# Uncultured Microbial Phyla Suggest Mechanisms for Multi-Thousand-Year Subsistence in Baltic Sea Sediments

**DOI:** 10.1128/mBio.02376-18

**Published:** 2019-04-16

**Authors:** Jordan T. Bird, Eric D. Tague, Laura Zinke, Jenna M. Schmidt, Andrew D. Steen, Brandi Reese, Ian P. G. Marshall, Gordon Webster, Andrew Weightman, Hector F. Castro, Shawn R. Campagna, Karen G. Lloyd

**Affiliations:** a University of Tennessee, Knoxville, Tennessee, USA; b University of Southern California, Los Angeles, California, USA; c Texas A&M Corpus Christi, Corpus Christi, Texas, USA; d Aarhus University, Aarhus, Denmark; e Cardiff University, Cardiff, Wales; Max Planck Institute for Marine Microbiology

**Keywords:** deep subsurface, enzyme assays, low energy, marine sediments, metabolomics, metatranscriptomics, single-cell genomics, subsistence

## Abstract

Energy-starved microbes in deep marine sediments subsist at near-zero growth for thousands of years, yet the mechanisms for their subsistence are unknown because no model strains have been cultivated from most of these groups. We investigated Baltic Sea sediments with single-cell genomics, metabolomics, metatranscriptomics, and enzyme assays to identify possible subsistence mechanisms employed by uncultured *Atribacteria*, *Aminicenantes*, *Actinobacteria* group OPB41, *Aerophobetes*, *Chloroflexi*, *Deltaproteobacteria*, *Desulfatiglans*, *Bathyarchaeota*, and *Euryarchaeota* marine group II lineages. Some functions appeared to be shared by multiple lineages, such as trehalose production and NAD^+^-consuming deacetylation, both of which have been shown to increase cellular life spans in other organisms by stabilizing proteins and nucleic acids, respectively. Other possible subsistence mechanisms differed between lineages, possibly providing them different physiological niches. Enzyme assays and transcripts suggested that *Atribacteria* and *Actinobacteria* group OPB41 catabolized sugars, whereas *Aminicenantes* and *Atribacteria* catabolized peptides. Metabolite and transcript data suggested that *Atribacteria* utilized allantoin, possibly as an energetic substrate or chemical protectant, and also possessed energy-efficient sodium pumps. *Atribacteria* single-cell amplified genomes (SAGs) recruited transcripts for full pathways for the production of all 20 canonical amino acids, and the gene for amino acid exporter YddG was one of their most highly transcribed genes, suggesting that they may benefit from metabolic interdependence with other cells. Subsistence of uncultured phyla in deep subsurface sediments may occur through shared strategies of using chemical protectants for biomolecular stabilization, but also by differentiating into physiological niches and metabolic interdependencies.

## Abstract (precis)

Much of life on Earth exists in a very slow-growing state, with microbes from deeply buried marine sediments representing an extreme example. These environments are like natural laboratories that have run multi-thousand-year experiments that are impossible to perform in a laboratory. We borrowed some techniques that are commonly used in laboratory experiments and applied them to these natural samples to make hypotheses about how these microbes subsist for so long at low activity. We found that some methods for stabilizing proteins and nucleic acids might be used by many members of the community. We also found evidence for niche differentiation strategies, and possibly cross-feeding, suggesting that even though they are barely growing, complex ecological interactions continue to occur over ultralong timescales.

## INTRODUCTION

The marine subseafloor is an enormous reservoir of organic carbon (1) and one of Earth’s largest biospheres (2). Here microbial biomass turns over orders of magnitude more slowly, and microbes subsist using orders of magnitude less energy, than any pure culture (3, 4). However, little is known about these ecosystems, because most of their microbial lineages have never been cultured (5). Success in an ecosystem that remains undisturbed for many thousands of years with no nutrient replenishment is likely to be determined by the ability to decrease maintenance energies and growth rates to levels supported by the environment (6). Therefore, these cells may not be persisting or starving but are instead subsisting on very low energies that nonetheless meet the per-cell requirements of at least a fraction of community members (7, 8). Since experiments on these timescales are unrealistic, we analyzed biomolecules and activities of these uncultured organisms in natural sediments to predict mechanisms for their subsistence.

We obtained sediments from IODP Expedition 347: Baltic Sea Paleoenvironment up to 85 m below seafloor (mbsf), representing >44,000 years of sedimentation (9). Samples from Lille Belt (M0059) spanned a glacial cycle, with lacustrine deposits below 50 mbsf, while samples for Anholt Basin (M0060) were entirely glacial deposits that were older and more organic poor than M0059, suggesting that the sediments may have been reworked before their final deposition (9). The total organic carbon was relatively high for the marine deposits above 50 mbsf in M0059 (3 to 8%) but were well below 1% for deeper M0059 samples and all depths of M0060. Metagenomic studies of these samples showed that, like in many marine sediments, the microbial communities were functionally diverse (10). Additionally, the majority of the microbes appear to be alive and able to adapt to changing environmental conditions, since lacustrine sediment layers that have experienced a salinity increase due to diffusion from the overlying glacial deposits contain more genes for salinity tolerance than lacustrine samples with the original freshwater (10).

While metagenomes have been extremely important for predicting the functions of uncultured microbes in deep subsurface sediments (10, 11), they are insufficient for inferring microbial activity *in situ*. This is due to the presence of extracellular DNA which may be sequenced alongside intracellular DNA obtained from lysed cells (12). In addition, the low biomass and DNA concentration and high species diversity in deep-marine sediment samples can make obtaining sufficient read coverage needed for genomic assembly from metagenomes more difficult. Assembled metagenomes from similar Baltic Sea sediment samples produced few long contigs necessary for metagenomic binning (10). We combined single-cell amplified genomics with transcriptomics, environmental metabolomics, and targeted enzyme activity assays to build a case for the strategies used by each of the uncultured microbial lineages *in situ*.

## RESULTS AND DISCUSSION

**FIG 1:**
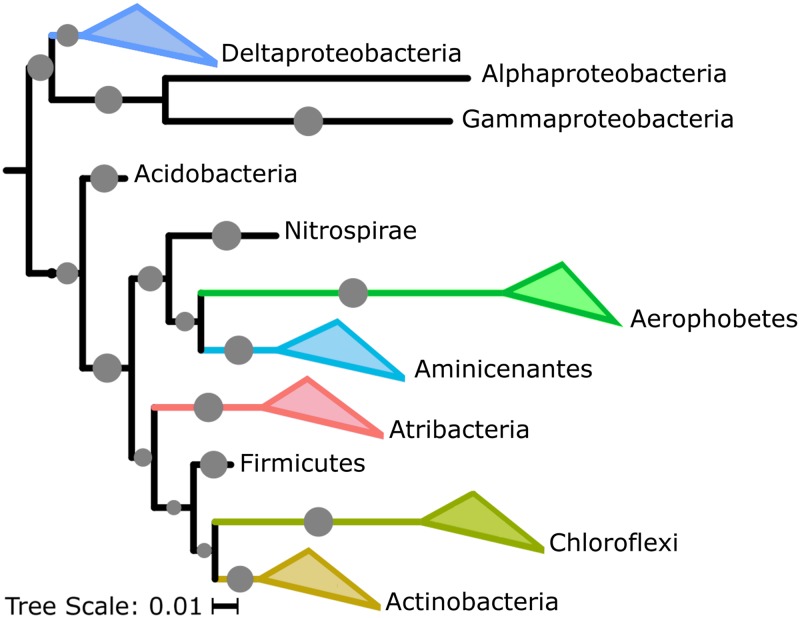
Phylogeny of SAGs from diverse and abundant bacterial lineages. Shown is a 16S rRNA gene maximum likelihood tree, with >80% bootstrap support indicated by gray dots; SAGs are in colored triangles.

**FIG 2:**
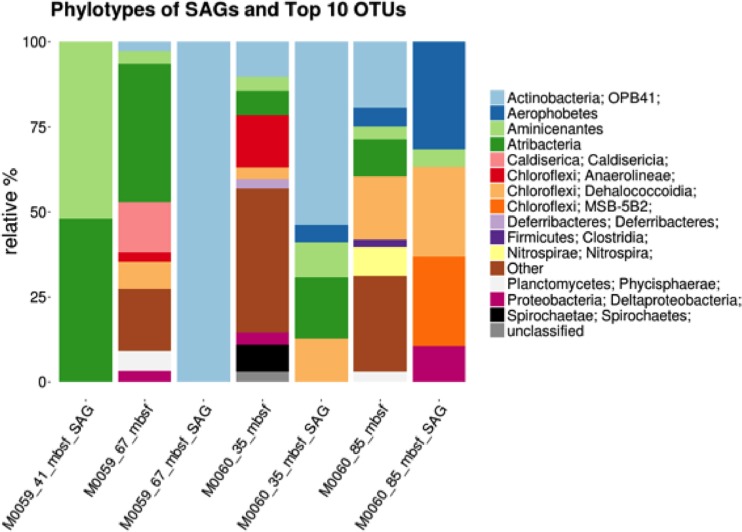
Operational taxonomic unit (OTU) composition for three 16S rRNA gene-based microbiomes of Baltic Sea sediment horizons. Relative abundances are displayed in the stacked bar graphs. The taxonomy of each of the top 10 most abundant OTUs is detailed based on its closest match in the SILVA 119 database, with some corrections for recently named taxonomies. The label “Other” represents the proportion of OTUs not within the top 10 in abundance. The taxonomy and composition of the SAGs recovered are represented in the stacked bar graphs with the “SAG” label.

**FIG 3:**
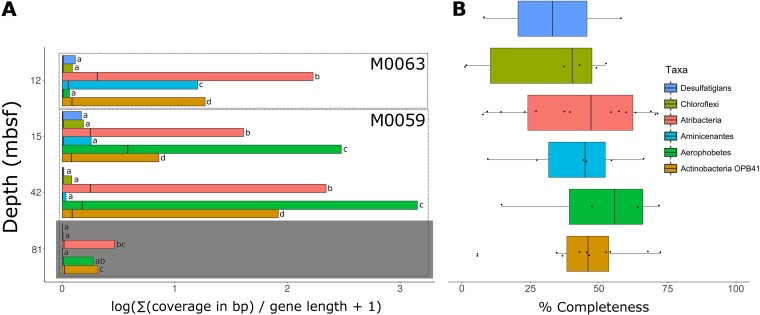
Recruitment of transcripts to SAG lineages and estimated genome completeness. (A) SAG transcript recruitment. Black bars show means, box edges are the 1st and 99th percentiles, and gray shading indicates lacustrine sample. (B) Genome completeness for each SAG.

Forty-six single-cell amplified genomes (SAGs) from M0059 at 41 and 68 mbsf and M0060 at 37 and 84 mbsf, metatranscriptomes from M0059 at 15, 41, and 81 mbsf and Landsort Deep (M0063) at 12 mbsf, and metabolites along a depth profile from M0059 and M0060 were analyzed (Fig. 1; see also Table S1 in the supplemental material). Metatranscriptomes from M0060 were not analyzed in this study due to insufficient mRNA read recovery in sequenced samples. Initial taxonomic classifications of SAGs were obtained by the alignment of partial 16S sequences to the SILVA short subunit database amplified from degenerate PCR primers at the Single-Cell Genomics Center at Bigelow Laboratories (13, 14). Of the 46 SAGs that were selected for sequencing, 31 contained full-length 16S rRNA gene sequences, which could be used for taxonomic classification. For 13 of the 15 remaining SAGs, maximum likelihood trees built from alignments of 15 single copy conserved ribosomal genes revealed relatively close relationships with other SAGs containing full-length 16S sequences within the context for a reference phylogeny (15). The MG2_P15 and Unk_M15 SAGs were not able to be included due to low genome completeness. SAG completeness, based on a larger set of single-copy conserved gene estimates, ranged from 1% to 73%, and percent redundancy ranged from 0% to 3% except for JS1_K04 at 14% and three other JS1 SAGs, at 50 to 58% (Table S1). The genomes with redundant markers, with one exception (JS1_K04), contained only strain-level contamination. The SAGs represented the dominant phyla in 16S rRNA gene libraries (Fig. 1 and 2) (16), which were uncultured phyla *Aminicenantes* (OP8), *Atribacteria* (JS1/OP9), and *Aerophobetes* (NT-B2) (Fig. 1). SAGs were also from uncultured groups OPB41 within *Actinobacteria*, *Desulfatiglans* within *Deltaproteobacteria*, and multiple *Chloroflexi* groups (Fig. 1). *Bathyarchaeota* (MCG) and *Euryarchaeota* MGII SAGs were also recovered despite a slightly (less than 10-fold) lower abundance of archaea than bacteria (17). M0059 SAGs included eight *Atribacteria* and four *Aminicenantes* at 41 mbsf and four *Actinobacteria* OPB41 at 68 mbsf. M0060 SAGs included seven *Atribacteria*, six *Actinobacteria* OPB41, two *Aerophobetes*, one *Chloroflexi*, one *Aminicenantes*, and one *Bathyarchaeota* at 37 mbsf. At 84 mbsf four *Chloroflexi*, two *Desulfatiglans*, two *Aerophobetes*, one *Amnicenantes*, one *Euryarchaeota* MGII, and one SAG for which a lineage could not be assigned were recovered. All SAGs recruited transcripts, suggesting that they represented living microbes (Fig. 3A). Metagenomes (10) were not used to normalize metatranscriptomes (18), because they were not extracted from the same samples with similar methods. Transcript read recruitment provides a combination of cellular abundance and transcriptional activity. SAGs within each lineage were considered together, to minimize the influence of various completeness levels (Fig. 3A). There was significantly more (*P* < 0.05; Tukey’s mean test) read recruitment among the *Atribacteria*, *Aminicenantes*, and *Actinobacteria* OPB41 in M0059 and *Atribacteria*, *Aerophobetes*, and *Aminicenantes* in M0063 than the other lineages.

10.1128/mBio.02376-18.1 TABLE S1 Genome sources and accessions. Download Table S1, DOCX file, 0.02 MB. Copyright © 2019 Bird et al. 2019 Bird et al. This content is distributed under the terms of the Creative Commons Attribution 4.0 International license.

**FIG 4:**
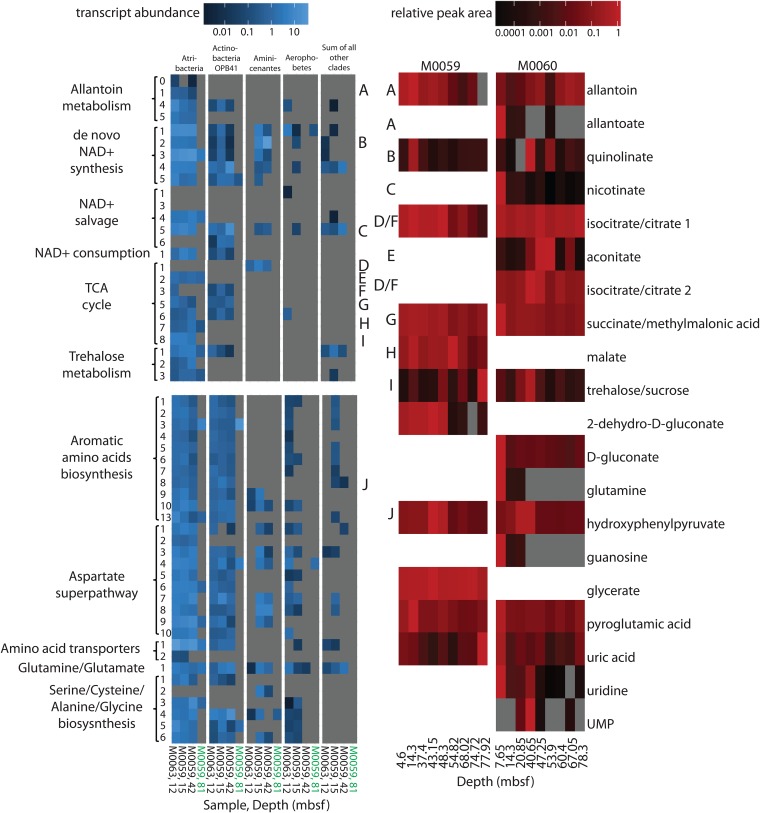
Metabolic pathways for most metabolites were transcribed differentially among deep subsurface community members. Transcript abundance (blue, coverage of each base pair in gene divided by gene length), summed from SAGs in each of the five lineages listed across the top, for the pathways for the metabolites (red, peak areas divided by largest peak area for that metabolite). Letters connect encoded enzymes to their metabolite. Enzymes for each numbered step in the pathway are listed in Table S2. All identified metabolites are shown. Products below detection limit are in gray. Samples for transcripts are marine (black) or lacustrine (green).

Twenty metabolites were identified in the samples (Fig. 4) and were absent in negative controls of extraction reagents. The metabolites represent a mixture of intra- and extracellular metabolite pools, and these methods are more likely to detect hydrophilic, dephosphorylated, polar, and small (<1,000 *m/z*) molecules (19). Metabolites were considered likely to indicate metabolic activity only when they were supported by genomic and transcriptomic data. Seventeen metabolites were identified at site M0060 and 11 at site M0059. This was far fewer than the number identifiable in pure microbial cultures with high biomass (20), supporting the fact that these subsurface populations functioned at a very low metabolic rate. Metabolites of the tricarboxylic acid (TCA) cycle were present at most depths, and the corresponding genes and transcripts were present in *Atribacteria* and *Actinobacteria* OPB41 (Fig. 4). This corroborates transcriptomic evidence (16) that these uncultured phyla were alive and metabolically active *in situ*. *Atribacteria* was the only lineage with a nearly complete TCA cycle, lacking genes only for first and sixth steps, citrate synthase and succinate dehydrogenase. However, *Atribacteria* had arginyl succinate synthase and arginosuccinate lyase genes, which were transcribed and were adjacent to fumarase genes, and may have provide fumarate for the TCA cycle (21). Citrate synthase was not found in the *Atribacteria* lineage; however, homologs to noncanonical citrate synthase such as Re-citrate synthase were found in the lineage (22, 23). Including arginyl succinate synthase and arginosuccinate lyase as a potential source of fumarate, the *Atribacteria* SAGs transcribe genes for a complete TCA cycle, apart from the missing citrate synthase, which is transcribed by *Aminicenantes*. However, it is important to consider that the missing genes in this and other lineages may be in the unsequenced portions of the SAGs, some genes maybe too divergent to be annotated, or lineages may have modified TCA cycles (Fig. 4).

10.1128/mBio.02376-18.2 TABLE S2 List of enzymes in pathways that are related to metabolites. When multiple enzymatic routes are possible for a single step, this is indicated by adding a digit after the decimal of the step. Only the enzymatic steps that were present in the SAGs are listed. Download Table S2, DOCX file, 0.02 MB. Copyright © 2019 Bird et al. 2019 Bird et al. This content is distributed under the terms of the Creative Commons Attribution 4.0 International license.

Two possible subsistence mechanisms appeared to be shared by multiple lineages. In the first, quinolinate was present in all samples except one depth at site M0060 and nicotinate was present in all depths at site M0060 (Fig. 4). These metabolites are precursors to NAD^+^ in the *de novo* synthesis and salvage pathways, respectively (24). A complete *de novo* pathway and a partial salvage pathway were expressed by *Atribacteria*, *Actinobacteria* OPB41, and *Aminicenantes* (Fig. 4). NAD^+^ is an electron carrier used nondestructively in energy conservation, but the presence of transcripts and metabolites for NAD^+^ synthesis pathways across many samples suggests that NAD^+^-consuming processes, such as deacetylation, may also occur. Transcripts for NAD^+^-consuming deacetylases were in the top 10% of the total transcriptome and recruited to *Atribacteria* and *Actinobacteria* OPB41. *Aminicenantes*, *Chloroflexi*, and *Desulfatiglans* had genes encoding the enzyme but did not recruit transcripts. NAD^+^-consuming deacetylases are important posttranslational modifiers of lysine residues in prokaryotes (25). Deacetylation of lysines restores their positive charge, causing tighter binding to negatively charged molecules like DNA and RNA (25). In bacteria, NAD^+^-consuming deacetylases target a wide variety of proteins, including those involved in metabolism and protein translation, and appear to be a global response to cellular energy levels (26). In eukaryotes and archaea, they have long been known to silence gene transcription, repair DNA breaks, and increase cellular life span (27). Cellular deacetylation may therefore be a subsistence mechanism employed by multiple lineages in deep marine sediments to maintain nucleic acid integrity or regulate gene expression.

In the second shared mechanism, trehalose was present in all samples and five of the six bacterial lineages encoded trehalose synthase and trehalose transporters SugA/SugB or MalG/MalF, all of which recruited transcripts in *Atribacteria*, *Actinobacteria* OPB41, and *Chloroflexi* (Fig. 4). In contrast, trehalose synthase was rare in cultures (2.9% of archaea and 0.1% of bacteria, EC 2.4.1.245 in complete and permanent draft genomes on the Joint Genome Institute Integrated Microbial Genomes [JGI/IMG]). Although the metabolite trehalose peak was indistinguishable from that of sucrose, sucrose-related genes were not widespread among lineages or highly expressed, suggesting that the peak was more likely to be trehalose. Trehalose increase with depth may indicate accumulation with sediment age or microbial responses to changes in salinity over those depths. Trehalose is a universal stress molecule and osmolyte that also stabilizes proteins. It is associated with increased life span and decreased growth rate in eukaryotes and bacteria (20, 28). Trehalose is more effective than other sugars at altering the water environment around proteins (29), so even if the cells’ primary usage of trehalose is osmoregulation, they would benefit from its protein-stabilizing properties. Protein repair is the largest energetic expenditure a cell must make (30), so trehalose may minimize these costs by stabilizing proteins and decreasing the repair rate required for proteins.

**FIG 5:**
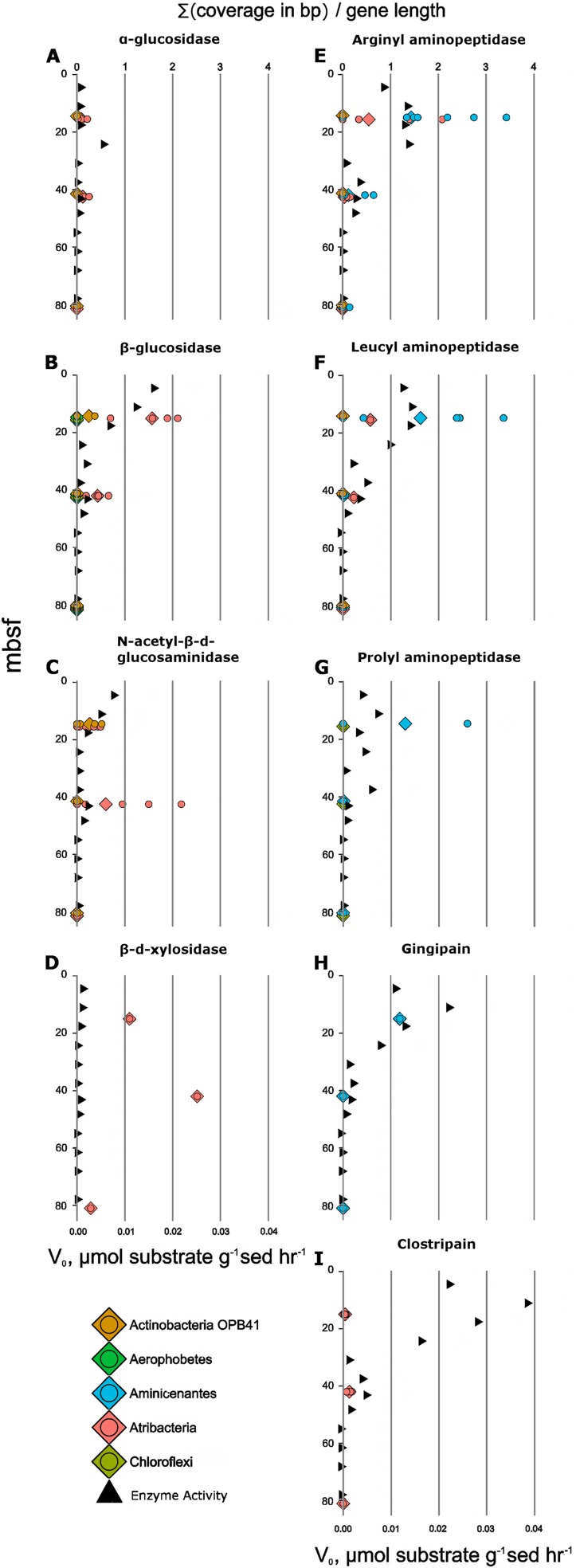
Enzyme activities (triangles) correlated with their transcript abundances (circles). Shown are the following proteins, identified by their domains in parentheses and substrate proxies: α-glucosidase (PS01324) and MUB-α-d-glucopyranoside (A), β-glucosidase (TIGR03356) and MUB-β-d-glucopyranoside (B), *N*-acetyl-β-d-glucosaminidase (PTHR30480) and MUB-*N*-acetyl-β-d-glucosaminide (C), β-d-xylosidase (PF04616) and MUB-β-d-xylopyranoside (D), arginyl aminopeptidase (PF03577) and l-arginine-AMC (E), leucyl aminopeptidase (PTHR12147) and leucine-AMC (F), prolyl aminopeptidase (PTHR10804:SF17) and H-proline-AMC (G), gingipain (PF01364) and Z-phenylalanine-arginine-AMC (H), and clostripain (PF03415) and Z-phenylalanine-valine-arginine-AMC (I).

Catabolic substrate utilization, on the other hand, appeared to differ between lineages. Previous metabolic reconstructions for some of these lineages predicted extracellular hydrolysis of peptides and carbohydrates (31–34). However, transcripts for hydrolytic enzymes have never been used to link enzyme activity measurements to specific lineages in the deep subsurface. Potential activity (v_0_) of five peptidases and four carbohydrate hydrolases decreased with depth, while transcript abundance of their homologs correlated with v_0_ for seven of them (Fig. 5). *Aminicenantes* and *Atribacteria* exopeptidase transcripts (leucyl aminopeptidase and arginyl aminopeptidase) also decreased with depth, with *Aminicenantes* transcripts being more abundant than transcripts from *Atribacteria*. Activity of the exopeptidase prolyl aminopeptidase and the endopeptidase gingipain decreased with depth, along with their transcripts in *Aminicenantes*. Only the depth trend for the activity of the endopeptidase clostripain did not correlate with changes in transcript abundance, although *Atribacteria* did produce some clostripain transcripts. Clostripain may have been produced by genes or lineages that had poor SAG recovery, such as *Bathyarchaeota* and *Euryarchaeota* MBG-D, which have previously been shown to encode clostripain (31). Multiple peptidases in *Aminicenantes* were in the top 10% of total community transcripts (see the supplemental data), which, together with our enzyme activity results, support the initial conclusion based on genes alone (32) that this group specializes in protein degradation.

*Atribacteria* and *Actinobacteria* OPB41 glycosyl hydrolase transcript abundance decreased with depth along with glycosyl hydrolase activity (α-glucosidase, β-glucosidase, and *N*-acetyl-β-glucosaminidase) (Fig. 5), with *Atribacteria* having greater transcript abundance than *Actinobacteria* OPB41. The genes for carbohydrate-active enzymes were some of the most highly expressed genes in these lineages, consistent with previous suggestions that *Atribacteria* consume sugars (33, 35). *Atribacteria* transcribed xylosidase-like genes, but the assays showed little activity for them, suggesting either that these transcripts were not translated into functional proteins or that these genes had a novel substrate specificity. Collectively, these data support catabolic niche partitioning, with specific lineages expressing hydrolases for distinct classes of the macromolecules within the shared detrital carbon pool. Establishing interspecific preferences for catabolic substrates allows the development of stable states, where microbial diversity is maintained by alleviating competition (36). In plants, interspecific competition has been shown to decrease during long periods of externally imposed resource limitation (37). This suggests that avoidance of competition may be a subsistence mechanism in the subsurface, where little catabolic energy is available to fuel competitive traits such as fast growth or antibiotic production. Catabolic niche partitioning could work together with intracellular nutrient recycling, which has also been hypothesized to allow organisms to avoid competition (38). Therefore, our observed catabolic niche partitioning among deep subsurface microbes may allow a high microbial diversity to be maintained through competition avoidance under extreme resource limitation.

Other possible subsistence mechanisms could only be ascribed to *Atribacteria*, possibly because *Atribacteria* had high transcript recruitment at all marine depths (Fig. 3A), in agreement with previous studies showing that they are very abundant (39, 40). The metabolite allantoin, a purine degradation product and potential catabolic substrate, was present in most samples (Fig. 4). Only *Atribacteria* encoded the allantoin-degrading enzyme allantoinase (step 1) and allantoin transporters (step 0), and total *Atribacteria* transcript abundances were higher when more allantoin metabolite was retrieved (Fig. 3 and 4). Intermediates in the purine degradation pathway to allantoin, guanosine and uric acid, as well as the product of allantoinase activity, allantoate, were also found in some samples (Fig. 4). Allantoin degradation yields energy through substrate-level phosphorylation and produces carbamoyl phosphate, a necessary anabolic substrate for proline, arginine, and nucleotides. Allantoin degradation genes (genes for carbamoyltransferase and allantoinase) appeared to be in operons in *Atribacteria* since they were adjacent, shared reading frames, and recruited similar amounts of transcripts. One allantoinase operon had an upstream transcription regulatory element from the GntR family, which represses allantoin degradation in Klebsiella pneumoniae (41). This operon had high transcript abundance for GntR and the lowest transcript abundance for allantoinase, consistent with GntR working as a regulatory element in *Atribacteria* as well. *Atribacteria* contained three possible transporters for allantoin. In Escherichia coli, allantoinase is in an operon with a purine transporter. In two *Atribacteria* SAGs, an ATP-dependent transporter with a purine binding protein was encoded directly behind allantoin degradation genes but did not recruit transcripts across the length of the gene. Instead, a nearby ATP-independent TRAP-type C_4_-dicarboxylate transporter which uses more energy-efficient Na^+^ gradients (42) recruited transcripts. The third potential allantoin transporter was an allantoin permease that recruited transcripts but was located on a separate contig from purine degradation genes. The sample that yielded no *Atribacteria* SAGs or 16S rRNA transcripts (M0059, 81 mbsf) also had undetectable allantoin and no transcripts for allantoinases. Allantoin catabolism may be *Atribacteria*’s sole route for carbamoyl phosphate production (steps 4 and 5), since the alternative l-glutamine pathway (43) is absent in all 15 *Atribacteria* SAGs. Transcript recruitment to the downstream pathways for UTP and CTP synthesis as well as the detection of orate and UMP metabolites suggest that the carbamoyl phosphate involving pathways was active.

**FIG 6:**
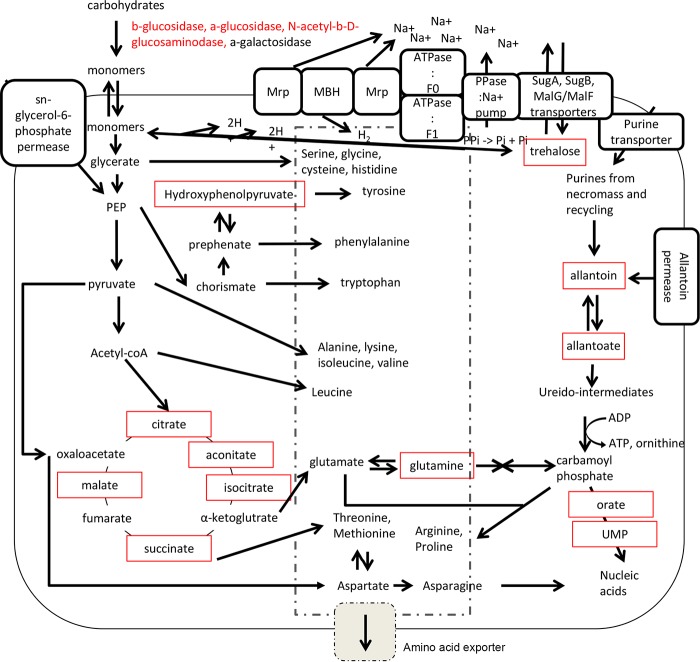
Unique attributes of *Atribacteria* provide advantages in energy-limited marine sediments. All elements include evidence at the transcript level; red boxes indicate detected metabolites, and red text indicates detected enzyme activity.

*Atribacteria* also appeared to catabolize organohalides, due to abundant transcripts for five reductive dehalogenases that were likely to be catabolic rather than respiratory. This is because they were not associated with a respiratory membrane complex and they lacked the necessary signaling motifs to target the enzyme to the membrane (44). Dehalogenation activity is consistent with the predictions from the metagenomes from these samples (10). *Atribacteria* were the only bacteria that encoded a membrane-bound hydrogenase complex cotranscribed with an adjacent oxidoreductase (45). This complex, which has been observed in previous atribacterial genomes (33, 35, 46), can accept electrons from ferredoxin or NADH to produce H_2_ from two protons and/or reduce sulfur compounds to create a proton motive force. This may then be converted to an Na^+^ motive force by *Atribacteria*’s two flanking multigene Mrp Na^+^/H^+^ antiporters. Atribacterial ATP synthases were specific for Na^+^ rather than H^+^ due to similarities in the conserved regions of the c subunits. At the conserved position 67, which has a threonine in Na^+^-specific ATP synthases (47), *Atribacteria* had either an asparagine or an serine, both of which would retain the properties of being polar and uncharged. So, the water molecule necessary during Na^+^ transport would most likely still be capable of coordinating at this site. These genes were adjacent to pyrophosphatase/Na^+^ pumps, which recycle pyrophosphates to maintain the Na^+^ gradient (Fig. 6). Cell membranes are less permeable to Na^+^ than H^+^; therefore, ATP generation via Na^+^ transport increases an organism’s energy efficiency (48, 49). Sodium pumps have been hypothesized to be a general strategy for life in energy-limited systems (50). We suggest that *Atribacteria*’s ability to utilize sodium motive force is a key factor allowing it to survive long-term energy limitation.

Although many lineages appeared capable of making at least some amino acid precursors from the TCA cycle, only *Atribacteria* contained all the genes necessary for *de novo* synthesis of 20 amino acids, all of which were transcribed (Fig. 4). The metabolite hydroxyphenylpyruvate, an intermediate in aromatic amino acid synthesis, was in high abundance in samples with high *Atribacteria* transcript abundance for the enzyme that produces it, chorismate mutase, at both sites (Fig. 4). The multi-amino acid exporter YddG (51) was one of the most highly transcribed genes in four atribacterial SAGs, and the gene for this protein was absent in all other SAGs (Fig. 4 and 6); a second predicted amino acid exporter was also transcribed in *Atribacteria*. Together with the transcript and metabolite evidence for amino acid synthesis, this suggests that *Atribacteria* may have exported amino acids. At first, releasing an energetic molecule while trying to survive under extreme energy limitation seems counterintuitive. However, when human kidney cells are placed under energy starvation conditions, they immediately metabolize free amino acids, not for their energetic gains but in order to halt protein production and cell proliferation (52). This is consistent with experiments showing that bacterial intracellular amino acid concentrations plunge upon energy limitation (15), and these low concentrations decrease growth rate (53). A possible subsistence mechanism for *Atribacteria* may therefore be the suppression of intracellular concentrations of amino acids to maintain a near-zero growth rate. The unique ability of *Atribacteria* to synthesize and export amino acids *de novo* in this energy-limited environment suggests that it may provide them as energetic substrates for other microbial lineages, which we observed to transcribe amino acid transporters. Such metabolic interdependencies have been observed in more energy-rich subsurface environments (54).

Metabolites, SAGs, transcripts, and enzyme activities suggest subsistence mechanisms for uncultured Baltic Sea microbial communities that have been buried for thousands of years under very low energy availability. Collectively, these mechanisms suggest that the majority of organisms in this environment are geared toward maintenance activities, rather than growth, consistent with environmental energetic assessments (4), metagenomes (10), and modeling (55). Some mechanisms appeared to be shared between multiple lineages, such as the stabilization of cellular components with trehalose and NAD^+^-consuming pathways. Other mechanisms differed between organisms, such as catabolic niche partitioning between community members, which may have helped to decrease competition. *Atribacteria*, for which most information was available, showed evidence of additional subsistence mechanisms, such as utilizing an energy-efficient Na^+^-motive force, depressing intracellular amino acid concentrations, and possibly contributing to metabolic interdependencies. Together, these biomolecules suggest a complex and interactive ecosystem that uses multiple mechanisms to subsist at near-zero growth over long-term burial in anoxic marine sediments.

## MATERIALS AND METHODS

### Data collection and division of labor.

Samples were collected during the IODP Expedition 347: Baltic Sea Paleoenvironment, 12 September through 1 November 2013, on the *Greatship Manisha*, which was outfitted as a mission-specific platform. This expedition was a collaboration of many different laboratories, which each received separate samples. Every effort was made to make sure samples for different laboratories were taken close to each other. RNA extraction and metatranscriptome sequencing were performed in the laboratory of Brandi Reese at Texas A&M Corpus Christi, along with Laura Zinke, with detailed methods described by Zinke et al. and below, with sequencing at the MR DNA Laboratory (16). DNA extraction and metagenome sequencing were performed in the laboratory of Bo Barker Jørgensen at Aarhus University, along with Ian Marshall, with detailed methods described by Marshall et al. (10). Single-cell amplified genomes were generated in the laboratory of Karen Lloyd at the University of Tennessee, along with Jordan Bird, with detailed methods described below, and sequencing at the University of Tennessee. DNA extractions for 16S rRNA gene libraries were performed in all three of these laboratories for different samples. Metabolite data were generated in the laboratory of Shawn Campagna, along with Hector Castro-Gonzalez and Eric Tague, at the University of Tennessee, with detailed methods described below. Enzyme assays were generated in the laboratory of Andrew Steen, along with Jenna Schmidt, at the University of Tennessee, with detailed methods described below. DNA and RNA data were acquired for fewer samples than for metabolites and enzyme assays because the former required more financial and time resources per sample. Sequencing of three *Chloroflexi* SAGs (Table S1) and the 16S rRNA genes at the Marine Biological Laboratory (Woods Hole, MA) was supported through the Census of Deep Life program within the Deep Carbon Observatory. Sequencing and initial analysis of 6 *Atribacteria* SAGs were performed in the Andrew Weightman lab at Cardiff University, along with Gordon Webster, with sequencing at Edinburgh Genomics.

### Metabolite extraction from marine sediments.

Sediment samples were removed from a −80°C freezer and within 5 min shavings were transferred to sterile plastic tubes in 100-mg triplicate subsamples and returned to the freezer. Samples were later ground to a powder using a mortar and pestle sitting in liquid nitrogen. Technical replicates were created by portioning out approximately 100 mg of ground sample. A total of 1.3 ml of chilled extraction solvent (40:40:20, acetonitrile-methanol-water plus 0.1 M formic acid) was added and the mixture was vortexed to suspend the sample. The extraction process lasted 20 min at 4°C on an orbital shaker, and then the samples were centrifuged at 16,800 × *g* for 5 min. The supernatant was removed and collected in a separate vial. Then a second extraction was performed using a 200 µl of extraction solvent and the same extraction process. After both volumes of supernatant were collected in the same vial, the solvent was evaporated to dryness using nitrogen gas. The dried samples were then suspended using 300 µl of sterile water and transferred to autosampler vials for analysis. The low metabolite yield relative to pure cultures (20) was likely due to low cell abundance and activity, rather than sediment matrix interference during extraction, since the removal of cells from sediment prior to extraction did not increase the number of metabolites even though 50 times more cells were used.

### UPLC-MS.

Mass spectrometric (MS) analysis was performed using an Exactive Plus orbitrap mass spectrometer (Thermo Scientific) coupled to an ultraperformance liquid chromatography (UPLC) system (Dionex). The separation was achieved using a Synergi HydroRP column (Phenomenex) with a solvent gradient modeled after that described in reference 19. The sample was introduced using electrospray ionization, and full scan data from 85 *m/z* to 1,000 *m/z* were collected in negative mode.

Data generated from the mass spectrometer’s software were converted using the MSConvert package of the ProteoWizard (56) toolkit software. Files were then imported into the MAVEN (57) software to visualize chromatograms for specific *m/z* ratios. Using a list of known retention times and exact *m/z* values (±5 ppm), metabolites were selected, and ion counts were compiled from areas under the curve.

### Single-cell extractions.

Cell extraction methods followed a previously published one (31), with the following minor modifications. Samples were preserved in 1:1 (glycerol-sediment) mixture frozen on the ship. The samples were shipped back to the University of Tennessee on dry ice. Three milliliters of the sample mixture was removed into a sterile 15-ml plastic tube. An additional artificial seawater buffer (ASW), either 30% or 15% depending on the sample salinity, was added to bring the volume up to 10 ml. The tip of a Misonix Microson ultrasonic cell disruptor was placed in an ice bath next to the plastic tube containing the sample and manually sonicated two times per second at 20% power to dislodge cells from their associated minerals. Next, the tube was vortexed for 10 s and the sediment was allowed to settle for 10 min. Five-milliliter aliquots of the supernatant were gently laid on top of an equal volume of 60% Nycodenz solution in a sterile 15-ml tubes. The tubes were then centrifuged at 11,617 × *g* and 4°C for 1 h in a Fiberlite rotor. When the sediment in the sample climbed up the sidewall past the Nycodenz boundary, the sample was again spun at 5,000 rpm in the swinging-bucket rotor. The layer above the Nycodenz gradient was carefully removed and pooled into a sterile 15-ml plastic tube. Lastly, 1.5-ml aliquots were mixed with 375 μl of a 5% glycerol 1× Tris-EDTA (TE) solution and immediately placed at −80°C.

### Transcriptomes.

On the ship, samples were frozen immediately upon retrieval at −80°C. Approximately 7.5 g of frozen sediment was chipped from whole round cores in a clean room using flame-sterilized instruments. Samples were extracted from the middle of the core, which was determined to have little or no contact with the drill fluid (9). RNA was extracted from sediment using a MoBio PowerSoil RNA kit following the manufacturer’s instructions (MoBio Laboratories, Carlsbad, CA).

Total RNA extractions were treated with Ambion Turbo DNase (Thermo Fisher Scientific, Waltham, MA) according to manufacturer protocols. Resulting RNA purity and quantity were checked using an Eppendorf BioSpectrometer (Eppendorf, Hauppauge, NY) and by reverse transcription followed by PCR amplification of 16S small-subunit (SSU) rRNA. DNase-treated RNA extract was PCR amplified to determine if DNase treatment was effective. RNA extracts were shipped on dry ice to the MR DNA Laboratory, LLC, in Shallowater, TX, for library preparation and sequencing analysis. rRNA was not removed and mRNA enrichment was not implemented.

For metatranscriptomes, cDNA synthesis was performed using the Nextera RNA sample preparation kit following manufacturer’s instructions (Illumina, San Diego, CA). Libraries were sequenced on the Illumina HiSeq 2500 platform (Illumina, San Diego, CA) for 500 cycles with 250-bp paired-end chemistry. Transcripts were trimmed with TRIMMOMATIC using the ILLUMINACLIP:NexteraPE-PE.fa:2:30:12:1:true LEADING:3 TRAILING:3 SLIDINGWINDOW:10:20 MINLEN:75 parameters. Bowtie2 and Samtools were used to filter reads matching the PhiX internal standard. And finally, reads were mapped to each of the SAG contigs with Bowtie2 using the default settings and combined by concatenating mapping from the same sample (58). Competing mapping to possible contaminants was not performed because (i) SAGs were phylogenetically divergent from common lab contaminants (59), making spurious overlaps across large parts of the genes unlikely, and (ii) any mismapping of reads from contaminants to SAGs would therefore be in genes that are highly evolutionarily conserved across the deepest branches of the tree of life, rather than the functional genes that are the focus of this study.

### Genome annotation and analysis.

Sequence data from 5 separate Illumina sequencing runs were combined to make the library of single-amplified-genome short-read data in the study. For all genome libraries sequenced at the University of Tennessee and MR DNA Laboratory, the Nextera library preparation was used. Genomes sequenced at the Marine Biological Laboratory used the TruSeq V2 library preparation kit (Table S1). Sequences from each Illumina sequencing run were quality trimmed using the TRIMMOMATIC software using the ILLUMINACLIP:{ADAPTER}:2:30:12:1:true LEADING:3 TRAILING:3 SLIDINGWINDOW:10:20 MINLEN:75 parameters (60). The remaining high-quality paired and single reads were assembled using the SPAdes 3.6 assembler with the following parameters: spades.py -m 62 -o <OUTPUT_DIR> –sc –12 <PAIRED_READS> -s <SINGLES> -t 15 -k 21,33,55,77,99,127 –careful (61). Pairwise comparsions of average nucleotide identity (ANI) between single-cell genomes were calculated use ANIcalculator from Lawerence Livermore National Lab (62). Coassembly of SAGs with ANIs of >97% resulted in small to moderately higher genome completeness values (98% for the *Atribacteria* M0060 cluster with greater than 99% ANI, 75% for the *Atribacteria* M0059 cluster, 68% for *Atribacteria* from M0060, 81% for OPB41 from M0060, 80% for OPB41 from M0059, 43% for *Desulfatiglans*, 66% for *Aerophobetes* 60, and 74% from OP8 from M0059), with no increase in contamination. However, the gene content of these coassemblies was not different enough from the individual assemblies to change their results.

Contigs with high sequence similarity to the PhiX internal standard, those with less than 1,000 bp and 5× coverage, and those contigs containing a only a single repeated nucleotide were removed. The remaining contigs from each genome were then annotated using the Prokka genome annotation software with a custom database which combined both of the *Bacteria*- and *Archaea*-specific databases provided with the software (63). Predicted proteomes of each genome were additionally annotated with INTERPROSCAN5 (64). Later, these annotations were combined in and visualized using the Anvi’o software (65). Anvi’o annotations of single-copy conserved genes were used to predict genome completeness and contamination levels. The Anvi’o single-copy-gene finder is a wrapper that uses hmmer3 to match “single copy genes” hmmpress profiles for genes described and calculates both completeness and “redundancy” values based on the presence and overlap of the hmm hits that are found (32, 66). The hmms can be found at https://github.com/merenlab/anvio/tree/master/anvio/data/hmm and are accessed when the anvi-run-hmm program is run. Potential contamination was further assessed with the CheckM software and manual checking of aligned redundant markers (67). The Anvi’o software profiling functions were used to analyze the read recruitment from the transcriptomes to the annotated genome features in each of the SAGs.

### Enzyme activities.

Enzymes were assayed according to the procedure described by Schmidt (68). All processing was performed under an atmosphere of 100% N_2_ in a glove box. Subsamples of –80°C-frozen core rounds were removed using a hand drill fitted with an ethyl alcohol (EtOH)-sterilized, 1-cm-interior-diameter hole saw bit. Subsamples were kept frozen until the time of the assay. Each depth was assayed on separate days. For each depth, 3 g of thawed sediment was mixed in a Waring blender for 1 min in 100 ml of sterile, anoxic, 0.2 M borate-buffed saline, per the best practice in soil enzyme assays (69). A separate slurry for each depth was autoclaved for 60 min on a liquid cycle to serve as a sterile control. Immediately after preparation, 960 µl of slurry was added to a 1-cm by 1-cm semimicro-style methacrylate cuvette and amended with 40 µl of a solution of fluorogenic enzyme substrate (Table S3). For each depth, three replicate “live” and three replicate “sterile” cuvettes were poured. Additionally, two 10-point calibration samples were created, using 7-amido-4-methylcoumarin (AMC) or 4-methylumbelliferone plus sediment. Cuvettes were capped and mixed, and fluorescence in each cuvette was measured immediately. The exact time of fluorescence measurement was noted. Samples were incubated at 20 to 22°C in the dark, and fluorescence was measured in each cuvette approximately 5 times over the course of 24 h. The ambient temperature was monitored during the incubation, and small variations in temperature between depths did not explain variation among depths. Calibration samples were also measured at each measurement time point, and sample fluorescence was calibrated separately for each time point.

10.1128/mBio.02376-18.3 TABLE S3 Enzyme substrates and nominal corresponding enzymes. Download Table S3, DOCX file, 0.01 MB. Copyright © 2019 Bird et al. 2019 Bird et al. This content is distributed under the terms of the Creative Commons Attribution 4.0 International license.

Changes in concentration of fluorophore over time were calculated using the R package enzalyze, available at https://github.com/adsteen/enzalyze. As described by Schmidt et al. (68), several lines of evidence suggested that increases in fluorescence over time in autoclaved sediment were due to incompletely denatured (or denatured and then renatured) enzymes, rather than abiotic processes. Therefore, uncorrected hydrolysis rates of “live” samples are reported as v_0_. In addition to the substrates listed in Table S3, a substrate for cellulobiose was assayed, but it did not have any detectable activity.

### Data visualization.

Software packages in the R statistical language (70) including ggplot2 (71) were utilized to produce the figures in this study. Analysis of variance (ANOVA) and Tukey’s mean testing comparing transcript recruitment between each microbial lineage used the base stats package in R. The Microsoft Office suite was used to produce tables and diagrams, while the GIMP image software was used to edit the appearance of figures (https://products.office.com/en-US/, https://www.gimp.org/).

### Data availability.

Sequencing reads for single-cell genomes were deposited in NCBI SRA and can be accessed under BioProject accession no. PRJNA417388. Metatranscriptome sequences were sequenced as described by Zinke et al. (16) and archived under the BioProject accession no. PRJNA388431.

10.1128/mBio.02376-18.4 DATA SET S1 A data file including the mean coverage values for transcripts recruitment from 4 transcriptomes to genes found within the 46 SAGs that were included in this study. Download Data Set S1, PDF file, 7.9 MB. Copyright © 2019 Bird et al. 2019 Bird et al. This content is distributed under the terms of the Creative Commons Attribution 4.0 International license.
