# Supplementary material for: Uncultured Microbial Phyla Suggest Mechanisms for Multi-Thousand-Year Subsistence in Baltic Sea Sediments
Source: mBio. 2019 Apr 16;10(2):e02376-18. doi: 10.1128/mBio.02376-18 (PMC6469976; doi:10.1128/mBio.02376-18)
Supplement: TABLE S1 [file mBio.02376-18-st001.docx]

**Supplemental Table S1.** Genome sources and accessions

| **Genome** | **Platform** | **Read Format** | **Location** | **Bioproject** | **Completeness** | **Redundancy** | **Site** | **mbsf** |
| --- | --- | --- | --- | --- | --- | --- | --- | --- |
| Chl_A19 | MiSeq | 2x250 | University of Tennessee, Knoxville, Tennessee | PRJNA417388 | 0.3741 | 0 | M0060 | 37 |
| Chl_A21 | MiSeq | 2x250 | University of Tennessee, Knoxville, Tennessee | PRJNA417388 | 0.4892 | 0 | M0060 | 84 |
| Chl_B06 | HiSeq | 2x125 | Marine Biological Laboratory, Woods Hole, Massachusetts | PRJNA417388 | 0.0144 | 0 | M0060 | 84 |
| Chl_C14 | MiSeq | 2x250 | University of Tennessee, Knoxville, Tennessee | PRJNA417388 | 0.5252 | 0 | M0060 | 84 |
| Chl_G11 | HiSeq | 2x125 | Marine Biological Laboratory, Woods Hole, Massachusetts | PRJNA417388 | 0.0144 | 0 | M0060 | 84 |
| Chl_N02 | HiSeq | 2x125 | Marine Biological Laboratory, Woods Hole, Massachusetts | PRJNA417388 | 0.4317 | 0 | M0060 | 84 |
| Dsu_D02 | HiSeq | 2x125 | Molecular Research DNA Laboratory, Shallowater, Texas | PRJNA417388 | 0.0791 | 0.0144 | M0060 | 84 |
| Dsu_E09 | HiSeq | 2x125 | Molecular Research DNA Laboratory, Shallowater, Texas | PRJNA417388 | 0.5827 | 0.0144 | M0060 | 84 |
| JS1_A09 | MiSeq | 2x300 | Edinburgh Genomics, University of Edinburgh, Edinburgh, UK | PRJNA417388 | 0.3957 | 0 | M0060 | 37 |
| JS1_C14 | MiSeq | 2x250 | University of Tennessee, Knoxville, Tennessee | PRJNA417388 | 0.1871 | 0 | M0059 | 41 |
| JS1_D03 | MiSeq | 2x300 | Edinburgh Genomics, University of Edinburgh, Edinburgh, UK | PRJNA417388 | 0.7122 | 0.4964 | M0060 | 37 |
| JS1_E13 | MiSeq | 2x300 | Edinburgh Genomics, University of Edinburgh, Edinburgh, UK | PRJNA417388 | 0.6906 | 0.5827 | M0060 | 37 |
| JS1_E15 | MiSeq | 2x250 | University of Tennessee, Knoxville, Tennessee | PRJNA417388 | 0.0791 | 0 | M0059 | 41 |
| JS1_E20 | MiSeq | 2x250 | University of Tennessee, Knoxville, Tennessee | PRJNA417388 | 0.3741 | 0.0144 | M0059 | 41 |
| JS1_F07 | MiSeq | 2x250 | University of Tennessee, Knoxville, Tennessee | PRJNA417388 | 0.2734 | 0 | M0059 | 41 |
| JS1_I07 | MiSeq | 2x300 | Edinburgh Genomics, University of Edinburgh, Edinburgh, UK | PRJNA417388 | 0.2302 | 0.0216 | M0060 | 37 |
| JS1_K04 | MiSeq | 2x250 | University of Tennessee, Knoxville, Tennessee | PRJNA417388 | 0.5755 | 0.1439 | M0059 | 41 |
| JS1_L14 | MiSeq | 2x250 | University of Tennessee, Knoxville, Tennessee | PRJNA417388 | 0.0935 | 0.0072 | M0059 | 41 |
| JS1_L23 | MiSeq | 2x250 | University of Tennessee, Knoxville, Tennessee | PRJNA417388 | 0.1439 | 0 | M0059 | 41 |
| JS1_M10 | MiSeq | 2x300 | Edinburgh Genomics, University of Edinburgh, Edinburgh, UK | PRJNA417388 | 0.705 | 0.0288 | M0060 | 37 |
| JS1_M21 | MiSeq | 2x300 | Edinburgh Genomics, University of Edinburgh, Edinburgh, UK | PRJNA417388 | 0.5468 | 0.0216 | M0060 | 37 |
| JS1_N06 | MiSeq | 2x300 | Edinburgh Genomics, University of Edinburgh, Edinburgh, UK | PRJNA417388 | 0.5971 | 0.5108 | M0060 | 37 |
| JS1_O21 | MiSeq | 2x250 | University of Tennessee, Knoxville, Tennessee | PRJNA417388 | 0.6331 | 0 | M0059 | 41 |
| MCG_K23 | MiSeq | 2x250 | University of Tennessee, Knoxville, Tennessee | PRJNA417388 | 0.358 | 0 | M0060 | 37 |
| MG2_P15 | HiSeq | 2x125 | Molecular Research DNA Laboratory, Shallowater, Texas | PRJNA417388 | 0.1727 | 0 | M0060 | 84 |
| NT-A5 | MiSeq | 2x250 | Molecular Research DNA Laboratory, Shallowater, Texas | PRJNA417388 | 0.4748 | 0.0072 | M0060 | 37 |
| NT-E05 | HiSeq | 2x125 | Molecular Research DNA Laboratory, Shallowater, Texas | PRJNA417388 | 0.7194 | 0.0144 | M0060 | 84 |
| NT-P03 | HiSeq | 2x125 | Molecular Research DNA Laboratory, Shallowater, Texas | PRJNA417388 | 0.1439 | 0 | M0060 | 84 |
| NT-P19 | HiSeq | 2x125 | Molecular Research DNA Laboratory, Shallowater, Texas | PRJNA417388 | 0.6403 | 0 | M0060 | 37 |
| OP8_C16 | HiSeq | 2x125 | Molecular Research DNA Laboratory, Shallowater, Texas | PRJNA417388 | 0.6619 | 0 | M0060 | 37 |
| OP8_E21 | MiSeq | 2x250 | University of Tennessee, Knoxville, Tennessee | PRJNA417388 | 0.446 | 0.0072 | M0059 | 41 |
| OP8_F13 | MiSeq | 2x250 | University of Tennessee, Knoxville, Tennessee | PRJNA417388 | 0.5468 | 0.0072 | M0059 | 41 |
| OP8_M19 | MiSeq | 2x250 | University of Tennessee, Knoxville, Tennessee | PRJNA417388 | 0.2734 | 0.0072 | M0059 | 41 |
| OP8_M21 | MiSeq | 2x250 | University of Tennessee, Knoxville, Tennessee | PRJNA417388 | 0.4532 | 0 | M0059 | 41 |
| OP8_P22 | HiSeq | 2x125 | Molecular Research DNA Laboratory, Shallowater, Texas | PRJNA417388 | 0.0935 | 0 | M0060 | 84 |
| OPBA10 | MiSeq | 2x250 | University of Tennessee, Knoxville, Tennessee | PRJNA417388 | 0.6763 | 0.0144 | M0060 | 37 |
| OPBB05 | MiSeq | 2x250 | University of Tennessee, Knoxville, Tennessee | PRJNA417388 | 0.4676 | 0.0072 | M0059 | 68 |
| OPBB07 | MiSeq | 2x250 | University of Tennessee, Knoxville, Tennessee | PRJNA417388 | 0.4317 | 0.0144 | M0060 | 37 |
| OPBC09 | MiSeq | 2x250 | University of Tennessee, Knoxville, Tennessee | PRJNA417388 | 0.7266 | 0.0144 | M0060 | 37 |
| OPBI09 | HiSeq | 2x125 | Molecular Research DNA Laboratory, Shallowater, Texas | PRJNA417388 | 0.3453 | 0.0144 | M0060 | 37 |
| OPBM06 | MiSeq | 2x250 | University of Tennessee, Knoxville, Tennessee | PRJNA417388 | 0.0576 | 0 | M0059 | 68 |
| OPBM19 | HiSeq | 2x125 | Molecular Research DNA Laboratory, Shallowater, Texas | PRJNA417388 | 0.3669 | 0 | M0060 | 37 |
| OPBM23 | MiSeq | 2x250 | University of Tennessee, Knoxville, Tennessee | PRJNA417388 | 0.5396 | 0 | M0059 | 68 |
| OPBO21 | MiSeq | 2x250 | University of Tennessee, Knoxville, Tennessee | PRJNA417388 | 0.5252 | 0.0144 | M0059 | 68 |
| OPBO22 | MiSeq | 2x250 | University of Tennessee, Knoxville, Tennessee | PRJNA417388 | 0.4532 | 0 | M0060 | 37 |
| Unk_M15 | MiSeq | 2x250 | University of Tennessee, Knoxville, Tennessee | PRJNA417388 | 0.0647 | 0 | M0060 | 84 |
