# Supplementary material for: Uncultured Microbial Phyla Suggest Mechanisms for Multi-Thousand-Year Subsistence in Baltic Sea Sediments
Source: mBio. 2019 Apr 16;10(2):e02376-18. doi: 10.1128/mBio.02376-18 (PMC6469976; doi:10.1128/mBio.02376-18)
Supplement: TABLE S2 [file mBio.02376-18-st002.docx]

**Supplemental Table S2.** List of enzymes in pathways that are related to metabolites. When multiple enzymatic routes are possible for a single step, this is indicated by adding a digit after the decimal of the step. Only the enzymatic steps that were present in the SAGs are listed.

| **Pathway** | **Annotation** | **Step** |
| --- | --- | --- |
| TCA | Citrate synthase | 1 |
| TCA | Aconitase/Citrate lyase | 2 |
| TCA | Isocitrate dehydrogenase | 3 |
| TCA | Succinyl-CoA synthetase | 5 |
| TCA | Succinate dehydrogenase or arginosuccinate lyase | 6 |
| TCA | Fumarate hydratase | 7 |
| TCA | Malate dehydrogenase | 8 |
| De Novo NAD+ biosynthesis | L-aspartate oxidase | 1 |
| De Novo NAD+ biosynthesis | Quinolinate synthase | 2 |
| De Novo NAD+ biosynthesis | Quinolinate phosphoribosyltransferase | 3 |
| De Novo NAD+ biosynthesis | Nicotinate-nucleotide adenylyltransferase | 4 |
| De Novo NAD+ biosynthesis | NH(3)-dependent NAD(+) synthetase | 5.1 |
| De Novo NAD+ biosynthesis | Glutamine-dependent NAD(+) synthetase | 5.2 |
| Aspartate Superpathway | Aspartate aminotransferase | 1 |
| Aspartate Superpathway | Aspartate kinase | 2 |
| Aspartate Superpathway | Aspartate-semialdehyde dehydrogenase | 3.1 |
| Aspartate Superpathway | Homoserine dehydrogenase | 3.2 |
| Aspartate Superpathway | 4-hydroxy-tetrahydrodipicolinate synthase | 4.1 |
| Aspartate Superpathway | Homoserine O-acetyltransferase | 4.2 |
| Aspartate Superpathway | 4-hydroxy-tetrahydrodipicolinate reductase | 5.1 |
| Aspartate Superpathway | Homoserine kinase | 5.2 |
| Aspartate Superpathway | O-succinylhomoserine(thiol)-lyase | 5.3 |
| Aspartate Superpathway | Tetrahydropicolinate succinylase | 6.1 |
| Aspartate Superpathway | Threonine synthase | 6.2 |
| Aspartate Superpathway | Cystathionine beta-lyase | 6.3 |
| Aspartate Superpathway | Succinyldiaminopimelate aminotransferase | 7.1 |
| Aspartate Superpathway | Methionine synthase | 7.2 |
| Aspartate Superpathway | Succinyl-diaminopimelate desuccinylase | 8.1 |
| Aspartate Superpathway | Methionine adenosyltransferase | 8.2 |
| Aspartate Superpathway | Diaminopimelate epimerase | 9.1 |
| Aspartate Superpathway | Diaminopimelate decarboxylase | 10.1 |
| Serine/Cysteine/Alanine/Glycine | D-3-phosphoglycerate dehydrogenase | 1 |
| Serine/Cysteine/Alanine/Glycine | Phosphoserine aminotransferase | 2 |
| Serine/Cysteine/Alanine/Glycine | Phosphoserine phosphatase | 3 |
| Serine/Cysteine/Alanine/Glycine | Serine acetyltransferase | 4.1 |
| Serine/Cysteine/Alanine/Glycine | Serine hydroxymethyltransferase | 4.2 |
| Serine/Cysteine/Alanine/Glycine | Cysteine synthase | 5 |
| Serine/Cysteine/Alanine/Glycine | Cysteine desulfurase | 6 |
| Branched Amino Acid Transport | High-affinity branched-chain amino acid transport system | 1 |
| Branched Amino Acid Transport | Leucine export protein LeuE | 2 |
| Aromatic Amino Acid Synthesis | 3-deoxy-7-phosphoheptulonate synthase | 1 |
| Aromatic Amino Acid Synthesis | 3-dehydroquinate synthase | 2 |
| Aromatic Amino Acid Synthesis | 3-dehydroquinate dehydratase | 3 |
| Aromatic Amino Acid Synthesis | Shikimate dehydrogenase | 4 |
| Aromatic Amino Acid Synthesis | Shikimate kinase | 5 |
| Aromatic Amino Acid Synthesis | 3-phosphoshikimate 1-carboxyvinyltransferase | 6 |
| Aromatic Amino Acid Synthesis | Chorismate synthase | 7 |
| Aromatic Amino Acid Synthesis | Anthranilate synthase/phosphoribosyltransferase | 8.1 |
| Aromatic Amino Acid Synthesis | Chorismate mutase | 8.2 |
| Aromatic Amino Acid Synthesis | Phosphoribosylanthranilate isomerase/Indole-3-glycerol phosphate synthase | 9.1 |
| Aromatic Amino Acid Synthesis | Prephenate dehydratase | 9.2 |
| Aromatic Amino Acid Synthesis | Prephenate dehydrogenase | 9.3 |
| Aromatic Amino Acid Synthesis | Branched Amino Acid/Tyrosine/Phenylalanine aminotransferase | 10 |
| Aromatic Amino Acid Synthesis | Tryptophan synthase | 13 |
| Allantoin Degradation | Permease for cytosine/purines, uracil, thiamine, allantoin | 0 |
| Allantoin Degradation | Allantoinase | 1 |
| Allantoin Degradation | Oxamate carbamoyltransferase | 4 |
| Allantoin Degradation | Carbamate kinas | 5 |
| Trehalose Metabolism | Trehalose synthase | 1.1 |
| Trehalose Metabolism | Bifunctional trehalose-6-phosphate synthase/phosphatase | 1.2 |
| Trehalose Metabolism | Maltooligosyl trehalose synthase | 1.3 |
| Trehalose Metabolism | Trehalase | 1.4 |
| Trehalose Metabolism | Trehalose-phosphate phosphatase | 2.1 |
| Trehalose Metabolism | Malto-oligosyltrehalose trehalohydrolase | 2.2 |
| Trehalose Metabolism | Bifunctional trehalose-6-phosphate synthase/phosphatase | 3 |
| Glutamine and Glutamate Synthesis | Glutamine synthetase | 1.1 |
| Glutamine and Glutamate Synthesis | Glutamate dehydrogenase | 1.2 |
| Glutamine and Glutamate Synthesis | Glutamate synthase | 1.3 |
| NAD+ Consumption | NAD+-dependent protein deacetylase | 1 |
| NAD+ Salvage | NAD(H) pyrophosphatase | 1 |
| NAD+ Salvage | Nicotinamidase | 3 |
| NAD+ Salvage | Nicotinate phosphoribosyltransferase | 4 |
| NAD+ Salvage | Nicotinate-nucleotide adenylytransferase | 5 |
| NAD+ Salvage | NAD+ synthetase | 6 |
