# Supplementary material for: Uncultured Microbial Phyla Suggest Mechanisms for Multi-Thousand-Year Subsistence in Baltic Sea Sediments
Source: mBio. 2019 Apr 16;10(2):e02376-18. doi: 10.1128/mBio.02376-18 (PMC6469976; doi:10.1128/mBio.02376-18)
Supplement: TABLE S3 [file mBio.02376-18-st003.docx]

**Supplemental Table S3:** Enzyme substrates and nominal corresponding enzymes

| Enzyme | Class | Substrate | EC |
| --- | --- | --- | --- |
| Leucyl aminopeptidase | Exopeptidase | Leucine-AMC | 3.4.11.1 |
| Arginyl aminopeptidase | Exopeptidase | L-arginine-AMC | 3.4.11.6 |
| Prolyl aminopeptidase | Exopeptidase | H-proline-AMC | 3.4.11.5 |
| Ornithyl aminopeptidase | Exopeptidase | Ornithine-AMC | -- |
| Gingipain R. | Endopeptidase | Z-phenylalanine-arginine-AMC | 3.4.22.37 |
| Clostripain | Endopeptidase | Z-phenylalanine-valine-arginine-AMC | 3.4.22.8 |
| β-d-xylosidase | Polysaccharide hydrolase | MUB-β-D-xylopyranoside | 3.2.1.37 |
| β-d-cellobiohydrolase | Polysaccharide hydrolase | MUB-β-D-cellobioside | 3.2.1.91 |
| N-acetyl- β-d- glucosaminidase | Polysaccharide hydrolase | MUB-N-acetyl-β-D-glucosaminide | 3.2.1.52 |
| β-glucosidase | Polysaccharide hydrolase | MUB-β-D-glucopyranoside | 3.2.1.21 |
| α-glucosidase | Polysaccharide hydrolase | MUB-α-D-glucopyranoside | 3.2.1.20 |
| Alkaline phosphatase | Phosphatase | MUB-PO_4_ | 3.1.3.1 |
