# Supplementary material for: Uncultured Microbial Phyla Suggest Mechanisms for Multi-Thousand-Year Subsistence in Baltic Sea Sediments
Source: mBio. 2019 Apr 16;10(2):e02376-18. doi: 10.1128/mBio.02376-18 (PMC6469976; doi:10.1128/mBio.02376-18)
Supplement: DATA SET S1 [file mBio.02376-18-sd001.pdf]

"pID" "gene_callers_id" "sample_id" "mean_transcript_coverage" "PROKKA_Annotation"
"Chl_A19_00001" 1 "s59E13H2" 0 "Integrase core domain protein"
"Chl_A19_00001" 1 "s59E25H2" 0 "Integrase core domain protein"
"Chl_A19_00001" 1 "s59E5H2" 0.0206553666056678 "Integrase core domain protein"
"Chl_A19_00001" 1 "s63E6H2" 0 "Integrase core domain protein"
"Chl_A19_00002" 2 "s59E13H2" 0 "Transposase"
"Chl_A19_00002" 2 "s59E25H2" 0 "Transposase"
"Chl_A19_00002" 2 "s59E5H2" 0.270603385438915 "Transposase"
"Chl_A19_00002" 2 "s63E6H2" 0.0116997647804295 "Transposase"
"Chl_A19_00004" 4 "s59E13H2" 0 "hypothetical protein"
"Chl_A19_00004" 4 "s59E25H2" 0 "hypothetical protein"
"Chl_A19_00004" 4 "s59E5H2" 0 "hypothetical protein"
"Chl_A19_00004" 4 "s63E6H2" 0 "hypothetical protein"
"Chl_A19_00005" 5 "s59E13H2" 0 "Transposase IS116/IS110/IS902 family protein"
"Chl_A19_00005" 5 "s59E25H2" 0 "Transposase IS116/IS110/IS902 family protein"
"Chl_A19_00005" 5 "s59E5H2" 0 "Transposase IS116/IS110/IS902 family protein"
"Chl_A19_00005" 5 "s63E6H2" 0 "Transposase IS116/IS110/IS902 family protein"
"Chl_A19_00006" 6 "s59E13H2" 0 "pyrroloquinoline quinone biosynthesis protein PqqE"
"Chl_A19_00006" 6 "s59E25H2" 0 "pyrroloquinoline quinone biosynthesis protein PqqE"
"Chl_A19_00006" 6 "s59E5H2" 0 "pyrroloquinoline quinone biosynthesis protein PqqE"
"Chl_A19_00006" 6 "s63E6H2" 0 "pyrroloquinoline quinone biosynthesis protein PqqE"
"Chl_A19_00007" 7 "s59E13H2" 0 "hypothetical protein"
"Chl_A19_00007" 7 "s59E25H2" 0 "hypothetical protein"
"Chl_A19_00007" 7 "s59E5H2" 0 "hypothetical protein"
"Chl_A19_00007" 7 "s63E6H2" 0 "hypothetical protein"
"Chl_A19_00008" 8 "s59E13H2" 0 "hypothetical protein"
"Chl_A19_00008" 8 "s59E25H2" 0 "hypothetical protein"
"Chl_A19_00008" 8 "s59E5H2" 0 "hypothetical protein"
"Chl_A19_00008" 8 "s63E6H2" 0 "hypothetical protein"
"Chl_A19_00009" 9 "s59E13H2" 0 "Diacetylchitobiose deacetylase"
"Chl_A19_00009" 9 "s59E25H2" 0 "Diacetylchitobiose deacetylase"
"Chl_A19_00009" 9 "s59E5H2" 0 "Diacetylchitobiose deacetylase"
"Chl_A19_00009" 9 "s63E6H2" 0 "Diacetylchitobiose deacetylase"
"Chl_A19_00010" 10 "s59E13H2" 0 "UDP-N-acetylglucosamine 2-epimerase"
"Chl_A19_00010" 10 "s59E25H2" 0 "UDP-N-acetylglucosamine 2-epimerase"
"Chl_A19_00010" 10 "s59E5H2" 0 "UDP-N-acetylglucosamine 2-epimerase"
"Chl_A19_00010" 10 "s63E6H2" 0 "UDP-N-acetylglucosamine 2-epimerase"
"Chl_A19_00011" 11 "s59E13H2" 0 "carbamoyl phosphate synthase-like protein"
"Chl_A19_00011" 11 "s59E25H2" 0 "carbamoyl phosphate synthase-like protein"
"Chl_A19_00011" 11 "s59E5H2" 0 "carbamoyl phosphate synthase-like protein"
"Chl_A19_00011" 11 "s63E6H2" 0 "carbamoyl phosphate synthase-like protein"
"Chl_A19_00012" 12 "s59E13H2" 0 "N,N'-diacetyllegionaminic acid synthase"
"Chl_A19_00012" 12 "s59E25H2" 0 "N,N'-diacetyllegionaminic acid synthase"
"Chl_A19_00012" 12 "s59E5H2" 0 "N,N'-diacetyllegionaminic acid synthase"
"Chl_A19_00012" 12 "s63E6H2" 0 "N,N'-diacetyllegionaminic acid synthase"
"Chl_A19_00013" 13 "s59E13H2" 0 "3-deoxy-D-manno-octulosonate 8-phosphate phosphatase KdsC"
"Chl_A19_00013" 13 "s59E25H2" 0 "3-deoxy-D-manno-octulosonate 8-phosphate phosphatase KdsC"

"Chl_A19_00013" 13 "s59E5H2" 0 "3-deoxy-D-manno-octulosonate 8-phosphate phosphatase KdsC"
"Chl_A19_00013" 13 "s63E6H2" 0 "3-deoxy-D-manno-octulosonate 8-phosphate phosphatase KdsC"
"Chl_A19_00014" 14 "s59E13H2" 0 "Ribosomal protein S12 methylthiotransferase RimO"
"Chl_A19_00014" 14 "s59E25H2" 0 "Ribosomal protein S12 methylthiotransferase RimO"
"Chl_A19_00014" 14 "s59E5H2" 0 "Ribosomal protein S12 methylthiotransferase RimO"
"Chl_A19_00014" 14 "s63E6H2" 0 "Ribosomal protein S12 methylthiotransferase RimO"
"Chl_A19_00015" 15 "s59E13H2" 0 "hypothetical protein"
"Chl_A19_00015" 15 "s59E25H2" 0 "hypothetical protein"
"Chl_A19_00015" 15 "s59E5H2" 0 "hypothetical protein"
"Chl_A19_00015" 15 "s63E6H2" 0 "hypothetical protein"
"Chl_A19_00016" 16 "s59E13H2" 0 "Macrocin-O-methyltransferase (TylF)"
"Chl_A19_00016" 16 "s59E25H2" 0 "Macrocin-O-methyltransferase (TylF)"
"Chl_A19_00016" 16 "s59E5H2" 0 "Macrocin-O-methyltransferase (TylF)"
"Chl_A19_00016" 16 "s63E6H2" 0 "Macrocin-O-methyltransferase (TylF)"
"Chl_A19_00017" 17 "s59E13H2" 0 "Radical SAM superfamily protein"
"Chl_A19_00017" 17 "s59E25H2" 0 "Radical SAM superfamily protein"
"Chl_A19_00017" 17 "s59E5H2" 0 "Radical SAM superfamily protein"
"Chl_A19_00017" 17 "s63E6H2" 0 "Radical SAM superfamily protein"
"Chl_A19_00018" 18 "s59E13H2" 0 "GDSL-like Lipase/Acylhydrolase"
"Chl_A19_00018" 18 "s59E25H2" 0 "GDSL-like Lipase/Acylhydrolase"
"Chl_A19_00018" 18 "s59E5H2" 0 "GDSL-like Lipase/Acylhydrolase"
"Chl_A19_00018" 18 "s63E6H2" 0 "GDSL-like Lipase/Acylhydrolase"
"Chl_A19_00019" 19 "s59E13H2" 0 "hypothetical protein"
"Chl_A19_00019" 19 "s59E25H2" 0 "hypothetical protein"
"Chl_A19_00019" 19 "s59E5H2" 0 "hypothetical protein"
"Chl_A19_00019" 19 "s63E6H2" 0 "hypothetical protein"
"Chl_A19_00020" 20 "s59E13H2" 0 "hypothetical protein"
"Chl_A19_00020" 20 "s59E25H2" 0 "hypothetical protein"
"Chl_A19_00020" 20 "s59E5H2" 0 "hypothetical protein"
"Chl_A19_00020" 20 "s63E6H2" 0 "hypothetical protein"
"Chl_A19_00021" 21 "s59E13H2" 0 "Decarbamoylnovobiocin carbamoyltransferase"
"Chl_A19_00021" 21 "s59E25H2" 0 "Decarbamoylnovobiocin carbamoyltransferase"
"Chl_A19_00021" 21 "s59E5H2" 0 "Decarbamoylnovobiocin carbamoyltransferase"
"Chl_A19_00021" 21 "s63E6H2" 0 "Decarbamoylnovobiocin carbamoyltransferase"
"Chl_A19_00022" 22 "s59E13H2" 0 "putative methyltransferase YcgJ"
"Chl_A19_00022" 22 "s59E25H2" 0 "putative methyltransferase YcgJ"
"Chl_A19_00022" 22 "s59E5H2" 0 "putative methyltransferase YcgJ"
"Chl_A19_00022" 22 "s63E6H2" 0 "putative methyltransferase YcgJ"
"Chl_A19_00023" 23 "s59E13H2" 0 "Cyclic pyranopterin monophosphate synthase"
"Chl_A19_00023" 23 "s59E25H2" 0 "Cyclic pyranopterin monophosphate synthase"
"Chl_A19_00023" 23 "s59E5H2" 0 "Cyclic pyranopterin monophosphate synthase"
"Chl_A19_00023" 23 "s63E6H2" 0 "Cyclic pyranopterin monophosphate synthase"
"Chl_A19_00024" 24 "s59E13H2" 0 "pyrroloquinoline quinone biosynthesis protein PqqE"
"Chl_A19_00024" 24 "s59E25H2" 0 "pyrroloquinoline quinone biosynthesis protein PqqE"
"Chl_A19_00024" 24 "s59E5H2" 0 "pyrroloquinoline quinone biosynthesis protein PqqE"
"Chl_A19_00024" 24 "s63E6H2" 0 "pyrroloquinoline quinone biosynthesis protein PqqE"
"Chl_A19_00025" 25 "s59E13H2" 0 "CMP-N,N'-diacetyllegionaminic acid synthase"

"Chl_A19_00025" 25 "s59E25H2" 0 "CMP-N,N'-diacetyllegionaminic acid synthase"
"Chl_A19_00025" 25 "s59E5H2" 0 "CMP-N,N'-diacetyllegionaminic acid synthase"
"Chl_A19_00025" 25 "s63E6H2" 0 "CMP-N,N'-diacetyllegionaminic acid synthase"
"Chl_A19_00026" 26 "s59E13H2" 0 "Teichoic acids export ATP-binding protein TagH"
"Chl_A19_00026" 26 "s59E25H2" 0 "Teichoic acids export ATP-binding protein TagH"
"Chl_A19_00026" 26 "s59E5H2" 0 "Teichoic acids export ATP-binding protein TagH"
"Chl_A19_00026" 26 "s63E6H2" 0 "Teichoic acids export ATP-binding protein TagH"
"Chl_A19_00027" 27 "s59E13H2" 0 "ABC-2 type transporter"
"Chl_A19_00027" 27 "s59E25H2" 0 "ABC-2 type transporter"
"Chl_A19_00027" 27 "s59E5H2" 0 "ABC-2 type transporter"
"Chl_A19_00027" 27 "s63E6H2" 0 "ABC-2 type transporter"
"Chl_A19_00028" 28 "s59E13H2" 0 "hypothetical protein"
"Chl_A19_00028" 28 "s59E25H2" 0 "hypothetical protein"
"Chl_A19_00028" 28 "s59E5H2" 0 "hypothetical protein"
"Chl_A19_00028" 28 "s63E6H2" 0 "hypothetical protein"
"Chl_A19_00029" 29 "s59E13H2" 0 "hypothetical protein"
"Chl_A19_00029" 29 "s59E25H2" 0 "hypothetical protein"
"Chl_A19_00029" 29 "s59E5H2" 0 "hypothetical protein"
"Chl_A19_00029" 29 "s63E6H2" 0 "hypothetical protein"
"Chl_A19_00031" 31 "s59E13H2" 0 "hypothetical protein"
"Chl_A19_00031" 31 "s59E25H2" 0 "hypothetical protein"
"Chl_A19_00031" 31 "s59E5H2" 0 "hypothetical protein"
"Chl_A19_00031" 31 "s63E6H2" 0 "hypothetical protein"
"Chl_A19_00034" 34 "s59E13H2" 0 "Pesticidal crystal protein cry11Bb"
"Chl_A19_00034" 34 "s59E25H2" 0 "Pesticidal crystal protein cry11Bb"
"Chl_A19_00034" 34 "s59E5H2" 0 "Pesticidal crystal protein cry11Bb"
"Chl_A19_00034" 34 "s63E6H2" 0 "Pesticidal crystal protein cry11Bb"
"Chl_A19_00037" 37 "s59E13H2" 0 "Acetoin utilization protein AcuC"
"Chl_A19_00037" 37 "s59E25H2" 0 "Acetoin utilization protein AcuC"
"Chl_A19_00037" 37 "s59E5H2" 0 "Acetoin utilization protein AcuC"
"Chl_A19_00037" 37 "s63E6H2" 0 "Acetoin utilization protein AcuC"
"Chl_A19_00039" 39 "s59E13H2" 0 "Chaperone protein DnaJ"
"Chl_A19_00039" 39 "s59E25H2" 0 "Chaperone protein DnaJ"
"Chl_A19_00039" 39 "s59E5H2" 0 "Chaperone protein DnaJ"
"Chl_A19_00039" 39 "s63E6H2" 0 "Chaperone protein DnaJ"
"Chl_A19_00040" 40 "s59E13H2" 0 "hypothetical protein"
"Chl_A19_00040" 40 "s59E25H2" 0 "hypothetical protein"
"Chl_A19_00040" 40 "s59E5H2" 0 "hypothetical protein"
"Chl_A19_00040" 40 "s63E6H2" 0 "hypothetical protein"
"Chl_A19_00041" 41 "s59E13H2" 0 "26 kDa periplasmic immunogenic protein precursor"
"Chl_A19_00041" 41 "s59E25H2" 0 "26 kDa periplasmic immunogenic protein precursor"
"Chl_A19_00041" 41 "s59E5H2" 0 "26 kDa periplasmic immunogenic protein precursor"
"Chl_A19_00041" 41 "s63E6H2" 0 "26 kDa periplasmic immunogenic protein precursor"
"Chl_A19_00042" 42 "s59E13H2" 0 "Macrolide export ATP-binding/permease protein MacB"
"Chl_A19_00042" 42 "s59E25H2" 0 "Macrolide export ATP-binding/permease protein MacB"
"Chl_A19_00042" 42 "s59E5H2" 0.0192752635619859 "Macrolide export ATP-binding/permease protein MacB"
"Chl_A19_00042" 42 "s63E6H2" 0 "Macrolide export ATP-binding/permease protein MacB"

"Chl_A19_00043" 43 "s59E13H2" 0 "putative ABC transporter ATP-binding protein"
"Chl_A19_00043" 43 "s59E25H2" 0 "putative ABC transporter ATP-binding protein"
"Chl_A19_00043" 43 "s59E5H2" 0 "putative ABC transporter ATP-binding protein"
"Chl_A19_00043" 43 "s63E6H2" 0 "putative ABC transporter ATP-binding protein"
"Chl_A19_00044" 44 "s59E13H2" 0 "macrolide transporter subunit MacA"
"Chl_A19_00044" 44 "s59E25H2" 0 "macrolide transporter subunit MacA"
"Chl_A19_00044" 44 "s59E5H2" 0 "macrolide transporter subunit MacA"
"Chl_A19_00044" 44 "s63E6H2" 0 "macrolide transporter subunit MacA"
"Chl_A19_00045" 45 "s59E13H2" 0 "Signal transduction histidine-protein kinase BaeS"
"Chl_A19_00045" 45 "s59E25H2" 0 "Signal transduction histidine-protein kinase BaeS"
"Chl_A19_00045" 45 "s59E5H2" 0 "Signal transduction histidine-protein kinase BaeS"
"Chl_A19_00045" 45 "s63E6H2" 0 "Signal transduction histidine-protein kinase BaeS"
"Chl_A19_00046" 46 "s59E13H2" 0 "Alkaline phosphatase synthesis transcriptional regulatory protein PhoP"
"Chl_A19_00046" 46 "s59E25H2" 0 "Alkaline phosphatase synthesis transcriptional regulatory protein PhoP"
"Chl_A19_00046" 46 "s59E5H2" 0 "Alkaline phosphatase synthesis transcriptional regulatory protein PhoP"
"Chl_A19_00046" 46 "s63E6H2" 0 "Alkaline phosphatase synthesis transcriptional regulatory protein PhoP"
"Chl_A19_00047" 47 "s59E13H2" 0 "hypothetical protein"
"Chl_A19_00047" 47 "s59E25H2" 0 "hypothetical protein"
"Chl_A19_00047" 47 "s59E5H2" 0 "hypothetical protein"
"Chl_A19_00047" 47 "s63E6H2" 0 "hypothetical protein"
"Chl_A19_00049" 49 "s59E13H2" 0 "Putative NAD(P)H nitroreductase"
"Chl_A19_00049" 49 "s59E25H2" 0 "Putative NAD(P)H nitroreductase"
"Chl_A19_00049" 49 "s59E5H2" 0 "Putative NAD(P)H nitroreductase"
"Chl_A19_00049" 49 "s63E6H2" 0 "Putative NAD(P)H nitroreductase"
"Chl_A19_00050" 50 "s59E13H2" 0 "hypothetical protein"
"Chl_A19_00050" 50 "s59E25H2" 0 "hypothetical protein"
"Chl_A19_00050" 50 "s59E5H2" 0 "hypothetical protein"
"Chl_A19_00050" 50 "s63E6H2" 0 "hypothetical protein"
"Chl_A19_00052" 52 "s59E13H2" 0 "hypothetical protein"
"Chl_A19_00052" 52 "s59E25H2" 0 "hypothetical protein"
"Chl_A19_00052" 52 "s59E5H2" 0 "hypothetical protein"
"Chl_A19_00052" 52 "s63E6H2" 0 "hypothetical protein"
"Chl_A19_00053" 53 "s59E13H2" 0 "hypothetical protein"
"Chl_A19_00053" 53 "s59E25H2" 0 "hypothetical protein"
"Chl_A19_00053" 53 "s59E5H2" 0 "hypothetical protein"
"Chl_A19_00053" 53 "s63E6H2" 0 "hypothetical protein"
"Chl_A19_00054" 54 "s59E13H2" 0 "CobQ/CobB/MinD/ParA nucleotide binding domain protein"
"Chl_A19_00054" 54 "s59E25H2" 0 "CobQ/CobB/MinD/ParA nucleotide binding domain protein"
"Chl_A19_00054" 54 "s59E5H2" 0 "CobQ/CobB/MinD/ParA nucleotide binding domain protein"
"Chl_A19_00054" 54 "s63E6H2" 0 "CobQ/CobB/MinD/ParA nucleotide binding domain protein"
"Chl_A19_00055" 55 "s59E13H2" 0 "Bifunctional protein FolD protein"
"Chl_A19_00055" 55 "s59E25H2" 0 "Bifunctional protein FolD protein"
"Chl_A19_00055" 55 "s59E5H2" 0 "Bifunctional protein FolD protein"
"Chl_A19_00055" 55 "s63E6H2" 0 "Bifunctional protein FolD protein"
"Chl_A19_00056" 56 "s59E13H2" 0 "putative enoyl-CoA hydratase echA8"
"Chl_A19_00056" 56 "s59E25H2" 0 "putative enoyl-CoA hydratase echA8"
"Chl_A19_00056" 56 "s59E5H2" 0 "putative enoyl-CoA hydratase echA8"

"Chl_A19_00056" 56 "s63E6H2" 0 "putative enoyl-CoA hydratase echA8"
"Chl_A19_00057" 57 "s59E13H2" 0 "putative enoyl-CoA hydratase echA8"
"Chl_A19_00057" 57 "s59E25H2" 0 "putative enoyl-CoA hydratase echA8"
"Chl_A19_00057" 57 "s59E5H2" 0 "putative enoyl-CoA hydratase echA8"
"Chl_A19_00057" 57 "s63E6H2" 0 "putative enoyl-CoA hydratase echA8"
"Chl_A19_00058" 58 "s59E13H2" 0 "hypothetical protein"
"Chl_A19_00058" 58 "s59E25H2" 0 "hypothetical protein"
"Chl_A19_00058" 58 "s59E5H2" 0 "hypothetical protein"
"Chl_A19_00058" 58 "s63E6H2" 0 "hypothetical protein"
"Chl_A19_00059" 59 "s59E13H2" 0 "bifunctional enoyl-CoA hydratase/phosphate acetyltransferase"
"Chl_A19_00059" 59 "s59E25H2" 0 "bifunctional enoyl-CoA hydratase/phosphate acetyltransferase"
"Chl_A19_00059" 59 "s59E5H2" 0 "bifunctional enoyl-CoA hydratase/phosphate acetyltransferase"
"Chl_A19_00059" 59 "s63E6H2" 0 "bifunctional enoyl-CoA hydratase/phosphate acetyltransferase"
"Chl_A19_00060" 60 "s59E13H2" 0 "putative enoyl-CoA hydratase echA8"
"Chl_A19_00060" 60 "s59E25H2" 0 "putative enoyl-CoA hydratase echA8"
"Chl_A19_00060" 60 "s59E5H2" 0 "putative enoyl-CoA hydratase echA8"
"Chl_A19_00060" 60 "s63E6H2" 0 "putative enoyl-CoA hydratase echA8"
"Chl_A19_00061" 61 "s59E13H2" 0 "putative enoyl-CoA hydratase echA8"
"Chl_A19_00061" 61 "s59E25H2" 0 "putative enoyl-CoA hydratase echA8"
"Chl_A19_00061" 61 "s59E5H2" 0 "putative enoyl-CoA hydratase echA8"
"Chl_A19_00061" 61 "s63E6H2" 0 "putative enoyl-CoA hydratase echA8"
"Chl_A19_00062" 62 "s59E13H2" 0 "3-hydroxypropionyl-coenzyme A dehydratase"
"Chl_A19_00062" 62 "s59E25H2" 0 "3-hydroxypropionyl-coenzyme A dehydratase"
"Chl_A19_00062" 62 "s59E5H2" 0 "3-hydroxypropionyl-coenzyme A dehydratase"
"Chl_A19_00062" 62 "s63E6H2" 0 "3-hydroxypropionyl-coenzyme A dehydratase"
"Chl_A19_00063" 63 "s59E13H2" 0 "Glyoxalase-like domain protein"
"Chl_A19_00063" 63 "s59E25H2" 0 "Glyoxalase-like domain protein"
"Chl_A19_00063" 63 "s59E5H2" 0 "Glyoxalase-like domain protein"
"Chl_A19_00063" 63 "s63E6H2" 0 "Glyoxalase-like domain protein"
"Chl_A19_00064" 64 "s59E13H2" 0 "Acryloyl-CoA reductase electron transfer subunit gamma"
"Chl_A19_00064" 64 "s59E25H2" 0 "Acryloyl-CoA reductase electron transfer subunit gamma"
"Chl_A19_00064" 64 "s59E5H2" 0 "Acryloyl-CoA reductase electron transfer subunit gamma"
"Chl_A19_00064" 64 "s63E6H2" 0 "Acryloyl-CoA reductase electron transfer subunit gamma"
"Chl_A19_00065" 65 "s59E13H2" 0 "D-glycero-alpha-D-manno-heptose 1-phosphate guanylyltransferase"
"Chl_A19_00065" 65 "s59E25H2" 0 "D-glycero-alpha-D-manno-heptose 1-phosphate guanylyltransferase"
"Chl_A19_00065" 65 "s59E5H2" 0 "D-glycero-alpha-D-manno-heptose 1-phosphate guanylyltransferase"
"Chl_A19_00065" 65 "s63E6H2" 0 "D-glycero-alpha-D-manno-heptose 1-phosphate guanylyltransferase"
"Chl_A19_00066" 66 "s59E13H2" 0 "hypothetical protein"
"Chl_A19_00066" 66 "s59E25H2" 0 "hypothetical protein"
"Chl_A19_00066" 66 "s59E5H2" 0 "hypothetical protein"
"Chl_A19_00066" 66 "s63E6H2" 0 "hypothetical protein"
"Chl_A19_00068" 68 "s59E13H2" 0 "hypothetical protein"
"Chl_A19_00068" 68 "s59E25H2" 0 "hypothetical protein"
"Chl_A19_00068" 68 "s59E5H2" 0 "hypothetical protein"
"Chl_A19_00068" 68 "s63E6H2" 0 "hypothetical protein"
"Chl_A19_00069" 69 "s59E13H2" 0 "ATP-dependent DNA helicase RecG"
"Chl_A19_00069" 69 "s59E25H2" 0 "ATP-dependent DNA helicase RecG"

"Chl_A19_00069" 69 "s59E5H2" 0 "ATP-dependent DNA helicase RecG"
"Chl_A19_00069" 69 "s63E6H2" 0 "ATP-dependent DNA helicase RecG"
"Chl_A19_00070" 70 "s59E13H2" 0 "DegV domain-containing protein"
"Chl_A19_00070" 70 "s59E25H2" 0 "DegV domain-containing protein"
"Chl_A19_00070" 70 "s59E5H2" 0 "DegV domain-containing protein"
"Chl_A19_00070" 70 "s63E6H2" 0 "DegV domain-containing protein"
"Chl_A19_00071" 71 "s59E13H2" 0 "DegV domain-containing protein"
"Chl_A19_00071" 71 "s59E25H2" 0 "DegV domain-containing protein"
"Chl_A19_00071" 71 "s59E5H2" 0 "DegV domain-containing protein"
"Chl_A19_00071" 71 "s63E6H2" 0 "DegV domain-containing protein"
"Chl_A19_00072" 72 "s59E13H2" 0 "DAK2 domain protein"
"Chl_A19_00072" 72 "s59E25H2" 0 "DAK2 domain protein"
"Chl_A19_00072" 72 "s59E5H2" 0 "DAK2 domain protein"
"Chl_A19_00072" 72 "s63E6H2" 0 "DAK2 domain protein"
"Chl_A19_00073" 73 "s59E13H2" 0 "Corrinoid/iron-sulfur protein small subunit"
"Chl_A19_00073" 73 "s59E25H2" 0 "Corrinoid/iron-sulfur protein small subunit"
"Chl_A19_00073" 73 "s59E5H2" 0 "Corrinoid/iron-sulfur protein small subunit"
"Chl_A19_00073" 73 "s63E6H2" 0.00270648596593473 "Corrinoid/iron-sulfur protein small subunit"
"Chl_A19_00074" 74 "s59E13H2" 0 "Helix-turn-helix"
"Chl_A19_00074" 74 "s59E25H2" 0 "Helix-turn-helix"
"Chl_A19_00074" 74 "s59E5H2" 0 "Helix-turn-helix"
"Chl_A19_00074" 74 "s63E6H2" 0 "Helix-turn-helix"
"Chl_A19_00075" 75 "s59E13H2" 0 "Transposase DDE domain protein"
"Chl_A19_00075" 75 "s59E25H2" 0 "Transposase DDE domain protein"
"Chl_A19_00075" 75 "s59E5H2" 0 "Transposase DDE domain protein"
"Chl_A19_00075" 75 "s63E6H2" 0 "Transposase DDE domain protein"
"Chl_A19_00076" 76 "s59E13H2" 0 "hypothetical protein"
"Chl_A19_00076" 76 "s59E25H2" 0 "hypothetical protein"
"Chl_A19_00076" 76 "s59E5H2" 0 "hypothetical protein"
"Chl_A19_00076" 76 "s63E6H2" 0 "hypothetical protein"
"Chl_A19_00078" 78 "s59E13H2" 0 "Phosphoribosylglycinamide formyltransferase"
"Chl_A19_00078" 78 "s59E25H2" 0 "Phosphoribosylglycinamide formyltransferase"
"Chl_A19_00078" 78 "s59E5H2" 0 "Phosphoribosylglycinamide formyltransferase"
"Chl_A19_00078" 78 "s63E6H2" 0 "Phosphoribosylglycinamide formyltransferase"
"Chl_A19_00079" 79 "s59E13H2" 0 "Phosphoribosylformylglycinamidine synthase"
"Chl_A19_00079" 79 "s59E25H2" 0 "Phosphoribosylformylglycinamidine synthase"
"Chl_A19_00079" 79 "s59E5H2" 0 "Phosphoribosylformylglycinamidine synthase"
"Chl_A19_00079" 79 "s63E6H2" 0 "Phosphoribosylformylglycinamidine synthase"
"Chl_A19_00080" 80 "s59E13H2" 0 "Glyoxylate reductase"
"Chl_A19_00080" 80 "s59E25H2" 0 "Glyoxylate reductase"
"Chl_A19_00080" 80 "s59E5H2" 0 "Glyoxylate reductase"
"Chl_A19_00080" 80 "s63E6H2" 0 "Glyoxylate reductase"
"Chl_A19_00081" 81 "s59E13H2" 0 "Acetophenone carboxylase gamma subunit"
"Chl_A19_00081" 81 "s59E25H2" 0 "Acetophenone carboxylase gamma subunit"
"Chl_A19_00081" 81 "s59E5H2" 0 "Acetophenone carboxylase gamma subunit"
"Chl_A19_00081" 81 "s63E6H2" 0 "Acetophenone carboxylase gamma subunit"
"Chl_A19_00082" 82 "s59E13H2" 0 "Acetophenone carboxylase delta subunit"

"Chl_A19_00082" 82 "s59E25H2" 0 "Acetophenone carboxylase delta subunit"
"Chl_A19_00082" 82 "s59E5H2" 0 "Acetophenone carboxylase delta subunit"
"Chl_A19_00082" 82 "s63E6H2" 0 "Acetophenone carboxylase delta subunit"
"Chl_A19_00083" 83 "s59E13H2" 0 "Histidinol-phosphate aminotransferase 2"
"Chl_A19_00083" 83 "s59E25H2" 0 "Histidinol-phosphate aminotransferase 2"
"Chl_A19_00083" 83 "s59E5H2" 0 "Histidinol-phosphate aminotransferase 2"
"Chl_A19_00083" 83 "s63E6H2" 0 "Histidinol-phosphate aminotransferase 2"
"Chl_A19_00085" 85 "s59E13H2" 0 "hypothetical protein"
"Chl_A19_00085" 85 "s59E25H2" 0 "hypothetical protein"
"Chl_A19_00085" 85 "s59E5H2" 0 "hypothetical protein"
"Chl_A19_00085" 85 "s63E6H2" 0 "hypothetical protein"
"Chl_A19_00086" 86 "s59E13H2" 0 "hypothetical protein"
"Chl_A19_00086" 86 "s59E25H2" 0 "hypothetical protein"
"Chl_A19_00086" 86 "s59E5H2" 0 "hypothetical protein"
"Chl_A19_00086" 86 "s63E6H2" 0 "hypothetical protein"
"Chl_A19_00087" 87 "s59E13H2" 0 "Bifunctional protein GlmU"
"Chl_A19_00087" 87 "s59E25H2" 0 "Bifunctional protein GlmU"
"Chl_A19_00087" 87 "s59E5H2" 0 "Bifunctional protein GlmU"
"Chl_A19_00087" 87 "s63E6H2" 0.000778229916531103 "Bifunctional protein GlmU"
"Chl_A19_00088" 88 "s59E13H2" 0 "Bifunctional protein GlmU"
"Chl_A19_00088" 88 "s59E25H2" 0 "Bifunctional protein GlmU"
"Chl_A19_00088" 88 "s59E5H2" 0 "Bifunctional protein GlmU"
"Chl_A19_00088" 88 "s63E6H2" 0 "Bifunctional protein GlmU"
"Chl_A19_00089" 89 "s59E13H2" 0 "Glutamine--fructose-6-phosphate aminotransferase [isomerizing]"
"Chl_A19_00089" 89 "s59E25H2" 0 "Glutamine--fructose-6-phosphate aminotransferase [isomerizing]"
"Chl_A19_00089" 89 "s59E5H2" 0 "Glutamine--fructose-6-phosphate aminotransferase [isomerizing]"
"Chl_A19_00089" 89 "s63E6H2" 0 "Glutamine--fructose-6-phosphate aminotransferase [isomerizing]"
"Chl_A19_00090" 90 "s59E13H2" 0 "Light-independent protochlorophyllide reductase iron-sulfur ATP-binding protein"
"Chl_A19_00090" 90 "s59E25H2" 0 "Light-independent protochlorophyllide reductase iron-sulfur ATP-binding protein"
"Chl_A19_00090" 90 "s59E5H2" 0 "Light-independent protochlorophyllide reductase iron-sulfur ATP-binding protein"
"Chl_A19_00090" 90 "s63E6H2" 0 "Light-independent protochlorophyllide reductase iron-sulfur ATP-binding protein"
"Chl_A19_00091" 91 "s59E13H2" 0 "Formate--tetrahydrofolate ligase"
"Chl_A19_00091" 91 "s59E25H2" 0 "Formate--tetrahydrofolate ligase"
"Chl_A19_00091" 91 "s59E5H2" 0 "Formate--tetrahydrofolate ligase"
"Chl_A19_00091" 91 "s63E6H2" 0 "Formate--tetrahydrofolate ligase"
"Chl_A19_00093" 93 "s59E13H2" 0 "Tryptophan synthase beta chain 1"
"Chl_A19_00093" 93 "s59E25H2" 0 "Tryptophan synthase beta chain 1"
"Chl_A19_00093" 93 "s59E5H2" 0 "Tryptophan synthase beta chain 1"
"Chl_A19_00093" 93 "s63E6H2" 0 "Tryptophan synthase beta chain 1"
"Chl_A19_00094" 94 "s59E13H2" 0 "Putative serine protease HhoB precursor"
"Chl_A19_00094" 94 "s59E25H2" 0 "Putative serine protease HhoB precursor"
"Chl_A19_00094" 94 "s59E5H2" 0 "Putative serine protease HhoB precursor"
"Chl_A19_00094" 94 "s63E6H2" 0 "Putative serine protease HhoB precursor"
"Chl_A19_00095" 95 "s59E13H2" 0 "Isoleucine--tRNA ligase"
"Chl_A19_00095" 95 "s59E25H2" 0 "Isoleucine--tRNA ligase"
"Chl_A19_00095" 95 "s59E5H2" 0 "Isoleucine--tRNA ligase"
"Chl_A19_00095" 95 "s63E6H2" 0 "Isoleucine--tRNA ligase"

"Chl_A19_00096" 96 "s59E13H2" 0 "DNA repair and recombination protein RadA"
"Chl_A19_00096" 96 "s59E25H2" 0 "DNA repair and recombination protein RadA"
"Chl_A19_00096" 96 "s59E5H2" 0 "DNA repair and recombination protein RadA"
"Chl_A19_00096" 96 "s63E6H2" 0 "DNA repair and recombination protein RadA"
"Chl_A19_00097" 97 "s59E13H2" 0 "Regulatory protein RecX"
"Chl_A19_00097" 97 "s59E25H2" 0 "Regulatory protein RecX"
"Chl_A19_00097" 97 "s59E5H2" 0 "Regulatory protein RecX"
"Chl_A19_00097" 97 "s63E6H2" 0 "Regulatory protein RecX"
"Chl_A19_00098" 98 "s59E13H2" 0 "Ribonuclease Y"
"Chl_A19_00098" 98 "s59E25H2" 0 "Ribonuclease Y"
"Chl_A19_00098" 98 "s59E5H2" 0 "Ribonuclease Y"
"Chl_A19_00098" 98 "s63E6H2" 0.001738068485263 "Ribonuclease Y"
"Chl_A19_00099" 99 "s59E13H2" 0 "hypothetical protein"
"Chl_A19_00099" 99 "s59E25H2" 0 "hypothetical protein"
"Chl_A19_00099" 99 "s59E5H2" 0 "hypothetical protein"
"Chl_A19_00099" 99 "s63E6H2" 0 "hypothetical protein"
"Chl_A19_00100" 100 "s59E13H2" 0 "hypothetical protein"
"Chl_A19_00100" 100 "s59E25H2" 0 "hypothetical protein"
"Chl_A19_00100" 100 "s59E5H2" 0 "hypothetical protein"
"Chl_A19_00100" 100 "s63E6H2" 0 "hypothetical protein"
"Chl_A19_00101" 101 "s59E13H2" 0 "putative glycerol-3-phosphate acyltransferase"
"Chl_A19_00101" 101 "s59E25H2" 0 "putative glycerol-3-phosphate acyltransferase"
"Chl_A19_00101" 101 "s59E5H2" 0 "putative glycerol-3-phosphate acyltransferase"
"Chl_A19_00101" 101 "s63E6H2" 0 "putative glycerol-3-phosphate acyltransferase"
"Chl_A19_00102" 102 "s59E13H2" 0 "hypothetical protein"
"Chl_A19_00102" 102 "s59E25H2" 0 "hypothetical protein"
"Chl_A19_00102" 102 "s59E5H2" 0 "hypothetical protein"
"Chl_A19_00102" 102 "s63E6H2" 0 "hypothetical protein"
"Chl_A19_00103" 103 "s59E13H2" 0 "putative acetyl-CoA acyltransferase"
"Chl_A19_00103" 103 "s59E25H2" 0 "putative acetyl-CoA acyltransferase"
"Chl_A19_00103" 103 "s59E5H2" 0 "putative acetyl-CoA acyltransferase"
"Chl_A19_00103" 103 "s63E6H2" 0 "putative acetyl-CoA acyltransferase"
"Chl_A19_00104" 104 "s59E13H2" 0 "hypothetical protein"
"Chl_A19_00104" 104 "s59E25H2" 0 "hypothetical protein"
"Chl_A19_00104" 104 "s59E5H2" 0 "hypothetical protein"
"Chl_A19_00104" 104 "s63E6H2" 0 "hypothetical protein"
"Chl_A19_00105" 105 "s59E13H2" 0 "R-phenyllactate dehydratase subunit alpha precursor"
"Chl_A19_00105" 105 "s59E25H2" 0 "R-phenyllactate dehydratase subunit alpha precursor"
"Chl_A19_00105" 105 "s59E5H2" 0 "R-phenyllactate dehydratase subunit alpha precursor"
"Chl_A19_00105" 105 "s63E6H2" 0 "R-phenyllactate dehydratase subunit alpha precursor"
"Chl_A19_00106" 106 "s59E13H2" 0 "Benzoyl-CoA reductase subunit C"
"Chl_A19_00106" 106 "s59E25H2" 0 "Benzoyl-CoA reductase subunit C"
"Chl_A19_00106" 106 "s59E5H2" 0 "Benzoyl-CoA reductase subunit C"
"Chl_A19_00106" 106 "s63E6H2" 0 "Benzoyl-CoA reductase subunit C"
"Chl_A19_00107" 107 "s59E13H2" 0 "R-phenyllactate dehydratase activator"
"Chl_A19_00107" 107 "s59E25H2" 0 "R-phenyllactate dehydratase activator"
"Chl_A19_00107" 107 "s59E5H2" 0 "R-phenyllactate dehydratase activator"

"Chl_A19_00107" 107 "s63E6H2" 0 "R-phenyllactate dehydratase activator"
"Chl_A19_00108" 108 "s59E13H2" 0 "Activator of (R)-2-hydroxyglutaryl-CoA dehydratase"
"Chl_A19_00108" 108 "s59E25H2" 0 "Activator of (R)-2-hydroxyglutaryl-CoA dehydratase"
"Chl_A19_00108" 108 "s59E5H2" 0 "Activator of (R)-2-hydroxyglutaryl-CoA dehydratase"
"Chl_A19_00108" 108 "s63E6H2" 0 "Activator of (R)-2-hydroxyglutaryl-CoA dehydratase"
"Chl_A19_00109" 109 "s59E13H2" 0 "(R)-specific enoyl-CoA hydratase"
"Chl_A19_00109" 109 "s59E25H2" 0 "(R)-specific enoyl-CoA hydratase"
"Chl_A19_00109" 109 "s59E5H2" 0 "(R)-specific enoyl-CoA hydratase"
"Chl_A19_00109" 109 "s63E6H2" 0 "(R)-specific enoyl-CoA hydratase"
"Chl_A19_00110" 110 "s59E13H2" 0 "Methylmalonyl-CoA mutase"
"Chl_A19_00110" 110 "s59E25H2" 0 "Methylmalonyl-CoA mutase"
"Chl_A19_00110" 110 "s59E5H2" 0 "Methylmalonyl-CoA mutase"
"Chl_A19_00110" 110 "s63E6H2" 0 "Methylmalonyl-CoA mutase"
"Chl_A19_00111" 111 "s59E13H2" 0 "Methylmalonyl-CoA mutase large subunit"
"Chl_A19_00111" 111 "s59E25H2" 0 "Methylmalonyl-CoA mutase large subunit"
"Chl_A19_00111" 111 "s59E5H2" 0 "Methylmalonyl-CoA mutase large subunit"
"Chl_A19_00111" 111 "s63E6H2" 0 "Methylmalonyl-CoA mutase large subunit"
"Chl_A19_00112" 112 "s59E13H2" 0 "Formyl-coenzyme A transferase"
"Chl_A19_00112" 112 "s59E25H2" 0 "Formyl-coenzyme A transferase"
"Chl_A19_00112" 112 "s59E5H2" 0 "Formyl-coenzyme A transferase"
"Chl_A19_00112" 112 "s63E6H2" 0 "Formyl-coenzyme A transferase"
"Chl_A19_00113" 113 "s59E13H2" 0 "AsnC family protein"
"Chl_A19_00113" 113 "s59E25H2" 0 "AsnC family protein"
"Chl_A19_00113" 113 "s59E5H2" 0 "AsnC family protein"
"Chl_A19_00113" 113 "s63E6H2" 0 "AsnC family protein"
"Chl_A19_00114" 114 "s59E13H2" 0 "Glutaryl-CoA dehydrogenase"
"Chl_A19_00114" 114 "s59E25H2" 0 "Glutaryl-CoA dehydrogenase"
"Chl_A19_00114" 114 "s59E5H2" 0 "Glutaryl-CoA dehydrogenase"
"Chl_A19_00114" 114 "s63E6H2" 0 "Glutaryl-CoA dehydrogenase"
"Chl_A19_00115" 115 "s59E13H2" 0 "Acyl-CoA dehydrogenase"
"Chl_A19_00115" 115 "s59E25H2" 0 "Acyl-CoA dehydrogenase"
"Chl_A19_00115" 115 "s59E5H2" 0 "Acyl-CoA dehydrogenase"
"Chl_A19_00115" 115 "s63E6H2" 0 "Acyl-CoA dehydrogenase"
"Chl_A19_00116" 116 "s59E13H2" 0 "4-hydroxy-3-methylbut-2-enyl diphosphate reductase"
"Chl_A19_00116" 116 "s59E25H2" 0 "4-hydroxy-3-methylbut-2-enyl diphosphate reductase"
"Chl_A19_00116" 116 "s59E5H2" 0 "4-hydroxy-3-methylbut-2-enyl diphosphate reductase"
"Chl_A19_00116" 116 "s63E6H2" 0 "4-hydroxy-3-methylbut-2-enyl diphosphate reductase"
"Chl_A19_00117" 117 "s59E13H2" 0 "1-acyl-sn-glycerol-3-phosphate acyltransferase"
"Chl_A19_00117" 117 "s59E25H2" 0 "1-acyl-sn-glycerol-3-phosphate acyltransferase"
"Chl_A19_00117" 117 "s59E5H2" 0 "1-acyl-sn-glycerol-3-phosphate acyltransferase"
"Chl_A19_00117" 117 "s63E6H2" 0 "1-acyl-sn-glycerol-3-phosphate acyltransferase"
"Chl_A19_00118" 118 "s59E13H2" 0 "Protease 4"
"Chl_A19_00118" 118 "s59E25H2" 0 "Protease 4"
"Chl_A19_00118" 118 "s59E5H2" 0 "Protease 4"
"Chl_A19_00118" 118 "s63E6H2" 0 "Protease 4"
"Chl_A19_00119" 119 "s59E13H2" 0 "hypothetical protein"
"Chl_A19_00119" 119 "s59E25H2" 0 "hypothetical protein"

"Chl_A19_00119" 119 "s59E5H2" 0 "hypothetical protein"
"Chl_A19_00119" 119 "s63E6H2" 0 "hypothetical protein"
"Chl_A19_00120" 120 "s59E13H2" 0 "Sialic acid TRAP transporter permease protein SiaT"
"Chl_A19_00120" 120 "s59E25H2" 0 "Sialic acid TRAP transporter permease protein SiaT"
"Chl_A19_00120" 120 "s59E5H2" 0 "Sialic acid TRAP transporter permease protein SiaT"
"Chl_A19_00120" 120 "s63E6H2" 0 "Sialic acid TRAP transporter permease protein SiaT"
"Chl_A19_00121" 121 "s59E13H2" 0 "Tripartite ATP-independent periplasmic transporters, DctQ component"
"Chl_A19_00121" 121 "s59E25H2" 0 "Tripartite ATP-independent periplasmic transporters, DctQ component"
"Chl_A19_00121" 121 "s59E5H2" 0 "Tripartite ATP-independent periplasmic transporters, DctQ component"
"Chl_A19_00121" 121 "s63E6H2" 0 "Tripartite ATP-independent periplasmic transporters, DctQ component"
"Chl_A19_00122" 122 "s59E13H2" 0 "Sialic acid-binding periplasmic protein SiaP precursor"
"Chl_A19_00122" 122 "s59E25H2" 0 "Sialic acid-binding periplasmic protein SiaP precursor"
"Chl_A19_00122" 122 "s59E5H2" 0 "Sialic acid-binding periplasmic protein SiaP precursor"
"Chl_A19_00122" 122 "s63E6H2" 0 "Sialic acid-binding periplasmic protein SiaP precursor"
"Chl_A19_00123" 123 "s59E13H2" 0 "Succinyl-CoA:(R)-benzylsuccinate CoA-transferase subunit BbsE"
"Chl_A19_00123" 123 "s59E25H2" 0 "Succinyl-CoA:(R)-benzylsuccinate CoA-transferase subunit BbsE"
"Chl_A19_00123" 123 "s59E5H2" 0 "Succinyl-CoA:(R)-benzylsuccinate CoA-transferase subunit BbsE"
"Chl_A19_00123" 123 "s63E6H2" 0 "Succinyl-CoA:(R)-benzylsuccinate CoA-transferase subunit BbsE"
"Chl_A19_00124" 124 "s59E13H2" 0 "Succinyl-CoA:(R)-benzylsuccinate CoA-transferase subunit BbsF"
"Chl_A19_00124" 124 "s59E25H2" 0 "Succinyl-CoA:(R)-benzylsuccinate CoA-transferase subunit BbsF"
"Chl_A19_00124" 124 "s59E5H2" 0 "Succinyl-CoA:(R)-benzylsuccinate CoA-transferase subunit BbsF"
"Chl_A19_00124" 124 "s63E6H2" 0 "Succinyl-CoA:(R)-benzylsuccinate CoA-transferase subunit BbsF"
"Chl_A19_00125" 125 "s59E13H2" 0 "NADH oxidase"
"Chl_A19_00125" 125 "s59E25H2" 0 "NADH oxidase"
"Chl_A19_00125" 125 "s59E5H2" 0 "NADH oxidase"
"Chl_A19_00125" 125 "s63E6H2" 0 "NADH oxidase"
"Chl_A19_00126" 126 "s59E13H2" 0 "NADH oxidase"
"Chl_A19_00126" 126 "s59E25H2" 0 "NADH oxidase"
"Chl_A19_00126" 126 "s59E5H2" 0 "NADH oxidase"
"Chl_A19_00126" 126 "s63E6H2" 0 "NADH oxidase"
"Chl_A19_00127" 127 "s59E13H2" 0 "Energy-coupling factor transporter ATP-binding protein EcfA2"
"Chl_A19_00127" 127 "s59E25H2" 0 "Energy-coupling factor transporter ATP-binding protein EcfA2"
"Chl_A19_00127" 127 "s59E5H2" 0 "Energy-coupling factor transporter ATP-binding protein EcfA2"
"Chl_A19_00127" 127 "s63E6H2" 0 "Energy-coupling factor transporter ATP-binding protein EcfA2"
"Chl_A19_00128" 128 "s59E13H2" 0 "ABC-2 family transporter protein"
"Chl_A19_00128" 128 "s59E25H2" 0 "ABC-2 family transporter protein"
"Chl_A19_00128" 128 "s59E5H2" 0 "ABC-2 family transporter protein"
"Chl_A19_00128" 128 "s63E6H2" 0 "ABC-2 family transporter protein"
"Chl_A19_00129" 129 "s59E13H2" 0 "hypothetical protein"
"Chl_A19_00129" 129 "s59E25H2" 0 "hypothetical protein"
"Chl_A19_00129" 129 "s59E5H2" 0 "hypothetical protein"
"Chl_A19_00129" 129 "s63E6H2" 0 "hypothetical protein"
"Chl_A19_00130" 130 "s59E13H2" 0 "Putative phosphatase"
"Chl_A19_00130" 130 "s59E25H2" 0 "Putative phosphatase"
"Chl_A19_00130" 130 "s59E5H2" 0 "Putative phosphatase"
"Chl_A19_00130" 130 "s63E6H2" 0 "Putative phosphatase"
"Chl_A19_00132" 132 "s59E13H2" 0 "hypothetical protein"

"Chl_A19_00132" 132 "s59E25H2" 0 "hypothetical protein"
"Chl_A19_00132" 132 "s59E5H2" 0 "hypothetical protein"
"Chl_A19_00132" 132 "s63E6H2" 0 "hypothetical protein"
"Chl_A19_00133" 133 "s59E13H2" 0 "Tyrosine recombinase XerD"
"Chl_A19_00133" 133 "s59E25H2" 0 "Tyrosine recombinase XerD"
"Chl_A19_00133" 133 "s59E5H2" 0 "Tyrosine recombinase XerD"
"Chl_A19_00133" 133 "s63E6H2" 0 "Tyrosine recombinase XerD"
"Chl_A19_00134" 134 "s59E13H2" 0 "Transposase"
"Chl_A19_00134" 134 "s59E25H2" 0 "Transposase"
"Chl_A19_00134" 134 "s59E5H2" 0 "Transposase"
"Chl_A19_00134" 134 "s63E6H2" 0 "Transposase"
"Chl_A19_00135" 135 "s59E13H2" 0 "IS2 transposase TnpB"
"Chl_A19_00135" 135 "s59E25H2" 0 "IS2 transposase TnpB"
"Chl_A19_00135" 135 "s59E5H2" 0 "IS2 transposase TnpB"
"Chl_A19_00135" 135 "s63E6H2" 0 "IS2 transposase TnpB"
"Chl_A19_00136" 136 "s59E13H2" 0 "hypothetical protein"
"Chl_A19_00136" 136 "s59E25H2" 0 "hypothetical protein"
"Chl_A19_00136" 136 "s59E5H2" 0 "hypothetical protein"
"Chl_A19_00136" 136 "s63E6H2" 0 "hypothetical protein"
"Chl_A19_00137" 137 "s59E13H2" 0 "hypothetical protein"
"Chl_A19_00137" 137 "s59E25H2" 0 "hypothetical protein"
"Chl_A19_00137" 137 "s59E5H2" 0 "hypothetical protein"
"Chl_A19_00137" 137 "s63E6H2" 0 "hypothetical protein"
"Chl_A19_00138" 138 "s59E13H2" 0 "hypothetical protein"
"Chl_A19_00138" 138 "s59E25H2" 0 "hypothetical protein"
"Chl_A19_00138" 138 "s59E5H2" 0.00508182345981144 "hypothetical protein"
"Chl_A19_00138" 138 "s63E6H2" 0 "hypothetical protein"
"Chl_A19_00141" 141 "s59E13H2" 0 "hypothetical protein"
"Chl_A19_00141" 141 "s59E25H2" 0 "hypothetical protein"
"Chl_A19_00141" 141 "s59E5H2" 0 "hypothetical protein"
"Chl_A19_00141" 141 "s63E6H2" 0 "hypothetical protein"
"Chl_A19_00142" 142 "s59E13H2" 0 "hypothetical protein"
"Chl_A19_00142" 142 "s59E25H2" 0 "hypothetical protein"
"Chl_A19_00142" 142 "s59E5H2" 0 "hypothetical protein"
"Chl_A19_00142" 142 "s63E6H2" 0 "hypothetical protein"
"Chl_A19_00143" 143 "s59E13H2" 0 "DNA methylase"
"Chl_A19_00143" 143 "s59E25H2" 0 "DNA methylase"
"Chl_A19_00143" 143 "s59E5H2" 0 "DNA methylase"
"Chl_A19_00143" 143 "s63E6H2" 0 "DNA methylase"
"Chl_A19_00144" 144 "s59E13H2" 0 "hypothetical protein"
"Chl_A19_00144" 144 "s59E25H2" 0 "hypothetical protein"
"Chl_A19_00144" 144 "s59E5H2" 0 "hypothetical protein"
"Chl_A19_00144" 144 "s63E6H2" 0 "hypothetical protein"
"Chl_A19_00145" 145 "s59E13H2" 0 "N-6 DNA Methylase"
"Chl_A19_00145" 145 "s59E25H2" 0 "N-6 DNA Methylase"
"Chl_A19_00145" 145 "s59E5H2" 0 "N-6 DNA Methylase"
"Chl_A19_00145" 145 "s63E6H2" 0 "N-6 DNA Methylase"

"Chl_A19_00146" 146 "s59E13H2" 0 "hypothetical protein"
"Chl_A19_00146" 146 "s59E25H2" 0 "hypothetical protein"
"Chl_A19_00146" 146 "s59E5H2" 0 "hypothetical protein"
"Chl_A19_00146" 146 "s63E6H2" 0 "hypothetical protein"
"Chl_A19_00147" 147 "s59E13H2" 0 "hypothetical protein"
"Chl_A19_00147" 147 "s59E25H2" 0 "hypothetical protein"
"Chl_A19_00147" 147 "s59E5H2" 0 "hypothetical protein"
"Chl_A19_00147" 147 "s63E6H2" 0 "hypothetical protein"
"Chl_A19_00149" 149 "s59E13H2" 0 "hypothetical protein"
"Chl_A19_00149" 149 "s59E25H2" 0 "hypothetical protein"
"Chl_A19_00149" 149 "s59E5H2" 0 "hypothetical protein"
"Chl_A19_00149" 149 "s63E6H2" 0 "hypothetical protein"
"Chl_A19_00151" 151 "s59E13H2" 0 "Putative ribosome biogenesis GTPase RsgA"
"Chl_A19_00151" 151 "s59E25H2" 0 "Putative ribosome biogenesis GTPase RsgA"
"Chl_A19_00151" 151 "s59E5H2" 0 "Putative ribosome biogenesis GTPase RsgA"
"Chl_A19_00151" 151 "s63E6H2" 0 "Putative ribosome biogenesis GTPase RsgA"
"Chl_A19_00153" 153 "s59E13H2" 0 "Lipoprotein-releasing system transmembrane protein LolE"
"Chl_A19_00153" 153 "s59E25H2" 0 "Lipoprotein-releasing system transmembrane protein LolE"
"Chl_A19_00153" 153 "s59E5H2" 0 "Lipoprotein-releasing system transmembrane protein LolE"
"Chl_A19_00153" 153 "s63E6H2" 0 "Lipoprotein-releasing system transmembrane protein LolE"
"Chl_A19_00154" 154 "s59E13H2" 0 "Lipoprotein-releasing system ATP-binding protein LolD"
"Chl_A19_00154" 154 "s59E25H2" 0 "Lipoprotein-releasing system ATP-binding protein LolD"
"Chl_A19_00154" 154 "s59E5H2" 0 "Lipoprotein-releasing system ATP-binding protein LolD"
"Chl_A19_00154" 154 "s63E6H2" 0 "Lipoprotein-releasing system ATP-binding protein LolD"
"Chl_A19_00155" 155 "s59E13H2" 0 "hypothetical protein"
"Chl_A19_00155" 155 "s59E25H2" 0 "hypothetical protein"
"Chl_A19_00155" 155 "s59E5H2" 0 "hypothetical protein"
"Chl_A19_00155" 155 "s63E6H2" 0 "hypothetical protein"
"Chl_A19_00157" 157 "s59E13H2" 0 "Tyrosine recombinase XerD"
"Chl_A19_00157" 157 "s59E25H2" 0 "Tyrosine recombinase XerD"
"Chl_A19_00157" 157 "s59E5H2" 0 "Tyrosine recombinase XerD"
"Chl_A19_00157" 157 "s63E6H2" 0 "Tyrosine recombinase XerD"
"Chl_A19_00158" 158 "s59E13H2" 0 "hypothetical protein"
"Chl_A19_00158" 158 "s59E25H2" 0 "hypothetical protein"
"Chl_A19_00158" 158 "s59E5H2" 0 "hypothetical protein"
"Chl_A19_00158" 158 "s63E6H2" 0 "hypothetical protein"
"Chl_A19_00159" 159 "s59E13H2" 0 "Alpha/beta hydrolase family protein"
"Chl_A19_00159" 159 "s59E25H2" 0 "Alpha/beta hydrolase family protein"
"Chl_A19_00159" 159 "s59E5H2" 0 "Alpha/beta hydrolase family protein"
"Chl_A19_00159" 159 "s63E6H2" 0 "Alpha/beta hydrolase family protein"
"Chl_A19_00160" 160 "s59E13H2" 0 "GYD domain protein"
"Chl_A19_00160" 160 "s59E25H2" 0 "GYD domain protein"
"Chl_A19_00160" 160 "s59E5H2" 0 "GYD domain protein"
"Chl_A19_00160" 160 "s63E6H2" 0 "GYD domain protein"
"Chl_A19_00161" 161 "s59E13H2" 0 "putative type I restriction enzymeP M protein"
"Chl_A19_00161" 161 "s59E25H2" 0 "putative type I restriction enzymeP M protein"
"Chl_A19_00161" 161 "s59E5H2" 0 "putative type I restriction enzymeP M protein"

"Chl_A19_00161" 161 "s63E6H2" 0 "putative type I restriction enzymeP M protein"
"Chl_A19_00162" 162 "s59E13H2" 0 "putative MFS-type transporter YhjX"
"Chl_A19_00162" 162 "s59E25H2" 0 "putative MFS-type transporter YhjX"
"Chl_A19_00162" 162 "s59E5H2" 0 "putative MFS-type transporter YhjX"
"Chl_A19_00162" 162 "s63E6H2" 0 "putative MFS-type transporter YhjX"
"Chl_A19_00163" 163 "s59E13H2" 0 "putative MFS-type transporter YhjX"
"Chl_A19_00163" 163 "s59E25H2" 0 "putative MFS-type transporter YhjX"
"Chl_A19_00163" 163 "s59E5H2" 0 "putative MFS-type transporter YhjX"
"Chl_A19_00163" 163 "s63E6H2" 0 "putative MFS-type transporter YhjX"
"Chl_A19_00164" 164 "s59E13H2" 0 "D12 class N6 adenine-specific DNA methyltransferase"
"Chl_A19_00164" 164 "s59E25H2" 0 "D12 class N6 adenine-specific DNA methyltransferase"
"Chl_A19_00164" 164 "s59E5H2" 0 "D12 class N6 adenine-specific DNA methyltransferase"
"Chl_A19_00164" 164 "s63E6H2" 0 "D12 class N6 adenine-specific DNA methyltransferase"
"Chl_A19_00166" 166 "s59E13H2" 0 "hypothetical protein"
"Chl_A19_00166" 166 "s59E25H2" 0 "hypothetical protein"
"Chl_A19_00166" 166 "s59E5H2" 0 "hypothetical protein"
"Chl_A19_00166" 166 "s63E6H2" 0 "hypothetical protein"
"Chl_A19_00167" 167 "s59E13H2" 0 "DegV domain-containing protein"
"Chl_A19_00167" 167 "s59E25H2" 0 "DegV domain-containing protein"
"Chl_A19_00167" 167 "s59E5H2" 0 "DegV domain-containing protein"
"Chl_A19_00167" 167 "s63E6H2" 0 "DegV domain-containing protein"
"Chl_A19_00168" 168 "s59E13H2" 0 "Adenylyl-sulfate kinase"
"Chl_A19_00168" 168 "s59E25H2" 0 "Adenylyl-sulfate kinase"
"Chl_A19_00168" 168 "s59E5H2" 0 "Adenylyl-sulfate kinase"
"Chl_A19_00168" 168 "s63E6H2" 0 "Adenylyl-sulfate kinase"
"Chl_A19_00169" 169 "s59E13H2" 0 "Tungsten-containing aldehyde ferredoxin oxidoreductase"
"Chl_A19_00169" 169 "s59E25H2" 0 "Tungsten-containing aldehyde ferredoxin oxidoreductase"
"Chl_A19_00169" 169 "s59E5H2" 0 "Tungsten-containing aldehyde ferredoxin oxidoreductase"
"Chl_A19_00169" 169 "s63E6H2" 0 "Tungsten-containing aldehyde ferredoxin oxidoreductase"
"Chl_A19_00170" 170 "s59E13H2" 0 "Cytosol aminopeptidase"
"Chl_A19_00170" 170 "s59E25H2" 0 "Cytosol aminopeptidase"
"Chl_A19_00170" 170 "s59E5H2" 0 "Cytosol aminopeptidase"
"Chl_A19_00170" 170 "s63E6H2" 0 "Cytosol aminopeptidase"
"Chl_A19_00171" 171 "s59E13H2" 0 "metal-dependent hydrolase"
"Chl_A19_00171" 171 "s59E25H2" 0 "metal-dependent hydrolase"
"Chl_A19_00171" 171 "s59E5H2" 0 "metal-dependent hydrolase"
"Chl_A19_00171" 171 "s63E6H2" 0 "metal-dependent hydrolase"
"Chl_A19_00172" 172 "s59E13H2" 0 "hypothetical protein"
"Chl_A19_00172" 172 "s59E25H2" 0 "hypothetical protein"
"Chl_A19_00172" 172 "s59E5H2" 0 "hypothetical protein"
"Chl_A19_00172" 172 "s63E6H2" 0 "hypothetical protein"
"Chl_A19_00174" 174 "s59E13H2" 0 "Acylphosphatase"
"Chl_A19_00174" 174 "s59E25H2" 0 "Acylphosphatase"
"Chl_A19_00174" 174 "s59E5H2" 0 "Acylphosphatase"
"Chl_A19_00174" 174 "s63E6H2" 0 "Acylphosphatase"
"Chl_A19_00176" 176 "s59E13H2" 0 "Ribonuclease 3"
"Chl_A19_00176" 176 "s59E25H2" 0 "Ribonuclease 3"

"Chl_A19_00176" 176 "s59E5H2" 0 "Ribonuclease 3"
"Chl_A19_00176" 176 "s63E6H2" 0 "Ribonuclease 3"
"Chl_A19_00177" 177 "s59E13H2" 0 "3-hydroxypropionyl-coenzyme A dehydratase"
"Chl_A19_00177" 177 "s59E25H2" 0 "3-hydroxypropionyl-coenzyme A dehydratase"
"Chl_A19_00177" 177 "s59E5H2" 0 "3-hydroxypropionyl-coenzyme A dehydratase"
"Chl_A19_00177" 177 "s63E6H2" 0 "3-hydroxypropionyl-coenzyme A dehydratase"
"Chl_A19_00178" 178 "s59E13H2" 0 "3-hydroxypropionyl-coenzyme A dehydratase"
"Chl_A19_00178" 178 "s59E25H2" 0 "3-hydroxypropionyl-coenzyme A dehydratase"
"Chl_A19_00178" 178 "s59E5H2" 0 "3-hydroxypropionyl-coenzyme A dehydratase"
"Chl_A19_00178" 178 "s63E6H2" 0 "3-hydroxypropionyl-coenzyme A dehydratase"
"Chl_A19_00179" 179 "s59E13H2" 0 "Chromosome partition protein Smc"
"Chl_A19_00179" 179 "s59E25H2" 0 "Chromosome partition protein Smc"
"Chl_A19_00179" 179 "s59E5H2" 0 "Chromosome partition protein Smc"
"Chl_A19_00179" 179 "s63E6H2" 0 "Chromosome partition protein Smc"
"Chl_A19_00180" 180 "s59E13H2" 0 "Beta-lactamase hydrolase-like protein"
"Chl_A19_00180" 180 "s59E25H2" 0 "Beta-lactamase hydrolase-like protein"
"Chl_A19_00180" 180 "s59E5H2" 0 "Beta-lactamase hydrolase-like protein"
"Chl_A19_00180" 180 "s63E6H2" 0 "Beta-lactamase hydrolase-like protein"
"Chl_A19_00181" 181 "s59E13H2" 0 "Diacetylchitobiose deacetylase"
"Chl_A19_00181" 181 "s59E25H2" 0 "Diacetylchitobiose deacetylase"
"Chl_A19_00181" 181 "s59E5H2" 0 "Diacetylchitobiose deacetylase"
"Chl_A19_00181" 181 "s63E6H2" 0 "Diacetylchitobiose deacetylase"
"Chl_A19_00182" 182 "s59E13H2" 0 "Mycothiol acetyltransferase"
"Chl_A19_00182" 182 "s59E25H2" 0 "Mycothiol acetyltransferase"
"Chl_A19_00182" 182 "s59E5H2" 0 "Mycothiol acetyltransferase"
"Chl_A19_00182" 182 "s63E6H2" 0 "Mycothiol acetyltransferase"
"Chl_A19_00183" 183 "s59E13H2" 0 "D-inositol 3-phosphate glycosyltransferase"
"Chl_A19_00183" 183 "s59E25H2" 0 "D-inositol 3-phosphate glycosyltransferase"
"Chl_A19_00183" 183 "s59E5H2" 0 "D-inositol 3-phosphate glycosyltransferase"
"Chl_A19_00183" 183 "s63E6H2" 0 "D-inositol 3-phosphate glycosyltransferase"
"Chl_A19_00184" 184 "s59E13H2" 0 "hypothetical protein"
"Chl_A19_00184" 184 "s59E25H2" 0 "hypothetical protein"
"Chl_A19_00184" 184 "s59E5H2" 0 "hypothetical protein"
"Chl_A19_00184" 184 "s63E6H2" 0 "hypothetical protein"
"Chl_A19_00186" 186 "s59E13H2" 0 "UDP-2-acetamido-2-deoxy-3-oxo-D-glucuronate aminotransferase"
"Chl_A19_00186" 186 "s59E25H2" 0 "UDP-2-acetamido-2-deoxy-3-oxo-D-glucuronate aminotransferase"
"Chl_A19_00186" 186 "s59E5H2" 0 "UDP-2-acetamido-2-deoxy-3-oxo-D-glucuronate aminotransferase"
"Chl_A19_00186" 186 "s63E6H2" 0 "UDP-2-acetamido-2-deoxy-3-oxo-D-glucuronate aminotransferase"
"Chl_A19_00187" 187 "s59E13H2" 0 "UDP-2-acetamido-3-amino-2,3-dideoxy-D-glucuronate N-acetyltransferase"
"Chl_A19_00187" 187 "s59E25H2" 0 "UDP-2-acetamido-3-amino-2,3-dideoxy-D-glucuronate N-acetyltransferase"
"Chl_A19_00187" 187 "s59E5H2" 0 "UDP-2-acetamido-3-amino-2,3-dideoxy-D-glucuronate N-acetyltransferase"
"Chl_A19_00187" 187 "s63E6H2" 0 "UDP-2-acetamido-3-amino-2,3-dideoxy-D-glucuronate N-acetyltransferase"
"Chl_A19_00188" 188 "s59E13H2" 0 "Chaperone protein TorD"
"Chl_A19_00188" 188 "s59E25H2" 0 "Chaperone protein TorD"
"Chl_A19_00188" 188 "s59E5H2" 0 "Chaperone protein TorD"
"Chl_A19_00188" 188 "s63E6H2" 0 "Chaperone protein TorD"
"Chl_A19_00191" 191 "s59E13H2" 0 "Pyrogallol hydroxytransferase large subunit"

"Chl_A19_00191" 191 "s59E25H2" 0 "Pyrogallol hydroxytransferase large subunit"
"Chl_A19_00191" 191 "s59E5H2" 0 "Pyrogallol hydroxytransferase large subunit"
"Chl_A19_00191" 191 "s63E6H2" 0 "Pyrogallol hydroxytransferase large subunit"
"Chl_A19_00192" 192 "s59E13H2" 0 "Pyrogallol hydroxytransferase small subunit"
"Chl_A19_00192" 192 "s59E25H2" 0 "Pyrogallol hydroxytransferase small subunit"
"Chl_A19_00192" 192 "s59E5H2" 0 "Pyrogallol hydroxytransferase small subunit"
"Chl_A19_00192" 192 "s63E6H2" 0 "Pyrogallol hydroxytransferase small subunit"
"Chl_A19_00193" 193 "s59E13H2" 0 "Deoxyuridine 5'-triphosphate nucleotidohydrolase"
"Chl_A19_00193" 193 "s59E25H2" 0 "Deoxyuridine 5'-triphosphate nucleotidohydrolase"
"Chl_A19_00193" 193 "s59E5H2" 0 "Deoxyuridine 5'-triphosphate nucleotidohydrolase"
"Chl_A19_00193" 193 "s63E6H2" 0 "Deoxyuridine 5'-triphosphate nucleotidohydrolase"
"Chl_A19_00194" 194 "s59E13H2" 0 "putative MFS-type transporter YhjX"
"Chl_A19_00194" 194 "s59E25H2" 0 "putative MFS-type transporter YhjX"
"Chl_A19_00194" 194 "s59E5H2" 0 "putative MFS-type transporter YhjX"
"Chl_A19_00194" 194 "s63E6H2" 0 "putative MFS-type transporter YhjX"
"Chl_A19_00195" 195 "s59E13H2" 0 "tRNA-specific adenosine deaminase"
"Chl_A19_00195" 195 "s59E25H2" 0 "tRNA-specific adenosine deaminase"
"Chl_A19_00195" 195 "s59E5H2" 0 "tRNA-specific adenosine deaminase"
"Chl_A19_00195" 195 "s63E6H2" 0 "tRNA-specific adenosine deaminase"
"Chl_A19_00196" 196 "s59E13H2" 0 "hypothetical protein"
"Chl_A19_00196" 196 "s59E25H2" 0 "hypothetical protein"
"Chl_A19_00196" 196 "s59E5H2" 0 "hypothetical protein"
"Chl_A19_00196" 196 "s63E6H2" 0 "hypothetical protein"
"Chl_A19_00197" 197 "s59E13H2" 0 "putative glutamine synthetase 2"
"Chl_A19_00197" 197 "s59E25H2" 0 "putative glutamine synthetase 2"
"Chl_A19_00197" 197 "s59E5H2" 0 "putative glutamine synthetase 2"
"Chl_A19_00197" 197 "s63E6H2" 0 "putative glutamine synthetase 2"
"Chl_A19_00198" 198 "s59E13H2" 0 "hypothetical protein"
"Chl_A19_00198" 198 "s59E25H2" 0 "hypothetical protein"
"Chl_A19_00198" 198 "s59E5H2" 0 "hypothetical protein"
"Chl_A19_00198" 198 "s63E6H2" 0 "hypothetical protein"
"Chl_A19_00199" 199 "s59E13H2" 0 "Imidazole glycerol phosphate synthase subunit HisF"
"Chl_A19_00199" 199 "s59E25H2" 0 "Imidazole glycerol phosphate synthase subunit HisF"
"Chl_A19_00199" 199 "s59E5H2" 0 "Imidazole glycerol phosphate synthase subunit HisF"
"Chl_A19_00199" 199 "s63E6H2" 0 "Imidazole glycerol phosphate synthase subunit HisF"
"Chl_A19_00200" 200 "s59E13H2" 0 "Ribonuclease R winged-helix domain protein"
"Chl_A19_00200" 200 "s59E25H2" 0 "Ribonuclease R winged-helix domain protein"
"Chl_A19_00200" 200 "s59E5H2" 0 "Ribonuclease R winged-helix domain protein"
"Chl_A19_00200" 200 "s63E6H2" 0 "Ribonuclease R winged-helix domain protein"
"Chl_A19_00201" 201 "s59E13H2" 0 "AP-4-A phosphorylase"
"Chl_A19_00201" 201 "s59E25H2" 0 "AP-4-A phosphorylase"
"Chl_A19_00201" 201 "s59E5H2" 0 "AP-4-A phosphorylase"
"Chl_A19_00201" 201 "s63E6H2" 0 "AP-4-A phosphorylase"
"Chl_A19_00202" 202 "s59E13H2" 0 "Transposase"
"Chl_A19_00202" 202 "s59E25H2" 0 "Transposase"
"Chl_A19_00202" 202 "s59E5H2" 0.0151388973418813 "Transposase"
"Chl_A19_00202" 202 "s63E6H2" 2.13366672709889 "Transposase"

"Chl_A19_00203" 203 "s59E13H2" 0.047428447202891 "IS2 transposase TnpB"
"Chl_A19_00203" 203 "s59E25H2" 0 "IS2 transposase TnpB"
"Chl_A19_00203" 203 "s59E5H2" 0.0685114573997515 "IS2 transposase TnpB"
"Chl_A19_00203" 203 "s63E6H2" 0.00268501954805771 "IS2 transposase TnpB"
"Chl_A19_00204" 204 "s59E13H2" 0 "O-acetylserine sulfhydrylase"
"Chl_A19_00204" 204 "s59E25H2" 0 "O-acetylserine sulfhydrylase"
"Chl_A19_00204" 204 "s59E5H2" 0 "O-acetylserine sulfhydrylase"
"Chl_A19_00204" 204 "s63E6H2" 0 "O-acetylserine sulfhydrylase"
"Chl_A19_00205" 205 "s59E13H2" 0 "Serine acetyltransferase"
"Chl_A19_00205" 205 "s59E25H2" 0 "Serine acetyltransferase"
"Chl_A19_00205" 205 "s59E5H2" 0 "Serine acetyltransferase"
"Chl_A19_00205" 205 "s63E6H2" 0 "Serine acetyltransferase"
"Chl_A19_00206" 206 "s59E13H2" 0 "hypothetical protein"
"Chl_A19_00206" 206 "s59E25H2" 0 "hypothetical protein"
"Chl_A19_00206" 206 "s59E5H2" 0 "hypothetical protein"
"Chl_A19_00206" 206 "s63E6H2" 0 "hypothetical protein"
"Chl_A19_00207" 207 "s59E13H2" 0 "Universal stress protein"
"Chl_A19_00207" 207 "s59E25H2" 0 "Universal stress protein"
"Chl_A19_00207" 207 "s59E5H2" 0 "Universal stress protein"
"Chl_A19_00207" 207 "s63E6H2" 0 "Universal stress protein"
"Chl_A19_00208" 208 "s59E13H2" 0 "putative cysteine desulfurase 2"
"Chl_A19_00208" 208 "s59E25H2" 0 "putative cysteine desulfurase 2"
"Chl_A19_00208" 208 "s59E5H2" 0 "putative cysteine desulfurase 2"
"Chl_A19_00208" 208 "s63E6H2" 0 "putative cysteine desulfurase 2"
"Chl_A19_00209" 209 "s59E13H2" 0 "NifU-like protein"
"Chl_A19_00209" 209 "s59E25H2" 0 "NifU-like protein"
"Chl_A19_00209" 209 "s59E5H2" 0 "NifU-like protein"
"Chl_A19_00209" 209 "s63E6H2" 0 "NifU-like protein"
"Chl_A19_00210" 210 "s59E13H2" 0 "Hydrogenase isoenzymes formation protein HypE"
"Chl_A19_00210" 210 "s59E25H2" 0 "Hydrogenase isoenzymes formation protein HypE"
"Chl_A19_00210" 210 "s59E5H2" 0 "Hydrogenase isoenzymes formation protein HypE"
"Chl_A19_00210" 210 "s63E6H2" 0 "Hydrogenase isoenzymes formation protein HypE"
"Chl_A19_00211" 211 "s59E13H2" 0 "hypothetical protein"
"Chl_A19_00211" 211 "s59E25H2" 0 "hypothetical protein"
"Chl_A19_00211" 211 "s59E5H2" 0 "hypothetical protein"
"Chl_A19_00211" 211 "s63E6H2" 0 "hypothetical protein"
"Chl_A19_00212" 212 "s59E13H2" 0 "formate dehydrogenase accessory protein"
"Chl_A19_00212" 212 "s59E25H2" 0 "formate dehydrogenase accessory protein"
"Chl_A19_00212" 212 "s59E5H2" 0 "formate dehydrogenase accessory protein"
"Chl_A19_00212" 212 "s63E6H2" 0 "formate dehydrogenase accessory protein"
"Chl_A19_00213" 213 "s59E13H2" 0 "Quinolinate synthase A"
"Chl_A19_00213" 213 "s59E25H2" 0 "Quinolinate synthase A"
"Chl_A19_00213" 213 "s59E5H2" 0 "Quinolinate synthase A"
"Chl_A19_00213" 213 "s63E6H2" 0 "Quinolinate synthase A"
"Chl_A19_00214" 214 "s59E13H2" 0 "DinB superfamily protein"
"Chl_A19_00214" 214 "s59E25H2" 0 "DinB superfamily protein"
"Chl_A19_00214" 214 "s59E5H2" 0 "DinB superfamily protein"

"Chl_A19_00214" 214 "s63E6H2" 0 "DinB superfamily protein"
"Chl_A19_00215" 215 "s59E13H2" 0 "Demethylrebeccamycin-D-glucose O-methyltransferase"
"Chl_A19_00215" 215 "s59E25H2" 0 "Demethylrebeccamycin-D-glucose O-methyltransferase"
"Chl_A19_00215" 215 "s59E5H2" 0 "Demethylrebeccamycin-D-glucose O-methyltransferase"
"Chl_A19_00215" 215 "s63E6H2" 0 "Demethylrebeccamycin-D-glucose O-methyltransferase"
"Chl_A19_00217" 217 "s59E13H2" 0 "DNA-invertase hin"
"Chl_A19_00217" 217 "s59E25H2" 0 "DNA-invertase hin"
"Chl_A19_00217" 217 "s59E5H2" 0 "DNA-invertase hin"
"Chl_A19_00217" 217 "s63E6H2" 0 "DNA-invertase hin"
"Chl_A19_00218" 218 "s59E13H2" 0 "Zonular occludens toxin (Zot)"
"Chl_A19_00218" 218 "s59E25H2" 0 "Zonular occludens toxin (Zot)"
"Chl_A19_00218" 218 "s59E5H2" 0 "Zonular occludens toxin (Zot)"
"Chl_A19_00218" 218 "s63E6H2" 0 "Zonular occludens toxin (Zot)"
"Chl_A19_00220" 220 "s59E13H2" 0 "Oxalate:formate antiporter"
"Chl_A19_00220" 220 "s59E25H2" 0 "Oxalate:formate antiporter"
"Chl_A19_00220" 220 "s59E5H2" 0 "Oxalate:formate antiporter"
"Chl_A19_00220" 220 "s63E6H2" 0 "Oxalate:formate antiporter"
"Chl_A19_00221" 221 "s59E13H2" 0 "DSBA-like thioredoxin domain protein"
"Chl_A19_00221" 221 "s59E25H2" 0 "DSBA-like thioredoxin domain protein"
"Chl_A19_00221" 221 "s59E5H2" 0 "DSBA-like thioredoxin domain protein"
"Chl_A19_00221" 221 "s63E6H2" 0 "DSBA-like thioredoxin domain protein"
"Chl_A19_00222" 222 "s59E13H2" 0 "MmgE/PrpD family protein"
"Chl_A19_00222" 222 "s59E25H2" 0 "MmgE/PrpD family protein"
"Chl_A19_00222" 222 "s59E5H2" 0 "MmgE/PrpD family protein"
"Chl_A19_00222" 222 "s63E6H2" 0 "MmgE/PrpD family protein"
"Chl_A19_00223" 223 "s59E13H2" 0 "putative MFS-type transporter YhjX"
"Chl_A19_00223" 223 "s59E25H2" 0 "putative MFS-type transporter YhjX"
"Chl_A19_00223" 223 "s59E5H2" 0 "putative MFS-type transporter YhjX"
"Chl_A19_00223" 223 "s63E6H2" 0 "putative MFS-type transporter YhjX"
"Chl_A19_00224" 224 "s59E13H2" 0 "hypothetical protein"
"Chl_A19_00224" 224 "s59E25H2" 0 "hypothetical protein"
"Chl_A19_00224" 224 "s59E5H2" 0 "hypothetical protein"
"Chl_A19_00224" 224 "s63E6H2" 0 "hypothetical protein"
"Chl_A19_00225" 225 "s59E13H2" 0 "Rubrerythrin"
"Chl_A19_00225" 225 "s59E25H2" 0 "Rubrerythrin"
"Chl_A19_00225" 225 "s59E5H2" 0 "Rubrerythrin"
"Chl_A19_00225" 225 "s63E6H2" 0 "Rubrerythrin"
"Chl_A19_00227" 227 "s59E13H2" 0 "putative MFS-type transporter YhjX"
"Chl_A19_00227" 227 "s59E25H2" 0 "putative MFS-type transporter YhjX"
"Chl_A19_00227" 227 "s59E5H2" 0 "putative MFS-type transporter YhjX"
"Chl_A19_00227" 227 "s63E6H2" 0 "putative MFS-type transporter YhjX"
"Chl_A19_00228" 228 "s59E13H2" 0 "putative dimethyl sulfoxide reductase chain YnfF precursor"
"Chl_A19_00228" 228 "s59E25H2" 0 "putative dimethyl sulfoxide reductase chain YnfF precursor"
"Chl_A19_00228" 228 "s59E5H2" 0 "putative dimethyl sulfoxide reductase chain YnfF precursor"
"Chl_A19_00228" 228 "s63E6H2" 0 "putative dimethyl sulfoxide reductase chain YnfF precursor"
"Chl_A19_00229" 229 "s59E13H2" 0 "Assimilatory nitrate reductase catalytic subunit"
"Chl_A19_00229" 229 "s59E25H2" 0 "Assimilatory nitrate reductase catalytic subunit"

"Chl_A19_00229" 229 "s59E5H2" 0 "Assimilatory nitrate reductase catalytic subunit"
"Chl_A19_00229" 229 "s63E6H2" 0 "Assimilatory nitrate reductase catalytic subunit"
"Chl_A19_00230" 230 "s59E13H2" 0 "Pyrogallol hydroxytransferase large subunit"
"Chl_A19_00230" 230 "s59E25H2" 0 "Pyrogallol hydroxytransferase large subunit"
"Chl_A19_00230" 230 "s59E5H2" 0 "Pyrogallol hydroxytransferase large subunit"
"Chl_A19_00230" 230 "s63E6H2" 0 "Pyrogallol hydroxytransferase large subunit"
"Chl_A19_00231" 231 "s59E13H2" 0 "putative MFS-type transporter YhjX"
"Chl_A19_00231" 231 "s59E25H2" 0 "putative MFS-type transporter YhjX"
"Chl_A19_00231" 231 "s59E5H2" 0 "putative MFS-type transporter YhjX"
"Chl_A19_00231" 231 "s63E6H2" 0 "putative MFS-type transporter YhjX"
"Chl_A19_00232" 232 "s59E13H2" 0 "5,6-dimethylbenzimidazole synthase"
"Chl_A19_00232" 232 "s59E25H2" 0 "5,6-dimethylbenzimidazole synthase"
"Chl_A19_00232" 232 "s59E5H2" 0 "5,6-dimethylbenzimidazole synthase"
"Chl_A19_00232" 232 "s63E6H2" 0 "5,6-dimethylbenzimidazole synthase"
"Chl_A19_00233" 233 "s59E13H2" 0 "Acetylene hydratase"
"Chl_A19_00233" 233 "s59E25H2" 0 "Acetylene hydratase"
"Chl_A19_00233" 233 "s59E5H2" 0 "Acetylene hydratase"
"Chl_A19_00233" 233 "s63E6H2" 0 "Acetylene hydratase"
"Chl_A19_00234" 234 "s59E13H2" 0 "Acetylene hydratase"
"Chl_A19_00234" 234 "s59E25H2" 0 "Acetylene hydratase"
"Chl_A19_00234" 234 "s59E5H2" 0 "Acetylene hydratase"
"Chl_A19_00234" 234 "s63E6H2" 0 "Acetylene hydratase"
"Chl_A19_00235" 235 "s59E13H2" 0 "hypothetical protein"
"Chl_A19_00235" 235 "s59E25H2" 0 "hypothetical protein"
"Chl_A19_00235" 235 "s59E5H2" 0 "hypothetical protein"
"Chl_A19_00235" 235 "s63E6H2" 0 "hypothetical protein"
"Chl_A19_00236" 236 "s59E13H2" 0 "Fumarate reductase flavoprotein subunit precursor"
"Chl_A19_00236" 236 "s59E25H2" 0 "Fumarate reductase flavoprotein subunit precursor"
"Chl_A19_00236" 236 "s59E5H2" 0 "Fumarate reductase flavoprotein subunit precursor"
"Chl_A19_00236" 236 "s63E6H2" 0 "Fumarate reductase flavoprotein subunit precursor"
"Chl_A19_00237" 237 "s59E13H2" 0 "Cysteine--tRNA ligase"
"Chl_A19_00237" 237 "s59E25H2" 0 "Cysteine--tRNA ligase"
"Chl_A19_00237" 237 "s59E5H2" 0 "Cysteine--tRNA ligase"
"Chl_A19_00237" 237 "s63E6H2" 0 "Cysteine--tRNA ligase"
"Chl_A19_00238" 238 "s59E13H2" 0 "hypothetical protein"
"Chl_A19_00238" 238 "s59E25H2" 0 "hypothetical protein"
"Chl_A19_00238" 238 "s59E5H2" 0 "hypothetical protein"
"Chl_A19_00238" 238 "s63E6H2" 0 "hypothetical protein"
"Chl_A19_00239" 239 "s59E13H2" 0 "hypothetical protein"
"Chl_A19_00239" 239 "s59E25H2" 0 "hypothetical protein"
"Chl_A19_00239" 239 "s59E5H2" 0 "hypothetical protein"
"Chl_A19_00239" 239 "s63E6H2" 0 "hypothetical protein"
"Chl_A19_00240" 240 "s59E13H2" 0 "B12 binding domain protein"
"Chl_A19_00240" 240 "s59E25H2" 0 "B12 binding domain protein"
"Chl_A19_00240" 240 "s59E5H2" 0 "B12 binding domain protein"
"Chl_A19_00240" 240 "s63E6H2" 0 "B12 binding domain protein"
"Chl_A19_00241" 241 "s59E13H2" 0 "Inner membrane transport permease YadH"

"Chl_A19_00241" 241 "s59E25H2" 0 "Inner membrane transport permease YadH"
"Chl_A19_00241" 241 "s59E5H2" 0 "Inner membrane transport permease YadH"
"Chl_A19_00241" 241 "s63E6H2" 0 "Inner membrane transport permease YadH"
"Chl_A19_00242" 242 "s59E13H2" 0 "Daunorubicin/doxorubicin resistance ATP-binding protein DrrA"
"Chl_A19_00242" 242 "s59E25H2" 0 "Daunorubicin/doxorubicin resistance ATP-binding protein DrrA"
"Chl_A19_00242" 242 "s59E5H2" 0 "Daunorubicin/doxorubicin resistance ATP-binding protein DrrA"
"Chl_A19_00242" 242 "s63E6H2" 0 "Daunorubicin/doxorubicin resistance ATP-binding protein DrrA"
"Chl_A19_00243" 243 "s59E13H2" 0 "1,4-dihydroxy-2-naphthoate octaprenyltransferase"
"Chl_A19_00243" 243 "s59E25H2" 0 "1,4-dihydroxy-2-naphthoate octaprenyltransferase"
"Chl_A19_00243" 243 "s59E5H2" 0 "1,4-dihydroxy-2-naphthoate octaprenyltransferase"
"Chl_A19_00243" 243 "s63E6H2" 0 "1,4-dihydroxy-2-naphthoate octaprenyltransferase"
"Chl_A19_00244" 244 "s59E13H2" 0 "preprotein translocase subunit SecG"
"Chl_A19_00244" 244 "s59E25H2" 0 "preprotein translocase subunit SecG"
"Chl_A19_00244" 244 "s59E5H2" 0 "preprotein translocase subunit SecG"
"Chl_A19_00244" 244 "s63E6H2" 0 "preprotein translocase subunit SecG"
"Chl_A19_00246" 246 "s59E13H2" 0 "Helix-turn-helix domain protein"
"Chl_A19_00246" 246 "s59E25H2" 0 "Helix-turn-helix domain protein"
"Chl_A19_00246" 246 "s59E5H2" 0 "Helix-turn-helix domain protein"
"Chl_A19_00246" 246 "s63E6H2" 0 "Helix-turn-helix domain protein"
"Chl_A19_00247" 247 "s59E13H2" 0 "50S ribosomal protein L2"
"Chl_A19_00247" 247 "s59E25H2" 0 "50S ribosomal protein L2"
"Chl_A19_00247" 247 "s59E5H2" 0 "50S ribosomal protein L2"
"Chl_A19_00247" 247 "s63E6H2" 0 "50S ribosomal protein L2"
"Chl_A19_00248" 248 "s59E13H2" 0 "30S ribosomal protein S19"
"Chl_A19_00248" 248 "s59E25H2" 0 "30S ribosomal protein S19"
"Chl_A19_00248" 248 "s59E5H2" 0 "30S ribosomal protein S19"
"Chl_A19_00248" 248 "s63E6H2" 0 "30S ribosomal protein S19"
"Chl_A19_00249" 249 "s59E13H2" 0 "50S ribosomal protein L22"
"Chl_A19_00249" 249 "s59E25H2" 0 "50S ribosomal protein L22"
"Chl_A19_00249" 249 "s59E5H2" 0 "50S ribosomal protein L22"
"Chl_A19_00249" 249 "s63E6H2" 0 "50S ribosomal protein L22"
"Chl_A19_00250" 250 "s59E13H2" 0 "30S ribosomal protein S3"
"Chl_A19_00250" 250 "s59E25H2" 0 "30S ribosomal protein S3"
"Chl_A19_00250" 250 "s59E5H2" 0 "30S ribosomal protein S3"
"Chl_A19_00250" 250 "s63E6H2" 0 "30S ribosomal protein S3"
"Chl_A19_00251" 251 "s59E13H2" 0 "50S ribosomal protein L16"
"Chl_A19_00251" 251 "s59E25H2" 0 "50S ribosomal protein L16"
"Chl_A19_00251" 251 "s59E5H2" 0 "50S ribosomal protein L16"
"Chl_A19_00251" 251 "s63E6H2" 0 "50S ribosomal protein L16"
"Chl_A19_00252" 252 "s59E13H2" 0 "50S ribosomal protein L29"
"Chl_A19_00252" 252 "s59E25H2" 0 "50S ribosomal protein L29"
"Chl_A19_00252" 252 "s59E5H2" 0 "50S ribosomal protein L29"
"Chl_A19_00252" 252 "s63E6H2" 0 "50S ribosomal protein L29"
"Chl_A19_00253" 253 "s59E13H2" 0 "30S ribosomal protein S17"
"Chl_A19_00253" 253 "s59E25H2" 0 "30S ribosomal protein S17"
"Chl_A19_00253" 253 "s59E5H2" 0 "30S ribosomal protein S17"
"Chl_A19_00253" 253 "s63E6H2" 0 "30S ribosomal protein S17"

"Chl_A19_00254" 254 "s59E13H2" 0 "50S ribosomal protein L14"
"Chl_A19_00254" 254 "s59E25H2" 0 "50S ribosomal protein L14"
"Chl_A19_00254" 254 "s59E5H2" 0 "50S ribosomal protein L14"
"Chl_A19_00254" 254 "s63E6H2" 0 "50S ribosomal protein L14"
"Chl_A19_00255" 255 "s59E13H2" 0 "50S ribosomal protein L24"
"Chl_A19_00255" 255 "s59E25H2" 0 "50S ribosomal protein L24"
"Chl_A19_00255" 255 "s59E5H2" 0 "50S ribosomal protein L24"
"Chl_A19_00255" 255 "s63E6H2" 0 "50S ribosomal protein L24"
"Chl_A19_00256" 256 "s59E13H2" 0 "50S ribosomal protein L5"
"Chl_A19_00256" 256 "s59E25H2" 0 "50S ribosomal protein L5"
"Chl_A19_00256" 256 "s59E5H2" 0 "50S ribosomal protein L5"
"Chl_A19_00256" 256 "s63E6H2" 0 "50S ribosomal protein L5"
"Chl_A19_00257" 257 "s59E13H2" 0 "30S ribosomal protein S14"
"Chl_A19_00257" 257 "s59E25H2" 0 "30S ribosomal protein S14"
"Chl_A19_00257" 257 "s59E5H2" 0 "30S ribosomal protein S14"
"Chl_A19_00257" 257 "s63E6H2" 0 "30S ribosomal protein S14"
"Chl_A19_00258" 258 "s59E13H2" 0 "30S ribosomal protein S8"
"Chl_A19_00258" 258 "s59E25H2" 0 "30S ribosomal protein S8"
"Chl_A19_00258" 258 "s59E5H2" 0 "30S ribosomal protein S8"
"Chl_A19_00258" 258 "s63E6H2" 0 "30S ribosomal protein S8"
"Chl_A19_00259" 259 "s59E13H2" 0 "50S ribosomal protein L6"
"Chl_A19_00259" 259 "s59E25H2" 0 "50S ribosomal protein L6"
"Chl_A19_00259" 259 "s59E5H2" 0 "50S ribosomal protein L6"
"Chl_A19_00259" 259 "s63E6H2" 0 "50S ribosomal protein L6"
"Chl_A19_00260" 260 "s59E13H2" 0 "50S ribosomal protein L18"
"Chl_A19_00260" 260 "s59E25H2" 0 "50S ribosomal protein L18"
"Chl_A19_00260" 260 "s59E5H2" 0 "50S ribosomal protein L18"
"Chl_A19_00260" 260 "s63E6H2" 0 "50S ribosomal protein L18"
"Chl_A19_00261" 261 "s59E13H2" 0 "30S ribosomal protein S5"
"Chl_A19_00261" 261 "s59E25H2" 0 "30S ribosomal protein S5"
"Chl_A19_00261" 261 "s59E5H2" 0 "30S ribosomal protein S5"
"Chl_A19_00261" 261 "s63E6H2" 0 "30S ribosomal protein S5"
"Chl_A19_00262" 262 "s59E13H2" 0 "50S ribosomal protein L30"
"Chl_A19_00262" 262 "s59E25H2" 0 "50S ribosomal protein L30"
"Chl_A19_00262" 262 "s59E5H2" 0 "50S ribosomal protein L30"
"Chl_A19_00262" 262 "s63E6H2" 0 "50S ribosomal protein L30"
"Chl_A19_00263" 263 "s59E13H2" 0 "50S ribosomal protein L15"
"Chl_A19_00263" 263 "s59E25H2" 0 "50S ribosomal protein L15"
"Chl_A19_00263" 263 "s59E5H2" 0 "50S ribosomal protein L15"
"Chl_A19_00263" 263 "s63E6H2" 0 "50S ribosomal protein L15"
"Chl_A19_00264" 264 "s59E13H2" 0 "preprotein translocase subunit SecY"
"Chl_A19_00264" 264 "s59E25H2" 0 "preprotein translocase subunit SecY"
"Chl_A19_00264" 264 "s59E5H2" 0 "preprotein translocase subunit SecY"
"Chl_A19_00264" 264 "s63E6H2" 0 "preprotein translocase subunit SecY"
"Chl_A19_00265" 265 "s59E13H2" 0 "Adenylate kinase"
"Chl_A19_00265" 265 "s59E25H2" 0 "Adenylate kinase"
"Chl_A19_00265" 265 "s59E5H2" 0 "Adenylate kinase"

"Chl_A19_00265" 265 "s63E6H2" 0 "Adenylate kinase"
"Chl_A19_00266" 266 "s59E13H2" 0 "Methionine aminopeptidase 1"
"Chl_A19_00266" 266 "s59E25H2" 0 "Methionine aminopeptidase 1"
"Chl_A19_00266" 266 "s59E5H2" 0 "Methionine aminopeptidase 1"
"Chl_A19_00266" 266 "s63E6H2" 0 "Methionine aminopeptidase 1"
"Chl_A19_00267" 267 "s59E13H2" 0 "Translation initiation factor IF-1"
"Chl_A19_00267" 267 "s59E25H2" 0 "Translation initiation factor IF-1"
"Chl_A19_00267" 267 "s59E5H2" 0 "Translation initiation factor IF-1"
"Chl_A19_00267" 267 "s63E6H2" 0 "Translation initiation factor IF-1"
"Chl_A19_00268" 268 "s59E13H2" 0 "30S ribosomal protein S13"
"Chl_A19_00268" 268 "s59E25H2" 0 "30S ribosomal protein S13"
"Chl_A19_00268" 268 "s59E5H2" 0 "30S ribosomal protein S13"
"Chl_A19_00268" 268 "s63E6H2" 0 "30S ribosomal protein S13"
"Chl_A19_00269" 269 "s59E13H2" 0 "30S ribosomal protein S11"
"Chl_A19_00269" 269 "s59E25H2" 0 "30S ribosomal protein S11"
"Chl_A19_00269" 269 "s59E5H2" 0 "30S ribosomal protein S11"
"Chl_A19_00269" 269 "s63E6H2" 0 "30S ribosomal protein S11"
"Chl_A19_00270" 270 "s59E13H2" 0 "30S ribosomal protein S4"
"Chl_A19_00270" 270 "s59E25H2" 0 "30S ribosomal protein S4"
"Chl_A19_00270" 270 "s59E5H2" 0 "30S ribosomal protein S4"
"Chl_A19_00270" 270 "s63E6H2" 0 "30S ribosomal protein S4"
"Chl_A19_00271" 271 "s59E13H2" 0 "DNA-directed RNA polymerase subunit alpha"
"Chl_A19_00271" 271 "s59E25H2" 0 "DNA-directed RNA polymerase subunit alpha"
"Chl_A19_00271" 271 "s59E5H2" 0 "DNA-directed RNA polymerase subunit alpha"
"Chl_A19_00271" 271 "s63E6H2" 0 "DNA-directed RNA polymerase subunit alpha"
"Chl_A19_00272" 272 "s59E13H2" 0 "50S ribosomal protein L17"
"Chl_A19_00272" 272 "s59E25H2" 0 "50S ribosomal protein L17"
"Chl_A19_00272" 272 "s59E5H2" 0 "50S ribosomal protein L17"
"Chl_A19_00272" 272 "s63E6H2" 0 "50S ribosomal protein L17"
"Chl_A19_00273" 273 "s59E13H2" 0 "Acyl-CoA dehydrogenase"
"Chl_A19_00273" 273 "s59E25H2" 0 "Acyl-CoA dehydrogenase"
"Chl_A19_00273" 273 "s59E5H2" 0 "Acyl-CoA dehydrogenase"
"Chl_A19_00273" 273 "s63E6H2" 0 "Acyl-CoA dehydrogenase"
"Chl_A19_00274" 274 "s59E13H2" 0 "Amidohydrolase"
"Chl_A19_00274" 274 "s59E25H2" 0 "Amidohydrolase"
"Chl_A19_00274" 274 "s59E5H2" 0 "Amidohydrolase"
"Chl_A19_00274" 274 "s63E6H2" 0 "Amidohydrolase"
"Chl_A19_00275" 275 "s59E13H2" 0 "tRNA pseudouridine synthase A"
"Chl_A19_00275" 275 "s59E25H2" 0 "tRNA pseudouridine synthase A"
"Chl_A19_00275" 275 "s59E5H2" 0 "tRNA pseudouridine synthase A"
"Chl_A19_00275" 275 "s63E6H2" 0 "tRNA pseudouridine synthase A"
"Chl_A19_00276" 276 "s59E13H2" 0 "Amidohydrolase"
"Chl_A19_00276" 276 "s59E25H2" 0 "Amidohydrolase"
"Chl_A19_00276" 276 "s59E5H2" 0 "Amidohydrolase"
"Chl_A19_00276" 276 "s63E6H2" 0 "Amidohydrolase"
"Chl_A19_00277" 277 "s59E13H2" 0 "putative propionyl-CoA carboxylase beta chain 5"
"Chl_A19_00277" 277 "s59E25H2" 0 "putative propionyl-CoA carboxylase beta chain 5"

"Chl_A19_00277" 277 "s59E5H2" 0 "putative propionyl-CoA carboxylase beta chain 5"
"Chl_A19_00277" 277 "s63E6H2" 0 "putative propionyl-CoA carboxylase beta chain 5"
"Chl_A19_00278" 278 "s59E13H2" 0 "Amidohydrolase"
"Chl_A19_00278" 278 "s59E25H2" 0 "Amidohydrolase"
"Chl_A19_00278" 278 "s59E5H2" 0 "Amidohydrolase"
"Chl_A19_00278" 278 "s63E6H2" 0 "Amidohydrolase"
"Chl_A19_00279" 279 "s59E13H2" 0 "3-oxoacyl-[acyl-carrier-protein] reductase FabG"
"Chl_A19_00279" 279 "s59E25H2" 0 "3-oxoacyl-[acyl-carrier-protein] reductase FabG"
"Chl_A19_00279" 279 "s59E5H2" 0 "3-oxoacyl-[acyl-carrier-protein] reductase FabG"
"Chl_A19_00279" 279 "s63E6H2" 0 "3-oxoacyl-[acyl-carrier-protein] reductase FabG"
"Chl_A19_00280" 280 "s59E13H2" 0 "putative NH(3)-dependent NAD(+) synthetase"
"Chl_A19_00280" 280 "s59E25H2" 0 "putative NH(3)-dependent NAD(+) synthetase"
"Chl_A19_00280" 280 "s59E5H2" 0 "putative NH(3)-dependent NAD(+) synthetase"
"Chl_A19_00280" 280 "s63E6H2" 0 "putative NH(3)-dependent NAD(+) synthetase"
"Chl_A19_00281" 281 "s59E13H2" 0 "2-methylcitrate dehydratase"
"Chl_A19_00281" 281 "s59E25H2" 0 "2-methylcitrate dehydratase"
"Chl_A19_00281" 281 "s59E5H2" 0 "2-methylcitrate dehydratase"
"Chl_A19_00281" 281 "s63E6H2" 0 "2-methylcitrate dehydratase"
"Chl_A19_00282" 282 "s59E13H2" 0 "3-hydroxypropionyl-coenzyme A dehydratase"
"Chl_A19_00282" 282 "s59E25H2" 0 "3-hydroxypropionyl-coenzyme A dehydratase"
"Chl_A19_00282" 282 "s59E5H2" 0 "3-hydroxypropionyl-coenzyme A dehydratase"
"Chl_A19_00282" 282 "s63E6H2" 0 "3-hydroxypropionyl-coenzyme A dehydratase"
"Chl_A19_00283" 283 "s59E13H2" 0 "putative acyl-CoA thioester hydrolase"
"Chl_A19_00283" 283 "s59E25H2" 0 "putative acyl-CoA thioester hydrolase"
"Chl_A19_00283" 283 "s59E5H2" 0 "putative acyl-CoA thioester hydrolase"
"Chl_A19_00283" 283 "s63E6H2" 0 "putative acyl-CoA thioester hydrolase"
"Chl_A19_00284" 284 "s59E13H2" 0 "N-substituted formamide deformylase precursor"
"Chl_A19_00284" 284 "s59E25H2" 0 "N-substituted formamide deformylase precursor"
"Chl_A19_00284" 284 "s59E5H2" 0 "N-substituted formamide deformylase precursor"
"Chl_A19_00284" 284 "s63E6H2" 0 "N-substituted formamide deformylase precursor"
"Chl_A19_00285" 285 "s59E13H2" 0 "Lactate utilization protein A"
"Chl_A19_00285" 285 "s59E25H2" 0 "Lactate utilization protein A"
"Chl_A19_00285" 285 "s59E5H2" 0 "Lactate utilization protein A"
"Chl_A19_00285" 285 "s63E6H2" 0 "Lactate utilization protein A"
"Chl_A19_00286" 286 "s59E13H2" 0 "Acetylene hydratase"
"Chl_A19_00286" 286 "s59E25H2" 0 "Acetylene hydratase"
"Chl_A19_00286" 286 "s59E5H2" 0 "Acetylene hydratase"
"Chl_A19_00286" 286 "s63E6H2" 0 "Acetylene hydratase"
"Chl_A19_00287" 287 "s59E13H2" 0 "hypothetical protein"
"Chl_A19_00287" 287 "s59E25H2" 0 "hypothetical protein"
"Chl_A19_00287" 287 "s59E5H2" 0.000634885702388896 "hypothetical protein"
"Chl_A19_00287" 287 "s63E6H2" 0 "hypothetical protein"
"Chl_A19_00288" 288 "s59E13H2" 0 "hypothetical protein"
"Chl_A19_00288" 288 "s59E25H2" 0 "hypothetical protein"
"Chl_A19_00288" 288 "s59E5H2" 0 "hypothetical protein"
"Chl_A19_00288" 288 "s63E6H2" 0 "hypothetical protein"
"Chl_A19_00289" 289 "s59E13H2" 0 "hypothetical protein"

"Chl_A19_00289" 289 "s59E25H2" 0 "hypothetical protein"
"Chl_A19_00289" 289 "s59E5H2" 0 "hypothetical protein"
"Chl_A19_00289" 289 "s63E6H2" 0 "hypothetical protein"
"Chl_A19_00290" 290 "s59E13H2" 0 "DNA polymerase III subunit alpha"
"Chl_A19_00290" 290 "s59E25H2" 0 "DNA polymerase III subunit alpha"
"Chl_A19_00290" 290 "s59E5H2" 0 "DNA polymerase III subunit alpha"
"Chl_A19_00290" 290 "s63E6H2" 0 "DNA polymerase III subunit alpha"
"Chl_A19_00291" 291 "s59E13H2" 0 "Demethylmenaquinone methyltransferase"
"Chl_A19_00291" 291 "s59E25H2" 0 "Demethylmenaquinone methyltransferase"
"Chl_A19_00291" 291 "s59E5H2" 0 "Demethylmenaquinone methyltransferase"
"Chl_A19_00291" 291 "s63E6H2" 0 "Demethylmenaquinone methyltransferase"
"Chl_A19_00292" 292 "s59E13H2" 0 "hypothetical protein"
"Chl_A19_00292" 292 "s59E25H2" 0 "hypothetical protein"
"Chl_A19_00292" 292 "s59E5H2" 0 "hypothetical protein"
"Chl_A19_00292" 292 "s63E6H2" 0.00172246537045212 "hypothetical protein"
"Chl_A19_00293" 293 "s59E13H2" 0 "Chagasin family peptidase inhibitor I42"
"Chl_A19_00293" 293 "s59E25H2" 0 "Chagasin family peptidase inhibitor I42"
"Chl_A19_00293" 293 "s59E5H2" 0 "Chagasin family peptidase inhibitor I42"
"Chl_A19_00293" 293 "s63E6H2" 0 "Chagasin family peptidase inhibitor I42"
"Chl_A19_00294" 294 "s59E13H2" 0 "Methylated-DNA--protein-cysteine methyltransferase"
"Chl_A19_00294" 294 "s59E25H2" 0 "Methylated-DNA--protein-cysteine methyltransferase"
"Chl_A19_00294" 294 "s59E5H2" 0 "Methylated-DNA--protein-cysteine methyltransferase"
"Chl_A19_00294" 294 "s63E6H2" 0 "Methylated-DNA--protein-cysteine methyltransferase"
"Chl_A19_00295" 295 "s59E13H2" 0 "Putative sigma-54 modulation protein"
"Chl_A19_00295" 295 "s59E25H2" 0 "Putative sigma-54 modulation protein"
"Chl_A19_00295" 295 "s59E5H2" 0 "Putative sigma-54 modulation protein"
"Chl_A19_00295" 295 "s63E6H2" 0 "Putative sigma-54 modulation protein"
"Chl_A19_00296" 296 "s59E13H2" 0 "DNA utilization protein GntX"
"Chl_A19_00296" 296 "s59E25H2" 0 "DNA utilization protein GntX"
"Chl_A19_00296" 296 "s59E5H2" 0 "DNA utilization protein GntX"
"Chl_A19_00296" 296 "s63E6H2" 0 "DNA utilization protein GntX"
"Chl_A19_00298" 298 "s59E13H2" 0 "2'-5'-RNA ligase"
"Chl_A19_00298" 298 "s59E25H2" 0 "2'-5'-RNA ligase"
"Chl_A19_00298" 298 "s59E5H2" 0 "2'-5'-RNA ligase"
"Chl_A19_00298" 298 "s63E6H2" 0 "2'-5'-RNA ligase"
"Chl_A19_00299" 299 "s59E13H2" 0 "Amidohydrolase"
"Chl_A19_00299" 299 "s59E25H2" 0 "Amidohydrolase"
"Chl_A19_00299" 299 "s59E5H2" 0 "Amidohydrolase"
"Chl_A19_00299" 299 "s63E6H2" 0 "Amidohydrolase"
"Chl_A19_00300" 300 "s59E13H2" 0 "10 kDa chaperonin"
"Chl_A19_00300" 300 "s59E25H2" 0 "10 kDa chaperonin"
"Chl_A19_00300" 300 "s59E5H2" 0 "10 kDa chaperonin"
"Chl_A19_00300" 300 "s63E6H2" 0 "10 kDa chaperonin"
"Chl_A19_00301" 301 "s59E13H2" 0 "60 kDa chaperonin"
"Chl_A19_00301" 301 "s59E25H2" 0 "60 kDa chaperonin"
"Chl_A19_00301" 301 "s59E5H2" 0 "60 kDa chaperonin"
"Chl_A19_00301" 301 "s63E6H2" 0 "60 kDa chaperonin"

"Chl_A19_00302" 302 "s59E13H2" 0 "Protease 3 precursor"
"Chl_A19_00302" 302 "s59E25H2" 0 "Protease 3 precursor"
"Chl_A19_00302" 302 "s59E5H2" 0 "Protease 3 precursor"
"Chl_A19_00302" 302 "s63E6H2" 0 "Protease 3 precursor"
"Chl_A19_00303" 303 "s59E13H2" 0 "Putative prophage phiRv2 integrase"
"Chl_A19_00303" 303 "s59E25H2" 0 "Putative prophage phiRv2 integrase"
"Chl_A19_00303" 303 "s59E5H2" 0 "Putative prophage phiRv2 integrase"
"Chl_A19_00303" 303 "s63E6H2" 0 "Putative prophage phiRv2 integrase"
"Chl_A19_00304" 304 "s59E13H2" 0 "hypothetical protein"
"Chl_A19_00304" 304 "s59E25H2" 0 "hypothetical protein"
"Chl_A19_00304" 304 "s59E5H2" 0 "hypothetical protein"
"Chl_A19_00304" 304 "s63E6H2" 0 "hypothetical protein"
"Chl_A19_00305" 305 "s59E13H2" 0 "Helix-turn-helix domain protein"
"Chl_A19_00305" 305 "s59E25H2" 0 "Helix-turn-helix domain protein"
"Chl_A19_00305" 305 "s59E5H2" 0 "Helix-turn-helix domain protein"
"Chl_A19_00305" 305 "s63E6H2" 0 "Helix-turn-helix domain protein"
"Chl_A19_00307" 307 "s59E13H2" 0 "Transcriptional regulatory protein DegU"
"Chl_A19_00307" 307 "s59E25H2" 0 "Transcriptional regulatory protein DegU"
"Chl_A19_00307" 307 "s59E5H2" 0 "Transcriptional regulatory protein DegU"
"Chl_A19_00307" 307 "s63E6H2" 0 "Transcriptional regulatory protein DegU"
"Chl_A19_00308" 308 "s59E13H2" 0 "hypothetical protein"
"Chl_A19_00308" 308 "s59E25H2" 0 "hypothetical protein"
"Chl_A19_00308" 308 "s59E5H2" 0 "hypothetical protein"
"Chl_A19_00308" 308 "s63E6H2" 0 "hypothetical protein"
"Chl_A19_00309" 309 "s59E13H2" 0 "hypothetical protein"
"Chl_A19_00309" 309 "s59E25H2" 0 "hypothetical protein"
"Chl_A19_00309" 309 "s59E5H2" 0 "hypothetical protein"
"Chl_A19_00309" 309 "s63E6H2" 0 "hypothetical protein"
"Chl_A19_00310" 310 "s59E13H2" 0 "hypothetical protein"
"Chl_A19_00310" 310 "s59E25H2" 0 "hypothetical protein"
"Chl_A19_00310" 310 "s59E5H2" 0 "hypothetical protein"
"Chl_A19_00310" 310 "s63E6H2" 0 "hypothetical protein"
"Chl_A19_00311" 311 "s59E13H2" 0 "hypothetical protein"
"Chl_A19_00311" 311 "s59E25H2" 0 "hypothetical protein"
"Chl_A19_00311" 311 "s59E5H2" 0 "hypothetical protein"
"Chl_A19_00311" 311 "s63E6H2" 0 "hypothetical protein"
"Chl_A19_00312" 312 "s59E13H2" 0 "Vegetative catalase"
"Chl_A19_00312" 312 "s59E25H2" 0 "Vegetative catalase"
"Chl_A19_00312" 312 "s59E5H2" 0 "Vegetative catalase"
"Chl_A19_00312" 312 "s63E6H2" 0 "Vegetative catalase"
"Chl_A19_00314" 314 "s59E13H2" 0 "Ribosomal RNA small subunit methyltransferase A"
"Chl_A19_00314" 314 "s59E25H2" 0 "Ribosomal RNA small subunit methyltransferase A"
"Chl_A19_00314" 314 "s59E5H2" 0 "Ribosomal RNA small subunit methyltransferase A"
"Chl_A19_00314" 314 "s63E6H2" 0 "Ribosomal RNA small subunit methyltransferase A"
"Chl_A19_00315" 315 "s59E13H2" 0 "Trigger factor"
"Chl_A19_00315" 315 "s59E25H2" 0 "Trigger factor"
"Chl_A19_00315" 315 "s59E5H2" 0 "Trigger factor"

"Chl_A19_00315" 315 "s63E6H2" 0 "Trigger factor"
"Chl_A19_00316" 316 "s59E13H2" 0 "3-hydroxypropionyl-coenzyme A dehydratase"
"Chl_A19_00316" 316 "s59E25H2" 0 "3-hydroxypropionyl-coenzyme A dehydratase"
"Chl_A19_00316" 316 "s59E5H2" 0 "3-hydroxypropionyl-coenzyme A dehydratase"
"Chl_A19_00316" 316 "s63E6H2" 0 "3-hydroxypropionyl-coenzyme A dehydratase"
"Chl_A19_00317" 317 "s59E13H2" 0 "3-hydroxypropionyl-coenzyme A dehydratase"
"Chl_A19_00317" 317 "s59E25H2" 0 "3-hydroxypropionyl-coenzyme A dehydratase"
"Chl_A19_00317" 317 "s59E5H2" 0 "3-hydroxypropionyl-coenzyme A dehydratase"
"Chl_A19_00317" 317 "s63E6H2" 0 "3-hydroxypropionyl-coenzyme A dehydratase"
"Chl_A19_00318" 318 "s59E13H2" 0 "Aspartate--tRNA ligase"
"Chl_A19_00318" 318 "s59E25H2" 0 "Aspartate--tRNA ligase"
"Chl_A19_00318" 318 "s59E5H2" 0 "Aspartate--tRNA ligase"
"Chl_A19_00318" 318 "s63E6H2" 0 "Aspartate--tRNA ligase"
"Chl_A19_00319" 319 "s59E13H2" 0 "hypothetical protein"
"Chl_A19_00319" 319 "s59E25H2" 0 "hypothetical protein"
"Chl_A19_00319" 319 "s59E5H2" 0 "hypothetical protein"
"Chl_A19_00319" 319 "s63E6H2" 0 "hypothetical protein"
"Chl_A19_00320" 320 "s59E13H2" 0 "hypothetical protein"
"Chl_A19_00320" 320 "s59E25H2" 0 "hypothetical protein"
"Chl_A19_00320" 320 "s59E5H2" 0 "hypothetical protein"
"Chl_A19_00320" 320 "s63E6H2" 0 "hypothetical protein"
"Chl_A19_00321" 321 "s59E13H2" 0 "ATP-dependent Clp protease proteolytic subunit"
"Chl_A19_00321" 321 "s59E25H2" 0 "ATP-dependent Clp protease proteolytic subunit"
"Chl_A19_00321" 321 "s59E5H2" 0 "ATP-dependent Clp protease proteolytic subunit"
"Chl_A19_00321" 321 "s63E6H2" 0 "ATP-dependent Clp protease proteolytic subunit"
"Chl_A19_00322" 322 "s59E13H2" 0 "PHP domain protein"
"Chl_A19_00322" 322 "s59E25H2" 0 "PHP domain protein"
"Chl_A19_00322" 322 "s59E5H2" 0 "PHP domain protein"
"Chl_A19_00322" 322 "s63E6H2" 0 "PHP domain protein"
"Chl_A19_00323" 323 "s59E13H2" 0 "Elongation factor P"
"Chl_A19_00323" 323 "s59E25H2" 0 "Elongation factor P"
"Chl_A19_00323" 323 "s59E5H2" 0 "Elongation factor P"
"Chl_A19_00323" 323 "s63E6H2" 0 "Elongation factor P"
"Chl_A19_00324" 324 "s59E13H2" 0 "Xaa-Pro dipeptidase"
"Chl_A19_00324" 324 "s59E25H2" 0 "Xaa-Pro dipeptidase"
"Chl_A19_00324" 324 "s59E5H2" 0 "Xaa-Pro dipeptidase"
"Chl_A19_00324" 324 "s63E6H2" 0 "Xaa-Pro dipeptidase"
"Chl_A19_00325" 325 "s59E13H2" 0 "Tyrosine recombinase XerD"
"Chl_A19_00325" 325 "s59E25H2" 0 "Tyrosine recombinase XerD"
"Chl_A19_00325" 325 "s59E5H2" 0 "Tyrosine recombinase XerD"
"Chl_A19_00325" 325 "s63E6H2" 0 "Tyrosine recombinase XerD"
"Chl_A19_00326" 326 "s59E13H2" 0 "DNA topoisomerase 1"
"Chl_A19_00326" 326 "s59E25H2" 0 "DNA topoisomerase 1"
"Chl_A19_00326" 326 "s59E5H2" 0 "DNA topoisomerase 1"
"Chl_A19_00326" 326 "s63E6H2" 0 "DNA topoisomerase 1"
"Chl_A19_00327" 327 "s59E13H2" 0 "hypothetical protein"
"Chl_A19_00327" 327 "s59E25H2" 0 "hypothetical protein"

"Chl_A19_00327" 327 "s59E5H2" 0 "hypothetical protein"
"Chl_A19_00327" 327 "s63E6H2" 0 "hypothetical protein"
"Chl_A19_00329" 329 "s59E13H2" 0 "4-diphosphocytidyl-2-C-methyl-D-erythritol kinase"
"Chl_A19_00329" 329 "s59E25H2" 0 "4-diphosphocytidyl-2-C-methyl-D-erythritol kinase"
"Chl_A19_00329" 329 "s59E5H2" 0 "4-diphosphocytidyl-2-C-methyl-D-erythritol kinase"
"Chl_A19_00329" 329 "s63E6H2" 0 "4-diphosphocytidyl-2-C-methyl-D-erythritol kinase"
"Chl_A19_00330" 330 "s59E13H2" 0 "hypothetical protein"
"Chl_A19_00330" 330 "s59E25H2" 0 "hypothetical protein"
"Chl_A19_00330" 330 "s59E5H2" 0 "hypothetical protein"
"Chl_A19_00330" 330 "s63E6H2" 0 "hypothetical protein"
"Chl_A19_00331" 331 "s59E13H2" 0 "Sialic acid-binding periplasmic protein SiaP precursor"
"Chl_A19_00331" 331 "s59E25H2" 0 "Sialic acid-binding periplasmic protein SiaP precursor"
"Chl_A19_00331" 331 "s59E5H2" 0 "Sialic acid-binding periplasmic protein SiaP precursor"
"Chl_A19_00331" 331 "s63E6H2" 0 "Sialic acid-binding periplasmic protein SiaP precursor"
"Chl_A19_00332" 332 "s59E13H2" 0 "2,3-diketo-L-gulonate TRAP transporter small permease protein YiaM"
"Chl_A19_00332" 332 "s59E25H2" 0 "2,3-diketo-L-gulonate TRAP transporter small permease protein YiaM"
"Chl_A19_00332" 332 "s59E5H2" 0 "2,3-diketo-L-gulonate TRAP transporter small permease protein YiaM"
"Chl_A19_00332" 332 "s63E6H2" 0 "2,3-diketo-L-gulonate TRAP transporter small permease protein YiaM"
"Chl_A19_00333" 333 "s59E13H2" 0 "Sialic acid TRAP transporter permease protein SiaT"
"Chl_A19_00333" 333 "s59E25H2" 0 "Sialic acid TRAP transporter permease protein SiaT"
"Chl_A19_00333" 333 "s59E5H2" 0 "Sialic acid TRAP transporter permease protein SiaT"
"Chl_A19_00333" 333 "s63E6H2" 0 "Sialic acid TRAP transporter permease protein SiaT"
"Chl_A19_00334" 334 "s59E13H2" 0 "2-methylcitrate dehydratase"
"Chl_A19_00334" 334 "s59E25H2" 0 "2-methylcitrate dehydratase"
"Chl_A19_00334" 334 "s59E5H2" 0 "2-methylcitrate dehydratase"
"Chl_A19_00334" 334 "s63E6H2" 0 "2-methylcitrate dehydratase"
"Chl_A19_00335" 335 "s59E13H2" 0 "hypothetical protein"
"Chl_A19_00335" 335 "s59E25H2" 0 "hypothetical protein"
"Chl_A19_00335" 335 "s59E5H2" 0 "hypothetical protein"
"Chl_A19_00335" 335 "s63E6H2" 0 "hypothetical protein"
"Chl_A19_00336" 336 "s59E13H2" 0 "Peptide deformylase 1"
"Chl_A19_00336" 336 "s59E25H2" 0 "Peptide deformylase 1"
"Chl_A19_00336" 336 "s59E5H2" 0 "Peptide deformylase 1"
"Chl_A19_00336" 336 "s63E6H2" 0 "Peptide deformylase 1"
"Chl_A19_00337" 337 "s59E13H2" 0 "Primosomal protein N'"
"Chl_A19_00337" 337 "s59E25H2" 0 "Primosomal protein N'"
"Chl_A19_00337" 337 "s59E5H2" 0 "Primosomal protein N'"
"Chl_A19_00337" 337 "s63E6H2" 0 "Primosomal protein N'"
"Chl_A19_00338" 338 "s59E13H2" 0 "50S ribosomal protein L19"
"Chl_A19_00338" 338 "s59E25H2" 0 "50S ribosomal protein L19"
"Chl_A19_00338" 338 "s59E5H2" 0 "50S ribosomal protein L19"
"Chl_A19_00338" 338 "s63E6H2" 0 "50S ribosomal protein L19"
"Chl_A19_00339" 339 "s59E13H2" 0 "tRNA (guanine-N(1)-)-methyltransferase"
"Chl_A19_00339" 339 "s59E25H2" 0 "tRNA (guanine-N(1)-)-methyltransferase"
"Chl_A19_00339" 339 "s59E5H2" 0 "tRNA (guanine-N(1)-)-methyltransferase"
"Chl_A19_00339" 339 "s63E6H2" 0 "tRNA (guanine-N(1)-)-methyltransferase"
"Chl_A19_00340" 340 "s59E13H2" 0 "cell division protein MraZ"

"Chl_A19_00340" 340 "s59E25H2" 0 "cell division protein MraZ"
"Chl_A19_00340" 340 "s59E5H2" 0 "cell division protein MraZ"
"Chl_A19_00340" 340 "s63E6H2" 0 "cell division protein MraZ"
"Chl_A19_00341" 341 "s59E13H2" 0 "Ribosomal RNA small subunit methyltransferase H"
"Chl_A19_00341" 341 "s59E25H2" 0 "Ribosomal RNA small subunit methyltransferase H"
"Chl_A19_00341" 341 "s59E5H2" 0 "Ribosomal RNA small subunit methyltransferase H"
"Chl_A19_00341" 341 "s63E6H2" 0 "Ribosomal RNA small subunit methyltransferase H"
"Chl_A19_00343" 343 "s59E13H2" 0 "Cell division protein FtsA"
"Chl_A19_00343" 343 "s59E25H2" 0 "Cell division protein FtsA"
"Chl_A19_00343" 343 "s59E5H2" 0 "Cell division protein FtsA"
"Chl_A19_00343" 343 "s63E6H2" 0 "Cell division protein FtsA"
"Chl_A19_00344" 344 "s59E13H2" 0 "Cell division protein FtsZ"
"Chl_A19_00344" 344 "s59E25H2" 0 "Cell division protein FtsZ"
"Chl_A19_00344" 344 "s59E5H2" 0 "Cell division protein FtsZ"
"Chl_A19_00344" 344 "s63E6H2" 0 "Cell division protein FtsZ"
"Chl_A19_00345" 345 "s59E13H2" 0 "DNA adenine methyltransferase YhdJ"
"Chl_A19_00345" 345 "s59E25H2" 0 "DNA adenine methyltransferase YhdJ"
"Chl_A19_00345" 345 "s59E5H2" 0 "DNA adenine methyltransferase YhdJ"
"Chl_A19_00345" 345 "s63E6H2" 0 "DNA adenine methyltransferase YhdJ"
"Chl_A19_00346" 346 "s59E13H2" 0 "Transposase DDE domain protein"
"Chl_A19_00346" 346 "s59E25H2" 0 "Transposase DDE domain protein"
"Chl_A19_00346" 346 "s59E5H2" 0 "Transposase DDE domain protein"
"Chl_A19_00346" 346 "s63E6H2" 0 "Transposase DDE domain protein"
"Chl_A19_00349" 349 "s59E13H2" 0 "hypothetical protein"
"Chl_A19_00349" 349 "s59E25H2" 0 "hypothetical protein"
"Chl_A19_00349" 349 "s59E5H2" 0 "hypothetical protein"
"Chl_A19_00349" 349 "s63E6H2" 0 "hypothetical protein"
"Chl_A19_00351" 351 "s59E13H2" 0 "Transcriptional repressor NrdR"
"Chl_A19_00351" 351 "s59E25H2" 0 "Transcriptional repressor NrdR"
"Chl_A19_00351" 351 "s59E5H2" 0 "Transcriptional repressor NrdR"
"Chl_A19_00351" 351 "s63E6H2" 0 "Transcriptional repressor NrdR"
"Chl_A19_00352" 352 "s59E13H2" 0 "Ribonucleoside-diphosphate reductase NrdZ"
"Chl_A19_00352" 352 "s59E25H2" 0 "Ribonucleoside-diphosphate reductase NrdZ"
"Chl_A19_00352" 352 "s59E5H2" 0 "Ribonucleoside-diphosphate reductase NrdZ"
"Chl_A19_00352" 352 "s63E6H2" 0 "Ribonucleoside-diphosphate reductase NrdZ"
"Chl_A19_00353" 353 "s59E13H2" 0 "NYN domain protein"
"Chl_A19_00353" 353 "s59E25H2" 0 "NYN domain protein"
"Chl_A19_00353" 353 "s59E5H2" 0 "NYN domain protein"
"Chl_A19_00353" 353 "s63E6H2" 0 "NYN domain protein"
"Chl_A19_00354" 354 "s59E13H2" 0 "Radical SAM superfamily protein"
"Chl_A19_00354" 354 "s59E25H2" 0 "Radical SAM superfamily protein"
"Chl_A19_00354" 354 "s59E5H2" 0 "Radical SAM superfamily protein"
"Chl_A19_00354" 354 "s63E6H2" 0.000580056127377272 "Radical SAM superfamily protein"
"Chl_A19_00355" 355 "s59E13H2" 0 "Chloroplast import component protein (Tic20)"
"Chl_A19_00355" 355 "s59E25H2" 0 "Chloroplast import component protein (Tic20)"
"Chl_A19_00355" 355 "s59E5H2" 0 "Chloroplast import component protein (Tic20)"
"Chl_A19_00355" 355 "s63E6H2" 0 "Chloroplast import component protein (Tic20)"

"Chl_A19_00356" 356 "s59E13H2" 0 "Yqey-like protein"
"Chl_A19_00356" 356 "s59E25H2" 0 "Yqey-like protein"
"Chl_A19_00356" 356 "s59E5H2" 0 "Yqey-like protein"
"Chl_A19_00356" 356 "s63E6H2" 0 "Yqey-like protein"
"Chl_A19_00357" 357 "s59E13H2" 0 "30S ribosomal protein S21"
"Chl_A19_00357" 357 "s59E25H2" 0 "30S ribosomal protein S21"
"Chl_A19_00357" 357 "s59E5H2" 0 "30S ribosomal protein S21"
"Chl_A19_00357" 357 "s63E6H2" 0 "30S ribosomal protein S21"
"Chl_A19_00358" 358 "s59E13H2" 0 "Acyl-CoA dehydrogenase"
"Chl_A19_00358" 358 "s59E25H2" 0 "Acyl-CoA dehydrogenase"
"Chl_A19_00358" 358 "s59E5H2" 0 "Acyl-CoA dehydrogenase"
"Chl_A19_00358" 358 "s63E6H2" 0 "Acyl-CoA dehydrogenase"
"Chl_A19_00359" 359 "s59E13H2" 0 "Methylmalonyl-CoA mutase large subunit"
"Chl_A19_00359" 359 "s59E25H2" 0 "Methylmalonyl-CoA mutase large subunit"
"Chl_A19_00359" 359 "s59E5H2" 0 "Methylmalonyl-CoA mutase large subunit"
"Chl_A19_00359" 359 "s63E6H2" 0 "Methylmalonyl-CoA mutase large subunit"
"Chl_A19_00360" 360 "s59E13H2" 0 "Methylmalonyl-CoA mutase"
"Chl_A19_00360" 360 "s59E25H2" 0 "Methylmalonyl-CoA mutase"
"Chl_A19_00360" 360 "s59E5H2" 0 "Methylmalonyl-CoA mutase"
"Chl_A19_00360" 360 "s63E6H2" 0 "Methylmalonyl-CoA mutase"
"Chl_A19_00361" 361 "s59E13H2" 0 "Acyl-CoA dehydrogenase"
"Chl_A19_00361" 361 "s59E25H2" 0 "Acyl-CoA dehydrogenase"
"Chl_A19_00361" 361 "s59E5H2" 0 "Acyl-CoA dehydrogenase"
"Chl_A19_00361" 361 "s63E6H2" 0 "Acyl-CoA dehydrogenase"
"Chl_A19_00362" 362 "s59E13H2" 0 "Acetylene hydratase"
"Chl_A19_00362" 362 "s59E25H2" 0 "Acetylene hydratase"
"Chl_A19_00362" 362 "s59E5H2" 0 "Acetylene hydratase"
"Chl_A19_00362" 362 "s63E6H2" 0 "Acetylene hydratase"
"Chl_A19_00363" 363 "s59E13H2" 0 "Modulator of FtsH protease HflK"
"Chl_A19_00363" 363 "s59E25H2" 0 "Modulator of FtsH protease HflK"
"Chl_A19_00363" 363 "s59E5H2" 0 "Modulator of FtsH protease HflK"
"Chl_A19_00363" 363 "s63E6H2" 0 "Modulator of FtsH protease HflK"
"Chl_A19_00364" 364 "s59E13H2" 0 "hypothetical protein"
"Chl_A19_00364" 364 "s59E25H2" 0 "hypothetical protein"
"Chl_A19_00364" 364 "s59E5H2" 0 "hypothetical protein"
"Chl_A19_00364" 364 "s63E6H2" 0 "hypothetical protein"
"Chl_A19_00365" 365 "s59E13H2" 0 "Diaminopimelate decarboxylase"
"Chl_A19_00365" 365 "s59E25H2" 0 "Diaminopimelate decarboxylase"
"Chl_A19_00365" 365 "s59E5H2" 0 "Diaminopimelate decarboxylase"
"Chl_A19_00365" 365 "s63E6H2" 0 "Diaminopimelate decarboxylase"
"Chl_A19_00366" 366 "s59E13H2" 0 "Acylamino-acid-releasing enzyme"
"Chl_A19_00366" 366 "s59E25H2" 0 "Acylamino-acid-releasing enzyme"
"Chl_A19_00366" 366 "s59E5H2" 0 "Acylamino-acid-releasing enzyme"
"Chl_A19_00366" 366 "s63E6H2" 0 "Acylamino-acid-releasing enzyme"
"Chl_A19_00367" 367 "s59E13H2" 0 "Transcriptional regulatory protein DegU"
"Chl_A19_00367" 367 "s59E25H2" 0 "Transcriptional regulatory protein DegU"
"Chl_A19_00367" 367 "s59E5H2" 0 "Transcriptional regulatory protein DegU"

"Chl_A19_00367" 367 "s63E6H2" 0 "Transcriptional regulatory protein DegU"
"Chl_A19_00368" 368 "s59E13H2" 0 "Transcriptional regulatory protein DegU"
"Chl_A19_00368" 368 "s59E25H2" 0 "Transcriptional regulatory protein DegU"
"Chl_A19_00368" 368 "s59E5H2" 0 "Transcriptional regulatory protein DegU"
"Chl_A19_00368" 368 "s63E6H2" 0 "Transcriptional regulatory protein DegU"
"Chl_A19_00369" 369 "s59E13H2" 0 "Oxygen sensor histidine kinase NreB"
"Chl_A19_00369" 369 "s59E25H2" 0 "Oxygen sensor histidine kinase NreB"
"Chl_A19_00369" 369 "s59E5H2" 0 "Oxygen sensor histidine kinase NreB"
"Chl_A19_00369" 369 "s63E6H2" 0 "Oxygen sensor histidine kinase NreB"
"Chl_A19_00370" 370 "s59E13H2" 0 "GH3 auxin-responsive promoter"
"Chl_A19_00370" 370 "s59E25H2" 0 "GH3 auxin-responsive promoter"
"Chl_A19_00370" 370 "s59E5H2" 0 "GH3 auxin-responsive promoter"
"Chl_A19_00370" 370 "s63E6H2" 0 "GH3 auxin-responsive promoter"
"Chl_A19_00371" 371 "s59E13H2" 0 "hypothetical protein"
"Chl_A19_00371" 371 "s59E25H2" 0 "hypothetical protein"
"Chl_A19_00371" 371 "s59E5H2" 0 "hypothetical protein"
"Chl_A19_00371" 371 "s63E6H2" 0 "hypothetical protein"
"Chl_A19_00372" 372 "s59E13H2" 0 "hypothetical protein"
"Chl_A19_00372" 372 "s59E25H2" 0 "hypothetical protein"
"Chl_A19_00372" 372 "s59E5H2" 0 "hypothetical protein"
"Chl_A19_00372" 372 "s63E6H2" 0 "hypothetical protein"
"Chl_A19_00373" 373 "s59E13H2" 0 "Type II secretion system protein E"
"Chl_A19_00373" 373 "s59E25H2" 0 "Type II secretion system protein E"
"Chl_A19_00373" 373 "s59E5H2" 0 "Type II secretion system protein E"
"Chl_A19_00373" 373 "s63E6H2" 0 "Type II secretion system protein E"
"Chl_A19_00374" 374 "s59E13H2" 0 "Putative type II secretion system protein F"
"Chl_A19_00374" 374 "s59E25H2" 0 "Putative type II secretion system protein F"
"Chl_A19_00374" 374 "s59E5H2" 0 "Putative type II secretion system protein F"
"Chl_A19_00374" 374 "s63E6H2" 0 "Putative type II secretion system protein F"
"Chl_A19_00375" 375 "s59E13H2" 0 "hypothetical protein"
"Chl_A19_00375" 375 "s59E25H2" 0 "hypothetical protein"
"Chl_A19_00375" 375 "s59E5H2" 0 "hypothetical protein"
"Chl_A19_00375" 375 "s63E6H2" 0 "hypothetical protein"
"Chl_A19_00376" 376 "s59E13H2" 0 "hypothetical protein"
"Chl_A19_00376" 376 "s59E25H2" 0 "hypothetical protein"
"Chl_A19_00376" 376 "s59E5H2" 0 "hypothetical protein"
"Chl_A19_00376" 376 "s63E6H2" 0 "hypothetical protein"
"Chl_A19_00377" 377 "s59E13H2" 0 "hypothetical protein"
"Chl_A19_00377" 377 "s59E25H2" 0 "hypothetical protein"
"Chl_A19_00377" 377 "s59E5H2" 0 "hypothetical protein"
"Chl_A19_00377" 377 "s63E6H2" 0 "hypothetical protein"
"Chl_A19_00378" 378 "s59E13H2" 0 "hypothetical protein"
"Chl_A19_00378" 378 "s59E25H2" 0 "hypothetical protein"
"Chl_A19_00378" 378 "s59E5H2" 0 "hypothetical protein"
"Chl_A19_00378" 378 "s63E6H2" 0 "hypothetical protein"
"Chl_A19_00379" 379 "s59E13H2" 0 "hypothetical protein"
"Chl_A19_00379" 379 "s59E25H2" 0 "hypothetical protein"

"Chl_A19_00379" 379 "s59E5H2" 0 "hypothetical protein"
"Chl_A19_00379" 379 "s63E6H2" 0 "hypothetical protein"
"Chl_A19_00380" 380 "s59E13H2" 0 "Competence protein A"
"Chl_A19_00380" 380 "s59E25H2" 0 "Competence protein A"
"Chl_A19_00380" 380 "s59E5H2" 0 "Competence protein A"
"Chl_A19_00380" 380 "s63E6H2" 0 "Competence protein A"
"Chl_A19_00382" 382 "s59E13H2" 0 "Fimbrial protein precursor"
"Chl_A19_00382" 382 "s59E25H2" 0 "Fimbrial protein precursor"
"Chl_A19_00382" 382 "s59E5H2" 0 "Fimbrial protein precursor"
"Chl_A19_00382" 382 "s63E6H2" 0 "Fimbrial protein precursor"
"Chl_A19_00383" 383 "s59E13H2" 0 "Fimbrial protein precursor"
"Chl_A19_00383" 383 "s59E25H2" 0 "Fimbrial protein precursor"
"Chl_A19_00383" 383 "s59E5H2" 0 "Fimbrial protein precursor"
"Chl_A19_00383" 383 "s63E6H2" 0 "Fimbrial protein precursor"
"Chl_A19_00384" 384 "s59E13H2" 0 "Transcriptional regulatory protein DegU"
"Chl_A19_00384" 384 "s59E25H2" 0 "Transcriptional regulatory protein DegU"
"Chl_A19_00384" 384 "s59E5H2" 0 "Transcriptional regulatory protein DegU"
"Chl_A19_00384" 384 "s63E6H2" 0 "Transcriptional regulatory protein DegU"
"Chl_A19_00385" 385 "s59E13H2" 0.00364932027961413 "Sensor histidine kinase LiaS"
"Chl_A19_00385" 385 "s59E25H2" 0.120748299319728 "Sensor histidine kinase LiaS"
"Chl_A19_00385" 385 "s59E5H2" 0 "Sensor histidine kinase LiaS"
"Chl_A19_00385" 385 "s63E6H2" 0 "Sensor histidine kinase LiaS"
"Chl_A19_00386" 386 "s59E13H2" 0 "Type 4 prepilin-like proteins leader peptide-processing enzyme"
"Chl_A19_00386" 386 "s59E25H2" 0 "Type 4 prepilin-like proteins leader peptide-processing enzyme"
"Chl_A19_00386" 386 "s59E5H2" 0.00337204037800897 "Type 4 prepilin-like proteins leader peptide-processing enzyme"
"Chl_A19_00386" 386 "s63E6H2" 0 "Type 4 prepilin-like proteins leader peptide-processing enzyme"
"Chl_A19_00387" 387 "s59E13H2" 0 "Signal peptidase I W"
"Chl_A19_00387" 387 "s59E25H2" 0 "Signal peptidase I W"
"Chl_A19_00387" 387 "s59E5H2" 0 "Signal peptidase I W"
"Chl_A19_00387" 387 "s63E6H2" 0 "Signal peptidase I W"
"Chl_A19_00388" 388 "s59E13H2" 0 "hypothetical protein"
"Chl_A19_00388" 388 "s59E25H2" 0 "hypothetical protein"
"Chl_A19_00388" 388 "s59E5H2" 0 "hypothetical protein"
"Chl_A19_00388" 388 "s63E6H2" 0 "hypothetical protein"
"Chl_A19_00390" 390 "s59E13H2" 0 "hypothetical protein"
"Chl_A19_00390" 390 "s59E25H2" 0 "hypothetical protein"
"Chl_A19_00390" 390 "s59E5H2" 0 "hypothetical protein"
"Chl_A19_00390" 390 "s63E6H2" 0 "hypothetical protein"
"Chl_A19_00391" 391 "s59E13H2" 0 "Transcriptional regulatory protein DegU"
"Chl_A19_00391" 391 "s59E25H2" 0 "Transcriptional regulatory protein DegU"
"Chl_A19_00391" 391 "s59E5H2" 0 "Transcriptional regulatory protein DegU"
"Chl_A19_00391" 391 "s63E6H2" 0 "Transcriptional regulatory protein DegU"
"Chl_A19_00392" 392 "s59E13H2" 0 "Nitrogenase molybdenum-iron protein alpha chain"
"Chl_A19_00392" 392 "s59E25H2" 0 "Nitrogenase molybdenum-iron protein alpha chain"
"Chl_A19_00392" 392 "s59E5H2" 0 "Nitrogenase molybdenum-iron protein alpha chain"
"Chl_A19_00392" 392 "s63E6H2" 0 "Nitrogenase molybdenum-iron protein alpha chain"
"Chl_A19_00393" 393 "s59E13H2" 0 "Nitrogenase molybdenum-iron protein alpha chain"

"Chl_A19_00393" 393 "s59E25H2" 0 "Nitrogenase molybdenum-iron protein alpha chain"
"Chl_A19_00393" 393 "s59E5H2" 0 "Nitrogenase molybdenum-iron protein alpha chain"
"Chl_A19_00393" 393 "s63E6H2" 0 "Nitrogenase molybdenum-iron protein alpha chain"
"Chl_A19_00394" 394 "s59E13H2" 0 "Succinyl-CoA:(R)-benzylsuccinate CoA-transferase subunit BbsE"
"Chl_A19_00394" 394 "s59E25H2" 0 "Succinyl-CoA:(R)-benzylsuccinate CoA-transferase subunit BbsE"
"Chl_A19_00394" 394 "s59E5H2" 0 "Succinyl-CoA:(R)-benzylsuccinate CoA-transferase subunit BbsE"
"Chl_A19_00394" 394 "s63E6H2" 0 "Succinyl-CoA:(R)-benzylsuccinate CoA-transferase subunit BbsE"
"Chl_A19_00395" 395 "s59E13H2" 0 "Succinyl-CoA:(R)-benzylsuccinate CoA-transferase subunit BbsF"
"Chl_A19_00395" 395 "s59E25H2" 0 "Succinyl-CoA:(R)-benzylsuccinate CoA-transferase subunit BbsF"
"Chl_A19_00395" 395 "s59E5H2" 0 "Succinyl-CoA:(R)-benzylsuccinate CoA-transferase subunit BbsF"
"Chl_A19_00395" 395 "s63E6H2" 0 "Succinyl-CoA:(R)-benzylsuccinate CoA-transferase subunit BbsF"
"Chl_A19_00396" 396 "s59E13H2" 0 "3-dehydroquinate synthase"
"Chl_A19_00396" 396 "s59E25H2" 0 "3-dehydroquinate synthase"
"Chl_A19_00396" 396 "s59E5H2" 0 "3-dehydroquinate synthase"
"Chl_A19_00396" 396 "s63E6H2" 0 "3-dehydroquinate synthase"
"Chl_A19_00397" 397 "s59E13H2" 0 "3-dehydroquinate dehydratase"
"Chl_A19_00397" 397 "s59E25H2" 0 "3-dehydroquinate dehydratase"
"Chl_A19_00397" 397 "s59E5H2" 0 "3-dehydroquinate dehydratase"
"Chl_A19_00397" 397 "s63E6H2" 0 "3-dehydroquinate dehydratase"
"Chl_A19_00398" 398 "s59E13H2" 0 "Shikimate dehydrogenase"
"Chl_A19_00398" 398 "s59E25H2" 0 "Shikimate dehydrogenase"
"Chl_A19_00398" 398 "s59E5H2" 0 "Shikimate dehydrogenase"
"Chl_A19_00398" 398 "s63E6H2" 0 "Shikimate dehydrogenase"
"Chl_A19_00399" 399 "s59E13H2" 0 "Shikimate kinase"
"Chl_A19_00399" 399 "s59E25H2" 0 "Shikimate kinase"
"Chl_A19_00399" 399 "s59E5H2" 0 "Shikimate kinase"
"Chl_A19_00399" 399 "s63E6H2" 0 "Shikimate kinase"
"Chl_A19_00400" 400 "s59E13H2" 0 "3-phosphoshikimate 1-carboxyvinyltransferase"
"Chl_A19_00400" 400 "s59E25H2" 0 "3-phosphoshikimate 1-carboxyvinyltransferase"
"Chl_A19_00400" 400 "s59E5H2" 0 "3-phosphoshikimate 1-carboxyvinyltransferase"
"Chl_A19_00400" 400 "s63E6H2" 0 "3-phosphoshikimate 1-carboxyvinyltransferase"
"Chl_A19_00401" 401 "s59E13H2" 0 "Chorismate synthase"
"Chl_A19_00401" 401 "s59E25H2" 0 "Chorismate synthase"
"Chl_A19_00401" 401 "s59E5H2" 0 "Chorismate synthase"
"Chl_A19_00401" 401 "s63E6H2" 0 "Chorismate synthase"
"Chl_A19_00402" 402 "s59E13H2" 0 "P-protein"
"Chl_A19_00402" 402 "s59E25H2" 0 "P-protein"
"Chl_A19_00402" 402 "s59E5H2" 0 "P-protein"
"Chl_A19_00402" 402 "s63E6H2" 0 "P-protein"
"Chl_A19_00403" 403 "s59E13H2" 0 "T-protein"
"Chl_A19_00403" 403 "s59E25H2" 0 "T-protein"
"Chl_A19_00403" 403 "s59E5H2" 0 "T-protein"
"Chl_A19_00403" 403 "s63E6H2" 0 "T-protein"
"Chl_A19_00404" 404 "s59E13H2" 0 "hypothetical protein"
"Chl_A19_00404" 404 "s59E25H2" 0 "hypothetical protein"
"Chl_A19_00404" 404 "s59E5H2" 0 "hypothetical protein"
"Chl_A19_00404" 404 "s63E6H2" 0 "hypothetical protein"

"Chl_A19_00405" 405 "s59E13H2" 0 "hypothetical protein"
"Chl_A19_00405" 405 "s59E25H2" 0 "hypothetical protein"
"Chl_A19_00405" 405 "s59E5H2" 0 "hypothetical protein"
"Chl_A19_00405" 405 "s63E6H2" 0 "hypothetical protein"
"Chl_A19_00406" 406 "s59E13H2" 0 "putative dimethyl sulfoxide reductase chain YnfF precursor"
"Chl_A19_00406" 406 "s59E25H2" 0 "putative dimethyl sulfoxide reductase chain YnfF precursor"
"Chl_A19_00406" 406 "s59E5H2" 0 "putative dimethyl sulfoxide reductase chain YnfF precursor"
"Chl_A19_00406" 406 "s63E6H2" 0 "putative dimethyl sulfoxide reductase chain YnfF precursor"
"Chl_A19_00407" 407 "s59E13H2" 0 "Sulfide dehydrogenase subunit alpha precursor"
"Chl_A19_00407" 407 "s59E25H2" 0 "Sulfide dehydrogenase subunit alpha precursor"
"Chl_A19_00407" 407 "s59E5H2" 0 "Sulfide dehydrogenase subunit alpha precursor"
"Chl_A19_00407" 407 "s63E6H2" 0 "Sulfide dehydrogenase subunit alpha precursor"
"Chl_A19_00408" 408 "s59E13H2" 0 "Carbamoyl-phosphate synthase large chain"
"Chl_A19_00408" 408 "s59E25H2" 0 "Carbamoyl-phosphate synthase large chain"
"Chl_A19_00408" 408 "s59E5H2" 0 "Carbamoyl-phosphate synthase large chain"
"Chl_A19_00408" 408 "s63E6H2" 0 "Carbamoyl-phosphate synthase large chain"
"Chl_A19_00409" 409 "s59E13H2" 0 "hypothetical protein"
"Chl_A19_00409" 409 "s59E25H2" 0 "hypothetical protein"
"Chl_A19_00409" 409 "s59E5H2" 0 "hypothetical protein"
"Chl_A19_00409" 409 "s63E6H2" 0 "hypothetical protein"
"Chl_A19_00410" 410 "s59E13H2" 0 "CoB--CoM heterodisulfide reductase iron-sulfur subunit D"
"Chl_A19_00410" 410 "s59E25H2" 0 "CoB--CoM heterodisulfide reductase iron-sulfur subunit D"
"Chl_A19_00410" 410 "s59E5H2" 0 "CoB--CoM heterodisulfide reductase iron-sulfur subunit D"
"Chl_A19_00410" 410 "s63E6H2" 0 "CoB--CoM heterodisulfide reductase iron-sulfur subunit D"
"Chl_A19_00411" 411 "s59E13H2" 0 "putative HTH-type transcriptional regulator/MT0088"
"Chl_A19_00411" 411 "s59E25H2" 0 "putative HTH-type transcriptional regulator/MT0088"
"Chl_A19_00411" 411 "s59E5H2" 0 "putative HTH-type transcriptional regulator/MT0088"
"Chl_A19_00411" 411 "s63E6H2" 0 "putative HTH-type transcriptional regulator/MT0088"
"Chl_A19_00412" 412 "s59E13H2" 0 "hypothetical protein"
"Chl_A19_00412" 412 "s59E25H2" 0 "hypothetical protein"
"Chl_A19_00412" 412 "s59E5H2" 0 "hypothetical protein"
"Chl_A19_00412" 412 "s63E6H2" 0 "hypothetical protein"
"Chl_A19_00413" 413 "s59E13H2" 0 "30S ribosomal protein S21"
"Chl_A19_00413" 413 "s59E25H2" 0 "30S ribosomal protein S21"
"Chl_A19_00413" 413 "s59E5H2" 0 "30S ribosomal protein S21"
"Chl_A19_00413" 413 "s63E6H2" 0 "30S ribosomal protein S21"
"Chl_A19_00414" 414 "s59E13H2" 0 "RNA recognition motif. (a.k.a. RRM, RBD, or RNP domain)"
"Chl_A19_00414" 414 "s59E25H2" 0 "RNA recognition motif. (a.k.a. RRM, RBD, or RNP domain)"
"Chl_A19_00414" 414 "s59E5H2" 0 "RNA recognition motif. (a.k.a. RRM, RBD, or RNP domain)"
"Chl_A19_00414" 414 "s63E6H2" 0 "RNA recognition motif. (a.k.a. RRM, RBD, or RNP domain)"
"Chl_A19_00415" 415 "s59E13H2" 0 "Iron-sulphur cluster biosynthesis"
"Chl_A19_00415" 415 "s59E25H2" 0 "Iron-sulphur cluster biosynthesis"
"Chl_A19_00415" 415 "s59E5H2" 0 "Iron-sulphur cluster biosynthesis"
"Chl_A19_00415" 415 "s63E6H2" 0 "Iron-sulphur cluster biosynthesis"
"Chl_A19_00417" 417 "s59E13H2" 0 "Multidrug resistance protein stp"
"Chl_A19_00417" 417 "s59E25H2" 0 "Multidrug resistance protein stp"
"Chl_A19_00417" 417 "s59E5H2" 0 "Multidrug resistance protein stp"

"Chl_A19_00417" 417 "s63E6H2" 0 "Multidrug resistance protein stp"
"Chl_A19_00418" 418 "s59E13H2" 0 "Phosphoribosylamine--glycine ligase"
"Chl_A19_00418" 418 "s59E25H2" 0 "Phosphoribosylamine--glycine ligase"
"Chl_A19_00418" 418 "s59E5H2" 0 "Phosphoribosylamine--glycine ligase"
"Chl_A19_00418" 418 "s63E6H2" 0 "Phosphoribosylamine--glycine ligase"
"Chl_A19_00419" 419 "s59E13H2" 0 "hypothetical protein"
"Chl_A19_00419" 419 "s59E25H2" 0 "hypothetical protein"
"Chl_A19_00419" 419 "s59E5H2" 0 "hypothetical protein"
"Chl_A19_00419" 419 "s63E6H2" 0 "hypothetical protein"
"Chl_A19_00420" 420 "s59E13H2" 0 "NAD-dependent alcohol dehydrogenase"
"Chl_A19_00420" 420 "s59E25H2" 0 "NAD-dependent alcohol dehydrogenase"
"Chl_A19_00420" 420 "s59E5H2" 0.0138672666432913 "NAD-dependent alcohol dehydrogenase"
"Chl_A19_00420" 420 "s63E6H2" 0.00291182381176883 "NAD-dependent alcohol dehydrogenase"
"Chl_A19_00421" 421 "s59E13H2" 0 "Alcohol dehydrogenase"
"Chl_A19_00421" 421 "s59E25H2" 0 "Alcohol dehydrogenase"
"Chl_A19_00421" 421 "s59E5H2" 0.0041441034219278 "Alcohol dehydrogenase"
"Chl_A19_00421" 421 "s63E6H2" 0.0135820528128397 "Alcohol dehydrogenase"
"Chl_A19_00422" 422 "s59E13H2" 0 "GMP synthase [glutamine-hydrolyzing]"
"Chl_A19_00422" 422 "s59E25H2" 0 "GMP synthase [glutamine-hydrolyzing]"
"Chl_A19_00422" 422 "s59E5H2" 0 "GMP synthase [glutamine-hydrolyzing]"
"Chl_A19_00422" 422 "s63E6H2" 0 "GMP synthase [glutamine-hydrolyzing]"
"Chl_A19_00424" 424 "s59E13H2" 0 "hypothetical protein"
"Chl_A19_00424" 424 "s59E25H2" 0 "hypothetical protein"
"Chl_A19_00424" 424 "s59E5H2" 0 "hypothetical protein"
"Chl_A19_00424" 424 "s63E6H2" 0 "hypothetical protein"
"Chl_A19_00425" 425 "s59E13H2" 0 "Helix-turn-helix domain protein"
"Chl_A19_00425" 425 "s59E25H2" 0 "Helix-turn-helix domain protein"
"Chl_A19_00425" 425 "s59E5H2" 0 "Helix-turn-helix domain protein"
"Chl_A19_00425" 425 "s63E6H2" 0 "Helix-turn-helix domain protein"
"Chl_A19_00426" 426 "s59E13H2" 0 "Glycerol-3-phosphate acyltransferase"
"Chl_A19_00426" 426 "s59E25H2" 0 "Glycerol-3-phosphate acyltransferase"
"Chl_A19_00426" 426 "s59E5H2" 0 "Glycerol-3-phosphate acyltransferase"
"Chl_A19_00426" 426 "s63E6H2" 0 "Glycerol-3-phosphate acyltransferase"
"Chl_A19_00427" 427 "s59E13H2" 0 "hypothetical protein"
"Chl_A19_00427" 427 "s59E25H2" 0 "hypothetical protein"
"Chl_A19_00427" 427 "s59E5H2" 0 "hypothetical protein"
"Chl_A19_00427" 427 "s63E6H2" 0 "hypothetical protein"
"Chl_A19_00428" 428 "s59E13H2" 0 "1-deoxy-D-xylulose-5-phosphate synthase"
"Chl_A19_00428" 428 "s59E25H2" 0 "1-deoxy-D-xylulose-5-phosphate synthase"
"Chl_A19_00428" 428 "s59E5H2" 0 "1-deoxy-D-xylulose-5-phosphate synthase"
"Chl_A19_00428" 428 "s63E6H2" 0 "1-deoxy-D-xylulose-5-phosphate synthase"
"Chl_A19_00429" 429 "s59E13H2" 0 "Transketolase 2"
"Chl_A19_00429" 429 "s59E25H2" 0 "Transketolase 2"
"Chl_A19_00429" 429 "s59E5H2" 0 "Transketolase 2"
"Chl_A19_00429" 429 "s63E6H2" 0 "Transketolase 2"
"Chl_A19_00431" 431 "s59E13H2" 0 "Pyrogallol hydroxytransferase large subunit"
"Chl_A19_00431" 431 "s59E25H2" 0 "Pyrogallol hydroxytransferase large subunit"

"Chl_A19_00431" 431 "s59E5H2" 0 "Pyrogallol hydroxytransferase large subunit"
"Chl_A19_00431" 431 "s63E6H2" 0 "Pyrogallol hydroxytransferase large subunit"
"Chl_A19_00432" 432 "s59E13H2" 0 "Pyrogallol hydroxytransferase large subunit"
"Chl_A19_00432" 432 "s59E25H2" 0 "Pyrogallol hydroxytransferase large subunit"
"Chl_A19_00432" 432 "s59E5H2" 0 "Pyrogallol hydroxytransferase large subunit"
"Chl_A19_00432" 432 "s63E6H2" 0 "Pyrogallol hydroxytransferase large subunit"
"Chl_A19_00433" 433 "s59E13H2" 0 "RDD family protein"
"Chl_A19_00433" 433 "s59E25H2" 0 "RDD family protein"
"Chl_A19_00433" 433 "s59E5H2" 0 "RDD family protein"
"Chl_A19_00433" 433 "s63E6H2" 0 "RDD family protein"
"Chl_A19_00434" 434 "s59E13H2" 0 "hypothetical protein"
"Chl_A19_00434" 434 "s59E25H2" 0 "hypothetical protein"
"Chl_A19_00434" 434 "s59E5H2" 0 "hypothetical protein"
"Chl_A19_00434" 434 "s63E6H2" 0 "hypothetical protein"
"Chl_A19_00435" 435 "s59E13H2" 0 "hypothetical protein"
"Chl_A19_00435" 435 "s59E25H2" 0 "hypothetical protein"
"Chl_A19_00435" 435 "s59E5H2" 0 "hypothetical protein"
"Chl_A19_00435" 435 "s63E6H2" 0 "hypothetical protein"
"Chl_A19_00436" 436 "s59E13H2" 0 "PD-(D/E)XK nuclease superfamily protein"
"Chl_A19_00436" 436 "s59E25H2" 0 "PD-(D/E)XK nuclease superfamily protein"
"Chl_A19_00436" 436 "s59E5H2" 0 "PD-(D/E)XK nuclease superfamily protein"
"Chl_A19_00436" 436 "s63E6H2" 0 "PD-(D/E)XK nuclease superfamily protein"
"Chl_A19_00437" 437 "s59E13H2" 0 "hypothetical protein"
"Chl_A19_00437" 437 "s59E25H2" 0 "hypothetical protein"
"Chl_A19_00437" 437 "s59E5H2" 0 "hypothetical protein"
"Chl_A19_00437" 437 "s63E6H2" 0 "hypothetical protein"
"Chl_A19_00438" 438 "s59E13H2" 0 "Tyrosine recombinase XerD"
"Chl_A19_00438" 438 "s59E25H2" 0 "Tyrosine recombinase XerD"
"Chl_A19_00438" 438 "s59E5H2" 0 "Tyrosine recombinase XerD"
"Chl_A19_00438" 438 "s63E6H2" 0 "Tyrosine recombinase XerD"
"Chl_A19_00439" 439 "s59E13H2" 0 "hypothetical protein"
"Chl_A19_00439" 439 "s59E25H2" 0 "hypothetical protein"
"Chl_A19_00439" 439 "s59E5H2" 0 "hypothetical protein"
"Chl_A19_00439" 439 "s63E6H2" 0 "hypothetical protein"
"Chl_A19_00440" 440 "s59E13H2" 0 "Protein-L-isoaspartate O-methyltransferase"
"Chl_A19_00440" 440 "s59E25H2" 0 "Protein-L-isoaspartate O-methyltransferase"
"Chl_A19_00440" 440 "s59E5H2" 0 "Protein-L-isoaspartate O-methyltransferase"
"Chl_A19_00440" 440 "s63E6H2" 0 "Protein-L-isoaspartate O-methyltransferase"
"Chl_A19_00441" 441 "s59E13H2" 0 "Glyoxalase-like domain protein"
"Chl_A19_00441" 441 "s59E25H2" 0 "Glyoxalase-like domain protein"
"Chl_A19_00441" 441 "s59E5H2" 0 "Glyoxalase-like domain protein"
"Chl_A19_00441" 441 "s63E6H2" 0 "Glyoxalase-like domain protein"
"Chl_A19_00442" 442 "s59E13H2" 0 "5,6-dimethylbenzimidazole synthase"
"Chl_A19_00442" 442 "s59E25H2" 0 "5,6-dimethylbenzimidazole synthase"
"Chl_A19_00442" 442 "s59E5H2" 0 "5,6-dimethylbenzimidazole synthase"
"Chl_A19_00442" 442 "s63E6H2" 0 "5,6-dimethylbenzimidazole synthase"
"Chl_A19_00443" 443 "s59E13H2" 0 "hypothetical protein"

"Chl_A19_00443" 443 "s59E25H2" 0 "hypothetical protein"
"Chl_A19_00443" 443 "s59E5H2" 0 "hypothetical protein"
"Chl_A19_00443" 443 "s63E6H2" 0 "hypothetical protein"
"Chl_A19_00444" 444 "s59E13H2" 0 "hypothetical protein"
"Chl_A19_00444" 444 "s59E25H2" 0 "hypothetical protein"
"Chl_A19_00444" 444 "s59E5H2" 0 "hypothetical protein"
"Chl_A19_00444" 444 "s63E6H2" 0 "hypothetical protein"
"Chl_A19_00445" 445 "s59E13H2" 0 "Transglutaminase-like superfamily protein"
"Chl_A19_00445" 445 "s59E25H2" 0 "Transglutaminase-like superfamily protein"
"Chl_A19_00445" 445 "s59E5H2" 0 "Transglutaminase-like superfamily protein"
"Chl_A19_00445" 445 "s63E6H2" 0 "Transglutaminase-like superfamily protein"
"Chl_A19_00446" 446 "s59E13H2" 0 "4-hydroxybutyryl-CoA dehydratase/vinylacetyl-CoA-Delta-isomerase"
"Chl_A19_00446" 446 "s59E25H2" 0 "4-hydroxybutyryl-CoA dehydratase/vinylacetyl-CoA-Delta-isomerase"
"Chl_A19_00446" 446 "s59E5H2" 0 "4-hydroxybutyryl-CoA dehydratase/vinylacetyl-CoA-Delta-isomerase"
"Chl_A19_00446" 446 "s63E6H2" 0 "4-hydroxybutyryl-CoA dehydratase/vinylacetyl-CoA-Delta-isomerase"
"Chl_A19_00447" 447 "s59E13H2" 0 "Elongation factor G"
"Chl_A19_00447" 447 "s59E25H2" 0 "Elongation factor G"
"Chl_A19_00447" 447 "s59E5H2" 0 "Elongation factor G"
"Chl_A19_00447" 447 "s63E6H2" 0 "Elongation factor G"
"Chl_A19_00448" 448 "s59E13H2" 0 "Acryloyl-CoA reductase electron transfer subunit beta"
"Chl_A19_00448" 448 "s59E25H2" 0 "Acryloyl-CoA reductase electron transfer subunit beta"
"Chl_A19_00448" 448 "s59E5H2" 0 "Acryloyl-CoA reductase electron transfer subunit beta"
"Chl_A19_00448" 448 "s63E6H2" 0 "Acryloyl-CoA reductase electron transfer subunit beta"
"Chl_A19_00449" 449 "s59E13H2" 0 "UDP-glucose 4-epimerase"
"Chl_A19_00449" 449 "s59E25H2" 0 "UDP-glucose 4-epimerase"
"Chl_A19_00449" 449 "s59E5H2" 0 "UDP-glucose 4-epimerase"
"Chl_A19_00449" 449 "s63E6H2" 0 "UDP-glucose 4-epimerase"
"Chl_A19_00450" 450 "s59E13H2" 0 "UDP-glucose 4-epimerase"
"Chl_A19_00450" 450 "s59E25H2" 0 "UDP-glucose 4-epimerase"
"Chl_A19_00450" 450 "s59E5H2" 0 "UDP-glucose 4-epimerase"
"Chl_A19_00450" 450 "s63E6H2" 0 "UDP-glucose 4-epimerase"
"Chl_A19_00451" 451 "s59E13H2" 0 "GDP-mannose-dependent alpha-(1-6)-phosphatidylinositol monomannoside mannosyltransferase"
"Chl_A19_00451" 451 "s59E25H2" 0 "GDP-mannose-dependent alpha-(1-6)-phosphatidylinositol monomannoside mannosyltransferase"
"Chl_A19_00451" 451 "s59E5H2" 0 "GDP-mannose-dependent alpha-(1-6)-phosphatidylinositol monomannoside mannosyltransferase"
"Chl_A19_00451" 451 "s63E6H2" 0 "GDP-mannose-dependent alpha-(1-6)-phosphatidylinositol monomannoside mannosyltransferase"
"Chl_A19_00452" 452 "s59E13H2" 0 "coproporphyrinogen III oxidase"
"Chl_A19_00452" 452 "s59E25H2" 0 "coproporphyrinogen III oxidase"
"Chl_A19_00452" 452 "s59E5H2" 0 "coproporphyrinogen III oxidase"
"Chl_A19_00452" 452 "s63E6H2" 0 "coproporphyrinogen III oxidase"
"Chl_A19_00453" 453 "s59E13H2" 0 "dTDP-glucose 4,6-dehydratase"
"Chl_A19_00453" 453 "s59E25H2" 0 "dTDP-glucose 4,6-dehydratase"
"Chl_A19_00453" 453 "s59E5H2" 0 "dTDP-glucose 4,6-dehydratase"
"Chl_A19_00453" 453 "s63E6H2" 0 "dTDP-glucose 4,6-dehydratase"
"Chl_A19_00454" 454 "s59E13H2" 0 "UDP-N-acetylglucosamine 2-epimerase"
"Chl_A19_00454" 454 "s59E25H2" 0 "UDP-N-acetylglucosamine 2-epimerase"
"Chl_A19_00454" 454 "s59E5H2" 0 "UDP-N-acetylglucosamine 2-epimerase"
"Chl_A19_00454" 454 "s63E6H2" 0 "UDP-N-acetylglucosamine 2-epimerase"

"Chl_A19_00455" 455 "s59E13H2" 0 "hypothetical protein"
"Chl_A19_00455" 455 "s59E25H2" 0 "hypothetical protein"
"Chl_A19_00455" 455 "s59E5H2" 0 "hypothetical protein"
"Chl_A19_00455" 455 "s63E6H2" 0 "hypothetical protein"
"Chl_A19_00456" 456 "s59E13H2" 0 "Glycosyltransferase AglJ"
"Chl_A19_00456" 456 "s59E25H2" 0 "Glycosyltransferase AglJ"
"Chl_A19_00456" 456 "s59E5H2" 0 "Glycosyltransferase AglJ"
"Chl_A19_00456" 456 "s63E6H2" 0 "Glycosyltransferase AglJ"
"Chl_A19_00457" 457 "s59E13H2" 0 "hypothetical protein"
"Chl_A19_00457" 457 "s59E25H2" 0 "hypothetical protein"
"Chl_A19_00457" 457 "s59E5H2" 0 "hypothetical protein"
"Chl_A19_00457" 457 "s63E6H2" 0 "hypothetical protein"
"Chl_A19_00458" 458 "s59E13H2" 0 "Benzylsuccinate synthase alpha subunit"
"Chl_A19_00458" 458 "s59E25H2" 0 "Benzylsuccinate synthase alpha subunit"
"Chl_A19_00458" 458 "s59E5H2" 0 "Benzylsuccinate synthase alpha subunit"
"Chl_A19_00458" 458 "s63E6H2" 0 "Benzylsuccinate synthase alpha subunit"
"Chl_A19_00459" 459 "s59E13H2" 0 "4-hydroxyphenylacetate decarboxylase activating enzyme"
"Chl_A19_00459" 459 "s59E25H2" 0 "4-hydroxyphenylacetate decarboxylase activating enzyme"
"Chl_A19_00459" 459 "s59E5H2" 0 "4-hydroxyphenylacetate decarboxylase activating enzyme"
"Chl_A19_00459" 459 "s63E6H2" 0 "4-hydroxyphenylacetate decarboxylase activating enzyme"
"Chl_A19_00460" 460 "s59E13H2" 0 "Isoprenylcysteine carboxyl methyltransferase (ICMT) family protein"
"Chl_A19_00460" 460 "s59E25H2" 0 "Isoprenylcysteine carboxyl methyltransferase (ICMT) family protein"
"Chl_A19_00460" 460 "s59E5H2" 0 "Isoprenylcysteine carboxyl methyltransferase (ICMT) family protein"
"Chl_A19_00460" 460 "s63E6H2" 0 "Isoprenylcysteine carboxyl methyltransferase (ICMT) family protein"
"Chl_A19_00461" 461 "s59E13H2" 0 "hypothetical protein"
"Chl_A19_00461" 461 "s59E25H2" 0 "hypothetical protein"
"Chl_A19_00461" 461 "s59E5H2" 0 "hypothetical protein"
"Chl_A19_00461" 461 "s63E6H2" 0 "hypothetical protein"
"Chl_A19_00462" 462 "s59E13H2" 0 "hypothetical protein"
"Chl_A19_00462" 462 "s59E25H2" 0 "hypothetical protein"
"Chl_A19_00462" 462 "s59E5H2" 0 "hypothetical protein"
"Chl_A19_00462" 462 "s63E6H2" 0 "hypothetical protein"
"Chl_A19_00465" 465 "s59E13H2" 0 "Undecaprenyl-phosphate mannosyltransferase"
"Chl_A19_00465" 465 "s59E25H2" 0 "Undecaprenyl-phosphate mannosyltransferase"
"Chl_A19_00465" 465 "s59E5H2" 0 "Undecaprenyl-phosphate mannosyltransferase"
"Chl_A19_00465" 465 "s63E6H2" 0 "Undecaprenyl-phosphate mannosyltransferase"
"Chl_A19_00466" 466 "s59E13H2" 0 "hypothetical protein"
"Chl_A19_00466" 466 "s59E25H2" 0 "hypothetical protein"
"Chl_A19_00466" 466 "s59E5H2" 0 "hypothetical protein"
"Chl_A19_00466" 466 "s63E6H2" 0 "hypothetical protein"
"Chl_A19_00467" 467 "s59E13H2" 0 "hypothetical protein"
"Chl_A19_00467" 467 "s59E25H2" 0 "hypothetical protein"
"Chl_A19_00467" 467 "s59E5H2" 0.208246564961486 "hypothetical protein"
"Chl_A19_00467" 467 "s63E6H2" 0 "hypothetical protein"
"Chl_A19_00470" 470 "s59E13H2" 0.00102728752596264 "Tryptophan--tRNA ligase"
"Chl_A19_00470" 470 "s59E25H2" 0 "Tryptophan--tRNA ligase"
"Chl_A19_00470" 470 "s59E5H2" 0 "Tryptophan--tRNA ligase"

"Chl_A19_00470" 470 "s63E6H2" 0 "Tryptophan--tRNA ligase"
"Chl_A19_00471" 471 "s59E13H2" 0 "Methylmalonyl-CoA carboxyltransferase 12S subunit"
"Chl_A19_00471" 471 "s59E25H2" 0 "Methylmalonyl-CoA carboxyltransferase 12S subunit"
"Chl_A19_00471" 471 "s59E5H2" 0 "Methylmalonyl-CoA carboxyltransferase 12S subunit"
"Chl_A19_00471" 471 "s63E6H2" 0 "Methylmalonyl-CoA carboxyltransferase 12S subunit"
"Chl_A19_00472" 472 "s59E13H2" 0 "1-(5-phosphoribosyl)-5-[(5-phosphoribosylamino)methylideneamino] imidazole-4-carboxamide isomerase"
"Chl_A19_00472" 472 "s59E25H2" 0 "1-(5-phosphoribosyl)-5-[(5-phosphoribosylamino)methylideneamino] imidazole-4-carboxamide isomerase"
"Chl_A19_00472" 472 "s59E5H2" 0 "1-(5-phosphoribosyl)-5-[(5-phosphoribosylamino)methylideneamino] imidazole-4-carboxamide isomerase"
"Chl_A19_00472" 472 "s63E6H2" 0 "1-(5-phosphoribosyl)-5-[(5-phosphoribosylamino)methylideneamino] imidazole-4-carboxamide isomerase"
"Chl_A19_00473" 473 "s59E13H2" 0 "Phosphoribosyl-AMP cyclohydrolase"
"Chl_A19_00473" 473 "s59E25H2" 0 "Phosphoribosyl-AMP cyclohydrolase"
"Chl_A19_00473" 473 "s59E5H2" 0 "Phosphoribosyl-AMP cyclohydrolase"
"Chl_A19_00473" 473 "s63E6H2" 0 "Phosphoribosyl-AMP cyclohydrolase"
"Chl_A19_00474" 474 "s59E13H2" 0 "hypothetical protein"
"Chl_A19_00474" 474 "s59E25H2" 0 "hypothetical protein"
"Chl_A19_00474" 474 "s59E5H2" 0 "hypothetical protein"
"Chl_A19_00474" 474 "s63E6H2" 0 "hypothetical protein"
"Chl_A19_00475" 475 "s59E13H2" 0 "50S ribosomal protein L27"
"Chl_A19_00475" 475 "s59E25H2" 0 "50S ribosomal protein L27"
"Chl_A19_00475" 475 "s59E5H2" 0 "50S ribosomal protein L27"
"Chl_A19_00475" 475 "s63E6H2" 0 "50S ribosomal protein L27"
"Chl_A19_00476" 476 "s59E13H2" 0 "50S ribosomal protein L21"
"Chl_A19_00476" 476 "s59E25H2" 0 "50S ribosomal protein L21"
"Chl_A19_00476" 476 "s59E5H2" 0 "50S ribosomal protein L21"
"Chl_A19_00476" 476 "s63E6H2" 0 "50S ribosomal protein L21"
"Chl_A19_00477" 477 "s59E13H2" 0 "30S ribosomal protein S15"
"Chl_A19_00477" 477 "s59E25H2" 0 "30S ribosomal protein S15"
"Chl_A19_00477" 477 "s59E5H2" 0 "30S ribosomal protein S15"
"Chl_A19_00477" 477 "s63E6H2" 0 "30S ribosomal protein S15"
"Chl_A19_00478" 478 "s59E13H2" 0 "Polyribonucleotide nucleotidyltransferase"
"Chl_A19_00478" 478 "s59E25H2" 0 "Polyribonucleotide nucleotidyltransferase"
"Chl_A19_00478" 478 "s59E5H2" 0 "Polyribonucleotide nucleotidyltransferase"
"Chl_A19_00478" 478 "s63E6H2" 0 "Polyribonucleotide nucleotidyltransferase"
"Chl_A19_00479" 479 "s59E13H2" 0 "Polyribonucleotide nucleotidyltransferase"
"Chl_A19_00479" 479 "s59E25H2" 0 "Polyribonucleotide nucleotidyltransferase"
"Chl_A19_00479" 479 "s59E5H2" 0 "Polyribonucleotide nucleotidyltransferase"
"Chl_A19_00479" 479 "s63E6H2" 0 "Polyribonucleotide nucleotidyltransferase"
"Chl_A19_00480" 480 "s59E13H2" 0 "4-hydroxy-tetrahydrodipicolinate reductase"
"Chl_A19_00480" 480 "s59E25H2" 0 "4-hydroxy-tetrahydrodipicolinate reductase"
"Chl_A19_00480" 480 "s59E5H2" 0 "4-hydroxy-tetrahydrodipicolinate reductase"
"Chl_A19_00480" 480 "s63E6H2" 0 "4-hydroxy-tetrahydrodipicolinate reductase"
"Chl_A19_00481" 481 "s59E13H2" 0 "Aspartate-semialdehyde dehydrogenase"
"Chl_A19_00481" 481 "s59E25H2" 0 "Aspartate-semialdehyde dehydrogenase"
"Chl_A19_00481" 481 "s59E5H2" 0.0037606163310004 "Aspartate-semialdehyde dehydrogenase"
"Chl_A19_00481" 481 "s63E6H2" 0 "Aspartate-semialdehyde dehydrogenase"
"Chl_A19_00482" 482 "s59E13H2" 0 "4-hydroxy-tetrahydrodipicolinate synthase"
"Chl_A19_00482" 482 "s59E25H2" 0 "4-hydroxy-tetrahydrodipicolinate synthase"

"Chl_A19_00482" 482 "s59E5H2" 0 "4-hydroxy-tetrahydrodipicolinate synthase"
"Chl_A19_00482" 482 "s63E6H2" 0 "4-hydroxy-tetrahydrodipicolinate synthase"
"Chl_A19_00483" 483 "s59E13H2" 0 "Adenylosuccinate synthetase"
"Chl_A19_00483" 483 "s59E25H2" 0 "Adenylosuccinate synthetase"
"Chl_A19_00483" 483 "s59E5H2" 0 "Adenylosuccinate synthetase"
"Chl_A19_00483" 483 "s63E6H2" 0 "Adenylosuccinate synthetase"
"Chl_A19_00484" 484 "s59E13H2" 0 "Archaetidylinositol phosphate synthase"
"Chl_A19_00484" 484 "s59E25H2" 0 "Archaetidylinositol phosphate synthase"
"Chl_A19_00484" 484 "s59E5H2" 0 "Archaetidylinositol phosphate synthase"
"Chl_A19_00484" 484 "s63E6H2" 0 "Archaetidylinositol phosphate synthase"
"Chl_A19_00485" 485 "s59E13H2" 0 "GDP-mannose-dependent alpha-(1-2)-phosphatidylinositol mannosyltransferase"
"Chl_A19_00485" 485 "s59E25H2" 0 "GDP-mannose-dependent alpha-(1-2)-phosphatidylinositol mannosyltransferase"
"Chl_A19_00485" 485 "s59E5H2" 0 "GDP-mannose-dependent alpha-(1-2)-phosphatidylinositol mannosyltransferase"
"Chl_A19_00485" 485 "s63E6H2" 0 "GDP-mannose-dependent alpha-(1-2)-phosphatidylinositol mannosyltransferase"
"Chl_A19_00486" 486 "s59E13H2" 0 "Inositol-3-phosphate synthase"
"Chl_A19_00486" 486 "s59E25H2" 0 "Inositol-3-phosphate synthase"
"Chl_A19_00486" 486 "s59E5H2" 0 "Inositol-3-phosphate synthase"
"Chl_A19_00486" 486 "s63E6H2" 0 "Inositol-3-phosphate synthase"
"Chl_A19_00487" 487 "s59E13H2" 0 "DNA polymerase/3'-5' exonuclease PolX"
"Chl_A19_00487" 487 "s59E25H2" 0 "DNA polymerase/3'-5' exonuclease PolX"
"Chl_A19_00487" 487 "s59E5H2" 0 "DNA polymerase/3'-5' exonuclease PolX"
"Chl_A19_00487" 487 "s63E6H2" 0 "DNA polymerase/3'-5' exonuclease PolX"
"Chl_A19_00488" 488 "s59E13H2" 0 "tRNA pseudouridine synthase B"
"Chl_A19_00488" 488 "s59E25H2" 0 "tRNA pseudouridine synthase B"
"Chl_A19_00488" 488 "s59E5H2" 0 "tRNA pseudouridine synthase B"
"Chl_A19_00488" 488 "s63E6H2" 0 "tRNA pseudouridine synthase B"
"Chl_A19_00489" 489 "s59E13H2" 0 "Ribosome-binding factor A"
"Chl_A19_00489" 489 "s59E25H2" 0 "Ribosome-binding factor A"
"Chl_A19_00489" 489 "s59E5H2" 0 "Ribosome-binding factor A"
"Chl_A19_00489" 489 "s63E6H2" 0 "Ribosome-binding factor A"
"Chl_A19_00490" 490 "s59E13H2" 0 "Translation initiation factor IF-2"
"Chl_A19_00490" 490 "s59E25H2" 0 "Translation initiation factor IF-2"
"Chl_A19_00490" 490 "s59E5H2" 0 "Translation initiation factor IF-2"
"Chl_A19_00490" 490 "s63E6H2" 0 "Translation initiation factor IF-2"
"Chl_A19_00491" 491 "s59E13H2" 0 "hypothetical protein"
"Chl_A19_00491" 491 "s59E25H2" 0 "hypothetical protein"
"Chl_A19_00491" 491 "s59E5H2" 0 "hypothetical protein"
"Chl_A19_00491" 491 "s63E6H2" 0 "hypothetical protein"
"Chl_A19_00492" 492 "s59E13H2" 0 "Integrase core domain protein"
"Chl_A19_00492" 492 "s59E25H2" 0 "Integrase core domain protein"
"Chl_A19_00492" 492 "s59E5H2" 0.0188321400670418 "Integrase core domain protein"
"Chl_A19_00492" 492 "s63E6H2" 0 "Integrase core domain protein"
"Chl_A19_00493" 493 "s59E13H2" 0 "transposase"
"Chl_A19_00493" 493 "s59E25H2" 0 "transposase"
"Chl_A19_00493" 493 "s59E5H2" 0.0296676859286977 "transposase"
"Chl_A19_00493" 493 "s63E6H2" 0 "transposase"
"Chl_A19_00494" 494 "s59E13H2" 0 "Plasmid pRiA4b ORF-3-like protein"

"Chl_A19_00494" 494 "s59E25H2" 0 "Plasmid pRiA4b ORF-3-like protein"
"Chl_A19_00494" 494 "s59E5H2" 0 "Plasmid pRiA4b ORF-3-like protein"
"Chl_A19_00494" 494 "s63E6H2" 0 "Plasmid pRiA4b ORF-3-like protein"
"Chl_A19_00495" 495 "s59E13H2" 0 "lipid-transfer protein"
"Chl_A19_00495" 495 "s59E25H2" 0 "lipid-transfer protein"
"Chl_A19_00495" 495 "s59E5H2" 0 "lipid-transfer protein"
"Chl_A19_00495" 495 "s63E6H2" 0 "lipid-transfer protein"
"Chl_A19_00497" 497 "s59E13H2" 0 "ATPase family associated with various cellular activities (AAA)"
"Chl_A19_00497" 497 "s59E25H2" 0 "ATPase family associated with various cellular activities (AAA)"
"Chl_A19_00497" 497 "s59E5H2" 0 "ATPase family associated with various cellular activities (AAA)"
"Chl_A19_00497" 497 "s63E6H2" 0 "ATPase family associated with various cellular activities (AAA)"
"Chl_A19_00498" 498 "s59E13H2" 0 "VWA domain containing CoxE-like protein"
"Chl_A19_00498" 498 "s59E25H2" 0 "VWA domain containing CoxE-like protein"
"Chl_A19_00498" 498 "s59E5H2" 0 "VWA domain containing CoxE-like protein"
"Chl_A19_00498" 498 "s63E6H2" 0 "VWA domain containing CoxE-like protein"
"Chl_A19_00499" 499 "s59E13H2" 0 "cAMP receptor protein"
"Chl_A19_00499" 499 "s59E25H2" 0 "cAMP receptor protein"
"Chl_A19_00499" 499 "s59E5H2" 0 "cAMP receptor protein"
"Chl_A19_00499" 499 "s63E6H2" 0 "cAMP receptor protein"
"Chl_A19_00500" 500 "s59E13H2" 0 "2-oxoglutarate-acceptor oxidoreductase subunit OorD"
"Chl_A19_00500" 500 "s59E25H2" 0 "2-oxoglutarate-acceptor oxidoreductase subunit OorD"
"Chl_A19_00500" 500 "s59E5H2" 0 "2-oxoglutarate-acceptor oxidoreductase subunit OorD"
"Chl_A19_00500" 500 "s63E6H2" 0 "2-oxoglutarate-acceptor oxidoreductase subunit OorD"
"Chl_A19_00502" 502 "s59E13H2" 0 "Spore protein SP21"
"Chl_A19_00502" 502 "s59E25H2" 0 "Spore protein SP21"
"Chl_A19_00502" 502 "s59E5H2" 0 "Spore protein SP21"
"Chl_A19_00502" 502 "s63E6H2" 0 "Spore protein SP21"
"Chl_A19_00503" 503 "s59E13H2" 0 "2-methylcitrate dehydratase"
"Chl_A19_00503" 503 "s59E25H2" 0 "2-methylcitrate dehydratase"
"Chl_A19_00503" 503 "s59E5H2" 0 "2-methylcitrate dehydratase"
"Chl_A19_00503" 503 "s63E6H2" 0 "2-methylcitrate dehydratase"
"Chl_A19_00504" 504 "s59E13H2" 0 "DNA alkylation repair enzyme"
"Chl_A19_00504" 504 "s59E25H2" 0 "DNA alkylation repair enzyme"
"Chl_A19_00504" 504 "s59E5H2" 0 "DNA alkylation repair enzyme"
"Chl_A19_00504" 504 "s63E6H2" 0 "DNA alkylation repair enzyme"
"Chl_A19_00505" 505 "s59E13H2" 0 "Pathogenicity locus"
"Chl_A19_00505" 505 "s59E25H2" 0 "Pathogenicity locus"
"Chl_A19_00505" 505 "s59E5H2" 0 "Pathogenicity locus"
"Chl_A19_00505" 505 "s63E6H2" 0 "Pathogenicity locus"
"Chl_A19_00506" 506 "s59E13H2" 0 "Phospholipase YtpA"
"Chl_A19_00506" 506 "s59E25H2" 0 "Phospholipase YtpA"
"Chl_A19_00506" 506 "s59E5H2" 0 "Phospholipase YtpA"
"Chl_A19_00506" 506 "s63E6H2" 0 "Phospholipase YtpA"
"Chl_A19_00507" 507 "s59E13H2" 0 "hypothetical protein"
"Chl_A19_00507" 507 "s59E25H2" 0 "hypothetical protein"
"Chl_A19_00507" 507 "s59E5H2" 0 "hypothetical protein"
"Chl_A19_00507" 507 "s63E6H2" 0.00107144566654222 "hypothetical protein"

"Chl_A19_00508" 508 "s59E13H2" 0 "hypothetical protein"
"Chl_A19_00508" 508 "s59E25H2" 0 "hypothetical protein"
"Chl_A19_00508" 508 "s59E5H2" 0.00325337490683099 "hypothetical protein"
"Chl_A19_00508" 508 "s63E6H2" 0 "hypothetical protein"
"Chl_A19_00509" 509 "s59E13H2" 0 "hypothetical protein"
"Chl_A19_00509" 509 "s59E25H2" 0 "hypothetical protein"
"Chl_A19_00509" 509 "s59E5H2" 0 "hypothetical protein"
"Chl_A19_00509" 509 "s63E6H2" 0.00277007382835797 "hypothetical protein"
"Chl_A19_00510" 510 "s59E13H2" 0 "hypothetical protein"
"Chl_A19_00510" 510 "s59E25H2" 0 "hypothetical protein"
"Chl_A19_00510" 510 "s59E5H2" 0 "hypothetical protein"
"Chl_A19_00510" 510 "s63E6H2" 0 "hypothetical protein"
"Chl_A19_00513" 513 "s59E13H2" 0 "putative tyrosine recombinase XerC-like protein"
"Chl_A19_00513" 513 "s59E25H2" 0 "putative tyrosine recombinase XerC-like protein"
"Chl_A19_00513" 513 "s59E5H2" 0 "putative tyrosine recombinase XerC-like protein"
"Chl_A19_00513" 513 "s63E6H2" 0 "putative tyrosine recombinase XerC-like protein"
"Chl_A19_00514" 514 "s59E13H2" 0 "Proteasome subunit alpha"
"Chl_A19_00514" 514 "s59E25H2" 0 "Proteasome subunit alpha"
"Chl_A19_00514" 514 "s59E5H2" 0 "Proteasome subunit alpha"
"Chl_A19_00514" 514 "s63E6H2" 0 "Proteasome subunit alpha"
"Chl_A19_00515" 515 "s59E13H2" 0 "hypothetical protein"
"Chl_A19_00515" 515 "s59E25H2" 0 "hypothetical protein"
"Chl_A19_00515" 515 "s59E5H2" 0 "hypothetical protein"
"Chl_A19_00515" 515 "s63E6H2" 0 "hypothetical protein"
"Chl_A19_00516" 516 "s59E13H2" 0 "Helix-turn-helix"
"Chl_A19_00516" 516 "s59E25H2" 0 "Helix-turn-helix"
"Chl_A19_00516" 516 "s59E5H2" 0 "Helix-turn-helix"
"Chl_A19_00516" 516 "s63E6H2" 0 "Helix-turn-helix"
"Chl_A19_00518" 518 "s59E13H2" 0 "putative HTH-type transcriptional regulator YusO"
"Chl_A19_00518" 518 "s59E25H2" 0 "putative HTH-type transcriptional regulator YusO"
"Chl_A19_00518" 518 "s59E5H2" 0 "putative HTH-type transcriptional regulator YusO"
"Chl_A19_00518" 518 "s63E6H2" 0 "putative HTH-type transcriptional regulator YusO"
"Chl_A19_00519" 519 "s59E13H2" 0 "Tyrosine recombinase XerC"
"Chl_A19_00519" 519 "s59E25H2" 0 "Tyrosine recombinase XerC"
"Chl_A19_00519" 519 "s59E5H2" 0 "Tyrosine recombinase XerC"
"Chl_A19_00519" 519 "s63E6H2" 0 "Tyrosine recombinase XerC"
"Chl_A19_00521" 521 "s59E13H2" 0 "Transcriptional regulatory protein AfsQ1"
"Chl_A19_00521" 521 "s59E25H2" 0 "Transcriptional regulatory protein AfsQ1"
"Chl_A19_00521" 521 "s59E5H2" 0 "Transcriptional regulatory protein AfsQ1"
"Chl_A19_00521" 521 "s63E6H2" 0.00622507586274274 "Transcriptional regulatory protein AfsQ1"
"Chl_A19_00523" 523 "s59E13H2" 0 "hypothetical protein"
"Chl_A19_00523" 523 "s59E25H2" 0 "hypothetical protein"
"Chl_A19_00523" 523 "s59E5H2" 0 "hypothetical protein"
"Chl_A19_00523" 523 "s63E6H2" 0 "hypothetical protein"
"Chl_A19_00524" 524 "s59E13H2" 0 "hypothetical protein"
"Chl_A19_00524" 524 "s59E25H2" 0 "hypothetical protein"
"Chl_A19_00524" 524 "s59E5H2" 0 "hypothetical protein"

"Chl_A19_00524" 524 "s63E6H2" 0 "hypothetical protein"
"Chl_A19_00525" 525 "s59E13H2" 0 "hypothetical protein"
"Chl_A19_00525" 525 "s59E25H2" 0 "hypothetical protein"
"Chl_A19_00525" 525 "s59E5H2" 0 "hypothetical protein"
"Chl_A19_00525" 525 "s63E6H2" 0 "hypothetical protein"
"Chl_A19_00526" 526 "s59E13H2" 0.465006670658056 "Transposase DDE domain protein"
"Chl_A19_00526" 526 "s59E25H2" 0 "Transposase DDE domain protein"
"Chl_A19_00526" 526 "s59E5H2" 0 "Transposase DDE domain protein"
"Chl_A19_00526" 526 "s63E6H2" 0.0833202120075375 "Transposase DDE domain protein"
"Chl_A19_00527" 527 "s59E13H2" 0 "hypothetical protein"
"Chl_A19_00527" 527 "s59E25H2" 0 "hypothetical protein"
"Chl_A19_00527" 527 "s59E5H2" 0 "hypothetical protein"
"Chl_A19_00527" 527 "s63E6H2" 0 "hypothetical protein"
"Chl_A19_00528" 528 "s59E13H2" 0 "Restriction endonuclease"
"Chl_A19_00528" 528 "s59E25H2" 0 "Restriction endonuclease"
"Chl_A19_00528" 528 "s59E5H2" 0 "Restriction endonuclease"
"Chl_A19_00528" 528 "s63E6H2" 0 "Restriction endonuclease"
"Chl_A19_00530" 530 "s59E13H2" 0 "hypothetical protein"
"Chl_A19_00530" 530 "s59E25H2" 0 "hypothetical protein"
"Chl_A19_00530" 530 "s59E5H2" 0 "hypothetical protein"
"Chl_A19_00530" 530 "s63E6H2" 0 "hypothetical protein"
"Chl_A19_00532" 532 "s59E13H2" 0 "hypothetical protein"
"Chl_A19_00532" 532 "s59E25H2" 0 "hypothetical protein"
"Chl_A19_00532" 532 "s59E5H2" 0 "hypothetical protein"
"Chl_A19_00532" 532 "s63E6H2" 0 "hypothetical protein"
"Chl_A19_00534" 534 "s59E13H2" 0 "hypothetical protein"
"Chl_A19_00534" 534 "s59E25H2" 0 "hypothetical protein"
"Chl_A19_00534" 534 "s59E5H2" 0 "hypothetical protein"
"Chl_A19_00534" 534 "s63E6H2" 0 "hypothetical protein"
"Chl_A19_00535" 535 "s59E13H2" 0 "hypothetical protein"
"Chl_A19_00535" 535 "s59E25H2" 0 "hypothetical protein"
"Chl_A19_00535" 535 "s59E5H2" 0 "hypothetical protein"
"Chl_A19_00535" 535 "s63E6H2" 0 "hypothetical protein"
"Chl_A19_00537" 537 "s59E13H2" 0 "hypothetical protein"
"Chl_A19_00537" 537 "s59E25H2" 0 "hypothetical protein"
"Chl_A19_00537" 537 "s59E5H2" 0 "hypothetical protein"
"Chl_A19_00537" 537 "s63E6H2" 0 "hypothetical protein"
"Chl_A19_00538" 538 "s59E13H2" 0 "Methyltransferase domain protein"
"Chl_A19_00538" 538 "s59E25H2" 0 "Methyltransferase domain protein"
"Chl_A19_00538" 538 "s59E5H2" 0 "Methyltransferase domain protein"
"Chl_A19_00538" 538 "s63E6H2" 0 "Methyltransferase domain protein"
"Chl_A19_00539" 539 "s59E13H2" 0 "hypothetical protein"
"Chl_A19_00539" 539 "s59E25H2" 0 "hypothetical protein"
"Chl_A19_00539" 539 "s59E5H2" 0 "hypothetical protein"
"Chl_A19_00539" 539 "s63E6H2" 0 "hypothetical protein"
"Chl_A19_00541" 541 "s59E13H2" 0 "Beta-galactosidase"
"Chl_A19_00541" 541 "s59E25H2" 0 "Beta-galactosidase"

"Chl_A19_00541" 541 "s59E5H2" 0.0106202673343766 "Beta-galactosidase"
"Chl_A19_00541" 541 "s63E6H2" 0 "Beta-galactosidase"
"Chl_A19_00545" 545 "s59E13H2" 0 "hypothetical protein"
"Chl_A19_00545" 545 "s59E25H2" 0 "hypothetical protein"
"Chl_A19_00545" 545 "s59E5H2" 0 "hypothetical protein"
"Chl_A19_00545" 545 "s63E6H2" 0 "hypothetical protein"
"Chl_A19_00546" 546 "s59E13H2" 0 "NAD-dependent protein deacylase 2"
"Chl_A19_00546" 546 "s59E25H2" 0 "NAD-dependent protein deacylase 2"
"Chl_A19_00546" 546 "s59E5H2" 0 "NAD-dependent protein deacylase 2"
"Chl_A19_00546" 546 "s63E6H2" 0 "NAD-dependent protein deacylase 2"
"Chl_A19_00547" 547 "s59E13H2" 0 "Phytoene desaturase (lycopene-forming)"
"Chl_A19_00547" 547 "s59E25H2" 0 "Phytoene desaturase (lycopene-forming)"
"Chl_A19_00547" 547 "s59E5H2" 0 "Phytoene desaturase (lycopene-forming)"
"Chl_A19_00547" 547 "s63E6H2" 0 "Phytoene desaturase (lycopene-forming)"
"Chl_A19_00548" 548 "s59E13H2" 0 "Phytoene desaturase (lycopene-forming)"
"Chl_A19_00548" 548 "s59E25H2" 0 "Phytoene desaturase (lycopene-forming)"
"Chl_A19_00548" 548 "s59E5H2" 0 "Phytoene desaturase (lycopene-forming)"
"Chl_A19_00548" 548 "s63E6H2" 0 "Phytoene desaturase (lycopene-forming)"
"Chl_A19_00549" 549 "s59E13H2" 0 "hypothetical protein"
"Chl_A19_00549" 549 "s59E25H2" 0 "hypothetical protein"
"Chl_A19_00549" 549 "s59E5H2" 0 "hypothetical protein"
"Chl_A19_00549" 549 "s63E6H2" 0 "hypothetical protein"
"Chl_A19_00550" 550 "s59E13H2" 0 "Putative thymidylate synthase"
"Chl_A19_00550" 550 "s59E25H2" 0 "Putative thymidylate synthase"
"Chl_A19_00550" 550 "s59E5H2" 0 "Putative thymidylate synthase"
"Chl_A19_00550" 550 "s63E6H2" 0 "Putative thymidylate synthase"
"Chl_A19_00552" 552 "s59E13H2" 0 "hypothetical protein"
"Chl_A19_00552" 552 "s59E25H2" 0 "hypothetical protein"
"Chl_A19_00552" 552 "s59E5H2" 0 "hypothetical protein"
"Chl_A19_00552" 552 "s63E6H2" 0 "hypothetical protein"
"Chl_A19_00553" 553 "s59E13H2" 0 "hypothetical protein"
"Chl_A19_00553" 553 "s59E25H2" 0 "hypothetical protein"
"Chl_A19_00553" 553 "s59E5H2" 0 "hypothetical protein"
"Chl_A19_00553" 553 "s63E6H2" 0 "hypothetical protein"
"Chl_A19_00554" 554 "s59E13H2" 0 "tRNA-splicing ligase RtcB"
"Chl_A19_00554" 554 "s59E25H2" 0 "tRNA-splicing ligase RtcB"
"Chl_A19_00554" 554 "s59E5H2" 0 "tRNA-splicing ligase RtcB"
"Chl_A19_00554" 554 "s63E6H2" 0 "tRNA-splicing ligase RtcB"
"Chl_A19_00555" 555 "s59E13H2" 0 "Cysteine synthase"
"Chl_A19_00555" 555 "s59E25H2" 0 "Cysteine synthase"
"Chl_A19_00555" 555 "s59E5H2" 0 "Cysteine synthase"
"Chl_A19_00555" 555 "s63E6H2" 0 "Cysteine synthase"
"Chl_A19_00556" 556 "s59E13H2" 0 "2-isopropylmalate synthase"
"Chl_A19_00556" 556 "s59E25H2" 0 "2-isopropylmalate synthase"
"Chl_A19_00556" 556 "s59E5H2" 0 "2-isopropylmalate synthase"
"Chl_A19_00556" 556 "s63E6H2" 0 "2-isopropylmalate synthase"
"Chl_A19_00557" 557 "s59E13H2" 0 "3-isopropylmalate dehydrogenase"

"Chl_A19_00557" 557 "s59E25H2" 0 "3-isopropylmalate dehydrogenase"
"Chl_A19_00557" 557 "s59E5H2" 0 "3-isopropylmalate dehydrogenase"
"Chl_A19_00557" 557 "s63E6H2" 0 "3-isopropylmalate dehydrogenase"
"Chl_A19_00558" 558 "s59E13H2" 0 "2,3-dimethylmalate dehydratase small subunit"
"Chl_A19_00558" 558 "s59E25H2" 0 "2,3-dimethylmalate dehydratase small subunit"
"Chl_A19_00558" 558 "s59E5H2" 0 "2,3-dimethylmalate dehydratase small subunit"
"Chl_A19_00558" 558 "s63E6H2" 0 "2,3-dimethylmalate dehydratase small subunit"
"Chl_A19_00559" 559 "s59E13H2" 0 "hypothetical protein"
"Chl_A19_00559" 559 "s59E25H2" 0 "hypothetical protein"
"Chl_A19_00559" 559 "s59E5H2" 0 "hypothetical protein"
"Chl_A19_00559" 559 "s63E6H2" 0 "hypothetical protein"
"Chl_A19_00560" 560 "s59E13H2" 0 "hypothetical protein"
"Chl_A19_00560" 560 "s59E25H2" 0 "hypothetical protein"
"Chl_A19_00560" 560 "s59E5H2" 0 "hypothetical protein"
"Chl_A19_00560" 560 "s63E6H2" 0 "hypothetical protein"
"Chl_A19_00562" 562 "s59E13H2" 0 "Polyketide cyclase / dehydrase and lipid transport"
"Chl_A19_00562" 562 "s59E25H2" 0 "Polyketide cyclase / dehydrase and lipid transport"
"Chl_A19_00562" 562 "s59E5H2" 0 "Polyketide cyclase / dehydrase and lipid transport"
"Chl_A19_00562" 562 "s63E6H2" 0 "Polyketide cyclase / dehydrase and lipid transport"
"Chl_A19_00563" 563 "s59E13H2" 0 "2,3-dimethylmalate dehydratase large subunit"
"Chl_A19_00563" 563 "s59E25H2" 0 "2,3-dimethylmalate dehydratase large subunit"
"Chl_A19_00563" 563 "s59E5H2" 0 "2,3-dimethylmalate dehydratase large subunit"
"Chl_A19_00563" 563 "s63E6H2" 0 "2,3-dimethylmalate dehydratase large subunit"
"Chl_A19_00564" 564 "s59E13H2" 0 "2-isopropylmalate synthase"
"Chl_A19_00564" 564 "s59E25H2" 0 "2-isopropylmalate synthase"
"Chl_A19_00564" 564 "s59E5H2" 0 "2-isopropylmalate synthase"
"Chl_A19_00564" 564 "s63E6H2" 0 "2-isopropylmalate synthase"
"Chl_A19_00565" 565 "s59E13H2" 0 "Ketol-acid reductoisomerase"
"Chl_A19_00565" 565 "s59E25H2" 0 "Ketol-acid reductoisomerase"
"Chl_A19_00565" 565 "s59E5H2" 0 "Ketol-acid reductoisomerase"
"Chl_A19_00565" 565 "s63E6H2" 0 "Ketol-acid reductoisomerase"
"Chl_A19_00566" 566 "s59E13H2" 0 "Putative acetolactate synthase small subunit"
"Chl_A19_00566" 566 "s59E25H2" 0 "Putative acetolactate synthase small subunit"
"Chl_A19_00566" 566 "s59E5H2" 0 "Putative acetolactate synthase small subunit"
"Chl_A19_00566" 566 "s63E6H2" 0 "Putative acetolactate synthase small subunit"
"Chl_A19_00567" 567 "s59E13H2" 0 "Acetolactate synthase large subunit IlvB1"
"Chl_A19_00567" 567 "s59E25H2" 0 "Acetolactate synthase large subunit IlvB1"
"Chl_A19_00567" 567 "s59E5H2" 0 "Acetolactate synthase large subunit IlvB1"
"Chl_A19_00567" 567 "s63E6H2" 0 "Acetolactate synthase large subunit IlvB1"
"Chl_A19_00568" 568 "s59E13H2" 0 "Dihydroxy-acid dehydratase"
"Chl_A19_00568" 568 "s59E25H2" 0 "Dihydroxy-acid dehydratase"
"Chl_A19_00568" 568 "s59E5H2" 0 "Dihydroxy-acid dehydratase"
"Chl_A19_00568" 568 "s63E6H2" 0 "Dihydroxy-acid dehydratase"
"Chl_A19_00569" 569 "s59E13H2" 0 "Putative sugar phosphate isomerase YwlF"
"Chl_A19_00569" 569 "s59E25H2" 0 "Putative sugar phosphate isomerase YwlF"
"Chl_A19_00569" 569 "s59E5H2" 0 "Putative sugar phosphate isomerase YwlF"
"Chl_A19_00569" 569 "s63E6H2" 0 "Putative sugar phosphate isomerase YwlF"

"Chl_A19_00573" 573 "s59E13H2" 0 "hypothetical protein"
"Chl_A19_00573" 573 "s59E25H2" 0 "hypothetical protein"
"Chl_A19_00573" 573 "s59E5H2" 0.0475544155873059 "hypothetical protein"
"Chl_A19_00573" 573 "s63E6H2" 0 "hypothetical protein"
"Chl_A19_00574" 574 "s59E13H2" 0 "hypothetical protein"
"Chl_A19_00574" 574 "s59E25H2" 0 "hypothetical protein"
"Chl_A19_00574" 574 "s59E5H2" 0 "hypothetical protein"
"Chl_A19_00574" 574 "s63E6H2" 0 "hypothetical protein"
"Chl_A19_00576" 576 "s59E13H2" 0 "hypothetical protein"
"Chl_A19_00576" 576 "s59E25H2" 0 "hypothetical protein"
"Chl_A19_00576" 576 "s59E5H2" 0 "hypothetical protein"
"Chl_A19_00576" 576 "s63E6H2" 0 "hypothetical protein"
"Chl_A19_00578" 578 "s59E13H2" 0 "putative deoxyribonuclease YjjV"
"Chl_A19_00578" 578 "s59E25H2" 0 "putative deoxyribonuclease YjjV"
"Chl_A19_00578" 578 "s59E5H2" 0 "putative deoxyribonuclease YjjV"
"Chl_A19_00578" 578 "s63E6H2" 0 "putative deoxyribonuclease YjjV"
"Chl_A19_00579" 579 "s59E13H2" 0 "hypothetical protein"
"Chl_A19_00579" 579 "s59E25H2" 0 "hypothetical protein"
"Chl_A19_00579" 579 "s59E5H2" 0 "hypothetical protein"
"Chl_A19_00579" 579 "s63E6H2" 0 "hypothetical protein"
"Chl_A19_00580" 580 "s59E13H2" 0 "6-hydroxynicotinate reductase"
"Chl_A19_00580" 580 "s59E25H2" 0 "6-hydroxynicotinate reductase"
"Chl_A19_00580" 580 "s59E5H2" 0 "6-hydroxynicotinate reductase"
"Chl_A19_00580" 580 "s63E6H2" 0 "6-hydroxynicotinate reductase"
"Chl_A19_00582" 582 "s59E13H2" 0 "hypothetical protein"
"Chl_A19_00582" 582 "s59E25H2" 0 "hypothetical protein"
"Chl_A19_00582" 582 "s59E5H2" 0 "hypothetical protein"
"Chl_A19_00582" 582 "s63E6H2" 0 "hypothetical protein"
"Chl_A19_00584" 584 "s59E13H2" 0 "Tyrosine recombinase XerC"
"Chl_A19_00584" 584 "s59E25H2" 0 "Tyrosine recombinase XerC"
"Chl_A19_00584" 584 "s59E5H2" 0 "Tyrosine recombinase XerC"
"Chl_A19_00584" 584 "s63E6H2" 0 "Tyrosine recombinase XerC"
"Chl_A19_00585" 585 "s59E13H2" 0 "N-6 DNA Methylase"
"Chl_A19_00585" 585 "s59E25H2" 0 "N-6 DNA Methylase"
"Chl_A19_00585" 585 "s59E5H2" 0 "N-6 DNA Methylase"
"Chl_A19_00585" 585 "s63E6H2" 0 "N-6 DNA Methylase"
"Chl_A19_00586" 586 "s59E13H2" 0 "F420-non-reducing hydrogenase iron-sulfur subunit A"
"Chl_A19_00586" 586 "s59E25H2" 0 "F420-non-reducing hydrogenase iron-sulfur subunit A"
"Chl_A19_00586" 586 "s59E5H2" 0 "F420-non-reducing hydrogenase iron-sulfur subunit A"
"Chl_A19_00586" 586 "s63E6H2" 0 "F420-non-reducing hydrogenase iron-sulfur subunit A"
"Chl_A19_00587" 587 "s59E13H2" 0 "Fe/S biogenesis protein NfuA"
"Chl_A19_00587" 587 "s59E25H2" 0 "Fe/S biogenesis protein NfuA"
"Chl_A19_00587" 587 "s59E5H2" 0 "Fe/S biogenesis protein NfuA"
"Chl_A19_00587" 587 "s63E6H2" 0 "Fe/S biogenesis protein NfuA"
"Chl_A19_00588" 588 "s59E13H2" 0 "Hydrogenase 2 maturation protease"
"Chl_A19_00588" 588 "s59E25H2" 0 "Hydrogenase 2 maturation protease"
"Chl_A19_00588" 588 "s59E5H2" 0 "Hydrogenase 2 maturation protease"

"Chl_A19_00588" 588 "s63E6H2" 0 "Hydrogenase 2 maturation protease"
"Chl_A19_00589" 589 "s59E13H2" 0 "F420-non-reducing hydrogenase vhc iron-sulfur subunit D"
"Chl_A19_00589" 589 "s59E25H2" 0 "F420-non-reducing hydrogenase vhc iron-sulfur subunit D"
"Chl_A19_00589" 589 "s59E5H2" 0 "F420-non-reducing hydrogenase vhc iron-sulfur subunit D"
"Chl_A19_00589" 589 "s63E6H2" 0 "F420-non-reducing hydrogenase vhc iron-sulfur subunit D"
"Chl_A19_00590" 590 "s59E13H2" 0 "hypothetical protein"
"Chl_A19_00590" 590 "s59E25H2" 0 "hypothetical protein"
"Chl_A19_00590" 590 "s59E5H2" 0 "hypothetical protein"
"Chl_A19_00590" 590 "s63E6H2" 0 "hypothetical protein"
"Chl_A19_00591" 591 "s59E13H2" 0 "Carbamoyltransferase HypF"
"Chl_A19_00591" 591 "s59E25H2" 0 "Carbamoyltransferase HypF"
"Chl_A19_00591" 591 "s59E5H2" 0 "Carbamoyltransferase HypF"
"Chl_A19_00591" 591 "s63E6H2" 0 "Carbamoyltransferase HypF"
"Chl_A19_00592" 592 "s59E13H2" 0 "Hydrogenase isoenzymes formation protein HypC"
"Chl_A19_00592" 592 "s59E25H2" 0 "Hydrogenase isoenzymes formation protein HypC"
"Chl_A19_00592" 592 "s59E5H2" 0 "Hydrogenase isoenzymes formation protein HypC"
"Chl_A19_00592" 592 "s63E6H2" 0 "Hydrogenase isoenzymes formation protein HypC"
"Chl_A19_00593" 593 "s59E13H2" 0 "Hydrogenase expression/formation protein HypD"
"Chl_A19_00593" 593 "s59E25H2" 0 "Hydrogenase expression/formation protein HypD"
"Chl_A19_00593" 593 "s59E5H2" 0 "Hydrogenase expression/formation protein HypD"
"Chl_A19_00593" 593 "s63E6H2" 0 "Hydrogenase expression/formation protein HypD"
"Chl_A19_00594" 594 "s59E13H2" 0 "Hydrogenase expression/formation protein HypE"
"Chl_A19_00594" 594 "s59E25H2" 0 "Hydrogenase expression/formation protein HypE"
"Chl_A19_00594" 594 "s59E5H2" 0 "Hydrogenase expression/formation protein HypE"
"Chl_A19_00594" 594 "s63E6H2" 0 "Hydrogenase expression/formation protein HypE"
"Chl_A19_00595" 595 "s59E13H2" 0 "putative hydrogenase nickel incorporation protein HypA"
"Chl_A19_00595" 595 "s59E25H2" 0 "putative hydrogenase nickel incorporation protein HypA"
"Chl_A19_00595" 595 "s59E5H2" 0 "putative hydrogenase nickel incorporation protein HypA"
"Chl_A19_00595" 595 "s63E6H2" 0 "putative hydrogenase nickel incorporation protein HypA"
"Chl_A19_00596" 596 "s59E13H2" 0 "Hydrogenase isoenzymes nickel incorporation protein HypB"
"Chl_A19_00596" 596 "s59E25H2" 0 "Hydrogenase isoenzymes nickel incorporation protein HypB"
"Chl_A19_00596" 596 "s59E5H2" 0 "Hydrogenase isoenzymes nickel incorporation protein HypB"
"Chl_A19_00596" 596 "s63E6H2" 0 "Hydrogenase isoenzymes nickel incorporation protein HypB"
"Chl_A19_00597" 597 "s59E13H2" 0 "PAC2 family protein"
"Chl_A19_00597" 597 "s59E25H2" 0 "PAC2 family protein"
"Chl_A19_00597" 597 "s59E5H2" 0 "PAC2 family protein"
"Chl_A19_00597" 597 "s63E6H2" 0 "PAC2 family protein"
"Chl_A19_00598" 598 "s59E13H2" 0 "Trm112p-like protein"
"Chl_A19_00598" 598 "s59E25H2" 0 "Trm112p-like protein"
"Chl_A19_00598" 598 "s59E5H2" 0 "Trm112p-like protein"
"Chl_A19_00598" 598 "s63E6H2" 0 "Trm112p-like protein"
"Chl_A19_00599" 599 "s59E13H2" 0 "6-carboxy-5,6,7,8-tetrahydropterin synthase"
"Chl_A19_00599" 599 "s59E25H2" 0 "6-carboxy-5,6,7,8-tetrahydropterin synthase"
"Chl_A19_00599" 599 "s59E5H2" 0 "6-carboxy-5,6,7,8-tetrahydropterin synthase"
"Chl_A19_00599" 599 "s63E6H2" 0 "6-carboxy-5,6,7,8-tetrahydropterin synthase"
"Chl_A19_00600" 600 "s59E13H2" 0 "Bifunctional folate synthesis protein"
"Chl_A19_00600" 600 "s59E25H2" 0 "Bifunctional folate synthesis protein"

"Chl_A19_00600" 600 "s59E5H2" 0 "Bifunctional folate synthesis protein"
"Chl_A19_00600" 600 "s63E6H2" 0 "Bifunctional folate synthesis protein"
"Chl_A19_00601" 601 "s59E13H2" 0 "Malate dehydrogenase"
"Chl_A19_00601" 601 "s59E25H2" 0 "Malate dehydrogenase"
"Chl_A19_00601" 601 "s59E5H2" 0 "Malate dehydrogenase"
"Chl_A19_00601" 601 "s63E6H2" 0 "Malate dehydrogenase"
"Chl_A19_00602" 602 "s59E13H2" 0 "L(+)-tartrate dehydratase subunit alpha"
"Chl_A19_00602" 602 "s59E25H2" 0 "L(+)-tartrate dehydratase subunit alpha"
"Chl_A19_00602" 602 "s59E5H2" 0 "L(+)-tartrate dehydratase subunit alpha"
"Chl_A19_00602" 602 "s63E6H2" 0 "L(+)-tartrate dehydratase subunit alpha"
"Chl_A19_00603" 603 "s59E13H2" 0 "Fumarate hydratase class I, aerobic"
"Chl_A19_00603" 603 "s59E25H2" 0 "Fumarate hydratase class I, aerobic"
"Chl_A19_00603" 603 "s59E5H2" 0 "Fumarate hydratase class I, aerobic"
"Chl_A19_00603" 603 "s63E6H2" 0 "Fumarate hydratase class I, aerobic"
"Chl_A19_00604" 604 "s59E13H2" 0 "HIT-like protein"
"Chl_A19_00604" 604 "s59E25H2" 0 "HIT-like protein"
"Chl_A19_00604" 604 "s59E5H2" 0 "HIT-like protein"
"Chl_A19_00604" 604 "s63E6H2" 0 "HIT-like protein"
"Chl_A19_00605" 605 "s59E13H2" 0 "hypothetical protein"
"Chl_A19_00605" 605 "s59E25H2" 0 "hypothetical protein"
"Chl_A19_00605" 605 "s59E5H2" 0 "hypothetical protein"
"Chl_A19_00605" 605 "s63E6H2" 0 "hypothetical protein"
"Chl_A19_00606" 606 "s59E13H2" 0 "ADP-ribose pyrophosphatase"
"Chl_A19_00606" 606 "s59E25H2" 0 "ADP-ribose pyrophosphatase"
"Chl_A19_00606" 606 "s59E5H2" 0 "ADP-ribose pyrophosphatase"
"Chl_A19_00606" 606 "s63E6H2" 0 "ADP-ribose pyrophosphatase"
"Chl_A19_00607" 607 "s59E13H2" 0 "Amino-acid acetyltransferase"
"Chl_A19_00607" 607 "s59E25H2" 0 "Amino-acid acetyltransferase"
"Chl_A19_00607" 607 "s59E5H2" 0 "Amino-acid acetyltransferase"
"Chl_A19_00607" 607 "s63E6H2" 0 "Amino-acid acetyltransferase"
"Chl_A19_00608" 608 "s59E13H2" 0 "putative inorganic polyphosphate/ATP-NAD kinase"
"Chl_A19_00608" 608 "s59E25H2" 0 "putative inorganic polyphosphate/ATP-NAD kinase"
"Chl_A19_00608" 608 "s59E5H2" 0 "putative inorganic polyphosphate/ATP-NAD kinase"
"Chl_A19_00608" 608 "s63E6H2" 0.000796097440124929 "putative inorganic polyphosphate/ATP-NAD kinase"
"Chl_A19_00609" 609 "s59E13H2" 0 "3-oxoacyl-[acyl-carrier-protein] synthase 3"
"Chl_A19_00609" 609 "s59E25H2" 0 "3-oxoacyl-[acyl-carrier-protein] synthase 3"
"Chl_A19_00609" 609 "s59E5H2" 0 "3-oxoacyl-[acyl-carrier-protein] synthase 3"
"Chl_A19_00609" 609 "s63E6H2" 0 "3-oxoacyl-[acyl-carrier-protein] synthase 3"
"Chl_A19_00610" 610 "s59E13H2" 0 "acetyl-CoA acetyltransferase"
"Chl_A19_00610" 610 "s59E25H2" 0 "acetyl-CoA acetyltransferase"
"Chl_A19_00610" 610 "s59E5H2" 0 "acetyl-CoA acetyltransferase"
"Chl_A19_00610" 610 "s63E6H2" 0 "acetyl-CoA acetyltransferase"
"Chl_A19_00611" 611 "s59E13H2" 0 "50S ribosomal protein L10"
"Chl_A19_00611" 611 "s59E25H2" 0 "50S ribosomal protein L10"
"Chl_A19_00611" 611 "s59E5H2" 0 "50S ribosomal protein L10"
"Chl_A19_00611" 611 "s63E6H2" 0 "50S ribosomal protein L10"
"Chl_A19_00612" 612 "s59E13H2" 0 "50S ribosomal protein L7/L12"

"Chl_A19_00612" 612 "s59E25H2" 0 "50S ribosomal protein L7/L12"
"Chl_A19_00612" 612 "s59E5H2" 0 "50S ribosomal protein L7/L12"
"Chl_A19_00612" 612 "s63E6H2" 0 "50S ribosomal protein L7/L12"
"Chl_A19_00613" 613 "s59E13H2" 0 "hypothetical protein"
"Chl_A19_00613" 613 "s59E25H2" 0 "hypothetical protein"
"Chl_A19_00613" 613 "s59E5H2" 0 "hypothetical protein"
"Chl_A19_00613" 613 "s63E6H2" 0 "hypothetical protein"
"Chl_A19_00614" 614 "s59E13H2" 0 "hypothetical protein"
"Chl_A19_00614" 614 "s59E25H2" 0 "hypothetical protein"
"Chl_A19_00614" 614 "s59E5H2" 0 "hypothetical protein"
"Chl_A19_00614" 614 "s63E6H2" 0 "hypothetical protein"
"Chl_A19_00615" 615 "s59E13H2" 0 "hypothetical protein"
"Chl_A19_00615" 615 "s59E25H2" 0 "hypothetical protein"
"Chl_A19_00615" 615 "s59E5H2" 0 "hypothetical protein"
"Chl_A19_00615" 615 "s63E6H2" 0 "hypothetical protein"
"Chl_A19_00616" 616 "s59E13H2" 0 "hypothetical protein"
"Chl_A19_00616" 616 "s59E25H2" 0 "hypothetical protein"
"Chl_A19_00616" 616 "s59E5H2" 0 "hypothetical protein"
"Chl_A19_00616" 616 "s63E6H2" 0 "hypothetical protein"
"Chl_A19_00617" 617 "s59E13H2" 0 "Sodium/glucose cotransporter"
"Chl_A19_00617" 617 "s59E25H2" 0 "Sodium/glucose cotransporter"
"Chl_A19_00617" 617 "s59E5H2" 0 "Sodium/glucose cotransporter"
"Chl_A19_00617" 617 "s63E6H2" 0 "Sodium/glucose cotransporter"
"Chl_A19_00618" 618 "s59E13H2" 0 "DNA mismatch repair protein MutL"
"Chl_A19_00618" 618 "s59E25H2" 0 "DNA mismatch repair protein MutL"
"Chl_A19_00618" 618 "s59E5H2" 0 "DNA mismatch repair protein MutL"
"Chl_A19_00618" 618 "s63E6H2" 0 "DNA mismatch repair protein MutL"
"Chl_A19_00619" 619 "s59E13H2" 0 "30S ribosomal protein S6"
"Chl_A19_00619" 619 "s59E25H2" 0 "30S ribosomal protein S6"
"Chl_A19_00619" 619 "s59E5H2" 0 "30S ribosomal protein S6"
"Chl_A19_00619" 619 "s63E6H2" 0 "30S ribosomal protein S6"
"Chl_A19_00620" 620 "s59E13H2" 0 "Single-stranded DNA-binding protein"
"Chl_A19_00620" 620 "s59E25H2" 0 "Single-stranded DNA-binding protein"
"Chl_A19_00620" 620 "s59E5H2" 0 "Single-stranded DNA-binding protein"
"Chl_A19_00620" 620 "s63E6H2" 0 "Single-stranded DNA-binding protein"
"Chl_A19_00621" 621 "s59E13H2" 0 "30S ribosomal protein S18"
"Chl_A19_00621" 621 "s59E25H2" 0 "30S ribosomal protein S18"
"Chl_A19_00621" 621 "s59E5H2" 0 "30S ribosomal protein S18"
"Chl_A19_00621" 621 "s63E6H2" 0 "30S ribosomal protein S18"
"Chl_A19_00622" 622 "s59E13H2" 0 "Ribonuclease P protein component"
"Chl_A19_00622" 622 "s59E25H2" 0 "Ribonuclease P protein component"
"Chl_A19_00622" 622 "s59E5H2" 0 "Ribonuclease P protein component"
"Chl_A19_00622" 622 "s63E6H2" 0 "Ribonuclease P protein component"
"Chl_A19_00623" 623 "s59E13H2" 0 "Serine/threonine-protein kinase AfsK"
"Chl_A19_00623" 623 "s59E25H2" 0 "Serine/threonine-protein kinase AfsK"
"Chl_A19_00623" 623 "s59E5H2" 0 "Serine/threonine-protein kinase AfsK"
"Chl_A19_00623" 623 "s63E6H2" 0 "Serine/threonine-protein kinase AfsK"

"Chl_A19_00624" 624 "s59E13H2" 0 "Membrane protein insertase YidC"
"Chl_A19_00624" 624 "s59E25H2" 0 "Membrane protein insertase YidC"
"Chl_A19_00624" 624 "s59E5H2" 0 "Membrane protein insertase YidC"
"Chl_A19_00624" 624 "s63E6H2" 0 "Membrane protein insertase YidC"
"Chl_A19_00625" 625 "s59E13H2" 0 "R3H domain protein"
"Chl_A19_00625" 625 "s59E25H2" 0 "R3H domain protein"
"Chl_A19_00625" 625 "s59E5H2" 0 "R3H domain protein"
"Chl_A19_00625" 625 "s63E6H2" 0 "R3H domain protein"
"Chl_A19_00626" 626 "s59E13H2" 0 "Mannosylfructose-phosphate phosphatase"
"Chl_A19_00626" 626 "s59E25H2" 0 "Mannosylfructose-phosphate phosphatase"
"Chl_A19_00626" 626 "s59E5H2" 0 "Mannosylfructose-phosphate phosphatase"
"Chl_A19_00626" 626 "s63E6H2" 0 "Mannosylfructose-phosphate phosphatase"
"Chl_A19_00627" 627 "s59E13H2" 0 "hypothetical protein"
"Chl_A19_00627" 627 "s59E25H2" 0 "hypothetical protein"
"Chl_A19_00627" 627 "s59E5H2" 0 "hypothetical protein"
"Chl_A19_00627" 627 "s63E6H2" 0 "hypothetical protein"
"Chl_A19_00628" 628 "s59E13H2" 0 "50S ribosomal protein L32"
"Chl_A19_00628" 628 "s59E25H2" 0 "50S ribosomal protein L32"
"Chl_A19_00628" 628 "s59E5H2" 0 "50S ribosomal protein L32"
"Chl_A19_00628" 628 "s63E6H2" 0 "50S ribosomal protein L32"
"Chl_A19_00629" 629 "s59E13H2" 0 "Polyketide biosynthesis malonyl CoA-acyl carrier protein transacylase PksC"
"Chl_A19_00629" 629 "s59E25H2" 0 "Polyketide biosynthesis malonyl CoA-acyl carrier protein transacylase PksC"
"Chl_A19_00629" 629 "s59E5H2" 0 "Polyketide biosynthesis malonyl CoA-acyl carrier protein transacylase PksC"
"Chl_A19_00629" 629 "s63E6H2" 0 "Polyketide biosynthesis malonyl CoA-acyl carrier protein transacylase PksC"
"Chl_A19_00630" 630 "s59E13H2" 0 "3-oxoacyl-[acyl-carrier-protein] reductase FabG"
"Chl_A19_00630" 630 "s59E25H2" 0 "3-oxoacyl-[acyl-carrier-protein] reductase FabG"
"Chl_A19_00630" 630 "s59E5H2" 0.00279883829516016 "3-oxoacyl-[acyl-carrier-protein] reductase FabG"
"Chl_A19_00630" 630 "s63E6H2" 0 "3-oxoacyl-[acyl-carrier-protein] reductase FabG"
"Chl_A19_00631" 631 "s59E13H2" 0 "hypothetical protein"
"Chl_A19_00631" 631 "s59E25H2" 0 "hypothetical protein"
"Chl_A19_00631" 631 "s59E5H2" 0 "hypothetical protein"
"Chl_A19_00631" 631 "s63E6H2" 0 "hypothetical protein"
"Chl_A19_00632" 632 "s59E13H2" 0 "Acyl carrier protein"
"Chl_A19_00632" 632 "s59E25H2" 0 "Acyl carrier protein"
"Chl_A19_00632" 632 "s59E5H2" 0 "Acyl carrier protein"
"Chl_A19_00632" 632 "s63E6H2" 0 "Acyl carrier protein"
"Chl_A19_00633" 633 "s59E13H2" 0 "putative HAD-hydrolase"
"Chl_A19_00633" 633 "s59E25H2" 0 "putative HAD-hydrolase"
"Chl_A19_00633" 633 "s59E5H2" 0 "putative HAD-hydrolase"
"Chl_A19_00633" 633 "s63E6H2" 0 "putative HAD-hydrolase"
"Chl_A19_00634" 634 "s59E13H2" 0 "Endonuclease MutS2"
"Chl_A19_00634" 634 "s59E25H2" 0 "Endonuclease MutS2"
"Chl_A19_00634" 634 "s59E5H2" 0 "Endonuclease MutS2"
"Chl_A19_00634" 634 "s63E6H2" 0 "Endonuclease MutS2"
"Chl_A19_00635" 635 "s59E13H2" 0 "putative MFS-type transporter YhjX"
"Chl_A19_00635" 635 "s59E25H2" 0 "putative MFS-type transporter YhjX"
"Chl_A19_00635" 635 "s59E5H2" 0 "putative MFS-type transporter YhjX"

"Chl_A19_00635" 635 "s63E6H2" 0 "putative MFS-type transporter YhjX"
"Chl_A19_00636" 636 "s59E13H2" 0 "Alcohol dehydrogenase"
"Chl_A19_00636" 636 "s59E25H2" 0 "Alcohol dehydrogenase"
"Chl_A19_00636" 636 "s59E5H2" 0 "Alcohol dehydrogenase"
"Chl_A19_00636" 636 "s63E6H2" 0 "Alcohol dehydrogenase"
"Chl_A19_00638" 638 "s59E13H2" 0 "Putative prophage phiRv2 integrase"
"Chl_A19_00638" 638 "s59E25H2" 0 "Putative prophage phiRv2 integrase"
"Chl_A19_00638" 638 "s59E5H2" 0 "Putative prophage phiRv2 integrase"
"Chl_A19_00638" 638 "s63E6H2" 0 "Putative prophage phiRv2 integrase"
"Chl_A19_00640" 640 "s59E13H2" 0 "Helix-turn-helix domain protein"
"Chl_A19_00640" 640 "s59E25H2" 0 "Helix-turn-helix domain protein"
"Chl_A19_00640" 640 "s59E5H2" 0 "Helix-turn-helix domain protein"
"Chl_A19_00640" 640 "s63E6H2" 0 "Helix-turn-helix domain protein"
"Chl_A21_00002" 642 "s59E13H2" 0 "Elongation factor G"
"Chl_A21_00002" 642 "s59E25H2" 0 "Elongation factor G"
"Chl_A21_00002" 642 "s59E5H2" 0 "Elongation factor G"
"Chl_A21_00002" 642 "s63E6H2" 0 "Elongation factor G"
"Chl_A21_00003" 643 "s59E13H2" 0 "Elongation factor Tu"
"Chl_A21_00003" 643 "s59E25H2" 0 "Elongation factor Tu"
"Chl_A21_00003" 643 "s59E5H2" 0 "Elongation factor Tu"
"Chl_A21_00003" 643 "s63E6H2" 0 "Elongation factor Tu"
"Chl_A21_00004" 644 "s59E13H2" 0 "30S ribosomal protein S10"
"Chl_A21_00004" 644 "s59E25H2" 0 "30S ribosomal protein S10"
"Chl_A21_00004" 644 "s59E5H2" 0 "30S ribosomal protein S10"
"Chl_A21_00004" 644 "s63E6H2" 0 "30S ribosomal protein S10"
"Chl_A21_00005" 645 "s59E13H2" 0 "50S ribosomal protein L3"
"Chl_A21_00005" 645 "s59E25H2" 0 "50S ribosomal protein L3"
"Chl_A21_00005" 645 "s59E5H2" 0 "50S ribosomal protein L3"
"Chl_A21_00005" 645 "s63E6H2" 0 "50S ribosomal protein L3"
"Chl_A21_00006" 646 "s59E13H2" 0 "50S ribosomal protein L4"
"Chl_A21_00006" 646 "s59E25H2" 0 "50S ribosomal protein L4"
"Chl_A21_00006" 646 "s59E5H2" 0 "50S ribosomal protein L4"
"Chl_A21_00006" 646 "s63E6H2" 0 "50S ribosomal protein L4"
"Chl_A21_00007" 647 "s59E13H2" 0 "50S ribosomal protein L23"
"Chl_A21_00007" 647 "s59E25H2" 0 "50S ribosomal protein L23"
"Chl_A21_00007" 647 "s59E5H2" 0 "50S ribosomal protein L23"
"Chl_A21_00007" 647 "s63E6H2" 0 "50S ribosomal protein L23"
"Chl_A21_00008" 648 "s59E13H2" 0 "hypothetical protein"
"Chl_A21_00008" 648 "s59E25H2" 0 "hypothetical protein"
"Chl_A21_00008" 648 "s59E5H2" 0 "hypothetical protein"
"Chl_A21_00008" 648 "s63E6H2" 0 "hypothetical protein"
"Chl_A21_00009" 649 "s59E13H2" 0 "50S ribosomal protein L2"
"Chl_A21_00009" 649 "s59E25H2" 0 "50S ribosomal protein L2"
"Chl_A21_00009" 649 "s59E5H2" 0 "50S ribosomal protein L2"
"Chl_A21_00009" 649 "s63E6H2" 0 "50S ribosomal protein L2"
"Chl_A21_00010" 650 "s59E13H2" 0 "30S ribosomal protein S19"
"Chl_A21_00010" 650 "s59E25H2" 0 "30S ribosomal protein S19"

"Chl_A21_00010" 650 "s59E5H2" 0 "30S ribosomal protein S19"
"Chl_A21_00010" 650 "s63E6H2" 0 "30S ribosomal protein S19"
"Chl_A21_00011" 651 "s59E13H2" 0 "50S ribosomal protein L22"
"Chl_A21_00011" 651 "s59E25H2" 0 "50S ribosomal protein L22"
"Chl_A21_00011" 651 "s59E5H2" 0 "50S ribosomal protein L22"
"Chl_A21_00011" 651 "s63E6H2" 0 "50S ribosomal protein L22"
"Chl_A21_00012" 652 "s59E13H2" 0 "30S ribosomal protein S3"
"Chl_A21_00012" 652 "s59E25H2" 0 "30S ribosomal protein S3"
"Chl_A21_00012" 652 "s59E5H2" 0 "30S ribosomal protein S3"
"Chl_A21_00012" 652 "s63E6H2" 0 "30S ribosomal protein S3"
"Chl_A21_00013" 653 "s59E13H2" 0 "50S ribosomal protein L16"
"Chl_A21_00013" 653 "s59E25H2" 0 "50S ribosomal protein L16"
"Chl_A21_00013" 653 "s59E5H2" 0 "50S ribosomal protein L16"
"Chl_A21_00013" 653 "s63E6H2" 0 "50S ribosomal protein L16"
"Chl_A21_00014" 654 "s59E13H2" 0 "50S ribosomal protein L29"
"Chl_A21_00014" 654 "s59E25H2" 0 "50S ribosomal protein L29"
"Chl_A21_00014" 654 "s59E5H2" 0 "50S ribosomal protein L29"
"Chl_A21_00014" 654 "s63E6H2" 0 "50S ribosomal protein L29"
"Chl_A21_00015" 655 "s59E13H2" 0 "30S ribosomal protein S17"
"Chl_A21_00015" 655 "s59E25H2" 0 "30S ribosomal protein S17"
"Chl_A21_00015" 655 "s59E5H2" 0 "30S ribosomal protein S17"
"Chl_A21_00015" 655 "s63E6H2" 0 "30S ribosomal protein S17"
"Chl_A21_00016" 656 "s59E13H2" 0 "50S ribosomal protein L14"
"Chl_A21_00016" 656 "s59E25H2" 0 "50S ribosomal protein L14"
"Chl_A21_00016" 656 "s59E5H2" 0 "50S ribosomal protein L14"
"Chl_A21_00016" 656 "s63E6H2" 0 "50S ribosomal protein L14"
"Chl_A21_00017" 657 "s59E13H2" 0 "50S ribosomal protein L24"
"Chl_A21_00017" 657 "s59E25H2" 0 "50S ribosomal protein L24"
"Chl_A21_00017" 657 "s59E5H2" 0 "50S ribosomal protein L24"
"Chl_A21_00017" 657 "s63E6H2" 0 "50S ribosomal protein L24"
"Chl_A21_00018" 658 "s59E13H2" 0 "50S ribosomal protein L5"
"Chl_A21_00018" 658 "s59E25H2" 0 "50S ribosomal protein L5"
"Chl_A21_00018" 658 "s59E5H2" 0 "50S ribosomal protein L5"
"Chl_A21_00018" 658 "s63E6H2" 0 "50S ribosomal protein L5"
"Chl_A21_00019" 659 "s59E13H2" 0 "30S ribosomal protein S8"
"Chl_A21_00019" 659 "s59E25H2" 0 "30S ribosomal protein S8"
"Chl_A21_00019" 659 "s59E5H2" 0 "30S ribosomal protein S8"
"Chl_A21_00019" 659 "s63E6H2" 0 "30S ribosomal protein S8"
"Chl_A21_00020" 660 "s59E13H2" 0 "50S ribosomal protein L6"
"Chl_A21_00020" 660 "s59E25H2" 0 "50S ribosomal protein L6"
"Chl_A21_00020" 660 "s59E5H2" 0 "50S ribosomal protein L6"
"Chl_A21_00020" 660 "s63E6H2" 0 "50S ribosomal protein L6"
"Chl_A21_00021" 661 "s59E13H2" 0 "50S ribosomal protein L18"
"Chl_A21_00021" 661 "s59E25H2" 0 "50S ribosomal protein L18"
"Chl_A21_00021" 661 "s59E5H2" 0 "50S ribosomal protein L18"
"Chl_A21_00021" 661 "s63E6H2" 0 "50S ribosomal protein L18"
"Chl_A21_00022" 662 "s59E13H2" 0 "30S ribosomal protein S5"

"Chl_A21_00022" 662 "s59E25H2" 0 "30S ribosomal protein S5"
"Chl_A21_00022" 662 "s59E5H2" 0 "30S ribosomal protein S5"
"Chl_A21_00022" 662 "s63E6H2" 0 "30S ribosomal protein S5"
"Chl_A21_00023" 663 "s59E13H2" 0 "50S ribosomal protein L30"
"Chl_A21_00023" 663 "s59E25H2" 0 "50S ribosomal protein L30"
"Chl_A21_00023" 663 "s59E5H2" 0 "50S ribosomal protein L30"
"Chl_A21_00023" 663 "s63E6H2" 0 "50S ribosomal protein L30"
"Chl_A21_00024" 664 "s59E13H2" 0 "50S ribosomal protein L15"
"Chl_A21_00024" 664 "s59E25H2" 0 "50S ribosomal protein L15"
"Chl_A21_00024" 664 "s59E5H2" 0 "50S ribosomal protein L15"
"Chl_A21_00024" 664 "s63E6H2" 0 "50S ribosomal protein L15"
"Chl_A21_00025" 665 "s59E13H2" 0 "preprotein translocase subunit SecY"
"Chl_A21_00025" 665 "s59E25H2" 0 "preprotein translocase subunit SecY"
"Chl_A21_00025" 665 "s59E5H2" 0 "preprotein translocase subunit SecY"
"Chl_A21_00025" 665 "s63E6H2" 0 "preprotein translocase subunit SecY"
"Chl_A21_00026" 666 "s59E13H2" 0 "Adenylate kinase"
"Chl_A21_00026" 666 "s59E25H2" 0 "Adenylate kinase"
"Chl_A21_00026" 666 "s59E5H2" 0 "Adenylate kinase"
"Chl_A21_00026" 666 "s63E6H2" 0 "Adenylate kinase"
"Chl_A21_00027" 667 "s59E13H2" 0 "Methionine aminopeptidase 1"
"Chl_A21_00027" 667 "s59E25H2" 0 "Methionine aminopeptidase 1"
"Chl_A21_00027" 667 "s59E5H2" 0 "Methionine aminopeptidase 1"
"Chl_A21_00027" 667 "s63E6H2" 0 "Methionine aminopeptidase 1"
"Chl_A21_00028" 668 "s59E13H2" 0 "50S ribosomal protein L36"
"Chl_A21_00028" 668 "s59E25H2" 0 "50S ribosomal protein L36"
"Chl_A21_00028" 668 "s59E5H2" 0 "50S ribosomal protein L36"
"Chl_A21_00028" 668 "s63E6H2" 0 "50S ribosomal protein L36"
"Chl_A21_00029" 669 "s59E13H2" 0 "30S ribosomal protein S13"
"Chl_A21_00029" 669 "s59E25H2" 0 "30S ribosomal protein S13"
"Chl_A21_00029" 669 "s59E5H2" 0 "30S ribosomal protein S13"
"Chl_A21_00029" 669 "s63E6H2" 0 "30S ribosomal protein S13"
"Chl_A21_00030" 670 "s59E13H2" 0 "30S ribosomal protein S11"
"Chl_A21_00030" 670 "s59E25H2" 0 "30S ribosomal protein S11"
"Chl_A21_00030" 670 "s59E5H2" 0 "30S ribosomal protein S11"
"Chl_A21_00030" 670 "s63E6H2" 0 "30S ribosomal protein S11"
"Chl_A21_00031" 671 "s59E13H2" 0 "30S ribosomal protein S4"
"Chl_A21_00031" 671 "s59E25H2" 0 "30S ribosomal protein S4"
"Chl_A21_00031" 671 "s59E5H2" 0 "30S ribosomal protein S4"
"Chl_A21_00031" 671 "s63E6H2" 0 "30S ribosomal protein S4"
"Chl_A21_00032" 672 "s59E13H2" 0 "DNA-directed RNA polymerase subunit alpha"
"Chl_A21_00032" 672 "s59E25H2" 0 "DNA-directed RNA polymerase subunit alpha"
"Chl_A21_00032" 672 "s59E5H2" 0 "DNA-directed RNA polymerase subunit alpha"
"Chl_A21_00032" 672 "s63E6H2" 0 "DNA-directed RNA polymerase subunit alpha"
"Chl_A21_00033" 673 "s59E13H2" 0 "50S ribosomal protein L17"
"Chl_A21_00033" 673 "s59E25H2" 0 "50S ribosomal protein L17"
"Chl_A21_00033" 673 "s59E5H2" 0 "50S ribosomal protein L17"
"Chl_A21_00033" 673 "s63E6H2" 0 "50S ribosomal protein L17"

"Chl_A21_00034" 674 "s59E13H2" 0 "tRNA pseudouridine synthase A"
"Chl_A21_00034" 674 "s59E25H2" 0 "tRNA pseudouridine synthase A"
"Chl_A21_00034" 674 "s59E5H2" 0 "tRNA pseudouridine synthase A"
"Chl_A21_00034" 674 "s63E6H2" 0 "tRNA pseudouridine synthase A"
"Chl_A21_00035" 675 "s59E13H2" 0 "50S ribosomal protein L13"
"Chl_A21_00035" 675 "s59E25H2" 0 "50S ribosomal protein L13"
"Chl_A21_00035" 675 "s59E5H2" 0 "50S ribosomal protein L13"
"Chl_A21_00035" 675 "s63E6H2" 0 "50S ribosomal protein L13"
"Chl_A21_00036" 676 "s59E13H2" 0 "30S ribosomal protein S9"
"Chl_A21_00036" 676 "s59E25H2" 0 "30S ribosomal protein S9"
"Chl_A21_00036" 676 "s59E5H2" 0 "30S ribosomal protein S9"
"Chl_A21_00036" 676 "s63E6H2" 0 "30S ribosomal protein S9"
"Chl_A21_00037" 677 "s59E13H2" 0 "hypothetical protein"
"Chl_A21_00037" 677 "s59E25H2" 0 "hypothetical protein"
"Chl_A21_00037" 677 "s59E5H2" 0 "hypothetical protein"
"Chl_A21_00037" 677 "s63E6H2" 0 "hypothetical protein"
"Chl_A21_00038" 678 "s59E13H2" 0 "Decaprenyl-phosphate phosphoribosyltransferase"
"Chl_A21_00038" 678 "s59E25H2" 0 "Decaprenyl-phosphate phosphoribosyltransferase"
"Chl_A21_00038" 678 "s59E5H2" 0 "Decaprenyl-phosphate phosphoribosyltransferase"
"Chl_A21_00038" 678 "s63E6H2" 0 "Decaprenyl-phosphate phosphoribosyltransferase"
"Chl_A21_00039" 679 "s59E13H2" 0 "16S/23S rRNA (cytidine-2'-O)-methyltransferase TlyA"
"Chl_A21_00039" 679 "s59E25H2" 0 "16S/23S rRNA (cytidine-2'-O)-methyltransferase TlyA"
"Chl_A21_00039" 679 "s59E5H2" 0 "16S/23S rRNA (cytidine-2'-O)-methyltransferase TlyA"
"Chl_A21_00039" 679 "s63E6H2" 0 "16S/23S rRNA (cytidine-2'-O)-methyltransferase TlyA"
"Chl_A21_00040" 680 "s59E13H2" 0 "hypothetical protein"
"Chl_A21_00040" 680 "s59E25H2" 0 "hypothetical protein"
"Chl_A21_00040" 680 "s59E5H2" 0 "hypothetical protein"
"Chl_A21_00040" 680 "s63E6H2" 0 "hypothetical protein"
"Chl_A21_00041" 681 "s59E13H2" 0 "Elongation factor 4"
"Chl_A21_00041" 681 "s59E25H2" 0 "Elongation factor 4"
"Chl_A21_00041" 681 "s59E5H2" 0 "Elongation factor 4"
"Chl_A21_00041" 681 "s63E6H2" 0 "Elongation factor 4"
"Chl_A21_00043" 683 "s59E13H2" 0 "Inner membrane transport protein YajR"
"Chl_A21_00043" 683 "s59E25H2" 0 "Inner membrane transport protein YajR"
"Chl_A21_00043" 683 "s59E5H2" 0 "Inner membrane transport protein YajR"
"Chl_A21_00043" 683 "s63E6H2" 0 "Inner membrane transport protein YajR"
"Chl_A21_00045" 685 "s59E13H2" 0 "Antibiotic biosynthesis monooxygenase"
"Chl_A21_00045" 685 "s59E25H2" 0 "Antibiotic biosynthesis monooxygenase"
"Chl_A21_00045" 685 "s59E5H2" 0 "Antibiotic biosynthesis monooxygenase"
"Chl_A21_00045" 685 "s63E6H2" 0 "Antibiotic biosynthesis monooxygenase"
"Chl_A21_00046" 686 "s59E13H2" 0 "hypothetical protein"
"Chl_A21_00046" 686 "s59E25H2" 0 "hypothetical protein"
"Chl_A21_00046" 686 "s59E5H2" 0 "hypothetical protein"
"Chl_A21_00046" 686 "s63E6H2" 0 "hypothetical protein"
"Chl_A21_00048" 688 "s59E13H2" 0 "hypothetical protein"
"Chl_A21_00048" 688 "s59E25H2" 0 "hypothetical protein"
"Chl_A21_00048" 688 "s59E5H2" 0 "hypothetical protein"

"Chl_A21_00048" 688 "s63E6H2" 0 "hypothetical protein"
"Chl_A21_00049" 689 "s59E13H2" 0 "DNA polymerase/3'-5' exonuclease PolX"
"Chl_A21_00049" 689 "s59E25H2" 0 "DNA polymerase/3'-5' exonuclease PolX"
"Chl_A21_00049" 689 "s59E5H2" 0 "DNA polymerase/3'-5' exonuclease PolX"
"Chl_A21_00049" 689 "s63E6H2" 0 "DNA polymerase/3'-5' exonuclease PolX"
"Chl_A21_00050" 690 "s59E13H2" 0 "hypothetical protein"
"Chl_A21_00050" 690 "s59E25H2" 0 "hypothetical protein"
"Chl_A21_00050" 690 "s59E5H2" 0 "hypothetical protein"
"Chl_A21_00050" 690 "s63E6H2" 0 "hypothetical protein"
"Chl_A21_00051" 691 "s59E13H2" 0 "Transposase IS200 like protein"
"Chl_A21_00051" 691 "s59E25H2" 0 "Transposase IS200 like protein"
"Chl_A21_00051" 691 "s59E5H2" 0 "Transposase IS200 like protein"
"Chl_A21_00051" 691 "s63E6H2" 0 "Transposase IS200 like protein"
"Chl_A21_00052" 692 "s59E13H2" 0 "hypothetical protein"
"Chl_A21_00052" 692 "s59E25H2" 0 "hypothetical protein"
"Chl_A21_00052" 692 "s59E5H2" 0 "hypothetical protein"
"Chl_A21_00052" 692 "s63E6H2" 0 "hypothetical protein"
"Chl_A21_00053" 693 "s59E13H2" 0 "hypothetical protein"
"Chl_A21_00053" 693 "s59E25H2" 0 "hypothetical protein"
"Chl_A21_00053" 693 "s59E5H2" 0 "hypothetical protein"
"Chl_A21_00053" 693 "s63E6H2" 0 "hypothetical protein"
"Chl_A21_00056" 696 "s59E13H2" 0 "hypothetical protein"
"Chl_A21_00056" 696 "s59E25H2" 0 "hypothetical protein"
"Chl_A21_00056" 696 "s59E5H2" 0 "hypothetical protein"
"Chl_A21_00056" 696 "s63E6H2" 0 "hypothetical protein"
"Chl_A21_00057" 697 "s59E13H2" 0 "hypothetical protein"
"Chl_A21_00057" 697 "s59E25H2" 0 "hypothetical protein"
"Chl_A21_00057" 697 "s59E5H2" 0 "hypothetical protein"
"Chl_A21_00057" 697 "s63E6H2" 0 "hypothetical protein"
"Chl_A21_00058" 698 "s59E13H2" 0 "HTH-type transcriptional regulator MalT"
"Chl_A21_00058" 698 "s59E25H2" 0 "HTH-type transcriptional regulator MalT"
"Chl_A21_00058" 698 "s59E5H2" 0 "HTH-type transcriptional regulator MalT"
"Chl_A21_00058" 698 "s63E6H2" 0 "HTH-type transcriptional regulator MalT"
"Chl_A21_00059" 699 "s59E13H2" 0 "hypothetical protein"
"Chl_A21_00059" 699 "s59E25H2" 0 "hypothetical protein"
"Chl_A21_00059" 699 "s59E5H2" 0 "hypothetical protein"
"Chl_A21_00059" 699 "s63E6H2" 0 "hypothetical protein"
"Chl_A21_00060" 700 "s59E13H2" 0 "Error-prone DNA polymerase"
"Chl_A21_00060" 700 "s59E25H2" 0 "Error-prone DNA polymerase"
"Chl_A21_00060" 700 "s59E5H2" 0 "Error-prone DNA polymerase"
"Chl_A21_00060" 700 "s63E6H2" 0 "Error-prone DNA polymerase"
"Chl_A21_00061" 701 "s59E13H2" 0 "DNA polymerase IV"
"Chl_A21_00061" 701 "s59E25H2" 0 "DNA polymerase IV"
"Chl_A21_00061" 701 "s59E5H2" 0 "DNA polymerase IV"
"Chl_A21_00061" 701 "s63E6H2" 0 "DNA polymerase IV"
"Chl_A21_00062" 702 "s59E13H2" 0 "hypothetical protein"
"Chl_A21_00062" 702 "s59E25H2" 0 "hypothetical protein"

"Chl_A21_00062" 702 "s59E5H2" 0 "hypothetical protein"
"Chl_A21_00062" 702 "s63E6H2" 0 "hypothetical protein"
"Chl_A21_00064" 704 "s59E13H2" 0 "hypothetical protein"
"Chl_A21_00064" 704 "s59E25H2" 0 "hypothetical protein"
"Chl_A21_00064" 704 "s59E5H2" 0 "hypothetical protein"
"Chl_A21_00064" 704 "s63E6H2" 0 "hypothetical protein"
"Chl_A21_00065" 705 "s59E13H2" 0 "hypothetical protein"
"Chl_A21_00065" 705 "s59E25H2" 0 "hypothetical protein"
"Chl_A21_00065" 705 "s59E5H2" 0 "hypothetical protein"
"Chl_A21_00065" 705 "s63E6H2" 0 "hypothetical protein"
"Chl_A21_00066" 706 "s59E13H2" 0 "DNA-invertase hin"
"Chl_A21_00066" 706 "s59E25H2" 0 "DNA-invertase hin"
"Chl_A21_00066" 706 "s59E5H2" 0 "DNA-invertase hin"
"Chl_A21_00066" 706 "s63E6H2" 0 "DNA-invertase hin"
"Chl_A21_00068" 708 "s59E13H2" 0 "Transposase, Mutator family"
"Chl_A21_00068" 708 "s59E25H2" 0 "Transposase, Mutator family"
"Chl_A21_00068" 708 "s59E5H2" 0 "Transposase, Mutator family"
"Chl_A21_00068" 708 "s63E6H2" 0 "Transposase, Mutator family"
"Chl_A21_00069" 709 "s59E13H2" 0 "Transposase, Mutator family"
"Chl_A21_00069" 709 "s59E25H2" 0 "Transposase, Mutator family"
"Chl_A21_00069" 709 "s59E5H2" 0 "Transposase, Mutator family"
"Chl_A21_00069" 709 "s63E6H2" 0 "Transposase, Mutator family"
"Chl_A21_00071" 711 "s59E13H2" 0 "Transposase IS116/IS110/IS902 family protein"
"Chl_A21_00071" 711 "s59E25H2" 0 "Transposase IS116/IS110/IS902 family protein"
"Chl_A21_00071" 711 "s59E5H2" 0 "Transposase IS116/IS110/IS902 family protein"
"Chl_A21_00071" 711 "s63E6H2" 0 "Transposase IS116/IS110/IS902 family protein"
"Chl_A21_00073" 713 "s59E13H2" 0 "D-inositol 3-phosphate glycosyltransferase"
"Chl_A21_00073" 713 "s59E25H2" 0 "D-inositol 3-phosphate glycosyltransferase"
"Chl_A21_00073" 713 "s59E5H2" 0 "D-inositol 3-phosphate glycosyltransferase"
"Chl_A21_00073" 713 "s63E6H2" 0 "D-inositol 3-phosphate glycosyltransferase"
"Chl_A21_00074" 714 "s59E13H2" 0 "hypothetical protein"
"Chl_A21_00074" 714 "s59E25H2" 0 "hypothetical protein"
"Chl_A21_00074" 714 "s59E5H2" 0 "hypothetical protein"
"Chl_A21_00074" 714 "s63E6H2" 0 "hypothetical protein"
"Chl_A21_00075" 715 "s59E13H2" 0 "threonine dehydratase"
"Chl_A21_00075" 715 "s59E25H2" 0 "threonine dehydratase"
"Chl_A21_00075" 715 "s59E5H2" 0 "threonine dehydratase"
"Chl_A21_00075" 715 "s63E6H2" 0 "threonine dehydratase"
"Chl_A21_00076" 716 "s59E13H2" 0 "Helix-turn-helix domain protein"
"Chl_A21_00076" 716 "s59E25H2" 0 "Helix-turn-helix domain protein"
"Chl_A21_00076" 716 "s59E5H2" 0 "Helix-turn-helix domain protein"
"Chl_A21_00076" 716 "s63E6H2" 0 "Helix-turn-helix domain protein"
"Chl_A21_00077" 717 "s59E13H2" 0 "Transcription antitermination protein RfaH"
"Chl_A21_00077" 717 "s59E25H2" 0 "Transcription antitermination protein RfaH"
"Chl_A21_00077" 717 "s59E5H2" 0 "Transcription antitermination protein RfaH"
"Chl_A21_00077" 717 "s63E6H2" 0 "Transcription antitermination protein RfaH"
"Chl_A21_00078" 718 "s59E13H2" 0 "tyrosine kinase"

"Chl_A21_00078" 718 "s59E25H2" 0 "tyrosine kinase"
"Chl_A21_00078" 718 "s59E5H2" 0 "tyrosine kinase"
"Chl_A21_00078" 718 "s63E6H2" 0 "tyrosine kinase"
"Chl_A21_00079" 719 "s59E13H2" 0 "O-Antigen ligase"
"Chl_A21_00079" 719 "s59E25H2" 0 "O-Antigen ligase"
"Chl_A21_00079" 719 "s59E5H2" 0 "O-Antigen ligase"
"Chl_A21_00079" 719 "s63E6H2" 0 "O-Antigen ligase"
"Chl_A21_00082" 722 "s59E13H2" 0 "putative peptidoglycan biosynthesis protein MurJ"
"Chl_A21_00082" 722 "s59E25H2" 0 "putative peptidoglycan biosynthesis protein MurJ"
"Chl_A21_00082" 722 "s59E5H2" 0 "putative peptidoglycan biosynthesis protein MurJ"
"Chl_A21_00082" 722 "s63E6H2" 0 "putative peptidoglycan biosynthesis protein MurJ"
"Chl_A21_00083" 723 "s59E13H2" 0 "Methyltransferase domain protein"
"Chl_A21_00083" 723 "s59E25H2" 0 "Methyltransferase domain protein"
"Chl_A21_00083" 723 "s59E5H2" 0 "Methyltransferase domain protein"
"Chl_A21_00083" 723 "s63E6H2" 0 "Methyltransferase domain protein"
"Chl_A21_00084" 724 "s59E13H2" 0 "2-deoxystreptamine glucosyltransferase"
"Chl_A21_00084" 724 "s59E25H2" 0 "2-deoxystreptamine glucosyltransferase"
"Chl_A21_00084" 724 "s59E5H2" 0 "2-deoxystreptamine glucosyltransferase"
"Chl_A21_00084" 724 "s63E6H2" 0 "2-deoxystreptamine glucosyltransferase"
"Chl_A21_00085" 725 "s59E13H2" 0 "D-inositol 3-phosphate glycosyltransferase"
"Chl_A21_00085" 725 "s59E25H2" 0 "D-inositol 3-phosphate glycosyltransferase"
"Chl_A21_00085" 725 "s59E5H2" 0 "D-inositol 3-phosphate glycosyltransferase"
"Chl_A21_00085" 725 "s63E6H2" 0 "D-inositol 3-phosphate glycosyltransferase"
"Chl_A21_00086" 726 "s59E13H2" 0 "GDP-mannose-dependent alpha-(1-6)-phosphatidylinositol monomannoside mannosyltransferase"
"Chl_A21_00086" 726 "s59E25H2" 0 "GDP-mannose-dependent alpha-(1-6)-phosphatidylinositol monomannoside mannosyltransferase"
"Chl_A21_00086" 726 "s59E5H2" 0 "GDP-mannose-dependent alpha-(1-6)-phosphatidylinositol monomannoside mannosyltransferase"
"Chl_A21_00086" 726 "s63E6H2" 0 "GDP-mannose-dependent alpha-(1-6)-phosphatidylinositol monomannoside mannosyltransferase"
"Chl_A21_00087" 727 "s59E13H2" 0 "Dimethylglycine N-methyltransferase"
"Chl_A21_00087" 727 "s59E25H2" 0 "Dimethylglycine N-methyltransferase"
"Chl_A21_00087" 727 "s59E5H2" 0 "Dimethylglycine N-methyltransferase"
"Chl_A21_00087" 727 "s63E6H2" 0 "Dimethylglycine N-methyltransferase"
"Chl_A21_00088" 728 "s59E13H2" 0 "bifunctional 3-demethylubiquinone-9 3-methyltransferase/ 2-octaprenyl-6-hydroxy phenol methylase"
"Chl_A21_00088" 728 "s59E25H2" 0 "bifunctional 3-demethylubiquinone-9 3-methyltransferase/ 2-octaprenyl-6-hydroxy phenol methylase"
"Chl_A21_00088" 728 "s59E5H2" 0 "bifunctional 3-demethylubiquinone-9 3-methyltransferase/ 2-octaprenyl-6-hydroxy phenol methylase"
"Chl_A21_00088" 728 "s63E6H2" 0 "bifunctional 3-demethylubiquinone-9 3-methyltransferase/ 2-octaprenyl-6-hydroxy phenol methylase"
"Chl_A21_00089" 729 "s59E13H2" 0 "GDP-mannose-dependent alpha-(1-2)-phosphatidylinositol mannosyltransferase"
"Chl_A21_00089" 729 "s59E25H2" 0 "GDP-mannose-dependent alpha-(1-2)-phosphatidylinositol mannosyltransferase"
"Chl_A21_00089" 729 "s59E5H2" 0 "GDP-mannose-dependent alpha-(1-2)-phosphatidylinositol mannosyltransferase"
"Chl_A21_00089" 729 "s63E6H2" 0 "GDP-mannose-dependent alpha-(1-2)-phosphatidylinositol mannosyltransferase"
"Chl_A21_00090" 730 "s59E13H2" 0 "UDP-N-acetylgalactosamine-undecaprenyl-phosphate N-acetylgalactosaminephosphotransferase"
"Chl_A21_00090" 730 "s59E25H2" 0 "UDP-N-acetylgalactosamine-undecaprenyl-phosphate N-acetylgalactosaminephosphotransferase"
"Chl_A21_00090" 730 "s59E5H2" 0 "UDP-N-acetylgalactosamine-undecaprenyl-phosphate N-acetylgalactosaminephosphotransferase"
"Chl_A21_00090" 730 "s63E6H2" 0 "UDP-N-acetylgalactosamine-undecaprenyl-phosphate N-acetylgalactosaminephosphotransferase"
"Chl_A21_00091" 731 "s59E13H2" 0 "Digeranylgeranylglycerophospholipid reductase"
"Chl_A21_00091" 731 "s59E25H2" 0 "Digeranylgeranylglycerophospholipid reductase"
"Chl_A21_00091" 731 "s59E5H2" 0 "Digeranylgeranylglycerophospholipid reductase"
"Chl_A21_00091" 731 "s63E6H2" 0 "Digeranylgeranylglycerophospholipid reductase"

"Chl_A21_00092" 732 "s59E13H2" 0 "UDP-glucose 4-epimerase"
"Chl_A21_00092" 732 "s59E25H2" 0 "UDP-glucose 4-epimerase"
"Chl_A21_00092" 732 "s59E5H2" 0 "UDP-glucose 4-epimerase"
"Chl_A21_00092" 732 "s63E6H2" 0 "UDP-glucose 4-epimerase"
"Chl_A21_00094" 734 "s59E13H2" 0 "Teichoic acid translocation permease protein TagG"
"Chl_A21_00094" 734 "s59E25H2" 0 "Teichoic acid translocation permease protein TagG"
"Chl_A21_00094" 734 "s59E5H2" 0 "Teichoic acid translocation permease protein TagG"
"Chl_A21_00094" 734 "s63E6H2" 0 "Teichoic acid translocation permease protein TagG"
"Chl_A21_00095" 735 "s59E13H2" 0 "Teichoic acids export ATP-binding protein TagH"
"Chl_A21_00095" 735 "s59E25H2" 0 "Teichoic acids export ATP-binding protein TagH"
"Chl_A21_00095" 735 "s59E5H2" 0 "Teichoic acids export ATP-binding protein TagH"
"Chl_A21_00095" 735 "s63E6H2" 0 "Teichoic acids export ATP-binding protein TagH"
"Chl_A21_00096" 736 "s59E13H2" 0 "putative S-adenosylmethionine-dependent methyltransferase/MSMEI_2290"
"Chl_A21_00096" 736 "s59E25H2" 0 "putative S-adenosylmethionine-dependent methyltransferase/MSMEI_2290"
"Chl_A21_00096" 736 "s59E5H2" 0 "putative S-adenosylmethionine-dependent methyltransferase/MSMEI_2290"
"Chl_A21_00096" 736 "s63E6H2" 0 "putative S-adenosylmethionine-dependent methyltransferase/MSMEI_2290"
"Chl_A21_00097" 737 "s59E13H2" 0 "hypothetical protein"
"Chl_A21_00097" 737 "s59E25H2" 0 "hypothetical protein"
"Chl_A21_00097" 737 "s59E5H2" 0 "hypothetical protein"
"Chl_A21_00097" 737 "s63E6H2" 0 "hypothetical protein"
"Chl_A21_00098" 738 "s59E13H2" 0 "Integrase core domain protein"
"Chl_A21_00098" 738 "s59E25H2" 0 "Integrase core domain protein"
"Chl_A21_00098" 738 "s59E5H2" 0 "Integrase core domain protein"
"Chl_A21_00098" 738 "s63E6H2" 0 "Integrase core domain protein"
"Chl_A21_00099" 739 "s59E13H2" 0 "hypothetical protein"
"Chl_A21_00099" 739 "s59E25H2" 0 "hypothetical protein"
"Chl_A21_00099" 739 "s59E5H2" 0 "hypothetical protein"
"Chl_A21_00099" 739 "s63E6H2" 0 "hypothetical protein"
"Chl_A21_00100" 740 "s59E13H2" 0 "hypothetical protein"
"Chl_A21_00100" 740 "s59E25H2" 0 "hypothetical protein"
"Chl_A21_00100" 740 "s59E5H2" 0 "hypothetical protein"
"Chl_A21_00100" 740 "s63E6H2" 0 "hypothetical protein"
"Chl_A21_00101" 741 "s59E13H2" 0 "hypothetical protein"
"Chl_A21_00101" 741 "s59E25H2" 0 "hypothetical protein"
"Chl_A21_00101" 741 "s59E5H2" 0 "hypothetical protein"
"Chl_A21_00101" 741 "s63E6H2" 0 "hypothetical protein"
"Chl_A21_00102" 742 "s59E13H2" 0 "hypothetical protein"
"Chl_A21_00102" 742 "s59E25H2" 0 "hypothetical protein"
"Chl_A21_00102" 742 "s59E5H2" 0 "hypothetical protein"
"Chl_A21_00102" 742 "s63E6H2" 0 "hypothetical protein"
"Chl_A21_00103" 743 "s59E13H2" 0 "Chondroitin synthase"
"Chl_A21_00103" 743 "s59E25H2" 0 "Chondroitin synthase"
"Chl_A21_00103" 743 "s59E5H2" 0 "Chondroitin synthase"
"Chl_A21_00103" 743 "s63E6H2" 0 "Chondroitin synthase"
"Chl_A21_00104" 744 "s59E13H2" 0 "D-inositol 3-phosphate glycosyltransferase"
"Chl_A21_00104" 744 "s59E25H2" 0 "D-inositol 3-phosphate glycosyltransferase"
"Chl_A21_00104" 744 "s59E5H2" 0 "D-inositol 3-phosphate glycosyltransferase"

"Chl_A21_00104" 744 "s63E6H2" 0 "D-inositol 3-phosphate glycosyltransferase"
"Chl_A21_00105" 745 "s59E13H2" 0 "N-acetylglucosaminyl-diphospho-decaprenol L-rhamnosyltransferase"
"Chl_A21_00105" 745 "s59E25H2" 0 "N-acetylglucosaminyl-diphospho-decaprenol L-rhamnosyltransferase"
"Chl_A21_00105" 745 "s59E5H2" 0 "N-acetylglucosaminyl-diphospho-decaprenol L-rhamnosyltransferase"
"Chl_A21_00105" 745 "s63E6H2" 0 "N-acetylglucosaminyl-diphospho-decaprenol L-rhamnosyltransferase"
"Chl_A21_00106" 746 "s59E13H2" 0 "Endoglucanase H precursor"
"Chl_A21_00106" 746 "s59E25H2" 0 "Endoglucanase H precursor"
"Chl_A21_00106" 746 "s59E5H2" 0 "Endoglucanase H precursor"
"Chl_A21_00106" 746 "s63E6H2" 0 "Endoglucanase H precursor"
"Chl_A21_00108" 748 "s59E13H2" 0 "GDP-mannose-dependent alpha-(1-2)-phosphatidylinositol mannosyltransferase"
"Chl_A21_00108" 748 "s59E25H2" 0 "GDP-mannose-dependent alpha-(1-2)-phosphatidylinositol mannosyltransferase"
"Chl_A21_00108" 748 "s59E5H2" 0 "GDP-mannose-dependent alpha-(1-2)-phosphatidylinositol mannosyltransferase"
"Chl_A21_00108" 748 "s63E6H2" 0 "GDP-mannose-dependent alpha-(1-2)-phosphatidylinositol mannosyltransferase"
"Chl_A21_00119" 759 "s59E13H2" 0 "DNA polymerase III subunit beta"
"Chl_A21_00119" 759 "s59E25H2" 0 "DNA polymerase III subunit beta"
"Chl_A21_00119" 759 "s59E5H2" 0 "DNA polymerase III subunit beta"
"Chl_A21_00119" 759 "s63E6H2" 0 "DNA polymerase III subunit beta"
"Chl_A21_00123" 763 "s59E13H2" 0 "ATP-dependent helicase HepA"
"Chl_A21_00123" 763 "s59E25H2" 0 "ATP-dependent helicase HepA"
"Chl_A21_00123" 763 "s59E5H2" 0 "ATP-dependent helicase HepA"
"Chl_A21_00123" 763 "s63E6H2" 0 "ATP-dependent helicase HepA"
"Chl_A21_00128" 768 "s59E13H2" 0 "GvpH"
"Chl_A21_00128" 768 "s59E25H2" 0 "GvpH"
"Chl_A21_00128" 768 "s59E5H2" 0 "GvpH"
"Chl_A21_00128" 768 "s63E6H2" 0 "GvpH"
"Chl_A21_00130" 770 "s59E13H2" 0 "VCP-like ATPase"
"Chl_A21_00130" 770 "s59E25H2" 0 "VCP-like ATPase"
"Chl_A21_00130" 770 "s59E5H2" 0 "VCP-like ATPase"
"Chl_A21_00130" 770 "s63E6H2" 0 "VCP-like ATPase"
"Chl_A21_00133" 773 "s59E13H2" 0 "Gas vesicle structural protein"
"Chl_A21_00133" 773 "s59E25H2" 0 "Gas vesicle structural protein"
"Chl_A21_00133" 773 "s59E5H2" 0 "Gas vesicle structural protein"
"Chl_A21_00133" 773 "s63E6H2" 0 "Gas vesicle structural protein"
"Chl_A21_00134" 774 "s59E13H2" 0 "Gas vesicle structural protein"
"Chl_A21_00134" 774 "s59E25H2" 0 "Gas vesicle structural protein"
"Chl_A21_00134" 774 "s59E5H2" 0 "Gas vesicle structural protein"
"Chl_A21_00134" 774 "s63E6H2" 0 "Gas vesicle structural protein"
"Chl_A21_00135" 775 "s59E13H2" 0 "hypothetical protein"
"Chl_A21_00135" 775 "s59E25H2" 0 "hypothetical protein"
"Chl_A21_00135" 775 "s59E5H2" 0 "hypothetical protein"
"Chl_A21_00135" 775 "s63E6H2" 0 "hypothetical protein"
"Chl_A21_00136" 776 "s59E13H2" 0 "Denitrification regulatory protein NirQ"
"Chl_A21_00136" 776 "s59E25H2" 0 "Denitrification regulatory protein NirQ"
"Chl_A21_00136" 776 "s59E5H2" 0 "Denitrification regulatory protein NirQ"
"Chl_A21_00136" 776 "s63E6H2" 0 "Denitrification regulatory protein NirQ"
"Chl_A21_00137" 777 "s59E13H2" 0 "hypothetical protein"
"Chl_A21_00137" 777 "s59E25H2" 0 "hypothetical protein"

"Chl_A21_00137" 777 "s59E5H2" 0 "hypothetical protein"
"Chl_A21_00137" 777 "s63E6H2" 0 "hypothetical protein"
"Chl_A21_00138" 778 "s59E13H2" 0 "Hsp20/alpha crystallin family protein"
"Chl_A21_00138" 778 "s59E25H2" 0 "Hsp20/alpha crystallin family protein"
"Chl_A21_00138" 778 "s59E5H2" 0 "Hsp20/alpha crystallin family protein"
"Chl_A21_00138" 778 "s63E6H2" 0 "Hsp20/alpha crystallin family protein"
"Chl_A21_00139" 779 "s59E13H2" 0 "Chaperone protein DnaK"
"Chl_A21_00139" 779 "s59E25H2" 0 "Chaperone protein DnaK"
"Chl_A21_00139" 779 "s59E5H2" 0 "Chaperone protein DnaK"
"Chl_A21_00139" 779 "s63E6H2" 0 "Chaperone protein DnaK"
"Chl_A21_00140" 780 "s59E13H2" 0 "chaperone protein DnaJ"
"Chl_A21_00140" 780 "s59E25H2" 0 "chaperone protein DnaJ"
"Chl_A21_00140" 780 "s59E5H2" 0 "chaperone protein DnaJ"
"Chl_A21_00140" 780 "s63E6H2" 0 "chaperone protein DnaJ"
"Chl_A21_00141" 781 "s59E13H2" 0 "Gas vesicle synthesis protein GvpL/GvpF"
"Chl_A21_00141" 781 "s59E25H2" 0 "Gas vesicle synthesis protein GvpL/GvpF"
"Chl_A21_00141" 781 "s59E5H2" 0 "Gas vesicle synthesis protein GvpL/GvpF"
"Chl_A21_00141" 781 "s63E6H2" 0 "Gas vesicle synthesis protein GvpL/GvpF"
"Chl_A21_00142" 782 "s59E13H2" 0 "chaperone protein DnaJ"
"Chl_A21_00142" 782 "s59E25H2" 0 "chaperone protein DnaJ"
"Chl_A21_00142" 782 "s59E5H2" 0 "chaperone protein DnaJ"
"Chl_A21_00142" 782 "s63E6H2" 0 "chaperone protein DnaJ"
"Chl_A21_00144" 784 "s59E13H2" 0 "chaperone protein DnaJ"
"Chl_A21_00144" 784 "s59E25H2" 0 "chaperone protein DnaJ"
"Chl_A21_00144" 784 "s59E5H2" 0 "chaperone protein DnaJ"
"Chl_A21_00144" 784 "s63E6H2" 0 "chaperone protein DnaJ"
"Chl_A21_00145" 785 "s59E13H2" 0 "Chaperone protein DnaJ"
"Chl_A21_00145" 785 "s59E25H2" 0 "Chaperone protein DnaJ"
"Chl_A21_00145" 785 "s59E5H2" 0 "Chaperone protein DnaJ"
"Chl_A21_00145" 785 "s63E6H2" 0 "Chaperone protein DnaJ"
"Chl_A21_00146" 786 "s59E13H2" 0 "Gas vesicle protein G"
"Chl_A21_00146" 786 "s59E25H2" 0 "Gas vesicle protein G"
"Chl_A21_00146" 786 "s59E5H2" 0 "Gas vesicle protein G"
"Chl_A21_00146" 786 "s63E6H2" 0 "Gas vesicle protein G"
"Chl_A21_00147" 787 "s59E13H2" 0 "Putative arsenical pump-driving ATPase"
"Chl_A21_00147" 787 "s59E25H2" 0 "Putative arsenical pump-driving ATPase"
"Chl_A21_00147" 787 "s59E5H2" 0 "Putative arsenical pump-driving ATPase"
"Chl_A21_00147" 787 "s63E6H2" 0 "Putative arsenical pump-driving ATPase"
"Chl_A21_00148" 788 "s59E13H2" 0 "hypothetical protein"
"Chl_A21_00148" 788 "s59E25H2" 0 "hypothetical protein"
"Chl_A21_00148" 788 "s59E5H2" 0 "hypothetical protein"
"Chl_A21_00148" 788 "s63E6H2" 0 "hypothetical protein"
"Chl_A21_00149" 789 "s59E13H2" 0 "Gas vesicle structural protein"
"Chl_A21_00149" 789 "s59E25H2" 0 "Gas vesicle structural protein"
"Chl_A21_00149" 789 "s59E5H2" 0 "Gas vesicle structural protein"
"Chl_A21_00149" 789 "s63E6H2" 0 "Gas vesicle structural protein"
"Chl_A21_00151" 791 "s59E13H2" 0 "Tyrosine phenol-lyase"

"Chl_A21_00151" 791 "s59E25H2" 0 "Tyrosine phenol-lyase"
"Chl_A21_00151" 791 "s59E5H2" 0 "Tyrosine phenol-lyase"
"Chl_A21_00151" 791 "s63E6H2" 0 "Tyrosine phenol-lyase"
"Chl_A21_00152" 792 "s59E13H2" 0 "Sugar-specific transcriptional regulator TrmB"
"Chl_A21_00152" 792 "s59E25H2" 0 "Sugar-specific transcriptional regulator TrmB"
"Chl_A21_00152" 792 "s59E5H2" 0 "Sugar-specific transcriptional regulator TrmB"
"Chl_A21_00152" 792 "s63E6H2" 0 "Sugar-specific transcriptional regulator TrmB"
"Chl_A21_00153" 793 "s59E13H2" 0 "Daunorubicin/doxorubicin resistance ATP-binding protein DrrA"
"Chl_A21_00153" 793 "s59E25H2" 0 "Daunorubicin/doxorubicin resistance ATP-binding protein DrrA"
"Chl_A21_00153" 793 "s59E5H2" 0 "Daunorubicin/doxorubicin resistance ATP-binding protein DrrA"
"Chl_A21_00153" 793 "s63E6H2" 0 "Daunorubicin/doxorubicin resistance ATP-binding protein DrrA"
"Chl_A21_00154" 794 "s59E13H2" 0 "ABC-2 family transporter protein"
"Chl_A21_00154" 794 "s59E25H2" 0 "ABC-2 family transporter protein"
"Chl_A21_00154" 794 "s59E5H2" 0 "ABC-2 family transporter protein"
"Chl_A21_00154" 794 "s63E6H2" 0 "ABC-2 family transporter protein"
"Chl_A21_00155" 795 "s59E13H2" 0 "Isoprenylcysteine carboxyl methyltransferase (ICMT) family protein"
"Chl_A21_00155" 795 "s59E25H2" 0 "Isoprenylcysteine carboxyl methyltransferase (ICMT) family protein"
"Chl_A21_00155" 795 "s59E5H2" 0 "Isoprenylcysteine carboxyl methyltransferase (ICMT) family protein"
"Chl_A21_00155" 795 "s63E6H2" 0 "Isoprenylcysteine carboxyl methyltransferase (ICMT) family protein"
"Chl_A21_00156" 796 "s59E13H2" 0 "Isoprenylcysteine carboxyl methyltransferase (ICMT) family protein"
"Chl_A21_00156" 796 "s59E25H2" 0 "Isoprenylcysteine carboxyl methyltransferase (ICMT) family protein"
"Chl_A21_00156" 796 "s59E5H2" 0 "Isoprenylcysteine carboxyl methyltransferase (ICMT) family protein"
"Chl_A21_00156" 796 "s63E6H2" 0 "Isoprenylcysteine carboxyl methyltransferase (ICMT) family protein"
"Chl_A21_00157" 797 "s59E13H2" 0 "Demethylrebeccamycin-D-glucose O-methyltransferase"
"Chl_A21_00157" 797 "s59E25H2" 0 "Demethylrebeccamycin-D-glucose O-methyltransferase"
"Chl_A21_00157" 797 "s59E5H2" 0 "Demethylrebeccamycin-D-glucose O-methyltransferase"
"Chl_A21_00157" 797 "s63E6H2" 0 "Demethylrebeccamycin-D-glucose O-methyltransferase"
"Chl_A21_00158" 798 "s59E13H2" 0 "Demethylrebeccamycin-D-glucose O-methyltransferase"
"Chl_A21_00158" 798 "s59E25H2" 0 "Demethylrebeccamycin-D-glucose O-methyltransferase"
"Chl_A21_00158" 798 "s59E5H2" 0 "Demethylrebeccamycin-D-glucose O-methyltransferase"
"Chl_A21_00158" 798 "s63E6H2" 0 "Demethylrebeccamycin-D-glucose O-methyltransferase"
"Chl_A21_00159" 799 "s59E13H2" 0 "Macrolide export ATP-binding/permease protein MacB"
"Chl_A21_00159" 799 "s59E25H2" 0 "Macrolide export ATP-binding/permease protein MacB"
"Chl_A21_00159" 799 "s59E5H2" 0 "Macrolide export ATP-binding/permease protein MacB"
"Chl_A21_00159" 799 "s63E6H2" 0 "Macrolide export ATP-binding/permease protein MacB"
"Chl_A21_00160" 800 "s59E13H2" 0 "Multidrug export protein EmrA"
"Chl_A21_00160" 800 "s59E25H2" 0 "Multidrug export protein EmrA"
"Chl_A21_00160" 800 "s59E5H2" 0 "Multidrug export protein EmrA"
"Chl_A21_00160" 800 "s63E6H2" 0 "Multidrug export protein EmrA"
"Chl_A21_00161" 801 "s59E13H2" 0 "putative ABC transporter ATP-binding protein"
"Chl_A21_00161" 801 "s59E25H2" 0 "putative ABC transporter ATP-binding protein"
"Chl_A21_00161" 801 "s59E5H2" 0 "putative ABC transporter ATP-binding protein"
"Chl_A21_00161" 801 "s63E6H2" 0 "putative ABC transporter ATP-binding protein"
"Chl_A21_00163" 803 "s59E13H2" 0 "Prolipoprotein diacylglyceryl transferase"
"Chl_A21_00163" 803 "s59E25H2" 0 "Prolipoprotein diacylglyceryl transferase"
"Chl_A21_00163" 803 "s59E5H2" 0 "Prolipoprotein diacylglyceryl transferase"
"Chl_A21_00163" 803 "s63E6H2" 0 "Prolipoprotein diacylglyceryl transferase"

"Chl_A21_00164" 804 "s59E13H2" 0 "Global nitrogen regulator"
"Chl_A21_00164" 804 "s59E25H2" 0 "Global nitrogen regulator"
"Chl_A21_00164" 804 "s59E5H2" 0 "Global nitrogen regulator"
"Chl_A21_00164" 804 "s63E6H2" 0 "Global nitrogen regulator"
"Chl_A21_00165" 805 "s59E13H2" 0 "Polymer-forming cytoskeletal"
"Chl_A21_00165" 805 "s59E25H2" 0 "Polymer-forming cytoskeletal"
"Chl_A21_00165" 805 "s59E5H2" 0 "Polymer-forming cytoskeletal"
"Chl_A21_00165" 805 "s63E6H2" 0 "Polymer-forming cytoskeletal"
"Chl_A21_00166" 806 "s59E13H2" 0 "hypothetical protein"
"Chl_A21_00166" 806 "s59E25H2" 0 "hypothetical protein"
"Chl_A21_00166" 806 "s59E5H2" 0 "hypothetical protein"
"Chl_A21_00166" 806 "s63E6H2" 0 "hypothetical protein"
"Chl_A21_00167" 807 "s59E13H2" 0 "dITP/XTP pyrophosphatase"
"Chl_A21_00167" 807 "s59E25H2" 0 "dITP/XTP pyrophosphatase"
"Chl_A21_00167" 807 "s59E5H2" 0 "dITP/XTP pyrophosphatase"
"Chl_A21_00167" 807 "s63E6H2" 0 "dITP/XTP pyrophosphatase"
"Chl_A21_00168" 808 "s59E13H2" 0 "2-hydroxy-6-oxo-6-phenylhexa-2,4-dienoate hydrolase"
"Chl_A21_00168" 808 "s59E25H2" 0 "2-hydroxy-6-oxo-6-phenylhexa-2,4-dienoate hydrolase"
"Chl_A21_00168" 808 "s59E5H2" 0 "2-hydroxy-6-oxo-6-phenylhexa-2,4-dienoate hydrolase"
"Chl_A21_00168" 808 "s63E6H2" 0 "2-hydroxy-6-oxo-6-phenylhexa-2,4-dienoate hydrolase"
"Chl_A21_00169" 809 "s59E13H2" 0 "Thioredoxin reductase"
"Chl_A21_00169" 809 "s59E25H2" 0 "Thioredoxin reductase"
"Chl_A21_00169" 809 "s59E5H2" 0 "Thioredoxin reductase"
"Chl_A21_00169" 809 "s63E6H2" 0 "Thioredoxin reductase"
"Chl_A21_00170" 810 "s59E13H2" 0 "putative S-adenosylmethionine-dependent methyltransferase/MSMEI_2290"
"Chl_A21_00170" 810 "s59E25H2" 0 "putative S-adenosylmethionine-dependent methyltransferase/MSMEI_2290"
"Chl_A21_00170" 810 "s59E5H2" 0 "putative S-adenosylmethionine-dependent methyltransferase/MSMEI_2290"
"Chl_A21_00170" 810 "s63E6H2" 0 "putative S-adenosylmethionine-dependent methyltransferase/MSMEI_2290"
"Chl_A21_00171" 811 "s59E13H2" 0 "hypothetical protein"
"Chl_A21_00171" 811 "s59E25H2" 0 "hypothetical protein"
"Chl_A21_00171" 811 "s59E5H2" 0 "hypothetical protein"
"Chl_A21_00171" 811 "s63E6H2" 0 "hypothetical protein"
"Chl_A21_00174" 814 "s59E13H2" 0 "Glutamate-1-semialdehyde 2,1-aminomutase 1"
"Chl_A21_00174" 814 "s59E25H2" 0 "Glutamate-1-semialdehyde 2,1-aminomutase 1"
"Chl_A21_00174" 814 "s59E5H2" 0 "Glutamate-1-semialdehyde 2,1-aminomutase 1"
"Chl_A21_00174" 814 "s63E6H2" 0 "Glutamate-1-semialdehyde 2,1-aminomutase 1"
"Chl_A21_00175" 815 "s59E13H2" 0 "hypothetical protein"
"Chl_A21_00175" 815 "s59E25H2" 0 "hypothetical protein"
"Chl_A21_00175" 815 "s59E5H2" 0 "hypothetical protein"
"Chl_A21_00175" 815 "s63E6H2" 0 "hypothetical protein"
"Chl_A21_00176" 816 "s59E13H2" 0 "CBS domain protein"
"Chl_A21_00176" 816 "s59E25H2" 0 "CBS domain protein"
"Chl_A21_00176" 816 "s59E5H2" 0 "CBS domain protein"
"Chl_A21_00176" 816 "s63E6H2" 0 "CBS domain protein"
"Chl_A21_00177" 817 "s59E13H2" 0 "Globin-coupled histidine kinase"
"Chl_A21_00177" 817 "s59E25H2" 0 "Globin-coupled histidine kinase"
"Chl_A21_00177" 817 "s59E5H2" 0 "Globin-coupled histidine kinase"

"Chl_A21_00177" 817 "s63E6H2" 0 "Globin-coupled histidine kinase"
"Chl_A21_00178" 818 "s59E13H2" 0 "Response regulator receiver protein"
"Chl_A21_00178" 818 "s59E25H2" 0 "Response regulator receiver protein"
"Chl_A21_00178" 818 "s59E5H2" 0 "Response regulator receiver protein"
"Chl_A21_00178" 818 "s63E6H2" 0 "Response regulator receiver protein"
"Chl_A21_00179" 819 "s59E13H2" 0 "Cyclic di-GMP phosphodiesterase response regulator RpfG"
"Chl_A21_00179" 819 "s59E25H2" 0 "Cyclic di-GMP phosphodiesterase response regulator RpfG"
"Chl_A21_00179" 819 "s59E5H2" 0 "Cyclic di-GMP phosphodiesterase response regulator RpfG"
"Chl_A21_00179" 819 "s63E6H2" 0.0001472956 "Cyclic di-GMP phosphodiesterase response regulator RpfG"
"Chl_A21_00180" 820 "s59E13H2" 0 "hypothetical protein"
"Chl_A21_00180" 820 "s59E25H2" 0 "hypothetical protein"
"Chl_A21_00180" 820 "s59E5H2" 0 "hypothetical protein"
"Chl_A21_00180" 820 "s63E6H2" 0 "hypothetical protein"
"Chl_A21_00181" 821 "s59E13H2" 0 "Tyrosine-protein kinase YwqD"
"Chl_A21_00181" 821 "s59E25H2" 0 "Tyrosine-protein kinase YwqD"
"Chl_A21_00181" 821 "s59E5H2" 0 "Tyrosine-protein kinase YwqD"
"Chl_A21_00181" 821 "s63E6H2" 0 "Tyrosine-protein kinase YwqD"
"Chl_A21_00182" 822 "s59E13H2" 0 "photosystem I assembly protein Ycf3"
"Chl_A21_00182" 822 "s59E25H2" 0 "photosystem I assembly protein Ycf3"
"Chl_A21_00182" 822 "s59E5H2" 0 "photosystem I assembly protein Ycf3"
"Chl_A21_00182" 822 "s63E6H2" 0 "photosystem I assembly protein Ycf3"
"Chl_A21_00183" 823 "s59E13H2" 0 "UDP-N-acetyl-D-glucosamine 6-dehydrogenase"
"Chl_A21_00183" 823 "s59E25H2" 0 "UDP-N-acetyl-D-glucosamine 6-dehydrogenase"
"Chl_A21_00183" 823 "s59E5H2" 0 "UDP-N-acetyl-D-glucosamine 6-dehydrogenase"
"Chl_A21_00183" 823 "s63E6H2" 0 "UDP-N-acetyl-D-glucosamine 6-dehydrogenase"
"Chl_A21_00184" 824 "s59E13H2" 0 "Teichoic acid translocation permease protein TagG"
"Chl_A21_00184" 824 "s59E25H2" 0 "Teichoic acid translocation permease protein TagG"
"Chl_A21_00184" 824 "s59E5H2" 0 "Teichoic acid translocation permease protein TagG"
"Chl_A21_00184" 824 "s63E6H2" 0 "Teichoic acid translocation permease protein TagG"
"Chl_A21_00185" 825 "s59E13H2" 0 "hypothetical protein"
"Chl_A21_00185" 825 "s59E25H2" 0 "hypothetical protein"
"Chl_A21_00185" 825 "s59E5H2" 0 "hypothetical protein"
"Chl_A21_00185" 825 "s63E6H2" 0 "hypothetical protein"
"Chl_A21_00186" 826 "s59E13H2" 0 "Phenylacetate-coenzyme A ligase"
"Chl_A21_00186" 826 "s59E25H2" 0 "Phenylacetate-coenzyme A ligase"
"Chl_A21_00186" 826 "s59E5H2" 0 "Phenylacetate-coenzyme A ligase"
"Chl_A21_00186" 826 "s63E6H2" 0 "Phenylacetate-coenzyme A ligase"
"Chl_A21_00187" 827 "s59E13H2" 0 "Alpha-D-kanosaminyltransferase"
"Chl_A21_00187" 827 "s59E25H2" 0 "Alpha-D-kanosaminyltransferase"
"Chl_A21_00187" 827 "s59E5H2" 0 "Alpha-D-kanosaminyltransferase"
"Chl_A21_00187" 827 "s63E6H2" 0 "Alpha-D-kanosaminyltransferase"
"Chl_A21_00188" 828 "s59E13H2" 0 "Peptidoglycan deacetylase"
"Chl_A21_00188" 828 "s59E25H2" 0 "Peptidoglycan deacetylase"
"Chl_A21_00188" 828 "s59E5H2" 0 "Peptidoglycan deacetylase"
"Chl_A21_00188" 828 "s63E6H2" 0 "Peptidoglycan deacetylase"
"Chl_A21_00189" 829 "s59E13H2" 0 "FemAB family protein"
"Chl_A21_00189" 829 "s59E25H2" 0 "FemAB family protein"

"Chl_A21_00189" 829 "s59E5H2" 0 "FemAB family protein"
"Chl_A21_00189" 829 "s63E6H2" 0 "FemAB family protein"
"Chl_A21_00190" 830 "s59E13H2" 0 "dTDP-4-amino-4,6-dideoxy-D-glucose transaminase"
"Chl_A21_00190" 830 "s59E25H2" 0 "dTDP-4-amino-4,6-dideoxy-D-glucose transaminase"
"Chl_A21_00190" 830 "s59E5H2" 0 "dTDP-4-amino-4,6-dideoxy-D-glucose transaminase"
"Chl_A21_00190" 830 "s63E6H2" 0 "dTDP-4-amino-4,6-dideoxy-D-glucose transaminase"
"Chl_A21_00191" 831 "s59E13H2" 0 "VanZ like family protein"
"Chl_A21_00191" 831 "s59E25H2" 0 "VanZ like family protein"
"Chl_A21_00191" 831 "s59E5H2" 0 "VanZ like family protein"
"Chl_A21_00191" 831 "s63E6H2" 0 "VanZ like family protein"
"Chl_A21_00192" 832 "s59E13H2" 0 "putative undecaprenyl-phosphate N-acetylglucosaminyl 1-phosphate transferase"
"Chl_A21_00192" 832 "s59E25H2" 0 "putative undecaprenyl-phosphate N-acetylglucosaminyl 1-phosphate transferase"
"Chl_A21_00192" 832 "s59E5H2" 0 "putative undecaprenyl-phosphate N-acetylglucosaminyl 1-phosphate transferase"
"Chl_A21_00192" 832 "s63E6H2" 0 "putative undecaprenyl-phosphate N-acetylglucosaminyl 1-phosphate transferase"
"Chl_A21_00193" 833 "s59E13H2" 0 "UDP-4-amino-4-deoxy-L-arabinose--oxoglutarate aminotransferase"
"Chl_A21_00193" 833 "s59E25H2" 0 "UDP-4-amino-4-deoxy-L-arabinose--oxoglutarate aminotransferase"
"Chl_A21_00193" 833 "s59E5H2" 0 "UDP-4-amino-4-deoxy-L-arabinose--oxoglutarate aminotransferase"
"Chl_A21_00193" 833 "s63E6H2" 0 "UDP-4-amino-4-deoxy-L-arabinose--oxoglutarate aminotransferase"
"Chl_A21_00194" 834 "s59E13H2" 0 "UDP-N-acetyl-alpha-D-glucosamine C6 dehydratase"
"Chl_A21_00194" 834 "s59E25H2" 0 "UDP-N-acetyl-alpha-D-glucosamine C6 dehydratase"
"Chl_A21_00194" 834 "s59E5H2" 0 "UDP-N-acetyl-alpha-D-glucosamine C6 dehydratase"
"Chl_A21_00194" 834 "s63E6H2" 0 "UDP-N-acetyl-alpha-D-glucosamine C6 dehydratase"
"Chl_A21_00195" 835 "s59E13H2" 0 "Macrolide export protein MacA"
"Chl_A21_00195" 835 "s59E25H2" 0 "Macrolide export protein MacA"
"Chl_A21_00195" 835 "s59E5H2" 0 "Macrolide export protein MacA"
"Chl_A21_00195" 835 "s63E6H2" 0 "Macrolide export protein MacA"
"Chl_A21_00196" 836 "s59E13H2" 0 "hypothetical protein"
"Chl_A21_00196" 836 "s59E25H2" 0 "hypothetical protein"
"Chl_A21_00196" 836 "s59E5H2" 0 "hypothetical protein"
"Chl_A21_00196" 836 "s63E6H2" 0 "hypothetical protein"
"Chl_A21_00198" 838 "s59E13H2" 0 "hypothetical protein"
"Chl_A21_00198" 838 "s59E25H2" 0 "hypothetical protein"
"Chl_A21_00198" 838 "s59E5H2" 0 "hypothetical protein"
"Chl_A21_00198" 838 "s63E6H2" 0 "hypothetical protein"
"Chl_A21_00199" 839 "s59E13H2" 0 "Digeranylgeranylglycerophospholipid reductase"
"Chl_A21_00199" 839 "s59E25H2" 0 "Digeranylgeranylglycerophospholipid reductase"
"Chl_A21_00199" 839 "s59E5H2" 0 "Digeranylgeranylglycerophospholipid reductase"
"Chl_A21_00199" 839 "s63E6H2" 0 "Digeranylgeranylglycerophospholipid reductase"
"Chl_A21_00200" 840 "s59E13H2" 0 "UDP-glucose 4-epimerase"
"Chl_A21_00200" 840 "s59E25H2" 0 "UDP-glucose 4-epimerase"
"Chl_A21_00200" 840 "s59E5H2" 0 "UDP-glucose 4-epimerase"
"Chl_A21_00200" 840 "s63E6H2" 0 "UDP-glucose 4-epimerase"
"Chl_A21_00201" 841 "s59E13H2" 0 "dTDP-4-dehydro-6-deoxyglucose reductase"
"Chl_A21_00201" 841 "s59E25H2" 0 "dTDP-4-dehydro-6-deoxyglucose reductase"
"Chl_A21_00201" 841 "s59E5H2" 0 "dTDP-4-dehydro-6-deoxyglucose reductase"
"Chl_A21_00201" 841 "s63E6H2" 0 "dTDP-4-dehydro-6-deoxyglucose reductase"
"Chl_A21_00202" 842 "s59E13H2" 0 "Phosphoheptose isomerase"

"Chl_A21_00202" 842 "s59E25H2" 0 "Phosphoheptose isomerase"
"Chl_A21_00202" 842 "s59E5H2" 0 "Phosphoheptose isomerase"
"Chl_A21_00202" 842 "s63E6H2" 0 "Phosphoheptose isomerase"
"Chl_A21_00203" 843 "s59E13H2" 0 "Phosphoglucomutase"
"Chl_A21_00203" 843 "s59E25H2" 0 "Phosphoglucomutase"
"Chl_A21_00203" 843 "s59E5H2" 0 "Phosphoglucomutase"
"Chl_A21_00203" 843 "s63E6H2" 0 "Phosphoglucomutase"
"Chl_A21_00205" 845 "s59E13H2" 0 "GDP-mannose-dependent alpha-(1-2)-phosphatidylinositol mannosyltransferase"
"Chl_A21_00205" 845 "s59E25H2" 0 "GDP-mannose-dependent alpha-(1-2)-phosphatidylinositol mannosyltransferase"
"Chl_A21_00205" 845 "s59E5H2" 0 "GDP-mannose-dependent alpha-(1-2)-phosphatidylinositol mannosyltransferase"
"Chl_A21_00205" 845 "s63E6H2" 0 "GDP-mannose-dependent alpha-(1-2)-phosphatidylinositol mannosyltransferase"
"Chl_A21_00207" 847 "s59E13H2" 0 "Acyl-coenzyme A thioesterase PaaI"
"Chl_A21_00207" 847 "s59E25H2" 0 "Acyl-coenzyme A thioesterase PaaI"
"Chl_A21_00207" 847 "s59E5H2" 0 "Acyl-coenzyme A thioesterase PaaI"
"Chl_A21_00207" 847 "s63E6H2" 0 "Acyl-coenzyme A thioesterase PaaI"
"Chl_A21_00208" 848 "s59E13H2" 0 "Translation initiation factor IF-1"
"Chl_A21_00208" 848 "s59E25H2" 0 "Translation initiation factor IF-1"
"Chl_A21_00208" 848 "s59E5H2" 0 "Translation initiation factor IF-1"
"Chl_A21_00208" 848 "s63E6H2" 0 "Translation initiation factor IF-1"
"Chl_A21_00209" 849 "s59E13H2" 0 "RNA polymerase-binding transcription factor CarD"
"Chl_A21_00209" 849 "s59E25H2" 0 "RNA polymerase-binding transcription factor CarD"
"Chl_A21_00209" 849 "s59E5H2" 0 "RNA polymerase-binding transcription factor CarD"
"Chl_A21_00209" 849 "s63E6H2" 0 "RNA polymerase-binding transcription factor CarD"
"Chl_A21_00210" 850 "s59E13H2" 0 "Bifunctional IPC transferase and DIPP synthase"
"Chl_A21_00210" 850 "s59E25H2" 0 "Bifunctional IPC transferase and DIPP synthase"
"Chl_A21_00210" 850 "s59E5H2" 0 "Bifunctional IPC transferase and DIPP synthase"
"Chl_A21_00210" 850 "s63E6H2" 0 "Bifunctional IPC transferase and DIPP synthase"
"Chl_A21_00211" 851 "s59E13H2" 0 "putative hydrolase"
"Chl_A21_00211" 851 "s59E25H2" 0 "putative hydrolase"
"Chl_A21_00211" 851 "s59E5H2" 0 "putative hydrolase"
"Chl_A21_00211" 851 "s63E6H2" 0 "putative hydrolase"
"Chl_A21_00213" 853 "s59E13H2" 0 "RNA polymerase sigma factor SigA"
"Chl_A21_00213" 853 "s59E25H2" 0 "RNA polymerase sigma factor SigA"
"Chl_A21_00213" 853 "s59E5H2" 0 "RNA polymerase sigma factor SigA"
"Chl_A21_00213" 853 "s63E6H2" 0 "RNA polymerase sigma factor SigA"
"Chl_A21_00214" 854 "s59E13H2" 0 "DNA primase"
"Chl_A21_00214" 854 "s59E25H2" 0 "DNA primase"
"Chl_A21_00214" 854 "s59E5H2" 0 "DNA primase"
"Chl_A21_00214" 854 "s63E6H2" 0 "DNA primase"
"Chl_A21_00215" 855 "s59E13H2" 0 "hypothetical protein"
"Chl_A21_00215" 855 "s59E25H2" 0 "hypothetical protein"
"Chl_A21_00215" 855 "s59E5H2" 0 "hypothetical protein"
"Chl_A21_00215" 855 "s63E6H2" 0 "hypothetical protein"
"Chl_A21_00216" 856 "s59E13H2" 0 "Nucleotidyltransferase domain protein"
"Chl_A21_00216" 856 "s59E25H2" 0 "Nucleotidyltransferase domain protein"
"Chl_A21_00216" 856 "s59E5H2" 0 "Nucleotidyltransferase domain protein"
"Chl_A21_00216" 856 "s63E6H2" 0 "Nucleotidyltransferase domain protein"

"Chl_A21_00217" 857 "s59E13H2" 0 "Ribonuclease Y"
"Chl_A21_00217" 857 "s59E25H2" 0 "Ribonuclease Y"
"Chl_A21_00217" 857 "s59E5H2" 0 "Ribonuclease Y"
"Chl_A21_00217" 857 "s63E6H2" 0 "Ribonuclease Y"
"Chl_A21_00218" 858 "s59E13H2" 0 "Regulatory protein RecX"
"Chl_A21_00218" 858 "s59E25H2" 0 "Regulatory protein RecX"
"Chl_A21_00218" 858 "s59E5H2" 0 "Regulatory protein RecX"
"Chl_A21_00218" 858 "s63E6H2" 0 "Regulatory protein RecX"
"Chl_A21_00219" 859 "s59E13H2" 0 "DNA repair and recombination protein RadA"
"Chl_A21_00219" 859 "s59E25H2" 0 "DNA repair and recombination protein RadA"
"Chl_A21_00219" 859 "s59E5H2" 0 "DNA repair and recombination protein RadA"
"Chl_A21_00219" 859 "s63E6H2" 0 "DNA repair and recombination protein RadA"
"Chl_A21_00222" 862 "s59E13H2" 0 "hypothetical protein"
"Chl_A21_00222" 862 "s59E25H2" 0 "hypothetical protein"
"Chl_A21_00222" 862 "s59E5H2" 0 "hypothetical protein"
"Chl_A21_00222" 862 "s63E6H2" 0 "hypothetical protein"
"Chl_A21_00223" 863 "s59E13H2" 0 "Dolichyl-phosphate-mannose-protein mannosyltransferase"
"Chl_A21_00223" 863 "s59E25H2" 0 "Dolichyl-phosphate-mannose-protein mannosyltransferase"
"Chl_A21_00223" 863 "s59E5H2" 0 "Dolichyl-phosphate-mannose-protein mannosyltransferase"
"Chl_A21_00223" 863 "s63E6H2" 0 "Dolichyl-phosphate-mannose-protein mannosyltransferase"
"Chl_A21_00224" 864 "s59E13H2" 0 "hypothetical protein"
"Chl_A21_00224" 864 "s59E25H2" 0.1186791 "hypothetical protein"
"Chl_A21_00224" 864 "s59E5H2" 0 "hypothetical protein"
"Chl_A21_00224" 864 "s63E6H2" 0 "hypothetical protein"
"Chl_A21_00225" 865 "s59E13H2" 0 "hypothetical protein"
"Chl_A21_00225" 865 "s59E25H2" 0 "hypothetical protein"
"Chl_A21_00225" 865 "s59E5H2" 0 "hypothetical protein"
"Chl_A21_00225" 865 "s63E6H2" 0 "hypothetical protein"
"Chl_A21_00226" 866 "s59E13H2" 0 "putative adenylyltransferase"
"Chl_A21_00226" 866 "s59E25H2" 0 "putative adenylyltransferase"
"Chl_A21_00226" 866 "s59E5H2" 0 "putative adenylyltransferase"
"Chl_A21_00226" 866 "s63E6H2" 0 "putative adenylyltransferase"
"Chl_A21_00227" 867 "s59E13H2" 0 "Putative peptidoglycan binding domain protein"
"Chl_A21_00227" 867 "s59E25H2" 0 "Putative peptidoglycan binding domain protein"
"Chl_A21_00227" 867 "s59E5H2" 0 "Putative peptidoglycan binding domain protein"
"Chl_A21_00227" 867 "s63E6H2" 0 "Putative peptidoglycan binding domain protein"
"Chl_A21_00228" 868 "s59E13H2" 0 "hypothetical protein"
"Chl_A21_00228" 868 "s59E25H2" 0 "hypothetical protein"
"Chl_A21_00228" 868 "s59E5H2" 0 "hypothetical protein"
"Chl_A21_00228" 868 "s63E6H2" 0 "hypothetical protein"
"Chl_A21_00229" 869 "s59E13H2" 0 "Methionine--tRNA ligase"
"Chl_A21_00229" 869 "s59E25H2" 0 "Methionine--tRNA ligase"
"Chl_A21_00229" 869 "s59E5H2" 0 "Methionine--tRNA ligase"
"Chl_A21_00229" 869 "s63E6H2" 0 "Methionine--tRNA ligase"
"Chl_A21_00230" 870 "s59E13H2" 0 "hypothetical protein"
"Chl_A21_00230" 870 "s59E25H2" 0 "hypothetical protein"
"Chl_A21_00230" 870 "s59E5H2" 0 "hypothetical protein"

"Chl_A21_00230" 870 "s63E6H2" 0 "hypothetical protein"
"Chl_A21_00233" 873 "s59E13H2" 0 "hypothetical protein"
"Chl_A21_00233" 873 "s59E25H2" 0 "hypothetical protein"
"Chl_A21_00233" 873 "s59E5H2" 0 "hypothetical protein"
"Chl_A21_00233" 873 "s63E6H2" 0 "hypothetical protein"
"Chl_A21_00234" 874 "s59E13H2" 0 "hypothetical protein"
"Chl_A21_00234" 874 "s59E25H2" 0 "hypothetical protein"
"Chl_A21_00234" 874 "s59E5H2" 0 "hypothetical protein"
"Chl_A21_00234" 874 "s63E6H2" 0 "hypothetical protein"
"Chl_A21_00235" 875 "s59E13H2" 0 "Chagasin family peptidase inhibitor I42"
"Chl_A21_00235" 875 "s59E25H2" 0 "Chagasin family peptidase inhibitor I42"
"Chl_A21_00235" 875 "s59E5H2" 0 "Chagasin family peptidase inhibitor I42"
"Chl_A21_00235" 875 "s63E6H2" 0 "Chagasin family peptidase inhibitor I42"
"Chl_A21_00236" 876 "s59E13H2" 0 "hypothetical protein"
"Chl_A21_00236" 876 "s59E25H2" 0 "hypothetical protein"
"Chl_A21_00236" 876 "s59E5H2" 0 "hypothetical protein"
"Chl_A21_00236" 876 "s63E6H2" 0 "hypothetical protein"
"Chl_A21_00237" 877 "s59E13H2" 0 "methionine synthase"
"Chl_A21_00237" 877 "s59E25H2" 0 "methionine synthase"
"Chl_A21_00237" 877 "s59E5H2" 0 "methionine synthase"
"Chl_A21_00237" 877 "s63E6H2" 0 "methionine synthase"
"Chl_A21_00238" 878 "s59E13H2" 0 "Transcription antitermination protein RfaH"
"Chl_A21_00238" 878 "s59E25H2" 0 "Transcription antitermination protein RfaH"
"Chl_A21_00238" 878 "s59E5H2" 0 "Transcription antitermination protein RfaH"
"Chl_A21_00238" 878 "s63E6H2" 0 "Transcription antitermination protein RfaH"
"Chl_A21_00239" 879 "s59E13H2" 0 "Transcriptional regulatory protein YycF"
"Chl_A21_00239" 879 "s59E25H2" 0 "Transcriptional regulatory protein YycF"
"Chl_A21_00239" 879 "s59E5H2" 0 "Transcriptional regulatory protein YycF"
"Chl_A21_00239" 879 "s63E6H2" 0 "Transcriptional regulatory protein YycF"
"Chl_A21_00240" 880 "s59E13H2" 0 "hypothetical protein"
"Chl_A21_00240" 880 "s59E25H2" 0 "hypothetical protein"
"Chl_A21_00240" 880 "s59E5H2" 0 "hypothetical protein"
"Chl_A21_00240" 880 "s63E6H2" 0 "hypothetical protein"
"Chl_A21_00241" 881 "s59E13H2" 0 "N-acetylmuramoyl-L-alanine amidase XlyA precursor"
"Chl_A21_00241" 881 "s59E25H2" 0 "N-acetylmuramoyl-L-alanine amidase XlyA precursor"
"Chl_A21_00241" 881 "s59E5H2" 0 "N-acetylmuramoyl-L-alanine amidase XlyA precursor"
"Chl_A21_00241" 881 "s63E6H2" 0 "N-acetylmuramoyl-L-alanine amidase XlyA precursor"
"Chl_A21_00242" 882 "s59E13H2" 0 "CysO-cysteine peptidase"
"Chl_A21_00242" 882 "s59E25H2" 0 "CysO-cysteine peptidase"
"Chl_A21_00242" 882 "s59E5H2" 0 "CysO-cysteine peptidase"
"Chl_A21_00242" 882 "s63E6H2" 0 "CysO-cysteine peptidase"
"Chl_A21_00243" 883 "s59E13H2" 0 "hypothetical protein"
"Chl_A21_00243" 883 "s59E25H2" 0 "hypothetical protein"
"Chl_A21_00243" 883 "s59E5H2" 0 "hypothetical protein"
"Chl_A21_00243" 883 "s63E6H2" 0 "hypothetical protein"
"Chl_A21_00245" 885 "s59E13H2" 0 "hypothetical protein"
"Chl_A21_00245" 885 "s59E25H2" 0 "hypothetical protein"

"Chl_A21_00245" 885 "s59E5H2" 0 "hypothetical protein"
"Chl_A21_00245" 885 "s63E6H2" 0 "hypothetical protein"
"Chl_A21_00246" 886 "s59E13H2" 0 "tRNA N6-adenosine threonylcarbamoyltransferase"
"Chl_A21_00246" 886 "s59E25H2" 0 "tRNA N6-adenosine threonylcarbamoyltransferase"
"Chl_A21_00246" 886 "s59E5H2" 0 "tRNA N6-adenosine threonylcarbamoyltransferase"
"Chl_A21_00246" 886 "s63E6H2" 0 "tRNA N6-adenosine threonylcarbamoyltransferase"
"Chl_A21_00247" 887 "s59E13H2" 0 "Glutaredoxin"
"Chl_A21_00247" 887 "s59E25H2" 0 "Glutaredoxin"
"Chl_A21_00247" 887 "s59E5H2" 0 "Glutaredoxin"
"Chl_A21_00247" 887 "s63E6H2" 0 "Glutaredoxin"
"Chl_A21_00248" 888 "s59E13H2" 0 "Bifunctional protein GlmU"
"Chl_A21_00248" 888 "s59E25H2" 0 "Bifunctional protein GlmU"
"Chl_A21_00248" 888 "s59E5H2" 0 "Bifunctional protein GlmU"
"Chl_A21_00248" 888 "s63E6H2" 0 "Bifunctional protein GlmU"
"Chl_A21_00249" 889 "s59E13H2" 0 "hypothetical protein"
"Chl_A21_00249" 889 "s59E25H2" 0 "hypothetical protein"
"Chl_A21_00249" 889 "s59E5H2" 0 "hypothetical protein"
"Chl_A21_00249" 889 "s63E6H2" 0 "hypothetical protein"
"Chl_A21_00250" 890 "s59E13H2" 0 "hypothetical protein"
"Chl_A21_00250" 890 "s59E25H2" 0 "hypothetical protein"
"Chl_A21_00250" 890 "s59E5H2" 0 "hypothetical protein"
"Chl_A21_00250" 890 "s63E6H2" 0 "hypothetical protein"
"Chl_A21_00251" 891 "s59E13H2" 0 "hypothetical protein"
"Chl_A21_00251" 891 "s59E25H2" 0 "hypothetical protein"
"Chl_A21_00251" 891 "s59E5H2" 0 "hypothetical protein"
"Chl_A21_00251" 891 "s63E6H2" 0 "hypothetical protein"
"Chl_A21_00252" 892 "s59E13H2" 0 "hypothetical protein"
"Chl_A21_00252" 892 "s59E25H2" 0 "hypothetical protein"
"Chl_A21_00252" 892 "s59E5H2" 0 "hypothetical protein"
"Chl_A21_00252" 892 "s63E6H2" 0 "hypothetical protein"
"Chl_A21_00253" 893 "s59E13H2" 0 "Phosphate-binding protein PstS 1 precursor"
"Chl_A21_00253" 893 "s59E25H2" 0 "Phosphate-binding protein PstS 1 precursor"
"Chl_A21_00253" 893 "s59E5H2" 0 "Phosphate-binding protein PstS 1 precursor"
"Chl_A21_00253" 893 "s63E6H2" 0 "Phosphate-binding protein PstS 1 precursor"
"Chl_A21_00254" 894 "s59E13H2" 0 "Undecaprenyl-diphosphatase"
"Chl_A21_00254" 894 "s59E25H2" 0 "Undecaprenyl-diphosphatase"
"Chl_A21_00254" 894 "s59E5H2" 0 "Undecaprenyl-diphosphatase"
"Chl_A21_00254" 894 "s63E6H2" 0 "Undecaprenyl-diphosphatase"
"Chl_A21_00255" 895 "s59E13H2" 0 "Queuine tRNA-ribosyltransferase"
"Chl_A21_00255" 895 "s59E25H2" 0 "Queuine tRNA-ribosyltransferase"
"Chl_A21_00255" 895 "s59E5H2" 0 "Queuine tRNA-ribosyltransferase"
"Chl_A21_00255" 895 "s63E6H2" 0 "Queuine tRNA-ribosyltransferase"
"Chl_A21_00256" 896 "s59E13H2" 0 "Zinc ribbon domain protein"
"Chl_A21_00256" 896 "s59E25H2" 0 "Zinc ribbon domain protein"
"Chl_A21_00256" 896 "s59E5H2" 0 "Zinc ribbon domain protein"
"Chl_A21_00256" 896 "s63E6H2" 0 "Zinc ribbon domain protein"
"Chl_A21_00257" 897 "s59E13H2" 0 "protease TldD"

"Chl_A21_00257" 897 "s59E25H2" 0 "protease TldD"
"Chl_A21_00257" 897 "s59E5H2" 0 "protease TldD"
"Chl_A21_00257" 897 "s63E6H2" 0 "protease TldD"
"Chl_A21_00258" 898 "s59E13H2" 0 "hypothetical protein"
"Chl_A21_00258" 898 "s59E25H2" 0 "hypothetical protein"
"Chl_A21_00258" 898 "s59E5H2" 0 "hypothetical protein"
"Chl_A21_00258" 898 "s63E6H2" 0 "hypothetical protein"
"Chl_A21_00259" 899 "s59E13H2" 0 "peptidase PmbA"
"Chl_A21_00259" 899 "s59E25H2" 0 "peptidase PmbA"
"Chl_A21_00259" 899 "s59E5H2" 0 "peptidase PmbA"
"Chl_A21_00259" 899 "s63E6H2" 0 "peptidase PmbA"
"Chl_A21_00260" 900 "s59E13H2" 0 "Acyl-CoA thioester hydrolase YbgC"
"Chl_A21_00260" 900 "s59E25H2" 0 "Acyl-CoA thioester hydrolase YbgC"
"Chl_A21_00260" 900 "s59E5H2" 0 "Acyl-CoA thioester hydrolase YbgC"
"Chl_A21_00260" 900 "s63E6H2" 0 "Acyl-CoA thioester hydrolase YbgC"
"Chl_A21_00261" 901 "s59E13H2" 0 "Possibl zinc metallo-peptidase"
"Chl_A21_00261" 901 "s59E25H2" 0 "Possibl zinc metallo-peptidase"
"Chl_A21_00261" 901 "s59E5H2" 0 "Possibl zinc metallo-peptidase"
"Chl_A21_00261" 901 "s63E6H2" 0 "Possibl zinc metallo-peptidase"
"Chl_A21_00262" 902 "s59E13H2" 0 "hypothetical protein"
"Chl_A21_00262" 902 "s59E25H2" 0 "hypothetical protein"
"Chl_A21_00262" 902 "s59E5H2" 0 "hypothetical protein"
"Chl_A21_00262" 902 "s63E6H2" 0 "hypothetical protein"
"Chl_A21_00263" 903 "s59E13H2" 0 "Formamidopyrimidine-DNA glycosylase"
"Chl_A21_00263" 903 "s59E25H2" 0 "Formamidopyrimidine-DNA glycosylase"
"Chl_A21_00263" 903 "s59E5H2" 0 "Formamidopyrimidine-DNA glycosylase"
"Chl_A21_00263" 903 "s63E6H2" 0 "Formamidopyrimidine-DNA glycosylase"
"Chl_A21_00265" 905 "s59E13H2" 0 "Acyl-CoA thioester hydrolase YbgC"
"Chl_A21_00265" 905 "s59E25H2" 0 "Acyl-CoA thioester hydrolase YbgC"
"Chl_A21_00265" 905 "s59E5H2" 0 "Acyl-CoA thioester hydrolase YbgC"
"Chl_A21_00265" 905 "s63E6H2" 0 "Acyl-CoA thioester hydrolase YbgC"
"Chl_A21_00266" 906 "s59E13H2" 0 "Coenzyme A biosynthesis bifunctional protein CoaBC"
"Chl_A21_00266" 906 "s59E25H2" 0 "Coenzyme A biosynthesis bifunctional protein CoaBC"
"Chl_A21_00266" 906 "s59E5H2" 0 "Coenzyme A biosynthesis bifunctional protein CoaBC"
"Chl_A21_00266" 906 "s63E6H2" 0 "Coenzyme A biosynthesis bifunctional protein CoaBC"
"Chl_A21_00267" 907 "s59E13H2" 0 "Coenzyme A biosynthesis bifunctional protein CoaBC"
"Chl_A21_00267" 907 "s59E25H2" 0 "Coenzyme A biosynthesis bifunctional protein CoaBC"
"Chl_A21_00267" 907 "s59E5H2" 0 "Coenzyme A biosynthesis bifunctional protein CoaBC"
"Chl_A21_00267" 907 "s63E6H2" 0 "Coenzyme A biosynthesis bifunctional protein CoaBC"
"Chl_A21_00268" 908 "s59E13H2" 0 "Acyl-coenzyme A:6-aminopenicillanic acid acyl-transferase"
"Chl_A21_00268" 908 "s59E25H2" 0 "Acyl-coenzyme A:6-aminopenicillanic acid acyl-transferase"
"Chl_A21_00268" 908 "s59E5H2" 0 "Acyl-coenzyme A:6-aminopenicillanic acid acyl-transferase"
"Chl_A21_00268" 908 "s63E6H2" 0 "Acyl-coenzyme A:6-aminopenicillanic acid acyl-transferase"
"Chl_A21_00269" 909 "s59E13H2" 0 "Radical SAM superfamily protein"
"Chl_A21_00269" 909 "s59E25H2" 0 "Radical SAM superfamily protein"
"Chl_A21_00269" 909 "s59E5H2" 0 "Radical SAM superfamily protein"
"Chl_A21_00269" 909 "s63E6H2" 0 "Radical SAM superfamily protein"

"Chl_A21_00270" 910 "s59E13H2" 0 "Trehalose synthase"
"Chl_A21_00270" 910 "s59E25H2" 0 "Trehalose synthase"
"Chl_A21_00270" 910 "s59E5H2" 0 "Trehalose synthase"
"Chl_A21_00270" 910 "s63E6H2" 0 "Trehalose synthase"
"Chl_A21_00271" 911 "s59E13H2" 0 "Pyruvate kinase"
"Chl_A21_00271" 911 "s59E25H2" 0 "Pyruvate kinase"
"Chl_A21_00271" 911 "s59E5H2" 0 "Pyruvate kinase"
"Chl_A21_00271" 911 "s63E6H2" 0 "Pyruvate kinase"
"Chl_A21_00272" 912 "s59E13H2" 0 "Glyceraldehyde-3-phosphate dehydrogenase"
"Chl_A21_00272" 912 "s59E25H2" 0 "Glyceraldehyde-3-phosphate dehydrogenase"
"Chl_A21_00272" 912 "s59E5H2" 0 "Glyceraldehyde-3-phosphate dehydrogenase"
"Chl_A21_00272" 912 "s63E6H2" 0 "Glyceraldehyde-3-phosphate dehydrogenase"
"Chl_A21_00273" 913 "s59E13H2" 0 "Phosphoglycerate kinase"
"Chl_A21_00273" 913 "s59E25H2" 0 "Phosphoglycerate kinase"
"Chl_A21_00273" 913 "s59E5H2" 0 "Phosphoglycerate kinase"
"Chl_A21_00273" 913 "s63E6H2" 0 "Phosphoglycerate kinase"
"Chl_A21_00274" 914 "s59E13H2" 0 "hypothetical protein"
"Chl_A21_00274" 914 "s59E25H2" 0 "hypothetical protein"
"Chl_A21_00274" 914 "s59E5H2" 0 "hypothetical protein"
"Chl_A21_00274" 914 "s63E6H2" 0 "hypothetical protein"
"Chl_A21_00275" 915 "s59E13H2" 0 "hypothetical protein"
"Chl_A21_00275" 915 "s59E25H2" 0 "hypothetical protein"
"Chl_A21_00275" 915 "s59E5H2" 0 "hypothetical protein"
"Chl_A21_00275" 915 "s63E6H2" 0 "hypothetical protein"
"Chl_A21_00277" 917 "s59E13H2" 0 "hypothetical protein"
"Chl_A21_00277" 917 "s59E25H2" 0 "hypothetical protein"
"Chl_A21_00277" 917 "s59E5H2" 0 "hypothetical protein"
"Chl_A21_00277" 917 "s63E6H2" 0 "hypothetical protein"
"Chl_A21_00278" 918 "s59E13H2" 0 "Glyoxylate reductase"
"Chl_A21_00278" 918 "s59E25H2" 0 "Glyoxylate reductase"
"Chl_A21_00278" 918 "s59E5H2" 0 "Glyoxylate reductase"
"Chl_A21_00278" 918 "s63E6H2" 0 "Glyoxylate reductase"
"Chl_A21_00280" 920 "s59E13H2" 0 "hypothetical protein"
"Chl_A21_00280" 920 "s59E25H2" 0 "hypothetical protein"
"Chl_A21_00280" 920 "s59E5H2" 0 "hypothetical protein"
"Chl_A21_00280" 920 "s63E6H2" 0 "hypothetical protein"
"Chl_A21_00285" 925 "s59E13H2" 0 "Ribosomal protein S12 methylthiotransferase RimO"
"Chl_A21_00285" 925 "s59E25H2" 0 "Ribosomal protein S12 methylthiotransferase RimO"
"Chl_A21_00285" 925 "s59E5H2" 0 "Ribosomal protein S12 methylthiotransferase RimO"
"Chl_A21_00285" 925 "s63E6H2" 0 "Ribosomal protein S12 methylthiotransferase RimO"
"Chl_A21_00286" 926 "s59E13H2" 0 "B12 binding domain protein"
"Chl_A21_00286" 926 "s59E25H2" 0 "B12 binding domain protein"
"Chl_A21_00286" 926 "s59E5H2" 0 "B12 binding domain protein"
"Chl_A21_00286" 926 "s63E6H2" 0 "B12 binding domain protein"
"Chl_A21_00287" 927 "s59E13H2" 0 "Endonuclease MutS2"
"Chl_A21_00287" 927 "s59E25H2" 0 "Endonuclease MutS2"
"Chl_A21_00287" 927 "s59E5H2" 0 "Endonuclease MutS2"

"Chl_A21_00287" 927 "s63E6H2" 0 "Endonuclease MutS2"
"Chl_A21_00288" 928 "s59E13H2" 0 "Colicin V production protein"
"Chl_A21_00288" 928 "s59E25H2" 0 "Colicin V production protein"
"Chl_A21_00288" 928 "s59E5H2" 0 "Colicin V production protein"
"Chl_A21_00288" 928 "s63E6H2" 0 "Colicin V production protein"
"Chl_A21_00289" 929 "s59E13H2" 0 "Phenylalanine--tRNA ligase beta subunit"
"Chl_A21_00289" 929 "s59E25H2" 0 "Phenylalanine--tRNA ligase beta subunit"
"Chl_A21_00289" 929 "s59E5H2" 0 "Phenylalanine--tRNA ligase beta subunit"
"Chl_A21_00289" 929 "s63E6H2" 0 "Phenylalanine--tRNA ligase beta subunit"
"Chl_A21_00291" 931 "s59E13H2" 0 "Phenylalanine--tRNA ligase alpha subunit"
"Chl_A21_00291" 931 "s59E25H2" 0 "Phenylalanine--tRNA ligase alpha subunit"
"Chl_A21_00291" 931 "s59E5H2" 0 "Phenylalanine--tRNA ligase alpha subunit"
"Chl_A21_00291" 931 "s63E6H2" 0 "Phenylalanine--tRNA ligase alpha subunit"
"Chl_A21_00292" 932 "s59E13H2" 0 "Transposase IS200 like protein"
"Chl_A21_00292" 932 "s59E25H2" 0 "Transposase IS200 like protein"
"Chl_A21_00292" 932 "s59E5H2" 0 "Transposase IS200 like protein"
"Chl_A21_00292" 932 "s63E6H2" 0 "Transposase IS200 like protein"
"Chl_A21_00293" 933 "s59E13H2" 0 "Putative TrmH family tRNA/rRNA methyltransferase"
"Chl_A21_00293" 933 "s59E25H2" 0 "Putative TrmH family tRNA/rRNA methyltransferase"
"Chl_A21_00293" 933 "s59E5H2" 0 "Putative TrmH family tRNA/rRNA methyltransferase"
"Chl_A21_00293" 933 "s63E6H2" 0 "Putative TrmH family tRNA/rRNA methyltransferase"
"Chl_A21_00294" 934 "s59E13H2" 0 "50S ribosomal protein L20"
"Chl_A21_00294" 934 "s59E25H2" 0 "50S ribosomal protein L20"
"Chl_A21_00294" 934 "s59E5H2" 0 "50S ribosomal protein L20"
"Chl_A21_00294" 934 "s63E6H2" 0 "50S ribosomal protein L20"
"Chl_A21_00295" 935 "s59E13H2" 0 "50S ribosomal protein L35"
"Chl_A21_00295" 935 "s59E25H2" 0 "50S ribosomal protein L35"
"Chl_A21_00295" 935 "s59E5H2" 0 "50S ribosomal protein L35"
"Chl_A21_00295" 935 "s63E6H2" 0 "50S ribosomal protein L35"
"Chl_A21_00296" 936 "s59E13H2" 0 "Translation initiation factor IF-3"
"Chl_A21_00296" 936 "s59E25H2" 0 "Translation initiation factor IF-3"
"Chl_A21_00296" 936 "s59E5H2" 0 "Translation initiation factor IF-3"
"Chl_A21_00296" 936 "s63E6H2" 0 "Translation initiation factor IF-3"
"Chl_A21_00297" 937 "s59E13H2" 0 "3-methyladenine DNA glycosylase"
"Chl_A21_00297" 937 "s59E25H2" 0 "3-methyladenine DNA glycosylase"
"Chl_A21_00297" 937 "s59E5H2" 0 "3-methyladenine DNA glycosylase"
"Chl_A21_00297" 937 "s63E6H2" 0 "3-methyladenine DNA glycosylase"
"Chl_A21_00298" 938 "s59E13H2" 0 "ThiS family protein"
"Chl_A21_00298" 938 "s59E25H2" 0 "ThiS family protein"
"Chl_A21_00298" 938 "s59E5H2" 0 "ThiS family protein"
"Chl_A21_00298" 938 "s63E6H2" 0 "ThiS family protein"
"Chl_A21_00299" 939 "s59E13H2" 0 "3-hydroxybutyryl-CoA dehydrogenase"
"Chl_A21_00299" 939 "s59E25H2" 0 "3-hydroxybutyryl-CoA dehydrogenase"
"Chl_A21_00299" 939 "s59E5H2" 0 "3-hydroxybutyryl-CoA dehydrogenase"
"Chl_A21_00299" 939 "s63E6H2" 0 "3-hydroxybutyryl-CoA dehydrogenase"
"Chl_A21_00301" 941 "s59E13H2" 0 "Redox-sensing transcriptional repressor Rex"
"Chl_A21_00301" 941 "s59E25H2" 0 "Redox-sensing transcriptional repressor Rex"

"Chl_A21_00301" 941 "s59E5H2" 0 "Redox-sensing transcriptional repressor Rex"
"Chl_A21_00301" 941 "s63E6H2" 0 "Redox-sensing transcriptional repressor Rex"
"Chl_A21_00302" 942 "s59E13H2" 0 "Rod shape-determining protein MreB"
"Chl_A21_00302" 942 "s59E25H2" 0 "Rod shape-determining protein MreB"
"Chl_A21_00302" 942 "s59E5H2" 0 "Rod shape-determining protein MreB"
"Chl_A21_00302" 942 "s63E6H2" 0 "Rod shape-determining protein MreB"
"Chl_A21_00303" 943 "s59E13H2" 0 "ATP synthase epsilon chain"
"Chl_A21_00303" 943 "s59E25H2" 0 "ATP synthase epsilon chain"
"Chl_A21_00303" 943 "s59E5H2" 0 "ATP synthase epsilon chain"
"Chl_A21_00303" 943 "s63E6H2" 0 "ATP synthase epsilon chain"
"Chl_A21_00304" 944 "s59E13H2" 0 "ATP synthase subunit beta"
"Chl_A21_00304" 944 "s59E25H2" 0 "ATP synthase subunit beta"
"Chl_A21_00304" 944 "s59E5H2" 0 "ATP synthase subunit beta"
"Chl_A21_00304" 944 "s63E6H2" 0 "ATP synthase subunit beta"
"Chl_A21_00306" 946 "s59E13H2" 0 "hypothetical protein"
"Chl_A21_00306" 946 "s59E25H2" 0 "hypothetical protein"
"Chl_A21_00306" 946 "s59E5H2" 0 "hypothetical protein"
"Chl_A21_00306" 946 "s63E6H2" 0 "hypothetical protein"
"Chl_A21_00308" 948 "s59E13H2" 0 "ATP synthase gamma chain"
"Chl_A21_00308" 948 "s59E25H2" 0 "ATP synthase gamma chain"
"Chl_A21_00308" 948 "s59E5H2" 0 "ATP synthase gamma chain"
"Chl_A21_00308" 948 "s63E6H2" 0 "ATP synthase gamma chain"
"Chl_A21_00309" 949 "s59E13H2" 0 "ATP synthase subunit alpha"
"Chl_A21_00309" 949 "s59E25H2" 0 "ATP synthase subunit alpha"
"Chl_A21_00309" 949 "s59E5H2" 0 "ATP synthase subunit alpha"
"Chl_A21_00309" 949 "s63E6H2" 0 "ATP synthase subunit alpha"
"Chl_A21_00310" 950 "s59E13H2" 0 "hypothetical protein"
"Chl_A21_00310" 950 "s59E25H2" 0 "hypothetical protein"
"Chl_A21_00310" 950 "s59E5H2" 0 "hypothetical protein"
"Chl_A21_00310" 950 "s63E6H2" 0 "hypothetical protein"
"Chl_A21_00312" 952 "s59E13H2" 0 "6-pyruvoyl-tetrahydropterin synthase related domain; membrane protein"
"Chl_A21_00312" 952 "s59E25H2" 0 "6-pyruvoyl-tetrahydropterin synthase related domain; membrane protein"
"Chl_A21_00312" 952 "s59E5H2" 0 "6-pyruvoyl-tetrahydropterin synthase related domain; membrane protein"
"Chl_A21_00312" 952 "s63E6H2" 0 "6-pyruvoyl-tetrahydropterin synthase related domain; membrane protein"
"Chl_A21_00313" 953 "s59E13H2" 0 "hypothetical protein"
"Chl_A21_00313" 953 "s59E25H2" 0 "hypothetical protein"
"Chl_A21_00313" 953 "s59E5H2" 0 "hypothetical protein"
"Chl_A21_00313" 953 "s63E6H2" 0 "hypothetical protein"
"Chl_A21_00315" 955 "s59E13H2" 0 "hypothetical protein"
"Chl_A21_00315" 955 "s59E25H2" 0 "hypothetical protein"
"Chl_A21_00315" 955 "s59E5H2" 0 "hypothetical protein"
"Chl_A21_00315" 955 "s63E6H2" 0 "hypothetical protein"
"Chl_A21_00316" 956 "s59E13H2" 0 "6-pyruvoyl-tetrahydropterin synthase related domain; membrane protein"
"Chl_A21_00316" 956 "s59E25H2" 0 "6-pyruvoyl-tetrahydropterin synthase related domain; membrane protein"
"Chl_A21_00316" 956 "s59E5H2" 0 "6-pyruvoyl-tetrahydropterin synthase related domain; membrane protein"
"Chl_A21_00316" 956 "s63E6H2" 0 "6-pyruvoyl-tetrahydropterin synthase related domain; membrane protein"
"Chl_A21_00318" 958 "s59E13H2" 0 "Radical SAM superfamily protein"

"Chl_A21_00318" 958 "s59E25H2" 0 "Radical SAM superfamily protein"
"Chl_A21_00318" 958 "s59E5H2" 0 "Radical SAM superfamily protein"
"Chl_A21_00318" 958 "s63E6H2" 0 "Radical SAM superfamily protein"
"Chl_A21_00319" 959 "s59E13H2" 0 "Radical SAM superfamily protein"
"Chl_A21_00319" 959 "s59E25H2" 0 "Radical SAM superfamily protein"
"Chl_A21_00319" 959 "s59E5H2" 0 "Radical SAM superfamily protein"
"Chl_A21_00319" 959 "s63E6H2" 0 "Radical SAM superfamily protein"
"Chl_A21_00321" 961 "s59E13H2" 0 "hypothetical protein"
"Chl_A21_00321" 961 "s59E25H2" 0 "hypothetical protein"
"Chl_A21_00321" 961 "s59E5H2" 0 "hypothetical protein"
"Chl_A21_00321" 961 "s63E6H2" 0 "hypothetical protein"
"Chl_A21_00323" 963 "s59E13H2" 0 "SNARE associated Golgi protein"
"Chl_A21_00323" 963 "s59E25H2" 0 "SNARE associated Golgi protein"
"Chl_A21_00323" 963 "s59E5H2" 0 "SNARE associated Golgi protein"
"Chl_A21_00323" 963 "s63E6H2" 0 "SNARE associated Golgi protein"
"Chl_A21_00324" 964 "s59E13H2" 0 "putative peptidyl-prolyl cis-trans isomerase"
"Chl_A21_00324" 964 "s59E25H2" 0 "putative peptidyl-prolyl cis-trans isomerase"
"Chl_A21_00324" 964 "s59E5H2" 0 "putative peptidyl-prolyl cis-trans isomerase"
"Chl_A21_00324" 964 "s63E6H2" 0 "putative peptidyl-prolyl cis-trans isomerase"
"Chl_A21_00325" 965 "s59E13H2" 0 "UDP-glucose 4-epimerase"
"Chl_A21_00325" 965 "s59E25H2" 0 "UDP-glucose 4-epimerase"
"Chl_A21_00325" 965 "s59E5H2" 0 "UDP-glucose 4-epimerase"
"Chl_A21_00325" 965 "s63E6H2" 0 "UDP-glucose 4-epimerase"
"Chl_A21_00326" 966 "s59E13H2" 0 "Uridine kinase"
"Chl_A21_00326" 966 "s59E25H2" 0 "Uridine kinase"
"Chl_A21_00326" 966 "s59E5H2" 0 "Uridine kinase"
"Chl_A21_00326" 966 "s63E6H2" 0 "Uridine kinase"
"Chl_A21_00327" 967 "s59E13H2" 0 "GTP pyrophosphokinase"
"Chl_A21_00327" 967 "s59E25H2" 0 "GTP pyrophosphokinase"
"Chl_A21_00327" 967 "s59E5H2" 0 "GTP pyrophosphokinase"
"Chl_A21_00327" 967 "s63E6H2" 0 "GTP pyrophosphokinase"
"Chl_A21_00328" 968 "s59E13H2" 0 "Foldase protein PrsA precursor"
"Chl_A21_00328" 968 "s59E25H2" 0 "Foldase protein PrsA precursor"
"Chl_A21_00328" 968 "s59E5H2" 0 "Foldase protein PrsA precursor"
"Chl_A21_00328" 968 "s63E6H2" 0 "Foldase protein PrsA precursor"
"Chl_A21_00329" 969 "s59E13H2" 0 "30S ribosomal protein S18"
"Chl_A21_00329" 969 "s59E25H2" 0 "30S ribosomal protein S18"
"Chl_A21_00329" 969 "s59E5H2" 0 "30S ribosomal protein S18"
"Chl_A21_00329" 969 "s63E6H2" 0 "30S ribosomal protein S18"
"Chl_A21_00330" 970 "s59E13H2" 0 "Single-stranded DNA-binding protein"
"Chl_A21_00330" 970 "s59E25H2" 0 "Single-stranded DNA-binding protein"
"Chl_A21_00330" 970 "s59E5H2" 0 "Single-stranded DNA-binding protein"
"Chl_A21_00330" 970 "s63E6H2" 0 "Single-stranded DNA-binding protein"
"Chl_A21_00331" 971 "s59E13H2" 0 "30S ribosomal protein S6"
"Chl_A21_00331" 971 "s59E25H2" 0 "30S ribosomal protein S6"
"Chl_A21_00331" 971 "s59E5H2" 0 "30S ribosomal protein S6"
"Chl_A21_00331" 971 "s63E6H2" 0 "30S ribosomal protein S6"

"Chl_A21_00332" 972 "s59E13H2" 0 "Ribosome-binding ATPase YchF"
"Chl_A21_00332" 972 "s59E25H2" 0 "Ribosome-binding ATPase YchF"
"Chl_A21_00332" 972 "s59E5H2" 0 "Ribosome-binding ATPase YchF"
"Chl_A21_00332" 972 "s63E6H2" 0 "Ribosome-binding ATPase YchF"
"Chl_A21_00333" 973 "s59E13H2" 0 "tRNA uridine 5-carboxymethylaminomethyl modification enzyme MnmG"
"Chl_A21_00333" 973 "s59E25H2" 0 "tRNA uridine 5-carboxymethylaminomethyl modification enzyme MnmG"
"Chl_A21_00333" 973 "s59E5H2" 0 "tRNA uridine 5-carboxymethylaminomethyl modification enzyme MnmG"
"Chl_A21_00333" 973 "s63E6H2" 0 "tRNA uridine 5-carboxymethylaminomethyl modification enzyme MnmG"
"Chl_A21_00334" 974 "s59E13H2" 0 "hypothetical protein"
"Chl_A21_00334" 974 "s59E25H2" 0 "hypothetical protein"
"Chl_A21_00334" 974 "s59E5H2" 0 "hypothetical protein"
"Chl_A21_00334" 974 "s63E6H2" 0 "hypothetical protein"
"Chl_A21_00335" 975 "s59E13H2" 0 "hypothetical protein"
"Chl_A21_00335" 975 "s59E25H2" 0 "hypothetical protein"
"Chl_A21_00335" 975 "s59E5H2" 0 "hypothetical protein"
"Chl_A21_00335" 975 "s63E6H2" 0 "hypothetical protein"
"Chl_A21_00336" 976 "s59E13H2" 0 "Dolichyl-phosphate-mannose-protein mannosyltransferase"
"Chl_A21_00336" 976 "s59E25H2" 0 "Dolichyl-phosphate-mannose-protein mannosyltransferase"
"Chl_A21_00336" 976 "s59E5H2" 0 "Dolichyl-phosphate-mannose-protein mannosyltransferase"
"Chl_A21_00336" 976 "s63E6H2" 0 "Dolichyl-phosphate-mannose-protein mannosyltransferase"
"Chl_A21_00337" 977 "s59E13H2" 0 "CDP-paratose 2-epimerase"
"Chl_A21_00337" 977 "s59E25H2" 0 "CDP-paratose 2-epimerase"
"Chl_A21_00337" 977 "s59E5H2" 0 "CDP-paratose 2-epimerase"
"Chl_A21_00337" 977 "s63E6H2" 0 "CDP-paratose 2-epimerase"
"Chl_A21_00338" 978 "s59E13H2" 0 "Phosphoadenosine phosphosulfate reductase"
"Chl_A21_00338" 978 "s59E25H2" 0 "Phosphoadenosine phosphosulfate reductase"
"Chl_A21_00338" 978 "s59E5H2" 0 "Phosphoadenosine phosphosulfate reductase"
"Chl_A21_00338" 978 "s63E6H2" 0 "Phosphoadenosine phosphosulfate reductase"
"Chl_A21_00339" 979 "s59E13H2" 0 "Bifunctional protein PaaZ"
"Chl_A21_00339" 979 "s59E25H2" 0 "Bifunctional protein PaaZ"
"Chl_A21_00339" 979 "s59E5H2" 0 "Bifunctional protein PaaZ"
"Chl_A21_00339" 979 "s63E6H2" 0 "Bifunctional protein PaaZ"
"Chl_A21_00340" 980 "s59E13H2" 0 "Spermidine synthase"
"Chl_A21_00340" 980 "s59E25H2" 0 "Spermidine synthase"
"Chl_A21_00340" 980 "s59E5H2" 0 "Spermidine synthase"
"Chl_A21_00340" 980 "s63E6H2" 0 "Spermidine synthase"
"Chl_A21_00341" 981 "s59E13H2" 0 "Protein-L-isoaspartate O-methyltransferase"
"Chl_A21_00341" 981 "s59E25H2" 0 "Protein-L-isoaspartate O-methyltransferase"
"Chl_A21_00341" 981 "s59E5H2" 0 "Protein-L-isoaspartate O-methyltransferase"
"Chl_A21_00341" 981 "s63E6H2" 0 "Protein-L-isoaspartate O-methyltransferase"
"Chl_A21_00342" 982 "s59E13H2" 0 "putative L-threonine 3-dehydrogenase"
"Chl_A21_00342" 982 "s59E25H2" 0 "putative L-threonine 3-dehydrogenase"
"Chl_A21_00342" 982 "s59E5H2" 0 "putative L-threonine 3-dehydrogenase"
"Chl_A21_00342" 982 "s63E6H2" 0 "putative L-threonine 3-dehydrogenase"
"Chl_A21_00343" 983 "s59E13H2" 0 "Putative pyridoxal phosphate-dependent acyltransferase"
"Chl_A21_00343" 983 "s59E25H2" 0 "Putative pyridoxal phosphate-dependent acyltransferase"
"Chl_A21_00343" 983 "s59E5H2" 0 "Putative pyridoxal phosphate-dependent acyltransferase"

"Chl_A21_00343" 983 "s63E6H2" 0 "Putative pyridoxal phosphate-dependent acyltransferase"
"Chl_A21_00344" 984 "s59E13H2" 0 "hypothetical protein"
"Chl_A21_00344" 984 "s59E25H2" 0 "hypothetical protein"
"Chl_A21_00344" 984 "s59E5H2" 0 "hypothetical protein"
"Chl_A21_00344" 984 "s63E6H2" 0 "hypothetical protein"
"Chl_A21_00345" 985 "s59E13H2" 0 "Peptidoglycan endopeptidase LytF precursor"
"Chl_A21_00345" 985 "s59E25H2" 0 "Peptidoglycan endopeptidase LytF precursor"
"Chl_A21_00345" 985 "s59E5H2" 0 "Peptidoglycan endopeptidase LytF precursor"
"Chl_A21_00345" 985 "s63E6H2" 0 "Peptidoglycan endopeptidase LytF precursor"
"Chl_A21_00346" 986 "s59E13H2" 0 "Transaldolase"
"Chl_A21_00346" 986 "s59E25H2" 0 "Transaldolase"
"Chl_A21_00346" 986 "s59E5H2" 0 "Transaldolase"
"Chl_A21_00346" 986 "s63E6H2" 0 "Transaldolase"
"Chl_A21_00347" 987 "s59E13H2" 0 "hypothetical protein"
"Chl_A21_00347" 987 "s59E25H2" 0 "hypothetical protein"
"Chl_A21_00347" 987 "s59E5H2" 0 "hypothetical protein"
"Chl_A21_00347" 987 "s63E6H2" 0 "hypothetical protein"
"Chl_A21_00351" 991 "s59E13H2" 0 "Cell division protein FtsL"
"Chl_A21_00351" 991 "s59E25H2" 0 "Cell division protein FtsL"
"Chl_A21_00351" 991 "s59E5H2" 0 "Cell division protein FtsL"
"Chl_A21_00351" 991 "s63E6H2" 0 "Cell division protein FtsL"
"Chl_A21_00354" 994 "s59E13H2" 0 "RNA recognition motif. (a.k.a. RRM, RBD, or RNP domain)"
"Chl_A21_00354" 994 "s59E25H2" 0 "RNA recognition motif. (a.k.a. RRM, RBD, or RNP domain)"
"Chl_A21_00354" 994 "s59E5H2" 0 "RNA recognition motif. (a.k.a. RRM, RBD, or RNP domain)"
"Chl_A21_00354" 994 "s63E6H2" 0 "RNA recognition motif. (a.k.a. RRM, RBD, or RNP domain)"
"Chl_A21_00355" 995 "s59E13H2" 0 "hypothetical protein"
"Chl_A21_00355" 995 "s59E25H2" 0 "hypothetical protein"
"Chl_A21_00355" 995 "s59E5H2" 0 "hypothetical protein"
"Chl_A21_00355" 995 "s63E6H2" 0 "hypothetical protein"
"Chl_A21_00357" 997 "s59E13H2" 0 "hypothetical protein"
"Chl_A21_00357" 997 "s59E25H2" 0 "hypothetical protein"
"Chl_A21_00357" 997 "s59E5H2" 0 "hypothetical protein"
"Chl_A21_00357" 997 "s63E6H2" 0 "hypothetical protein"
"Chl_A21_00358" 998 "s59E13H2" 0 "GDP-mannose-dependent alpha-(1-2)-phosphatidylinositol mannosyltransferase"
"Chl_A21_00358" 998 "s59E25H2" 0 "GDP-mannose-dependent alpha-(1-2)-phosphatidylinositol mannosyltransferase"
"Chl_A21_00358" 998 "s59E5H2" 0 "GDP-mannose-dependent alpha-(1-2)-phosphatidylinositol mannosyltransferase"
"Chl_A21_00358" 998 "s63E6H2" 0 "GDP-mannose-dependent alpha-(1-2)-phosphatidylinositol mannosyltransferase"
"Chl_A21_00359" 999 "s59E13H2" 0 "Sugar-binding cellulase-like protein"
"Chl_A21_00359" 999 "s59E25H2" 0 "Sugar-binding cellulase-like protein"
"Chl_A21_00359" 999 "s59E5H2" 0 "Sugar-binding cellulase-like protein"
"Chl_A21_00359" 999 "s63E6H2" 0 "Sugar-binding cellulase-like protein"
"Chl_A21_00360" 1000 "s59E13H2" 0 "Glycogen synthase"
"Chl_A21_00360" 1000 "s59E25H2" 0 "Glycogen synthase"
"Chl_A21_00360" 1000 "s59E5H2" 0 "Glycogen synthase"
"Chl_A21_00360" 1000 "s63E6H2" 0 "Glycogen synthase"
"Chl_A21_00361" 1001 "s59E13H2" 0 "Beta-1,4-mannooligosaccharide phosphorylase"
"Chl_A21_00361" 1001 "s59E25H2" 0 "Beta-1,4-mannooligosaccharide phosphorylase"

"Chl_A21_00361" 1001 "s59E5H2" 0 "Beta-1,4-mannooligosaccharide phosphorylase"
"Chl_A21_00361" 1001 "s63E6H2" 0 "Beta-1,4-mannooligosaccharide phosphorylase"
"Chl_A21_00362" 1002 "s59E13H2" 0 "Copper-sensing transcriptional repressor CsoR"
"Chl_A21_00362" 1002 "s59E25H2" 0 "Copper-sensing transcriptional repressor CsoR"
"Chl_A21_00362" 1002 "s59E5H2" 0 "Copper-sensing transcriptional repressor CsoR"
"Chl_A21_00362" 1002 "s63E6H2" 0 "Copper-sensing transcriptional repressor CsoR"
"Chl_A21_00363" 1003 "s59E13H2" 0 "Copper-exporting P-type ATPase A"
"Chl_A21_00363" 1003 "s59E25H2" 0 "Copper-exporting P-type ATPase A"
"Chl_A21_00363" 1003 "s59E5H2" 0 "Copper-exporting P-type ATPase A"
"Chl_A21_00363" 1003 "s63E6H2" 0 "Copper-exporting P-type ATPase A"
"Chl_A21_00364" 1004 "s59E13H2" 0 "hypothetical protein"
"Chl_A21_00364" 1004 "s59E25H2" 0 "hypothetical protein"
"Chl_A21_00364" 1004 "s59E5H2" 0 "hypothetical protein"
"Chl_A21_00364" 1004 "s63E6H2" 0 "hypothetical protein"
"Chl_A21_00365" 1005 "s59E13H2" 0 "hypothetical protein"
"Chl_A21_00365" 1005 "s59E25H2" 0 "hypothetical protein"
"Chl_A21_00365" 1005 "s59E5H2" 0 "hypothetical protein"
"Chl_A21_00365" 1005 "s63E6H2" 0 "hypothetical protein"
"Chl_A21_00366" 1006 "s59E13H2" 0 "CCA-adding enzyme"
"Chl_A21_00366" 1006 "s59E25H2" 0 "CCA-adding enzyme"
"Chl_A21_00366" 1006 "s59E5H2" 0 "CCA-adding enzyme"
"Chl_A21_00366" 1006 "s63E6H2" 0 "CCA-adding enzyme"
"Chl_A21_00367" 1007 "s59E13H2" 0 "2-oxoglutarate synthase subunit KorA"
"Chl_A21_00367" 1007 "s59E25H2" 0 "2-oxoglutarate synthase subunit KorA"
"Chl_A21_00367" 1007 "s59E5H2" 0 "2-oxoglutarate synthase subunit KorA"
"Chl_A21_00367" 1007 "s63E6H2" 0 "2-oxoglutarate synthase subunit KorA"
"Chl_A21_00368" 1008 "s59E13H2" 0 "lipoprotein NlpI"
"Chl_A21_00368" 1008 "s59E25H2" 0 "lipoprotein NlpI"
"Chl_A21_00368" 1008 "s59E5H2" 0 "lipoprotein NlpI"
"Chl_A21_00368" 1008 "s63E6H2" 0 "lipoprotein NlpI"
"Chl_A21_00370" 1010 "s59E13H2" 0 "hypothetical protein"
"Chl_A21_00370" 1010 "s59E25H2" 0 "hypothetical protein"
"Chl_A21_00370" 1010 "s59E5H2" 0 "hypothetical protein"
"Chl_A21_00370" 1010 "s63E6H2" 0 "hypothetical protein"
"Chl_A21_00371" 1011 "s59E13H2" 0 "Divalent-cation tolerance protein CutA"
"Chl_A21_00371" 1011 "s59E25H2" 0 "Divalent-cation tolerance protein CutA"
"Chl_A21_00371" 1011 "s59E5H2" 0 "Divalent-cation tolerance protein CutA"
"Chl_A21_00371" 1011 "s63E6H2" 0 "Divalent-cation tolerance protein CutA"
"Chl_A21_00372" 1012 "s59E13H2" 0 "Trimethylamine corrinoid protein"
"Chl_A21_00372" 1012 "s59E25H2" 0 "Trimethylamine corrinoid protein"
"Chl_A21_00372" 1012 "s59E5H2" 0 "Trimethylamine corrinoid protein"
"Chl_A21_00372" 1012 "s63E6H2" 0 "Trimethylamine corrinoid protein"
"Chl_A21_00373" 1013 "s59E13H2" 0 "Serine/threonine-protein kinase pkn1"
"Chl_A21_00373" 1013 "s59E25H2" 0 "Serine/threonine-protein kinase pkn1"
"Chl_A21_00373" 1013 "s59E5H2" 0 "Serine/threonine-protein kinase pkn1"
"Chl_A21_00373" 1013 "s63E6H2" 0 "Serine/threonine-protein kinase pkn1"
"Chl_A21_00374" 1014 "s59E13H2" 0 "Multiple sugar-binding protein precursor"

"Chl_A21_00374" 1014 "s59E25H2" 0 "Multiple sugar-binding protein precursor"
"Chl_A21_00374" 1014 "s59E5H2" 0 "Multiple sugar-binding protein precursor"
"Chl_A21_00374" 1014 "s63E6H2" 0 "Multiple sugar-binding protein precursor"
"Chl_A21_00375" 1015 "s59E13H2" 0 "Lactose transport system permease protein LacF"
"Chl_A21_00375" 1015 "s59E25H2" 0 "Lactose transport system permease protein LacF"
"Chl_A21_00375" 1015 "s59E5H2" 0 "Lactose transport system permease protein LacF"
"Chl_A21_00375" 1015 "s63E6H2" 0 "Lactose transport system permease protein LacF"
"Chl_A21_00376" 1016 "s59E13H2" 0 "L-arabinose transport system permease protein AraQ"
"Chl_A21_00376" 1016 "s59E25H2" 0 "L-arabinose transport system permease protein AraQ"
"Chl_A21_00376" 1016 "s59E5H2" 0 "L-arabinose transport system permease protein AraQ"
"Chl_A21_00376" 1016 "s63E6H2" 0 "L-arabinose transport system permease protein AraQ"
"Chl_A21_00377" 1017 "s59E13H2" 0 "DNA adenine methyltransferase YhdJ"
"Chl_A21_00377" 1017 "s59E25H2" 0 "DNA adenine methyltransferase YhdJ"
"Chl_A21_00377" 1017 "s59E5H2" 0 "DNA adenine methyltransferase YhdJ"
"Chl_A21_00377" 1017 "s63E6H2" 0 "DNA adenine methyltransferase YhdJ"
"Chl_A21_00378" 1018 "s59E13H2" 0 "Type III restriction enzyme, res subunit"
"Chl_A21_00378" 1018 "s59E25H2" 0 "Type III restriction enzyme, res subunit"
"Chl_A21_00378" 1018 "s59E5H2" 0 "Type III restriction enzyme, res subunit"
"Chl_A21_00378" 1018 "s63E6H2" 0 "Type III restriction enzyme, res subunit"
"Chl_A21_00379" 1019 "s59E13H2" 0 "ThiS family protein"
"Chl_A21_00379" 1019 "s59E25H2" 0 "ThiS family protein"
"Chl_A21_00379" 1019 "s59E5H2" 0 "ThiS family protein"
"Chl_A21_00379" 1019 "s63E6H2" 0 "ThiS family protein"
"Chl_A21_00380" 1020 "s59E13H2" 0 "sn-glycerol-3-phosphate dehydrogenase subunit C"
"Chl_A21_00380" 1020 "s59E25H2" 0 "sn-glycerol-3-phosphate dehydrogenase subunit C"
"Chl_A21_00380" 1020 "s59E5H2" 0 "sn-glycerol-3-phosphate dehydrogenase subunit C"
"Chl_A21_00380" 1020 "s63E6H2" 0 "sn-glycerol-3-phosphate dehydrogenase subunit C"
"Chl_A21_00381" 1021 "s59E13H2" 0 "Sulfhydrogenase 1 subunit gamma"
"Chl_A21_00381" 1021 "s59E25H2" 0 "Sulfhydrogenase 1 subunit gamma"
"Chl_A21_00381" 1021 "s59E5H2" 0 "Sulfhydrogenase 1 subunit gamma"
"Chl_A21_00381" 1021 "s63E6H2" 0 "Sulfhydrogenase 1 subunit gamma"
"Chl_A21_00382" 1022 "s59E13H2" 0 "Sulfhydrogenase 2 subunit beta"
"Chl_A21_00382" 1022 "s59E25H2" 0 "Sulfhydrogenase 2 subunit beta"
"Chl_A21_00382" 1022 "s59E5H2" 0 "Sulfhydrogenase 2 subunit beta"
"Chl_A21_00382" 1022 "s63E6H2" 0 "Sulfhydrogenase 2 subunit beta"
"Chl_A21_00383" 1023 "s59E13H2" 0 "Formate dehydrogenase subunit beta"
"Chl_A21_00383" 1023 "s59E25H2" 0 "Formate dehydrogenase subunit beta"
"Chl_A21_00383" 1023 "s59E5H2" 0 "Formate dehydrogenase subunit beta"
"Chl_A21_00383" 1023 "s63E6H2" 0 "Formate dehydrogenase subunit beta"
"Chl_A21_00384" 1024 "s59E13H2" 0 "F420-non-reducing hydrogenase vhc iron-sulfur subunit D"
"Chl_A21_00384" 1024 "s59E25H2" 0 "F420-non-reducing hydrogenase vhc iron-sulfur subunit D"
"Chl_A21_00384" 1024 "s59E5H2" 0 "F420-non-reducing hydrogenase vhc iron-sulfur subunit D"
"Chl_A21_00384" 1024 "s63E6H2" 0 "F420-non-reducing hydrogenase vhc iron-sulfur subunit D"
"Chl_A21_00385" 1025 "s59E13H2" 0 "Heterodisulfide reductase subunit A-like protein"
"Chl_A21_00385" 1025 "s59E25H2" 0 "Heterodisulfide reductase subunit A-like protein"
"Chl_A21_00385" 1025 "s59E5H2" 0 "Heterodisulfide reductase subunit A-like protein"
"Chl_A21_00385" 1025 "s63E6H2" 0 "Heterodisulfide reductase subunit A-like protein"

"Chl_A21_00386" 1026 "s59E13H2" 0 "CoB--CoM heterodisulfide reductase iron-sulfur subunit A"
"Chl_A21_00386" 1026 "s59E25H2" 0 "CoB--CoM heterodisulfide reductase iron-sulfur subunit A"
"Chl_A21_00386" 1026 "s59E5H2" 0 "CoB--CoM heterodisulfide reductase iron-sulfur subunit A"
"Chl_A21_00386" 1026 "s63E6H2" 0 "CoB--CoM heterodisulfide reductase iron-sulfur subunit A"
"Chl_A21_00387" 1027 "s59E13H2" 0 "hypothetical protein"
"Chl_A21_00387" 1027 "s59E25H2" 0 "hypothetical protein"
"Chl_A21_00387" 1027 "s59E5H2" 0 "hypothetical protein"
"Chl_A21_00387" 1027 "s63E6H2" 0 "hypothetical protein"
"Chl_A21_00388" 1028 "s59E13H2" 0 "Electron transport protein HydN"
"Chl_A21_00388" 1028 "s59E25H2" 0 "Electron transport protein HydN"
"Chl_A21_00388" 1028 "s59E5H2" 0 "Electron transport protein HydN"
"Chl_A21_00388" 1028 "s63E6H2" 0 "Electron transport protein HydN"
"Chl_A21_00389" 1029 "s59E13H2" 0 "Tungsten-containing aldehyde ferredoxin oxidoreductase"
"Chl_A21_00389" 1029 "s59E25H2" 0 "Tungsten-containing aldehyde ferredoxin oxidoreductase"
"Chl_A21_00389" 1029 "s59E5H2" 0 "Tungsten-containing aldehyde ferredoxin oxidoreductase"
"Chl_A21_00389" 1029 "s63E6H2" 0 "Tungsten-containing aldehyde ferredoxin oxidoreductase"
"Chl_A21_00391" 1031 "s59E13H2" 0 "Acetyl-CoA acetyltransferase"
"Chl_A21_00391" 1031 "s59E25H2" 0 "Acetyl-CoA acetyltransferase"
"Chl_A21_00391" 1031 "s59E5H2" 0 "Acetyl-CoA acetyltransferase"
"Chl_A21_00391" 1031 "s63E6H2" 0 "Acetyl-CoA acetyltransferase"
"Chl_A21_00392" 1032 "s59E13H2" 0 "R-phenyllactate dehydratase activator"
"Chl_A21_00392" 1032 "s59E25H2" 0 "R-phenyllactate dehydratase activator"
"Chl_A21_00392" 1032 "s59E5H2" 0 "R-phenyllactate dehydratase activator"
"Chl_A21_00392" 1032 "s63E6H2" 0 "R-phenyllactate dehydratase activator"
"Chl_A21_00393" 1033 "s59E13H2" 0 "R-phenyllactate dehydratase subunit alpha precursor"
"Chl_A21_00393" 1033 "s59E25H2" 0 "R-phenyllactate dehydratase subunit alpha precursor"
"Chl_A21_00393" 1033 "s59E5H2" 0 "R-phenyllactate dehydratase subunit alpha precursor"
"Chl_A21_00393" 1033 "s63E6H2" 0 "R-phenyllactate dehydratase subunit alpha precursor"
"Chl_A21_00394" 1034 "s59E13H2" 0 "CoB--CoM heterodisulfide reductase iron-sulfur subunit D"
"Chl_A21_00394" 1034 "s59E25H2" 0 "CoB--CoM heterodisulfide reductase iron-sulfur subunit D"
"Chl_A21_00394" 1034 "s59E5H2" 0 "CoB--CoM heterodisulfide reductase iron-sulfur subunit D"
"Chl_A21_00394" 1034 "s63E6H2" 0 "CoB--CoM heterodisulfide reductase iron-sulfur subunit D"
"Chl_A21_00395" 1035 "s59E13H2" 0 "Long-chain-fatty-acid--CoA ligase"
"Chl_A21_00395" 1035 "s59E25H2" 0 "Long-chain-fatty-acid--CoA ligase"
"Chl_A21_00395" 1035 "s59E5H2" 0 "Long-chain-fatty-acid--CoA ligase"
"Chl_A21_00395" 1035 "s63E6H2" 0 "Long-chain-fatty-acid--CoA ligase"
"Chl_A21_00396" 1036 "s59E13H2" 0 "3-hydroxypropionyl-coenzyme A dehydratase"
"Chl_A21_00396" 1036 "s59E25H2" 0 "3-hydroxypropionyl-coenzyme A dehydratase"
"Chl_A21_00396" 1036 "s59E5H2" 0 "3-hydroxypropionyl-coenzyme A dehydratase"
"Chl_A21_00396" 1036 "s63E6H2" 0 "3-hydroxypropionyl-coenzyme A dehydratase"
"Chl_A21_00397" 1037 "s59E13H2" 0 "3-hydroxybutyryl-CoA dehydrogenase"
"Chl_A21_00397" 1037 "s59E25H2" 0 "3-hydroxybutyryl-CoA dehydrogenase"
"Chl_A21_00397" 1037 "s59E5H2" 0 "3-hydroxybutyryl-CoA dehydrogenase"
"Chl_A21_00397" 1037 "s63E6H2" 0 "3-hydroxybutyryl-CoA dehydrogenase"
"Chl_A21_00398" 1038 "s59E13H2" 0 "1,4-Dihydroxy-2-naphthoyl-CoA synthase"
"Chl_A21_00398" 1038 "s59E25H2" 0 "1,4-Dihydroxy-2-naphthoyl-CoA synthase"
"Chl_A21_00398" 1038 "s59E5H2" 0 "1,4-Dihydroxy-2-naphthoyl-CoA synthase"

"Chl_A21_00398" 1038 "s63E6H2" 0 "1,4-Dihydroxy-2-naphthoyl-CoA synthase"
"Chl_A21_00399" 1039 "s59E13H2" 0 "Galactitol-1-phosphate 5-dehydrogenase"
"Chl_A21_00399" 1039 "s59E25H2" 0 "Galactitol-1-phosphate 5-dehydrogenase"
"Chl_A21_00399" 1039 "s59E5H2" 0 "Galactitol-1-phosphate 5-dehydrogenase"
"Chl_A21_00399" 1039 "s63E6H2" 0 "Galactitol-1-phosphate 5-dehydrogenase"
"Chl_A21_00400" 1040 "s59E13H2" 0 "hypothetical protein"
"Chl_A21_00400" 1040 "s59E25H2" 0 "hypothetical protein"
"Chl_A21_00400" 1040 "s59E5H2" 0 "hypothetical protein"
"Chl_A21_00400" 1040 "s63E6H2" 0 "hypothetical protein"
"Chl_A21_00401" 1041 "s59E13H2" 0 "CRISPR-associated endonuclease Cas9"
"Chl_A21_00401" 1041 "s59E25H2" 0 "CRISPR-associated endonuclease Cas9"
"Chl_A21_00401" 1041 "s59E5H2" 0 "CRISPR-associated endonuclease Cas9"
"Chl_A21_00401" 1041 "s63E6H2" 0 "CRISPR-associated endonuclease Cas9"
"Chl_A21_00402" 1042 "s59E13H2" 0 "putative tyrosine recombinase XerC-like protein"
"Chl_A21_00402" 1042 "s59E25H2" 0 "putative tyrosine recombinase XerC-like protein"
"Chl_A21_00402" 1042 "s59E5H2" 0 "putative tyrosine recombinase XerC-like protein"
"Chl_A21_00402" 1042 "s63E6H2" 0 "putative tyrosine recombinase XerC-like protein"
"Chl_A21_00403" 1043 "s59E13H2" 0 "Tyrosine recombinase XerD"
"Chl_A21_00403" 1043 "s59E25H2" 0 "Tyrosine recombinase XerD"
"Chl_A21_00403" 1043 "s59E5H2" 0 "Tyrosine recombinase XerD"
"Chl_A21_00403" 1043 "s63E6H2" 0 "Tyrosine recombinase XerD"
"Chl_A21_00404" 1044 "s59E13H2" 0 "Hint module"
"Chl_A21_00404" 1044 "s59E25H2" 0 "Hint module"
"Chl_A21_00404" 1044 "s59E5H2" 0 "Hint module"
"Chl_A21_00404" 1044 "s63E6H2" 0 "Hint module"
"Chl_A21_00406" 1046 "s59E13H2" 0 "type I restriction enzyme EcoKI subunit R"
"Chl_A21_00406" 1046 "s59E25H2" 0 "type I restriction enzyme EcoKI subunit R"
"Chl_A21_00406" 1046 "s59E5H2" 0 "type I restriction enzyme EcoKI subunit R"
"Chl_A21_00406" 1046 "s63E6H2" 0 "type I restriction enzyme EcoKI subunit R"
"Chl_A21_00408" 1048 "s59E13H2" 0 "DNA double-strand break repair Rad50 ATPase"
"Chl_A21_00408" 1048 "s59E25H2" 0 "DNA double-strand break repair Rad50 ATPase"
"Chl_A21_00408" 1048 "s59E5H2" 0 "DNA double-strand break repair Rad50 ATPase"
"Chl_A21_00408" 1048 "s63E6H2" 0 "DNA double-strand break repair Rad50 ATPase"
"Chl_A21_00410" 1050 "s59E13H2" 0 "B12 binding domain protein"
"Chl_A21_00410" 1050 "s59E25H2" 0 "B12 binding domain protein"
"Chl_A21_00410" 1050 "s59E5H2" 0 "B12 binding domain protein"
"Chl_A21_00410" 1050 "s63E6H2" 0 "B12 binding domain protein"
"Chl_A21_00411" 1051 "s59E13H2" 0 "hypothetical protein"
"Chl_A21_00411" 1051 "s59E25H2" 0 "hypothetical protein"
"Chl_A21_00411" 1051 "s59E5H2" 0 "hypothetical protein"
"Chl_A21_00411" 1051 "s63E6H2" 0 "hypothetical protein"
"Chl_A21_00412" 1052 "s59E13H2" 0 "putative FAD-linked oxidoreductase"
"Chl_A21_00412" 1052 "s59E25H2" 0 "putative FAD-linked oxidoreductase"
"Chl_A21_00412" 1052 "s59E5H2" 0 "putative FAD-linked oxidoreductase"
"Chl_A21_00412" 1052 "s63E6H2" 0 "putative FAD-linked oxidoreductase"
"Chl_A21_00413" 1053 "s59E13H2" 0 "glycolate oxidase subunit GlcD"
"Chl_A21_00413" 1053 "s59E25H2" 0 "glycolate oxidase subunit GlcD"

"Chl_A21_00413" 1053 "s59E5H2" 0 "glycolate oxidase subunit GlcD"
"Chl_A21_00413" 1053 "s63E6H2" 0 "glycolate oxidase subunit GlcD"
"Chl_A21_00414" 1054 "s59E13H2" 0 "putative FAD-linked oxidoreductase"
"Chl_A21_00414" 1054 "s59E25H2" 0 "putative FAD-linked oxidoreductase"
"Chl_A21_00414" 1054 "s59E5H2" 0 "putative FAD-linked oxidoreductase"
"Chl_A21_00414" 1054 "s63E6H2" 0 "putative FAD-linked oxidoreductase"
"Chl_A21_00415" 1055 "s59E13H2" 0 "hypothetical protein"
"Chl_A21_00415" 1055 "s59E25H2" 0 "hypothetical protein"
"Chl_A21_00415" 1055 "s59E5H2" 0 "hypothetical protein"
"Chl_A21_00415" 1055 "s63E6H2" 0 "hypothetical protein"
"Chl_A21_00416" 1056 "s59E13H2" 0 "hypothetical protein"
"Chl_A21_00416" 1056 "s59E25H2" 0 "hypothetical protein"
"Chl_A21_00416" 1056 "s59E5H2" 0 "hypothetical protein"
"Chl_A21_00416" 1056 "s63E6H2" 0 "hypothetical protein"
"Chl_A21_00417" 1057 "s59E13H2" 0 "5-methyltetrahydrofolate:corrinoid/iron-sulfur protein co-methyltransferase"
"Chl_A21_00417" 1057 "s59E25H2" 0 "5-methyltetrahydrofolate:corrinoid/iron-sulfur protein co-methyltransferase"
"Chl_A21_00417" 1057 "s59E5H2" 0 "5-methyltetrahydrofolate:corrinoid/iron-sulfur protein co-methyltransferase"
"Chl_A21_00417" 1057 "s63E6H2" 0 "5-methyltetrahydrofolate:corrinoid/iron-sulfur protein co-methyltransferase"
"Chl_A21_00418" 1058 "s59E13H2" 0 "Alkaline phosphatase synthesis transcriptional regulatory protein PhoP"
"Chl_A21_00418" 1058 "s59E25H2" 0 "Alkaline phosphatase synthesis transcriptional regulatory protein PhoP"
"Chl_A21_00418" 1058 "s59E5H2" 0 "Alkaline phosphatase synthesis transcriptional regulatory protein PhoP"
"Chl_A21_00418" 1058 "s63E6H2" 0 "Alkaline phosphatase synthesis transcriptional regulatory protein PhoP"
"Chl_A21_00419" 1059 "s59E13H2" 0 "hypothetical protein"
"Chl_A21_00419" 1059 "s59E25H2" 0 "hypothetical protein"
"Chl_A21_00419" 1059 "s59E5H2" 0 "hypothetical protein"
"Chl_A21_00419" 1059 "s63E6H2" 0 "hypothetical protein"
"Chl_A21_00421" 1061 "s59E13H2" 0 "hypothetical protein"
"Chl_A21_00421" 1061 "s59E25H2" 0 "hypothetical protein"
"Chl_A21_00421" 1061 "s59E5H2" 0 "hypothetical protein"
"Chl_A21_00421" 1061 "s63E6H2" 0 "hypothetical protein"
"Chl_A21_00422" 1062 "s59E13H2" 0 "hypothetical protein"
"Chl_A21_00422" 1062 "s59E25H2" 0 "hypothetical protein"
"Chl_A21_00422" 1062 "s59E5H2" 0 "hypothetical protein"
"Chl_A21_00422" 1062 "s63E6H2" 0 "hypothetical protein"
"Chl_A21_00423" 1063 "s59E13H2" 0 "translocation protein TolB"
"Chl_A21_00423" 1063 "s59E25H2" 0 "translocation protein TolB"
"Chl_A21_00423" 1063 "s59E5H2" 0 "translocation protein TolB"
"Chl_A21_00423" 1063 "s63E6H2" 0 "translocation protein TolB"
"Chl_A21_00424" 1064 "s59E13H2" 0 "O-Antigen ligase"
"Chl_A21_00424" 1064 "s59E25H2" 0 "O-Antigen ligase"
"Chl_A21_00424" 1064 "s59E5H2" 0 "O-Antigen ligase"
"Chl_A21_00424" 1064 "s63E6H2" 0 "O-Antigen ligase"
"Chl_A21_00426" 1066 "s59E13H2" 0 "UDP-glucose 4-epimerase"
"Chl_A21_00426" 1066 "s59E25H2" 0 "UDP-glucose 4-epimerase"
"Chl_A21_00426" 1066 "s59E5H2" 0 "UDP-glucose 4-epimerase"
"Chl_A21_00426" 1066 "s63E6H2" 0 "UDP-glucose 4-epimerase"
"Chl_A21_00427" 1067 "s59E13H2" 0 "GDP-L-fucose synthase"

"Chl_A21_00427" 1067 "s59E25H2" 0 "GDP-L-fucose synthase"
"Chl_A21_00427" 1067 "s59E5H2" 0 "GDP-L-fucose synthase"
"Chl_A21_00427" 1067 "s63E6H2" 0 "GDP-L-fucose synthase"
"Chl_A21_00428" 1068 "s59E13H2" 0 "hypothetical protein"
"Chl_A21_00428" 1068 "s59E25H2" 0 "hypothetical protein"
"Chl_A21_00428" 1068 "s59E5H2" 0 "hypothetical protein"
"Chl_A21_00428" 1068 "s63E6H2" 0 "hypothetical protein"
"Chl_A21_00429" 1069 "s59E13H2" 0 "hypothetical protein"
"Chl_A21_00429" 1069 "s59E25H2" 0 "hypothetical protein"
"Chl_A21_00429" 1069 "s59E5H2" 0 "hypothetical protein"
"Chl_A21_00429" 1069 "s63E6H2" 0 "hypothetical protein"
"Chl_A21_00430" 1070 "s59E13H2" 0 "Pyruvate kinase, alpha/beta domain"
"Chl_A21_00430" 1070 "s59E25H2" 0 "Pyruvate kinase, alpha/beta domain"
"Chl_A21_00430" 1070 "s59E5H2" 0 "Pyruvate kinase, alpha/beta domain"
"Chl_A21_00430" 1070 "s63E6H2" 0 "Pyruvate kinase, alpha/beta domain"
"Chl_A21_00432" 1072 "s59E13H2" 0 "hypothetical protein"
"Chl_A21_00432" 1072 "s59E25H2" 0 "hypothetical protein"
"Chl_A21_00432" 1072 "s59E5H2" 0 "hypothetical protein"
"Chl_A21_00432" 1072 "s63E6H2" 0 "hypothetical protein"
"Chl_A21_00433" 1073 "s59E13H2" 0 "hypothetical protein"
"Chl_A21_00433" 1073 "s59E25H2" 0 "hypothetical protein"
"Chl_A21_00433" 1073 "s59E5H2" 0 "hypothetical protein"
"Chl_A21_00433" 1073 "s63E6H2" 0 "hypothetical protein"
"Chl_A21_00434" 1074 "s59E13H2" 0 "hypothetical protein"
"Chl_A21_00434" 1074 "s59E25H2" 0 "hypothetical protein"
"Chl_A21_00434" 1074 "s59E5H2" 0 "hypothetical protein"
"Chl_A21_00434" 1074 "s63E6H2" 0 "hypothetical protein"
"Chl_A21_00437" 1077 "s59E13H2" 0 "hypothetical protein"
"Chl_A21_00437" 1077 "s59E25H2" 0 "hypothetical protein"
"Chl_A21_00437" 1077 "s59E5H2" 0 "hypothetical protein"
"Chl_A21_00437" 1077 "s63E6H2" 0 "hypothetical protein"
"Chl_A21_00440" 1080 "s59E13H2" 0 "hypothetical protein"
"Chl_A21_00440" 1080 "s59E25H2" 0 "hypothetical protein"
"Chl_A21_00440" 1080 "s59E5H2" 0 "hypothetical protein"
"Chl_A21_00440" 1080 "s63E6H2" 0 "hypothetical protein"
"Chl_A21_00446" 1086 "s59E13H2" 0 "CoB--CoM heterodisulfide reductase iron-sulfur subunit A"
"Chl_A21_00446" 1086 "s59E25H2" 0 "CoB--CoM heterodisulfide reductase iron-sulfur subunit A"
"Chl_A21_00446" 1086 "s59E5H2" 0 "CoB--CoM heterodisulfide reductase iron-sulfur subunit A"
"Chl_A21_00446" 1086 "s63E6H2" 0 "CoB--CoM heterodisulfide reductase iron-sulfur subunit A"
"Chl_A21_00447" 1087 "s59E13H2" 0 "acetyl-CoA decarbonylase/synthase complex subunit alpha"
"Chl_A21_00447" 1087 "s59E25H2" 0 "acetyl-CoA decarbonylase/synthase complex subunit alpha"
"Chl_A21_00447" 1087 "s59E5H2" 0 "acetyl-CoA decarbonylase/synthase complex subunit alpha"
"Chl_A21_00447" 1087 "s63E6H2" 0 "acetyl-CoA decarbonylase/synthase complex subunit alpha"
"Chl_A21_00448" 1088 "s59E13H2" 0 "L-aspartate oxidase"
"Chl_A21_00448" 1088 "s59E25H2" 0 "L-aspartate oxidase"
"Chl_A21_00448" 1088 "s59E5H2" 0 "L-aspartate oxidase"
"Chl_A21_00448" 1088 "s63E6H2" 0 "L-aspartate oxidase"

"Chl_A21_00449" 1089 "s59E13H2" 0 "hypothetical protein"
"Chl_A21_00449" 1089 "s59E25H2" 0 "hypothetical protein"
"Chl_A21_00449" 1089 "s59E5H2" 0 "hypothetical protein"
"Chl_A21_00449" 1089 "s63E6H2" 0 "hypothetical protein"
"Chl_A21_00450" 1090 "s59E13H2" 0 "hypothetical protein"
"Chl_A21_00450" 1090 "s59E25H2" 0 "hypothetical protein"
"Chl_A21_00450" 1090 "s59E5H2" 0 "hypothetical protein"
"Chl_A21_00450" 1090 "s63E6H2" 0 "hypothetical protein"
"Chl_A21_00451" 1091 "s59E13H2" 0 "HTH-type transcriptional regulator CymR"
"Chl_A21_00451" 1091 "s59E25H2" 0 "HTH-type transcriptional regulator CymR"
"Chl_A21_00451" 1091 "s59E5H2" 0 "HTH-type transcriptional regulator CymR"
"Chl_A21_00451" 1091 "s63E6H2" 0 "HTH-type transcriptional regulator CymR"
"Chl_A21_00454" 1094 "s59E13H2" 0 "hypothetical protein"
"Chl_A21_00454" 1094 "s59E25H2" 0 "hypothetical protein"
"Chl_A21_00454" 1094 "s59E5H2" 0 "hypothetical protein"
"Chl_A21_00454" 1094 "s63E6H2" 0 "hypothetical protein"
"Chl_A21_00455" 1095 "s59E13H2" 0 "hypothetical protein"
"Chl_A21_00455" 1095 "s59E25H2" 0 "hypothetical protein"
"Chl_A21_00455" 1095 "s59E5H2" 0 "hypothetical protein"
"Chl_A21_00455" 1095 "s63E6H2" 0 "hypothetical protein"
"Chl_A21_00457" 1097 "s59E13H2" 0 "4-amino-4-deoxy-L-arabinose-phosphoundecaprenol flippase subunit ArnE"
"Chl_A21_00457" 1097 "s59E25H2" 0 "4-amino-4-deoxy-L-arabinose-phosphoundecaprenol flippase subunit ArnE"
"Chl_A21_00457" 1097 "s59E5H2" 0 "4-amino-4-deoxy-L-arabinose-phosphoundecaprenol flippase subunit ArnE"
"Chl_A21_00457" 1097 "s63E6H2" 0 "4-amino-4-deoxy-L-arabinose-phosphoundecaprenol flippase subunit ArnE"
"Chl_A21_00460" 1100 "s59E13H2" 0 "Tungsten-containing aldehyde ferredoxin oxidoreductase"
"Chl_A21_00460" 1100 "s59E25H2" 0 "Tungsten-containing aldehyde ferredoxin oxidoreductase"
"Chl_A21_00460" 1100 "s59E5H2" 0 "Tungsten-containing aldehyde ferredoxin oxidoreductase"
"Chl_A21_00460" 1100 "s63E6H2" 0 "Tungsten-containing aldehyde ferredoxin oxidoreductase"
"Chl_A21_00461" 1101 "s59E13H2" 0 "Deoxyribose-phosphate aldolase"
"Chl_A21_00461" 1101 "s59E25H2" 0 "Deoxyribose-phosphate aldolase"
"Chl_A21_00461" 1101 "s59E5H2" 0 "Deoxyribose-phosphate aldolase"
"Chl_A21_00461" 1101 "s63E6H2" 0 "Deoxyribose-phosphate aldolase"
"Chl_A21_00462" 1102 "s59E13H2" 0 "Signal peptidase I T"
"Chl_A21_00462" 1102 "s59E25H2" 0 "Signal peptidase I T"
"Chl_A21_00462" 1102 "s59E5H2" 0 "Signal peptidase I T"
"Chl_A21_00462" 1102 "s63E6H2" 0 "Signal peptidase I T"
"Chl_A21_00463" 1103 "s59E13H2" 0 "hypothetical protein"
"Chl_A21_00463" 1103 "s59E25H2" 0 "hypothetical protein"
"Chl_A21_00463" 1103 "s59E5H2" 0 "hypothetical protein"
"Chl_A21_00463" 1103 "s63E6H2" 0 "hypothetical protein"
"Chl_A21_00464" 1104 "s59E13H2" 0 "Dihydrolipoyl dehydrogenase"
"Chl_A21_00464" 1104 "s59E25H2" 0 "Dihydrolipoyl dehydrogenase"
"Chl_A21_00464" 1104 "s59E5H2" 0 "Dihydrolipoyl dehydrogenase"
"Chl_A21_00464" 1104 "s63E6H2" 0 "Dihydrolipoyl dehydrogenase"
"Chl_A21_00465" 1105 "s59E13H2" 0 "hypothetical protein"
"Chl_A21_00465" 1105 "s59E25H2" 0 "hypothetical protein"
"Chl_A21_00465" 1105 "s59E5H2" 0 "hypothetical protein"

"Chl_A21_00465" 1105 "s63E6H2" 0 "hypothetical protein"
"Chl_A21_00466" 1106 "s59E13H2" 0 "hypothetical protein"
"Chl_A21_00466" 1106 "s59E25H2" 0 "hypothetical protein"
"Chl_A21_00466" 1106 "s59E5H2" 0 "hypothetical protein"
"Chl_A21_00466" 1106 "s63E6H2" 0 "hypothetical protein"
"Chl_A21_00467" 1107 "s59E13H2" 0 "hypothetical protein"
"Chl_A21_00467" 1107 "s59E25H2" 0 "hypothetical protein"
"Chl_A21_00467" 1107 "s59E5H2" 0 "hypothetical protein"
"Chl_A21_00467" 1107 "s63E6H2" 0 "hypothetical protein"
"Chl_A21_00468" 1108 "s59E13H2" 0 "DNA-3-methyladenine glycosylase"
"Chl_A21_00468" 1108 "s59E25H2" 0 "DNA-3-methyladenine glycosylase"
"Chl_A21_00468" 1108 "s59E5H2" 0 "DNA-3-methyladenine glycosylase"
"Chl_A21_00468" 1108 "s63E6H2" 0 "DNA-3-methyladenine glycosylase"
"Chl_A21_00469" 1109 "s59E13H2" 0 "hypothetical protein"
"Chl_A21_00469" 1109 "s59E25H2" 0 "hypothetical protein"
"Chl_A21_00469" 1109 "s59E5H2" 0 "hypothetical protein"
"Chl_A21_00469" 1109 "s63E6H2" 0 "hypothetical protein"
"Chl_A21_00470" 1110 "s59E13H2" 0 "hypothetical protein"
"Chl_A21_00470" 1110 "s59E25H2" 0 "hypothetical protein"
"Chl_A21_00470" 1110 "s59E5H2" 0 "hypothetical protein"
"Chl_A21_00470" 1110 "s63E6H2" 0 "hypothetical protein"
"Chl_A21_00471" 1111 "s59E13H2" 0 "Ferredoxin--NADP reductase"
"Chl_A21_00471" 1111 "s59E25H2" 0 "Ferredoxin--NADP reductase"
"Chl_A21_00471" 1111 "s59E5H2" 0 "Ferredoxin--NADP reductase"
"Chl_A21_00471" 1111 "s63E6H2" 0 "Ferredoxin--NADP reductase"
"Chl_A21_00474" 1114 "s59E13H2" 0 "hypothetical protein"
"Chl_A21_00474" 1114 "s59E25H2" 0 "hypothetical protein"
"Chl_A21_00474" 1114 "s59E5H2" 0 "hypothetical protein"
"Chl_A21_00474" 1114 "s63E6H2" 0 "hypothetical protein"
"Chl_A21_00475" 1115 "s59E13H2" 0 "Arginine-binding extracellular protein ArtP precursor"
"Chl_A21_00475" 1115 "s59E25H2" 0 "Arginine-binding extracellular protein ArtP precursor"
"Chl_A21_00475" 1115 "s59E5H2" 0 "Arginine-binding extracellular protein ArtP precursor"
"Chl_A21_00475" 1115 "s63E6H2" 0 "Arginine-binding extracellular protein ArtP precursor"
"Chl_A21_00476" 1116 "s59E13H2" 0 "Serine dehydrogenase proteinase"
"Chl_A21_00476" 1116 "s59E25H2" 0 "Serine dehydrogenase proteinase"
"Chl_A21_00476" 1116 "s59E5H2" 0 "Serine dehydrogenase proteinase"
"Chl_A21_00476" 1116 "s63E6H2" 0 "Serine dehydrogenase proteinase"
"Chl_A21_00478" 1118 "s59E13H2" 0 "hypothetical protein"
"Chl_A21_00478" 1118 "s59E25H2" 0 "hypothetical protein"
"Chl_A21_00478" 1118 "s59E5H2" 0 "hypothetical protein"
"Chl_A21_00478" 1118 "s63E6H2" 0 "hypothetical protein"
"Chl_A21_00479" 1119 "s59E13H2" 0 "L-asparaginase precursor"
"Chl_A21_00479" 1119 "s59E25H2" 0 "L-asparaginase precursor"
"Chl_A21_00479" 1119 "s59E5H2" 0 "L-asparaginase precursor"
"Chl_A21_00479" 1119 "s63E6H2" 0 "L-asparaginase precursor"
"Chl_A21_00480" 1120 "s59E13H2" 0 "Chromosome partition protein Smc"
"Chl_A21_00480" 1120 "s59E25H2" 0 "Chromosome partition protein Smc"

"Chl_A21_00480" 1120 "s59E5H2" 0 "Chromosome partition protein Smc"
"Chl_A21_00480" 1120 "s63E6H2" 0 "Chromosome partition protein Smc"
"Chl_A21_00481" 1121 "s59E13H2" 0 "Cupin domain protein"
"Chl_A21_00481" 1121 "s59E25H2" 0 "Cupin domain protein"
"Chl_A21_00481" 1121 "s59E5H2" 0 "Cupin domain protein"
"Chl_A21_00481" 1121 "s63E6H2" 0 "Cupin domain protein"
"Chl_A21_00482" 1122 "s59E13H2" 0 "Ribonuclease 3"
"Chl_A21_00482" 1122 "s59E25H2" 0 "Ribonuclease 3"
"Chl_A21_00482" 1122 "s59E5H2" 0 "Ribonuclease 3"
"Chl_A21_00482" 1122 "s63E6H2" 0 "Ribonuclease 3"
"Chl_A21_00483" 1123 "s59E13H2" 0 "3-oxoacyl-[acyl-carrier-protein] synthase 2"
"Chl_A21_00483" 1123 "s59E25H2" 0 "3-oxoacyl-[acyl-carrier-protein] synthase 2"
"Chl_A21_00483" 1123 "s59E5H2" 0 "3-oxoacyl-[acyl-carrier-protein] synthase 2"
"Chl_A21_00483" 1123 "s63E6H2" 0 "3-oxoacyl-[acyl-carrier-protein] synthase 2"
"Chl_A21_00484" 1124 "s59E13H2" 0 "Threonine synthase"
"Chl_A21_00484" 1124 "s59E25H2" 0 "Threonine synthase"
"Chl_A21_00484" 1124 "s59E5H2" 0 "Threonine synthase"
"Chl_A21_00484" 1124 "s63E6H2" 0 "Threonine synthase"
"Chl_A21_00485" 1125 "s59E13H2" 0 "Cyclic pyranopterin monophosphate synthase accessory protein"
"Chl_A21_00485" 1125 "s59E25H2" 0 "Cyclic pyranopterin monophosphate synthase accessory protein"
"Chl_A21_00485" 1125 "s59E5H2" 0 "Cyclic pyranopterin monophosphate synthase accessory protein"
"Chl_A21_00485" 1125 "s63E6H2" 0 "Cyclic pyranopterin monophosphate synthase accessory protein"
"Chl_A21_00486" 1126 "s59E13H2" 0 "hypothetical protein"
"Chl_A21_00486" 1126 "s59E25H2" 0 "hypothetical protein"
"Chl_A21_00486" 1126 "s59E5H2" 0 "hypothetical protein"
"Chl_A21_00486" 1126 "s63E6H2" 0 "hypothetical protein"
"Chl_A21_00487" 1127 "s59E13H2" 0 "hypothetical protein"
"Chl_A21_00487" 1127 "s59E25H2" 0 "hypothetical protein"
"Chl_A21_00487" 1127 "s59E5H2" 0 "hypothetical protein"
"Chl_A21_00487" 1127 "s63E6H2" 0 "hypothetical protein"
"Chl_A21_00488" 1128 "s59E13H2" 0 "Glycosyltransferase AglD"
"Chl_A21_00488" 1128 "s59E25H2" 0 "Glycosyltransferase AglD"
"Chl_A21_00488" 1128 "s59E5H2" 0 "Glycosyltransferase AglD"
"Chl_A21_00488" 1128 "s63E6H2" 0 "Glycosyltransferase AglD"
"Chl_A21_00489" 1129 "s59E13H2" 0 "Signal recognition particle receptor FtsY"
"Chl_A21_00489" 1129 "s59E25H2" 0 "Signal recognition particle receptor FtsY"
"Chl_A21_00489" 1129 "s59E5H2" 0 "Signal recognition particle receptor FtsY"
"Chl_A21_00489" 1129 "s63E6H2" 0 "Signal recognition particle receptor FtsY"
"Chl_A21_00490" 1130 "s59E13H2" 0 "Peptide chain release factor 2"
"Chl_A21_00490" 1130 "s59E25H2" 0 "Peptide chain release factor 2"
"Chl_A21_00490" 1130 "s59E5H2" 0 "Peptide chain release factor 2"
"Chl_A21_00490" 1130 "s63E6H2" 0 "Peptide chain release factor 2"
"Chl_A21_00491" 1131 "s59E13H2" 0 "8-oxoguanine deaminase"
"Chl_A21_00491" 1131 "s59E25H2" 0 "8-oxoguanine deaminase"
"Chl_A21_00491" 1131 "s59E5H2" 0 "8-oxoguanine deaminase"
"Chl_A21_00491" 1131 "s63E6H2" 0 "8-oxoguanine deaminase"
"Chl_A21_00492" 1132 "s59E13H2" 0 "Secreted effector protein pipB2"

"Chl_A21_00492" 1132 "s59E25H2" 0 "Secreted effector protein pipB2"
"Chl_A21_00492" 1132 "s59E5H2" 0 "Secreted effector protein pipB2"
"Chl_A21_00492" 1132 "s63E6H2" 0 "Secreted effector protein pipB2"
"Chl_A21_00494" 1134 "s59E13H2" 0 "Major Facilitator Superfamily protein"
"Chl_A21_00494" 1134 "s59E25H2" 0 "Major Facilitator Superfamily protein"
"Chl_A21_00494" 1134 "s59E5H2" 0 "Major Facilitator Superfamily protein"
"Chl_A21_00494" 1134 "s63E6H2" 0 "Major Facilitator Superfamily protein"
"Chl_A21_00495" 1135 "s59E13H2" 0 "hypothetical protein"
"Chl_A21_00495" 1135 "s59E25H2" 0 "hypothetical protein"
"Chl_A21_00495" 1135 "s59E5H2" 0 "hypothetical protein"
"Chl_A21_00495" 1135 "s63E6H2" 0 "hypothetical protein"
"Chl_A21_00496" 1136 "s59E13H2" 0 "Oxygen-independent coproporphyrinogen-III oxidase 1"
"Chl_A21_00496" 1136 "s59E25H2" 0 "Oxygen-independent coproporphyrinogen-III oxidase 1"
"Chl_A21_00496" 1136 "s59E5H2" 0 "Oxygen-independent coproporphyrinogen-III oxidase 1"
"Chl_A21_00496" 1136 "s63E6H2" 0 "Oxygen-independent coproporphyrinogen-III oxidase 1"
"Chl_A21_00497" 1137 "s59E13H2" 0 "(2E,6E)-farnesyl diphosphate synthase"
"Chl_A21_00497" 1137 "s59E25H2" 0 "(2E,6E)-farnesyl diphosphate synthase"
"Chl_A21_00497" 1137 "s59E5H2" 0 "(2E,6E)-farnesyl diphosphate synthase"
"Chl_A21_00497" 1137 "s63E6H2" 0 "(2E,6E)-farnesyl diphosphate synthase"
"Chl_A21_00498" 1138 "s59E13H2" 0 "Isopentenyl-diphosphate delta-isomerase"
"Chl_A21_00498" 1138 "s59E25H2" 0 "Isopentenyl-diphosphate delta-isomerase"
"Chl_A21_00498" 1138 "s59E5H2" 0 "Isopentenyl-diphosphate delta-isomerase"
"Chl_A21_00498" 1138 "s63E6H2" 0 "Isopentenyl-diphosphate delta-isomerase"
"Chl_A21_00499" 1139 "s59E13H2" 0 "Acyl carrier protein"
"Chl_A21_00499" 1139 "s59E25H2" 0 "Acyl carrier protein"
"Chl_A21_00499" 1139 "s59E5H2" 0 "Acyl carrier protein"
"Chl_A21_00499" 1139 "s63E6H2" 0 "Acyl carrier protein"
"Chl_A21_00500" 1140 "s59E13H2" 0 "putative molybdenum cofactor guanylyltransferase"
"Chl_A21_00500" 1140 "s59E25H2" 0 "putative molybdenum cofactor guanylyltransferase"
"Chl_A21_00500" 1140 "s59E5H2" 0 "putative molybdenum cofactor guanylyltransferase"
"Chl_A21_00500" 1140 "s63E6H2" 0 "putative molybdenum cofactor guanylyltransferase"
"Chl_A21_00501" 1141 "s59E13H2" 0 "6-hydroxypseudooxynicotine dehydrogenase complex subunit alpha"
"Chl_A21_00501" 1141 "s59E25H2" 0 "6-hydroxypseudooxynicotine dehydrogenase complex subunit alpha"
"Chl_A21_00501" 1141 "s59E5H2" 0 "6-hydroxypseudooxynicotine dehydrogenase complex subunit alpha"
"Chl_A21_00501" 1141 "s63E6H2" 0 "6-hydroxypseudooxynicotine dehydrogenase complex subunit alpha"
"Chl_A21_00502" 1142 "s59E13H2" 0 "Nicotinate dehydrogenase small FeS subunit"
"Chl_A21_00502" 1142 "s59E25H2" 0 "Nicotinate dehydrogenase small FeS subunit"
"Chl_A21_00502" 1142 "s59E5H2" 0 "Nicotinate dehydrogenase small FeS subunit"
"Chl_A21_00502" 1142 "s63E6H2" 0 "Nicotinate dehydrogenase small FeS subunit"
"Chl_A21_00503" 1143 "s59E13H2" 0 "putative xanthine dehydrogenase subunit D"
"Chl_A21_00503" 1143 "s59E25H2" 0 "putative xanthine dehydrogenase subunit D"
"Chl_A21_00503" 1143 "s59E5H2" 0 "putative xanthine dehydrogenase subunit D"
"Chl_A21_00503" 1143 "s63E6H2" 0 "putative xanthine dehydrogenase subunit D"
"Chl_A21_00504" 1144 "s59E13H2" 0 "HEAT repeat protein"
"Chl_A21_00504" 1144 "s59E25H2" 0 "HEAT repeat protein"
"Chl_A21_00504" 1144 "s59E5H2" 0 "HEAT repeat protein"
"Chl_A21_00504" 1144 "s63E6H2" 0 "HEAT repeat protein"

"Chl_A21_00505" 1145 "s59E13H2" 0 "putative xanthine dehydrogenase subunit D"
"Chl_A21_00505" 1145 "s59E25H2" 0 "putative xanthine dehydrogenase subunit D"
"Chl_A21_00505" 1145 "s59E5H2" 0 "putative xanthine dehydrogenase subunit D"
"Chl_A21_00505" 1145 "s63E6H2" 0 "putative xanthine dehydrogenase subunit D"
"Chl_A21_00506" 1146 "s59E13H2" 0 "translocation protein TolB"
"Chl_A21_00506" 1146 "s59E25H2" 0 "translocation protein TolB"
"Chl_A21_00506" 1146 "s59E5H2" 0 "translocation protein TolB"
"Chl_A21_00506" 1146 "s63E6H2" 0 "translocation protein TolB"
"Chl_A21_00508" 1148 "s59E13H2" 0 "Molybdopterin molybdenumtransferase"
"Chl_A21_00508" 1148 "s59E25H2" 0 "Molybdopterin molybdenumtransferase"
"Chl_A21_00508" 1148 "s59E5H2" 0 "Molybdopterin molybdenumtransferase"
"Chl_A21_00508" 1148 "s63E6H2" 0 "Molybdopterin molybdenumtransferase"
"Chl_A21_00509" 1149 "s59E13H2" 0 "Molybdopterin-guanine dinucleotide biosynthesis adapter protein"
"Chl_A21_00509" 1149 "s59E25H2" 0 "Molybdopterin-guanine dinucleotide biosynthesis adapter protein"
"Chl_A21_00509" 1149 "s59E5H2" 0 "Molybdopterin-guanine dinucleotide biosynthesis adapter protein"
"Chl_A21_00509" 1149 "s63E6H2" 0 "Molybdopterin-guanine dinucleotide biosynthesis adapter protein"
"Chl_A21_00510" 1150 "s59E13H2" 0 "2-oxoglutarate synthase subunit KorB"
"Chl_A21_00510" 1150 "s59E25H2" 0 "2-oxoglutarate synthase subunit KorB"
"Chl_A21_00510" 1150 "s59E5H2" 0 "2-oxoglutarate synthase subunit KorB"
"Chl_A21_00510" 1150 "s63E6H2" 0 "2-oxoglutarate synthase subunit KorB"
"Chl_A21_00511" 1151 "s59E13H2" 0 "S-methyl-5'-thioadenosine phosphorylase"
"Chl_A21_00511" 1151 "s59E25H2" 0 "S-methyl-5'-thioadenosine phosphorylase"
"Chl_A21_00511" 1151 "s59E5H2" 0 "S-methyl-5'-thioadenosine phosphorylase"
"Chl_A21_00511" 1151 "s63E6H2" 0 "S-methyl-5'-thioadenosine phosphorylase"
"Chl_A21_00512" 1152 "s59E13H2" 0 "hypothetical protein"
"Chl_A21_00512" 1152 "s59E25H2" 0 "hypothetical protein"
"Chl_A21_00512" 1152 "s59E5H2" 0 "hypothetical protein"
"Chl_A21_00512" 1152 "s63E6H2" 0 "hypothetical protein"
"Chl_A21_00513" 1153 "s59E13H2" 0 "SsrA-binding protein"
"Chl_A21_00513" 1153 "s59E25H2" 0 "SsrA-binding protein"
"Chl_A21_00513" 1153 "s59E5H2" 0 "SsrA-binding protein"
"Chl_A21_00513" 1153 "s63E6H2" 0 "SsrA-binding protein"
"Chl_A21_00514" 1154 "s59E13H2" 0 "putative CtpA-like serine protease"
"Chl_A21_00514" 1154 "s59E25H2" 0 "putative CtpA-like serine protease"
"Chl_A21_00514" 1154 "s59E5H2" 0 "putative CtpA-like serine protease"
"Chl_A21_00514" 1154 "s63E6H2" 0 "putative CtpA-like serine protease"
"Chl_A21_00515" 1155 "s59E13H2" 0 "putative CtpA-like serine protease"
"Chl_A21_00515" 1155 "s59E25H2" 0 "putative CtpA-like serine protease"
"Chl_A21_00515" 1155 "s59E5H2" 0 "putative CtpA-like serine protease"
"Chl_A21_00515" 1155 "s63E6H2" 0 "putative CtpA-like serine protease"
"Chl_A21_00516" 1156 "s59E13H2" 0 "GTPase HflX"
"Chl_A21_00516" 1156 "s59E25H2" 0 "GTPase HflX"
"Chl_A21_00516" 1156 "s59E5H2" 0 "GTPase HflX"
"Chl_A21_00516" 1156 "s63E6H2" 0 "GTPase HflX"
"Chl_A21_00517" 1157 "s59E13H2" 0 "Methylenetetrahydrofolate--tRNA-(uracil-5-)-methyltransferase TrmFO"
"Chl_A21_00517" 1157 "s59E25H2" 0 "Methylenetetrahydrofolate--tRNA-(uracil-5-)-methyltransferase TrmFO"
"Chl_A21_00517" 1157 "s59E5H2" 0 "Methylenetetrahydrofolate--tRNA-(uracil-5-)-methyltransferase TrmFO"

"Chl_A21_00517" 1157 "s63E6H2" 0 "Methylenetetrahydrofolate--tRNA-(uracil-5-)-methyltransferase TrmFO"
"Chl_A21_00518" 1158 "s59E13H2" 0 "Erythromycin 3''-O-methyltransferase"
"Chl_A21_00518" 1158 "s59E25H2" 0 "Erythromycin 3''-O-methyltransferase"
"Chl_A21_00518" 1158 "s59E5H2" 0 "Erythromycin 3''-O-methyltransferase"
"Chl_A21_00518" 1158 "s63E6H2" 0 "Erythromycin 3''-O-methyltransferase"
"Chl_A21_00520" 1160 "s59E13H2" 0 "Transcriptional repressor SdpR"
"Chl_A21_00520" 1160 "s59E25H2" 0 "Transcriptional repressor SdpR"
"Chl_A21_00520" 1160 "s59E5H2" 0 "Transcriptional repressor SdpR"
"Chl_A21_00520" 1160 "s63E6H2" 0 "Transcriptional repressor SdpR"
"Chl_A21_00521" 1161 "s59E13H2" 0 "hypothetical protein"
"Chl_A21_00521" 1161 "s59E25H2" 0 "hypothetical protein"
"Chl_A21_00521" 1161 "s59E5H2" 0 "hypothetical protein"
"Chl_A21_00521" 1161 "s63E6H2" 0 "hypothetical protein"
"Chl_A21_00522" 1162 "s59E13H2" 0 "Pyrimidine-nucleoside phosphorylase"
"Chl_A21_00522" 1162 "s59E25H2" 0 "Pyrimidine-nucleoside phosphorylase"
"Chl_A21_00522" 1162 "s59E5H2" 0 "Pyrimidine-nucleoside phosphorylase"
"Chl_A21_00522" 1162 "s63E6H2" 0 "Pyrimidine-nucleoside phosphorylase"
"Chl_A21_00523" 1163 "s59E13H2" 0 "Serine/threonine-protein kinase AfsK"
"Chl_A21_00523" 1163 "s59E25H2" 0 "Serine/threonine-protein kinase AfsK"
"Chl_A21_00523" 1163 "s59E5H2" 0 "Serine/threonine-protein kinase AfsK"
"Chl_A21_00523" 1163 "s63E6H2" 0 "Serine/threonine-protein kinase AfsK"
"Chl_A21_00524" 1164 "s59E13H2" 0 "Soluble lytic murein transglycosylase precursor"
"Chl_A21_00524" 1164 "s59E25H2" 0 "Soluble lytic murein transglycosylase precursor"
"Chl_A21_00524" 1164 "s59E5H2" 0 "Soluble lytic murein transglycosylase precursor"
"Chl_A21_00524" 1164 "s63E6H2" 0 "Soluble lytic murein transglycosylase precursor"
"Chl_A21_00525" 1165 "s59E13H2" 0 "hypothetical protein"
"Chl_A21_00525" 1165 "s59E25H2" 0 "hypothetical protein"
"Chl_A21_00525" 1165 "s59E5H2" 0 "hypothetical protein"
"Chl_A21_00525" 1165 "s63E6H2" 0 "hypothetical protein"
"Chl_A21_00526" 1166 "s59E13H2" 0 "CDP-alcohol phosphatidyltransferase"
"Chl_A21_00526" 1166 "s59E25H2" 0 "CDP-alcohol phosphatidyltransferase"
"Chl_A21_00526" 1166 "s59E5H2" 0 "CDP-alcohol phosphatidyltransferase"
"Chl_A21_00526" 1166 "s63E6H2" 0 "CDP-alcohol phosphatidyltransferase"
"Chl_A21_00527" 1167 "s59E13H2" 0 "hypothetical protein"
"Chl_A21_00527" 1167 "s59E25H2" 0 "hypothetical protein"
"Chl_A21_00527" 1167 "s59E5H2" 0 "hypothetical protein"
"Chl_A21_00527" 1167 "s63E6H2" 0 "hypothetical protein"
"Chl_A21_00528" 1168 "s59E13H2" 0 "L-arabinose transport system permease protein AraQ"
"Chl_A21_00528" 1168 "s59E25H2" 0 "L-arabinose transport system permease protein AraQ"
"Chl_A21_00528" 1168 "s59E5H2" 0 "L-arabinose transport system permease protein AraQ"
"Chl_A21_00528" 1168 "s63E6H2" 0 "L-arabinose transport system permease protein AraQ"
"Chl_A21_00529" 1169 "s59E13H2" 0 "Lactose transport system permease protein LacF"
"Chl_A21_00529" 1169 "s59E25H2" 0 "Lactose transport system permease protein LacF"
"Chl_A21_00529" 1169 "s59E5H2" 0 "Lactose transport system permease protein LacF"
"Chl_A21_00529" 1169 "s63E6H2" 0 "Lactose transport system permease protein LacF"
"Chl_A21_00530" 1170 "s59E13H2" 0 "Bacterial extracellular solute-binding protein"
"Chl_A21_00530" 1170 "s59E25H2" 0 "Bacterial extracellular solute-binding protein"

"Chl_A21_00530" 1170 "s59E5H2" 0 "Bacterial extracellular solute-binding protein"
"Chl_A21_00530" 1170 "s63E6H2" 0 "Bacterial extracellular solute-binding protein"
"Chl_A21_00532" 1172 "s59E13H2" 0 "Xaa-Pro dipeptidase"
"Chl_A21_00532" 1172 "s59E25H2" 0 "Xaa-Pro dipeptidase"
"Chl_A21_00532" 1172 "s59E5H2" 0 "Xaa-Pro dipeptidase"
"Chl_A21_00532" 1172 "s63E6H2" 0 "Xaa-Pro dipeptidase"
"Chl_A21_00534" 1174 "s59E13H2" 0 "hypothetical protein"
"Chl_A21_00534" 1174 "s59E25H2" 0 "hypothetical protein"
"Chl_A21_00534" 1174 "s59E5H2" 0 "hypothetical protein"
"Chl_A21_00534" 1174 "s63E6H2" 0 "hypothetical protein"
"Chl_A21_00535" 1175 "s59E13H2" 0 "Alkaline phosphatase synthesis transcriptional regulatory protein PhoP"
"Chl_A21_00535" 1175 "s59E25H2" 0 "Alkaline phosphatase synthesis transcriptional regulatory protein PhoP"
"Chl_A21_00535" 1175 "s59E5H2" 0 "Alkaline phosphatase synthesis transcriptional regulatory protein PhoP"
"Chl_A21_00535" 1175 "s63E6H2" 0 "Alkaline phosphatase synthesis transcriptional regulatory protein PhoP"
"Chl_A21_00536" 1176 "s59E13H2" 0 "Phytochrome-like protein cph2"
"Chl_A21_00536" 1176 "s59E25H2" 0 "Phytochrome-like protein cph2"
"Chl_A21_00536" 1176 "s59E5H2" 0 "Phytochrome-like protein cph2"
"Chl_A21_00536" 1176 "s63E6H2" 0 "Phytochrome-like protein cph2"
"Chl_A21_00537" 1177 "s59E13H2" 0 "hypothetical protein"
"Chl_A21_00537" 1177 "s59E25H2" 0 "hypothetical protein"
"Chl_A21_00537" 1177 "s59E5H2" 0 "hypothetical protein"
"Chl_A21_00537" 1177 "s63E6H2" 0 "hypothetical protein"
"Chl_A21_00538" 1178 "s59E13H2" 0 "Tyrosine-protein kinase YwqD"
"Chl_A21_00538" 1178 "s59E25H2" 0 "Tyrosine-protein kinase YwqD"
"Chl_A21_00538" 1178 "s59E5H2" 0 "Tyrosine-protein kinase YwqD"
"Chl_A21_00538" 1178 "s63E6H2" 0 "Tyrosine-protein kinase YwqD"
"Chl_A21_00539" 1179 "s59E13H2" 0 "UDP-N-acetylgalactosamine-undecaprenyl-phosphate N-acetylgalactosaminephosphotransferase"
"Chl_A21_00539" 1179 "s59E25H2" 0 "UDP-N-acetylgalactosamine-undecaprenyl-phosphate N-acetylgalactosaminephosphotransferase"
"Chl_A21_00539" 1179 "s59E5H2" 0 "UDP-N-acetylgalactosamine-undecaprenyl-phosphate N-acetylgalactosaminephosphotransferase"
"Chl_A21_00539" 1179 "s63E6H2" 0 "UDP-N-acetylgalactosamine-undecaprenyl-phosphate N-acetylgalactosaminephosphotransferase"
"Chl_A21_00540" 1180 "s59E13H2" 0 "putative oxidoreductase YdgJ"
"Chl_A21_00540" 1180 "s59E25H2" 0 "putative oxidoreductase YdgJ"
"Chl_A21_00540" 1180 "s59E5H2" 0 "putative oxidoreductase YdgJ"
"Chl_A21_00540" 1180 "s63E6H2" 0 "putative oxidoreductase YdgJ"
"Chl_A21_00541" 1181 "s59E13H2" 0 "dTDP-3-amino-3,6-dideoxy-alpha-D-galactopyranose 3-N-acetyltransferase"
"Chl_A21_00541" 1181 "s59E25H2" 0 "dTDP-3-amino-3,6-dideoxy-alpha-D-galactopyranose 3-N-acetyltransferase"
"Chl_A21_00541" 1181 "s59E5H2" 0 "dTDP-3-amino-3,6-dideoxy-alpha-D-galactopyranose 3-N-acetyltransferase"
"Chl_A21_00541" 1181 "s63E6H2" 0 "dTDP-3-amino-3,6-dideoxy-alpha-D-galactopyranose 3-N-acetyltransferase"
"Chl_A21_00542" 1182 "s59E13H2" 0 "dTDP-3-amino-3,6-dideoxy-alpha-D-galactopyranose transaminase"
"Chl_A21_00542" 1182 "s59E25H2" 0 "dTDP-3-amino-3,6-dideoxy-alpha-D-galactopyranose transaminase"
"Chl_A21_00542" 1182 "s59E5H2" 0 "dTDP-3-amino-3,6-dideoxy-alpha-D-galactopyranose transaminase"
"Chl_A21_00542" 1182 "s63E6H2" 0 "dTDP-3-amino-3,6-dideoxy-alpha-D-galactopyranose transaminase"
"Chl_A21_00543" 1183 "s59E13H2" 0 "hypothetical protein"
"Chl_A21_00543" 1183 "s59E25H2" 0 "hypothetical protein"
"Chl_A21_00543" 1183 "s59E5H2" 0 "hypothetical protein"
"Chl_A21_00543" 1183 "s63E6H2" 0 "hypothetical protein"
"Chl_A21_00544" 1184 "s59E13H2" 0 "hypothetical protein"

"Chl_A21_00544" 1184 "s59E25H2" 0 "hypothetical protein"
"Chl_A21_00544" 1184 "s59E5H2" 0 "hypothetical protein"
"Chl_A21_00544" 1184 "s63E6H2" 0 "hypothetical protein"
"Chl_A21_00545" 1185 "s59E13H2" 0 "hypothetical protein"
"Chl_A21_00545" 1185 "s59E25H2" 0 "hypothetical protein"
"Chl_A21_00545" 1185 "s59E5H2" 0 "hypothetical protein"
"Chl_A21_00545" 1185 "s63E6H2" 0 "hypothetical protein"
"Chl_A21_00546" 1186 "s59E13H2" 0 "hypothetical protein"
"Chl_A21_00546" 1186 "s59E25H2" 0 "hypothetical protein"
"Chl_A21_00546" 1186 "s59E5H2" 0 "hypothetical protein"
"Chl_A21_00546" 1186 "s63E6H2" 0 "hypothetical protein"
"Chl_A21_00547" 1187 "s59E13H2" 0 "Ribose operon repressor"
"Chl_A21_00547" 1187 "s59E25H2" 0 "Ribose operon repressor"
"Chl_A21_00547" 1187 "s59E5H2" 0 "Ribose operon repressor"
"Chl_A21_00547" 1187 "s63E6H2" 0 "Ribose operon repressor"
"Chl_A21_00548" 1188 "s59E13H2" 0 "Bacterial extracellular solute-binding protein"
"Chl_A21_00548" 1188 "s59E25H2" 0 "Bacterial extracellular solute-binding protein"
"Chl_A21_00548" 1188 "s59E5H2" 0 "Bacterial extracellular solute-binding protein"
"Chl_A21_00548" 1188 "s63E6H2" 0 "Bacterial extracellular solute-binding protein"
"Chl_A21_00549" 1189 "s59E13H2" 0 "L-arabinose transport system permease protein AraP"
"Chl_A21_00549" 1189 "s59E25H2" 0 "L-arabinose transport system permease protein AraP"
"Chl_A21_00549" 1189 "s59E5H2" 0 "L-arabinose transport system permease protein AraP"
"Chl_A21_00549" 1189 "s63E6H2" 0 "L-arabinose transport system permease protein AraP"
"Chl_A21_00550" 1190 "s59E13H2" 0 "Trehalose/maltose transport system permease protein MalG"
"Chl_A21_00550" 1190 "s59E25H2" 0 "Trehalose/maltose transport system permease protein MalG"
"Chl_A21_00550" 1190 "s59E5H2" 0 "Trehalose/maltose transport system permease protein MalG"
"Chl_A21_00550" 1190 "s63E6H2" 0 "Trehalose/maltose transport system permease protein MalG"
"Chl_A21_00551" 1191 "s59E13H2" 0 "N,N'-diacetylchitobiose phosphorylase"
"Chl_A21_00551" 1191 "s59E25H2" 0 "N,N'-diacetylchitobiose phosphorylase"
"Chl_A21_00551" 1191 "s59E5H2" 0 "N,N'-diacetylchitobiose phosphorylase"
"Chl_A21_00551" 1191 "s63E6H2" 0 "N,N'-diacetylchitobiose phosphorylase"
"Chl_A21_00552" 1192 "s59E13H2" 0 "General stress protein 69"
"Chl_A21_00552" 1192 "s59E25H2" 0 "General stress protein 69"
"Chl_A21_00552" 1192 "s59E5H2" 0 "General stress protein 69"
"Chl_A21_00552" 1192 "s63E6H2" 0 "General stress protein 69"
"Chl_A21_00553" 1193 "s59E13H2" 0 "Beta-xylosidase"
"Chl_A21_00553" 1193 "s59E25H2" 0 "Beta-xylosidase"
"Chl_A21_00553" 1193 "s59E5H2" 0 "Beta-xylosidase"
"Chl_A21_00553" 1193 "s63E6H2" 0 "Beta-xylosidase"
"Chl_A21_00554" 1194 "s59E13H2" 0 "Periplasmic beta-glucosidase precursor"
"Chl_A21_00554" 1194 "s59E25H2" 0 "Periplasmic beta-glucosidase precursor"
"Chl_A21_00554" 1194 "s59E5H2" 0 "Periplasmic beta-glucosidase precursor"
"Chl_A21_00554" 1194 "s63E6H2" 0 "Periplasmic beta-glucosidase precursor"
"Chl_A21_00555" 1195 "s59E13H2" 0 "N,N'-diacetylchitobiose phosphorylase"
"Chl_A21_00555" 1195 "s59E25H2" 0 "N,N'-diacetylchitobiose phosphorylase"
"Chl_A21_00555" 1195 "s59E5H2" 0 "N,N'-diacetylchitobiose phosphorylase"
"Chl_A21_00555" 1195 "s63E6H2" 0 "N,N'-diacetylchitobiose phosphorylase"

"Chl_A21_00556" 1196 "s59E13H2" 0 "Beta-glucosidase A"
"Chl_A21_00556" 1196 "s59E25H2" 0 "Beta-glucosidase A"
"Chl_A21_00556" 1196 "s59E5H2" 0 "Beta-glucosidase A"
"Chl_A21_00556" 1196 "s63E6H2" 0 "Beta-glucosidase A"
"Chl_A21_00557" 1197 "s59E13H2" 0 "DinB superfamily protein"
"Chl_A21_00557" 1197 "s59E25H2" 0 "DinB superfamily protein"
"Chl_A21_00557" 1197 "s59E5H2" 0 "DinB superfamily protein"
"Chl_A21_00557" 1197 "s63E6H2" 0 "DinB superfamily protein"
"Chl_A21_00558" 1198 "s59E13H2" 0 "hypothetical protein"
"Chl_A21_00558" 1198 "s59E25H2" 0 "hypothetical protein"
"Chl_A21_00558" 1198 "s59E5H2" 0 "hypothetical protein"
"Chl_A21_00558" 1198 "s63E6H2" 0 "hypothetical protein"
"Chl_A21_00559" 1199 "s59E13H2" 0 "Acyl-CoA thioester hydrolase YbgC"
"Chl_A21_00559" 1199 "s59E25H2" 0 "Acyl-CoA thioester hydrolase YbgC"
"Chl_A21_00559" 1199 "s59E5H2" 0 "Acyl-CoA thioester hydrolase YbgC"
"Chl_A21_00559" 1199 "s63E6H2" 0 "Acyl-CoA thioester hydrolase YbgC"
"Chl_A21_00560" 1200 "s59E13H2" 0 "Xaa-Pro aminopeptidase"
"Chl_A21_00560" 1200 "s59E25H2" 0 "Xaa-Pro aminopeptidase"
"Chl_A21_00560" 1200 "s59E5H2" 0 "Xaa-Pro aminopeptidase"
"Chl_A21_00560" 1200 "s63E6H2" 0 "Xaa-Pro aminopeptidase"
"Chl_A21_00564" 1204 "s59E13H2" 0 "Long-chain-fatty-acid--CoA ligase"
"Chl_A21_00564" 1204 "s59E25H2" 0 "Long-chain-fatty-acid--CoA ligase"
"Chl_A21_00564" 1204 "s59E5H2" 0 "Long-chain-fatty-acid--CoA ligase"
"Chl_A21_00564" 1204 "s63E6H2" 0 "Long-chain-fatty-acid--CoA ligase"
"Chl_A21_00565" 1205 "s59E13H2" 0 "hypothetical protein"
"Chl_A21_00565" 1205 "s59E25H2" 0 "hypothetical protein"
"Chl_A21_00565" 1205 "s59E5H2" 0 "hypothetical protein"
"Chl_A21_00565" 1205 "s63E6H2" 0 "hypothetical protein"
"Chl_A21_00566" 1206 "s59E13H2" 0 "Transposase DDE domain protein"
"Chl_A21_00566" 1206 "s59E25H2" 0 "Transposase DDE domain protein"
"Chl_A21_00566" 1206 "s59E5H2" 0 "Transposase DDE domain protein"
"Chl_A21_00566" 1206 "s63E6H2" 0 "Transposase DDE domain protein"
"Chl_A21_00567" 1207 "s59E13H2" 0 "Oligopeptide-binding protein OppA precursor"
"Chl_A21_00567" 1207 "s59E25H2" 0 "Oligopeptide-binding protein OppA precursor"
"Chl_A21_00567" 1207 "s59E5H2" 0 "Oligopeptide-binding protein OppA precursor"
"Chl_A21_00567" 1207 "s63E6H2" 0 "Oligopeptide-binding protein OppA precursor"
"Chl_A21_00568" 1208 "s59E13H2" 0 "Glutathione transport system permease protein GsiC"
"Chl_A21_00568" 1208 "s59E25H2" 0 "Glutathione transport system permease protein GsiC"
"Chl_A21_00568" 1208 "s59E5H2" 0 "Glutathione transport system permease protein GsiC"
"Chl_A21_00568" 1208 "s63E6H2" 0 "Glutathione transport system permease protein GsiC"
"Chl_A21_00569" 1209 "s59E13H2" 0 "Glutathione transport system permease protein GsiD"
"Chl_A21_00569" 1209 "s59E25H2" 0 "Glutathione transport system permease protein GsiD"
"Chl_A21_00569" 1209 "s59E5H2" 0 "Glutathione transport system permease protein GsiD"
"Chl_A21_00569" 1209 "s63E6H2" 0 "Glutathione transport system permease protein GsiD"
"Chl_A21_00570" 1210 "s59E13H2" 0 "hypothetical protein"
"Chl_A21_00570" 1210 "s59E25H2" 0 "hypothetical protein"
"Chl_A21_00570" 1210 "s59E5H2" 0 "hypothetical protein"

"Chl_A21_00570" 1210 "s63E6H2" 0 "hypothetical protein"
"Chl_A21_00571" 1211 "s59E13H2" 0 "Carboxy-terminal processing protease CtpB precursor"
"Chl_A21_00571" 1211 "s59E25H2" 0 "Carboxy-terminal processing protease CtpB precursor"
"Chl_A21_00571" 1211 "s59E5H2" 0 "Carboxy-terminal processing protease CtpB precursor"
"Chl_A21_00571" 1211 "s63E6H2" 0 "Carboxy-terminal processing protease CtpB precursor"
"Chl_A21_00572" 1212 "s59E13H2" 0 "hypothetical protein"
"Chl_A21_00572" 1212 "s59E25H2" 0 "hypothetical protein"
"Chl_A21_00572" 1212 "s59E5H2" 0 "hypothetical protein"
"Chl_A21_00572" 1212 "s63E6H2" 0 "hypothetical protein"
"Chl_A21_00573" 1213 "s59E13H2" 0 "Rubrerythrin"
"Chl_A21_00573" 1213 "s59E25H2" 0 "Rubrerythrin"
"Chl_A21_00573" 1213 "s59E5H2" 0 "Rubrerythrin"
"Chl_A21_00573" 1213 "s63E6H2" 0 "Rubrerythrin"
"Chl_A21_00574" 1214 "s59E13H2" 0 "Methylmalonate semialdehyde dehydrogenase [acylating]"
"Chl_A21_00574" 1214 "s59E25H2" 0 "Methylmalonate semialdehyde dehydrogenase [acylating]"
"Chl_A21_00574" 1214 "s59E5H2" 0 "Methylmalonate semialdehyde dehydrogenase [acylating]"
"Chl_A21_00574" 1214 "s63E6H2" 0 "Methylmalonate semialdehyde dehydrogenase [acylating]"
"Chl_A21_00575" 1215 "s59E13H2" 0 "5-aminovalerate aminotransferase DavT"
"Chl_A21_00575" 1215 "s59E25H2" 0 "5-aminovalerate aminotransferase DavT"
"Chl_A21_00575" 1215 "s59E5H2" 0 "5-aminovalerate aminotransferase DavT"
"Chl_A21_00575" 1215 "s63E6H2" 0 "5-aminovalerate aminotransferase DavT"
"Chl_A21_00576" 1216 "s59E13H2" 0 "D-hydantoinase"
"Chl_A21_00576" 1216 "s59E25H2" 0 "D-hydantoinase"
"Chl_A21_00576" 1216 "s59E5H2" 0 "D-hydantoinase"
"Chl_A21_00576" 1216 "s63E6H2" 0 "D-hydantoinase"
"Chl_A21_00577" 1217 "s59E13H2" 0 "NAD-dependent dihydropyrimidine dehydrogenase subunit PreA"
"Chl_A21_00577" 1217 "s59E25H2" 0 "NAD-dependent dihydropyrimidine dehydrogenase subunit PreA"
"Chl_A21_00577" 1217 "s59E5H2" 0 "NAD-dependent dihydropyrimidine dehydrogenase subunit PreA"
"Chl_A21_00577" 1217 "s63E6H2" 0 "NAD-dependent dihydropyrimidine dehydrogenase subunit PreA"
"Chl_A21_00578" 1218 "s59E13H2" 0 "N-carbamoyl-D-amino acid hydrolase"
"Chl_A21_00578" 1218 "s59E25H2" 0 "N-carbamoyl-D-amino acid hydrolase"
"Chl_A21_00578" 1218 "s59E5H2" 0 "N-carbamoyl-D-amino acid hydrolase"
"Chl_A21_00578" 1218 "s63E6H2" 0 "N-carbamoyl-D-amino acid hydrolase"
"Chl_A21_00579" 1219 "s59E13H2" 0 "Helix-turn-helix domain protein"
"Chl_A21_00579" 1219 "s59E25H2" 0 "Helix-turn-helix domain protein"
"Chl_A21_00579" 1219 "s59E5H2" 0 "Helix-turn-helix domain protein"
"Chl_A21_00579" 1219 "s63E6H2" 0 "Helix-turn-helix domain protein"
"Chl_A21_00580" 1220 "s59E13H2" 0 "hypothetical protein"
"Chl_A21_00580" 1220 "s59E25H2" 0 "hypothetical protein"
"Chl_A21_00580" 1220 "s59E5H2" 0 "hypothetical protein"
"Chl_A21_00580" 1220 "s63E6H2" 0 "hypothetical protein"
"Chl_A21_00581" 1221 "s59E13H2" 0 "hypothetical protein"
"Chl_A21_00581" 1221 "s59E25H2" 0 "hypothetical protein"
"Chl_A21_00581" 1221 "s59E5H2" 0 "hypothetical protein"
"Chl_A21_00581" 1221 "s63E6H2" 0 "hypothetical protein"
"Chl_A21_00583" 1223 "s59E13H2" 0 "ABC transporter arginine-binding protein 1 precursor"
"Chl_A21_00583" 1223 "s59E25H2" 0 "ABC transporter arginine-binding protein 1 precursor"

"Chl_A21_00583" 1223 "s59E5H2" 0 "ABC transporter arginine-binding protein 1 precursor"
"Chl_A21_00583" 1223 "s63E6H2" 0 "ABC transporter arginine-binding protein 1 precursor"
"Chl_A21_00584" 1224 "s59E13H2" 0 "Glutamine transport system permease protein GlnP"
"Chl_A21_00584" 1224 "s59E25H2" 0 "Glutamine transport system permease protein GlnP"
"Chl_A21_00584" 1224 "s59E5H2" 0 "Glutamine transport system permease protein GlnP"
"Chl_A21_00584" 1224 "s63E6H2" 0 "Glutamine transport system permease protein GlnP"
"Chl_A21_00585" 1225 "s59E13H2" 0 "Glutamine transport ATP-binding protein GlnQ"
"Chl_A21_00585" 1225 "s59E25H2" 0 "Glutamine transport ATP-binding protein GlnQ"
"Chl_A21_00585" 1225 "s59E5H2" 0 "Glutamine transport ATP-binding protein GlnQ"
"Chl_A21_00585" 1225 "s63E6H2" 0 "Glutamine transport ATP-binding protein GlnQ"
"Chl_A21_00586" 1226 "s59E13H2" 0 "Antibiotic biosynthesis monooxygenase"
"Chl_A21_00586" 1226 "s59E25H2" 0 "Antibiotic biosynthesis monooxygenase"
"Chl_A21_00586" 1226 "s59E5H2" 0 "Antibiotic biosynthesis monooxygenase"
"Chl_A21_00586" 1226 "s63E6H2" 0 "Antibiotic biosynthesis monooxygenase"
"Chl_A21_00587" 1227 "s59E13H2" 0 "NYN domain protein"
"Chl_A21_00587" 1227 "s59E25H2" 0 "NYN domain protein"
"Chl_A21_00587" 1227 "s59E5H2" 0 "NYN domain protein"
"Chl_A21_00587" 1227 "s63E6H2" 0 "NYN domain protein"
"Chl_A21_00589" 1229 "s59E13H2" 0 "Malate dehydrogenase"
"Chl_A21_00589" 1229 "s59E25H2" 0 "Malate dehydrogenase"
"Chl_A21_00589" 1229 "s59E5H2" 0 "Malate dehydrogenase"
"Chl_A21_00589" 1229 "s63E6H2" 0 "Malate dehydrogenase"
"Chl_A21_00591" 1231 "s59E13H2" 0 "Universal stress protein family protein"
"Chl_A21_00591" 1231 "s59E25H2" 0 "Universal stress protein family protein"
"Chl_A21_00591" 1231 "s59E5H2" 0 "Universal stress protein family protein"
"Chl_A21_00591" 1231 "s63E6H2" 0 "Universal stress protein family protein"
"Chl_A21_00592" 1232 "s59E13H2" 0 "hypothetical protein"
"Chl_A21_00592" 1232 "s59E25H2" 0 "hypothetical protein"
"Chl_A21_00592" 1232 "s59E5H2" 0 "hypothetical protein"
"Chl_A21_00592" 1232 "s63E6H2" 0 "hypothetical protein"
"Chl_A21_00593" 1233 "s59E13H2" 0 "Endonuclease 4"
"Chl_A21_00593" 1233 "s59E25H2" 0 "Endonuclease 4"
"Chl_A21_00593" 1233 "s59E5H2" 0 "Endonuclease 4"
"Chl_A21_00593" 1233 "s63E6H2" 0 "Endonuclease 4"
"Chl_A21_00594" 1234 "s59E13H2" 0 "Carbonic anhydrase precursor"
"Chl_A21_00594" 1234 "s59E25H2" 0 "Carbonic anhydrase precursor"
"Chl_A21_00594" 1234 "s59E5H2" 0 "Carbonic anhydrase precursor"
"Chl_A21_00594" 1234 "s63E6H2" 0 "Carbonic anhydrase precursor"
"Chl_A21_00595" 1235 "s59E13H2" 0 "S-adenosyl-L-methionine-binding protein"
"Chl_A21_00595" 1235 "s59E25H2" 0 "S-adenosyl-L-methionine-binding protein"
"Chl_A21_00595" 1235 "s59E5H2" 0 "S-adenosyl-L-methionine-binding protein"
"Chl_A21_00595" 1235 "s63E6H2" 0 "S-adenosyl-L-methionine-binding protein"
"Chl_A21_00596" 1236 "s59E13H2" 0 "putative hydrogenase nickel incorporation protein HypA"
"Chl_A21_00596" 1236 "s59E25H2" 0 "putative hydrogenase nickel incorporation protein HypA"
"Chl_A21_00596" 1236 "s59E5H2" 0 "putative hydrogenase nickel incorporation protein HypA"
"Chl_A21_00596" 1236 "s63E6H2" 0 "putative hydrogenase nickel incorporation protein HypA"
"Chl_A21_00597" 1237 "s59E13H2" 0 "Carbamoyltransferase HypF"

"Chl_A21_00597" 1237 "s59E25H2" 0 "Carbamoyltransferase HypF"
"Chl_A21_00597" 1237 "s59E5H2" 0 "Carbamoyltransferase HypF"
"Chl_A21_00597" 1237 "s63E6H2" 0 "Carbamoyltransferase HypF"
"Chl_A21_00598" 1238 "s59E13H2" 0 "Hydrogenase isoenzymes formation protein HypC"
"Chl_A21_00598" 1238 "s59E25H2" 0 "Hydrogenase isoenzymes formation protein HypC"
"Chl_A21_00598" 1238 "s59E5H2" 0 "Hydrogenase isoenzymes formation protein HypC"
"Chl_A21_00598" 1238 "s63E6H2" 0 "Hydrogenase isoenzymes formation protein HypC"
"Chl_A21_00599" 1239 "s59E13H2" 0 "Hydrogenase expression/formation protein HypD"
"Chl_A21_00599" 1239 "s59E25H2" 0 "Hydrogenase expression/formation protein HypD"
"Chl_A21_00599" 1239 "s59E5H2" 0 "Hydrogenase expression/formation protein HypD"
"Chl_A21_00599" 1239 "s63E6H2" 0 "Hydrogenase expression/formation protein HypD"
"Chl_A21_00600" 1240 "s59E13H2" 0 "Hydrogenase expression/formation protein HypE"
"Chl_A21_00600" 1240 "s59E25H2" 0 "Hydrogenase expression/formation protein HypE"
"Chl_A21_00600" 1240 "s59E5H2" 0 "Hydrogenase expression/formation protein HypE"
"Chl_A21_00600" 1240 "s63E6H2" 0 "Hydrogenase expression/formation protein HypE"
"Chl_A21_00601" 1241 "s59E13H2" 0 "Formate dehydrogenase subunit alpha"
"Chl_A21_00601" 1241 "s59E25H2" 0 "Formate dehydrogenase subunit alpha"
"Chl_A21_00601" 1241 "s59E5H2" 0 "Formate dehydrogenase subunit alpha"
"Chl_A21_00601" 1241 "s63E6H2" 0 "Formate dehydrogenase subunit alpha"
"Chl_A21_00603" 1243 "s59E13H2" 0 "Formate dehydrogenase subunit alpha"
"Chl_A21_00603" 1243 "s59E25H2" 0 "Formate dehydrogenase subunit alpha"
"Chl_A21_00603" 1243 "s59E5H2" 0 "Formate dehydrogenase subunit alpha"
"Chl_A21_00603" 1243 "s63E6H2" 0 "Formate dehydrogenase subunit alpha"
"Chl_A21_00604" 1244 "s59E13H2" 0 "Formate dehydrogenase subunit beta"
"Chl_A21_00604" 1244 "s59E25H2" 0 "Formate dehydrogenase subunit beta"
"Chl_A21_00604" 1244 "s59E5H2" 0 "Formate dehydrogenase subunit beta"
"Chl_A21_00604" 1244 "s63E6H2" 0 "Formate dehydrogenase subunit beta"
"Chl_A21_00605" 1245 "s59E13H2" 0 "F420-non-reducing hydrogenase iron-sulfur subunit D"
"Chl_A21_00605" 1245 "s59E25H2" 0 "F420-non-reducing hydrogenase iron-sulfur subunit D"
"Chl_A21_00605" 1245 "s59E5H2" 0 "F420-non-reducing hydrogenase iron-sulfur subunit D"
"Chl_A21_00605" 1245 "s63E6H2" 0 "F420-non-reducing hydrogenase iron-sulfur subunit D"
"Chl_A21_00606" 1246 "s59E13H2" 0 "CoB--CoM heterodisulfide reductase iron-sulfur subunit A"
"Chl_A21_00606" 1246 "s59E25H2" 0 "CoB--CoM heterodisulfide reductase iron-sulfur subunit A"
"Chl_A21_00606" 1246 "s59E5H2" 0 "CoB--CoM heterodisulfide reductase iron-sulfur subunit A"
"Chl_A21_00606" 1246 "s63E6H2" 0 "CoB--CoM heterodisulfide reductase iron-sulfur subunit A"
"Chl_A21_00607" 1247 "s59E13H2" 0 "Heterodisulfide reductase subunit A-like protein"
"Chl_A21_00607" 1247 "s59E25H2" 0 "Heterodisulfide reductase subunit A-like protein"
"Chl_A21_00607" 1247 "s59E5H2" 0 "Heterodisulfide reductase subunit A-like protein"
"Chl_A21_00607" 1247 "s63E6H2" 0 "Heterodisulfide reductase subunit A-like protein"
"Chl_A21_00608" 1248 "s59E13H2" 0 "CoB--CoM heterodisulfide reductase subunit B"
"Chl_A21_00608" 1248 "s59E25H2" 0 "CoB--CoM heterodisulfide reductase subunit B"
"Chl_A21_00608" 1248 "s59E5H2" 0 "CoB--CoM heterodisulfide reductase subunit B"
"Chl_A21_00608" 1248 "s63E6H2" 0 "CoB--CoM heterodisulfide reductase subunit B"
"Chl_A21_00609" 1249 "s59E13H2" 0 "CoB--CoM heterodisulfide reductase iron-sulfur subunit C"
"Chl_A21_00609" 1249 "s59E25H2" 0 "CoB--CoM heterodisulfide reductase iron-sulfur subunit C"
"Chl_A21_00609" 1249 "s59E5H2" 0 "CoB--CoM heterodisulfide reductase iron-sulfur subunit C"
"Chl_A21_00609" 1249 "s63E6H2" 0 "CoB--CoM heterodisulfide reductase iron-sulfur subunit C"

"Chl_A21_00610" 1250 "s59E13H2" 0 "Lipoate-protein ligase LplJ"
"Chl_A21_00610" 1250 "s59E25H2" 0 "Lipoate-protein ligase LplJ"
"Chl_A21_00610" 1250 "s59E5H2" 0 "Lipoate-protein ligase LplJ"
"Chl_A21_00610" 1250 "s63E6H2" 0 "Lipoate-protein ligase LplJ"
"Chl_A21_00611" 1251 "s59E13H2" 0 "CoB--CoM heterodisulfide reductase iron-sulfur subunit D"
"Chl_A21_00611" 1251 "s59E25H2" 0 "CoB--CoM heterodisulfide reductase iron-sulfur subunit D"
"Chl_A21_00611" 1251 "s59E5H2" 0 "CoB--CoM heterodisulfide reductase iron-sulfur subunit D"
"Chl_A21_00611" 1251 "s63E6H2" 0 "CoB--CoM heterodisulfide reductase iron-sulfur subunit D"
"Chl_A21_00612" 1252 "s59E13H2" 0 "Glycine cleavage system H protein"
"Chl_A21_00612" 1252 "s59E25H2" 0 "Glycine cleavage system H protein"
"Chl_A21_00612" 1252 "s59E5H2" 0 "Glycine cleavage system H protein"
"Chl_A21_00612" 1252 "s63E6H2" 0 "Glycine cleavage system H protein"
"Chl_A21_00613" 1253 "s59E13H2" 0 "Phosphate import ATP-binding protein PstB 3"
"Chl_A21_00613" 1253 "s59E25H2" 0 "Phosphate import ATP-binding protein PstB 3"
"Chl_A21_00613" 1253 "s59E5H2" 0 "Phosphate import ATP-binding protein PstB 3"
"Chl_A21_00613" 1253 "s63E6H2" 0 "Phosphate import ATP-binding protein PstB 3"
"Chl_A21_00614" 1254 "s59E13H2" 0 "Phosphate transport system permease protein PstA"
"Chl_A21_00614" 1254 "s59E25H2" 0 "Phosphate transport system permease protein PstA"
"Chl_A21_00614" 1254 "s59E5H2" 0 "Phosphate transport system permease protein PstA"
"Chl_A21_00614" 1254 "s63E6H2" 0 "Phosphate transport system permease protein PstA"
"Chl_A21_00615" 1255 "s59E13H2" 0 "Phosphate transport system permease protein PstC"
"Chl_A21_00615" 1255 "s59E25H2" 0 "Phosphate transport system permease protein PstC"
"Chl_A21_00615" 1255 "s59E5H2" 0 "Phosphate transport system permease protein PstC"
"Chl_A21_00615" 1255 "s63E6H2" 0 "Phosphate transport system permease protein PstC"
"Chl_A21_00616" 1256 "s59E13H2" 0 "Phosphate-binding protein PstS 1 precursor"
"Chl_A21_00616" 1256 "s59E25H2" 0 "Phosphate-binding protein PstS 1 precursor"
"Chl_A21_00616" 1256 "s59E5H2" 0 "Phosphate-binding protein PstS 1 precursor"
"Chl_A21_00616" 1256 "s63E6H2" 0 "Phosphate-binding protein PstS 1 precursor"
"Chl_A21_00618" 1258 "s59E13H2" 0 "HTH-type transcriptional regulator CymR"
"Chl_A21_00618" 1258 "s59E25H2" 0 "HTH-type transcriptional regulator CymR"
"Chl_A21_00618" 1258 "s59E5H2" 0 "HTH-type transcriptional regulator CymR"
"Chl_A21_00618" 1258 "s63E6H2" 0 "HTH-type transcriptional regulator CymR"
"Chl_A21_00619" 1259 "s59E13H2" 0 "putative formate transporter 1"
"Chl_A21_00619" 1259 "s59E25H2" 0 "putative formate transporter 1"
"Chl_A21_00619" 1259 "s59E5H2" 0 "putative formate transporter 1"
"Chl_A21_00619" 1259 "s63E6H2" 0 "putative formate transporter 1"
"Chl_A21_00620" 1260 "s59E13H2" 0 "hypothetical protein"
"Chl_A21_00620" 1260 "s59E25H2" 0 "hypothetical protein"
"Chl_A21_00620" 1260 "s59E5H2" 0 "hypothetical protein"
"Chl_A21_00620" 1260 "s63E6H2" 0 "hypothetical protein"
"Chl_A21_00621" 1261 "s59E13H2" 0 "hypothetical protein"
"Chl_A21_00621" 1261 "s59E25H2" 0 "hypothetical protein"
"Chl_A21_00621" 1261 "s59E5H2" 0 "hypothetical protein"
"Chl_A21_00621" 1261 "s63E6H2" 0 "hypothetical protein"
"Chl_A21_00622" 1262 "s59E13H2" 0 "hypothetical protein"
"Chl_A21_00622" 1262 "s59E25H2" 0 "hypothetical protein"
"Chl_A21_00622" 1262 "s59E5H2" 0 "hypothetical protein"

"Chl_A21_00622" 1262 "s63E6H2" 0 "hypothetical protein"
"Chl_A21_00623" 1263 "s59E13H2" 0 "Transcriptional regulatory protein LiaR"
"Chl_A21_00623" 1263 "s59E25H2" 0 "Transcriptional regulatory protein LiaR"
"Chl_A21_00623" 1263 "s59E5H2" 0 "Transcriptional regulatory protein LiaR"
"Chl_A21_00623" 1263 "s63E6H2" 0 "Transcriptional regulatory protein LiaR"
"Chl_A21_00624" 1264 "s59E13H2" 0 "Signal transduction histidine-protein kinase/phosphatase DegS"
"Chl_A21_00624" 1264 "s59E25H2" 0 "Signal transduction histidine-protein kinase/phosphatase DegS"
"Chl_A21_00624" 1264 "s59E5H2" 0 "Signal transduction histidine-protein kinase/phosphatase DegS"
"Chl_A21_00624" 1264 "s63E6H2" 0 "Signal transduction histidine-protein kinase/phosphatase DegS"
"Chl_A21_00625" 1265 "s59E13H2" 0 "Hydrogenase 2 maturation protease"
"Chl_A21_00625" 1265 "s59E25H2" 0 "Hydrogenase 2 maturation protease"
"Chl_A21_00625" 1265 "s59E5H2" 0 "Hydrogenase 2 maturation protease"
"Chl_A21_00625" 1265 "s63E6H2" 0 "Hydrogenase 2 maturation protease"
"Chl_A21_00626" 1266 "s59E13H2" 0 "Periplasmic [NiFeSe] hydrogenase large subunit"
"Chl_A21_00626" 1266 "s59E25H2" 0 "Periplasmic [NiFeSe] hydrogenase large subunit"
"Chl_A21_00626" 1266 "s59E5H2" 0 "Periplasmic [NiFeSe] hydrogenase large subunit"
"Chl_A21_00626" 1266 "s63E6H2" 0 "Periplasmic [NiFeSe] hydrogenase large subunit"
"Chl_A21_00627" 1267 "s59E13H2" 0 "Formate dehydrogenase subunit alpha precursor"
"Chl_A21_00627" 1267 "s59E25H2" 0 "Formate dehydrogenase subunit alpha precursor"
"Chl_A21_00627" 1267 "s59E5H2" 0 "Formate dehydrogenase subunit alpha precursor"
"Chl_A21_00627" 1267 "s63E6H2" 0 "Formate dehydrogenase subunit alpha precursor"
"Chl_A21_00628" 1268 "s59E13H2" 0 "Formate dehydrogenase subunit alpha precursor"
"Chl_A21_00628" 1268 "s59E25H2" 0 "Formate dehydrogenase subunit alpha precursor"
"Chl_A21_00628" 1268 "s59E5H2" 0 "Formate dehydrogenase subunit alpha precursor"
"Chl_A21_00628" 1268 "s63E6H2" 0 "Formate dehydrogenase subunit alpha precursor"
"Chl_A21_00629" 1269 "s59E13H2" 0 "Formate dehydrogenase-O iron-sulfur subunit"
"Chl_A21_00629" 1269 "s59E25H2" 0 "Formate dehydrogenase-O iron-sulfur subunit"
"Chl_A21_00629" 1269 "s59E5H2" 0 "Formate dehydrogenase-O iron-sulfur subunit"
"Chl_A21_00629" 1269 "s63E6H2" 0 "Formate dehydrogenase-O iron-sulfur subunit"
"Chl_A21_00630" 1270 "s59E13H2" 0 "Formate dehydrogenase, cytochrome b556(fdo) subunit"
"Chl_A21_00630" 1270 "s59E25H2" 0 "Formate dehydrogenase, cytochrome b556(fdo) subunit"
"Chl_A21_00630" 1270 "s59E5H2" 0 "Formate dehydrogenase, cytochrome b556(fdo) subunit"
"Chl_A21_00630" 1270 "s63E6H2" 0 "Formate dehydrogenase, cytochrome b556(fdo) subunit"
"Chl_A21_00631" 1271 "s59E13H2" 0 "formate dehydrogenase accessory protein FdhE"
"Chl_A21_00631" 1271 "s59E25H2" 0 "formate dehydrogenase accessory protein FdhE"
"Chl_A21_00631" 1271 "s59E5H2" 0 "formate dehydrogenase accessory protein FdhE"
"Chl_A21_00631" 1271 "s63E6H2" 0 "formate dehydrogenase accessory protein FdhE"
"Chl_A21_00632" 1272 "s59E13H2" 0 "Hydrogenase isoenzymes formation protein HypC"
"Chl_A21_00632" 1272 "s59E25H2" 0 "Hydrogenase isoenzymes formation protein HypC"
"Chl_A21_00632" 1272 "s59E5H2" 0 "Hydrogenase isoenzymes formation protein HypC"
"Chl_A21_00632" 1272 "s63E6H2" 0 "Hydrogenase isoenzymes formation protein HypC"
"Chl_A21_00633" 1273 "s59E13H2" 0 "Protoheme IX farnesyltransferase 1"
"Chl_A21_00633" 1273 "s59E25H2" 0 "Protoheme IX farnesyltransferase 1"
"Chl_A21_00633" 1273 "s59E5H2" 0 "Protoheme IX farnesyltransferase 1"
"Chl_A21_00633" 1273 "s63E6H2" 0 "Protoheme IX farnesyltransferase 1"
"Chl_A21_00634" 1274 "s59E13H2" 0 "formate dehydrogenase accessory protein"
"Chl_A21_00634" 1274 "s59E25H2" 0 "formate dehydrogenase accessory protein"

"Chl_A21_00634" 1274 "s59E5H2" 0 "formate dehydrogenase accessory protein"
"Chl_A21_00634" 1274 "s63E6H2" 0 "formate dehydrogenase accessory protein"
"Chl_A21_00635" 1275 "s59E13H2" 0 "hypothetical protein"
"Chl_A21_00635" 1275 "s59E25H2" 0 "hypothetical protein"
"Chl_A21_00635" 1275 "s59E5H2" 0 "hypothetical protein"
"Chl_A21_00635" 1275 "s63E6H2" 0 "hypothetical protein"
"Chl_A21_00636" 1276 "s59E13H2" 0 "Sec-independent protein translocase protein TatAd"
"Chl_A21_00636" 1276 "s59E25H2" 0 "Sec-independent protein translocase protein TatAd"
"Chl_A21_00636" 1276 "s59E5H2" 0 "Sec-independent protein translocase protein TatAd"
"Chl_A21_00636" 1276 "s63E6H2" 0 "Sec-independent protein translocase protein TatAd"
"Chl_A21_00637" 1277 "s59E13H2" 0 "Bifunctional protein FolD protein"
"Chl_A21_00637" 1277 "s59E25H2" 0 "Bifunctional protein FolD protein"
"Chl_A21_00637" 1277 "s59E5H2" 0 "Bifunctional protein FolD protein"
"Chl_A21_00637" 1277 "s63E6H2" 0 "Bifunctional protein FolD protein"
"Chl_A21_00638" 1278 "s59E13H2" 0 "CoB--CoM heterodisulfide reductase subunit B"
"Chl_A21_00638" 1278 "s59E25H2" 0 "CoB--CoM heterodisulfide reductase subunit B"
"Chl_A21_00638" 1278 "s59E5H2" 0 "CoB--CoM heterodisulfide reductase subunit B"
"Chl_A21_00638" 1278 "s63E6H2" 0 "CoB--CoM heterodisulfide reductase subunit B"
"Chl_A21_00639" 1279 "s59E13H2" 0 "Septum site-determining protein MinD"
"Chl_A21_00639" 1279 "s59E25H2" 0 "Septum site-determining protein MinD"
"Chl_A21_00639" 1279 "s59E5H2" 0 "Septum site-determining protein MinD"
"Chl_A21_00639" 1279 "s63E6H2" 0 "Septum site-determining protein MinD"
"Chl_A21_00640" 1280 "s59E13H2" 0 "phosphodiesterase"
"Chl_A21_00640" 1280 "s59E25H2" 0 "phosphodiesterase"
"Chl_A21_00640" 1280 "s59E5H2" 0 "phosphodiesterase"
"Chl_A21_00640" 1280 "s63E6H2" 0 "phosphodiesterase"
"Chl_A21_00641" 1281 "s59E13H2" 0 "Bifunctional homocysteine S-methyltransferase/5,10-methylenetetrahydrofolate reductase"
"Chl_A21_00641" 1281 "s59E25H2" 0 "Bifunctional homocysteine S-methyltransferase/5,10-methylenetetrahydrofolate reductase"
"Chl_A21_00641" 1281 "s59E5H2" 0 "Bifunctional homocysteine S-methyltransferase/5,10-methylenetetrahydrofolate reductase"
"Chl_A21_00641" 1281 "s63E6H2" 0 "Bifunctional homocysteine S-methyltransferase/5,10-methylenetetrahydrofolate reductase"
"Chl_A21_00642" 1282 "s59E13H2" 0 "hypothetical protein"
"Chl_A21_00642" 1282 "s59E25H2" 0 "hypothetical protein"
"Chl_A21_00642" 1282 "s59E5H2" 0 "hypothetical protein"
"Chl_A21_00642" 1282 "s63E6H2" 0 "hypothetical protein"
"Chl_A21_00643" 1283 "s59E13H2" 0 "Alkaline phosphatase synthesis transcriptional regulatory protein PhoP"
"Chl_A21_00643" 1283 "s59E25H2" 0 "Alkaline phosphatase synthesis transcriptional regulatory protein PhoP"
"Chl_A21_00643" 1283 "s59E5H2" 0 "Alkaline phosphatase synthesis transcriptional regulatory protein PhoP"
"Chl_A21_00643" 1283 "s63E6H2" 0 "Alkaline phosphatase synthesis transcriptional regulatory protein PhoP"
"Chl_A21_00644" 1284 "s59E13H2" 0 "Response regulator MprA"
"Chl_A21_00644" 1284 "s59E25H2" 0 "Response regulator MprA"
"Chl_A21_00644" 1284 "s59E5H2" 0 "Response regulator MprA"
"Chl_A21_00644" 1284 "s63E6H2" 0 "Response regulator MprA"
"Chl_A21_00645" 1285 "s59E13H2" 0 "Bifunctional homocysteine S-methyltransferase/5,10-methylenetetrahydrofolate reductase"
"Chl_A21_00645" 1285 "s59E25H2" 0 "Bifunctional homocysteine S-methyltransferase/5,10-methylenetetrahydrofolate reductase"
"Chl_A21_00645" 1285 "s59E5H2" 0 "Bifunctional homocysteine S-methyltransferase/5,10-methylenetetrahydrofolate reductase"
"Chl_A21_00645" 1285 "s63E6H2" 0 "Bifunctional homocysteine S-methyltransferase/5,10-methylenetetrahydrofolate reductase"
"Chl_A21_00646" 1286 "s59E13H2" 0 "Alkaline phosphatase synthesis sensor protein PhoR"

"Chl_A21_00646" 1286 "s59E25H2" 0 "Alkaline phosphatase synthesis sensor protein PhoR"
"Chl_A21_00646" 1286 "s59E5H2" 0 "Alkaline phosphatase synthesis sensor protein PhoR"
"Chl_A21_00646" 1286 "s63E6H2" 0 "Alkaline phosphatase synthesis sensor protein PhoR"
"Chl_A21_00647" 1287 "s59E13H2" 0 "Periplasmic [NiFeSe] hydrogenase small subunit precursor"
"Chl_A21_00647" 1287 "s59E25H2" 0 "Periplasmic [NiFeSe] hydrogenase small subunit precursor"
"Chl_A21_00647" 1287 "s59E5H2" 0 "Periplasmic [NiFeSe] hydrogenase small subunit precursor"
"Chl_A21_00647" 1287 "s63E6H2" 0 "Periplasmic [NiFeSe] hydrogenase small subunit precursor"
"Chl_A21_00648" 1288 "s59E13H2" 0 "hypothetical protein"
"Chl_A21_00648" 1288 "s59E25H2" 0 "hypothetical protein"
"Chl_A21_00648" 1288 "s59E5H2" 0 "hypothetical protein"
"Chl_A21_00648" 1288 "s63E6H2" 0 "hypothetical protein"
"Chl_A21_00649" 1289 "s59E13H2" 0 "hypothetical protein"
"Chl_A21_00649" 1289 "s59E25H2" 0 "hypothetical protein"
"Chl_A21_00649" 1289 "s59E5H2" 0 "hypothetical protein"
"Chl_A21_00649" 1289 "s63E6H2" 0 "hypothetical protein"
"Chl_A21_00650" 1290 "s59E13H2" 0 "F420-non-reducing hydrogenase iron-sulfur subunit D"
"Chl_A21_00650" 1290 "s59E25H2" 0 "F420-non-reducing hydrogenase iron-sulfur subunit D"
"Chl_A21_00650" 1290 "s59E5H2" 0 "F420-non-reducing hydrogenase iron-sulfur subunit D"
"Chl_A21_00650" 1290 "s63E6H2" 0 "F420-non-reducing hydrogenase iron-sulfur subunit D"
"Chl_A21_00651" 1291 "s59E13H2" 0 "CoB--CoM heterodisulfide reductase iron-sulfur subunit A"
"Chl_A21_00651" 1291 "s59E25H2" 0 "CoB--CoM heterodisulfide reductase iron-sulfur subunit A"
"Chl_A21_00651" 1291 "s59E5H2" 0 "CoB--CoM heterodisulfide reductase iron-sulfur subunit A"
"Chl_A21_00651" 1291 "s63E6H2" 0 "CoB--CoM heterodisulfide reductase iron-sulfur subunit A"
"Chl_A21_00652" 1292 "s59E13H2" 0 "NAD-dependent malic enzyme"
"Chl_A21_00652" 1292 "s59E25H2" 0 "NAD-dependent malic enzyme"
"Chl_A21_00652" 1292 "s59E5H2" 0 "NAD-dependent malic enzyme"
"Chl_A21_00652" 1292 "s63E6H2" 0 "NAD-dependent malic enzyme"
"Chl_A21_00653" 1293 "s59E13H2" 0 "5-methyltetrahydrofolate:corrinoid/iron-sulfur protein co-methyltransferase"
"Chl_A21_00653" 1293 "s59E25H2" 0 "5-methyltetrahydrofolate:corrinoid/iron-sulfur protein co-methyltransferase"
"Chl_A21_00653" 1293 "s59E5H2" 0 "5-methyltetrahydrofolate:corrinoid/iron-sulfur protein co-methyltransferase"
"Chl_A21_00653" 1293 "s63E6H2" 0 "5-methyltetrahydrofolate:corrinoid/iron-sulfur protein co-methyltransferase"
"Chl_A21_00654" 1294 "s59E13H2" 0 "Corrinoid/iron-sulfur protein large subunit"
"Chl_A21_00654" 1294 "s59E25H2" 0 "Corrinoid/iron-sulfur protein large subunit"
"Chl_A21_00654" 1294 "s59E5H2" 0 "Corrinoid/iron-sulfur protein large subunit"
"Chl_A21_00654" 1294 "s63E6H2" 0 "Corrinoid/iron-sulfur protein large subunit"
"Chl_A21_00655" 1295 "s59E13H2" 0 "hypothetical protein"
"Chl_A21_00655" 1295 "s59E25H2" 0 "hypothetical protein"
"Chl_A21_00655" 1295 "s59E5H2" 0 "hypothetical protein"
"Chl_A21_00655" 1295 "s63E6H2" 0 "hypothetical protein"
"Chl_A21_00656" 1296 "s59E13H2" 0 "metal-dependent hydrolase"
"Chl_A21_00656" 1296 "s59E25H2" 0 "metal-dependent hydrolase"
"Chl_A21_00656" 1296 "s59E5H2" 0 "metal-dependent hydrolase"
"Chl_A21_00656" 1296 "s63E6H2" 0 "metal-dependent hydrolase"
"Chl_A21_00657" 1297 "s59E13H2" 0 "GTP cyclohydrolase 1"
"Chl_A21_00657" 1297 "s59E25H2" 0 "GTP cyclohydrolase 1"
"Chl_A21_00657" 1297 "s59E5H2" 0 "GTP cyclohydrolase 1"
"Chl_A21_00657" 1297 "s63E6H2" 0 "GTP cyclohydrolase 1"

"Chl_A21_00658" 1298 "s59E13H2" 0 "Dihydropteroate synthase"
"Chl_A21_00658" 1298 "s59E25H2" 0 "Dihydropteroate synthase"
"Chl_A21_00658" 1298 "s59E5H2" 0 "Dihydropteroate synthase"
"Chl_A21_00658" 1298 "s63E6H2" 0 "Dihydropteroate synthase"
"Chl_A21_00659" 1299 "s59E13H2" 0 "Protease 3 precursor"
"Chl_A21_00659" 1299 "s59E25H2" 0 "Protease 3 precursor"
"Chl_A21_00659" 1299 "s59E5H2" 0 "Protease 3 precursor"
"Chl_A21_00659" 1299 "s63E6H2" 0 "Protease 3 precursor"
"Chl_A21_00660" 1300 "s59E13H2" 0 "hypothetical protein"
"Chl_A21_00660" 1300 "s59E25H2" 0 "hypothetical protein"
"Chl_A21_00660" 1300 "s59E5H2" 0 "hypothetical protein"
"Chl_A21_00660" 1300 "s63E6H2" 0 "hypothetical protein"
"Chl_A21_00661" 1301 "s59E13H2" 0 "Exodeoxyribonuclease 7 small subunit"
"Chl_A21_00661" 1301 "s59E25H2" 0 "Exodeoxyribonuclease 7 small subunit"
"Chl_A21_00661" 1301 "s59E5H2" 0 "Exodeoxyribonuclease 7 small subunit"
"Chl_A21_00661" 1301 "s63E6H2" 0 "Exodeoxyribonuclease 7 small subunit"
"Chl_A21_00662" 1302 "s59E13H2" 0 "Exodeoxyribonuclease 7 large subunit"
"Chl_A21_00662" 1302 "s59E25H2" 0 "Exodeoxyribonuclease 7 large subunit"
"Chl_A21_00662" 1302 "s59E5H2" 0 "Exodeoxyribonuclease 7 large subunit"
"Chl_A21_00662" 1302 "s63E6H2" 0 "Exodeoxyribonuclease 7 large subunit"
"Chl_A21_00663" 1303 "s59E13H2" 0 "Exodeoxyribonuclease 7 large subunit"
"Chl_A21_00663" 1303 "s59E25H2" 0 "Exodeoxyribonuclease 7 large subunit"
"Chl_A21_00663" 1303 "s59E5H2" 0 "Exodeoxyribonuclease 7 large subunit"
"Chl_A21_00663" 1303 "s63E6H2" 0 "Exodeoxyribonuclease 7 large subunit"
"Chl_A21_00664" 1304 "s59E13H2" 0 "hypothetical protein"
"Chl_A21_00664" 1304 "s59E25H2" 0 "hypothetical protein"
"Chl_A21_00664" 1304 "s59E5H2" 0 "hypothetical protein"
"Chl_A21_00664" 1304 "s63E6H2" 0 "hypothetical protein"
"Chl_A21_00665" 1305 "s59E13H2" 0 "hypothetical protein"
"Chl_A21_00665" 1305 "s59E25H2" 0 "hypothetical protein"
"Chl_A21_00665" 1305 "s59E5H2" 0 "hypothetical protein"
"Chl_A21_00665" 1305 "s63E6H2" 0 "hypothetical protein"
"Chl_A21_00667" 1307 "s59E13H2" 0 "Trigger factor"
"Chl_A21_00667" 1307 "s59E25H2" 0 "Trigger factor"
"Chl_A21_00667" 1307 "s59E5H2" 0 "Trigger factor"
"Chl_A21_00667" 1307 "s63E6H2" 0 "Trigger factor"
"Chl_A21_00668" 1308 "s59E13H2" 0 "ATP-dependent Clp protease proteolytic subunit precursor"
"Chl_A21_00668" 1308 "s59E25H2" 360.2596 "ATP-dependent Clp protease proteolytic subunit precursor"
"Chl_A21_00668" 1308 "s59E5H2" 0 "ATP-dependent Clp protease proteolytic subunit precursor"
"Chl_A21_00668" 1308 "s63E6H2" 0 "ATP-dependent Clp protease proteolytic subunit precursor"
"Chl_A21_00669" 1309 "s59E13H2" 0 "ATP-dependent Clp protease ATP-binding subunit ClpX"
"Chl_A21_00669" 1309 "s59E25H2" 308.6438 "ATP-dependent Clp protease ATP-binding subunit ClpX"
"Chl_A21_00669" 1309 "s59E5H2" 0 "ATP-dependent Clp protease ATP-binding subunit ClpX"
"Chl_A21_00669" 1309 "s63E6H2" 0 "ATP-dependent Clp protease ATP-binding subunit ClpX"
"Chl_A21_00670" 1310 "s59E13H2" 0 "translocation protein TolB"
"Chl_A21_00670" 1310 "s59E25H2" 0 "translocation protein TolB"
"Chl_A21_00670" 1310 "s59E5H2" 0 "translocation protein TolB"

"Chl_A21_00670" 1310 "s63E6H2" 0 "translocation protein TolB"
"Chl_A21_00671" 1311 "s59E13H2" 0 "Cyclic pyranopterin monophosphate synthase"
"Chl_A21_00671" 1311 "s59E25H2" 0 "Cyclic pyranopterin monophosphate synthase"
"Chl_A21_00671" 1311 "s59E5H2" 0 "Cyclic pyranopterin monophosphate synthase"
"Chl_A21_00671" 1311 "s63E6H2" 0 "Cyclic pyranopterin monophosphate synthase"
"Chl_A21_00673" 1313 "s59E13H2" 0 "D-inositol 3-phosphate glycosyltransferase"
"Chl_A21_00673" 1313 "s59E25H2" 0 "D-inositol 3-phosphate glycosyltransferase"
"Chl_A21_00673" 1313 "s59E5H2" 0 "D-inositol 3-phosphate glycosyltransferase"
"Chl_A21_00673" 1313 "s63E6H2" 0 "D-inositol 3-phosphate glycosyltransferase"
"Chl_A21_00674" 1314 "s59E13H2" 0 "hypothetical protein"
"Chl_A21_00674" 1314 "s59E25H2" 0 "hypothetical protein"
"Chl_A21_00674" 1314 "s59E5H2" 0 "hypothetical protein"
"Chl_A21_00674" 1314 "s63E6H2" 0 "hypothetical protein"
"Chl_A21_00675" 1315 "s59E13H2" 0 "30S ribosomal protein S1"
"Chl_A21_00675" 1315 "s59E25H2" 0 "30S ribosomal protein S1"
"Chl_A21_00675" 1315 "s59E5H2" 0 "30S ribosomal protein S1"
"Chl_A21_00675" 1315 "s63E6H2" 0 "30S ribosomal protein S1"
"Chl_A21_00676" 1316 "s59E13H2" 0 "DNA-directed RNA polymerase subunit beta'"
"Chl_A21_00676" 1316 "s59E25H2" 0 "DNA-directed RNA polymerase subunit beta'"
"Chl_A21_00676" 1316 "s59E5H2" 0 "DNA-directed RNA polymerase subunit beta'"
"Chl_A21_00676" 1316 "s63E6H2" 0 "DNA-directed RNA polymerase subunit beta'"
"Chl_A21_00677" 1317 "s59E13H2" 0 "CoB--CoM heterodisulfide reductase iron-sulfur subunit D"
"Chl_A21_00677" 1317 "s59E25H2" 0 "CoB--CoM heterodisulfide reductase iron-sulfur subunit D"
"Chl_A21_00677" 1317 "s59E5H2" 0 "CoB--CoM heterodisulfide reductase iron-sulfur subunit D"
"Chl_A21_00677" 1317 "s63E6H2" 0 "CoB--CoM heterodisulfide reductase iron-sulfur subunit D"
"Chl_A21_00678" 1318 "s59E13H2" 0 "Demethylmenaquinone methyltransferase"
"Chl_A21_00678" 1318 "s59E25H2" 0 "Demethylmenaquinone methyltransferase"
"Chl_A21_00678" 1318 "s59E5H2" 0 "Demethylmenaquinone methyltransferase"
"Chl_A21_00678" 1318 "s63E6H2" 0 "Demethylmenaquinone methyltransferase"
"Chl_A21_00679" 1319 "s59E13H2" 0 "1,4-dihydroxy-2-naphthoate octaprenyltransferase"
"Chl_A21_00679" 1319 "s59E25H2" 0 "1,4-dihydroxy-2-naphthoate octaprenyltransferase"
"Chl_A21_00679" 1319 "s59E5H2" 0 "1,4-dihydroxy-2-naphthoate octaprenyltransferase"
"Chl_A21_00679" 1319 "s63E6H2" 0 "1,4-dihydroxy-2-naphthoate octaprenyltransferase"
"Chl_A21_00680" 1320 "s59E13H2" 0 "Branched-chain amino acid transport system / permease component"
"Chl_A21_00680" 1320 "s59E25H2" 0 "Branched-chain amino acid transport system / permease component"
"Chl_A21_00680" 1320 "s59E5H2" 0 "Branched-chain amino acid transport system / permease component"
"Chl_A21_00680" 1320 "s63E6H2" 0 "Branched-chain amino acid transport system / permease component"
"Chl_A21_00681" 1321 "s59E13H2" 0 "beta-methylgalactoside transporter inner membrane component"
"Chl_A21_00681" 1321 "s59E25H2" 0 "beta-methylgalactoside transporter inner membrane component"
"Chl_A21_00681" 1321 "s59E5H2" 0 "beta-methylgalactoside transporter inner membrane component"
"Chl_A21_00681" 1321 "s63E6H2" 0 "beta-methylgalactoside transporter inner membrane component"
"Chl_A21_00682" 1322 "s59E13H2" 0 "Ribose import ATP-binding protein RbsA"
"Chl_A21_00682" 1322 "s59E25H2" 0 "Ribose import ATP-binding protein RbsA"
"Chl_A21_00682" 1322 "s59E5H2" 0 "Ribose import ATP-binding protein RbsA"
"Chl_A21_00682" 1322 "s63E6H2" 0 "Ribose import ATP-binding protein RbsA"
"Chl_A21_00683" 1323 "s59E13H2" 0 "Membrane lipoprotein TmpC precursor"
"Chl_A21_00683" 1323 "s59E25H2" 0 "Membrane lipoprotein TmpC precursor"

"Chl_A21_00683" 1323 "s59E5H2" 0 "Membrane lipoprotein TmpC precursor"
"Chl_A21_00683" 1323 "s63E6H2" 0 "Membrane lipoprotein TmpC precursor"
"Chl_A21_00684" 1324 "s59E13H2" 0 "Uracil DNA glycosylase superfamily protein"
"Chl_A21_00684" 1324 "s59E25H2" 0 "Uracil DNA glycosylase superfamily protein"
"Chl_A21_00684" 1324 "s59E5H2" 0 "Uracil DNA glycosylase superfamily protein"
"Chl_A21_00684" 1324 "s63E6H2" 0 "Uracil DNA glycosylase superfamily protein"
"Chl_A21_00686" 1326 "s59E13H2" 0 "hypothetical protein"
"Chl_A21_00686" 1326 "s59E25H2" 0 "hypothetical protein"
"Chl_A21_00686" 1326 "s59E5H2" 0 "hypothetical protein"
"Chl_A21_00686" 1326 "s63E6H2" 0 "hypothetical protein"
"Chl_A21_00688" 1328 "s59E13H2" 0 "Helix-turn-helix domain protein"
"Chl_A21_00688" 1328 "s59E25H2" 0 "Helix-turn-helix domain protein"
"Chl_A21_00688" 1328 "s59E5H2" 0 "Helix-turn-helix domain protein"
"Chl_A21_00688" 1328 "s63E6H2" 0 "Helix-turn-helix domain protein"
"Chl_A21_00689" 1329 "s59E13H2" 0 "hypothetical protein"
"Chl_A21_00689" 1329 "s59E25H2" 0 "hypothetical protein"
"Chl_A21_00689" 1329 "s59E5H2" 0 "hypothetical protein"
"Chl_A21_00689" 1329 "s63E6H2" 0 "hypothetical protein"
"Chl_A21_00690" 1330 "s59E13H2" 0 "hypothetical protein"
"Chl_A21_00690" 1330 "s59E25H2" 0 "hypothetical protein"
"Chl_A21_00690" 1330 "s59E5H2" 0 "hypothetical protein"
"Chl_A21_00690" 1330 "s63E6H2" 0 "hypothetical protein"
"Chl_A21_00691" 1331 "s59E13H2" 0 "DNA repair protein RecN"
"Chl_A21_00691" 1331 "s59E25H2" 0 "DNA repair protein RecN"
"Chl_A21_00691" 1331 "s59E5H2" 0 "DNA repair protein RecN"
"Chl_A21_00691" 1331 "s63E6H2" 0 "DNA repair protein RecN"
"Chl_A21_00692" 1332 "s59E13H2" 0 "hypothetical protein"
"Chl_A21_00692" 1332 "s59E25H2" 0 "hypothetical protein"
"Chl_A21_00692" 1332 "s59E5H2" 0 "hypothetical protein"
"Chl_A21_00692" 1332 "s63E6H2" 0 "hypothetical protein"
"Chl_A21_00693" 1333 "s59E13H2" 0 "putative inorganic polyphosphate/ATP-NAD kinase"
"Chl_A21_00693" 1333 "s59E25H2" 0 "putative inorganic polyphosphate/ATP-NAD kinase"
"Chl_A21_00693" 1333 "s59E5H2" 0 "putative inorganic polyphosphate/ATP-NAD kinase"
"Chl_A21_00693" 1333 "s63E6H2" 0 "putative inorganic polyphosphate/ATP-NAD kinase"
"Chl_A21_00694" 1334 "s59E13H2" 0 "Segregation and condensation protein B"
"Chl_A21_00694" 1334 "s59E25H2" 0 "Segregation and condensation protein B"
"Chl_A21_00694" 1334 "s59E5H2" 0 "Segregation and condensation protein B"
"Chl_A21_00694" 1334 "s63E6H2" 0 "Segregation and condensation protein B"
"Chl_A21_00695" 1335 "s59E13H2" 0 "Cytidylate kinase"
"Chl_A21_00695" 1335 "s59E25H2" 0 "Cytidylate kinase"
"Chl_A21_00695" 1335 "s59E5H2" 0 "Cytidylate kinase"
"Chl_A21_00695" 1335 "s63E6H2" 0 "Cytidylate kinase"
"Chl_A21_00696" 1336 "s59E13H2" 0 "hypothetical protein"
"Chl_A21_00696" 1336 "s59E25H2" 0 "hypothetical protein"
"Chl_A21_00696" 1336 "s59E5H2" 0 "hypothetical protein"
"Chl_A21_00696" 1336 "s63E6H2" 0 "hypothetical protein"
"Chl_A21_00697" 1337 "s59E13H2" 0 "hypothetical protein"

"Chl_A21_00697" 1337 "s59E25H2" 0 "hypothetical protein"
"Chl_A21_00697" 1337 "s59E5H2" 0 "hypothetical protein"
"Chl_A21_00697" 1337 "s63E6H2" 0 "hypothetical protein"
"Chl_A21_00698" 1338 "s59E13H2" 0 "Glycosyltransferase AglJ"
"Chl_A21_00698" 1338 "s59E25H2" 0 "Glycosyltransferase AglJ"
"Chl_A21_00698" 1338 "s59E5H2" 0 "Glycosyltransferase AglJ"
"Chl_A21_00698" 1338 "s63E6H2" 0 "Glycosyltransferase AglJ"
"Chl_A21_00699" 1339 "s59E13H2" 0 "hypothetical protein"
"Chl_A21_00699" 1339 "s59E25H2" 0 "hypothetical protein"
"Chl_A21_00699" 1339 "s59E5H2" 0 "hypothetical protein"
"Chl_A21_00699" 1339 "s63E6H2" 0 "hypothetical protein"
"Chl_A21_00701" 1341 "s59E13H2" 0 "D-alanine--D-alanine ligase"
"Chl_A21_00701" 1341 "s59E25H2" 0 "D-alanine--D-alanine ligase"
"Chl_A21_00701" 1341 "s59E5H2" 0 "D-alanine--D-alanine ligase"
"Chl_A21_00701" 1341 "s63E6H2" 0 "D-alanine--D-alanine ligase"
"Chl_A21_00702" 1342 "s59E13H2" 0 "L-lysine 2,3-aminomutase"
"Chl_A21_00702" 1342 "s59E25H2" 0 "L-lysine 2,3-aminomutase"
"Chl_A21_00702" 1342 "s59E5H2" 0 "L-lysine 2,3-aminomutase"
"Chl_A21_00702" 1342 "s63E6H2" 0 "L-lysine 2,3-aminomutase"
"Chl_A21_00703" 1343 "s59E13H2" 0 "Acetyltransferase (GNAT) family protein"
"Chl_A21_00703" 1343 "s59E25H2" 0 "Acetyltransferase (GNAT) family protein"
"Chl_A21_00703" 1343 "s59E5H2" 0 "Acetyltransferase (GNAT) family protein"
"Chl_A21_00703" 1343 "s63E6H2" 0 "Acetyltransferase (GNAT) family protein"
"Chl_A21_00704" 1344 "s59E13H2" 0 "hypothetical protein"
"Chl_A21_00704" 1344 "s59E25H2" 0 "hypothetical protein"
"Chl_A21_00704" 1344 "s59E5H2" 0.001508068 "hypothetical protein"
"Chl_A21_00704" 1344 "s63E6H2" 0 "hypothetical protein"
"Chl_A21_00705" 1345 "s59E13H2" 0 "Phosphate regulon transcriptional regulatory protein PhoB"
"Chl_A21_00705" 1345 "s59E25H2" 0 "Phosphate regulon transcriptional regulatory protein PhoB"
"Chl_A21_00705" 1345 "s59E5H2" 0 "Phosphate regulon transcriptional regulatory protein PhoB"
"Chl_A21_00705" 1345 "s63E6H2" 0 "Phosphate regulon transcriptional regulatory protein PhoB"
"Chl_A21_00706" 1346 "s59E13H2" 0 "Dinitrogenase iron-molybdenum cofactor"
"Chl_A21_00706" 1346 "s59E25H2" 0 "Dinitrogenase iron-molybdenum cofactor"
"Chl_A21_00706" 1346 "s59E5H2" 0 "Dinitrogenase iron-molybdenum cofactor"
"Chl_A21_00706" 1346 "s63E6H2" 0 "Dinitrogenase iron-molybdenum cofactor"
"Chl_A21_00707" 1347 "s59E13H2" 0 "Thiol:disulfide interchange protein DsbD"
"Chl_A21_00707" 1347 "s59E25H2" 0 "Thiol:disulfide interchange protein DsbD"
"Chl_A21_00707" 1347 "s59E5H2" 0 "Thiol:disulfide interchange protein DsbD"
"Chl_A21_00707" 1347 "s63E6H2" 0 "Thiol:disulfide interchange protein DsbD"
"Chl_A21_00708" 1348 "s59E13H2" 0 "lineage-specific thermal regulator protein"
"Chl_A21_00708" 1348 "s59E25H2" 0 "lineage-specific thermal regulator protein"
"Chl_A21_00708" 1348 "s59E5H2" 0 "lineage-specific thermal regulator protein"
"Chl_A21_00708" 1348 "s63E6H2" 0 "lineage-specific thermal regulator protein"
"Chl_A21_00709" 1349 "s59E13H2" 0 "Competence protein ComM"
"Chl_A21_00709" 1349 "s59E25H2" 0 "Competence protein ComM"
"Chl_A21_00709" 1349 "s59E5H2" 0 "Competence protein ComM"
"Chl_A21_00709" 1349 "s63E6H2" 0 "Competence protein ComM"

"Chl_A21_00710" 1350 "s59E13H2" 0 "L-threonine dehydratase catabolic TdcB"
"Chl_A21_00710" 1350 "s59E25H2" 0 "L-threonine dehydratase catabolic TdcB"
"Chl_A21_00710" 1350 "s59E5H2" 0 "L-threonine dehydratase catabolic TdcB"
"Chl_A21_00710" 1350 "s63E6H2" 0 "L-threonine dehydratase catabolic TdcB"
"Chl_A21_00711" 1351 "s59E13H2" 0 "D-aminopeptidase"
"Chl_A21_00711" 1351 "s59E25H2" 0 "D-aminopeptidase"
"Chl_A21_00711" 1351 "s59E5H2" 0 "D-aminopeptidase"
"Chl_A21_00711" 1351 "s63E6H2" 0 "D-aminopeptidase"
"Chl_A21_00712" 1352 "s59E13H2" 0 "Proline dehydrogenase 1"
"Chl_A21_00712" 1352 "s59E25H2" 0 "Proline dehydrogenase 1"
"Chl_A21_00712" 1352 "s59E5H2" 0 "Proline dehydrogenase 1"
"Chl_A21_00712" 1352 "s63E6H2" 0 "Proline dehydrogenase 1"
"Chl_A21_00713" 1353 "s59E13H2" 0 "Serine dehydrogenase proteinase"
"Chl_A21_00713" 1353 "s59E25H2" 0 "Serine dehydrogenase proteinase"
"Chl_A21_00713" 1353 "s59E5H2" 0 "Serine dehydrogenase proteinase"
"Chl_A21_00713" 1353 "s63E6H2" 0 "Serine dehydrogenase proteinase"
"Chl_A21_00714" 1354 "s59E13H2" 0 "Vitamin B12 dependent methionine synthase, activation domain"
"Chl_A21_00714" 1354 "s59E25H2" 0 "Vitamin B12 dependent methionine synthase, activation domain"
"Chl_A21_00714" 1354 "s59E5H2" 0 "Vitamin B12 dependent methionine synthase, activation domain"
"Chl_A21_00714" 1354 "s63E6H2" 0 "Vitamin B12 dependent methionine synthase, activation domain"
"Chl_A21_00715" 1355 "s59E13H2" 0 "NifU-like protein"
"Chl_A21_00715" 1355 "s59E25H2" 0 "NifU-like protein"
"Chl_A21_00715" 1355 "s59E5H2" 0 "NifU-like protein"
"Chl_A21_00715" 1355 "s63E6H2" 0 "NifU-like protein"
"Chl_A21_00716" 1356 "s59E13H2" 0 "Putative arsenical pump-driving ATPase"
"Chl_A21_00716" 1356 "s59E25H2" 0 "Putative arsenical pump-driving ATPase"
"Chl_A21_00716" 1356 "s59E5H2" 0 "Putative arsenical pump-driving ATPase"
"Chl_A21_00716" 1356 "s63E6H2" 0 "Putative arsenical pump-driving ATPase"
"Chl_A21_00717" 1357 "s59E13H2" 0 "putative cysteine desulfurase 2"
"Chl_A21_00717" 1357 "s59E25H2" 0 "putative cysteine desulfurase 2"
"Chl_A21_00717" 1357 "s59E5H2" 0 "putative cysteine desulfurase 2"
"Chl_A21_00717" 1357 "s63E6H2" 0 "putative cysteine desulfurase 2"
"Chl_A21_00718" 1358 "s59E13H2" 0 "O-acetylserine sulfhydrylase"
"Chl_A21_00718" 1358 "s59E25H2" 0 "O-acetylserine sulfhydrylase"
"Chl_A21_00718" 1358 "s59E5H2" 0 "O-acetylserine sulfhydrylase"
"Chl_A21_00718" 1358 "s63E6H2" 0 "O-acetylserine sulfhydrylase"
"Chl_A21_00719" 1359 "s59E13H2" 0 "Serine acetyltransferase"
"Chl_A21_00719" 1359 "s59E25H2" 0 "Serine acetyltransferase"
"Chl_A21_00719" 1359 "s59E5H2" 0 "Serine acetyltransferase"
"Chl_A21_00719" 1359 "s63E6H2" 0 "Serine acetyltransferase"
"Chl_A21_00720" 1360 "s59E13H2" 0 "HTH-type transcriptional regulator CymR"
"Chl_A21_00720" 1360 "s59E25H2" 0 "HTH-type transcriptional regulator CymR"
"Chl_A21_00720" 1360 "s59E5H2" 0 "HTH-type transcriptional regulator CymR"
"Chl_A21_00720" 1360 "s63E6H2" 0 "HTH-type transcriptional regulator CymR"
"Chl_A21_00721" 1361 "s59E13H2" 0 "DegV domain-containing protein"
"Chl_A21_00721" 1361 "s59E25H2" 0 "DegV domain-containing protein"
"Chl_A21_00721" 1361 "s59E5H2" 0 "DegV domain-containing protein"

"Chl_A21_00721" 1361 "s63E6H2" 0 "DegV domain-containing protein"
"Chl_A21_00722" 1362 "s59E13H2" 0 "Phosphate acyltransferase"
"Chl_A21_00722" 1362 "s59E25H2" 0 "Phosphate acyltransferase"
"Chl_A21_00722" 1362 "s59E5H2" 0 "Phosphate acyltransferase"
"Chl_A21_00722" 1362 "s63E6H2" 0 "Phosphate acyltransferase"
"Chl_A21_00723" 1363 "s59E13H2" 0 "3-oxoacyl-[acyl-carrier-protein] synthase 2"
"Chl_A21_00723" 1363 "s59E25H2" 0 "3-oxoacyl-[acyl-carrier-protein] synthase 2"
"Chl_A21_00723" 1363 "s59E5H2" 0 "3-oxoacyl-[acyl-carrier-protein] synthase 2"
"Chl_A21_00723" 1363 "s63E6H2" 0 "3-oxoacyl-[acyl-carrier-protein] synthase 2"
"Chl_A21_00724" 1364 "s59E13H2" 0 "hypothetical protein"
"Chl_A21_00724" 1364 "s59E25H2" 0 "hypothetical protein"
"Chl_A21_00724" 1364 "s59E5H2" 0 "hypothetical protein"
"Chl_A21_00724" 1364 "s63E6H2" 0 "hypothetical protein"
"Chl_A21_00725" 1365 "s59E13H2" 0 "hypothetical protein"
"Chl_A21_00725" 1365 "s59E25H2" 0 "hypothetical protein"
"Chl_A21_00725" 1365 "s59E5H2" 0 "hypothetical protein"
"Chl_A21_00725" 1365 "s63E6H2" 0 "hypothetical protein"
"Chl_A21_00726" 1366 "s59E13H2" 0 "Peptidase family M50"
"Chl_A21_00726" 1366 "s59E25H2" 0 "Peptidase family M50"
"Chl_A21_00726" 1366 "s59E5H2" 0 "Peptidase family M50"
"Chl_A21_00726" 1366 "s63E6H2" 0 "Peptidase family M50"
"Chl_A21_00727" 1367 "s59E13H2" 0 "Galactokinase"
"Chl_A21_00727" 1367 "s59E25H2" 0 "Galactokinase"
"Chl_A21_00727" 1367 "s59E5H2" 0 "Galactokinase"
"Chl_A21_00727" 1367 "s63E6H2" 0 "Galactokinase"
"Chl_A21_00728" 1368 "s59E13H2" 0 "hypothetical protein"
"Chl_A21_00728" 1368 "s59E25H2" 0 "hypothetical protein"
"Chl_A21_00728" 1368 "s59E5H2" 0 "hypothetical protein"
"Chl_A21_00728" 1368 "s63E6H2" 0 "hypothetical protein"
"Chl_A21_00729" 1369 "s59E13H2" 0 "Pyridoxal biosynthesis lyase PdxS"
"Chl_A21_00729" 1369 "s59E25H2" 0 "Pyridoxal biosynthesis lyase PdxS"
"Chl_A21_00729" 1369 "s59E5H2" 0 "Pyridoxal biosynthesis lyase PdxS"
"Chl_A21_00729" 1369 "s63E6H2" 0 "Pyridoxal biosynthesis lyase PdxS"
"Chl_A21_00730" 1370 "s59E13H2" 0 "Glutamine amidotransferase subunit PdxT"
"Chl_A21_00730" 1370 "s59E25H2" 0 "Glutamine amidotransferase subunit PdxT"
"Chl_A21_00730" 1370 "s59E5H2" 0 "Glutamine amidotransferase subunit PdxT"
"Chl_A21_00730" 1370 "s63E6H2" 0 "Glutamine amidotransferase subunit PdxT"
"Chl_A21_00732" 1372 "s59E13H2" 0 "R3H domain protein"
"Chl_A21_00732" 1372 "s59E25H2" 0 "R3H domain protein"
"Chl_A21_00732" 1372 "s59E5H2" 0 "R3H domain protein"
"Chl_A21_00732" 1372 "s63E6H2" 0 "R3H domain protein"
"Chl_A21_00733" 1373 "s59E13H2" 0 "Thymidylate kinase"
"Chl_A21_00733" 1373 "s59E25H2" 0 "Thymidylate kinase"
"Chl_A21_00733" 1373 "s59E5H2" 0 "Thymidylate kinase"
"Chl_A21_00733" 1373 "s63E6H2" 0 "Thymidylate kinase"
"Chl_A21_00734" 1374 "s59E13H2" 0 "Zinc ribbon domain protein"
"Chl_A21_00734" 1374 "s59E25H2" 0 "Zinc ribbon domain protein"

"Chl_A21_00734" 1374 "s59E5H2" 0 "Zinc ribbon domain protein"
"Chl_A21_00734" 1374 "s63E6H2" 0 "Zinc ribbon domain protein"
"Chl_A21_00735" 1375 "s59E13H2" 0 "hypothetical protein"
"Chl_A21_00735" 1375 "s59E25H2" 0 "hypothetical protein"
"Chl_A21_00735" 1375 "s59E5H2" 0 "hypothetical protein"
"Chl_A21_00735" 1375 "s63E6H2" 0 "hypothetical protein"
"Chl_A21_00736" 1376 "s59E13H2" 0 "Guanylate kinase"
"Chl_A21_00736" 1376 "s59E25H2" 0 "Guanylate kinase"
"Chl_A21_00736" 1376 "s59E5H2" 0 "Guanylate kinase"
"Chl_A21_00736" 1376 "s63E6H2" 0 "Guanylate kinase"
"Chl_A21_00737" 1377 "s59E13H2" 0 "hypothetical protein"
"Chl_A21_00737" 1377 "s59E25H2" 0 "hypothetical protein"
"Chl_A21_00737" 1377 "s59E5H2" 0 "hypothetical protein"
"Chl_A21_00737" 1377 "s63E6H2" 0 "hypothetical protein"
"Chl_A21_00738" 1378 "s59E13H2" 0 "RNA recognition motif. (a.k.a. RRM, RBD, or RNP domain)"
"Chl_A21_00738" 1378 "s59E25H2" 0 "RNA recognition motif. (a.k.a. RRM, RBD, or RNP domain)"
"Chl_A21_00738" 1378 "s59E5H2" 0 "RNA recognition motif. (a.k.a. RRM, RBD, or RNP domain)"
"Chl_A21_00738" 1378 "s63E6H2" 0 "RNA recognition motif. (a.k.a. RRM, RBD, or RNP domain)"
"Chl_A21_00739" 1379 "s59E13H2" 0 "hypothetical protein"
"Chl_A21_00739" 1379 "s59E25H2" 0 "hypothetical protein"
"Chl_A21_00739" 1379 "s59E5H2" 0 "hypothetical protein"
"Chl_A21_00739" 1379 "s63E6H2" 0 "hypothetical protein"
"Chl_A21_00741" 1381 "s59E13H2" 0 "hypothetical protein"
"Chl_A21_00741" 1381 "s59E25H2" 0 "hypothetical protein"
"Chl_A21_00741" 1381 "s59E5H2" 0 "hypothetical protein"
"Chl_A21_00741" 1381 "s63E6H2" 0 "hypothetical protein"
"Chl_A21_00742" 1382 "s59E13H2" 0 "Primosomal protein N'"
"Chl_A21_00742" 1382 "s59E25H2" 0 "Primosomal protein N'"
"Chl_A21_00742" 1382 "s59E5H2" 0 "Primosomal protein N'"
"Chl_A21_00742" 1382 "s63E6H2" 0 "Primosomal protein N'"
"Chl_A21_00747" 1387 "s59E13H2" 0 "putative ATP-dependent helicase DinG"
"Chl_A21_00747" 1387 "s59E25H2" 0 "putative ATP-dependent helicase DinG"
"Chl_A21_00747" 1387 "s59E5H2" 0 "putative ATP-dependent helicase DinG"
"Chl_A21_00747" 1387 "s63E6H2" 0 "putative ATP-dependent helicase DinG"
"Chl_A21_00748" 1388 "s59E13H2" 0 "Elongation factor P"
"Chl_A21_00748" 1388 "s59E25H2" 0 "Elongation factor P"
"Chl_A21_00748" 1388 "s59E5H2" 0 "Elongation factor P"
"Chl_A21_00748" 1388 "s63E6H2" 0 "Elongation factor P"
"Chl_A21_00749" 1389 "s59E13H2" 0 "DNA recombination protein RmuC"
"Chl_A21_00749" 1389 "s59E25H2" 0 "DNA recombination protein RmuC"
"Chl_A21_00749" 1389 "s59E5H2" 0 "DNA recombination protein RmuC"
"Chl_A21_00749" 1389 "s63E6H2" 0 "DNA recombination protein RmuC"
"Chl_A21_00750" 1390 "s59E13H2" 0 "hypothetical protein"
"Chl_A21_00750" 1390 "s59E25H2" 0 "hypothetical protein"
"Chl_A21_00750" 1390 "s59E5H2" 0 "hypothetical protein"
"Chl_A21_00750" 1390 "s63E6H2" 0 "hypothetical protein"
"Chl_A21_00751" 1391 "s59E13H2" 0 "Oxygen-independent coproporphyrinogen-III oxidase 1"

"Chl_A21_00751" 1391 "s59E25H2" 0 "Oxygen-independent coproporphyrinogen-III oxidase 1"
"Chl_A21_00751" 1391 "s59E5H2" 0 "Oxygen-independent coproporphyrinogen-III oxidase 1"
"Chl_A21_00751" 1391 "s63E6H2" 0 "Oxygen-independent coproporphyrinogen-III oxidase 1"
"Chl_A21_00752" 1392 "s59E13H2" 0 "hypothetical protein"
"Chl_A21_00752" 1392 "s59E25H2" 0 "hypothetical protein"
"Chl_A21_00752" 1392 "s59E5H2" 0 "hypothetical protein"
"Chl_A21_00752" 1392 "s63E6H2" 0 "hypothetical protein"
"Chl_A21_00754" 1394 "s59E13H2" 0 "Retroviral aspartyl protease"
"Chl_A21_00754" 1394 "s59E25H2" 0 "Retroviral aspartyl protease"
"Chl_A21_00754" 1394 "s59E5H2" 0 "Retroviral aspartyl protease"
"Chl_A21_00754" 1394 "s63E6H2" 0 "Retroviral aspartyl protease"
"Chl_A21_00755" 1395 "s59E13H2" 0 "hypothetical protein"
"Chl_A21_00755" 1395 "s59E25H2" 0 "hypothetical protein"
"Chl_A21_00755" 1395 "s59E5H2" 0 "hypothetical protein"
"Chl_A21_00755" 1395 "s63E6H2" 0 "hypothetical protein"
"Chl_A21_00757" 1397 "s59E13H2" 0 "Cysteine-rich secretory protein family protein"
"Chl_A21_00757" 1397 "s59E25H2" 0 "Cysteine-rich secretory protein family protein"
"Chl_A21_00757" 1397 "s59E5H2" 0 "Cysteine-rich secretory protein family protein"
"Chl_A21_00757" 1397 "s63E6H2" 0 "Cysteine-rich secretory protein family protein"
"Chl_A21_00758" 1398 "s59E13H2" 0 "Sortase family protein"
"Chl_A21_00758" 1398 "s59E25H2" 0 "Sortase family protein"
"Chl_A21_00758" 1398 "s59E5H2" 0 "Sortase family protein"
"Chl_A21_00758" 1398 "s63E6H2" 0 "Sortase family protein"
"Chl_A21_00759" 1399 "s59E13H2" 0 "Disulfide bond formation protein D precursor"
"Chl_A21_00759" 1399 "s59E25H2" 0 "Disulfide bond formation protein D precursor"
"Chl_A21_00759" 1399 "s59E5H2" 0 "Disulfide bond formation protein D precursor"
"Chl_A21_00759" 1399 "s63E6H2" 0 "Disulfide bond formation protein D precursor"
"Chl_A21_00760" 1400 "s59E13H2" 0 "hypothetical protein"
"Chl_A21_00760" 1400 "s59E25H2" 0 "hypothetical protein"
"Chl_A21_00760" 1400 "s59E5H2" 0 "hypothetical protein"
"Chl_A21_00760" 1400 "s63E6H2" 0 "hypothetical protein"
"Chl_A21_00762" 1402 "s59E13H2" 0 "Sensory transduction protein regX3"
"Chl_A21_00762" 1402 "s59E25H2" 0 "Sensory transduction protein regX3"
"Chl_A21_00762" 1402 "s59E5H2" 0 "Sensory transduction protein regX3"
"Chl_A21_00762" 1402 "s63E6H2" 0 "Sensory transduction protein regX3"
"Chl_A21_00764" 1404 "s59E13H2" 0 "hypothetical protein"
"Chl_A21_00764" 1404 "s59E25H2" 0 "hypothetical protein"
"Chl_A21_00764" 1404 "s59E5H2" 0 "hypothetical protein"
"Chl_A21_00764" 1404 "s63E6H2" 0 "hypothetical protein"
"Chl_A21_00770" 1410 "s59E13H2" 0 "putative aldolase LsrF"
"Chl_A21_00770" 1410 "s59E25H2" 0 "putative aldolase LsrF"
"Chl_A21_00770" 1410 "s59E5H2" 0 "putative aldolase LsrF"
"Chl_A21_00770" 1410 "s63E6H2" 0 "putative aldolase LsrF"
"Chl_A21_00771" 1411 "s59E13H2" 0 "putative NAD-dependent oxidoreductase"
"Chl_A21_00771" 1411 "s59E25H2" 0 "putative NAD-dependent oxidoreductase"
"Chl_A21_00771" 1411 "s59E5H2" 0 "putative NAD-dependent oxidoreductase"
"Chl_A21_00771" 1411 "s63E6H2" 0 "putative NAD-dependent oxidoreductase"

"Chl_A21_00773" 1413 "s59E13H2" 0 "Xylose import ATP-binding protein XylG"
"Chl_A21_00773" 1413 "s59E25H2" 0 "Xylose import ATP-binding protein XylG"
"Chl_A21_00773" 1413 "s59E5H2" 0 "Xylose import ATP-binding protein XylG"
"Chl_A21_00773" 1413 "s63E6H2" 0 "Xylose import ATP-binding protein XylG"
"Chl_A21_00774" 1414 "s59E13H2" 0 "Periplasmic binding proteins and sugar binding domain of LacI family protein"
"Chl_A21_00774" 1414 "s59E25H2" 0 "Periplasmic binding proteins and sugar binding domain of LacI family protein"
"Chl_A21_00774" 1414 "s59E5H2" 0 "Periplasmic binding proteins and sugar binding domain of LacI family protein"
"Chl_A21_00774" 1414 "s63E6H2" 0 "Periplasmic binding proteins and sugar binding domain of LacI family protein"
"Chl_A21_00775" 1415 "s59E13H2" 0 "Catabolite control protein A"
"Chl_A21_00775" 1415 "s59E25H2" 0 "Catabolite control protein A"
"Chl_A21_00775" 1415 "s59E5H2" 0 "Catabolite control protein A"
"Chl_A21_00775" 1415 "s63E6H2" 0 "Catabolite control protein A"
"Chl_A21_00776" 1416 "s59E13H2" 0 "Competence protein ComM"
"Chl_A21_00776" 1416 "s59E25H2" 0 "Competence protein ComM"
"Chl_A21_00776" 1416 "s59E5H2" 0 "Competence protein ComM"
"Chl_A21_00776" 1416 "s63E6H2" 0 "Competence protein ComM"
"Chl_A21_00777" 1417 "s59E13H2" 0 "Copper-sensing transcriptional repressor CsoR"
"Chl_A21_00777" 1417 "s59E25H2" 0 "Copper-sensing transcriptional repressor CsoR"
"Chl_A21_00777" 1417 "s59E5H2" 0 "Copper-sensing transcriptional repressor CsoR"
"Chl_A21_00777" 1417 "s63E6H2" 0 "Copper-sensing transcriptional repressor CsoR"
"Chl_A21_00778" 1418 "s59E13H2" 0 "Sensor histidine kinase YycG"
"Chl_A21_00778" 1418 "s59E25H2" 0 "Sensor histidine kinase YycG"
"Chl_A21_00778" 1418 "s59E5H2" 0 "Sensor histidine kinase YycG"
"Chl_A21_00778" 1418 "s63E6H2" 0 "Sensor histidine kinase YycG"
"Chl_A21_00779" 1419 "s59E13H2" 0 "Transcriptional regulatory protein YycF"
"Chl_A21_00779" 1419 "s59E25H2" 0 "Transcriptional regulatory protein YycF"
"Chl_A21_00779" 1419 "s59E5H2" 0 "Transcriptional regulatory protein YycF"
"Chl_A21_00779" 1419 "s63E6H2" 0 "Transcriptional regulatory protein YycF"
"Chl_A21_00781" 1421 "s59E13H2" 0 "Glutamate synthase [NADPH] large chain precursor"
"Chl_A21_00781" 1421 "s59E25H2" 0 "Glutamate synthase [NADPH] large chain precursor"
"Chl_A21_00781" 1421 "s59E5H2" 0 "Glutamate synthase [NADPH] large chain precursor"
"Chl_A21_00781" 1421 "s63E6H2" 0 "Glutamate synthase [NADPH] large chain precursor"
"Chl_A21_00782" 1422 "s59E13H2" 0 "Molybdenum-containing formylmethanofuran dehydrogenase 1 subunit C"
"Chl_A21_00782" 1422 "s59E25H2" 0 "Molybdenum-containing formylmethanofuran dehydrogenase 1 subunit C"
"Chl_A21_00782" 1422 "s59E5H2" 0 "Molybdenum-containing formylmethanofuran dehydrogenase 1 subunit C"
"Chl_A21_00782" 1422 "s63E6H2" 0 "Molybdenum-containing formylmethanofuran dehydrogenase 1 subunit C"
"Chl_A21_00783" 1423 "s59E13H2" 0 "Coenzyme F420 hydrogenase subunit beta"
"Chl_A21_00783" 1423 "s59E25H2" 0 "Coenzyme F420 hydrogenase subunit beta"
"Chl_A21_00783" 1423 "s59E5H2" 0 "Coenzyme F420 hydrogenase subunit beta"
"Chl_A21_00783" 1423 "s63E6H2" 0 "Coenzyme F420 hydrogenase subunit beta"
"Chl_A21_00784" 1424 "s59E13H2" 0 "Heterodisulfide reductase subunit A-like protein"
"Chl_A21_00784" 1424 "s59E25H2" 0 "Heterodisulfide reductase subunit A-like protein"
"Chl_A21_00784" 1424 "s59E5H2" 0 "Heterodisulfide reductase subunit A-like protein"
"Chl_A21_00784" 1424 "s63E6H2" 0 "Heterodisulfide reductase subunit A-like protein"
"Chl_A21_00785" 1425 "s59E13H2" 0 "NADP-reducing hydrogenase subunit HndC"
"Chl_A21_00785" 1425 "s59E25H2" 0 "NADP-reducing hydrogenase subunit HndC"
"Chl_A21_00785" 1425 "s59E5H2" 0 "NADP-reducing hydrogenase subunit HndC"

"Chl_A21_00785" 1425 "s63E6H2" 0 "NADP-reducing hydrogenase subunit HndC"
"Chl_A21_00786" 1426 "s59E13H2" 0 "NADP-reducing hydrogenase subunit HndC"
"Chl_A21_00786" 1426 "s59E25H2" 0 "NADP-reducing hydrogenase subunit HndC"
"Chl_A21_00786" 1426 "s59E5H2" 0 "NADP-reducing hydrogenase subunit HndC"
"Chl_A21_00786" 1426 "s63E6H2" 0 "NADP-reducing hydrogenase subunit HndC"
"Chl_A21_00787" 1427 "s59E13H2" 0 "NADP-reducing hydrogenase subunit HndA"
"Chl_A21_00787" 1427 "s59E25H2" 0 "NADP-reducing hydrogenase subunit HndA"
"Chl_A21_00787" 1427 "s59E5H2" 0 "NADP-reducing hydrogenase subunit HndA"
"Chl_A21_00787" 1427 "s63E6H2" 0 "NADP-reducing hydrogenase subunit HndA"
"Chl_A21_00788" 1428 "s59E13H2" 0 "Competence protein ComM"
"Chl_A21_00788" 1428 "s59E25H2" 0 "Competence protein ComM"
"Chl_A21_00788" 1428 "s59E5H2" 0 "Competence protein ComM"
"Chl_A21_00788" 1428 "s63E6H2" 0 "Competence protein ComM"
"Chl_A21_00789" 1429 "s59E13H2" 0 "D-alanine--D-alanine ligase B"
"Chl_A21_00789" 1429 "s59E25H2" 0 "D-alanine--D-alanine ligase B"
"Chl_A21_00789" 1429 "s59E5H2" 0 "D-alanine--D-alanine ligase B"
"Chl_A21_00789" 1429 "s63E6H2" 0 "D-alanine--D-alanine ligase B"
"Chl_A21_00790" 1430 "s59E13H2" 0 "Sporulation and spore germination"
"Chl_A21_00790" 1430 "s59E25H2" 0 "Sporulation and spore germination"
"Chl_A21_00790" 1430 "s59E5H2" 0 "Sporulation and spore germination"
"Chl_A21_00790" 1430 "s63E6H2" 0 "Sporulation and spore germination"
"Chl_A21_00791" 1431 "s59E13H2" 0 "Tungsten-containing formaldehyde ferredoxin oxidoreductase"
"Chl_A21_00791" 1431 "s59E25H2" 0 "Tungsten-containing formaldehyde ferredoxin oxidoreductase"
"Chl_A21_00791" 1431 "s59E5H2" 0 "Tungsten-containing formaldehyde ferredoxin oxidoreductase"
"Chl_A21_00791" 1431 "s63E6H2" 0 "Tungsten-containing formaldehyde ferredoxin oxidoreductase"
"Chl_A21_00792" 1432 "s59E13H2" 0 "Tungsten-containing aldehyde ferredoxin oxidoreductase"
"Chl_A21_00792" 1432 "s59E25H2" 0 "Tungsten-containing aldehyde ferredoxin oxidoreductase"
"Chl_A21_00792" 1432 "s59E5H2" 0 "Tungsten-containing aldehyde ferredoxin oxidoreductase"
"Chl_A21_00792" 1432 "s63E6H2" 0 "Tungsten-containing aldehyde ferredoxin oxidoreductase"
"Chl_A21_00793" 1433 "s59E13H2" 0 "RNA polymerase sigma factor SigA"
"Chl_A21_00793" 1433 "s59E25H2" 0 "RNA polymerase sigma factor SigA"
"Chl_A21_00793" 1433 "s59E5H2" 0 "RNA polymerase sigma factor SigA"
"Chl_A21_00793" 1433 "s63E6H2" 0 "RNA polymerase sigma factor SigA"
"Chl_A21_00794" 1434 "s59E13H2" 0 "Alpha/beta hydrolase family protein"
"Chl_A21_00794" 1434 "s59E25H2" 0 "Alpha/beta hydrolase family protein"
"Chl_A21_00794" 1434 "s59E5H2" 0 "Alpha/beta hydrolase family protein"
"Chl_A21_00794" 1434 "s63E6H2" 0 "Alpha/beta hydrolase family protein"
"Chl_A21_00795" 1435 "s59E13H2" 0 "Undecaprenyl-diphosphatase BcrC"
"Chl_A21_00795" 1435 "s59E25H2" 0 "Undecaprenyl-diphosphatase BcrC"
"Chl_A21_00795" 1435 "s59E5H2" 0 "Undecaprenyl-diphosphatase BcrC"
"Chl_A21_00795" 1435 "s63E6H2" 0 "Undecaprenyl-diphosphatase BcrC"
"Chl_A21_00796" 1436 "s59E13H2" 0 "Poly(hydroxyalcanoate) granule associated protein (phasin)"
"Chl_A21_00796" 1436 "s59E25H2" 0 "Poly(hydroxyalcanoate) granule associated protein (phasin)"
"Chl_A21_00796" 1436 "s59E5H2" 0 "Poly(hydroxyalcanoate) granule associated protein (phasin)"
"Chl_A21_00796" 1436 "s63E6H2" 0 "Poly(hydroxyalcanoate) granule associated protein (phasin)"
"Chl_A21_00797" 1437 "s59E13H2" 0 "PHB/PHA accumulation regulator DNA-binding domain protein"
"Chl_A21_00797" 1437 "s59E25H2" 0 "PHB/PHA accumulation regulator DNA-binding domain protein"

"Chl_A21_00797" 1437 "s59E5H2" 0 "PHB/PHA accumulation regulator DNA-binding domain protein"
"Chl_A21_00797" 1437 "s63E6H2" 0 "PHB/PHA accumulation regulator DNA-binding domain protein"
"Chl_A21_00798" 1438 "s59E13H2" 0 "putative HTH-type transcriptional regulator YfiR"
"Chl_A21_00798" 1438 "s59E25H2" 0 "putative HTH-type transcriptional regulator YfiR"
"Chl_A21_00798" 1438 "s59E5H2" 0 "putative HTH-type transcriptional regulator YfiR"
"Chl_A21_00798" 1438 "s63E6H2" 0 "putative HTH-type transcriptional regulator YfiR"
"Chl_A21_00799" 1439 "s59E13H2" 0 "hypothetical protein"
"Chl_A21_00799" 1439 "s59E25H2" 0 "hypothetical protein"
"Chl_A21_00799" 1439 "s59E5H2" 0 "hypothetical protein"
"Chl_A21_00799" 1439 "s63E6H2" 0 "hypothetical protein"
"Chl_A21_00800" 1440 "s59E13H2" 0 "26 kDa periplasmic immunogenic protein precursor"
"Chl_A21_00800" 1440 "s59E25H2" 0 "26 kDa periplasmic immunogenic protein precursor"
"Chl_A21_00800" 1440 "s59E5H2" 0 "26 kDa periplasmic immunogenic protein precursor"
"Chl_A21_00800" 1440 "s63E6H2" 0 "26 kDa periplasmic immunogenic protein precursor"
"Chl_A21_00801" 1441 "s59E13H2" 0 "PHP domain protein"
"Chl_A21_00801" 1441 "s59E25H2" 0 "PHP domain protein"
"Chl_A21_00801" 1441 "s59E5H2" 0 "PHP domain protein"
"Chl_A21_00801" 1441 "s63E6H2" 0 "PHP domain protein"
"Chl_A21_00802" 1442 "s59E13H2" 0 "putative nucleotide-binding protein containing TIR-like domain protein"
"Chl_A21_00802" 1442 "s59E25H2" 0 "putative nucleotide-binding protein containing TIR-like domain protein"
"Chl_A21_00802" 1442 "s59E5H2" 0 "putative nucleotide-binding protein containing TIR-like domain protein"
"Chl_A21_00802" 1442 "s63E6H2" 0 "putative nucleotide-binding protein containing TIR-like domain protein"
"Chl_A21_00803" 1443 "s59E13H2" 0 "UvrABC system protein B"
"Chl_A21_00803" 1443 "s59E25H2" 0 "UvrABC system protein B"
"Chl_A21_00803" 1443 "s59E5H2" 0 "UvrABC system protein B"
"Chl_A21_00803" 1443 "s63E6H2" 0 "UvrABC system protein B"
"Chl_A21_00804" 1444 "s59E13H2" 0 "hypothetical protein"
"Chl_A21_00804" 1444 "s59E25H2" 0 "hypothetical protein"
"Chl_A21_00804" 1444 "s59E5H2" 0 "hypothetical protein"
"Chl_A21_00804" 1444 "s63E6H2" 0 "hypothetical protein"
"Chl_A21_00805" 1445 "s59E13H2" 0 "hypothetical protein"
"Chl_A21_00805" 1445 "s59E25H2" 0 "hypothetical protein"
"Chl_A21_00805" 1445 "s59E5H2" 0 "hypothetical protein"
"Chl_A21_00805" 1445 "s63E6H2" 0 "hypothetical protein"
"Chl_A21_00806" 1446 "s59E13H2" 0 "hypothetical protein"
"Chl_A21_00806" 1446 "s59E25H2" 0 "hypothetical protein"
"Chl_A21_00806" 1446 "s59E5H2" 0 "hypothetical protein"
"Chl_A21_00806" 1446 "s63E6H2" 0 "hypothetical protein"
"Chl_A21_00807" 1447 "s59E13H2" 0 "UvrABC system protein A"
"Chl_A21_00807" 1447 "s59E25H2" 0 "UvrABC system protein A"
"Chl_A21_00807" 1447 "s59E5H2" 0 "UvrABC system protein A"
"Chl_A21_00807" 1447 "s63E6H2" 0 "UvrABC system protein A"
"Chl_A21_00808" 1448 "s59E13H2" 0 "Prenyltransferase and squalene oxidase repeat protein"
"Chl_A21_00808" 1448 "s59E25H2" 0 "Prenyltransferase and squalene oxidase repeat protein"
"Chl_A21_00808" 1448 "s59E5H2" 0 "Prenyltransferase and squalene oxidase repeat protein"
"Chl_A21_00808" 1448 "s63E6H2" 0 "Prenyltransferase and squalene oxidase repeat protein"
"Chl_A21_00809" 1449 "s59E13H2" 0 "8-oxo-dGTP diphosphatase"

"Chl_A21_00809" 1449 "s59E25H2" 0 "8-oxo-dGTP diphosphatase"
"Chl_A21_00809" 1449 "s59E5H2" 0 "8-oxo-dGTP diphosphatase"
"Chl_A21_00809" 1449 "s63E6H2" 0 "8-oxo-dGTP diphosphatase"
"Chl_A21_00810" 1450 "s59E13H2" 0 "SEC-C motif protein"
"Chl_A21_00810" 1450 "s59E25H2" 0 "SEC-C motif protein"
"Chl_A21_00810" 1450 "s59E5H2" 0 "SEC-C motif protein"
"Chl_A21_00810" 1450 "s63E6H2" 0 "SEC-C motif protein"
"Chl_A21_00811" 1451 "s59E13H2" 0 "(Dimethylallyl)adenosine tRNA methylthiotransferase MiaB"
"Chl_A21_00811" 1451 "s59E25H2" 0 "(Dimethylallyl)adenosine tRNA methylthiotransferase MiaB"
"Chl_A21_00811" 1451 "s59E5H2" 0 "(Dimethylallyl)adenosine tRNA methylthiotransferase MiaB"
"Chl_A21_00811" 1451 "s63E6H2" 0 "(Dimethylallyl)adenosine tRNA methylthiotransferase MiaB"
"Chl_A21_00812" 1452 "s59E13H2" 0 "Acyl-coenzyme A:6-aminopenicillanic acid acyl-transferase"
"Chl_A21_00812" 1452 "s59E25H2" 0 "Acyl-coenzyme A:6-aminopenicillanic acid acyl-transferase"
"Chl_A21_00812" 1452 "s59E5H2" 0 "Acyl-coenzyme A:6-aminopenicillanic acid acyl-transferase"
"Chl_A21_00812" 1452 "s63E6H2" 0 "Acyl-coenzyme A:6-aminopenicillanic acid acyl-transferase"
"Chl_A21_00813" 1453 "s59E13H2" 0 "hypothetical protein"
"Chl_A21_00813" 1453 "s59E25H2" 0 "hypothetical protein"
"Chl_A21_00813" 1453 "s59E5H2" 0 "hypothetical protein"
"Chl_A21_00813" 1453 "s63E6H2" 0 "hypothetical protein"
"Chl_A21_00814" 1454 "s59E13H2" 0 "L-allo-threonine aldolase"
"Chl_A21_00814" 1454 "s59E25H2" 0 "L-allo-threonine aldolase"
"Chl_A21_00814" 1454 "s59E5H2" 0 "L-allo-threonine aldolase"
"Chl_A21_00814" 1454 "s63E6H2" 0 "L-allo-threonine aldolase"
"Chl_A21_00815" 1455 "s59E13H2" 0 "Ribosomal large subunit pseudouridine synthase D"
"Chl_A21_00815" 1455 "s59E25H2" 0 "Ribosomal large subunit pseudouridine synthase D"
"Chl_A21_00815" 1455 "s59E5H2" 0 "Ribosomal large subunit pseudouridine synthase D"
"Chl_A21_00815" 1455 "s63E6H2" 0 "Ribosomal large subunit pseudouridine synthase D"
"Chl_A21_00816" 1456 "s59E13H2" 0 "Lipoprotein signal peptidase"
"Chl_A21_00816" 1456 "s59E25H2" 0 "Lipoprotein signal peptidase"
"Chl_A21_00816" 1456 "s59E5H2" 0 "Lipoprotein signal peptidase"
"Chl_A21_00816" 1456 "s63E6H2" 0 "Lipoprotein signal peptidase"
"Chl_A21_00817" 1457 "s59E13H2" 0 "General stress protein 16O"
"Chl_A21_00817" 1457 "s59E25H2" 0 "General stress protein 16O"
"Chl_A21_00817" 1457 "s59E5H2" 0 "General stress protein 16O"
"Chl_A21_00817" 1457 "s63E6H2" 0 "General stress protein 16O"
"Chl_A21_00818" 1458 "s59E13H2" 0 "Undecaprenyl-phosphate mannosyltransferase"
"Chl_A21_00818" 1458 "s59E25H2" 0 "Undecaprenyl-phosphate mannosyltransferase"
"Chl_A21_00818" 1458 "s59E5H2" 0 "Undecaprenyl-phosphate mannosyltransferase"
"Chl_A21_00818" 1458 "s63E6H2" 0 "Undecaprenyl-phosphate mannosyltransferase"
"Chl_A21_00819" 1459 "s59E13H2" 0 "Polysaccharide biosynthesis protein"
"Chl_A21_00819" 1459 "s59E25H2" 0 "Polysaccharide biosynthesis protein"
"Chl_A21_00819" 1459 "s59E5H2" 0 "Polysaccharide biosynthesis protein"
"Chl_A21_00819" 1459 "s63E6H2" 0 "Polysaccharide biosynthesis protein"
"Chl_A21_00820" 1460 "s59E13H2" 0 "Transposase IS200 like protein"
"Chl_A21_00820" 1460 "s59E25H2" 0 "Transposase IS200 like protein"
"Chl_A21_00820" 1460 "s59E5H2" 0 "Transposase IS200 like protein"
"Chl_A21_00820" 1460 "s63E6H2" 0 "Transposase IS200 like protein"

"Chl_A21_00824" 1464 "s59E13H2" 0 "hypothetical protein"
"Chl_A21_00824" 1464 "s59E25H2" 0 "hypothetical protein"
"Chl_A21_00824" 1464 "s59E5H2" 0 "hypothetical protein"
"Chl_A21_00824" 1464 "s63E6H2" 0 "hypothetical protein"
"Chl_A21_00825" 1465 "s59E13H2" 0 "L-asparaginase II"
"Chl_A21_00825" 1465 "s59E25H2" 0 "L-asparaginase II"
"Chl_A21_00825" 1465 "s59E5H2" 0 "L-asparaginase II"
"Chl_A21_00825" 1465 "s63E6H2" 0 "L-asparaginase II"
"Chl_A21_00826" 1466 "s59E13H2" 0 "Fosmidomycin resistance protein"
"Chl_A21_00826" 1466 "s59E25H2" 0 "Fosmidomycin resistance protein"
"Chl_A21_00826" 1466 "s59E5H2" 0 "Fosmidomycin resistance protein"
"Chl_A21_00826" 1466 "s63E6H2" 0 "Fosmidomycin resistance protein"
"Chl_A21_00827" 1467 "s59E13H2" 0 "Sulfurtransferase TusA"
"Chl_A21_00827" 1467 "s59E25H2" 0 "Sulfurtransferase TusA"
"Chl_A21_00827" 1467 "s59E5H2" 0 "Sulfurtransferase TusA"
"Chl_A21_00827" 1467 "s63E6H2" 0 "Sulfurtransferase TusA"
"Chl_A21_00828" 1468 "s59E13H2" 0 "putative cysteine desulfurase 2"
"Chl_A21_00828" 1468 "s59E25H2" 0 "putative cysteine desulfurase 2"
"Chl_A21_00828" 1468 "s59E5H2" 0 "putative cysteine desulfurase 2"
"Chl_A21_00828" 1468 "s63E6H2" 0 "putative cysteine desulfurase 2"
"Chl_A21_00829" 1469 "s59E13H2" 0 "Nucleoside 2-deoxyribosyltransferase"
"Chl_A21_00829" 1469 "s59E25H2" 0 "Nucleoside 2-deoxyribosyltransferase"
"Chl_A21_00829" 1469 "s59E5H2" 0 "Nucleoside 2-deoxyribosyltransferase"
"Chl_A21_00829" 1469 "s63E6H2" 0 "Nucleoside 2-deoxyribosyltransferase"
"Chl_A21_00830" 1470 "s59E13H2" 0 "Acyl-coenzyme A:6-aminopenicillanic acid acyl-transferase"
"Chl_A21_00830" 1470 "s59E25H2" 0 "Acyl-coenzyme A:6-aminopenicillanic acid acyl-transferase"
"Chl_A21_00830" 1470 "s59E5H2" 0 "Acyl-coenzyme A:6-aminopenicillanic acid acyl-transferase"
"Chl_A21_00830" 1470 "s63E6H2" 0 "Acyl-coenzyme A:6-aminopenicillanic acid acyl-transferase"
"Chl_A21_00831" 1471 "s59E13H2" 0 "hypothetical protein"
"Chl_A21_00831" 1471 "s59E25H2" 0 "hypothetical protein"
"Chl_A21_00831" 1471 "s59E5H2" 0 "hypothetical protein"
"Chl_A21_00831" 1471 "s63E6H2" 0 "hypothetical protein"
"Chl_A21_00832" 1472 "s59E13H2" 0 "dihydropteridine reductase"
"Chl_A21_00832" 1472 "s59E25H2" 0 "dihydropteridine reductase"
"Chl_A21_00832" 1472 "s59E5H2" 0 "dihydropteridine reductase"
"Chl_A21_00832" 1472 "s63E6H2" 0 "dihydropteridine reductase"
"Chl_A21_00833" 1473 "s59E13H2" 0 "Demethylmenaquinone methyltransferase"
"Chl_A21_00833" 1473 "s59E25H2" 0 "Demethylmenaquinone methyltransferase"
"Chl_A21_00833" 1473 "s59E5H2" 0 "Demethylmenaquinone methyltransferase"
"Chl_A21_00833" 1473 "s63E6H2" 0 "Demethylmenaquinone methyltransferase"
"Chl_A21_00834" 1474 "s59E13H2" 0 "tRNA-specific 2-thiouridylase MnmA"
"Chl_A21_00834" 1474 "s59E25H2" 0 "tRNA-specific 2-thiouridylase MnmA"
"Chl_A21_00834" 1474 "s59E5H2" 0 "tRNA-specific 2-thiouridylase MnmA"
"Chl_A21_00834" 1474 "s63E6H2" 0 "tRNA-specific 2-thiouridylase MnmA"
"Chl_A21_00835" 1475 "s59E13H2" 0 "hypothetical protein"
"Chl_A21_00835" 1475 "s59E25H2" 0 "hypothetical protein"
"Chl_A21_00835" 1475 "s59E5H2" 0 "hypothetical protein"

"Chl_A21_00835" 1475 "s63E6H2" 0 "hypothetical protein"
"Chl_A21_00836" 1476 "s59E13H2" 0 "hypothetical protein"
"Chl_A21_00836" 1476 "s59E25H2" 0 "hypothetical protein"
"Chl_A21_00836" 1476 "s59E5H2" 0 "hypothetical protein"
"Chl_A21_00836" 1476 "s63E6H2" 0 "hypothetical protein"
"Chl_A21_00837" 1477 "s59E13H2" 0 "YtxH-like protein"
"Chl_A21_00837" 1477 "s59E25H2" 0 "YtxH-like protein"
"Chl_A21_00837" 1477 "s59E5H2" 0 "YtxH-like protein"
"Chl_A21_00837" 1477 "s63E6H2" 0 "YtxH-like protein"
"Chl_A21_00838" 1478 "s59E13H2" 0 "hypothetical protein"
"Chl_A21_00838" 1478 "s59E25H2" 0 "hypothetical protein"
"Chl_A21_00838" 1478 "s59E5H2" 0 "hypothetical protein"
"Chl_A21_00838" 1478 "s63E6H2" 0 "hypothetical protein"
"Chl_A21_00839" 1479 "s59E13H2" 0 "hypothetical protein"
"Chl_A21_00839" 1479 "s59E25H2" 0 "hypothetical protein"
"Chl_A21_00839" 1479 "s59E5H2" 0 "hypothetical protein"
"Chl_A21_00839" 1479 "s63E6H2" 0 "hypothetical protein"
"Chl_A21_00840" 1480 "s59E13H2" 0 "hypothetical protein"
"Chl_A21_00840" 1480 "s59E25H2" 0 "hypothetical protein"
"Chl_A21_00840" 1480 "s59E5H2" 0 "hypothetical protein"
"Chl_A21_00840" 1480 "s63E6H2" 0 "hypothetical protein"
"Chl_A21_00841" 1481 "s59E13H2" 0 "Bifunctional NAD(P)H-hydrate repair enzyme Nnr"
"Chl_A21_00841" 1481 "s59E25H2" 0 "Bifunctional NAD(P)H-hydrate repair enzyme Nnr"
"Chl_A21_00841" 1481 "s59E5H2" 0 "Bifunctional NAD(P)H-hydrate repair enzyme Nnr"
"Chl_A21_00841" 1481 "s63E6H2" 0 "Bifunctional NAD(P)H-hydrate repair enzyme Nnr"
"Chl_A21_00845" 1485 "s59E13H2" 0 "hypothetical protein"
"Chl_A21_00845" 1485 "s59E25H2" 0 "hypothetical protein"
"Chl_A21_00845" 1485 "s59E5H2" 0 "hypothetical protein"
"Chl_A21_00845" 1485 "s63E6H2" 0 "hypothetical protein"
"Chl_A21_00846" 1486 "s59E13H2" 0 "hypothetical protein"
"Chl_A21_00846" 1486 "s59E25H2" 0 "hypothetical protein"
"Chl_A21_00846" 1486 "s59E5H2" 0 "hypothetical protein"
"Chl_A21_00846" 1486 "s63E6H2" 0 "hypothetical protein"
"Chl_A21_00847" 1487 "s59E13H2" 0 "ComEC family competence protein"
"Chl_A21_00847" 1487 "s59E25H2" 0 "ComEC family competence protein"
"Chl_A21_00847" 1487 "s59E5H2" 0 "ComEC family competence protein"
"Chl_A21_00847" 1487 "s63E6H2" 0 "ComEC family competence protein"
"Chl_A21_00848" 1488 "s59E13H2" 0 "Putative nickel-responsive regulator"
"Chl_A21_00848" 1488 "s59E25H2" 0 "Putative nickel-responsive regulator"
"Chl_A21_00848" 1488 "s59E5H2" 0 "Putative nickel-responsive regulator"
"Chl_A21_00848" 1488 "s63E6H2" 0 "Putative nickel-responsive regulator"
"Chl_A21_00849" 1489 "s59E13H2" 0 "mRNA interferase EndoA"
"Chl_A21_00849" 1489 "s59E25H2" 0 "mRNA interferase EndoA"
"Chl_A21_00849" 1489 "s59E5H2" 0 "mRNA interferase EndoA"
"Chl_A21_00849" 1489 "s63E6H2" 0 "mRNA interferase EndoA"
"Chl_A21_00850" 1490 "s59E13H2" 0 "NYN domain protein"
"Chl_A21_00850" 1490 "s59E25H2" 0 "NYN domain protein"

"Chl_A21_00850" 1490 "s59E5H2" 0 "NYN domain protein"
"Chl_A21_00850" 1490 "s63E6H2" 0 "NYN domain protein"
"Chl_A21_00851" 1491 "s59E13H2" 0 "hypothetical protein"
"Chl_A21_00851" 1491 "s59E25H2" 0 "hypothetical protein"
"Chl_A21_00851" 1491 "s59E5H2" 0 "hypothetical protein"
"Chl_A21_00851" 1491 "s63E6H2" 0 "hypothetical protein"
"Chl_A21_00852" 1492 "s59E13H2" 0 "Sporulation initiation phosphotransferase F"
"Chl_A21_00852" 1492 "s59E25H2" 0 "Sporulation initiation phosphotransferase F"
"Chl_A21_00852" 1492 "s59E5H2" 0 "Sporulation initiation phosphotransferase F"
"Chl_A21_00852" 1492 "s63E6H2" 0 "Sporulation initiation phosphotransferase F"
"Chl_A21_00853" 1493 "s59E13H2" 0 "hypothetical protein"
"Chl_A21_00853" 1493 "s59E25H2" 0 "hypothetical protein"
"Chl_A21_00853" 1493 "s59E5H2" 0 "hypothetical protein"
"Chl_A21_00853" 1493 "s63E6H2" 0 "hypothetical protein"
"Chl_A21_00854" 1494 "s59E13H2" 0 "30S ribosomal protein S2"
"Chl_A21_00854" 1494 "s59E25H2" 0 "30S ribosomal protein S2"
"Chl_A21_00854" 1494 "s59E5H2" 0 "30S ribosomal protein S2"
"Chl_A21_00854" 1494 "s63E6H2" 0 "30S ribosomal protein S2"
"Chl_A21_00855" 1495 "s59E13H2" 0 "Elongation factor Ts"
"Chl_A21_00855" 1495 "s59E25H2" 0 "Elongation factor Ts"
"Chl_A21_00855" 1495 "s59E5H2" 0 "Elongation factor Ts"
"Chl_A21_00855" 1495 "s63E6H2" 0 "Elongation factor Ts"
"Chl_A21_00856" 1496 "s59E13H2" 0 "Uridylate kinase"
"Chl_A21_00856" 1496 "s59E25H2" 0 "Uridylate kinase"
"Chl_A21_00856" 1496 "s59E5H2" 0 "Uridylate kinase"
"Chl_A21_00856" 1496 "s63E6H2" 0 "Uridylate kinase"
"Chl_A21_00857" 1497 "s59E13H2" 0 "Ribosome-recycling factor"
"Chl_A21_00857" 1497 "s59E25H2" 0 "Ribosome-recycling factor"
"Chl_A21_00857" 1497 "s59E5H2" 0 "Ribosome-recycling factor"
"Chl_A21_00857" 1497 "s63E6H2" 0 "Ribosome-recycling factor"
"Chl_A21_00858" 1498 "s59E13H2" 0 "Isoprenyl transferase"
"Chl_A21_00858" 1498 "s59E25H2" 0 "Isoprenyl transferase"
"Chl_A21_00858" 1498 "s59E5H2" 0 "Isoprenyl transferase"
"Chl_A21_00858" 1498 "s63E6H2" 0 "Isoprenyl transferase"
"Chl_A21_00859" 1499 "s59E13H2" 0 "Phosphatidate cytidylyltransferase"
"Chl_A21_00859" 1499 "s59E25H2" 0 "Phosphatidate cytidylyltransferase"
"Chl_A21_00859" 1499 "s59E5H2" 0 "Phosphatidate cytidylyltransferase"
"Chl_A21_00859" 1499 "s63E6H2" 0 "Phosphatidate cytidylyltransferase"
"Chl_A21_00860" 1500 "s59E13H2" 0 "hypothetical protein"
"Chl_A21_00860" 1500 "s59E25H2" 0 "hypothetical protein"
"Chl_A21_00860" 1500 "s59E5H2" 0 "hypothetical protein"
"Chl_A21_00860" 1500 "s63E6H2" 0 "hypothetical protein"
"Chl_A21_00861" 1501 "s59E13H2" 0 "O-acetyl-ADP-ribose deacetylase"
"Chl_A21_00861" 1501 "s59E25H2" 0 "O-acetyl-ADP-ribose deacetylase"
"Chl_A21_00861" 1501 "s59E5H2" 0 "O-acetyl-ADP-ribose deacetylase"
"Chl_A21_00861" 1501 "s63E6H2" 0 "O-acetyl-ADP-ribose deacetylase"
"Chl_A21_00862" 1502 "s59E13H2" 0 "Spore germination protein YaaH"

"Chl_A21_00862" 1502 "s59E25H2" 0 "Spore germination protein YaaH"
"Chl_A21_00862" 1502 "s59E5H2" 0 "Spore germination protein YaaH"
"Chl_A21_00862" 1502 "s63E6H2" 0 "Spore germination protein YaaH"
"Chl_A21_00863" 1503 "s59E13H2" 0 "Glycine cleavage system H protein"
"Chl_A21_00863" 1503 "s59E25H2" 0 "Glycine cleavage system H protein"
"Chl_A21_00863" 1503 "s59E5H2" 0 "Glycine cleavage system H protein"
"Chl_A21_00863" 1503 "s63E6H2" 0 "Glycine cleavage system H protein"
"Chl_A21_00864" 1504 "s59E13H2" 0 "Deoxyguanosinetriphosphate triphosphohydrolase"
"Chl_A21_00864" 1504 "s59E25H2" 0 "Deoxyguanosinetriphosphate triphosphohydrolase"
"Chl_A21_00864" 1504 "s59E5H2" 0 "Deoxyguanosinetriphosphate triphosphohydrolase"
"Chl_A21_00864" 1504 "s63E6H2" 0 "Deoxyguanosinetriphosphate triphosphohydrolase"
"Chl_A21_00865" 1505 "s59E13H2" 0 "hypothetical protein"
"Chl_A21_00865" 1505 "s59E25H2" 0 "hypothetical protein"
"Chl_A21_00865" 1505 "s59E5H2" 0 "hypothetical protein"
"Chl_A21_00865" 1505 "s63E6H2" 0 "hypothetical protein"
"Chl_A21_00866" 1506 "s59E13H2" 0 "putative glycine dehydrogenase (decarboxylating) subunit 1"
"Chl_A21_00866" 1506 "s59E25H2" 0 "putative glycine dehydrogenase (decarboxylating) subunit 1"
"Chl_A21_00866" 1506 "s59E5H2" 0 "putative glycine dehydrogenase (decarboxylating) subunit 1"
"Chl_A21_00866" 1506 "s63E6H2" 0 "putative glycine dehydrogenase (decarboxylating) subunit 1"
"Chl_A21_00867" 1507 "s59E13H2" 0 "Soluble hydrogenase 42 kDa subunit"
"Chl_A21_00867" 1507 "s59E25H2" 0 "Soluble hydrogenase 42 kDa subunit"
"Chl_A21_00867" 1507 "s59E5H2" 0 "Soluble hydrogenase 42 kDa subunit"
"Chl_A21_00867" 1507 "s63E6H2" 0 "Soluble hydrogenase 42 kDa subunit"
"Chl_A21_00868" 1508 "s59E13H2" 0 "putative glycine dehydrogenase (decarboxylating) subunit 2"
"Chl_A21_00868" 1508 "s59E25H2" 0 "putative glycine dehydrogenase (decarboxylating) subunit 2"
"Chl_A21_00868" 1508 "s59E5H2" 0 "putative glycine dehydrogenase (decarboxylating) subunit 2"
"Chl_A21_00868" 1508 "s63E6H2" 0 "putative glycine dehydrogenase (decarboxylating) subunit 2"
"Chl_A21_00869" 1509 "s59E13H2" 0 "ECF RNA polymerase sigma factor SigW"
"Chl_A21_00869" 1509 "s59E25H2" 0 "ECF RNA polymerase sigma factor SigW"
"Chl_A21_00869" 1509 "s59E5H2" 0 "ECF RNA polymerase sigma factor SigW"
"Chl_A21_00869" 1509 "s63E6H2" 0 "ECF RNA polymerase sigma factor SigW"
"Chl_A21_00871" 1511 "s59E13H2" 0 "Oligopeptide transport ATP-binding protein OppD"
"Chl_A21_00871" 1511 "s59E25H2" 0 "Oligopeptide transport ATP-binding protein OppD"
"Chl_A21_00871" 1511 "s59E5H2" 0 "Oligopeptide transport ATP-binding protein OppD"
"Chl_A21_00871" 1511 "s63E6H2" 0 "Oligopeptide transport ATP-binding protein OppD"
"Chl_A21_00872" 1512 "s59E13H2" 0 "Oligopeptide transport ATP-binding protein OppF"
"Chl_A21_00872" 1512 "s59E25H2" 0 "Oligopeptide transport ATP-binding protein OppF"
"Chl_A21_00872" 1512 "s59E5H2" 0 "Oligopeptide transport ATP-binding protein OppF"
"Chl_A21_00872" 1512 "s63E6H2" 0 "Oligopeptide transport ATP-binding protein OppF"
"Chl_A21_00873" 1513 "s59E13H2" 0 "Heme-binding protein A precursor"
"Chl_A21_00873" 1513 "s59E25H2" 0 "Heme-binding protein A precursor"
"Chl_A21_00873" 1513 "s59E5H2" 0 "Heme-binding protein A precursor"
"Chl_A21_00873" 1513 "s63E6H2" 0 "Heme-binding protein A precursor"
"Chl_A21_00874" 1514 "s59E13H2" 0 "Dipeptide transport system permease protein DppB"
"Chl_A21_00874" 1514 "s59E25H2" 0 "Dipeptide transport system permease protein DppB"
"Chl_A21_00874" 1514 "s59E5H2" 0 "Dipeptide transport system permease protein DppB"
"Chl_A21_00874" 1514 "s63E6H2" 0 "Dipeptide transport system permease protein DppB"

"Chl_A21_00875" 1515 "s59E13H2" 0 "Glutathione transport system permease protein GsiD"
"Chl_A21_00875" 1515 "s59E25H2" 0 "Glutathione transport system permease protein GsiD"
"Chl_A21_00875" 1515 "s59E5H2" 0 "Glutathione transport system permease protein GsiD"
"Chl_A21_00875" 1515 "s63E6H2" 0 "Glutathione transport system permease protein GsiD"
"Chl_A21_00876" 1516 "s59E13H2" 0 "Oligopeptide-binding protein AppA precursor"
"Chl_A21_00876" 1516 "s59E25H2" 0 "Oligopeptide-binding protein AppA precursor"
"Chl_A21_00876" 1516 "s59E5H2" 0 "Oligopeptide-binding protein AppA precursor"
"Chl_A21_00876" 1516 "s63E6H2" 0 "Oligopeptide-binding protein AppA precursor"
"Chl_A21_00877" 1517 "s59E13H2" 0 "Dipeptide transport system permease protein DppB"
"Chl_A21_00877" 1517 "s59E25H2" 0 "Dipeptide transport system permease protein DppB"
"Chl_A21_00877" 1517 "s59E5H2" 0 "Dipeptide transport system permease protein DppB"
"Chl_A21_00877" 1517 "s63E6H2" 0 "Dipeptide transport system permease protein DppB"
"Chl_A21_00878" 1518 "s59E13H2" 0 "Glutathione transport system permease protein GsiD"
"Chl_A21_00878" 1518 "s59E25H2" 0 "Glutathione transport system permease protein GsiD"
"Chl_A21_00878" 1518 "s59E5H2" 0 "Glutathione transport system permease protein GsiD"
"Chl_A21_00878" 1518 "s63E6H2" 0 "Glutathione transport system permease protein GsiD"
"Chl_A21_00879" 1519 "s59E13H2" 0 "Alpha-galactosidase"
"Chl_A21_00879" 1519 "s59E25H2" 0 "Alpha-galactosidase"
"Chl_A21_00879" 1519 "s59E5H2" 0 "Alpha-galactosidase"
"Chl_A21_00879" 1519 "s63E6H2" 0 "Alpha-galactosidase"
"Chl_A21_00880" 1520 "s59E13H2" 0 "Ultraviolet N-glycosylase/AP lyase"
"Chl_A21_00880" 1520 "s59E25H2" 0 "Ultraviolet N-glycosylase/AP lyase"
"Chl_A21_00880" 1520 "s59E5H2" 0 "Ultraviolet N-glycosylase/AP lyase"
"Chl_A21_00880" 1520 "s63E6H2" 0 "Ultraviolet N-glycosylase/AP lyase"
"Chl_A21_00881" 1521 "s59E13H2" 0 "DNA double-strand break repair Rad50 ATPase"
"Chl_A21_00881" 1521 "s59E25H2" 0 "DNA double-strand break repair Rad50 ATPase"
"Chl_A21_00881" 1521 "s59E5H2" 0 "DNA double-strand break repair Rad50 ATPase"
"Chl_A21_00881" 1521 "s63E6H2" 0 "DNA double-strand break repair Rad50 ATPase"
"Chl_A21_00882" 1522 "s59E13H2" 0 "Ribonuclease BN"
"Chl_A21_00882" 1522 "s59E25H2" 0 "Ribonuclease BN"
"Chl_A21_00882" 1522 "s59E5H2" 0 "Ribonuclease BN"
"Chl_A21_00882" 1522 "s63E6H2" 0 "Ribonuclease BN"
"Chl_A21_00883" 1523 "s59E13H2" 0 "hypothetical protein"
"Chl_A21_00883" 1523 "s59E25H2" 0 "hypothetical protein"
"Chl_A21_00883" 1523 "s59E5H2" 0 "hypothetical protein"
"Chl_A21_00883" 1523 "s63E6H2" 0 "hypothetical protein"
"Chl_A21_00884" 1524 "s59E13H2" 0 "Sorbitol dehydrogenase"
"Chl_A21_00884" 1524 "s59E25H2" 0 "Sorbitol dehydrogenase"
"Chl_A21_00884" 1524 "s59E5H2" 0 "Sorbitol dehydrogenase"
"Chl_A21_00884" 1524 "s63E6H2" 0 "Sorbitol dehydrogenase"
"Chl_A21_00885" 1525 "s59E13H2" 0 "putative L-threonine 3-dehydrogenase"
"Chl_A21_00885" 1525 "s59E25H2" 0 "putative L-threonine 3-dehydrogenase"
"Chl_A21_00885" 1525 "s59E5H2" 0 "putative L-threonine 3-dehydrogenase"
"Chl_A21_00885" 1525 "s63E6H2" 0 "putative L-threonine 3-dehydrogenase"
"Chl_A21_00886" 1526 "s59E13H2" 0 "Putative ketoacyl reductase"
"Chl_A21_00886" 1526 "s59E25H2" 0 "Putative ketoacyl reductase"
"Chl_A21_00886" 1526 "s59E5H2" 0 "Putative ketoacyl reductase"

"Chl_A21_00886" 1526 "s63E6H2" 0 "Putative ketoacyl reductase"
"Chl_A21_00888" 1528 "s59E13H2" 0 "6-pyruvoyl-tetrahydropterin synthase related domain; membrane protein"
"Chl_A21_00888" 1528 "s59E25H2" 0 "6-pyruvoyl-tetrahydropterin synthase related domain; membrane protein"
"Chl_A21_00888" 1528 "s59E5H2" 0 "6-pyruvoyl-tetrahydropterin synthase related domain; membrane protein"
"Chl_A21_00888" 1528 "s63E6H2" 0 "6-pyruvoyl-tetrahydropterin synthase related domain; membrane protein"
"Chl_A21_00889" 1529 "s59E13H2" 0 "hypothetical protein"
"Chl_A21_00889" 1529 "s59E25H2" 0 "hypothetical protein"
"Chl_A21_00889" 1529 "s59E5H2" 0 "hypothetical protein"
"Chl_A21_00889" 1529 "s63E6H2" 0 "hypothetical protein"
"Chl_A21_00890" 1530 "s59E13H2" 0 "6-pyruvoyl-tetrahydropterin synthase related domain; membrane protein"
"Chl_A21_00890" 1530 "s59E25H2" 0 "6-pyruvoyl-tetrahydropterin synthase related domain; membrane protein"
"Chl_A21_00890" 1530 "s59E5H2" 0 "6-pyruvoyl-tetrahydropterin synthase related domain; membrane protein"
"Chl_A21_00890" 1530 "s63E6H2" 0 "6-pyruvoyl-tetrahydropterin synthase related domain; membrane protein"
"Chl_B06_00001" 1532 "s59E13H2" 0.02577325 "Sensor histidine kinase CitA"
"Chl_B06_00001" 1532 "s59E25H2" 0 "Sensor histidine kinase CitA"
"Chl_B06_00001" 1532 "s59E5H2" 0.2120084 "Sensor histidine kinase CitA"
"Chl_B06_00001" 1532 "s63E6H2" 0.02622229 "Sensor histidine kinase CitA"
"Chl_B06_00002" 1533 "s59E13H2" 0.02220637 "50S ribosomal protein L34"
"Chl_B06_00002" 1533 "s59E25H2" 0 "50S ribosomal protein L34"
"Chl_B06_00002" 1533 "s59E5H2" 0.04743433 "50S ribosomal protein L34"
"Chl_B06_00002" 1533 "s63E6H2" 0.02341652 "50S ribosomal protein L34"
"Chl_B06_00003" 1534 "s59E13H2" 0.01791303 "Ribonuclease P protein component"
"Chl_B06_00003" 1534 "s59E25H2" 0 "Ribonuclease P protein component"
"Chl_B06_00003" 1534 "s59E5H2" 0.04019621 "Ribonuclease P protein component"
"Chl_B06_00003" 1534 "s63E6H2" 0.0322008 "Ribonuclease P protein component"
"Chl_B06_00004" 1535 "s59E13H2" 0.03361059 "Membrane protein insertase YidC"
"Chl_B06_00004" 1535 "s59E25H2" 0 "Membrane protein insertase YidC"
"Chl_B06_00004" 1535 "s59E5H2" 0.1089663 "Membrane protein insertase YidC"
"Chl_B06_00004" 1535 "s63E6H2" 0.05217703 "Membrane protein insertase YidC"
"Chl_B06_00005" 1536 "s59E13H2" 0.0008782314 "R3H domain protein"
"Chl_B06_00005" 1536 "s59E25H2" 0 "R3H domain protein"
"Chl_B06_00005" 1536 "s59E5H2" 0.01435584 "R3H domain protein"
"Chl_B06_00005" 1536 "s63E6H2" 0.02067368 "R3H domain protein"
"Chl_B06_00006" 1537 "s59E13H2" 0.00951429 "tRNA modification GTPase MnmE"
"Chl_B06_00006" 1537 "s59E25H2" 0 "tRNA modification GTPase MnmE"
"Chl_B06_00006" 1537 "s59E5H2" 0.04262846 "tRNA modification GTPase MnmE"
"Chl_B06_00006" 1537 "s63E6H2" 0.0535914 "tRNA modification GTPase MnmE"
"Chl_B06_00007" 1538 "s59E13H2" 0.7667416 "tRNA modification GTPase MnmE"
"Chl_B06_00007" 1538 "s59E25H2" 0 "tRNA modification GTPase MnmE"
"Chl_B06_00007" 1538 "s59E5H2" 0.003737033 "tRNA modification GTPase MnmE"
"Chl_B06_00007" 1538 "s63E6H2" 0.03223132 "tRNA modification GTPase MnmE"
"Chl_B06_00008" 1539 "s59E13H2" 1.673349 "tRNA uridine 5-carboxymethylaminomethyl modification enzyme MnmG"
"Chl_B06_00008" 1539 "s59E25H2" 0 "tRNA uridine 5-carboxymethylaminomethyl modification enzyme MnmG"
"Chl_B06_00008" 1539 "s59E5H2" 0.07211621 "tRNA uridine 5-carboxymethylaminomethyl modification enzyme MnmG"
"Chl_B06_00008" 1539 "s63E6H2" 0.1019574 "tRNA uridine 5-carboxymethylaminomethyl modification enzyme MnmG"
"Chl_B06_00009" 1540 "s59E13H2" 1.62305 "tRNA uridine 5-carboxymethylaminomethyl modification enzyme MnmG"
"Chl_B06_00009" 1540 "s59E25H2" 0 "tRNA uridine 5-carboxymethylaminomethyl modification enzyme MnmG"

"Chl_B06_00009" 1540 "s59E5H2" 0.05601678 "tRNA uridine 5-carboxymethylaminomethyl modification enzyme MnmG"
"Chl_B06_00009" 1540 "s63E6H2" 0.2790391 "tRNA uridine 5-carboxymethylaminomethyl modification enzyme MnmG"
"Chl_B06_00010" 1541 "s59E13H2" 1.217666 "Ribosomal RNA small subunit methyltransferase G"
"Chl_B06_00010" 1541 "s59E25H2" 0 "Ribosomal RNA small subunit methyltransferase G"
"Chl_B06_00010" 1541 "s59E5H2" 0.04294337 "Ribosomal RNA small subunit methyltransferase G"
"Chl_B06_00010" 1541 "s63E6H2" 0.1474892 "Ribosomal RNA small subunit methyltransferase G"
"Chl_B06_00011" 1542 "s59E13H2" 1.881762 "Sporulation initiation inhibitor protein Soj"
"Chl_B06_00011" 1542 "s59E25H2" 0 "Sporulation initiation inhibitor protein Soj"
"Chl_B06_00011" 1542 "s59E5H2" 0.04722036 "Sporulation initiation inhibitor protein Soj"
"Chl_B06_00011" 1542 "s63E6H2" 0.04412459 "Sporulation initiation inhibitor protein Soj"
"Chl_B06_00012" 1543 "s59E13H2" 2.033982 "putative chromosome-partitioning protein ParB"
"Chl_B06_00012" 1543 "s59E25H2" 0 "putative chromosome-partitioning protein ParB"
"Chl_B06_00012" 1543 "s59E5H2" 0.05720237 "putative chromosome-partitioning protein ParB"
"Chl_B06_00012" 1543 "s63E6H2" 0.02274968 "putative chromosome-partitioning protein ParB"
"Chl_B06_00013" 1544 "s59E13H2" 2.306387 "Lipoyl synthase"
"Chl_B06_00013" 1544 "s59E25H2" 0 "Lipoyl synthase"
"Chl_B06_00013" 1544 "s59E5H2" 0.07679144 "Lipoyl synthase"
"Chl_B06_00013" 1544 "s63E6H2" 0.02792979 "Lipoyl synthase"
"Chl_B06_00014" 1545 "s59E13H2" 2.354923 "AsnC family protein"
"Chl_B06_00014" 1545 "s59E25H2" 0 "AsnC family protein"
"Chl_B06_00014" 1545 "s59E5H2" 0.0521922 "AsnC family protein"
"Chl_B06_00014" 1545 "s63E6H2" 0.04699265 "AsnC family protein"
"Chl_B06_00015" 1546 "s59E13H2" 2.800257 "SPFH domain / Band 7 family protein"
"Chl_B06_00015" 1546 "s59E25H2" 0 "SPFH domain / Band 7 family protein"
"Chl_B06_00015" 1546 "s59E5H2" 0.0907429 "SPFH domain / Band 7 family protein"
"Chl_B06_00015" 1546 "s63E6H2" 0.0278818 "SPFH domain / Band 7 family protein"
"Chl_B06_00016" 1547 "s59E13H2" 0.03692565 "Cyclic di-GMP phosphodiesterase response regulator RpfG"
"Chl_B06_00016" 1547 "s59E25H2" 0 "Cyclic di-GMP phosphodiesterase response regulator RpfG"
"Chl_B06_00016" 1547 "s59E5H2" 0 "Cyclic di-GMP phosphodiesterase response regulator RpfG"
"Chl_B06_00016" 1547 "s63E6H2" 0.008404351 "Cyclic di-GMP phosphodiesterase response regulator RpfG"
"Chl_B06_00017" 1548 "s59E13H2" 3.646814 "Trehalose/maltose import ATP-binding protein MalK"
"Chl_B06_00017" 1548 "s59E25H2" 0 "Trehalose/maltose import ATP-binding protein MalK"
"Chl_B06_00017" 1548 "s59E5H2" 0.1721849 "Trehalose/maltose import ATP-binding protein MalK"
"Chl_B06_00017" 1548 "s63E6H2" 0.002781483 "Trehalose/maltose import ATP-binding protein MalK"
"Chl_B06_00018" 1549 "s59E13H2" 3.426272 "VIT family protein"
"Chl_B06_00018" 1549 "s59E25H2" 0 "VIT family protein"
"Chl_B06_00018" 1549 "s59E5H2" 0.1250925 "VIT family protein"
"Chl_B06_00018" 1549 "s63E6H2" 0 "VIT family protein"
"Chl_B06_00019" 1550 "s59E13H2" 2.377674 "putative glutamine synthetase 2"
"Chl_B06_00019" 1550 "s59E25H2" 0 "putative glutamine synthetase 2"
"Chl_B06_00019" 1550 "s59E5H2" 0.1871256 "putative glutamine synthetase 2"
"Chl_B06_00019" 1550 "s63E6H2" 0.01717467 "putative glutamine synthetase 2"
"Chl_B06_00020" 1551 "s59E13H2" 0.002963329 "HTH-type transcriptional repressor PurR"
"Chl_B06_00020" 1551 "s59E25H2" 0 "HTH-type transcriptional repressor PurR"
"Chl_B06_00020" 1551 "s59E5H2" 0.09258594 "HTH-type transcriptional repressor PurR"
"Chl_B06_00020" 1551 "s63E6H2" 0.007650072 "HTH-type transcriptional repressor PurR"
"Chl_B06_00021" 1552 "s59E13H2" 1.017295 "L-arabinose isomerase"

"Chl_B06_00021" 1552 "s59E25H2" 0.1795977 "L-arabinose isomerase"
"Chl_B06_00021" 1552 "s59E5H2" 0.02693803 "L-arabinose isomerase"
"Chl_B06_00021" 1552 "s63E6H2" 0.7876654 "L-arabinose isomerase"
"Chl_B06_00022" 1553 "s59E13H2" 0 "Modification methylase MjaII"
"Chl_B06_00022" 1553 "s59E25H2" 0 "Modification methylase MjaII"
"Chl_B06_00022" 1553 "s59E5H2" 0 "Modification methylase MjaII"
"Chl_B06_00022" 1553 "s63E6H2" 0 "Modification methylase MjaII"
"Chl_B06_00023" 1554 "s59E13H2" 0 "hypothetical protein"
"Chl_B06_00023" 1554 "s59E25H2" 0 "hypothetical protein"
"Chl_B06_00023" 1554 "s59E5H2" 0 "hypothetical protein"
"Chl_B06_00023" 1554 "s63E6H2" 0 "hypothetical protein"
"Chl_B06_00024" 1555 "s59E13H2" 0.01101609 "Tyrosine recombinase XerD"
"Chl_B06_00024" 1555 "s59E25H2" 0 "Tyrosine recombinase XerD"
"Chl_B06_00024" 1555 "s59E5H2" 0.03945713 "Tyrosine recombinase XerD"
"Chl_B06_00024" 1555 "s63E6H2" 0 "Tyrosine recombinase XerD"
"Chl_B06_00025" 1556 "s59E13H2" 0 "hypothetical protein"
"Chl_B06_00025" 1556 "s59E25H2" 0 "hypothetical protein"
"Chl_B06_00025" 1556 "s59E5H2" 0 "hypothetical protein"
"Chl_B06_00025" 1556 "s63E6H2" 0 "hypothetical protein"
"Chl_B06_00026" 1557 "s59E13H2" 0 "hypothetical protein"
"Chl_B06_00026" 1557 "s59E25H2" 0 "hypothetical protein"
"Chl_B06_00026" 1557 "s59E5H2" 0 "hypothetical protein"
"Chl_B06_00026" 1557 "s63E6H2" 0 "hypothetical protein"
"Chl_B06_00027" 1558 "s59E13H2" 0 "hypothetical protein"
"Chl_B06_00027" 1558 "s59E25H2" 0 "hypothetical protein"
"Chl_B06_00027" 1558 "s59E5H2" 0 "hypothetical protein"
"Chl_B06_00027" 1558 "s63E6H2" 0 "hypothetical protein"
"Chl_B06_00028" 1559 "s59E13H2" 0 "hypothetical protein"
"Chl_B06_00028" 1559 "s59E25H2" 0 "hypothetical protein"
"Chl_B06_00028" 1559 "s59E5H2" 0 "hypothetical protein"
"Chl_B06_00028" 1559 "s63E6H2" 0.008979181 "hypothetical protein"
"Chl_B06_00029" 1560 "s59E13H2" 0.001458524 "putative transposase"
"Chl_B06_00029" 1560 "s59E25H2" 0 "putative transposase"
"Chl_B06_00029" 1560 "s59E5H2" 0.0234034 "putative transposase"
"Chl_B06_00029" 1560 "s63E6H2" 0.003900497 "putative transposase"
"Chl_B06_00031" 1562 "s59E13H2" 0.892201 "Phd_YefM"
"Chl_B06_00031" 1562 "s59E25H2" 0 "Phd_YefM"
"Chl_B06_00031" 1562 "s59E5H2" 0.04383733 "Phd_YefM"
"Chl_B06_00031" 1562 "s63E6H2" 0.01259417 "Phd_YefM"
"Chl_B06_00032" 1563 "s59E13H2" 0 "hypothetical protein"
"Chl_B06_00032" 1563 "s59E25H2" 0 "hypothetical protein"
"Chl_B06_00032" 1563 "s59E5H2" 0 "hypothetical protein"
"Chl_B06_00032" 1563 "s63E6H2" 0.02782536 "hypothetical protein"
"Chl_B06_00033" 1564 "s59E13H2" 0 "hypothetical protein"
"Chl_B06_00033" 1564 "s59E25H2" 0 "hypothetical protein"
"Chl_B06_00033" 1564 "s59E5H2" 0 "hypothetical protein"
"Chl_B06_00033" 1564 "s63E6H2" 0 "hypothetical protein"

"Chl_B06_00034" 1565 "s59E13H2" 0.01515495 "Glucokinase"
"Chl_B06_00034" 1565 "s59E25H2" 0 "Glucokinase"
"Chl_B06_00034" 1565 "s59E5H2" 0.01488833 "Glucokinase"
"Chl_B06_00034" 1565 "s63E6H2" 0.1627643 "Glucokinase"
"Chl_C14_00001" 1566 "s59E13H2" 0.04086411 "hypothetical protein"
"Chl_C14_00001" 1566 "s59E25H2" 0 "hypothetical protein"
"Chl_C14_00001" 1566 "s59E5H2" 0 "hypothetical protein"
"Chl_C14_00001" 1566 "s63E6H2" 0 "hypothetical protein"
"Chl_C14_00002" 1567 "s59E13H2" 0.005183707 "hypothetical protein"
"Chl_C14_00002" 1567 "s59E25H2" 0 "hypothetical protein"
"Chl_C14_00002" 1567 "s59E5H2" 0 "hypothetical protein"
"Chl_C14_00002" 1567 "s63E6H2" 0 "hypothetical protein"
"Chl_C14_00003" 1568 "s59E13H2" 0 "Tyrosine recombinase XerD"
"Chl_C14_00003" 1568 "s59E25H2" 0 "Tyrosine recombinase XerD"
"Chl_C14_00003" 1568 "s59E5H2" 0 "Tyrosine recombinase XerD"
"Chl_C14_00003" 1568 "s63E6H2" 0 "Tyrosine recombinase XerD"
"Chl_C14_00005" 1570 "s59E13H2" 0 "Tyrosine recombinase XerD"
"Chl_C14_00005" 1570 "s59E25H2" 0 "Tyrosine recombinase XerD"
"Chl_C14_00005" 1570 "s59E5H2" 0 "Tyrosine recombinase XerD"
"Chl_C14_00005" 1570 "s63E6H2" 0 "Tyrosine recombinase XerD"
"Chl_C14_00007" 1572 "s59E13H2" 0 "Tyrosine recombinase XerC"
"Chl_C14_00007" 1572 "s59E25H2" 0 "Tyrosine recombinase XerC"
"Chl_C14_00007" 1572 "s59E5H2" 0 "Tyrosine recombinase XerC"
"Chl_C14_00007" 1572 "s63E6H2" 0 "Tyrosine recombinase XerC"
"Chl_C14_00008" 1573 "s59E13H2" 0 "Adenylate cyclase 2"
"Chl_C14_00008" 1573 "s59E25H2" 0 "Adenylate cyclase 2"
"Chl_C14_00008" 1573 "s59E5H2" 0 "Adenylate cyclase 2"
"Chl_C14_00008" 1573 "s63E6H2" 0 "Adenylate cyclase 2"
"Chl_C14_00009" 1574 "s59E13H2" 0 "RNA polymerase sigma factor SigM"
"Chl_C14_00009" 1574 "s59E25H2" 0 "RNA polymerase sigma factor SigM"
"Chl_C14_00009" 1574 "s59E5H2" 0 "RNA polymerase sigma factor SigM"
"Chl_C14_00009" 1574 "s63E6H2" 0 "RNA polymerase sigma factor SigM"
"Chl_C14_00010" 1575 "s59E13H2" 0 "FecR protein"
"Chl_C14_00010" 1575 "s59E25H2" 0 "FecR protein"
"Chl_C14_00010" 1575 "s59E5H2" 0 "FecR protein"
"Chl_C14_00010" 1575 "s63E6H2" 0 "FecR protein"
"Chl_C14_00011" 1576 "s59E13H2" 0 "Cyclic di-GMP phosphodiesterase response regulator RpfG"
"Chl_C14_00011" 1576 "s59E25H2" 0 "Cyclic di-GMP phosphodiesterase response regulator RpfG"
"Chl_C14_00011" 1576 "s59E5H2" 0 "Cyclic di-GMP phosphodiesterase response regulator RpfG"
"Chl_C14_00011" 1576 "s63E6H2" 0 "Cyclic di-GMP phosphodiesterase response regulator RpfG"
"Chl_C14_00012" 1577 "s59E13H2" 0 "Phospholipase YtpA"
"Chl_C14_00012" 1577 "s59E25H2" 0 "Phospholipase YtpA"
"Chl_C14_00012" 1577 "s59E5H2" 0 "Phospholipase YtpA"
"Chl_C14_00012" 1577 "s63E6H2" 0 "Phospholipase YtpA"
"Chl_C14_00013" 1578 "s59E13H2" 0 "hypothetical protein"
"Chl_C14_00013" 1578 "s59E25H2" 0 "hypothetical protein"
"Chl_C14_00013" 1578 "s59E5H2" 0 "hypothetical protein"

"Chl_C14_00013" 1578 "s63E6H2" 0 "hypothetical protein"
"Chl_C14_00015" 1580 "s59E13H2" 0 "Tungsten-containing aldehyde ferredoxin oxidoreductase"
"Chl_C14_00015" 1580 "s59E25H2" 0 "Tungsten-containing aldehyde ferredoxin oxidoreductase"
"Chl_C14_00015" 1580 "s59E5H2" 0 "Tungsten-containing aldehyde ferredoxin oxidoreductase"
"Chl_C14_00015" 1580 "s63E6H2" 0 "Tungsten-containing aldehyde ferredoxin oxidoreductase"
"Chl_C14_00016" 1581 "s59E13H2" 0 "hypothetical protein"
"Chl_C14_00016" 1581 "s59E25H2" 0 "hypothetical protein"
"Chl_C14_00016" 1581 "s59E5H2" 0 "hypothetical protein"
"Chl_C14_00016" 1581 "s63E6H2" 0 "hypothetical protein"
"Chl_C14_00017" 1582 "s59E13H2" 0 "Sialic acid TRAP transporter permease protein SiaT"
"Chl_C14_00017" 1582 "s59E25H2" 0 "Sialic acid TRAP transporter permease protein SiaT"
"Chl_C14_00017" 1582 "s59E5H2" 0 "Sialic acid TRAP transporter permease protein SiaT"
"Chl_C14_00017" 1582 "s63E6H2" 0 "Sialic acid TRAP transporter permease protein SiaT"
"Chl_C14_00018" 1583 "s59E13H2" 0 "Tripartite ATP-independent periplasmic transporters, DctQ component"
"Chl_C14_00018" 1583 "s59E25H2" 0 "Tripartite ATP-independent periplasmic transporters, DctQ component"
"Chl_C14_00018" 1583 "s59E5H2" 0 "Tripartite ATP-independent periplasmic transporters, DctQ component"
"Chl_C14_00018" 1583 "s63E6H2" 0 "Tripartite ATP-independent periplasmic transporters, DctQ component"
"Chl_C14_00019" 1584 "s59E13H2" 0 "C4-dicarboxylate-binding periplasmic protein precursor"
"Chl_C14_00019" 1584 "s59E25H2" 0 "C4-dicarboxylate-binding periplasmic protein precursor"
"Chl_C14_00019" 1584 "s59E5H2" 0 "C4-dicarboxylate-binding periplasmic protein precursor"
"Chl_C14_00019" 1584 "s63E6H2" 0 "C4-dicarboxylate-binding periplasmic protein precursor"
"Chl_C14_00020" 1585 "s59E13H2" 0 "hypothetical protein"
"Chl_C14_00020" 1585 "s59E25H2" 0 "hypothetical protein"
"Chl_C14_00020" 1585 "s59E5H2" 0 "hypothetical protein"
"Chl_C14_00020" 1585 "s63E6H2" 0 "hypothetical protein"
"Chl_C14_00022" 1587 "s59E13H2" 0 "metal-dependent hydrolase"
"Chl_C14_00022" 1587 "s59E25H2" 0 "metal-dependent hydrolase"
"Chl_C14_00022" 1587 "s59E5H2" 0 "metal-dependent hydrolase"
"Chl_C14_00022" 1587 "s63E6H2" 0 "metal-dependent hydrolase"
"Chl_C14_00023" 1588 "s59E13H2" 0 "L-hydantoinase"
"Chl_C14_00023" 1588 "s59E25H2" 0 "L-hydantoinase"
"Chl_C14_00023" 1588 "s59E5H2" 0 "L-hydantoinase"
"Chl_C14_00023" 1588 "s63E6H2" 0 "L-hydantoinase"
"Chl_C14_00024" 1589 "s59E13H2" 0 "NADH oxidase"
"Chl_C14_00024" 1589 "s59E25H2" 0 "NADH oxidase"
"Chl_C14_00024" 1589 "s59E5H2" 0 "NADH oxidase"
"Chl_C14_00024" 1589 "s63E6H2" 0 "NADH oxidase"
"Chl_C14_00025" 1590 "s59E13H2" 0 "(R)-stereoselective amidase"
"Chl_C14_00025" 1590 "s59E25H2" 0 "(R)-stereoselective amidase"
"Chl_C14_00025" 1590 "s59E5H2" 0 "(R)-stereoselective amidase"
"Chl_C14_00025" 1590 "s63E6H2" 0 "(R)-stereoselective amidase"
"Chl_C14_00026" 1591 "s59E13H2" 0 "CAAX amino terminal protease self- immunity"
"Chl_C14_00026" 1591 "s59E25H2" 0 "CAAX amino terminal protease self- immunity"
"Chl_C14_00026" 1591 "s59E5H2" 0 "CAAX amino terminal protease self- immunity"
"Chl_C14_00026" 1591 "s63E6H2" 0 "CAAX amino terminal protease self- immunity"
"Chl_C14_00027" 1592 "s59E13H2" 0 "hypothetical protein"
"Chl_C14_00027" 1592 "s59E25H2" 0 "hypothetical protein"

"Chl_C14_00027" 1592 "s59E5H2" 0 "hypothetical protein"
"Chl_C14_00027" 1592 "s63E6H2" 0 "hypothetical protein"
"Chl_C14_00028" 1593 "s59E13H2" 0 "Propionyl-CoA:succinate CoA transferase"
"Chl_C14_00028" 1593 "s59E25H2" 0 "Propionyl-CoA:succinate CoA transferase"
"Chl_C14_00028" 1593 "s59E5H2" 0 "Propionyl-CoA:succinate CoA transferase"
"Chl_C14_00028" 1593 "s63E6H2" 0 "Propionyl-CoA:succinate CoA transferase"
"Chl_C14_00029" 1594 "s59E13H2" 0 "bifunctional enoyl-CoA hydratase/phosphate acetyltransferase"
"Chl_C14_00029" 1594 "s59E25H2" 0 "bifunctional enoyl-CoA hydratase/phosphate acetyltransferase"
"Chl_C14_00029" 1594 "s59E5H2" 0 "bifunctional enoyl-CoA hydratase/phosphate acetyltransferase"
"Chl_C14_00029" 1594 "s63E6H2" 0 "bifunctional enoyl-CoA hydratase/phosphate acetyltransferase"
"Chl_C14_00032" 1597 "s59E13H2" 0 "GTPase ObgE"
"Chl_C14_00032" 1597 "s59E25H2" 0 "GTPase ObgE"
"Chl_C14_00032" 1597 "s59E5H2" 0 "GTPase ObgE"
"Chl_C14_00032" 1597 "s63E6H2" 0 "GTPase ObgE"
"Chl_C14_00033" 1598 "s59E13H2" 0 "Nicotinate-nucleotide adenylyltransferase"
"Chl_C14_00033" 1598 "s59E25H2" 0 "Nicotinate-nucleotide adenylyltransferase"
"Chl_C14_00033" 1598 "s59E5H2" 0 "Nicotinate-nucleotide adenylyltransferase"
"Chl_C14_00033" 1598 "s63E6H2" 0 "Nicotinate-nucleotide adenylyltransferase"
"Chl_C14_00034" 1599 "s59E13H2" 0 "Orotate phosphoribosyltransferase"
"Chl_C14_00034" 1599 "s59E25H2" 0 "Orotate phosphoribosyltransferase"
"Chl_C14_00034" 1599 "s59E5H2" 0 "Orotate phosphoribosyltransferase"
"Chl_C14_00034" 1599 "s63E6H2" 0 "Orotate phosphoribosyltransferase"
"Chl_C14_00035" 1600 "s59E13H2" 0 "60 kDa chaperonin"
"Chl_C14_00035" 1600 "s59E25H2" 0 "60 kDa chaperonin"
"Chl_C14_00035" 1600 "s59E5H2" 0.004974395 "60 kDa chaperonin"
"Chl_C14_00035" 1600 "s63E6H2" 0 "60 kDa chaperonin"
"Chl_C14_00036" 1601 "s59E13H2" 0 "10 kDa chaperonin"
"Chl_C14_00036" 1601 "s59E25H2" 0 "10 kDa chaperonin"
"Chl_C14_00036" 1601 "s59E5H2" 0 "10 kDa chaperonin"
"Chl_C14_00036" 1601 "s63E6H2" 0 "10 kDa chaperonin"
"Chl_C14_00037" 1602 "s59E13H2" 0 "transcriptional repressor DicA"
"Chl_C14_00037" 1602 "s59E25H2" 0 "transcriptional repressor DicA"
"Chl_C14_00037" 1602 "s59E5H2" 0 "transcriptional repressor DicA"
"Chl_C14_00037" 1602 "s63E6H2" 0 "transcriptional repressor DicA"
"Chl_C14_00038" 1603 "s59E13H2" 0 "hypothetical protein"
"Chl_C14_00038" 1603 "s59E25H2" 0 "hypothetical protein"
"Chl_C14_00038" 1603 "s59E5H2" 0 "hypothetical protein"
"Chl_C14_00038" 1603 "s63E6H2" 0 "hypothetical protein"
"Chl_C14_00039" 1604 "s59E13H2" 0 "tRNA N6-adenosine threonylcarbamoyltransferase"
"Chl_C14_00039" 1604 "s59E25H2" 0 "tRNA N6-adenosine threonylcarbamoyltransferase"
"Chl_C14_00039" 1604 "s59E5H2" 0 "tRNA N6-adenosine threonylcarbamoyltransferase"
"Chl_C14_00039" 1604 "s63E6H2" 0 "tRNA N6-adenosine threonylcarbamoyltransferase"
"Chl_C14_00040" 1605 "s59E13H2" 0 "Magnesium and cobalt efflux protein CorC"
"Chl_C14_00040" 1605 "s59E25H2" 0 "Magnesium and cobalt efflux protein CorC"
"Chl_C14_00040" 1605 "s59E5H2" 0 "Magnesium and cobalt efflux protein CorC"
"Chl_C14_00040" 1605 "s63E6H2" 0 "Magnesium and cobalt efflux protein CorC"
"Chl_C14_00041" 1606 "s59E13H2" 0 "hypothetical protein"

"Chl_C14_00041" 1606 "s59E25H2" 0 "hypothetical protein"
"Chl_C14_00041" 1606 "s59E5H2" 0 "hypothetical protein"
"Chl_C14_00041" 1606 "s63E6H2" 0 "hypothetical protein"
"Chl_C14_00042" 1607 "s59E13H2" 0 "hypothetical protein"
"Chl_C14_00042" 1607 "s59E25H2" 0 "hypothetical protein"
"Chl_C14_00042" 1607 "s59E5H2" 0 "hypothetical protein"
"Chl_C14_00042" 1607 "s63E6H2" 0 "hypothetical protein"
"Chl_C14_00044" 1609 "s59E13H2" 0 "Tryptophan synthase beta chain 1"
"Chl_C14_00044" 1609 "s59E25H2" 0 "Tryptophan synthase beta chain 1"
"Chl_C14_00044" 1609 "s59E5H2" 0 "Tryptophan synthase beta chain 1"
"Chl_C14_00044" 1609 "s63E6H2" 0 "Tryptophan synthase beta chain 1"
"Chl_C14_00045" 1610 "s59E13H2" 0 "Serine protease Do-like HtrB"
"Chl_C14_00045" 1610 "s59E25H2" 0 "Serine protease Do-like HtrB"
"Chl_C14_00045" 1610 "s59E5H2" 0 "Serine protease Do-like HtrB"
"Chl_C14_00045" 1610 "s63E6H2" 0 "Serine protease Do-like HtrB"
"Chl_C14_00046" 1611 "s59E13H2" 0 "Isoleucine--tRNA ligase"
"Chl_C14_00046" 1611 "s59E25H2" 0 "Isoleucine--tRNA ligase"
"Chl_C14_00046" 1611 "s59E5H2" 0.001314181 "Isoleucine--tRNA ligase"
"Chl_C14_00046" 1611 "s63E6H2" 0 "Isoleucine--tRNA ligase"
"Chl_C14_00048" 1613 "s59E13H2" 0 "R3H domain protein"
"Chl_C14_00048" 1613 "s59E25H2" 0 "R3H domain protein"
"Chl_C14_00048" 1613 "s59E5H2" 0 "R3H domain protein"
"Chl_C14_00048" 1613 "s63E6H2" 0 "R3H domain protein"
"Chl_C14_00049" 1614 "s59E13H2" 0 "Membrane protein insertase MisCA precursor"
"Chl_C14_00049" 1614 "s59E25H2" 0 "Membrane protein insertase MisCA precursor"
"Chl_C14_00049" 1614 "s59E5H2" 0 "Membrane protein insertase MisCA precursor"
"Chl_C14_00049" 1614 "s63E6H2" 0 "Membrane protein insertase MisCA precursor"
"Chl_C14_00050" 1615 "s59E13H2" 0 "outer membrane biogenesis protein BamB"
"Chl_C14_00050" 1615 "s59E25H2" 0 "outer membrane biogenesis protein BamB"
"Chl_C14_00050" 1615 "s59E5H2" 0 "outer membrane biogenesis protein BamB"
"Chl_C14_00050" 1615 "s63E6H2" 0 "outer membrane biogenesis protein BamB"
"Chl_C14_00052" 1617 "s59E13H2" 0 "hypothetical protein"
"Chl_C14_00052" 1617 "s59E25H2" 0 "hypothetical protein"
"Chl_C14_00052" 1617 "s59E5H2" 0 "hypothetical protein"
"Chl_C14_00052" 1617 "s63E6H2" 0 "hypothetical protein"
"Chl_C14_00054" 1619 "s59E13H2" 0 "hypothetical protein"
"Chl_C14_00054" 1619 "s59E25H2" 0 "hypothetical protein"
"Chl_C14_00054" 1619 "s59E5H2" 0 "hypothetical protein"
"Chl_C14_00054" 1619 "s63E6H2" 0 "hypothetical protein"
"Chl_C14_00056" 1621 "s59E13H2" 0 "hypothetical protein"
"Chl_C14_00056" 1621 "s59E25H2" 0 "hypothetical protein"
"Chl_C14_00056" 1621 "s59E5H2" 0 "hypothetical protein"
"Chl_C14_00056" 1621 "s63E6H2" 0 "hypothetical protein"
"Chl_C14_00058" 1623 "s59E13H2" 0 "putative tyrosine recombinase XerC-like protein"
"Chl_C14_00058" 1623 "s59E25H2" 0 "putative tyrosine recombinase XerC-like protein"
"Chl_C14_00058" 1623 "s59E5H2" 0 "putative tyrosine recombinase XerC-like protein"
"Chl_C14_00058" 1623 "s63E6H2" 0 "putative tyrosine recombinase XerC-like protein"

"Chl_C14_00060" 1625 "s59E13H2" 0 "Tyrosine recombinase XerD"
"Chl_C14_00060" 1625 "s59E25H2" 0 "Tyrosine recombinase XerD"
"Chl_C14_00060" 1625 "s59E5H2" 0 "Tyrosine recombinase XerD"
"Chl_C14_00060" 1625 "s63E6H2" 0 "Tyrosine recombinase XerD"
"Chl_C14_00062" 1627 "s59E13H2" 0 "chromosome segregation protein"
"Chl_C14_00062" 1627 "s59E25H2" 0 "chromosome segregation protein"
"Chl_C14_00062" 1627 "s59E5H2" 0 "chromosome segregation protein"
"Chl_C14_00062" 1627 "s63E6H2" 0 "chromosome segregation protein"
"Chl_C14_00063" 1628 "s59E13H2" 0 "Modification methylase MjaII"
"Chl_C14_00063" 1628 "s59E25H2" 0 "Modification methylase MjaII"
"Chl_C14_00063" 1628 "s59E5H2" 0 "Modification methylase MjaII"
"Chl_C14_00063" 1628 "s63E6H2" 0 "Modification methylase MjaII"
"Chl_C14_00064" 1629 "s59E13H2" 0.02010024 "Oxygen sensor histidine kinase NreB"
"Chl_C14_00064" 1629 "s59E25H2" 0 "Oxygen sensor histidine kinase NreB"
"Chl_C14_00064" 1629 "s59E5H2" 0.2083924 "Oxygen sensor histidine kinase NreB"
"Chl_C14_00064" 1629 "s63E6H2" 0 "Oxygen sensor histidine kinase NreB"
"Chl_C14_00065" 1630 "s59E13H2" 0.007509116 "Transcriptional regulatory protein DegU"
"Chl_C14_00065" 1630 "s59E25H2" 0 "Transcriptional regulatory protein DegU"
"Chl_C14_00065" 1630 "s59E5H2" 0 "Transcriptional regulatory protein DegU"
"Chl_C14_00065" 1630 "s63E6H2" 0 "Transcriptional regulatory protein DegU"
"Chl_C14_00067" 1632 "s59E13H2" 0.0120396 "NADP-reducing hydrogenase subunit HndA"
"Chl_C14_00067" 1632 "s59E25H2" 0 "NADP-reducing hydrogenase subunit HndA"
"Chl_C14_00067" 1632 "s59E5H2" 0 "NADP-reducing hydrogenase subunit HndA"
"Chl_C14_00067" 1632 "s63E6H2" 0 "NADP-reducing hydrogenase subunit HndA"
"Chl_C14_00068" 1633 "s59E13H2" 0 "NADP-reducing hydrogenase subunit HndC"
"Chl_C14_00068" 1633 "s59E25H2" 0 "NADP-reducing hydrogenase subunit HndC"
"Chl_C14_00068" 1633 "s59E5H2" 0 "NADP-reducing hydrogenase subunit HndC"
"Chl_C14_00068" 1633 "s63E6H2" 0 "NADP-reducing hydrogenase subunit HndC"
"Chl_C14_00069" 1634 "s59E13H2" 0 "NADP-reducing hydrogenase subunit HndC"
"Chl_C14_00069" 1634 "s59E25H2" 0 "NADP-reducing hydrogenase subunit HndC"
"Chl_C14_00069" 1634 "s59E5H2" 0 "NADP-reducing hydrogenase subunit HndC"
"Chl_C14_00069" 1634 "s63E6H2" 0 "NADP-reducing hydrogenase subunit HndC"
"Chl_C14_00070" 1635 "s59E13H2" 0 "hypothetical protein"
"Chl_C14_00070" 1635 "s59E25H2" 0 "hypothetical protein"
"Chl_C14_00070" 1635 "s59E5H2" 0 "hypothetical protein"
"Chl_C14_00070" 1635 "s63E6H2" 0 "hypothetical protein"
"Chl_C14_00071" 1636 "s59E13H2" 0 "hypothetical protein"
"Chl_C14_00071" 1636 "s59E25H2" 0 "hypothetical protein"
"Chl_C14_00071" 1636 "s59E5H2" 0 "hypothetical protein"
"Chl_C14_00071" 1636 "s63E6H2" 0 "hypothetical protein"
"Chl_C14_00074" 1639 "s59E13H2" 0 "hypothetical protein"
"Chl_C14_00074" 1639 "s59E25H2" 0 "hypothetical protein"
"Chl_C14_00074" 1639 "s59E5H2" 0 "hypothetical protein"
"Chl_C14_00074" 1639 "s63E6H2" 0 "hypothetical protein"
"Chl_C14_00075" 1640 "s59E13H2" 0 "Recombinase"
"Chl_C14_00075" 1640 "s59E25H2" 0 "Recombinase"
"Chl_C14_00075" 1640 "s59E5H2" 0 "Recombinase"

"Chl_C14_00075" 1640 "s63E6H2" 0 "Recombinase"
"Chl_C14_00076" 1641 "s59E13H2" 0 "Response regulator protein VraR"
"Chl_C14_00076" 1641 "s59E25H2" 0 "Response regulator protein VraR"
"Chl_C14_00076" 1641 "s59E5H2" 0 "Response regulator protein VraR"
"Chl_C14_00076" 1641 "s63E6H2" 0 "Response regulator protein VraR"
"Chl_C14_00077" 1642 "s59E13H2" 0 "Bifunctional homocysteine S-methyltransferase/5,10-methylenetetrahydrofolate reductase"
"Chl_C14_00077" 1642 "s59E25H2" 0 "Bifunctional homocysteine S-methyltransferase/5,10-methylenetetrahydrofolate reductase"
"Chl_C14_00077" 1642 "s59E5H2" 0 "Bifunctional homocysteine S-methyltransferase/5,10-methylenetetrahydrofolate reductase"
"Chl_C14_00077" 1642 "s63E6H2" 0 "Bifunctional homocysteine S-methyltransferase/5,10-methylenetetrahydrofolate reductase"
"Chl_C14_00078" 1643 "s59E13H2" 0 "hypothetical protein"
"Chl_C14_00078" 1643 "s59E25H2" 0 "hypothetical protein"
"Chl_C14_00078" 1643 "s59E5H2" 0 "hypothetical protein"
"Chl_C14_00078" 1643 "s63E6H2" 0 "hypothetical protein"
"Chl_C14_00079" 1644 "s59E13H2" 0 "Sulfide dehydrogenase subunit alpha precursor"
"Chl_C14_00079" 1644 "s59E25H2" 0 "Sulfide dehydrogenase subunit alpha precursor"
"Chl_C14_00079" 1644 "s59E5H2" 0 "Sulfide dehydrogenase subunit alpha precursor"
"Chl_C14_00079" 1644 "s63E6H2" 0 "Sulfide dehydrogenase subunit alpha precursor"
"Chl_C14_00080" 1645 "s59E13H2" 0 "Oxygen regulatory protein NreC"
"Chl_C14_00080" 1645 "s59E25H2" 0 "Oxygen regulatory protein NreC"
"Chl_C14_00080" 1645 "s59E5H2" 0 "Oxygen regulatory protein NreC"
"Chl_C14_00080" 1645 "s63E6H2" 0 "Oxygen regulatory protein NreC"
"Chl_C14_00081" 1646 "s59E13H2" 0 "Signal transduction histidine-protein kinase/phosphatase DegS"
"Chl_C14_00081" 1646 "s59E25H2" 0 "Signal transduction histidine-protein kinase/phosphatase DegS"
"Chl_C14_00081" 1646 "s59E5H2" 0 "Signal transduction histidine-protein kinase/phosphatase DegS"
"Chl_C14_00081" 1646 "s63E6H2" 0 "Signal transduction histidine-protein kinase/phosphatase DegS"
"Chl_C14_00082" 1647 "s59E13H2" 0 "Tripartite tricarboxylate transporter TctB family protein"
"Chl_C14_00082" 1647 "s59E25H2" 0 "Tripartite tricarboxylate transporter TctB family protein"
"Chl_C14_00082" 1647 "s59E5H2" 0 "Tripartite tricarboxylate transporter TctB family protein"
"Chl_C14_00082" 1647 "s63E6H2" 0 "Tripartite tricarboxylate transporter TctB family protein"
"Chl_C14_00083" 1648 "s59E13H2" 0 "Tripartite tricarboxylate transporter TctA family protein"
"Chl_C14_00083" 1648 "s59E25H2" 0 "Tripartite tricarboxylate transporter TctA family protein"
"Chl_C14_00083" 1648 "s59E5H2" 0 "Tripartite tricarboxylate transporter TctA family protein"
"Chl_C14_00083" 1648 "s63E6H2" 0 "Tripartite tricarboxylate transporter TctA family protein"
"Chl_C14_00084" 1649 "s59E13H2" 0 "Succinyl-CoA:coenzyme A transferase"
"Chl_C14_00084" 1649 "s59E25H2" 0 "Succinyl-CoA:coenzyme A transferase"
"Chl_C14_00084" 1649 "s59E5H2" 0 "Succinyl-CoA:coenzyme A transferase"
"Chl_C14_00084" 1649 "s63E6H2" 0 "Succinyl-CoA:coenzyme A transferase"
"Chl_C14_00085" 1650 "s59E13H2" 0 "bifunctional enoyl-CoA hydratase/phosphate acetyltransferase"
"Chl_C14_00085" 1650 "s59E25H2" 0 "bifunctional enoyl-CoA hydratase/phosphate acetyltransferase"
"Chl_C14_00085" 1650 "s59E5H2" 0 "bifunctional enoyl-CoA hydratase/phosphate acetyltransferase"
"Chl_C14_00085" 1650 "s63E6H2" 0 "bifunctional enoyl-CoA hydratase/phosphate acetyltransferase"
"Chl_C14_00086" 1651 "s59E13H2" 0 "Succinyl-CoA:(R)-benzylsuccinate CoA-transferase subunit BbsE"
"Chl_C14_00086" 1651 "s59E25H2" 0 "Succinyl-CoA:(R)-benzylsuccinate CoA-transferase subunit BbsE"
"Chl_C14_00086" 1651 "s59E5H2" 0 "Succinyl-CoA:(R)-benzylsuccinate CoA-transferase subunit BbsE"
"Chl_C14_00086" 1651 "s63E6H2" 0 "Succinyl-CoA:(R)-benzylsuccinate CoA-transferase subunit BbsE"
"Chl_C14_00087" 1652 "s59E13H2" 0 "Succinyl-CoA:(R)-benzylsuccinate CoA-transferase subunit BbsF"
"Chl_C14_00087" 1652 "s59E25H2" 0 "Succinyl-CoA:(R)-benzylsuccinate CoA-transferase subunit BbsF"

"Chl_C14_00087" 1652 "s59E5H2" 0 "Succinyl-CoA:(R)-benzylsuccinate CoA-transferase subunit BbsF"
"Chl_C14_00087" 1652 "s63E6H2" 0 "Succinyl-CoA:(R)-benzylsuccinate CoA-transferase subunit BbsF"
"Chl_C14_00088" 1653 "s59E13H2" 0 "metal-dependent hydrolase"
"Chl_C14_00088" 1653 "s59E25H2" 0 "metal-dependent hydrolase"
"Chl_C14_00088" 1653 "s59E5H2" 0 "metal-dependent hydrolase"
"Chl_C14_00088" 1653 "s63E6H2" 0 "metal-dependent hydrolase"
"Chl_C14_00090" 1655 "s59E13H2" 0 "Glycine cleavage system H protein"
"Chl_C14_00090" 1655 "s59E25H2" 0 "Glycine cleavage system H protein"
"Chl_C14_00090" 1655 "s59E5H2" 0 "Glycine cleavage system H protein"
"Chl_C14_00090" 1655 "s63E6H2" 0 "Glycine cleavage system H protein"
"Chl_C14_00094" 1659 "s59E13H2" 0 "Antibiotic biosynthesis monooxygenase"
"Chl_C14_00094" 1659 "s59E25H2" 0 "Antibiotic biosynthesis monooxygenase"
"Chl_C14_00094" 1659 "s59E5H2" 0 "Antibiotic biosynthesis monooxygenase"
"Chl_C14_00094" 1659 "s63E6H2" 0 "Antibiotic biosynthesis monooxygenase"
"Chl_C14_00095" 1660 "s59E13H2" 0 "Oxygen sensor histidine kinase NreB"
"Chl_C14_00095" 1660 "s59E25H2" 0 "Oxygen sensor histidine kinase NreB"
"Chl_C14_00095" 1660 "s59E5H2" 0 "Oxygen sensor histidine kinase NreB"
"Chl_C14_00095" 1660 "s63E6H2" 0 "Oxygen sensor histidine kinase NreB"
"Chl_C14_00096" 1661 "s59E13H2" 0 "Transcriptional regulatory protein DegU"
"Chl_C14_00096" 1661 "s59E25H2" 0 "Transcriptional regulatory protein DegU"
"Chl_C14_00096" 1661 "s59E5H2" 0 "Transcriptional regulatory protein DegU"
"Chl_C14_00096" 1661 "s63E6H2" 0 "Transcriptional regulatory protein DegU"
"Chl_C14_00097" 1662 "s59E13H2" 0 "hypothetical protein"
"Chl_C14_00097" 1662 "s59E25H2" 0 "hypothetical protein"
"Chl_C14_00097" 1662 "s59E5H2" 0 "hypothetical protein"
"Chl_C14_00097" 1662 "s63E6H2" 0 "hypothetical protein"
"Chl_C14_00098" 1663 "s59E13H2" 0 "NADH-quinone oxidoreductase subunit E"
"Chl_C14_00098" 1663 "s59E25H2" 0 "NADH-quinone oxidoreductase subunit E"
"Chl_C14_00098" 1663 "s59E5H2" 0 "NADH-quinone oxidoreductase subunit E"
"Chl_C14_00098" 1663 "s63E6H2" 0 "NADH-quinone oxidoreductase subunit E"
"Chl_C14_00099" 1664 "s59E13H2" 0 "NADP-reducing hydrogenase subunit HndC"
"Chl_C14_00099" 1664 "s59E25H2" 0 "NADP-reducing hydrogenase subunit HndC"
"Chl_C14_00099" 1664 "s59E5H2" 0 "NADP-reducing hydrogenase subunit HndC"
"Chl_C14_00099" 1664 "s63E6H2" 0 "NADP-reducing hydrogenase subunit HndC"
"Chl_C14_00100" 1665 "s59E13H2" 0 "Putative formate dehydrogenase"
"Chl_C14_00100" 1665 "s59E25H2" 0 "Putative formate dehydrogenase"
"Chl_C14_00100" 1665 "s59E5H2" 0.002194927 "Putative formate dehydrogenase"
"Chl_C14_00100" 1665 "s63E6H2" 0 "Putative formate dehydrogenase"
"Chl_C14_00101" 1666 "s59E13H2" 0 "putative MFS-type transporter YhjX"
"Chl_C14_00101" 1666 "s59E25H2" 0 "putative MFS-type transporter YhjX"
"Chl_C14_00101" 1666 "s59E5H2" 0 "putative MFS-type transporter YhjX"
"Chl_C14_00101" 1666 "s63E6H2" 0 "putative MFS-type transporter YhjX"
"Chl_C14_00104" 1669 "s59E13H2" 0.001001076 "Anthranilate synthase component 1"
"Chl_C14_00104" 1669 "s59E25H2" 0 "Anthranilate synthase component 1"
"Chl_C14_00104" 1669 "s59E5H2" 0.009718235 "Anthranilate synthase component 1"
"Chl_C14_00104" 1669 "s63E6H2" 0 "Anthranilate synthase component 1"
"Chl_C14_00105" 1670 "s59E13H2" 0 "Aminodeoxychorismate synthase component 2"

"Chl_C14_00105" 1670 "s59E25H2" 0 "Aminodeoxychorismate synthase component 2"
"Chl_C14_00105" 1670 "s59E5H2" 0.0003981829 "Aminodeoxychorismate synthase component 2"
"Chl_C14_00105" 1670 "s63E6H2" 0 "Aminodeoxychorismate synthase component 2"
"Chl_C14_00106" 1671 "s59E13H2" 0 "Anthranilate phosphoribosyltransferase"
"Chl_C14_00106" 1671 "s59E25H2" 0 "Anthranilate phosphoribosyltransferase"
"Chl_C14_00106" 1671 "s59E5H2" 0 "Anthranilate phosphoribosyltransferase"
"Chl_C14_00106" 1671 "s63E6H2" 0 "Anthranilate phosphoribosyltransferase"
"Chl_C14_00107" 1672 "s59E13H2" 0 "Indole-3-glycerol phosphate synthase"
"Chl_C14_00107" 1672 "s59E25H2" 0 "Indole-3-glycerol phosphate synthase"
"Chl_C14_00107" 1672 "s59E5H2" 0 "Indole-3-glycerol phosphate synthase"
"Chl_C14_00107" 1672 "s63E6H2" 0 "Indole-3-glycerol phosphate synthase"
"Chl_C14_00108" 1673 "s59E13H2" 0 "N-(5'-phosphoribosyl)anthranilate isomerase"
"Chl_C14_00108" 1673 "s59E25H2" 0 "N-(5'-phosphoribosyl)anthranilate isomerase"
"Chl_C14_00108" 1673 "s59E5H2" 0 "N-(5'-phosphoribosyl)anthranilate isomerase"
"Chl_C14_00108" 1673 "s63E6H2" 0 "N-(5'-phosphoribosyl)anthranilate isomerase"
"Chl_C14_00109" 1674 "s59E13H2" 0 "Tryptophan synthase beta chain"
"Chl_C14_00109" 1674 "s59E25H2" 0 "Tryptophan synthase beta chain"
"Chl_C14_00109" 1674 "s59E5H2" 0 "Tryptophan synthase beta chain"
"Chl_C14_00109" 1674 "s63E6H2" 0 "Tryptophan synthase beta chain"
"Chl_C14_00110" 1675 "s59E13H2" 0 "Tryptophan synthase alpha chain"
"Chl_C14_00110" 1675 "s59E25H2" 0 "Tryptophan synthase alpha chain"
"Chl_C14_00110" 1675 "s59E5H2" 0 "Tryptophan synthase alpha chain"
"Chl_C14_00110" 1675 "s63E6H2" 0 "Tryptophan synthase alpha chain"
"Chl_C14_00113" 1678 "s59E13H2" 0 "hypothetical protein"
"Chl_C14_00113" 1678 "s59E25H2" 0 "hypothetical protein"
"Chl_C14_00113" 1678 "s59E5H2" 0 "hypothetical protein"
"Chl_C14_00113" 1678 "s63E6H2" 0 "hypothetical protein"
"Chl_C14_00115" 1680 "s59E13H2" 0 "putative cold shock protein A"
"Chl_C14_00115" 1680 "s59E25H2" 0 "putative cold shock protein A"
"Chl_C14_00115" 1680 "s59E5H2" 0 "putative cold shock protein A"
"Chl_C14_00115" 1680 "s63E6H2" 0.6933953 "putative cold shock protein A"
"Chl_C14_00116" 1681 "s59E13H2" 0 "hypothetical protein"
"Chl_C14_00116" 1681 "s59E25H2" 0 "hypothetical protein"
"Chl_C14_00116" 1681 "s59E5H2" 0 "hypothetical protein"
"Chl_C14_00116" 1681 "s63E6H2" 0 "hypothetical protein"
"Chl_C14_00117" 1682 "s59E13H2" 0 "putative solute-binding protein precursor"
"Chl_C14_00117" 1682 "s59E25H2" 0 "putative solute-binding protein precursor"
"Chl_C14_00117" 1682 "s59E5H2" 0 "putative solute-binding protein precursor"
"Chl_C14_00117" 1682 "s63E6H2" 0 "putative solute-binding protein precursor"
"Chl_C14_00118" 1683 "s59E13H2" 0 "Sulfate/thiosulfate import ATP-binding protein CysA"
"Chl_C14_00118" 1683 "s59E25H2" 0 "Sulfate/thiosulfate import ATP-binding protein CysA"
"Chl_C14_00118" 1683 "s59E5H2" 0 "Sulfate/thiosulfate import ATP-binding protein CysA"
"Chl_C14_00118" 1683 "s63E6H2" 0 "Sulfate/thiosulfate import ATP-binding protein CysA"
"Chl_C14_00119" 1684 "s59E13H2" 0 "Molybdate/tungstate transport system permease protein WtpB"
"Chl_C14_00119" 1684 "s59E25H2" 0 "Molybdate/tungstate transport system permease protein WtpB"
"Chl_C14_00119" 1684 "s59E5H2" 0 "Molybdate/tungstate transport system permease protein WtpB"
"Chl_C14_00119" 1684 "s63E6H2" 0 "Molybdate/tungstate transport system permease protein WtpB"

"Chl_C14_00121" 1686 "s59E13H2" 0 "hydroxyacylglutathione hydrolase"
"Chl_C14_00121" 1686 "s59E25H2" 0 "hydroxyacylglutathione hydrolase"
"Chl_C14_00121" 1686 "s59E5H2" 0 "hydroxyacylglutathione hydrolase"
"Chl_C14_00121" 1686 "s63E6H2" 0 "hydroxyacylglutathione hydrolase"
"Chl_C14_00123" 1688 "s59E13H2" 0.0006970267 "Magnesium transport protein CorA"
"Chl_C14_00123" 1688 "s59E25H2" 0 "Magnesium transport protein CorA"
"Chl_C14_00123" 1688 "s59E5H2" 0 "Magnesium transport protein CorA"
"Chl_C14_00123" 1688 "s63E6H2" 0 "Magnesium transport protein CorA"
"Chl_C14_00124" 1689 "s59E13H2" 0 "hypothetical protein"
"Chl_C14_00124" 1689 "s59E25H2" 0 "hypothetical protein"
"Chl_C14_00124" 1689 "s59E5H2" 0 "hypothetical protein"
"Chl_C14_00124" 1689 "s63E6H2" 0 "hypothetical protein"
"Chl_C14_00125" 1690 "s59E13H2" 0 "ribonuclease Z"
"Chl_C14_00125" 1690 "s59E25H2" 0 "ribonuclease Z"
"Chl_C14_00125" 1690 "s59E5H2" 0 "ribonuclease Z"
"Chl_C14_00125" 1690 "s63E6H2" 0 "ribonuclease Z"
"Chl_C14_00126" 1691 "s59E13H2" 0 "hypothetical protein"
"Chl_C14_00126" 1691 "s59E25H2" 0 "hypothetical protein"
"Chl_C14_00126" 1691 "s59E5H2" 0 "hypothetical protein"
"Chl_C14_00126" 1691 "s63E6H2" 0 "hypothetical protein"
"Chl_C14_00127" 1692 "s59E13H2" 0 "Bifunctional NAD(P)H-hydrate repair enzyme Nnr"
"Chl_C14_00127" 1692 "s59E25H2" 0 "Bifunctional NAD(P)H-hydrate repair enzyme Nnr"
"Chl_C14_00127" 1692 "s59E5H2" 0 "Bifunctional NAD(P)H-hydrate repair enzyme Nnr"
"Chl_C14_00127" 1692 "s63E6H2" 0 "Bifunctional NAD(P)H-hydrate repair enzyme Nnr"
"Chl_C14_00128" 1693 "s59E13H2" 0 "Type III pantothenate kinase"
"Chl_C14_00128" 1693 "s59E25H2" 0 "Type III pantothenate kinase"
"Chl_C14_00128" 1693 "s59E5H2" 0 "Type III pantothenate kinase"
"Chl_C14_00128" 1693 "s63E6H2" 0 "Type III pantothenate kinase"
"Chl_C14_00129" 1694 "s59E13H2" 0 "Coenzyme A biosynthesis bifunctional protein CoaBC"
"Chl_C14_00129" 1694 "s59E25H2" 0 "Coenzyme A biosynthesis bifunctional protein CoaBC"
"Chl_C14_00129" 1694 "s59E5H2" 0 "Coenzyme A biosynthesis bifunctional protein CoaBC"
"Chl_C14_00129" 1694 "s63E6H2" 0 "Coenzyme A biosynthesis bifunctional protein CoaBC"
"Chl_C14_00132" 1697 "s59E13H2" 0 "Valine--tRNA ligase"
"Chl_C14_00132" 1697 "s59E25H2" 0 "Valine--tRNA ligase"
"Chl_C14_00132" 1697 "s59E5H2" 0 "Valine--tRNA ligase"
"Chl_C14_00132" 1697 "s63E6H2" 0 "Valine--tRNA ligase"
"Chl_C14_00133" 1698 "s59E13H2" 0 "Universal stress protein"
"Chl_C14_00133" 1698 "s59E25H2" 0 "Universal stress protein"
"Chl_C14_00133" 1698 "s59E5H2" 0 "Universal stress protein"
"Chl_C14_00133" 1698 "s63E6H2" 0 "Universal stress protein"
"Chl_C14_00135" 1700 "s59E13H2" 0 "NAD-dependent methanol dehydrogenase"
"Chl_C14_00135" 1700 "s59E25H2" 0 "NAD-dependent methanol dehydrogenase"
"Chl_C14_00135" 1700 "s59E5H2" 0 "NAD-dependent methanol dehydrogenase"
"Chl_C14_00135" 1700 "s63E6H2" 0 "NAD-dependent methanol dehydrogenase"
"Chl_C14_00136" 1701 "s59E13H2" 0 "hypothetical protein"
"Chl_C14_00136" 1701 "s59E25H2" 0 "hypothetical protein"
"Chl_C14_00136" 1701 "s59E5H2" 0 "hypothetical protein"

"Chl_C14_00136" 1701 "s63E6H2" 0 "hypothetical protein"
"Chl_C14_00137" 1702 "s59E13H2" 0 "Isopropylmalate/citramalate isomerase small subunit"
"Chl_C14_00137" 1702 "s59E25H2" 0 "Isopropylmalate/citramalate isomerase small subunit"
"Chl_C14_00137" 1702 "s59E5H2" 0 "Isopropylmalate/citramalate isomerase small subunit"
"Chl_C14_00137" 1702 "s63E6H2" 0 "Isopropylmalate/citramalate isomerase small subunit"
"Chl_C14_00138" 1703 "s59E13H2" 0 "Isopropylmalate/citramalate isomerase large subunit"
"Chl_C14_00138" 1703 "s59E25H2" 0 "Isopropylmalate/citramalate isomerase large subunit"
"Chl_C14_00138" 1703 "s59E5H2" 0 "Isopropylmalate/citramalate isomerase large subunit"
"Chl_C14_00138" 1703 "s63E6H2" 0 "Isopropylmalate/citramalate isomerase large subunit"
"Chl_C14_00139" 1704 "s59E13H2" 0 "D-gluconate dehydratase"
"Chl_C14_00139" 1704 "s59E25H2" 0 "D-gluconate dehydratase"
"Chl_C14_00139" 1704 "s59E5H2" 0 "D-gluconate dehydratase"
"Chl_C14_00139" 1704 "s63E6H2" 0 "D-gluconate dehydratase"
"Chl_C14_00140" 1705 "s59E13H2" 0 "D-gluconate dehydratase"
"Chl_C14_00140" 1705 "s59E25H2" 0 "D-gluconate dehydratase"
"Chl_C14_00140" 1705 "s59E5H2" 0 "D-gluconate dehydratase"
"Chl_C14_00140" 1705 "s63E6H2" 0 "D-gluconate dehydratase"
"Chl_C14_00141" 1706 "s59E13H2" 0 "Thioredoxin reductase"
"Chl_C14_00141" 1706 "s59E25H2" 0 "Thioredoxin reductase"
"Chl_C14_00141" 1706 "s59E5H2" 0 "Thioredoxin reductase"
"Chl_C14_00141" 1706 "s63E6H2" 0 "Thioredoxin reductase"
"Chl_C14_00142" 1707 "s59E13H2" 0 "hypothetical protein"
"Chl_C14_00142" 1707 "s59E25H2" 0 "hypothetical protein"
"Chl_C14_00142" 1707 "s59E5H2" 0 "hypothetical protein"
"Chl_C14_00142" 1707 "s63E6H2" 0 "hypothetical protein"
"Chl_C14_00144" 1709 "s59E13H2" 0 "Tetrathionate reductase subunit B precursor"
"Chl_C14_00144" 1709 "s59E25H2" 0 "Tetrathionate reductase subunit B precursor"
"Chl_C14_00144" 1709 "s59E5H2" 0.03879236 "Tetrathionate reductase subunit B precursor"
"Chl_C14_00144" 1709 "s63E6H2" 0 "Tetrathionate reductase subunit B precursor"
"Chl_C14_00145" 1710 "s59E13H2" 0 "Acetylene hydratase"
"Chl_C14_00145" 1710 "s59E25H2" 0 "Acetylene hydratase"
"Chl_C14_00145" 1710 "s59E5H2" 0 "Acetylene hydratase"
"Chl_C14_00145" 1710 "s63E6H2" 0 "Acetylene hydratase"
"Chl_C14_00146" 1711 "s59E13H2" 0 "Xaa-Pro dipeptidase"
"Chl_C14_00146" 1711 "s59E25H2" 0 "Xaa-Pro dipeptidase"
"Chl_C14_00146" 1711 "s59E5H2" 0 "Xaa-Pro dipeptidase"
"Chl_C14_00146" 1711 "s63E6H2" 0 "Xaa-Pro dipeptidase"
"Chl_C14_00147" 1712 "s59E13H2" 0 "Xaa-Pro dipeptidase"
"Chl_C14_00147" 1712 "s59E25H2" 0 "Xaa-Pro dipeptidase"
"Chl_C14_00147" 1712 "s59E5H2" 0 "Xaa-Pro dipeptidase"
"Chl_C14_00147" 1712 "s63E6H2" 0 "Xaa-Pro dipeptidase"
"Chl_C14_00148" 1713 "s59E13H2" 0 "NMT1/THI5 like protein"
"Chl_C14_00148" 1713 "s59E25H2" 0 "NMT1/THI5 like protein"
"Chl_C14_00148" 1713 "s59E5H2" 0 "NMT1/THI5 like protein"
"Chl_C14_00148" 1713 "s63E6H2" 0 "NMT1/THI5 like protein"
"Chl_C14_00149" 1714 "s59E13H2" 0 "Sialic acid TRAP transporter permease protein SiaT"
"Chl_C14_00149" 1714 "s59E25H2" 0 "Sialic acid TRAP transporter permease protein SiaT"

"Chl_C14_00149" 1714 "s59E5H2" 0 "Sialic acid TRAP transporter permease protein SiaT"
"Chl_C14_00149" 1714 "s63E6H2" 0 "Sialic acid TRAP transporter permease protein SiaT"
"Chl_C14_00150" 1715 "s59E13H2" 0 "hypothetical protein"
"Chl_C14_00150" 1715 "s59E25H2" 0 "hypothetical protein"
"Chl_C14_00150" 1715 "s59E5H2" 0 "hypothetical protein"
"Chl_C14_00150" 1715 "s63E6H2" 0 "hypothetical protein"
"Chl_C14_00151" 1716 "s59E13H2" 0 "EthD protein"
"Chl_C14_00151" 1716 "s59E25H2" 0 "EthD protein"
"Chl_C14_00151" 1716 "s59E5H2" 0 "EthD protein"
"Chl_C14_00151" 1716 "s63E6H2" 0 "EthD protein"
"Chl_C14_00152" 1717 "s59E13H2" 0 "2-hydroxymuconate tautomerase"
"Chl_C14_00152" 1717 "s59E25H2" 0 "2-hydroxymuconate tautomerase"
"Chl_C14_00152" 1717 "s59E5H2" 0 "2-hydroxymuconate tautomerase"
"Chl_C14_00152" 1717 "s63E6H2" 0 "2-hydroxymuconate tautomerase"
"Chl_C14_00153" 1718 "s59E13H2" 0 "metal-dependent hydrolase"
"Chl_C14_00153" 1718 "s59E25H2" 0 "metal-dependent hydrolase"
"Chl_C14_00153" 1718 "s59E5H2" 0 "metal-dependent hydrolase"
"Chl_C14_00153" 1718 "s63E6H2" 0 "metal-dependent hydrolase"
"Chl_C14_00154" 1719 "s59E13H2" 0 "Digeranylgeranylglycerophospholipid reductase"
"Chl_C14_00154" 1719 "s59E25H2" 0 "Digeranylgeranylglycerophospholipid reductase"
"Chl_C14_00154" 1719 "s59E5H2" 0 "Digeranylgeranylglycerophospholipid reductase"
"Chl_C14_00154" 1719 "s63E6H2" 0 "Digeranylgeranylglycerophospholipid reductase"
"Chl_C14_00155" 1720 "s59E13H2" 0 "2-methylcitrate dehydratase"
"Chl_C14_00155" 1720 "s59E25H2" 0 "2-methylcitrate dehydratase"
"Chl_C14_00155" 1720 "s59E5H2" 0 "2-methylcitrate dehydratase"
"Chl_C14_00155" 1720 "s63E6H2" 0 "2-methylcitrate dehydratase"
"Chl_C14_00156" 1721 "s59E13H2" 0 "Tungsten-containing aldehyde ferredoxin oxidoreductase"
"Chl_C14_00156" 1721 "s59E25H2" 0 "Tungsten-containing aldehyde ferredoxin oxidoreductase"
"Chl_C14_00156" 1721 "s59E5H2" 0 "Tungsten-containing aldehyde ferredoxin oxidoreductase"
"Chl_C14_00156" 1721 "s63E6H2" 0 "Tungsten-containing aldehyde ferredoxin oxidoreductase"
"Chl_C14_00157" 1722 "s59E13H2" 0 "Putative redox-active protein (C_GCAxxG_C_C)"
"Chl_C14_00157" 1722 "s59E25H2" 0 "Putative redox-active protein (C_GCAxxG_C_C)"
"Chl_C14_00157" 1722 "s59E5H2" 0 "Putative redox-active protein (C_GCAxxG_C_C)"
"Chl_C14_00157" 1722 "s63E6H2" 0 "Putative redox-active protein (C_GCAxxG_C_C)"
"Chl_C14_00158" 1723 "s59E13H2" 0 "2-methylcitrate dehydratase"
"Chl_C14_00158" 1723 "s59E25H2" 0 "2-methylcitrate dehydratase"
"Chl_C14_00158" 1723 "s59E5H2" 0 "2-methylcitrate dehydratase"
"Chl_C14_00158" 1723 "s63E6H2" 0 "2-methylcitrate dehydratase"
"Chl_C14_00159" 1724 "s59E13H2" 0 "hypothetical protein"
"Chl_C14_00159" 1724 "s59E25H2" 0 "hypothetical protein"
"Chl_C14_00159" 1724 "s59E5H2" 0 "hypothetical protein"
"Chl_C14_00159" 1724 "s63E6H2" 0 "hypothetical protein"
"Chl_C14_00160" 1725 "s59E13H2" 0 "hypothetical protein"
"Chl_C14_00160" 1725 "s59E25H2" 0 "hypothetical protein"
"Chl_C14_00160" 1725 "s59E5H2" 0 "hypothetical protein"
"Chl_C14_00160" 1725 "s63E6H2" 0 "hypothetical protein"
"Chl_C14_00161" 1726 "s59E13H2" 0 "CoB--CoM heterodisulfide reductase iron-sulfur subunit A"

"Chl_C14_00161" 1726 "s59E25H2" 0 "CoB--CoM heterodisulfide reductase iron-sulfur subunit A"
"Chl_C14_00161" 1726 "s59E5H2" 0 "CoB--CoM heterodisulfide reductase iron-sulfur subunit A"
"Chl_C14_00161" 1726 "s63E6H2" 0 "CoB--CoM heterodisulfide reductase iron-sulfur subunit A"
"Chl_C14_00162" 1727 "s59E13H2" 0 "Light-independent protochlorophyllide reductase iron-sulfur ATP-binding protein"
"Chl_C14_00162" 1727 "s59E25H2" 0 "Light-independent protochlorophyllide reductase iron-sulfur ATP-binding protein"
"Chl_C14_00162" 1727 "s59E5H2" 0 "Light-independent protochlorophyllide reductase iron-sulfur ATP-binding protein"
"Chl_C14_00162" 1727 "s63E6H2" 0.0007165703 "Light-independent protochlorophyllide reductase iron-sulfur ATP-binding protein"
"Chl_C14_00163" 1728 "s59E13H2" 0 "Formate--tetrahydrofolate ligase"
"Chl_C14_00163" 1728 "s59E25H2" 0 "Formate--tetrahydrofolate ligase"
"Chl_C14_00163" 1728 "s59E5H2" 0 "Formate--tetrahydrofolate ligase"
"Chl_C14_00163" 1728 "s63E6H2" 0.002191853 "Formate--tetrahydrofolate ligase"
"Chl_C14_00164" 1729 "s59E13H2" 0 "CobQ/CobB/MinD/ParA nucleotide binding domain protein"
"Chl_C14_00164" 1729 "s59E25H2" 0 "CobQ/CobB/MinD/ParA nucleotide binding domain protein"
"Chl_C14_00164" 1729 "s59E5H2" 0 "CobQ/CobB/MinD/ParA nucleotide binding domain protein"
"Chl_C14_00164" 1729 "s63E6H2" 0 "CobQ/CobB/MinD/ParA nucleotide binding domain protein"
"Chl_C14_00165" 1730 "s59E13H2" 0 "Bifunctional protein FolD protein"
"Chl_C14_00165" 1730 "s59E25H2" 0 "Bifunctional protein FolD protein"
"Chl_C14_00165" 1730 "s59E5H2" 0 "Bifunctional protein FolD protein"
"Chl_C14_00165" 1730 "s63E6H2" 0 "Bifunctional protein FolD protein"
"Chl_C14_00167" 1732 "s59E13H2" 0 "Corrinoid/iron-sulfur protein small subunit"
"Chl_C14_00167" 1732 "s59E25H2" 0 "Corrinoid/iron-sulfur protein small subunit"
"Chl_C14_00167" 1732 "s59E5H2" 0 "Corrinoid/iron-sulfur protein small subunit"
"Chl_C14_00167" 1732 "s63E6H2" 0 "Corrinoid/iron-sulfur protein small subunit"
"Chl_C14_00168" 1733 "s59E13H2" 0 "Carbon monoxide dehydrogenase 1"
"Chl_C14_00168" 1733 "s59E25H2" 0 "Carbon monoxide dehydrogenase 1"
"Chl_C14_00168" 1733 "s59E5H2" 0 "Carbon monoxide dehydrogenase 1"
"Chl_C14_00168" 1733 "s63E6H2" 0 "Carbon monoxide dehydrogenase 1"
"Chl_C14_00169" 1734 "s59E13H2" 0.001036409 "Carbon monoxide dehydrogenase/acetyl-CoA synthase subunit alpha"
"Chl_C14_00169" 1734 "s59E25H2" 0 "Carbon monoxide dehydrogenase/acetyl-CoA synthase subunit alpha"
"Chl_C14_00169" 1734 "s59E5H2" 0 "Carbon monoxide dehydrogenase/acetyl-CoA synthase subunit alpha"
"Chl_C14_00169" 1734 "s63E6H2" 0 "Carbon monoxide dehydrogenase/acetyl-CoA synthase subunit alpha"
"Chl_C14_00170" 1735 "s59E13H2" 0 "Corrinoid/iron-sulfur protein large subunit"
"Chl_C14_00170" 1735 "s59E25H2" 0 "Corrinoid/iron-sulfur protein large subunit"
"Chl_C14_00170" 1735 "s59E5H2" 0 "Corrinoid/iron-sulfur protein large subunit"
"Chl_C14_00170" 1735 "s63E6H2" 0.02285037 "Corrinoid/iron-sulfur protein large subunit"
"Chl_C14_00171" 1736 "s59E13H2" 0 "5-methyltetrahydrofolate:corrinoid/iron-sulfur protein co-methyltransferase"
"Chl_C14_00171" 1736 "s59E25H2" 0 "5-methyltetrahydrofolate:corrinoid/iron-sulfur protein co-methyltransferase"
"Chl_C14_00171" 1736 "s59E5H2" 0 "5-methyltetrahydrofolate:corrinoid/iron-sulfur protein co-methyltransferase"
"Chl_C14_00171" 1736 "s63E6H2" 0.008565322 "5-methyltetrahydrofolate:corrinoid/iron-sulfur protein co-methyltransferase"
"Chl_C14_00172" 1737 "s59E13H2" 0 "hypothetical protein"
"Chl_C14_00172" 1737 "s59E25H2" 0 "hypothetical protein"
"Chl_C14_00172" 1737 "s59E5H2" 0 "hypothetical protein"
"Chl_C14_00172" 1737 "s63E6H2" 0 "hypothetical protein"
"Chl_C14_00173" 1738 "s59E13H2" 0 "D-tyrosyl-tRNA(Tyr) deacylase"
"Chl_C14_00173" 1738 "s59E25H2" 0 "D-tyrosyl-tRNA(Tyr) deacylase"
"Chl_C14_00173" 1738 "s59E5H2" 0 "D-tyrosyl-tRNA(Tyr) deacylase"
"Chl_C14_00173" 1738 "s63E6H2" 0 "D-tyrosyl-tRNA(Tyr) deacylase"

"Chl_C14_00174" 1739 "s59E13H2" 0 "hydroxyacylglutathione hydrolase"
"Chl_C14_00174" 1739 "s59E25H2" 0 "hydroxyacylglutathione hydrolase"
"Chl_C14_00174" 1739 "s59E5H2" 0 "hydroxyacylglutathione hydrolase"
"Chl_C14_00174" 1739 "s63E6H2" 0 "hydroxyacylglutathione hydrolase"
"Chl_C14_00175" 1740 "s59E13H2" 0 "Ferric uptake regulation protein"
"Chl_C14_00175" 1740 "s59E25H2" 0 "Ferric uptake regulation protein"
"Chl_C14_00175" 1740 "s59E5H2" 0 "Ferric uptake regulation protein"
"Chl_C14_00175" 1740 "s63E6H2" 0 "Ferric uptake regulation protein"
"Chl_C14_00176" 1741 "s59E13H2" 0 "putative periplasmic iron-binding protein precursor"
"Chl_C14_00176" 1741 "s59E25H2" 0 "putative periplasmic iron-binding protein precursor"
"Chl_C14_00176" 1741 "s59E5H2" 0.006132097 "putative periplasmic iron-binding protein precursor"
"Chl_C14_00176" 1741 "s63E6H2" 0.0004024116 "putative periplasmic iron-binding protein precursor"
"Chl_C14_00177" 1742 "s59E13H2" 0 "Iron(3+)-hydroxamate import ATP-binding protein FhuC"
"Chl_C14_00177" 1742 "s59E25H2" 0 "Iron(3+)-hydroxamate import ATP-binding protein FhuC"
"Chl_C14_00177" 1742 "s59E5H2" 0 "Iron(3+)-hydroxamate import ATP-binding protein FhuC"
"Chl_C14_00177" 1742 "s63E6H2" 0.009568817 "Iron(3+)-hydroxamate import ATP-binding protein FhuC"
"Chl_C14_00178" 1743 "s59E13H2" 0 "High-affinity zinc uptake system membrane protein ZnuB"
"Chl_C14_00178" 1743 "s59E25H2" 0.1170213 "High-affinity zinc uptake system membrane protein ZnuB"
"Chl_C14_00178" 1743 "s59E5H2" 0 "High-affinity zinc uptake system membrane protein ZnuB"
"Chl_C14_00178" 1743 "s63E6H2" 0.04024835 "High-affinity zinc uptake system membrane protein ZnuB"
"Chl_C14_00179" 1744 "s59E13H2" 0 "Ubiquinone/menaquinone biosynthesis C-methyltransferase UbiE"
"Chl_C14_00179" 1744 "s59E25H2" 0 "Ubiquinone/menaquinone biosynthesis C-methyltransferase UbiE"
"Chl_C14_00179" 1744 "s59E5H2" 0 "Ubiquinone/menaquinone biosynthesis C-methyltransferase UbiE"
"Chl_C14_00179" 1744 "s63E6H2" 0.01367591 "Ubiquinone/menaquinone biosynthesis C-methyltransferase UbiE"
"Chl_C14_00180" 1745 "s59E13H2" 0 "putative nicotinate-nucleotide pyrophosphorylase [carboxylating]"
"Chl_C14_00180" 1745 "s59E25H2" 0 "putative nicotinate-nucleotide pyrophosphorylase [carboxylating]"
"Chl_C14_00180" 1745 "s59E5H2" 0 "putative nicotinate-nucleotide pyrophosphorylase [carboxylating]"
"Chl_C14_00180" 1745 "s63E6H2" 0.008588203 "putative nicotinate-nucleotide pyrophosphorylase [carboxylating]"
"Chl_C14_00181" 1746 "s59E13H2" 0 "L-aspartate oxidase"
"Chl_C14_00181" 1746 "s59E25H2" 0 "L-aspartate oxidase"
"Chl_C14_00181" 1746 "s59E5H2" 0.005152078 "L-aspartate oxidase"
"Chl_C14_00181" 1746 "s63E6H2" 0 "L-aspartate oxidase"
"Chl_C14_00182" 1747 "s59E13H2" 0 "Quinolinate synthase A"
"Chl_C14_00182" 1747 "s59E25H2" 0 "Quinolinate synthase A"
"Chl_C14_00182" 1747 "s59E5H2" 0 "Quinolinate synthase A"
"Chl_C14_00182" 1747 "s63E6H2" 0.01229856 "Quinolinate synthase A"
"Chl_C14_00183" 1748 "s59E13H2" 0 "preprotein translocase subunit YajC"
"Chl_C14_00183" 1748 "s59E25H2" 0 "preprotein translocase subunit YajC"
"Chl_C14_00183" 1748 "s59E5H2" 0 "preprotein translocase subunit YajC"
"Chl_C14_00183" 1748 "s63E6H2" 0 "preprotein translocase subunit YajC"
"Chl_C14_00184" 1749 "s59E13H2" 0 "(Dimethylallyl)adenosine tRNA methylthiotransferase MiaB"
"Chl_C14_00184" 1749 "s59E25H2" 0 "(Dimethylallyl)adenosine tRNA methylthiotransferase MiaB"
"Chl_C14_00184" 1749 "s59E5H2" 0 "(Dimethylallyl)adenosine tRNA methylthiotransferase MiaB"
"Chl_C14_00184" 1749 "s63E6H2" 0 "(Dimethylallyl)adenosine tRNA methylthiotransferase MiaB"
"Chl_C14_00186" 1751 "s59E13H2" 0 "NTE family protein RssA"
"Chl_C14_00186" 1751 "s59E25H2" 0 "NTE family protein RssA"
"Chl_C14_00186" 1751 "s59E5H2" 0 "NTE family protein RssA"

"Chl_C14_00186" 1751 "s63E6H2" 0 "NTE family protein RssA"
"Chl_C14_00187" 1752 "s59E13H2" 0 "Endoribonuclease YbeY"
"Chl_C14_00187" 1752 "s59E25H2" 0 "Endoribonuclease YbeY"
"Chl_C14_00187" 1752 "s59E5H2" 0 "Endoribonuclease YbeY"
"Chl_C14_00187" 1752 "s63E6H2" 0 "Endoribonuclease YbeY"
"Chl_C14_00188" 1753 "s59E13H2" 0 "Iron-sulfur flavoprotein"
"Chl_C14_00188" 1753 "s59E25H2" 0 "Iron-sulfur flavoprotein"
"Chl_C14_00188" 1753 "s59E5H2" 0 "Iron-sulfur flavoprotein"
"Chl_C14_00188" 1753 "s63E6H2" 0 "Iron-sulfur flavoprotein"
"Chl_C14_00189" 1754 "s59E13H2" 0 "Ferredoxin-2"
"Chl_C14_00189" 1754 "s59E25H2" 0 "Ferredoxin-2"
"Chl_C14_00189" 1754 "s59E5H2" 0 "Ferredoxin-2"
"Chl_C14_00189" 1754 "s63E6H2" 0 "Ferredoxin-2"
"Chl_C14_00190" 1755 "s59E13H2" 0 "hypothetical protein"
"Chl_C14_00190" 1755 "s59E25H2" 0 "hypothetical protein"
"Chl_C14_00190" 1755 "s59E5H2" 0 "hypothetical protein"
"Chl_C14_00190" 1755 "s63E6H2" 0 "hypothetical protein"
"Chl_C14_00191" 1756 "s59E13H2" 0 "cAMP receptor protein"
"Chl_C14_00191" 1756 "s59E25H2" 0 "cAMP receptor protein"
"Chl_C14_00191" 1756 "s59E5H2" 0.01185528 "cAMP receptor protein"
"Chl_C14_00191" 1756 "s63E6H2" 0 "cAMP receptor protein"
"Chl_C14_00192" 1757 "s59E13H2" 0 "TspO/MBR family protein"
"Chl_C14_00192" 1757 "s59E25H2" 0 "TspO/MBR family protein"
"Chl_C14_00192" 1757 "s59E5H2" 0 "TspO/MBR family protein"
"Chl_C14_00192" 1757 "s63E6H2" 0 "TspO/MBR family protein"
"Chl_C14_00193" 1758 "s59E13H2" 0 "Hydroxyacylglutathione hydrolase"
"Chl_C14_00193" 1758 "s59E25H2" 0 "Hydroxyacylglutathione hydrolase"
"Chl_C14_00193" 1758 "s59E5H2" 0 "Hydroxyacylglutathione hydrolase"
"Chl_C14_00193" 1758 "s63E6H2" 0 "Hydroxyacylglutathione hydrolase"
"Chl_C14_00195" 1760 "s59E13H2" 0 "Glucokinase"
"Chl_C14_00195" 1760 "s59E25H2" 0 "Glucokinase"
"Chl_C14_00195" 1760 "s59E5H2" 0 "Glucokinase"
"Chl_C14_00195" 1760 "s63E6H2" 0 "Glucokinase"
"Chl_C14_00196" 1761 "s59E13H2" 0 "UTP--glucose-1-phosphate uridylyltransferase"
"Chl_C14_00196" 1761 "s59E25H2" 0 "UTP--glucose-1-phosphate uridylyltransferase"
"Chl_C14_00196" 1761 "s59E5H2" 0 "UTP--glucose-1-phosphate uridylyltransferase"
"Chl_C14_00196" 1761 "s63E6H2" 0 "UTP--glucose-1-phosphate uridylyltransferase"
"Chl_C14_00197" 1762 "s59E13H2" 0 "Alanine--tRNA ligase"
"Chl_C14_00197" 1762 "s59E25H2" 0 "Alanine--tRNA ligase"
"Chl_C14_00197" 1762 "s59E5H2" 0 "Alanine--tRNA ligase"
"Chl_C14_00197" 1762 "s63E6H2" 0 "Alanine--tRNA ligase"
"Chl_C14_00198" 1763 "s59E13H2" 0 "Putative Holliday junction resolvase"
"Chl_C14_00198" 1763 "s59E25H2" 0 "Putative Holliday junction resolvase"
"Chl_C14_00198" 1763 "s59E5H2" 0 "Putative Holliday junction resolvase"
"Chl_C14_00198" 1763 "s63E6H2" 0 "Putative Holliday junction resolvase"
"Chl_C14_00199" 1764 "s59E13H2" 0 "HPP family protein"
"Chl_C14_00199" 1764 "s59E25H2" 0 "HPP family protein"

"Chl_C14_00199" 1764 "s59E5H2" 0 "HPP family protein"
"Chl_C14_00199" 1764 "s63E6H2" 0 "HPP family protein"
"Chl_C14_00200" 1765 "s59E13H2" 0 "Protoheme IX farnesyltransferase 1"
"Chl_C14_00200" 1765 "s59E25H2" 0 "Protoheme IX farnesyltransferase 1"
"Chl_C14_00200" 1765 "s59E5H2" 0 "Protoheme IX farnesyltransferase 1"
"Chl_C14_00200" 1765 "s63E6H2" 0 "Protoheme IX farnesyltransferase 1"
"Chl_C14_00201" 1766 "s59E13H2" 0.000611998 "Queuine tRNA-ribosyltransferase"
"Chl_C14_00201" 1766 "s59E25H2" 0 "Queuine tRNA-ribosyltransferase"
"Chl_C14_00201" 1766 "s59E5H2" 0 "Queuine tRNA-ribosyltransferase"
"Chl_C14_00201" 1766 "s63E6H2" 0.001280663 "Queuine tRNA-ribosyltransferase"
"Chl_C14_00202" 1767 "s59E13H2" 0 "NIL domain protein"
"Chl_C14_00202" 1767 "s59E25H2" 0 "NIL domain protein"
"Chl_C14_00202" 1767 "s59E5H2" 0 "NIL domain protein"
"Chl_C14_00202" 1767 "s63E6H2" 0 "NIL domain protein"
"Chl_C14_00203" 1768 "s59E13H2" 0 "Nitronate monooxygenase"
"Chl_C14_00203" 1768 "s59E25H2" 0 "Nitronate monooxygenase"
"Chl_C14_00203" 1768 "s59E5H2" 0 "Nitronate monooxygenase"
"Chl_C14_00203" 1768 "s63E6H2" 0 "Nitronate monooxygenase"
"Chl_C14_00204" 1769 "s59E13H2" 0 "Sulfur carrier protein CysO"
"Chl_C14_00204" 1769 "s59E25H2" 0 "Sulfur carrier protein CysO"
"Chl_C14_00204" 1769 "s59E5H2" 0 "Sulfur carrier protein CysO"
"Chl_C14_00204" 1769 "s63E6H2" 0 "Sulfur carrier protein CysO"
"Chl_C14_00205" 1770 "s59E13H2" 0 "putative adenylyltransferase"
"Chl_C14_00205" 1770 "s59E25H2" 0 "putative adenylyltransferase"
"Chl_C14_00205" 1770 "s59E5H2" 0 "putative adenylyltransferase"
"Chl_C14_00205" 1770 "s63E6H2" 0 "putative adenylyltransferase"
"Chl_C14_00206" 1771 "s59E13H2" 0 "Divalent-cation tolerance protein CutA"
"Chl_C14_00206" 1771 "s59E25H2" 0 "Divalent-cation tolerance protein CutA"
"Chl_C14_00206" 1771 "s59E5H2" 0 "Divalent-cation tolerance protein CutA"
"Chl_C14_00206" 1771 "s63E6H2" 0 "Divalent-cation tolerance protein CutA"
"Chl_C14_00207" 1772 "s59E13H2" 0 "formate dehydrogenase accessory protein"
"Chl_C14_00207" 1772 "s59E25H2" 0 "formate dehydrogenase accessory protein"
"Chl_C14_00207" 1772 "s59E5H2" 0 "formate dehydrogenase accessory protein"
"Chl_C14_00207" 1772 "s63E6H2" 0 "formate dehydrogenase accessory protein"
"Chl_C14_00208" 1773 "s59E13H2" 0 "MOSC domain protein"
"Chl_C14_00208" 1773 "s59E25H2" 0 "MOSC domain protein"
"Chl_C14_00208" 1773 "s59E5H2" 0 "MOSC domain protein"
"Chl_C14_00208" 1773 "s63E6H2" 0 "MOSC domain protein"
"Chl_C14_00209" 1774 "s59E13H2" 0 "putative permease"
"Chl_C14_00209" 1774 "s59E25H2" 0 "putative permease"
"Chl_C14_00209" 1774 "s59E5H2" 0 "putative permease"
"Chl_C14_00209" 1774 "s63E6H2" 0 "putative permease"
"Chl_C14_00210" 1775 "s59E13H2" 0 "putative methyltransferase YcgJ"
"Chl_C14_00210" 1775 "s59E25H2" 0 "putative methyltransferase YcgJ"
"Chl_C14_00210" 1775 "s59E5H2" 0 "putative methyltransferase YcgJ"
"Chl_C14_00210" 1775 "s63E6H2" 0 "putative methyltransferase YcgJ"
"Chl_C14_00211" 1776 "s59E13H2" 0 "hypothetical protein"

"Chl_C14_00211" 1776 "s59E25H2" 0 "hypothetical protein"
"Chl_C14_00211" 1776 "s59E5H2" 0 "hypothetical protein"
"Chl_C14_00211" 1776 "s63E6H2" 0 "hypothetical protein"
"Chl_C14_00212" 1777 "s59E13H2" 0 "PKD domain protein"
"Chl_C14_00212" 1777 "s59E25H2" 0 "PKD domain protein"
"Chl_C14_00212" 1777 "s59E5H2" 0 "PKD domain protein"
"Chl_C14_00212" 1777 "s63E6H2" 0 "PKD domain protein"
"Chl_C14_00213" 1778 "s59E13H2" 0 "DoxX"
"Chl_C14_00213" 1778 "s59E25H2" 0 "DoxX"
"Chl_C14_00213" 1778 "s59E5H2" 0 "DoxX"
"Chl_C14_00213" 1778 "s63E6H2" 0 "DoxX"
"Chl_C14_00214" 1779 "s59E13H2" 0 "hypothetical protein"
"Chl_C14_00214" 1779 "s59E25H2" 0 "hypothetical protein"
"Chl_C14_00214" 1779 "s59E5H2" 0 "hypothetical protein"
"Chl_C14_00214" 1779 "s63E6H2" 0 "hypothetical protein"
"Chl_C14_00215" 1780 "s59E13H2" 0 "Ferredoxin"
"Chl_C14_00215" 1780 "s59E25H2" 0 "Ferredoxin"
"Chl_C14_00215" 1780 "s59E5H2" 0 "Ferredoxin"
"Chl_C14_00215" 1780 "s63E6H2" 0 "Ferredoxin"
"Chl_C14_00216" 1781 "s59E13H2" 0 "hypothetical protein"
"Chl_C14_00216" 1781 "s59E25H2" 0 "hypothetical protein"
"Chl_C14_00216" 1781 "s59E5H2" 0 "hypothetical protein"
"Chl_C14_00216" 1781 "s63E6H2" 0 "hypothetical protein"
"Chl_C14_00217" 1782 "s59E13H2" 0 "Aspartate--tRNA ligase"
"Chl_C14_00217" 1782 "s59E25H2" 0 "Aspartate--tRNA ligase"
"Chl_C14_00217" 1782 "s59E5H2" 0 "Aspartate--tRNA ligase"
"Chl_C14_00217" 1782 "s63E6H2" 0 "Aspartate--tRNA ligase"
"Chl_C14_00218" 1783 "s59E13H2" 0 "Trigger factor"
"Chl_C14_00218" 1783 "s59E25H2" 0 "Trigger factor"
"Chl_C14_00218" 1783 "s59E5H2" 0 "Trigger factor"
"Chl_C14_00218" 1783 "s63E6H2" 0 "Trigger factor"
"Chl_C14_00219" 1784 "s59E13H2" 0 "ATP-dependent Clp protease proteolytic subunit"
"Chl_C14_00219" 1784 "s59E25H2" 0 "ATP-dependent Clp protease proteolytic subunit"
"Chl_C14_00219" 1784 "s59E5H2" 0 "ATP-dependent Clp protease proteolytic subunit"
"Chl_C14_00219" 1784 "s63E6H2" 0.001570726 "ATP-dependent Clp protease proteolytic subunit"
"Chl_C14_00220" 1785 "s59E13H2" 0 "putative sugar kinase"
"Chl_C14_00220" 1785 "s59E25H2" 0 "putative sugar kinase"
"Chl_C14_00220" 1785 "s59E5H2" 0 "putative sugar kinase"
"Chl_C14_00220" 1785 "s63E6H2" 0 "putative sugar kinase"
"Chl_C14_00221" 1786 "s59E13H2" 0 "phosphoserine phosphatase"
"Chl_C14_00221" 1786 "s59E25H2" 0 "phosphoserine phosphatase"
"Chl_C14_00221" 1786 "s59E5H2" 0 "phosphoserine phosphatase"
"Chl_C14_00221" 1786 "s63E6H2" 0 "phosphoserine phosphatase"
"Chl_C14_00222" 1787 "s59E13H2" 0 "hypothetical protein"
"Chl_C14_00222" 1787 "s59E25H2" 0 "hypothetical protein"
"Chl_C14_00222" 1787 "s59E5H2" 0 "hypothetical protein"
"Chl_C14_00222" 1787 "s63E6H2" 0 "hypothetical protein"

"Chl_C14_00223" 1788 "s59E13H2" 0 "Phosphoribosylglycinamide formyltransferase"
"Chl_C14_00223" 1788 "s59E25H2" 0 "Phosphoribosylglycinamide formyltransferase"
"Chl_C14_00223" 1788 "s59E5H2" 0 "Phosphoribosylglycinamide formyltransferase"
"Chl_C14_00223" 1788 "s63E6H2" 0 "Phosphoribosylglycinamide formyltransferase"
"Chl_C14_00224" 1789 "s59E13H2" 0 "PHP domain protein"
"Chl_C14_00224" 1789 "s59E25H2" 0 "PHP domain protein"
"Chl_C14_00224" 1789 "s59E5H2" 0 "PHP domain protein"
"Chl_C14_00224" 1789 "s63E6H2" 0 "PHP domain protein"
"Chl_C14_00225" 1790 "s59E13H2" 0 "Elongation factor P"
"Chl_C14_00225" 1790 "s59E25H2" 0 "Elongation factor P"
"Chl_C14_00225" 1790 "s59E5H2" 0 "Elongation factor P"
"Chl_C14_00225" 1790 "s63E6H2" 0 "Elongation factor P"
"Chl_C14_00226" 1791 "s59E13H2" 0 "Xaa-Pro dipeptidase"
"Chl_C14_00226" 1791 "s59E25H2" 0 "Xaa-Pro dipeptidase"
"Chl_C14_00226" 1791 "s59E5H2" 0 "Xaa-Pro dipeptidase"
"Chl_C14_00226" 1791 "s63E6H2" 0 "Xaa-Pro dipeptidase"
"Chl_C14_00227" 1792 "s59E13H2" 0 "Tyrosine recombinase XerC"
"Chl_C14_00227" 1792 "s59E25H2" 0 "Tyrosine recombinase XerC"
"Chl_C14_00227" 1792 "s59E5H2" 0 "Tyrosine recombinase XerC"
"Chl_C14_00227" 1792 "s63E6H2" 0 "Tyrosine recombinase XerC"
"Chl_C14_00228" 1793 "s59E13H2" 0 "DNA topoisomerase 1"
"Chl_C14_00228" 1793 "s59E25H2" 0 "DNA topoisomerase 1"
"Chl_C14_00228" 1793 "s59E5H2" 0.008299677 "DNA topoisomerase 1"
"Chl_C14_00228" 1793 "s63E6H2" 0 "DNA topoisomerase 1"
"Chl_C14_00229" 1794 "s59E13H2" 0 "hypothetical protein"
"Chl_C14_00229" 1794 "s59E25H2" 0 "hypothetical protein"
"Chl_C14_00229" 1794 "s59E5H2" 0 "hypothetical protein"
"Chl_C14_00229" 1794 "s63E6H2" 0 "hypothetical protein"
"Chl_C14_00230" 1795 "s59E13H2" 0 "hypothetical protein"
"Chl_C14_00230" 1795 "s59E25H2" 0 "hypothetical protein"
"Chl_C14_00230" 1795 "s59E5H2" 0 "hypothetical protein"
"Chl_C14_00230" 1795 "s63E6H2" 0 "hypothetical protein"
"Chl_C14_00231" 1796 "s59E13H2" 0 "hypothetical protein"
"Chl_C14_00231" 1796 "s59E25H2" 0 "hypothetical protein"
"Chl_C14_00231" 1796 "s59E5H2" 0 "hypothetical protein"
"Chl_C14_00231" 1796 "s63E6H2" 0 "hypothetical protein"
"Chl_C14_00232" 1797 "s59E13H2" 0 "hypothetical protein"
"Chl_C14_00232" 1797 "s59E25H2" 0 "hypothetical protein"
"Chl_C14_00232" 1797 "s59E5H2" 0 "hypothetical protein"
"Chl_C14_00232" 1797 "s63E6H2" 0 "hypothetical protein"
"Chl_C14_00235" 1800 "s59E13H2" 0 "Putative prophage phiRv2 integrase"
"Chl_C14_00235" 1800 "s59E25H2" 0 "Putative prophage phiRv2 integrase"
"Chl_C14_00235" 1800 "s59E5H2" 0 "Putative prophage phiRv2 integrase"
"Chl_C14_00235" 1800 "s63E6H2" 0.005951097 "Putative prophage phiRv2 integrase"
"Chl_C14_00237" 1802 "s59E13H2" 0 "Helix-turn-helix domain protein"
"Chl_C14_00237" 1802 "s59E25H2" 0 "Helix-turn-helix domain protein"
"Chl_C14_00237" 1802 "s59E5H2" 0 "Helix-turn-helix domain protein"

"Chl_C14_00237" 1802 "s63E6H2" 0 "Helix-turn-helix domain protein"
"Chl_C14_00238" 1803 "s59E13H2" 0 "putative tyrosine recombinase XerC-like protein"
"Chl_C14_00238" 1803 "s59E25H2" 0 "putative tyrosine recombinase XerC-like protein"
"Chl_C14_00238" 1803 "s59E5H2" 0.006287728 "putative tyrosine recombinase XerC-like protein"
"Chl_C14_00238" 1803 "s63E6H2" 0 "putative tyrosine recombinase XerC-like protein"
"Chl_C14_00240" 1805 "s59E13H2" 0 "Chromosome partition protein Smc"
"Chl_C14_00240" 1805 "s59E25H2" 0 "Chromosome partition protein Smc"
"Chl_C14_00240" 1805 "s59E5H2" 0 "Chromosome partition protein Smc"
"Chl_C14_00240" 1805 "s63E6H2" 0 "Chromosome partition protein Smc"
"Chl_C14_00242" 1807 "s59E13H2" 0 "hypothetical protein"
"Chl_C14_00242" 1807 "s59E25H2" 0 "hypothetical protein"
"Chl_C14_00242" 1807 "s59E5H2" 0 "hypothetical protein"
"Chl_C14_00242" 1807 "s63E6H2" 0 "hypothetical protein"
"Chl_C14_00243" 1808 "s59E13H2" 0 "hypothetical protein"
"Chl_C14_00243" 1808 "s59E25H2" 0 "hypothetical protein"
"Chl_C14_00243" 1808 "s59E5H2" 0 "hypothetical protein"
"Chl_C14_00243" 1808 "s63E6H2" 0 "hypothetical protein"
"Chl_C14_00244" 1809 "s59E13H2" 0 "Cell division protein FtsZ 1"
"Chl_C14_00244" 1809 "s59E25H2" 0 "Cell division protein FtsZ 1"
"Chl_C14_00244" 1809 "s59E5H2" 0 "Cell division protein FtsZ 1"
"Chl_C14_00244" 1809 "s63E6H2" 0 "Cell division protein FtsZ 1"
"Chl_C14_00245" 1810 "s59E13H2" 0 "cell division protein FtsZ"
"Chl_C14_00245" 1810 "s59E25H2" 0 "cell division protein FtsZ"
"Chl_C14_00245" 1810 "s59E5H2" 0.0018658 "cell division protein FtsZ"
"Chl_C14_00245" 1810 "s63E6H2" 0 "cell division protein FtsZ"
"Chl_C14_00246" 1811 "s59E13H2" 0 "Glycosyltransferase AglJ"
"Chl_C14_00246" 1811 "s59E25H2" 0 "Glycosyltransferase AglJ"
"Chl_C14_00246" 1811 "s59E5H2" 0 "Glycosyltransferase AglJ"
"Chl_C14_00246" 1811 "s63E6H2" 0 "Glycosyltransferase AglJ"
"Chl_C14_00247" 1812 "s59E13H2" 0 "hypothetical protein"
"Chl_C14_00247" 1812 "s59E25H2" 0 "hypothetical protein"
"Chl_C14_00247" 1812 "s59E5H2" 0 "hypothetical protein"
"Chl_C14_00247" 1812 "s63E6H2" 0 "hypothetical protein"
"Chl_C14_00248" 1813 "s59E13H2" 0 "UDP-glucose 4-epimerase"
"Chl_C14_00248" 1813 "s59E25H2" 0 "UDP-glucose 4-epimerase"
"Chl_C14_00248" 1813 "s59E5H2" 0 "UDP-glucose 4-epimerase"
"Chl_C14_00248" 1813 "s63E6H2" 0 "UDP-glucose 4-epimerase"
"Chl_C14_00249" 1814 "s59E13H2" 0 "ATP synthase epsilon chain"
"Chl_C14_00249" 1814 "s59E25H2" 0 "ATP synthase epsilon chain"
"Chl_C14_00249" 1814 "s59E5H2" 0 "ATP synthase epsilon chain"
"Chl_C14_00249" 1814 "s63E6H2" 0 "ATP synthase epsilon chain"
"Chl_C14_00250" 1815 "s59E13H2" 0 "ATP synthase subunit beta"
"Chl_C14_00250" 1815 "s59E25H2" 0 "ATP synthase subunit beta"
"Chl_C14_00250" 1815 "s59E5H2" 0.04181043 "ATP synthase subunit beta"
"Chl_C14_00250" 1815 "s63E6H2" 0 "ATP synthase subunit beta"
"Chl_C14_00251" 1816 "s59E13H2" 0 "ATP synthase gamma chain"
"Chl_C14_00251" 1816 "s59E25H2" 0 "ATP synthase gamma chain"

"Chl_C14_00251" 1816 "s59E5H2" 0 "ATP synthase gamma chain"
"Chl_C14_00251" 1816 "s63E6H2" 0 "ATP synthase gamma chain"
"Chl_C14_00252" 1817 "s59E13H2" 0 "ATP synthase subunit alpha"
"Chl_C14_00252" 1817 "s59E25H2" 0 "ATP synthase subunit alpha"
"Chl_C14_00252" 1817 "s59E5H2" 0 "ATP synthase subunit alpha"
"Chl_C14_00252" 1817 "s63E6H2" 0 "ATP synthase subunit alpha"
"Chl_C14_00253" 1818 "s59E13H2" 0.0004948084 "Acetyl-coenzyme A synthetase"
"Chl_C14_00253" 1818 "s59E25H2" 0 "Acetyl-coenzyme A synthetase"
"Chl_C14_00253" 1818 "s59E5H2" 0 "Acetyl-coenzyme A synthetase"
"Chl_C14_00253" 1818 "s63E6H2" 0 "Acetyl-coenzyme A synthetase"
"Chl_C14_00254" 1819 "s59E13H2" 0 "Cyclic nucleotide-binding domain protein"
"Chl_C14_00254" 1819 "s59E25H2" 0 "Cyclic nucleotide-binding domain protein"
"Chl_C14_00254" 1819 "s59E5H2" 0 "Cyclic nucleotide-binding domain protein"
"Chl_C14_00254" 1819 "s63E6H2" 0 "Cyclic nucleotide-binding domain protein"
"Chl_C14_00255" 1820 "s59E13H2" 0 "ATP synthase subunit delta"
"Chl_C14_00255" 1820 "s59E25H2" 0 "ATP synthase subunit delta"
"Chl_C14_00255" 1820 "s59E5H2" 0 "ATP synthase subunit delta"
"Chl_C14_00255" 1820 "s63E6H2" 0 "ATP synthase subunit delta"
"Chl_C14_00256" 1821 "s59E13H2" 0 "DNA recombination-mediator protein A"
"Chl_C14_00256" 1821 "s59E25H2" 0 "DNA recombination-mediator protein A"
"Chl_C14_00256" 1821 "s59E5H2" 0 "DNA recombination-mediator protein A"
"Chl_C14_00256" 1821 "s63E6H2" 0 "DNA recombination-mediator protein A"
"Chl_C14_00257" 1822 "s59E13H2" 0 "Enolase"
"Chl_C14_00257" 1822 "s59E25H2" 0 "Enolase"
"Chl_C14_00257" 1822 "s59E5H2" 0 "Enolase"
"Chl_C14_00257" 1822 "s63E6H2" 0 "Enolase"
"Chl_C14_00258" 1823 "s59E13H2" 0 "hypothetical protein"
"Chl_C14_00258" 1823 "s59E25H2" 0 "hypothetical protein"
"Chl_C14_00258" 1823 "s59E5H2" 0 "hypothetical protein"
"Chl_C14_00258" 1823 "s63E6H2" 0 "hypothetical protein"
"Chl_C14_00259" 1824 "s59E13H2" 0 "2-hydroxyglutaryl-CoA dehydratase, D-component"
"Chl_C14_00259" 1824 "s59E25H2" 0 "2-hydroxyglutaryl-CoA dehydratase, D-component"
"Chl_C14_00259" 1824 "s59E5H2" 0 "2-hydroxyglutaryl-CoA dehydratase, D-component"
"Chl_C14_00259" 1824 "s63E6H2" 0 "2-hydroxyglutaryl-CoA dehydratase, D-component"
"Chl_C14_00260" 1825 "s59E13H2" 0 "Glyceraldehyde-3-phosphate dehydrogenase"
"Chl_C14_00260" 1825 "s59E25H2" 0 "Glyceraldehyde-3-phosphate dehydrogenase"
"Chl_C14_00260" 1825 "s59E5H2" 0 "Glyceraldehyde-3-phosphate dehydrogenase"
"Chl_C14_00260" 1825 "s63E6H2" 0 "Glyceraldehyde-3-phosphate dehydrogenase"
"Chl_C14_00261" 1826 "s59E13H2" 0 "5,6-dimethylbenzimidazole synthase"
"Chl_C14_00261" 1826 "s59E25H2" 0 "5,6-dimethylbenzimidazole synthase"
"Chl_C14_00261" 1826 "s59E5H2" 0 "5,6-dimethylbenzimidazole synthase"
"Chl_C14_00261" 1826 "s63E6H2" 0 "5,6-dimethylbenzimidazole synthase"
"Chl_C14_00262" 1827 "s59E13H2" 0 "Ribonuclease 3"
"Chl_C14_00262" 1827 "s59E25H2" 0 "Ribonuclease 3"
"Chl_C14_00262" 1827 "s59E5H2" 0 "Ribonuclease 3"
"Chl_C14_00262" 1827 "s63E6H2" 0 "Ribonuclease 3"
"Chl_C14_00264" 1829 "s59E13H2" 0.001000462 "Radical SAM superfamily protein"

"Chl_C14_00264" 1829 "s59E25H2" 0 "Radical SAM superfamily protein"
"Chl_C14_00264" 1829 "s59E5H2" 0 "Radical SAM superfamily protein"
"Chl_C14_00264" 1829 "s63E6H2" 0 "Radical SAM superfamily protein"
"Chl_C14_00265" 1830 "s59E13H2" 0 "Acylphosphatase"
"Chl_C14_00265" 1830 "s59E25H2" 0 "Acylphosphatase"
"Chl_C14_00265" 1830 "s59E5H2" 0 "Acylphosphatase"
"Chl_C14_00265" 1830 "s63E6H2" 0 "Acylphosphatase"
"Chl_C14_00267" 1832 "s59E13H2" 0 "hypothetical protein"
"Chl_C14_00267" 1832 "s59E25H2" 0 "hypothetical protein"
"Chl_C14_00267" 1832 "s59E5H2" 0 "hypothetical protein"
"Chl_C14_00267" 1832 "s63E6H2" 0 "hypothetical protein"
"Chl_C14_00268" 1833 "s59E13H2" 0 "Molybdopterin-guanine dinucleotide biosynthesis adapter protein"
"Chl_C14_00268" 1833 "s59E25H2" 0 "Molybdopterin-guanine dinucleotide biosynthesis adapter protein"
"Chl_C14_00268" 1833 "s59E5H2" 0 "Molybdopterin-guanine dinucleotide biosynthesis adapter protein"
"Chl_C14_00268" 1833 "s63E6H2" 0 "Molybdopterin-guanine dinucleotide biosynthesis adapter protein"
"Chl_C14_00269" 1834 "s59E13H2" 0 "Molybdopterin molybdenumtransferase"
"Chl_C14_00269" 1834 "s59E25H2" 0 "Molybdopterin molybdenumtransferase"
"Chl_C14_00269" 1834 "s59E5H2" 0 "Molybdopterin molybdenumtransferase"
"Chl_C14_00269" 1834 "s63E6H2" 0 "Molybdopterin molybdenumtransferase"
"Chl_C14_00270" 1835 "s59E13H2" 0 "putative molybdenum cofactor guanylyltransferase"
"Chl_C14_00270" 1835 "s59E25H2" 0 "putative molybdenum cofactor guanylyltransferase"
"Chl_C14_00270" 1835 "s59E5H2" 0 "putative molybdenum cofactor guanylyltransferase"
"Chl_C14_00270" 1835 "s63E6H2" 0 "putative molybdenum cofactor guanylyltransferase"
"Chl_C14_00271" 1836 "s59E13H2" 0 "Enhancing lycopene biosynthesis protein 2"
"Chl_C14_00271" 1836 "s59E25H2" 0 "Enhancing lycopene biosynthesis protein 2"
"Chl_C14_00271" 1836 "s59E5H2" 0 "Enhancing lycopene biosynthesis protein 2"
"Chl_C14_00271" 1836 "s63E6H2" 0 "Enhancing lycopene biosynthesis protein 2"
"Chl_C14_00272" 1837 "s59E13H2" 0 "Cytosol aminopeptidase"
"Chl_C14_00272" 1837 "s59E25H2" 0 "Cytosol aminopeptidase"
"Chl_C14_00272" 1837 "s59E5H2" 0 "Cytosol aminopeptidase"
"Chl_C14_00272" 1837 "s63E6H2" 0 "Cytosol aminopeptidase"
"Chl_C14_00274" 1839 "s59E13H2" 0 "Double zinc ribbon"
"Chl_C14_00274" 1839 "s59E25H2" 0 "Double zinc ribbon"
"Chl_C14_00274" 1839 "s59E5H2" 0 "Double zinc ribbon"
"Chl_C14_00274" 1839 "s63E6H2" 0 "Double zinc ribbon"
"Chl_C14_00277" 1842 "s59E13H2" 0 "Elongation factor Tu-B"
"Chl_C14_00277" 1842 "s59E25H2" 0 "Elongation factor Tu-B"
"Chl_C14_00277" 1842 "s59E5H2" 0 "Elongation factor Tu-B"
"Chl_C14_00277" 1842 "s63E6H2" 0.0005760923 "Elongation factor Tu-B"
"Chl_C14_00278" 1843 "s59E13H2" 0 "preprotein translocase subunit SecE"
"Chl_C14_00278" 1843 "s59E25H2" 0 "preprotein translocase subunit SecE"
"Chl_C14_00278" 1843 "s59E5H2" 0 "preprotein translocase subunit SecE"
"Chl_C14_00278" 1843 "s63E6H2" 0 "preprotein translocase subunit SecE"
"Chl_C14_00279" 1844 "s59E13H2" 0 "hypothetical protein"
"Chl_C14_00279" 1844 "s59E25H2" 0 "hypothetical protein"
"Chl_C14_00279" 1844 "s59E5H2" 0 "hypothetical protein"
"Chl_C14_00279" 1844 "s63E6H2" 0 "hypothetical protein"

"Chl_C14_00280" 1845 "s59E13H2" 0 "50S ribosomal protein L11"
"Chl_C14_00280" 1845 "s59E25H2" 0 "50S ribosomal protein L11"
"Chl_C14_00280" 1845 "s59E5H2" 0 "50S ribosomal protein L11"
"Chl_C14_00280" 1845 "s63E6H2" 0 "50S ribosomal protein L11"
"Chl_C14_00281" 1846 "s59E13H2" 0 "50S ribosomal protein L1"
"Chl_C14_00281" 1846 "s59E25H2" 0 "50S ribosomal protein L1"
"Chl_C14_00281" 1846 "s59E5H2" 0 "50S ribosomal protein L1"
"Chl_C14_00281" 1846 "s63E6H2" 0 "50S ribosomal protein L1"
"Chl_C14_00282" 1847 "s59E13H2" 0 "putative acetyl-CoA acyltransferase"
"Chl_C14_00282" 1847 "s59E25H2" 0 "putative acetyl-CoA acyltransferase"
"Chl_C14_00282" 1847 "s59E5H2" 0 "putative acetyl-CoA acyltransferase"
"Chl_C14_00282" 1847 "s63E6H2" 0 "putative acetyl-CoA acyltransferase"
"Chl_C14_00283" 1848 "s59E13H2" 0 "hypothetical protein"
"Chl_C14_00283" 1848 "s59E25H2" 0 "hypothetical protein"
"Chl_C14_00283" 1848 "s59E5H2" 0 "hypothetical protein"
"Chl_C14_00283" 1848 "s63E6H2" 0 "hypothetical protein"
"Chl_C14_00284" 1849 "s59E13H2" 0 "50S ribosomal protein L10"
"Chl_C14_00284" 1849 "s59E25H2" 0 "50S ribosomal protein L10"
"Chl_C14_00284" 1849 "s59E5H2" 0 "50S ribosomal protein L10"
"Chl_C14_00284" 1849 "s63E6H2" 0 "50S ribosomal protein L10"
"Chl_C14_00285" 1850 "s59E13H2" 0 "50S ribosomal protein L7/L12"
"Chl_C14_00285" 1850 "s59E25H2" 0 "50S ribosomal protein L7/L12"
"Chl_C14_00285" 1850 "s59E5H2" 0 "50S ribosomal protein L7/L12"
"Chl_C14_00285" 1850 "s63E6H2" 0 "50S ribosomal protein L7/L12"
"Chl_C14_00286" 1851 "s59E13H2" 0 "hypothetical protein"
"Chl_C14_00286" 1851 "s59E25H2" 0 "hypothetical protein"
"Chl_C14_00286" 1851 "s59E5H2" 0 "hypothetical protein"
"Chl_C14_00286" 1851 "s63E6H2" 0 "hypothetical protein"
"Chl_C14_00287" 1852 "s59E13H2" 0 "hypothetical protein"
"Chl_C14_00287" 1852 "s59E25H2" 0 "hypothetical protein"
"Chl_C14_00287" 1852 "s59E5H2" 0 "hypothetical protein"
"Chl_C14_00287" 1852 "s63E6H2" 0 "hypothetical protein"
"Chl_C14_00288" 1853 "s59E13H2" 0 "Competence protein ComM"
"Chl_C14_00288" 1853 "s59E25H2" 0 "Competence protein ComM"
"Chl_C14_00288" 1853 "s59E5H2" 0.0104363 "Competence protein ComM"
"Chl_C14_00288" 1853 "s63E6H2" 0 "Competence protein ComM"
"Chl_C14_00289" 1854 "s59E13H2" 0 "Transposon gamma-delta resolvase"
"Chl_C14_00289" 1854 "s59E25H2" 0 "Transposon gamma-delta resolvase"
"Chl_C14_00289" 1854 "s59E5H2" 0.005312319 "Transposon gamma-delta resolvase"
"Chl_C14_00289" 1854 "s63E6H2" 0 "Transposon gamma-delta resolvase"
"Chl_C14_00291" 1856 "s59E13H2" 0 "hypothetical protein"
"Chl_C14_00291" 1856 "s59E25H2" 0 "hypothetical protein"
"Chl_C14_00291" 1856 "s59E5H2" 0 "hypothetical protein"
"Chl_C14_00291" 1856 "s63E6H2" 0 "hypothetical protein"
"Chl_C14_00295" 1860 "s59E13H2" 0.0002637192 "Transposase"
"Chl_C14_00295" 1860 "s59E25H2" 0 "Transposase"
"Chl_C14_00295" 1860 "s59E5H2" 0.003214174 "Transposase"

"Chl_C14_00295" 1860 "s63E6H2" 0.002900388 "Transposase"
"Chl_C14_00296" 1861 "s59E13H2" 0 "hypothetical protein"
"Chl_C14_00296" 1861 "s59E25H2" 0 "hypothetical protein"
"Chl_C14_00296" 1861 "s59E5H2" 0 "hypothetical protein"
"Chl_C14_00296" 1861 "s63E6H2" 0 "hypothetical protein"
"Chl_C14_00297" 1862 "s59E13H2" 0 "hypothetical protein"
"Chl_C14_00297" 1862 "s59E25H2" 0 "hypothetical protein"
"Chl_C14_00297" 1862 "s59E5H2" 0 "hypothetical protein"
"Chl_C14_00297" 1862 "s63E6H2" 0 "hypothetical protein"
"Chl_C14_00298" 1863 "s59E13H2" 0 "Beta-galactosidase"
"Chl_C14_00298" 1863 "s59E25H2" 0 "Beta-galactosidase"
"Chl_C14_00298" 1863 "s59E5H2" 0 "Beta-galactosidase"
"Chl_C14_00298" 1863 "s63E6H2" 0 "Beta-galactosidase"
"Chl_C14_00299" 1864 "s59E13H2" 0 "LexA repressor"
"Chl_C14_00299" 1864 "s59E25H2" 0 "LexA repressor"
"Chl_C14_00299" 1864 "s59E5H2" 0 "LexA repressor"
"Chl_C14_00299" 1864 "s63E6H2" 0 "LexA repressor"
"Chl_C14_00300" 1865 "s59E13H2" 0 "hypothetical protein"
"Chl_C14_00300" 1865 "s59E25H2" 0 "hypothetical protein"
"Chl_C14_00300" 1865 "s59E5H2" 0 "hypothetical protein"
"Chl_C14_00300" 1865 "s63E6H2" 0 "hypothetical protein"
"Chl_C14_00301" 1866 "s59E13H2" 0 "Thermonuclease precursor"
"Chl_C14_00301" 1866 "s59E25H2" 0 "Thermonuclease precursor"
"Chl_C14_00301" 1866 "s59E5H2" 0 "Thermonuclease precursor"
"Chl_C14_00301" 1866 "s63E6H2" 0 "Thermonuclease precursor"
"Chl_C14_00302" 1867 "s59E13H2" 0 "Peptidoglycan deacetylase"
"Chl_C14_00302" 1867 "s59E25H2" 0 "Peptidoglycan deacetylase"
"Chl_C14_00302" 1867 "s59E5H2" 0 "Peptidoglycan deacetylase"
"Chl_C14_00302" 1867 "s63E6H2" 0 "Peptidoglycan deacetylase"
"Chl_C14_00303" 1868 "s59E13H2" 0 "Putative acetyltransferase"
"Chl_C14_00303" 1868 "s59E25H2" 0 "Putative acetyltransferase"
"Chl_C14_00303" 1868 "s59E5H2" 0 "Putative acetyltransferase"
"Chl_C14_00303" 1868 "s63E6H2" 0 "Putative acetyltransferase"
"Chl_C14_00304" 1869 "s59E13H2" 0 "D-glycero-alpha-D-manno-heptose 7-phosphate kinase"
"Chl_C14_00304" 1869 "s59E25H2" 0 "D-glycero-alpha-D-manno-heptose 7-phosphate kinase"
"Chl_C14_00304" 1869 "s59E5H2" 0 "D-glycero-alpha-D-manno-heptose 7-phosphate kinase"
"Chl_C14_00304" 1869 "s63E6H2" 0 "D-glycero-alpha-D-manno-heptose 7-phosphate kinase"
"Chl_C14_00305" 1870 "s59E13H2" 0 "Phosphoheptose isomerase"
"Chl_C14_00305" 1870 "s59E25H2" 0 "Phosphoheptose isomerase"
"Chl_C14_00305" 1870 "s59E5H2" 0 "Phosphoheptose isomerase"
"Chl_C14_00305" 1870 "s63E6H2" 0 "Phosphoheptose isomerase"
"Chl_C14_00306" 1871 "s59E13H2" 0 "D-glycero-beta-D-manno-heptose-1,7-bisphosphate 7-phosphatase"
"Chl_C14_00306" 1871 "s59E25H2" 0 "D-glycero-beta-D-manno-heptose-1,7-bisphosphate 7-phosphatase"
"Chl_C14_00306" 1871 "s59E5H2" 0 "D-glycero-beta-D-manno-heptose-1,7-bisphosphate 7-phosphatase"
"Chl_C14_00306" 1871 "s63E6H2" 0 "D-glycero-beta-D-manno-heptose-1,7-bisphosphate 7-phosphatase"
"Chl_C14_00307" 1872 "s59E13H2" 0 "D-inositol 3-phosphate glycosyltransferase"
"Chl_C14_00307" 1872 "s59E25H2" 0 "D-inositol 3-phosphate glycosyltransferase"

"Chl_C14_00307" 1872 "s59E5H2" 0 "D-inositol 3-phosphate glycosyltransferase"
"Chl_C14_00307" 1872 "s63E6H2" 0 "D-inositol 3-phosphate glycosyltransferase"
"Chl_C14_00308" 1873 "s59E13H2" 0 "putative S-adenosylmethionine-dependent methyltransferase/MSMEI_2290"
"Chl_C14_00308" 1873 "s59E25H2" 0 "putative S-adenosylmethionine-dependent methyltransferase/MSMEI_2290"
"Chl_C14_00308" 1873 "s59E5H2" 0 "putative S-adenosylmethionine-dependent methyltransferase/MSMEI_2290"
"Chl_C14_00308" 1873 "s63E6H2" 0 "putative S-adenosylmethionine-dependent methyltransferase/MSMEI_2290"
"Chl_C14_00309" 1874 "s59E13H2" 0 "putative oxidoreductase YcjS"
"Chl_C14_00309" 1874 "s59E25H2" 0 "putative oxidoreductase YcjS"
"Chl_C14_00309" 1874 "s59E5H2" 0 "putative oxidoreductase YcjS"
"Chl_C14_00309" 1874 "s63E6H2" 0 "putative oxidoreductase YcjS"
"Chl_C14_00310" 1875 "s59E13H2" 0 "UDP-2-acetamido-2-deoxy-3-oxo-D-glucuronate aminotransferase"
"Chl_C14_00310" 1875 "s59E25H2" 0 "UDP-2-acetamido-2-deoxy-3-oxo-D-glucuronate aminotransferase"
"Chl_C14_00310" 1875 "s59E5H2" 0 "UDP-2-acetamido-2-deoxy-3-oxo-D-glucuronate aminotransferase"
"Chl_C14_00310" 1875 "s63E6H2" 0 "UDP-2-acetamido-2-deoxy-3-oxo-D-glucuronate aminotransferase"
"Chl_C14_00311" 1876 "s59E13H2" 0 "UDP-glucose 4-epimerase"
"Chl_C14_00311" 1876 "s59E25H2" 0 "UDP-glucose 4-epimerase"
"Chl_C14_00311" 1876 "s59E5H2" 0 "UDP-glucose 4-epimerase"
"Chl_C14_00311" 1876 "s63E6H2" 0 "UDP-glucose 4-epimerase"
"Chl_C14_00314" 1879 "s59E13H2" 0 "putative cold shock protein A"
"Chl_C14_00314" 1879 "s59E25H2" 0 "putative cold shock protein A"
"Chl_C14_00314" 1879 "s59E5H2" 0 "putative cold shock protein A"
"Chl_C14_00314" 1879 "s63E6H2" 0 "putative cold shock protein A"
"Chl_C14_00315" 1880 "s59E13H2" 0 "hypothetical protein"
"Chl_C14_00315" 1880 "s59E25H2" 0 "hypothetical protein"
"Chl_C14_00315" 1880 "s59E5H2" 0 "hypothetical protein"
"Chl_C14_00315" 1880 "s63E6H2" 0 "hypothetical protein"
"Chl_C14_00316" 1881 "s59E13H2" 0 "Modification methylase VspI"
"Chl_C14_00316" 1881 "s59E25H2" 0 "Modification methylase VspI"
"Chl_C14_00316" 1881 "s59E5H2" 0 "Modification methylase VspI"
"Chl_C14_00316" 1881 "s63E6H2" 0 "Modification methylase VspI"
"Chl_C14_00319" 1884 "s59E13H2" 0 "hypothetical protein"
"Chl_C14_00319" 1884 "s59E25H2" 0 "hypothetical protein"
"Chl_C14_00319" 1884 "s59E5H2" 0 "hypothetical protein"
"Chl_C14_00319" 1884 "s63E6H2" 0 "hypothetical protein"
"Chl_C14_00321" 1886 "s59E13H2" 0 "hypothetical protein"
"Chl_C14_00321" 1886 "s59E25H2" 0 "hypothetical protein"
"Chl_C14_00321" 1886 "s59E5H2" 0 "hypothetical protein"
"Chl_C14_00321" 1886 "s63E6H2" 0 "hypothetical protein"
"Chl_C14_00323" 1888 "s59E13H2" 0 "Glutathione transport system permease protein GsiC"
"Chl_C14_00323" 1888 "s59E25H2" 0 "Glutathione transport system permease protein GsiC"
"Chl_C14_00323" 1888 "s59E5H2" 0 "Glutathione transport system permease protein GsiC"
"Chl_C14_00323" 1888 "s63E6H2" 0 "Glutathione transport system permease protein GsiC"
"Chl_C14_00324" 1889 "s59E13H2" 0 "Dipeptide transport system permease protein DppC"
"Chl_C14_00324" 1889 "s59E25H2" 0 "Dipeptide transport system permease protein DppC"
"Chl_C14_00324" 1889 "s59E5H2" 0 "Dipeptide transport system permease protein DppC"
"Chl_C14_00324" 1889 "s63E6H2" 0 "Dipeptide transport system permease protein DppC"
"Chl_C14_00325" 1890 "s59E13H2" 0 "Oligopeptide transport ATP-binding protein OppD"

"Chl_C14_00325" 1890 "s59E25H2" 0 "Oligopeptide transport ATP-binding protein OppD"
"Chl_C14_00325" 1890 "s59E5H2" 0 "Oligopeptide transport ATP-binding protein OppD"
"Chl_C14_00325" 1890 "s63E6H2" 0 "Oligopeptide transport ATP-binding protein OppD"
"Chl_C14_00326" 1891 "s59E13H2" 0 "Oligopeptide transport ATP-binding protein OppF"
"Chl_C14_00326" 1891 "s59E25H2" 0 "Oligopeptide transport ATP-binding protein OppF"
"Chl_C14_00326" 1891 "s59E5H2" 0 "Oligopeptide transport ATP-binding protein OppF"
"Chl_C14_00326" 1891 "s63E6H2" 0 "Oligopeptide transport ATP-binding protein OppF"
"Chl_C14_00328" 1893 "s59E13H2" 0 "hypothetical protein"
"Chl_C14_00328" 1893 "s59E25H2" 0 "hypothetical protein"
"Chl_C14_00328" 1893 "s59E5H2" 0 "hypothetical protein"
"Chl_C14_00328" 1893 "s63E6H2" 0 "hypothetical protein"
"Chl_C14_00329" 1894 "s59E13H2" 0 "Tungsten-containing aldehyde ferredoxin oxidoreductase"
"Chl_C14_00329" 1894 "s59E25H2" 0 "Tungsten-containing aldehyde ferredoxin oxidoreductase"
"Chl_C14_00329" 1894 "s59E5H2" 0 "Tungsten-containing aldehyde ferredoxin oxidoreductase"
"Chl_C14_00329" 1894 "s63E6H2" 0 "Tungsten-containing aldehyde ferredoxin oxidoreductase"
"Chl_C14_00330" 1895 "s59E13H2" 0 "Putative redox-active protein (C_GCAxxG_C_C)"
"Chl_C14_00330" 1895 "s59E25H2" 0 "Putative redox-active protein (C_GCAxxG_C_C)"
"Chl_C14_00330" 1895 "s59E5H2" 0 "Putative redox-active protein (C_GCAxxG_C_C)"
"Chl_C14_00330" 1895 "s63E6H2" 0 "Putative redox-active protein (C_GCAxxG_C_C)"
"Chl_C14_00334" 1899 "s59E13H2" 0 "Glutaredoxin-3"
"Chl_C14_00334" 1899 "s59E25H2" 0 "Glutaredoxin-3"
"Chl_C14_00334" 1899 "s59E5H2" 0 "Glutaredoxin-3"
"Chl_C14_00334" 1899 "s63E6H2" 0 "Glutaredoxin-3"
"Chl_C14_00335" 1900 "s59E13H2" 0 "Alkyl hydroperoxide reductase subunit F"
"Chl_C14_00335" 1900 "s59E25H2" 0 "Alkyl hydroperoxide reductase subunit F"
"Chl_C14_00335" 1900 "s59E5H2" 0 "Alkyl hydroperoxide reductase subunit F"
"Chl_C14_00335" 1900 "s63E6H2" 0 "Alkyl hydroperoxide reductase subunit F"
"Chl_C14_00336" 1901 "s59E13H2" 0 "Rubrerythrin"
"Chl_C14_00336" 1901 "s59E25H2" 0 "Rubrerythrin"
"Chl_C14_00336" 1901 "s59E5H2" 0 "Rubrerythrin"
"Chl_C14_00336" 1901 "s63E6H2" 0 "Rubrerythrin"
"Chl_C14_00337" 1902 "s59E13H2" 0 "putative trifunctional 2-polyprenylphenol hydroxylase/glutamate synthase subunit beta/ferritin domain-containing protein"
"Chl_C14_00337" 1902 "s59E25H2" 0 "putative trifunctional 2-polyprenylphenol hydroxylase/glutamate synthase subunit beta/ferritin domain-containing protein"
"Chl_C14_00337" 1902 "s59E5H2" 0 "putative trifunctional 2-polyprenylphenol hydroxylase/glutamate synthase subunit beta/ferritin domain-containing protein"
"Chl_C14_00337" 1902 "s63E6H2" 0 "putative trifunctional 2-polyprenylphenol hydroxylase/glutamate synthase subunit beta/ferritin domain-containing protein"
"Chl_C14_00338" 1903 "s59E13H2" 0 "putative cysteine desulfurase 2"
"Chl_C14_00338" 1903 "s59E25H2" 0 "putative cysteine desulfurase 2"
"Chl_C14_00338" 1903 "s59E5H2" 0 "putative cysteine desulfurase 2"
"Chl_C14_00338" 1903 "s63E6H2" 0 "putative cysteine desulfurase 2"
"Chl_C14_00339" 1904 "s59E13H2" 0 "NifU-like protein"
"Chl_C14_00339" 1904 "s59E25H2" 0 "NifU-like protein"
"Chl_C14_00339" 1904 "s59E5H2" 0 "NifU-like protein"
"Chl_C14_00339" 1904 "s63E6H2" 0 "NifU-like protein"
"Chl_C14_00341" 1906 "s59E13H2" 0 "hypothetical protein"
"Chl_C14_00341" 1906 "s59E25H2" 0 "hypothetical protein"
"Chl_C14_00341" 1906 "s59E5H2" 0 "hypothetical protein"
"Chl_C14_00341" 1906 "s63E6H2" 0 "hypothetical protein"

"Chl_C14_00342" 1907 "s59E13H2" 0 "Sulfurtransferase TusA"
"Chl_C14_00342" 1907 "s59E25H2" 0 "Sulfurtransferase TusA"
"Chl_C14_00342" 1907 "s59E5H2" 0 "Sulfurtransferase TusA"
"Chl_C14_00342" 1907 "s63E6H2" 0 "Sulfurtransferase TusA"
"Chl_C14_00343" 1908 "s59E13H2" 0 "High molecular weight rubredoxin"
"Chl_C14_00343" 1908 "s59E25H2" 0 "High molecular weight rubredoxin"
"Chl_C14_00343" 1908 "s59E5H2" 0.005710683 "High molecular weight rubredoxin"
"Chl_C14_00343" 1908 "s63E6H2" 0 "High molecular weight rubredoxin"
"Chl_C14_00344" 1909 "s59E13H2" 0 "3-oxoacyl-[acyl-carrier-protein] reductase FabG"
"Chl_C14_00344" 1909 "s59E25H2" 0 "3-oxoacyl-[acyl-carrier-protein] reductase FabG"
"Chl_C14_00344" 1909 "s59E5H2" 0 "3-oxoacyl-[acyl-carrier-protein] reductase FabG"
"Chl_C14_00344" 1909 "s63E6H2" 0 "3-oxoacyl-[acyl-carrier-protein] reductase FabG"
"Chl_C14_00345" 1910 "s59E13H2" 0 "Desulfoferrodoxin"
"Chl_C14_00345" 1910 "s59E25H2" 0 "Desulfoferrodoxin"
"Chl_C14_00345" 1910 "s59E5H2" 0 "Desulfoferrodoxin"
"Chl_C14_00345" 1910 "s63E6H2" 0 "Desulfoferrodoxin"
"Chl_C14_00346" 1911 "s59E13H2" 0 "hypothetical protein"
"Chl_C14_00346" 1911 "s59E25H2" 0 "hypothetical protein"
"Chl_C14_00346" 1911 "s59E5H2" 0 "hypothetical protein"
"Chl_C14_00346" 1911 "s63E6H2" 0 "hypothetical protein"
"Chl_C14_00347" 1912 "s59E13H2" 0 "Copper-exporting P-type ATPase A"
"Chl_C14_00347" 1912 "s59E25H2" 0 "Copper-exporting P-type ATPase A"
"Chl_C14_00347" 1912 "s59E5H2" 0 "Copper-exporting P-type ATPase A"
"Chl_C14_00347" 1912 "s63E6H2" 0.0002591092 "Copper-exporting P-type ATPase A"
"Chl_C14_00348" 1913 "s59E13H2" 0 "Copper-transporting P-type ATPase"
"Chl_C14_00348" 1913 "s59E25H2" 0 "Copper-transporting P-type ATPase"
"Chl_C14_00348" 1913 "s59E5H2" 0 "Copper-transporting P-type ATPase"
"Chl_C14_00348" 1913 "s63E6H2" 0 "Copper-transporting P-type ATPase"
"Chl_C14_00349" 1914 "s59E13H2" 0 "Bacterioferritin"
"Chl_C14_00349" 1914 "s59E25H2" 0 "Bacterioferritin"
"Chl_C14_00349" 1914 "s59E5H2" 0.005017917 "Bacterioferritin"
"Chl_C14_00349" 1914 "s63E6H2" 0 "Bacterioferritin"
"Chl_C14_00350" 1915 "s59E13H2" 0 "tRNA-binding protein YgjH"
"Chl_C14_00350" 1915 "s59E25H2" 0 "tRNA-binding protein YgjH"
"Chl_C14_00350" 1915 "s59E5H2" 0 "tRNA-binding protein YgjH"
"Chl_C14_00350" 1915 "s63E6H2" 0 "tRNA-binding protein YgjH"
"Chl_C14_00351" 1916 "s59E13H2" 0 "3-oxoadipate CoA-transferase subunit A"
"Chl_C14_00351" 1916 "s59E25H2" 0 "3-oxoadipate CoA-transferase subunit A"
"Chl_C14_00351" 1916 "s59E5H2" 0 "3-oxoadipate CoA-transferase subunit A"
"Chl_C14_00351" 1916 "s63E6H2" 0 "3-oxoadipate CoA-transferase subunit A"
"Chl_C14_00352" 1917 "s59E13H2" 0 "3-oxoadipate CoA-transferase subunit B"
"Chl_C14_00352" 1917 "s59E25H2" 0 "3-oxoadipate CoA-transferase subunit B"
"Chl_C14_00352" 1917 "s59E5H2" 0 "3-oxoadipate CoA-transferase subunit B"
"Chl_C14_00352" 1917 "s63E6H2" 0 "3-oxoadipate CoA-transferase subunit B"
"Chl_C14_00353" 1918 "s59E13H2" 0 "Transposase"
"Chl_C14_00353" 1918 "s59E25H2" 0 "Transposase"
"Chl_C14_00353" 1918 "s59E5H2" 0 "Transposase"

"Chl_C14_00353" 1918 "s63E6H2" 0 "Transposase"
"Chl_C14_00354" 1919 "s59E13H2" 0 "IS2 transposase TnpB"
"Chl_C14_00354" 1919 "s59E25H2" 0 "IS2 transposase TnpB"
"Chl_C14_00354" 1919 "s59E5H2" 0 "IS2 transposase TnpB"
"Chl_C14_00354" 1919 "s63E6H2" 0 "IS2 transposase TnpB"
"Chl_C14_00355" 1920 "s59E13H2" 0 "Butyrate--acetoacetate CoA-transferase subunit B"
"Chl_C14_00355" 1920 "s59E25H2" 0 "Butyrate--acetoacetate CoA-transferase subunit B"
"Chl_C14_00355" 1920 "s59E5H2" 0 "Butyrate--acetoacetate CoA-transferase subunit B"
"Chl_C14_00355" 1920 "s63E6H2" 0 "Butyrate--acetoacetate CoA-transferase subunit B"
"Chl_C14_00356" 1921 "s59E13H2" 0 "hypothetical protein"
"Chl_C14_00356" 1921 "s59E25H2" 0 "hypothetical protein"
"Chl_C14_00356" 1921 "s59E5H2" 0 "hypothetical protein"
"Chl_C14_00356" 1921 "s63E6H2" 0 "hypothetical protein"
"Chl_C14_00357" 1922 "s59E13H2" 0 "5,6-dimethylbenzimidazole synthase"
"Chl_C14_00357" 1922 "s59E25H2" 0 "5,6-dimethylbenzimidazole synthase"
"Chl_C14_00357" 1922 "s59E5H2" 0 "5,6-dimethylbenzimidazole synthase"
"Chl_C14_00357" 1922 "s63E6H2" 0 "5,6-dimethylbenzimidazole synthase"
"Chl_C14_00358" 1923 "s59E13H2" 0 "Tungsten-containing aldehyde ferredoxin oxidoreductase"
"Chl_C14_00358" 1923 "s59E25H2" 0 "Tungsten-containing aldehyde ferredoxin oxidoreductase"
"Chl_C14_00358" 1923 "s59E5H2" 0 "Tungsten-containing aldehyde ferredoxin oxidoreductase"
"Chl_C14_00358" 1923 "s63E6H2" 0 "Tungsten-containing aldehyde ferredoxin oxidoreductase"
"Chl_C14_00359" 1924 "s59E13H2" 0 "Quinolinate synthase A"
"Chl_C14_00359" 1924 "s59E25H2" 0 "Quinolinate synthase A"
"Chl_C14_00359" 1924 "s59E5H2" 0 "Quinolinate synthase A"
"Chl_C14_00359" 1924 "s63E6H2" 0 "Quinolinate synthase A"
"Chl_C14_00360" 1925 "s59E13H2" 0 "formate dehydrogenase accessory protein"
"Chl_C14_00360" 1925 "s59E25H2" 0 "formate dehydrogenase accessory protein"
"Chl_C14_00360" 1925 "s59E5H2" 0 "formate dehydrogenase accessory protein"
"Chl_C14_00360" 1925 "s63E6H2" 0 "formate dehydrogenase accessory protein"
"Chl_C14_00361" 1926 "s59E13H2" 0 "Hydrogenase isoenzymes formation protein HypE"
"Chl_C14_00361" 1926 "s59E25H2" 0 "Hydrogenase isoenzymes formation protein HypE"
"Chl_C14_00361" 1926 "s59E5H2" 0 "Hydrogenase isoenzymes formation protein HypE"
"Chl_C14_00361" 1926 "s63E6H2" 0 "Hydrogenase isoenzymes formation protein HypE"
"Chl_C14_00362" 1927 "s59E13H2" 0 "NifU-like protein"
"Chl_C14_00362" 1927 "s59E25H2" 0 "NifU-like protein"
"Chl_C14_00362" 1927 "s59E5H2" 0 "NifU-like protein"
"Chl_C14_00362" 1927 "s63E6H2" 0 "NifU-like protein"
"Chl_C14_00363" 1928 "s59E13H2" 0 "hypothetical protein"
"Chl_C14_00363" 1928 "s59E25H2" 0 "hypothetical protein"
"Chl_C14_00363" 1928 "s59E5H2" 0 "hypothetical protein"
"Chl_C14_00363" 1928 "s63E6H2" 0 "hypothetical protein"
"Chl_C14_00364" 1929 "s59E13H2" 0 "putative cysteine desulfurase 2"
"Chl_C14_00364" 1929 "s59E25H2" 0 "putative cysteine desulfurase 2"
"Chl_C14_00364" 1929 "s59E5H2" 0 "putative cysteine desulfurase 2"
"Chl_C14_00364" 1929 "s63E6H2" 0 "putative cysteine desulfurase 2"
"Chl_C14_00365" 1930 "s59E13H2" 0 "NIL domain protein"
"Chl_C14_00365" 1930 "s59E25H2" 0 "NIL domain protein"

"Chl_C14_00365" 1930 "s59E5H2" 0 "NIL domain protein"
"Chl_C14_00365" 1930 "s63E6H2" 0 "NIL domain protein"
"Chl_C14_00366" 1931 "s59E13H2" 0 "Threonine synthase"
"Chl_C14_00366" 1931 "s59E25H2" 0 "Threonine synthase"
"Chl_C14_00366" 1931 "s59E5H2" 0 "Threonine synthase"
"Chl_C14_00366" 1931 "s63E6H2" 0 "Threonine synthase"
"Chl_C14_00367" 1932 "s59E13H2" 0 "Cystathionine beta-lyase"
"Chl_C14_00367" 1932 "s59E25H2" 0 "Cystathionine beta-lyase"
"Chl_C14_00367" 1932 "s59E5H2" 0 "Cystathionine beta-lyase"
"Chl_C14_00367" 1932 "s63E6H2" 0 "Cystathionine beta-lyase"
"Chl_C14_00368" 1933 "s59E13H2" 0 "Serine acetyltransferase"
"Chl_C14_00368" 1933 "s59E25H2" 0 "Serine acetyltransferase"
"Chl_C14_00368" 1933 "s59E5H2" 0 "Serine acetyltransferase"
"Chl_C14_00368" 1933 "s63E6H2" 0 "Serine acetyltransferase"
"Chl_C14_00369" 1934 "s59E13H2" 0 "O-acetylserine sulfhydrylase"
"Chl_C14_00369" 1934 "s59E25H2" 0 "O-acetylserine sulfhydrylase"
"Chl_C14_00369" 1934 "s59E5H2" 0 "O-acetylserine sulfhydrylase"
"Chl_C14_00369" 1934 "s63E6H2" 0 "O-acetylserine sulfhydrylase"
"Chl_C14_00370" 1935 "s59E13H2" 0 "HTH-type transcriptional regulator CymR"
"Chl_C14_00370" 1935 "s59E25H2" 0 "HTH-type transcriptional regulator CymR"
"Chl_C14_00370" 1935 "s59E5H2" 0 "HTH-type transcriptional regulator CymR"
"Chl_C14_00370" 1935 "s63E6H2" 0 "HTH-type transcriptional regulator CymR"
"Chl_C14_00371" 1936 "s59E13H2" 0 "hypothetical protein"
"Chl_C14_00371" 1936 "s59E25H2" 0 "hypothetical protein"
"Chl_C14_00371" 1936 "s59E5H2" 0 "hypothetical protein"
"Chl_C14_00371" 1936 "s63E6H2" 0 "hypothetical protein"
"Chl_C14_00372" 1937 "s59E13H2" 0 "V4R domain protein"
"Chl_C14_00372" 1937 "s59E25H2" 0 "V4R domain protein"
"Chl_C14_00372" 1937 "s59E5H2" 0 "V4R domain protein"
"Chl_C14_00372" 1937 "s63E6H2" 0 "V4R domain protein"
"Chl_C14_00373" 1938 "s59E13H2" 0 "Cyclic di-GMP phosphodiesterase response regulator RpfG"
"Chl_C14_00373" 1938 "s59E25H2" 0 "Cyclic di-GMP phosphodiesterase response regulator RpfG"
"Chl_C14_00373" 1938 "s59E5H2" 0 "Cyclic di-GMP phosphodiesterase response regulator RpfG"
"Chl_C14_00373" 1938 "s63E6H2" 0 "Cyclic di-GMP phosphodiesterase response regulator RpfG"
"Chl_C14_00374" 1939 "s59E13H2" 0 "Type II secretion system protein E"
"Chl_C14_00374" 1939 "s59E25H2" 0 "Type II secretion system protein E"
"Chl_C14_00374" 1939 "s59E5H2" 0 "Type II secretion system protein E"
"Chl_C14_00374" 1939 "s63E6H2" 0 "Type II secretion system protein E"
"Chl_C14_00375" 1940 "s59E13H2" 0 "Type II secretion system protein F"
"Chl_C14_00375" 1940 "s59E25H2" 0 "Type II secretion system protein F"
"Chl_C14_00375" 1940 "s59E5H2" 0 "Type II secretion system protein F"
"Chl_C14_00375" 1940 "s63E6H2" 0 "Type II secretion system protein F"
"Chl_C14_00376" 1941 "s59E13H2" 0 "hypothetical protein"
"Chl_C14_00376" 1941 "s59E25H2" 0 "hypothetical protein"
"Chl_C14_00376" 1941 "s59E5H2" 0 "hypothetical protein"
"Chl_C14_00376" 1941 "s63E6H2" 0 "hypothetical protein"
"Chl_C14_00377" 1942 "s59E13H2" 0 "hypothetical protein"

"Chl_C14_00377" 1942 "s59E25H2" 0 "hypothetical protein"
"Chl_C14_00377" 1942 "s59E5H2" 0 "hypothetical protein"
"Chl_C14_00377" 1942 "s63E6H2" 0 "hypothetical protein"
"Chl_C14_00378" 1943 "s59E13H2" 0 "hypothetical protein"
"Chl_C14_00378" 1943 "s59E25H2" 0 "hypothetical protein"
"Chl_C14_00378" 1943 "s59E5H2" 0 "hypothetical protein"
"Chl_C14_00378" 1943 "s63E6H2" 0 "hypothetical protein"
"Chl_C14_00379" 1944 "s59E13H2" 0 "hypothetical protein"
"Chl_C14_00379" 1944 "s59E25H2" 0 "hypothetical protein"
"Chl_C14_00379" 1944 "s59E5H2" 0 "hypothetical protein"
"Chl_C14_00379" 1944 "s63E6H2" 0 "hypothetical protein"
"Chl_C14_00380" 1945 "s59E13H2" 0 "Competence protein A"
"Chl_C14_00380" 1945 "s59E25H2" 0 "Competence protein A"
"Chl_C14_00380" 1945 "s59E5H2" 0 "Competence protein A"
"Chl_C14_00380" 1945 "s63E6H2" 0 "Competence protein A"
"Chl_C14_00381" 1946 "s59E13H2" 0 "Twitching mobility protein"
"Chl_C14_00381" 1946 "s59E25H2" 0 "Twitching mobility protein"
"Chl_C14_00381" 1946 "s59E5H2" 0 "Twitching mobility protein"
"Chl_C14_00381" 1946 "s63E6H2" 0 "Twitching mobility protein"
"Chl_C14_00382" 1947 "s59E13H2" 0 "Phytochrome-like protein cph2"
"Chl_C14_00382" 1947 "s59E25H2" 0 "Phytochrome-like protein cph2"
"Chl_C14_00382" 1947 "s59E5H2" 0 "Phytochrome-like protein cph2"
"Chl_C14_00382" 1947 "s63E6H2" 0 "Phytochrome-like protein cph2"
"Chl_C14_00383" 1948 "s59E13H2" 0 "Cysteine-rich secretory protein family protein"
"Chl_C14_00383" 1948 "s59E25H2" 0 "Cysteine-rich secretory protein family protein"
"Chl_C14_00383" 1948 "s59E5H2" 0 "Cysteine-rich secretory protein family protein"
"Chl_C14_00383" 1948 "s63E6H2" 0 "Cysteine-rich secretory protein family protein"
"Chl_C14_00384" 1949 "s59E13H2" 0 "hypothetical protein"
"Chl_C14_00384" 1949 "s59E25H2" 0 "hypothetical protein"
"Chl_C14_00384" 1949 "s59E5H2" 0 "hypothetical protein"
"Chl_C14_00384" 1949 "s63E6H2" 0 "hypothetical protein"
"Chl_C14_00385" 1950 "s59E13H2" 0 "hypothetical protein"
"Chl_C14_00385" 1950 "s59E25H2" 0 "hypothetical protein"
"Chl_C14_00385" 1950 "s59E5H2" 0 "hypothetical protein"
"Chl_C14_00385" 1950 "s63E6H2" 0 "hypothetical protein"
"Chl_C14_00386" 1951 "s59E13H2" 0 "hypothetical protein"
"Chl_C14_00386" 1951 "s59E25H2" 0 "hypothetical protein"
"Chl_C14_00386" 1951 "s59E5H2" 0 "hypothetical protein"
"Chl_C14_00386" 1951 "s63E6H2" 0 "hypothetical protein"
"Chl_C14_00387" 1952 "s59E13H2" 0 "Chagasin family peptidase inhibitor I42"
"Chl_C14_00387" 1952 "s59E25H2" 0 "Chagasin family peptidase inhibitor I42"
"Chl_C14_00387" 1952 "s59E5H2" 0 "Chagasin family peptidase inhibitor I42"
"Chl_C14_00387" 1952 "s63E6H2" 0 "Chagasin family peptidase inhibitor I42"
"Chl_C14_00388" 1953 "s59E13H2" 0 "Multidrug resistance protein stp"
"Chl_C14_00388" 1953 "s59E25H2" 0 "Multidrug resistance protein stp"
"Chl_C14_00388" 1953 "s59E5H2" 0 "Multidrug resistance protein stp"
"Chl_C14_00388" 1953 "s63E6H2" 0 "Multidrug resistance protein stp"

"Chl_C14_00390" 1955 "s59E13H2" 0 "hypothetical protein"
"Chl_C14_00390" 1955 "s59E25H2" 0 "hypothetical protein"
"Chl_C14_00390" 1955 "s59E5H2" 0 "hypothetical protein"
"Chl_C14_00390" 1955 "s63E6H2" 0 "hypothetical protein"
"Chl_C14_00391" 1956 "s59E13H2" 0 "hypothetical protein"
"Chl_C14_00391" 1956 "s59E25H2" 0 "hypothetical protein"
"Chl_C14_00391" 1956 "s59E5H2" 0 "hypothetical protein"
"Chl_C14_00391" 1956 "s63E6H2" 0 "hypothetical protein"
"Chl_C14_00392" 1957 "s59E13H2" 0 "1-deoxy-D-xylulose-5-phosphate synthase"
"Chl_C14_00392" 1957 "s59E25H2" 0 "1-deoxy-D-xylulose-5-phosphate synthase"
"Chl_C14_00392" 1957 "s59E5H2" 0 "1-deoxy-D-xylulose-5-phosphate synthase"
"Chl_C14_00392" 1957 "s63E6H2" 0 "1-deoxy-D-xylulose-5-phosphate synthase"
"Chl_C14_00393" 1958 "s59E13H2" 0.006228054 "Transketolase 2"
"Chl_C14_00393" 1958 "s59E25H2" 0 "Transketolase 2"
"Chl_C14_00393" 1958 "s59E5H2" 0.01505641 "Transketolase 2"
"Chl_C14_00393" 1958 "s63E6H2" 0 "Transketolase 2"
"Chl_C14_00394" 1959 "s59E13H2" 0 "hypothetical protein"
"Chl_C14_00394" 1959 "s59E25H2" 0 "hypothetical protein"
"Chl_C14_00394" 1959 "s59E5H2" 0 "hypothetical protein"
"Chl_C14_00394" 1959 "s63E6H2" 0 "hypothetical protein"
"Chl_C14_00395" 1960 "s59E13H2" 0 "Sodium:neurotransmitter symporter family protein"
"Chl_C14_00395" 1960 "s59E25H2" 0 "Sodium:neurotransmitter symporter family protein"
"Chl_C14_00395" 1960 "s59E5H2" 0.001142724 "Sodium:neurotransmitter symporter family protein"
"Chl_C14_00395" 1960 "s63E6H2" 0.0004405282 "Sodium:neurotransmitter symporter family protein"
"Chl_C14_00396" 1961 "s59E13H2" 0 "Amidohydrolase"
"Chl_C14_00396" 1961 "s59E25H2" 0 "Amidohydrolase"
"Chl_C14_00396" 1961 "s59E5H2" 0 "Amidohydrolase"
"Chl_C14_00396" 1961 "s63E6H2" 0 "Amidohydrolase"
"Chl_C14_00397" 1962 "s59E13H2" 0 "hypothetical protein"
"Chl_C14_00397" 1962 "s59E25H2" 0 "hypothetical protein"
"Chl_C14_00397" 1962 "s59E5H2" 0 "hypothetical protein"
"Chl_C14_00397" 1962 "s63E6H2" 0 "hypothetical protein"
"Chl_C14_00398" 1963 "s59E13H2" 0 "D-2-hydroxyacid dehydrogenase"
"Chl_C14_00398" 1963 "s59E25H2" 0 "D-2-hydroxyacid dehydrogenase"
"Chl_C14_00398" 1963 "s59E5H2" 0 "D-2-hydroxyacid dehydrogenase"
"Chl_C14_00398" 1963 "s63E6H2" 0 "D-2-hydroxyacid dehydrogenase"
"Chl_C14_00399" 1964 "s59E13H2" 0 "Glutamate-1-semialdehyde 2,1-aminomutase"
"Chl_C14_00399" 1964 "s59E25H2" 0 "Glutamate-1-semialdehyde 2,1-aminomutase"
"Chl_C14_00399" 1964 "s59E5H2" 0 "Glutamate-1-semialdehyde 2,1-aminomutase"
"Chl_C14_00399" 1964 "s63E6H2" 0 "Glutamate-1-semialdehyde 2,1-aminomutase"
"Chl_C14_00400" 1965 "s59E13H2" 0 "putative amino-acid racemase"
"Chl_C14_00400" 1965 "s59E25H2" 0 "putative amino-acid racemase"
"Chl_C14_00400" 1965 "s59E5H2" 0 "putative amino-acid racemase"
"Chl_C14_00400" 1965 "s63E6H2" 0 "putative amino-acid racemase"
"Chl_C14_00401" 1966 "s59E13H2" 0 "3-keto-5-aminohexanoate cleavage enzyme"
"Chl_C14_00401" 1966 "s59E25H2" 0 "3-keto-5-aminohexanoate cleavage enzyme"
"Chl_C14_00401" 1966 "s59E5H2" 0 "3-keto-5-aminohexanoate cleavage enzyme"

"Chl_C14_00401" 1966 "s63E6H2" 0 "3-keto-5-aminohexanoate cleavage enzyme"
"Chl_C14_00402" 1967 "s59E13H2" 0 "3-chloro-4-hydroxyphenylacetate reductive dehalogenase precursor"
"Chl_C14_00402" 1967 "s59E25H2" 0 "3-chloro-4-hydroxyphenylacetate reductive dehalogenase precursor"
"Chl_C14_00402" 1967 "s59E5H2" 0 "3-chloro-4-hydroxyphenylacetate reductive dehalogenase precursor"
"Chl_C14_00402" 1967 "s63E6H2" 0 "3-chloro-4-hydroxyphenylacetate reductive dehalogenase precursor"
"Chl_C14_00403" 1968 "s59E13H2" 0 "Methylthioribose-1-phosphate isomerase"
"Chl_C14_00403" 1968 "s59E25H2" 0 "Methylthioribose-1-phosphate isomerase"
"Chl_C14_00403" 1968 "s59E5H2" 0.003895127 "Methylthioribose-1-phosphate isomerase"
"Chl_C14_00403" 1968 "s63E6H2" 0.0005369173 "Methylthioribose-1-phosphate isomerase"
"Chl_C14_00404" 1969 "s59E13H2" 0 "Lactoylglutathione lyase"
"Chl_C14_00404" 1969 "s59E25H2" 0 "Lactoylglutathione lyase"
"Chl_C14_00404" 1969 "s59E5H2" 0 "Lactoylglutathione lyase"
"Chl_C14_00404" 1969 "s63E6H2" 0 "Lactoylglutathione lyase"
"Chl_C14_00405" 1970 "s59E13H2" 0 "Molybdopterin adenylyltransferase"
"Chl_C14_00405" 1970 "s59E25H2" 0 "Molybdopterin adenylyltransferase"
"Chl_C14_00405" 1970 "s59E5H2" 0 "Molybdopterin adenylyltransferase"
"Chl_C14_00405" 1970 "s63E6H2" 0 "Molybdopterin adenylyltransferase"
"Chl_C14_00406" 1971 "s59E13H2" 0 "tRNA (guanine-N(1)-)-methyltransferase"
"Chl_C14_00406" 1971 "s59E25H2" 0 "tRNA (guanine-N(1)-)-methyltransferase"
"Chl_C14_00406" 1971 "s59E5H2" 0 "tRNA (guanine-N(1)-)-methyltransferase"
"Chl_C14_00406" 1971 "s63E6H2" 0 "tRNA (guanine-N(1)-)-methyltransferase"
"Chl_C14_00407" 1972 "s59E13H2" 0 "50S ribosomal protein L19"
"Chl_C14_00407" 1972 "s59E25H2" 0 "50S ribosomal protein L19"
"Chl_C14_00407" 1972 "s59E5H2" 0 "50S ribosomal protein L19"
"Chl_C14_00407" 1972 "s63E6H2" 0 "50S ribosomal protein L19"
"Chl_C14_00408" 1973 "s59E13H2" 0.0006750514 "Primosomal protein N'"
"Chl_C14_00408" 1973 "s59E25H2" 0 "Primosomal protein N'"
"Chl_C14_00408" 1973 "s59E5H2" 0 "Primosomal protein N'"
"Chl_C14_00408" 1973 "s63E6H2" 0 "Primosomal protein N'"
"Chl_C14_00409" 1974 "s59E13H2" 0 "Alpha-keto acid-binding periplasmic protein TakP precursor"
"Chl_C14_00409" 1974 "s59E25H2" 0 "Alpha-keto acid-binding periplasmic protein TakP precursor"
"Chl_C14_00409" 1974 "s59E5H2" 0 "Alpha-keto acid-binding periplasmic protein TakP precursor"
"Chl_C14_00409" 1974 "s63E6H2" 0 "Alpha-keto acid-binding periplasmic protein TakP precursor"
"Chl_C14_00410" 1975 "s59E13H2" 0 "Tripartite ATP-independent periplasmic transporters, DctQ component"
"Chl_C14_00410" 1975 "s59E25H2" 0 "Tripartite ATP-independent periplasmic transporters, DctQ component"
"Chl_C14_00410" 1975 "s59E5H2" 0 "Tripartite ATP-independent periplasmic transporters, DctQ component"
"Chl_C14_00410" 1975 "s63E6H2" 0 "Tripartite ATP-independent periplasmic transporters, DctQ component"
"Chl_C14_00411" 1976 "s59E13H2" 0 "Sialic acid TRAP transporter permease protein SiaT"
"Chl_C14_00411" 1976 "s59E25H2" 0 "Sialic acid TRAP transporter permease protein SiaT"
"Chl_C14_00411" 1976 "s59E5H2" 0 "Sialic acid TRAP transporter permease protein SiaT"
"Chl_C14_00411" 1976 "s63E6H2" 0 "Sialic acid TRAP transporter permease protein SiaT"
"Chl_C14_00413" 1978 "s59E13H2" 0 "SsrA-binding protein"
"Chl_C14_00413" 1978 "s59E25H2" 0 "SsrA-binding protein"
"Chl_C14_00413" 1978 "s59E5H2" 0.008750849 "SsrA-binding protein"
"Chl_C14_00413" 1978 "s63E6H2" 0 "SsrA-binding protein"
"Chl_C14_00414" 1979 "s59E13H2" 0 "Tetracycline resistance protein, class B"
"Chl_C14_00414" 1979 "s59E25H2" 0 "Tetracycline resistance protein, class B"

"Chl_C14_00414" 1979 "s59E5H2" 0 "Tetracycline resistance protein, class B"
"Chl_C14_00414" 1979 "s63E6H2" 0 "Tetracycline resistance protein, class B"
"Chl_C14_00415" 1980 "s59E13H2" 0 "Threonine--tRNA ligase 1"
"Chl_C14_00415" 1980 "s59E25H2" 0 "Threonine--tRNA ligase 1"
"Chl_C14_00415" 1980 "s59E5H2" 0 "Threonine--tRNA ligase 1"
"Chl_C14_00415" 1980 "s63E6H2" 0 "Threonine--tRNA ligase 1"
"Chl_C14_00416" 1981 "s59E13H2" 0 "Translation initiation factor IF-3"
"Chl_C14_00416" 1981 "s59E25H2" 0 "Translation initiation factor IF-3"
"Chl_C14_00416" 1981 "s59E5H2" 0 "Translation initiation factor IF-3"
"Chl_C14_00416" 1981 "s63E6H2" 0 "Translation initiation factor IF-3"
"Chl_C14_00417" 1982 "s59E13H2" 0 "50S ribosomal protein L35"
"Chl_C14_00417" 1982 "s59E25H2" 0 "50S ribosomal protein L35"
"Chl_C14_00417" 1982 "s59E5H2" 0 "50S ribosomal protein L35"
"Chl_C14_00417" 1982 "s63E6H2" 0 "50S ribosomal protein L35"
"Chl_C14_00418" 1983 "s59E13H2" 0 "50S ribosomal protein L20"
"Chl_C14_00418" 1983 "s59E25H2" 0 "50S ribosomal protein L20"
"Chl_C14_00418" 1983 "s59E5H2" 0 "50S ribosomal protein L20"
"Chl_C14_00418" 1983 "s63E6H2" 0 "50S ribosomal protein L20"
"Chl_C14_00419" 1984 "s59E13H2" 0 "Major Facilitator Superfamily protein"
"Chl_C14_00419" 1984 "s59E25H2" 0 "Major Facilitator Superfamily protein"
"Chl_C14_00419" 1984 "s59E5H2" 0 "Major Facilitator Superfamily protein"
"Chl_C14_00419" 1984 "s63E6H2" 0 "Major Facilitator Superfamily protein"
"Chl_C14_00420" 1985 "s59E13H2" 0 "Phenylalanine--tRNA ligase alpha subunit"
"Chl_C14_00420" 1985 "s59E25H2" 0 "Phenylalanine--tRNA ligase alpha subunit"
"Chl_C14_00420" 1985 "s59E5H2" 0 "Phenylalanine--tRNA ligase alpha subunit"
"Chl_C14_00420" 1985 "s63E6H2" 0 "Phenylalanine--tRNA ligase alpha subunit"
"Chl_C14_00421" 1986 "s59E13H2" 0 "Phenylalanine--tRNA ligase beta subunit"
"Chl_C14_00421" 1986 "s59E25H2" 0 "Phenylalanine--tRNA ligase beta subunit"
"Chl_C14_00421" 1986 "s59E5H2" 0 "Phenylalanine--tRNA ligase beta subunit"
"Chl_C14_00421" 1986 "s63E6H2" 0 "Phenylalanine--tRNA ligase beta subunit"
"Chl_C14_00422" 1987 "s59E13H2" 0 "Proline--tRNA ligase"
"Chl_C14_00422" 1987 "s59E25H2" 0 "Proline--tRNA ligase"
"Chl_C14_00422" 1987 "s59E5H2" 0 "Proline--tRNA ligase"
"Chl_C14_00422" 1987 "s63E6H2" 0 "Proline--tRNA ligase"
"Chl_C14_00423" 1988 "s59E13H2" 0 "50S ribosomal protein L15"
"Chl_C14_00423" 1988 "s59E25H2" 0 "50S ribosomal protein L15"
"Chl_C14_00423" 1988 "s59E5H2" 0 "50S ribosomal protein L15"
"Chl_C14_00423" 1988 "s63E6H2" 0 "50S ribosomal protein L15"
"Chl_C14_00424" 1989 "s59E13H2" 0 "preprotein translocase subunit SecY"
"Chl_C14_00424" 1989 "s59E25H2" 0 "preprotein translocase subunit SecY"
"Chl_C14_00424" 1989 "s59E5H2" 0 "preprotein translocase subunit SecY"
"Chl_C14_00424" 1989 "s63E6H2" 0 "preprotein translocase subunit SecY"
"Chl_C14_00425" 1990 "s59E13H2" 0 "Adenylate kinase"
"Chl_C14_00425" 1990 "s59E25H2" 0 "Adenylate kinase"
"Chl_C14_00425" 1990 "s59E5H2" 0 "Adenylate kinase"
"Chl_C14_00425" 1990 "s63E6H2" 0.0007542317 "Adenylate kinase"
"Chl_C14_00426" 1991 "s59E13H2" 0 "Methionine aminopeptidase 1"

"Chl_C14_00426" 1991 "s59E25H2" 0 "Methionine aminopeptidase 1"
"Chl_C14_00426" 1991 "s59E5H2" 0.001923792 "Methionine aminopeptidase 1"
"Chl_C14_00426" 1991 "s63E6H2" 0.00231013 "Methionine aminopeptidase 1"
"Chl_C14_00427" 1992 "s59E13H2" 0 "Translation initiation factor IF-1"
"Chl_C14_00427" 1992 "s59E25H2" 0 "Translation initiation factor IF-1"
"Chl_C14_00427" 1992 "s59E5H2" 0 "Translation initiation factor IF-1"
"Chl_C14_00427" 1992 "s63E6H2" 0 "Translation initiation factor IF-1"
"Chl_C14_00428" 1993 "s59E13H2" 0 "30S ribosomal protein S13"
"Chl_C14_00428" 1993 "s59E25H2" 0 "30S ribosomal protein S13"
"Chl_C14_00428" 1993 "s59E5H2" 0 "30S ribosomal protein S13"
"Chl_C14_00428" 1993 "s63E6H2" 0 "30S ribosomal protein S13"
"Chl_C14_00429" 1994 "s59E13H2" 0 "30S ribosomal protein S11"
"Chl_C14_00429" 1994 "s59E25H2" 0 "30S ribosomal protein S11"
"Chl_C14_00429" 1994 "s59E5H2" 0 "30S ribosomal protein S11"
"Chl_C14_00429" 1994 "s63E6H2" 0.009358703 "30S ribosomal protein S11"
"Chl_C14_00430" 1995 "s59E13H2" 0 "30S ribosomal protein S4"
"Chl_C14_00430" 1995 "s59E25H2" 0 "30S ribosomal protein S4"
"Chl_C14_00430" 1995 "s59E5H2" 0 "30S ribosomal protein S4"
"Chl_C14_00430" 1995 "s63E6H2" 0 "30S ribosomal protein S4"
"Chl_C14_00431" 1996 "s59E13H2" 0 "DNA-directed RNA polymerase subunit alpha"
"Chl_C14_00431" 1996 "s59E25H2" 0 "DNA-directed RNA polymerase subunit alpha"
"Chl_C14_00431" 1996 "s59E5H2" 0 "DNA-directed RNA polymerase subunit alpha"
"Chl_C14_00431" 1996 "s63E6H2" 0 "DNA-directed RNA polymerase subunit alpha"
"Chl_C14_00432" 1997 "s59E13H2" 0 "50S ribosomal protein L17"
"Chl_C14_00432" 1997 "s59E25H2" 0 "50S ribosomal protein L17"
"Chl_C14_00432" 1997 "s59E5H2" 0 "50S ribosomal protein L17"
"Chl_C14_00432" 1997 "s63E6H2" 0 "50S ribosomal protein L17"
"Chl_C14_00433" 1998 "s59E13H2" 0 "tRNA pseudouridine synthase A"
"Chl_C14_00433" 1998 "s59E25H2" 0 "tRNA pseudouridine synthase A"
"Chl_C14_00433" 1998 "s59E5H2" 0 "tRNA pseudouridine synthase A"
"Chl_C14_00433" 1998 "s63E6H2" 0 "tRNA pseudouridine synthase A"
"Chl_C14_00434" 1999 "s59E13H2" 0 "50S ribosomal protein L13"
"Chl_C14_00434" 1999 "s59E25H2" 0 "50S ribosomal protein L13"
"Chl_C14_00434" 1999 "s59E5H2" 0 "50S ribosomal protein L13"
"Chl_C14_00434" 1999 "s63E6H2" 0 "50S ribosomal protein L13"
"Chl_C14_00435" 2000 "s59E13H2" 0 "30S ribosomal protein S9"
"Chl_C14_00435" 2000 "s59E25H2" 0 "30S ribosomal protein S9"
"Chl_C14_00435" 2000 "s59E5H2" 0 "30S ribosomal protein S9"
"Chl_C14_00435" 2000 "s63E6H2" 0 "30S ribosomal protein S9"
"Chl_C14_00437" 2002 "s59E13H2" 0 "AIG2-like family protein"
"Chl_C14_00437" 2002 "s59E25H2" 0 "AIG2-like family protein"
"Chl_C14_00437" 2002 "s59E5H2" 0 "AIG2-like family protein"
"Chl_C14_00437" 2002 "s63E6H2" 0 "AIG2-like family protein"
"Chl_C14_00438" 2003 "s59E13H2" 0 "Bifunctional phosphoglucose/phosphomannose isomerase"
"Chl_C14_00438" 2003 "s59E25H2" 0 "Bifunctional phosphoglucose/phosphomannose isomerase"
"Chl_C14_00438" 2003 "s59E5H2" 0 "Bifunctional phosphoglucose/phosphomannose isomerase"
"Chl_C14_00438" 2003 "s63E6H2" 0 "Bifunctional phosphoglucose/phosphomannose isomerase"

"Chl_C14_00439" 2004 "s59E13H2" 0.0004369737 "Phosphoglucosamine mutase"
"Chl_C14_00439" 2004 "s59E25H2" 0 "Phosphoglucosamine mutase"
"Chl_C14_00439" 2004 "s59E5H2" 0 "Phosphoglucosamine mutase"
"Chl_C14_00439" 2004 "s63E6H2" 0 "Phosphoglucosamine mutase"
"Chl_C14_00440" 2005 "s59E13H2" 0 "S-adenosylmethionine synthase"
"Chl_C14_00440" 2005 "s59E25H2" 0 "S-adenosylmethionine synthase"
"Chl_C14_00440" 2005 "s59E5H2" 0.008859745 "S-adenosylmethionine synthase"
"Chl_C14_00440" 2005 "s63E6H2" 0 "S-adenosylmethionine synthase"
"Chl_C14_00441" 2006 "s59E13H2" 0 "Adenosylhomocysteinase"
"Chl_C14_00441" 2006 "s59E25H2" 0 "Adenosylhomocysteinase"
"Chl_C14_00441" 2006 "s59E5H2" 0 "Adenosylhomocysteinase"
"Chl_C14_00441" 2006 "s63E6H2" 0 "Adenosylhomocysteinase"
"Chl_C14_00442" 2007 "s59E13H2" 0 "Chromatin associated protein KTI12"
"Chl_C14_00442" 2007 "s59E25H2" 0 "Chromatin associated protein KTI12"
"Chl_C14_00442" 2007 "s59E5H2" 0 "Chromatin associated protein KTI12"
"Chl_C14_00442" 2007 "s63E6H2" 0 "Chromatin associated protein KTI12"
"Chl_C14_00443" 2008 "s59E13H2" 0.007142141 "GIY-YIG nuclease superfamily protein"
"Chl_C14_00443" 2008 "s59E25H2" 0 "GIY-YIG nuclease superfamily protein"
"Chl_C14_00443" 2008 "s59E5H2" 0.01043514 "GIY-YIG nuclease superfamily protein"
"Chl_C14_00443" 2008 "s63E6H2" 0 "GIY-YIG nuclease superfamily protein"
"Chl_C14_00445" 2010 "s59E13H2" 0 "Acetylene hydratase"
"Chl_C14_00445" 2010 "s59E25H2" 0 "Acetylene hydratase"
"Chl_C14_00445" 2010 "s59E5H2" 0 "Acetylene hydratase"
"Chl_C14_00445" 2010 "s63E6H2" 0 "Acetylene hydratase"
"Chl_C14_00446" 2011 "s59E13H2" 0 "Coenzyme F420 hydrogenase subunit beta"
"Chl_C14_00446" 2011 "s59E25H2" 0 "Coenzyme F420 hydrogenase subunit beta"
"Chl_C14_00446" 2011 "s59E5H2" 0 "Coenzyme F420 hydrogenase subunit beta"
"Chl_C14_00446" 2011 "s63E6H2" 0 "Coenzyme F420 hydrogenase subunit beta"
"Chl_C14_00448" 2013 "s59E13H2" 0 "hypothetical protein"
"Chl_C14_00448" 2013 "s59E25H2" 0 "hypothetical protein"
"Chl_C14_00448" 2013 "s59E5H2" 0 "hypothetical protein"
"Chl_C14_00448" 2013 "s63E6H2" 0 "hypothetical protein"
"Chl_C14_00449" 2014 "s59E13H2" 0 "hypothetical protein"
"Chl_C14_00449" 2014 "s59E25H2" 0 "hypothetical protein"
"Chl_C14_00449" 2014 "s59E5H2" 0 "hypothetical protein"
"Chl_C14_00449" 2014 "s63E6H2" 0 "hypothetical protein"
"Chl_C14_00451" 2016 "s59E13H2" 0 "Ribonuclease Y"
"Chl_C14_00451" 2016 "s59E25H2" 0 "Ribonuclease Y"
"Chl_C14_00451" 2016 "s59E5H2" 0 "Ribonuclease Y"
"Chl_C14_00451" 2016 "s63E6H2" 0 "Ribonuclease Y"
"Chl_C14_00452" 2017 "s59E13H2" 0 "Regulatory protein RecX"
"Chl_C14_00452" 2017 "s59E25H2" 0 "Regulatory protein RecX"
"Chl_C14_00452" 2017 "s59E5H2" 0 "Regulatory protein RecX"
"Chl_C14_00452" 2017 "s63E6H2" 0 "Regulatory protein RecX"
"Chl_C14_00453" 2018 "s59E13H2" 0 "DNA repair and recombination protein RadA"
"Chl_C14_00453" 2018 "s59E25H2" 0 "DNA repair and recombination protein RadA"
"Chl_C14_00453" 2018 "s59E5H2" 0 "DNA repair and recombination protein RadA"

"Chl_C14_00453" 2018 "s63E6H2" 0.001077413 "DNA repair and recombination protein RadA"
"Chl_C14_00454" 2019 "s59E13H2" 0 "Methylmalonyl-CoA carboxyltransferase 12S subunit"
"Chl_C14_00454" 2019 "s59E25H2" 0 "Methylmalonyl-CoA carboxyltransferase 12S subunit"
"Chl_C14_00454" 2019 "s59E5H2" 0 "Methylmalonyl-CoA carboxyltransferase 12S subunit"
"Chl_C14_00454" 2019 "s63E6H2" 0 "Methylmalonyl-CoA carboxyltransferase 12S subunit"
"Chl_C14_00455" 2020 "s59E13H2" 0 "Glyoxalase/Bleomycin resistance protein/Dioxygenase superfamily protein"
"Chl_C14_00455" 2020 "s59E25H2" 0 "Glyoxalase/Bleomycin resistance protein/Dioxygenase superfamily protein"
"Chl_C14_00455" 2020 "s59E5H2" 0 "Glyoxalase/Bleomycin resistance protein/Dioxygenase superfamily protein"
"Chl_C14_00455" 2020 "s63E6H2" 0 "Glyoxalase/Bleomycin resistance protein/Dioxygenase superfamily protein"
"Chl_C14_00456" 2021 "s59E13H2" 0 "Methylmalonyl-CoA mutase"
"Chl_C14_00456" 2021 "s59E25H2" 0 "Methylmalonyl-CoA mutase"
"Chl_C14_00456" 2021 "s59E5H2" 0 "Methylmalonyl-CoA mutase"
"Chl_C14_00456" 2021 "s63E6H2" 0 "Methylmalonyl-CoA mutase"
"Chl_C14_00457" 2022 "s59E13H2" 0 "Methylmalonyl-CoA mutase"
"Chl_C14_00457" 2022 "s59E25H2" 0 "Methylmalonyl-CoA mutase"
"Chl_C14_00457" 2022 "s59E5H2" 0 "Methylmalonyl-CoA mutase"
"Chl_C14_00457" 2022 "s63E6H2" 0 "Methylmalonyl-CoA mutase"
"Chl_C14_00458" 2023 "s59E13H2" 0 "Methylmalonyl-CoA mutase"
"Chl_C14_00458" 2023 "s59E25H2" 0 "Methylmalonyl-CoA mutase"
"Chl_C14_00458" 2023 "s59E5H2" 0 "Methylmalonyl-CoA mutase"
"Chl_C14_00458" 2023 "s63E6H2" 0 "Methylmalonyl-CoA mutase"
"Chl_C14_00459" 2024 "s59E13H2" 0 "3-hydroxybutyryl-CoA dehydrogenase"
"Chl_C14_00459" 2024 "s59E25H2" 0 "3-hydroxybutyryl-CoA dehydrogenase"
"Chl_C14_00459" 2024 "s59E5H2" 0 "3-hydroxybutyryl-CoA dehydrogenase"
"Chl_C14_00459" 2024 "s63E6H2" 0 "3-hydroxybutyryl-CoA dehydrogenase"
"Chl_C14_00460" 2025 "s59E13H2" 0 "3-hydroxypropionyl-coenzyme A dehydratase"
"Chl_C14_00460" 2025 "s59E25H2" 0 "3-hydroxypropionyl-coenzyme A dehydratase"
"Chl_C14_00460" 2025 "s59E5H2" 0 "3-hydroxypropionyl-coenzyme A dehydratase"
"Chl_C14_00460" 2025 "s63E6H2" 0 "3-hydroxypropionyl-coenzyme A dehydratase"
"Chl_C14_00461" 2026 "s59E13H2" 0 "Carnitinyl-CoA dehydratase"
"Chl_C14_00461" 2026 "s59E25H2" 0 "Carnitinyl-CoA dehydratase"
"Chl_C14_00461" 2026 "s59E5H2" 0 "Carnitinyl-CoA dehydratase"
"Chl_C14_00461" 2026 "s63E6H2" 0 "Carnitinyl-CoA dehydratase"
"Chl_C14_00462" 2027 "s59E13H2" 0 "hypothetical protein"
"Chl_C14_00462" 2027 "s59E25H2" 0 "hypothetical protein"
"Chl_C14_00462" 2027 "s59E5H2" 0 "hypothetical protein"
"Chl_C14_00462" 2027 "s63E6H2" 0 "hypothetical protein"
"Chl_C14_00463" 2028 "s59E13H2" 0 "3-oxoacyl-[acyl-carrier-protein] synthase 3"
"Chl_C14_00463" 2028 "s59E25H2" 0 "3-oxoacyl-[acyl-carrier-protein] synthase 3"
"Chl_C14_00463" 2028 "s59E5H2" 0 "3-oxoacyl-[acyl-carrier-protein] synthase 3"
"Chl_C14_00463" 2028 "s63E6H2" 0 "3-oxoacyl-[acyl-carrier-protein] synthase 3"
"Chl_C14_00464" 2029 "s59E13H2" 0 "putative acetyl-CoA acyltransferase"
"Chl_C14_00464" 2029 "s59E25H2" 0 "putative acetyl-CoA acyltransferase"
"Chl_C14_00464" 2029 "s59E5H2" 0 "putative acetyl-CoA acyltransferase"
"Chl_C14_00464" 2029 "s63E6H2" 0 "putative acetyl-CoA acyltransferase"
"Chl_C14_00465" 2030 "s59E13H2" 0 "Tetratricopeptide repeat protein"
"Chl_C14_00465" 2030 "s59E25H2" 0 "Tetratricopeptide repeat protein"

"Chl_C14_00465" 2030 "s59E5H2" 0 "Tetratricopeptide repeat protein"
"Chl_C14_00465" 2030 "s63E6H2" 0 "Tetratricopeptide repeat protein"
"Chl_C14_00466" 2031 "s59E13H2" 0 "Thiol:disulfide interchange protein DsbD precursor"
"Chl_C14_00466" 2031 "s59E25H2" 0 "Thiol:disulfide interchange protein DsbD precursor"
"Chl_C14_00466" 2031 "s59E5H2" 0 "Thiol:disulfide interchange protein DsbD precursor"
"Chl_C14_00466" 2031 "s63E6H2" 0 "Thiol:disulfide interchange protein DsbD precursor"
"Chl_C14_00467" 2032 "s59E13H2" 0 "Thiol-disulfide oxidoreductase ResA"
"Chl_C14_00467" 2032 "s59E25H2" 0 "Thiol-disulfide oxidoreductase ResA"
"Chl_C14_00467" 2032 "s59E5H2" 0 "Thiol-disulfide oxidoreductase ResA"
"Chl_C14_00467" 2032 "s63E6H2" 0 "Thiol-disulfide oxidoreductase ResA"
"Chl_C14_00471" 2036 "s59E13H2" 0 "Zinc ribbon domain protein"
"Chl_C14_00471" 2036 "s59E25H2" 0 "Zinc ribbon domain protein"
"Chl_C14_00471" 2036 "s59E5H2" 0 "Zinc ribbon domain protein"
"Chl_C14_00471" 2036 "s63E6H2" 0.002786341 "Zinc ribbon domain protein"
"Chl_C14_00472" 2037 "s59E13H2" 0 "DNA gyrase subunit B"
"Chl_C14_00472" 2037 "s59E25H2" 0 "DNA gyrase subunit B"
"Chl_C14_00472" 2037 "s59E5H2" 0 "DNA gyrase subunit B"
"Chl_C14_00472" 2037 "s63E6H2" 0 "DNA gyrase subunit B"
"Chl_C14_00473" 2038 "s59E13H2" 0 "Radical SAM superfamily protein"
"Chl_C14_00473" 2038 "s59E25H2" 0 "Radical SAM superfamily protein"
"Chl_C14_00473" 2038 "s59E5H2" 0.002849901 "Radical SAM superfamily protein"
"Chl_C14_00473" 2038 "s63E6H2" 0 "Radical SAM superfamily protein"
"Chl_C14_00474" 2039 "s59E13H2" 0 "hypothetical protein"
"Chl_C14_00474" 2039 "s59E25H2" 0 "hypothetical protein"
"Chl_C14_00474" 2039 "s59E5H2" 0 "hypothetical protein"
"Chl_C14_00474" 2039 "s63E6H2" 0.000474073 "hypothetical protein"
"Chl_C14_00475" 2040 "s59E13H2" 0 "Nitroreductase family protein"
"Chl_C14_00475" 2040 "s59E25H2" 0 "Nitroreductase family protein"
"Chl_C14_00475" 2040 "s59E5H2" 0 "Nitroreductase family protein"
"Chl_C14_00475" 2040 "s63E6H2" 0 "Nitroreductase family protein"
"Chl_C14_00477" 2042 "s59E13H2" 0 "Replication-associated recombination protein A"
"Chl_C14_00477" 2042 "s59E25H2" 0 "Replication-associated recombination protein A"
"Chl_C14_00477" 2042 "s59E5H2" 0 "Replication-associated recombination protein A"
"Chl_C14_00477" 2042 "s63E6H2" 0 "Replication-associated recombination protein A"
"Chl_C14_00478" 2043 "s59E13H2" 0 "Folylpolyglutamate synthase"
"Chl_C14_00478" 2043 "s59E25H2" 0 "Folylpolyglutamate synthase"
"Chl_C14_00478" 2043 "s59E5H2" 0 "Folylpolyglutamate synthase"
"Chl_C14_00478" 2043 "s63E6H2" 0 "Folylpolyglutamate synthase"
"Chl_C14_00479" 2044 "s59E13H2" 0 "hypothetical protein"
"Chl_C14_00479" 2044 "s59E25H2" 0 "hypothetical protein"
"Chl_C14_00479" 2044 "s59E5H2" 0 "hypothetical protein"
"Chl_C14_00479" 2044 "s63E6H2" 0.0006055997 "hypothetical protein"
"Chl_C14_00480" 2045 "s59E13H2" 0 "Thiosulfate sulfurtransferase PspE precursor"
"Chl_C14_00480" 2045 "s59E25H2" 0 "Thiosulfate sulfurtransferase PspE precursor"
"Chl_C14_00480" 2045 "s59E5H2" 0 "Thiosulfate sulfurtransferase PspE precursor"
"Chl_C14_00480" 2045 "s63E6H2" 0 "Thiosulfate sulfurtransferase PspE precursor"
"Chl_C14_00481" 2046 "s59E13H2" 0 "DNA polymerase I"

"Chl_C14_00481" 2046 "s59E25H2" 0 "DNA polymerase I"
"Chl_C14_00481" 2046 "s59E5H2" 0 "DNA polymerase I"
"Chl_C14_00481" 2046 "s63E6H2" 0 "DNA polymerase I"
"Chl_C14_00482" 2047 "s59E13H2" 0 "Formamidopyrimidine-DNA glycosylase"
"Chl_C14_00482" 2047 "s59E25H2" 0 "Formamidopyrimidine-DNA glycosylase"
"Chl_C14_00482" 2047 "s59E5H2" 0 "Formamidopyrimidine-DNA glycosylase"
"Chl_C14_00482" 2047 "s63E6H2" 0 "Formamidopyrimidine-DNA glycosylase"
"Chl_C14_00483" 2048 "s59E13H2" 0 "hypothetical protein"
"Chl_C14_00483" 2048 "s59E25H2" 0 "hypothetical protein"
"Chl_C14_00483" 2048 "s59E5H2" 0 "hypothetical protein"
"Chl_C14_00483" 2048 "s63E6H2" 0 "hypothetical protein"
"Chl_C14_00484" 2049 "s59E13H2" 0 "Succinyl-CoA:(R)-benzylsuccinate CoA-transferase subunit BbsF"
"Chl_C14_00484" 2049 "s59E25H2" 0 "Succinyl-CoA:(R)-benzylsuccinate CoA-transferase subunit BbsF"
"Chl_C14_00484" 2049 "s59E5H2" 0 "Succinyl-CoA:(R)-benzylsuccinate CoA-transferase subunit BbsF"
"Chl_C14_00484" 2049 "s63E6H2" 0 "Succinyl-CoA:(R)-benzylsuccinate CoA-transferase subunit BbsF"
"Chl_C14_00486" 2051 "s59E13H2" 0 "hypothetical protein"
"Chl_C14_00486" 2051 "s59E25H2" 0 "hypothetical protein"
"Chl_C14_00486" 2051 "s59E5H2" 0 "hypothetical protein"
"Chl_C14_00486" 2051 "s63E6H2" 0 "hypothetical protein"
"Chl_C14_00487" 2052 "s59E13H2" 0 "Succinyl-CoA:(R)-benzylsuccinate CoA-transferase subunit BbsE"
"Chl_C14_00487" 2052 "s59E25H2" 0 "Succinyl-CoA:(R)-benzylsuccinate CoA-transferase subunit BbsE"
"Chl_C14_00487" 2052 "s59E5H2" 0 "Succinyl-CoA:(R)-benzylsuccinate CoA-transferase subunit BbsE"
"Chl_C14_00487" 2052 "s63E6H2" 0 "Succinyl-CoA:(R)-benzylsuccinate CoA-transferase subunit BbsE"
"Chl_C14_00488" 2053 "s59E13H2" 0 "Glutamate--tRNA ligase"
"Chl_C14_00488" 2053 "s59E25H2" 0 "Glutamate--tRNA ligase"
"Chl_C14_00488" 2053 "s59E5H2" 0 "Glutamate--tRNA ligase"
"Chl_C14_00488" 2053 "s63E6H2" 0 "Glutamate--tRNA ligase"
"Chl_C14_00489" 2054 "s59E13H2" 0 "Ribosomal large subunit pseudouridine synthase D"
"Chl_C14_00489" 2054 "s59E25H2" 0 "Ribosomal large subunit pseudouridine synthase D"
"Chl_C14_00489" 2054 "s59E5H2" 0 "Ribosomal large subunit pseudouridine synthase D"
"Chl_C14_00489" 2054 "s63E6H2" 0 "Ribosomal large subunit pseudouridine synthase D"
"Chl_C14_00490" 2055 "s59E13H2" 0 "Lipoprotein signal peptidase"
"Chl_C14_00490" 2055 "s59E25H2" 0 "Lipoprotein signal peptidase"
"Chl_C14_00490" 2055 "s59E5H2" 0 "Lipoprotein signal peptidase"
"Chl_C14_00490" 2055 "s63E6H2" 0 "Lipoprotein signal peptidase"
"Chl_C14_00491" 2056 "s59E13H2" 0 "RNA polymerase-binding transcription factor DksA"
"Chl_C14_00491" 2056 "s59E25H2" 0 "RNA polymerase-binding transcription factor DksA"
"Chl_C14_00491" 2056 "s59E5H2" 0 "RNA polymerase-binding transcription factor DksA"
"Chl_C14_00491" 2056 "s63E6H2" 0 "RNA polymerase-binding transcription factor DksA"
"Chl_C14_00492" 2057 "s59E13H2" 0 "hypothetical protein"
"Chl_C14_00492" 2057 "s59E25H2" 0 "hypothetical protein"
"Chl_C14_00492" 2057 "s59E5H2" 0 "hypothetical protein"
"Chl_C14_00492" 2057 "s63E6H2" 0 "hypothetical protein"
"Chl_C14_00493" 2058 "s59E13H2" 0 "hypothetical protein"
"Chl_C14_00493" 2058 "s59E25H2" 0 "hypothetical protein"
"Chl_C14_00493" 2058 "s59E5H2" 0 "hypothetical protein"
"Chl_C14_00493" 2058 "s63E6H2" 0 "hypothetical protein"

"Chl_C14_00494" 2059 "s59E13H2" 0 "Dihydroorotate dehydrogenase B (NAD(+)), catalytic subunit"
"Chl_C14_00494" 2059 "s59E25H2" 0 "Dihydroorotate dehydrogenase B (NAD(+)), catalytic subunit"
"Chl_C14_00494" 2059 "s59E5H2" 0.001701881 "Dihydroorotate dehydrogenase B (NAD(+)), catalytic subunit"
"Chl_C14_00494" 2059 "s63E6H2" 0 "Dihydroorotate dehydrogenase B (NAD(+)), catalytic subunit"
"Chl_C14_00495" 2060 "s59E13H2" 0 "Xaa-Pro dipeptidase"
"Chl_C14_00495" 2060 "s59E25H2" 0 "Xaa-Pro dipeptidase"
"Chl_C14_00495" 2060 "s59E5H2" 0 "Xaa-Pro dipeptidase"
"Chl_C14_00495" 2060 "s63E6H2" 0 "Xaa-Pro dipeptidase"
"Chl_C14_00496" 2061 "s59E13H2" 0 "Asp/Glu/Hydantoin racemase"
"Chl_C14_00496" 2061 "s59E25H2" 0 "Asp/Glu/Hydantoin racemase"
"Chl_C14_00496" 2061 "s59E5H2" 0 "Asp/Glu/Hydantoin racemase"
"Chl_C14_00496" 2061 "s63E6H2" 0 "Asp/Glu/Hydantoin racemase"
"Chl_C14_00497" 2062 "s59E13H2" 0 "DctM-like transporters"
"Chl_C14_00497" 2062 "s59E25H2" 0 "DctM-like transporters"
"Chl_C14_00497" 2062 "s59E5H2" 0 "DctM-like transporters"
"Chl_C14_00497" 2062 "s63E6H2" 0 "DctM-like transporters"
"Chl_C14_00498" 2063 "s59E13H2" 0 "NMT1/THI5 like protein"
"Chl_C14_00498" 2063 "s59E25H2" 0 "NMT1/THI5 like protein"
"Chl_C14_00498" 2063 "s59E5H2" 0 "NMT1/THI5 like protein"
"Chl_C14_00498" 2063 "s63E6H2" 0 "NMT1/THI5 like protein"
"Chl_C14_00499" 2064 "s59E13H2" 0 "hypothetical protein"
"Chl_C14_00499" 2064 "s59E25H2" 0 "hypothetical protein"
"Chl_C14_00499" 2064 "s59E5H2" 0 "hypothetical protein"
"Chl_C14_00499" 2064 "s63E6H2" 0 "hypothetical protein"
"Chl_C14_00500" 2065 "s59E13H2" 0 "hypothetical protein"
"Chl_C14_00500" 2065 "s59E25H2" 0 "hypothetical protein"
"Chl_C14_00500" 2065 "s59E5H2" 0 "hypothetical protein"
"Chl_C14_00500" 2065 "s63E6H2" 0 "hypothetical protein"
"Chl_C14_00501" 2066 "s59E13H2" 0 "hypothetical protein"
"Chl_C14_00501" 2066 "s59E25H2" 0 "hypothetical protein"
"Chl_C14_00501" 2066 "s59E5H2" 0 "hypothetical protein"
"Chl_C14_00501" 2066 "s63E6H2" 0 "hypothetical protein"
"Chl_C14_00505" 2070 "s59E13H2" 0 "hypothetical protein"
"Chl_C14_00505" 2070 "s59E25H2" 0 "hypothetical protein"
"Chl_C14_00505" 2070 "s59E5H2" 0 "hypothetical protein"
"Chl_C14_00505" 2070 "s63E6H2" 0 "hypothetical protein"
"Chl_C14_00507" 2072 "s59E13H2" 0 "hypothetical protein"
"Chl_C14_00507" 2072 "s59E25H2" 0 "hypothetical protein"
"Chl_C14_00507" 2072 "s59E5H2" 0 "hypothetical protein"
"Chl_C14_00507" 2072 "s63E6H2" 0 "hypothetical protein"
"Chl_C14_00509" 2074 "s59E13H2" 0.00522025 "hypothetical protein"
"Chl_C14_00509" 2074 "s59E25H2" 0 "hypothetical protein"
"Chl_C14_00509" 2074 "s59E5H2" 0 "hypothetical protein"
"Chl_C14_00509" 2074 "s63E6H2" 0.1853873 "hypothetical protein"
"Chl_C14_00512" 2077 "s59E13H2" 0.1628755 "DNA polymerase III subunit beta"
"Chl_C14_00512" 2077 "s59E25H2" 0 "DNA polymerase III subunit beta"
"Chl_C14_00512" 2077 "s59E5H2" 0.1486137 "DNA polymerase III subunit beta"

"Chl_C14_00512" 2077 "s63E6H2" 0.004641776 "DNA polymerase III subunit beta"
"Chl_C14_00514" 2079 "s59E13H2" 0.1419012 "thiamine biosynthesis protein ThiF"
"Chl_C14_00514" 2079 "s59E25H2" 0 "thiamine biosynthesis protein ThiF"
"Chl_C14_00514" 2079 "s59E5H2" 0.3600951 "thiamine biosynthesis protein ThiF"
"Chl_C14_00514" 2079 "s63E6H2" 0.008113483 "thiamine biosynthesis protein ThiF"
"Chl_C14_00516" 2081 "s59E13H2" 0.1014508 "hypothetical protein"
"Chl_C14_00516" 2081 "s59E25H2" 0 "hypothetical protein"
"Chl_C14_00516" 2081 "s59E5H2" 0.401722 "hypothetical protein"
"Chl_C14_00516" 2081 "s63E6H2" 0 "hypothetical protein"
"Chl_C14_00519" 2084 "s59E13H2" 0 "hypothetical protein"
"Chl_C14_00519" 2084 "s59E25H2" 0 "hypothetical protein"
"Chl_C14_00519" 2084 "s59E5H2" 0.01568877 "hypothetical protein"
"Chl_C14_00519" 2084 "s63E6H2" 0 "hypothetical protein"
"Chl_C14_00524" 2089 "s59E13H2" 0 "Kynurenine formamidase"
"Chl_C14_00524" 2089 "s59E25H2" 0 "Kynurenine formamidase"
"Chl_C14_00524" 2089 "s59E5H2" 0 "Kynurenine formamidase"
"Chl_C14_00524" 2089 "s63E6H2" 0 "Kynurenine formamidase"
"Chl_C14_00525" 2090 "s59E13H2" 0 "Methylmalonate semialdehyde dehydrogenase [acylating]"
"Chl_C14_00525" 2090 "s59E25H2" 0 "Methylmalonate semialdehyde dehydrogenase [acylating]"
"Chl_C14_00525" 2090 "s59E5H2" 0 "Methylmalonate semialdehyde dehydrogenase [acylating]"
"Chl_C14_00525" 2090 "s63E6H2" 0 "Methylmalonate semialdehyde dehydrogenase [acylating]"
"Chl_C14_00526" 2091 "s59E13H2" 0 "hypothetical protein"
"Chl_C14_00526" 2091 "s59E25H2" 0 "hypothetical protein"
"Chl_C14_00526" 2091 "s59E5H2" 0 "hypothetical protein"
"Chl_C14_00526" 2091 "s63E6H2" 0 "hypothetical protein"
"Chl_C14_00527" 2092 "s59E13H2" 0 "putative membrane protein"
"Chl_C14_00527" 2092 "s59E25H2" 0 "putative membrane protein"
"Chl_C14_00527" 2092 "s59E5H2" 0 "putative membrane protein"
"Chl_C14_00527" 2092 "s63E6H2" 0 "putative membrane protein"
"Chl_C14_00528" 2093 "s59E13H2" 0 "Calcium-transporting ATPase 1"
"Chl_C14_00528" 2093 "s59E25H2" 0 "Calcium-transporting ATPase 1"
"Chl_C14_00528" 2093 "s59E5H2" 0 "Calcium-transporting ATPase 1"
"Chl_C14_00528" 2093 "s63E6H2" 0 "Calcium-transporting ATPase 1"
"Chl_C14_00529" 2094 "s59E13H2" 0 "Arsenical pump membrane protein"
"Chl_C14_00529" 2094 "s59E25H2" 0 "Arsenical pump membrane protein"
"Chl_C14_00529" 2094 "s59E5H2" 0 "Arsenical pump membrane protein"
"Chl_C14_00529" 2094 "s63E6H2" 0 "Arsenical pump membrane protein"
"Chl_C14_00530" 2095 "s59E13H2" 0 "Inosine-5'-monophosphate dehydrogenase"
"Chl_C14_00530" 2095 "s59E25H2" 0 "Inosine-5'-monophosphate dehydrogenase"
"Chl_C14_00530" 2095 "s59E5H2" 0 "Inosine-5'-monophosphate dehydrogenase"
"Chl_C14_00530" 2095 "s63E6H2" 0 "Inosine-5'-monophosphate dehydrogenase"
"Chl_C14_00532" 2097 "s59E13H2" 0 "Pyruvate/ketoisovalerate oxidoreductases common subunit gamma"
"Chl_C14_00532" 2097 "s59E25H2" 0 "Pyruvate/ketoisovalerate oxidoreductases common subunit gamma"
"Chl_C14_00532" 2097 "s59E5H2" 0 "Pyruvate/ketoisovalerate oxidoreductases common subunit gamma"
"Chl_C14_00532" 2097 "s63E6H2" 0 "Pyruvate/ketoisovalerate oxidoreductases common subunit gamma"
"Chl_C14_00533" 2098 "s59E13H2" 0 "Pyruvate synthase subunit PorD"
"Chl_C14_00533" 2098 "s59E25H2" 0 "Pyruvate synthase subunit PorD"

"Chl_C14_00533" 2098 "s59E5H2" 0 "Pyruvate synthase subunit PorD"
"Chl_C14_00533" 2098 "s63E6H2" 0 "Pyruvate synthase subunit PorD"
"Chl_C14_00534" 2099 "s59E13H2" 0 "Pyruvate synthase subunit PorA"
"Chl_C14_00534" 2099 "s59E25H2" 0 "Pyruvate synthase subunit PorA"
"Chl_C14_00534" 2099 "s59E5H2" 0 "Pyruvate synthase subunit PorA"
"Chl_C14_00534" 2099 "s63E6H2" 0 "Pyruvate synthase subunit PorA"
"Chl_C14_00535" 2100 "s59E13H2" 0 "Pyruvate synthase subunit PorB"
"Chl_C14_00535" 2100 "s59E25H2" 0 "Pyruvate synthase subunit PorB"
"Chl_C14_00535" 2100 "s59E5H2" 0 "Pyruvate synthase subunit PorB"
"Chl_C14_00535" 2100 "s63E6H2" 0 "Pyruvate synthase subunit PorB"
"Chl_C14_00536" 2101 "s59E13H2" 0 "NADP-reducing hydrogenase subunit HndA"
"Chl_C14_00536" 2101 "s59E25H2" 0 "NADP-reducing hydrogenase subunit HndA"
"Chl_C14_00536" 2101 "s59E5H2" 0 "NADP-reducing hydrogenase subunit HndA"
"Chl_C14_00536" 2101 "s63E6H2" 0 "NADP-reducing hydrogenase subunit HndA"
"Chl_C14_00537" 2102 "s59E13H2" 0 "NADP-reducing hydrogenase subunit HndC"
"Chl_C14_00537" 2102 "s59E25H2" 0 "NADP-reducing hydrogenase subunit HndC"
"Chl_C14_00537" 2102 "s59E5H2" 0 "NADP-reducing hydrogenase subunit HndC"
"Chl_C14_00537" 2102 "s63E6H2" 0 "NADP-reducing hydrogenase subunit HndC"
"Chl_C14_00538" 2103 "s59E13H2" 0 "NADP-reducing hydrogenase subunit HndC"
"Chl_C14_00538" 2103 "s59E25H2" 0 "NADP-reducing hydrogenase subunit HndC"
"Chl_C14_00538" 2103 "s59E5H2" 0 "NADP-reducing hydrogenase subunit HndC"
"Chl_C14_00538" 2103 "s63E6H2" 0.0007599112 "NADP-reducing hydrogenase subunit HndC"
"Chl_C14_00540" 2105 "s59E13H2" 0 "hypothetical protein"
"Chl_C14_00540" 2105 "s59E25H2" 0 "hypothetical protein"
"Chl_C14_00540" 2105 "s59E5H2" 0 "hypothetical protein"
"Chl_C14_00540" 2105 "s63E6H2" 0 "hypothetical protein"
"Chl_C14_00541" 2106 "s59E13H2" 0 "lipid-transfer protein"
"Chl_C14_00541" 2106 "s59E25H2" 0 "lipid-transfer protein"
"Chl_C14_00541" 2106 "s59E5H2" 0 "lipid-transfer protein"
"Chl_C14_00541" 2106 "s63E6H2" 0 "lipid-transfer protein"
"Chl_C14_00544" 2109 "s59E13H2" 0 "hypothetical protein"
"Chl_C14_00544" 2109 "s59E25H2" 0 "hypothetical protein"
"Chl_C14_00544" 2109 "s59E5H2" 0 "hypothetical protein"
"Chl_C14_00544" 2109 "s63E6H2" 0 "hypothetical protein"
"Chl_C14_00546" 2111 "s59E13H2" 0 "hypothetical protein"
"Chl_C14_00546" 2111 "s59E25H2" 0 "hypothetical protein"
"Chl_C14_00546" 2111 "s59E5H2" 0 "hypothetical protein"
"Chl_C14_00546" 2111 "s63E6H2" 0 "hypothetical protein"
"Chl_C14_00547" 2112 "s59E13H2" 0 "Flavoredoxin"
"Chl_C14_00547" 2112 "s59E25H2" 0 "Flavoredoxin"
"Chl_C14_00547" 2112 "s59E5H2" 0 "Flavoredoxin"
"Chl_C14_00547" 2112 "s63E6H2" 0 "Flavoredoxin"
"Chl_C14_00548" 2113 "s59E13H2" 0 "hypothetical protein"
"Chl_C14_00548" 2113 "s59E25H2" 0 "hypothetical protein"
"Chl_C14_00548" 2113 "s59E5H2" 0 "hypothetical protein"
"Chl_C14_00548" 2113 "s63E6H2" 0 "hypothetical protein"
"Chl_C14_00549" 2114 "s59E13H2" 0 "hypothetical protein"

"Chl_C14_00549" 2114 "s59E25H2" 0 "hypothetical protein"
"Chl_C14_00549" 2114 "s59E5H2" 0 "hypothetical protein"
"Chl_C14_00549" 2114 "s63E6H2" 0 "hypothetical protein"
"Chl_C14_00550" 2115 "s59E13H2" 0 "hypothetical protein"
"Chl_C14_00550" 2115 "s59E25H2" 0 "hypothetical protein"
"Chl_C14_00550" 2115 "s59E5H2" 0 "hypothetical protein"
"Chl_C14_00550" 2115 "s63E6H2" 0 "hypothetical protein"
"Chl_C14_00551" 2116 "s59E13H2" 0 "GTPase HflX"
"Chl_C14_00551" 2116 "s59E25H2" 0 "GTPase HflX"
"Chl_C14_00551" 2116 "s59E5H2" 0 "GTPase HflX"
"Chl_C14_00551" 2116 "s63E6H2" 0.003039645 "GTPase HflX"
"Chl_C14_00552" 2117 "s59E13H2" 0 "LL-diaminopimelate aminotransferase"
"Chl_C14_00552" 2117 "s59E25H2" 0 "LL-diaminopimelate aminotransferase"
"Chl_C14_00552" 2117 "s59E5H2" 0 "LL-diaminopimelate aminotransferase"
"Chl_C14_00552" 2117 "s63E6H2" 0 "LL-diaminopimelate aminotransferase"
"Chl_C14_00553" 2118 "s59E13H2" 0 "Diaminopimelate epimerase"
"Chl_C14_00553" 2118 "s59E25H2" 0 "Diaminopimelate epimerase"
"Chl_C14_00553" 2118 "s59E5H2" 0 "Diaminopimelate epimerase"
"Chl_C14_00553" 2118 "s63E6H2" 0 "Diaminopimelate epimerase"
"Chl_C14_00554" 2119 "s59E13H2" 0 "tRNA dimethylallyltransferase"
"Chl_C14_00554" 2119 "s59E25H2" 0 "tRNA dimethylallyltransferase"
"Chl_C14_00554" 2119 "s59E5H2" 0 "tRNA dimethylallyltransferase"
"Chl_C14_00554" 2119 "s63E6H2" 0 "tRNA dimethylallyltransferase"
"Chl_C14_00555" 2120 "s59E13H2" 0 "Triosephosphate isomerase"
"Chl_C14_00555" 2120 "s59E25H2" 0 "Triosephosphate isomerase"
"Chl_C14_00555" 2120 "s59E5H2" 0 "Triosephosphate isomerase"
"Chl_C14_00555" 2120 "s63E6H2" 0.009679183 "Triosephosphate isomerase"
"Chl_C14_00556" 2121 "s59E13H2" 0 "2,3-bisphosphoglycerate-independent phosphoglycerate mutase 1"
"Chl_C14_00556" 2121 "s59E25H2" 0 "2,3-bisphosphoglycerate-independent phosphoglycerate mutase 1"
"Chl_C14_00556" 2121 "s59E5H2" 0 "2,3-bisphosphoglycerate-independent phosphoglycerate mutase 1"
"Chl_C14_00556" 2121 "s63E6H2" 0.03589032 "2,3-bisphosphoglycerate-independent phosphoglycerate mutase 1"
"Chl_C14_00557" 2122 "s59E13H2" 0 "Phosphoglycerate kinase"
"Chl_C14_00557" 2122 "s59E25H2" 0 "Phosphoglycerate kinase"
"Chl_C14_00557" 2122 "s59E5H2" 0 "Phosphoglycerate kinase"
"Chl_C14_00557" 2122 "s63E6H2" 0 "Phosphoglycerate kinase"
"Chl_C14_00558" 2123 "s59E13H2" 0 "1-deoxy-D-xylulose-5-phosphate synthase"
"Chl_C14_00558" 2123 "s59E25H2" 0 "1-deoxy-D-xylulose-5-phosphate synthase"
"Chl_C14_00558" 2123 "s59E5H2" 0 "1-deoxy-D-xylulose-5-phosphate synthase"
"Chl_C14_00558" 2123 "s63E6H2" 0 "1-deoxy-D-xylulose-5-phosphate synthase"
"Chl_C14_00559" 2124 "s59E13H2" 0 "Putative small multi-drug export protein"
"Chl_C14_00559" 2124 "s59E25H2" 0 "Putative small multi-drug export protein"
"Chl_C14_00559" 2124 "s59E5H2" 0 "Putative small multi-drug export protein"
"Chl_C14_00559" 2124 "s63E6H2" 0 "Putative small multi-drug export protein"
"Chl_C14_00561" 2126 "s59E13H2" 0 "Tyrosine recombinase XerC"
"Chl_C14_00561" 2126 "s59E25H2" 0 "Tyrosine recombinase XerC"
"Chl_C14_00561" 2126 "s59E5H2" 0 "Tyrosine recombinase XerC"
"Chl_C14_00561" 2126 "s63E6H2" 0 "Tyrosine recombinase XerC"

"Chl_C14_00564" 2129 "s59E13H2" 0 "Tyrosine recombinase XerD"
"Chl_C14_00564" 2129 "s59E25H2" 0 "Tyrosine recombinase XerD"
"Chl_C14_00564" 2129 "s59E5H2" 0 "Tyrosine recombinase XerD"
"Chl_C14_00564" 2129 "s63E6H2" 0 "Tyrosine recombinase XerD"
"Chl_C14_00565" 2130 "s59E13H2" 0 "site-specific tyrosine recombinase XerC"
"Chl_C14_00565" 2130 "s59E25H2" 0 "site-specific tyrosine recombinase XerC"
"Chl_C14_00565" 2130 "s59E5H2" 0 "site-specific tyrosine recombinase XerC"
"Chl_C14_00565" 2130 "s63E6H2" 0 "site-specific tyrosine recombinase XerC"
"Chl_C14_00566" 2131 "s59E13H2" 0 "Integrase core domain protein"
"Chl_C14_00566" 2131 "s59E25H2" 0 "Integrase core domain protein"
"Chl_C14_00566" 2131 "s59E5H2" 0 "Integrase core domain protein"
"Chl_C14_00566" 2131 "s63E6H2" 0 "Integrase core domain protein"
"Chl_C14_00567" 2132 "s59E13H2" 0 "Tripartite tricarboxylate transporter family receptor"
"Chl_C14_00567" 2132 "s59E25H2" 0 "Tripartite tricarboxylate transporter family receptor"
"Chl_C14_00567" 2132 "s59E5H2" 0 "Tripartite tricarboxylate transporter family receptor"
"Chl_C14_00567" 2132 "s63E6H2" 0 "Tripartite tricarboxylate transporter family receptor"
"Chl_C14_00568" 2133 "s59E13H2" 0 "Tripartite tricarboxylate transporter TctB family protein"
"Chl_C14_00568" 2133 "s59E25H2" 0 "Tripartite tricarboxylate transporter TctB family protein"
"Chl_C14_00568" 2133 "s59E5H2" 0 "Tripartite tricarboxylate transporter TctB family protein"
"Chl_C14_00568" 2133 "s63E6H2" 0 "Tripartite tricarboxylate transporter TctB family protein"
"Chl_C14_00569" 2134 "s59E13H2" 0 "Tripartite tricarboxylate transporter TctA family protein"
"Chl_C14_00569" 2134 "s59E25H2" 0 "Tripartite tricarboxylate transporter TctA family protein"
"Chl_C14_00569" 2134 "s59E5H2" 0 "Tripartite tricarboxylate transporter TctA family protein"
"Chl_C14_00569" 2134 "s63E6H2" 0 "Tripartite tricarboxylate transporter TctA family protein"
"Chl_C14_00570" 2135 "s59E13H2" 0 "Thermophilic metalloprotease (M29)"
"Chl_C14_00570" 2135 "s59E25H2" 0 "Thermophilic metalloprotease (M29)"
"Chl_C14_00570" 2135 "s59E5H2" 0 "Thermophilic metalloprotease (M29)"
"Chl_C14_00570" 2135 "s63E6H2" 0 "Thermophilic metalloprotease (M29)"
"Chl_C14_00572" 2137 "s59E13H2" 0 "putative adenylyltransferase/sulfurtransferase MoeZ"
"Chl_C14_00572" 2137 "s59E25H2" 0 "putative adenylyltransferase/sulfurtransferase MoeZ"
"Chl_C14_00572" 2137 "s59E5H2" 0 "putative adenylyltransferase/sulfurtransferase MoeZ"
"Chl_C14_00572" 2137 "s63E6H2" 0 "putative adenylyltransferase/sulfurtransferase MoeZ"
"Chl_C14_00573" 2138 "s59E13H2" 0.001232931 "Acetyltransferase Pat"
"Chl_C14_00573" 2138 "s59E25H2" 0 "Acetyltransferase Pat"
"Chl_C14_00573" 2138 "s59E5H2" 0.07401008 "Acetyltransferase Pat"
"Chl_C14_00573" 2138 "s63E6H2" 0 "Acetyltransferase Pat"
"Chl_C14_00576" 2141 "s59E13H2" 0 "hypothetical protein"
"Chl_C14_00576" 2141 "s59E25H2" 0 "hypothetical protein"
"Chl_C14_00576" 2141 "s59E5H2" 0 "hypothetical protein"
"Chl_C14_00576" 2141 "s63E6H2" 0 "hypothetical protein"
"Chl_C14_00577" 2142 "s59E13H2" 0 "Pyridoxal biosynthesis lyase PdxS"
"Chl_C14_00577" 2142 "s59E25H2" 0 "Pyridoxal biosynthesis lyase PdxS"
"Chl_C14_00577" 2142 "s59E5H2" 0 "Pyridoxal biosynthesis lyase PdxS"
"Chl_C14_00577" 2142 "s63E6H2" 0 "Pyridoxal biosynthesis lyase PdxS"
"Chl_C14_00578" 2143 "s59E13H2" 0.006280416 "hypothetical protein"
"Chl_C14_00578" 2143 "s59E25H2" 0 "hypothetical protein"
"Chl_C14_00578" 2143 "s59E5H2" 0 "hypothetical protein"

"Chl_C14_00578" 2143 "s63E6H2" 0 "hypothetical protein"
"Chl_C14_00579" 2144 "s59E13H2" 0.03509776 "Trehalase"
"Chl_C14_00579" 2144 "s59E25H2" 0 "Trehalase"
"Chl_C14_00579" 2144 "s59E5H2" 1.841543 "Trehalase"
"Chl_C14_00579" 2144 "s63E6H2" 0.04249123 "Trehalase"
"Chl_C14_00580" 2145 "s59E13H2" 0.001250387 "Lipopolysaccharide core biosynthesis protein RfaG"
"Chl_C14_00580" 2145 "s59E25H2" 0 "Lipopolysaccharide core biosynthesis protein RfaG"
"Chl_C14_00580" 2145 "s59E5H2" 0.9773763 "Lipopolysaccharide core biosynthesis protein RfaG"
"Chl_C14_00580" 2145 "s63E6H2" 0.001092856 "Lipopolysaccharide core biosynthesis protein RfaG"
"Chl_C14_00584" 2149 "s59E13H2" 0 "hypothetical protein"
"Chl_C14_00584" 2149 "s59E25H2" 0 "hypothetical protein"
"Chl_C14_00584" 2149 "s59E5H2" 0.005010899 "hypothetical protein"
"Chl_C14_00584" 2149 "s63E6H2" 0 "hypothetical protein"
"Chl_C14_00585" 2150 "s59E13H2" 0 "Acetyltransferase (GNAT) family protein"
"Chl_C14_00585" 2150 "s59E25H2" 0 "Acetyltransferase (GNAT) family protein"
"Chl_C14_00585" 2150 "s59E5H2" 0.00356862 "Acetyltransferase (GNAT) family protein"
"Chl_C14_00585" 2150 "s63E6H2" 0 "Acetyltransferase (GNAT) family protein"
"Chl_C14_00586" 2151 "s59E13H2" 0 "Epoxyqueuosine reductase"
"Chl_C14_00586" 2151 "s59E25H2" 0 "Epoxyqueuosine reductase"
"Chl_C14_00586" 2151 "s59E5H2" 0 "Epoxyqueuosine reductase"
"Chl_C14_00586" 2151 "s63E6H2" 0 "Epoxyqueuosine reductase"
"Chl_C14_00587" 2152 "s59E13H2" 0 "hypothetical protein"
"Chl_C14_00587" 2152 "s59E25H2" 0 "hypothetical protein"
"Chl_C14_00587" 2152 "s59E5H2" 0 "hypothetical protein"
"Chl_C14_00587" 2152 "s63E6H2" 0 "hypothetical protein"
"Chl_C14_00588" 2153 "s59E13H2" 0 "hypothetical protein"
"Chl_C14_00588" 2153 "s59E25H2" 0 "hypothetical protein"
"Chl_C14_00588" 2153 "s59E5H2" 0 "hypothetical protein"
"Chl_C14_00588" 2153 "s63E6H2" 0 "hypothetical protein"
"Chl_C14_00589" 2154 "s59E13H2" 0 "Threonylcarbamoyladenosine tRNA methylthiotransferase MtaB"
"Chl_C14_00589" 2154 "s59E25H2" 0 "Threonylcarbamoyladenosine tRNA methylthiotransferase MtaB"
"Chl_C14_00589" 2154 "s59E5H2" 0 "Threonylcarbamoyladenosine tRNA methylthiotransferase MtaB"
"Chl_C14_00589" 2154 "s63E6H2" 0 "Threonylcarbamoyladenosine tRNA methylthiotransferase MtaB"
"Chl_C14_00590" 2155 "s59E13H2" 0 "3-oxoacyl-[acyl-carrier-protein] synthase 2"
"Chl_C14_00590" 2155 "s59E25H2" 0 "3-oxoacyl-[acyl-carrier-protein] synthase 2"
"Chl_C14_00590" 2155 "s59E5H2" 0 "3-oxoacyl-[acyl-carrier-protein] synthase 2"
"Chl_C14_00590" 2155 "s63E6H2" 0.0008047544 "3-oxoacyl-[acyl-carrier-protein] synthase 2"
"Chl_C14_00591" 2156 "s59E13H2" 0 "Single-stranded-DNA-specific exonuclease RecJ"
"Chl_C14_00591" 2156 "s59E25H2" 0 "Single-stranded-DNA-specific exonuclease RecJ"
"Chl_C14_00591" 2156 "s59E5H2" 0 "Single-stranded-DNA-specific exonuclease RecJ"
"Chl_C14_00591" 2156 "s63E6H2" 0.005456886 "Single-stranded-DNA-specific exonuclease RecJ"
"Chl_C14_00592" 2157 "s59E13H2" 0 "Oxygen sensor histidine kinase NreB"
"Chl_C14_00592" 2157 "s59E25H2" 0 "Oxygen sensor histidine kinase NreB"
"Chl_C14_00592" 2157 "s59E5H2" 0 "Oxygen sensor histidine kinase NreB"
"Chl_C14_00592" 2157 "s63E6H2" 0 "Oxygen sensor histidine kinase NreB"
"Chl_C14_00593" 2158 "s59E13H2" 0 "Transcriptional regulatory protein LiaR"
"Chl_C14_00593" 2158 "s59E25H2" 0 "Transcriptional regulatory protein LiaR"

"Chl_C14_00593" 2158 "s59E5H2" 0 "Transcriptional regulatory protein LiaR"
"Chl_C14_00593" 2158 "s63E6H2" 0 "Transcriptional regulatory protein LiaR"
"Chl_C14_00595" 2160 "s59E13H2" 0 "putative formate transporter 1"
"Chl_C14_00595" 2160 "s59E25H2" 0 "putative formate transporter 1"
"Chl_C14_00595" 2160 "s59E5H2" 0 "putative formate transporter 1"
"Chl_C14_00595" 2160 "s63E6H2" 0 "putative formate transporter 1"
"Chl_C14_00596" 2161 "s59E13H2" 0 "Prolipoprotein diacylglyceryl transferase"
"Chl_C14_00596" 2161 "s59E25H2" 0 "Prolipoprotein diacylglyceryl transferase"
"Chl_C14_00596" 2161 "s59E5H2" 0 "Prolipoprotein diacylglyceryl transferase"
"Chl_C14_00596" 2161 "s63E6H2" 0 "Prolipoprotein diacylglyceryl transferase"
"Chl_C14_00597" 2162 "s59E13H2" 0 "Signal recognition particle receptor FtsY"
"Chl_C14_00597" 2162 "s59E25H2" 0 "Signal recognition particle receptor FtsY"
"Chl_C14_00597" 2162 "s59E5H2" 0 "Signal recognition particle receptor FtsY"
"Chl_C14_00597" 2162 "s63E6H2" 0 "Signal recognition particle receptor FtsY"
"Chl_C14_00598" 2163 "s59E13H2" 0 "hypothetical protein"
"Chl_C14_00598" 2163 "s59E25H2" 0 "hypothetical protein"
"Chl_C14_00598" 2163 "s59E5H2" 0 "hypothetical protein"
"Chl_C14_00598" 2163 "s63E6H2" 0 "hypothetical protein"
"Chl_C14_00599" 2164 "s59E13H2" 0 "Putative ski2-type helicase"
"Chl_C14_00599" 2164 "s59E25H2" 0 "Putative ski2-type helicase"
"Chl_C14_00599" 2164 "s59E5H2" 0 "Putative ski2-type helicase"
"Chl_C14_00599" 2164 "s63E6H2" 0 "Putative ski2-type helicase"
"Chl_C14_00600" 2165 "s59E13H2" 0 "hypothetical protein"
"Chl_C14_00600" 2165 "s59E25H2" 0 "hypothetical protein"
"Chl_C14_00600" 2165 "s59E5H2" 0 "hypothetical protein"
"Chl_C14_00600" 2165 "s63E6H2" 0 "hypothetical protein"
"Chl_C14_00601" 2166 "s59E13H2" 0 "hypothetical protein"
"Chl_C14_00601" 2166 "s59E25H2" 0 "hypothetical protein"
"Chl_C14_00601" 2166 "s59E5H2" 0 "hypothetical protein"
"Chl_C14_00601" 2166 "s63E6H2" 0 "hypothetical protein"
"Chl_C14_00602" 2167 "s59E13H2" 0 "30S ribosomal protein S15"
"Chl_C14_00602" 2167 "s59E25H2" 0 "30S ribosomal protein S15"
"Chl_C14_00602" 2167 "s59E5H2" 0 "30S ribosomal protein S15"
"Chl_C14_00602" 2167 "s63E6H2" 0 "30S ribosomal protein S15"
"Chl_C14_00603" 2168 "s59E13H2" 0 "Polyribonucleotide nucleotidyltransferase"
"Chl_C14_00603" 2168 "s59E25H2" 0 "Polyribonucleotide nucleotidyltransferase"
"Chl_C14_00603" 2168 "s59E5H2" 0 "Polyribonucleotide nucleotidyltransferase"
"Chl_C14_00603" 2168 "s63E6H2" 0 "Polyribonucleotide nucleotidyltransferase"
"Chl_C14_00604" 2169 "s59E13H2" 0 "4-hydroxy-tetrahydrodipicolinate reductase"
"Chl_C14_00604" 2169 "s59E25H2" 0 "4-hydroxy-tetrahydrodipicolinate reductase"
"Chl_C14_00604" 2169 "s59E5H2" 0 "4-hydroxy-tetrahydrodipicolinate reductase"
"Chl_C14_00604" 2169 "s63E6H2" 0 "4-hydroxy-tetrahydrodipicolinate reductase"
"Chl_C14_00605" 2170 "s59E13H2" 0 "Aspartate-semialdehyde dehydrogenase"
"Chl_C14_00605" 2170 "s59E25H2" 0 "Aspartate-semialdehyde dehydrogenase"
"Chl_C14_00605" 2170 "s59E5H2" 0.001327276 "Aspartate-semialdehyde dehydrogenase"
"Chl_C14_00605" 2170 "s63E6H2" 0.001481308 "Aspartate-semialdehyde dehydrogenase"
"Chl_C14_00606" 2171 "s59E13H2" 0 "4-hydroxy-tetrahydrodipicolinate synthase"

"Chl_C14_00606" 2171 "s59E25H2" 0 "4-hydroxy-tetrahydrodipicolinate synthase"
"Chl_C14_00606" 2171 "s59E5H2" 0 "4-hydroxy-tetrahydrodipicolinate synthase"
"Chl_C14_00606" 2171 "s63E6H2" 0 "4-hydroxy-tetrahydrodipicolinate synthase"
"Chl_C14_00607" 2172 "s59E13H2" 0.00371615 "Adenylosuccinate synthetase"
"Chl_C14_00607" 2172 "s59E25H2" 0 "Adenylosuccinate synthetase"
"Chl_C14_00607" 2172 "s59E5H2" 0 "Adenylosuccinate synthetase"
"Chl_C14_00607" 2172 "s63E6H2" 0 "Adenylosuccinate synthetase"
"Chl_C14_00608" 2173 "s59E13H2" 0 "Archaetidylinositol phosphate synthase"
"Chl_C14_00608" 2173 "s59E25H2" 0 "Archaetidylinositol phosphate synthase"
"Chl_C14_00608" 2173 "s59E5H2" 0 "Archaetidylinositol phosphate synthase"
"Chl_C14_00608" 2173 "s63E6H2" 0 "Archaetidylinositol phosphate synthase"
"Chl_C14_00609" 2174 "s59E13H2" 0 "GDP-mannose-dependent alpha-(1-2)-phosphatidylinositol mannosyltransferase"
"Chl_C14_00609" 2174 "s59E25H2" 0 "GDP-mannose-dependent alpha-(1-2)-phosphatidylinositol mannosyltransferase"
"Chl_C14_00609" 2174 "s59E5H2" 0 "GDP-mannose-dependent alpha-(1-2)-phosphatidylinositol mannosyltransferase"
"Chl_C14_00609" 2174 "s63E6H2" 0 "GDP-mannose-dependent alpha-(1-2)-phosphatidylinositol mannosyltransferase"
"Chl_C14_00610" 2175 "s59E13H2" 0 "Inositol-3-phosphate synthase"
"Chl_C14_00610" 2175 "s59E25H2" 0 "Inositol-3-phosphate synthase"
"Chl_C14_00610" 2175 "s59E5H2" 0.007347552 "Inositol-3-phosphate synthase"
"Chl_C14_00610" 2175 "s63E6H2" 0.07365272 "Inositol-3-phosphate synthase"
"Chl_C14_00611" 2176 "s59E13H2" 0 "tRNA pseudouridine synthase B"
"Chl_C14_00611" 2176 "s59E25H2" 0 "tRNA pseudouridine synthase B"
"Chl_C14_00611" 2176 "s59E5H2" 0 "tRNA pseudouridine synthase B"
"Chl_C14_00611" 2176 "s63E6H2" 0 "tRNA pseudouridine synthase B"
"Chl_C14_00612" 2177 "s59E13H2" 0 "Ribosome-binding factor A"
"Chl_C14_00612" 2177 "s59E25H2" 0 "Ribosome-binding factor A"
"Chl_C14_00612" 2177 "s59E5H2" 0 "Ribosome-binding factor A"
"Chl_C14_00612" 2177 "s63E6H2" 0 "Ribosome-binding factor A"
"Chl_C14_00613" 2178 "s59E13H2" 0.003632241 "Translation initiation factor IF-2"
"Chl_C14_00613" 2178 "s59E25H2" 0 "Translation initiation factor IF-2"
"Chl_C14_00613" 2178 "s59E5H2" 0 "Translation initiation factor IF-2"
"Chl_C14_00613" 2178 "s63E6H2" 0 "Translation initiation factor IF-2"
"Chl_C14_00614" 2179 "s59E13H2" 0 "hypothetical protein"
"Chl_C14_00614" 2179 "s59E25H2" 0 "hypothetical protein"
"Chl_C14_00614" 2179 "s59E5H2" 0 "hypothetical protein"
"Chl_C14_00614" 2179 "s63E6H2" 0 "hypothetical protein"
"Chl_C14_00615" 2180 "s59E13H2" 0.001440449 "hypothetical protein"
"Chl_C14_00615" 2180 "s59E25H2" 0 "hypothetical protein"
"Chl_C14_00615" 2180 "s59E5H2" 0 "hypothetical protein"
"Chl_C14_00615" 2180 "s63E6H2" 0 "hypothetical protein"
"Chl_C14_00616" 2181 "s59E13H2" 0 "5'/3'-nucleotidase SurE"
"Chl_C14_00616" 2181 "s59E25H2" 0 "5'/3'-nucleotidase SurE"
"Chl_C14_00616" 2181 "s59E5H2" 0 "5'/3'-nucleotidase SurE"
"Chl_C14_00616" 2181 "s63E6H2" 0 "5'/3'-nucleotidase SurE"
"Chl_C14_00617" 2182 "s59E13H2" 0 "CoB--CoM heterodisulfide reductase iron-sulfur subunit C"
"Chl_C14_00617" 2182 "s59E25H2" 0 "CoB--CoM heterodisulfide reductase iron-sulfur subunit C"
"Chl_C14_00617" 2182 "s59E5H2" 0 "CoB--CoM heterodisulfide reductase iron-sulfur subunit C"
"Chl_C14_00617" 2182 "s63E6H2" 0 "CoB--CoM heterodisulfide reductase iron-sulfur subunit C"

"Chl_C14_00618" 2183 "s59E13H2" 0 "CoB--CoM heterodisulfide reductase subunit B"
"Chl_C14_00618" 2183 "s59E25H2" 0 "CoB--CoM heterodisulfide reductase subunit B"
"Chl_C14_00618" 2183 "s59E5H2" 0 "CoB--CoM heterodisulfide reductase subunit B"
"Chl_C14_00618" 2183 "s63E6H2" 0 "CoB--CoM heterodisulfide reductase subunit B"
"Chl_C14_00619" 2184 "s59E13H2" 0 "CoB--CoM heterodisulfide reductase iron-sulfur subunit A"
"Chl_C14_00619" 2184 "s59E25H2" 0 "CoB--CoM heterodisulfide reductase iron-sulfur subunit A"
"Chl_C14_00619" 2184 "s59E5H2" 0 "CoB--CoM heterodisulfide reductase iron-sulfur subunit A"
"Chl_C14_00619" 2184 "s63E6H2" 0 "CoB--CoM heterodisulfide reductase iron-sulfur subunit A"
"Chl_C14_00620" 2185 "s59E13H2" 0 "NADP-reducing hydrogenase subunit HndC"
"Chl_C14_00620" 2185 "s59E25H2" 0 "NADP-reducing hydrogenase subunit HndC"
"Chl_C14_00620" 2185 "s59E5H2" 0 "NADP-reducing hydrogenase subunit HndC"
"Chl_C14_00620" 2185 "s63E6H2" 0 "NADP-reducing hydrogenase subunit HndC"
"Chl_C14_00621" 2186 "s59E13H2" 0 "NADP-reducing hydrogenase subunit HndA"
"Chl_C14_00621" 2186 "s59E25H2" 0 "NADP-reducing hydrogenase subunit HndA"
"Chl_C14_00621" 2186 "s59E5H2" 0 "NADP-reducing hydrogenase subunit HndA"
"Chl_C14_00621" 2186 "s63E6H2" 0 "NADP-reducing hydrogenase subunit HndA"
"Chl_C14_00622" 2187 "s59E13H2" 0 "NADP-reducing hydrogenase subunit HndC"
"Chl_C14_00622" 2187 "s59E25H2" 0 "NADP-reducing hydrogenase subunit HndC"
"Chl_C14_00622" 2187 "s59E5H2" 0 "NADP-reducing hydrogenase subunit HndC"
"Chl_C14_00622" 2187 "s63E6H2" 0 "NADP-reducing hydrogenase subunit HndC"
"Chl_C14_00623" 2188 "s59E13H2" 0 "hypothetical protein"
"Chl_C14_00623" 2188 "s59E25H2" 0 "hypothetical protein"
"Chl_C14_00623" 2188 "s59E5H2" 0 "hypothetical protein"
"Chl_C14_00623" 2188 "s63E6H2" 0 "hypothetical protein"
"Chl_C14_00624" 2189 "s59E13H2" 0 "Alkaline phosphatase synthesis sensor protein PhoR"
"Chl_C14_00624" 2189 "s59E25H2" 0 "Alkaline phosphatase synthesis sensor protein PhoR"
"Chl_C14_00624" 2189 "s59E5H2" 0 "Alkaline phosphatase synthesis sensor protein PhoR"
"Chl_C14_00624" 2189 "s63E6H2" 0 "Alkaline phosphatase synthesis sensor protein PhoR"
"Chl_C14_00625" 2190 "s59E13H2" 0 "Bifunctional homocysteine S-methyltransferase/5,10-methylenetetrahydrofolate reductase"
"Chl_C14_00625" 2190 "s59E25H2" 0 "Bifunctional homocysteine S-methyltransferase/5,10-methylenetetrahydrofolate reductase"
"Chl_C14_00625" 2190 "s59E5H2" 0 "Bifunctional homocysteine S-methyltransferase/5,10-methylenetetrahydrofolate reductase"
"Chl_C14_00625" 2190 "s63E6H2" 0 "Bifunctional homocysteine S-methyltransferase/5,10-methylenetetrahydrofolate reductase"
"Chl_C14_00626" 2191 "s59E13H2" 0 "Alkaline phosphatase synthesis transcriptional regulatory protein PhoP"
"Chl_C14_00626" 2191 "s59E25H2" 0 "Alkaline phosphatase synthesis transcriptional regulatory protein PhoP"
"Chl_C14_00626" 2191 "s59E5H2" 0 "Alkaline phosphatase synthesis transcriptional regulatory protein PhoP"
"Chl_C14_00626" 2191 "s63E6H2" 0 "Alkaline phosphatase synthesis transcriptional regulatory protein PhoP"
"Chl_C14_00627" 2192 "s59E13H2" 0 "Alkaline phosphatase synthesis transcriptional regulatory protein PhoP"
"Chl_C14_00627" 2192 "s59E25H2" 0 "Alkaline phosphatase synthesis transcriptional regulatory protein PhoP"
"Chl_C14_00627" 2192 "s59E5H2" 0 "Alkaline phosphatase synthesis transcriptional regulatory protein PhoP"
"Chl_C14_00627" 2192 "s63E6H2" 0 "Alkaline phosphatase synthesis transcriptional regulatory protein PhoP"
"Chl_C14_00628" 2193 "s59E13H2" 0 "Bifunctional homocysteine S-methyltransferase/5,10-methylenetetrahydrofolate reductase"
"Chl_C14_00628" 2193 "s59E25H2" 0 "Bifunctional homocysteine S-methyltransferase/5,10-methylenetetrahydrofolate reductase"
"Chl_C14_00628" 2193 "s59E5H2" 0 "Bifunctional homocysteine S-methyltransferase/5,10-methylenetetrahydrofolate reductase"
"Chl_C14_00628" 2193 "s63E6H2" 0 "Bifunctional homocysteine S-methyltransferase/5,10-methylenetetrahydrofolate reductase"
"Chl_C14_00629" 2194 "s59E13H2" 0 "Sulfhydrogenase 1 subunit gamma"
"Chl_C14_00629" 2194 "s59E25H2" 0 "Sulfhydrogenase 1 subunit gamma"
"Chl_C14_00629" 2194 "s59E5H2" 0 "Sulfhydrogenase 1 subunit gamma"

"Chl_C14_00629" 2194 "s63E6H2" 0 "Sulfhydrogenase 1 subunit gamma"
"Chl_C14_00630" 2195 "s59E13H2" 0 "Sulfhydrogenase 2 subunit beta"
"Chl_C14_00630" 2195 "s59E25H2" 0 "Sulfhydrogenase 2 subunit beta"
"Chl_C14_00630" 2195 "s59E5H2" 0 "Sulfhydrogenase 2 subunit beta"
"Chl_C14_00630" 2195 "s63E6H2" 0 "Sulfhydrogenase 2 subunit beta"
"Chl_C14_00631" 2196 "s59E13H2" 0 "Formate dehydrogenase subunit beta"
"Chl_C14_00631" 2196 "s59E25H2" 0 "Formate dehydrogenase subunit beta"
"Chl_C14_00631" 2196 "s59E5H2" 0 "Formate dehydrogenase subunit beta"
"Chl_C14_00631" 2196 "s63E6H2" 0 "Formate dehydrogenase subunit beta"
"Chl_C14_00632" 2197 "s59E13H2" 0 "F420-non-reducing hydrogenase vhc iron-sulfur subunit D"
"Chl_C14_00632" 2197 "s59E25H2" 0 "F420-non-reducing hydrogenase vhc iron-sulfur subunit D"
"Chl_C14_00632" 2197 "s59E5H2" 0 "F420-non-reducing hydrogenase vhc iron-sulfur subunit D"
"Chl_C14_00632" 2197 "s63E6H2" 0 "F420-non-reducing hydrogenase vhc iron-sulfur subunit D"
"Chl_C14_00633" 2198 "s59E13H2" 0 "S-adenosylmethionine:tRNA ribosyltransferase-isomerase"
"Chl_C14_00633" 2198 "s59E25H2" 0 "S-adenosylmethionine:tRNA ribosyltransferase-isomerase"
"Chl_C14_00633" 2198 "s59E5H2" 0 "S-adenosylmethionine:tRNA ribosyltransferase-isomerase"
"Chl_C14_00633" 2198 "s63E6H2" 0 "S-adenosylmethionine:tRNA ribosyltransferase-isomerase"
"Chl_C14_00634" 2199 "s59E13H2" 0 "Recombinase"
"Chl_C14_00634" 2199 "s59E25H2" 0 "Recombinase"
"Chl_C14_00634" 2199 "s59E5H2" 0 "Recombinase"
"Chl_C14_00634" 2199 "s63E6H2" 0 "Recombinase"
"Chl_C14_00636" 2201 "s59E13H2" 0 "hypothetical protein"
"Chl_C14_00636" 2201 "s59E25H2" 0 "hypothetical protein"
"Chl_C14_00636" 2201 "s59E5H2" 0 "hypothetical protein"
"Chl_C14_00636" 2201 "s63E6H2" 0 "hypothetical protein"
"Chl_C14_00637" 2202 "s59E13H2" 0 "hypothetical protein"
"Chl_C14_00637" 2202 "s59E25H2" 0 "hypothetical protein"
"Chl_C14_00637" 2202 "s59E5H2" 0 "hypothetical protein"
"Chl_C14_00637" 2202 "s63E6H2" 0 "hypothetical protein"
"Chl_C14_00639" 2204 "s59E13H2" 0 "AIPR protein"
"Chl_C14_00639" 2204 "s59E25H2" 0 "AIPR protein"
"Chl_C14_00639" 2204 "s59E5H2" 0 "AIPR protein"
"Chl_C14_00639" 2204 "s63E6H2" 0 "AIPR protein"
"Chl_C14_00640" 2205 "s59E13H2" 0 "Transposase IS116/IS110/IS902 family protein"
"Chl_C14_00640" 2205 "s59E25H2" 0 "Transposase IS116/IS110/IS902 family protein"
"Chl_C14_00640" 2205 "s59E5H2" 0.02473819 "Transposase IS116/IS110/IS902 family protein"
"Chl_C14_00640" 2205 "s63E6H2" 0.4434823 "Transposase IS116/IS110/IS902 family protein"
"Chl_C14_00641" 2206 "s59E13H2" 0 "Oxalate:formate antiporter"
"Chl_C14_00641" 2206 "s59E25H2" 0 "Oxalate:formate antiporter"
"Chl_C14_00641" 2206 "s59E5H2" 0 "Oxalate:formate antiporter"
"Chl_C14_00641" 2206 "s63E6H2" 0 "Oxalate:formate antiporter"
"Chl_C14_00642" 2207 "s59E13H2" 0 "Pyruvate kinase"
"Chl_C14_00642" 2207 "s59E25H2" 0 "Pyruvate kinase"
"Chl_C14_00642" 2207 "s59E5H2" 0 "Pyruvate kinase"
"Chl_C14_00642" 2207 "s63E6H2" 0 "Pyruvate kinase"
"Chl_C14_00643" 2208 "s59E13H2" 0 "3-keto-5-aminohexanoate cleavage enzyme"
"Chl_C14_00643" 2208 "s59E25H2" 0 "3-keto-5-aminohexanoate cleavage enzyme"

"Chl_C14_00643" 2208 "s59E5H2" 0 "3-keto-5-aminohexanoate cleavage enzyme"
"Chl_C14_00643" 2208 "s63E6H2" 0 "3-keto-5-aminohexanoate cleavage enzyme"
"Chl_C14_00644" 2209 "s59E13H2" 0 "3-keto-5-aminohexanoate cleavage enzyme"
"Chl_C14_00644" 2209 "s59E25H2" 0 "3-keto-5-aminohexanoate cleavage enzyme"
"Chl_C14_00644" 2209 "s59E5H2" 0 "3-keto-5-aminohexanoate cleavage enzyme"
"Chl_C14_00644" 2209 "s63E6H2" 0 "3-keto-5-aminohexanoate cleavage enzyme"
"Chl_C14_00646" 2211 "s59E13H2" 0 "hypothetical protein"
"Chl_C14_00646" 2211 "s59E25H2" 0 "hypothetical protein"
"Chl_C14_00646" 2211 "s59E5H2" 0 "hypothetical protein"
"Chl_C14_00646" 2211 "s63E6H2" 0 "hypothetical protein"
"Chl_C14_00647" 2212 "s59E13H2" 0 "hypothetical protein"
"Chl_C14_00647" 2212 "s59E25H2" 0 "hypothetical protein"
"Chl_C14_00647" 2212 "s59E5H2" 0 "hypothetical protein"
"Chl_C14_00647" 2212 "s63E6H2" 0 "hypothetical protein"
"Chl_C14_00648" 2213 "s59E13H2" 0 "metal-dependent hydrolase"
"Chl_C14_00648" 2213 "s59E25H2" 0 "metal-dependent hydrolase"
"Chl_C14_00648" 2213 "s59E5H2" 0.005531966 "metal-dependent hydrolase"
"Chl_C14_00648" 2213 "s63E6H2" 0.002400983 "metal-dependent hydrolase"
"Chl_C14_00649" 2214 "s59E13H2" 0 "Heat-inducible transcription repressor HrcA"
"Chl_C14_00649" 2214 "s59E25H2" 0 "Heat-inducible transcription repressor HrcA"
"Chl_C14_00649" 2214 "s59E5H2" 0 "Heat-inducible transcription repressor HrcA"
"Chl_C14_00649" 2214 "s63E6H2" 0 "Heat-inducible transcription repressor HrcA"
"Chl_C14_00650" 2215 "s59E13H2" 0 "heat shock protein GrpE"
"Chl_C14_00650" 2215 "s59E25H2" 0 "heat shock protein GrpE"
"Chl_C14_00650" 2215 "s59E5H2" 0 "heat shock protein GrpE"
"Chl_C14_00650" 2215 "s63E6H2" 0 "heat shock protein GrpE"
"Chl_C14_00651" 2216 "s59E13H2" 0 "Chaperone protein DnaK"
"Chl_C14_00651" 2216 "s59E25H2" 0 "Chaperone protein DnaK"
"Chl_C14_00651" 2216 "s59E5H2" 0.00133177 "Chaperone protein DnaK"
"Chl_C14_00651" 2216 "s63E6H2" 0 "Chaperone protein DnaK"
"Chl_C14_00652" 2217 "s59E13H2" 0 "Chaperone protein DnaJ"
"Chl_C14_00652" 2217 "s59E25H2" 0 "Chaperone protein DnaJ"
"Chl_C14_00652" 2217 "s59E5H2" 0 "Chaperone protein DnaJ"
"Chl_C14_00652" 2217 "s63E6H2" 0 "Chaperone protein DnaJ"
"Chl_C14_00653" 2218 "s59E13H2" 0 "Glycerol-3-phosphate dehydrogenase [NAD(P)+]"
"Chl_C14_00653" 2218 "s59E25H2" 0 "Glycerol-3-phosphate dehydrogenase [NAD(P)+]"
"Chl_C14_00653" 2218 "s59E5H2" 0 "Glycerol-3-phosphate dehydrogenase [NAD(P)+]"
"Chl_C14_00653" 2218 "s63E6H2" 0 "Glycerol-3-phosphate dehydrogenase [NAD(P)+]"
"Chl_C14_00654" 2219 "s59E13H2" 0 "Glycerol-3-phosphate acyltransferase"
"Chl_C14_00654" 2219 "s59E25H2" 0 "Glycerol-3-phosphate acyltransferase"
"Chl_C14_00654" 2219 "s59E5H2" 0 "Glycerol-3-phosphate acyltransferase"
"Chl_C14_00654" 2219 "s63E6H2" 0 "Glycerol-3-phosphate acyltransferase"
"Chl_C14_00655" 2220 "s59E13H2" 0 "GTPase Der"
"Chl_C14_00655" 2220 "s59E25H2" 0 "GTPase Der"
"Chl_C14_00655" 2220 "s59E5H2" 0 "GTPase Der"
"Chl_C14_00655" 2220 "s63E6H2" 0 "GTPase Der"
"Chl_C14_00656" 2221 "s59E13H2" 0 "hypothetical protein"

"Chl_C14_00656" 2221 "s59E25H2" 0 "hypothetical protein"
"Chl_C14_00656" 2221 "s59E5H2" 0 "hypothetical protein"
"Chl_C14_00656" 2221 "s63E6H2" 0 "hypothetical protein"
"Chl_C14_00657" 2222 "s59E13H2" 0 "Ornithine carbamoyltransferase"
"Chl_C14_00657" 2222 "s59E25H2" 0 "Ornithine carbamoyltransferase"
"Chl_C14_00657" 2222 "s59E5H2" 0.001691959 "Ornithine carbamoyltransferase"
"Chl_C14_00657" 2222 "s63E6H2" 0 "Ornithine carbamoyltransferase"
"Chl_C14_00658" 2223 "s59E13H2" 0 "NADH dehydrogenase"
"Chl_C14_00658" 2223 "s59E25H2" 0 "NADH dehydrogenase"
"Chl_C14_00658" 2223 "s59E5H2" 0 "NADH dehydrogenase"
"Chl_C14_00658" 2223 "s63E6H2" 0 "NADH dehydrogenase"
"Chl_C14_00659" 2224 "s59E13H2" 0 "Phosphoserine phosphatase 1"
"Chl_C14_00659" 2224 "s59E25H2" 0 "Phosphoserine phosphatase 1"
"Chl_C14_00659" 2224 "s59E5H2" 0 "Phosphoserine phosphatase 1"
"Chl_C14_00659" 2224 "s63E6H2" 0 "Phosphoserine phosphatase 1"
"Chl_C14_00660" 2225 "s59E13H2" 0 "Transposase IS116/IS110/IS902 family protein"
"Chl_C14_00660" 2225 "s59E25H2" 0 "Transposase IS116/IS110/IS902 family protein"
"Chl_C14_00660" 2225 "s59E5H2" 0.07186864 "Transposase IS116/IS110/IS902 family protein"
"Chl_C14_00660" 2225 "s63E6H2" 0 "Transposase IS116/IS110/IS902 family protein"
"Chl_C14_00661" 2226 "s59E13H2" 0.003935401 "hypothetical protein"
"Chl_C14_00661" 2226 "s59E25H2" 0 "hypothetical protein"
"Chl_C14_00661" 2226 "s59E5H2" 0 "hypothetical protein"
"Chl_C14_00661" 2226 "s63E6H2" 0.0100255 "hypothetical protein"
"Chl_C14_00663" 2228 "s59E13H2" 0.001786127 "Transposase"
"Chl_C14_00663" 2228 "s59E25H2" 0 "Transposase"
"Chl_C14_00663" 2228 "s59E5H2" 0 "Transposase"
"Chl_C14_00663" 2228 "s63E6H2" 1.093296 "Transposase"
"Chl_C14_00664" 2229 "s59E13H2" 0 "hypothetical protein"
"Chl_C14_00664" 2229 "s59E25H2" 0 "hypothetical protein"
"Chl_C14_00664" 2229 "s59E5H2" 0 "hypothetical protein"
"Chl_C14_00664" 2229 "s63E6H2" 0 "hypothetical protein"
"Chl_C14_00666" 2231 "s59E13H2" 0 "hypothetical protein"
"Chl_C14_00666" 2231 "s59E25H2" 0 "hypothetical protein"
"Chl_C14_00666" 2231 "s59E5H2" 0 "hypothetical protein"
"Chl_C14_00666" 2231 "s63E6H2" 0 "hypothetical protein"
"Chl_C14_00667" 2232 "s59E13H2" 0 "hypothetical protein"
"Chl_C14_00667" 2232 "s59E25H2" 0 "hypothetical protein"
"Chl_C14_00667" 2232 "s59E5H2" 0 "hypothetical protein"
"Chl_C14_00667" 2232 "s63E6H2" 0 "hypothetical protein"
"Chl_C14_00668" 2233 "s59E13H2" 0 "GIY-YIG nuclease superfamily protein"
"Chl_C14_00668" 2233 "s59E25H2" 0 "GIY-YIG nuclease superfamily protein"
"Chl_C14_00668" 2233 "s59E5H2" 0 "GIY-YIG nuclease superfamily protein"
"Chl_C14_00668" 2233 "s63E6H2" 0 "GIY-YIG nuclease superfamily protein"
"Chl_C14_00671" 2236 "s59E13H2" 0 "hypothetical protein"
"Chl_C14_00671" 2236 "s59E25H2" 0 "hypothetical protein"
"Chl_C14_00671" 2236 "s59E5H2" 0 "hypothetical protein"
"Chl_C14_00671" 2236 "s63E6H2" 0 "hypothetical protein"

"Chl_C14_00672" 2237 "s59E13H2" 0 "Glycine reductase complex component B subunit gamma"
"Chl_C14_00672" 2237 "s59E25H2" 0 "Glycine reductase complex component B subunit gamma"
"Chl_C14_00672" 2237 "s59E5H2" 0 "Glycine reductase complex component B subunit gamma"
"Chl_C14_00672" 2237 "s63E6H2" 0.05707273 "Glycine reductase complex component B subunit gamma"
"Chl_C14_00673" 2238 "s59E13H2" 0 "Glycine reductase complex component B subunit gamma"
"Chl_C14_00673" 2238 "s59E25H2" 0 "Glycine reductase complex component B subunit gamma"
"Chl_C14_00673" 2238 "s59E5H2" 0 "Glycine reductase complex component B subunit gamma"
"Chl_C14_00673" 2238 "s63E6H2" 0.09445351 "Glycine reductase complex component B subunit gamma"
"Chl_C14_00674" 2239 "s59E13H2" 0 "Glycine reductase complex component B subunits alpha and beta"
"Chl_C14_00674" 2239 "s59E25H2" 0 "Glycine reductase complex component B subunits alpha and beta"
"Chl_C14_00674" 2239 "s59E5H2" 0 "Glycine reductase complex component B subunits alpha and beta"
"Chl_C14_00674" 2239 "s63E6H2" 0.05064163 "Glycine reductase complex component B subunits alpha and beta"
"Chl_C14_00675" 2240 "s59E13H2" 0 "Glycine/sarcosine/betaine reductase complex component A"
"Chl_C14_00675" 2240 "s59E25H2" 0 "Glycine/sarcosine/betaine reductase complex component A"
"Chl_C14_00675" 2240 "s59E5H2" 0 "Glycine/sarcosine/betaine reductase complex component A"
"Chl_C14_00675" 2240 "s63E6H2" 0 "Glycine/sarcosine/betaine reductase complex component A"
"Chl_C14_00676" 2241 "s59E13H2" 0 "Glycine/sarcosine/betaine reductase complex component A"
"Chl_C14_00676" 2241 "s59E25H2" 0 "Glycine/sarcosine/betaine reductase complex component A"
"Chl_C14_00676" 2241 "s59E5H2" 0 "Glycine/sarcosine/betaine reductase complex component A"
"Chl_C14_00676" 2241 "s63E6H2" 0 "Glycine/sarcosine/betaine reductase complex component A"
"Chl_C14_00677" 2242 "s59E13H2" 0 "Thioredoxin"
"Chl_C14_00677" 2242 "s59E25H2" 0 "Thioredoxin"
"Chl_C14_00677" 2242 "s59E5H2" 0 "Thioredoxin"
"Chl_C14_00677" 2242 "s63E6H2" 0 "Thioredoxin"
"Chl_C14_00678" 2243 "s59E13H2" 0 "Glycine/sarcosine/betaine reductase complex component C subunit beta"
"Chl_C14_00678" 2243 "s59E25H2" 0 "Glycine/sarcosine/betaine reductase complex component C subunit beta"
"Chl_C14_00678" 2243 "s59E5H2" 0 "Glycine/sarcosine/betaine reductase complex component C subunit beta"
"Chl_C14_00678" 2243 "s63E6H2" 0 "Glycine/sarcosine/betaine reductase complex component C subunit beta"
"Chl_C14_00680" 2245 "s59E13H2" 0 "D-3-phosphoglycerate dehydrogenase"
"Chl_C14_00680" 2245 "s59E25H2" 0 "D-3-phosphoglycerate dehydrogenase"
"Chl_C14_00680" 2245 "s59E5H2" 0 "D-3-phosphoglycerate dehydrogenase"
"Chl_C14_00680" 2245 "s63E6H2" 0 "D-3-phosphoglycerate dehydrogenase"
"Chl_C14_00683" 2248 "s59E13H2" 0 "hypothetical protein"
"Chl_C14_00683" 2248 "s59E25H2" 0 "hypothetical protein"
"Chl_C14_00683" 2248 "s59E5H2" 0 "hypothetical protein"
"Chl_C14_00683" 2248 "s63E6H2" 0 "hypothetical protein"
"Chl_C14_00684" 2249 "s59E13H2" 0 "hypothetical protein"
"Chl_C14_00684" 2249 "s59E25H2" 0 "hypothetical protein"
"Chl_C14_00684" 2249 "s59E5H2" 0 "hypothetical protein"
"Chl_C14_00684" 2249 "s63E6H2" 0 "hypothetical protein"
"Chl_C14_00685" 2250 "s59E13H2" 0 "DctM-like transporters"
"Chl_C14_00685" 2250 "s59E25H2" 0 "DctM-like transporters"
"Chl_C14_00685" 2250 "s59E5H2" 0 "DctM-like transporters"
"Chl_C14_00685" 2250 "s63E6H2" 0 "DctM-like transporters"
"Chl_C14_00686" 2251 "s59E13H2" 0 "Thermophilic metalloprotease (M29)"
"Chl_C14_00686" 2251 "s59E25H2" 0 "Thermophilic metalloprotease (M29)"
"Chl_C14_00686" 2251 "s59E5H2" 0 "Thermophilic metalloprotease (M29)"

"Chl_C14_00686" 2251 "s63E6H2" 0 "Thermophilic metalloprotease (M29)"
"Chl_C14_00687" 2252 "s59E13H2" 0 "hypothetical protein"
"Chl_C14_00687" 2252 "s59E25H2" 0 "hypothetical protein"
"Chl_C14_00687" 2252 "s59E5H2" 0 "hypothetical protein"
"Chl_C14_00687" 2252 "s63E6H2" 0 "hypothetical protein"
"Chl_C14_00688" 2253 "s59E13H2" 0 "Enamine/imine deaminase"
"Chl_C14_00688" 2253 "s59E25H2" 0 "Enamine/imine deaminase"
"Chl_C14_00688" 2253 "s59E5H2" 0 "Enamine/imine deaminase"
"Chl_C14_00688" 2253 "s63E6H2" 0 "Enamine/imine deaminase"
"Chl_C14_00689" 2254 "s59E13H2" 0 "Acetylene hydratase"
"Chl_C14_00689" 2254 "s59E25H2" 0 "Acetylene hydratase"
"Chl_C14_00689" 2254 "s59E5H2" 0.005658055 "Acetylene hydratase"
"Chl_C14_00689" 2254 "s63E6H2" 0.404669 "Acetylene hydratase"
"Chl_C14_00690" 2255 "s59E13H2" 0 "L-hydantoinase"
"Chl_C14_00690" 2255 "s59E25H2" 0 "L-hydantoinase"
"Chl_C14_00690" 2255 "s59E5H2" 0 "L-hydantoinase"
"Chl_C14_00690" 2255 "s63E6H2" 0.0004387939 "L-hydantoinase"
"Chl_C14_00691" 2256 "s59E13H2" 0 "hypothetical protein"
"Chl_C14_00691" 2256 "s59E25H2" 0 "hypothetical protein"
"Chl_C14_00691" 2256 "s59E5H2" 0 "hypothetical protein"
"Chl_C14_00691" 2256 "s63E6H2" 0 "hypothetical protein"
"Chl_C14_00692" 2257 "s59E13H2" 0 "Putative serine protease HhoA precursor"
"Chl_C14_00692" 2257 "s59E25H2" 0 "Putative serine protease HhoA precursor"
"Chl_C14_00692" 2257 "s59E5H2" 0 "Putative serine protease HhoA precursor"
"Chl_C14_00692" 2257 "s63E6H2" 0 "Putative serine protease HhoA precursor"
"Chl_C14_00693" 2258 "s59E13H2" 0 "hypothetical protein"
"Chl_C14_00693" 2258 "s59E25H2" 0 "hypothetical protein"
"Chl_C14_00693" 2258 "s59E5H2" 0 "hypothetical protein"
"Chl_C14_00693" 2258 "s63E6H2" 0 "hypothetical protein"
"Chl_C14_00696" 2261 "s59E13H2" 0 "Antilisterial bacteriocin subtilosin biosynthesis protein AlbA"
"Chl_C14_00696" 2261 "s59E25H2" 0 "Antilisterial bacteriocin subtilosin biosynthesis protein AlbA"
"Chl_C14_00696" 2261 "s59E5H2" 0 "Antilisterial bacteriocin subtilosin biosynthesis protein AlbA"
"Chl_C14_00696" 2261 "s63E6H2" 0 "Antilisterial bacteriocin subtilosin biosynthesis protein AlbA"
"Chl_C14_00697" 2262 "s59E13H2" 0 "hypothetical protein"
"Chl_C14_00697" 2262 "s59E25H2" 0 "hypothetical protein"
"Chl_C14_00697" 2262 "s59E5H2" 0 "hypothetical protein"
"Chl_C14_00697" 2262 "s63E6H2" 0 "hypothetical protein"
"Chl_C14_00699" 2264 "s59E13H2" 0 "hypothetical protein"
"Chl_C14_00699" 2264 "s59E25H2" 0 "hypothetical protein"
"Chl_C14_00699" 2264 "s59E5H2" 0 "hypothetical protein"
"Chl_C14_00699" 2264 "s63E6H2" 0 "hypothetical protein"
"Chl_C14_00702" 2267 "s59E13H2" 0 "hypothetical protein"
"Chl_C14_00702" 2267 "s59E25H2" 0 "hypothetical protein"
"Chl_C14_00702" 2267 "s59E5H2" 0 "hypothetical protein"
"Chl_C14_00702" 2267 "s63E6H2" 0 "hypothetical protein"
"Chl_C14_00704" 2269 "s59E13H2" 0 "Recombinase"
"Chl_C14_00704" 2269 "s59E25H2" 0 "Recombinase"

"Chl_C14_00704" 2269 "s59E5H2" 0 "Recombinase"
"Chl_C14_00704" 2269 "s63E6H2" 0 "Recombinase"
"Chl_C14_00706" 2271 "s59E13H2" 0 "DNA polymerase III subunit alpha"
"Chl_C14_00706" 2271 "s59E25H2" 0 "DNA polymerase III subunit alpha"
"Chl_C14_00706" 2271 "s59E5H2" 0 "DNA polymerase III subunit alpha"
"Chl_C14_00706" 2271 "s63E6H2" 0.0008603257 "DNA polymerase III subunit alpha"
"Chl_C14_00707" 2272 "s59E13H2" 0 "hypothetical protein"
"Chl_C14_00707" 2272 "s59E25H2" 0 "hypothetical protein"
"Chl_C14_00707" 2272 "s59E5H2" 0 "hypothetical protein"
"Chl_C14_00707" 2272 "s63E6H2" 0 "hypothetical protein"
"Chl_C14_00709" 2274 "s59E13H2" 0 "hypothetical protein"
"Chl_C14_00709" 2274 "s59E25H2" 0 "hypothetical protein"
"Chl_C14_00709" 2274 "s59E5H2" 0 "hypothetical protein"
"Chl_C14_00709" 2274 "s63E6H2" 0 "hypothetical protein"
"Chl_C14_00710" 2275 "s59E13H2" 0 "Zinc ribbon domain protein"
"Chl_C14_00710" 2275 "s59E25H2" 0 "Zinc ribbon domain protein"
"Chl_C14_00710" 2275 "s59E5H2" 0 "Zinc ribbon domain protein"
"Chl_C14_00710" 2275 "s63E6H2" 0 "Zinc ribbon domain protein"
"Chl_C14_00711" 2276 "s59E13H2" 0 "N-substituted formamide deformylase precursor"
"Chl_C14_00711" 2276 "s59E25H2" 0 "N-substituted formamide deformylase precursor"
"Chl_C14_00711" 2276 "s59E5H2" 0.009016991 "N-substituted formamide deformylase precursor"
"Chl_C14_00711" 2276 "s63E6H2" 0 "N-substituted formamide deformylase precursor"
"Chl_C14_00712" 2277 "s59E13H2" 0 "putative MFS-type transporter YhjX"
"Chl_C14_00712" 2277 "s59E25H2" 0 "putative MFS-type transporter YhjX"
"Chl_C14_00712" 2277 "s59E5H2" 0 "putative MFS-type transporter YhjX"
"Chl_C14_00712" 2277 "s63E6H2" 0 "putative MFS-type transporter YhjX"
"Chl_C14_00713" 2278 "s59E13H2" 0 "putative NH(3)-dependent NAD(+) synthetase"
"Chl_C14_00713" 2278 "s59E25H2" 0 "putative NH(3)-dependent NAD(+) synthetase"
"Chl_C14_00713" 2278 "s59E5H2" 0 "putative NH(3)-dependent NAD(+) synthetase"
"Chl_C14_00713" 2278 "s63E6H2" 0 "putative NH(3)-dependent NAD(+) synthetase"
"Chl_C14_00714" 2279 "s59E13H2" 0 "Nitrilase, bromoxynil-specific"
"Chl_C14_00714" 2279 "s59E25H2" 0 "Nitrilase, bromoxynil-specific"
"Chl_C14_00714" 2279 "s59E5H2" 0 "Nitrilase, bromoxynil-specific"
"Chl_C14_00714" 2279 "s63E6H2" 0 "Nitrilase, bromoxynil-specific"
"Chl_C14_00716" 2281 "s59E13H2" 0 "Amidohydrolase"
"Chl_C14_00716" 2281 "s59E25H2" 0 "Amidohydrolase"
"Chl_C14_00716" 2281 "s59E5H2" 0 "Amidohydrolase"
"Chl_C14_00716" 2281 "s63E6H2" 0 "Amidohydrolase"
"Chl_C14_00717" 2282 "s59E13H2" 0 "Phosphate acetyltransferase"
"Chl_C14_00717" 2282 "s59E25H2" 0 "Phosphate acetyltransferase"
"Chl_C14_00717" 2282 "s59E5H2" 0 "Phosphate acetyltransferase"
"Chl_C14_00717" 2282 "s63E6H2" 0 "Phosphate acetyltransferase"
"Chl_C14_00718" 2283 "s59E13H2" 0 "Radical SAM superfamily protein"
"Chl_C14_00718" 2283 "s59E25H2" 0 "Radical SAM superfamily protein"
"Chl_C14_00718" 2283 "s59E5H2" 0 "Radical SAM superfamily protein"
"Chl_C14_00718" 2283 "s63E6H2" 0 "Radical SAM superfamily protein"
"Chl_C14_00720" 2285 "s59E13H2" 0 "hypothetical protein"

"Chl_C14_00720" 2285 "s59E25H2" 0 "hypothetical protein"
"Chl_C14_00720" 2285 "s59E5H2" 0 "hypothetical protein"
"Chl_C14_00720" 2285 "s63E6H2" 0 "hypothetical protein"
"Chl_C14_00721" 2286 "s59E13H2" 0 "Divergent AAA domain protein"
"Chl_C14_00721" 2286 "s59E25H2" 0 "Divergent AAA domain protein"
"Chl_C14_00721" 2286 "s59E5H2" 0 "Divergent AAA domain protein"
"Chl_C14_00721" 2286 "s63E6H2" 0 "Divergent AAA domain protein"
"Chl_C14_00722" 2287 "s59E13H2" 0 "hypothetical protein"
"Chl_C14_00722" 2287 "s59E25H2" 0 "hypothetical protein"
"Chl_C14_00722" 2287 "s59E5H2" 0 "hypothetical protein"
"Chl_C14_00722" 2287 "s63E6H2" 0 "hypothetical protein"
"Chl_C14_00723" 2288 "s59E13H2" 0 "Transcription-repair-coupling factor"
"Chl_C14_00723" 2288 "s59E25H2" 0 "Transcription-repair-coupling factor"
"Chl_C14_00723" 2288 "s59E5H2" 0 "Transcription-repair-coupling factor"
"Chl_C14_00723" 2288 "s63E6H2" 0.0002506743 "Transcription-repair-coupling factor"
"Chl_C14_00724" 2289 "s59E13H2" 0 "Inner membrane transport protein YajR"
"Chl_C14_00724" 2289 "s59E25H2" 0 "Inner membrane transport protein YajR"
"Chl_C14_00724" 2289 "s59E5H2" 0 "Inner membrane transport protein YajR"
"Chl_C14_00724" 2289 "s63E6H2" 0 "Inner membrane transport protein YajR"
"Chl_C14_00725" 2290 "s59E13H2" 0 "Glutamate 5-kinase"
"Chl_C14_00725" 2290 "s59E25H2" 0 "Glutamate 5-kinase"
"Chl_C14_00725" 2290 "s59E5H2" 0 "Glutamate 5-kinase"
"Chl_C14_00725" 2290 "s63E6H2" 0.002627444 "Glutamate 5-kinase"
"Chl_C14_00726" 2291 "s59E13H2" 0 "Gamma-glutamyl phosphate reductase"
"Chl_C14_00726" 2291 "s59E25H2" 0 "Gamma-glutamyl phosphate reductase"
"Chl_C14_00726" 2291 "s59E5H2" 0 "Gamma-glutamyl phosphate reductase"
"Chl_C14_00726" 2291 "s63E6H2" 0 "Gamma-glutamyl phosphate reductase"
"Chl_C14_00727" 2292 "s59E13H2" 0 "Ribonuclease PH"
"Chl_C14_00727" 2292 "s59E25H2" 0 "Ribonuclease PH"
"Chl_C14_00727" 2292 "s59E5H2" 0 "Ribonuclease PH"
"Chl_C14_00727" 2292 "s63E6H2" 0 "Ribonuclease PH"
"Chl_C14_00729" 2294 "s59E13H2" 0 "Nicotinamide-nucleotide amidohydrolase PncC"
"Chl_C14_00729" 2294 "s59E25H2" 0 "Nicotinamide-nucleotide amidohydrolase PncC"
"Chl_C14_00729" 2294 "s59E5H2" 0 "Nicotinamide-nucleotide amidohydrolase PncC"
"Chl_C14_00729" 2294 "s63E6H2" 0 "Nicotinamide-nucleotide amidohydrolase PncC"
"Chl_C14_00730" 2295 "s59E13H2" 0 "Radical SAM superfamily protein"
"Chl_C14_00730" 2295 "s59E25H2" 0 "Radical SAM superfamily protein"
"Chl_C14_00730" 2295 "s59E5H2" 0 "Radical SAM superfamily protein"
"Chl_C14_00730" 2295 "s63E6H2" 0 "Radical SAM superfamily protein"
"Chl_C14_00731" 2296 "s59E13H2" 0 "hypothetical protein"
"Chl_C14_00731" 2296 "s59E25H2" 0 "hypothetical protein"
"Chl_C14_00731" 2296 "s59E5H2" 0 "hypothetical protein"
"Chl_C14_00731" 2296 "s63E6H2" 0 "hypothetical protein"
"Chl_C14_00732" 2297 "s59E13H2" 0 "hypothetical protein"
"Chl_C14_00732" 2297 "s59E25H2" 0 "hypothetical protein"
"Chl_C14_00732" 2297 "s59E5H2" 0 "hypothetical protein"
"Chl_C14_00732" 2297 "s63E6H2" 0 "hypothetical protein"

"Chl_C14_00733" 2298 "s59E13H2" 0 "hypothetical protein"
"Chl_C14_00733" 2298 "s59E25H2" 0 "hypothetical protein"
"Chl_C14_00733" 2298 "s59E5H2" 0 "hypothetical protein"
"Chl_C14_00733" 2298 "s63E6H2" 0 "hypothetical protein"
"Chl_C14_00734" 2299 "s59E13H2" 0 "Protease 1 precursor"
"Chl_C14_00734" 2299 "s59E25H2" 0 "Protease 1 precursor"
"Chl_C14_00734" 2299 "s59E5H2" 0.00702227 "Protease 1 precursor"
"Chl_C14_00734" 2299 "s63E6H2" 0 "Protease 1 precursor"
"Chl_C14_00735" 2300 "s59E13H2" 0 "YcfA-like protein"
"Chl_C14_00735" 2300 "s59E25H2" 0 "YcfA-like protein"
"Chl_C14_00735" 2300 "s59E5H2" 0 "YcfA-like protein"
"Chl_C14_00735" 2300 "s63E6H2" 0 "YcfA-like protein"
"Chl_C14_00736" 2301 "s59E13H2" 0 "hypothetical protein"
"Chl_C14_00736" 2301 "s59E25H2" 0 "hypothetical protein"
"Chl_C14_00736" 2301 "s59E5H2" 0 "hypothetical protein"
"Chl_C14_00736" 2301 "s63E6H2" 0 "hypothetical protein"
"Chl_C14_00737" 2302 "s59E13H2" 0 "Chaperone protein DnaJ"
"Chl_C14_00737" 2302 "s59E25H2" 0 "Chaperone protein DnaJ"
"Chl_C14_00737" 2302 "s59E5H2" 0 "Chaperone protein DnaJ"
"Chl_C14_00737" 2302 "s63E6H2" 0 "Chaperone protein DnaJ"
"Chl_C14_00738" 2303 "s59E13H2" 0 "hypothetical protein"
"Chl_C14_00738" 2303 "s59E25H2" 0 "hypothetical protein"
"Chl_C14_00738" 2303 "s59E5H2" 0 "hypothetical protein"
"Chl_C14_00738" 2303 "s63E6H2" 0 "hypothetical protein"
"Chl_C14_00739" 2304 "s59E13H2" 0 "ComEC family competence protein"
"Chl_C14_00739" 2304 "s59E25H2" 0 "ComEC family competence protein"
"Chl_C14_00739" 2304 "s59E5H2" 0 "ComEC family competence protein"
"Chl_C14_00739" 2304 "s63E6H2" 0 "ComEC family competence protein"
"Chl_C14_00740" 2305 "s59E13H2" 0 "putative tyrosine recombinase XerC-like protein"
"Chl_C14_00740" 2305 "s59E25H2" 0 "putative tyrosine recombinase XerC-like protein"
"Chl_C14_00740" 2305 "s59E5H2" 0 "putative tyrosine recombinase XerC-like protein"
"Chl_C14_00740" 2305 "s63E6H2" 0 "putative tyrosine recombinase XerC-like protein"
"Chl_C14_00742" 2307 "s59E13H2" 0 "hypothetical protein"
"Chl_C14_00742" 2307 "s59E25H2" 0 "hypothetical protein"
"Chl_C14_00742" 2307 "s59E5H2" 0 "hypothetical protein"
"Chl_C14_00742" 2307 "s63E6H2" 0 "hypothetical protein"
"Chl_C14_00744" 2309 "s59E13H2" 0.01402055 "Transposase"
"Chl_C14_00744" 2309 "s59E25H2" 0 "Transposase"
"Chl_C14_00744" 2309 "s59E5H2" 1.390487 "Transposase"
"Chl_C14_00744" 2309 "s63E6H2" 0.0006117524 "Transposase"
"Chl_C14_00745" 2310 "s59E13H2" 0.003732269 "Integrase core domain protein"
"Chl_C14_00745" 2310 "s59E25H2" 0 "Integrase core domain protein"
"Chl_C14_00745" 2310 "s59E5H2" 1.232468 "Integrase core domain protein"
"Chl_C14_00745" 2310 "s63E6H2" 0.8650314 "Integrase core domain protein"
"Chl_C14_00746" 2311 "s59E13H2" 0 "hypothetical protein"
"Chl_C14_00746" 2311 "s59E25H2" 0 "hypothetical protein"
"Chl_C14_00746" 2311 "s59E5H2" 0 "hypothetical protein"

"Chl_C14_00746" 2311 "s63E6H2" 0.008319444 "hypothetical protein"
"Chl_C14_00747" 2312 "s59E13H2" 0 "hypothetical protein"
"Chl_C14_00747" 2312 "s59E25H2" 0 "hypothetical protein"
"Chl_C14_00747" 2312 "s59E5H2" 0 "hypothetical protein"
"Chl_C14_00747" 2312 "s63E6H2" 0 "hypothetical protein"
"Chl_C14_00748" 2313 "s59E13H2" 0 "Glycerol-3-phosphate acyltransferase"
"Chl_C14_00748" 2313 "s59E25H2" 0 "Glycerol-3-phosphate acyltransferase"
"Chl_C14_00748" 2313 "s59E5H2" 0 "Glycerol-3-phosphate acyltransferase"
"Chl_C14_00748" 2313 "s63E6H2" 0 "Glycerol-3-phosphate acyltransferase"
"Chl_C14_00749" 2314 "s59E13H2" 0 "hypothetical protein"
"Chl_C14_00749" 2314 "s59E25H2" 0 "hypothetical protein"
"Chl_C14_00749" 2314 "s59E5H2" 0 "hypothetical protein"
"Chl_C14_00749" 2314 "s63E6H2" 0 "hypothetical protein"
"Chl_C14_00750" 2315 "s59E13H2" 0 "CoB--CoM heterodisulfide reductase iron-sulfur subunit D"
"Chl_C14_00750" 2315 "s59E25H2" 0 "CoB--CoM heterodisulfide reductase iron-sulfur subunit D"
"Chl_C14_00750" 2315 "s59E5H2" 0 "CoB--CoM heterodisulfide reductase iron-sulfur subunit D"
"Chl_C14_00750" 2315 "s63E6H2" 0 "CoB--CoM heterodisulfide reductase iron-sulfur subunit D"
"Chl_C14_00751" 2316 "s59E13H2" 0 "Acyl-CoA dehydrogenase"
"Chl_C14_00751" 2316 "s59E25H2" 0 "Acyl-CoA dehydrogenase"
"Chl_C14_00751" 2316 "s59E5H2" 0 "Acyl-CoA dehydrogenase"
"Chl_C14_00751" 2316 "s63E6H2" 0 "Acyl-CoA dehydrogenase"
"Chl_C14_00752" 2317 "s59E13H2" 0 "Long-chain-fatty-acid--CoA ligase"
"Chl_C14_00752" 2317 "s59E25H2" 0 "Long-chain-fatty-acid--CoA ligase"
"Chl_C14_00752" 2317 "s59E5H2" 0 "Long-chain-fatty-acid--CoA ligase"
"Chl_C14_00752" 2317 "s63E6H2" 0 "Long-chain-fatty-acid--CoA ligase"
"Chl_C14_00753" 2318 "s59E13H2" 0 "NAD-dependent protein deacylase 2"
"Chl_C14_00753" 2318 "s59E25H2" 0 "NAD-dependent protein deacylase 2"
"Chl_C14_00753" 2318 "s59E5H2" 0 "NAD-dependent protein deacylase 2"
"Chl_C14_00753" 2318 "s63E6H2" 0 "NAD-dependent protein deacylase 2"
"Chl_C14_00754" 2319 "s59E13H2" 0 "3-hydroxypropionyl-coenzyme A dehydratase"
"Chl_C14_00754" 2319 "s59E25H2" 0 "3-hydroxypropionyl-coenzyme A dehydratase"
"Chl_C14_00754" 2319 "s59E5H2" 0 "3-hydroxypropionyl-coenzyme A dehydratase"
"Chl_C14_00754" 2319 "s63E6H2" 0 "3-hydroxypropionyl-coenzyme A dehydratase"
"Chl_C14_00755" 2320 "s59E13H2" 0 "3-hydroxypropionyl-coenzyme A dehydratase"
"Chl_C14_00755" 2320 "s59E25H2" 0 "3-hydroxypropionyl-coenzyme A dehydratase"
"Chl_C14_00755" 2320 "s59E5H2" 0 "3-hydroxypropionyl-coenzyme A dehydratase"
"Chl_C14_00755" 2320 "s63E6H2" 0 "3-hydroxypropionyl-coenzyme A dehydratase"
"Chl_C14_00756" 2321 "s59E13H2" 0.002432387 "Endonuclease III"
"Chl_C14_00756" 2321 "s59E25H2" 0 "Endonuclease III"
"Chl_C14_00756" 2321 "s59E5H2" 0 "Endonuclease III"
"Chl_C14_00756" 2321 "s63E6H2" 0 "Endonuclease III"
"Chl_C14_00757" 2322 "s59E13H2" 0 "S-adenosyl-L-methionine-binding protein"
"Chl_C14_00757" 2322 "s59E25H2" 0 "S-adenosyl-L-methionine-binding protein"
"Chl_C14_00757" 2322 "s59E5H2" 0 "S-adenosyl-L-methionine-binding protein"
"Chl_C14_00757" 2322 "s63E6H2" 0 "S-adenosyl-L-methionine-binding protein"
"Chl_C14_00758" 2323 "s59E13H2" 0 "2-oxoglutarate synthase subunit KorC"
"Chl_C14_00758" 2323 "s59E25H2" 0 "2-oxoglutarate synthase subunit KorC"

"Chl_C14_00758" 2323 "s59E5H2" 0 "2-oxoglutarate synthase subunit KorC"
"Chl_C14_00758" 2323 "s63E6H2" 0 "2-oxoglutarate synthase subunit KorC"
"Chl_C14_00759" 2324 "s59E13H2" 0 "2-oxoglutarate synthase subunit KorB"
"Chl_C14_00759" 2324 "s59E25H2" 0 "2-oxoglutarate synthase subunit KorB"
"Chl_C14_00759" 2324 "s59E5H2" 0 "2-oxoglutarate synthase subunit KorB"
"Chl_C14_00759" 2324 "s63E6H2" 0 "2-oxoglutarate synthase subunit KorB"
"Chl_C14_00760" 2325 "s59E13H2" 0 "2-oxoglutarate synthase subunit KorA"
"Chl_C14_00760" 2325 "s59E25H2" 0 "2-oxoglutarate synthase subunit KorA"
"Chl_C14_00760" 2325 "s59E5H2" 0 "2-oxoglutarate synthase subunit KorA"
"Chl_C14_00760" 2325 "s63E6H2" 0 "2-oxoglutarate synthase subunit KorA"
"Chl_C14_00761" 2326 "s59E13H2" 0 "Methionyl-tRNA formyltransferase"
"Chl_C14_00761" 2326 "s59E25H2" 0 "Methionyl-tRNA formyltransferase"
"Chl_C14_00761" 2326 "s59E5H2" 0 "Methionyl-tRNA formyltransferase"
"Chl_C14_00761" 2326 "s63E6H2" 0 "Methionyl-tRNA formyltransferase"
"Chl_C14_00762" 2327 "s59E13H2" 0 "Nucleoside triphosphate pyrophosphohydrolase/pyrophosphatase MazG"
"Chl_C14_00762" 2327 "s59E25H2" 0 "Nucleoside triphosphate pyrophosphohydrolase/pyrophosphatase MazG"
"Chl_C14_00762" 2327 "s59E5H2" 0 "Nucleoside triphosphate pyrophosphohydrolase/pyrophosphatase MazG"
"Chl_C14_00762" 2327 "s63E6H2" 0 "Nucleoside triphosphate pyrophosphohydrolase/pyrophosphatase MazG"
"Chl_C14_00764" 2329 "s59E13H2" 0 "2,3-bisphosphoglycerate-independent phosphoglycerate mutase"
"Chl_C14_00764" 2329 "s59E25H2" 0 "2,3-bisphosphoglycerate-independent phosphoglycerate mutase"
"Chl_C14_00764" 2329 "s59E5H2" 0 "2,3-bisphosphoglycerate-independent phosphoglycerate mutase"
"Chl_C14_00764" 2329 "s63E6H2" 0 "2,3-bisphosphoglycerate-independent phosphoglycerate mutase"
"Chl_C14_00765" 2330 "s59E13H2" 0 "hypothetical protein"
"Chl_C14_00765" 2330 "s59E25H2" 0 "hypothetical protein"
"Chl_C14_00765" 2330 "s59E5H2" 0 "hypothetical protein"
"Chl_C14_00765" 2330 "s63E6H2" 0 "hypothetical protein"
"Chl_C14_00766" 2331 "s59E13H2" 0 "Aspartokinase"
"Chl_C14_00766" 2331 "s59E25H2" 0 "Aspartokinase"
"Chl_C14_00766" 2331 "s59E5H2" 0 "Aspartokinase"
"Chl_C14_00766" 2331 "s63E6H2" 0 "Aspartokinase"
"Chl_C14_00767" 2332 "s59E13H2" 0 "Aspartokinase"
"Chl_C14_00767" 2332 "s59E25H2" 0 "Aspartokinase"
"Chl_C14_00767" 2332 "s59E5H2" 0 "Aspartokinase"
"Chl_C14_00767" 2332 "s63E6H2" 0 "Aspartokinase"
"Chl_C14_00768" 2333 "s59E13H2" 0 "Tetratricopeptide repeat protein"
"Chl_C14_00768" 2333 "s59E25H2" 0 "Tetratricopeptide repeat protein"
"Chl_C14_00768" 2333 "s59E5H2" 0 "Tetratricopeptide repeat protein"
"Chl_C14_00768" 2333 "s63E6H2" 0 "Tetratricopeptide repeat protein"
"Chl_C14_00769" 2334 "s59E13H2" 0 "DNA gyrase subunit A"
"Chl_C14_00769" 2334 "s59E25H2" 0 "DNA gyrase subunit A"
"Chl_C14_00769" 2334 "s59E5H2" 0 "DNA gyrase subunit A"
"Chl_C14_00769" 2334 "s63E6H2" 0 "DNA gyrase subunit A"
"Chl_C14_00771" 2336 "s59E13H2" 0 "tRNA(Ile)-lysidine synthase"
"Chl_C14_00771" 2336 "s59E25H2" 0 "tRNA(Ile)-lysidine synthase"
"Chl_C14_00771" 2336 "s59E5H2" 0 "tRNA(Ile)-lysidine synthase"
"Chl_C14_00771" 2336 "s63E6H2" 0 "tRNA(Ile)-lysidine synthase"
"Chl_C14_00772" 2337 "s59E13H2" 0 "hypothetical protein"

"Chl_C14_00772" 2337 "s59E25H2" 0 "hypothetical protein"
"Chl_C14_00772" 2337 "s59E5H2" 0 "hypothetical protein"
"Chl_C14_00772" 2337 "s63E6H2" 0 "hypothetical protein"
"Chl_C14_00773" 2338 "s59E13H2" 0 "Putative nicotinate phosphoribosyltransferase"
"Chl_C14_00773" 2338 "s59E25H2" 0 "Putative nicotinate phosphoribosyltransferase"
"Chl_C14_00773" 2338 "s59E5H2" 0.002288715 "Putative nicotinate phosphoribosyltransferase"
"Chl_C14_00773" 2338 "s63E6H2" 0 "Putative nicotinate phosphoribosyltransferase"
"Chl_C14_00774" 2339 "s59E13H2" 0 "hypothetical protein"
"Chl_C14_00774" 2339 "s59E25H2" 0 "hypothetical protein"
"Chl_C14_00774" 2339 "s59E5H2" 0 "hypothetical protein"
"Chl_C14_00774" 2339 "s63E6H2" 0 "hypothetical protein"
"Chl_C14_00775" 2340 "s59E13H2" 0 "Bifunctional purine biosynthesis protein PurH"
"Chl_C14_00775" 2340 "s59E25H2" 0 "Bifunctional purine biosynthesis protein PurH"
"Chl_C14_00775" 2340 "s59E5H2" 0 "Bifunctional purine biosynthesis protein PurH"
"Chl_C14_00775" 2340 "s63E6H2" 0 "Bifunctional purine biosynthesis protein PurH"
"Chl_C14_00776" 2341 "s59E13H2" 0.001633052 "Phosphoribosylformylglycinamidine cyclo-ligase"
"Chl_C14_00776" 2341 "s59E25H2" 0 "Phosphoribosylformylglycinamidine cyclo-ligase"
"Chl_C14_00776" 2341 "s59E5H2" 0 "Phosphoribosylformylglycinamidine cyclo-ligase"
"Chl_C14_00776" 2341 "s63E6H2" 0.007301021 "Phosphoribosylformylglycinamidine cyclo-ligase"
"Chl_C14_00777" 2342 "s59E13H2" 0 "hypothetical protein"
"Chl_C14_00777" 2342 "s59E25H2" 0 "hypothetical protein"
"Chl_C14_00777" 2342 "s59E5H2" 0 "hypothetical protein"
"Chl_C14_00777" 2342 "s63E6H2" 0 "hypothetical protein"
"Chl_C14_00778" 2343 "s59E13H2" 0.01120134 "Amidophosphoribosyltransferase precursor"
"Chl_C14_00778" 2343 "s59E25H2" 0 "Amidophosphoribosyltransferase precursor"
"Chl_C14_00778" 2343 "s59E5H2" 0 "Amidophosphoribosyltransferase precursor"
"Chl_C14_00778" 2343 "s63E6H2" 0 "Amidophosphoribosyltransferase precursor"
"Chl_C14_00779" 2344 "s59E13H2" 0 "Amidohydrolase"
"Chl_C14_00779" 2344 "s59E25H2" 0 "Amidohydrolase"
"Chl_C14_00779" 2344 "s59E5H2" 0.01013949 "Amidohydrolase"
"Chl_C14_00779" 2344 "s63E6H2" 0 "Amidohydrolase"
"Chl_C14_00780" 2345 "s59E13H2" 0 "2'-5'-RNA ligase"
"Chl_C14_00780" 2345 "s59E25H2" 0 "2'-5'-RNA ligase"
"Chl_C14_00780" 2345 "s59E5H2" 0 "2'-5'-RNA ligase"
"Chl_C14_00780" 2345 "s63E6H2" 0 "2'-5'-RNA ligase"
"Chl_C14_00781" 2346 "s59E13H2" 0 "HTH-type transcriptional activator RhaS"
"Chl_C14_00781" 2346 "s59E25H2" 0 "HTH-type transcriptional activator RhaS"
"Chl_C14_00781" 2346 "s59E5H2" 0 "HTH-type transcriptional activator RhaS"
"Chl_C14_00781" 2346 "s63E6H2" 0 "HTH-type transcriptional activator RhaS"
"Chl_C14_00783" 2348 "s59E13H2" 0 "DNA utilization protein GntX"
"Chl_C14_00783" 2348 "s59E25H2" 0 "DNA utilization protein GntX"
"Chl_C14_00783" 2348 "s59E5H2" 0.00404158 "DNA utilization protein GntX"
"Chl_C14_00783" 2348 "s63E6H2" 0 "DNA utilization protein GntX"
"Chl_C14_00784" 2349 "s59E13H2" 0 "hypothetical protein"
"Chl_C14_00784" 2349 "s59E25H2" 0 "hypothetical protein"
"Chl_C14_00784" 2349 "s59E5H2" 0 "hypothetical protein"
"Chl_C14_00784" 2349 "s63E6H2" 0 "hypothetical protein"

"Chl_C14_00785" 2350 "s59E13H2" 0 "hypothetical protein"
"Chl_C14_00785" 2350 "s59E25H2" 0 "hypothetical protein"
"Chl_C14_00785" 2350 "s59E5H2" 0 "hypothetical protein"
"Chl_C14_00785" 2350 "s63E6H2" 0 "hypothetical protein"
"Chl_C14_00786" 2351 "s59E13H2" 0 "hypothetical protein"
"Chl_C14_00786" 2351 "s59E25H2" 0 "hypothetical protein"
"Chl_C14_00786" 2351 "s59E5H2" 0 "hypothetical protein"
"Chl_C14_00786" 2351 "s63E6H2" 0 "hypothetical protein"
"Chl_C14_00787" 2352 "s59E13H2" 0 "Methylated-DNA--protein-cysteine methyltransferase"
"Chl_C14_00787" 2352 "s59E25H2" 0 "Methylated-DNA--protein-cysteine methyltransferase"
"Chl_C14_00787" 2352 "s59E5H2" 0 "Methylated-DNA--protein-cysteine methyltransferase"
"Chl_C14_00787" 2352 "s63E6H2" 0 "Methylated-DNA--protein-cysteine methyltransferase"
"Chl_C14_00788" 2353 "s59E13H2" 0 "Chagasin family peptidase inhibitor I42"
"Chl_C14_00788" 2353 "s59E25H2" 0 "Chagasin family peptidase inhibitor I42"
"Chl_C14_00788" 2353 "s59E5H2" 0 "Chagasin family peptidase inhibitor I42"
"Chl_C14_00788" 2353 "s63E6H2" 0 "Chagasin family peptidase inhibitor I42"
"Chl_C14_00789" 2354 "s59E13H2" 0 "hypothetical protein"
"Chl_C14_00789" 2354 "s59E25H2" 0 "hypothetical protein"
"Chl_C14_00789" 2354 "s59E5H2" 0 "hypothetical protein"
"Chl_C14_00789" 2354 "s63E6H2" 0 "hypothetical protein"
"Chl_C14_00790" 2355 "s59E13H2" 0 "hypothetical protein"
"Chl_C14_00790" 2355 "s59E25H2" 0 "hypothetical protein"
"Chl_C14_00790" 2355 "s59E5H2" 0.005095703 "hypothetical protein"
"Chl_C14_00790" 2355 "s63E6H2" 0 "hypothetical protein"
"Chl_C14_00791" 2356 "s59E13H2" 0 "hypothetical protein"
"Chl_C14_00791" 2356 "s59E25H2" 0 "hypothetical protein"
"Chl_C14_00791" 2356 "s59E5H2" 0 "hypothetical protein"
"Chl_C14_00791" 2356 "s63E6H2" 0 "hypothetical protein"
"Chl_C14_00792" 2357 "s59E13H2" 0 "NAD-dependent protein deacylase 2"
"Chl_C14_00792" 2357 "s59E25H2" 0 "NAD-dependent protein deacylase 2"
"Chl_C14_00792" 2357 "s59E5H2" 0 "NAD-dependent protein deacylase 2"
"Chl_C14_00792" 2357 "s63E6H2" 0 "NAD-dependent protein deacylase 2"
"Chl_C14_00793" 2358 "s59E13H2" 0 "hypothetical protein"
"Chl_C14_00793" 2358 "s59E25H2" 0 "hypothetical protein"
"Chl_C14_00793" 2358 "s59E5H2" 0 "hypothetical protein"
"Chl_C14_00793" 2358 "s63E6H2" 0 "hypothetical protein"
"Chl_C14_00794" 2359 "s59E13H2" 0 "Acetoin utilization protein AcuC"
"Chl_C14_00794" 2359 "s59E25H2" 0 "Acetoin utilization protein AcuC"
"Chl_C14_00794" 2359 "s59E5H2" 0 "Acetoin utilization protein AcuC"
"Chl_C14_00794" 2359 "s63E6H2" 0 "Acetoin utilization protein AcuC"
"Chl_C14_00795" 2360 "s59E13H2" 0 "hypothetical protein"
"Chl_C14_00795" 2360 "s59E25H2" 0 "hypothetical protein"
"Chl_C14_00795" 2360 "s59E5H2" 0 "hypothetical protein"
"Chl_C14_00795" 2360 "s63E6H2" 0 "hypothetical protein"
"Chl_C14_00796" 2361 "s59E13H2" 0 "hypothetical protein"
"Chl_C14_00796" 2361 "s59E25H2" 0 "hypothetical protein"
"Chl_C14_00796" 2361 "s59E5H2" 0 "hypothetical protein"

"Chl_C14_00796" 2361 "s63E6H2" 0 "hypothetical protein"
"Chl_C14_00797" 2362 "s59E13H2" 0 "Ribonuclease HI"
"Chl_C14_00797" 2362 "s59E25H2" 0 "Ribonuclease HI"
"Chl_C14_00797" 2362 "s59E5H2" 0 "Ribonuclease HI"
"Chl_C14_00797" 2362 "s63E6H2" 0 "Ribonuclease HI"
"Chl_C14_00798" 2363 "s59E13H2" 0 "IPT/TIG domain protein"
"Chl_C14_00798" 2363 "s59E25H2" 0 "IPT/TIG domain protein"
"Chl_C14_00798" 2363 "s59E5H2" 0 "IPT/TIG domain protein"
"Chl_C14_00798" 2363 "s63E6H2" 0 "IPT/TIG domain protein"
"Chl_C14_00799" 2364 "s59E13H2" 0 "Alpha-monoglucosyldiacylglycerol synthase"
"Chl_C14_00799" 2364 "s59E25H2" 0 "Alpha-monoglucosyldiacylglycerol synthase"
"Chl_C14_00799" 2364 "s59E5H2" 0.05201264 "Alpha-monoglucosyldiacylglycerol synthase"
"Chl_C14_00799" 2364 "s63E6H2" 0 "Alpha-monoglucosyldiacylglycerol synthase"
"Chl_C14_00800" 2365 "s59E13H2" 0.5002689 "Transposase"
"Chl_C14_00800" 2365 "s59E25H2" 0 "Transposase"
"Chl_C14_00800" 2365 "s59E5H2" 0 "Transposase"
"Chl_C14_00800" 2365 "s63E6H2" 0 "Transposase"
"Chl_C14_00802" 2367 "s59E13H2" 0 "hypothetical protein"
"Chl_C14_00802" 2367 "s59E25H2" 0 "hypothetical protein"
"Chl_C14_00802" 2367 "s59E5H2" 0 "hypothetical protein"
"Chl_C14_00802" 2367 "s63E6H2" 0 "hypothetical protein"
"Chl_C14_00803" 2368 "s59E13H2" 0 "Polyketide cyclase / dehydrase and lipid transport"
"Chl_C14_00803" 2368 "s59E25H2" 0 "Polyketide cyclase / dehydrase and lipid transport"
"Chl_C14_00803" 2368 "s59E5H2" 0 "Polyketide cyclase / dehydrase and lipid transport"
"Chl_C14_00803" 2368 "s63E6H2" 0 "Polyketide cyclase / dehydrase and lipid transport"
"Chl_C14_00804" 2369 "s59E13H2" 0 "hypothetical protein"
"Chl_C14_00804" 2369 "s59E25H2" 0 "hypothetical protein"
"Chl_C14_00804" 2369 "s59E5H2" 0 "hypothetical protein"
"Chl_C14_00804" 2369 "s63E6H2" 0 "hypothetical protein"
"Chl_C14_00805" 2370 "s59E13H2" 0 "hypothetical protein"
"Chl_C14_00805" 2370 "s59E25H2" 0 "hypothetical protein"
"Chl_C14_00805" 2370 "s59E5H2" 0 "hypothetical protein"
"Chl_C14_00805" 2370 "s63E6H2" 0 "hypothetical protein"
"Chl_C14_00806" 2371 "s59E13H2" 0 "Peptide chain release factor 2"
"Chl_C14_00806" 2371 "s59E25H2" 0 "Peptide chain release factor 2"
"Chl_C14_00806" 2371 "s59E5H2" 0.00400163 "Peptide chain release factor 2"
"Chl_C14_00806" 2371 "s63E6H2" 0 "Peptide chain release factor 2"
"Chl_C14_00807" 2372 "s59E13H2" 0 "Endonuclease V"
"Chl_C14_00807" 2372 "s59E25H2" 0 "Endonuclease V"
"Chl_C14_00807" 2372 "s59E5H2" 0 "Endonuclease V"
"Chl_C14_00807" 2372 "s63E6H2" 0 "Endonuclease V"
"Chl_C14_00808" 2373 "s59E13H2" 0 "30S ribosomal protein S16"
"Chl_C14_00808" 2373 "s59E25H2" 0 "30S ribosomal protein S16"
"Chl_C14_00808" 2373 "s59E5H2" 0 "30S ribosomal protein S16"
"Chl_C14_00808" 2373 "s63E6H2" 0 "30S ribosomal protein S16"
"Chl_C14_00809" 2374 "s59E13H2" 0 "hypothetical protein"
"Chl_C14_00809" 2374 "s59E25H2" 0 "hypothetical protein"

"Chl_C14_00809" 2374 "s59E5H2" 0 "hypothetical protein"
"Chl_C14_00809" 2374 "s63E6H2" 0 "hypothetical protein"
"Chl_C14_00810" 2375 "s59E13H2" 0 "hypothetical protein"
"Chl_C14_00810" 2375 "s59E25H2" 0 "hypothetical protein"
"Chl_C14_00810" 2375 "s59E5H2" 0 "hypothetical protein"
"Chl_C14_00810" 2375 "s63E6H2" 0 "hypothetical protein"
"Chl_C14_00811" 2376 "s59E13H2" 0 "RNA 3'-terminal phosphate cyclase"
"Chl_C14_00811" 2376 "s59E25H2" 0 "RNA 3'-terminal phosphate cyclase"
"Chl_C14_00811" 2376 "s59E5H2" 0 "RNA 3'-terminal phosphate cyclase"
"Chl_C14_00811" 2376 "s63E6H2" 0 "RNA 3'-terminal phosphate cyclase"
"Chl_C14_00812" 2377 "s59E13H2" 0 "3-oxoadipate CoA-transferase subunit B"
"Chl_C14_00812" 2377 "s59E25H2" 0 "3-oxoadipate CoA-transferase subunit B"
"Chl_C14_00812" 2377 "s59E5H2" 0 "3-oxoadipate CoA-transferase subunit B"
"Chl_C14_00812" 2377 "s63E6H2" 0 "3-oxoadipate CoA-transferase subunit B"
"Chl_C14_00813" 2378 "s59E13H2" 0 "3-oxoadipate CoA-transferase subunit A"
"Chl_C14_00813" 2378 "s59E25H2" 0 "3-oxoadipate CoA-transferase subunit A"
"Chl_C14_00813" 2378 "s59E5H2" 0 "3-oxoadipate CoA-transferase subunit A"
"Chl_C14_00813" 2378 "s63E6H2" 0 "3-oxoadipate CoA-transferase subunit A"
"Chl_C14_00814" 2379 "s59E13H2" 0 "4-hydroxybutyryl-CoA dehydratase/vinylacetyl-CoA-Delta-isomerase"
"Chl_C14_00814" 2379 "s59E25H2" 0 "4-hydroxybutyryl-CoA dehydratase/vinylacetyl-CoA-Delta-isomerase"
"Chl_C14_00814" 2379 "s59E5H2" 0 "4-hydroxybutyryl-CoA dehydratase/vinylacetyl-CoA-Delta-isomerase"
"Chl_C14_00814" 2379 "s63E6H2" 0 "4-hydroxybutyryl-CoA dehydratase/vinylacetyl-CoA-Delta-isomerase"
"Chl_C14_00816" 2381 "s59E13H2" 0 "p-aminobenzoyl-glutamate hydrolase subunit B"
"Chl_C14_00816" 2381 "s59E25H2" 0 "p-aminobenzoyl-glutamate hydrolase subunit B"
"Chl_C14_00816" 2381 "s59E5H2" 0 "p-aminobenzoyl-glutamate hydrolase subunit B"
"Chl_C14_00816" 2381 "s63E6H2" 0 "p-aminobenzoyl-glutamate hydrolase subunit B"
"Chl_C14_00817" 2382 "s59E13H2" 0 "hypothetical protein"
"Chl_C14_00817" 2382 "s59E25H2" 0 "hypothetical protein"
"Chl_C14_00817" 2382 "s59E5H2" 0 "hypothetical protein"
"Chl_C14_00817" 2382 "s63E6H2" 0 "hypothetical protein"
"Chl_C14_00818" 2383 "s59E13H2" 0 "hypothetical protein"
"Chl_C14_00818" 2383 "s59E25H2" 0 "hypothetical protein"
"Chl_C14_00818" 2383 "s59E5H2" 0 "hypothetical protein"
"Chl_C14_00818" 2383 "s63E6H2" 0 "hypothetical protein"
"Chl_C14_00819" 2384 "s59E13H2" 0 "R-phenyllactate dehydratase activator"
"Chl_C14_00819" 2384 "s59E25H2" 0 "R-phenyllactate dehydratase activator"
"Chl_C14_00819" 2384 "s59E5H2" 0 "R-phenyllactate dehydratase activator"
"Chl_C14_00819" 2384 "s63E6H2" 0 "R-phenyllactate dehydratase activator"
"Chl_C14_00820" 2385 "s59E13H2" 0 "Phosphate acyltransferase"
"Chl_C14_00820" 2385 "s59E25H2" 0 "Phosphate acyltransferase"
"Chl_C14_00820" 2385 "s59E5H2" 0 "Phosphate acyltransferase"
"Chl_C14_00820" 2385 "s63E6H2" 0 "Phosphate acyltransferase"
"Chl_C14_00821" 2386 "s59E13H2" 0 "Thioredoxin-1"
"Chl_C14_00821" 2386 "s59E25H2" 0 "Thioredoxin-1"
"Chl_C14_00821" 2386 "s59E5H2" 0 "Thioredoxin-1"
"Chl_C14_00821" 2386 "s63E6H2" 0 "Thioredoxin-1"
"Chl_C14_00822" 2387 "s59E13H2" 0 "hypothetical protein"

"Chl_C14_00822" 2387 "s59E25H2" 0 "hypothetical protein"
"Chl_C14_00822" 2387 "s59E5H2" 0 "hypothetical protein"
"Chl_C14_00822" 2387 "s63E6H2" 0 "hypothetical protein"
"Chl_C14_00823" 2388 "s59E13H2" 0 "Thioredoxin reductase"
"Chl_C14_00823" 2388 "s59E25H2" 0 "Thioredoxin reductase"
"Chl_C14_00823" 2388 "s59E5H2" 0 "Thioredoxin reductase"
"Chl_C14_00823" 2388 "s63E6H2" 0 "Thioredoxin reductase"
"Chl_C14_00824" 2389 "s59E13H2" 0 "50S ribosomal protein L9"
"Chl_C14_00824" 2389 "s59E25H2" 0 "50S ribosomal protein L9"
"Chl_C14_00824" 2389 "s59E5H2" 0 "50S ribosomal protein L9"
"Chl_C14_00824" 2389 "s63E6H2" 0 "50S ribosomal protein L9"
"Chl_C14_00825" 2390 "s59E13H2" 0 "Replicative DNA helicase"
"Chl_C14_00825" 2390 "s59E25H2" 0 "Replicative DNA helicase"
"Chl_C14_00825" 2390 "s59E5H2" 0 "Replicative DNA helicase"
"Chl_C14_00825" 2390 "s63E6H2" 0 "Replicative DNA helicase"
"Chl_C14_00826" 2391 "s59E13H2" 0 "Replication initiation and membrane attachment"
"Chl_C14_00826" 2391 "s59E25H2" 0 "Replication initiation and membrane attachment"
"Chl_C14_00826" 2391 "s59E5H2" 0.007137666 "Replication initiation and membrane attachment"
"Chl_C14_00826" 2391 "s63E6H2" 0 "Replication initiation and membrane attachment"
"Chl_C14_00827" 2392 "s59E13H2" 0 "Succinyl-CoA:(R)-benzylsuccinate CoA-transferase subunit BbsE"
"Chl_C14_00827" 2392 "s59E25H2" 0 "Succinyl-CoA:(R)-benzylsuccinate CoA-transferase subunit BbsE"
"Chl_C14_00827" 2392 "s59E5H2" 0 "Succinyl-CoA:(R)-benzylsuccinate CoA-transferase subunit BbsE"
"Chl_C14_00827" 2392 "s63E6H2" 0 "Succinyl-CoA:(R)-benzylsuccinate CoA-transferase subunit BbsE"
"Chl_C14_00828" 2393 "s59E13H2" 0 "Succinyl-CoA:(R)-benzylsuccinate CoA-transferase subunit BbsF"
"Chl_C14_00828" 2393 "s59E25H2" 0 "Succinyl-CoA:(R)-benzylsuccinate CoA-transferase subunit BbsF"
"Chl_C14_00828" 2393 "s59E5H2" 0 "Succinyl-CoA:(R)-benzylsuccinate CoA-transferase subunit BbsF"
"Chl_C14_00828" 2393 "s63E6H2" 0 "Succinyl-CoA:(R)-benzylsuccinate CoA-transferase subunit BbsF"
"Chl_C14_00831" 2396 "s59E13H2" 0 "putative transcriptional regulatory protein"
"Chl_C14_00831" 2396 "s59E25H2" 0 "putative transcriptional regulatory protein"
"Chl_C14_00831" 2396 "s59E5H2" 0 "putative transcriptional regulatory protein"
"Chl_C14_00831" 2396 "s63E6H2" 0 "putative transcriptional regulatory protein"
"Chl_C14_00832" 2397 "s59E13H2" 0 "Crossover junction endodeoxyribonuclease RuvC"
"Chl_C14_00832" 2397 "s59E25H2" 0 "Crossover junction endodeoxyribonuclease RuvC"
"Chl_C14_00832" 2397 "s59E5H2" 0 "Crossover junction endodeoxyribonuclease RuvC"
"Chl_C14_00832" 2397 "s63E6H2" 0 "Crossover junction endodeoxyribonuclease RuvC"
"Chl_C14_00833" 2398 "s59E13H2" 0 "Holliday junction ATP-dependent DNA helicase RuvA"
"Chl_C14_00833" 2398 "s59E25H2" 0 "Holliday junction ATP-dependent DNA helicase RuvA"
"Chl_C14_00833" 2398 "s59E5H2" 0 "Holliday junction ATP-dependent DNA helicase RuvA"
"Chl_C14_00833" 2398 "s63E6H2" 0 "Holliday junction ATP-dependent DNA helicase RuvA"
"Chl_C14_00834" 2399 "s59E13H2" 0 "hypothetical protein"
"Chl_C14_00834" 2399 "s59E25H2" 0 "hypothetical protein"
"Chl_C14_00834" 2399 "s59E5H2" 0 "hypothetical protein"
"Chl_C14_00834" 2399 "s63E6H2" 0 "hypothetical protein"
"Chl_C14_00836" 2401 "s59E13H2" 0 "UvrABC system protein B"
"Chl_C14_00836" 2401 "s59E25H2" 0 "UvrABC system protein B"
"Chl_C14_00836" 2401 "s59E5H2" 0.002065715 "UvrABC system protein B"
"Chl_C14_00836" 2401 "s63E6H2" 0 "UvrABC system protein B"

"Chl_C14_00837" 2402 "s59E13H2" 0 "hypothetical protein"
"Chl_C14_00837" 2402 "s59E25H2" 0 "hypothetical protein"
"Chl_C14_00837" 2402 "s59E5H2" 0 "hypothetical protein"
"Chl_C14_00837" 2402 "s63E6H2" 0 "hypothetical protein"
"Chl_C14_00838" 2403 "s59E13H2" 0 "hypothetical protein"
"Chl_C14_00838" 2403 "s59E25H2" 0 "hypothetical protein"
"Chl_C14_00838" 2403 "s59E5H2" 0 "hypothetical protein"
"Chl_C14_00838" 2403 "s63E6H2" 0 "hypothetical protein"
"Chl_C14_00839" 2404 "s59E13H2" 0 "DNA translocase SpoIIIE"
"Chl_C14_00839" 2404 "s59E25H2" 0 "DNA translocase SpoIIIE"
"Chl_C14_00839" 2404 "s59E5H2" 0.004315623 "DNA translocase SpoIIIE"
"Chl_C14_00839" 2404 "s63E6H2" 0 "DNA translocase SpoIIIE"
"Chl_C14_00840" 2405 "s59E13H2" 0 "Ribonuclease J 1"
"Chl_C14_00840" 2405 "s59E25H2" 0 "Ribonuclease J 1"
"Chl_C14_00840" 2405 "s59E5H2" 0 "Ribonuclease J 1"
"Chl_C14_00840" 2405 "s63E6H2" 0 "Ribonuclease J 1"
"Chl_C14_00841" 2406 "s59E13H2" 0 "Uracil DNA glycosylase superfamily protein"
"Chl_C14_00841" 2406 "s59E25H2" 0 "Uracil DNA glycosylase superfamily protein"
"Chl_C14_00841" 2406 "s59E5H2" 0 "Uracil DNA glycosylase superfamily protein"
"Chl_C14_00841" 2406 "s63E6H2" 0 "Uracil DNA glycosylase superfamily protein"
"Chl_C14_00842" 2407 "s59E13H2" 0 "hypothetical protein"
"Chl_C14_00842" 2407 "s59E25H2" 0 "hypothetical protein"
"Chl_C14_00842" 2407 "s59E5H2" 0 "hypothetical protein"
"Chl_C14_00842" 2407 "s63E6H2" 0 "hypothetical protein"
"Chl_C14_00843" 2408 "s59E13H2" 0 "hypothetical protein"
"Chl_C14_00843" 2408 "s59E25H2" 0 "hypothetical protein"
"Chl_C14_00843" 2408 "s59E5H2" 0 "hypothetical protein"
"Chl_C14_00843" 2408 "s63E6H2" 0 "hypothetical protein"
"Chl_C14_00844" 2409 "s59E13H2" 0 "Phosphoribosylamine--glycine ligase"
"Chl_C14_00844" 2409 "s59E25H2" 0 "Phosphoribosylamine--glycine ligase"
"Chl_C14_00844" 2409 "s59E5H2" 0 "Phosphoribosylamine--glycine ligase"
"Chl_C14_00844" 2409 "s63E6H2" 0 "Phosphoribosylamine--glycine ligase"
"Chl_C14_00845" 2410 "s59E13H2" 0 "N5-carboxyaminoimidazole ribonucleotide mutase"
"Chl_C14_00845" 2410 "s59E25H2" 0 "N5-carboxyaminoimidazole ribonucleotide mutase"
"Chl_C14_00845" 2410 "s59E5H2" 0 "N5-carboxyaminoimidazole ribonucleotide mutase"
"Chl_C14_00845" 2410 "s63E6H2" 0 "N5-carboxyaminoimidazole ribonucleotide mutase"
"Chl_C14_00846" 2411 "s59E13H2" 0 "Adenylosuccinate lyase"
"Chl_C14_00846" 2411 "s59E25H2" 0 "Adenylosuccinate lyase"
"Chl_C14_00846" 2411 "s59E5H2" 0 "Adenylosuccinate lyase"
"Chl_C14_00846" 2411 "s63E6H2" 0.001121291 "Adenylosuccinate lyase"
"Chl_C14_00847" 2412 "s59E13H2" 0 "DNA polymerase IV"
"Chl_C14_00847" 2412 "s59E25H2" 0 "DNA polymerase IV"
"Chl_C14_00847" 2412 "s59E5H2" 0 "DNA polymerase IV"
"Chl_C14_00847" 2412 "s63E6H2" 0 "DNA polymerase IV"
"Chl_C14_00848" 2413 "s59E13H2" 0 "hypothetical protein"
"Chl_C14_00848" 2413 "s59E25H2" 0 "hypothetical protein"
"Chl_C14_00848" 2413 "s59E5H2" 0 "hypothetical protein"

"Chl_C14_00848" 2413 "s63E6H2" 0.003767893 "hypothetical protein"
"Chl_C14_00849" 2414 "s59E13H2" 0 "putative MFS-type transporter YhjX"
"Chl_C14_00849" 2414 "s59E25H2" 0 "putative MFS-type transporter YhjX"
"Chl_C14_00849" 2414 "s59E5H2" 0 "putative MFS-type transporter YhjX"
"Chl_C14_00849" 2414 "s63E6H2" 0 "putative MFS-type transporter YhjX"
"Chl_C14_00851" 2416 "s59E13H2" 0 "Orotidine 5'-phosphate decarboxylase"
"Chl_C14_00851" 2416 "s59E25H2" 0 "Orotidine 5'-phosphate decarboxylase"
"Chl_C14_00851" 2416 "s59E5H2" 0 "Orotidine 5'-phosphate decarboxylase"
"Chl_C14_00851" 2416 "s63E6H2" 0 "Orotidine 5'-phosphate decarboxylase"
"Chl_C14_00853" 2418 "s59E13H2" 0 "hypothetical protein"
"Chl_C14_00853" 2418 "s59E25H2" 0 "hypothetical protein"
"Chl_C14_00853" 2418 "s59E5H2" 0 "hypothetical protein"
"Chl_C14_00853" 2418 "s63E6H2" 0 "hypothetical protein"
"Chl_C14_00854" 2419 "s59E13H2" 0.0007232115 "hypothetical protein"
"Chl_C14_00854" 2419 "s59E25H2" 0 "hypothetical protein"
"Chl_C14_00854" 2419 "s59E5H2" 0 "hypothetical protein"
"Chl_C14_00854" 2419 "s63E6H2" 0 "hypothetical protein"
"Chl_C14_00855" 2420 "s59E13H2" 0 "hypothetical protein"
"Chl_C14_00855" 2420 "s59E25H2" 0 "hypothetical protein"
"Chl_C14_00855" 2420 "s59E5H2" 0 "hypothetical protein"
"Chl_C14_00855" 2420 "s63E6H2" 0 "hypothetical protein"
"Chl_C14_00856" 2421 "s59E13H2" 0.0639987 "IS2 transposase TnpB"
"Chl_C14_00856" 2421 "s59E25H2" 0 "IS2 transposase TnpB"
"Chl_C14_00856" 2421 "s59E5H2" 0.07573919 "IS2 transposase TnpB"
"Chl_C14_00856" 2421 "s63E6H2" 0.004914092 "IS2 transposase TnpB"
"Chl_C14_00857" 2422 "s59E13H2" 0.001501207 "Transposase"
"Chl_C14_00857" 2422 "s59E25H2" 0 "Transposase"
"Chl_C14_00857" 2422 "s59E5H2" 0 "Transposase"
"Chl_C14_00857" 2422 "s63E6H2" 0.882882 "Transposase"
"Chl_C14_00858" 2423 "s59E13H2" 0.0003622704 "hypothetical protein"
"Chl_C14_00858" 2423 "s59E25H2" 0 "hypothetical protein"
"Chl_C14_00858" 2423 "s59E5H2" 0 "hypothetical protein"
"Chl_C14_00858" 2423 "s63E6H2" 0.0268502 "hypothetical protein"
"Chl_C14_00859" 2424 "s59E13H2" 0.03018265 "30S ribosomal protein S1"
"Chl_C14_00859" 2424 "s59E25H2" 0.1158818 "30S ribosomal protein S1"
"Chl_C14_00859" 2424 "s59E5H2" 0.848429 "30S ribosomal protein S1"
"Chl_C14_00859" 2424 "s63E6H2" 0.4226453 "30S ribosomal protein S1"
"Chl_C14_00860" 2425 "s59E13H2" 0 "GIY-YIG nuclease superfamily protein"
"Chl_C14_00860" 2425 "s59E25H2" 0 "GIY-YIG nuclease superfamily protein"
"Chl_C14_00860" 2425 "s59E5H2" 0.6539846 "GIY-YIG nuclease superfamily protein"
"Chl_C14_00860" 2425 "s63E6H2" 0.3491309 "GIY-YIG nuclease superfamily protein"
"Chl_C14_00861" 2426 "s59E13H2" 0 "hypothetical protein"
"Chl_C14_00861" 2426 "s59E25H2" 0 "hypothetical protein"
"Chl_C14_00861" 2426 "s59E5H2" 0.003756377 "hypothetical protein"
"Chl_C14_00861" 2426 "s63E6H2" 0.02540451 "hypothetical protein"
"Chl_C14_00862" 2427 "s59E13H2" 0 "hypothetical protein"
"Chl_C14_00862" 2427 "s59E25H2" 0 "hypothetical protein"

"Chl_C14_00862" 2427 "s59E5H2" 0.04584284 "hypothetical protein"
"Chl_C14_00862" 2427 "s63E6H2" 0 "hypothetical protein"
"Chl_C14_00863" 2428 "s59E13H2" 0.08962062 "hypothetical protein"
"Chl_C14_00863" 2428 "s59E25H2" 0 "hypothetical protein"
"Chl_C14_00863" 2428 "s59E5H2" 0 "hypothetical protein"
"Chl_C14_00863" 2428 "s63E6H2" 0.03999187 "hypothetical protein"
"Chl_C14_00864" 2429 "s59E13H2" 0 "hypothetical protein"
"Chl_C14_00864" 2429 "s59E25H2" 0 "hypothetical protein"
"Chl_C14_00864" 2429 "s59E5H2" 0.2088071 "hypothetical protein"
"Chl_C14_00864" 2429 "s63E6H2" 0.08421842 "hypothetical protein"
"Chl_C14_00865" 2430 "s59E13H2" 0 "30S ribosomal protein S21"
"Chl_C14_00865" 2430 "s59E25H2" 0 "30S ribosomal protein S21"
"Chl_C14_00865" 2430 "s59E5H2" 0.08473632 "30S ribosomal protein S21"
"Chl_C14_00865" 2430 "s63E6H2" 0.005094219 "30S ribosomal protein S21"
"Chl_C14_00866" 2431 "s59E13H2" 0.1793452 "RNA recognition motif. (a.k.a. RRM, RBD, or RNP domain)"
"Chl_C14_00866" 2431 "s59E25H2" 0 "RNA recognition motif. (a.k.a. RRM, RBD, or RNP domain)"
"Chl_C14_00866" 2431 "s59E5H2" 0.02410568 "RNA recognition motif. (a.k.a. RRM, RBD, or RNP domain)"
"Chl_C14_00866" 2431 "s63E6H2" 0.1740346 "RNA recognition motif. (a.k.a. RRM, RBD, or RNP domain)"
"Chl_C14_00867" 2432 "s59E13H2" 0 "RNA recognition motif. (a.k.a. RRM, RBD, or RNP domain)"
"Chl_C14_00867" 2432 "s59E25H2" 0 "RNA recognition motif. (a.k.a. RRM, RBD, or RNP domain)"
"Chl_C14_00867" 2432 "s59E5H2" 0.00961797 "RNA recognition motif. (a.k.a. RRM, RBD, or RNP domain)"
"Chl_C14_00867" 2432 "s63E6H2" 0.4619145 "RNA recognition motif. (a.k.a. RRM, RBD, or RNP domain)"
"Chl_C14_00870" 2435 "s59E13H2" 0 "Negative regulator of genetic competence ClpC/MecB"
"Chl_C14_00870" 2435 "s59E25H2" 0 "Negative regulator of genetic competence ClpC/MecB"
"Chl_C14_00870" 2435 "s59E5H2" 0.004144747 "Negative regulator of genetic competence ClpC/MecB"
"Chl_C14_00870" 2435 "s63E6H2" 0 "Negative regulator of genetic competence ClpC/MecB"
"Chl_C14_00871" 2436 "s59E13H2" 0 "Biotinylated protein TB7.3"
"Chl_C14_00871" 2436 "s59E25H2" 0 "Biotinylated protein TB7.3"
"Chl_C14_00871" 2436 "s59E5H2" 0 "Biotinylated protein TB7.3"
"Chl_C14_00871" 2436 "s63E6H2" 0 "Biotinylated protein TB7.3"
"Chl_C14_00872" 2437 "s59E13H2" 0 "hypothetical protein"
"Chl_C14_00872" 2437 "s59E25H2" 0 "hypothetical protein"
"Chl_C14_00872" 2437 "s59E5H2" 0 "hypothetical protein"
"Chl_C14_00872" 2437 "s63E6H2" 0 "hypothetical protein"
"Chl_C14_00873" 2438 "s59E13H2" 0 "Glutaconyl-CoA decarboxylase subunit beta"
"Chl_C14_00873" 2438 "s59E25H2" 0 "Glutaconyl-CoA decarboxylase subunit beta"
"Chl_C14_00873" 2438 "s59E5H2" 0 "Glutaconyl-CoA decarboxylase subunit beta"
"Chl_C14_00873" 2438 "s63E6H2" 0 "Glutaconyl-CoA decarboxylase subunit beta"
"Chl_C14_00874" 2439 "s59E13H2" 0 "hypothetical protein"
"Chl_C14_00874" 2439 "s59E25H2" 0 "hypothetical protein"
"Chl_C14_00874" 2439 "s59E5H2" 0 "hypothetical protein"
"Chl_C14_00874" 2439 "s63E6H2" 0 "hypothetical protein"
"Chl_C14_00875" 2440 "s59E13H2" 0 "2-C-methyl-D-erythritol 4-phosphate cytidylyltransferase"
"Chl_C14_00875" 2440 "s59E25H2" 0 "2-C-methyl-D-erythritol 4-phosphate cytidylyltransferase"
"Chl_C14_00875" 2440 "s59E5H2" 0 "2-C-methyl-D-erythritol 4-phosphate cytidylyltransferase"
"Chl_C14_00875" 2440 "s63E6H2" 0 "2-C-methyl-D-erythritol 4-phosphate cytidylyltransferase"
"Chl_C14_00876" 2441 "s59E13H2" 0 "2-C-methyl-D-erythritol 2,4-cyclodiphosphate synthase"

"Chl_C14_00876" 2441 "s59E25H2" 0 "2-C-methyl-D-erythritol 2,4-cyclodiphosphate synthase"
"Chl_C14_00876" 2441 "s59E5H2" 0 "2-C-methyl-D-erythritol 2,4-cyclodiphosphate synthase"
"Chl_C14_00876" 2441 "s63E6H2" 0 "2-C-methyl-D-erythritol 2,4-cyclodiphosphate synthase"
"Chl_C14_00877" 2442 "s59E13H2" 0 "Serine acetyltransferase"
"Chl_C14_00877" 2442 "s59E25H2" 0 "Serine acetyltransferase"
"Chl_C14_00877" 2442 "s59E5H2" 0 "Serine acetyltransferase"
"Chl_C14_00877" 2442 "s63E6H2" 0 "Serine acetyltransferase"
"Chl_C14_00878" 2443 "s59E13H2" 0 "hypothetical protein"
"Chl_C14_00878" 2443 "s59E25H2" 0 "hypothetical protein"
"Chl_C14_00878" 2443 "s59E5H2" 0 "hypothetical protein"
"Chl_C14_00878" 2443 "s63E6H2" 0 "hypothetical protein"
"Chl_C14_00879" 2444 "s59E13H2" 0 "Cysteine--tRNA ligase"
"Chl_C14_00879" 2444 "s59E25H2" 0 "Cysteine--tRNA ligase"
"Chl_C14_00879" 2444 "s59E5H2" 0 "Cysteine--tRNA ligase"
"Chl_C14_00879" 2444 "s63E6H2" 0 "Cysteine--tRNA ligase"
"Chl_C14_00880" 2445 "s59E13H2" 0 "hypothetical protein"
"Chl_C14_00880" 2445 "s59E25H2" 0 "hypothetical protein"
"Chl_C14_00880" 2445 "s59E5H2" 0 "hypothetical protein"
"Chl_C14_00880" 2445 "s63E6H2" 0 "hypothetical protein"
"Chl_C14_00881" 2446 "s59E13H2" 0 "hypothetical protein"
"Chl_C14_00881" 2446 "s59E25H2" 0 "hypothetical protein"
"Chl_C14_00881" 2446 "s59E5H2" 0 "hypothetical protein"
"Chl_C14_00881" 2446 "s63E6H2" 0 "hypothetical protein"
"Chl_C14_00882" 2447 "s59E13H2" 0 "Chlorinase"
"Chl_C14_00882" 2447 "s59E25H2" 0 "Chlorinase"
"Chl_C14_00882" 2447 "s59E5H2" 0 "Chlorinase"
"Chl_C14_00882" 2447 "s63E6H2" 0 "Chlorinase"
"Chl_C14_00883" 2448 "s59E13H2" 0 "preprotein translocase subunit SecG"
"Chl_C14_00883" 2448 "s59E25H2" 0 "preprotein translocase subunit SecG"
"Chl_C14_00883" 2448 "s59E5H2" 0 "preprotein translocase subunit SecG"
"Chl_C14_00883" 2448 "s63E6H2" 0 "preprotein translocase subunit SecG"
"Chl_C14_00884" 2449 "s59E13H2" 0 "Aspartyl/glutamyl-tRNA(Asn/Gln) amidotransferase subunit B"
"Chl_C14_00884" 2449 "s59E25H2" 0 "Aspartyl/glutamyl-tRNA(Asn/Gln) amidotransferase subunit B"
"Chl_C14_00884" 2449 "s59E5H2" 0.001032326 "Aspartyl/glutamyl-tRNA(Asn/Gln) amidotransferase subunit B"
"Chl_C14_00884" 2449 "s63E6H2" 0 "Aspartyl/glutamyl-tRNA(Asn/Gln) amidotransferase subunit B"
"Chl_C14_00886" 2451 "s59E13H2" 0 "Glycine betaine transport system permease protein OpuAB"
"Chl_C14_00886" 2451 "s59E25H2" 0 "Glycine betaine transport system permease protein OpuAB"
"Chl_C14_00886" 2451 "s59E5H2" 0 "Glycine betaine transport system permease protein OpuAB"
"Chl_C14_00886" 2451 "s63E6H2" 0 "Glycine betaine transport system permease protein OpuAB"
"Chl_C14_00887" 2452 "s59E13H2" 0 "hypothetical protein"
"Chl_C14_00887" 2452 "s59E25H2" 0 "hypothetical protein"
"Chl_C14_00887" 2452 "s59E5H2" 0 "hypothetical protein"
"Chl_C14_00887" 2452 "s63E6H2" 0 "hypothetical protein"
"Chl_C14_00888" 2453 "s59E13H2" 0 "Macrolide export ATP-binding/permease protein MacB"
"Chl_C14_00888" 2453 "s59E25H2" 0 "Macrolide export ATP-binding/permease protein MacB"
"Chl_C14_00888" 2453 "s59E5H2" 0 "Macrolide export ATP-binding/permease protein MacB"
"Chl_C14_00888" 2453 "s63E6H2" 0 "Macrolide export ATP-binding/permease protein MacB"

"Chl_C14_00889" 2454 "s59E13H2" 0 "putative ABC transporter ATP-binding protein"
"Chl_C14_00889" 2454 "s59E25H2" 0 "putative ABC transporter ATP-binding protein"
"Chl_C14_00889" 2454 "s59E5H2" 0.002205183 "putative ABC transporter ATP-binding protein"
"Chl_C14_00889" 2454 "s63E6H2" 0 "putative ABC transporter ATP-binding protein"
"Chl_C14_00890" 2455 "s59E13H2" 0 "Macrolide export protein MacA"
"Chl_C14_00890" 2455 "s59E25H2" 0 "Macrolide export protein MacA"
"Chl_C14_00890" 2455 "s59E5H2" 0 "Macrolide export protein MacA"
"Chl_C14_00890" 2455 "s63E6H2" 0 "Macrolide export protein MacA"
"Chl_C14_00891" 2456 "s59E13H2" 0 "Signal transduction histidine-protein kinase BaeS"
"Chl_C14_00891" 2456 "s59E25H2" 0 "Signal transduction histidine-protein kinase BaeS"
"Chl_C14_00891" 2456 "s59E5H2" 0 "Signal transduction histidine-protein kinase BaeS"
"Chl_C14_00891" 2456 "s63E6H2" 0 "Signal transduction histidine-protein kinase BaeS"
"Chl_C14_00892" 2457 "s59E13H2" 0 "Transcriptional regulatory protein YycF"
"Chl_C14_00892" 2457 "s59E25H2" 0 "Transcriptional regulatory protein YycF"
"Chl_C14_00892" 2457 "s59E5H2" 0 "Transcriptional regulatory protein YycF"
"Chl_C14_00892" 2457 "s63E6H2" 0 "Transcriptional regulatory protein YycF"
"Chl_C14_00893" 2458 "s59E13H2" 0 "hypothetical protein"
"Chl_C14_00893" 2458 "s59E25H2" 0 "hypothetical protein"
"Chl_C14_00893" 2458 "s59E5H2" 0 "hypothetical protein"
"Chl_C14_00893" 2458 "s63E6H2" 0 "hypothetical protein"
"Chl_C14_00894" 2459 "s59E13H2" 0 "hypothetical protein"
"Chl_C14_00894" 2459 "s59E25H2" 0 "hypothetical protein"
"Chl_C14_00894" 2459 "s59E5H2" 0 "hypothetical protein"
"Chl_C14_00894" 2459 "s63E6H2" 0 "hypothetical protein"
"Chl_C14_00896" 2461 "s59E13H2" 0 "Putative serine protease HtrA"
"Chl_C14_00896" 2461 "s59E25H2" 0 "Putative serine protease HtrA"
"Chl_C14_00896" 2461 "s59E5H2" 0 "Putative serine protease HtrA"
"Chl_C14_00896" 2461 "s63E6H2" 0 "Putative serine protease HtrA"
"Chl_C14_00898" 2463 "s59E13H2" 0.2867935 "Holliday junction ATP-dependent DNA helicase RuvB"
"Chl_C14_00898" 2463 "s59E25H2" 0 "Holliday junction ATP-dependent DNA helicase RuvB"
"Chl_C14_00898" 2463 "s59E5H2" 0.03196174 "Holliday junction ATP-dependent DNA helicase RuvB"
"Chl_C14_00898" 2463 "s63E6H2" 0 "Holliday junction ATP-dependent DNA helicase RuvB"
"Chl_C14_00899" 2464 "s59E13H2" 0 "hypothetical protein"
"Chl_C14_00899" 2464 "s59E25H2" 0 "hypothetical protein"
"Chl_C14_00899" 2464 "s59E5H2" 0.01610577 "hypothetical protein"
"Chl_C14_00899" 2464 "s63E6H2" 0 "hypothetical protein"
"Chl_C14_00900" 2465 "s59E13H2" 0 "hypothetical protein"
"Chl_C14_00900" 2465 "s59E25H2" 0 "hypothetical protein"
"Chl_C14_00900" 2465 "s59E5H2" 0.05607323 "hypothetical protein"
"Chl_C14_00900" 2465 "s63E6H2" 0 "hypothetical protein"
"Chl_C14_00905" 2470 "s59E13H2" 0 "hypothetical protein"
"Chl_C14_00905" 2470 "s59E25H2" 0 "hypothetical protein"
"Chl_C14_00905" 2470 "s59E5H2" 0.02820209 "hypothetical protein"
"Chl_C14_00905" 2470 "s63E6H2" 0 "hypothetical protein"
"Chl_C14_00906" 2471 "s59E13H2" 0 "hypothetical protein"
"Chl_C14_00906" 2471 "s59E25H2" 0 "hypothetical protein"
"Chl_C14_00906" 2471 "s59E5H2" 0.05689469 "hypothetical protein"

"Chl_C14_00906" 2471 "s63E6H2" 0 "hypothetical protein"
"Chl_C14_00907" 2472 "s59E13H2" 0 "hypothetical protein"
"Chl_C14_00907" 2472 "s59E25H2" 0 "hypothetical protein"
"Chl_C14_00907" 2472 "s59E5H2" 0.08417331 "hypothetical protein"
"Chl_C14_00907" 2472 "s63E6H2" 0 "hypothetical protein"
"Chl_C14_00908" 2473 "s59E13H2" 0.00545048 "Beta-1,4-mannooligosaccharide phosphorylase"
"Chl_C14_00908" 2473 "s59E25H2" 0 "Beta-1,4-mannooligosaccharide phosphorylase"
"Chl_C14_00908" 2473 "s59E5H2" 0 "Beta-1,4-mannooligosaccharide phosphorylase"
"Chl_C14_00908" 2473 "s63E6H2" 0.1532386 "Beta-1,4-mannooligosaccharide phosphorylase"
"Chl_C14_00909" 2474 "s59E13H2" 0 "Zinc ribbon domain protein"
"Chl_C14_00909" 2474 "s59E25H2" 0 "Zinc ribbon domain protein"
"Chl_C14_00909" 2474 "s59E5H2" 0 "Zinc ribbon domain protein"
"Chl_C14_00909" 2474 "s63E6H2" 0 "Zinc ribbon domain protein"
"Chl_C14_00911" 2476 "s59E13H2" 0 "hypothetical protein"
"Chl_C14_00911" 2476 "s59E25H2" 0 "hypothetical protein"
"Chl_C14_00911" 2476 "s59E5H2" 0 "hypothetical protein"
"Chl_C14_00911" 2476 "s63E6H2" 0.002093978 "hypothetical protein"
"Chl_C14_00912" 2477 "s59E13H2" 0 "hypothetical protein"
"Chl_C14_00912" 2477 "s59E25H2" 0 "hypothetical protein"
"Chl_C14_00912" 2477 "s59E5H2" 0 "hypothetical protein"
"Chl_C14_00912" 2477 "s63E6H2" 0.002747513 "hypothetical protein"
"Chl_C14_00915" 2480 "s59E13H2" 0 "hypothetical protein"
"Chl_C14_00915" 2480 "s59E25H2" 0 "hypothetical protein"
"Chl_C14_00915" 2480 "s59E5H2" 0.06768398 "hypothetical protein"
"Chl_C14_00915" 2480 "s63E6H2" 0 "hypothetical protein"
"Chl_C14_00916" 2481 "s59E13H2" 0.3306347 "Helix-turn-helix domain protein"
"Chl_C14_00916" 2481 "s59E25H2" 0.2105263 "Helix-turn-helix domain protein"
"Chl_C14_00916" 2481 "s59E5H2" 0 "Helix-turn-helix domain protein"
"Chl_C14_00916" 2481 "s63E6H2" 0.1722732 "Helix-turn-helix domain protein"
"Chl_C14_00917" 2482 "s59E13H2" 0 "Sensory transduction protein regX3"
"Chl_C14_00917" 2482 "s59E25H2" 0 "Sensory transduction protein regX3"
"Chl_C14_00917" 2482 "s59E5H2" 0 "Sensory transduction protein regX3"
"Chl_C14_00917" 2482 "s63E6H2" 0 "Sensory transduction protein regX3"
"Chl_C14_00919" 2484 "s59E13H2" 0 "Response regulator ArlR"
"Chl_C14_00919" 2484 "s59E25H2" 0 "Response regulator ArlR"
"Chl_C14_00919" 2484 "s59E5H2" 0 "Response regulator ArlR"
"Chl_C14_00919" 2484 "s63E6H2" 0 "Response regulator ArlR"
"Chl_C14_00920" 2485 "s59E13H2" 0 "cell division protein FtsZ"
"Chl_C14_00920" 2485 "s59E25H2" 0 "cell division protein FtsZ"
"Chl_C14_00920" 2485 "s59E5H2" 0 "cell division protein FtsZ"
"Chl_C14_00920" 2485 "s63E6H2" 0.07701953 "cell division protein FtsZ"
"Chl_C14_00921" 2486 "s59E13H2" 0 "General stress protein CTC"
"Chl_C14_00921" 2486 "s59E25H2" 0 "General stress protein CTC"
"Chl_C14_00921" 2486 "s59E5H2" 0 "General stress protein CTC"
"Chl_C14_00921" 2486 "s63E6H2" 0 "General stress protein CTC"
"Chl_C14_00922" 2487 "s59E13H2" 0 "Cold shock-like protein CspC"
"Chl_C14_00922" 2487 "s59E25H2" 0 "Cold shock-like protein CspC"

"Chl_C14_00922" 2487 "s59E5H2" 0 "Cold shock-like protein CspC"
"Chl_C14_00922" 2487 "s63E6H2" 0 "Cold shock-like protein CspC"
"Chl_C14_00924" 2489 "s59E13H2" 0 "hypothetical protein"
"Chl_C14_00924" 2489 "s59E25H2" 0 "hypothetical protein"
"Chl_C14_00924" 2489 "s59E5H2" 0 "hypothetical protein"
"Chl_C14_00924" 2489 "s63E6H2" 0 "hypothetical protein"
"Chl_C14_00926" 2491 "s59E13H2" 0 "AsnC family protein"
"Chl_C14_00926" 2491 "s59E25H2" 0 "AsnC family protein"
"Chl_C14_00926" 2491 "s59E5H2" 0 "AsnC family protein"
"Chl_C14_00926" 2491 "s63E6H2" 0 "AsnC family protein"
"Chl_C14_00928" 2493 "s59E13H2" 0 "Methyltransferase domain protein"
"Chl_C14_00928" 2493 "s59E25H2" 0 "Methyltransferase domain protein"
"Chl_C14_00928" 2493 "s59E5H2" 0 "Methyltransferase domain protein"
"Chl_C14_00928" 2493 "s63E6H2" 0 "Methyltransferase domain protein"
"Chl_C14_00929" 2494 "s59E13H2" 0 "Trimethylamine corrinoid protein"
"Chl_C14_00929" 2494 "s59E25H2" 0 "Trimethylamine corrinoid protein"
"Chl_C14_00929" 2494 "s59E5H2" 0 "Trimethylamine corrinoid protein"
"Chl_C14_00929" 2494 "s63E6H2" 0 "Trimethylamine corrinoid protein"
"Chl_C14_00930" 2495 "s59E13H2" 0 "Trimethylamine methyltransferase MttB"
"Chl_C14_00930" 2495 "s59E25H2" 0 "Trimethylamine methyltransferase MttB"
"Chl_C14_00930" 2495 "s59E5H2" 0 "Trimethylamine methyltransferase MttB"
"Chl_C14_00930" 2495 "s63E6H2" 0 "Trimethylamine methyltransferase MttB"
"Chl_C14_00931" 2496 "s59E13H2" 0 "5-methyltetrahydrofolate:corrinoid/iron-sulfur protein co-methyltransferase"
"Chl_C14_00931" 2496 "s59E25H2" 0 "5-methyltetrahydrofolate:corrinoid/iron-sulfur protein co-methyltransferase"
"Chl_C14_00931" 2496 "s59E5H2" 0 "5-methyltetrahydrofolate:corrinoid/iron-sulfur protein co-methyltransferase"
"Chl_C14_00931" 2496 "s63E6H2" 0 "5-methyltetrahydrofolate:corrinoid/iron-sulfur protein co-methyltransferase"
"Chl_C14_00932" 2497 "s59E13H2" 0 "Trimethylamine methyltransferase MttB"
"Chl_C14_00932" 2497 "s59E25H2" 0 "Trimethylamine methyltransferase MttB"
"Chl_C14_00932" 2497 "s59E5H2" 0 "Trimethylamine methyltransferase MttB"
"Chl_C14_00932" 2497 "s63E6H2" 0 "Trimethylamine methyltransferase MttB"
"Chl_C14_00933" 2498 "s59E13H2" 0 "Vitamin B12 dependent methionine synthase, activation domain"
"Chl_C14_00933" 2498 "s59E25H2" 0 "Vitamin B12 dependent methionine synthase, activation domain"
"Chl_C14_00933" 2498 "s59E5H2" 0 "Vitamin B12 dependent methionine synthase, activation domain"
"Chl_C14_00933" 2498 "s63E6H2" 0 "Vitamin B12 dependent methionine synthase, activation domain"
"Chl_C14_00934" 2499 "s59E13H2" 0 "Response regulator protein VraR"
"Chl_C14_00934" 2499 "s59E25H2" 0 "Response regulator protein VraR"
"Chl_C14_00934" 2499 "s59E5H2" 0 "Response regulator protein VraR"
"Chl_C14_00934" 2499 "s63E6H2" 0 "Response regulator protein VraR"
"Chl_C14_00936" 2501 "s59E13H2" 0 "Polyketide cyclase / dehydrase and lipid transport"
"Chl_C14_00936" 2501 "s59E25H2" 0 "Polyketide cyclase / dehydrase and lipid transport"
"Chl_C14_00936" 2501 "s59E5H2" 0 "Polyketide cyclase / dehydrase and lipid transport"
"Chl_C14_00936" 2501 "s63E6H2" 0 "Polyketide cyclase / dehydrase and lipid transport"
"Chl_C14_00939" 2504 "s59E13H2" 0 "RNA polymerase sigma factor SigA"
"Chl_C14_00939" 2504 "s59E25H2" 0 "RNA polymerase sigma factor SigA"
"Chl_C14_00939" 2504 "s59E5H2" 0 "RNA polymerase sigma factor SigA"
"Chl_C14_00939" 2504 "s63E6H2" 0 "RNA polymerase sigma factor SigA"
"Chl_C14_00942" 2507 "s59E13H2" 0 "Iron-sulfur cluster insertion protein ErpA"

"Chl_C14_00942" 2507 "s59E25H2" 0 "Iron-sulfur cluster insertion protein ErpA"
"Chl_C14_00942" 2507 "s59E5H2" 0 "Iron-sulfur cluster insertion protein ErpA"
"Chl_C14_00942" 2507 "s63E6H2" 0 "Iron-sulfur cluster insertion protein ErpA"
"Chl_C14_00946" 2511 "s59E13H2" 0 "hypothetical protein"
"Chl_C14_00946" 2511 "s59E25H2" 0 "hypothetical protein"
"Chl_C14_00946" 2511 "s59E5H2" 0 "hypothetical protein"
"Chl_C14_00946" 2511 "s63E6H2" 0 "hypothetical protein"
"Chl_C14_00947" 2512 "s59E13H2" 0 "IPT/TIG domain protein"
"Chl_C14_00947" 2512 "s59E25H2" 0 "IPT/TIG domain protein"
"Chl_C14_00947" 2512 "s59E5H2" 0 "IPT/TIG domain protein"
"Chl_C14_00947" 2512 "s63E6H2" 0 "IPT/TIG domain protein"
"Chl_C14_00948" 2513 "s59E13H2" 0 "hypothetical protein"
"Chl_C14_00948" 2513 "s59E25H2" 0 "hypothetical protein"
"Chl_C14_00948" 2513 "s59E5H2" 0 "hypothetical protein"
"Chl_C14_00948" 2513 "s63E6H2" 0 "hypothetical protein"
"Chl_C14_00949" 2514 "s59E13H2" 0 "hypothetical protein"
"Chl_C14_00949" 2514 "s59E25H2" 0 "hypothetical protein"
"Chl_C14_00949" 2514 "s59E5H2" 0 "hypothetical protein"
"Chl_C14_00949" 2514 "s63E6H2" 0 "hypothetical protein"
"Chl_C14_00951" 2516 "s59E13H2" 0 "hypothetical protein"
"Chl_C14_00951" 2516 "s59E25H2" 0 "hypothetical protein"
"Chl_C14_00951" 2516 "s59E5H2" 0 "hypothetical protein"
"Chl_C14_00951" 2516 "s63E6H2" 0 "hypothetical protein"
"Chl_C14_00956" 2521 "s59E13H2" 0 "Spore photoproduct lyase"
"Chl_C14_00956" 2521 "s59E25H2" 0 "Spore photoproduct lyase"
"Chl_C14_00956" 2521 "s59E5H2" 0 "Spore photoproduct lyase"
"Chl_C14_00956" 2521 "s63E6H2" 0 "Spore photoproduct lyase"
"Chl_C14_00957" 2522 "s59E13H2" 0 "hypothetical protein"
"Chl_C14_00957" 2522 "s59E25H2" 0 "hypothetical protein"
"Chl_C14_00957" 2522 "s59E5H2" 0.008387889 "hypothetical protein"
"Chl_C14_00957" 2522 "s63E6H2" 0 "hypothetical protein"
"Chl_C14_00958" 2523 "s59E13H2" 0 "putative glycosyl hydrolase/MT2062"
"Chl_C14_00958" 2523 "s59E25H2" 0 "putative glycosyl hydrolase/MT2062"
"Chl_C14_00958" 2523 "s59E5H2" 0 "putative glycosyl hydrolase/MT2062"
"Chl_C14_00958" 2523 "s63E6H2" 0 "putative glycosyl hydrolase/MT2062"
"Chl_C14_00959" 2524 "s59E13H2" 0 "Putative prophage phiRv2 integrase"
"Chl_C14_00959" 2524 "s59E25H2" 0 "Putative prophage phiRv2 integrase"
"Chl_C14_00959" 2524 "s59E5H2" 0 "Putative prophage phiRv2 integrase"
"Chl_C14_00959" 2524 "s63E6H2" 0 "Putative prophage phiRv2 integrase"
"Chl_C14_00962" 2527 "s59E13H2" 0 "Tyrosine recombinase XerD"
"Chl_C14_00962" 2527 "s59E25H2" 0 "Tyrosine recombinase XerD"
"Chl_C14_00962" 2527 "s59E5H2" 0 "Tyrosine recombinase XerD"
"Chl_C14_00962" 2527 "s63E6H2" 0 "Tyrosine recombinase XerD"
"Chl_C14_00964" 2529 "s59E13H2" 0 "Ribosome-binding ATPase YchF"
"Chl_C14_00964" 2529 "s59E25H2" 0 "Ribosome-binding ATPase YchF"
"Chl_C14_00964" 2529 "s59E5H2" 0 "Ribosome-binding ATPase YchF"
"Chl_C14_00964" 2529 "s63E6H2" 0 "Ribosome-binding ATPase YchF"

"Chl_C14_00966" 2531 "s59E13H2" 0 "DNA polymerase III subunit delta"
"Chl_C14_00966" 2531 "s59E25H2" 0 "DNA polymerase III subunit delta"
"Chl_C14_00966" 2531 "s59E5H2" 0 "DNA polymerase III subunit delta"
"Chl_C14_00966" 2531 "s63E6H2" 0 "DNA polymerase III subunit delta"
"Chl_C14_00967" 2532 "s59E13H2" 0 "Bifunctional trehalose-6-phosphate synthase/phosphatase"
"Chl_C14_00967" 2532 "s59E25H2" 0 "Bifunctional trehalose-6-phosphate synthase/phosphatase"
"Chl_C14_00967" 2532 "s59E5H2" 0 "Bifunctional trehalose-6-phosphate synthase/phosphatase"
"Chl_C14_00967" 2532 "s63E6H2" 0 "Bifunctional trehalose-6-phosphate synthase/phosphatase"
"Chl_C14_00968" 2533 "s59E13H2" 0 "Trehalose-phosphate phosphatase"
"Chl_C14_00968" 2533 "s59E25H2" 0 "Trehalose-phosphate phosphatase"
"Chl_C14_00968" 2533 "s59E5H2" 0 "Trehalose-phosphate phosphatase"
"Chl_C14_00968" 2533 "s63E6H2" 0 "Trehalose-phosphate phosphatase"
"Chl_C14_00969" 2534 "s59E13H2" 0 "Glycosyl hydrolase family 57"
"Chl_C14_00969" 2534 "s59E25H2" 0 "Glycosyl hydrolase family 57"
"Chl_C14_00969" 2534 "s59E5H2" 0 "Glycosyl hydrolase family 57"
"Chl_C14_00969" 2534 "s63E6H2" 0 "Glycosyl hydrolase family 57"
"Chl_C14_00970" 2535 "s59E13H2" 0 "hypothetical protein"
"Chl_C14_00970" 2535 "s59E25H2" 0 "hypothetical protein"
"Chl_C14_00970" 2535 "s59E5H2" 0 "hypothetical protein"
"Chl_C14_00970" 2535 "s63E6H2" 0 "hypothetical protein"
"Chl_C14_00971" 2536 "s59E13H2" 0 "putative MscS family protein YkuT"
"Chl_C14_00971" 2536 "s59E25H2" 0 "putative MscS family protein YkuT"
"Chl_C14_00971" 2536 "s59E5H2" 0 "putative MscS family protein YkuT"
"Chl_C14_00971" 2536 "s63E6H2" 0 "putative MscS family protein YkuT"
"Chl_C14_00972" 2537 "s59E13H2" 0 "Carbonic anhydrase 2"
"Chl_C14_00972" 2537 "s59E25H2" 0 "Carbonic anhydrase 2"
"Chl_C14_00972" 2537 "s59E5H2" 0.009965276 "Carbonic anhydrase 2"
"Chl_C14_00972" 2537 "s63E6H2" 0 "Carbonic anhydrase 2"
"Chl_C14_00973" 2538 "s59E13H2" 0 "Magnesium transport protein CorA"
"Chl_C14_00973" 2538 "s59E25H2" 0 "Magnesium transport protein CorA"
"Chl_C14_00973" 2538 "s59E5H2" 0.04732976 "Magnesium transport protein CorA"
"Chl_C14_00973" 2538 "s63E6H2" 0 "Magnesium transport protein CorA"
"Chl_C14_00974" 2539 "s59E13H2" 0 "NAD(P)H dehydrogenase (quinone)"
"Chl_C14_00974" 2539 "s59E25H2" 0 "NAD(P)H dehydrogenase (quinone)"
"Chl_C14_00974" 2539 "s59E5H2" 0 "NAD(P)H dehydrogenase (quinone)"
"Chl_C14_00974" 2539 "s63E6H2" 0 "NAD(P)H dehydrogenase (quinone)"
"Chl_C14_00975" 2540 "s59E13H2" 0 "hypothetical protein"
"Chl_C14_00975" 2540 "s59E25H2" 0 "hypothetical protein"
"Chl_C14_00975" 2540 "s59E5H2" 0 "hypothetical protein"
"Chl_C14_00975" 2540 "s63E6H2" 0 "hypothetical protein"
"Chl_C14_00977" 2542 "s59E13H2" 0 "Desulforedoxin"
"Chl_C14_00977" 2542 "s59E25H2" 0 "Desulforedoxin"
"Chl_C14_00977" 2542 "s59E5H2" 0 "Desulforedoxin"
"Chl_C14_00977" 2542 "s63E6H2" 0 "Desulforedoxin"
"Chl_C14_00978" 2543 "s59E13H2" 0 "hypothetical protein"
"Chl_C14_00978" 2543 "s59E25H2" 0 "hypothetical protein"
"Chl_C14_00978" 2543 "s59E5H2" 0 "hypothetical protein"

"Chl_C14_00978" 2543 "s63E6H2" 0 "hypothetical protein"
"Chl_C14_00979" 2544 "s59E13H2" 0 "Succinyl-CoA ligase [ADP-forming] subunit alpha"
"Chl_C14_00979" 2544 "s59E25H2" 0 "Succinyl-CoA ligase [ADP-forming] subunit alpha"
"Chl_C14_00979" 2544 "s59E5H2" 0 "Succinyl-CoA ligase [ADP-forming] subunit alpha"
"Chl_C14_00979" 2544 "s63E6H2" 0 "Succinyl-CoA ligase [ADP-forming] subunit alpha"
"Chl_C14_00980" 2545 "s59E13H2" 0.0008760458 "Alpha-1,4-glucan:maltose-1-phosphate maltosyltransferase 1"
"Chl_C14_00980" 2545 "s59E25H2" 0 "Alpha-1,4-glucan:maltose-1-phosphate maltosyltransferase 1"
"Chl_C14_00980" 2545 "s59E5H2" 0 "Alpha-1,4-glucan:maltose-1-phosphate maltosyltransferase 1"
"Chl_C14_00980" 2545 "s63E6H2" 0 "Alpha-1,4-glucan:maltose-1-phosphate maltosyltransferase 1"
"Chl_C14_00981" 2546 "s59E13H2" 0 "Trehalose synthase/amylase TreS"
"Chl_C14_00981" 2546 "s59E25H2" 0 "Trehalose synthase/amylase TreS"
"Chl_C14_00981" 2546 "s59E5H2" 0 "Trehalose synthase/amylase TreS"
"Chl_C14_00981" 2546 "s63E6H2" 0 "Trehalose synthase/amylase TreS"
"Chl_C14_00982" 2547 "s59E13H2" 0 "4-alpha-glucanotransferase"
"Chl_C14_00982" 2547 "s59E25H2" 0 "4-alpha-glucanotransferase"
"Chl_C14_00982" 2547 "s59E5H2" 0 "4-alpha-glucanotransferase"
"Chl_C14_00982" 2547 "s63E6H2" 0 "4-alpha-glucanotransferase"
"Chl_C14_00983" 2548 "s59E13H2" 0 "1,4-alpha-glucan branching enzyme GlgB"
"Chl_C14_00983" 2548 "s59E25H2" 0 "1,4-alpha-glucan branching enzyme GlgB"
"Chl_C14_00983" 2548 "s59E5H2" 0 "1,4-alpha-glucan branching enzyme GlgB"
"Chl_C14_00983" 2548 "s63E6H2" 0.000357243 "1,4-alpha-glucan branching enzyme GlgB"
"Chl_C14_00984" 2549 "s59E13H2" 0 "Malto-oligosyltrehalose trehalohydrolase"
"Chl_C14_00984" 2549 "s59E25H2" 0 "Malto-oligosyltrehalose trehalohydrolase"
"Chl_C14_00984" 2549 "s59E5H2" 0 "Malto-oligosyltrehalose trehalohydrolase"
"Chl_C14_00984" 2549 "s63E6H2" 0 "Malto-oligosyltrehalose trehalohydrolase"
"Chl_C14_00985" 2550 "s59E13H2" 0 "Glycosyl hydrolase family 57"
"Chl_C14_00985" 2550 "s59E25H2" 0 "Glycosyl hydrolase family 57"
"Chl_C14_00985" 2550 "s59E5H2" 0 "Glycosyl hydrolase family 57"
"Chl_C14_00985" 2550 "s63E6H2" 0 "Glycosyl hydrolase family 57"
"Chl_C14_00986" 2551 "s59E13H2" 0 "Maltooligosyl trehalose synthase"
"Chl_C14_00986" 2551 "s59E25H2" 0 "Maltooligosyl trehalose synthase"
"Chl_C14_00986" 2551 "s59E5H2" 0 "Maltooligosyl trehalose synthase"
"Chl_C14_00986" 2551 "s63E6H2" 0 "Maltooligosyl trehalose synthase"
"Chl_C14_00987" 2552 "s59E13H2" 0 "pH-gated potassium channel KcsA"
"Chl_C14_00987" 2552 "s59E25H2" 0 "pH-gated potassium channel KcsA"
"Chl_C14_00987" 2552 "s59E5H2" 0 "pH-gated potassium channel KcsA"
"Chl_C14_00987" 2552 "s63E6H2" 0 "pH-gated potassium channel KcsA"
"Chl_C14_00988" 2553 "s59E13H2" 0 "Maltodextrin phosphorylase"
"Chl_C14_00988" 2553 "s59E25H2" 0 "Maltodextrin phosphorylase"
"Chl_C14_00988" 2553 "s59E5H2" 0 "Maltodextrin phosphorylase"
"Chl_C14_00988" 2553 "s63E6H2" 0 "Maltodextrin phosphorylase"
"Chl_C14_00990" 2555 "s59E13H2" 0 "Lactate-binding periplasmic protein precursor"
"Chl_C14_00990" 2555 "s59E25H2" 0 "Lactate-binding periplasmic protein precursor"
"Chl_C14_00990" 2555 "s59E5H2" 0 "Lactate-binding periplasmic protein precursor"
"Chl_C14_00990" 2555 "s63E6H2" 0 "Lactate-binding periplasmic protein precursor"
"Chl_C14_00991" 2556 "s59E13H2" 0 "Tripartite ATP-independent periplasmic transporters, DctQ component"
"Chl_C14_00991" 2556 "s59E25H2" 0 "Tripartite ATP-independent periplasmic transporters, DctQ component"

"Chl_C14_00991" 2556 "s59E5H2" 0 "Tripartite ATP-independent periplasmic transporters, DctQ component"
"Chl_C14_00991" 2556 "s63E6H2" 0 "Tripartite ATP-independent periplasmic transporters, DctQ component"
"Chl_C14_00992" 2557 "s59E13H2" 0 "Sialic acid TRAP transporter permease protein SiaT"
"Chl_C14_00992" 2557 "s59E25H2" 0 "Sialic acid TRAP transporter permease protein SiaT"
"Chl_C14_00992" 2557 "s59E5H2" 0 "Sialic acid TRAP transporter permease protein SiaT"
"Chl_C14_00992" 2557 "s63E6H2" 0 "Sialic acid TRAP transporter permease protein SiaT"
"Chl_C14_00993" 2558 "s59E13H2" 0 "Aldehyde-alcohol dehydrogenase"
"Chl_C14_00993" 2558 "s59E25H2" 0 "Aldehyde-alcohol dehydrogenase"
"Chl_C14_00993" 2558 "s59E5H2" 0 "Aldehyde-alcohol dehydrogenase"
"Chl_C14_00993" 2558 "s63E6H2" 0 "Aldehyde-alcohol dehydrogenase"
"Chl_C14_00994" 2559 "s59E13H2" 0 "Tungsten-containing aldehyde ferredoxin oxidoreductase"
"Chl_C14_00994" 2559 "s59E25H2" 0 "Tungsten-containing aldehyde ferredoxin oxidoreductase"
"Chl_C14_00994" 2559 "s59E5H2" 0 "Tungsten-containing aldehyde ferredoxin oxidoreductase"
"Chl_C14_00994" 2559 "s63E6H2" 0 "Tungsten-containing aldehyde ferredoxin oxidoreductase"
"Chl_C14_00995" 2560 "s59E13H2" 0 "2-dehydro-3-deoxy-D-gluconate 5-dehydrogenase"
"Chl_C14_00995" 2560 "s59E25H2" 0 "2-dehydro-3-deoxy-D-gluconate 5-dehydrogenase"
"Chl_C14_00995" 2560 "s59E5H2" 0 "2-dehydro-3-deoxy-D-gluconate 5-dehydrogenase"
"Chl_C14_00995" 2560 "s63E6H2" 0 "2-dehydro-3-deoxy-D-gluconate 5-dehydrogenase"
"Chl_C14_00996" 2561 "s59E13H2" 0 "NADH oxidase"
"Chl_C14_00996" 2561 "s59E25H2" 0 "NADH oxidase"
"Chl_C14_00996" 2561 "s59E5H2" 0 "NADH oxidase"
"Chl_C14_00996" 2561 "s63E6H2" 0 "NADH oxidase"
"Chl_C14_00997" 2562 "s59E13H2" 0 "2-isopropylmalate synthase"
"Chl_C14_00997" 2562 "s59E25H2" 0 "2-isopropylmalate synthase"
"Chl_C14_00997" 2562 "s59E5H2" 0 "2-isopropylmalate synthase"
"Chl_C14_00997" 2562 "s63E6H2" 0 "2-isopropylmalate synthase"
"Chl_C14_00998" 2563 "s59E13H2" 0 "Tungsten-containing aldehyde ferredoxin oxidoreductase"
"Chl_C14_00998" 2563 "s59E25H2" 0 "Tungsten-containing aldehyde ferredoxin oxidoreductase"
"Chl_C14_00998" 2563 "s59E5H2" 0 "Tungsten-containing aldehyde ferredoxin oxidoreductase"
"Chl_C14_00998" 2563 "s63E6H2" 0 "Tungsten-containing aldehyde ferredoxin oxidoreductase"
"Chl_C14_00999" 2564 "s59E13H2" 0 "2,6-dihydropseudooxynicotine hydrolase"
"Chl_C14_00999" 2564 "s59E25H2" 0 "2,6-dihydropseudooxynicotine hydrolase"
"Chl_C14_00999" 2564 "s59E5H2" 0 "2,6-dihydropseudooxynicotine hydrolase"
"Chl_C14_00999" 2564 "s63E6H2" 0 "2,6-dihydropseudooxynicotine hydrolase"
"Chl_C14_01000" 2565 "s59E13H2" 0 "Sialic acid TRAP transporter permease protein SiaT"
"Chl_C14_01000" 2565 "s59E25H2" 0 "Sialic acid TRAP transporter permease protein SiaT"
"Chl_C14_01000" 2565 "s59E5H2" 0 "Sialic acid TRAP transporter permease protein SiaT"
"Chl_C14_01000" 2565 "s63E6H2" 0 "Sialic acid TRAP transporter permease protein SiaT"
"Chl_C14_01001" 2566 "s59E13H2" 0 "Tripartite ATP-independent periplasmic transporters, DctQ component"
"Chl_C14_01001" 2566 "s59E25H2" 0 "Tripartite ATP-independent periplasmic transporters, DctQ component"
"Chl_C14_01001" 2566 "s59E5H2" 0 "Tripartite ATP-independent periplasmic transporters, DctQ component"
"Chl_C14_01001" 2566 "s63E6H2" 0 "Tripartite ATP-independent periplasmic transporters, DctQ component"
"Chl_C14_01002" 2567 "s59E13H2" 0 "Lactate-binding periplasmic protein precursor"
"Chl_C14_01002" 2567 "s59E25H2" 0 "Lactate-binding periplasmic protein precursor"
"Chl_C14_01002" 2567 "s59E5H2" 0 "Lactate-binding periplasmic protein precursor"
"Chl_C14_01002" 2567 "s63E6H2" 0 "Lactate-binding periplasmic protein precursor"
"Chl_C14_01004" 2569 "s59E13H2" 0 "hypothetical protein"

"Chl_C14_01004" 2569 "s59E25H2" 0 "hypothetical protein"
"Chl_C14_01004" 2569 "s59E5H2" 0 "hypothetical protein"
"Chl_C14_01004" 2569 "s63E6H2" 0 "hypothetical protein"
"Chl_C14_01005" 2570 "s59E13H2" 0 "D-inositol 3-phosphate glycosyltransferase"
"Chl_C14_01005" 2570 "s59E25H2" 0 "D-inositol 3-phosphate glycosyltransferase"
"Chl_C14_01005" 2570 "s59E5H2" 0 "D-inositol 3-phosphate glycosyltransferase"
"Chl_C14_01005" 2570 "s63E6H2" 0 "D-inositol 3-phosphate glycosyltransferase"
"Chl_C14_01006" 2571 "s59E13H2" 0 "putative acetyltransferase YhhY"
"Chl_C14_01006" 2571 "s59E25H2" 0 "putative acetyltransferase YhhY"
"Chl_C14_01006" 2571 "s59E5H2" 0 "putative acetyltransferase YhhY"
"Chl_C14_01006" 2571 "s63E6H2" 0 "putative acetyltransferase YhhY"
"Chl_C14_01007" 2572 "s59E13H2" 0 "hypothetical protein"
"Chl_C14_01007" 2572 "s59E25H2" 0 "hypothetical protein"
"Chl_C14_01007" 2572 "s59E5H2" 0 "hypothetical protein"
"Chl_C14_01007" 2572 "s63E6H2" 0 "hypothetical protein"
"Chl_C14_01008" 2573 "s59E13H2" 0 "Alpha-D-kanosaminyltransferase"
"Chl_C14_01008" 2573 "s59E25H2" 0 "Alpha-D-kanosaminyltransferase"
"Chl_C14_01008" 2573 "s59E5H2" 0 "Alpha-D-kanosaminyltransferase"
"Chl_C14_01008" 2573 "s63E6H2" 0 "Alpha-D-kanosaminyltransferase"
"Chl_C14_01010" 2575 "s59E13H2" 0 "Dimethyl sulfoxide reductase DmsA precursor"
"Chl_C14_01010" 2575 "s59E25H2" 0 "Dimethyl sulfoxide reductase DmsA precursor"
"Chl_C14_01010" 2575 "s59E5H2" 0 "Dimethyl sulfoxide reductase DmsA precursor"
"Chl_C14_01010" 2575 "s63E6H2" 0 "Dimethyl sulfoxide reductase DmsA precursor"
"Chl_C14_01011" 2576 "s59E13H2" 0 "Sulfide dehydrogenase subunit alpha precursor"
"Chl_C14_01011" 2576 "s59E25H2" 0 "Sulfide dehydrogenase subunit alpha precursor"
"Chl_C14_01011" 2576 "s59E5H2" 0.01101283 "Sulfide dehydrogenase subunit alpha precursor"
"Chl_C14_01011" 2576 "s63E6H2" 0 "Sulfide dehydrogenase subunit alpha precursor"
"Chl_C14_01012" 2577 "s59E13H2" 0 "Branched-chain-amino-acid aminotransferase"
"Chl_C14_01012" 2577 "s59E25H2" 0 "Branched-chain-amino-acid aminotransferase"
"Chl_C14_01012" 2577 "s59E5H2" 0 "Branched-chain-amino-acid aminotransferase"
"Chl_C14_01012" 2577 "s63E6H2" 0 "Branched-chain-amino-acid aminotransferase"
"Chl_C14_01013" 2578 "s59E13H2" 0 "Glycerol-3-phosphate acyltransferase"
"Chl_C14_01013" 2578 "s59E25H2" 0 "Glycerol-3-phosphate acyltransferase"
"Chl_C14_01013" 2578 "s59E5H2" 0 "Glycerol-3-phosphate acyltransferase"
"Chl_C14_01013" 2578 "s63E6H2" 0 "Glycerol-3-phosphate acyltransferase"
"Chl_C14_01014" 2579 "s59E13H2" 0 "L-fuculose phosphate aldolase"
"Chl_C14_01014" 2579 "s59E25H2" 0 "L-fuculose phosphate aldolase"
"Chl_C14_01014" 2579 "s59E5H2" 0 "L-fuculose phosphate aldolase"
"Chl_C14_01014" 2579 "s63E6H2" 0 "L-fuculose phosphate aldolase"
"Chl_C14_01015" 2580 "s59E13H2" 0 "LexA repressor"
"Chl_C14_01015" 2580 "s59E25H2" 0 "LexA repressor"
"Chl_C14_01015" 2580 "s59E5H2" 0 "LexA repressor"
"Chl_C14_01015" 2580 "s63E6H2" 0 "LexA repressor"
"Chl_C14_01016" 2581 "s59E13H2" 0 "30S ribosomal protein S20"
"Chl_C14_01016" 2581 "s59E25H2" 0 "30S ribosomal protein S20"
"Chl_C14_01016" 2581 "s59E5H2" 0 "30S ribosomal protein S20"
"Chl_C14_01016" 2581 "s63E6H2" 0 "30S ribosomal protein S20"

"Chl_C14_01017" 2582 "s59E13H2" 0 "GTP pyrophosphokinase"
"Chl_C14_01017" 2582 "s59E25H2" 0 "GTP pyrophosphokinase"
"Chl_C14_01017" 2582 "s59E5H2" 0 "GTP pyrophosphokinase"
"Chl_C14_01017" 2582 "s63E6H2" 0 "GTP pyrophosphokinase"
"Chl_C14_01018" 2583 "s59E13H2" 0 "Histidine--tRNA ligase"
"Chl_C14_01018" 2583 "s59E25H2" 0 "Histidine--tRNA ligase"
"Chl_C14_01018" 2583 "s59E5H2" 0 "Histidine--tRNA ligase"
"Chl_C14_01018" 2583 "s63E6H2" 0 "Histidine--tRNA ligase"
"Chl_C14_01019" 2584 "s59E13H2" 0 "30S ribosomal protein S21"
"Chl_C14_01019" 2584 "s59E25H2" 0 "30S ribosomal protein S21"
"Chl_C14_01019" 2584 "s59E5H2" 0 "30S ribosomal protein S21"
"Chl_C14_01019" 2584 "s63E6H2" 0 "30S ribosomal protein S21"
"Chl_C14_01020" 2585 "s59E13H2" 0 "Yqey-like protein"
"Chl_C14_01020" 2585 "s59E25H2" 0 "Yqey-like protein"
"Chl_C14_01020" 2585 "s59E5H2" 0 "Yqey-like protein"
"Chl_C14_01020" 2585 "s63E6H2" 0 "Yqey-like protein"
"Chl_C14_01021" 2586 "s59E13H2" 0 "Phosphoribosylformylglycinamidine synthase 2"
"Chl_C14_01021" 2586 "s59E25H2" 0 "Phosphoribosylformylglycinamidine synthase 2"
"Chl_C14_01021" 2586 "s59E5H2" 0 "Phosphoribosylformylglycinamidine synthase 2"
"Chl_C14_01021" 2586 "s63E6H2" 0 "Phosphoribosylformylglycinamidine synthase 2"
"Chl_C14_01022" 2587 "s59E13H2" 0 "Radical SAM superfamily protein"
"Chl_C14_01022" 2587 "s59E25H2" 0 "Radical SAM superfamily protein"
"Chl_C14_01022" 2587 "s59E5H2" 0 "Radical SAM superfamily protein"
"Chl_C14_01022" 2587 "s63E6H2" 0 "Radical SAM superfamily protein"
"Chl_C14_01023" 2588 "s59E13H2" 0 "NYN domain protein"
"Chl_C14_01023" 2588 "s59E25H2" 0 "NYN domain protein"
"Chl_C14_01023" 2588 "s59E5H2" 0 "NYN domain protein"
"Chl_C14_01023" 2588 "s63E6H2" 0 "NYN domain protein"
"Chl_C14_01024" 2589 "s59E13H2" 0 "Ribonucleoside-diphosphate reductase NrdZ"
"Chl_C14_01024" 2589 "s59E25H2" 0 "Ribonucleoside-diphosphate reductase NrdZ"
"Chl_C14_01024" 2589 "s59E5H2" 0 "Ribonucleoside-diphosphate reductase NrdZ"
"Chl_C14_01024" 2589 "s63E6H2" 0 "Ribonucleoside-diphosphate reductase NrdZ"
"Chl_C14_01025" 2590 "s59E13H2" 0 "Transcriptional repressor NrdR"
"Chl_C14_01025" 2590 "s59E25H2" 0 "Transcriptional repressor NrdR"
"Chl_C14_01025" 2590 "s59E5H2" 0 "Transcriptional repressor NrdR"
"Chl_C14_01025" 2590 "s63E6H2" 0.001681016 "Transcriptional repressor NrdR"
"Chl_C14_01026" 2591 "s59E13H2" 0 "Transaldolase"
"Chl_C14_01026" 2591 "s59E25H2" 0 "Transaldolase"
"Chl_C14_01026" 2591 "s59E5H2" 0 "Transaldolase"
"Chl_C14_01026" 2591 "s63E6H2" 0 "Transaldolase"
"Chl_C14_01027" 2592 "s59E13H2" 0 "CTP synthase"
"Chl_C14_01027" 2592 "s59E25H2" 0 "CTP synthase"
"Chl_C14_01027" 2592 "s59E5H2" 0.004977547 "CTP synthase"
"Chl_C14_01027" 2592 "s63E6H2" 0.1992259 "CTP synthase"
"Chl_C14_01028" 2593 "s59E13H2" 0 "putative NUDIX hydrolase"
"Chl_C14_01028" 2593 "s59E25H2" 0 "putative NUDIX hydrolase"
"Chl_C14_01028" 2593 "s59E5H2" 0 "putative NUDIX hydrolase"

"Chl_C14_01028" 2593 "s63E6H2" 0 "putative NUDIX hydrolase"
"Chl_C14_01029" 2594 "s59E13H2" 0.0003916246 "ATP-dependent Clp protease ATP-binding subunit ClpC"
"Chl_C14_01029" 2594 "s59E25H2" 0 "ATP-dependent Clp protease ATP-binding subunit ClpC"
"Chl_C14_01029" 2594 "s59E5H2" 0 "ATP-dependent Clp protease ATP-binding subunit ClpC"
"Chl_C14_01029" 2594 "s63E6H2" 0.000307561 "ATP-dependent Clp protease ATP-binding subunit ClpC"
"Chl_C14_01030" 2595 "s59E13H2" 0 "hypothetical protein"
"Chl_C14_01030" 2595 "s59E25H2" 0 "hypothetical protein"
"Chl_C14_01030" 2595 "s59E5H2" 0 "hypothetical protein"
"Chl_C14_01030" 2595 "s63E6H2" 0 "hypothetical protein"
"Chl_C14_01031" 2596 "s59E13H2" 0.0008963393 "putative peptidyl-prolyl cis-trans isomerase"
"Chl_C14_01031" 2596 "s59E25H2" 0 "putative peptidyl-prolyl cis-trans isomerase"
"Chl_C14_01031" 2596 "s59E5H2" 0 "putative peptidyl-prolyl cis-trans isomerase"
"Chl_C14_01031" 2596 "s63E6H2" 0 "putative peptidyl-prolyl cis-trans isomerase"
"Chl_C14_01033" 2598 "s59E13H2" 0 "NADH dehydrogenase"
"Chl_C14_01033" 2598 "s59E25H2" 0 "NADH dehydrogenase"
"Chl_C14_01033" 2598 "s59E5H2" 0 "NADH dehydrogenase"
"Chl_C14_01033" 2598 "s63E6H2" 0 "NADH dehydrogenase"
"Chl_C14_01034" 2599 "s59E13H2" 0 "Succinyl-diaminopimelate desuccinylase"
"Chl_C14_01034" 2599 "s59E25H2" 0 "Succinyl-diaminopimelate desuccinylase"
"Chl_C14_01034" 2599 "s59E5H2" 0 "Succinyl-diaminopimelate desuccinylase"
"Chl_C14_01034" 2599 "s63E6H2" 0 "Succinyl-diaminopimelate desuccinylase"
"Chl_C14_01036" 2601 "s59E13H2" 0 "Leucine--tRNA ligase"
"Chl_C14_01036" 2601 "s59E25H2" 0 "Leucine--tRNA ligase"
"Chl_C14_01036" 2601 "s59E5H2" 0.00165561 "Leucine--tRNA ligase"
"Chl_C14_01036" 2601 "s63E6H2" 0 "Leucine--tRNA ligase"
"Chl_C14_01037" 2602 "s59E13H2" 0 "Pyrroline-5-carboxylate reductase"
"Chl_C14_01037" 2602 "s59E25H2" 0 "Pyrroline-5-carboxylate reductase"
"Chl_C14_01037" 2602 "s59E5H2" 0 "Pyrroline-5-carboxylate reductase"
"Chl_C14_01037" 2602 "s63E6H2" 0 "Pyrroline-5-carboxylate reductase"
"Chl_C14_01038" 2603 "s59E13H2" 0 "MMPL family protein"
"Chl_C14_01038" 2603 "s59E25H2" 0 "MMPL family protein"
"Chl_C14_01038" 2603 "s59E5H2" 0 "MMPL family protein"
"Chl_C14_01038" 2603 "s63E6H2" 0 "MMPL family protein"
"Chl_C14_01039" 2604 "s59E13H2" 0 "Macrolide export protein MacA"
"Chl_C14_01039" 2604 "s59E25H2" 0 "Macrolide export protein MacA"
"Chl_C14_01039" 2604 "s59E5H2" 0 "Macrolide export protein MacA"
"Chl_C14_01039" 2604 "s63E6H2" 0 "Macrolide export protein MacA"
"Chl_C14_01040" 2605 "s59E13H2" 0 "putative ABC transporter ATP-binding protein"
"Chl_C14_01040" 2605 "s59E25H2" 0 "putative ABC transporter ATP-binding protein"
"Chl_C14_01040" 2605 "s59E5H2" 0 "putative ABC transporter ATP-binding protein"
"Chl_C14_01040" 2605 "s63E6H2" 0 "putative ABC transporter ATP-binding protein"
"Chl_C14_01041" 2606 "s59E13H2" 0.001289926 "Macrolide export ATP-binding/permease protein MacB"
"Chl_C14_01041" 2606 "s59E25H2" 0 "Macrolide export ATP-binding/permease protein MacB"
"Chl_C14_01041" 2606 "s59E5H2" 0.006453591 "Macrolide export ATP-binding/permease protein MacB"
"Chl_C14_01041" 2606 "s63E6H2" 0.003289911 "Macrolide export ATP-binding/permease protein MacB"
"Chl_C14_01042" 2607 "s59E13H2" 0 "colicin V production protein"
"Chl_C14_01042" 2607 "s59E25H2" 0 "colicin V production protein"

"Chl_C14_01042" 2607 "s59E5H2" 0 "colicin V production protein"
"Chl_C14_01042" 2607 "s63E6H2" 0 "colicin V production protein"
"Chl_C14_01043" 2608 "s59E13H2" 0 "putative ferredoxin-like protein YfhL"
"Chl_C14_01043" 2608 "s59E25H2" 0 "putative ferredoxin-like protein YfhL"
"Chl_C14_01043" 2608 "s59E5H2" 0 "putative ferredoxin-like protein YfhL"
"Chl_C14_01043" 2608 "s63E6H2" 0 "putative ferredoxin-like protein YfhL"
"Chl_C14_01044" 2609 "s59E13H2" 0 "Phosphopantetheine adenylyltransferase"
"Chl_C14_01044" 2609 "s59E25H2" 0 "Phosphopantetheine adenylyltransferase"
"Chl_C14_01044" 2609 "s59E5H2" 0 "Phosphopantetheine adenylyltransferase"
"Chl_C14_01044" 2609 "s63E6H2" 0 "Phosphopantetheine adenylyltransferase"
"Chl_C14_01045" 2610 "s59E13H2" 0 "Ribosomal RNA small subunit methyltransferase D"
"Chl_C14_01045" 2610 "s59E25H2" 0 "Ribosomal RNA small subunit methyltransferase D"
"Chl_C14_01045" 2610 "s59E5H2" 0 "Ribosomal RNA small subunit methyltransferase D"
"Chl_C14_01045" 2610 "s63E6H2" 0 "Ribosomal RNA small subunit methyltransferase D"
"Chl_C14_01046" 2611 "s59E13H2" 0 "NAD(P)H-quinone oxidoreductase subunit 3"
"Chl_C14_01046" 2611 "s59E25H2" 0 "NAD(P)H-quinone oxidoreductase subunit 3"
"Chl_C14_01046" 2611 "s59E5H2" 0 "NAD(P)H-quinone oxidoreductase subunit 3"
"Chl_C14_01046" 2611 "s63E6H2" 0 "NAD(P)H-quinone oxidoreductase subunit 3"
"Chl_C14_01047" 2612 "s59E13H2" 0 "NADH-quinone oxidoreductase subunit B"
"Chl_C14_01047" 2612 "s59E25H2" 0 "NADH-quinone oxidoreductase subunit B"
"Chl_C14_01047" 2612 "s59E5H2" 0 "NADH-quinone oxidoreductase subunit B"
"Chl_C14_01047" 2612 "s63E6H2" 0 "NADH-quinone oxidoreductase subunit B"
"Chl_C14_01048" 2613 "s59E13H2" 0 "NADH-quinone oxidoreductase subunit 5"
"Chl_C14_01048" 2613 "s59E25H2" 0 "NADH-quinone oxidoreductase subunit 5"
"Chl_C14_01048" 2613 "s59E5H2" 0 "NADH-quinone oxidoreductase subunit 5"
"Chl_C14_01048" 2613 "s63E6H2" 0 "NADH-quinone oxidoreductase subunit 5"
"Chl_C14_01049" 2614 "s59E13H2" 0 "NADH-quinone oxidoreductase subunit 4"
"Chl_C14_01049" 2614 "s59E25H2" 0 "NADH-quinone oxidoreductase subunit 4"
"Chl_C14_01049" 2614 "s59E5H2" 0 "NADH-quinone oxidoreductase subunit 4"
"Chl_C14_01049" 2614 "s63E6H2" 0 "NADH-quinone oxidoreductase subunit 4"
"Chl_C14_01050" 2615 "s59E13H2" 0 "NADH-quinone oxidoreductase subunit H"
"Chl_C14_01050" 2615 "s59E25H2" 0 "NADH-quinone oxidoreductase subunit H"
"Chl_C14_01050" 2615 "s59E5H2" 0 "NADH-quinone oxidoreductase subunit H"
"Chl_C14_01050" 2615 "s63E6H2" 0 "NADH-quinone oxidoreductase subunit H"
"Chl_C14_01051" 2616 "s59E13H2" 0 "NADH-quinone oxidoreductase subunit I"
"Chl_C14_01051" 2616 "s59E25H2" 0 "NADH-quinone oxidoreductase subunit I"
"Chl_C14_01051" 2616 "s59E5H2" 0 "NADH-quinone oxidoreductase subunit I"
"Chl_C14_01051" 2616 "s63E6H2" 0 "NADH-quinone oxidoreductase subunit I"
"Chl_C14_01052" 2617 "s59E13H2" 0 "F(420)H(2) dehydrogenase subunit J"
"Chl_C14_01052" 2617 "s59E25H2" 0 "F(420)H(2) dehydrogenase subunit J"
"Chl_C14_01052" 2617 "s59E5H2" 0 "F(420)H(2) dehydrogenase subunit J"
"Chl_C14_01052" 2617 "s63E6H2" 0 "F(420)H(2) dehydrogenase subunit J"
"Chl_C14_01053" 2618 "s59E13H2" 0 "F(420)H(2) dehydrogenase subunit K"
"Chl_C14_01053" 2618 "s59E25H2" 0 "F(420)H(2) dehydrogenase subunit K"
"Chl_C14_01053" 2618 "s59E5H2" 0 "F(420)H(2) dehydrogenase subunit K"
"Chl_C14_01053" 2618 "s63E6H2" 0 "F(420)H(2) dehydrogenase subunit K"
"Chl_C14_01054" 2619 "s59E13H2" 0 "hypothetical protein"

"Chl_C14_01054" 2619 "s59E25H2" 0 "hypothetical protein"
"Chl_C14_01054" 2619 "s59E5H2" 0 "hypothetical protein"
"Chl_C14_01054" 2619 "s63E6H2" 0 "hypothetical protein"
"Chl_C14_01056" 2621 "s59E13H2" 0 "F(420)H(2) dehydrogenase subunit L"
"Chl_C14_01056" 2621 "s59E25H2" 0 "F(420)H(2) dehydrogenase subunit L"
"Chl_C14_01056" 2621 "s59E5H2" 0.04654888 "F(420)H(2) dehydrogenase subunit L"
"Chl_C14_01056" 2621 "s63E6H2" 0 "F(420)H(2) dehydrogenase subunit L"
"Chl_C14_01057" 2622 "s59E13H2" 0.00066043 "F(420)H(2) dehydrogenase subunit M"
"Chl_C14_01057" 2622 "s59E25H2" 0 "F(420)H(2) dehydrogenase subunit M"
"Chl_C14_01057" 2622 "s59E5H2" 0.02424664 "F(420)H(2) dehydrogenase subunit M"
"Chl_C14_01057" 2622 "s63E6H2" 0 "F(420)H(2) dehydrogenase subunit M"
"Chl_C14_01058" 2623 "s59E13H2" 0 "ADP-ribose pyrophosphatase"
"Chl_C14_01058" 2623 "s59E25H2" 0 "ADP-ribose pyrophosphatase"
"Chl_C14_01058" 2623 "s59E5H2" 0 "ADP-ribose pyrophosphatase"
"Chl_C14_01058" 2623 "s63E6H2" 0 "ADP-ribose pyrophosphatase"
"Chl_C14_01059" 2624 "s59E13H2" 0 "F(420)H(2) dehydrogenase subunit N"
"Chl_C14_01059" 2624 "s59E25H2" 0 "F(420)H(2) dehydrogenase subunit N"
"Chl_C14_01059" 2624 "s59E5H2" 0 "F(420)H(2) dehydrogenase subunit N"
"Chl_C14_01059" 2624 "s63E6H2" 0 "F(420)H(2) dehydrogenase subunit N"
"Chl_C14_01060" 2625 "s59E13H2" 0 "DNA recombination protein RmuC"
"Chl_C14_01060" 2625 "s59E25H2" 0 "DNA recombination protein RmuC"
"Chl_C14_01060" 2625 "s59E5H2" 0 "DNA recombination protein RmuC"
"Chl_C14_01060" 2625 "s63E6H2" 0 "DNA recombination protein RmuC"
"Chl_C14_01061" 2626 "s59E13H2" 0 "hypothetical protein"
"Chl_C14_01061" 2626 "s59E25H2" 0 "hypothetical protein"
"Chl_C14_01061" 2626 "s59E5H2" 0 "hypothetical protein"
"Chl_C14_01061" 2626 "s63E6H2" 0 "hypothetical protein"
"Chl_C14_01062" 2627 "s59E13H2" 0 "hypothetical protein"
"Chl_C14_01062" 2627 "s59E25H2" 0 "hypothetical protein"
"Chl_C14_01062" 2627 "s59E5H2" 0 "hypothetical protein"
"Chl_C14_01062" 2627 "s63E6H2" 0 "hypothetical protein"
"Chl_C14_01063" 2628 "s59E13H2" 0 "hypothetical protein"
"Chl_C14_01063" 2628 "s59E25H2" 0 "hypothetical protein"
"Chl_C14_01063" 2628 "s59E5H2" 0 "hypothetical protein"
"Chl_C14_01063" 2628 "s63E6H2" 0 "hypothetical protein"
"Chl_C14_01064" 2629 "s59E13H2" 0 "hypothetical protein"
"Chl_C14_01064" 2629 "s59E25H2" 0 "hypothetical protein"
"Chl_C14_01064" 2629 "s59E5H2" 0 "hypothetical protein"
"Chl_C14_01064" 2629 "s63E6H2" 0 "hypothetical protein"
"Chl_C14_01065" 2630 "s59E13H2" 0 "hypothetical protein"
"Chl_C14_01065" 2630 "s59E25H2" 0 "hypothetical protein"
"Chl_C14_01065" 2630 "s59E5H2" 0 "hypothetical protein"
"Chl_C14_01065" 2630 "s63E6H2" 0 "hypothetical protein"
"Chl_C14_01068" 2633 "s59E13H2" 0 "hypothetical protein"
"Chl_C14_01068" 2633 "s59E25H2" 0 "hypothetical protein"
"Chl_C14_01068" 2633 "s59E5H2" 0 "hypothetical protein"
"Chl_C14_01068" 2633 "s63E6H2" 0 "hypothetical protein"

"Chl_C14_01069" 2634 "s59E13H2" 0 "putative inorganic polyphosphate/ATP-NAD kinase"
"Chl_C14_01069" 2634 "s59E25H2" 0 "putative inorganic polyphosphate/ATP-NAD kinase"
"Chl_C14_01069" 2634 "s59E5H2" 0 "putative inorganic polyphosphate/ATP-NAD kinase"
"Chl_C14_01069" 2634 "s63E6H2" 0.03285141 "putative inorganic polyphosphate/ATP-NAD kinase"
"Chl_C14_01070" 2635 "s59E13H2" 0 "Amino-acid acetyltransferase"
"Chl_C14_01070" 2635 "s59E25H2" 0 "Amino-acid acetyltransferase"
"Chl_C14_01070" 2635 "s59E5H2" 0.006519557 "Amino-acid acetyltransferase"
"Chl_C14_01070" 2635 "s63E6H2" 0.0007190558 "Amino-acid acetyltransferase"
"Chl_C14_01071" 2636 "s59E13H2" 0 "ADP-ribose pyrophosphatase"
"Chl_C14_01071" 2636 "s59E25H2" 0 "ADP-ribose pyrophosphatase"
"Chl_C14_01071" 2636 "s59E5H2" 0 "ADP-ribose pyrophosphatase"
"Chl_C14_01071" 2636 "s63E6H2" 0 "ADP-ribose pyrophosphatase"
"Chl_C14_01072" 2637 "s59E13H2" 0 "hypothetical protein"
"Chl_C14_01072" 2637 "s59E25H2" 0 "hypothetical protein"
"Chl_C14_01072" 2637 "s59E5H2" 0 "hypothetical protein"
"Chl_C14_01072" 2637 "s63E6H2" 0 "hypothetical protein"
"Chl_C14_01073" 2638 "s59E13H2" 0 "HIT-like protein"
"Chl_C14_01073" 2638 "s59E25H2" 0 "HIT-like protein"
"Chl_C14_01073" 2638 "s59E5H2" 0 "HIT-like protein"
"Chl_C14_01073" 2638 "s63E6H2" 0 "HIT-like protein"
"Chl_C14_01074" 2639 "s59E13H2" 0 "Fumarate hydratase class I, aerobic"
"Chl_C14_01074" 2639 "s59E25H2" 0 "Fumarate hydratase class I, aerobic"
"Chl_C14_01074" 2639 "s59E5H2" 0.00543328 "Fumarate hydratase class I, aerobic"
"Chl_C14_01074" 2639 "s63E6H2" 0 "Fumarate hydratase class I, aerobic"
"Chl_C14_01075" 2640 "s59E13H2" 0 "L(+)-tartrate dehydratase subunit alpha"
"Chl_C14_01075" 2640 "s59E25H2" 0 "L(+)-tartrate dehydratase subunit alpha"
"Chl_C14_01075" 2640 "s59E5H2" 0 "L(+)-tartrate dehydratase subunit alpha"
"Chl_C14_01075" 2640 "s63E6H2" 0 "L(+)-tartrate dehydratase subunit alpha"
"Chl_C14_01077" 2642 "s59E13H2" 0 "Malate dehydrogenase"
"Chl_C14_01077" 2642 "s59E25H2" 0 "Malate dehydrogenase"
"Chl_C14_01077" 2642 "s59E5H2" 0 "Malate dehydrogenase"
"Chl_C14_01077" 2642 "s63E6H2" 0 "Malate dehydrogenase"
"Chl_C14_01078" 2643 "s59E13H2" 0 "Bifunctional folate synthesis protein"
"Chl_C14_01078" 2643 "s59E25H2" 0 "Bifunctional folate synthesis protein"
"Chl_C14_01078" 2643 "s59E5H2" 0 "Bifunctional folate synthesis protein"
"Chl_C14_01078" 2643 "s63E6H2" 0 "Bifunctional folate synthesis protein"
"Chl_C14_01079" 2644 "s59E13H2" 0 "6-carboxy-5,6,7,8-tetrahydropterin synthase"
"Chl_C14_01079" 2644 "s59E25H2" 0 "6-carboxy-5,6,7,8-tetrahydropterin synthase"
"Chl_C14_01079" 2644 "s59E5H2" 0 "6-carboxy-5,6,7,8-tetrahydropterin synthase"
"Chl_C14_01079" 2644 "s63E6H2" 0 "6-carboxy-5,6,7,8-tetrahydropterin synthase"
"Chl_C14_01080" 2645 "s59E13H2" 0 "hypothetical protein"
"Chl_C14_01080" 2645 "s59E25H2" 0 "hypothetical protein"
"Chl_C14_01080" 2645 "s59E5H2" 0 "hypothetical protein"
"Chl_C14_01080" 2645 "s63E6H2" 0 "hypothetical protein"
"Chl_C14_01081" 2646 "s59E13H2" 0 "fermentation/respiration switch protein"
"Chl_C14_01081" 2646 "s59E25H2" 0 "fermentation/respiration switch protein"
"Chl_C14_01081" 2646 "s59E5H2" 7.133876e-05 "fermentation/respiration switch protein"

"Chl_C14_01081" 2646 "s63E6H2" 0 "fermentation/respiration switch protein"
"Chl_C14_01082" 2647 "s59E13H2" 0 "hypothetical protein"
"Chl_C14_01082" 2647 "s59E25H2" 0 "hypothetical protein"
"Chl_C14_01082" 2647 "s59E5H2" 0.07633975 "hypothetical protein"
"Chl_C14_01082" 2647 "s63E6H2" 0 "hypothetical protein"
"Chl_C14_01083" 2648 "s59E13H2" 0 "Vegetative catalase"
"Chl_C14_01083" 2648 "s59E25H2" 0 "Vegetative catalase"
"Chl_C14_01083" 2648 "s59E5H2" 0.0158051 "Vegetative catalase"
"Chl_C14_01083" 2648 "s63E6H2" 0 "Vegetative catalase"
"Chl_C14_01084" 2649 "s59E13H2" 0 "DNA polymerase III subunit beta"
"Chl_C14_01084" 2649 "s59E25H2" 0 "DNA polymerase III subunit beta"
"Chl_C14_01084" 2649 "s59E5H2" 0 "DNA polymerase III subunit beta"
"Chl_C14_01084" 2649 "s63E6H2" 0 "DNA polymerase III subunit beta"
"Chl_C14_01085" 2650 "s59E13H2" 0 "Cyclic pyranopterin monophosphate synthase accessory protein"
"Chl_C14_01085" 2650 "s59E25H2" 0 "Cyclic pyranopterin monophosphate synthase accessory protein"
"Chl_C14_01085" 2650 "s59E5H2" 0 "Cyclic pyranopterin monophosphate synthase accessory protein"
"Chl_C14_01085" 2650 "s63E6H2" 0.03496585 "Cyclic pyranopterin monophosphate synthase accessory protein"
"Chl_C14_01086" 2651 "s59E13H2" 0 "putative tyrosine recombinase XerC-like protein"
"Chl_C14_01086" 2651 "s59E25H2" 0 "putative tyrosine recombinase XerC-like protein"
"Chl_C14_01086" 2651 "s59E5H2" 0 "putative tyrosine recombinase XerC-like protein"
"Chl_C14_01086" 2651 "s63E6H2" 0 "putative tyrosine recombinase XerC-like protein"
"Chl_C14_01087" 2652 "s59E13H2" 0 "hypothetical protein"
"Chl_C14_01087" 2652 "s59E25H2" 0 "hypothetical protein"
"Chl_C14_01087" 2652 "s59E5H2" 0 "hypothetical protein"
"Chl_C14_01087" 2652 "s63E6H2" 0 "hypothetical protein"
"Chl_C14_01088" 2653 "s59E13H2" 0 "Succinyl-CoA ligase [ADP-forming] subunit alpha"
"Chl_C14_01088" 2653 "s59E25H2" 0 "Succinyl-CoA ligase [ADP-forming] subunit alpha"
"Chl_C14_01088" 2653 "s59E5H2" 0 "Succinyl-CoA ligase [ADP-forming] subunit alpha"
"Chl_C14_01088" 2653 "s63E6H2" 0 "Succinyl-CoA ligase [ADP-forming] subunit alpha"
"Chl_C14_01089" 2654 "s59E13H2" 0 "Argininosuccinate lyase 1"
"Chl_C14_01089" 2654 "s59E25H2" 0 "Argininosuccinate lyase 1"
"Chl_C14_01089" 2654 "s59E5H2" 0 "Argininosuccinate lyase 1"
"Chl_C14_01089" 2654 "s63E6H2" 0 "Argininosuccinate lyase 1"
"Chl_C14_01090" 2655 "s59E13H2" 0 "2-methylcitrate dehydratase"
"Chl_C14_01090" 2655 "s59E25H2" 0 "2-methylcitrate dehydratase"
"Chl_C14_01090" 2655 "s59E5H2" 0 "2-methylcitrate dehydratase"
"Chl_C14_01090" 2655 "s63E6H2" 0 "2-methylcitrate dehydratase"
"Chl_C14_01091" 2656 "s59E13H2" 0 "Aldehyde ferredoxin oxidoreductase, domains 2 & 3"
"Chl_C14_01091" 2656 "s59E25H2" 0 "Aldehyde ferredoxin oxidoreductase, domains 2 & 3"
"Chl_C14_01091" 2656 "s59E5H2" 0 "Aldehyde ferredoxin oxidoreductase, domains 2 & 3"
"Chl_C14_01091" 2656 "s63E6H2" 0 "Aldehyde ferredoxin oxidoreductase, domains 2 & 3"
"Chl_C14_01092" 2657 "s59E13H2" 0 "Alpha/beta hydrolase family protein"
"Chl_C14_01092" 2657 "s59E25H2" 0 "Alpha/beta hydrolase family protein"
"Chl_C14_01092" 2657 "s59E5H2" 0 "Alpha/beta hydrolase family protein"
"Chl_C14_01092" 2657 "s63E6H2" 0 "Alpha/beta hydrolase family protein"
"Chl_C14_01093" 2658 "s59E13H2" 0 "2-methylcitrate dehydratase"
"Chl_C14_01093" 2658 "s59E25H2" 0 "2-methylcitrate dehydratase"

"Chl_C14_01093" 2658 "s59E5H2" 0 "2-methylcitrate dehydratase"
"Chl_C14_01093" 2658 "s63E6H2" 0 "2-methylcitrate dehydratase"
"Chl_C14_01094" 2659 "s59E13H2" 0 "Acryloyl-CoA reductase electron transfer subunit beta"
"Chl_C14_01094" 2659 "s59E25H2" 0 "Acryloyl-CoA reductase electron transfer subunit beta"
"Chl_C14_01094" 2659 "s59E5H2" 0 "Acryloyl-CoA reductase electron transfer subunit beta"
"Chl_C14_01094" 2659 "s63E6H2" 0 "Acryloyl-CoA reductase electron transfer subunit beta"
"Chl_C14_01095" 2660 "s59E13H2" 0 "Electron transfer flavoprotein subunit beta"
"Chl_C14_01095" 2660 "s59E25H2" 0 "Electron transfer flavoprotein subunit beta"
"Chl_C14_01095" 2660 "s59E5H2" 0 "Electron transfer flavoprotein subunit beta"
"Chl_C14_01095" 2660 "s63E6H2" 0 "Electron transfer flavoprotein subunit beta"
"Chl_C14_01096" 2661 "s59E13H2" 0 "Fumarate reductase flavoprotein subunit precursor"
"Chl_C14_01096" 2661 "s59E25H2" 0 "Fumarate reductase flavoprotein subunit precursor"
"Chl_C14_01096" 2661 "s59E5H2" 0 "Fumarate reductase flavoprotein subunit precursor"
"Chl_C14_01096" 2661 "s63E6H2" 0 "Fumarate reductase flavoprotein subunit precursor"
"Chl_C14_01097" 2662 "s59E13H2" 0 "Capsule biosynthesis protein CapA"
"Chl_C14_01097" 2662 "s59E25H2" 0 "Capsule biosynthesis protein CapA"
"Chl_C14_01097" 2662 "s59E5H2" 0 "Capsule biosynthesis protein CapA"
"Chl_C14_01097" 2662 "s63E6H2" 0 "Capsule biosynthesis protein CapA"
"Chl_C14_01098" 2663 "s59E13H2" 0 "2,3-dimethylmalate lyase"
"Chl_C14_01098" 2663 "s59E25H2" 0 "2,3-dimethylmalate lyase"
"Chl_C14_01098" 2663 "s59E5H2" 0 "2,3-dimethylmalate lyase"
"Chl_C14_01098" 2663 "s63E6H2" 0 "2,3-dimethylmalate lyase"
"Chl_C14_01099" 2664 "s59E13H2" 0 "Isopropylmalate/citramalate isomerase small subunit"
"Chl_C14_01099" 2664 "s59E25H2" 0 "Isopropylmalate/citramalate isomerase small subunit"
"Chl_C14_01099" 2664 "s59E5H2" 0 "Isopropylmalate/citramalate isomerase small subunit"
"Chl_C14_01099" 2664 "s63E6H2" 0 "Isopropylmalate/citramalate isomerase small subunit"
"Chl_C14_01100" 2665 "s59E13H2" 0 "Isopropylmalate/citramalate isomerase large subunit"
"Chl_C14_01100" 2665 "s59E25H2" 0 "Isopropylmalate/citramalate isomerase large subunit"
"Chl_C14_01100" 2665 "s59E5H2" 0 "Isopropylmalate/citramalate isomerase large subunit"
"Chl_C14_01100" 2665 "s63E6H2" 0 "Isopropylmalate/citramalate isomerase large subunit"
"Chl_C14_01101" 2666 "s59E13H2" 0 "3-oxosteroid 1-dehydrogenase"
"Chl_C14_01101" 2666 "s59E25H2" 0 "3-oxosteroid 1-dehydrogenase"
"Chl_C14_01101" 2666 "s59E5H2" 0 "3-oxosteroid 1-dehydrogenase"
"Chl_C14_01101" 2666 "s63E6H2" 0 "3-oxosteroid 1-dehydrogenase"
"Chl_C14_01102" 2667 "s59E13H2" 0 "3-isopropylmalate/3-methylmalate dehydrogenase"
"Chl_C14_01102" 2667 "s59E25H2" 0 "3-isopropylmalate/3-methylmalate dehydrogenase"
"Chl_C14_01102" 2667 "s59E5H2" 0 "3-isopropylmalate/3-methylmalate dehydrogenase"
"Chl_C14_01102" 2667 "s63E6H2" 0 "3-isopropylmalate/3-methylmalate dehydrogenase"
"Chl_C14_01103" 2668 "s59E13H2" 0 "Indolepyruvate oxidoreductase subunit IorB"
"Chl_C14_01103" 2668 "s59E25H2" 0 "Indolepyruvate oxidoreductase subunit IorB"
"Chl_C14_01103" 2668 "s59E5H2" 0 "Indolepyruvate oxidoreductase subunit IorB"
"Chl_C14_01103" 2668 "s63E6H2" 0 "Indolepyruvate oxidoreductase subunit IorB"
"Chl_G11_00001" 2670 "s59E13H2" 0 "hypothetical protein"
"Chl_G11_00001" 2670 "s59E25H2" 0 "hypothetical protein"
"Chl_G11_00001" 2670 "s59E5H2" 0 "hypothetical protein"
"Chl_G11_00001" 2670 "s63E6H2" 0 "hypothetical protein"
"Chl_G11_00003" 2672 "s59E13H2" 0 "L-2-keto-3-deoxyarabonate dehydratase"

"Chl_G11_00003" 2672 "s59E25H2" 0 "L-2-keto-3-deoxyarabonate dehydratase"
"Chl_G11_00003" 2672 "s59E5H2" 0 "L-2-keto-3-deoxyarabonate dehydratase"
"Chl_G11_00003" 2672 "s63E6H2" 0 "L-2-keto-3-deoxyarabonate dehydratase"
"Chl_G11_00004" 2673 "s59E13H2" 0 "Trehalose synthase"
"Chl_G11_00004" 2673 "s59E25H2" 0 "Trehalose synthase"
"Chl_G11_00004" 2673 "s59E5H2" 0.003068027 "Trehalose synthase"
"Chl_G11_00004" 2673 "s63E6H2" 0 "Trehalose synthase"
"Chl_G11_00005" 2674 "s59E13H2" 0 "Bifunctional trehalose-6-phosphate synthase/phosphatase"
"Chl_G11_00005" 2674 "s59E25H2" 0 "Bifunctional trehalose-6-phosphate synthase/phosphatase"
"Chl_G11_00005" 2674 "s59E5H2" 0.001821125 "Bifunctional trehalose-6-phosphate synthase/phosphatase"
"Chl_G11_00005" 2674 "s63E6H2" 0 "Bifunctional trehalose-6-phosphate synthase/phosphatase"
"Chl_G11_00006" 2675 "s59E13H2" 0 "Glucose-1-phosphate thymidylyltransferase"
"Chl_G11_00006" 2675 "s59E25H2" 0 "Glucose-1-phosphate thymidylyltransferase"
"Chl_G11_00006" 2675 "s59E5H2" 0 "Glucose-1-phosphate thymidylyltransferase"
"Chl_G11_00006" 2675 "s63E6H2" 0 "Glucose-1-phosphate thymidylyltransferase"
"Chl_G11_00007" 2676 "s59E13H2" 0 "NADP-reducing hydrogenase subunit HndC"
"Chl_G11_00007" 2676 "s59E25H2" 0 "NADP-reducing hydrogenase subunit HndC"
"Chl_G11_00007" 2676 "s59E5H2" 0 "NADP-reducing hydrogenase subunit HndC"
"Chl_G11_00007" 2676 "s63E6H2" 0 "NADP-reducing hydrogenase subunit HndC"
"Chl_G11_00008" 2677 "s59E13H2" 0 "NADP-reducing hydrogenase subunit HndC"
"Chl_G11_00008" 2677 "s59E25H2" 0 "NADP-reducing hydrogenase subunit HndC"
"Chl_G11_00008" 2677 "s59E5H2" 0.00210905 "NADP-reducing hydrogenase subunit HndC"
"Chl_G11_00008" 2677 "s63E6H2" 0 "NADP-reducing hydrogenase subunit HndC"
"Chl_G11_00009" 2678 "s59E13H2" 0 "NADP-reducing hydrogenase subunit HndA"
"Chl_G11_00009" 2678 "s59E25H2" 0 "NADP-reducing hydrogenase subunit HndA"
"Chl_G11_00009" 2678 "s59E5H2" 0 "NADP-reducing hydrogenase subunit HndA"
"Chl_G11_00009" 2678 "s63E6H2" 0 "NADP-reducing hydrogenase subunit HndA"
"Chl_G11_00010" 2679 "s59E13H2" 0 "hypothetical protein"
"Chl_G11_00010" 2679 "s59E25H2" 0 "hypothetical protein"
"Chl_G11_00010" 2679 "s59E5H2" 0 "hypothetical protein"
"Chl_G11_00010" 2679 "s63E6H2" 0 "hypothetical protein"
"Chl_G11_00011" 2680 "s59E13H2" 0 "hypothetical protein"
"Chl_G11_00011" 2680 "s59E25H2" 0 "hypothetical protein"
"Chl_G11_00011" 2680 "s59E5H2" 0 "hypothetical protein"
"Chl_G11_00011" 2680 "s63E6H2" 0 "hypothetical protein"
"Chl_G11_00012" 2681 "s59E13H2" 0 "acetyl-CoA acetyltransferase"
"Chl_G11_00012" 2681 "s59E25H2" 0 "acetyl-CoA acetyltransferase"
"Chl_G11_00012" 2681 "s59E5H2" 0 "acetyl-CoA acetyltransferase"
"Chl_G11_00012" 2681 "s63E6H2" 0 "acetyl-CoA acetyltransferase"
"Chl_G11_00013" 2682 "s59E13H2" 0 "Acyl-CoA dehydrogenase"
"Chl_G11_00013" 2682 "s59E25H2" 0 "Acyl-CoA dehydrogenase"
"Chl_G11_00013" 2682 "s59E5H2" 0 "Acyl-CoA dehydrogenase"
"Chl_G11_00013" 2682 "s63E6H2" 0 "Acyl-CoA dehydrogenase"
"Chl_G11_00014" 2683 "s59E13H2" 0 "Acyl-CoA dehydrogenase"
"Chl_G11_00014" 2683 "s59E25H2" 0 "Acyl-CoA dehydrogenase"
"Chl_G11_00014" 2683 "s59E5H2" 0 "Acyl-CoA dehydrogenase"
"Chl_G11_00014" 2683 "s63E6H2" 0 "Acyl-CoA dehydrogenase"

"Chl_G11_00015" 2684 "s59E13H2" 0 "2-dehydro-3-deoxy-D-gluconate 5-dehydrogenase"
"Chl_G11_00015" 2684 "s59E25H2" 0 "2-dehydro-3-deoxy-D-gluconate 5-dehydrogenase"
"Chl_G11_00015" 2684 "s59E5H2" 0 "2-dehydro-3-deoxy-D-gluconate 5-dehydrogenase"
"Chl_G11_00015" 2684 "s63E6H2" 0 "2-dehydro-3-deoxy-D-gluconate 5-dehydrogenase"
"Chl_G11_00016" 2685 "s59E13H2" 0 "3-hydroxypropionyl-coenzyme A dehydratase"
"Chl_G11_00016" 2685 "s59E25H2" 0 "3-hydroxypropionyl-coenzyme A dehydratase"
"Chl_G11_00016" 2685 "s59E5H2" 0 "3-hydroxypropionyl-coenzyme A dehydratase"
"Chl_G11_00016" 2685 "s63E6H2" 0 "3-hydroxypropionyl-coenzyme A dehydratase"
"Chl_G11_00017" 2686 "s59E13H2" 0 "4-hydroxybutyryl-CoA dehydratase/vinylacetyl-CoA-Delta-isomerase"
"Chl_G11_00017" 2686 "s59E25H2" 0 "4-hydroxybutyryl-CoA dehydratase/vinylacetyl-CoA-Delta-isomerase"
"Chl_G11_00017" 2686 "s59E5H2" 0 "4-hydroxybutyryl-CoA dehydratase/vinylacetyl-CoA-Delta-isomerase"
"Chl_G11_00017" 2686 "s63E6H2" 0 "4-hydroxybutyryl-CoA dehydratase/vinylacetyl-CoA-Delta-isomerase"
"Chl_G11_00018" 2687 "s59E13H2" 0 "Enamine/imine deaminase"
"Chl_G11_00018" 2687 "s59E25H2" 0 "Enamine/imine deaminase"
"Chl_G11_00018" 2687 "s59E5H2" 0 "Enamine/imine deaminase"
"Chl_G11_00018" 2687 "s63E6H2" 0 "Enamine/imine deaminase"
"Chl_G11_00019" 2688 "s59E13H2" 0.6584998 "putative peptidyl-prolyl cis-trans isomerase"
"Chl_G11_00019" 2688 "s59E25H2" 0 "putative peptidyl-prolyl cis-trans isomerase"
"Chl_G11_00019" 2688 "s59E5H2" 0 "putative peptidyl-prolyl cis-trans isomerase"
"Chl_G11_00019" 2688 "s63E6H2" 0 "putative peptidyl-prolyl cis-trans isomerase"
"Chl_G11_00020" 2689 "s59E13H2" 0 "Alkaline phosphatase synthesis sensor protein PhoR"
"Chl_G11_00020" 2689 "s59E25H2" 0 "Alkaline phosphatase synthesis sensor protein PhoR"
"Chl_G11_00020" 2689 "s59E5H2" 0 "Alkaline phosphatase synthesis sensor protein PhoR"
"Chl_G11_00020" 2689 "s63E6H2" 0 "Alkaline phosphatase synthesis sensor protein PhoR"
"Chl_G11_00022" 2691 "s59E13H2" 0 "Alkaline phosphatase synthesis transcriptional regulatory protein PhoP"
"Chl_G11_00022" 2691 "s59E25H2" 0 "Alkaline phosphatase synthesis transcriptional regulatory protein PhoP"
"Chl_G11_00022" 2691 "s59E5H2" 0 "Alkaline phosphatase synthesis transcriptional regulatory protein PhoP"
"Chl_G11_00022" 2691 "s63E6H2" 0 "Alkaline phosphatase synthesis transcriptional regulatory protein PhoP"
"Chl_G11_00023" 2692 "s59E13H2" 0 "Alkaline phosphatase synthesis transcriptional regulatory protein PhoP"
"Chl_G11_00023" 2692 "s59E25H2" 0 "Alkaline phosphatase synthesis transcriptional regulatory protein PhoP"
"Chl_G11_00023" 2692 "s59E5H2" 0 "Alkaline phosphatase synthesis transcriptional regulatory protein PhoP"
"Chl_G11_00023" 2692 "s63E6H2" 0 "Alkaline phosphatase synthesis transcriptional regulatory protein PhoP"
"Chl_G11_00024" 2693 "s59E13H2" 0 "Putative formate dehydrogenase"
"Chl_G11_00024" 2693 "s59E25H2" 0 "Putative formate dehydrogenase"
"Chl_G11_00024" 2693 "s59E5H2" 0 "Putative formate dehydrogenase"
"Chl_G11_00024" 2693 "s63E6H2" 0 "Putative formate dehydrogenase"
"Chl_G11_00025" 2694 "s59E13H2" 0 "NADP-reducing hydrogenase subunit HndA"
"Chl_G11_00025" 2694 "s59E25H2" 0 "NADP-reducing hydrogenase subunit HndA"
"Chl_G11_00025" 2694 "s59E5H2" 0 "NADP-reducing hydrogenase subunit HndA"
"Chl_G11_00025" 2694 "s63E6H2" 0 "NADP-reducing hydrogenase subunit HndA"
"Chl_G11_00026" 2695 "s59E13H2" 0 "NADP-reducing hydrogenase subunit HndC"
"Chl_G11_00026" 2695 "s59E25H2" 0 "NADP-reducing hydrogenase subunit HndC"
"Chl_G11_00026" 2695 "s59E5H2" 0 "NADP-reducing hydrogenase subunit HndC"
"Chl_G11_00026" 2695 "s63E6H2" 0 "NADP-reducing hydrogenase subunit HndC"
"Chl_G11_00027" 2696 "s59E13H2" 0 "hypothetical protein"
"Chl_G11_00027" 2696 "s59E25H2" 0 "hypothetical protein"
"Chl_G11_00027" 2696 "s59E5H2" 0 "hypothetical protein"

"Chl_G11_00027" 2696 "s63E6H2" 0 "hypothetical protein"
"Chl_G11_00028" 2697 "s59E13H2" 0 "Bifunctional homocysteine S-methyltransferase/5,10-methylenetetrahydrofolate reductase"
"Chl_G11_00028" 2697 "s59E25H2" 0 "Bifunctional homocysteine S-methyltransferase/5,10-methylenetetrahydrofolate reductase"
"Chl_G11_00028" 2697 "s59E5H2" 0.009018567 "Bifunctional homocysteine S-methyltransferase/5,10-methylenetetrahydrofolate reductase"
"Chl_G11_00028" 2697 "s63E6H2" 0 "Bifunctional homocysteine S-methyltransferase/5,10-methylenetetrahydrofolate reductase"
"Chl_G11_00029" 2698 "s59E13H2" 0 "Demethylmenaquinone methyltransferase"
"Chl_G11_00029" 2698 "s59E25H2" 0 "Demethylmenaquinone methyltransferase"
"Chl_G11_00029" 2698 "s59E5H2" 0 "Demethylmenaquinone methyltransferase"
"Chl_G11_00029" 2698 "s63E6H2" 0 "Demethylmenaquinone methyltransferase"
"Chl_G11_00030" 2699 "s59E13H2" 0 "putative polyketide biosynthesis zinc-dependent hydrolase BaeB"
"Chl_G11_00030" 2699 "s59E25H2" 0 "putative polyketide biosynthesis zinc-dependent hydrolase BaeB"
"Chl_G11_00030" 2699 "s59E5H2" 0 "putative polyketide biosynthesis zinc-dependent hydrolase BaeB"
"Chl_G11_00030" 2699 "s63E6H2" 0 "putative polyketide biosynthesis zinc-dependent hydrolase BaeB"
"Chl_G11_00031" 2700 "s59E13H2" 0 "Diacetylchitobiose deacetylase"
"Chl_G11_00031" 2700 "s59E25H2" 0 "Diacetylchitobiose deacetylase"
"Chl_G11_00031" 2700 "s59E5H2" 0.004916863 "Diacetylchitobiose deacetylase"
"Chl_G11_00031" 2700 "s63E6H2" 0 "Diacetylchitobiose deacetylase"
"Chl_G11_00032" 2701 "s59E13H2" 0 "Mycothiol acetyltransferase"
"Chl_G11_00032" 2701 "s59E25H2" 0 "Mycothiol acetyltransferase"
"Chl_G11_00032" 2701 "s59E5H2" 0 "Mycothiol acetyltransferase"
"Chl_G11_00032" 2701 "s63E6H2" 0 "Mycothiol acetyltransferase"
"Chl_G11_00033" 2702 "s59E13H2" 0 "D-inositol 3-phosphate glycosyltransferase"
"Chl_G11_00033" 2702 "s59E25H2" 0 "D-inositol 3-phosphate glycosyltransferase"
"Chl_G11_00033" 2702 "s59E5H2" 0 "D-inositol 3-phosphate glycosyltransferase"
"Chl_G11_00033" 2702 "s63E6H2" 0 "D-inositol 3-phosphate glycosyltransferase"
"Chl_G11_00034" 2703 "s59E13H2" 0 "hypothetical protein"
"Chl_G11_00034" 2703 "s59E25H2" 0 "hypothetical protein"
"Chl_G11_00034" 2703 "s59E5H2" 0.002380062 "hypothetical protein"
"Chl_G11_00034" 2703 "s63E6H2" 0 "hypothetical protein"
"Chl_G11_00036" 2705 "s59E13H2" 0 "hypothetical protein"
"Chl_G11_00036" 2705 "s59E25H2" 0 "hypothetical protein"
"Chl_G11_00036" 2705 "s59E5H2" 0 "hypothetical protein"
"Chl_G11_00036" 2705 "s63E6H2" 0 "hypothetical protein"
"Chl_G11_00037" 2706 "s59E13H2" 0 "hypothetical protein"
"Chl_G11_00037" 2706 "s59E25H2" 0 "hypothetical protein"
"Chl_G11_00037" 2706 "s59E5H2" 0 "hypothetical protein"
"Chl_G11_00037" 2706 "s63E6H2" 0 "hypothetical protein"
"Chl_G11_00039" 2708 "s59E13H2" 0 "hypothetical protein"
"Chl_G11_00039" 2708 "s59E25H2" 0 "hypothetical protein"
"Chl_G11_00039" 2708 "s59E5H2" 0 "hypothetical protein"
"Chl_G11_00039" 2708 "s63E6H2" 0 "hypothetical protein"
"Chl_G11_00047" 2716 "s59E13H2" 0.001163305 "putative tyrosine recombinase XerC-like protein"
"Chl_G11_00047" 2716 "s59E25H2" 0 "putative tyrosine recombinase XerC-like protein"
"Chl_G11_00047" 2716 "s59E5H2" 0 "putative tyrosine recombinase XerC-like protein"
"Chl_G11_00047" 2716 "s63E6H2" 0 "putative tyrosine recombinase XerC-like protein"
"Chl_G11_00049" 2718 "s59E13H2" 0.001436917 "Transposase"
"Chl_G11_00049" 2718 "s59E25H2" 0 "Transposase"

"Chl_G11_00049" 2718 "s59E5H2" 0 "Transposase"
"Chl_G11_00049" 2718 "s63E6H2" 1.024933 "Transposase"
"Chl_G11_00050" 2719 "s59E13H2" 0 "hypothetical protein"
"Chl_G11_00050" 2719 "s59E25H2" 0 "hypothetical protein"
"Chl_G11_00050" 2719 "s59E5H2" 0 "hypothetical protein"
"Chl_G11_00050" 2719 "s63E6H2" 0.00120411 "hypothetical protein"
"Chl_G11_00052" 2721 "s59E13H2" 0 "hypothetical protein"
"Chl_G11_00052" 2721 "s59E25H2" 0 "hypothetical protein"
"Chl_G11_00052" 2721 "s59E5H2" 0 "hypothetical protein"
"Chl_G11_00052" 2721 "s63E6H2" 0 "hypothetical protein"
"Chl_G11_00053" 2722 "s59E13H2" 0 "NTE family protein RssA"
"Chl_G11_00053" 2722 "s59E25H2" 0 "NTE family protein RssA"
"Chl_G11_00053" 2722 "s59E5H2" 0 "NTE family protein RssA"
"Chl_G11_00053" 2722 "s63E6H2" 0 "NTE family protein RssA"
"Chl_G11_00055" 2724 "s59E13H2" 0 "DegV domain-containing protein/M5005_Spy1226"
"Chl_G11_00055" 2724 "s59E25H2" 0 "DegV domain-containing protein/M5005_Spy1226"
"Chl_G11_00055" 2724 "s59E5H2" 0 "DegV domain-containing protein/M5005_Spy1226"
"Chl_G11_00055" 2724 "s63E6H2" 0 "DegV domain-containing protein/M5005_Spy1226"
"Chl_G11_00057" 2726 "s59E13H2" 0 "hypothetical protein"
"Chl_G11_00057" 2726 "s59E25H2" 0 "hypothetical protein"
"Chl_G11_00057" 2726 "s59E5H2" 0.469881 "hypothetical protein"
"Chl_G11_00057" 2726 "s63E6H2" 0 "hypothetical protein"
"Chl_G11_00058" 2727 "s59E13H2" 0 "hypothetical protein"
"Chl_G11_00058" 2727 "s59E25H2" 0 "hypothetical protein"
"Chl_G11_00058" 2727 "s59E5H2" 1.415418 "hypothetical protein"
"Chl_G11_00058" 2727 "s63E6H2" 0 "hypothetical protein"
"Chl_G11_00059" 2728 "s59E13H2" 0 "hypothetical protein"
"Chl_G11_00059" 2728 "s59E25H2" 0 "hypothetical protein"
"Chl_G11_00059" 2728 "s59E5H2" 1.064193 "hypothetical protein"
"Chl_G11_00059" 2728 "s63E6H2" 0 "hypothetical protein"
"Chl_G11_00060" 2729 "s59E13H2" 0.00613947 "Protease 1 precursor"
"Chl_G11_00060" 2729 "s59E25H2" 0 "Protease 1 precursor"
"Chl_G11_00060" 2729 "s59E5H2" 0.1880561 "Protease 1 precursor"
"Chl_G11_00060" 2729 "s63E6H2" 0.01409314 "Protease 1 precursor"
"Chl_G11_00061" 2730 "s59E13H2" 0 "hypothetical protein"
"Chl_G11_00061" 2730 "s59E25H2" 0 "hypothetical protein"
"Chl_G11_00061" 2730 "s59E5H2" 0 "hypothetical protein"
"Chl_G11_00061" 2730 "s63E6H2" 0 "hypothetical protein"
"Chl_G11_00062" 2731 "s59E13H2" 0 "hypothetical protein"
"Chl_G11_00062" 2731 "s59E25H2" 0 "hypothetical protein"
"Chl_G11_00062" 2731 "s59E5H2" 0 "hypothetical protein"
"Chl_G11_00062" 2731 "s63E6H2" 0 "hypothetical protein"
"Chl_G11_00063" 2732 "s59E13H2" 0 "Nitronate monooxygenase"
"Chl_G11_00063" 2732 "s59E25H2" 0 "Nitronate monooxygenase"
"Chl_G11_00063" 2732 "s59E5H2" 0 "Nitronate monooxygenase"
"Chl_G11_00063" 2732 "s63E6H2" 0 "Nitronate monooxygenase"
"Chl_G11_00064" 2733 "s59E13H2" 0 "Phytoene desaturase (neurosporene-forming)"

"Chl_G11_00064" 2733 "s59E25H2" 0 "Phytoene desaturase (neurosporene-forming)"
"Chl_G11_00064" 2733 "s59E5H2" 0 "Phytoene desaturase (neurosporene-forming)"
"Chl_G11_00064" 2733 "s63E6H2" 0 "Phytoene desaturase (neurosporene-forming)"
"Chl_G11_00065" 2734 "s59E13H2" 0 "2-methylcitrate dehydratase"
"Chl_G11_00065" 2734 "s59E25H2" 0 "2-methylcitrate dehydratase"
"Chl_G11_00065" 2734 "s59E5H2" 0 "2-methylcitrate dehydratase"
"Chl_G11_00065" 2734 "s63E6H2" 0 "2-methylcitrate dehydratase"
"Chl_G11_00066" 2735 "s59E13H2" 0 "2-methylcitrate dehydratase"
"Chl_G11_00066" 2735 "s59E25H2" 0 "2-methylcitrate dehydratase"
"Chl_G11_00066" 2735 "s59E5H2" 0 "2-methylcitrate dehydratase"
"Chl_G11_00066" 2735 "s63E6H2" 0 "2-methylcitrate dehydratase"
"Chl_G11_00067" 2736 "s59E13H2" 0 "NADH oxidase"
"Chl_G11_00067" 2736 "s59E25H2" 0 "NADH oxidase"
"Chl_G11_00067" 2736 "s59E5H2" 0 "NADH oxidase"
"Chl_G11_00067" 2736 "s63E6H2" 0 "NADH oxidase"
"Chl_G11_00068" 2737 "s59E13H2" 0 "hypothetical protein"
"Chl_G11_00068" 2737 "s59E25H2" 0 "hypothetical protein"
"Chl_G11_00068" 2737 "s59E5H2" 0 "hypothetical protein"
"Chl_G11_00068" 2737 "s63E6H2" 0 "hypothetical protein"
"Chl_G11_00069" 2738 "s59E13H2" 0 "MmgE/PrpD family protein"
"Chl_G11_00069" 2738 "s59E25H2" 0 "MmgE/PrpD family protein"
"Chl_G11_00069" 2738 "s59E5H2" 0 "MmgE/PrpD family protein"
"Chl_G11_00069" 2738 "s63E6H2" 0 "MmgE/PrpD family protein"
"Chl_G11_00070" 2739 "s59E13H2" 0 "R-phenyllactate dehydratase subunit alpha precursor"
"Chl_G11_00070" 2739 "s59E25H2" 0 "R-phenyllactate dehydratase subunit alpha precursor"
"Chl_G11_00070" 2739 "s59E5H2" 0 "R-phenyllactate dehydratase subunit alpha precursor"
"Chl_G11_00070" 2739 "s63E6H2" 0 "R-phenyllactate dehydratase subunit alpha precursor"
"Chl_G11_00071" 2740 "s59E13H2" 0 "Benzoyl-CoA reductase subunit C"
"Chl_G11_00071" 2740 "s59E25H2" 0 "Benzoyl-CoA reductase subunit C"
"Chl_G11_00071" 2740 "s59E5H2" 0 "Benzoyl-CoA reductase subunit C"
"Chl_G11_00071" 2740 "s63E6H2" 0 "Benzoyl-CoA reductase subunit C"
"Chl_G11_00072" 2741 "s59E13H2" 0 "Succinyl-CoA:(R)-benzylsuccinate CoA-transferase subunit BbsF"
"Chl_G11_00072" 2741 "s59E25H2" 0 "Succinyl-CoA:(R)-benzylsuccinate CoA-transferase subunit BbsF"
"Chl_G11_00072" 2741 "s59E5H2" 0 "Succinyl-CoA:(R)-benzylsuccinate CoA-transferase subunit BbsF"
"Chl_G11_00072" 2741 "s63E6H2" 0 "Succinyl-CoA:(R)-benzylsuccinate CoA-transferase subunit BbsF"
"Chl_G11_00073" 2742 "s59E13H2" 0 "Formyl-coenzyme A transferase"
"Chl_G11_00073" 2742 "s59E25H2" 0 "Formyl-coenzyme A transferase"
"Chl_G11_00073" 2742 "s59E5H2" 0 "Formyl-coenzyme A transferase"
"Chl_G11_00073" 2742 "s63E6H2" 0 "Formyl-coenzyme A transferase"
"Chl_G11_00074" 2743 "s59E13H2" 0 "R-phenyllactate dehydratase activator"
"Chl_G11_00074" 2743 "s59E25H2" 0 "R-phenyllactate dehydratase activator"
"Chl_G11_00074" 2743 "s59E5H2" 0 "R-phenyllactate dehydratase activator"
"Chl_G11_00074" 2743 "s63E6H2" 0 "R-phenyllactate dehydratase activator"
"Chl_G11_00075" 2744 "s59E13H2" 0 "Sialic acid TRAP transporter permease protein SiaT"
"Chl_G11_00075" 2744 "s59E25H2" 0 "Sialic acid TRAP transporter permease protein SiaT"
"Chl_G11_00075" 2744 "s59E5H2" 0 "Sialic acid TRAP transporter permease protein SiaT"
"Chl_G11_00075" 2744 "s63E6H2" 0 "Sialic acid TRAP transporter permease protein SiaT"

"Chl_G11_00076" 2745 "s59E13H2" 0 "2,3-diketo-L-gulonate TRAP transporter small permease protein YiaM"
"Chl_G11_00076" 2745 "s59E25H2" 0 "2,3-diketo-L-gulonate TRAP transporter small permease protein YiaM"
"Chl_G11_00076" 2745 "s59E5H2" 0 "2,3-diketo-L-gulonate TRAP transporter small permease protein YiaM"
"Chl_G11_00076" 2745 "s63E6H2" 0 "2,3-diketo-L-gulonate TRAP transporter small permease protein YiaM"
"Chl_G11_00077" 2746 "s59E13H2" 0 "Lactate-binding periplasmic protein precursor"
"Chl_G11_00077" 2746 "s59E25H2" 0 "Lactate-binding periplasmic protein precursor"
"Chl_G11_00077" 2746 "s59E5H2" 0 "Lactate-binding periplasmic protein precursor"
"Chl_G11_00077" 2746 "s63E6H2" 0 "Lactate-binding periplasmic protein precursor"
"Chl_G11_00080" 2749 "s59E13H2" 0 "hypothetical protein"
"Chl_G11_00080" 2749 "s59E25H2" 0 "hypothetical protein"
"Chl_G11_00080" 2749 "s59E5H2" 0 "hypothetical protein"
"Chl_G11_00080" 2749 "s63E6H2" 0 "hypothetical protein"
"Chl_G11_00081" 2750 "s59E13H2" 0 "DNA-invertase hin"
"Chl_G11_00081" 2750 "s59E25H2" 0 "DNA-invertase hin"
"Chl_G11_00081" 2750 "s59E5H2" 0.00481573 "DNA-invertase hin"
"Chl_G11_00081" 2750 "s63E6H2" 0 "DNA-invertase hin"
"Chl_G11_00082" 2751 "s59E13H2" 0 "hypothetical protein"
"Chl_G11_00082" 2751 "s59E25H2" 0 "hypothetical protein"
"Chl_G11_00082" 2751 "s59E5H2" 0 "hypothetical protein"
"Chl_G11_00082" 2751 "s63E6H2" 0 "hypothetical protein"
"Chl_G11_00084" 2753 "s59E13H2" 0 "Helix-turn-helix"
"Chl_G11_00084" 2753 "s59E25H2" 0 "Helix-turn-helix"
"Chl_G11_00084" 2753 "s59E5H2" 0 "Helix-turn-helix"
"Chl_G11_00084" 2753 "s63E6H2" 0 "Helix-turn-helix"
"Chl_G11_00087" 2756 "s59E13H2" 0 "Signal transduction histidine-protein kinase/phosphatase DegS"
"Chl_G11_00087" 2756 "s59E25H2" 0 "Signal transduction histidine-protein kinase/phosphatase DegS"
"Chl_G11_00087" 2756 "s59E5H2" 0 "Signal transduction histidine-protein kinase/phosphatase DegS"
"Chl_G11_00087" 2756 "s63E6H2" 0 "Signal transduction histidine-protein kinase/phosphatase DegS"
"Chl_G11_00088" 2757 "s59E13H2" 0 "Oxygen regulatory protein NreC"
"Chl_G11_00088" 2757 "s59E25H2" 0 "Oxygen regulatory protein NreC"
"Chl_G11_00088" 2757 "s59E5H2" 0 "Oxygen regulatory protein NreC"
"Chl_G11_00088" 2757 "s63E6H2" 0 "Oxygen regulatory protein NreC"
"Chl_G11_00089" 2758 "s59E13H2" 0 "Sulfide dehydrogenase subunit alpha precursor"
"Chl_G11_00089" 2758 "s59E25H2" 0 "Sulfide dehydrogenase subunit alpha precursor"
"Chl_G11_00089" 2758 "s59E5H2" 0 "Sulfide dehydrogenase subunit alpha precursor"
"Chl_G11_00089" 2758 "s63E6H2" 0 "Sulfide dehydrogenase subunit alpha precursor"
"Chl_G11_00090" 2759 "s59E13H2" 0 "hypothetical protein"
"Chl_G11_00090" 2759 "s59E25H2" 0 "hypothetical protein"
"Chl_G11_00090" 2759 "s59E5H2" 0 "hypothetical protein"
"Chl_G11_00090" 2759 "s63E6H2" 0 "hypothetical protein"
"Chl_G11_00091" 2760 "s59E13H2" 0 "Bifunctional homocysteine S-methyltransferase/5,10-methylenetetrahydrofolate reductase"
"Chl_G11_00091" 2760 "s59E25H2" 0 "Bifunctional homocysteine S-methyltransferase/5,10-methylenetetrahydrofolate reductase"
"Chl_G11_00091" 2760 "s59E5H2" 0 "Bifunctional homocysteine S-methyltransferase/5,10-methylenetetrahydrofolate reductase"
"Chl_G11_00091" 2760 "s63E6H2" 0 "Bifunctional homocysteine S-methyltransferase/5,10-methylenetetrahydrofolate reductase"
"Chl_G11_00092" 2761 "s59E13H2" 0 "Response regulator protein VraR"
"Chl_G11_00092" 2761 "s59E25H2" 0 "Response regulator protein VraR"
"Chl_G11_00092" 2761 "s59E5H2" 0 "Response regulator protein VraR"

"Chl_G11_00092" 2761 "s63E6H2" 0 "Response regulator protein VraR"
"Chl_G11_00093" 2762 "s59E13H2" 0 "YcfA-like protein"
"Chl_G11_00093" 2762 "s59E25H2" 0 "YcfA-like protein"
"Chl_G11_00093" 2762 "s59E5H2" 0 "YcfA-like protein"
"Chl_G11_00093" 2762 "s63E6H2" 0 "YcfA-like protein"
"Chl_G11_00094" 2763 "s59E13H2" 0 "hypothetical protein"
"Chl_G11_00094" 2763 "s59E25H2" 0 "hypothetical protein"
"Chl_G11_00094" 2763 "s59E5H2" 0 "hypothetical protein"
"Chl_G11_00094" 2763 "s63E6H2" 0 "hypothetical protein"
"Chl_G11_00095" 2764 "s59E13H2" 0 "YcfA-like protein"
"Chl_G11_00095" 2764 "s59E25H2" 0 "YcfA-like protein"
"Chl_G11_00095" 2764 "s59E5H2" 0 "YcfA-like protein"
"Chl_G11_00095" 2764 "s63E6H2" 0 "YcfA-like protein"
"Chl_G11_00096" 2765 "s59E13H2" 0 "putative aldolase LsrF"
"Chl_G11_00096" 2765 "s59E25H2" 0 "putative aldolase LsrF"
"Chl_G11_00096" 2765 "s59E5H2" 0 "putative aldolase LsrF"
"Chl_G11_00096" 2765 "s63E6H2" 0 "putative aldolase LsrF"
"Chl_G11_00097" 2766 "s59E13H2" 0 "Sorbitol dehydrogenase"
"Chl_G11_00097" 2766 "s59E25H2" 0 "Sorbitol dehydrogenase"
"Chl_G11_00097" 2766 "s59E5H2" 0.009665059 "Sorbitol dehydrogenase"
"Chl_G11_00097" 2766 "s63E6H2" 0.002142641 "Sorbitol dehydrogenase"
"Chl_G11_00098" 2767 "s59E13H2" 0 "hypothetical protein"
"Chl_G11_00098" 2767 "s59E25H2" 0 "hypothetical protein"
"Chl_G11_00098" 2767 "s59E5H2" 0.002041477 "hypothetical protein"
"Chl_G11_00098" 2767 "s63E6H2" 0 "hypothetical protein"
"Chl_G11_00099" 2768 "s59E13H2" 0 "Holliday junction ATP-dependent DNA helicase RuvB"
"Chl_G11_00099" 2768 "s59E25H2" 0 "Holliday junction ATP-dependent DNA helicase RuvB"
"Chl_G11_00099" 2768 "s59E5H2" 0.1918566 "Holliday junction ATP-dependent DNA helicase RuvB"
"Chl_G11_00099" 2768 "s63E6H2" 0.001586481 "Holliday junction ATP-dependent DNA helicase RuvB"
"Chl_G11_00102" 2771 "s59E13H2" 0 "Alginate biosynthesis sensor protein KinB"
"Chl_G11_00102" 2771 "s59E25H2" 0 "Alginate biosynthesis sensor protein KinB"
"Chl_G11_00102" 2771 "s59E5H2" 0 "Alginate biosynthesis sensor protein KinB"
"Chl_G11_00102" 2771 "s63E6H2" 0 "Alginate biosynthesis sensor protein KinB"
"Chl_G11_00103" 2772 "s59E13H2" 0 "ATP-dependent helicase/deoxyribonuclease subunit B"
"Chl_G11_00103" 2772 "s59E25H2" 0 "ATP-dependent helicase/deoxyribonuclease subunit B"
"Chl_G11_00103" 2772 "s59E5H2" 0 "ATP-dependent helicase/deoxyribonuclease subunit B"
"Chl_G11_00103" 2772 "s63E6H2" 0 "ATP-dependent helicase/deoxyribonuclease subunit B"
"Chl_G11_00104" 2773 "s59E13H2" 0 "Tetratricopeptide repeat protein"
"Chl_G11_00104" 2773 "s59E25H2" 0 "Tetratricopeptide repeat protein"
"Chl_G11_00104" 2773 "s59E5H2" 0 "Tetratricopeptide repeat protein"
"Chl_G11_00104" 2773 "s63E6H2" 0 "Tetratricopeptide repeat protein"
"Chl_G11_00105" 2774 "s59E13H2" 0 "Ribosomal RNA small subunit methyltransferase G"
"Chl_G11_00105" 2774 "s59E25H2" 0 "Ribosomal RNA small subunit methyltransferase G"
"Chl_G11_00105" 2774 "s59E5H2" 0 "Ribosomal RNA small subunit methyltransferase G"
"Chl_G11_00105" 2774 "s63E6H2" 0 "Ribosomal RNA small subunit methyltransferase G"
"Chl_G11_00106" 2775 "s59E13H2" 0 "4-hydroxybutyryl-CoA dehydratase/vinylacetyl-CoA-Delta-isomerase"
"Chl_G11_00106" 2775 "s59E25H2" 0 "4-hydroxybutyryl-CoA dehydratase/vinylacetyl-CoA-Delta-isomerase"

"Chl_G11_00106" 2775 "s59E5H2" 0 "4-hydroxybutyryl-CoA dehydratase/vinylacetyl-CoA-Delta-isomerase"
"Chl_G11_00106" 2775 "s63E6H2" 0 "4-hydroxybutyryl-CoA dehydratase/vinylacetyl-CoA-Delta-isomerase"
"Chl_G11_00107" 2776 "s59E13H2" 0 "Pyridoxal biosynthesis lyase PdxS"
"Chl_G11_00107" 2776 "s59E25H2" 0 "Pyridoxal biosynthesis lyase PdxS"
"Chl_G11_00107" 2776 "s59E5H2" 0.01670801 "Pyridoxal biosynthesis lyase PdxS"
"Chl_G11_00107" 2776 "s63E6H2" 0 "Pyridoxal biosynthesis lyase PdxS"
"Chl_G11_00108" 2777 "s59E13H2" 0 "hypothetical protein"
"Chl_G11_00108" 2777 "s59E25H2" 0 "hypothetical protein"
"Chl_G11_00108" 2777 "s59E5H2" 0 "hypothetical protein"
"Chl_G11_00108" 2777 "s63E6H2" 0 "hypothetical protein"
"Chl_G11_00109" 2778 "s59E13H2" 0 "Glutamine amidotransferase subunit PdxT"
"Chl_G11_00109" 2778 "s59E25H2" 0 "Glutamine amidotransferase subunit PdxT"
"Chl_G11_00109" 2778 "s59E5H2" 0 "Glutamine amidotransferase subunit PdxT"
"Chl_G11_00109" 2778 "s63E6H2" 0 "Glutamine amidotransferase subunit PdxT"
"Chl_G11_00110" 2779 "s59E13H2" 0.001147788 "R3H domain protein"
"Chl_G11_00110" 2779 "s59E25H2" 0 "R3H domain protein"
"Chl_G11_00110" 2779 "s59E5H2" 0.009438118 "R3H domain protein"
"Chl_G11_00110" 2779 "s63E6H2" 0 "R3H domain protein"
"Chl_G11_00112" 2781 "s59E13H2" 0 "Thymidylate kinase"
"Chl_G11_00112" 2781 "s59E25H2" 0 "Thymidylate kinase"
"Chl_G11_00112" 2781 "s59E5H2" 0 "Thymidylate kinase"
"Chl_G11_00112" 2781 "s63E6H2" 0 "Thymidylate kinase"
"Chl_G11_00113" 2782 "s59E13H2" 0 "tRNA-specific 2-thiouridylase MnmA"
"Chl_G11_00113" 2782 "s59E25H2" 0 "tRNA-specific 2-thiouridylase MnmA"
"Chl_G11_00113" 2782 "s59E5H2" 0 "tRNA-specific 2-thiouridylase MnmA"
"Chl_G11_00113" 2782 "s63E6H2" 0 "tRNA-specific 2-thiouridylase MnmA"
"Chl_G11_00114" 2783 "s59E13H2" 0 "Non-heme chloroperoxidase"
"Chl_G11_00114" 2783 "s59E25H2" 0 "Non-heme chloroperoxidase"
"Chl_G11_00114" 2783 "s59E5H2" 0 "Non-heme chloroperoxidase"
"Chl_G11_00114" 2783 "s63E6H2" 0 "Non-heme chloroperoxidase"
"Chl_G11_00115" 2784 "s59E13H2" 0 "Ribonuclease HII"
"Chl_G11_00115" 2784 "s59E25H2" 0 "Ribonuclease HII"
"Chl_G11_00115" 2784 "s59E5H2" 0 "Ribonuclease HII"
"Chl_G11_00115" 2784 "s63E6H2" 0 "Ribonuclease HII"
"Chl_N02_00001" 2785 "s59E13H2" 0 "CoB--CoM heterodisulfide reductase iron-sulfur subunit D"
"Chl_N02_00001" 2785 "s59E25H2" 0 "CoB--CoM heterodisulfide reductase iron-sulfur subunit D"
"Chl_N02_00001" 2785 "s59E5H2" 0 "CoB--CoM heterodisulfide reductase iron-sulfur subunit D"
"Chl_N02_00001" 2785 "s63E6H2" 0 "CoB--CoM heterodisulfide reductase iron-sulfur subunit D"
"Chl_N02_00002" 2786 "s59E13H2" 0 "CoB--CoM heterodisulfide reductase iron-sulfur subunit A"
"Chl_N02_00002" 2786 "s59E25H2" 0 "CoB--CoM heterodisulfide reductase iron-sulfur subunit A"
"Chl_N02_00002" 2786 "s59E5H2" 0 "CoB--CoM heterodisulfide reductase iron-sulfur subunit A"
"Chl_N02_00002" 2786 "s63E6H2" 0 "CoB--CoM heterodisulfide reductase iron-sulfur subunit A"
"Chl_N02_00003" 2787 "s59E13H2" 0 "Sulfhydrogenase 1 subunit gamma"
"Chl_N02_00003" 2787 "s59E25H2" 0 "Sulfhydrogenase 1 subunit gamma"
"Chl_N02_00003" 2787 "s59E5H2" 0 "Sulfhydrogenase 1 subunit gamma"
"Chl_N02_00003" 2787 "s63E6H2" 0 "Sulfhydrogenase 1 subunit gamma"
"Chl_N02_00004" 2788 "s59E13H2" 0 "Sulfhydrogenase 2 subunit beta"

"Chl_N02_00004" 2788 "s59E25H2" 0 "Sulfhydrogenase 2 subunit beta"
"Chl_N02_00004" 2788 "s59E5H2" 0 "Sulfhydrogenase 2 subunit beta"
"Chl_N02_00004" 2788 "s63E6H2" 0 "Sulfhydrogenase 2 subunit beta"
"Chl_N02_00005" 2789 "s59E13H2" 0 "Formate dehydrogenase subunit beta"
"Chl_N02_00005" 2789 "s59E25H2" 0 "Formate dehydrogenase subunit beta"
"Chl_N02_00005" 2789 "s59E5H2" 0 "Formate dehydrogenase subunit beta"
"Chl_N02_00005" 2789 "s63E6H2" 0 "Formate dehydrogenase subunit beta"
"Chl_N02_00006" 2790 "s59E13H2" 0 "F420-non-reducing hydrogenase iron-sulfur subunit D"
"Chl_N02_00006" 2790 "s59E25H2" 0 "F420-non-reducing hydrogenase iron-sulfur subunit D"
"Chl_N02_00006" 2790 "s59E5H2" 0 "F420-non-reducing hydrogenase iron-sulfur subunit D"
"Chl_N02_00006" 2790 "s63E6H2" 0 "F420-non-reducing hydrogenase iron-sulfur subunit D"
"Chl_N02_00007" 2791 "s59E13H2" 0 "fused phosphoenolpyruvate-protein phosphotransferase PtsP/GAF domain protein"
"Chl_N02_00007" 2791 "s59E25H2" 0 "fused phosphoenolpyruvate-protein phosphotransferase PtsP/GAF domain protein"
"Chl_N02_00007" 2791 "s59E5H2" 0 "fused phosphoenolpyruvate-protein phosphotransferase PtsP/GAF domain protein"
"Chl_N02_00007" 2791 "s63E6H2" 0 "fused phosphoenolpyruvate-protein phosphotransferase PtsP/GAF domain protein"
"Chl_N02_00008" 2792 "s59E13H2" 0 "CoB--CoM heterodisulfide reductase iron-sulfur subunit D"
"Chl_N02_00008" 2792 "s59E25H2" 0 "CoB--CoM heterodisulfide reductase iron-sulfur subunit D"
"Chl_N02_00008" 2792 "s59E5H2" 0 "CoB--CoM heterodisulfide reductase iron-sulfur subunit D"
"Chl_N02_00008" 2792 "s63E6H2" 0 "CoB--CoM heterodisulfide reductase iron-sulfur subunit D"
"Chl_N02_00009" 2793 "s59E13H2" 0 "CoB--CoM heterodisulfide reductase iron-sulfur subunit A"
"Chl_N02_00009" 2793 "s59E25H2" 0 "CoB--CoM heterodisulfide reductase iron-sulfur subunit A"
"Chl_N02_00009" 2793 "s59E5H2" 0 "CoB--CoM heterodisulfide reductase iron-sulfur subunit A"
"Chl_N02_00009" 2793 "s63E6H2" 0 "CoB--CoM heterodisulfide reductase iron-sulfur subunit A"
"Chl_N02_00010" 2794 "s59E13H2" 0 "NADP-reducing hydrogenase subunit HndA"
"Chl_N02_00010" 2794 "s59E25H2" 0 "NADP-reducing hydrogenase subunit HndA"
"Chl_N02_00010" 2794 "s59E5H2" 0 "NADP-reducing hydrogenase subunit HndA"
"Chl_N02_00010" 2794 "s63E6H2" 0 "NADP-reducing hydrogenase subunit HndA"
"Chl_N02_00011" 2795 "s59E13H2" 0 "NADP-reducing hydrogenase subunit HndC"
"Chl_N02_00011" 2795 "s59E25H2" 0 "NADP-reducing hydrogenase subunit HndC"
"Chl_N02_00011" 2795 "s59E5H2" 0 "NADP-reducing hydrogenase subunit HndC"
"Chl_N02_00011" 2795 "s63E6H2" 0 "NADP-reducing hydrogenase subunit HndC"
"Chl_N02_00012" 2796 "s59E13H2" 0 "CoB--CoM heterodisulfide reductase iron-sulfur subunit A"
"Chl_N02_00012" 2796 "s59E25H2" 0 "CoB--CoM heterodisulfide reductase iron-sulfur subunit A"
"Chl_N02_00012" 2796 "s59E5H2" 0 "CoB--CoM heterodisulfide reductase iron-sulfur subunit A"
"Chl_N02_00012" 2796 "s63E6H2" 0 "CoB--CoM heterodisulfide reductase iron-sulfur subunit A"
"Chl_N02_00013" 2797 "s59E13H2" 0 "F420-non-reducing hydrogenase vhc iron-sulfur subunit D"
"Chl_N02_00013" 2797 "s59E25H2" 0 "F420-non-reducing hydrogenase vhc iron-sulfur subunit D"
"Chl_N02_00013" 2797 "s59E5H2" 0 "F420-non-reducing hydrogenase vhc iron-sulfur subunit D"
"Chl_N02_00013" 2797 "s63E6H2" 0 "F420-non-reducing hydrogenase vhc iron-sulfur subunit D"
"Chl_N02_00015" 2799 "s59E13H2" 0 "F420-non-reducing hydrogenase subunit G"
"Chl_N02_00015" 2799 "s59E25H2" 0 "F420-non-reducing hydrogenase subunit G"
"Chl_N02_00015" 2799 "s59E5H2" 0 "F420-non-reducing hydrogenase subunit G"
"Chl_N02_00015" 2799 "s63E6H2" 0 "F420-non-reducing hydrogenase subunit G"
"Chl_N02_00016" 2800 "s59E13H2" 0 "F420-non-reducing hydrogenase subunit A"
"Chl_N02_00016" 2800 "s59E25H2" 0 "F420-non-reducing hydrogenase subunit A"
"Chl_N02_00016" 2800 "s59E5H2" 0 "F420-non-reducing hydrogenase subunit A"
"Chl_N02_00016" 2800 "s63E6H2" 0 "F420-non-reducing hydrogenase subunit A"

"Chl_N02_00017" 2801 "s59E13H2" 0 "Hydrogenase 2 maturation protease"
"Chl_N02_00017" 2801 "s59E25H2" 0 "Hydrogenase 2 maturation protease"
"Chl_N02_00017" 2801 "s59E5H2" 0 "Hydrogenase 2 maturation protease"
"Chl_N02_00017" 2801 "s63E6H2" 0 "Hydrogenase 2 maturation protease"
"Chl_N02_00018" 2802 "s59E13H2" 0 "Electron transfer flavoprotein subunit beta"
"Chl_N02_00018" 2802 "s59E25H2" 0 "Electron transfer flavoprotein subunit beta"
"Chl_N02_00018" 2802 "s59E5H2" 0 "Electron transfer flavoprotein subunit beta"
"Chl_N02_00018" 2802 "s63E6H2" 0 "Electron transfer flavoprotein subunit beta"
"Chl_N02_00019" 2803 "s59E13H2" 0 "Acryloyl-CoA reductase electron transfer subunit beta"
"Chl_N02_00019" 2803 "s59E25H2" 0 "Acryloyl-CoA reductase electron transfer subunit beta"
"Chl_N02_00019" 2803 "s59E5H2" 0 "Acryloyl-CoA reductase electron transfer subunit beta"
"Chl_N02_00019" 2803 "s63E6H2" 0 "Acryloyl-CoA reductase electron transfer subunit beta"
"Chl_N02_00020" 2804 "s59E13H2" 0 "putative sensor histidine kinase TcrY"
"Chl_N02_00020" 2804 "s59E25H2" 0 "putative sensor histidine kinase TcrY"
"Chl_N02_00020" 2804 "s59E5H2" 0 "putative sensor histidine kinase TcrY"
"Chl_N02_00020" 2804 "s63E6H2" 0 "putative sensor histidine kinase TcrY"
"Chl_N02_00021" 2805 "s59E13H2" 0 "Response regulator MprA"
"Chl_N02_00021" 2805 "s59E25H2" 0 "Response regulator MprA"
"Chl_N02_00021" 2805 "s59E5H2" 0 "Response regulator MprA"
"Chl_N02_00021" 2805 "s63E6H2" 0 "Response regulator MprA"
"Chl_N02_00022" 2806 "s59E13H2" 0 "Collagen triple helix repeat (20 copies)"
"Chl_N02_00022" 2806 "s59E25H2" 0 "Collagen triple helix repeat (20 copies)"
"Chl_N02_00022" 2806 "s59E5H2" 0 "Collagen triple helix repeat (20 copies)"
"Chl_N02_00022" 2806 "s63E6H2" 0 "Collagen triple helix repeat (20 copies)"
"Chl_N02_00023" 2807 "s59E13H2" 0 "hypothetical protein"
"Chl_N02_00023" 2807 "s59E25H2" 0 "hypothetical protein"
"Chl_N02_00023" 2807 "s59E5H2" 0 "hypothetical protein"
"Chl_N02_00023" 2807 "s63E6H2" 0 "hypothetical protein"
"Chl_N02_00026" 2810 "s59E13H2" 0 "Recombinase"
"Chl_N02_00026" 2810 "s59E25H2" 0 "Recombinase"
"Chl_N02_00026" 2810 "s59E5H2" 0 "Recombinase"
"Chl_N02_00026" 2810 "s63E6H2" 0 "Recombinase"
"Chl_N02_00028" 2812 "s59E13H2" 0 "30S ribosomal protein S6"
"Chl_N02_00028" 2812 "s59E25H2" 0 "30S ribosomal protein S6"
"Chl_N02_00028" 2812 "s59E5H2" 0 "30S ribosomal protein S6"
"Chl_N02_00028" 2812 "s63E6H2" 0 "30S ribosomal protein S6"
"Chl_N02_00029" 2813 "s59E13H2" 0 "Single-stranded DNA-binding protein"
"Chl_N02_00029" 2813 "s59E25H2" 0 "Single-stranded DNA-binding protein"
"Chl_N02_00029" 2813 "s59E5H2" 0 "Single-stranded DNA-binding protein"
"Chl_N02_00029" 2813 "s63E6H2" 0 "Single-stranded DNA-binding protein"
"Chl_N02_00030" 2814 "s59E13H2" 0 "30S ribosomal protein S18"
"Chl_N02_00030" 2814 "s59E25H2" 0 "30S ribosomal protein S18"
"Chl_N02_00030" 2814 "s59E5H2" 0 "30S ribosomal protein S18"
"Chl_N02_00030" 2814 "s63E6H2" 0 "30S ribosomal protein S18"
"Chl_N02_00031" 2815 "s59E13H2" 0 "Ribonuclease P protein component"
"Chl_N02_00031" 2815 "s59E25H2" 0 "Ribonuclease P protein component"
"Chl_N02_00031" 2815 "s59E5H2" 0 "Ribonuclease P protein component"

"Chl_N02_00031" 2815 "s63E6H2" 0 "Ribonuclease P protein component"
"Chl_N02_00032" 2816 "s59E13H2" 0 "Putative membrane protein insertion efficiency factor"
"Chl_N02_00032" 2816 "s59E25H2" 0 "Putative membrane protein insertion efficiency factor"
"Chl_N02_00032" 2816 "s59E5H2" 0 "Putative membrane protein insertion efficiency factor"
"Chl_N02_00032" 2816 "s63E6H2" 0 "Putative membrane protein insertion efficiency factor"
"Chl_N02_00033" 2817 "s59E13H2" 0 "Serine/threonine-protein kinase AfsK"
"Chl_N02_00033" 2817 "s59E25H2" 0 "Serine/threonine-protein kinase AfsK"
"Chl_N02_00033" 2817 "s59E5H2" 0 "Serine/threonine-protein kinase AfsK"
"Chl_N02_00033" 2817 "s63E6H2" 0 "Serine/threonine-protein kinase AfsK"
"Chl_N02_00034" 2818 "s59E13H2" 0 "Membrane protein insertase YidC"
"Chl_N02_00034" 2818 "s59E25H2" 0 "Membrane protein insertase YidC"
"Chl_N02_00034" 2818 "s59E5H2" 0 "Membrane protein insertase YidC"
"Chl_N02_00034" 2818 "s63E6H2" 0 "Membrane protein insertase YidC"
"Chl_N02_00035" 2819 "s59E13H2" 0 "R3H domain protein"
"Chl_N02_00035" 2819 "s59E25H2" 0 "R3H domain protein"
"Chl_N02_00035" 2819 "s59E5H2" 0 "R3H domain protein"
"Chl_N02_00035" 2819 "s63E6H2" 0 "R3H domain protein"
"Chl_N02_00036" 2820 "s59E13H2" 0 "Isoleucine--tRNA ligase"
"Chl_N02_00036" 2820 "s59E25H2" 0 "Isoleucine--tRNA ligase"
"Chl_N02_00036" 2820 "s59E5H2" 0 "Isoleucine--tRNA ligase"
"Chl_N02_00036" 2820 "s63E6H2" 0 "Isoleucine--tRNA ligase"
"Chl_N02_00037" 2821 "s59E13H2" 0 "Putative serine protease HtrA"
"Chl_N02_00037" 2821 "s59E25H2" 0 "Putative serine protease HtrA"
"Chl_N02_00037" 2821 "s59E5H2" 0 "Putative serine protease HtrA"
"Chl_N02_00037" 2821 "s63E6H2" 0 "Putative serine protease HtrA"
"Chl_N02_00038" 2822 "s59E13H2" 0 "Tryptophan synthase beta chain 1"
"Chl_N02_00038" 2822 "s59E25H2" 0 "Tryptophan synthase beta chain 1"
"Chl_N02_00038" 2822 "s59E5H2" 0 "Tryptophan synthase beta chain 1"
"Chl_N02_00038" 2822 "s63E6H2" 0 "Tryptophan synthase beta chain 1"
"Chl_N02_00039" 2823 "s59E13H2" 0 "Tryptophan synthase alpha chain"
"Chl_N02_00039" 2823 "s59E25H2" 0 "Tryptophan synthase alpha chain"
"Chl_N02_00039" 2823 "s59E5H2" 0 "Tryptophan synthase alpha chain"
"Chl_N02_00039" 2823 "s63E6H2" 0 "Tryptophan synthase alpha chain"
"Chl_N02_00040" 2824 "s59E13H2" 0 "Tryptophan synthase beta chain"
"Chl_N02_00040" 2824 "s59E25H2" 0 "Tryptophan synthase beta chain"
"Chl_N02_00040" 2824 "s59E5H2" 0.01008192 "Tryptophan synthase beta chain"
"Chl_N02_00040" 2824 "s63E6H2" 0 "Tryptophan synthase beta chain"
"Chl_N02_00041" 2825 "s59E13H2" 0 "N-(5'-phosphoribosyl)anthranilate isomerase"
"Chl_N02_00041" 2825 "s59E25H2" 0 "N-(5'-phosphoribosyl)anthranilate isomerase"
"Chl_N02_00041" 2825 "s59E5H2" 0 "N-(5'-phosphoribosyl)anthranilate isomerase"
"Chl_N02_00041" 2825 "s63E6H2" 0 "N-(5'-phosphoribosyl)anthranilate isomerase"
"Chl_N02_00042" 2826 "s59E13H2" 0 "NADP-reducing hydrogenase subunit HndA"
"Chl_N02_00042" 2826 "s59E25H2" 0 "NADP-reducing hydrogenase subunit HndA"
"Chl_N02_00042" 2826 "s59E5H2" 0 "NADP-reducing hydrogenase subunit HndA"
"Chl_N02_00042" 2826 "s63E6H2" 0 "NADP-reducing hydrogenase subunit HndA"
"Chl_N02_00043" 2827 "s59E13H2" 0 "L-2-hydroxyglutarate oxidase LhgO"
"Chl_N02_00043" 2827 "s59E25H2" 0 "L-2-hydroxyglutarate oxidase LhgO"

"Chl_N02_00043" 2827 "s59E5H2" 0 "L-2-hydroxyglutarate oxidase LhgO"
"Chl_N02_00043" 2827 "s63E6H2" 0 "L-2-hydroxyglutarate oxidase LhgO"
"Chl_N02_00046" 2830 "s59E13H2" 0 "hypothetical protein"
"Chl_N02_00046" 2830 "s59E25H2" 0 "hypothetical protein"
"Chl_N02_00046" 2830 "s59E5H2" 0 "hypothetical protein"
"Chl_N02_00046" 2830 "s63E6H2" 0 "hypothetical protein"
"Chl_N02_00047" 2831 "s59E13H2" 0 "hypothetical protein"
"Chl_N02_00047" 2831 "s59E25H2" 0 "hypothetical protein"
"Chl_N02_00047" 2831 "s59E5H2" 0.007318103 "hypothetical protein"
"Chl_N02_00047" 2831 "s63E6H2" 0 "hypothetical protein"
"Chl_N02_00048" 2832 "s59E13H2" 0.001323362 "Ribosomal protein S12 methylthiotransferase RimO"
"Chl_N02_00048" 2832 "s59E25H2" 0 "Ribosomal protein S12 methylthiotransferase RimO"
"Chl_N02_00048" 2832 "s59E5H2" 0.01807472 "Ribosomal protein S12 methylthiotransferase RimO"
"Chl_N02_00048" 2832 "s63E6H2" 0.5403443 "Ribosomal protein S12 methylthiotransferase RimO"
"Chl_N02_00049" 2833 "s59E13H2" 0 "GTPase ObgE"
"Chl_N02_00049" 2833 "s59E25H2" 0 "GTPase ObgE"
"Chl_N02_00049" 2833 "s59E5H2" 0.01948839 "GTPase ObgE"
"Chl_N02_00049" 2833 "s63E6H2" 0 "GTPase ObgE"
"Chl_N02_00050" 2834 "s59E13H2" 0 "UvrABC system protein C"
"Chl_N02_00050" 2834 "s59E25H2" 0 "UvrABC system protein C"
"Chl_N02_00050" 2834 "s59E5H2" 0 "UvrABC system protein C"
"Chl_N02_00050" 2834 "s63E6H2" 0 "UvrABC system protein C"
"Chl_N02_00051" 2835 "s59E13H2" 0 "hypothetical protein"
"Chl_N02_00051" 2835 "s59E25H2" 0 "hypothetical protein"
"Chl_N02_00051" 2835 "s59E5H2" 0 "hypothetical protein"
"Chl_N02_00051" 2835 "s63E6H2" 0 "hypothetical protein"
"Chl_N02_00052" 2836 "s59E13H2" 0 "hypothetical protein"
"Chl_N02_00052" 2836 "s59E25H2" 0 "hypothetical protein"
"Chl_N02_00052" 2836 "s59E5H2" 0 "hypothetical protein"
"Chl_N02_00052" 2836 "s63E6H2" 0 "hypothetical protein"
"Chl_N02_00053" 2837 "s59E13H2" 0 "Cysteine--tRNA ligase"
"Chl_N02_00053" 2837 "s59E25H2" 0 "Cysteine--tRNA ligase"
"Chl_N02_00053" 2837 "s59E5H2" 0 "Cysteine--tRNA ligase"
"Chl_N02_00053" 2837 "s63E6H2" 0.0005863882 "Cysteine--tRNA ligase"
"Chl_N02_00054" 2838 "s59E13H2" 0 "2-C-methyl-D-erythritol 2,4-cyclodiphosphate synthase"
"Chl_N02_00054" 2838 "s59E25H2" 0 "2-C-methyl-D-erythritol 2,4-cyclodiphosphate synthase"
"Chl_N02_00054" 2838 "s59E5H2" 0 "2-C-methyl-D-erythritol 2,4-cyclodiphosphate synthase"
"Chl_N02_00054" 2838 "s63E6H2" 0 "2-C-methyl-D-erythritol 2,4-cyclodiphosphate synthase"
"Chl_N02_00055" 2839 "s59E13H2" 0 "2-C-methyl-D-erythritol 4-phosphate cytidylyltransferase"
"Chl_N02_00055" 2839 "s59E25H2" 0 "2-C-methyl-D-erythritol 4-phosphate cytidylyltransferase"
"Chl_N02_00055" 2839 "s59E5H2" 0 "2-C-methyl-D-erythritol 4-phosphate cytidylyltransferase"
"Chl_N02_00055" 2839 "s63E6H2" 0 "2-C-methyl-D-erythritol 4-phosphate cytidylyltransferase"
"Chl_N02_00056" 2840 "s59E13H2" 0 "Cupin domain protein"
"Chl_N02_00056" 2840 "s59E25H2" 0 "Cupin domain protein"
"Chl_N02_00056" 2840 "s59E5H2" 0 "Cupin domain protein"
"Chl_N02_00056" 2840 "s63E6H2" 0 "Cupin domain protein"
"Chl_N02_00057" 2841 "s59E13H2" 0 "hypothetical protein"

"Chl_N02_00057" 2841 "s59E25H2" 0 "hypothetical protein"
"Chl_N02_00057" 2841 "s59E5H2" 0 "hypothetical protein"
"Chl_N02_00057" 2841 "s63E6H2" 0 "hypothetical protein"
"Chl_N02_00058" 2842 "s59E13H2" 0 "Glutaconyl-CoA decarboxylase subunit beta"
"Chl_N02_00058" 2842 "s59E25H2" 0 "Glutaconyl-CoA decarboxylase subunit beta"
"Chl_N02_00058" 2842 "s59E5H2" 0 "Glutaconyl-CoA decarboxylase subunit beta"
"Chl_N02_00058" 2842 "s63E6H2" 0 "Glutaconyl-CoA decarboxylase subunit beta"
"Chl_N02_00059" 2843 "s59E13H2" 0 "Glutaconyl-CoA decarboxylase subunit gamma"
"Chl_N02_00059" 2843 "s59E25H2" 0 "Glutaconyl-CoA decarboxylase subunit gamma"
"Chl_N02_00059" 2843 "s59E5H2" 0 "Glutaconyl-CoA decarboxylase subunit gamma"
"Chl_N02_00059" 2843 "s63E6H2" 0 "Glutaconyl-CoA decarboxylase subunit gamma"
"Chl_N02_00060" 2844 "s59E13H2" 0 "hypothetical protein"
"Chl_N02_00060" 2844 "s59E25H2" 0 "hypothetical protein"
"Chl_N02_00060" 2844 "s59E5H2" 0 "hypothetical protein"
"Chl_N02_00060" 2844 "s63E6H2" 0 "hypothetical protein"
"Chl_N02_00061" 2845 "s59E13H2" 0 "Negative regulator of genetic competence ClpC/MecB"
"Chl_N02_00061" 2845 "s59E25H2" 0 "Negative regulator of genetic competence ClpC/MecB"
"Chl_N02_00061" 2845 "s59E5H2" 0.01808964 "Negative regulator of genetic competence ClpC/MecB"
"Chl_N02_00061" 2845 "s63E6H2" 0.0006032507 "Negative regulator of genetic competence ClpC/MecB"
"Chl_N02_00063" 2847 "s59E13H2" 0 "hypothetical protein"
"Chl_N02_00063" 2847 "s59E25H2" 0 "hypothetical protein"
"Chl_N02_00063" 2847 "s59E5H2" 0 "hypothetical protein"
"Chl_N02_00063" 2847 "s63E6H2" 0 "hypothetical protein"
"Chl_N02_00067" 2851 "s59E13H2" 0 "Heat-inducible transcription repressor HrcA"
"Chl_N02_00067" 2851 "s59E25H2" 0 "Heat-inducible transcription repressor HrcA"
"Chl_N02_00067" 2851 "s59E5H2" 0 "Heat-inducible transcription repressor HrcA"
"Chl_N02_00067" 2851 "s63E6H2" 0 "Heat-inducible transcription repressor HrcA"
"Chl_N02_00068" 2852 "s59E13H2" 0 "heat shock protein GrpE"
"Chl_N02_00068" 2852 "s59E25H2" 0 "heat shock protein GrpE"
"Chl_N02_00068" 2852 "s59E5H2" 0 "heat shock protein GrpE"
"Chl_N02_00068" 2852 "s63E6H2" 0 "heat shock protein GrpE"
"Chl_N02_00069" 2853 "s59E13H2" 0 "Chaperone protein DnaK"
"Chl_N02_00069" 2853 "s59E25H2" 0 "Chaperone protein DnaK"
"Chl_N02_00069" 2853 "s59E5H2" 0 "Chaperone protein DnaK"
"Chl_N02_00069" 2853 "s63E6H2" 0.004263031 "Chaperone protein DnaK"
"Chl_N02_00070" 2854 "s59E13H2" 0 "Chaperone protein DnaJ"
"Chl_N02_00070" 2854 "s59E25H2" 0 "Chaperone protein DnaJ"
"Chl_N02_00070" 2854 "s59E5H2" 0 "Chaperone protein DnaJ"
"Chl_N02_00070" 2854 "s63E6H2" 0 "Chaperone protein DnaJ"
"Chl_N02_00071" 2855 "s59E13H2" 0 "Glycerol-3-phosphate dehydrogenase [NAD(P)+]"
"Chl_N02_00071" 2855 "s59E25H2" 0 "Glycerol-3-phosphate dehydrogenase [NAD(P)+]"
"Chl_N02_00071" 2855 "s59E5H2" 0 "Glycerol-3-phosphate dehydrogenase [NAD(P)+]"
"Chl_N02_00071" 2855 "s63E6H2" 0 "Glycerol-3-phosphate dehydrogenase [NAD(P)+]"
"Chl_N02_00072" 2856 "s59E13H2" 0 "Glycerol-3-phosphate acyltransferase"
"Chl_N02_00072" 2856 "s59E25H2" 0 "Glycerol-3-phosphate acyltransferase"
"Chl_N02_00072" 2856 "s59E5H2" 0 "Glycerol-3-phosphate acyltransferase"
"Chl_N02_00072" 2856 "s63E6H2" 0 "Glycerol-3-phosphate acyltransferase"

"Chl_N02_00073" 2857 "s59E13H2" 0 "GTPase Der"
"Chl_N02_00073" 2857 "s59E25H2" 0 "GTPase Der"
"Chl_N02_00073" 2857 "s59E5H2" 0 "GTPase Der"
"Chl_N02_00073" 2857 "s63E6H2" 0 "GTPase Der"
"Chl_N02_00074" 2858 "s59E13H2" 0 "hypothetical protein"
"Chl_N02_00074" 2858 "s59E25H2" 0 "hypothetical protein"
"Chl_N02_00074" 2858 "s59E5H2" 0 "hypothetical protein"
"Chl_N02_00074" 2858 "s63E6H2" 0 "hypothetical protein"
"Chl_N02_00075" 2859 "s59E13H2" 0 "Xaa-Pro dipeptidase"
"Chl_N02_00075" 2859 "s59E25H2" 0 "Xaa-Pro dipeptidase"
"Chl_N02_00075" 2859 "s59E5H2" 0.03387952 "Xaa-Pro dipeptidase"
"Chl_N02_00075" 2859 "s63E6H2" 0 "Xaa-Pro dipeptidase"
"Chl_N02_00076" 2860 "s59E13H2" 0 "Elongation factor P"
"Chl_N02_00076" 2860 "s59E25H2" 0 "Elongation factor P"
"Chl_N02_00076" 2860 "s59E5H2" 0.07754322 "Elongation factor P"
"Chl_N02_00076" 2860 "s63E6H2" 0 "Elongation factor P"
"Chl_N02_00077" 2861 "s59E13H2" 0 "putative hydrolase"
"Chl_N02_00077" 2861 "s59E25H2" 0 "putative hydrolase"
"Chl_N02_00077" 2861 "s59E5H2" 0.01118309 "putative hydrolase"
"Chl_N02_00077" 2861 "s63E6H2" 0 "putative hydrolase"
"Chl_N02_00078" 2862 "s59E13H2" 0 "Phosphoribosylglycinamide formyltransferase"
"Chl_N02_00078" 2862 "s59E25H2" 0 "Phosphoribosylglycinamide formyltransferase"
"Chl_N02_00078" 2862 "s59E5H2" 0.1793705 "Phosphoribosylglycinamide formyltransferase"
"Chl_N02_00078" 2862 "s63E6H2" 0 "Phosphoribosylglycinamide formyltransferase"
"Chl_N02_00080" 2864 "s59E13H2" 0 "hypothetical protein"
"Chl_N02_00080" 2864 "s59E25H2" 0 "hypothetical protein"
"Chl_N02_00080" 2864 "s59E5H2" 0 "hypothetical protein"
"Chl_N02_00080" 2864 "s63E6H2" 0 "hypothetical protein"
"Chl_N02_00081" 2865 "s59E13H2" 0 "Glyoxylate reductase"
"Chl_N02_00081" 2865 "s59E25H2" 0 "Glyoxylate reductase"
"Chl_N02_00081" 2865 "s59E5H2" 0 "Glyoxylate reductase"
"Chl_N02_00081" 2865 "s63E6H2" 0 "Glyoxylate reductase"
"Chl_N02_00082" 2866 "s59E13H2" 0 "4-hydroxy-tetrahydrodipicolinate synthase"
"Chl_N02_00082" 2866 "s59E25H2" 0 "4-hydroxy-tetrahydrodipicolinate synthase"
"Chl_N02_00082" 2866 "s59E5H2" 0 "4-hydroxy-tetrahydrodipicolinate synthase"
"Chl_N02_00082" 2866 "s63E6H2" 0 "4-hydroxy-tetrahydrodipicolinate synthase"
"Chl_N02_00083" 2867 "s59E13H2" 0 "putative sugar kinase YdjH"
"Chl_N02_00083" 2867 "s59E25H2" 0 "putative sugar kinase YdjH"
"Chl_N02_00083" 2867 "s59E5H2" 0.4130324 "putative sugar kinase YdjH"
"Chl_N02_00083" 2867 "s63E6H2" 0 "putative sugar kinase YdjH"
"Chl_N02_00084" 2868 "s59E13H2" 0 "hypothetical protein"
"Chl_N02_00084" 2868 "s59E25H2" 0 "hypothetical protein"
"Chl_N02_00084" 2868 "s59E5H2" 1.066682 "hypothetical protein"
"Chl_N02_00084" 2868 "s63E6H2" 0 "hypothetical protein"
"Chl_N02_00085" 2869 "s59E13H2" 0 "hypothetical protein"
"Chl_N02_00085" 2869 "s59E25H2" 0 "hypothetical protein"
"Chl_N02_00085" 2869 "s59E5H2" 0.6219727 "hypothetical protein"

"Chl_N02_00085" 2869 "s63E6H2" 0 "hypothetical protein"
"Chl_N02_00086" 2870 "s59E13H2" 0 "ATP-dependent Clp protease proteolytic subunit"
"Chl_N02_00086" 2870 "s59E25H2" 0 "ATP-dependent Clp protease proteolytic subunit"
"Chl_N02_00086" 2870 "s59E5H2" 0 "ATP-dependent Clp protease proteolytic subunit"
"Chl_N02_00086" 2870 "s63E6H2" 0 "ATP-dependent Clp protease proteolytic subunit"
"Chl_N02_00087" 2871 "s59E13H2" 0 "Trigger factor"
"Chl_N02_00087" 2871 "s59E25H2" 0 "Trigger factor"
"Chl_N02_00087" 2871 "s59E5H2" 0 "Trigger factor"
"Chl_N02_00087" 2871 "s63E6H2" 0 "Trigger factor"
"Chl_N02_00088" 2872 "s59E13H2" 0 "Aspartate--tRNA ligase"
"Chl_N02_00088" 2872 "s59E25H2" 0 "Aspartate--tRNA ligase"
"Chl_N02_00088" 2872 "s59E5H2" 0.04839162 "Aspartate--tRNA ligase"
"Chl_N02_00088" 2872 "s63E6H2" 0 "Aspartate--tRNA ligase"
"Chl_N02_00089" 2873 "s59E13H2" 0 "hypothetical protein"
"Chl_N02_00089" 2873 "s59E25H2" 0 "hypothetical protein"
"Chl_N02_00089" 2873 "s59E5H2" 0.02002105 "hypothetical protein"
"Chl_N02_00089" 2873 "s63E6H2" 0 "hypothetical protein"
"Chl_N02_00090" 2874 "s59E13H2" 0 "(R)-citramalate synthase CimA"
"Chl_N02_00090" 2874 "s59E25H2" 0 "(R)-citramalate synthase CimA"
"Chl_N02_00090" 2874 "s59E5H2" 0.01486839 "(R)-citramalate synthase CimA"
"Chl_N02_00090" 2874 "s63E6H2" 0.001007491 "(R)-citramalate synthase CimA"
"Chl_N02_00091" 2875 "s59E13H2" 0 "Glyoxylate reductase"
"Chl_N02_00091" 2875 "s59E25H2" 0 "Glyoxylate reductase"
"Chl_N02_00091" 2875 "s59E5H2" 0.01171377 "Glyoxylate reductase"
"Chl_N02_00091" 2875 "s63E6H2" 0 "Glyoxylate reductase"
"Chl_N02_00092" 2876 "s59E13H2" 0 "3-isopropylmalate dehydrogenase"
"Chl_N02_00092" 2876 "s59E25H2" 0 "3-isopropylmalate dehydrogenase"
"Chl_N02_00092" 2876 "s59E5H2" 0 "3-isopropylmalate dehydrogenase"
"Chl_N02_00092" 2876 "s63E6H2" 0 "3-isopropylmalate dehydrogenase"
"Chl_N02_00093" 2877 "s59E13H2" 0 "2,3-dimethylmalate dehydratase small subunit"
"Chl_N02_00093" 2877 "s59E25H2" 0 "2,3-dimethylmalate dehydratase small subunit"
"Chl_N02_00093" 2877 "s59E5H2" 0 "2,3-dimethylmalate dehydratase small subunit"
"Chl_N02_00093" 2877 "s63E6H2" 0 "2,3-dimethylmalate dehydratase small subunit"
"Chl_N02_00094" 2878 "s59E13H2" 0 "hypothetical protein"
"Chl_N02_00094" 2878 "s59E25H2" 0 "hypothetical protein"
"Chl_N02_00094" 2878 "s59E5H2" 0 "hypothetical protein"
"Chl_N02_00094" 2878 "s63E6H2" 0 "hypothetical protein"
"Chl_N02_00096" 2880 "s59E13H2" 0 "Acetyltransferase (GNAT) family protein"
"Chl_N02_00096" 2880 "s59E25H2" 0 "Acetyltransferase (GNAT) family protein"
"Chl_N02_00096" 2880 "s59E5H2" 0 "Acetyltransferase (GNAT) family protein"
"Chl_N02_00096" 2880 "s63E6H2" 0 "Acetyltransferase (GNAT) family protein"
"Chl_N02_00097" 2881 "s59E13H2" 0 "2,3-dimethylmalate dehydratase large subunit"
"Chl_N02_00097" 2881 "s59E25H2" 0 "2,3-dimethylmalate dehydratase large subunit"
"Chl_N02_00097" 2881 "s59E5H2" 0 "2,3-dimethylmalate dehydratase large subunit"
"Chl_N02_00097" 2881 "s63E6H2" 0 "2,3-dimethylmalate dehydratase large subunit"
"Chl_N02_00098" 2882 "s59E13H2" 0 "2-isopropylmalate synthase"
"Chl_N02_00098" 2882 "s59E25H2" 0 "2-isopropylmalate synthase"

"Chl_N02_00098" 2882 "s59E5H2" 0 "2-isopropylmalate synthase"
"Chl_N02_00098" 2882 "s63E6H2" 0 "2-isopropylmalate synthase"
"Chl_N02_00099" 2883 "s59E13H2" 0 "Ketol-acid reductoisomerase"
"Chl_N02_00099" 2883 "s59E25H2" 0 "Ketol-acid reductoisomerase"
"Chl_N02_00099" 2883 "s59E5H2" 0 "Ketol-acid reductoisomerase"
"Chl_N02_00099" 2883 "s63E6H2" 0 "Ketol-acid reductoisomerase"
"Chl_N02_00100" 2884 "s59E13H2" 0 "Putative acetolactate synthase small subunit"
"Chl_N02_00100" 2884 "s59E25H2" 0 "Putative acetolactate synthase small subunit"
"Chl_N02_00100" 2884 "s59E5H2" 0 "Putative acetolactate synthase small subunit"
"Chl_N02_00100" 2884 "s63E6H2" 0 "Putative acetolactate synthase small subunit"
"Chl_N02_00101" 2885 "s59E13H2" 0 "Acetolactate synthase large subunit IlvB1"
"Chl_N02_00101" 2885 "s59E25H2" 0 "Acetolactate synthase large subunit IlvB1"
"Chl_N02_00101" 2885 "s59E5H2" 0 "Acetolactate synthase large subunit IlvB1"
"Chl_N02_00101" 2885 "s63E6H2" 0 "Acetolactate synthase large subunit IlvB1"
"Chl_N02_00102" 2886 "s59E13H2" 0.003128972 "Dihydroxy-acid dehydratase"
"Chl_N02_00102" 2886 "s59E25H2" 0 "Dihydroxy-acid dehydratase"
"Chl_N02_00102" 2886 "s59E5H2" 0 "Dihydroxy-acid dehydratase"
"Chl_N02_00102" 2886 "s63E6H2" 0 "Dihydroxy-acid dehydratase"
"Chl_N02_00103" 2887 "s59E13H2" 0 "Putative sugar phosphate isomerase YwlF"
"Chl_N02_00103" 2887 "s59E25H2" 0 "Putative sugar phosphate isomerase YwlF"
"Chl_N02_00103" 2887 "s59E5H2" 0 "Putative sugar phosphate isomerase YwlF"
"Chl_N02_00103" 2887 "s63E6H2" 0 "Putative sugar phosphate isomerase YwlF"
"Chl_N02_00104" 2888 "s59E13H2" 0 "Threonylcarbamoyl-AMP synthase"
"Chl_N02_00104" 2888 "s59E25H2" 0 "Threonylcarbamoyl-AMP synthase"
"Chl_N02_00104" 2888 "s59E5H2" 0 "Threonylcarbamoyl-AMP synthase"
"Chl_N02_00104" 2888 "s63E6H2" 0 "Threonylcarbamoyl-AMP synthase"
"Chl_N02_00105" 2889 "s59E13H2" 0 "GMP synthase [glutamine-hydrolyzing]"
"Chl_N02_00105" 2889 "s59E25H2" 0 "GMP synthase [glutamine-hydrolyzing]"
"Chl_N02_00105" 2889 "s59E5H2" 0 "GMP synthase [glutamine-hydrolyzing]"
"Chl_N02_00105" 2889 "s63E6H2" 0 "GMP synthase [glutamine-hydrolyzing]"
"Chl_N02_00106" 2890 "s59E13H2" 0 "hypothetical protein"
"Chl_N02_00106" 2890 "s59E25H2" 0 "hypothetical protein"
"Chl_N02_00106" 2890 "s59E5H2" 0 "hypothetical protein"
"Chl_N02_00106" 2890 "s63E6H2" 0 "hypothetical protein"
"Chl_N02_00108" 2892 "s59E13H2" 0 "PAC2 family protein"
"Chl_N02_00108" 2892 "s59E25H2" 0 "PAC2 family protein"
"Chl_N02_00108" 2892 "s59E5H2" 0.01390237 "PAC2 family protein"
"Chl_N02_00108" 2892 "s63E6H2" 0 "PAC2 family protein"
"Chl_N02_00109" 2893 "s59E13H2" 0 "HEAT repeat protein"
"Chl_N02_00109" 2893 "s59E25H2" 0 "HEAT repeat protein"
"Chl_N02_00109" 2893 "s59E5H2" 0.002257555 "HEAT repeat protein"
"Chl_N02_00109" 2893 "s63E6H2" 0 "HEAT repeat protein"
"Chl_N02_00110" 2894 "s59E13H2" 0 "Acetyltransferase (GNAT) family protein"
"Chl_N02_00110" 2894 "s59E25H2" 0 "Acetyltransferase (GNAT) family protein"
"Chl_N02_00110" 2894 "s59E5H2" 0.003162516 "Acetyltransferase (GNAT) family protein"
"Chl_N02_00110" 2894 "s63E6H2" 0 "Acetyltransferase (GNAT) family protein"
"Chl_N02_00111" 2895 "s59E13H2" 0 "Methylthioribose-1-phosphate isomerase"

"Chl_N02_00111" 2895 "s59E25H2" 0 "Methylthioribose-1-phosphate isomerase"
"Chl_N02_00111" 2895 "s59E5H2" 0.001197368 "Methylthioribose-1-phosphate isomerase"
"Chl_N02_00111" 2895 "s63E6H2" 0 "Methylthioribose-1-phosphate isomerase"
"Chl_N02_00113" 2897 "s59E13H2" 0 "S-methyl-5'-thioadenosine phosphorylase"
"Chl_N02_00113" 2897 "s59E25H2" 0 "S-methyl-5'-thioadenosine phosphorylase"
"Chl_N02_00113" 2897 "s59E5H2" 0 "S-methyl-5'-thioadenosine phosphorylase"
"Chl_N02_00113" 2897 "s63E6H2" 0 "S-methyl-5'-thioadenosine phosphorylase"
"Chl_N02_00114" 2898 "s59E13H2" 0 "methionine synthase"
"Chl_N02_00114" 2898 "s59E25H2" 0 "methionine synthase"
"Chl_N02_00114" 2898 "s59E5H2" 0 "methionine synthase"
"Chl_N02_00114" 2898 "s63E6H2" 0 "methionine synthase"
"Chl_N02_00116" 2900 "s59E13H2" 0 "Nucleoside-triphosphatase THEP1"
"Chl_N02_00116" 2900 "s59E25H2" 0 "Nucleoside-triphosphatase THEP1"
"Chl_N02_00116" 2900 "s59E5H2" 0 "Nucleoside-triphosphatase THEP1"
"Chl_N02_00116" 2900 "s63E6H2" 0 "Nucleoside-triphosphatase THEP1"
"Chl_N02_00117" 2901 "s59E13H2" 0 "Glycerol-3-phosphate acyltransferase"
"Chl_N02_00117" 2901 "s59E25H2" 0 "Glycerol-3-phosphate acyltransferase"
"Chl_N02_00117" 2901 "s59E5H2" 0 "Glycerol-3-phosphate acyltransferase"
"Chl_N02_00117" 2901 "s63E6H2" 0 "Glycerol-3-phosphate acyltransferase"
"Chl_N02_00118" 2902 "s59E13H2" 0.001742369 "hypothetical protein"
"Chl_N02_00118" 2902 "s59E25H2" 0 "hypothetical protein"
"Chl_N02_00118" 2902 "s59E5H2" 0 "hypothetical protein"
"Chl_N02_00118" 2902 "s63E6H2" 0 "hypothetical protein"
"Chl_N02_00119" 2903 "s59E13H2" 0 "hypothetical protein"
"Chl_N02_00119" 2903 "s59E25H2" 0 "hypothetical protein"
"Chl_N02_00119" 2903 "s59E5H2" 0 "hypothetical protein"
"Chl_N02_00119" 2903 "s63E6H2" 0 "hypothetical protein"
"Chl_N02_00120" 2904 "s59E13H2" 0 "Chromatin associated protein KTI12"
"Chl_N02_00120" 2904 "s59E25H2" 0 "Chromatin associated protein KTI12"
"Chl_N02_00120" 2904 "s59E5H2" 0 "Chromatin associated protein KTI12"
"Chl_N02_00120" 2904 "s63E6H2" 0 "Chromatin associated protein KTI12"
"Chl_N02_00121" 2905 "s59E13H2" 0 "DNA polymerase III PolC-type"
"Chl_N02_00121" 2905 "s59E25H2" 0 "DNA polymerase III PolC-type"
"Chl_N02_00121" 2905 "s59E5H2" 0 "DNA polymerase III PolC-type"
"Chl_N02_00121" 2905 "s63E6H2" 0 "DNA polymerase III PolC-type"
"Chl_N02_00122" 2906 "s59E13H2" 0 "Adenosylhomocysteinase"
"Chl_N02_00122" 2906 "s59E25H2" 0 "Adenosylhomocysteinase"
"Chl_N02_00122" 2906 "s59E5H2" 0.003374015 "Adenosylhomocysteinase"
"Chl_N02_00122" 2906 "s63E6H2" 0 "Adenosylhomocysteinase"
"Chl_N02_00123" 2907 "s59E13H2" 0 "S-adenosylmethionine synthase"
"Chl_N02_00123" 2907 "s59E25H2" 0 "S-adenosylmethionine synthase"
"Chl_N02_00123" 2907 "s59E5H2" 0.1077572 "S-adenosylmethionine synthase"
"Chl_N02_00123" 2907 "s63E6H2" 0 "S-adenosylmethionine synthase"
"Chl_N02_00125" 2909 "s59E13H2" 0 "ATP-binding region"
"Chl_N02_00125" 2909 "s59E25H2" 0 "ATP-binding region"
"Chl_N02_00125" 2909 "s59E5H2" 0.04481342 "ATP-binding region"
"Chl_N02_00125" 2909 "s63E6H2" 0 "ATP-binding region"

"Chl_N02_00126" 2910 "s59E13H2" 0 "Phosphoglucomutase"
"Chl_N02_00126" 2910 "s59E25H2" 0 "Phosphoglucomutase"
"Chl_N02_00126" 2910 "s59E5H2" 0.007250247 "Phosphoglucomutase"
"Chl_N02_00126" 2910 "s63E6H2" 0 "Phosphoglucomutase"
"Chl_N02_00127" 2911 "s59E13H2" 0 "Mannose-6-phosphate isomerase"
"Chl_N02_00127" 2911 "s59E25H2" 0 "Mannose-6-phosphate isomerase"
"Chl_N02_00127" 2911 "s59E5H2" 0.07083143 "Mannose-6-phosphate isomerase"
"Chl_N02_00127" 2911 "s63E6H2" 0 "Mannose-6-phosphate isomerase"
"Chl_N02_00128" 2912 "s59E13H2" 0 "30S ribosomal protein S9"
"Chl_N02_00128" 2912 "s59E25H2" 0 "30S ribosomal protein S9"
"Chl_N02_00128" 2912 "s59E5H2" 0.4738758 "30S ribosomal protein S9"
"Chl_N02_00128" 2912 "s63E6H2" 0 "30S ribosomal protein S9"
"Chl_N02_00129" 2913 "s59E13H2" 0 "50S ribosomal protein L13"
"Chl_N02_00129" 2913 "s59E25H2" 1.009592 "50S ribosomal protein L13"
"Chl_N02_00129" 2913 "s59E5H2" 0.7544093 "50S ribosomal protein L13"
"Chl_N02_00129" 2913 "s63E6H2" 0 "50S ribosomal protein L13"
"Chl_N02_00130" 2914 "s59E13H2" 0 "tRNA pseudouridine synthase A"
"Chl_N02_00130" 2914 "s59E25H2" 0.1024643 "tRNA pseudouridine synthase A"
"Chl_N02_00130" 2914 "s59E5H2" 0.3117447 "tRNA pseudouridine synthase A"
"Chl_N02_00130" 2914 "s63E6H2" 0.0009935026 "tRNA pseudouridine synthase A"
"Chl_N02_00131" 2915 "s59E13H2" 0 "50S ribosomal protein L17"
"Chl_N02_00131" 2915 "s59E25H2" 0 "50S ribosomal protein L17"
"Chl_N02_00131" 2915 "s59E5H2" 0.05710743 "50S ribosomal protein L17"
"Chl_N02_00131" 2915 "s63E6H2" 0.003091164 "50S ribosomal protein L17"
"Chl_N02_00132" 2916 "s59E13H2" 0 "DNA-directed RNA polymerase subunit alpha"
"Chl_N02_00132" 2916 "s59E25H2" 0 "DNA-directed RNA polymerase subunit alpha"
"Chl_N02_00132" 2916 "s59E5H2" 0.1029057 "DNA-directed RNA polymerase subunit alpha"
"Chl_N02_00132" 2916 "s63E6H2" 0 "DNA-directed RNA polymerase subunit alpha"
"Chl_N02_00133" 2917 "s59E13H2" 0 "30S ribosomal protein S4"
"Chl_N02_00133" 2917 "s59E25H2" 0 "30S ribosomal protein S4"
"Chl_N02_00133" 2917 "s59E5H2" 0.01080782 "30S ribosomal protein S4"
"Chl_N02_00133" 2917 "s63E6H2" 0 "30S ribosomal protein S4"
"Chl_N02_00134" 2918 "s59E13H2" 0 "30S ribosomal protein S11"
"Chl_N02_00134" 2918 "s59E25H2" 0 "30S ribosomal protein S11"
"Chl_N02_00134" 2918 "s59E5H2" 0 "30S ribosomal protein S11"
"Chl_N02_00134" 2918 "s63E6H2" 0 "30S ribosomal protein S11"
"Chl_N02_00135" 2919 "s59E13H2" 0 "30S ribosomal protein S13"
"Chl_N02_00135" 2919 "s59E25H2" 0 "30S ribosomal protein S13"
"Chl_N02_00135" 2919 "s59E5H2" 0 "30S ribosomal protein S13"
"Chl_N02_00135" 2919 "s63E6H2" 0 "30S ribosomal protein S13"
"Chl_N02_00136" 2920 "s59E13H2" 0 "50S ribosomal protein L36"
"Chl_N02_00136" 2920 "s59E25H2" 0 "50S ribosomal protein L36"
"Chl_N02_00136" 2920 "s59E5H2" 0 "50S ribosomal protein L36"
"Chl_N02_00136" 2920 "s63E6H2" 0 "50S ribosomal protein L36"
"Chl_N02_00137" 2921 "s59E13H2" 0 "Translation initiation factor IF-1"
"Chl_N02_00137" 2921 "s59E25H2" 0 "Translation initiation factor IF-1"
"Chl_N02_00137" 2921 "s59E5H2" 0 "Translation initiation factor IF-1"

"Chl_N02_00137" 2921 "s63E6H2" 0.009021777 "Translation initiation factor IF-1"
"Chl_N02_00138" 2922 "s59E13H2" 0 "Methionine aminopeptidase 1"
"Chl_N02_00138" 2922 "s59E25H2" 0 "Methionine aminopeptidase 1"
"Chl_N02_00138" 2922 "s59E5H2" 0 "Methionine aminopeptidase 1"
"Chl_N02_00138" 2922 "s63E6H2" 0.001248281 "Methionine aminopeptidase 1"
"Chl_N02_00139" 2923 "s59E13H2" 0 "Adenylate kinase"
"Chl_N02_00139" 2923 "s59E25H2" 0 "Adenylate kinase"
"Chl_N02_00139" 2923 "s59E5H2" 0 "Adenylate kinase"
"Chl_N02_00139" 2923 "s63E6H2" 0 "Adenylate kinase"
"Chl_N02_00140" 2924 "s59E13H2" 0 "preprotein translocase subunit SecY"
"Chl_N02_00140" 2924 "s59E25H2" 0 "preprotein translocase subunit SecY"
"Chl_N02_00140" 2924 "s59E5H2" 0 "preprotein translocase subunit SecY"
"Chl_N02_00140" 2924 "s63E6H2" 0.0002067786 "preprotein translocase subunit SecY"
"Chl_N02_00141" 2925 "s59E13H2" 0 "50S ribosomal protein L15"
"Chl_N02_00141" 2925 "s59E25H2" 0 "50S ribosomal protein L15"
"Chl_N02_00141" 2925 "s59E5H2" 0 "50S ribosomal protein L15"
"Chl_N02_00141" 2925 "s63E6H2" 0.00866789 "50S ribosomal protein L15"
"Chl_N02_00142" 2926 "s59E13H2" 0 "50S ribosomal protein L30"
"Chl_N02_00142" 2926 "s59E25H2" 0 "50S ribosomal protein L30"
"Chl_N02_00142" 2926 "s59E5H2" 0 "50S ribosomal protein L30"
"Chl_N02_00142" 2926 "s63E6H2" 0 "50S ribosomal protein L30"
"Chl_N02_00143" 2927 "s59E13H2" 0 "30S ribosomal protein S5"
"Chl_N02_00143" 2927 "s59E25H2" 0 "30S ribosomal protein S5"
"Chl_N02_00143" 2927 "s59E5H2" 0 "30S ribosomal protein S5"
"Chl_N02_00143" 2927 "s63E6H2" 0 "30S ribosomal protein S5"
"Chl_N02_00144" 2928 "s59E13H2" 0 "50S ribosomal protein L18"
"Chl_N02_00144" 2928 "s59E25H2" 0 "50S ribosomal protein L18"
"Chl_N02_00144" 2928 "s59E5H2" 0 "50S ribosomal protein L18"
"Chl_N02_00144" 2928 "s63E6H2" 0 "50S ribosomal protein L18"
"Chl_N02_00145" 2929 "s59E13H2" 0 "50S ribosomal protein L6"
"Chl_N02_00145" 2929 "s59E25H2" 0 "50S ribosomal protein L6"
"Chl_N02_00145" 2929 "s59E5H2" 0 "50S ribosomal protein L6"
"Chl_N02_00145" 2929 "s63E6H2" 0 "50S ribosomal protein L6"
"Chl_N02_00146" 2930 "s59E13H2" 0 "30S ribosomal protein S8"
"Chl_N02_00146" 2930 "s59E25H2" 0 "30S ribosomal protein S8"
"Chl_N02_00146" 2930 "s59E5H2" 0 "30S ribosomal protein S8"
"Chl_N02_00146" 2930 "s63E6H2" 0 "30S ribosomal protein S8"
"Chl_N02_00147" 2931 "s59E13H2" 0 "30S ribosomal protein S14"
"Chl_N02_00147" 2931 "s59E25H2" 0 "30S ribosomal protein S14"
"Chl_N02_00147" 2931 "s59E5H2" 0 "30S ribosomal protein S14"
"Chl_N02_00147" 2931 "s63E6H2" 0 "30S ribosomal protein S14"
"Chl_N02_00148" 2932 "s59E13H2" 0 "50S ribosomal protein L5"
"Chl_N02_00148" 2932 "s59E25H2" 0 "50S ribosomal protein L5"
"Chl_N02_00148" 2932 "s59E5H2" 0 "50S ribosomal protein L5"
"Chl_N02_00148" 2932 "s63E6H2" 0 "50S ribosomal protein L5"
"Chl_N02_00149" 2933 "s59E13H2" 0 "50S ribosomal protein L24"
"Chl_N02_00149" 2933 "s59E25H2" 0 "50S ribosomal protein L24"

"Chl_N02_00149" 2933 "s59E5H2" 0 "50S ribosomal protein L24"
"Chl_N02_00149" 2933 "s63E6H2" 0 "50S ribosomal protein L24"
"Chl_N02_00150" 2934 "s59E13H2" 0 "50S ribosomal protein L14"
"Chl_N02_00150" 2934 "s59E25H2" 0 "50S ribosomal protein L14"
"Chl_N02_00150" 2934 "s59E5H2" 0 "50S ribosomal protein L14"
"Chl_N02_00150" 2934 "s63E6H2" 0 "50S ribosomal protein L14"
"Chl_N02_00151" 2935 "s59E13H2" 0 "30S ribosomal protein S17"
"Chl_N02_00151" 2935 "s59E25H2" 0 "30S ribosomal protein S17"
"Chl_N02_00151" 2935 "s59E5H2" 0 "30S ribosomal protein S17"
"Chl_N02_00151" 2935 "s63E6H2" 0 "30S ribosomal protein S17"
"Chl_N02_00152" 2936 "s59E13H2" 0 "50S ribosomal protein L29"
"Chl_N02_00152" 2936 "s59E25H2" 0 "50S ribosomal protein L29"
"Chl_N02_00152" 2936 "s59E5H2" 0 "50S ribosomal protein L29"
"Chl_N02_00152" 2936 "s63E6H2" 0 "50S ribosomal protein L29"
"Chl_N02_00153" 2937 "s59E13H2" 0 "50S ribosomal protein L16"
"Chl_N02_00153" 2937 "s59E25H2" 0 "50S ribosomal protein L16"
"Chl_N02_00153" 2937 "s59E5H2" 0 "50S ribosomal protein L16"
"Chl_N02_00153" 2937 "s63E6H2" 0 "50S ribosomal protein L16"
"Chl_N02_00154" 2938 "s59E13H2" 0 "30S ribosomal protein S3"
"Chl_N02_00154" 2938 "s59E25H2" 0 "30S ribosomal protein S3"
"Chl_N02_00154" 2938 "s59E5H2" 0 "30S ribosomal protein S3"
"Chl_N02_00154" 2938 "s63E6H2" 0 "30S ribosomal protein S3"
"Chl_N02_00155" 2939 "s59E13H2" 0 "50S ribosomal protein L22"
"Chl_N02_00155" 2939 "s59E25H2" 0 "50S ribosomal protein L22"
"Chl_N02_00155" 2939 "s59E5H2" 0 "50S ribosomal protein L22"
"Chl_N02_00155" 2939 "s63E6H2" 0 "50S ribosomal protein L22"
"Chl_N02_00156" 2940 "s59E13H2" 0 "30S ribosomal protein S19"
"Chl_N02_00156" 2940 "s59E25H2" 0 "30S ribosomal protein S19"
"Chl_N02_00156" 2940 "s59E5H2" 0 "30S ribosomal protein S19"
"Chl_N02_00156" 2940 "s63E6H2" 0 "30S ribosomal protein S19"
"Chl_N02_00157" 2941 "s59E13H2" 0 "50S ribosomal protein L2"
"Chl_N02_00157" 2941 "s59E25H2" 0 "50S ribosomal protein L2"
"Chl_N02_00157" 2941 "s59E5H2" 0 "50S ribosomal protein L2"
"Chl_N02_00157" 2941 "s63E6H2" 0 "50S ribosomal protein L2"
"Chl_N02_00158" 2942 "s59E13H2" 0 "50S ribosomal protein L23"
"Chl_N02_00158" 2942 "s59E25H2" 0 "50S ribosomal protein L23"
"Chl_N02_00158" 2942 "s59E5H2" 0 "50S ribosomal protein L23"
"Chl_N02_00158" 2942 "s63E6H2" 0 "50S ribosomal protein L23"
"Chl_N02_00159" 2943 "s59E13H2" 0 "50S ribosomal protein L4"
"Chl_N02_00159" 2943 "s59E25H2" 0 "50S ribosomal protein L4"
"Chl_N02_00159" 2943 "s59E5H2" 0 "50S ribosomal protein L4"
"Chl_N02_00159" 2943 "s63E6H2" 0 "50S ribosomal protein L4"
"Chl_N02_00160" 2944 "s59E13H2" 0 "50S ribosomal protein L3"
"Chl_N02_00160" 2944 "s59E25H2" 0 "50S ribosomal protein L3"
"Chl_N02_00160" 2944 "s59E5H2" 0 "50S ribosomal protein L3"
"Chl_N02_00160" 2944 "s63E6H2" 0 "50S ribosomal protein L3"
"Chl_N02_00161" 2945 "s59E13H2" 0 "30S ribosomal protein S10"

"Chl_N02_00161" 2945 "s59E25H2" 0 "30S ribosomal protein S10"
"Chl_N02_00161" 2945 "s59E5H2" 0 "30S ribosomal protein S10"
"Chl_N02_00161" 2945 "s63E6H2" 0 "30S ribosomal protein S10"
"Chl_N02_00162" 2946 "s59E13H2" 0 "Elongation factor G"
"Chl_N02_00162" 2946 "s59E25H2" 0 "Elongation factor G"
"Chl_N02_00162" 2946 "s59E5H2" 0 "Elongation factor G"
"Chl_N02_00162" 2946 "s63E6H2" 0 "Elongation factor G"
"Chl_N02_00163" 2947 "s59E13H2" 0 "30S ribosomal protein S7"
"Chl_N02_00163" 2947 "s59E25H2" 0 "30S ribosomal protein S7"
"Chl_N02_00163" 2947 "s59E5H2" 0 "30S ribosomal protein S7"
"Chl_N02_00163" 2947 "s63E6H2" 0 "30S ribosomal protein S7"
"Chl_N02_00164" 2948 "s59E13H2" 0.0008659573 "30S ribosomal protein S12"
"Chl_N02_00164" 2948 "s59E25H2" 0 "30S ribosomal protein S12"
"Chl_N02_00164" 2948 "s59E5H2" 0 "30S ribosomal protein S12"
"Chl_N02_00164" 2948 "s63E6H2" 0 "30S ribosomal protein S12"
"Chl_N02_00165" 2949 "s59E13H2" 0 "Deoxyuridine 5'-triphosphate nucleotidohydrolase"
"Chl_N02_00165" 2949 "s59E25H2" 0 "Deoxyuridine 5'-triphosphate nucleotidohydrolase"
"Chl_N02_00165" 2949 "s59E5H2" 0 "Deoxyuridine 5'-triphosphate nucleotidohydrolase"
"Chl_N02_00165" 2949 "s63E6H2" 0 "Deoxyuridine 5'-triphosphate nucleotidohydrolase"
"Chl_N02_00166" 2950 "s59E13H2" 0 "Type II secretion system protein G precursor"
"Chl_N02_00166" 2950 "s59E25H2" 0 "Type II secretion system protein G precursor"
"Chl_N02_00166" 2950 "s59E5H2" 0 "Type II secretion system protein G precursor"
"Chl_N02_00166" 2950 "s63E6H2" 0 "Type II secretion system protein G precursor"
"Chl_N02_00167" 2951 "s59E13H2" 0 "hypothetical protein"
"Chl_N02_00167" 2951 "s59E25H2" 0 "hypothetical protein"
"Chl_N02_00167" 2951 "s59E5H2" 0 "hypothetical protein"
"Chl_N02_00167" 2951 "s63E6H2" 0 "hypothetical protein"
"Chl_N02_00168" 2952 "s59E13H2" 0 "Tungsten-containing aldehyde ferredoxin oxidoreductase"
"Chl_N02_00168" 2952 "s59E25H2" 0 "Tungsten-containing aldehyde ferredoxin oxidoreductase"
"Chl_N02_00168" 2952 "s59E5H2" 0 "Tungsten-containing aldehyde ferredoxin oxidoreductase"
"Chl_N02_00168" 2952 "s63E6H2" 0 "Tungsten-containing aldehyde ferredoxin oxidoreductase"
"Chl_N02_00169" 2953 "s59E13H2" 0 "NADH-dependent phenylglyoxylate dehydrogenase subunit beta"
"Chl_N02_00169" 2953 "s59E25H2" 0 "NADH-dependent phenylglyoxylate dehydrogenase subunit beta"
"Chl_N02_00169" 2953 "s59E5H2" 0 "NADH-dependent phenylglyoxylate dehydrogenase subunit beta"
"Chl_N02_00169" 2953 "s63E6H2" 0 "NADH-dependent phenylglyoxylate dehydrogenase subunit beta"
"Chl_N02_00170" 2954 "s59E13H2" 0 "(R)-citramalate synthase CimA"
"Chl_N02_00170" 2954 "s59E25H2" 0 "(R)-citramalate synthase CimA"
"Chl_N02_00170" 2954 "s59E5H2" 0 "(R)-citramalate synthase CimA"
"Chl_N02_00170" 2954 "s63E6H2" 0 "(R)-citramalate synthase CimA"
"Chl_N02_00171" 2955 "s59E13H2" 0 "putative phosphoenolpyruvate synthase"
"Chl_N02_00171" 2955 "s59E25H2" 0 "putative phosphoenolpyruvate synthase"
"Chl_N02_00171" 2955 "s59E5H2" 0.003767727 "putative phosphoenolpyruvate synthase"
"Chl_N02_00171" 2955 "s63E6H2" 0 "putative phosphoenolpyruvate synthase"
"Chl_N02_00173" 2957 "s59E13H2" 0 "hypothetical protein"
"Chl_N02_00173" 2957 "s59E25H2" 0 "hypothetical protein"
"Chl_N02_00173" 2957 "s59E5H2" 0 "hypothetical protein"
"Chl_N02_00173" 2957 "s63E6H2" 0 "hypothetical protein"

"Chl_N02_00174" 2958 "s59E13H2" 0 "High-affinity branched-chain amino acid transport system permease protein LivH"
"Chl_N02_00174" 2958 "s59E25H2" 0 "High-affinity branched-chain amino acid transport system permease protein LivH"
"Chl_N02_00174" 2958 "s59E5H2" 0 "High-affinity branched-chain amino acid transport system permease protein LivH"
"Chl_N02_00174" 2958 "s63E6H2" 0 "High-affinity branched-chain amino acid transport system permease protein LivH"
"Chl_N02_00175" 2959 "s59E13H2" 0 "leucine/isoleucine/valine transporter permease subunit"
"Chl_N02_00175" 2959 "s59E25H2" 0 "leucine/isoleucine/valine transporter permease subunit"
"Chl_N02_00175" 2959 "s59E5H2" 0 "leucine/isoleucine/valine transporter permease subunit"
"Chl_N02_00175" 2959 "s63E6H2" 0 "leucine/isoleucine/valine transporter permease subunit"
"Chl_N02_00176" 2960 "s59E13H2" 0.004329132 "Lipopolysaccharide export system ATP-binding protein LptB"
"Chl_N02_00176" 2960 "s59E25H2" 0 "Lipopolysaccharide export system ATP-binding protein LptB"
"Chl_N02_00176" 2960 "s59E5H2" 0 "Lipopolysaccharide export system ATP-binding protein LptB"
"Chl_N02_00176" 2960 "s63E6H2" 0 "Lipopolysaccharide export system ATP-binding protein LptB"
"Chl_N02_00177" 2961 "s59E13H2" 0 "High-affinity branched-chain amino acid transport ATP-binding protein LivF"
"Chl_N02_00177" 2961 "s59E25H2" 0 "High-affinity branched-chain amino acid transport ATP-binding protein LivF"
"Chl_N02_00177" 2961 "s59E5H2" 0 "High-affinity branched-chain amino acid transport ATP-binding protein LivF"
"Chl_N02_00177" 2961 "s63E6H2" 0 "High-affinity branched-chain amino acid transport ATP-binding protein LivF"
"Chl_N02_00179" 2963 "s59E13H2" 0 "Chondramide synthase cmdD"
"Chl_N02_00179" 2963 "s59E25H2" 0 "Chondramide synthase cmdD"
"Chl_N02_00179" 2963 "s59E5H2" 0 "Chondramide synthase cmdD"
"Chl_N02_00179" 2963 "s63E6H2" 0 "Chondramide synthase cmdD"
"Chl_N02_00181" 2965 "s59E13H2" 0 "HTH-type transcriptional activator RhaS"
"Chl_N02_00181" 2965 "s59E25H2" 0 "HTH-type transcriptional activator RhaS"
"Chl_N02_00181" 2965 "s59E5H2" 0 "HTH-type transcriptional activator RhaS"
"Chl_N02_00181" 2965 "s63E6H2" 0 "HTH-type transcriptional activator RhaS"
"Chl_N02_00183" 2967 "s59E13H2" 0 "hypothetical protein"
"Chl_N02_00183" 2967 "s59E25H2" 0 "hypothetical protein"
"Chl_N02_00183" 2967 "s59E5H2" 0 "hypothetical protein"
"Chl_N02_00183" 2967 "s63E6H2" 0 "hypothetical protein"
"Chl_N02_00184" 2968 "s59E13H2" 0 "Tungsten-containing aldehyde ferredoxin oxidoreductase"
"Chl_N02_00184" 2968 "s59E25H2" 0 "Tungsten-containing aldehyde ferredoxin oxidoreductase"
"Chl_N02_00184" 2968 "s59E5H2" 0 "Tungsten-containing aldehyde ferredoxin oxidoreductase"
"Chl_N02_00184" 2968 "s63E6H2" 0 "Tungsten-containing aldehyde ferredoxin oxidoreductase"
"Chl_N02_00186" 2970 "s59E13H2" 0 "Kynurenine formamidase"
"Chl_N02_00186" 2970 "s59E25H2" 0 "Kynurenine formamidase"
"Chl_N02_00186" 2970 "s59E5H2" 0 "Kynurenine formamidase"
"Chl_N02_00186" 2970 "s63E6H2" 0 "Kynurenine formamidase"
"Chl_N02_00187" 2971 "s59E13H2" 0 "hypothetical protein"
"Chl_N02_00187" 2971 "s59E25H2" 0 "hypothetical protein"
"Chl_N02_00187" 2971 "s59E5H2" 0 "hypothetical protein"
"Chl_N02_00187" 2971 "s63E6H2" 0 "hypothetical protein"
"Chl_N02_00188" 2972 "s59E13H2" 0 "putative adenylyltransferase"
"Chl_N02_00188" 2972 "s59E25H2" 0 "putative adenylyltransferase"
"Chl_N02_00188" 2972 "s59E5H2" 0 "putative adenylyltransferase"
"Chl_N02_00188" 2972 "s63E6H2" 0 "putative adenylyltransferase"
"Chl_N02_00189" 2973 "s59E13H2" 0 "Sulfur carrier protein CysO"
"Chl_N02_00189" 2973 "s59E25H2" 0 "Sulfur carrier protein CysO"
"Chl_N02_00189" 2973 "s59E5H2" 0 "Sulfur carrier protein CysO"

"Chl_N02_00189" 2973 "s63E6H2" 0 "Sulfur carrier protein CysO"
"Chl_N02_00191" 2975 "s59E13H2" 0.02379871 "chromosome segregation protein"
"Chl_N02_00191" 2975 "s59E25H2" 0 "chromosome segregation protein"
"Chl_N02_00191" 2975 "s59E5H2" 0 "chromosome segregation protein"
"Chl_N02_00191" 2975 "s63E6H2" 0 "chromosome segregation protein"
"Chl_N02_00192" 2976 "s59E13H2" 0.0003389665 "hypothetical protein"
"Chl_N02_00192" 2976 "s59E25H2" 0 "hypothetical protein"
"Chl_N02_00192" 2976 "s59E5H2" 0 "hypothetical protein"
"Chl_N02_00192" 2976 "s63E6H2" 0 "hypothetical protein"
"Chl_N02_00193" 2977 "s59E13H2" 0 "Tyrosine recombinase XerD"
"Chl_N02_00193" 2977 "s59E25H2" 0 "Tyrosine recombinase XerD"
"Chl_N02_00193" 2977 "s59E5H2" 0 "Tyrosine recombinase XerD"
"Chl_N02_00193" 2977 "s63E6H2" 0 "Tyrosine recombinase XerD"
"Chl_N02_00194" 2978 "s59E13H2" 0.03444915 "hypothetical protein"
"Chl_N02_00194" 2978 "s59E25H2" 0 "hypothetical protein"
"Chl_N02_00194" 2978 "s59E5H2" 0 "hypothetical protein"
"Chl_N02_00194" 2978 "s63E6H2" 0 "hypothetical protein"
"Chl_N02_00195" 2979 "s59E13H2" 0 "hypothetical protein"
"Chl_N02_00195" 2979 "s59E25H2" 0 "hypothetical protein"
"Chl_N02_00195" 2979 "s59E5H2" 0 "hypothetical protein"
"Chl_N02_00195" 2979 "s63E6H2" 0 "hypothetical protein"
"Chl_N02_00196" 2980 "s59E13H2" 0 "hypothetical protein"
"Chl_N02_00196" 2980 "s59E25H2" 0 "hypothetical protein"
"Chl_N02_00196" 2980 "s59E5H2" 0 "hypothetical protein"
"Chl_N02_00196" 2980 "s63E6H2" 0 "hypothetical protein"
"Chl_N02_00197" 2981 "s59E13H2" 0 "Ribonuclease PH"
"Chl_N02_00197" 2981 "s59E25H2" 0 "Ribonuclease PH"
"Chl_N02_00197" 2981 "s59E5H2" 0 "Ribonuclease PH"
"Chl_N02_00197" 2981 "s63E6H2" 0 "Ribonuclease PH"
"Chl_N02_00198" 2982 "s59E13H2" 0 "hypothetical protein"
"Chl_N02_00198" 2982 "s59E25H2" 0 "hypothetical protein"
"Chl_N02_00198" 2982 "s59E5H2" 0 "hypothetical protein"
"Chl_N02_00198" 2982 "s63E6H2" 0 "hypothetical protein"
"Chl_N02_00199" 2983 "s59E13H2" 0 "Galactose/methyl galactoside import ATP-binding protein MglA"
"Chl_N02_00199" 2983 "s59E25H2" 0 "Galactose/methyl galactoside import ATP-binding protein MglA"
"Chl_N02_00199" 2983 "s59E5H2" 0.04022328 "Galactose/methyl galactoside import ATP-binding protein MglA"
"Chl_N02_00199" 2983 "s63E6H2" 0.009325512 "Galactose/methyl galactoside import ATP-binding protein MglA"
"Chl_N02_00200" 2984 "s59E13H2" 0 "Ribose transport system permease protein RbsC"
"Chl_N02_00200" 2984 "s59E25H2" 0 "Ribose transport system permease protein RbsC"
"Chl_N02_00200" 2984 "s59E5H2" 0 "Ribose transport system permease protein RbsC"
"Chl_N02_00200" 2984 "s63E6H2" 0.003289821 "Ribose transport system permease protein RbsC"
"Chl_N02_00201" 2985 "s59E13H2" 0 "Inner membrane ABC transporter permease protein YjfF"
"Chl_N02_00201" 2985 "s59E25H2" 0 "Inner membrane ABC transporter permease protein YjfF"
"Chl_N02_00201" 2985 "s59E5H2" 0 "Inner membrane ABC transporter permease protein YjfF"
"Chl_N02_00201" 2985 "s63E6H2" 0.004543927 "Inner membrane ABC transporter permease protein YjfF"
"Chl_N02_00203" 2987 "s59E13H2" 0 "hypothetical protein"
"Chl_N02_00203" 2987 "s59E25H2" 0 "hypothetical protein"

"Chl_N02_00203" 2987 "s59E5H2" 0 "hypothetical protein"
"Chl_N02_00203" 2987 "s63E6H2" 0 "hypothetical protein"
"Chl_N02_00204" 2988 "s59E13H2" 0 "hypothetical protein"
"Chl_N02_00204" 2988 "s59E25H2" 0 "hypothetical protein"
"Chl_N02_00204" 2988 "s59E5H2" 0 "hypothetical protein"
"Chl_N02_00204" 2988 "s63E6H2" 0 "hypothetical protein"
"Chl_N02_00205" 2989 "s59E13H2" 0 "hypothetical protein"
"Chl_N02_00205" 2989 "s59E25H2" 0 "hypothetical protein"
"Chl_N02_00205" 2989 "s59E5H2" 0 "hypothetical protein"
"Chl_N02_00205" 2989 "s63E6H2" 0 "hypothetical protein"
"Chl_N02_00206" 2990 "s59E13H2" 0 "putative ABC transporter ATP-binding protein YxlF"
"Chl_N02_00206" 2990 "s59E25H2" 0 "putative ABC transporter ATP-binding protein YxlF"
"Chl_N02_00206" 2990 "s59E5H2" 0 "putative ABC transporter ATP-binding protein YxlF"
"Chl_N02_00206" 2990 "s63E6H2" 0 "putative ABC transporter ATP-binding protein YxlF"
"Chl_N02_00207" 2991 "s59E13H2" 0 "ABC-2 type transporter"
"Chl_N02_00207" 2991 "s59E25H2" 0 "ABC-2 type transporter"
"Chl_N02_00207" 2991 "s59E5H2" 0 "ABC-2 type transporter"
"Chl_N02_00207" 2991 "s63E6H2" 0 "ABC-2 type transporter"
"Chl_N02_00208" 2992 "s59E13H2" 0 "Non-motile and phage-resistance protein"
"Chl_N02_00208" 2992 "s59E25H2" 0 "Non-motile and phage-resistance protein"
"Chl_N02_00208" 2992 "s59E5H2" 0 "Non-motile and phage-resistance protein"
"Chl_N02_00208" 2992 "s63E6H2" 0 "Non-motile and phage-resistance protein"
"Chl_N02_00209" 2993 "s59E13H2" 0 "Polar-differentiation response regulator DivK"
"Chl_N02_00209" 2993 "s59E25H2" 0 "Polar-differentiation response regulator DivK"
"Chl_N02_00209" 2993 "s59E5H2" 0 "Polar-differentiation response regulator DivK"
"Chl_N02_00209" 2993 "s63E6H2" 0 "Polar-differentiation response regulator DivK"
"Chl_N02_00210" 2994 "s59E13H2" 0 "hypothetical protein"
"Chl_N02_00210" 2994 "s59E25H2" 0 "hypothetical protein"
"Chl_N02_00210" 2994 "s59E5H2" 0.002488774 "hypothetical protein"
"Chl_N02_00210" 2994 "s63E6H2" 0 "hypothetical protein"
"Chl_N02_00211" 2995 "s59E13H2" 0 "Threonine--tRNA ligase 1"
"Chl_N02_00211" 2995 "s59E25H2" 0 "Threonine--tRNA ligase 1"
"Chl_N02_00211" 2995 "s59E5H2" 0 "Threonine--tRNA ligase 1"
"Chl_N02_00211" 2995 "s63E6H2" 0 "Threonine--tRNA ligase 1"
"Chl_N02_00213" 2997 "s59E13H2" 0 "Translation initiation factor IF-3"
"Chl_N02_00213" 2997 "s59E25H2" 0 "Translation initiation factor IF-3"
"Chl_N02_00213" 2997 "s59E5H2" 0 "Translation initiation factor IF-3"
"Chl_N02_00213" 2997 "s63E6H2" 0 "Translation initiation factor IF-3"
"Chl_N02_00214" 2998 "s59E13H2" 0 "50S ribosomal protein L35"
"Chl_N02_00214" 2998 "s59E25H2" 0 "50S ribosomal protein L35"
"Chl_N02_00214" 2998 "s59E5H2" 0 "50S ribosomal protein L35"
"Chl_N02_00214" 2998 "s63E6H2" 0 "50S ribosomal protein L35"
"Chl_N02_00215" 2999 "s59E13H2" 0 "50S ribosomal protein L20"
"Chl_N02_00215" 2999 "s59E25H2" 0 "50S ribosomal protein L20"
"Chl_N02_00215" 2999 "s59E5H2" 0 "50S ribosomal protein L20"
"Chl_N02_00215" 2999 "s63E6H2" 0 "50S ribosomal protein L20"
"Chl_N02_00216" 3000 "s59E13H2" 0 "hypothetical protein"

"Chl_N02_00216" 3000 "s59E25H2" 0 "hypothetical protein"
"Chl_N02_00216" 3000 "s59E5H2" 0 "hypothetical protein"
"Chl_N02_00216" 3000 "s63E6H2" 0 "hypothetical protein"
"Chl_N02_00217" 3001 "s59E13H2" 0 "Phenylalanine--tRNA ligase alpha subunit"
"Chl_N02_00217" 3001 "s59E25H2" 0 "Phenylalanine--tRNA ligase alpha subunit"
"Chl_N02_00217" 3001 "s59E5H2" 0 "Phenylalanine--tRNA ligase alpha subunit"
"Chl_N02_00217" 3001 "s63E6H2" 0 "Phenylalanine--tRNA ligase alpha subunit"
"Chl_N02_00218" 3002 "s59E13H2" 0 "Phenylalanine--tRNA ligase beta subunit"
"Chl_N02_00218" 3002 "s59E25H2" 0 "Phenylalanine--tRNA ligase beta subunit"
"Chl_N02_00218" 3002 "s59E5H2" 0 "Phenylalanine--tRNA ligase beta subunit"
"Chl_N02_00218" 3002 "s63E6H2" 0 "Phenylalanine--tRNA ligase beta subunit"
"Chl_N02_00219" 3003 "s59E13H2" 0 "Ubiquinone/menaquinone biosynthesis C-methyltransferase UbiE"
"Chl_N02_00219" 3003 "s59E25H2" 0 "Ubiquinone/menaquinone biosynthesis C-methyltransferase UbiE"
"Chl_N02_00219" 3003 "s59E5H2" 0 "Ubiquinone/menaquinone biosynthesis C-methyltransferase UbiE"
"Chl_N02_00219" 3003 "s63E6H2" 0.004682004 "Ubiquinone/menaquinone biosynthesis C-methyltransferase UbiE"
"Chl_N02_00220" 3004 "s59E13H2" 0 "Proline--tRNA ligase"
"Chl_N02_00220" 3004 "s59E25H2" 0 "Proline--tRNA ligase"
"Chl_N02_00220" 3004 "s59E5H2" 0 "Proline--tRNA ligase"
"Chl_N02_00220" 3004 "s63E6H2" 0 "Proline--tRNA ligase"
"Chl_N02_00221" 3005 "s59E13H2" 0 "hypothetical protein"
"Chl_N02_00221" 3005 "s59E25H2" 0 "hypothetical protein"
"Chl_N02_00221" 3005 "s59E5H2" 0 "hypothetical protein"
"Chl_N02_00221" 3005 "s63E6H2" 0 "hypothetical protein"
"Chl_N02_00222" 3006 "s59E13H2" 0 "hypothetical protein"
"Chl_N02_00222" 3006 "s59E25H2" 0 "hypothetical protein"
"Chl_N02_00222" 3006 "s59E5H2" 0 "hypothetical protein"
"Chl_N02_00222" 3006 "s63E6H2" 0 "hypothetical protein"
"Chl_N02_00223" 3007 "s59E13H2" 0 "hypothetical protein"
"Chl_N02_00223" 3007 "s59E25H2" 0 "hypothetical protein"
"Chl_N02_00223" 3007 "s59E5H2" 0 "hypothetical protein"
"Chl_N02_00223" 3007 "s63E6H2" 0 "hypothetical protein"
"Chl_N02_00224" 3008 "s59E13H2" 0 "hypothetical protein"
"Chl_N02_00224" 3008 "s59E25H2" 0 "hypothetical protein"
"Chl_N02_00224" 3008 "s59E5H2" 0.0122746 "hypothetical protein"
"Chl_N02_00224" 3008 "s63E6H2" 0 "hypothetical protein"
"Chl_N02_00226" 3010 "s59E13H2" 0 "Phosphoglycerate kinase"
"Chl_N02_00226" 3010 "s59E25H2" 0 "Phosphoglycerate kinase"
"Chl_N02_00226" 3010 "s59E5H2" 0 "Phosphoglycerate kinase"
"Chl_N02_00226" 3010 "s63E6H2" 0 "Phosphoglycerate kinase"
"Chl_N02_00227" 3011 "s59E13H2" 0 "2,3-bisphosphoglycerate-independent phosphoglycerate mutase 1"
"Chl_N02_00227" 3011 "s59E25H2" 0 "2,3-bisphosphoglycerate-independent phosphoglycerate mutase 1"
"Chl_N02_00227" 3011 "s59E5H2" 0 "2,3-bisphosphoglycerate-independent phosphoglycerate mutase 1"
"Chl_N02_00227" 3011 "s63E6H2" 0 "2,3-bisphosphoglycerate-independent phosphoglycerate mutase 1"
"Chl_N02_00228" 3012 "s59E13H2" 0 "Doxorubicin resistance ATP-binding protein DrrA"
"Chl_N02_00228" 3012 "s59E25H2" 0 "Doxorubicin resistance ATP-binding protein DrrA"
"Chl_N02_00228" 3012 "s59E5H2" 0 "Doxorubicin resistance ATP-binding protein DrrA"
"Chl_N02_00228" 3012 "s63E6H2" 0 "Doxorubicin resistance ATP-binding protein DrrA"

"Chl_N02_00229" 3013 "s59E13H2" 0 "Daunorubicin/doxorubicin resistance ATP-binding protein DrrA"
"Chl_N02_00229" 3013 "s59E25H2" 0 "Daunorubicin/doxorubicin resistance ATP-binding protein DrrA"
"Chl_N02_00229" 3013 "s59E5H2" 0 "Daunorubicin/doxorubicin resistance ATP-binding protein DrrA"
"Chl_N02_00229" 3013 "s63E6H2" 0 "Daunorubicin/doxorubicin resistance ATP-binding protein DrrA"
"Chl_N02_00230" 3014 "s59E13H2" 0 "Inner membrane transport permease YbhR"
"Chl_N02_00230" 3014 "s59E25H2" 0 "Inner membrane transport permease YbhR"
"Chl_N02_00230" 3014 "s59E5H2" 0 "Inner membrane transport permease YbhR"
"Chl_N02_00230" 3014 "s63E6H2" 0 "Inner membrane transport permease YbhR"
"Chl_N02_00231" 3015 "s59E13H2" 0 "Triosephosphate isomerase"
"Chl_N02_00231" 3015 "s59E25H2" 0 "Triosephosphate isomerase"
"Chl_N02_00231" 3015 "s59E5H2" 0 "Triosephosphate isomerase"
"Chl_N02_00231" 3015 "s63E6H2" 0 "Triosephosphate isomerase"
"Chl_N02_00232" 3016 "s59E13H2" 0 "tRNA dimethylallyltransferase"
"Chl_N02_00232" 3016 "s59E25H2" 0 "tRNA dimethylallyltransferase"
"Chl_N02_00232" 3016 "s59E5H2" 0 "tRNA dimethylallyltransferase"
"Chl_N02_00232" 3016 "s63E6H2" 0 "tRNA dimethylallyltransferase"
"Chl_N02_00233" 3017 "s59E13H2" 0.00174412 "Demethylmenaquinone methyltransferase"
"Chl_N02_00233" 3017 "s59E25H2" 0 "Demethylmenaquinone methyltransferase"
"Chl_N02_00233" 3017 "s59E5H2" 0 "Demethylmenaquinone methyltransferase"
"Chl_N02_00233" 3017 "s63E6H2" 0 "Demethylmenaquinone methyltransferase"
"Chl_N02_00234" 3018 "s59E13H2" 0 "Diaminopimelate epimerase"
"Chl_N02_00234" 3018 "s59E25H2" 0 "Diaminopimelate epimerase"
"Chl_N02_00234" 3018 "s59E5H2" 0 "Diaminopimelate epimerase"
"Chl_N02_00234" 3018 "s63E6H2" 0 "Diaminopimelate epimerase"
"Chl_N02_00235" 3019 "s59E13H2" 0 "LL-diaminopimelate aminotransferase"
"Chl_N02_00235" 3019 "s59E25H2" 0 "LL-diaminopimelate aminotransferase"
"Chl_N02_00235" 3019 "s59E5H2" 0 "LL-diaminopimelate aminotransferase"
"Chl_N02_00235" 3019 "s63E6H2" 0 "LL-diaminopimelate aminotransferase"
"Chl_N02_00236" 3020 "s59E13H2" 0 "GTPase HflX"
"Chl_N02_00236" 3020 "s59E25H2" 0.04014939 "GTPase HflX"
"Chl_N02_00236" 3020 "s59E5H2" 0.01250317 "GTPase HflX"
"Chl_N02_00236" 3020 "s63E6H2" 0.0005846562 "GTPase HflX"
"Chl_N02_00237" 3021 "s59E13H2" 0 "Carboxymuconolactone decarboxylase family protein"
"Chl_N02_00237" 3021 "s59E25H2" 0 "Carboxymuconolactone decarboxylase family protein"
"Chl_N02_00237" 3021 "s59E5H2" 0 "Carboxymuconolactone decarboxylase family protein"
"Chl_N02_00237" 3021 "s63E6H2" 0 "Carboxymuconolactone decarboxylase family protein"
"Chl_N02_00239" 3023 "s59E13H2" 0 "ADP-ribosyl-[dinitrogen reductase] glycohydrolase"
"Chl_N02_00239" 3023 "s59E25H2" 0 "ADP-ribosyl-[dinitrogen reductase] glycohydrolase"
"Chl_N02_00239" 3023 "s59E5H2" 0 "ADP-ribosyl-[dinitrogen reductase] glycohydrolase"
"Chl_N02_00239" 3023 "s63E6H2" 0.0006190059 "ADP-ribosyl-[dinitrogen reductase] glycohydrolase"
"Chl_N02_00240" 3024 "s59E13H2" 0 "Radical SAM superfamily protein"
"Chl_N02_00240" 3024 "s59E25H2" 0 "Radical SAM superfamily protein"
"Chl_N02_00240" 3024 "s59E5H2" 0 "Radical SAM superfamily protein"
"Chl_N02_00240" 3024 "s63E6H2" 0 "Radical SAM superfamily protein"
"Chl_N02_00241" 3025 "s59E13H2" 0 "pyruvate formate lyase-activating enzyme 1"
"Chl_N02_00241" 3025 "s59E25H2" 0 "pyruvate formate lyase-activating enzyme 1"
"Chl_N02_00241" 3025 "s59E5H2" 0 "pyruvate formate lyase-activating enzyme 1"

"Chl_N02_00241" 3025 "s63E6H2" 0 "pyruvate formate lyase-activating enzyme 1"
"Chl_N02_00243" 3027 "s59E13H2" 0 "30S ribosomal protein S2"
"Chl_N02_00243" 3027 "s59E25H2" 0 "30S ribosomal protein S2"
"Chl_N02_00243" 3027 "s59E5H2" 0 "30S ribosomal protein S2"
"Chl_N02_00243" 3027 "s63E6H2" 0 "30S ribosomal protein S2"
"Chl_N02_00244" 3028 "s59E13H2" 0 "Elongation factor Ts"
"Chl_N02_00244" 3028 "s59E25H2" 0 "Elongation factor Ts"
"Chl_N02_00244" 3028 "s59E5H2" 0 "Elongation factor Ts"
"Chl_N02_00244" 3028 "s63E6H2" 0 "Elongation factor Ts"
"Chl_N02_00245" 3029 "s59E13H2" 0 "Uridylate kinase"
"Chl_N02_00245" 3029 "s59E25H2" 0 "Uridylate kinase"
"Chl_N02_00245" 3029 "s59E5H2" 0 "Uridylate kinase"
"Chl_N02_00245" 3029 "s63E6H2" 0 "Uridylate kinase"
"Chl_N02_00246" 3030 "s59E13H2" 0 "Ribosome-recycling factor"
"Chl_N02_00246" 3030 "s59E25H2" 0 "Ribosome-recycling factor"
"Chl_N02_00246" 3030 "s59E5H2" 0 "Ribosome-recycling factor"
"Chl_N02_00246" 3030 "s63E6H2" 0 "Ribosome-recycling factor"
"Chl_N02_00247" 3031 "s59E13H2" 0 "Isoprenyl transferase"
"Chl_N02_00247" 3031 "s59E25H2" 0 "Isoprenyl transferase"
"Chl_N02_00247" 3031 "s59E5H2" 0 "Isoprenyl transferase"
"Chl_N02_00247" 3031 "s63E6H2" 0 "Isoprenyl transferase"
"Chl_N02_00248" 3032 "s59E13H2" 0 "Phosphatidate cytidylyltransferase"
"Chl_N02_00248" 3032 "s59E25H2" 0 "Phosphatidate cytidylyltransferase"
"Chl_N02_00248" 3032 "s59E5H2" 0.004779632 "Phosphatidate cytidylyltransferase"
"Chl_N02_00248" 3032 "s63E6H2" 0 "Phosphatidate cytidylyltransferase"
"Chl_N02_00249" 3033 "s59E13H2" 0.001046686 "1-deoxy-D-xylulose 5-phosphate reductoisomerase"
"Chl_N02_00249" 3033 "s59E25H2" 0 "1-deoxy-D-xylulose 5-phosphate reductoisomerase"
"Chl_N02_00249" 3033 "s59E5H2" 0.004113213 "1-deoxy-D-xylulose 5-phosphate reductoisomerase"
"Chl_N02_00249" 3033 "s63E6H2" 0 "1-deoxy-D-xylulose 5-phosphate reductoisomerase"
"Chl_N02_00250" 3034 "s59E13H2" 0 "Regulator of sigma-W protease RasP"
"Chl_N02_00250" 3034 "s59E25H2" 0 "Regulator of sigma-W protease RasP"
"Chl_N02_00250" 3034 "s59E5H2" 0 "Regulator of sigma-W protease RasP"
"Chl_N02_00250" 3034 "s63E6H2" 0 "Regulator of sigma-W protease RasP"
"Chl_N02_00251" 3035 "s59E13H2" 0 "4-hydroxy-3-methylbut-2-en-1-yl diphosphate synthase"
"Chl_N02_00251" 3035 "s59E25H2" 0 "4-hydroxy-3-methylbut-2-en-1-yl diphosphate synthase"
"Chl_N02_00251" 3035 "s59E5H2" 0 "4-hydroxy-3-methylbut-2-en-1-yl diphosphate synthase"
"Chl_N02_00251" 3035 "s63E6H2" 0 "4-hydroxy-3-methylbut-2-en-1-yl diphosphate synthase"
"Chl_N02_00252" 3036 "s59E13H2" 0 "hypothetical protein"
"Chl_N02_00252" 3036 "s59E25H2" 0 "hypothetical protein"
"Chl_N02_00252" 3036 "s59E5H2" 0 "hypothetical protein"
"Chl_N02_00252" 3036 "s63E6H2" 0 "hypothetical protein"
"Chl_N02_00253" 3037 "s59E13H2" 0 "hypothetical protein"
"Chl_N02_00253" 3037 "s59E25H2" 0 "hypothetical protein"
"Chl_N02_00253" 3037 "s59E5H2" 0 "hypothetical protein"
"Chl_N02_00253" 3037 "s63E6H2" 0 "hypothetical protein"
"Chl_N02_00254" 3038 "s59E13H2" 0 "putative ABC transporter ATP-binding protein"
"Chl_N02_00254" 3038 "s59E25H2" 0 "putative ABC transporter ATP-binding protein"

"Chl_N02_00254" 3038 "s59E5H2" 0 "putative ABC transporter ATP-binding protein"
"Chl_N02_00254" 3038 "s63E6H2" 0 "putative ABC transporter ATP-binding protein"
"Chl_N02_00255" 3039 "s59E13H2" 0 "ABC transporter permease YtrF precursor"
"Chl_N02_00255" 3039 "s59E25H2" 0 "ABC transporter permease YtrF precursor"
"Chl_N02_00255" 3039 "s59E5H2" 0 "ABC transporter permease YtrF precursor"
"Chl_N02_00255" 3039 "s63E6H2" 0 "ABC transporter permease YtrF precursor"
"Chl_N02_00256" 3040 "s59E13H2" 0 "Macrolide export ATP-binding/permease protein MacB"
"Chl_N02_00256" 3040 "s59E25H2" 0 "Macrolide export ATP-binding/permease protein MacB"
"Chl_N02_00256" 3040 "s59E5H2" 0 "Macrolide export ATP-binding/permease protein MacB"
"Chl_N02_00256" 3040 "s63E6H2" 0 "Macrolide export ATP-binding/permease protein MacB"
"Chl_N02_00257" 3041 "s59E13H2" 0 "hypothetical protein"
"Chl_N02_00257" 3041 "s59E25H2" 0 "hypothetical protein"
"Chl_N02_00257" 3041 "s59E5H2" 0 "hypothetical protein"
"Chl_N02_00257" 3041 "s63E6H2" 0 "hypothetical protein"
"Chl_N02_00258" 3042 "s59E13H2" 0 "hypothetical protein"
"Chl_N02_00258" 3042 "s59E25H2" 0 "hypothetical protein"
"Chl_N02_00258" 3042 "s59E5H2" 0 "hypothetical protein"
"Chl_N02_00258" 3042 "s63E6H2" 0 "hypothetical protein"
"Chl_N02_00259" 3043 "s59E13H2" 0 "Ferredoxin"
"Chl_N02_00259" 3043 "s59E25H2" 0 "Ferredoxin"
"Chl_N02_00259" 3043 "s59E5H2" 0 "Ferredoxin"
"Chl_N02_00259" 3043 "s63E6H2" 0 "Ferredoxin"
"Chl_N02_00260" 3044 "s59E13H2" 0 "hypothetical protein"
"Chl_N02_00260" 3044 "s59E25H2" 0 "hypothetical protein"
"Chl_N02_00260" 3044 "s59E5H2" 0 "hypothetical protein"
"Chl_N02_00260" 3044 "s63E6H2" 0 "hypothetical protein"
"Chl_N02_00264" 3048 "s59E13H2" 0 "hypothetical protein"
"Chl_N02_00264" 3048 "s59E25H2" 0 "hypothetical protein"
"Chl_N02_00264" 3048 "s59E5H2" 0.01208577 "hypothetical protein"
"Chl_N02_00264" 3048 "s63E6H2" 0 "hypothetical protein"
"Chl_N02_00265" 3049 "s59E13H2" 0 "Methylmalonate semialdehyde dehydrogenase [acylating]"
"Chl_N02_00265" 3049 "s59E25H2" 0 "Methylmalonate semialdehyde dehydrogenase [acylating]"
"Chl_N02_00265" 3049 "s59E5H2" 0.01088409 "Methylmalonate semialdehyde dehydrogenase [acylating]"
"Chl_N02_00265" 3049 "s63E6H2" 0 "Methylmalonate semialdehyde dehydrogenase [acylating]"
"Chl_N02_00266" 3050 "s59E13H2" 0 "1,3-propanediol dehydrogenase"
"Chl_N02_00266" 3050 "s59E25H2" 0 "1,3-propanediol dehydrogenase"
"Chl_N02_00266" 3050 "s59E5H2" 0 "1,3-propanediol dehydrogenase"
"Chl_N02_00266" 3050 "s63E6H2" 0 "1,3-propanediol dehydrogenase"
"Chl_N02_00268" 3052 "s59E13H2" 0 "High-affinity branched-chain amino acid transport ATP-binding protein LivF"
"Chl_N02_00268" 3052 "s59E25H2" 0 "High-affinity branched-chain amino acid transport ATP-binding protein LivF"
"Chl_N02_00268" 3052 "s59E5H2" 0 "High-affinity branched-chain amino acid transport ATP-binding protein LivF"
"Chl_N02_00268" 3052 "s63E6H2" 0 "High-affinity branched-chain amino acid transport ATP-binding protein LivF"
"Chl_N02_00269" 3053 "s59E13H2" 0 "Long-chain-fatty-acid--CoA ligase FadD15"
"Chl_N02_00269" 3053 "s59E25H2" 0 "Long-chain-fatty-acid--CoA ligase FadD15"
"Chl_N02_00269" 3053 "s59E5H2" 0 "Long-chain-fatty-acid--CoA ligase FadD15"
"Chl_N02_00269" 3053 "s63E6H2" 0 "Long-chain-fatty-acid--CoA ligase FadD15"
"Chl_N02_00270" 3054 "s59E13H2" 0 "putative branched-chain amino acid transport ATP-binding protein LivG"

"Chl_N02_00270" 3054 "s59E25H2" 0 "putative branched-chain amino acid transport ATP-binding protein LivG"
"Chl_N02_00270" 3054 "s59E5H2" 0 "putative branched-chain amino acid transport ATP-binding protein LivG"
"Chl_N02_00270" 3054 "s63E6H2" 0 "putative branched-chain amino acid transport ATP-binding protein LivG"
"Chl_N02_00271" 3055 "s59E13H2" 0 "hypothetical protein"
"Chl_N02_00271" 3055 "s59E25H2" 0 "hypothetical protein"
"Chl_N02_00271" 3055 "s59E5H2" 0 "hypothetical protein"
"Chl_N02_00271" 3055 "s63E6H2" 0 "hypothetical protein"
"Chl_N02_00272" 3056 "s59E13H2" 0 "leucine/isoleucine/valine transporter permease subunit"
"Chl_N02_00272" 3056 "s59E25H2" 0 "leucine/isoleucine/valine transporter permease subunit"
"Chl_N02_00272" 3056 "s59E5H2" 0 "leucine/isoleucine/valine transporter permease subunit"
"Chl_N02_00272" 3056 "s63E6H2" 0 "leucine/isoleucine/valine transporter permease subunit"
"Chl_N02_00273" 3057 "s59E13H2" 0 "High-affinity branched-chain amino acid transport system permease protein LivH"
"Chl_N02_00273" 3057 "s59E25H2" 0 "High-affinity branched-chain amino acid transport system permease protein LivH"
"Chl_N02_00273" 3057 "s59E5H2" 0 "High-affinity branched-chain amino acid transport system permease protein LivH"
"Chl_N02_00273" 3057 "s63E6H2" 0 "High-affinity branched-chain amino acid transport system permease protein LivH"
"Chl_N02_00274" 3058 "s59E13H2" 0 "hypothetical protein"
"Chl_N02_00274" 3058 "s59E25H2" 0 "hypothetical protein"
"Chl_N02_00274" 3058 "s59E5H2" 0 "hypothetical protein"
"Chl_N02_00274" 3058 "s63E6H2" 0 "hypothetical protein"
"Chl_N02_00275" 3059 "s59E13H2" 0 "hypothetical protein"
"Chl_N02_00275" 3059 "s59E25H2" 0 "hypothetical protein"
"Chl_N02_00275" 3059 "s59E5H2" 0 "hypothetical protein"
"Chl_N02_00275" 3059 "s63E6H2" 0 "hypothetical protein"
"Chl_N02_00276" 3060 "s59E13H2" 0 "hypothetical protein"
"Chl_N02_00276" 3060 "s59E25H2" 0 "hypothetical protein"
"Chl_N02_00276" 3060 "s59E5H2" 0 "hypothetical protein"
"Chl_N02_00276" 3060 "s63E6H2" 0 "hypothetical protein"
"Chl_N02_00277" 3061 "s59E13H2" 0 "putative HTH-type transcriptional regulator"
"Chl_N02_00277" 3061 "s59E25H2" 0 "putative HTH-type transcriptional regulator"
"Chl_N02_00277" 3061 "s59E5H2" 0 "putative HTH-type transcriptional regulator"
"Chl_N02_00277" 3061 "s63E6H2" 0 "putative HTH-type transcriptional regulator"
"Chl_N02_00278" 3062 "s59E13H2" 0 "Macrolide export ATP-binding/permease protein MacB"
"Chl_N02_00278" 3062 "s59E25H2" 0 "Macrolide export ATP-binding/permease protein MacB"
"Chl_N02_00278" 3062 "s59E5H2" 0 "Macrolide export ATP-binding/permease protein MacB"
"Chl_N02_00278" 3062 "s63E6H2" 0 "Macrolide export ATP-binding/permease protein MacB"
"Chl_N02_00279" 3063 "s59E13H2" 0 "hypothetical protein"
"Chl_N02_00279" 3063 "s59E25H2" 0 "hypothetical protein"
"Chl_N02_00279" 3063 "s59E5H2" 0 "hypothetical protein"
"Chl_N02_00279" 3063 "s63E6H2" 0 "hypothetical protein"
"Chl_N02_00280" 3064 "s59E13H2" 0 "Helix-turn-helix domain protein"
"Chl_N02_00280" 3064 "s59E25H2" 0 "Helix-turn-helix domain protein"
"Chl_N02_00280" 3064 "s59E5H2" 0 "Helix-turn-helix domain protein"
"Chl_N02_00280" 3064 "s63E6H2" 0 "Helix-turn-helix domain protein"
"Chl_N02_00282" 3066 "s59E13H2" 0 "Cyclic di-GMP phosphodiesterase response regulator RpfG"
"Chl_N02_00282" 3066 "s59E25H2" 0 "Cyclic di-GMP phosphodiesterase response regulator RpfG"
"Chl_N02_00282" 3066 "s59E5H2" 0 "Cyclic di-GMP phosphodiesterase response regulator RpfG"
"Chl_N02_00282" 3066 "s63E6H2" 0 "Cyclic di-GMP phosphodiesterase response regulator RpfG"

"Chl_N02_00283" 3067 "s59E13H2" 0 "Bifunctional homocysteine S-methyltransferase/5,10-methylenetetrahydrofolate reductase"
"Chl_N02_00283" 3067 "s59E25H2" 0 "Bifunctional homocysteine S-methyltransferase/5,10-methylenetetrahydrofolate reductase"
"Chl_N02_00283" 3067 "s59E5H2" 0 "Bifunctional homocysteine S-methyltransferase/5,10-methylenetetrahydrofolate reductase"
"Chl_N02_00283" 3067 "s63E6H2" 0 "Bifunctional homocysteine S-methyltransferase/5,10-methylenetetrahydrofolate reductase"
"Chl_N02_00284" 3068 "s59E13H2" 0 "hypothetical protein"
"Chl_N02_00284" 3068 "s59E25H2" 0 "hypothetical protein"
"Chl_N02_00284" 3068 "s59E5H2" 0 "hypothetical protein"
"Chl_N02_00284" 3068 "s63E6H2" 0 "hypothetical protein"
"Chl_N02_00285" 3069 "s59E13H2" 0 "NADP-reducing hydrogenase subunit HndC"
"Chl_N02_00285" 3069 "s59E25H2" 0 "NADP-reducing hydrogenase subunit HndC"
"Chl_N02_00285" 3069 "s59E5H2" 0 "NADP-reducing hydrogenase subunit HndC"
"Chl_N02_00285" 3069 "s63E6H2" 0 "NADP-reducing hydrogenase subunit HndC"
"Chl_N02_00286" 3070 "s59E13H2" 0 "NADP-reducing hydrogenase subunit HndA"
"Chl_N02_00286" 3070 "s59E25H2" 0 "NADP-reducing hydrogenase subunit HndA"
"Chl_N02_00286" 3070 "s59E5H2" 0 "NADP-reducing hydrogenase subunit HndA"
"Chl_N02_00286" 3070 "s63E6H2" 0 "NADP-reducing hydrogenase subunit HndA"
"Chl_N02_00287" 3071 "s59E13H2" 0 "Putative formate dehydrogenase"
"Chl_N02_00287" 3071 "s59E25H2" 0 "Putative formate dehydrogenase"
"Chl_N02_00287" 3071 "s59E5H2" 0 "Putative formate dehydrogenase"
"Chl_N02_00287" 3071 "s63E6H2" 0 "Putative formate dehydrogenase"
"Chl_N02_00288" 3072 "s59E13H2" 0 "Response regulator protein VraR"
"Chl_N02_00288" 3072 "s59E25H2" 0 "Response regulator protein VraR"
"Chl_N02_00288" 3072 "s59E5H2" 0 "Response regulator protein VraR"
"Chl_N02_00288" 3072 "s63E6H2" 0 "Response regulator protein VraR"
"Chl_N02_00289" 3073 "s59E13H2" 0 "Oxygen regulatory protein NreC"
"Chl_N02_00289" 3073 "s59E25H2" 0 "Oxygen regulatory protein NreC"
"Chl_N02_00289" 3073 "s59E5H2" 0 "Oxygen regulatory protein NreC"
"Chl_N02_00289" 3073 "s63E6H2" 0 "Oxygen regulatory protein NreC"
"Chl_N02_00290" 3074 "s59E13H2" 0 "Signal transduction histidine-protein kinase/phosphatase DegS"
"Chl_N02_00290" 3074 "s59E25H2" 0 "Signal transduction histidine-protein kinase/phosphatase DegS"
"Chl_N02_00290" 3074 "s59E5H2" 0 "Signal transduction histidine-protein kinase/phosphatase DegS"
"Chl_N02_00290" 3074 "s63E6H2" 0 "Signal transduction histidine-protein kinase/phosphatase DegS"
"Chl_N02_00293" 3077 "s59E13H2" 0 "acyl-CoA esterase"
"Chl_N02_00293" 3077 "s59E25H2" 0 "acyl-CoA esterase"
"Chl_N02_00293" 3077 "s59E5H2" 0 "acyl-CoA esterase"
"Chl_N02_00293" 3077 "s63E6H2" 0 "acyl-CoA esterase"
"Chl_N02_00295" 3079 "s59E13H2" 0 "Modification methylase MjaII"
"Chl_N02_00295" 3079 "s59E25H2" 0 "Modification methylase MjaII"
"Chl_N02_00295" 3079 "s59E5H2" 0 "Modification methylase MjaII"
"Chl_N02_00295" 3079 "s63E6H2" 0 "Modification methylase MjaII"
"Chl_N02_00296" 3080 "s59E13H2" 0 "hypothetical protein"
"Chl_N02_00296" 3080 "s59E25H2" 0 "hypothetical protein"
"Chl_N02_00296" 3080 "s59E5H2" 0 "hypothetical protein"
"Chl_N02_00296" 3080 "s63E6H2" 0 "hypothetical protein"
"Chl_N02_00298" 3082 "s59E13H2" 0 "hypothetical protein"
"Chl_N02_00298" 3082 "s59E25H2" 0 "hypothetical protein"
"Chl_N02_00298" 3082 "s59E5H2" 0 "hypothetical protein"

"Chl_N02_00298" 3082 "s63E6H2" 0 "hypothetical protein"
"Chl_N02_00299" 3083 "s59E13H2" 0 "hypothetical protein"
"Chl_N02_00299" 3083 "s59E25H2" 0 "hypothetical protein"
"Chl_N02_00299" 3083 "s59E5H2" 0 "hypothetical protein"
"Chl_N02_00299" 3083 "s63E6H2" 0 "hypothetical protein"
"Chl_N02_00301" 3085 "s59E13H2" 0 "hypothetical protein"
"Chl_N02_00301" 3085 "s59E25H2" 0 "hypothetical protein"
"Chl_N02_00301" 3085 "s59E5H2" 0 "hypothetical protein"
"Chl_N02_00301" 3085 "s63E6H2" 0 "hypothetical protein"
"Chl_N02_00302" 3086 "s59E13H2" 0 "Type-1 restriction enzyme R protein"
"Chl_N02_00302" 3086 "s59E25H2" 0 "Type-1 restriction enzyme R protein"
"Chl_N02_00302" 3086 "s59E5H2" 0 "Type-1 restriction enzyme R protein"
"Chl_N02_00302" 3086 "s63E6H2" 0 "Type-1 restriction enzyme R protein"
"Chl_N02_00303" 3087 "s59E13H2" 0 "hypothetical protein"
"Chl_N02_00303" 3087 "s59E25H2" 0 "hypothetical protein"
"Chl_N02_00303" 3087 "s59E5H2" 0 "hypothetical protein"
"Chl_N02_00303" 3087 "s63E6H2" 0 "hypothetical protein"
"Chl_N02_00304" 3088 "s59E13H2" 0 "Type-1 restriction enzyme MjaXIP specificity protein"
"Chl_N02_00304" 3088 "s59E25H2" 0 "Type-1 restriction enzyme MjaXIP specificity protein"
"Chl_N02_00304" 3088 "s59E5H2" 0 "Type-1 restriction enzyme MjaXIP specificity protein"
"Chl_N02_00304" 3088 "s63E6H2" 0 "Type-1 restriction enzyme MjaXIP specificity protein"
"Chl_N02_00305" 3089 "s59E13H2" 0 "Type I restriction enzyme EcoKI M protein"
"Chl_N02_00305" 3089 "s59E25H2" 0 "Type I restriction enzyme EcoKI M protein"
"Chl_N02_00305" 3089 "s59E5H2" 0 "Type I restriction enzyme EcoKI M protein"
"Chl_N02_00305" 3089 "s63E6H2" 0 "Type I restriction enzyme EcoKI M protein"
"Chl_N02_00306" 3090 "s59E13H2" 0 "Helix-turn-helix domain protein"
"Chl_N02_00306" 3090 "s59E25H2" 0 "Helix-turn-helix domain protein"
"Chl_N02_00306" 3090 "s59E5H2" 0 "Helix-turn-helix domain protein"
"Chl_N02_00306" 3090 "s63E6H2" 0 "Helix-turn-helix domain protein"
"Chl_N02_00309" 3093 "s59E13H2" 0 "RES domain protein"
"Chl_N02_00309" 3093 "s59E25H2" 0 "RES domain protein"
"Chl_N02_00309" 3093 "s59E5H2" 0 "RES domain protein"
"Chl_N02_00309" 3093 "s63E6H2" 0 "RES domain protein"
"Chl_N02_00311" 3095 "s59E13H2" 0 "hypothetical protein"
"Chl_N02_00311" 3095 "s59E25H2" 0 "hypothetical protein"
"Chl_N02_00311" 3095 "s59E5H2" 0 "hypothetical protein"
"Chl_N02_00311" 3095 "s63E6H2" 0 "hypothetical protein"
"Chl_N02_00312" 3096 "s59E13H2" 0 "6-aminohexanoate-dimer hydrolase"
"Chl_N02_00312" 3096 "s59E25H2" 0 "6-aminohexanoate-dimer hydrolase"
"Chl_N02_00312" 3096 "s59E5H2" 0 "6-aminohexanoate-dimer hydrolase"
"Chl_N02_00312" 3096 "s63E6H2" 0 "6-aminohexanoate-dimer hydrolase"
"Chl_N02_00313" 3097 "s59E13H2" 0 "Tyrosine recombinase XerD"
"Chl_N02_00313" 3097 "s59E25H2" 0 "Tyrosine recombinase XerD"
"Chl_N02_00313" 3097 "s59E5H2" 0 "Tyrosine recombinase XerD"
"Chl_N02_00313" 3097 "s63E6H2" 0 "Tyrosine recombinase XerD"
"Chl_N02_00314" 3098 "s59E13H2" 0 "hypothetical protein"
"Chl_N02_00314" 3098 "s59E25H2" 0 "hypothetical protein"

"Chl_N02_00314" 3098 "s59E5H2" 0 "hypothetical protein"
"Chl_N02_00314" 3098 "s63E6H2" 0 "hypothetical protein"
"Chl_N02_00319" 3103 "s59E13H2" 0 "DNA repair protein RadA"
"Chl_N02_00319" 3103 "s59E25H2" 0 "DNA repair protein RadA"
"Chl_N02_00319" 3103 "s59E5H2" 0 "DNA repair protein RadA"
"Chl_N02_00319" 3103 "s63E6H2" 0 "DNA repair protein RadA"
"Chl_N02_00320" 3104 "s59E13H2" 0 "Tyrosine recombinase XerD"
"Chl_N02_00320" 3104 "s59E25H2" 0 "Tyrosine recombinase XerD"
"Chl_N02_00320" 3104 "s59E5H2" 0 "Tyrosine recombinase XerD"
"Chl_N02_00320" 3104 "s63E6H2" 0 "Tyrosine recombinase XerD"
"Chl_N02_00321" 3105 "s59E13H2" 0 "MerR family regulatory protein"
"Chl_N02_00321" 3105 "s59E25H2" 0 "MerR family regulatory protein"
"Chl_N02_00321" 3105 "s59E5H2" 0 "MerR family regulatory protein"
"Chl_N02_00321" 3105 "s63E6H2" 0 "MerR family regulatory protein"
"Chl_N02_00323" 3107 "s59E13H2" 0 "Tyrosine recombinase XerD"
"Chl_N02_00323" 3107 "s59E25H2" 0 "Tyrosine recombinase XerD"
"Chl_N02_00323" 3107 "s59E5H2" 0 "Tyrosine recombinase XerD"
"Chl_N02_00323" 3107 "s63E6H2" 0 "Tyrosine recombinase XerD"
"Chl_N02_00325" 3109 "s59E13H2" 0 "Methionyl-tRNA formyltransferase"
"Chl_N02_00325" 3109 "s59E25H2" 0 "Methionyl-tRNA formyltransferase"
"Chl_N02_00325" 3109 "s59E5H2" 0 "Methionyl-tRNA formyltransferase"
"Chl_N02_00325" 3109 "s63E6H2" 0 "Methionyl-tRNA formyltransferase"
"Chl_N02_00326" 3110 "s59E13H2" 0 "Lipoyl synthase"
"Chl_N02_00326" 3110 "s59E25H2" 0 "Lipoyl synthase"
"Chl_N02_00326" 3110 "s59E5H2" 0 "Lipoyl synthase"
"Chl_N02_00326" 3110 "s63E6H2" 0 "Lipoyl synthase"
"Chl_N02_00327" 3111 "s59E13H2" 0 "Octanoyltransferase"
"Chl_N02_00327" 3111 "s59E25H2" 0 "Octanoyltransferase"
"Chl_N02_00327" 3111 "s59E5H2" 0 "Octanoyltransferase"
"Chl_N02_00327" 3111 "s63E6H2" 0 "Octanoyltransferase"
"Chl_N02_00328" 3112 "s59E13H2" 0 "Dihydrolipoyl dehydrogenase"
"Chl_N02_00328" 3112 "s59E25H2" 0 "Dihydrolipoyl dehydrogenase"
"Chl_N02_00328" 3112 "s59E5H2" 0 "Dihydrolipoyl dehydrogenase"
"Chl_N02_00328" 3112 "s63E6H2" 0 "Dihydrolipoyl dehydrogenase"
"Chl_N02_00329" 3113 "s59E13H2" 0 "Dihydrolipoyllysine-residue acetyltransferase component of pyruvate dehydrogenase complex"
"Chl_N02_00329" 3113 "s59E25H2" 0 "Dihydrolipoyllysine-residue acetyltransferase component of pyruvate dehydrogenase complex"
"Chl_N02_00329" 3113 "s59E5H2" 0 "Dihydrolipoyllysine-residue acetyltransferase component of pyruvate dehydrogenase complex"
"Chl_N02_00329" 3113 "s63E6H2" 0 "Dihydrolipoyllysine-residue acetyltransferase component of pyruvate dehydrogenase complex"
"Chl_N02_00330" 3114 "s59E13H2" 0 "Acetoin:2,6-dichlorophenolindophenol oxidoreductase subunit beta"
"Chl_N02_00330" 3114 "s59E25H2" 0 "Acetoin:2,6-dichlorophenolindophenol oxidoreductase subunit beta"
"Chl_N02_00330" 3114 "s59E5H2" 0 "Acetoin:2,6-dichlorophenolindophenol oxidoreductase subunit beta"
"Chl_N02_00330" 3114 "s63E6H2" 0 "Acetoin:2,6-dichlorophenolindophenol oxidoreductase subunit beta"
"Chl_N02_00331" 3115 "s59E13H2" 0 "Acetoin:2,6-dichlorophenolindophenol oxidoreductase subunit alpha"
"Chl_N02_00331" 3115 "s59E25H2" 0 "Acetoin:2,6-dichlorophenolindophenol oxidoreductase subunit alpha"
"Chl_N02_00331" 3115 "s59E5H2" 0 "Acetoin:2,6-dichlorophenolindophenol oxidoreductase subunit alpha"
"Chl_N02_00331" 3115 "s63E6H2" 0 "Acetoin:2,6-dichlorophenolindophenol oxidoreductase subunit alpha"
"Chl_N02_00332" 3116 "s59E13H2" 0 "Queuine tRNA-ribosyltransferase"

"Chl_N02_00332" 3116 "s59E25H2" 0 "Queuine tRNA-ribosyltransferase"
"Chl_N02_00332" 3116 "s59E5H2" 0 "Queuine tRNA-ribosyltransferase"
"Chl_N02_00332" 3116 "s63E6H2" 0 "Queuine tRNA-ribosyltransferase"
"Chl_N02_00333" 3117 "s59E13H2" 0 "Inner membrane protein YhhQ"
"Chl_N02_00333" 3117 "s59E25H2" 0 "Inner membrane protein YhhQ"
"Chl_N02_00333" 3117 "s59E5H2" 0 "Inner membrane protein YhhQ"
"Chl_N02_00333" 3117 "s63E6H2" 0 "Inner membrane protein YhhQ"
"Chl_N02_00334" 3118 "s59E13H2" 0 "hypothetical protein"
"Chl_N02_00334" 3118 "s59E25H2" 0 "hypothetical protein"
"Chl_N02_00334" 3118 "s59E5H2" 0 "hypothetical protein"
"Chl_N02_00334" 3118 "s63E6H2" 0 "hypothetical protein"
"Chl_N02_00335" 3119 "s59E13H2" 0 "Sulfopyruvate decarboxylase subunit beta"
"Chl_N02_00335" 3119 "s59E25H2" 0 "Sulfopyruvate decarboxylase subunit beta"
"Chl_N02_00335" 3119 "s59E5H2" 0 "Sulfopyruvate decarboxylase subunit beta"
"Chl_N02_00335" 3119 "s63E6H2" 0 "Sulfopyruvate decarboxylase subunit beta"
"Chl_N02_00336" 3120 "s59E13H2" 0 "Sulfopyruvate decarboxylase subunit alpha"
"Chl_N02_00336" 3120 "s59E25H2" 0 "Sulfopyruvate decarboxylase subunit alpha"
"Chl_N02_00336" 3120 "s59E5H2" 0 "Sulfopyruvate decarboxylase subunit alpha"
"Chl_N02_00336" 3120 "s63E6H2" 0 "Sulfopyruvate decarboxylase subunit alpha"
"Chl_N02_00337" 3121 "s59E13H2" 0 "S-adenosylmethionine:tRNA ribosyltransferase-isomerase"
"Chl_N02_00337" 3121 "s59E25H2" 0 "S-adenosylmethionine:tRNA ribosyltransferase-isomerase"
"Chl_N02_00337" 3121 "s59E5H2" 0 "S-adenosylmethionine:tRNA ribosyltransferase-isomerase"
"Chl_N02_00337" 3121 "s63E6H2" 0 "S-adenosylmethionine:tRNA ribosyltransferase-isomerase"
"Chl_N02_00338" 3122 "s59E13H2" 0 "Bacterioferritin"
"Chl_N02_00338" 3122 "s59E25H2" 0 "Bacterioferritin"
"Chl_N02_00338" 3122 "s59E5H2" 0.0115935 "Bacterioferritin"
"Chl_N02_00338" 3122 "s63E6H2" 0 "Bacterioferritin"
"Chl_N02_00339" 3123 "s59E13H2" 0 "Desulfoferrodoxin"
"Chl_N02_00339" 3123 "s59E25H2" 0 "Desulfoferrodoxin"
"Chl_N02_00339" 3123 "s59E5H2" 0 "Desulfoferrodoxin"
"Chl_N02_00339" 3123 "s63E6H2" 0 "Desulfoferrodoxin"
"Chl_N02_00340" 3124 "s59E13H2" 0 "hypothetical protein"
"Chl_N02_00340" 3124 "s59E25H2" 0 "hypothetical protein"
"Chl_N02_00340" 3124 "s59E5H2" 0 "hypothetical protein"
"Chl_N02_00340" 3124 "s63E6H2" 0 "hypothetical protein"
"Chl_N02_00341" 3125 "s59E13H2" 0 "Rubredoxin"
"Chl_N02_00341" 3125 "s59E25H2" 0 "Rubredoxin"
"Chl_N02_00341" 3125 "s59E5H2" 0 "Rubredoxin"
"Chl_N02_00341" 3125 "s63E6H2" 0 "Rubredoxin"
"Chl_N02_00342" 3126 "s59E13H2" 0 "High molecular weight rubredoxin"
"Chl_N02_00342" 3126 "s59E25H2" 0 "High molecular weight rubredoxin"
"Chl_N02_00342" 3126 "s59E5H2" 0 "High molecular weight rubredoxin"
"Chl_N02_00342" 3126 "s63E6H2" 0 "High molecular weight rubredoxin"
"Chl_N02_00343" 3127 "s59E13H2" 0 "FeS cluster assembly protein SufB"
"Chl_N02_00343" 3127 "s59E25H2" 0 "FeS cluster assembly protein SufB"
"Chl_N02_00343" 3127 "s59E5H2" 0 "FeS cluster assembly protein SufB"
"Chl_N02_00343" 3127 "s63E6H2" 0 "FeS cluster assembly protein SufB"

"Chl_N02_00344" 3128 "s59E13H2" 0 "putative ABC transporter ATP-binding protein"
"Chl_N02_00344" 3128 "s59E25H2" 0 "putative ABC transporter ATP-binding protein"
"Chl_N02_00344" 3128 "s59E5H2" 0 "putative ABC transporter ATP-binding protein"
"Chl_N02_00344" 3128 "s63E6H2" 0 "putative ABC transporter ATP-binding protein"
"Chl_N02_00345" 3129 "s59E13H2" 0 "putative trifunctional 2-polyprenylphenol hydroxylase/glutamate synthase subunit beta/ferritin domain-containing protein"
"Chl_N02_00345" 3129 "s59E25H2" 0 "putative trifunctional 2-polyprenylphenol hydroxylase/glutamate synthase subunit beta/ferritin domain-containing protein"
"Chl_N02_00345" 3129 "s59E5H2" 0 "putative trifunctional 2-polyprenylphenol hydroxylase/glutamate synthase subunit beta/ferritin domain-containing protein"
"Chl_N02_00345" 3129 "s63E6H2" 0 "putative trifunctional 2-polyprenylphenol hydroxylase/glutamate synthase subunit beta/ferritin domain-containing protein"
"Chl_N02_00347" 3131 "s59E13H2" 0 "Protease 1 precursor"
"Chl_N02_00347" 3131 "s59E25H2" 0 "Protease 1 precursor"
"Chl_N02_00347" 3131 "s59E5H2" 0.00271285 "Protease 1 precursor"
"Chl_N02_00347" 3131 "s63E6H2" 0 "Protease 1 precursor"
"Chl_N02_00348" 3132 "s59E13H2" 0 "putative peptidyl-prolyl cis-trans isomerase"
"Chl_N02_00348" 3132 "s59E25H2" 0 "putative peptidyl-prolyl cis-trans isomerase"
"Chl_N02_00348" 3132 "s59E5H2" 0 "putative peptidyl-prolyl cis-trans isomerase"
"Chl_N02_00348" 3132 "s63E6H2" 0 "putative peptidyl-prolyl cis-trans isomerase"
"Chl_N02_00349" 3133 "s59E13H2" 0 "cell division protein FtsZ"
"Chl_N02_00349" 3133 "s59E25H2" 0 "cell division protein FtsZ"
"Chl_N02_00349" 3133 "s59E5H2" 0 "cell division protein FtsZ"
"Chl_N02_00349" 3133 "s63E6H2" 0 "cell division protein FtsZ"
"Chl_N02_00350" 3134 "s59E13H2" 0 "hypothetical protein"
"Chl_N02_00350" 3134 "s59E25H2" 0 "hypothetical protein"
"Chl_N02_00350" 3134 "s59E5H2" 0 "hypothetical protein"
"Chl_N02_00350" 3134 "s63E6H2" 0 "hypothetical protein"
"Chl_N02_00353" 3137 "s59E13H2" 0 "Cell division protein FtsA"
"Chl_N02_00353" 3137 "s59E25H2" 0 "Cell division protein FtsA"
"Chl_N02_00353" 3137 "s59E5H2" 0 "Cell division protein FtsA"
"Chl_N02_00353" 3137 "s63E6H2" 0 "Cell division protein FtsA"
"Chl_N02_00354" 3138 "s59E13H2" 0 "Cell division protein FtsZ"
"Chl_N02_00354" 3138 "s59E25H2" 0 "Cell division protein FtsZ"
"Chl_N02_00354" 3138 "s59E5H2" 0 "Cell division protein FtsZ"
"Chl_N02_00354" 3138 "s63E6H2" 0 "Cell division protein FtsZ"
"Chl_N02_00355" 3139 "s59E13H2" 0 "hypothetical protein"
"Chl_N02_00355" 3139 "s59E25H2" 0 "hypothetical protein"
"Chl_N02_00355" 3139 "s59E5H2" 0 "hypothetical protein"
"Chl_N02_00355" 3139 "s63E6H2" 0 "hypothetical protein"
"Chl_N02_00359" 3143 "s59E13H2" 0 "Major Facilitator Superfamily protein"
"Chl_N02_00359" 3143 "s59E25H2" 0 "Major Facilitator Superfamily protein"
"Chl_N02_00359" 3143 "s59E5H2" 0 "Major Facilitator Superfamily protein"
"Chl_N02_00359" 3143 "s63E6H2" 0 "Major Facilitator Superfamily protein"
"Chl_N02_00361" 3145 "s59E13H2" 0 "L-lysine 2,3-aminomutase"
"Chl_N02_00361" 3145 "s59E25H2" 0 "L-lysine 2,3-aminomutase"
"Chl_N02_00361" 3145 "s59E5H2" 0 "L-lysine 2,3-aminomutase"
"Chl_N02_00361" 3145 "s63E6H2" 0 "L-lysine 2,3-aminomutase"
"Chl_N02_00362" 3146 "s59E13H2" 0 "D-alanine--D-alanine ligase"
"Chl_N02_00362" 3146 "s59E25H2" 0 "D-alanine--D-alanine ligase"
"Chl_N02_00362" 3146 "s59E5H2" 0 "D-alanine--D-alanine ligase"

"Chl_N02_00362" 3146 "s63E6H2" 0 "D-alanine--D-alanine ligase"
"Chl_N02_00363" 3147 "s59E13H2" 0 "Vancomycin B-type resistance protein VanB"
"Chl_N02_00363" 3147 "s59E25H2" 0 "Vancomycin B-type resistance protein VanB"
"Chl_N02_00363" 3147 "s59E5H2" 0 "Vancomycin B-type resistance protein VanB"
"Chl_N02_00363" 3147 "s63E6H2" 0 "Vancomycin B-type resistance protein VanB"
"Chl_N02_00364" 3148 "s59E13H2" 0 "aminoalkylphosphonic acid N-acetyltransferase"
"Chl_N02_00364" 3148 "s59E25H2" 0 "aminoalkylphosphonic acid N-acetyltransferase"
"Chl_N02_00364" 3148 "s59E5H2" 0 "aminoalkylphosphonic acid N-acetyltransferase"
"Chl_N02_00364" 3148 "s63E6H2" 0 "aminoalkylphosphonic acid N-acetyltransferase"
"Chl_N02_00365" 3149 "s59E13H2" 0 "Glycerol-3-phosphate acyltransferase"
"Chl_N02_00365" 3149 "s59E25H2" 0 "Glycerol-3-phosphate acyltransferase"
"Chl_N02_00365" 3149 "s59E5H2" 0 "Glycerol-3-phosphate acyltransferase"
"Chl_N02_00365" 3149 "s63E6H2" 0 "Glycerol-3-phosphate acyltransferase"
"Chl_N02_00366" 3150 "s59E13H2" 0 "SnoaL-like polyketide cyclase"
"Chl_N02_00366" 3150 "s59E25H2" 0 "SnoaL-like polyketide cyclase"
"Chl_N02_00366" 3150 "s59E5H2" 0 "SnoaL-like polyketide cyclase"
"Chl_N02_00366" 3150 "s63E6H2" 0 "SnoaL-like polyketide cyclase"
"Chl_N02_00367" 3151 "s59E13H2" 0 "hypothetical protein"
"Chl_N02_00367" 3151 "s59E25H2" 0 "hypothetical protein"
"Chl_N02_00367" 3151 "s59E5H2" 0 "hypothetical protein"
"Chl_N02_00367" 3151 "s63E6H2" 0 "hypothetical protein"
"Chl_N02_00369" 3153 "s59E13H2" 0 "hypothetical protein"
"Chl_N02_00369" 3153 "s59E25H2" 0 "hypothetical protein"
"Chl_N02_00369" 3153 "s59E5H2" 0 "hypothetical protein"
"Chl_N02_00369" 3153 "s63E6H2" 0 "hypothetical protein"
"Chl_N02_00372" 3156 "s59E13H2" 0 "Tyrosine recombinase XerC"
"Chl_N02_00372" 3156 "s59E25H2" 0 "Tyrosine recombinase XerC"
"Chl_N02_00372" 3156 "s59E5H2" 0 "Tyrosine recombinase XerC"
"Chl_N02_00372" 3156 "s63E6H2" 0 "Tyrosine recombinase XerC"
"Chl_N02_00374" 3158 "s59E13H2" 0 "hypothetical protein"
"Chl_N02_00374" 3158 "s59E25H2" 0 "hypothetical protein"
"Chl_N02_00374" 3158 "s59E5H2" 0 "hypothetical protein"
"Chl_N02_00374" 3158 "s63E6H2" 0 "hypothetical protein"
"Chl_N02_00375" 3159 "s59E13H2" 0 "Putative thymidylate synthase"
"Chl_N02_00375" 3159 "s59E25H2" 0 "Putative thymidylate synthase"
"Chl_N02_00375" 3159 "s59E5H2" 0 "Putative thymidylate synthase"
"Chl_N02_00375" 3159 "s63E6H2" 0 "Putative thymidylate synthase"
"Chl_N02_00376" 3160 "s59E13H2" 0 "hypothetical protein"
"Chl_N02_00376" 3160 "s59E25H2" 0 "hypothetical protein"
"Chl_N02_00376" 3160 "s59E5H2" 0 "hypothetical protein"
"Chl_N02_00376" 3160 "s63E6H2" 0 "hypothetical protein"
"Chl_N02_00379" 3163 "s59E13H2" 0 "hypothetical protein"
"Chl_N02_00379" 3163 "s59E25H2" 0 "hypothetical protein"
"Chl_N02_00379" 3163 "s59E5H2" 0 "hypothetical protein"
"Chl_N02_00379" 3163 "s63E6H2" 0 "hypothetical protein"
"Chl_N02_00380" 3164 "s59E13H2" 0 "hypothetical protein"
"Chl_N02_00380" 3164 "s59E25H2" 0 "hypothetical protein"

"Chl_N02_00380" 3164 "s59E5H2" 0 "hypothetical protein"
"Chl_N02_00380" 3164 "s63E6H2" 0 "hypothetical protein"
"Chl_N02_00381" 3165 "s59E13H2" 0 "tRNA-splicing ligase RtcB"
"Chl_N02_00381" 3165 "s59E25H2" 0 "tRNA-splicing ligase RtcB"
"Chl_N02_00381" 3165 "s59E5H2" 0 "tRNA-splicing ligase RtcB"
"Chl_N02_00381" 3165 "s63E6H2" 0 "tRNA-splicing ligase RtcB"
"Chl_N02_00382" 3166 "s59E13H2" 0 "Cysteine synthase"
"Chl_N02_00382" 3166 "s59E25H2" 0 "Cysteine synthase"
"Chl_N02_00382" 3166 "s59E5H2" 0 "Cysteine synthase"
"Chl_N02_00382" 3166 "s63E6H2" 0 "Cysteine synthase"
"Chl_N02_00383" 3167 "s59E13H2" 0 "Nitroreductase family protein"
"Chl_N02_00383" 3167 "s59E25H2" 0 "Nitroreductase family protein"
"Chl_N02_00383" 3167 "s59E5H2" 0 "Nitroreductase family protein"
"Chl_N02_00383" 3167 "s63E6H2" 0 "Nitroreductase family protein"
"Chl_N02_00384" 3168 "s59E13H2" 0 "hypothetical protein"
"Chl_N02_00384" 3168 "s59E25H2" 0 "hypothetical protein"
"Chl_N02_00384" 3168 "s59E5H2" 0 "hypothetical protein"
"Chl_N02_00384" 3168 "s63E6H2" 0 "hypothetical protein"
"Chl_N02_00385" 3169 "s59E13H2" 0 "Heterodisulfide reductase subunit A-like protein"
"Chl_N02_00385" 3169 "s59E25H2" 0 "Heterodisulfide reductase subunit A-like protein"
"Chl_N02_00385" 3169 "s59E5H2" 0 "Heterodisulfide reductase subunit A-like protein"
"Chl_N02_00385" 3169 "s63E6H2" 0 "Heterodisulfide reductase subunit A-like protein"
"Chl_N02_00386" 3170 "s59E13H2" 0 "hypothetical protein"
"Chl_N02_00386" 3170 "s59E25H2" 0 "hypothetical protein"
"Chl_N02_00386" 3170 "s59E5H2" 0 "hypothetical protein"
"Chl_N02_00386" 3170 "s63E6H2" 0 "hypothetical protein"
"Chl_N02_00388" 3172 "s59E13H2" 0 "Divalent-cation tolerance protein CutA"
"Chl_N02_00388" 3172 "s59E25H2" 0 "Divalent-cation tolerance protein CutA"
"Chl_N02_00388" 3172 "s59E5H2" 0 "Divalent-cation tolerance protein CutA"
"Chl_N02_00388" 3172 "s63E6H2" 0 "Divalent-cation tolerance protein CutA"
"Chl_N02_00389" 3173 "s59E13H2" 0 "NIL domain protein"
"Chl_N02_00389" 3173 "s59E25H2" 0 "NIL domain protein"
"Chl_N02_00389" 3173 "s59E5H2" 0 "NIL domain protein"
"Chl_N02_00389" 3173 "s63E6H2" 0 "NIL domain protein"
"Chl_N02_00390" 3174 "s59E13H2" 0 "Hydrogenase isoenzymes nickel incorporation protein HypB"
"Chl_N02_00390" 3174 "s59E25H2" 0 "Hydrogenase isoenzymes nickel incorporation protein HypB"
"Chl_N02_00390" 3174 "s59E5H2" 0 "Hydrogenase isoenzymes nickel incorporation protein HypB"
"Chl_N02_00390" 3174 "s63E6H2" 0 "Hydrogenase isoenzymes nickel incorporation protein HypB"
"Chl_N02_00391" 3175 "s59E13H2" 0 "putative hydrogenase nickel incorporation protein HypA"
"Chl_N02_00391" 3175 "s59E25H2" 0 "putative hydrogenase nickel incorporation protein HypA"
"Chl_N02_00391" 3175 "s59E5H2" 0 "putative hydrogenase nickel incorporation protein HypA"
"Chl_N02_00391" 3175 "s63E6H2" 0 "putative hydrogenase nickel incorporation protein HypA"
"Chl_N02_00392" 3176 "s59E13H2" 0 "Hydrogenase expression/formation protein HypE"
"Chl_N02_00392" 3176 "s59E25H2" 0 "Hydrogenase expression/formation protein HypE"
"Chl_N02_00392" 3176 "s59E5H2" 0 "Hydrogenase expression/formation protein HypE"
"Chl_N02_00392" 3176 "s63E6H2" 0 "Hydrogenase expression/formation protein HypE"
"Chl_N02_00393" 3177 "s59E13H2" 0 "Hydrogenase expression/formation protein HypD"

"Chl_N02_00393" 3177 "s59E25H2" 0 "Hydrogenase expression/formation protein HypD"
"Chl_N02_00393" 3177 "s59E5H2" 0 "Hydrogenase expression/formation protein HypD"
"Chl_N02_00393" 3177 "s63E6H2" 0 "Hydrogenase expression/formation protein HypD"
"Chl_N02_00394" 3178 "s59E13H2" 0 "Hydrogenase isoenzymes formation protein HypC"
"Chl_N02_00394" 3178 "s59E25H2" 0 "Hydrogenase isoenzymes formation protein HypC"
"Chl_N02_00394" 3178 "s59E5H2" 0 "Hydrogenase isoenzymes formation protein HypC"
"Chl_N02_00394" 3178 "s63E6H2" 0 "Hydrogenase isoenzymes formation protein HypC"
"Chl_N02_00395" 3179 "s59E13H2" 0 "Carbamoyltransferase HypF"
"Chl_N02_00395" 3179 "s59E25H2" 0 "Carbamoyltransferase HypF"
"Chl_N02_00395" 3179 "s59E5H2" 0 "Carbamoyltransferase HypF"
"Chl_N02_00395" 3179 "s63E6H2" 0 "Carbamoyltransferase HypF"
"Chl_N02_00396" 3180 "s59E13H2" 0 "hypothetical protein"
"Chl_N02_00396" 3180 "s59E25H2" 0 "hypothetical protein"
"Chl_N02_00396" 3180 "s59E5H2" 0 "hypothetical protein"
"Chl_N02_00396" 3180 "s63E6H2" 0 "hypothetical protein"
"Chl_N02_00397" 3181 "s59E13H2" 0 "Sulfide dehydrogenase subunit alpha precursor"
"Chl_N02_00397" 3181 "s59E25H2" 0 "Sulfide dehydrogenase subunit alpha precursor"
"Chl_N02_00397" 3181 "s59E5H2" 0 "Sulfide dehydrogenase subunit alpha precursor"
"Chl_N02_00397" 3181 "s63E6H2" 0 "Sulfide dehydrogenase subunit alpha precursor"
"Chl_N02_00398" 3182 "s59E13H2" 0 "F420-non-reducing hydrogenase vhc iron-sulfur subunit D"
"Chl_N02_00398" 3182 "s59E25H2" 0 "F420-non-reducing hydrogenase vhc iron-sulfur subunit D"
"Chl_N02_00398" 3182 "s59E5H2" 0 "F420-non-reducing hydrogenase vhc iron-sulfur subunit D"
"Chl_N02_00398" 3182 "s63E6H2" 0 "F420-non-reducing hydrogenase vhc iron-sulfur subunit D"
"Chl_N02_00399" 3183 "s59E13H2" 0 "Hydrogenase 2 maturation protease"
"Chl_N02_00399" 3183 "s59E25H2" 0 "Hydrogenase 2 maturation protease"
"Chl_N02_00399" 3183 "s59E5H2" 0 "Hydrogenase 2 maturation protease"
"Chl_N02_00399" 3183 "s63E6H2" 0 "Hydrogenase 2 maturation protease"
"Chl_N02_00400" 3184 "s59E13H2" 0 "Cysteine synthase"
"Chl_N02_00400" 3184 "s59E25H2" 0 "Cysteine synthase"
"Chl_N02_00400" 3184 "s59E5H2" 0 "Cysteine synthase"
"Chl_N02_00400" 3184 "s63E6H2" 0 "Cysteine synthase"
"Chl_N02_00401" 3185 "s59E13H2" 0 "FeS cluster assembly protein SufB"
"Chl_N02_00401" 3185 "s59E25H2" 0 "FeS cluster assembly protein SufB"
"Chl_N02_00401" 3185 "s59E5H2" 0 "FeS cluster assembly protein SufB"
"Chl_N02_00401" 3185 "s63E6H2" 0 "FeS cluster assembly protein SufB"
"Chl_N02_00402" 3186 "s59E13H2" 0 "Vegetative protein 296"
"Chl_N02_00402" 3186 "s59E25H2" 0 "Vegetative protein 296"
"Chl_N02_00402" 3186 "s59E5H2" 0 "Vegetative protein 296"
"Chl_N02_00402" 3186 "s63E6H2" 0 "Vegetative protein 296"
"Chl_N02_00403" 3187 "s59E13H2" 0 "Fe/S biogenesis protein NfuA"
"Chl_N02_00403" 3187 "s59E25H2" 0 "Fe/S biogenesis protein NfuA"
"Chl_N02_00403" 3187 "s59E5H2" 0 "Fe/S biogenesis protein NfuA"
"Chl_N02_00403" 3187 "s63E6H2" 0 "Fe/S biogenesis protein NfuA"
"Chl_N02_00404" 3188 "s59E13H2" 0 "Sulfide dehydrogenase subunit alpha precursor"
"Chl_N02_00404" 3188 "s59E25H2" 0 "Sulfide dehydrogenase subunit alpha precursor"
"Chl_N02_00404" 3188 "s59E5H2" 0 "Sulfide dehydrogenase subunit alpha precursor"
"Chl_N02_00404" 3188 "s63E6H2" 0 "Sulfide dehydrogenase subunit alpha precursor"

"Chl_N02_00405" 3189 "s59E13H2" 0 "F420-non-reducing hydrogenase iron-sulfur subunit A"
"Chl_N02_00405" 3189 "s59E25H2" 0 "F420-non-reducing hydrogenase iron-sulfur subunit A"
"Chl_N02_00405" 3189 "s59E5H2" 0 "F420-non-reducing hydrogenase iron-sulfur subunit A"
"Chl_N02_00405" 3189 "s63E6H2" 0 "F420-non-reducing hydrogenase iron-sulfur subunit A"
"Chl_N02_00406" 3190 "s59E13H2" 0 "F420-non-reducing hydrogenase iron-sulfur subunit G"
"Chl_N02_00406" 3190 "s59E25H2" 0 "F420-non-reducing hydrogenase iron-sulfur subunit G"
"Chl_N02_00406" 3190 "s59E5H2" 0 "F420-non-reducing hydrogenase iron-sulfur subunit G"
"Chl_N02_00406" 3190 "s63E6H2" 0 "F420-non-reducing hydrogenase iron-sulfur subunit G"
"Chl_N02_00407" 3191 "s59E13H2" 0 "Glucose 1-dehydrogenase"
"Chl_N02_00407" 3191 "s59E25H2" 0 "Glucose 1-dehydrogenase"
"Chl_N02_00407" 3191 "s59E5H2" 0.0138899 "Glucose 1-dehydrogenase"
"Chl_N02_00407" 3191 "s63E6H2" 0 "Glucose 1-dehydrogenase"
"Chl_N02_00408" 3192 "s59E13H2" 0 "Phosphoenolpyruvate carboxykinase [ATP]"
"Chl_N02_00408" 3192 "s59E25H2" 0 "Phosphoenolpyruvate carboxykinase [ATP]"
"Chl_N02_00408" 3192 "s59E5H2" 0 "Phosphoenolpyruvate carboxykinase [ATP]"
"Chl_N02_00408" 3192 "s63E6H2" 0 "Phosphoenolpyruvate carboxykinase [ATP]"
"Chl_N02_00409" 3193 "s59E13H2" 0 "Acetyltransferase (GNAT) family protein"
"Chl_N02_00409" 3193 "s59E25H2" 0 "Acetyltransferase (GNAT) family protein"
"Chl_N02_00409" 3193 "s59E5H2" 0 "Acetyltransferase (GNAT) family protein"
"Chl_N02_00409" 3193 "s63E6H2" 0 "Acetyltransferase (GNAT) family protein"
"Chl_N02_00410" 3194 "s59E13H2" 0 "hypothetical protein"
"Chl_N02_00410" 3194 "s59E25H2" 0 "hypothetical protein"
"Chl_N02_00410" 3194 "s59E5H2" 0 "hypothetical protein"
"Chl_N02_00410" 3194 "s63E6H2" 0 "hypothetical protein"
"Chl_N02_00411" 3195 "s59E13H2" 0 "Threonylcarbamoyladenosine tRNA methylthiotransferase MtaB"
"Chl_N02_00411" 3195 "s59E25H2" 0 "Threonylcarbamoyladenosine tRNA methylthiotransferase MtaB"
"Chl_N02_00411" 3195 "s59E5H2" 0 "Threonylcarbamoyladenosine tRNA methylthiotransferase MtaB"
"Chl_N02_00411" 3195 "s63E6H2" 0 "Threonylcarbamoyladenosine tRNA methylthiotransferase MtaB"
"Chl_N02_00412" 3196 "s59E13H2" 0 "CoB--CoM heterodisulfide reductase iron-sulfur subunit A"
"Chl_N02_00412" 3196 "s59E25H2" 0 "CoB--CoM heterodisulfide reductase iron-sulfur subunit A"
"Chl_N02_00412" 3196 "s59E5H2" 0 "CoB--CoM heterodisulfide reductase iron-sulfur subunit A"
"Chl_N02_00412" 3196 "s63E6H2" 0 "CoB--CoM heterodisulfide reductase iron-sulfur subunit A"
"Chl_N02_00413" 3197 "s59E13H2" 0 "F420-non-reducing hydrogenase iron-sulfur subunit D"
"Chl_N02_00413" 3197 "s59E25H2" 0 "F420-non-reducing hydrogenase iron-sulfur subunit D"
"Chl_N02_00413" 3197 "s59E5H2" 0 "F420-non-reducing hydrogenase iron-sulfur subunit D"
"Chl_N02_00413" 3197 "s63E6H2" 0 "F420-non-reducing hydrogenase iron-sulfur subunit D"
"Chl_N02_00414" 3198 "s59E13H2" 0 "CoB--CoM heterodisulfide reductase iron-sulfur subunit A"
"Chl_N02_00414" 3198 "s59E25H2" 0 "CoB--CoM heterodisulfide reductase iron-sulfur subunit A"
"Chl_N02_00414" 3198 "s59E5H2" 0 "CoB--CoM heterodisulfide reductase iron-sulfur subunit A"
"Chl_N02_00414" 3198 "s63E6H2" 0 "CoB--CoM heterodisulfide reductase iron-sulfur subunit A"
"Chl_N02_00415" 3199 "s59E13H2" 0 "F420-non-reducing hydrogenase vhc iron-sulfur subunit D"
"Chl_N02_00415" 3199 "s59E25H2" 0 "F420-non-reducing hydrogenase vhc iron-sulfur subunit D"
"Chl_N02_00415" 3199 "s59E5H2" 0 "F420-non-reducing hydrogenase vhc iron-sulfur subunit D"
"Chl_N02_00415" 3199 "s63E6H2" 0 "F420-non-reducing hydrogenase vhc iron-sulfur subunit D"
"Chl_N02_00416" 3200 "s59E13H2" 0 "Formate dehydrogenase subunit beta"
"Chl_N02_00416" 3200 "s59E25H2" 0 "Formate dehydrogenase subunit beta"
"Chl_N02_00416" 3200 "s59E5H2" 0 "Formate dehydrogenase subunit beta"

"Chl_N02_00416" 3200 "s63E6H2" 0 "Formate dehydrogenase subunit beta"
"Chl_N02_00417" 3201 "s59E13H2" 0 "Sulfhydrogenase 1 subunit beta"
"Chl_N02_00417" 3201 "s59E25H2" 0 "Sulfhydrogenase 1 subunit beta"
"Chl_N02_00417" 3201 "s59E5H2" 0 "Sulfhydrogenase 1 subunit beta"
"Chl_N02_00417" 3201 "s63E6H2" 0 "Sulfhydrogenase 1 subunit beta"
"Chl_N02_00418" 3202 "s59E13H2" 0 "Sulfhydrogenase 1 subunit gamma"
"Chl_N02_00418" 3202 "s59E25H2" 0 "Sulfhydrogenase 1 subunit gamma"
"Chl_N02_00418" 3202 "s59E5H2" 0 "Sulfhydrogenase 1 subunit gamma"
"Chl_N02_00418" 3202 "s63E6H2" 0 "Sulfhydrogenase 1 subunit gamma"
"Chl_N02_00419" 3203 "s59E13H2" 0 "CoB--CoM heterodisulfide reductase iron-sulfur subunit D"
"Chl_N02_00419" 3203 "s59E25H2" 0 "CoB--CoM heterodisulfide reductase iron-sulfur subunit D"
"Chl_N02_00419" 3203 "s59E5H2" 0 "CoB--CoM heterodisulfide reductase iron-sulfur subunit D"
"Chl_N02_00419" 3203 "s63E6H2" 0 "CoB--CoM heterodisulfide reductase iron-sulfur subunit D"
"Chl_N02_00422" 3206 "s59E13H2" 0 "Demethylrebeccamycin-D-glucose O-methyltransferase"
"Chl_N02_00422" 3206 "s59E25H2" 0 "Demethylrebeccamycin-D-glucose O-methyltransferase"
"Chl_N02_00422" 3206 "s59E5H2" 0 "Demethylrebeccamycin-D-glucose O-methyltransferase"
"Chl_N02_00422" 3206 "s63E6H2" 0 "Demethylrebeccamycin-D-glucose O-methyltransferase"
"Chl_N02_00423" 3207 "s59E13H2" 0 "Cyclic pyranopterin monophosphate synthase 1"
"Chl_N02_00423" 3207 "s59E25H2" 0 "Cyclic pyranopterin monophosphate synthase 1"
"Chl_N02_00423" 3207 "s59E5H2" 0 "Cyclic pyranopterin monophosphate synthase 1"
"Chl_N02_00423" 3207 "s63E6H2" 0 "Cyclic pyranopterin monophosphate synthase 1"
"Chl_N02_00424" 3208 "s59E13H2" 0 "hypothetical protein"
"Chl_N02_00424" 3208 "s59E25H2" 0 "hypothetical protein"
"Chl_N02_00424" 3208 "s59E5H2" 0 "hypothetical protein"
"Chl_N02_00424" 3208 "s63E6H2" 0 "hypothetical protein"
"Chl_N02_00425" 3209 "s59E13H2" 0 "Phenylacetate-coenzyme A ligase"
"Chl_N02_00425" 3209 "s59E25H2" 0 "Phenylacetate-coenzyme A ligase"
"Chl_N02_00425" 3209 "s59E5H2" 0 "Phenylacetate-coenzyme A ligase"
"Chl_N02_00425" 3209 "s63E6H2" 0 "Phenylacetate-coenzyme A ligase"
"Chl_N02_00426" 3210 "s59E13H2" 0 "Putative redox-active protein (C_GCAxxG_C_C)"
"Chl_N02_00426" 3210 "s59E25H2" 0 "Putative redox-active protein (C_GCAxxG_C_C)"
"Chl_N02_00426" 3210 "s59E5H2" 0.002384722 "Putative redox-active protein (C_GCAxxG_C_C)"
"Chl_N02_00426" 3210 "s63E6H2" 0 "Putative redox-active protein (C_GCAxxG_C_C)"
"Chl_N02_00428" 3212 "s59E13H2" 0 "3-oxoacyl-[acyl-carrier-protein] synthase 2"
"Chl_N02_00428" 3212 "s59E25H2" 0 "3-oxoacyl-[acyl-carrier-protein] synthase 2"
"Chl_N02_00428" 3212 "s59E5H2" 2.259287 "3-oxoacyl-[acyl-carrier-protein] synthase 2"
"Chl_N02_00428" 3212 "s63E6H2" 0 "3-oxoacyl-[acyl-carrier-protein] synthase 2"
"Chl_N02_00429" 3213 "s59E13H2" 0 "Single-stranded-DNA-specific exonuclease RecJ"
"Chl_N02_00429" 3213 "s59E25H2" 0 "Single-stranded-DNA-specific exonuclease RecJ"
"Chl_N02_00429" 3213 "s59E5H2" 1.788051 "Single-stranded-DNA-specific exonuclease RecJ"
"Chl_N02_00429" 3213 "s63E6H2" 0 "Single-stranded-DNA-specific exonuclease RecJ"
"Chl_N02_00430" 3214 "s59E13H2" 0 "Cohesin domain protein"
"Chl_N02_00430" 3214 "s59E25H2" 0 "Cohesin domain protein"
"Chl_N02_00430" 3214 "s59E5H2" 0 "Cohesin domain protein"
"Chl_N02_00430" 3214 "s63E6H2" 0 "Cohesin domain protein"
"Chl_N02_00431" 3215 "s59E13H2" 0 "hypothetical protein"
"Chl_N02_00431" 3215 "s59E25H2" 0 "hypothetical protein"

"Chl_N02_00431" 3215 "s59E5H2" 0 "hypothetical protein"
"Chl_N02_00431" 3215 "s63E6H2" 0 "hypothetical protein"
"Chl_N02_00432" 3216 "s59E13H2" 0 "Cohesin domain protein"
"Chl_N02_00432" 3216 "s59E25H2" 0 "Cohesin domain protein"
"Chl_N02_00432" 3216 "s59E5H2" 0 "Cohesin domain protein"
"Chl_N02_00432" 3216 "s63E6H2" 0 "Cohesin domain protein"
"DsuD02_00001" 3217 "s59E13H2" 0 "Alkaline phosphatase synthesis transcriptional regulatory protein PhoP"
"DsuD02_00001" 3217 "s59E25H2" 0 "Alkaline phosphatase synthesis transcriptional regulatory protein PhoP"
"DsuD02_00001" 3217 "s59E5H2" 0 "Alkaline phosphatase synthesis transcriptional regulatory protein PhoP"
"DsuD02_00001" 3217 "s63E6H2" 0 "Alkaline phosphatase synthesis transcriptional regulatory protein PhoP"
"DsuD02_00002" 3218 "s59E13H2" 0 "Type II secretion system protein E"
"DsuD02_00002" 3218 "s59E25H2" 0 "Type II secretion system protein E"
"DsuD02_00002" 3218 "s59E5H2" 0 "Type II secretion system protein E"
"DsuD02_00002" 3218 "s63E6H2" 0 "Type II secretion system protein E"
"DsuD02_00003" 3219 "s59E13H2" 0 "hypothetical protein"
"DsuD02_00003" 3219 "s59E25H2" 0 "hypothetical protein"
"DsuD02_00003" 3219 "s59E5H2" 0 "hypothetical protein"
"DsuD02_00003" 3219 "s63E6H2" 0 "hypothetical protein"
"DsuD02_00005" 3221 "s59E13H2" 0 "Sensor protein ZraS"
"DsuD02_00005" 3221 "s59E25H2" 0 "Sensor protein ZraS"
"DsuD02_00005" 3221 "s59E5H2" 0 "Sensor protein ZraS"
"DsuD02_00005" 3221 "s63E6H2" 0 "Sensor protein ZraS"
"DsuD02_00006" 3222 "s59E13H2" 0 "hypothetical protein"
"DsuD02_00006" 3222 "s59E25H2" 0 "hypothetical protein"
"DsuD02_00006" 3222 "s59E5H2" 0 "hypothetical protein"
"DsuD02_00006" 3222 "s63E6H2" 0 "hypothetical protein"
"DsuD02_00007" 3223 "s59E13H2" 0 "Sensor protein ZraS"
"DsuD02_00007" 3223 "s59E25H2" 0 "Sensor protein ZraS"
"DsuD02_00007" 3223 "s59E5H2" 0 "Sensor protein ZraS"
"DsuD02_00007" 3223 "s63E6H2" 0 "Sensor protein ZraS"
"DsuD02_00008" 3224 "s59E13H2" 0 "Response regulator PleD"
"DsuD02_00008" 3224 "s59E25H2" 0 "Response regulator PleD"
"DsuD02_00008" 3224 "s59E5H2" 0 "Response regulator PleD"
"DsuD02_00008" 3224 "s63E6H2" 0 "Response regulator PleD"
"DsuD02_00009" 3225 "s59E13H2" 0 "Methylated-DNA--protein-cysteine methyltransferase"
"DsuD02_00009" 3225 "s59E25H2" 0 "Methylated-DNA--protein-cysteine methyltransferase"
"DsuD02_00009" 3225 "s59E5H2" 0 "Methylated-DNA--protein-cysteine methyltransferase"
"DsuD02_00009" 3225 "s63E6H2" 0 "Methylated-DNA--protein-cysteine methyltransferase"
"DsuD02_00010" 3226 "s59E13H2" 0 "DNA-3-methyladenine glycosylase"
"DsuD02_00010" 3226 "s59E25H2" 0 "DNA-3-methyladenine glycosylase"
"DsuD02_00010" 3226 "s59E5H2" 0 "DNA-3-methyladenine glycosylase"
"DsuD02_00010" 3226 "s63E6H2" 0 "DNA-3-methyladenine glycosylase"
"DsuD02_00011" 3227 "s59E13H2" 0 "putative MFS-type transporter YhjX"
"DsuD02_00011" 3227 "s59E25H2" 0 "putative MFS-type transporter YhjX"
"DsuD02_00011" 3227 "s59E5H2" 0 "putative MFS-type transporter YhjX"
"DsuD02_00011" 3227 "s63E6H2" 0.00628892 "putative MFS-type transporter YhjX"
"DsuD02_00013" 3229 "s59E13H2" 0 "Transglutaminase-like superfamily protein"

"DsuD02_00013" 3229 "s59E25H2" 0 "Transglutaminase-like superfamily protein"
"DsuD02_00013" 3229 "s59E5H2" 0 "Transglutaminase-like superfamily protein"
"DsuD02_00013" 3229 "s63E6H2" 0 "Transglutaminase-like superfamily protein"
"DsuD02_00015" 3231 "s59E13H2" 0 "Group II intron-encoded protein LtrA"
"DsuD02_00015" 3231 "s59E25H2" 0 "Group II intron-encoded protein LtrA"
"DsuD02_00015" 3231 "s59E5H2" 0 "Group II intron-encoded protein LtrA"
"DsuD02_00015" 3231 "s63E6H2" 0 "Group II intron-encoded protein LtrA"
"DsuD02_00016" 3232 "s59E13H2" 0 "hypothetical protein"
"DsuD02_00016" 3232 "s59E25H2" 0 "hypothetical protein"
"DsuD02_00016" 3232 "s59E5H2" 0 "hypothetical protein"
"DsuD02_00016" 3232 "s63E6H2" 0 "hypothetical protein"
"DsuD02_00017" 3233 "s59E13H2" 0 "thiamine biosynthesis protein ThiF"
"DsuD02_00017" 3233 "s59E25H2" 0 "thiamine biosynthesis protein ThiF"
"DsuD02_00017" 3233 "s59E5H2" 0 "thiamine biosynthesis protein ThiF"
"DsuD02_00017" 3233 "s63E6H2" 0 "thiamine biosynthesis protein ThiF"
"DsuD02_00018" 3234 "s59E13H2" 0 "hypothetical protein"
"DsuD02_00018" 3234 "s59E25H2" 0 "hypothetical protein"
"DsuD02_00018" 3234 "s59E5H2" 0 "hypothetical protein"
"DsuD02_00018" 3234 "s63E6H2" 0 "hypothetical protein"
"DsuD02_00019" 3235 "s59E13H2" 0 "hypothetical protein"
"DsuD02_00019" 3235 "s59E25H2" 0 "hypothetical protein"
"DsuD02_00019" 3235 "s59E5H2" 0 "hypothetical protein"
"DsuD02_00019" 3235 "s63E6H2" 0 "hypothetical protein"
"DsuD02_00020" 3236 "s59E13H2" 0 "hypothetical protein"
"DsuD02_00020" 3236 "s59E25H2" 0 "hypothetical protein"
"DsuD02_00020" 3236 "s59E5H2" 0 "hypothetical protein"
"DsuD02_00020" 3236 "s63E6H2" 0 "hypothetical protein"
"DsuD02_00021" 3237 "s59E13H2" 0 "RNA polymerase sigma factor RpoE"
"DsuD02_00021" 3237 "s59E25H2" 0 "RNA polymerase sigma factor RpoE"
"DsuD02_00021" 3237 "s59E5H2" 0 "RNA polymerase sigma factor RpoE"
"DsuD02_00021" 3237 "s63E6H2" 0 "RNA polymerase sigma factor RpoE"
"DsuD02_00022" 3238 "s59E13H2" 0 "hypothetical protein"
"DsuD02_00022" 3238 "s59E25H2" 0 "hypothetical protein"
"DsuD02_00022" 3238 "s59E5H2" 0 "hypothetical protein"
"DsuD02_00022" 3238 "s63E6H2" 0 "hypothetical protein"
"DsuD02_00023" 3239 "s59E13H2" 0 "Methyl-accepting chemotaxis protein McpB"
"DsuD02_00023" 3239 "s59E25H2" 0 "Methyl-accepting chemotaxis protein McpB"
"DsuD02_00023" 3239 "s59E5H2" 0 "Methyl-accepting chemotaxis protein McpB"
"DsuD02_00023" 3239 "s63E6H2" 0 "Methyl-accepting chemotaxis protein McpB"
"DsuD02_00024" 3240 "s59E13H2" 0 "Alkaline phosphatase synthesis transcriptional regulatory protein PhoP"
"DsuD02_00024" 3240 "s59E25H2" 0 "Alkaline phosphatase synthesis transcriptional regulatory protein PhoP"
"DsuD02_00024" 3240 "s59E5H2" 0 "Alkaline phosphatase synthesis transcriptional regulatory protein PhoP"
"DsuD02_00024" 3240 "s63E6H2" 0 "Alkaline phosphatase synthesis transcriptional regulatory protein PhoP"
"DsuD02_00025" 3241 "s59E13H2" 0 "Putative type II secretion system protein E"
"DsuD02_00025" 3241 "s59E25H2" 0 "Putative type II secretion system protein E"
"DsuD02_00025" 3241 "s59E5H2" 0 "Putative type II secretion system protein E"
"DsuD02_00025" 3241 "s63E6H2" 0 "Putative type II secretion system protein E"

"DsuD02_00026" 3242 "s59E13H2" 0 "Mutual gliding-motility protein MglA"
"DsuD02_00026" 3242 "s59E25H2" 0 "Mutual gliding-motility protein MglA"
"DsuD02_00026" 3242 "s59E5H2" 0 "Mutual gliding-motility protein MglA"
"DsuD02_00026" 3242 "s63E6H2" 0 "Mutual gliding-motility protein MglA"
"DsuD02_00027" 3243 "s59E13H2" 0 "Cyclic di-GMP phosphodiesterase response regulator RpfG"
"DsuD02_00027" 3243 "s59E25H2" 0 "Cyclic di-GMP phosphodiesterase response regulator RpfG"
"DsuD02_00027" 3243 "s59E5H2" 0 "Cyclic di-GMP phosphodiesterase response regulator RpfG"
"DsuD02_00027" 3243 "s63E6H2" 0 "Cyclic di-GMP phosphodiesterase response regulator RpfG"
"DsuD02_00028" 3244 "s59E13H2" 0 "Cyclic di-GMP phosphodiesterase response regulator RpfG"
"DsuD02_00028" 3244 "s59E25H2" 0 "Cyclic di-GMP phosphodiesterase response regulator RpfG"
"DsuD02_00028" 3244 "s59E5H2" 0 "Cyclic di-GMP phosphodiesterase response regulator RpfG"
"DsuD02_00028" 3244 "s63E6H2" 0 "Cyclic di-GMP phosphodiesterase response regulator RpfG"
"DsuD02_00029" 3245 "s59E13H2" 0 "hypothetical protein"
"DsuD02_00029" 3245 "s59E25H2" 0 "hypothetical protein"
"DsuD02_00029" 3245 "s59E5H2" 0 "hypothetical protein"
"DsuD02_00029" 3245 "s63E6H2" 0 "hypothetical protein"
"DsuD02_00030" 3246 "s59E13H2" 0 "Phosphate regulon transcriptional regulatory protein PhoB"
"DsuD02_00030" 3246 "s59E25H2" 0 "Phosphate regulon transcriptional regulatory protein PhoB"
"DsuD02_00030" 3246 "s59E5H2" 0 "Phosphate regulon transcriptional regulatory protein PhoB"
"DsuD02_00030" 3246 "s63E6H2" 0 "Phosphate regulon transcriptional regulatory protein PhoB"
"DsuD02_00031" 3247 "s59E13H2" 0 "hypothetical protein"
"DsuD02_00031" 3247 "s59E25H2" 0 "hypothetical protein"
"DsuD02_00031" 3247 "s59E5H2" 0 "hypothetical protein"
"DsuD02_00031" 3247 "s63E6H2" 0 "hypothetical protein"
"DsuD02_00032" 3248 "s59E13H2" 0 "Integrase core domain protein"
"DsuD02_00032" 3248 "s59E25H2" 0 "Integrase core domain protein"
"DsuD02_00032" 3248 "s59E5H2" 0 "Integrase core domain protein"
"DsuD02_00032" 3248 "s63E6H2" 0 "Integrase core domain protein"
"DsuD02_00034" 3250 "s59E13H2" 0 "30S ribosomal protein S1"
"DsuD02_00034" 3250 "s59E25H2" 0 "30S ribosomal protein S1"
"DsuD02_00034" 3250 "s59E5H2" 0 "30S ribosomal protein S1"
"DsuD02_00034" 3250 "s63E6H2" 0 "30S ribosomal protein S1"
"DsuD02_00035" 3251 "s59E13H2" 0 "Putative signal peptide peptidase SppA"
"DsuD02_00035" 3251 "s59E25H2" 0 "Putative signal peptide peptidase SppA"
"DsuD02_00035" 3251 "s59E5H2" 0 "Putative signal peptide peptidase SppA"
"DsuD02_00035" 3251 "s63E6H2" 0 "Putative signal peptide peptidase SppA"
"DsuD02_00036" 3252 "s59E13H2" 0 "Threonylcarbamoyl-AMP synthase"
"DsuD02_00036" 3252 "s59E25H2" 0 "Threonylcarbamoyl-AMP synthase"
"DsuD02_00036" 3252 "s59E5H2" 0 "Threonylcarbamoyl-AMP synthase"
"DsuD02_00036" 3252 "s63E6H2" 0 "Threonylcarbamoyl-AMP synthase"
"DsuD02_00037" 3253 "s59E13H2" 0 "Type 4 prepilin-like proteins leader peptide-processing enzyme"
"DsuD02_00037" 3253 "s59E25H2" 0 "Type 4 prepilin-like proteins leader peptide-processing enzyme"
"DsuD02_00037" 3253 "s59E5H2" 0 "Type 4 prepilin-like proteins leader peptide-processing enzyme"
"DsuD02_00037" 3253 "s63E6H2" 0 "Type 4 prepilin-like proteins leader peptide-processing enzyme"
"DsuD02_00038" 3254 "s59E13H2" 0 "Sensor protein ZraS"
"DsuD02_00038" 3254 "s59E25H2" 0 "Sensor protein ZraS"
"DsuD02_00038" 3254 "s59E5H2" 0 "Sensor protein ZraS"

"DsuD02_00038" 3254 "s63E6H2" 0 "Sensor protein ZraS"
"DsuD02_00039" 3255 "s59E13H2" 0 "Transcriptional regulatory protein ZraR"
"DsuD02_00039" 3255 "s59E25H2" 0 "Transcriptional regulatory protein ZraR"
"DsuD02_00039" 3255 "s59E5H2" 0 "Transcriptional regulatory protein ZraR"
"DsuD02_00039" 3255 "s63E6H2" 0 "Transcriptional regulatory protein ZraR"
"DsuD02_00040" 3256 "s59E13H2" 0 "hypothetical protein"
"DsuD02_00040" 3256 "s59E25H2" 0 "hypothetical protein"
"DsuD02_00040" 3256 "s59E5H2" 0 "hypothetical protein"
"DsuD02_00040" 3256 "s63E6H2" 0 "hypothetical protein"
"DsuD02_00041" 3257 "s59E13H2" 0 "hypothetical protein"
"DsuD02_00041" 3257 "s59E25H2" 0 "hypothetical protein"
"DsuD02_00041" 3257 "s59E5H2" 0 "hypothetical protein"
"DsuD02_00041" 3257 "s63E6H2" 0 "hypothetical protein"
"DsuD02_00042" 3258 "s59E13H2" 0 "hypothetical protein"
"DsuD02_00042" 3258 "s59E25H2" 0 "hypothetical protein"
"DsuD02_00042" 3258 "s59E5H2" 0 "hypothetical protein"
"DsuD02_00042" 3258 "s63E6H2" 0 "hypothetical protein"
"DsuD02_00043" 3259 "s59E13H2" 0 "hypothetical protein"
"DsuD02_00043" 3259 "s59E25H2" 0 "hypothetical protein"
"DsuD02_00043" 3259 "s59E5H2" 0 "hypothetical protein"
"DsuD02_00043" 3259 "s63E6H2" 0 "hypothetical protein"
"DsuD02_00044" 3260 "s59E13H2" 0 "Neisseria PilC protein"
"DsuD02_00044" 3260 "s59E25H2" 0 "Neisseria PilC protein"
"DsuD02_00044" 3260 "s59E5H2" 0 "Neisseria PilC protein"
"DsuD02_00044" 3260 "s63E6H2" 0 "Neisseria PilC protein"
"DsuD02_00045" 3261 "s59E13H2" 0 "Fimbrial protein precursor"
"DsuD02_00045" 3261 "s59E25H2" 0 "Fimbrial protein precursor"
"DsuD02_00045" 3261 "s59E5H2" 0 "Fimbrial protein precursor"
"DsuD02_00045" 3261 "s63E6H2" 0 "Fimbrial protein precursor"
"DsuD02_00046" 3262 "s59E13H2" 0 "hypothetical protein"
"DsuD02_00046" 3262 "s59E25H2" 0 "hypothetical protein"
"DsuD02_00046" 3262 "s59E5H2" 0 "hypothetical protein"
"DsuD02_00046" 3262 "s63E6H2" 0 "hypothetical protein"
"DsuD02_00047" 3263 "s59E13H2" 0 "Type II secretion system protein G precursor"
"DsuD02_00047" 3263 "s59E25H2" 0 "Type II secretion system protein G precursor"
"DsuD02_00047" 3263 "s59E5H2" 0 "Type II secretion system protein G precursor"
"DsuD02_00047" 3263 "s63E6H2" 0 "Type II secretion system protein G precursor"
"DsuD02_00049" 3265 "s59E13H2" 0 "MORN repeat protein"
"DsuD02_00049" 3265 "s59E25H2" 0 "MORN repeat protein"
"DsuD02_00049" 3265 "s59E5H2" 0 "MORN repeat protein"
"DsuD02_00049" 3265 "s63E6H2" 0 "MORN repeat protein"
"DsuD02_00050" 3266 "s59E13H2" 0 "Serine/threonine-protein phosphatase 1"
"DsuD02_00050" 3266 "s59E25H2" 0 "Serine/threonine-protein phosphatase 1"
"DsuD02_00050" 3266 "s59E5H2" 0 "Serine/threonine-protein phosphatase 1"
"DsuD02_00050" 3266 "s63E6H2" 0 "Serine/threonine-protein phosphatase 1"
"DsuD02_00051" 3267 "s59E13H2" 0 "hypothetical protein"
"DsuD02_00051" 3267 "s59E25H2" 0 "hypothetical protein"

"DsuD02_00051" 3267 "s59E5H2" 0 "hypothetical protein"
"DsuD02_00051" 3267 "s63E6H2" 0 "hypothetical protein"
"DsuD02_00052" 3268 "s59E13H2" 0 "hypothetical protein"
"DsuD02_00052" 3268 "s59E25H2" 0 "hypothetical protein"
"DsuD02_00052" 3268 "s59E5H2" 0 "hypothetical protein"
"DsuD02_00052" 3268 "s63E6H2" 0 "hypothetical protein"
"DsuD02_00053" 3269 "s59E13H2" 0 "Fimbrial protein precursor"
"DsuD02_00053" 3269 "s59E25H2" 0 "Fimbrial protein precursor"
"DsuD02_00053" 3269 "s59E5H2" 0 "Fimbrial protein precursor"
"DsuD02_00053" 3269 "s63E6H2" 0 "Fimbrial protein precursor"
"DsuD02_00054" 3270 "s59E13H2" 0 "PilZ domain protein"
"DsuD02_00054" 3270 "s59E25H2" 0 "PilZ domain protein"
"DsuD02_00054" 3270 "s59E5H2" 0 "PilZ domain protein"
"DsuD02_00054" 3270 "s63E6H2" 0 "PilZ domain protein"
"DsuD02_00055" 3271 "s59E13H2" 0 "hypothetical protein"
"DsuD02_00055" 3271 "s59E25H2" 0 "hypothetical protein"
"DsuD02_00055" 3271 "s59E5H2" 0 "hypothetical protein"
"DsuD02_00055" 3271 "s63E6H2" 0 "hypothetical protein"
"DsuD02_00056" 3272 "s59E13H2" 0 "hypothetical protein"
"DsuD02_00056" 3272 "s59E25H2" 0 "hypothetical protein"
"DsuD02_00056" 3272 "s59E5H2" 0 "hypothetical protein"
"DsuD02_00056" 3272 "s63E6H2" 0 "hypothetical protein"
"DsuD02_00057" 3273 "s59E13H2" 0 "hypothetical protein"
"DsuD02_00057" 3273 "s59E25H2" 0 "hypothetical protein"
"DsuD02_00057" 3273 "s59E5H2" 0 "hypothetical protein"
"DsuD02_00057" 3273 "s63E6H2" 0 "hypothetical protein"
"DsuD02_00058" 3274 "s59E13H2" 0 "Methionine--tRNA ligase"
"DsuD02_00058" 3274 "s59E25H2" 0 "Methionine--tRNA ligase"
"DsuD02_00058" 3274 "s59E5H2" 0 "Methionine--tRNA ligase"
"DsuD02_00058" 3274 "s63E6H2" 0 "Methionine--tRNA ligase"
"DsuD02_00059" 3275 "s59E13H2" 0 "hypothetical protein"
"DsuD02_00059" 3275 "s59E25H2" 0 "hypothetical protein"
"DsuD02_00059" 3275 "s59E5H2" 0 "hypothetical protein"
"DsuD02_00059" 3275 "s63E6H2" 0 "hypothetical protein"
"DsuD02_00060" 3276 "s59E13H2" 0 "DNA polymerase III subunit tau"
"DsuD02_00060" 3276 "s59E25H2" 0 "DNA polymerase III subunit tau"
"DsuD02_00060" 3276 "s59E5H2" 0 "DNA polymerase III subunit tau"
"DsuD02_00060" 3276 "s63E6H2" 0 "DNA polymerase III subunit tau"
"DsuD02_00061" 3277 "s59E13H2" 0 "Thymidylate kinase"
"DsuD02_00061" 3277 "s59E25H2" 0 "Thymidylate kinase"
"DsuD02_00061" 3277 "s59E5H2" 0 "Thymidylate kinase"
"DsuD02_00061" 3277 "s63E6H2" 0 "Thymidylate kinase"
"DsuD02_00062" 3278 "s59E13H2" 0 "Thiamine-monophosphate kinase"
"DsuD02_00062" 3278 "s59E25H2" 0 "Thiamine-monophosphate kinase"
"DsuD02_00062" 3278 "s59E5H2" 0 "Thiamine-monophosphate kinase"
"DsuD02_00062" 3278 "s63E6H2" 0 "Thiamine-monophosphate kinase"
"DsuD02_00063" 3279 "s59E13H2" 0 "Methylated-DNA--protein-cysteine methyltransferase"

"DsuD02_00063" 3279 "s59E25H2" 0 "Methylated-DNA--protein-cysteine methyltransferase"
"DsuD02_00063" 3279 "s59E5H2" 0 "Methylated-DNA--protein-cysteine methyltransferase"
"DsuD02_00063" 3279 "s63E6H2" 0 "Methylated-DNA--protein-cysteine methyltransferase"
"DsuD02_00064" 3280 "s59E13H2" 0 "hypothetical protein"
"DsuD02_00064" 3280 "s59E25H2" 0 "hypothetical protein"
"DsuD02_00064" 3280 "s59E5H2" 0 "hypothetical protein"
"DsuD02_00064" 3280 "s63E6H2" 0 "hypothetical protein"
"DsuD02_00065" 3281 "s59E13H2" 0 "Acetyl-CoA decarbonylase/synthase complex subunit gamma"
"DsuD02_00065" 3281 "s59E25H2" 0 "Acetyl-CoA decarbonylase/synthase complex subunit gamma"
"DsuD02_00065" 3281 "s59E5H2" 0 "Acetyl-CoA decarbonylase/synthase complex subunit gamma"
"DsuD02_00065" 3281 "s63E6H2" 0 "Acetyl-CoA decarbonylase/synthase complex subunit gamma"
"DsuD02_00066" 3282 "s59E13H2" 0 "hypothetical protein"
"DsuD02_00066" 3282 "s59E25H2" 0 "hypothetical protein"
"DsuD02_00066" 3282 "s59E5H2" 0 "hypothetical protein"
"DsuD02_00066" 3282 "s63E6H2" 0 "hypothetical protein"
"DsuD02_00067" 3283 "s59E13H2" 0 "Transposase IS116/IS110/IS902 family protein"
"DsuD02_00067" 3283 "s59E25H2" 0 "Transposase IS116/IS110/IS902 family protein"
"DsuD02_00067" 3283 "s59E5H2" 0 "Transposase IS116/IS110/IS902 family protein"
"DsuD02_00067" 3283 "s63E6H2" 0 "Transposase IS116/IS110/IS902 family protein"
"DsuD02_00069" 3285 "s59E13H2" 0 "hypothetical protein"
"DsuD02_00069" 3285 "s59E25H2" 0 "hypothetical protein"
"DsuD02_00069" 3285 "s59E5H2" 0 "hypothetical protein"
"DsuD02_00069" 3285 "s63E6H2" 0.07548018 "hypothetical protein"
"DsuD02_00070" 3286 "s59E13H2" 0 "hypothetical protein"
"DsuD02_00070" 3286 "s59E25H2" 0 "hypothetical protein"
"DsuD02_00070" 3286 "s59E5H2" 0 "hypothetical protein"
"DsuD02_00070" 3286 "s63E6H2" 0 "hypothetical protein"
"DsuD02_00071" 3287 "s59E13H2" 0 "ATP-dependent DNA helicase RecQ"
"DsuD02_00071" 3287 "s59E25H2" 0 "ATP-dependent DNA helicase RecQ"
"DsuD02_00071" 3287 "s59E5H2" 0 "ATP-dependent DNA helicase RecQ"
"DsuD02_00071" 3287 "s63E6H2" 0 "ATP-dependent DNA helicase RecQ"
"DsuD02_00073" 3289 "s59E13H2" 0 "2-aminoadipate transaminase"
"DsuD02_00073" 3289 "s59E25H2" 0 "2-aminoadipate transaminase"
"DsuD02_00073" 3289 "s59E5H2" 0 "2-aminoadipate transaminase"
"DsuD02_00073" 3289 "s63E6H2" 0.006268423 "2-aminoadipate transaminase"
"DsuD02_00074" 3290 "s59E13H2" 0 "ATP-dependent DNA helicase RecQ"
"DsuD02_00074" 3290 "s59E25H2" 0 "ATP-dependent DNA helicase RecQ"
"DsuD02_00074" 3290 "s59E5H2" 0 "ATP-dependent DNA helicase RecQ"
"DsuD02_00074" 3290 "s63E6H2" 0 "ATP-dependent DNA helicase RecQ"
"DsuD02_00075" 3291 "s59E13H2" 0 "hypothetical protein"
"DsuD02_00075" 3291 "s59E25H2" 0 "hypothetical protein"
"DsuD02_00075" 3291 "s59E5H2" 0 "hypothetical protein"
"DsuD02_00075" 3291 "s63E6H2" 0 "hypothetical protein"
"DsuD02_00078" 3294 "s59E13H2" 0 "10 kDa chaperonin 1"
"DsuD02_00078" 3294 "s59E25H2" 0 "10 kDa chaperonin 1"
"DsuD02_00078" 3294 "s59E5H2" 0 "10 kDa chaperonin 1"
"DsuD02_00078" 3294 "s63E6H2" 0 "10 kDa chaperonin 1"

"DsuD02_00079" 3295 "s59E13H2" 0 "Transcriptional regulatory protein ZraR"
"DsuD02_00079" 3295 "s59E25H2" 0 "Transcriptional regulatory protein ZraR"
"DsuD02_00079" 3295 "s59E5H2" 0 "Transcriptional regulatory protein ZraR"
"DsuD02_00079" 3295 "s63E6H2" 0 "Transcriptional regulatory protein ZraR"
"DsuD02_00082" 3298 "s59E13H2" 0 "L-lysine 2,3-aminomutase"
"DsuD02_00082" 3298 "s59E25H2" 0 "L-lysine 2,3-aminomutase"
"DsuD02_00082" 3298 "s59E5H2" 0 "L-lysine 2,3-aminomutase"
"DsuD02_00082" 3298 "s63E6H2" 0 "L-lysine 2,3-aminomutase"
"DsuD02_00083" 3299 "s59E13H2" 0 "D-alanine--D-alanine ligase"
"DsuD02_00083" 3299 "s59E25H2" 0 "D-alanine--D-alanine ligase"
"DsuD02_00083" 3299 "s59E5H2" 0 "D-alanine--D-alanine ligase"
"DsuD02_00083" 3299 "s63E6H2" 0 "D-alanine--D-alanine ligase"
"DsuD02_00084" 3300 "s59E13H2" 0 "D-alanine--D-alanine ligase"
"DsuD02_00084" 3300 "s59E25H2" 0 "D-alanine--D-alanine ligase"
"DsuD02_00084" 3300 "s59E5H2" 0 "D-alanine--D-alanine ligase"
"DsuD02_00084" 3300 "s63E6H2" 0 "D-alanine--D-alanine ligase"
"DsuD02_00085" 3301 "s59E13H2" 0 "Acetyltransferase (GNAT) family protein"
"DsuD02_00085" 3301 "s59E25H2" 0 "Acetyltransferase (GNAT) family protein"
"DsuD02_00085" 3301 "s59E5H2" 0 "Acetyltransferase (GNAT) family protein"
"DsuD02_00085" 3301 "s63E6H2" 0 "Acetyltransferase (GNAT) family protein"
"DsuD02_00086" 3302 "s59E13H2" 0 "hypothetical protein"
"DsuD02_00086" 3302 "s59E25H2" 0 "hypothetical protein"
"DsuD02_00086" 3302 "s59E5H2" 0 "hypothetical protein"
"DsuD02_00086" 3302 "s63E6H2" 0 "hypothetical protein"
"DsuD02_00087" 3303 "s59E13H2" 0 "Diacylglycerol kinase"
"DsuD02_00087" 3303 "s59E25H2" 0 "Diacylglycerol kinase"
"DsuD02_00087" 3303 "s59E5H2" 0 "Diacylglycerol kinase"
"DsuD02_00087" 3303 "s63E6H2" 0 "Diacylglycerol kinase"
"DsuD02_00088" 3304 "s59E13H2" 0 "hypothetical protein"
"DsuD02_00088" 3304 "s59E25H2" 0 "hypothetical protein"
"DsuD02_00088" 3304 "s59E5H2" 0 "hypothetical protein"
"DsuD02_00088" 3304 "s63E6H2" 0 "hypothetical protein"
"DsuD02_00089" 3305 "s59E13H2" 0 "Inner membrane ABC transporter permease protein YcjP"
"DsuD02_00089" 3305 "s59E25H2" 0 "Inner membrane ABC transporter permease protein YcjP"
"DsuD02_00089" 3305 "s59E5H2" 0 "Inner membrane ABC transporter permease protein YcjP"
"DsuD02_00089" 3305 "s63E6H2" 0 "Inner membrane ABC transporter permease protein YcjP"
"DsuD02_00090" 3306 "s59E13H2" 0 "Inner membrane ABC transporter permease protein YcjO"
"DsuD02_00090" 3306 "s59E25H2" 0 "Inner membrane ABC transporter permease protein YcjO"
"DsuD02_00090" 3306 "s59E5H2" 0 "Inner membrane ABC transporter permease protein YcjO"
"DsuD02_00090" 3306 "s63E6H2" 0 "Inner membrane ABC transporter permease protein YcjO"
"DsuD02_00091" 3307 "s59E13H2" 0 "Bacterial extracellular solute-binding protein"
"DsuD02_00091" 3307 "s59E25H2" 0 "Bacterial extracellular solute-binding protein"
"DsuD02_00091" 3307 "s59E5H2" 0 "Bacterial extracellular solute-binding protein"
"DsuD02_00091" 3307 "s63E6H2" 0 "Bacterial extracellular solute-binding protein"
"DsuD02_00092" 3308 "s59E13H2" 0 "NAD-dependent protein deacylase 2"
"DsuD02_00092" 3308 "s59E25H2" 0 "NAD-dependent protein deacylase 2"
"DsuD02_00092" 3308 "s59E5H2" 0 "NAD-dependent protein deacylase 2"

"DsuD02_00092" 3308 "s63E6H2" 0 "NAD-dependent protein deacylase 2"
"DsuD02_00093" 3309 "s59E13H2" 0 "putative nicotinate-nucleotide pyrophosphorylase [carboxylating]"
"DsuD02_00093" 3309 "s59E25H2" 0 "putative nicotinate-nucleotide pyrophosphorylase [carboxylating]"
"DsuD02_00093" 3309 "s59E5H2" 0 "putative nicotinate-nucleotide pyrophosphorylase [carboxylating]"
"DsuD02_00093" 3309 "s63E6H2" 0 "putative nicotinate-nucleotide pyrophosphorylase [carboxylating]"
"DsuD02_00094" 3310 "s59E13H2" 0 "Valine--tRNA ligase"
"DsuD02_00094" 3310 "s59E25H2" 0 "Valine--tRNA ligase"
"DsuD02_00094" 3310 "s59E5H2" 0 "Valine--tRNA ligase"
"DsuD02_00094" 3310 "s63E6H2" 0 "Valine--tRNA ligase"
"DsuD02_00095" 3311 "s59E13H2" 0 "hypothetical protein"
"DsuD02_00095" 3311 "s59E25H2" 0 "hypothetical protein"
"DsuD02_00095" 3311 "s59E5H2" 0 "hypothetical protein"
"DsuD02_00095" 3311 "s63E6H2" 0 "hypothetical protein"
"DsuD02_00096" 3312 "s59E13H2" 0 "putative acyl-CoA dehydrogenase"
"DsuD02_00096" 3312 "s59E25H2" 0 "putative acyl-CoA dehydrogenase"
"DsuD02_00096" 3312 "s59E5H2" 0 "putative acyl-CoA dehydrogenase"
"DsuD02_00096" 3312 "s63E6H2" 0 "putative acyl-CoA dehydrogenase"
"DsuD02_00097" 3313 "s59E13H2" 0 "Archaeal Lon protease"
"DsuD02_00097" 3313 "s59E25H2" 0 "Archaeal Lon protease"
"DsuD02_00097" 3313 "s59E5H2" 0 "Archaeal Lon protease"
"DsuD02_00097" 3313 "s63E6H2" 0 "Archaeal Lon protease"
"DsuD02_00099" 3315 "s59E13H2" 0 "hypothetical protein"
"DsuD02_00099" 3315 "s59E25H2" 0 "hypothetical protein"
"DsuD02_00099" 3315 "s59E5H2" 0 "hypothetical protein"
"DsuD02_00099" 3315 "s63E6H2" 0 "hypothetical protein"
"DsuD02_00100" 3316 "s59E13H2" 0 "Ribosomal RNA small subunit methyltransferase E"
"DsuD02_00100" 3316 "s59E25H2" 0 "Ribosomal RNA small subunit methyltransferase E"
"DsuD02_00100" 3316 "s59E5H2" 0 "Ribosomal RNA small subunit methyltransferase E"
"DsuD02_00100" 3316 "s63E6H2" 0.002190103 "Ribosomal RNA small subunit methyltransferase E"
"DsuD02_00107" 3323 "s59E13H2" 0 "Corrinoid/iron-sulfur protein small subunit"
"DsuD02_00107" 3323 "s59E25H2" 0 "Corrinoid/iron-sulfur protein small subunit"
"DsuD02_00107" 3323 "s59E5H2" 0 "Corrinoid/iron-sulfur protein small subunit"
"DsuD02_00107" 3323 "s63E6H2" 0 "Corrinoid/iron-sulfur protein small subunit"
"DsuD02_00108" 3324 "s59E13H2" 0 "Carbon monoxide dehydrogenase 1"
"DsuD02_00108" 3324 "s59E25H2" 0 "Carbon monoxide dehydrogenase 1"
"DsuD02_00108" 3324 "s59E5H2" 0 "Carbon monoxide dehydrogenase 1"
"DsuD02_00108" 3324 "s63E6H2" 0 "Carbon monoxide dehydrogenase 1"
"DsuD02_00109" 3325 "s59E13H2" 0 "Carbon monoxide dehydrogenase/acetyl-CoA synthase subunit alpha"
"DsuD02_00109" 3325 "s59E25H2" 0 "Carbon monoxide dehydrogenase/acetyl-CoA synthase subunit alpha"
"DsuD02_00109" 3325 "s59E5H2" 0 "Carbon monoxide dehydrogenase/acetyl-CoA synthase subunit alpha"
"DsuD02_00109" 3325 "s63E6H2" 0 "Carbon monoxide dehydrogenase/acetyl-CoA synthase subunit alpha"
"DsuD02_00110" 3326 "s59E13H2" 0 "Corrinoid/iron-sulfur protein large subunit"
"DsuD02_00110" 3326 "s59E25H2" 0 "Corrinoid/iron-sulfur protein large subunit"
"DsuD02_00110" 3326 "s59E5H2" 0 "Corrinoid/iron-sulfur protein large subunit"
"DsuD02_00110" 3326 "s63E6H2" 0 "Corrinoid/iron-sulfur protein large subunit"
"DsuD02_00111" 3327 "s59E13H2" 0 "5-methyltetrahydrofolate:corrinoid/iron-sulfur protein co-methyltransferase"
"DsuD02_00111" 3327 "s59E25H2" 0 "5-methyltetrahydrofolate:corrinoid/iron-sulfur protein co-methyltransferase"

"DsuD02_00111" 3327 "s59E5H2" 0 "5-methyltetrahydrofolate:corrinoid/iron-sulfur protein co-methyltransferase"
"DsuD02_00111" 3327 "s63E6H2" 0 "5-methyltetrahydrofolate:corrinoid/iron-sulfur protein co-methyltransferase"
"DsuD02_00112" 3328 "s59E13H2" 0 "ComEC family competence protein"
"DsuD02_00112" 3328 "s59E25H2" 0 "ComEC family competence protein"
"DsuD02_00112" 3328 "s59E5H2" 0 "ComEC family competence protein"
"DsuD02_00112" 3328 "s63E6H2" 0 "ComEC family competence protein"
"DsuD02_00113" 3329 "s59E13H2" 0 "UDP-N-acetylglucosamine 1-carboxyvinyltransferase"
"DsuD02_00113" 3329 "s59E25H2" 0 "UDP-N-acetylglucosamine 1-carboxyvinyltransferase"
"DsuD02_00113" 3329 "s59E5H2" 0 "UDP-N-acetylglucosamine 1-carboxyvinyltransferase"
"DsuD02_00113" 3329 "s63E6H2" 0 "UDP-N-acetylglucosamine 1-carboxyvinyltransferase"
"DsuD02_00114" 3330 "s59E13H2" 0 "Release factor glutamine methyltransferase"
"DsuD02_00114" 3330 "s59E25H2" 0 "Release factor glutamine methyltransferase"
"DsuD02_00114" 3330 "s59E5H2" 0 "Release factor glutamine methyltransferase"
"DsuD02_00114" 3330 "s63E6H2" 0 "Release factor glutamine methyltransferase"
"DsuD02_00115" 3331 "s59E13H2" 0 "Putative redox-active protein (C_GCAxxG_C_C)"
"DsuD02_00115" 3331 "s59E25H2" 0 "Putative redox-active protein (C_GCAxxG_C_C)"
"DsuD02_00115" 3331 "s59E5H2" 0 "Putative redox-active protein (C_GCAxxG_C_C)"
"DsuD02_00115" 3331 "s63E6H2" 0 "Putative redox-active protein (C_GCAxxG_C_C)"
"DsuD02_00116" 3332 "s59E13H2" 0 "GMP synthase [glutamine-hydrolyzing] subunit B"
"DsuD02_00116" 3332 "s59E25H2" 0 "GMP synthase [glutamine-hydrolyzing] subunit B"
"DsuD02_00116" 3332 "s59E5H2" 0 "GMP synthase [glutamine-hydrolyzing] subunit B"
"DsuD02_00116" 3332 "s63E6H2" 0 "GMP synthase [glutamine-hydrolyzing] subunit B"
"DsuD02_00117" 3333 "s59E13H2" 0 "Zinc ribbon domain protein"
"DsuD02_00117" 3333 "s59E25H2" 0 "Zinc ribbon domain protein"
"DsuD02_00117" 3333 "s59E5H2" 0 "Zinc ribbon domain protein"
"DsuD02_00117" 3333 "s63E6H2" 0 "Zinc ribbon domain protein"
"DsuD02_00119" 3335 "s59E13H2" 0 "4-chlorobenzoyl coenzyme A dehalogenase-2"
"DsuD02_00119" 3335 "s59E25H2" 0 "4-chlorobenzoyl coenzyme A dehalogenase-2"
"DsuD02_00119" 3335 "s59E5H2" 0 "4-chlorobenzoyl coenzyme A dehalogenase-2"
"DsuD02_00119" 3335 "s63E6H2" 0 "4-chlorobenzoyl coenzyme A dehalogenase-2"
"DsuD02_00120" 3336 "s59E13H2" 0 "Queuine tRNA-ribosyltransferase"
"DsuD02_00120" 3336 "s59E25H2" 0 "Queuine tRNA-ribosyltransferase"
"DsuD02_00120" 3336 "s59E5H2" 0 "Queuine tRNA-ribosyltransferase"
"DsuD02_00120" 3336 "s63E6H2" 0 "Queuine tRNA-ribosyltransferase"
"DsuD02_00121" 3337 "s59E13H2" 0 "hypothetical protein"
"DsuD02_00121" 3337 "s59E25H2" 0 "hypothetical protein"
"DsuD02_00121" 3337 "s59E5H2" 0 "hypothetical protein"
"DsuD02_00121" 3337 "s63E6H2" 0 "hypothetical protein"
"DsuD02_00122" 3338 "s59E13H2" 0 "Methylmalonyl-CoA mutase"
"DsuD02_00122" 3338 "s59E25H2" 0 "Methylmalonyl-CoA mutase"
"DsuD02_00122" 3338 "s59E5H2" 0 "Methylmalonyl-CoA mutase"
"DsuD02_00122" 3338 "s63E6H2" 0 "Methylmalonyl-CoA mutase"
"DsuD02_00123" 3339 "s59E13H2" 0 "Methylmalonyl-CoA mutase large subunit"
"DsuD02_00123" 3339 "s59E25H2" 0 "Methylmalonyl-CoA mutase large subunit"
"DsuD02_00123" 3339 "s59E5H2" 0 "Methylmalonyl-CoA mutase large subunit"
"DsuD02_00123" 3339 "s63E6H2" 0 "Methylmalonyl-CoA mutase large subunit"
"DsuD02_00124" 3340 "s59E13H2" 0 "Acyl-CoA dehydrogenase"

"DsuD02_00124" 3340 "s59E25H2" 0 "Acyl-CoA dehydrogenase"
"DsuD02_00124" 3340 "s59E5H2" 0 "Acyl-CoA dehydrogenase"
"DsuD02_00124" 3340 "s63E6H2" 0 "Acyl-CoA dehydrogenase"
"DsuD02_00125" 3341 "s59E13H2" 0 "Long-chain-fatty-acid--CoA ligase"
"DsuD02_00125" 3341 "s59E25H2" 0 "Long-chain-fatty-acid--CoA ligase"
"DsuD02_00125" 3341 "s59E5H2" 0 "Long-chain-fatty-acid--CoA ligase"
"DsuD02_00125" 3341 "s63E6H2" 0 "Long-chain-fatty-acid--CoA ligase"
"DsuD02_00126" 3342 "s59E13H2" 0 "Long-chain-fatty-acid--CoA ligase FadD13"
"DsuD02_00126" 3342 "s59E25H2" 0 "Long-chain-fatty-acid--CoA ligase FadD13"
"DsuD02_00126" 3342 "s59E5H2" 0 "Long-chain-fatty-acid--CoA ligase FadD13"
"DsuD02_00126" 3342 "s63E6H2" 0 "Long-chain-fatty-acid--CoA ligase FadD13"
"DsuD02_00127" 3343 "s59E13H2" 0 "2-enoate reductase FldZ"
"DsuD02_00127" 3343 "s59E25H2" 0 "2-enoate reductase FldZ"
"DsuD02_00127" 3343 "s59E5H2" 0 "2-enoate reductase FldZ"
"DsuD02_00127" 3343 "s63E6H2" 0 "2-enoate reductase FldZ"
"DsuD02_00128" 3344 "s59E13H2" 0 "hypothetical protein"
"DsuD02_00128" 3344 "s59E25H2" 0 "hypothetical protein"
"DsuD02_00128" 3344 "s59E5H2" 0 "hypothetical protein"
"DsuD02_00128" 3344 "s63E6H2" 0 "hypothetical protein"
"DsuD02_00129" 3345 "s59E13H2" 0 "hypothetical protein"
"DsuD02_00129" 3345 "s59E25H2" 0 "hypothetical protein"
"DsuD02_00129" 3345 "s59E5H2" 0 "hypothetical protein"
"DsuD02_00129" 3345 "s63E6H2" 0 "hypothetical protein"
"DsuD02_00132" 3348 "s59E13H2" 0 "hypothetical protein"
"DsuD02_00132" 3348 "s59E25H2" 0 "hypothetical protein"
"DsuD02_00132" 3348 "s59E5H2" 0 "hypothetical protein"
"DsuD02_00132" 3348 "s63E6H2" 0 "hypothetical protein"
"DsuD02_00135" 3351 "s59E13H2" 0 "PHP domain protein"
"DsuD02_00135" 3351 "s59E25H2" 0 "PHP domain protein"
"DsuD02_00135" 3351 "s59E5H2" 0 "PHP domain protein"
"DsuD02_00135" 3351 "s63E6H2" 0 "PHP domain protein"
"DsuD02_00138" 3354 "s59E13H2" 0.002955553 "putative BsuMI modification methylase subunit YdiO"
"DsuD02_00138" 3354 "s59E25H2" 0 "putative BsuMI modification methylase subunit YdiO"
"DsuD02_00138" 3354 "s59E5H2" 0 "putative BsuMI modification methylase subunit YdiO"
"DsuD02_00138" 3354 "s63E6H2" 0 "putative BsuMI modification methylase subunit YdiO"
"DsuD02_00139" 3355 "s59E13H2" 0.01438161 "Very short patch repair protein"
"DsuD02_00139" 3355 "s59E25H2" 0 "Very short patch repair protein"
"DsuD02_00139" 3355 "s59E5H2" 0 "Very short patch repair protein"
"DsuD02_00139" 3355 "s63E6H2" 0 "Very short patch repair protein"
"DsuD02_00140" 3356 "s59E13H2" 0.001011588 "HNH endonuclease"
"DsuD02_00140" 3356 "s59E25H2" 0 "HNH endonuclease"
"DsuD02_00140" 3356 "s59E5H2" 0 "HNH endonuclease"
"DsuD02_00140" 3356 "s63E6H2" 0 "HNH endonuclease"
"DsuD02_00142" 3358 "s59E13H2" 0 "Amidohydrolase"
"DsuD02_00142" 3358 "s59E25H2" 0 "Amidohydrolase"
"DsuD02_00142" 3358 "s59E5H2" 0 "Amidohydrolase"
"DsuD02_00142" 3358 "s63E6H2" 0 "Amidohydrolase"

"DsuD02_00147" 3363 "s59E13H2" 0 "hypothetical protein"
"DsuD02_00147" 3363 "s59E25H2" 0 "hypothetical protein"
"DsuD02_00147" 3363 "s59E5H2" 0 "hypothetical protein"
"DsuD02_00147" 3363 "s63E6H2" 0 "hypothetical protein"
"DsuD02_00148" 3364 "s59E13H2" 0 "putative BsuMI modification methylase subunit YdiO"
"DsuD02_00148" 3364 "s59E25H2" 0 "putative BsuMI modification methylase subunit YdiO"
"DsuD02_00148" 3364 "s59E5H2" 0 "putative BsuMI modification methylase subunit YdiO"
"DsuD02_00148" 3364 "s63E6H2" 0 "putative BsuMI modification methylase subunit YdiO"
"DsuD02_00149" 3365 "s59E13H2" 0 "Very short patch repair protein"
"DsuD02_00149" 3365 "s59E25H2" 0 "Very short patch repair protein"
"DsuD02_00149" 3365 "s59E5H2" 0 "Very short patch repair protein"
"DsuD02_00149" 3365 "s63E6H2" 0 "Very short patch repair protein"
"DsuD02_00150" 3366 "s59E13H2" 0 "HNH endonuclease"
"DsuD02_00150" 3366 "s59E25H2" 0 "HNH endonuclease"
"DsuD02_00150" 3366 "s59E5H2" 0 "HNH endonuclease"
"DsuD02_00150" 3366 "s63E6H2" 0 "HNH endonuclease"
"DsuD02_00152" 3368 "s59E13H2" 0 "Transposase IS200 like protein"
"DsuD02_00152" 3368 "s59E25H2" 0 "Transposase IS200 like protein"
"DsuD02_00152" 3368 "s59E5H2" 0 "Transposase IS200 like protein"
"DsuD02_00152" 3368 "s63E6H2" 0 "Transposase IS200 like protein"
"DsuD02_00153" 3369 "s59E13H2" 0 "hypothetical protein"
"DsuD02_00153" 3369 "s59E25H2" 0 "hypothetical protein"
"DsuD02_00153" 3369 "s59E5H2" 0 "hypothetical protein"
"DsuD02_00153" 3369 "s63E6H2" 0 "hypothetical protein"
"DsuD02_00154" 3370 "s59E13H2" 0 "hypothetical protein"
"DsuD02_00154" 3370 "s59E25H2" 0 "hypothetical protein"
"DsuD02_00154" 3370 "s59E5H2" 0 "hypothetical protein"
"DsuD02_00154" 3370 "s63E6H2" 0 "hypothetical protein"
"DsuD02_00157" 3373 "s59E13H2" 0 "Toxin RelE3"
"DsuD02_00157" 3373 "s59E25H2" 0 "Toxin RelE3"
"DsuD02_00157" 3373 "s59E5H2" 0 "Toxin RelE3"
"DsuD02_00157" 3373 "s63E6H2" 0 "Toxin RelE3"
"DsuD02_00158" 3374 "s59E13H2" 0 "Phd_YefM"
"DsuD02_00158" 3374 "s59E25H2" 0 "Phd_YefM"
"DsuD02_00158" 3374 "s59E5H2" 0 "Phd_YefM"
"DsuD02_00158" 3374 "s63E6H2" 0 "Phd_YefM"
"DsuD02_00159" 3375 "s59E13H2" 0 "hypothetical protein"
"DsuD02_00159" 3375 "s59E25H2" 0 "hypothetical protein"
"DsuD02_00159" 3375 "s59E5H2" 0 "hypothetical protein"
"DsuD02_00159" 3375 "s63E6H2" 0 "hypothetical protein"
"DsuD02_00160" 3376 "s59E13H2" 0 "Plasmid stabilisation system protein"
"DsuD02_00160" 3376 "s59E25H2" 0 "Plasmid stabilisation system protein"
"DsuD02_00160" 3376 "s59E5H2" 0 "Plasmid stabilisation system protein"
"DsuD02_00160" 3376 "s63E6H2" 0 "Plasmid stabilisation system protein"
"DsuD02_00162" 3378 "s59E13H2" 0 "Acyl-CoA dehydrogenase"
"DsuD02_00162" 3378 "s59E25H2" 0 "Acyl-CoA dehydrogenase"
"DsuD02_00162" 3378 "s59E5H2" 0 "Acyl-CoA dehydrogenase"

"DsuD02_00162" 3378 "s63E6H2" 0 "Acyl-CoA dehydrogenase"
"DsuD02_00163" 3379 "s59E13H2" 0 "4-aminobutyrate aminotransferase GabT"
"DsuD02_00163" 3379 "s59E25H2" 0 "4-aminobutyrate aminotransferase GabT"
"DsuD02_00163" 3379 "s59E5H2" 0 "4-aminobutyrate aminotransferase GabT"
"DsuD02_00163" 3379 "s63E6H2" 0 "4-aminobutyrate aminotransferase GabT"
"DsuD02_00164" 3380 "s59E13H2" 0 "putative hydrolase"
"DsuD02_00164" 3380 "s59E25H2" 0 "putative hydrolase"
"DsuD02_00164" 3380 "s59E5H2" 0 "putative hydrolase"
"DsuD02_00164" 3380 "s63E6H2" 0 "putative hydrolase"
"DsuD02_00165" 3381 "s59E13H2" 0 "hypothetical protein"
"DsuD02_00165" 3381 "s59E25H2" 0 "hypothetical protein"
"DsuD02_00165" 3381 "s59E5H2" 0 "hypothetical protein"
"DsuD02_00165" 3381 "s63E6H2" 0 "hypothetical protein"
"DsuD02_00166" 3382 "s59E13H2" 0 "p-aminobenzoyl-glutamate hydrolase subunit B"
"DsuD02_00166" 3382 "s59E25H2" 0 "p-aminobenzoyl-glutamate hydrolase subunit B"
"DsuD02_00166" 3382 "s59E5H2" 0 "p-aminobenzoyl-glutamate hydrolase subunit B"
"DsuD02_00166" 3382 "s63E6H2" 0 "p-aminobenzoyl-glutamate hydrolase subunit B"
"DsuD02_00168" 3384 "s59E13H2" 0 "2-oxoglutaramate amidase"
"DsuD02_00168" 3384 "s59E25H2" 0 "2-oxoglutaramate amidase"
"DsuD02_00168" 3384 "s59E5H2" 0 "2-oxoglutaramate amidase"
"DsuD02_00168" 3384 "s63E6H2" 0 "2-oxoglutaramate amidase"
"DsuD02_00169" 3385 "s59E13H2" 0 "leucine/isoleucine/valine transporter permease subunit"
"DsuD02_00169" 3385 "s59E25H2" 0 "leucine/isoleucine/valine transporter permease subunit"
"DsuD02_00169" 3385 "s59E5H2" 0 "leucine/isoleucine/valine transporter permease subunit"
"DsuD02_00169" 3385 "s63E6H2" 0 "leucine/isoleucine/valine transporter permease subunit"
"DsuD02_00170" 3386 "s59E13H2" 0 "High-affinity branched-chain amino acid transport system permease protein LivH"
"DsuD02_00170" 3386 "s59E25H2" 0 "High-affinity branched-chain amino acid transport system permease protein LivH"
"DsuD02_00170" 3386 "s59E5H2" 0 "High-affinity branched-chain amino acid transport system permease protein LivH"
"DsuD02_00170" 3386 "s63E6H2" 0 "High-affinity branched-chain amino acid transport system permease protein LivH"
"DsuD02_00171" 3387 "s59E13H2" 0 "High-affinity branched-chain amino acid transport ATP-binding protein LivF"
"DsuD02_00171" 3387 "s59E25H2" 0 "High-affinity branched-chain amino acid transport ATP-binding protein LivF"
"DsuD02_00171" 3387 "s59E5H2" 0 "High-affinity branched-chain amino acid transport ATP-binding protein LivF"
"DsuD02_00171" 3387 "s63E6H2" 0 "High-affinity branched-chain amino acid transport ATP-binding protein LivF"
"DsuD02_00172" 3388 "s59E13H2" 0 "putative branched-chain amino acid transport ATP-binding protein LivG"
"DsuD02_00172" 3388 "s59E25H2" 0 "putative branched-chain amino acid transport ATP-binding protein LivG"
"DsuD02_00172" 3388 "s59E5H2" 0 "putative branched-chain amino acid transport ATP-binding protein LivG"
"DsuD02_00172" 3388 "s63E6H2" 0 "putative branched-chain amino acid transport ATP-binding protein LivG"
"DsuD02_00173" 3389 "s59E13H2" 0 "Receptor family ligand binding region"
"DsuD02_00173" 3389 "s59E25H2" 0 "Receptor family ligand binding region"
"DsuD02_00173" 3389 "s59E5H2" 0 "Receptor family ligand binding region"
"DsuD02_00173" 3389 "s63E6H2" 0 "Receptor family ligand binding region"
"DsuD02_00175" 3391 "s59E13H2" 0 "Tungsten-containing aldehyde ferredoxin oxidoreductase"
"DsuD02_00175" 3391 "s59E25H2" 0 "Tungsten-containing aldehyde ferredoxin oxidoreductase"
"DsuD02_00175" 3391 "s59E5H2" 0 "Tungsten-containing aldehyde ferredoxin oxidoreductase"
"DsuD02_00175" 3391 "s63E6H2" 0 "Tungsten-containing aldehyde ferredoxin oxidoreductase"
"DsuD02_00176" 3392 "s59E13H2" 0 "Histone deacetylase-like amidohydrolase"
"DsuD02_00176" 3392 "s59E25H2" 0 "Histone deacetylase-like amidohydrolase"

"DsuD02_00176" 3392 "s59E5H2" 0 "Histone deacetylase-like amidohydrolase"
"DsuD02_00176" 3392 "s63E6H2" 0 "Histone deacetylase-like amidohydrolase"
"DsuD02_00177" 3393 "s59E13H2" 0 "Phosphoribosylformylglycinamidine synthase 1"
"DsuD02_00177" 3393 "s59E25H2" 0 "Phosphoribosylformylglycinamidine synthase 1"
"DsuD02_00177" 3393 "s59E5H2" 0 "Phosphoribosylformylglycinamidine synthase 1"
"DsuD02_00177" 3393 "s63E6H2" 0 "Phosphoribosylformylglycinamidine synthase 1"
"DsuD02_00178" 3394 "s59E13H2" 0 "Phosphoribosylformylglycinamidine synthase 2"
"DsuD02_00178" 3394 "s59E25H2" 0 "Phosphoribosylformylglycinamidine synthase 2"
"DsuD02_00178" 3394 "s59E5H2" 0 "Phosphoribosylformylglycinamidine synthase 2"
"DsuD02_00178" 3394 "s63E6H2" 0 "Phosphoribosylformylglycinamidine synthase 2"
"DsuD02_00179" 3395 "s59E13H2" 0 "phosphohistidine phosphatase"
"DsuD02_00179" 3395 "s59E25H2" 0 "phosphohistidine phosphatase"
"DsuD02_00179" 3395 "s59E5H2" 0 "phosphohistidine phosphatase"
"DsuD02_00179" 3395 "s63E6H2" 0 "phosphohistidine phosphatase"
"DsuD02_00180" 3396 "s59E13H2" 0 "Tetracycline resistance protein, class B"
"DsuD02_00180" 3396 "s59E25H2" 0 "Tetracycline resistance protein, class B"
"DsuD02_00180" 3396 "s59E5H2" 0 "Tetracycline resistance protein, class B"
"DsuD02_00180" 3396 "s63E6H2" 0 "Tetracycline resistance protein, class B"
"DsuD02_00181" 3397 "s59E13H2" 0 "hypothetical protein"
"DsuD02_00181" 3397 "s59E25H2" 0 "hypothetical protein"
"DsuD02_00181" 3397 "s59E5H2" 0 "hypothetical protein"
"DsuD02_00181" 3397 "s63E6H2" 0 "hypothetical protein"
"DsuD02_00182" 3398 "s59E13H2" 0 "Glycosyltransferase AglJ"
"DsuD02_00182" 3398 "s59E25H2" 0 "Glycosyltransferase AglJ"
"DsuD02_00182" 3398 "s59E5H2" 0 "Glycosyltransferase AglJ"
"DsuD02_00182" 3398 "s63E6H2" 0 "Glycosyltransferase AglJ"
"DsuD02_00183" 3399 "s59E13H2" 0 "Undecaprenyl-phosphate 4-deoxy-4-formamido-L-arabinose transferase"
"DsuD02_00183" 3399 "s59E25H2" 0 "Undecaprenyl-phosphate 4-deoxy-4-formamido-L-arabinose transferase"
"DsuD02_00183" 3399 "s59E5H2" 0 "Undecaprenyl-phosphate 4-deoxy-4-formamido-L-arabinose transferase"
"DsuD02_00183" 3399 "s63E6H2" 0 "Undecaprenyl-phosphate 4-deoxy-4-formamido-L-arabinose transferase"
"DsuD02_00184" 3400 "s59E13H2" 0 "Glycosyltransferase AglD"
"DsuD02_00184" 3400 "s59E25H2" 0 "Glycosyltransferase AglD"
"DsuD02_00184" 3400 "s59E5H2" 0 "Glycosyltransferase AglD"
"DsuD02_00184" 3400 "s63E6H2" 0 "Glycosyltransferase AglD"
"DsuD02_00185" 3401 "s59E13H2" 0 "hypothetical protein"
"DsuD02_00185" 3401 "s59E25H2" 0 "hypothetical protein"
"DsuD02_00185" 3401 "s59E5H2" 0 "hypothetical protein"
"DsuD02_00185" 3401 "s63E6H2" 0 "hypothetical protein"
"DsuD02_00186" 3402 "s59E13H2" 0 "FecR protein"
"DsuD02_00186" 3402 "s59E25H2" 0 "FecR protein"
"DsuD02_00186" 3402 "s59E5H2" 0 "FecR protein"
"DsuD02_00186" 3402 "s63E6H2" 0 "FecR protein"
"DsuD02_00187" 3403 "s59E13H2" 0 "photosystem I assembly protein Ycf3"
"DsuD02_00187" 3403 "s59E25H2" 0 "photosystem I assembly protein Ycf3"
"DsuD02_00187" 3403 "s59E5H2" 0 "photosystem I assembly protein Ycf3"
"DsuD02_00187" 3403 "s63E6H2" 0 "photosystem I assembly protein Ycf3"
"DsuD02_00188" 3404 "s59E13H2" 0 "Ribosomal protein S12 methylthiotransferase RimO"

"DsuD02_00188" 3404 "s59E25H2" 0 "Ribosomal protein S12 methylthiotransferase RimO"
"DsuD02_00188" 3404 "s59E5H2" 0 "Ribosomal protein S12 methylthiotransferase RimO"
"DsuD02_00188" 3404 "s63E6H2" 0 "Ribosomal protein S12 methylthiotransferase RimO"
"DsuD02_00189" 3405 "s59E13H2" 0 "Outer membrane protein assembly factor BamD"
"DsuD02_00189" 3405 "s59E25H2" 0 "Outer membrane protein assembly factor BamD"
"DsuD02_00189" 3405 "s59E5H2" 0 "Outer membrane protein assembly factor BamD"
"DsuD02_00189" 3405 "s63E6H2" 0 "Outer membrane protein assembly factor BamD"
"DsuD02_00190" 3406 "s59E13H2" 0 "hypothetical protein"
"DsuD02_00190" 3406 "s59E25H2" 0 "hypothetical protein"
"DsuD02_00190" 3406 "s59E5H2" 0 "hypothetical protein"
"DsuD02_00190" 3406 "s63E6H2" 0 "hypothetical protein"
"DsuD02_00191" 3407 "s59E13H2" 0 "DNA polymerase IV"
"DsuD02_00191" 3407 "s59E25H2" 0 "DNA polymerase IV"
"DsuD02_00191" 3407 "s59E5H2" 0 "DNA polymerase IV"
"DsuD02_00191" 3407 "s63E6H2" 0 "DNA polymerase IV"
"DsuD02_00192" 3408 "s59E13H2" 0 "Elongation factor G"
"DsuD02_00192" 3408 "s59E25H2" 0 "Elongation factor G"
"DsuD02_00192" 3408 "s59E5H2" 0 "Elongation factor G"
"DsuD02_00192" 3408 "s63E6H2" 0 "Elongation factor G"
"DsuD02_00193" 3409 "s59E13H2" 0 "hypothetical protein"
"DsuD02_00193" 3409 "s59E25H2" 0 "hypothetical protein"
"DsuD02_00193" 3409 "s59E5H2" 0 "hypothetical protein"
"DsuD02_00193" 3409 "s63E6H2" 0 "hypothetical protein"
"DsuD02_00194" 3410 "s59E13H2" 0 "hypothetical protein"
"DsuD02_00194" 3410 "s59E25H2" 0 "hypothetical protein"
"DsuD02_00194" 3410 "s59E5H2" 0 "hypothetical protein"
"DsuD02_00194" 3410 "s63E6H2" 0 "hypothetical protein"
"DsuD02_00195" 3411 "s59E13H2" 0 "Integration host factor subunit alpha"
"DsuD02_00195" 3411 "s59E25H2" 0 "Integration host factor subunit alpha"
"DsuD02_00195" 3411 "s59E5H2" 0 "Integration host factor subunit alpha"
"DsuD02_00195" 3411 "s63E6H2" 0 "Integration host factor subunit alpha"
"DsuD02_00196" 3412 "s59E13H2" 0 "DNA-binding protein HU"
"DsuD02_00196" 3412 "s59E25H2" 0 "DNA-binding protein HU"
"DsuD02_00196" 3412 "s59E5H2" 0 "DNA-binding protein HU"
"DsuD02_00196" 3412 "s63E6H2" 0 "DNA-binding protein HU"
"DsuD02_00197" 3413 "s59E13H2" 0 "Diaminopimelate epimerase"
"DsuD02_00197" 3413 "s59E25H2" 0 "Diaminopimelate epimerase"
"DsuD02_00197" 3413 "s59E5H2" 0 "Diaminopimelate epimerase"
"DsuD02_00197" 3413 "s63E6H2" 0 "Diaminopimelate epimerase"
"DsuD02_00198" 3414 "s59E13H2" 0 "hypothetical protein"
"DsuD02_00198" 3414 "s59E25H2" 0 "hypothetical protein"
"DsuD02_00198" 3414 "s59E5H2" 0 "hypothetical protein"
"DsuD02_00198" 3414 "s63E6H2" 0 "hypothetical protein"
"DsuD02_00199" 3415 "s59E13H2" 0 "Lipopolysaccharide export system permease protein LptF"
"DsuD02_00199" 3415 "s59E25H2" 0 "Lipopolysaccharide export system permease protein LptF"
"DsuD02_00199" 3415 "s59E5H2" 0 "Lipopolysaccharide export system permease protein LptF"
"DsuD02_00199" 3415 "s63E6H2" 0 "Lipopolysaccharide export system permease protein LptF"

"DsuD02_00200" 3416 "s59E13H2" 0 "PAP2 superfamily protein"
"DsuD02_00200" 3416 "s59E25H2" 0 "PAP2 superfamily protein"
"DsuD02_00200" 3416 "s59E5H2" 0 "PAP2 superfamily protein"
"DsuD02_00200" 3416 "s63E6H2" 0 "PAP2 superfamily protein"
"DsuD02_00201" 3417 "s59E13H2" 0 "hypothetical protein"
"DsuD02_00201" 3417 "s59E25H2" 0 "hypothetical protein"
"DsuD02_00201" 3417 "s59E5H2" 0 "hypothetical protein"
"DsuD02_00201" 3417 "s63E6H2" 0 "hypothetical protein"
"DsuD02_00202" 3418 "s59E13H2" 0 "Undecaprenyl-phosphate 4-deoxy-4-formamido-L-arabinose transferase"
"DsuD02_00202" 3418 "s59E25H2" 0 "Undecaprenyl-phosphate 4-deoxy-4-formamido-L-arabinose transferase"
"DsuD02_00202" 3418 "s59E5H2" 0 "Undecaprenyl-phosphate 4-deoxy-4-formamido-L-arabinose transferase"
"DsuD02_00202" 3418 "s63E6H2" 0 "Undecaprenyl-phosphate 4-deoxy-4-formamido-L-arabinose transferase"
"DsuD02_00203" 3419 "s59E13H2" 0 "hypothetical protein"
"DsuD02_00203" 3419 "s59E25H2" 0 "hypothetical protein"
"DsuD02_00203" 3419 "s59E5H2" 0 "hypothetical protein"
"DsuD02_00203" 3419 "s63E6H2" 0 "hypothetical protein"
"DsuD02_00204" 3420 "s59E13H2" 0 "hypothetical protein"
"DsuD02_00204" 3420 "s59E25H2" 0 "hypothetical protein"
"DsuD02_00204" 3420 "s59E5H2" 0 "hypothetical protein"
"DsuD02_00204" 3420 "s63E6H2" 0 "hypothetical protein"
"DsuD02_00205" 3421 "s59E13H2" 0 "Undecaprenyl phosphate-alpha-4-amino-4-deoxy-L-arabinose arabinosyl transferase"
"DsuD02_00205" 3421 "s59E25H2" 0 "Undecaprenyl phosphate-alpha-4-amino-4-deoxy-L-arabinose arabinosyl transferase"
"DsuD02_00205" 3421 "s59E5H2" 0 "Undecaprenyl phosphate-alpha-4-amino-4-deoxy-L-arabinose arabinosyl transferase"
"DsuD02_00205" 3421 "s63E6H2" 0 "Undecaprenyl phosphate-alpha-4-amino-4-deoxy-L-arabinose arabinosyl transferase"
"DsuD02_00206" 3422 "s59E13H2" 0 "serine/threonine protein kinase"
"DsuD02_00206" 3422 "s59E25H2" 0 "serine/threonine protein kinase"
"DsuD02_00206" 3422 "s59E5H2" 0 "serine/threonine protein kinase"
"DsuD02_00206" 3422 "s63E6H2" 0 "serine/threonine protein kinase"
"DsuD02_00207" 3423 "s59E13H2" 0 "hypothetical protein"
"DsuD02_00207" 3423 "s59E25H2" 0 "hypothetical protein"
"DsuD02_00207" 3423 "s59E5H2" 0 "hypothetical protein"
"DsuD02_00207" 3423 "s63E6H2" 0 "hypothetical protein"
"DsuD02_00208" 3424 "s59E13H2" 0 "GMP synthase [glutamine-hydrolyzing]"
"DsuD02_00208" 3424 "s59E25H2" 0 "GMP synthase [glutamine-hydrolyzing]"
"DsuD02_00208" 3424 "s59E5H2" 0 "GMP synthase [glutamine-hydrolyzing]"
"DsuD02_00208" 3424 "s63E6H2" 0 "GMP synthase [glutamine-hydrolyzing]"
"DsuD02_00209" 3425 "s59E13H2" 0 "GMP synthase [glutamine-hydrolyzing]"
"DsuD02_00209" 3425 "s59E25H2" 0 "GMP synthase [glutamine-hydrolyzing]"
"DsuD02_00209" 3425 "s59E5H2" 0 "GMP synthase [glutamine-hydrolyzing]"
"DsuD02_00209" 3425 "s63E6H2" 0 "GMP synthase [glutamine-hydrolyzing]"
"DsuD02_00211" 3427 "s59E13H2" 0 "3-deoxy-D-manno-octulosonic acid transferase"
"DsuD02_00211" 3427 "s59E25H2" 0 "3-deoxy-D-manno-octulosonic acid transferase"
"DsuD02_00211" 3427 "s59E5H2" 0 "3-deoxy-D-manno-octulosonic acid transferase"
"DsuD02_00211" 3427 "s63E6H2" 0 "3-deoxy-D-manno-octulosonic acid transferase"
"DsuD02_00212" 3428 "s59E13H2" 0 "hypothetical protein"
"DsuD02_00212" 3428 "s59E25H2" 0 "hypothetical protein"
"DsuD02_00212" 3428 "s59E5H2" 0 "hypothetical protein"

"DsuD02_00212" 3428 "s63E6H2" 0 "hypothetical protein"
"DsuD02_00214" 3430 "s59E13H2" 0 "Phosphoglucosamine mutase"
"DsuD02_00214" 3430 "s59E25H2" 0 "Phosphoglucosamine mutase"
"DsuD02_00214" 3430 "s59E5H2" 0 "Phosphoglucosamine mutase"
"DsuD02_00214" 3430 "s63E6H2" 0 "Phosphoglucosamine mutase"
"DsuD02_00215" 3431 "s59E13H2" 0 "Glucosamine-6-phosphate deaminase 1"
"DsuD02_00215" 3431 "s59E25H2" 0 "Glucosamine-6-phosphate deaminase 1"
"DsuD02_00215" 3431 "s59E5H2" 0 "Glucosamine-6-phosphate deaminase 1"
"DsuD02_00215" 3431 "s63E6H2" 0 "Glucosamine-6-phosphate deaminase 1"
"DsuD02_00216" 3432 "s59E13H2" 0 "Glutamine--fructose-6-phosphate aminotransferase [isomerizing]"
"DsuD02_00216" 3432 "s59E25H2" 0 "Glutamine--fructose-6-phosphate aminotransferase [isomerizing]"
"DsuD02_00216" 3432 "s59E5H2" 0 "Glutamine--fructose-6-phosphate aminotransferase [isomerizing]"
"DsuD02_00216" 3432 "s63E6H2" 0 "Glutamine--fructose-6-phosphate aminotransferase [isomerizing]"
"DsuD02_00217" 3433 "s59E13H2" 0 "putative transcriptional regulatory protein pdtaR"
"DsuD02_00217" 3433 "s59E25H2" 0 "putative transcriptional regulatory protein pdtaR"
"DsuD02_00217" 3433 "s59E5H2" 0 "putative transcriptional regulatory protein pdtaR"
"DsuD02_00217" 3433 "s63E6H2" 0 "putative transcriptional regulatory protein pdtaR"
"DsuD02_00218" 3434 "s59E13H2" 0 "Glutamate dehydrogenase"
"DsuD02_00218" 3434 "s59E25H2" 0 "Glutamate dehydrogenase"
"DsuD02_00218" 3434 "s59E5H2" 0 "Glutamate dehydrogenase"
"DsuD02_00218" 3434 "s63E6H2" 0 "Glutamate dehydrogenase"
"DsuD02_00219" 3435 "s59E13H2" 0 "phosphoenolpyruvate synthase"
"DsuD02_00219" 3435 "s59E25H2" 0 "phosphoenolpyruvate synthase"
"DsuD02_00219" 3435 "s59E5H2" 0 "phosphoenolpyruvate synthase"
"DsuD02_00219" 3435 "s63E6H2" 0 "phosphoenolpyruvate synthase"
"DsuD02_00220" 3436 "s59E13H2" 0 "hypothetical protein"
"DsuD02_00220" 3436 "s59E25H2" 0 "hypothetical protein"
"DsuD02_00220" 3436 "s59E5H2" 0 "hypothetical protein"
"DsuD02_00220" 3436 "s63E6H2" 0 "hypothetical protein"
"DsuD02_00221" 3437 "s59E13H2" 0 "hypothetical protein"
"DsuD02_00221" 3437 "s59E25H2" 0 "hypothetical protein"
"DsuD02_00221" 3437 "s59E5H2" 0 "hypothetical protein"
"DsuD02_00221" 3437 "s63E6H2" 0 "hypothetical protein"
"DsuD02_00223" 3439 "s59E13H2" 0 "Carbamoyl-phosphate synthase large chain"
"DsuD02_00223" 3439 "s59E25H2" 0 "Carbamoyl-phosphate synthase large chain"
"DsuD02_00223" 3439 "s59E5H2" 0 "Carbamoyl-phosphate synthase large chain"
"DsuD02_00223" 3439 "s63E6H2" 0 "Carbamoyl-phosphate synthase large chain"
"DsuD02_00224" 3440 "s59E13H2" 0 "Amidophosphoribosyltransferase precursor"
"DsuD02_00224" 3440 "s59E25H2" 0 "Amidophosphoribosyltransferase precursor"
"DsuD02_00224" 3440 "s59E5H2" 0.005473789 "Amidophosphoribosyltransferase precursor"
"DsuD02_00224" 3440 "s63E6H2" 0 "Amidophosphoribosyltransferase precursor"
"DsuD02_00225" 3441 "s59E13H2" 0 "Glutamine-dependent NAD(+) synthetase"
"DsuD02_00225" 3441 "s59E25H2" 0 "Glutamine-dependent NAD(+) synthetase"
"DsuD02_00225" 3441 "s59E5H2" 0 "Glutamine-dependent NAD(+) synthetase"
"DsuD02_00225" 3441 "s63E6H2" 0 "Glutamine-dependent NAD(+) synthetase"
"DsuD02_00226" 3442 "s59E13H2" 0 "putative glutamine synthetase 2"
"DsuD02_00226" 3442 "s59E25H2" 0 "putative glutamine synthetase 2"

"DsuD02_00226" 3442 "s59E5H2" 0 "putative glutamine synthetase 2"
"DsuD02_00226" 3442 "s63E6H2" 0 "putative glutamine synthetase 2"
"DsuD02_00227" 3443 "s59E13H2" 0 "asparagine synthetase A"
"DsuD02_00227" 3443 "s59E25H2" 0 "asparagine synthetase A"
"DsuD02_00227" 3443 "s59E5H2" 0 "asparagine synthetase A"
"DsuD02_00227" 3443 "s63E6H2" 0 "asparagine synthetase A"
"DsuD02_00228" 3444 "s59E13H2" 0 "Bifunctional ligase/repressor BirA"
"DsuD02_00228" 3444 "s59E25H2" 0 "Bifunctional ligase/repressor BirA"
"DsuD02_00228" 3444 "s59E5H2" 0 "Bifunctional ligase/repressor BirA"
"DsuD02_00228" 3444 "s63E6H2" 0 "Bifunctional ligase/repressor BirA"
"DsuD02_00230" 3446 "s59E13H2" 0 "DNA gyrase subunit A"
"DsuD02_00230" 3446 "s59E25H2" 0 "DNA gyrase subunit A"
"DsuD02_00230" 3446 "s59E5H2" 0.001669935 "DNA gyrase subunit A"
"DsuD02_00230" 3446 "s63E6H2" 0 "DNA gyrase subunit A"
"DsuD02_00231" 3447 "s59E13H2" 0 "DNA gyrase subunit B"
"DsuD02_00231" 3447 "s59E25H2" 0 "DNA gyrase subunit B"
"DsuD02_00231" 3447 "s59E5H2" 0 "DNA gyrase subunit B"
"DsuD02_00231" 3447 "s63E6H2" 0 "DNA gyrase subunit B"
"DsuD02_00232" 3448 "s59E13H2" 0 "DNA polymerase III subunit beta"
"DsuD02_00232" 3448 "s59E25H2" 0 "DNA polymerase III subunit beta"
"DsuD02_00232" 3448 "s59E5H2" 0 "DNA polymerase III subunit beta"
"DsuD02_00232" 3448 "s63E6H2" 0 "DNA polymerase III subunit beta"
"DsuD02_00233" 3449 "s59E13H2" 0 "Glutaryl-CoA dehydrogenase"
"DsuD02_00233" 3449 "s59E25H2" 0 "Glutaryl-CoA dehydrogenase"
"DsuD02_00233" 3449 "s59E5H2" 0 "Glutaryl-CoA dehydrogenase"
"DsuD02_00233" 3449 "s63E6H2" 0 "Glutaryl-CoA dehydrogenase"
"DsuD02_00234" 3450 "s59E13H2" 0 "tRNA modification GTPase MnmE"
"DsuD02_00234" 3450 "s59E25H2" 0 "tRNA modification GTPase MnmE"
"DsuD02_00234" 3450 "s59E5H2" 0 "tRNA modification GTPase MnmE"
"DsuD02_00234" 3450 "s63E6H2" 0 "tRNA modification GTPase MnmE"
"DsuD02_00235" 3451 "s59E13H2" 0 "ATP-dependent zinc metalloprotease FtsH"
"DsuD02_00235" 3451 "s59E25H2" 0 "ATP-dependent zinc metalloprotease FtsH"
"DsuD02_00235" 3451 "s59E5H2" 0 "ATP-dependent zinc metalloprotease FtsH"
"DsuD02_00235" 3451 "s63E6H2" 0 "ATP-dependent zinc metalloprotease FtsH"
"DsuD02_00236" 3452 "s59E13H2" 0 "Dihydropteroate synthase"
"DsuD02_00236" 3452 "s59E25H2" 0 "Dihydropteroate synthase"
"DsuD02_00236" 3452 "s59E5H2" 0 "Dihydropteroate synthase"
"DsuD02_00236" 3452 "s63E6H2" 0 "Dihydropteroate synthase"
"DsuD02_00237" 3453 "s59E13H2" 0 "Type III pantothenate kinase"
"DsuD02_00237" 3453 "s59E25H2" 0 "Type III pantothenate kinase"
"DsuD02_00237" 3453 "s59E5H2" 0 "Type III pantothenate kinase"
"DsuD02_00237" 3453 "s63E6H2" 0 "Type III pantothenate kinase"
"DsuD02_00238" 3454 "s59E13H2" 0 "hypothetical protein"
"DsuD02_00238" 3454 "s59E25H2" 0 "hypothetical protein"
"DsuD02_00238" 3454 "s59E5H2" 0 "hypothetical protein"
"DsuD02_00238" 3454 "s63E6H2" 0 "hypothetical protein"
"DsuD02_00239" 3455 "s59E13H2" 0 "Gas vesicle protein K"

"DsuD02_00239" 3455 "s59E25H2" 0 "Gas vesicle protein K"
"DsuD02_00239" 3455 "s59E5H2" 0 "Gas vesicle protein K"
"DsuD02_00239" 3455 "s63E6H2" 0 "Gas vesicle protein K"
"DsuD02_00240" 3456 "s59E13H2" 0 "gas vesicle synthesis protein GvpA"
"DsuD02_00240" 3456 "s59E25H2" 0 "gas vesicle synthesis protein GvpA"
"DsuD02_00240" 3456 "s59E5H2" 0 "gas vesicle synthesis protein GvpA"
"DsuD02_00240" 3456 "s63E6H2" 0 "gas vesicle synthesis protein GvpA"
"DsuD02_00241" 3457 "s59E13H2" 0 "Putative arsenical pump-driving ATPase"
"DsuD02_00241" 3457 "s59E25H2" 0 "Putative arsenical pump-driving ATPase"
"DsuD02_00241" 3457 "s59E5H2" 0 "Putative arsenical pump-driving ATPase"
"DsuD02_00241" 3457 "s63E6H2" 0 "Putative arsenical pump-driving ATPase"
"DsuD02_00243" 3459 "s59E13H2" 0 "Gas vesicle protein G"
"DsuD02_00243" 3459 "s59E25H2" 0 "Gas vesicle protein G"
"DsuD02_00243" 3459 "s59E5H2" 0 "Gas vesicle protein G"
"DsuD02_00243" 3459 "s63E6H2" 0 "Gas vesicle protein G"
"DsuD02_00244" 3460 "s59E13H2" 0 "Gas vesicle synthesis protein GvpL/GvpF"
"DsuD02_00244" 3460 "s59E25H2" 0 "Gas vesicle synthesis protein GvpL/GvpF"
"DsuD02_00244" 3460 "s59E5H2" 0 "Gas vesicle synthesis protein GvpL/GvpF"
"DsuD02_00244" 3460 "s63E6H2" 0 "Gas vesicle synthesis protein GvpL/GvpF"
"DsuD02_00246" 3462 "s59E13H2" 0 "chaperone protein DnaJ"
"DsuD02_00246" 3462 "s59E25H2" 0 "chaperone protein DnaJ"
"DsuD02_00246" 3462 "s59E5H2" 0 "chaperone protein DnaJ"
"DsuD02_00246" 3462 "s63E6H2" 0 "chaperone protein DnaJ"
"DsuD02_00248" 3464 "s59E13H2" 0 "Denitrification regulatory protein NirQ"
"DsuD02_00248" 3464 "s59E25H2" 0 "Denitrification regulatory protein NirQ"
"DsuD02_00248" 3464 "s59E5H2" 0 "Denitrification regulatory protein NirQ"
"DsuD02_00248" 3464 "s63E6H2" 0 "Denitrification regulatory protein NirQ"
"DsuD02_00249" 3465 "s59E13H2" 0 "chaperone protein DnaJ"
"DsuD02_00249" 3465 "s59E25H2" 0 "chaperone protein DnaJ"
"DsuD02_00249" 3465 "s59E5H2" 0 "chaperone protein DnaJ"
"DsuD02_00249" 3465 "s63E6H2" 0 "chaperone protein DnaJ"
"DsuD02_00250" 3466 "s59E13H2" 0 "50S ribosomal protein L21/unknown domain fusion protein"
"DsuD02_00250" 3466 "s59E25H2" 0 "50S ribosomal protein L21/unknown domain fusion protein"
"DsuD02_00250" 3466 "s59E5H2" 0 "50S ribosomal protein L21/unknown domain fusion protein"
"DsuD02_00250" 3466 "s63E6H2" 0 "50S ribosomal protein L21/unknown domain fusion protein"
"DsuD02_00251" 3467 "s59E13H2" 0 "Gas vesicle structural protein"
"DsuD02_00251" 3467 "s59E25H2" 0 "Gas vesicle structural protein"
"DsuD02_00251" 3467 "s59E5H2" 0 "Gas vesicle structural protein"
"DsuD02_00251" 3467 "s63E6H2" 0 "Gas vesicle structural protein"
"DsuD02_00252" 3468 "s59E13H2" 0 "hypothetical protein"
"DsuD02_00252" 3468 "s59E25H2" 0 "hypothetical protein"
"DsuD02_00252" 3468 "s59E5H2" 0 "hypothetical protein"
"DsuD02_00252" 3468 "s63E6H2" 0 "hypothetical protein"
"DsuD02_00253" 3469 "s59E13H2" 0 "hypothetical protein"
"DsuD02_00253" 3469 "s59E25H2" 0 "hypothetical protein"
"DsuD02_00253" 3469 "s59E5H2" 0 "hypothetical protein"
"DsuD02_00253" 3469 "s63E6H2" 0 "hypothetical protein"

"DsuD02_00254" 3470 "s59E13H2" 0 "Sporulation initiation phosphotransferase F"
"DsuD02_00254" 3470 "s59E25H2" 0 "Sporulation initiation phosphotransferase F"
"DsuD02_00254" 3470 "s59E5H2" 0 "Sporulation initiation phosphotransferase F"
"DsuD02_00254" 3470 "s63E6H2" 0 "Sporulation initiation phosphotransferase F"
"DsuD02_00255" 3471 "s59E13H2" 0 "hypothetical protein"
"DsuD02_00255" 3471 "s59E25H2" 0 "hypothetical protein"
"DsuD02_00255" 3471 "s59E5H2" 0 "hypothetical protein"
"DsuD02_00255" 3471 "s63E6H2" 0 "hypothetical protein"
"DsuD02_00256" 3472 "s59E13H2" 0 "Sporulation kinase A"
"DsuD02_00256" 3472 "s59E25H2" 0 "Sporulation kinase A"
"DsuD02_00256" 3472 "s59E5H2" 0 "Sporulation kinase A"
"DsuD02_00256" 3472 "s63E6H2" 0 "Sporulation kinase A"
"DsuD02_00259" 3475 "s59E13H2" 0 "Gas vesicle synthesis protein GvpL/GvpF"
"DsuD02_00259" 3475 "s59E25H2" 0 "Gas vesicle synthesis protein GvpL/GvpF"
"DsuD02_00259" 3475 "s59E5H2" 0 "Gas vesicle synthesis protein GvpL/GvpF"
"DsuD02_00259" 3475 "s63E6H2" 0 "Gas vesicle synthesis protein GvpL/GvpF"
"DsuD02_00260" 3476 "s59E13H2" 0 "Gas vesicle synthesis protein GvpL/GvpF"
"DsuD02_00260" 3476 "s59E25H2" 0 "Gas vesicle synthesis protein GvpL/GvpF"
"DsuD02_00260" 3476 "s59E5H2" 0 "Gas vesicle synthesis protein GvpL/GvpF"
"DsuD02_00260" 3476 "s63E6H2" 0 "Gas vesicle synthesis protein GvpL/GvpF"
"DsuD02_00261" 3477 "s59E13H2" 0 "Sporulation initiation phosphotransferase F"
"DsuD02_00261" 3477 "s59E25H2" 0 "Sporulation initiation phosphotransferase F"
"DsuD02_00261" 3477 "s59E5H2" 0 "Sporulation initiation phosphotransferase F"
"DsuD02_00261" 3477 "s63E6H2" 0 "Sporulation initiation phosphotransferase F"
"DsuD02_00262" 3478 "s59E13H2" 0 "hypothetical protein"
"DsuD02_00262" 3478 "s59E25H2" 0 "hypothetical protein"
"DsuD02_00262" 3478 "s59E5H2" 0 "hypothetical protein"
"DsuD02_00262" 3478 "s63E6H2" 0 "hypothetical protein"
"DsuD02_00263" 3479 "s59E13H2" 0 "VCP-like ATPase"
"DsuD02_00263" 3479 "s59E25H2" 0 "VCP-like ATPase"
"DsuD02_00263" 3479 "s59E5H2" 0 "VCP-like ATPase"
"DsuD02_00263" 3479 "s63E6H2" 0 "VCP-like ATPase"
"DsuD02_00264" 3480 "s59E13H2" 0 "GIY-YIG nuclease superfamily protein"
"DsuD02_00264" 3480 "s59E25H2" 0 "GIY-YIG nuclease superfamily protein"
"DsuD02_00264" 3480 "s59E5H2" 0 "GIY-YIG nuclease superfamily protein"
"DsuD02_00264" 3480 "s63E6H2" 0.001072816 "GIY-YIG nuclease superfamily protein"
"DsuD02_00265" 3481 "s59E13H2" 0 "Hsp20/alpha crystallin family protein"
"DsuD02_00265" 3481 "s59E25H2" 0 "Hsp20/alpha crystallin family protein"
"DsuD02_00265" 3481 "s59E5H2" 0 "Hsp20/alpha crystallin family protein"
"DsuD02_00265" 3481 "s63E6H2" 0 "Hsp20/alpha crystallin family protein"
"DsuD02_00266" 3482 "s59E13H2" 0 "Nitrogen regulation protein NR(I)"
"DsuD02_00266" 3482 "s59E25H2" 0 "Nitrogen regulation protein NR(I)"
"DsuD02_00266" 3482 "s59E5H2" 0 "Nitrogen regulation protein NR(I)"
"DsuD02_00266" 3482 "s63E6H2" 0 "Nitrogen regulation protein NR(I)"
"DsuD02_00268" 3484 "s59E13H2" 0 "Transcriptional regulatory protein ZraR"
"DsuD02_00268" 3484 "s59E25H2" 0 "Transcriptional regulatory protein ZraR"
"DsuD02_00268" 3484 "s59E5H2" 0 "Transcriptional regulatory protein ZraR"

"DsuD02_00268" 3484 "s63E6H2" 0 "Transcriptional regulatory protein ZraR"
"DsuD02_00269" 3485 "s59E13H2" 0 "Fe/S biogenesis protein NfuA"
"DsuD02_00269" 3485 "s59E25H2" 0 "Fe/S biogenesis protein NfuA"
"DsuD02_00269" 3485 "s59E5H2" 0 "Fe/S biogenesis protein NfuA"
"DsuD02_00269" 3485 "s63E6H2" 0 "Fe/S biogenesis protein NfuA"
"DsuD02_00270" 3486 "s59E13H2" 0 "Sulfurtransferase TusA"
"DsuD02_00270" 3486 "s59E25H2" 0 "Sulfurtransferase TusA"
"DsuD02_00270" 3486 "s59E5H2" 0 "Sulfurtransferase TusA"
"DsuD02_00270" 3486 "s63E6H2" 0 "Sulfurtransferase TusA"
"DsuD02_00271" 3487 "s59E13H2" 0 "DsrE/DsrF-like family protein"
"DsuD02_00271" 3487 "s59E25H2" 0 "DsrE/DsrF-like family protein"
"DsuD02_00271" 3487 "s59E5H2" 0 "DsrE/DsrF-like family protein"
"DsuD02_00271" 3487 "s63E6H2" 0 "DsrE/DsrF-like family protein"
"DsuD02_00272" 3488 "s59E13H2" 0 "putative cysteine desulfurase 2"
"DsuD02_00272" 3488 "s59E25H2" 0 "putative cysteine desulfurase 2"
"DsuD02_00272" 3488 "s59E5H2" 0 "putative cysteine desulfurase 2"
"DsuD02_00272" 3488 "s63E6H2" 0 "putative cysteine desulfurase 2"
"DsuD02_00273" 3489 "s59E13H2" 0 "NifU-like protein"
"DsuD02_00273" 3489 "s59E25H2" 0 "NifU-like protein"
"DsuD02_00273" 3489 "s59E5H2" 0 "NifU-like protein"
"DsuD02_00273" 3489 "s63E6H2" 0 "NifU-like protein"
"DsuD02_00274" 3490 "s59E13H2" 0 "Copper chaperone CopZ"
"DsuD02_00274" 3490 "s59E25H2" 0 "Copper chaperone CopZ"
"DsuD02_00274" 3490 "s59E5H2" 0 "Copper chaperone CopZ"
"DsuD02_00274" 3490 "s63E6H2" 0 "Copper chaperone CopZ"
"DsuD02_00275" 3491 "s59E13H2" 0 "Copper-exporting P-type ATPase A"
"DsuD02_00275" 3491 "s59E25H2" 0 "Copper-exporting P-type ATPase A"
"DsuD02_00275" 3491 "s59E5H2" 0 "Copper-exporting P-type ATPase A"
"DsuD02_00275" 3491 "s63E6H2" 0 "Copper-exporting P-type ATPase A"
"DsuD02_00276" 3492 "s59E13H2" 0 "hypothetical protein"
"DsuD02_00276" 3492 "s59E25H2" 0 "hypothetical protein"
"DsuD02_00276" 3492 "s59E5H2" 0 "hypothetical protein"
"DsuD02_00276" 3492 "s63E6H2" 0 "hypothetical protein"
"DsuD02_00277" 3493 "s59E13H2" 0 "3-oxoadipate CoA-transferase subunit B"
"DsuD02_00277" 3493 "s59E25H2" 0 "3-oxoadipate CoA-transferase subunit B"
"DsuD02_00277" 3493 "s59E5H2" 0 "3-oxoadipate CoA-transferase subunit B"
"DsuD02_00277" 3493 "s63E6H2" 0 "3-oxoadipate CoA-transferase subunit B"
"DsuD02_00278" 3494 "s59E13H2" 0 "Glutaconate CoA-transferase subunit A"
"DsuD02_00278" 3494 "s59E25H2" 0 "Glutaconate CoA-transferase subunit A"
"DsuD02_00278" 3494 "s59E5H2" 0 "Glutaconate CoA-transferase subunit A"
"DsuD02_00278" 3494 "s63E6H2" 0 "Glutaconate CoA-transferase subunit A"
"DsuD02_00279" 3495 "s59E13H2" 0 "Sulfur carrier protein CysO"
"DsuD02_00279" 3495 "s59E25H2" 0 "Sulfur carrier protein CysO"
"DsuD02_00279" 3495 "s59E5H2" 0 "Sulfur carrier protein CysO"
"DsuD02_00279" 3495 "s63E6H2" 0 "Sulfur carrier protein CysO"
"DsuD02_00280" 3496 "s59E13H2" 0 "hypothetical protein"
"DsuD02_00280" 3496 "s59E25H2" 0 "hypothetical protein"

"DsuD02_00280" 3496 "s59E5H2" 0 "hypothetical protein"
"DsuD02_00280" 3496 "s63E6H2" 0 "hypothetical protein"
"DsuD02_00281" 3497 "s59E13H2" 0 "Double zinc ribbon"
"DsuD02_00281" 3497 "s59E25H2" 0 "Double zinc ribbon"
"DsuD02_00281" 3497 "s59E5H2" 0 "Double zinc ribbon"
"DsuD02_00281" 3497 "s63E6H2" 0 "Double zinc ribbon"
"DsuD02_00283" 3499 "s59E13H2" 0 "hypothetical protein"
"DsuD02_00283" 3499 "s59E25H2" 0 "hypothetical protein"
"DsuD02_00283" 3499 "s59E5H2" 0 "hypothetical protein"
"DsuD02_00283" 3499 "s63E6H2" 0 "hypothetical protein"
"DsuD02_00284" 3500 "s59E13H2" 0 "Ubiquinone/menaquinone biosynthesis C-methyltransferase UbiE"
"DsuD02_00284" 3500 "s59E25H2" 0 "Ubiquinone/menaquinone biosynthesis C-methyltransferase UbiE"
"DsuD02_00284" 3500 "s59E5H2" 0 "Ubiquinone/menaquinone biosynthesis C-methyltransferase UbiE"
"DsuD02_00284" 3500 "s63E6H2" 0 "Ubiquinone/menaquinone biosynthesis C-methyltransferase UbiE"
"DsuD02_00285" 3501 "s59E13H2" 0 "Helix-turn-helix domain protein"
"DsuD02_00285" 3501 "s59E25H2" 0 "Helix-turn-helix domain protein"
"DsuD02_00285" 3501 "s59E5H2" 0 "Helix-turn-helix domain protein"
"DsuD02_00285" 3501 "s63E6H2" 0 "Helix-turn-helix domain protein"
"DsuD02_00286" 3502 "s59E13H2" 0 "hypothetical protein"
"DsuD02_00286" 3502 "s59E25H2" 0 "hypothetical protein"
"DsuD02_00286" 3502 "s59E5H2" 0 "hypothetical protein"
"DsuD02_00286" 3502 "s63E6H2" 0 "hypothetical protein"
"DsuD02_00287" 3503 "s59E13H2" 0 "Methylamine utilisation protein MauE"
"DsuD02_00287" 3503 "s59E25H2" 0 "Methylamine utilisation protein MauE"
"DsuD02_00287" 3503 "s59E5H2" 0 "Methylamine utilisation protein MauE"
"DsuD02_00287" 3503 "s63E6H2" 0 "Methylamine utilisation protein MauE"
"DsuD02_00288" 3504 "s59E13H2" 0 "Rhodanese-like domain protein"
"DsuD02_00288" 3504 "s59E25H2" 0 "Rhodanese-like domain protein"
"DsuD02_00288" 3504 "s59E5H2" 0 "Rhodanese-like domain protein"
"DsuD02_00288" 3504 "s63E6H2" 0 "Rhodanese-like domain protein"
"DsuD02_00289" 3505 "s59E13H2" 0 "thiol-disulfide oxidoreductase"
"DsuD02_00289" 3505 "s59E25H2" 0 "thiol-disulfide oxidoreductase"
"DsuD02_00289" 3505 "s59E5H2" 0 "thiol-disulfide oxidoreductase"
"DsuD02_00289" 3505 "s63E6H2" 0 "thiol-disulfide oxidoreductase"
"DsuD02_00290" 3506 "s59E13H2" 0 "Nitrogen regulation protein NR(I)"
"DsuD02_00290" 3506 "s59E25H2" 0 "Nitrogen regulation protein NR(I)"
"DsuD02_00290" 3506 "s59E5H2" 0 "Nitrogen regulation protein NR(I)"
"DsuD02_00290" 3506 "s63E6H2" 0 "Nitrogen regulation protein NR(I)"
"DsuD02_00291" 3507 "s59E13H2" 0 "Sporulation kinase E"
"DsuD02_00291" 3507 "s59E25H2" 0 "Sporulation kinase E"
"DsuD02_00291" 3507 "s59E5H2" 0 "Sporulation kinase E"
"DsuD02_00291" 3507 "s63E6H2" 0 "Sporulation kinase E"
"DsuD02_00292" 3508 "s59E13H2" 0 "hypothetical protein"
"DsuD02_00292" 3508 "s59E25H2" 0 "hypothetical protein"
"DsuD02_00292" 3508 "s59E5H2" 0 "hypothetical protein"
"DsuD02_00292" 3508 "s63E6H2" 0 "hypothetical protein"
"DsuD02_00293" 3509 "s59E13H2" 0 "Quinolinate synthase A"

"DsuD02_00293" 3509 "s59E25H2" 0 "Quinolinate synthase A"
"DsuD02_00293" 3509 "s59E5H2" 0 "Quinolinate synthase A"
"DsuD02_00293" 3509 "s63E6H2" 0 "Quinolinate synthase A"
"DsuD02_00294" 3510 "s59E13H2" 0 "formate dehydrogenase accessory protein"
"DsuD02_00294" 3510 "s59E25H2" 0 "formate dehydrogenase accessory protein"
"DsuD02_00294" 3510 "s59E5H2" 0 "formate dehydrogenase accessory protein"
"DsuD02_00294" 3510 "s63E6H2" 0 "formate dehydrogenase accessory protein"
"DsuD02_00295" 3511 "s59E13H2" 0 "MOSC domain protein"
"DsuD02_00295" 3511 "s59E25H2" 0 "MOSC domain protein"
"DsuD02_00295" 3511 "s59E5H2" 0 "MOSC domain protein"
"DsuD02_00295" 3511 "s63E6H2" 0 "MOSC domain protein"
"DsuD02_00296" 3512 "s59E13H2" 0 "tRNA-specific 2-thiouridylase MnmA"
"DsuD02_00296" 3512 "s59E25H2" 0 "tRNA-specific 2-thiouridylase MnmA"
"DsuD02_00296" 3512 "s59E5H2" 0 "tRNA-specific 2-thiouridylase MnmA"
"DsuD02_00296" 3512 "s63E6H2" 0 "tRNA-specific 2-thiouridylase MnmA"
"DsuD02_00297" 3513 "s59E13H2" 0 "hypothetical protein"
"DsuD02_00297" 3513 "s59E25H2" 0 "hypothetical protein"
"DsuD02_00297" 3513 "s59E5H2" 0 "hypothetical protein"
"DsuD02_00297" 3513 "s63E6H2" 0 "hypothetical protein"
"DsuD02_00299" 3515 "s59E13H2" 0 "Gas vesicle structural protein"
"DsuD02_00299" 3515 "s59E25H2" 0 "Gas vesicle structural protein"
"DsuD02_00299" 3515 "s59E5H2" 0 "Gas vesicle structural protein"
"DsuD02_00299" 3515 "s63E6H2" 0 "Gas vesicle structural protein"
"DsuD02_00300" 3516 "s59E13H2" 0 "hypothetical protein"
"DsuD02_00300" 3516 "s59E25H2" 0 "hypothetical protein"
"DsuD02_00300" 3516 "s59E5H2" 0 "hypothetical protein"
"DsuD02_00300" 3516 "s63E6H2" 0 "hypothetical protein"
"DsuD02_00301" 3517 "s59E13H2" 0 "ISXO2-like transposase domain protein"
"DsuD02_00301" 3517 "s59E25H2" 0 "ISXO2-like transposase domain protein"
"DsuD02_00301" 3517 "s59E5H2" 0 "ISXO2-like transposase domain protein"
"DsuD02_00301" 3517 "s63E6H2" 0 "ISXO2-like transposase domain protein"
"DsuD02_00303" 3519 "s59E13H2" 0 "hypothetical protein"
"DsuD02_00303" 3519 "s59E25H2" 0 "hypothetical protein"
"DsuD02_00303" 3519 "s59E5H2" 0 "hypothetical protein"
"DsuD02_00303" 3519 "s63E6H2" 0 "hypothetical protein"
"DsuD02_00304" 3520 "s59E13H2" 0 "hypothetical protein"
"DsuD02_00304" 3520 "s59E25H2" 0 "hypothetical protein"
"DsuD02_00304" 3520 "s59E5H2" 0 "hypothetical protein"
"DsuD02_00304" 3520 "s63E6H2" 0 "hypothetical protein"
"DsuD02_00307" 3523 "s59E13H2" 0 "Helix-turn-helix domain protein"
"DsuD02_00307" 3523 "s59E25H2" 0 "Helix-turn-helix domain protein"
"DsuD02_00307" 3523 "s59E5H2" 0 "Helix-turn-helix domain protein"
"DsuD02_00307" 3523 "s63E6H2" 0 "Helix-turn-helix domain protein"
"DsuD02_00308" 3524 "s59E13H2" 0 "Chemotaxis protein methyltransferase"
"DsuD02_00308" 3524 "s59E25H2" 0 "Chemotaxis protein methyltransferase"
"DsuD02_00308" 3524 "s59E5H2" 0 "Chemotaxis protein methyltransferase"
"DsuD02_00308" 3524 "s63E6H2" 0 "Chemotaxis protein methyltransferase"

"DsuD02_00309" 3525 "s59E13H2" 0 "Chemotaxis response regulator protein-glutamate methylesterase"
"DsuD02_00309" 3525 "s59E25H2" 0 "Chemotaxis response regulator protein-glutamate methylesterase"
"DsuD02_00309" 3525 "s59E5H2" 0 "Chemotaxis response regulator protein-glutamate methylesterase"
"DsuD02_00309" 3525 "s63E6H2" 0 "Chemotaxis response regulator protein-glutamate methylesterase"
"DsuD02_00311" 3527 "s59E13H2" 0 "Phytochrome-like protein cph1"
"DsuD02_00311" 3527 "s59E25H2" 0 "Phytochrome-like protein cph1"
"DsuD02_00311" 3527 "s59E5H2" 0 "Phytochrome-like protein cph1"
"DsuD02_00311" 3527 "s63E6H2" 0 "Phytochrome-like protein cph1"
"DsuD02_00312" 3528 "s59E13H2" 0 "Response regulator rcp1"
"DsuD02_00312" 3528 "s59E25H2" 0 "Response regulator rcp1"
"DsuD02_00312" 3528 "s59E5H2" 0 "Response regulator rcp1"
"DsuD02_00312" 3528 "s63E6H2" 0 "Response regulator rcp1"
"DsuD02_00313" 3529 "s59E13H2" 0 "hypothetical protein"
"DsuD02_00313" 3529 "s59E25H2" 0 "hypothetical protein"
"DsuD02_00313" 3529 "s59E5H2" 0 "hypothetical protein"
"DsuD02_00313" 3529 "s63E6H2" 0 "hypothetical protein"
"DsuD02_00314" 3530 "s59E13H2" 0 "SpoVT / AbrB like domain protein"
"DsuD02_00314" 3530 "s59E25H2" 0 "SpoVT / AbrB like domain protein"
"DsuD02_00314" 3530 "s59E5H2" 0 "SpoVT / AbrB like domain protein"
"DsuD02_00314" 3530 "s63E6H2" 0 "SpoVT / AbrB like domain protein"
"DsuD02_00315" 3531 "s59E13H2" 0 "Luminescence regulatory protein LuxO"
"DsuD02_00315" 3531 "s59E25H2" 0 "Luminescence regulatory protein LuxO"
"DsuD02_00315" 3531 "s59E5H2" 0.223765 "Luminescence regulatory protein LuxO"
"DsuD02_00315" 3531 "s63E6H2" 0.001554011 "Luminescence regulatory protein LuxO"
"DsuD02_00316" 3532 "s59E13H2" 0 "Circadian clock protein KaiB"
"DsuD02_00316" 3532 "s59E25H2" 0 "Circadian clock protein KaiB"
"DsuD02_00316" 3532 "s59E5H2" 0.4890807 "Circadian clock protein KaiB"
"DsuD02_00316" 3532 "s63E6H2" 0 "Circadian clock protein KaiB"
"DsuD02_00317" 3533 "s59E13H2" 0 "Circadian clock protein kinase KaiC"
"DsuD02_00317" 3533 "s59E25H2" 0 "Circadian clock protein kinase KaiC"
"DsuD02_00317" 3533 "s59E5H2" 0.3309976 "Circadian clock protein kinase KaiC"
"DsuD02_00317" 3533 "s63E6H2" 0.0056098 "Circadian clock protein kinase KaiC"
"DsuD02_00319" 3535 "s59E13H2" 0 "hypothetical protein"
"DsuD02_00319" 3535 "s59E25H2" 0 "hypothetical protein"
"DsuD02_00319" 3535 "s59E5H2" 0.0170594 "hypothetical protein"
"DsuD02_00319" 3535 "s63E6H2" 0 "hypothetical protein"
"DsuD02_00321" 3537 "s59E13H2" 0 "Non-motile and phage-resistance protein"
"DsuD02_00321" 3537 "s59E25H2" 0 "Non-motile and phage-resistance protein"
"DsuD02_00321" 3537 "s59E5H2" 0.0342117 "Non-motile and phage-resistance protein"
"DsuD02_00321" 3537 "s63E6H2" 0 "Non-motile and phage-resistance protein"
"DsuD02_00322" 3538 "s59E13H2" 0 "PemK-like protein"
"DsuD02_00322" 3538 "s59E25H2" 0 "PemK-like protein"
"DsuD02_00322" 3538 "s59E5H2" 0 "PemK-like protein"
"DsuD02_00322" 3538 "s63E6H2" 0 "PemK-like protein"
"DsuD02_00323" 3539 "s59E13H2" 0 "hypothetical protein"
"DsuD02_00323" 3539 "s59E25H2" 0 "hypothetical protein"
"DsuD02_00323" 3539 "s59E5H2" 0 "hypothetical protein"

"DsuD02_00323" 3539 "s63E6H2" 0 "hypothetical protein"
"DsuD02_00324" 3540 "s59E13H2" 0 "RNA polymerase sigma factor RpoD"
"DsuD02_00324" 3540 "s59E25H2" 0 "RNA polymerase sigma factor RpoD"
"DsuD02_00324" 3540 "s59E5H2" 0 "RNA polymerase sigma factor RpoD"
"DsuD02_00324" 3540 "s63E6H2" 0 "RNA polymerase sigma factor RpoD"
"DsuD02_00325" 3541 "s59E13H2" 0 "Putative zinc ribbon domain protein"
"DsuD02_00325" 3541 "s59E25H2" 0 "Putative zinc ribbon domain protein"
"DsuD02_00325" 3541 "s59E5H2" 0 "Putative zinc ribbon domain protein"
"DsuD02_00325" 3541 "s63E6H2" 0 "Putative zinc ribbon domain protein"
"DsuD02_00329" 3545 "s59E13H2" 0 "YGGT family protein"
"DsuD02_00329" 3545 "s59E25H2" 0 "YGGT family protein"
"DsuD02_00329" 3545 "s59E5H2" 0 "YGGT family protein"
"DsuD02_00329" 3545 "s63E6H2" 0 "YGGT family protein"
"DsuD02_00330" 3546 "s59E13H2" 0 "hypothetical protein"
"DsuD02_00330" 3546 "s59E25H2" 0 "hypothetical protein"
"DsuD02_00330" 3546 "s59E5H2" 0 "hypothetical protein"
"DsuD02_00330" 3546 "s63E6H2" 0 "hypothetical protein"
"DsuD02_00331" 3547 "s59E13H2" 0 "Coenzyme A biosynthesis bifunctional protein CoaBC"
"DsuD02_00331" 3547 "s59E25H2" 0 "Coenzyme A biosynthesis bifunctional protein CoaBC"
"DsuD02_00331" 3547 "s59E5H2" 0 "Coenzyme A biosynthesis bifunctional protein CoaBC"
"DsuD02_00331" 3547 "s63E6H2" 0 "Coenzyme A biosynthesis bifunctional protein CoaBC"
"DsuD02_00332" 3548 "s59E13H2" 0 "Uracil DNA glycosylase superfamily protein"
"DsuD02_00332" 3548 "s59E25H2" 0 "Uracil DNA glycosylase superfamily protein"
"DsuD02_00332" 3548 "s59E5H2" 0 "Uracil DNA glycosylase superfamily protein"
"DsuD02_00332" 3548 "s63E6H2" 0 "Uracil DNA glycosylase superfamily protein"
"DsuD02_00333" 3549 "s59E13H2" 0 "hypothetical protein"
"DsuD02_00333" 3549 "s59E25H2" 0 "hypothetical protein"
"DsuD02_00333" 3549 "s59E5H2" 0 "hypothetical protein"
"DsuD02_00333" 3549 "s63E6H2" 0 "hypothetical protein"
"DsuD02_00334" 3550 "s59E13H2" 0 "Modulator of FtsH protease HflC"
"DsuD02_00334" 3550 "s59E25H2" 0 "Modulator of FtsH protease HflC"
"DsuD02_00334" 3550 "s59E5H2" 0.005513746 "Modulator of FtsH protease HflC"
"DsuD02_00334" 3550 "s63E6H2" 0 "Modulator of FtsH protease HflC"
"DsuD02_00335" 3551 "s59E13H2" 0 "hypothetical protein"
"DsuD02_00335" 3551 "s59E25H2" 0 "hypothetical protein"
"DsuD02_00335" 3551 "s59E5H2" 0 "hypothetical protein"
"DsuD02_00335" 3551 "s63E6H2" 0 "hypothetical protein"
"DsuD02_00336" 3552 "s59E13H2" 0 "2-C-methyl-D-erythritol 4-phosphate cytidylyltransferase"
"DsuD02_00336" 3552 "s59E25H2" 0 "2-C-methyl-D-erythritol 4-phosphate cytidylyltransferase"
"DsuD02_00336" 3552 "s59E5H2" 0 "2-C-methyl-D-erythritol 4-phosphate cytidylyltransferase"
"DsuD02_00336" 3552 "s63E6H2" 0 "2-C-methyl-D-erythritol 4-phosphate cytidylyltransferase"
"DsuD02_00337" 3553 "s59E13H2" 0 "Porphobilinogen deaminase"
"DsuD02_00337" 3553 "s59E25H2" 0 "Porphobilinogen deaminase"
"DsuD02_00337" 3553 "s59E5H2" 0 "Porphobilinogen deaminase"
"DsuD02_00337" 3553 "s63E6H2" 0 "Porphobilinogen deaminase"
"DsuD02_00338" 3554 "s59E13H2" 0 "Uroporphyrinogen-III C-methyltransferase"
"DsuD02_00338" 3554 "s59E25H2" 0 "Uroporphyrinogen-III C-methyltransferase"

"DsuD02_00338" 3554 "s59E5H2" 0 "Uroporphyrinogen-III C-methyltransferase"
"DsuD02_00338" 3554 "s63E6H2" 0 "Uroporphyrinogen-III C-methyltransferase"
"DsuD02_00339" 3555 "s59E13H2" 0 "3'(2'),5'-bisphosphate nucleotidase CysQ"
"DsuD02_00339" 3555 "s59E25H2" 0 "3'(2'),5'-bisphosphate nucleotidase CysQ"
"DsuD02_00339" 3555 "s59E5H2" 0 "3'(2'),5'-bisphosphate nucleotidase CysQ"
"DsuD02_00339" 3555 "s63E6H2" 0 "3'(2'),5'-bisphosphate nucleotidase CysQ"
"DsuD02_00341" 3557 "s59E13H2" 0 "Xaa-Pro dipeptidase"
"DsuD02_00341" 3557 "s59E25H2" 0 "Xaa-Pro dipeptidase"
"DsuD02_00341" 3557 "s59E5H2" 0 "Xaa-Pro dipeptidase"
"DsuD02_00341" 3557 "s63E6H2" 0 "Xaa-Pro dipeptidase"
"DsuD02_00342" 3558 "s59E13H2" 0 "Tyrosine recombinase XerD"
"DsuD02_00342" 3558 "s59E25H2" 0 "Tyrosine recombinase XerD"
"DsuD02_00342" 3558 "s59E5H2" 0 "Tyrosine recombinase XerD"
"DsuD02_00342" 3558 "s63E6H2" 0 "Tyrosine recombinase XerD"
"DsuD02_00346" 3562 "s59E13H2" 0 "outer membrane protease"
"DsuD02_00346" 3562 "s59E25H2" 0 "outer membrane protease"
"DsuD02_00346" 3562 "s59E5H2" 0 "outer membrane protease"
"DsuD02_00346" 3562 "s63E6H2" 0 "outer membrane protease"
"DsuD02_00347" 3563 "s59E13H2" 0 "Inositol-1-monophosphatase"
"DsuD02_00347" 3563 "s59E25H2" 0 "Inositol-1-monophosphatase"
"DsuD02_00347" 3563 "s59E5H2" 0 "Inositol-1-monophosphatase"
"DsuD02_00347" 3563 "s63E6H2" 0 "Inositol-1-monophosphatase"
"DsuD02_00348" 3564 "s59E13H2" 0 "Sec-independent protein translocase protein TatAy"
"DsuD02_00348" 3564 "s59E25H2" 0 "Sec-independent protein translocase protein TatAy"
"DsuD02_00348" 3564 "s59E5H2" 0 "Sec-independent protein translocase protein TatAy"
"DsuD02_00348" 3564 "s63E6H2" 0 "Sec-independent protein translocase protein TatAy"
"DsuD02_00349" 3565 "s59E13H2" 0 "Putative ski2-type helicase"
"DsuD02_00349" 3565 "s59E25H2" 0 "Putative ski2-type helicase"
"DsuD02_00349" 3565 "s59E5H2" 0 "Putative ski2-type helicase"
"DsuD02_00349" 3565 "s63E6H2" 0 "Putative ski2-type helicase"
"DsuD02_00350" 3566 "s59E13H2" 0 "D-inositol 3-phosphate glycosyltransferase"
"DsuD02_00350" 3566 "s59E25H2" 0 "D-inositol 3-phosphate glycosyltransferase"
"DsuD02_00350" 3566 "s59E5H2" 0 "D-inositol 3-phosphate glycosyltransferase"
"DsuD02_00350" 3566 "s63E6H2" 0 "D-inositol 3-phosphate glycosyltransferase"
"DsuD02_00351" 3567 "s59E13H2" 0 "GDP-mannose-dependent alpha-(1-6)-phosphatidylinositol monomannoside mannosyltransferase"
"DsuD02_00351" 3567 "s59E25H2" 0 "GDP-mannose-dependent alpha-(1-6)-phosphatidylinositol monomannoside mannosyltransferase"
"DsuD02_00351" 3567 "s59E5H2" 0 "GDP-mannose-dependent alpha-(1-6)-phosphatidylinositol monomannoside mannosyltransferase"
"DsuD02_00351" 3567 "s63E6H2" 0 "GDP-mannose-dependent alpha-(1-6)-phosphatidylinositol monomannoside mannosyltransferase"
"DsuD02_00352" 3568 "s59E13H2" 0 "Asparagine synthetase [glutamine-hydrolyzing] 1"
"DsuD02_00352" 3568 "s59E25H2" 0 "Asparagine synthetase [glutamine-hydrolyzing] 1"
"DsuD02_00352" 3568 "s59E5H2" 0 "Asparagine synthetase [glutamine-hydrolyzing] 1"
"DsuD02_00352" 3568 "s63E6H2" 0 "Asparagine synthetase [glutamine-hydrolyzing] 1"
"DsuD02_00353" 3569 "s59E13H2" 0 "hypothetical protein"
"DsuD02_00353" 3569 "s59E25H2" 0 "hypothetical protein"
"DsuD02_00353" 3569 "s59E5H2" 0 "hypothetical protein"
"DsuD02_00353" 3569 "s63E6H2" 0 "hypothetical protein"
"DsuD02_00354" 3570 "s59E13H2" 0 "Alpha-D-kanosaminyltransferase"

"DsuD02_00354" 3570 "s59E25H2" 0 "Alpha-D-kanosaminyltransferase"
"DsuD02_00354" 3570 "s59E5H2" 0 "Alpha-D-kanosaminyltransferase"
"DsuD02_00354" 3570 "s63E6H2" 0 "Alpha-D-kanosaminyltransferase"
"DsuD02_00355" 3571 "s59E13H2" 0 "Asparagine synthetase [glutamine-hydrolyzing] 1"
"DsuD02_00355" 3571 "s59E25H2" 0 "Asparagine synthetase [glutamine-hydrolyzing] 1"
"DsuD02_00355" 3571 "s59E5H2" 0 "Asparagine synthetase [glutamine-hydrolyzing] 1"
"DsuD02_00355" 3571 "s63E6H2" 0 "Asparagine synthetase [glutamine-hydrolyzing] 1"
"DsuD02_00356" 3572 "s59E13H2" 0 "Glycosyl transferase family 2"
"DsuD02_00356" 3572 "s59E25H2" 0 "Glycosyl transferase family 2"
"DsuD02_00356" 3572 "s59E5H2" 0 "Glycosyl transferase family 2"
"DsuD02_00356" 3572 "s63E6H2" 0 "Glycosyl transferase family 2"
"DsuD02_00357" 3573 "s59E13H2" 0 "GDP-mannose-dependent alpha-(1-6)-phosphatidylinositol monomannoside mannosyltransferase"
"DsuD02_00357" 3573 "s59E25H2" 0 "GDP-mannose-dependent alpha-(1-6)-phosphatidylinositol monomannoside mannosyltransferase"
"DsuD02_00357" 3573 "s59E5H2" 0 "GDP-mannose-dependent alpha-(1-6)-phosphatidylinositol monomannoside mannosyltransferase"
"DsuD02_00357" 3573 "s63E6H2" 0 "GDP-mannose-dependent alpha-(1-6)-phosphatidylinositol monomannoside mannosyltransferase"
"DsuD02_00358" 3574 "s59E13H2" 0 "Phd_YefM"
"DsuD02_00358" 3574 "s59E25H2" 0 "Phd_YefM"
"DsuD02_00358" 3574 "s59E5H2" 0 "Phd_YefM"
"DsuD02_00358" 3574 "s63E6H2" 0 "Phd_YefM"
"DsuD02_00359" 3575 "s59E13H2" 0 "Ribonuclease VapC22"
"DsuD02_00359" 3575 "s59E25H2" 0 "Ribonuclease VapC22"
"DsuD02_00359" 3575 "s59E5H2" 0 "Ribonuclease VapC22"
"DsuD02_00359" 3575 "s63E6H2" 0 "Ribonuclease VapC22"
"DsuD02_00360" 3576 "s59E13H2" 0 "Putative glycosyltransferase EpsD"
"DsuD02_00360" 3576 "s59E25H2" 0 "Putative glycosyltransferase EpsD"
"DsuD02_00360" 3576 "s59E5H2" 0 "Putative glycosyltransferase EpsD"
"DsuD02_00360" 3576 "s63E6H2" 0 "Putative glycosyltransferase EpsD"
"DsuD02_00361" 3577 "s59E13H2" 0 "hypothetical protein"
"DsuD02_00361" 3577 "s59E25H2" 0 "hypothetical protein"
"DsuD02_00361" 3577 "s59E5H2" 0 "hypothetical protein"
"DsuD02_00361" 3577 "s63E6H2" 0 "hypothetical protein"
"DsuD02_00362" 3578 "s59E13H2" 0 "hypothetical protein"
"DsuD02_00362" 3578 "s59E25H2" 0 "hypothetical protein"
"DsuD02_00362" 3578 "s59E5H2" 0 "hypothetical protein"
"DsuD02_00362" 3578 "s63E6H2" 0 "hypothetical protein"
"DsuD02_00363" 3579 "s59E13H2" 0 "WbqC-like protein family protein"
"DsuD02_00363" 3579 "s59E25H2" 0 "WbqC-like protein family protein"
"DsuD02_00363" 3579 "s59E5H2" 0 "WbqC-like protein family protein"
"DsuD02_00363" 3579 "s63E6H2" 0 "WbqC-like protein family protein"
"DsuD02_00364" 3580 "s59E13H2" 0 "Diacetylchitobiose deacetylase"
"DsuD02_00364" 3580 "s59E25H2" 0 "Diacetylchitobiose deacetylase"
"DsuD02_00364" 3580 "s59E5H2" 0 "Diacetylchitobiose deacetylase"
"DsuD02_00364" 3580 "s63E6H2" 0 "Diacetylchitobiose deacetylase"
"DsuD02_00366" 3582 "s59E13H2" 0 "UDP-4-amino-4-deoxy-L-arabinose--oxoglutarate aminotransferase"
"DsuD02_00366" 3582 "s59E25H2" 0 "UDP-4-amino-4-deoxy-L-arabinose--oxoglutarate aminotransferase"
"DsuD02_00366" 3582 "s59E5H2" 0 "UDP-4-amino-4-deoxy-L-arabinose--oxoglutarate aminotransferase"
"DsuD02_00366" 3582 "s63E6H2" 0 "UDP-4-amino-4-deoxy-L-arabinose--oxoglutarate aminotransferase"

"DsuD02_00367" 3583 "s59E13H2" 0 "putative undecaprenyl-phosphate N-acetylglucosaminyl 1-phosphate transferase"
"DsuD02_00367" 3583 "s59E25H2" 0 "putative undecaprenyl-phosphate N-acetylglucosaminyl 1-phosphate transferase"
"DsuD02_00367" 3583 "s59E5H2" 0 "putative undecaprenyl-phosphate N-acetylglucosaminyl 1-phosphate transferase"
"DsuD02_00367" 3583 "s63E6H2" 0 "putative undecaprenyl-phosphate N-acetylglucosaminyl 1-phosphate transferase"
"DsuD02_00368" 3584 "s59E13H2" 0 "hypothetical protein"
"DsuD02_00368" 3584 "s59E25H2" 0 "hypothetical protein"
"DsuD02_00368" 3584 "s59E5H2" 0 "hypothetical protein"
"DsuD02_00368" 3584 "s63E6H2" 0 "hypothetical protein"
"DsuD02_00369" 3585 "s59E13H2" 0 "Antilisterial bacteriocin subtilosin biosynthesis protein AlbA"
"DsuD02_00369" 3585 "s59E25H2" 0 "Antilisterial bacteriocin subtilosin biosynthesis protein AlbA"
"DsuD02_00369" 3585 "s59E5H2" 0 "Antilisterial bacteriocin subtilosin biosynthesis protein AlbA"
"DsuD02_00369" 3585 "s63E6H2" 0 "Antilisterial bacteriocin subtilosin biosynthesis protein AlbA"
"DsuD02_00370" 3586 "s59E13H2" 0 "Coenzyme PQQ synthesis protein D"
"DsuD02_00370" 3586 "s59E25H2" 0 "Coenzyme PQQ synthesis protein D"
"DsuD02_00370" 3586 "s59E5H2" 0 "Coenzyme PQQ synthesis protein D"
"DsuD02_00370" 3586 "s63E6H2" 0 "Coenzyme PQQ synthesis protein D"
"DsuD02_00371" 3587 "s59E13H2" 0 "Peptidase S24-like protein"
"DsuD02_00371" 3587 "s59E25H2" 0 "Peptidase S24-like protein"
"DsuD02_00371" 3587 "s59E5H2" 0 "Peptidase S24-like protein"
"DsuD02_00371" 3587 "s63E6H2" 0 "Peptidase S24-like protein"
"DsuD02_00372" 3588 "s59E13H2" 0 "Antilisterial bacteriocin subtilosin biosynthesis protein AlbA"
"DsuD02_00372" 3588 "s59E25H2" 0 "Antilisterial bacteriocin subtilosin biosynthesis protein AlbA"
"DsuD02_00372" 3588 "s59E5H2" 0 "Antilisterial bacteriocin subtilosin biosynthesis protein AlbA"
"DsuD02_00372" 3588 "s63E6H2" 0 "Antilisterial bacteriocin subtilosin biosynthesis protein AlbA"
"DsuD02_00375" 3591 "s59E13H2" 0 "Digeranylgeranylglycerophospholipid reductase"
"DsuD02_00375" 3591 "s59E25H2" 0 "Digeranylgeranylglycerophospholipid reductase"
"DsuD02_00375" 3591 "s59E5H2" 0 "Digeranylgeranylglycerophospholipid reductase"
"DsuD02_00375" 3591 "s63E6H2" 0 "Digeranylgeranylglycerophospholipid reductase"
"DsuD02_00376" 3592 "s59E13H2" 0 "hypothetical protein"
"DsuD02_00376" 3592 "s59E25H2" 0 "hypothetical protein"
"DsuD02_00376" 3592 "s59E5H2" 0 "hypothetical protein"
"DsuD02_00376" 3592 "s63E6H2" 0 "hypothetical protein"
"DsuD02_00377" 3593 "s59E13H2" 0 "hypothetical protein"
"DsuD02_00377" 3593 "s59E25H2" 0 "hypothetical protein"
"DsuD02_00377" 3593 "s59E5H2" 0 "hypothetical protein"
"DsuD02_00377" 3593 "s63E6H2" 0 "hypothetical protein"
"DsuD02_00378" 3594 "s59E13H2" 0 "Bacillopeptidase F precursor"
"DsuD02_00378" 3594 "s59E25H2" 0 "Bacillopeptidase F precursor"
"DsuD02_00378" 3594 "s59E5H2" 0 "Bacillopeptidase F precursor"
"DsuD02_00378" 3594 "s63E6H2" 0 "Bacillopeptidase F precursor"
"DsuD02_00379" 3595 "s59E13H2" 0 "hypothetical protein"
"DsuD02_00379" 3595 "s59E25H2" 0 "hypothetical protein"
"DsuD02_00379" 3595 "s59E5H2" 0 "hypothetical protein"
"DsuD02_00379" 3595 "s63E6H2" 0 "hypothetical protein"
"DsuD02_00380" 3596 "s59E13H2" 0 "hypothetical protein"
"DsuD02_00380" 3596 "s59E25H2" 0 "hypothetical protein"
"DsuD02_00380" 3596 "s59E5H2" 0 "hypothetical protein"

"DsuD02_00380" 3596 "s63E6H2" 0 "hypothetical protein"
"DsuD02_00381" 3597 "s59E13H2" 0 "PilZ domain protein"
"DsuD02_00381" 3597 "s59E25H2" 0 "PilZ domain protein"
"DsuD02_00381" 3597 "s59E5H2" 0 "PilZ domain protein"
"DsuD02_00381" 3597 "s63E6H2" 0 "PilZ domain protein"
"DsuD02_00382" 3598 "s59E13H2" 0 "Type II secretion system protein G precursor"
"DsuD02_00382" 3598 "s59E25H2" 0 "Type II secretion system protein G precursor"
"DsuD02_00382" 3598 "s59E5H2" 0 "Type II secretion system protein G precursor"
"DsuD02_00382" 3598 "s63E6H2" 0 "Type II secretion system protein G precursor"
"DsuD02_00383" 3599 "s59E13H2" 0 "Nitrogen fixation protein VnfA"
"DsuD02_00383" 3599 "s59E25H2" 0 "Nitrogen fixation protein VnfA"
"DsuD02_00383" 3599 "s59E5H2" 0 "Nitrogen fixation protein VnfA"
"DsuD02_00383" 3599 "s63E6H2" 0 "Nitrogen fixation protein VnfA"
"DsuD02_00384" 3600 "s59E13H2" 0 "Type II secretion system protein F"
"DsuD02_00384" 3600 "s59E25H2" 0 "Type II secretion system protein F"
"DsuD02_00384" 3600 "s59E5H2" 0 "Type II secretion system protein F"
"DsuD02_00384" 3600 "s63E6H2" 0 "Type II secretion system protein F"
"DsuD02_00385" 3601 "s59E13H2" 0 "Type II secretion system protein E"
"DsuD02_00385" 3601 "s59E25H2" 0 "Type II secretion system protein E"
"DsuD02_00385" 3601 "s59E5H2" 0 "Type II secretion system protein E"
"DsuD02_00385" 3601 "s63E6H2" 0 "Type II secretion system protein E"
"DsuD02_00386" 3602 "s59E13H2" 0 "hypothetical protein"
"DsuD02_00386" 3602 "s59E25H2" 0 "hypothetical protein"
"DsuD02_00386" 3602 "s59E5H2" 0 "hypothetical protein"
"DsuD02_00386" 3602 "s63E6H2" 0 "hypothetical protein"
"DsuD02_00387" 3603 "s59E13H2" 0 "Type IV pilus biogenesis and competence protein PilQ precursor"
"DsuD02_00387" 3603 "s59E25H2" 0 "Type IV pilus biogenesis and competence protein PilQ precursor"
"DsuD02_00387" 3603 "s59E5H2" 0 "Type IV pilus biogenesis and competence protein PilQ precursor"
"DsuD02_00387" 3603 "s63E6H2" 0 "Type IV pilus biogenesis and competence protein PilQ precursor"
"DsuD02_00388" 3604 "s59E13H2" 0 "hypothetical protein"
"DsuD02_00388" 3604 "s59E25H2" 0 "hypothetical protein"
"DsuD02_00388" 3604 "s59E5H2" 0 "hypothetical protein"
"DsuD02_00388" 3604 "s63E6H2" 0 "hypothetical protein"
"DsuD02_00389" 3605 "s59E13H2" 0 "hypothetical protein"
"DsuD02_00389" 3605 "s59E25H2" 0 "hypothetical protein"
"DsuD02_00389" 3605 "s59E5H2" 0 "hypothetical protein"
"DsuD02_00389" 3605 "s63E6H2" 0 "hypothetical protein"
"DsuD02_00390" 3606 "s59E13H2" 0 "Competence protein A"
"DsuD02_00390" 3606 "s59E25H2" 0 "Competence protein A"
"DsuD02_00390" 3606 "s59E5H2" 0 "Competence protein A"
"DsuD02_00390" 3606 "s63E6H2" 0 "Competence protein A"
"DsuD02_00392" 3608 "s59E13H2" 0 "CAAX amino terminal protease self- immunity"
"DsuD02_00392" 3608 "s59E25H2" 0 "CAAX amino terminal protease self- immunity"
"DsuD02_00392" 3608 "s59E5H2" 0 "CAAX amino terminal protease self- immunity"
"DsuD02_00392" 3608 "s63E6H2" 0 "CAAX amino terminal protease self- immunity"
"DsuD02_00393" 3609 "s59E13H2" 0 "hypothetical protein"
"DsuD02_00393" 3609 "s59E25H2" 0 "hypothetical protein"

"DsuD02_00393" 3609 "s59E5H2" 0 "hypothetical protein"
"DsuD02_00393" 3609 "s63E6H2" 0 "hypothetical protein"
"DsuD02_00394" 3610 "s59E13H2" 0 "Bifunctional purine biosynthesis protein PurH"
"DsuD02_00394" 3610 "s59E25H2" 0 "Bifunctional purine biosynthesis protein PurH"
"DsuD02_00394" 3610 "s59E5H2" 0 "Bifunctional purine biosynthesis protein PurH"
"DsuD02_00394" 3610 "s63E6H2" 0 "Bifunctional purine biosynthesis protein PurH"
"DsuD02_00395" 3611 "s59E13H2" 0 "GTP cyclohydrolase FolE2"
"DsuD02_00395" 3611 "s59E25H2" 0 "GTP cyclohydrolase FolE2"
"DsuD02_00395" 3611 "s59E5H2" 0 "GTP cyclohydrolase FolE2"
"DsuD02_00395" 3611 "s63E6H2" 0 "GTP cyclohydrolase FolE2"
"DsuD02_00397" 3613 "s59E13H2" 0 "CRISPR-associated endonuclease Cas9"
"DsuD02_00397" 3613 "s59E25H2" 0 "CRISPR-associated endonuclease Cas9"
"DsuD02_00397" 3613 "s59E5H2" 0 "CRISPR-associated endonuclease Cas9"
"DsuD02_00397" 3613 "s63E6H2" 0 "CRISPR-associated endonuclease Cas9"
"DsuD02_00398" 3614 "s59E13H2" 0 "Alpha/beta hydrolase family protein"
"DsuD02_00398" 3614 "s59E25H2" 0 "Alpha/beta hydrolase family protein"
"DsuD02_00398" 3614 "s59E5H2" 0 "Alpha/beta hydrolase family protein"
"DsuD02_00398" 3614 "s63E6H2" 0 "Alpha/beta hydrolase family protein"
"DsuD02_00399" 3615 "s59E13H2" 0 "hypothetical protein"
"DsuD02_00399" 3615 "s59E25H2" 0 "hypothetical protein"
"DsuD02_00399" 3615 "s59E5H2" 0 "hypothetical protein"
"DsuD02_00399" 3615 "s63E6H2" 0 "hypothetical protein"
"DsuD02_00400" 3616 "s59E13H2" 0 "hypothetical protein"
"DsuD02_00400" 3616 "s59E25H2" 0 "hypothetical protein"
"DsuD02_00400" 3616 "s59E5H2" 0 "hypothetical protein"
"DsuD02_00400" 3616 "s63E6H2" 0 "hypothetical protein"
"DsuD02_00402" 3618 "s59E13H2" 0 "Glucose 1-dehydrogenase 1"
"DsuD02_00402" 3618 "s59E25H2" 0 "Glucose 1-dehydrogenase 1"
"DsuD02_00402" 3618 "s59E5H2" 0 "Glucose 1-dehydrogenase 1"
"DsuD02_00402" 3618 "s63E6H2" 0 "Glucose 1-dehydrogenase 1"
"DsuD02_00403" 3619 "s59E13H2" 0 "hybrid sensory kinase in two-component regulatory system with RcsB and YojN"
"DsuD02_00403" 3619 "s59E25H2" 0 "hybrid sensory kinase in two-component regulatory system with RcsB and YojN"
"DsuD02_00403" 3619 "s59E5H2" 0 "hybrid sensory kinase in two-component regulatory system with RcsB and YojN"
"DsuD02_00403" 3619 "s63E6H2" 0 "hybrid sensory kinase in two-component regulatory system with RcsB and YojN"
"DsuD02_00404" 3620 "s59E13H2" 0 "Sorbitol dehydrogenase"
"DsuD02_00404" 3620 "s59E25H2" 0 "Sorbitol dehydrogenase"
"DsuD02_00404" 3620 "s59E5H2" 0 "Sorbitol dehydrogenase"
"DsuD02_00404" 3620 "s63E6H2" 0 "Sorbitol dehydrogenase"
"DsuD02_00405" 3621 "s59E13H2" 0 "putative aldolase LsrF"
"DsuD02_00405" 3621 "s59E25H2" 0 "putative aldolase LsrF"
"DsuD02_00405" 3621 "s59E5H2" 0 "putative aldolase LsrF"
"DsuD02_00405" 3621 "s63E6H2" 0 "putative aldolase LsrF"
"DsuD02_00406" 3622 "s59E13H2" 0 "Inositol-3-phosphate synthase"
"DsuD02_00406" 3622 "s59E25H2" 0 "Inositol-3-phosphate synthase"
"DsuD02_00406" 3622 "s59E5H2" 0 "Inositol-3-phosphate synthase"
"DsuD02_00406" 3622 "s63E6H2" 0 "Inositol-3-phosphate synthase"
"DsuD02_00407" 3623 "s59E13H2" 0 "hypothetical protein"

"DsuD02_00407" 3623 "s59E25H2" 0 "hypothetical protein"
"DsuD02_00407" 3623 "s59E5H2" 0 "hypothetical protein"
"DsuD02_00407" 3623 "s63E6H2" 0 "hypothetical protein"
"DsuD02_00408" 3624 "s59E13H2" 0 "3'-5' exoribonuclease YhaM"
"DsuD02_00408" 3624 "s59E25H2" 0 "3'-5' exoribonuclease YhaM"
"DsuD02_00408" 3624 "s59E5H2" 0 "3'-5' exoribonuclease YhaM"
"DsuD02_00408" 3624 "s63E6H2" 0 "3'-5' exoribonuclease YhaM"
"DsuD02_00409" 3625 "s59E13H2" 0 "L-threonine dehydratase catabolic TdcB"
"DsuD02_00409" 3625 "s59E25H2" 0 "L-threonine dehydratase catabolic TdcB"
"DsuD02_00409" 3625 "s59E5H2" 0 "L-threonine dehydratase catabolic TdcB"
"DsuD02_00409" 3625 "s63E6H2" 0 "L-threonine dehydratase catabolic TdcB"
"DsuD02_00410" 3626 "s59E13H2" 0 "Streptothricin hydrolase"
"DsuD02_00410" 3626 "s59E25H2" 0 "Streptothricin hydrolase"
"DsuD02_00410" 3626 "s59E5H2" 0 "Streptothricin hydrolase"
"DsuD02_00410" 3626 "s63E6H2" 0 "Streptothricin hydrolase"
"DsuD02_00411" 3627 "s59E13H2" 0 "hypothetical protein"
"DsuD02_00411" 3627 "s59E25H2" 0 "hypothetical protein"
"DsuD02_00411" 3627 "s59E5H2" 0 "hypothetical protein"
"DsuD02_00411" 3627 "s63E6H2" 0 "hypothetical protein"
"DsuD02_00412" 3628 "s59E13H2" 0 "hypothetical protein"
"DsuD02_00412" 3628 "s59E25H2" 0 "hypothetical protein"
"DsuD02_00412" 3628 "s59E5H2" 0 "hypothetical protein"
"DsuD02_00412" 3628 "s63E6H2" 0 "hypothetical protein"
"DsuD02_00413" 3629 "s59E13H2" 0 "hypothetical protein"
"DsuD02_00413" 3629 "s59E25H2" 0 "hypothetical protein"
"DsuD02_00413" 3629 "s59E5H2" 0 "hypothetical protein"
"DsuD02_00413" 3629 "s63E6H2" 0 "hypothetical protein"
"DsuD02_00414" 3630 "s59E13H2" 0 "31-O-demethyl-FK506 methyltransferase FkbM"
"DsuD02_00414" 3630 "s59E25H2" 0 "31-O-demethyl-FK506 methyltransferase FkbM"
"DsuD02_00414" 3630 "s59E5H2" 0 "31-O-demethyl-FK506 methyltransferase FkbM"
"DsuD02_00414" 3630 "s63E6H2" 0 "31-O-demethyl-FK506 methyltransferase FkbM"
"DsuD02_00415" 3631 "s59E13H2" 0 "putative hydrolase YxeP"
"DsuD02_00415" 3631 "s59E25H2" 0 "putative hydrolase YxeP"
"DsuD02_00415" 3631 "s59E5H2" 0 "putative hydrolase YxeP"
"DsuD02_00415" 3631 "s63E6H2" 0 "putative hydrolase YxeP"
"DsuD02_00416" 3632 "s59E13H2" 0 "hypothetical protein"
"DsuD02_00416" 3632 "s59E25H2" 0 "hypothetical protein"
"DsuD02_00416" 3632 "s59E5H2" 0 "hypothetical protein"
"DsuD02_00416" 3632 "s63E6H2" 0 "hypothetical protein"
"DsuD02_00417" 3633 "s59E13H2" 0 "General stress protein 69"
"DsuD02_00417" 3633 "s59E25H2" 0 "General stress protein 69"
"DsuD02_00417" 3633 "s59E5H2" 0 "General stress protein 69"
"DsuD02_00417" 3633 "s63E6H2" 0 "General stress protein 69"
"DsuD02_00418" 3634 "s59E13H2" 0 "Rhomboid protease GluP"
"DsuD02_00418" 3634 "s59E25H2" 0 "Rhomboid protease GluP"
"DsuD02_00418" 3634 "s59E5H2" 0 "Rhomboid protease GluP"
"DsuD02_00418" 3634 "s63E6H2" 0 "Rhomboid protease GluP"

"DsuD02_00419" 3635 "s59E13H2" 0 "Prolipoprotein diacylglyceryl transferase"
"DsuD02_00419" 3635 "s59E25H2" 0 "Prolipoprotein diacylglyceryl transferase"
"DsuD02_00419" 3635 "s59E5H2" 0 "Prolipoprotein diacylglyceryl transferase"
"DsuD02_00419" 3635 "s63E6H2" 0 "Prolipoprotein diacylglyceryl transferase"
"DsuD02_00420" 3636 "s59E13H2" 0 "L-carnitine dehydrogenase"
"DsuD02_00420" 3636 "s59E25H2" 0 "L-carnitine dehydrogenase"
"DsuD02_00420" 3636 "s59E5H2" 0 "L-carnitine dehydrogenase"
"DsuD02_00420" 3636 "s63E6H2" 0 "L-carnitine dehydrogenase"
"DsuD02_00425" 3641 "s59E13H2" 0 "Diaminopropionate ammonia-lyase"
"DsuD02_00425" 3641 "s59E25H2" 0 "Diaminopropionate ammonia-lyase"
"DsuD02_00425" 3641 "s59E5H2" 0 "Diaminopropionate ammonia-lyase"
"DsuD02_00425" 3641 "s63E6H2" 0 "Diaminopropionate ammonia-lyase"
"DsuD02_00426" 3642 "s59E13H2" 0 "shikimate kinase"
"DsuD02_00426" 3642 "s59E25H2" 0 "shikimate kinase"
"DsuD02_00426" 3642 "s59E5H2" 0 "shikimate kinase"
"DsuD02_00426" 3642 "s63E6H2" 0 "shikimate kinase"
"DsuD02_00427" 3643 "s59E13H2" 0 "hypothetical protein"
"DsuD02_00427" 3643 "s59E25H2" 0 "hypothetical protein"
"DsuD02_00427" 3643 "s59E5H2" 0 "hypothetical protein"
"DsuD02_00427" 3643 "s63E6H2" 0 "hypothetical protein"
"DsuD02_00428" 3644 "s59E13H2" 0 "Prolyl-tRNA editing protein ProX"
"DsuD02_00428" 3644 "s59E25H2" 0 "Prolyl-tRNA editing protein ProX"
"DsuD02_00428" 3644 "s59E5H2" 0 "Prolyl-tRNA editing protein ProX"
"DsuD02_00428" 3644 "s63E6H2" 0 "Prolyl-tRNA editing protein ProX"
"DsuD02_00429" 3645 "s59E13H2" 0 "Glutamyl-tRNA(Gln) amidotransferase subunit A"
"DsuD02_00429" 3645 "s59E25H2" 0 "Glutamyl-tRNA(Gln) amidotransferase subunit A"
"DsuD02_00429" 3645 "s59E5H2" 0 "Glutamyl-tRNA(Gln) amidotransferase subunit A"
"DsuD02_00429" 3645 "s63E6H2" 0 "Glutamyl-tRNA(Gln) amidotransferase subunit A"
"DsuD02_00430" 3646 "s59E13H2" 0 "hypothetical protein"
"DsuD02_00430" 3646 "s59E25H2" 0 "hypothetical protein"
"DsuD02_00430" 3646 "s59E5H2" 0 "hypothetical protein"
"DsuD02_00430" 3646 "s63E6H2" 0 "hypothetical protein"
"DsuD02_00431" 3647 "s59E13H2" 0 "putative periplasmic serine endoprotease DegP-like precursor"
"DsuD02_00431" 3647 "s59E25H2" 0 "putative periplasmic serine endoprotease DegP-like precursor"
"DsuD02_00431" 3647 "s59E5H2" 0 "putative periplasmic serine endoprotease DegP-like precursor"
"DsuD02_00431" 3647 "s63E6H2" 0 "putative periplasmic serine endoprotease DegP-like precursor"
"DsuD02_00432" 3648 "s59E13H2" 0 "DNA-3-methyladenine glycosylase 1"
"DsuD02_00432" 3648 "s59E25H2" 0 "DNA-3-methyladenine glycosylase 1"
"DsuD02_00432" 3648 "s59E5H2" 0 "DNA-3-methyladenine glycosylase 1"
"DsuD02_00432" 3648 "s63E6H2" 0 "DNA-3-methyladenine glycosylase 1"
"DsuD02_00433" 3649 "s59E13H2" 0 "Glutamate dehydrogenase"
"DsuD02_00433" 3649 "s59E25H2" 0 "Glutamate dehydrogenase"
"DsuD02_00433" 3649 "s59E5H2" 0 "Glutamate dehydrogenase"
"DsuD02_00433" 3649 "s63E6H2" 0 "Glutamate dehydrogenase"
"DsuD02_00436" 3652 "s59E13H2" 0 "Phosphoglycolate phosphatase"
"DsuD02_00436" 3652 "s59E25H2" 0 "Phosphoglycolate phosphatase"
"DsuD02_00436" 3652 "s59E5H2" 0 "Phosphoglycolate phosphatase"

"DsuD02_00436" 3652 "s63E6H2" 0 "Phosphoglycolate phosphatase"
"DsuD02_00438" 3654 "s59E13H2" 0 "FecR protein"
"DsuD02_00438" 3654 "s59E25H2" 0 "FecR protein"
"DsuD02_00438" 3654 "s59E5H2" 0 "FecR protein"
"DsuD02_00438" 3654 "s63E6H2" 0 "FecR protein"
"DsuD02_00439" 3655 "s59E13H2" 0 "Adenylate cyclase 2"
"DsuD02_00439" 3655 "s59E25H2" 0 "Adenylate cyclase 2"
"DsuD02_00439" 3655 "s59E5H2" 0 "Adenylate cyclase 2"
"DsuD02_00439" 3655 "s63E6H2" 0 "Adenylate cyclase 2"
"DsuD02_00440" 3656 "s59E13H2" 0 "Sulfide dehydrogenase subunit alpha precursor"
"DsuD02_00440" 3656 "s59E25H2" 0 "Sulfide dehydrogenase subunit alpha precursor"
"DsuD02_00440" 3656 "s59E5H2" 0 "Sulfide dehydrogenase subunit alpha precursor"
"DsuD02_00440" 3656 "s63E6H2" 0 "Sulfide dehydrogenase subunit alpha precursor"
"DsuD02_00442" 3658 "s59E13H2" 0 "Altronate dehydratase"
"DsuD02_00442" 3658 "s59E25H2" 0 "Altronate dehydratase"
"DsuD02_00442" 3658 "s59E5H2" 0 "Altronate dehydratase"
"DsuD02_00442" 3658 "s63E6H2" 0 "Altronate dehydratase"
"DsuD02_00443" 3659 "s59E13H2" 0 "4-hydroxybutyryl-CoA dehydratase/vinylacetyl-CoA-Delta-isomerase"
"DsuD02_00443" 3659 "s59E25H2" 0 "4-hydroxybutyryl-CoA dehydratase/vinylacetyl-CoA-Delta-isomerase"
"DsuD02_00443" 3659 "s59E5H2" 0 "4-hydroxybutyryl-CoA dehydratase/vinylacetyl-CoA-Delta-isomerase"
"DsuD02_00443" 3659 "s63E6H2" 0 "4-hydroxybutyryl-CoA dehydratase/vinylacetyl-CoA-Delta-isomerase"
"DsuD02_00444" 3660 "s59E13H2" 0 "hypothetical protein"
"DsuD02_00444" 3660 "s59E25H2" 0 "hypothetical protein"
"DsuD02_00444" 3660 "s59E5H2" 0 "hypothetical protein"
"DsuD02_00444" 3660 "s63E6H2" 0 "hypothetical protein"
"DsuD02_00445" 3661 "s59E13H2" 0 "Phosphate acyltransferase"
"DsuD02_00445" 3661 "s59E25H2" 0 "Phosphate acyltransferase"
"DsuD02_00445" 3661 "s59E5H2" 0 "Phosphate acyltransferase"
"DsuD02_00445" 3661 "s63E6H2" 0 "Phosphate acyltransferase"
"DsuD02_00446" 3662 "s59E13H2" 0 "3-oxoacyl-[acyl-carrier-protein] reductase FabG"
"DsuD02_00446" 3662 "s59E25H2" 0 "3-oxoacyl-[acyl-carrier-protein] reductase FabG"
"DsuD02_00446" 3662 "s59E5H2" 0 "3-oxoacyl-[acyl-carrier-protein] reductase FabG"
"DsuD02_00446" 3662 "s63E6H2" 0 "3-oxoacyl-[acyl-carrier-protein] reductase FabG"
"DsuD02_00447" 3663 "s59E13H2" 0 "Acyl carrier protein"
"DsuD02_00447" 3663 "s59E25H2" 0 "Acyl carrier protein"
"DsuD02_00447" 3663 "s59E5H2" 0 "Acyl carrier protein"
"DsuD02_00447" 3663 "s63E6H2" 0 "Acyl carrier protein"
"DsuD02_00448" 3664 "s59E13H2" 0 "3-oxoacyl-[acyl-carrier-protein] synthase 2"
"DsuD02_00448" 3664 "s59E25H2" 0 "3-oxoacyl-[acyl-carrier-protein] synthase 2"
"DsuD02_00448" 3664 "s59E5H2" 0 "3-oxoacyl-[acyl-carrier-protein] synthase 2"
"DsuD02_00448" 3664 "s63E6H2" 0 "3-oxoacyl-[acyl-carrier-protein] synthase 2"
"DsuD02_00449" 3665 "s59E13H2" 0 "Ribose-5-phosphate isomerase B"
"DsuD02_00449" 3665 "s59E25H2" 0 "Ribose-5-phosphate isomerase B"
"DsuD02_00449" 3665 "s59E5H2" 0 "Ribose-5-phosphate isomerase B"
"DsuD02_00449" 3665 "s63E6H2" 0 "Ribose-5-phosphate isomerase B"
"DsuD02_00450" 3666 "s59E13H2" 0 "tRNA-specific adenosine deaminase"
"DsuD02_00450" 3666 "s59E25H2" 0 "tRNA-specific adenosine deaminase"

"DsuD02_00450" 3666 "s59E5H2" 0 "tRNA-specific adenosine deaminase"
"DsuD02_00450" 3666 "s63E6H2" 0 "tRNA-specific adenosine deaminase"
"DsuD02_00451" 3667 "s59E13H2" 0 "Transcriptional repressor NrdR"
"DsuD02_00451" 3667 "s59E25H2" 0 "Transcriptional repressor NrdR"
"DsuD02_00451" 3667 "s59E5H2" 0 "Transcriptional repressor NrdR"
"DsuD02_00451" 3667 "s63E6H2" 0 "Transcriptional repressor NrdR"
"DsuD02_00452" 3668 "s59E13H2" 0 "Riboflavin biosynthesis protein RibD"
"DsuD02_00452" 3668 "s59E25H2" 0 "Riboflavin biosynthesis protein RibD"
"DsuD02_00452" 3668 "s59E5H2" 0 "Riboflavin biosynthesis protein RibD"
"DsuD02_00452" 3668 "s63E6H2" 0 "Riboflavin biosynthesis protein RibD"
"DsuD02_00454" 3670 "s59E13H2" 0 "Riboflavin synthase"
"DsuD02_00454" 3670 "s59E25H2" 0 "Riboflavin synthase"
"DsuD02_00454" 3670 "s59E5H2" 0 "Riboflavin synthase"
"DsuD02_00454" 3670 "s63E6H2" 0 "Riboflavin synthase"
"DsuD02_00455" 3671 "s59E13H2" 0 "Riboflavin biosynthesis protein RibBA"
"DsuD02_00455" 3671 "s59E25H2" 0 "Riboflavin biosynthesis protein RibBA"
"DsuD02_00455" 3671 "s59E5H2" 0 "Riboflavin biosynthesis protein RibBA"
"DsuD02_00455" 3671 "s63E6H2" 0 "Riboflavin biosynthesis protein RibBA"
"DsuD02_00456" 3672 "s59E13H2" 0 "6,7-dimethyl-8-ribityllumazine synthase"
"DsuD02_00456" 3672 "s59E25H2" 0 "6,7-dimethyl-8-ribityllumazine synthase"
"DsuD02_00456" 3672 "s59E5H2" 0 "6,7-dimethyl-8-ribityllumazine synthase"
"DsuD02_00456" 3672 "s63E6H2" 0 "6,7-dimethyl-8-ribityllumazine synthase"
"DsuD02_00457" 3673 "s59E13H2" 0 "hypothetical protein"
"DsuD02_00457" 3673 "s59E25H2" 0 "hypothetical protein"
"DsuD02_00457" 3673 "s59E5H2" 0 "hypothetical protein"
"DsuD02_00457" 3673 "s63E6H2" 0 "hypothetical protein"
"DsuD02_00458" 3674 "s59E13H2" 0 "hypothetical protein"
"DsuD02_00458" 3674 "s59E25H2" 0 "hypothetical protein"
"DsuD02_00458" 3674 "s59E5H2" 0 "hypothetical protein"
"DsuD02_00458" 3674 "s63E6H2" 0 "hypothetical protein"
"DsuD02_00459" 3675 "s59E13H2" 0 "Leucine--tRNA ligase"
"DsuD02_00459" 3675 "s59E25H2" 0 "Leucine--tRNA ligase"
"DsuD02_00459" 3675 "s59E5H2" 0 "Leucine--tRNA ligase"
"DsuD02_00459" 3675 "s63E6H2" 0 "Leucine--tRNA ligase"
"DsuD02_00460" 3676 "s59E13H2" 0 "hypothetical protein"
"DsuD02_00460" 3676 "s59E25H2" 0 "hypothetical protein"
"DsuD02_00460" 3676 "s59E5H2" 0 "hypothetical protein"
"DsuD02_00460" 3676 "s63E6H2" 0 "hypothetical protein"
"DsuD02_00461" 3677 "s59E13H2" 0 "DNA polymerase III subunit delta"
"DsuD02_00461" 3677 "s59E25H2" 0 "DNA polymerase III subunit delta"
"DsuD02_00461" 3677 "s59E5H2" 0 "DNA polymerase III subunit delta"
"DsuD02_00461" 3677 "s63E6H2" 0 "DNA polymerase III subunit delta"
"DsuD02_00462" 3678 "s59E13H2" 0 "30S ribosomal protein S20"
"DsuD02_00462" 3678 "s59E25H2" 0 "30S ribosomal protein S20"
"DsuD02_00462" 3678 "s59E5H2" 0 "30S ribosomal protein S20"
"DsuD02_00462" 3678 "s63E6H2" 0 "30S ribosomal protein S20"
"DsuD02_00463" 3679 "s59E13H2" 0 "putative peptidoglycan biosynthesis protein MurJ"

"DsuD02_00463" 3679 "s59E25H2" 0 "putative peptidoglycan biosynthesis protein MurJ"
"DsuD02_00463" 3679 "s59E5H2" 0 "putative peptidoglycan biosynthesis protein MurJ"
"DsuD02_00463" 3679 "s63E6H2" 0 "putative peptidoglycan biosynthesis protein MurJ"
"DsuD02_00465" 3681 "s59E13H2" 0 "Transketolase"
"DsuD02_00465" 3681 "s59E25H2" 0 "Transketolase"
"DsuD02_00465" 3681 "s59E5H2" 0 "Transketolase"
"DsuD02_00465" 3681 "s63E6H2" 0 "Transketolase"
"DsuD02_00466" 3682 "s59E13H2" 0 "putative ATPase of the ABC class"
"DsuD02_00466" 3682 "s59E25H2" 0 "putative ATPase of the ABC class"
"DsuD02_00466" 3682 "s59E5H2" 0 "putative ATPase of the ABC class"
"DsuD02_00466" 3682 "s63E6H2" 0 "putative ATPase of the ABC class"
"DsuD02_00467" 3683 "s59E13H2" 0 "hypothetical protein"
"DsuD02_00467" 3683 "s59E25H2" 0 "hypothetical protein"
"DsuD02_00467" 3683 "s59E5H2" 0 "hypothetical protein"
"DsuD02_00467" 3683 "s63E6H2" 0 "hypothetical protein"
"DsuD02_00468" 3684 "s59E13H2" 0 "tRNA(fMet)-specific endonuclease VapC"
"DsuD02_00468" 3684 "s59E25H2" 0 "tRNA(fMet)-specific endonuclease VapC"
"DsuD02_00468" 3684 "s59E5H2" 0 "tRNA(fMet)-specific endonuclease VapC"
"DsuD02_00468" 3684 "s63E6H2" 0 "tRNA(fMet)-specific endonuclease VapC"
"DsuD02_00470" 3686 "s59E13H2" 0 "hypothetical protein"
"DsuD02_00470" 3686 "s59E25H2" 0 "hypothetical protein"
"DsuD02_00470" 3686 "s59E5H2" 0 "hypothetical protein"
"DsuD02_00470" 3686 "s63E6H2" 0 "hypothetical protein"
"DsuD02_00471" 3687 "s59E13H2" 0 "Oxygen sensor histidine kinase NreB"
"DsuD02_00471" 3687 "s59E25H2" 0 "Oxygen sensor histidine kinase NreB"
"DsuD02_00471" 3687 "s59E5H2" 0 "Oxygen sensor histidine kinase NreB"
"DsuD02_00471" 3687 "s63E6H2" 0 "Oxygen sensor histidine kinase NreB"
"DsuD02_00473" 3689 "s59E13H2" 0 "Response regulator UvrY"
"DsuD02_00473" 3689 "s59E25H2" 0 "Response regulator UvrY"
"DsuD02_00473" 3689 "s59E5H2" 0 "Response regulator UvrY"
"DsuD02_00473" 3689 "s63E6H2" 0 "Response regulator UvrY"
"DsuD02_00474" 3690 "s59E13H2" 0 "hypothetical protein"
"DsuD02_00474" 3690 "s59E25H2" 0 "hypothetical protein"
"DsuD02_00474" 3690 "s59E5H2" 0 "hypothetical protein"
"DsuD02_00474" 3690 "s63E6H2" 0 "hypothetical protein"
"DsuD02_00475" 3691 "s59E13H2" 0 "Transcriptional regulatory protein DevR (DosR)"
"DsuD02_00475" 3691 "s59E25H2" 0 "Transcriptional regulatory protein DevR (DosR)"
"DsuD02_00475" 3691 "s59E5H2" 0 "Transcriptional regulatory protein DevR (DosR)"
"DsuD02_00475" 3691 "s63E6H2" 0 "Transcriptional regulatory protein DevR (DosR)"
"DsuD02_00476" 3692 "s59E13H2" 0 "Cyclic di-GMP phosphodiesterase response regulator RpfG"
"DsuD02_00476" 3692 "s59E25H2" 0 "Cyclic di-GMP phosphodiesterase response regulator RpfG"
"DsuD02_00476" 3692 "s59E5H2" 0 "Cyclic di-GMP phosphodiesterase response regulator RpfG"
"DsuD02_00476" 3692 "s63E6H2" 0 "Cyclic di-GMP phosphodiesterase response regulator RpfG"
"DsuD02_00477" 3693 "s59E13H2" 0 "HEPN domain protein"
"DsuD02_00477" 3693 "s59E25H2" 0 "HEPN domain protein"
"DsuD02_00477" 3693 "s59E5H2" 0 "HEPN domain protein"
"DsuD02_00477" 3693 "s63E6H2" 0 "HEPN domain protein"

"DsuD02_00478" 3694 "s59E13H2" 0 "Nucleotidyltransferase domain protein"
"DsuD02_00478" 3694 "s59E25H2" 0 "Nucleotidyltransferase domain protein"
"DsuD02_00478" 3694 "s59E5H2" 0 "Nucleotidyltransferase domain protein"
"DsuD02_00478" 3694 "s63E6H2" 0 "Nucleotidyltransferase domain protein"
"DsuD02_00479" 3695 "s59E13H2" 0 "hypothetical protein"
"DsuD02_00479" 3695 "s59E25H2" 0 "hypothetical protein"
"DsuD02_00479" 3695 "s59E5H2" 0 "hypothetical protein"
"DsuD02_00479" 3695 "s63E6H2" 0 "hypothetical protein"
"DsuD02_00480" 3696 "s59E13H2" 0 "Nucleotidyltransferase domain protein"
"DsuD02_00480" 3696 "s59E25H2" 0 "Nucleotidyltransferase domain protein"
"DsuD02_00480" 3696 "s59E5H2" 0 "Nucleotidyltransferase domain protein"
"DsuD02_00480" 3696 "s63E6H2" 0 "Nucleotidyltransferase domain protein"
"DsuD02_00481" 3697 "s59E13H2" 0 "hypothetical protein"
"DsuD02_00481" 3697 "s59E25H2" 0 "hypothetical protein"
"DsuD02_00481" 3697 "s59E5H2" 0 "hypothetical protein"
"DsuD02_00481" 3697 "s63E6H2" 0 "hypothetical protein"
"DsuD02_00484" 3700 "s59E13H2" 0 "hypothetical protein"
"DsuD02_00484" 3700 "s59E25H2" 0 "hypothetical protein"
"DsuD02_00484" 3700 "s59E5H2" 0 "hypothetical protein"
"DsuD02_00484" 3700 "s63E6H2" 0 "hypothetical protein"
"DsuD02_00485" 3701 "s59E13H2" 0 "hypothetical protein"
"DsuD02_00485" 3701 "s59E25H2" 0 "hypothetical protein"
"DsuD02_00485" 3701 "s59E5H2" 0 "hypothetical protein"
"DsuD02_00485" 3701 "s63E6H2" 0 "hypothetical protein"
"DsuD02_00486" 3702 "s59E13H2" 0 "Archaeal ATPase"
"DsuD02_00486" 3702 "s59E25H2" 0 "Archaeal ATPase"
"DsuD02_00486" 3702 "s59E5H2" 0 "Archaeal ATPase"
"DsuD02_00486" 3702 "s63E6H2" 0 "Archaeal ATPase"
"DsuD02_00487" 3703 "s59E13H2" 0 "Nucleotidyltransferase domain protein"
"DsuD02_00487" 3703 "s59E25H2" 0 "Nucleotidyltransferase domain protein"
"DsuD02_00487" 3703 "s59E5H2" 0 "Nucleotidyltransferase domain protein"
"DsuD02_00487" 3703 "s63E6H2" 0 "Nucleotidyltransferase domain protein"
"DsuD02_00488" 3704 "s59E13H2" 0 "HEPN domain protein"
"DsuD02_00488" 3704 "s59E25H2" 0 "HEPN domain protein"
"DsuD02_00488" 3704 "s59E5H2" 0 "HEPN domain protein"
"DsuD02_00488" 3704 "s63E6H2" 0 "HEPN domain protein"
"DsuD02_00489" 3705 "s59E13H2" 0 "hypothetical protein"
"DsuD02_00489" 3705 "s59E25H2" 0 "hypothetical protein"
"DsuD02_00489" 3705 "s59E5H2" 0 "hypothetical protein"
"DsuD02_00489" 3705 "s63E6H2" 0 "hypothetical protein"
"DsuD02_00493" 3709 "s59E13H2" 0 "Serine/threonine-protein kinase pkn1"
"DsuD02_00493" 3709 "s59E25H2" 0 "Serine/threonine-protein kinase pkn1"
"DsuD02_00493" 3709 "s59E5H2" 0 "Serine/threonine-protein kinase pkn1"
"DsuD02_00493" 3709 "s63E6H2" 0 "Serine/threonine-protein kinase pkn1"
"DsuD02_00494" 3710 "s59E13H2" 0 "Phosphatidylinositol mannoside acyltransferase"
"DsuD02_00494" 3710 "s59E25H2" 0 "Phosphatidylinositol mannoside acyltransferase"
"DsuD02_00494" 3710 "s59E5H2" 0 "Phosphatidylinositol mannoside acyltransferase"

"DsuD02_00494" 3710 "s63E6H2" 0 "Phosphatidylinositol mannoside acyltransferase"
"DsuD02_00495" 3711 "s59E13H2" 0 "GYD domain protein"
"DsuD02_00495" 3711 "s59E25H2" 0 "GYD domain protein"
"DsuD02_00495" 3711 "s59E5H2" 0 "GYD domain protein"
"DsuD02_00495" 3711 "s63E6H2" 0 "GYD domain protein"
"DsuD02_00497" 3713 "s59E13H2" 0 "hypothetical protein"
"DsuD02_00497" 3713 "s59E25H2" 0 "hypothetical protein"
"DsuD02_00497" 3713 "s59E5H2" 0.1856084 "hypothetical protein"
"DsuD02_00497" 3713 "s63E6H2" 0 "hypothetical protein"
"DsuD02_00498" 3714 "s59E13H2" 0 "Ribonucleoside-diphosphate reductase NrdZ"
"DsuD02_00498" 3714 "s59E25H2" 0 "Ribonucleoside-diphosphate reductase NrdZ"
"DsuD02_00498" 3714 "s59E5H2" 0.002820255 "Ribonucleoside-diphosphate reductase NrdZ"
"DsuD02_00498" 3714 "s63E6H2" 0 "Ribonucleoside-diphosphate reductase NrdZ"
"DsuD02_00499" 3715 "s59E13H2" 0 "Flagellar brake protein YcgR"
"DsuD02_00499" 3715 "s59E25H2" 0 "Flagellar brake protein YcgR"
"DsuD02_00499" 3715 "s59E5H2" 0 "Flagellar brake protein YcgR"
"DsuD02_00499" 3715 "s63E6H2" 0 "Flagellar brake protein YcgR"
"DsuD02_00500" 3716 "s59E13H2" 0 "putative glutamate synthase (NADPH) small subunit"
"DsuD02_00500" 3716 "s59E25H2" 0 "putative glutamate synthase (NADPH) small subunit"
"DsuD02_00500" 3716 "s59E5H2" 0 "putative glutamate synthase (NADPH) small subunit"
"DsuD02_00500" 3716 "s63E6H2" 0 "putative glutamate synthase (NADPH) small subunit"
"DsuD02_00501" 3717 "s59E13H2" 0 "N5-carboxyaminoimidazole ribonucleotide mutase"
"DsuD02_00501" 3717 "s59E25H2" 0 "N5-carboxyaminoimidazole ribonucleotide mutase"
"DsuD02_00501" 3717 "s59E5H2" 0 "N5-carboxyaminoimidazole ribonucleotide mutase"
"DsuD02_00501" 3717 "s63E6H2" 0 "N5-carboxyaminoimidazole ribonucleotide mutase"
"DsuD02_00502" 3718 "s59E13H2" 0 "coproporphyrinogen III oxidase"
"DsuD02_00502" 3718 "s59E25H2" 0 "coproporphyrinogen III oxidase"
"DsuD02_00502" 3718 "s59E5H2" 0 "coproporphyrinogen III oxidase"
"DsuD02_00502" 3718 "s63E6H2" 0 "coproporphyrinogen III oxidase"
"DsuD02_00503" 3719 "s59E13H2" 0 "D-tyrosyl-tRNA(Tyr) deacylase"
"DsuD02_00503" 3719 "s59E25H2" 0 "D-tyrosyl-tRNA(Tyr) deacylase"
"DsuD02_00503" 3719 "s59E5H2" 0 "D-tyrosyl-tRNA(Tyr) deacylase"
"DsuD02_00503" 3719 "s63E6H2" 0 "D-tyrosyl-tRNA(Tyr) deacylase"
"DsuD02_00504" 3720 "s59E13H2" 0 "hypothetical protein"
"DsuD02_00504" 3720 "s59E25H2" 0 "hypothetical protein"
"DsuD02_00504" 3720 "s59E5H2" 0 "hypothetical protein"
"DsuD02_00504" 3720 "s63E6H2" 0 "hypothetical protein"
"DsuD02_00505" 3721 "s59E13H2" 0 "putative phospholipid ABC transporter-binding protein MlaD"
"DsuD02_00505" 3721 "s59E25H2" 0 "putative phospholipid ABC transporter-binding protein MlaD"
"DsuD02_00505" 3721 "s59E5H2" 0 "putative phospholipid ABC transporter-binding protein MlaD"
"DsuD02_00505" 3721 "s63E6H2" 0 "putative phospholipid ABC transporter-binding protein MlaD"
"DsuD02_00506" 3722 "s59E13H2" 0 "putative ABC transporter ATP-binding protein"
"DsuD02_00506" 3722 "s59E25H2" 0 "putative ABC transporter ATP-binding protein"
"DsuD02_00506" 3722 "s59E5H2" 0 "putative ABC transporter ATP-binding protein"
"DsuD02_00506" 3722 "s63E6H2" 0 "putative ABC transporter ATP-binding protein"
"DsuD02_00507" 3723 "s59E13H2" 0 "putative phospholipid ABC transporter permease protein MlaE"
"DsuD02_00507" 3723 "s59E25H2" 0 "putative phospholipid ABC transporter permease protein MlaE"

"DsuD02_00507" 3723 "s59E5H2" 0 "putative phospholipid ABC transporter permease protein MlaE"
"DsuD02_00507" 3723 "s63E6H2" 0 "putative phospholipid ABC transporter permease protein MlaE"
"DsuD02_00508" 3724 "s59E13H2" 0 "Adenylate cyclase 1"
"DsuD02_00508" 3724 "s59E25H2" 0 "Adenylate cyclase 1"
"DsuD02_00508" 3724 "s59E5H2" 0 "Adenylate cyclase 1"
"DsuD02_00508" 3724 "s63E6H2" 0 "Adenylate cyclase 1"
"DsuD02_00509" 3725 "s59E13H2" 0 "Hydroxymethylpyrimidine/phosphomethylpyrimidine kinase"
"DsuD02_00509" 3725 "s59E25H2" 0 "Hydroxymethylpyrimidine/phosphomethylpyrimidine kinase"
"DsuD02_00509" 3725 "s59E5H2" 0 "Hydroxymethylpyrimidine/phosphomethylpyrimidine kinase"
"DsuD02_00509" 3725 "s63E6H2" 0 "Hydroxymethylpyrimidine/phosphomethylpyrimidine kinase"
"DsuD02_00511" 3727 "s59E13H2" 0 "Phosphomethylpyrimidine synthase"
"DsuD02_00511" 3727 "s59E25H2" 0 "Phosphomethylpyrimidine synthase"
"DsuD02_00511" 3727 "s59E5H2" 0 "Phosphomethylpyrimidine synthase"
"DsuD02_00511" 3727 "s63E6H2" 0 "Phosphomethylpyrimidine synthase"
"DsuD02_00512" 3728 "s59E13H2" 0 "Glucosyl-3-phosphoglycerate/mannosyl-3-phosphoglycerate phosphatase"
"DsuD02_00512" 3728 "s59E25H2" 0 "Glucosyl-3-phosphoglycerate/mannosyl-3-phosphoglycerate phosphatase"
"DsuD02_00512" 3728 "s59E5H2" 0 "Glucosyl-3-phosphoglycerate/mannosyl-3-phosphoglycerate phosphatase"
"DsuD02_00512" 3728 "s63E6H2" 0 "Glucosyl-3-phosphoglycerate/mannosyl-3-phosphoglycerate phosphatase"
"DsuD02_00513" 3729 "s59E13H2" 0 "Methylthioribose-1-phosphate isomerase"
"DsuD02_00513" 3729 "s59E25H2" 0 "Methylthioribose-1-phosphate isomerase"
"DsuD02_00513" 3729 "s59E5H2" 0 "Methylthioribose-1-phosphate isomerase"
"DsuD02_00513" 3729 "s63E6H2" 0 "Methylthioribose-1-phosphate isomerase"
"DsuD02_00514" 3730 "s59E13H2" 0 "Ribonuclease VapC11"
"DsuD02_00514" 3730 "s59E25H2" 0 "Ribonuclease VapC11"
"DsuD02_00514" 3730 "s59E5H2" 0 "Ribonuclease VapC11"
"DsuD02_00514" 3730 "s63E6H2" 0 "Ribonuclease VapC11"
"DsuD02_00515" 3731 "s59E13H2" 0 "hypothetical protein"
"DsuD02_00515" 3731 "s59E25H2" 0 "hypothetical protein"
"DsuD02_00515" 3731 "s59E5H2" 0 "hypothetical protein"
"DsuD02_00515" 3731 "s63E6H2" 0 "hypothetical protein"
"DsuD02_00516" 3732 "s59E13H2" 0 "Glutamate-1-semialdehyde 2,1-aminomutase"
"DsuD02_00516" 3732 "s59E25H2" 0 "Glutamate-1-semialdehyde 2,1-aminomutase"
"DsuD02_00516" 3732 "s59E5H2" 0 "Glutamate-1-semialdehyde 2,1-aminomutase"
"DsuD02_00516" 3732 "s63E6H2" 0 "Glutamate-1-semialdehyde 2,1-aminomutase"
"DsuD02_00518" 3734 "s59E13H2" 0 "Pyruvate synthase subunit PorD"
"DsuD02_00518" 3734 "s59E25H2" 0 "Pyruvate synthase subunit PorD"
"DsuD02_00518" 3734 "s59E5H2" 0 "Pyruvate synthase subunit PorD"
"DsuD02_00518" 3734 "s63E6H2" 0 "Pyruvate synthase subunit PorD"
"DsuD02_00519" 3735 "s59E13H2" 0 "Adenylate cyclase 2"
"DsuD02_00519" 3735 "s59E25H2" 0 "Adenylate cyclase 2"
"DsuD02_00519" 3735 "s59E5H2" 0 "Adenylate cyclase 2"
"DsuD02_00519" 3735 "s63E6H2" 0 "Adenylate cyclase 2"
"DsuD02_00521" 3737 "s59E13H2" 0 "Phytochrome-like protein cph1"
"DsuD02_00521" 3737 "s59E25H2" 0 "Phytochrome-like protein cph1"
"DsuD02_00521" 3737 "s59E5H2" 0 "Phytochrome-like protein cph1"
"DsuD02_00521" 3737 "s63E6H2" 0 "Phytochrome-like protein cph1"
"DsuD02_00522" 3738 "s59E13H2" 0 "HicB family protein"

"DsuD02_00522" 3738 "s59E25H2" 0 "HicB family protein"
"DsuD02_00522" 3738 "s59E5H2" 0 "HicB family protein"
"DsuD02_00522" 3738 "s63E6H2" 0 "HicB family protein"
"DsuD02_00523" 3739 "s59E13H2" 0 "hypothetical protein"
"DsuD02_00523" 3739 "s59E25H2" 0 "hypothetical protein"
"DsuD02_00523" 3739 "s59E5H2" 0 "hypothetical protein"
"DsuD02_00523" 3739 "s63E6H2" 0 "hypothetical protein"
"DsuD02_00525" 3741 "s59E13H2" 0 "Opine dehydrogenase"
"DsuD02_00525" 3741 "s59E25H2" 0 "Opine dehydrogenase"
"DsuD02_00525" 3741 "s59E5H2" 0 "Opine dehydrogenase"
"DsuD02_00525" 3741 "s63E6H2" 0 "Opine dehydrogenase"
"DsuD02_00526" 3742 "s59E13H2" 0 "Glyoxylate reductase"
"DsuD02_00526" 3742 "s59E25H2" 0 "Glyoxylate reductase"
"DsuD02_00526" 3742 "s59E5H2" 0 "Glyoxylate reductase"
"DsuD02_00526" 3742 "s63E6H2" 0 "Glyoxylate reductase"
"DsuD02_00527" 3743 "s59E13H2" 0 "Coenzyme F420 hydrogenase subunit beta"
"DsuD02_00527" 3743 "s59E25H2" 0 "Coenzyme F420 hydrogenase subunit beta"
"DsuD02_00527" 3743 "s59E5H2" 0 "Coenzyme F420 hydrogenase subunit beta"
"DsuD02_00527" 3743 "s63E6H2" 0 "Coenzyme F420 hydrogenase subunit beta"
"DsuD02_00528" 3744 "s59E13H2" 0 "Indolepyruvate oxidoreductase subunit IorA"
"DsuD02_00528" 3744 "s59E25H2" 0 "Indolepyruvate oxidoreductase subunit IorA"
"DsuD02_00528" 3744 "s59E5H2" 0 "Indolepyruvate oxidoreductase subunit IorA"
"DsuD02_00528" 3744 "s63E6H2" 0 "Indolepyruvate oxidoreductase subunit IorA"
"DsuD02_00529" 3745 "s59E13H2" 0 "Indolepyruvate oxidoreductase subunit IorB"
"DsuD02_00529" 3745 "s59E25H2" 0 "Indolepyruvate oxidoreductase subunit IorB"
"DsuD02_00529" 3745 "s59E5H2" 0 "Indolepyruvate oxidoreductase subunit IorB"
"DsuD02_00529" 3745 "s63E6H2" 0 "Indolepyruvate oxidoreductase subunit IorB"
"DsuD02_00530" 3746 "s59E13H2" 0 "Aspartate aminotransferase"
"DsuD02_00530" 3746 "s59E25H2" 0 "Aspartate aminotransferase"
"DsuD02_00530" 3746 "s59E5H2" 0 "Aspartate aminotransferase"
"DsuD02_00530" 3746 "s63E6H2" 0 "Aspartate aminotransferase"
"DsuD02_00531" 3747 "s59E13H2" 0 "Threonylcarbamoyladenosine tRNA methylthiotransferase MtaB"
"DsuD02_00531" 3747 "s59E25H2" 0 "Threonylcarbamoyladenosine tRNA methylthiotransferase MtaB"
"DsuD02_00531" 3747 "s59E5H2" 0 "Threonylcarbamoyladenosine tRNA methylthiotransferase MtaB"
"DsuD02_00531" 3747 "s63E6H2" 0 "Threonylcarbamoyladenosine tRNA methylthiotransferase MtaB"
"DsuD02_00532" 3748 "s59E13H2" 0 "Transcription elongation factor GreA"
"DsuD02_00532" 3748 "s59E25H2" 0 "Transcription elongation factor GreA"
"DsuD02_00532" 3748 "s59E5H2" 0 "Transcription elongation factor GreA"
"DsuD02_00532" 3748 "s63E6H2" 0 "Transcription elongation factor GreA"
"DsuD02_00533" 3749 "s59E13H2" 0 "hypothetical protein"
"DsuD02_00533" 3749 "s59E25H2" 0 "hypothetical protein"
"DsuD02_00533" 3749 "s59E5H2" 0 "hypothetical protein"
"DsuD02_00533" 3749 "s63E6H2" 0 "hypothetical protein"
"DsuD02_00534" 3750 "s59E13H2" 0 "2-hydroxy-3-oxopropionate reductase"
"DsuD02_00534" 3750 "s59E25H2" 0 "2-hydroxy-3-oxopropionate reductase"
"DsuD02_00534" 3750 "s59E5H2" 0 "2-hydroxy-3-oxopropionate reductase"
"DsuD02_00534" 3750 "s63E6H2" 0 "2-hydroxy-3-oxopropionate reductase"

"DsuD02_00535" 3751 "s59E13H2" 0 "Sialic acid TRAP transporter permease protein SiaT"
"DsuD02_00535" 3751 "s59E25H2" 0 "Sialic acid TRAP transporter permease protein SiaT"
"DsuD02_00535" 3751 "s59E5H2" 0 "Sialic acid TRAP transporter permease protein SiaT"
"DsuD02_00535" 3751 "s63E6H2" 0 "Sialic acid TRAP transporter permease protein SiaT"
"DsuD02_00536" 3752 "s59E13H2" 0 "Sialic acid TRAP transporter permease protein SiaT"
"DsuD02_00536" 3752 "s59E25H2" 0 "Sialic acid TRAP transporter permease protein SiaT"
"DsuD02_00536" 3752 "s59E5H2" 0 "Sialic acid TRAP transporter permease protein SiaT"
"DsuD02_00536" 3752 "s63E6H2" 0 "Sialic acid TRAP transporter permease protein SiaT"
"DsuD02_00537" 3753 "s59E13H2" 0 "2,3-diketo-L-gulonate-binding periplasmic protein YiaO precursor"
"DsuD02_00537" 3753 "s59E25H2" 0 "2,3-diketo-L-gulonate-binding periplasmic protein YiaO precursor"
"DsuD02_00537" 3753 "s59E5H2" 0 "2,3-diketo-L-gulonate-binding periplasmic protein YiaO precursor"
"DsuD02_00537" 3753 "s63E6H2" 0 "2,3-diketo-L-gulonate-binding periplasmic protein YiaO precursor"
"DsuD02_00539" 3755 "s59E13H2" 0 "Ferredoxin"
"DsuD02_00539" 3755 "s59E25H2" 0 "Ferredoxin"
"DsuD02_00539" 3755 "s59E5H2" 0 "Ferredoxin"
"DsuD02_00539" 3755 "s63E6H2" 0 "Ferredoxin"
"DsuD02_00540" 3756 "s59E13H2" 0 "putative HTH-type transcriptional regulator YdfH"
"DsuD02_00540" 3756 "s59E25H2" 0 "putative HTH-type transcriptional regulator YdfH"
"DsuD02_00540" 3756 "s59E5H2" 0 "putative HTH-type transcriptional regulator YdfH"
"DsuD02_00540" 3756 "s63E6H2" 0 "putative HTH-type transcriptional regulator YdfH"
"DsuD02_00542" 3758 "s59E13H2" 0 "Hypoxanthine phosphoribosyltransferase"
"DsuD02_00542" 3758 "s59E25H2" 0 "Hypoxanthine phosphoribosyltransferase"
"DsuD02_00542" 3758 "s59E5H2" 0 "Hypoxanthine phosphoribosyltransferase"
"DsuD02_00542" 3758 "s63E6H2" 0 "Hypoxanthine phosphoribosyltransferase"
"DsuD02_00543" 3759 "s59E13H2" 0 "hypothetical protein"
"DsuD02_00543" 3759 "s59E25H2" 0 "hypothetical protein"
"DsuD02_00543" 3759 "s59E5H2" 0 "hypothetical protein"
"DsuD02_00543" 3759 "s63E6H2" 0 "hypothetical protein"
"DsuD02_00544" 3760 "s59E13H2" 0 "hypothetical protein"
"DsuD02_00544" 3760 "s59E25H2" 0 "hypothetical protein"
"DsuD02_00544" 3760 "s59E5H2" 0 "hypothetical protein"
"DsuD02_00544" 3760 "s63E6H2" 0 "hypothetical protein"
"DsuD02_00545" 3761 "s59E13H2" 0 "hypothetical protein"
"DsuD02_00545" 3761 "s59E25H2" 0 "hypothetical protein"
"DsuD02_00545" 3761 "s59E5H2" 0 "hypothetical protein"
"DsuD02_00545" 3761 "s63E6H2" 0 "hypothetical protein"
"DsuD02_00546" 3762 "s59E13H2" 0 "Long-chain-fatty-acid--CoA ligase FadD15"
"DsuD02_00546" 3762 "s59E25H2" 0 "Long-chain-fatty-acid--CoA ligase FadD15"
"DsuD02_00546" 3762 "s59E5H2" 0 "Long-chain-fatty-acid--CoA ligase FadD15"
"DsuD02_00546" 3762 "s63E6H2" 0 "Long-chain-fatty-acid--CoA ligase FadD15"
"DsuD02_00547" 3763 "s59E13H2" 0 "hypothetical protein"
"DsuD02_00547" 3763 "s59E25H2" 0 "hypothetical protein"
"DsuD02_00547" 3763 "s59E5H2" 0 "hypothetical protein"
"DsuD02_00547" 3763 "s63E6H2" 0 "hypothetical protein"
"DsuD02_00548" 3764 "s59E13H2" 0 "Phosphopantetheine adenylyltransferase"
"DsuD02_00548" 3764 "s59E25H2" 0 "Phosphopantetheine adenylyltransferase"
"DsuD02_00548" 3764 "s59E5H2" 0 "Phosphopantetheine adenylyltransferase"

"DsuD02_00548" 3764 "s63E6H2" 0 "Phosphopantetheine adenylyltransferase"
"DsuD02_00549" 3765 "s59E13H2" 0 "Aspartate--tRNA ligase"
"DsuD02_00549" 3765 "s59E25H2" 0 "Aspartate--tRNA ligase"
"DsuD02_00549" 3765 "s59E5H2" 0 "Aspartate--tRNA ligase"
"DsuD02_00549" 3765 "s63E6H2" 0 "Aspartate--tRNA ligase"
"DsuD02_00550" 3766 "s59E13H2" 0 "Histidine--tRNA ligase"
"DsuD02_00550" 3766 "s59E25H2" 0 "Histidine--tRNA ligase"
"DsuD02_00550" 3766 "s59E5H2" 0 "Histidine--tRNA ligase"
"DsuD02_00550" 3766 "s63E6H2" 0 "Histidine--tRNA ligase"
"DsuD02_00552" 3768 "s59E13H2" 0 "hypothetical protein"
"DsuD02_00552" 3768 "s59E25H2" 0 "hypothetical protein"
"DsuD02_00552" 3768 "s59E5H2" 0 "hypothetical protein"
"DsuD02_00552" 3768 "s63E6H2" 0 "hypothetical protein"
"DsuD02_00553" 3769 "s59E13H2" 0 "Glutamate 5-kinase 1"
"DsuD02_00553" 3769 "s59E25H2" 0 "Glutamate 5-kinase 1"
"DsuD02_00553" 3769 "s59E5H2" 0.04415457 "Glutamate 5-kinase 1"
"DsuD02_00553" 3769 "s63E6H2" 0.1602611 "Glutamate 5-kinase 1"
"DsuD02_00554" 3770 "s59E13H2" 0.08931047 "Gamma-glutamyl phosphate reductase"
"DsuD02_00554" 3770 "s59E25H2" 0 "Gamma-glutamyl phosphate reductase"
"DsuD02_00554" 3770 "s59E5H2" 0.07092149 "Gamma-glutamyl phosphate reductase"
"DsuD02_00554" 3770 "s63E6H2" 0.205037 "Gamma-glutamyl phosphate reductase"
"DsuD02_00555" 3771 "s59E13H2" 1.580888 "Nicotinate-nucleotide adenylyltransferase"
"DsuD02_00555" 3771 "s59E25H2" 0 "Nicotinate-nucleotide adenylyltransferase"
"DsuD02_00555" 3771 "s59E5H2" 0.1115194 "Nicotinate-nucleotide adenylyltransferase"
"DsuD02_00555" 3771 "s63E6H2" 0.183889 "Nicotinate-nucleotide adenylyltransferase"
"DsuD02_00556" 3772 "s59E13H2" 1.263952 "putative nicotinate-nucleotide adenylyltransferase"
"DsuD02_00556" 3772 "s59E25H2" 0 "putative nicotinate-nucleotide adenylyltransferase"
"DsuD02_00556" 3772 "s59E5H2" 0.04278096 "putative nicotinate-nucleotide adenylyltransferase"
"DsuD02_00556" 3772 "s63E6H2" 0.1530253 "putative nicotinate-nucleotide adenylyltransferase"
"DsuD02_00557" 3773 "s59E13H2" 0.6695776 "Cell envelope-related transcriptional attenuator domain protein"
"DsuD02_00557" 3773 "s59E25H2" 0 "Cell envelope-related transcriptional attenuator domain protein"
"DsuD02_00557" 3773 "s59E5H2" 0.04572329 "Cell envelope-related transcriptional attenuator domain protein"
"DsuD02_00557" 3773 "s63E6H2" 0.06873089 "Cell envelope-related transcriptional attenuator domain protein"
"DsuD02_00558" 3774 "s59E13H2" 2.318071 "Ribosomal silencing factor RsfS"
"DsuD02_00558" 3774 "s59E25H2" 0 "Ribosomal silencing factor RsfS"
"DsuD02_00558" 3774 "s59E5H2" 0.1841455 "Ribosomal silencing factor RsfS"
"DsuD02_00558" 3774 "s63E6H2" 0.1374414 "Ribosomal silencing factor RsfS"
"DsuD02_00559" 3775 "s59E13H2" 2.731775 "Leucine--tRNA ligase"
"DsuD02_00559" 3775 "s59E25H2" 0 "Leucine--tRNA ligase"
"DsuD02_00559" 3775 "s59E5H2" 0.2173008 "Leucine--tRNA ligase"
"DsuD02_00559" 3775 "s63E6H2" 0.3190769 "Leucine--tRNA ligase"
"DsuD02_00560" 3776 "s59E13H2" 2.021891 "Intracellular protease 1"
"DsuD02_00560" 3776 "s59E25H2" 0 "Intracellular protease 1"
"DsuD02_00560" 3776 "s59E5H2" 0.2322446 "Intracellular protease 1"
"DsuD02_00560" 3776 "s63E6H2" 0.2079117 "Intracellular protease 1"
"DsuD02_00562" 3778 "s59E13H2" 1.187861 "ComE operon protein 1"
"DsuD02_00562" 3778 "s59E25H2" 0 "ComE operon protein 1"

"DsuD02_00562" 3778 "s59E5H2" 0.05954422 "ComE operon protein 1"
"DsuD02_00562" 3778 "s63E6H2" 0.1450605 "ComE operon protein 1"
"DsuD02_00563" 3779 "s59E13H2" 0.9601061 "ComEC family competence protein"
"DsuD02_00563" 3779 "s59E25H2" 0.2259001 "ComEC family competence protein"
"DsuD02_00563" 3779 "s59E5H2" 0.07798013 "ComEC family competence protein"
"DsuD02_00563" 3779 "s63E6H2" 0.1362403 "ComEC family competence protein"
"DsuD02_00564" 3780 "s59E13H2" 0.00755353 "hypothetical protein"
"DsuD02_00564" 3780 "s59E25H2" 0.3122807 "hypothetical protein"
"DsuD02_00564" 3780 "s59E5H2" 0.0243176 "hypothetical protein"
"DsuD02_00564" 3780 "s63E6H2" 0.04117919 "hypothetical protein"
"DsuD02_00565" 3781 "s59E13H2" 0 "DNA ligase"
"DsuD02_00565" 3781 "s59E25H2" 0 "DNA ligase"
"DsuD02_00565" 3781 "s59E5H2" 0.05351477 "DNA ligase"
"DsuD02_00565" 3781 "s63E6H2" 0.1852358 "DNA ligase"
"DsuD02_00566" 3782 "s59E13H2" 0 "putative adenylyltransferase/sulfurtransferase MoeZ"
"DsuD02_00566" 3782 "s59E25H2" 0 "putative adenylyltransferase/sulfurtransferase MoeZ"
"DsuD02_00566" 3782 "s59E5H2" 0.1047723 "putative adenylyltransferase/sulfurtransferase MoeZ"
"DsuD02_00566" 3782 "s63E6H2" 0.001025135 "putative adenylyltransferase/sulfurtransferase MoeZ"
"DsuD02_00567" 3783 "s59E13H2" 0 "hypothetical protein"
"DsuD02_00567" 3783 "s59E25H2" 0 "hypothetical protein"
"DsuD02_00567" 3783 "s59E5H2" 0.01320098 "hypothetical protein"
"DsuD02_00567" 3783 "s63E6H2" 0 "hypothetical protein"
"DsuD02_00568" 3784 "s59E13H2" 0 "5-methyltetrahydrofolate:corrinoid/iron-sulfur protein co-methyltransferase"
"DsuD02_00568" 3784 "s59E25H2" 0 "5-methyltetrahydrofolate:corrinoid/iron-sulfur protein co-methyltransferase"
"DsuD02_00568" 3784 "s59E5H2" 0.04026523 "5-methyltetrahydrofolate:corrinoid/iron-sulfur protein co-methyltransferase"
"DsuD02_00568" 3784 "s63E6H2" 0.1117812 "5-methyltetrahydrofolate:corrinoid/iron-sulfur protein co-methyltransferase"
"DsuD02_00569" 3785 "s59E13H2" 0 "Arginine-binding extracellular protein ArtP precursor"
"DsuD02_00569" 3785 "s59E25H2" 0 "Arginine-binding extracellular protein ArtP precursor"
"DsuD02_00569" 3785 "s59E5H2" 0.0118886 "Arginine-binding extracellular protein ArtP precursor"
"DsuD02_00569" 3785 "s63E6H2" 0.01845552 "Arginine-binding extracellular protein ArtP precursor"
"DsuD02_00570" 3786 "s59E13H2" 0.9256005 "Na(+)-translocating NADH-quinone reductase subunit F"
"DsuD02_00570" 3786 "s59E25H2" 0 "Na(+)-translocating NADH-quinone reductase subunit F"
"DsuD02_00570" 3786 "s59E5H2" 0.00814835 "Na(+)-translocating NADH-quinone reductase subunit F"
"DsuD02_00570" 3786 "s63E6H2" 0.02215054 "Na(+)-translocating NADH-quinone reductase subunit F"
"DsuD02_00571" 3787 "s59E13H2" 0 "hypothetical protein"
"DsuD02_00571" 3787 "s59E25H2" 0 "hypothetical protein"
"DsuD02_00571" 3787 "s59E5H2" 0.003216614 "hypothetical protein"
"DsuD02_00571" 3787 "s63E6H2" 0 "hypothetical protein"
"DsuD02_00572" 3788 "s59E13H2" 0 "Riboflavin synthase"
"DsuD02_00572" 3788 "s59E25H2" 0 "Riboflavin synthase"
"DsuD02_00572" 3788 "s59E5H2" 0 "Riboflavin synthase"
"DsuD02_00572" 3788 "s63E6H2" 0 "Riboflavin synthase"
"DsuD02_00573" 3789 "s59E13H2" 0 "Riboflavin biosynthesis protein RibBA"
"DsuD02_00573" 3789 "s59E25H2" 0 "Riboflavin biosynthesis protein RibBA"
"DsuD02_00573" 3789 "s59E5H2" 0 "Riboflavin biosynthesis protein RibBA"
"DsuD02_00573" 3789 "s63E6H2" 0 "Riboflavin biosynthesis protein RibBA"
"DsuD02_00574" 3790 "s59E13H2" 0 "6,7-dimethyl-8-ribityllumazine synthase"

"DsuD02_00574" 3790 "s59E25H2" 0 "6,7-dimethyl-8-ribityllumazine synthase"
"DsuD02_00574" 3790 "s59E5H2" 0 "6,7-dimethyl-8-ribityllumazine synthase"
"DsuD02_00574" 3790 "s63E6H2" 0 "6,7-dimethyl-8-ribityllumazine synthase"
"DsuD02_00575" 3791 "s59E13H2" 0 "Phosphodiesterase YfcE"
"DsuD02_00575" 3791 "s59E25H2" 0 "Phosphodiesterase YfcE"
"DsuD02_00575" 3791 "s59E5H2" 0 "Phosphodiesterase YfcE"
"DsuD02_00575" 3791 "s63E6H2" 0 "Phosphodiesterase YfcE"
"DsuD02_00576" 3792 "s59E13H2" 0 "hypothetical protein"
"DsuD02_00576" 3792 "s59E25H2" 0 "hypothetical protein"
"DsuD02_00576" 3792 "s59E5H2" 0 "hypothetical protein"
"DsuD02_00576" 3792 "s63E6H2" 0 "hypothetical protein"
"DsuD02_00577" 3793 "s59E13H2" 0 "DAK2 domain protein"
"DsuD02_00577" 3793 "s59E25H2" 0 "DAK2 domain protein"
"DsuD02_00577" 3793 "s59E5H2" 0 "DAK2 domain protein"
"DsuD02_00577" 3793 "s63E6H2" 0 "DAK2 domain protein"
"DsuD02_00578" 3794 "s59E13H2" 0 "ATP-dependent DNA helicase RecG"
"DsuD02_00578" 3794 "s59E25H2" 0 "ATP-dependent DNA helicase RecG"
"DsuD02_00578" 3794 "s59E5H2" 0 "ATP-dependent DNA helicase RecG"
"DsuD02_00578" 3794 "s63E6H2" 0 "ATP-dependent DNA helicase RecG"
"DsuD02_00579" 3795 "s59E13H2" 0 "Ribosomal RNA small subunit methyltransferase D"
"DsuD02_00579" 3795 "s59E25H2" 0 "Ribosomal RNA small subunit methyltransferase D"
"DsuD02_00579" 3795 "s59E5H2" 0 "Ribosomal RNA small subunit methyltransferase D"
"DsuD02_00579" 3795 "s63E6H2" 0 "Ribosomal RNA small subunit methyltransferase D"
"DsuD02_00580" 3796 "s59E13H2" 0 "malonic semialdehyde reductase"
"DsuD02_00580" 3796 "s59E25H2" 0 "malonic semialdehyde reductase"
"DsuD02_00580" 3796 "s59E5H2" 0 "malonic semialdehyde reductase"
"DsuD02_00580" 3796 "s63E6H2" 0 "malonic semialdehyde reductase"
"DsuD02_00581" 3797 "s59E13H2" 0 "B3/4 domain protein"
"DsuD02_00581" 3797 "s59E25H2" 0 "B3/4 domain protein"
"DsuD02_00581" 3797 "s59E5H2" 0 "B3/4 domain protein"
"DsuD02_00581" 3797 "s63E6H2" 0 "B3/4 domain protein"
"DsuD02_00582" 3798 "s59E13H2" 0 "hypothetical protein"
"DsuD02_00582" 3798 "s59E25H2" 0 "hypothetical protein"
"DsuD02_00582" 3798 "s59E5H2" 0 "hypothetical protein"
"DsuD02_00582" 3798 "s63E6H2" 0 "hypothetical protein"
"DsuD02_00583" 3799 "s59E13H2" 0 "Hydroxyacylglutathione hydrolase"
"DsuD02_00583" 3799 "s59E25H2" 0 "Hydroxyacylglutathione hydrolase"
"DsuD02_00583" 3799 "s59E5H2" 0 "Hydroxyacylglutathione hydrolase"
"DsuD02_00583" 3799 "s63E6H2" 0 "Hydroxyacylglutathione hydrolase"
"DsuD02_00584" 3800 "s59E13H2" 0 "High-affinity branched-chain amino acid transport ATP-binding protein LivF"
"DsuD02_00584" 3800 "s59E25H2" 0 "High-affinity branched-chain amino acid transport ATP-binding protein LivF"
"DsuD02_00584" 3800 "s59E5H2" 0 "High-affinity branched-chain amino acid transport ATP-binding protein LivF"
"DsuD02_00584" 3800 "s63E6H2" 0 "High-affinity branched-chain amino acid transport ATP-binding protein LivF"
"DsuD02_00585" 3801 "s59E13H2" 0 "putative branched-chain amino acid transport ATP-binding protein LivG"
"DsuD02_00585" 3801 "s59E25H2" 0 "putative branched-chain amino acid transport ATP-binding protein LivG"
"DsuD02_00585" 3801 "s59E5H2" 0 "putative branched-chain amino acid transport ATP-binding protein LivG"
"DsuD02_00585" 3801 "s63E6H2" 0 "putative branched-chain amino acid transport ATP-binding protein LivG"

"DsuD02_00586" 3802 "s59E13H2" 0 "leucine/isoleucine/valine transporter permease subunit"
"DsuD02_00586" 3802 "s59E25H2" 0 "leucine/isoleucine/valine transporter permease subunit"
"DsuD02_00586" 3802 "s59E5H2" 0 "leucine/isoleucine/valine transporter permease subunit"
"DsuD02_00586" 3802 "s63E6H2" 0 "leucine/isoleucine/valine transporter permease subunit"
"DsuD02_00587" 3803 "s59E13H2" 0 "High-affinity branched-chain amino acid transport system permease protein LivH"
"DsuD02_00587" 3803 "s59E25H2" 0 "High-affinity branched-chain amino acid transport system permease protein LivH"
"DsuD02_00587" 3803 "s59E5H2" 0 "High-affinity branched-chain amino acid transport system permease protein LivH"
"DsuD02_00587" 3803 "s63E6H2" 0 "High-affinity branched-chain amino acid transport system permease protein LivH"
"DsuD02_00588" 3804 "s59E13H2" 0 "hypothetical protein"
"DsuD02_00588" 3804 "s59E25H2" 0 "hypothetical protein"
"DsuD02_00588" 3804 "s59E5H2" 0 "hypothetical protein"
"DsuD02_00588" 3804 "s63E6H2" 0 "hypothetical protein"
"DsuD02_00589" 3805 "s59E13H2" 0 "hypothetical protein"
"DsuD02_00589" 3805 "s59E25H2" 0 "hypothetical protein"
"DsuD02_00589" 3805 "s59E5H2" 0 "hypothetical protein"
"DsuD02_00589" 3805 "s63E6H2" 0 "hypothetical protein"
"DsuD02_00590" 3806 "s59E13H2" 0 "metal-dependent hydrolase"
"DsuD02_00590" 3806 "s59E25H2" 0 "metal-dependent hydrolase"
"DsuD02_00590" 3806 "s59E5H2" 0 "metal-dependent hydrolase"
"DsuD02_00590" 3806 "s63E6H2" 0 "metal-dependent hydrolase"
"DsuD02_00591" 3807 "s59E13H2" 0 "hypothetical protein"
"DsuD02_00591" 3807 "s59E25H2" 0 "hypothetical protein"
"DsuD02_00591" 3807 "s59E5H2" 0 "hypothetical protein"
"DsuD02_00591" 3807 "s63E6H2" 0 "hypothetical protein"
"DsuD02_00592" 3808 "s59E13H2" 0 "hypothetical protein"
"DsuD02_00592" 3808 "s59E25H2" 0 "hypothetical protein"
"DsuD02_00592" 3808 "s59E5H2" 0 "hypothetical protein"
"DsuD02_00592" 3808 "s63E6H2" 0 "hypothetical protein"
"DsuD02_00593" 3809 "s59E13H2" 0 "Transposase DDE domain protein"
"DsuD02_00593" 3809 "s59E25H2" 0 "Transposase DDE domain protein"
"DsuD02_00593" 3809 "s59E5H2" 0 "Transposase DDE domain protein"
"DsuD02_00593" 3809 "s63E6H2" 0 "Transposase DDE domain protein"
"DsuD02_00594" 3810 "s59E13H2" 0 "Psp operon transcriptional activator"
"DsuD02_00594" 3810 "s59E25H2" 0 "Psp operon transcriptional activator"
"DsuD02_00594" 3810 "s59E5H2" 0 "Psp operon transcriptional activator"
"DsuD02_00594" 3810 "s63E6H2" 0 "Psp operon transcriptional activator"
"DsuD02_00595" 3811 "s59E13H2" 0 "Phage shock protein A"
"DsuD02_00595" 3811 "s59E25H2" 0 "Phage shock protein A"
"DsuD02_00595" 3811 "s59E5H2" 0 "Phage shock protein A"
"DsuD02_00595" 3811 "s63E6H2" 0 "Phage shock protein A"
"DsuD02_00596" 3812 "s59E13H2" 0 "hypothetical protein"
"DsuD02_00596" 3812 "s59E25H2" 0 "hypothetical protein"
"DsuD02_00596" 3812 "s59E5H2" 0 "hypothetical protein"
"DsuD02_00596" 3812 "s63E6H2" 0 "hypothetical protein"
"DsuD02_00597" 3813 "s59E13H2" 0 "Phage shock protein C"
"DsuD02_00597" 3813 "s59E25H2" 0 "Phage shock protein C"
"DsuD02_00597" 3813 "s59E5H2" 0 "Phage shock protein C"

"DsuD02_00597" 3813 "s63E6H2" 0 "Phage shock protein C"
"DsuD02_00598" 3814 "s59E13H2" 0 "DSBA-like thioredoxin domain protein"
"DsuD02_00598" 3814 "s59E25H2" 0 "DSBA-like thioredoxin domain protein"
"DsuD02_00598" 3814 "s59E5H2" 0 "DSBA-like thioredoxin domain protein"
"DsuD02_00598" 3814 "s63E6H2" 0 "DSBA-like thioredoxin domain protein"
"DsuD02_00599" 3815 "s59E13H2" 0 "Dimethylamine corrinoid protein"
"DsuD02_00599" 3815 "s59E25H2" 0 "Dimethylamine corrinoid protein"
"DsuD02_00599" 3815 "s59E5H2" 0 "Dimethylamine corrinoid protein"
"DsuD02_00599" 3815 "s63E6H2" 0 "Dimethylamine corrinoid protein"
"DsuD02_00600" 3816 "s59E13H2" 0 "methylcobalamin:coenzyme M methyltransferase"
"DsuD02_00600" 3816 "s59E25H2" 0 "methylcobalamin:coenzyme M methyltransferase"
"DsuD02_00600" 3816 "s59E5H2" 0 "methylcobalamin:coenzyme M methyltransferase"
"DsuD02_00600" 3816 "s63E6H2" 0 "methylcobalamin:coenzyme M methyltransferase"
"DsuD02_00601" 3817 "s59E13H2" 0 "Sensor protein FixL"
"DsuD02_00601" 3817 "s59E25H2" 0 "Sensor protein FixL"
"DsuD02_00601" 3817 "s59E5H2" 0 "Sensor protein FixL"
"DsuD02_00601" 3817 "s63E6H2" 0 "Sensor protein FixL"
"DsuD02_00602" 3818 "s59E13H2" 0 "Putative phosphoribosyl transferase/MT0597"
"DsuD02_00602" 3818 "s59E25H2" 0 "Putative phosphoribosyl transferase/MT0597"
"DsuD02_00602" 3818 "s59E5H2" 0 "Putative phosphoribosyl transferase/MT0597"
"DsuD02_00602" 3818 "s63E6H2" 0 "Putative phosphoribosyl transferase/MT0597"
"DsuD02_00603" 3819 "s59E13H2" 0 "Leucine dehydrogenase"
"DsuD02_00603" 3819 "s59E25H2" 0 "Leucine dehydrogenase"
"DsuD02_00603" 3819 "s59E5H2" 0 "Leucine dehydrogenase"
"DsuD02_00603" 3819 "s63E6H2" 0 "Leucine dehydrogenase"
"DsuD02_00604" 3820 "s59E13H2" 0 "SdiA-regulated"
"DsuD02_00604" 3820 "s59E25H2" 0 "SdiA-regulated"
"DsuD02_00604" 3820 "s59E5H2" 0 "SdiA-regulated"
"DsuD02_00604" 3820 "s63E6H2" 0 "SdiA-regulated"
"DsuD02_00605" 3821 "s59E13H2" 0 "putative 3-hydroxybutyryl-CoA dehydrogenase"
"DsuD02_00605" 3821 "s59E25H2" 0 "putative 3-hydroxybutyryl-CoA dehydrogenase"
"DsuD02_00605" 3821 "s59E5H2" 0 "putative 3-hydroxybutyryl-CoA dehydrogenase"
"DsuD02_00605" 3821 "s63E6H2" 0 "putative 3-hydroxybutyryl-CoA dehydrogenase"
"DsuD02_00606" 3822 "s59E13H2" 0 "Glycosyl transferase family 2"
"DsuD02_00606" 3822 "s59E25H2" 0 "Glycosyl transferase family 2"
"DsuD02_00606" 3822 "s59E5H2" 0 "Glycosyl transferase family 2"
"DsuD02_00606" 3822 "s63E6H2" 0 "Glycosyl transferase family 2"
"DsuD02_00607" 3823 "s59E13H2" 0 "Alkaline phosphatase synthesis transcriptional regulatory protein PhoP"
"DsuD02_00607" 3823 "s59E25H2" 0 "Alkaline phosphatase synthesis transcriptional regulatory protein PhoP"
"DsuD02_00607" 3823 "s59E5H2" 0 "Alkaline phosphatase synthesis transcriptional regulatory protein PhoP"
"DsuD02_00607" 3823 "s63E6H2" 0 "Alkaline phosphatase synthesis transcriptional regulatory protein PhoP"
"DsuD02_00608" 3824 "s59E13H2" 0 "Lipopolysaccharide export system permease protein LptG"
"DsuD02_00608" 3824 "s59E25H2" 0 "Lipopolysaccharide export system permease protein LptG"
"DsuD02_00608" 3824 "s59E5H2" 0 "Lipopolysaccharide export system permease protein LptG"
"DsuD02_00608" 3824 "s63E6H2" 0 "Lipopolysaccharide export system permease protein LptG"
"DsuD02_00609" 3825 "s59E13H2" 0 "Threonine synthase"
"DsuD02_00609" 3825 "s59E25H2" 0 "Threonine synthase"

"DsuD02_00609" 3825 "s59E5H2" 0 "Threonine synthase"
"DsuD02_00609" 3825 "s63E6H2" 0 "Threonine synthase"
"DsuD02_00610" 3826 "s59E13H2" 0 "Protein-L-isoaspartate O-methyltransferase"
"DsuD02_00610" 3826 "s59E25H2" 0 "Protein-L-isoaspartate O-methyltransferase"
"DsuD02_00610" 3826 "s59E5H2" 0 "Protein-L-isoaspartate O-methyltransferase"
"DsuD02_00610" 3826 "s63E6H2" 0 "Protein-L-isoaspartate O-methyltransferase"
"DsuD02_00611" 3827 "s59E13H2" 0 "Phosphoribosylglycinamide formyltransferase"
"DsuD02_00611" 3827 "s59E25H2" 0 "Phosphoribosylglycinamide formyltransferase"
"DsuD02_00611" 3827 "s59E5H2" 0 "Phosphoribosylglycinamide formyltransferase"
"DsuD02_00611" 3827 "s63E6H2" 0 "Phosphoribosylglycinamide formyltransferase"
"DsuD02_00612" 3828 "s59E13H2" 0 "hypothetical protein"
"DsuD02_00612" 3828 "s59E25H2" 0 "hypothetical protein"
"DsuD02_00612" 3828 "s59E5H2" 0 "hypothetical protein"
"DsuD02_00612" 3828 "s63E6H2" 0 "hypothetical protein"
"DsuD02_00613" 3829 "s59E13H2" 0 "Aspartate racemase"
"DsuD02_00613" 3829 "s59E25H2" 0 "Aspartate racemase"
"DsuD02_00613" 3829 "s59E5H2" 0 "Aspartate racemase"
"DsuD02_00613" 3829 "s63E6H2" 0 "Aspartate racemase"
"DsuD02_00615" 3831 "s59E13H2" 0 "4-hydroxy-tetrahydrodipicolinate synthase"
"DsuD02_00615" 3831 "s59E25H2" 0 "4-hydroxy-tetrahydrodipicolinate synthase"
"DsuD02_00615" 3831 "s59E5H2" 0 "4-hydroxy-tetrahydrodipicolinate synthase"
"DsuD02_00615" 3831 "s63E6H2" 0 "4-hydroxy-tetrahydrodipicolinate synthase"
"DsuD02_00616" 3832 "s59E13H2" 0 "lipoprotein NlpI"
"DsuD02_00616" 3832 "s59E25H2" 0 "lipoprotein NlpI"
"DsuD02_00616" 3832 "s59E5H2" 0 "lipoprotein NlpI"
"DsuD02_00616" 3832 "s63E6H2" 0 "lipoprotein NlpI"
"DsuD02_00617" 3833 "s59E13H2" 0 "Epoxyqueuosine reductase"
"DsuD02_00617" 3833 "s59E25H2" 0 "Epoxyqueuosine reductase"
"DsuD02_00617" 3833 "s59E5H2" 0 "Epoxyqueuosine reductase"
"DsuD02_00617" 3833 "s63E6H2" 0 "Epoxyqueuosine reductase"
"DsuD02_00619" 3835 "s59E13H2" 0 "D-alanine--D-alanine ligase A"
"DsuD02_00619" 3835 "s59E25H2" 0 "D-alanine--D-alanine ligase A"
"DsuD02_00619" 3835 "s59E5H2" 0 "D-alanine--D-alanine ligase A"
"DsuD02_00619" 3835 "s63E6H2" 0 "D-alanine--D-alanine ligase A"
"DsuD02_00620" 3836 "s59E13H2" 0 "Purine efflux pump PbuE"
"DsuD02_00620" 3836 "s59E25H2" 0 "Purine efflux pump PbuE"
"DsuD02_00620" 3836 "s59E5H2" 0 "Purine efflux pump PbuE"
"DsuD02_00620" 3836 "s63E6H2" 0 "Purine efflux pump PbuE"
"DsuD02_00621" 3837 "s59E13H2" 0 "3-hydroxypropionyl-coenzyme A dehydratase"
"DsuD02_00621" 3837 "s59E25H2" 0 "3-hydroxypropionyl-coenzyme A dehydratase"
"DsuD02_00621" 3837 "s59E5H2" 0 "3-hydroxypropionyl-coenzyme A dehydratase"
"DsuD02_00621" 3837 "s63E6H2" 0 "3-hydroxypropionyl-coenzyme A dehydratase"
"DsuD02_00622" 3838 "s59E13H2" 0 "hypothetical protein"
"DsuD02_00622" 3838 "s59E25H2" 0 "hypothetical protein"
"DsuD02_00622" 3838 "s59E5H2" 0 "hypothetical protein"
"DsuD02_00622" 3838 "s63E6H2" 0 "hypothetical protein"
"DsuD02_00623" 3839 "s59E13H2" 0 "2-hydroxymuconate tautomerase"

"DsuD02_00623" 3839 "s59E25H2" 0 "2-hydroxymuconate tautomerase"
"DsuD02_00623" 3839 "s59E5H2" 0 "2-hydroxymuconate tautomerase"
"DsuD02_00623" 3839 "s63E6H2" 0 "2-hydroxymuconate tautomerase"
"DsuD02_00624" 3840 "s59E13H2" 0 "Acryloyl-CoA reductase electron transfer subunit beta"
"DsuD02_00624" 3840 "s59E25H2" 0 "Acryloyl-CoA reductase electron transfer subunit beta"
"DsuD02_00624" 3840 "s59E5H2" 0 "Acryloyl-CoA reductase electron transfer subunit beta"
"DsuD02_00624" 3840 "s63E6H2" 0 "Acryloyl-CoA reductase electron transfer subunit beta"
"DsuD02_00625" 3841 "s59E13H2" 0 "Acryloyl-CoA reductase electron transfer subunit gamma"
"DsuD02_00625" 3841 "s59E25H2" 0 "Acryloyl-CoA reductase electron transfer subunit gamma"
"DsuD02_00625" 3841 "s59E5H2" 0 "Acryloyl-CoA reductase electron transfer subunit gamma"
"DsuD02_00625" 3841 "s63E6H2" 0 "Acryloyl-CoA reductase electron transfer subunit gamma"
"DsuD02_00626" 3842 "s59E13H2" 0 "Acyl-CoA dehydrogenase"
"DsuD02_00626" 3842 "s59E25H2" 0 "Acyl-CoA dehydrogenase"
"DsuD02_00626" 3842 "s59E5H2" 0 "Acyl-CoA dehydrogenase"
"DsuD02_00626" 3842 "s63E6H2" 0 "Acyl-CoA dehydrogenase"
"DsuD02_00627" 3843 "s59E13H2" 0 "Lon protease 2"
"DsuD02_00627" 3843 "s59E25H2" 0 "Lon protease 2"
"DsuD02_00627" 3843 "s59E5H2" 0 "Lon protease 2"
"DsuD02_00627" 3843 "s63E6H2" 0 "Lon protease 2"
"DsuD02_00628" 3844 "s59E13H2" 0 "hypothetical protein"
"DsuD02_00628" 3844 "s59E25H2" 0 "hypothetical protein"
"DsuD02_00628" 3844 "s59E5H2" 0 "hypothetical protein"
"DsuD02_00628" 3844 "s63E6H2" 0 "hypothetical protein"
"DsuD02_00629" 3845 "s59E13H2" 0 "Arylsulfotransferase (ASST)"
"DsuD02_00629" 3845 "s59E25H2" 0 "Arylsulfotransferase (ASST)"
"DsuD02_00629" 3845 "s59E5H2" 0 "Arylsulfotransferase (ASST)"
"DsuD02_00629" 3845 "s63E6H2" 0 "Arylsulfotransferase (ASST)"
"DsuD02_00630" 3846 "s59E13H2" 0 "mRNA interferase EndoA"
"DsuD02_00630" 3846 "s59E25H2" 0 "mRNA interferase EndoA"
"DsuD02_00630" 3846 "s59E5H2" 0 "mRNA interferase EndoA"
"DsuD02_00630" 3846 "s63E6H2" 0 "mRNA interferase EndoA"
"DsuD02_00631" 3847 "s59E13H2" 0 "SpoVT / AbrB like domain protein"
"DsuD02_00631" 3847 "s59E25H2" 0 "SpoVT / AbrB like domain protein"
"DsuD02_00631" 3847 "s59E5H2" 0 "SpoVT / AbrB like domain protein"
"DsuD02_00631" 3847 "s63E6H2" 0 "SpoVT / AbrB like domain protein"
"DsuD02_00632" 3848 "s59E13H2" 0 "hypothetical protein"
"DsuD02_00632" 3848 "s59E25H2" 0 "hypothetical protein"
"DsuD02_00632" 3848 "s59E5H2" 0.02583939 "hypothetical protein"
"DsuD02_00632" 3848 "s63E6H2" 0 "hypothetical protein"
"DsuD02_00634" 3850 "s59E13H2" 0 "HicB family protein"
"DsuD02_00634" 3850 "s59E25H2" 0 "HicB family protein"
"DsuD02_00634" 3850 "s59E5H2" 0 "HicB family protein"
"DsuD02_00634" 3850 "s63E6H2" 0 "HicB family protein"
"DsuD02_00635" 3851 "s59E13H2" 0 "hypothetical protein"
"DsuD02_00635" 3851 "s59E25H2" 0 "hypothetical protein"
"DsuD02_00635" 3851 "s59E5H2" 0 "hypothetical protein"
"DsuD02_00635" 3851 "s63E6H2" 0 "hypothetical protein"

"DsuD02_00636" 3852 "s59E13H2" 0 "Helix-turn-helix"
"DsuD02_00636" 3852 "s59E25H2" 0 "Helix-turn-helix"
"DsuD02_00636" 3852 "s59E5H2" 0 "Helix-turn-helix"
"DsuD02_00636" 3852 "s63E6H2" 0 "Helix-turn-helix"
"DsuD02_00637" 3853 "s59E13H2" 0 "hypothetical protein"
"DsuD02_00637" 3853 "s59E25H2" 0 "hypothetical protein"
"DsuD02_00637" 3853 "s59E5H2" 0 "hypothetical protein"
"DsuD02_00637" 3853 "s63E6H2" 0 "hypothetical protein"
"DsuD02_00638" 3854 "s59E13H2" 0 "hypothetical protein"
"DsuD02_00638" 3854 "s59E25H2" 0 "hypothetical protein"
"DsuD02_00638" 3854 "s59E5H2" 0 "hypothetical protein"
"DsuD02_00638" 3854 "s63E6H2" 0 "hypothetical protein"
"DsuD02_00640" 3856 "s59E13H2" 0 "hypothetical protein"
"DsuD02_00640" 3856 "s59E25H2" 0 "hypothetical protein"
"DsuD02_00640" 3856 "s59E5H2" 0 "hypothetical protein"
"DsuD02_00640" 3856 "s63E6H2" 0 "hypothetical protein"
"DsuD02_00641" 3857 "s59E13H2" 0 "DNA utilization protein GntX"
"DsuD02_00641" 3857 "s59E25H2" 0 "DNA utilization protein GntX"
"DsuD02_00641" 3857 "s59E5H2" 0 "DNA utilization protein GntX"
"DsuD02_00641" 3857 "s63E6H2" 0 "DNA utilization protein GntX"
"DsuD02_00642" 3858 "s59E13H2" 0 "Metal binding domain of Ada"
"DsuD02_00642" 3858 "s59E25H2" 0 "Metal binding domain of Ada"
"DsuD02_00642" 3858 "s59E5H2" 0 "Metal binding domain of Ada"
"DsuD02_00642" 3858 "s63E6H2" 0 "Metal binding domain of Ada"
"DsuD02_00643" 3859 "s59E13H2" 0 "Cyclic nucleotide-gated potassium channel"
"DsuD02_00643" 3859 "s59E25H2" 0 "Cyclic nucleotide-gated potassium channel"
"DsuD02_00643" 3859 "s59E5H2" 0 "Cyclic nucleotide-gated potassium channel"
"DsuD02_00643" 3859 "s63E6H2" 0 "Cyclic nucleotide-gated potassium channel"
"DsuD02_00646" 3862 "s59E13H2" 0 "hypothetical protein"
"DsuD02_00646" 3862 "s59E25H2" 0 "hypothetical protein"
"DsuD02_00646" 3862 "s59E5H2" 0 "hypothetical protein"
"DsuD02_00646" 3862 "s63E6H2" 0 "hypothetical protein"
"DsuD02_00648" 3864 "s59E13H2" 0 "hypothetical protein"
"DsuD02_00648" 3864 "s59E25H2" 0 "hypothetical protein"
"DsuD02_00648" 3864 "s59E5H2" 0 "hypothetical protein"
"DsuD02_00648" 3864 "s63E6H2" 0 "hypothetical protein"
"DsuD02_00649" 3865 "s59E13H2" 0 "hypothetical protein"
"DsuD02_00649" 3865 "s59E25H2" 0 "hypothetical protein"
"DsuD02_00649" 3865 "s59E5H2" 0 "hypothetical protein"
"DsuD02_00649" 3865 "s63E6H2" 0 "hypothetical protein"
"DsuD02_00650" 3866 "s59E13H2" 0 "Helix-turn-helix domain protein"
"DsuD02_00650" 3866 "s59E25H2" 0 "Helix-turn-helix domain protein"
"DsuD02_00650" 3866 "s59E5H2" 0 "Helix-turn-helix domain protein"
"DsuD02_00650" 3866 "s63E6H2" 0 "Helix-turn-helix domain protein"
"DsuD02_00651" 3867 "s59E13H2" 0 "hypothetical protein"
"DsuD02_00651" 3867 "s59E25H2" 0 "hypothetical protein"
"DsuD02_00651" 3867 "s59E5H2" 0 "hypothetical protein"

"DsuD02_00651" 3867 "s63E6H2" 0 "hypothetical protein"
"DsuD02_00656" 3872 "s59E13H2" 0 "hypothetical protein"
"DsuD02_00656" 3872 "s59E25H2" 0 "hypothetical protein"
"DsuD02_00656" 3872 "s59E5H2" 0 "hypothetical protein"
"DsuD02_00656" 3872 "s63E6H2" 0 "hypothetical protein"
"DsuD02_00657" 3873 "s59E13H2" 0 "hypothetical protein"
"DsuD02_00657" 3873 "s59E25H2" 0 "hypothetical protein"
"DsuD02_00657" 3873 "s59E5H2" 0 "hypothetical protein"
"DsuD02_00657" 3873 "s63E6H2" 0 "hypothetical protein"
"DsuD02_00660" 3876 "s59E13H2" 0 "hypothetical protein"
"DsuD02_00660" 3876 "s59E25H2" 0 "hypothetical protein"
"DsuD02_00660" 3876 "s59E5H2" 0 "hypothetical protein"
"DsuD02_00660" 3876 "s63E6H2" 0 "hypothetical protein"
"DsuD02_00662" 3878 "s59E13H2" 0 "hypothetical protein"
"DsuD02_00662" 3878 "s59E25H2" 0 "hypothetical protein"
"DsuD02_00662" 3878 "s59E5H2" 0 "hypothetical protein"
"DsuD02_00662" 3878 "s63E6H2" 0 "hypothetical protein"
"DsuD02_00667" 3883 "s59E13H2" 0 "hypothetical protein"
"DsuD02_00667" 3883 "s59E25H2" 0 "hypothetical protein"
"DsuD02_00667" 3883 "s59E5H2" 0 "hypothetical protein"
"DsuD02_00667" 3883 "s63E6H2" 0 "hypothetical protein"
"DsuD02_00668" 3884 "s59E13H2" 0 "hypothetical protein"
"DsuD02_00668" 3884 "s59E25H2" 0 "hypothetical protein"
"DsuD02_00668" 3884 "s59E5H2" 0 "hypothetical protein"
"DsuD02_00668" 3884 "s63E6H2" 0 "hypothetical protein"
"DsuD02_00672" 3888 "s59E13H2" 0 "hypothetical protein"
"DsuD02_00672" 3888 "s59E25H2" 0 "hypothetical protein"
"DsuD02_00672" 3888 "s59E5H2" 0 "hypothetical protein"
"DsuD02_00672" 3888 "s63E6H2" 0 "hypothetical protein"
"DsuD02_00673" 3889 "s59E13H2" 0 "hypothetical protein"
"DsuD02_00673" 3889 "s59E25H2" 0 "hypothetical protein"
"DsuD02_00673" 3889 "s59E5H2" 0 "hypothetical protein"
"DsuD02_00673" 3889 "s63E6H2" 0 "hypothetical protein"
"DsuD02_00675" 3891 "s59E13H2" 0 "hypothetical protein"
"DsuD02_00675" 3891 "s59E25H2" 0 "hypothetical protein"
"DsuD02_00675" 3891 "s59E5H2" 0 "hypothetical protein"
"DsuD02_00675" 3891 "s63E6H2" 0 "hypothetical protein"
"DsuD02_00676" 3892 "s59E13H2" 0 "hypothetical protein"
"DsuD02_00676" 3892 "s59E25H2" 0 "hypothetical protein"
"DsuD02_00676" 3892 "s59E5H2" 0 "hypothetical protein"
"DsuD02_00676" 3892 "s63E6H2" 0 "hypothetical protein"
"DsuD02_00677" 3893 "s59E13H2" 0 "Tyrosine recombinase XerD"
"DsuD02_00677" 3893 "s59E25H2" 0 "Tyrosine recombinase XerD"
"DsuD02_00677" 3893 "s59E5H2" 0 "Tyrosine recombinase XerD"
"DsuD02_00677" 3893 "s63E6H2" 0 "Tyrosine recombinase XerD"
"DsuD02_00678" 3894 "s59E13H2" 0 "hypothetical protein"
"DsuD02_00678" 3894 "s59E25H2" 0 "hypothetical protein"

"DsuD02_00678" 3894 "s59E5H2" 0 "hypothetical protein"
"DsuD02_00678" 3894 "s63E6H2" 0 "hypothetical protein"
"DsuD02_00679" 3895 "s59E13H2" 0 "UDP-3-O-[3-hydroxymyristoyl] N-acetylglucosamine deacetylase"
"DsuD02_00679" 3895 "s59E25H2" 0 "UDP-3-O-[3-hydroxymyristoyl] N-acetylglucosamine deacetylase"
"DsuD02_00679" 3895 "s59E5H2" 0 "UDP-3-O-[3-hydroxymyristoyl] N-acetylglucosamine deacetylase"
"DsuD02_00679" 3895 "s63E6H2" 0 "UDP-3-O-[3-hydroxymyristoyl] N-acetylglucosamine deacetylase"
"DsuD02_00680" 3896 "s59E13H2" 0 "Membrane-bound lytic murein transglycosylase A precursor"
"DsuD02_00680" 3896 "s59E25H2" 0 "Membrane-bound lytic murein transglycosylase A precursor"
"DsuD02_00680" 3896 "s59E5H2" 0 "Membrane-bound lytic murein transglycosylase A precursor"
"DsuD02_00680" 3896 "s63E6H2" 0 "Membrane-bound lytic murein transglycosylase A precursor"
"DsuD02_00681" 3897 "s59E13H2" 0 "Glycine--tRNA ligase beta subunit"
"DsuD02_00681" 3897 "s59E25H2" 0 "Glycine--tRNA ligase beta subunit"
"DsuD02_00681" 3897 "s59E5H2" 0 "Glycine--tRNA ligase beta subunit"
"DsuD02_00681" 3897 "s63E6H2" 0 "Glycine--tRNA ligase beta subunit"
"DsuD02_00682" 3898 "s59E13H2" 0 "hypothetical protein"
"DsuD02_00682" 3898 "s59E25H2" 0 "hypothetical protein"
"DsuD02_00682" 3898 "s59E5H2" 0 "hypothetical protein"
"DsuD02_00682" 3898 "s63E6H2" 0 "hypothetical protein"
"DsuD02_00683" 3899 "s59E13H2" 0 "Glycine--tRNA ligase alpha subunit"
"DsuD02_00683" 3899 "s59E25H2" 0 "Glycine--tRNA ligase alpha subunit"
"DsuD02_00683" 3899 "s59E5H2" 0 "Glycine--tRNA ligase alpha subunit"
"DsuD02_00683" 3899 "s63E6H2" 0 "Glycine--tRNA ligase alpha subunit"
"DsuD02_00684" 3900 "s59E13H2" 0 "DNA repair protein RecO"
"DsuD02_00684" 3900 "s59E25H2" 0 "DNA repair protein RecO"
"DsuD02_00684" 3900 "s59E5H2" 0 "DNA repair protein RecO"
"DsuD02_00684" 3900 "s63E6H2" 0 "DNA repair protein RecO"
"DsuD02_00685" 3901 "s59E13H2" 0 "hypothetical protein"
"DsuD02_00685" 3901 "s59E25H2" 0 "hypothetical protein"
"DsuD02_00685" 3901 "s59E5H2" 0 "hypothetical protein"
"DsuD02_00685" 3901 "s63E6H2" 0 "hypothetical protein"
"DsuD02_00686" 3902 "s59E13H2" 0 "High-affinity branched-chain amino acid transport ATP-binding protein LivF"
"DsuD02_00686" 3902 "s59E25H2" 0 "High-affinity branched-chain amino acid transport ATP-binding protein LivF"
"DsuD02_00686" 3902 "s59E5H2" 0 "High-affinity branched-chain amino acid transport ATP-binding protein LivF"
"DsuD02_00686" 3902 "s63E6H2" 0 "High-affinity branched-chain amino acid transport ATP-binding protein LivF"
"DsuD02_00687" 3903 "s59E13H2" 0 "hypothetical protein"
"DsuD02_00687" 3903 "s59E25H2" 0 "hypothetical protein"
"DsuD02_00687" 3903 "s59E5H2" 0 "hypothetical protein"
"DsuD02_00687" 3903 "s63E6H2" 0 "hypothetical protein"
"DsuD02_00688" 3904 "s59E13H2" 0 "leucine/isoleucine/valine transporter permease subunit"
"DsuD02_00688" 3904 "s59E25H2" 0 "leucine/isoleucine/valine transporter permease subunit"
"DsuD02_00688" 3904 "s59E5H2" 0 "leucine/isoleucine/valine transporter permease subunit"
"DsuD02_00688" 3904 "s63E6H2" 0.005010708 "leucine/isoleucine/valine transporter permease subunit"
"DsuD02_00689" 3905 "s59E13H2" 0 "High-affinity branched-chain amino acid transport system permease protein LivH"
"DsuD02_00689" 3905 "s59E25H2" 0 "High-affinity branched-chain amino acid transport system permease protein LivH"
"DsuD02_00689" 3905 "s59E5H2" 0 "High-affinity branched-chain amino acid transport system permease protein LivH"
"DsuD02_00689" 3905 "s63E6H2" 0 "High-affinity branched-chain amino acid transport system permease protein LivH"
"DsuD02_00690" 3906 "s59E13H2" 0 "Long-chain-fatty-acid--CoA ligase FadD15"

"DsuD02_00690" 3906 "s59E25H2" 0 "Long-chain-fatty-acid--CoA ligase FadD15"
"DsuD02_00690" 3906 "s59E5H2" 0 "Long-chain-fatty-acid--CoA ligase FadD15"
"DsuD02_00690" 3906 "s63E6H2" 0 "Long-chain-fatty-acid--CoA ligase FadD15"
"DsuD02_00691" 3907 "s59E13H2" 0 "putative branched-chain amino acid transport ATP-binding protein LivG"
"DsuD02_00691" 3907 "s59E25H2" 0 "putative branched-chain amino acid transport ATP-binding protein LivG"
"DsuD02_00691" 3907 "s59E5H2" 0 "putative branched-chain amino acid transport ATP-binding protein LivG"
"DsuD02_00691" 3907 "s63E6H2" 0 "putative branched-chain amino acid transport ATP-binding protein LivG"
"DsuD02_00692" 3908 "s59E13H2" 0 "Deoxyguanosinetriphosphate triphosphohydrolase"
"DsuD02_00692" 3908 "s59E25H2" 0 "Deoxyguanosinetriphosphate triphosphohydrolase"
"DsuD02_00692" 3908 "s59E5H2" 0 "Deoxyguanosinetriphosphate triphosphohydrolase"
"DsuD02_00692" 3908 "s63E6H2" 0 "Deoxyguanosinetriphosphate triphosphohydrolase"
"DsuD02_00693" 3909 "s59E13H2" 0 "tRNA(Ile)-lysidine synthase"
"DsuD02_00693" 3909 "s59E25H2" 0 "tRNA(Ile)-lysidine synthase"
"DsuD02_00693" 3909 "s59E5H2" 0 "tRNA(Ile)-lysidine synthase"
"DsuD02_00693" 3909 "s63E6H2" 0 "tRNA(Ile)-lysidine synthase"
"DsuD02_00694" 3910 "s59E13H2" 0 "Acyl-coenzyme A:6-aminopenicillanic acid acyl-transferase"
"DsuD02_00694" 3910 "s59E25H2" 0 "Acyl-coenzyme A:6-aminopenicillanic acid acyl-transferase"
"DsuD02_00694" 3910 "s59E5H2" 0 "Acyl-coenzyme A:6-aminopenicillanic acid acyl-transferase"
"DsuD02_00694" 3910 "s63E6H2" 0 "Acyl-coenzyme A:6-aminopenicillanic acid acyl-transferase"
"DsuD02_00695" 3911 "s59E13H2" 0 "pyrroline-5-carboxylate reductase"
"DsuD02_00695" 3911 "s59E25H2" 0 "pyrroline-5-carboxylate reductase"
"DsuD02_00695" 3911 "s59E5H2" 0 "pyrroline-5-carboxylate reductase"
"DsuD02_00695" 3911 "s63E6H2" 0 "pyrroline-5-carboxylate reductase"
"DsuD02_00696" 3912 "s59E13H2" 0 "3-methyl-2-oxobutanoate hydroxymethyltransferase"
"DsuD02_00696" 3912 "s59E25H2" 0 "3-methyl-2-oxobutanoate hydroxymethyltransferase"
"DsuD02_00696" 3912 "s59E5H2" 0.05520542 "3-methyl-2-oxobutanoate hydroxymethyltransferase"
"DsuD02_00696" 3912 "s63E6H2" 0 "3-methyl-2-oxobutanoate hydroxymethyltransferase"
"DsuD02_00697" 3913 "s59E13H2" 0 "Acetylene hydratase"
"DsuD02_00697" 3913 "s59E25H2" 0 "Acetylene hydratase"
"DsuD02_00697" 3913 "s59E5H2" 0 "Acetylene hydratase"
"DsuD02_00697" 3913 "s63E6H2" 0 "Acetylene hydratase"
"DsuD02_00698" 3914 "s59E13H2" 0 "3-oxoacyl-[acyl-carrier-protein] reductase FabG"
"DsuD02_00698" 3914 "s59E25H2" 0 "3-oxoacyl-[acyl-carrier-protein] reductase FabG"
"DsuD02_00698" 3914 "s59E5H2" 0 "3-oxoacyl-[acyl-carrier-protein] reductase FabG"
"DsuD02_00698" 3914 "s63E6H2" 0 "3-oxoacyl-[acyl-carrier-protein] reductase FabG"
"DsuD02_00699" 3915 "s59E13H2" 0 "Signal recognition particle receptor FtsY"
"DsuD02_00699" 3915 "s59E25H2" 0 "Signal recognition particle receptor FtsY"
"DsuD02_00699" 3915 "s59E5H2" 0 "Signal recognition particle receptor FtsY"
"DsuD02_00699" 3915 "s63E6H2" 0 "Signal recognition particle receptor FtsY"
"DsuD02_00700" 3916 "s59E13H2" 0 "Chromosome partition protein Smc"
"DsuD02_00700" 3916 "s59E25H2" 0 "Chromosome partition protein Smc"
"DsuD02_00700" 3916 "s59E5H2" 0 "Chromosome partition protein Smc"
"DsuD02_00700" 3916 "s63E6H2" 0 "Chromosome partition protein Smc"
"DsuD02_00701" 3917 "s59E13H2" 0 "LysM domain/BON superfamily protein"
"DsuD02_00701" 3917 "s59E25H2" 0 "LysM domain/BON superfamily protein"
"DsuD02_00701" 3917 "s59E5H2" 0 "LysM domain/BON superfamily protein"
"DsuD02_00701" 3917 "s63E6H2" 0 "LysM domain/BON superfamily protein"

"DsuE09_00001" 3918 "s59E13H2" 0 "hypothetical protein"
"DsuE09_00001" 3918 "s59E25H2" 0 "hypothetical protein"
"DsuE09_00001" 3918 "s59E5H2" 0 "hypothetical protein"
"DsuE09_00001" 3918 "s63E6H2" 0 "hypothetical protein"
"DsuE09_00002" 3919 "s59E13H2" 0 "hypothetical protein"
"DsuE09_00002" 3919 "s59E25H2" 0 "hypothetical protein"
"DsuE09_00002" 3919 "s59E5H2" 0 "hypothetical protein"
"DsuE09_00002" 3919 "s63E6H2" 0 "hypothetical protein"
"DsuE09_00003" 3920 "s59E13H2" 0 "dTDP-glucose 4,6-dehydratase"
"DsuE09_00003" 3920 "s59E25H2" 0 "dTDP-glucose 4,6-dehydratase"
"DsuE09_00003" 3920 "s59E5H2" 0 "dTDP-glucose 4,6-dehydratase"
"DsuE09_00003" 3920 "s63E6H2" 0 "dTDP-glucose 4,6-dehydratase"
"DsuE09_00005" 3922 "s59E13H2" 0 "YcfA-like protein"
"DsuE09_00005" 3922 "s59E25H2" 0 "YcfA-like protein"
"DsuE09_00005" 3922 "s59E5H2" 0 "YcfA-like protein"
"DsuE09_00005" 3922 "s63E6H2" 0 "YcfA-like protein"
"DsuE09_00006" 3923 "s59E13H2" 0 "hypothetical protein"
"DsuE09_00006" 3923 "s59E25H2" 0 "hypothetical protein"
"DsuE09_00006" 3923 "s59E5H2" 0 "hypothetical protein"
"DsuE09_00006" 3923 "s63E6H2" 0 "hypothetical protein"
"DsuE09_00007" 3924 "s59E13H2" 0 "hypothetical protein"
"DsuE09_00007" 3924 "s59E25H2" 0 "hypothetical protein"
"DsuE09_00007" 3924 "s59E5H2" 0 "hypothetical protein"
"DsuE09_00007" 3924 "s63E6H2" 0 "hypothetical protein"
"DsuE09_00009" 3926 "s59E13H2" 0 "hypothetical protein"
"DsuE09_00009" 3926 "s59E25H2" 0 "hypothetical protein"
"DsuE09_00009" 3926 "s59E5H2" 0 "hypothetical protein"
"DsuE09_00009" 3926 "s63E6H2" 0 "hypothetical protein"
"DsuE09_00010" 3927 "s59E13H2" 0 "Nucleotidyltransferase domain protein"
"DsuE09_00010" 3927 "s59E25H2" 0 "Nucleotidyltransferase domain protein"
"DsuE09_00010" 3927 "s59E5H2" 0 "Nucleotidyltransferase domain protein"
"DsuE09_00010" 3927 "s63E6H2" 0 "Nucleotidyltransferase domain protein"
"DsuE09_00011" 3928 "s59E13H2" 0 "hypothetical protein"
"DsuE09_00011" 3928 "s59E25H2" 0 "hypothetical protein"
"DsuE09_00011" 3928 "s59E5H2" 0 "hypothetical protein"
"DsuE09_00011" 3928 "s63E6H2" 0 "hypothetical protein"
"DsuE09_00012" 3929 "s59E13H2" 0 "DAK2 domain protein"
"DsuE09_00012" 3929 "s59E25H2" 0 "DAK2 domain protein"
"DsuE09_00012" 3929 "s59E5H2" 0 "DAK2 domain protein"
"DsuE09_00012" 3929 "s63E6H2" 0 "DAK2 domain protein"
"DsuE09_00013" 3930 "s59E13H2" 0 "ATP-dependent DNA helicase RecG"
"DsuE09_00013" 3930 "s59E25H2" 0 "ATP-dependent DNA helicase RecG"
"DsuE09_00013" 3930 "s59E5H2" 0 "ATP-dependent DNA helicase RecG"
"DsuE09_00013" 3930 "s63E6H2" 0 "ATP-dependent DNA helicase RecG"
"DsuE09_00014" 3931 "s59E13H2" 0 "Ribosomal RNA small subunit methyltransferase D"
"DsuE09_00014" 3931 "s59E25H2" 0 "Ribosomal RNA small subunit methyltransferase D"
"DsuE09_00014" 3931 "s59E5H2" 0 "Ribosomal RNA small subunit methyltransferase D"

"DsuE09_00014" 3931 "s63E6H2" 0 "Ribosomal RNA small subunit methyltransferase D"
"DsuE09_00015" 3932 "s59E13H2" 0 "Succinyl-CoA ligase [ADP-forming] subunit alpha"
"DsuE09_00015" 3932 "s59E25H2" 0 "Succinyl-CoA ligase [ADP-forming] subunit alpha"
"DsuE09_00015" 3932 "s59E5H2" 0 "Succinyl-CoA ligase [ADP-forming] subunit alpha"
"DsuE09_00015" 3932 "s63E6H2" 0 "Succinyl-CoA ligase [ADP-forming] subunit alpha"
"DsuE09_00016" 3933 "s59E13H2" 0 "hypothetical protein"
"DsuE09_00016" 3933 "s59E25H2" 0 "hypothetical protein"
"DsuE09_00016" 3933 "s59E5H2" 0 "hypothetical protein"
"DsuE09_00016" 3933 "s63E6H2" 0 "hypothetical protein"
"DsuE09_00017" 3934 "s59E13H2" 0 "Phosphopantetheine adenylyltransferase"
"DsuE09_00017" 3934 "s59E25H2" 0 "Phosphopantetheine adenylyltransferase"
"DsuE09_00017" 3934 "s59E5H2" 0 "Phosphopantetheine adenylyltransferase"
"DsuE09_00017" 3934 "s63E6H2" 0 "Phosphopantetheine adenylyltransferase"
"DsuE09_00018" 3935 "s59E13H2" 0 "Leucine-specific-binding protein precursor"
"DsuE09_00018" 3935 "s59E25H2" 0 "Leucine-specific-binding protein precursor"
"DsuE09_00018" 3935 "s59E5H2" 0 "Leucine-specific-binding protein precursor"
"DsuE09_00018" 3935 "s63E6H2" 0 "Leucine-specific-binding protein precursor"
"DsuE09_00019" 3936 "s59E13H2" 0 "putative branched-chain amino acid transport ATP-binding protein LivG"
"DsuE09_00019" 3936 "s59E25H2" 0 "putative branched-chain amino acid transport ATP-binding protein LivG"
"DsuE09_00019" 3936 "s59E5H2" 0 "putative branched-chain amino acid transport ATP-binding protein LivG"
"DsuE09_00019" 3936 "s63E6H2" 0 "putative branched-chain amino acid transport ATP-binding protein LivG"
"DsuE09_00020" 3937 "s59E13H2" 0 "High-affinity branched-chain amino acid transport ATP-binding protein LivF"
"DsuE09_00020" 3937 "s59E25H2" 0 "High-affinity branched-chain amino acid transport ATP-binding protein LivF"
"DsuE09_00020" 3937 "s59E5H2" 0 "High-affinity branched-chain amino acid transport ATP-binding protein LivF"
"DsuE09_00020" 3937 "s63E6H2" 0 "High-affinity branched-chain amino acid transport ATP-binding protein LivF"
"DsuE09_00021" 3938 "s59E13H2" 0 "High-affinity branched-chain amino acid transport system permease protein LivH"
"DsuE09_00021" 3938 "s59E25H2" 0 "High-affinity branched-chain amino acid transport system permease protein LivH"
"DsuE09_00021" 3938 "s59E5H2" 0 "High-affinity branched-chain amino acid transport system permease protein LivH"
"DsuE09_00021" 3938 "s63E6H2" 0 "High-affinity branched-chain amino acid transport system permease protein LivH"
"DsuE09_00022" 3939 "s59E13H2" 0 "leucine/isoleucine/valine transporter permease subunit"
"DsuE09_00022" 3939 "s59E25H2" 0 "leucine/isoleucine/valine transporter permease subunit"
"DsuE09_00022" 3939 "s59E5H2" 0 "leucine/isoleucine/valine transporter permease subunit"
"DsuE09_00022" 3939 "s63E6H2" 0 "leucine/isoleucine/valine transporter permease subunit"
"DsuE09_00023" 3940 "s59E13H2" 0 "hypothetical protein"
"DsuE09_00023" 3940 "s59E25H2" 0 "hypothetical protein"
"DsuE09_00023" 3940 "s59E5H2" 0 "hypothetical protein"
"DsuE09_00023" 3940 "s63E6H2" 0 "hypothetical protein"
"DsuE09_00024" 3941 "s59E13H2" 0 "Long-chain-fatty-acid--CoA ligase"
"DsuE09_00024" 3941 "s59E25H2" 0 "Long-chain-fatty-acid--CoA ligase"
"DsuE09_00024" 3941 "s59E5H2" 0 "Long-chain-fatty-acid--CoA ligase"
"DsuE09_00024" 3941 "s63E6H2" 0 "Long-chain-fatty-acid--CoA ligase"
"DsuE09_00026" 3943 "s59E13H2" 0 "Leucine-, isoleucine-, valine-, threonine-, and alanine-binding protein precursor"
"DsuE09_00026" 3943 "s59E25H2" 0 "Leucine-, isoleucine-, valine-, threonine-, and alanine-binding protein precursor"
"DsuE09_00026" 3943 "s59E5H2" 0 "Leucine-, isoleucine-, valine-, threonine-, and alanine-binding protein precursor"
"DsuE09_00026" 3943 "s63E6H2" 0 "Leucine-, isoleucine-, valine-, threonine-, and alanine-binding protein precursor"
"DsuE09_00027" 3944 "s59E13H2" 0 "4-hydroxybutyryl-CoA dehydratase/vinylacetyl-CoA-Delta-isomerase"
"DsuE09_00027" 3944 "s59E25H2" 0 "4-hydroxybutyryl-CoA dehydratase/vinylacetyl-CoA-Delta-isomerase"

"DsuE09_00027" 3944 "s59E5H2" 0 "4-hydroxybutyryl-CoA dehydratase/vinylacetyl-CoA-Delta-isomerase"
"DsuE09_00027" 3944 "s63E6H2" 0 "4-hydroxybutyryl-CoA dehydratase/vinylacetyl-CoA-Delta-isomerase"
"DsuE09_00028" 3945 "s59E13H2" 0 "hypothetical protein"
"DsuE09_00028" 3945 "s59E25H2" 0 "hypothetical protein"
"DsuE09_00028" 3945 "s59E5H2" 0 "hypothetical protein"
"DsuE09_00028" 3945 "s63E6H2" 0 "hypothetical protein"
"DsuE09_00029" 3946 "s59E13H2" 0 "Long-chain-fatty-acid--CoA ligase"
"DsuE09_00029" 3946 "s59E25H2" 0 "Long-chain-fatty-acid--CoA ligase"
"DsuE09_00029" 3946 "s59E5H2" 0 "Long-chain-fatty-acid--CoA ligase"
"DsuE09_00029" 3946 "s63E6H2" 0 "Long-chain-fatty-acid--CoA ligase"
"DsuE09_00030" 3947 "s59E13H2" 0 "1,3-propanediol dehydrogenase"
"DsuE09_00030" 3947 "s59E25H2" 0 "1,3-propanediol dehydrogenase"
"DsuE09_00030" 3947 "s59E5H2" 0 "1,3-propanediol dehydrogenase"
"DsuE09_00030" 3947 "s63E6H2" 0 "1,3-propanediol dehydrogenase"
"DsuE09_00031" 3948 "s59E13H2" 0 "NDMA-dependent alcohol dehydrogenase"
"DsuE09_00031" 3948 "s59E25H2" 0 "NDMA-dependent alcohol dehydrogenase"
"DsuE09_00031" 3948 "s59E5H2" 0 "NDMA-dependent alcohol dehydrogenase"
"DsuE09_00031" 3948 "s63E6H2" 0 "NDMA-dependent alcohol dehydrogenase"
"DsuE09_00032" 3949 "s59E13H2" 0 "NDMA-dependent alcohol dehydrogenase"
"DsuE09_00032" 3949 "s59E25H2" 0 "NDMA-dependent alcohol dehydrogenase"
"DsuE09_00032" 3949 "s59E5H2" 0 "NDMA-dependent alcohol dehydrogenase"
"DsuE09_00032" 3949 "s63E6H2" 0 "NDMA-dependent alcohol dehydrogenase"
"DsuE09_00033" 3950 "s59E13H2" 0 "Lipoate-protein ligase A subunit 2"
"DsuE09_00033" 3950 "s59E25H2" 0 "Lipoate-protein ligase A subunit 2"
"DsuE09_00033" 3950 "s59E5H2" 0 "Lipoate-protein ligase A subunit 2"
"DsuE09_00033" 3950 "s63E6H2" 0 "Lipoate-protein ligase A subunit 2"
"DsuE09_00034" 3951 "s59E13H2" 0 "Glyoxalase/Bleomycin resistance protein/Dioxygenase superfamily protein"
"DsuE09_00034" 3951 "s59E25H2" 0 "Glyoxalase/Bleomycin resistance protein/Dioxygenase superfamily protein"
"DsuE09_00034" 3951 "s59E5H2" 0 "Glyoxalase/Bleomycin resistance protein/Dioxygenase superfamily protein"
"DsuE09_00034" 3951 "s63E6H2" 0 "Glyoxalase/Bleomycin resistance protein/Dioxygenase superfamily protein"
"DsuE09_00035" 3952 "s59E13H2" 0 "Octanoyltransferase LipM"
"DsuE09_00035" 3952 "s59E25H2" 0 "Octanoyltransferase LipM"
"DsuE09_00035" 3952 "s59E5H2" 0 "Octanoyltransferase LipM"
"DsuE09_00035" 3952 "s63E6H2" 0 "Octanoyltransferase LipM"
"DsuE09_00036" 3953 "s59E13H2" 0 "Acyl-CoA dehydrogenase"
"DsuE09_00036" 3953 "s59E25H2" 0 "Acyl-CoA dehydrogenase"
"DsuE09_00036" 3953 "s59E5H2" 0 "Acyl-CoA dehydrogenase"
"DsuE09_00036" 3953 "s63E6H2" 0 "Acyl-CoA dehydrogenase"
"DsuE09_00037" 3954 "s59E13H2" 0 "Lipoate-protein ligase A subunit 1"
"DsuE09_00037" 3954 "s59E25H2" 0 "Lipoate-protein ligase A subunit 1"
"DsuE09_00037" 3954 "s59E5H2" 0 "Lipoate-protein ligase A subunit 1"
"DsuE09_00037" 3954 "s63E6H2" 0 "Lipoate-protein ligase A subunit 1"
"DsuE09_00038" 3955 "s59E13H2" 0 "Methylmalonyl-CoA mutase"
"DsuE09_00038" 3955 "s59E25H2" 0 "Methylmalonyl-CoA mutase"
"DsuE09_00038" 3955 "s59E5H2" 0 "Methylmalonyl-CoA mutase"
"DsuE09_00038" 3955 "s63E6H2" 0 "Methylmalonyl-CoA mutase"
"DsuE09_00039" 3956 "s59E13H2" 0 "Methylmalonyl-CoA mutase"

"DsuE09_00039" 3956 "s59E25H2" 0 "Methylmalonyl-CoA mutase"
"DsuE09_00039" 3956 "s59E5H2" 0 "Methylmalonyl-CoA mutase"
"DsuE09_00039" 3956 "s63E6H2" 0 "Methylmalonyl-CoA mutase"
"DsuE09_00042" 3959 "s59E13H2" 0 "Acyl-CoA dehydrogenase"
"DsuE09_00042" 3959 "s59E25H2" 0 "Acyl-CoA dehydrogenase"
"DsuE09_00042" 3959 "s59E5H2" 0 "Acyl-CoA dehydrogenase"
"DsuE09_00042" 3959 "s63E6H2" 0 "Acyl-CoA dehydrogenase"
"DsuE09_00043" 3960 "s59E13H2" 0 "Glutaconate CoA-transferase subunit B"
"DsuE09_00043" 3960 "s59E25H2" 0 "Glutaconate CoA-transferase subunit B"
"DsuE09_00043" 3960 "s59E5H2" 0 "Glutaconate CoA-transferase subunit B"
"DsuE09_00043" 3960 "s63E6H2" 0 "Glutaconate CoA-transferase subunit B"
"DsuE09_00044" 3961 "s59E13H2" 0 "Purine nucleoside phosphorylase"
"DsuE09_00044" 3961 "s59E25H2" 0 "Purine nucleoside phosphorylase"
"DsuE09_00044" 3961 "s59E5H2" 0 "Purine nucleoside phosphorylase"
"DsuE09_00044" 3961 "s63E6H2" 0 "Purine nucleoside phosphorylase"
"DsuE09_00045" 3962 "s59E13H2" 0 "hypothetical protein"
"DsuE09_00045" 3962 "s59E25H2" 0 "hypothetical protein"
"DsuE09_00045" 3962 "s59E5H2" 0 "hypothetical protein"
"DsuE09_00045" 3962 "s63E6H2" 0 "hypothetical protein"
"DsuE09_00046" 3963 "s59E13H2" 0 "Acylphosphatase"
"DsuE09_00046" 3963 "s59E25H2" 0 "Acylphosphatase"
"DsuE09_00046" 3963 "s59E5H2" 0 "Acylphosphatase"
"DsuE09_00046" 3963 "s63E6H2" 0 "Acylphosphatase"
"DsuE09_00047" 3964 "s59E13H2" 0 "hypothetical protein"
"DsuE09_00047" 3964 "s59E25H2" 0 "hypothetical protein"
"DsuE09_00047" 3964 "s59E5H2" 0 "hypothetical protein"
"DsuE09_00047" 3964 "s63E6H2" 0 "hypothetical protein"
"DsuE09_00048" 3965 "s59E13H2" 0 "DNA repair protein RecN"
"DsuE09_00048" 3965 "s59E25H2" 0 "DNA repair protein RecN"
"DsuE09_00048" 3965 "s59E5H2" 0 "DNA repair protein RecN"
"DsuE09_00048" 3965 "s63E6H2" 0 "DNA repair protein RecN"
"DsuE09_00049" 3966 "s59E13H2" 0 "Type II secretion system protein E"
"DsuE09_00049" 3966 "s59E25H2" 0 "Type II secretion system protein E"
"DsuE09_00049" 3966 "s59E5H2" 0 "Type II secretion system protein E"
"DsuE09_00049" 3966 "s63E6H2" 0 "Type II secretion system protein E"
"DsuE09_00050" 3967 "s59E13H2" 0 "Bifunctional protein GlmU"
"DsuE09_00050" 3967 "s59E25H2" 0 "Bifunctional protein GlmU"
"DsuE09_00050" 3967 "s59E5H2" 0 "Bifunctional protein GlmU"
"DsuE09_00050" 3967 "s63E6H2" 0 "Bifunctional protein GlmU"
"DsuE09_00051" 3968 "s59E13H2" 0 "hypothetical protein"
"DsuE09_00051" 3968 "s59E25H2" 0 "hypothetical protein"
"DsuE09_00051" 3968 "s59E5H2" 0 "hypothetical protein"
"DsuE09_00051" 3968 "s63E6H2" 0 "hypothetical protein"
"DsuE09_00052" 3969 "s59E13H2" 0 "Nitronate monooxygenase"
"DsuE09_00052" 3969 "s59E25H2" 0 "Nitronate monooxygenase"
"DsuE09_00052" 3969 "s59E5H2" 0 "Nitronate monooxygenase"
"DsuE09_00052" 3969 "s63E6H2" 0 "Nitronate monooxygenase"

"DsuE09_00053" 3970 "s59E13H2" 0 "tRNA 2'-O-methylase"
"DsuE09_00053" 3970 "s59E25H2" 0 "tRNA 2'-O-methylase"
"DsuE09_00053" 3970 "s59E5H2" 0 "tRNA 2'-O-methylase"
"DsuE09_00053" 3970 "s63E6H2" 0 "tRNA 2'-O-methylase"
"DsuE09_00054" 3971 "s59E13H2" 0 "Primosomal protein N'"
"DsuE09_00054" 3971 "s59E25H2" 0 "Primosomal protein N'"
"DsuE09_00054" 3971 "s59E5H2" 0 "Primosomal protein N'"
"DsuE09_00054" 3971 "s63E6H2" 0 "Primosomal protein N'"
"DsuE09_00055" 3972 "s59E13H2" 0 "hypothetical protein"
"DsuE09_00055" 3972 "s59E25H2" 0 "hypothetical protein"
"DsuE09_00055" 3972 "s59E5H2" 0 "hypothetical protein"
"DsuE09_00055" 3972 "s63E6H2" 0 "hypothetical protein"
"DsuE09_00056" 3973 "s59E13H2" 0 "hypothetical protein"
"DsuE09_00056" 3973 "s59E25H2" 0 "hypothetical protein"
"DsuE09_00056" 3973 "s59E5H2" 0 "hypothetical protein"
"DsuE09_00056" 3973 "s63E6H2" 0 "hypothetical protein"
"DsuE09_00057" 3974 "s59E13H2" 0 "Murein DD-endopeptidase MepM"
"DsuE09_00057" 3974 "s59E25H2" 0 "Murein DD-endopeptidase MepM"
"DsuE09_00057" 3974 "s59E5H2" 0 "Murein DD-endopeptidase MepM"
"DsuE09_00057" 3974 "s63E6H2" 0 "Murein DD-endopeptidase MepM"
"DsuE09_00058" 3975 "s59E13H2" 0 "Ferredoxin"
"DsuE09_00058" 3975 "s59E25H2" 0 "Ferredoxin"
"DsuE09_00058" 3975 "s59E5H2" 0 "Ferredoxin"
"DsuE09_00058" 3975 "s63E6H2" 0 "Ferredoxin"
"DsuE09_00059" 3976 "s59E13H2" 0 "thiosulfate sulfurtransferase"
"DsuE09_00059" 3976 "s59E25H2" 0 "thiosulfate sulfurtransferase"
"DsuE09_00059" 3976 "s59E5H2" 0 "thiosulfate sulfurtransferase"
"DsuE09_00059" 3976 "s63E6H2" 0 "thiosulfate sulfurtransferase"
"DsuE09_00060" 3977 "s59E13H2" 0 "ADP-ribose pyrophosphatase"
"DsuE09_00060" 3977 "s59E25H2" 0 "ADP-ribose pyrophosphatase"
"DsuE09_00060" 3977 "s59E5H2" 0 "ADP-ribose pyrophosphatase"
"DsuE09_00060" 3977 "s63E6H2" 0 "ADP-ribose pyrophosphatase"
"DsuE09_00061" 3978 "s59E13H2" 0 "hypothetical protein"
"DsuE09_00061" 3978 "s59E25H2" 0 "hypothetical protein"
"DsuE09_00061" 3978 "s59E5H2" 0 "hypothetical protein"
"DsuE09_00061" 3978 "s63E6H2" 0 "hypothetical protein"
"DsuE09_00062" 3979 "s59E13H2" 0 "DNA polymerase III subunit alpha"
"DsuE09_00062" 3979 "s59E25H2" 0 "DNA polymerase III subunit alpha"
"DsuE09_00062" 3979 "s59E5H2" 0 "DNA polymerase III subunit alpha"
"DsuE09_00062" 3979 "s63E6H2" 0 "DNA polymerase III subunit alpha"
"DsuE09_00064" 3981 "s59E13H2" 0 "Transcriptional regulatory protein AfsQ1"
"DsuE09_00064" 3981 "s59E25H2" 0 "Transcriptional regulatory protein AfsQ1"
"DsuE09_00064" 3981 "s59E5H2" 0 "Transcriptional regulatory protein AfsQ1"
"DsuE09_00064" 3981 "s63E6H2" 0 "Transcriptional regulatory protein AfsQ1"
"DsuE09_00065" 3982 "s59E13H2" 0 "Replication-associated recombination protein A"
"DsuE09_00065" 3982 "s59E25H2" 0 "Replication-associated recombination protein A"
"DsuE09_00065" 3982 "s59E5H2" 0 "Replication-associated recombination protein A"

"DsuE09_00065" 3982 "s63E6H2" 0 "Replication-associated recombination protein A"
"DsuE09_00066" 3983 "s59E13H2" 0 "Radical SAM superfamily protein"
"DsuE09_00066" 3983 "s59E25H2" 0 "Radical SAM superfamily protein"
"DsuE09_00066" 3983 "s59E5H2" 0 "Radical SAM superfamily protein"
"DsuE09_00066" 3983 "s63E6H2" 0 "Radical SAM superfamily protein"
"DsuE09_00067" 3984 "s59E13H2" 0 "Endonuclease MutS2"
"DsuE09_00067" 3984 "s59E25H2" 0 "Endonuclease MutS2"
"DsuE09_00067" 3984 "s59E5H2" 0 "Endonuclease MutS2"
"DsuE09_00067" 3984 "s63E6H2" 0 "Endonuclease MutS2"
"DsuE09_00069" 3986 "s59E13H2" 0 "Putative arsenical pump-driving ATPase"
"DsuE09_00069" 3986 "s59E25H2" 0 "Putative arsenical pump-driving ATPase"
"DsuE09_00069" 3986 "s59E5H2" 0 "Putative arsenical pump-driving ATPase"
"DsuE09_00069" 3986 "s63E6H2" 0 "Putative arsenical pump-driving ATPase"
"DsuE09_00070" 3987 "s59E13H2" 0 "hypothetical protein"
"DsuE09_00070" 3987 "s59E25H2" 0 "hypothetical protein"
"DsuE09_00070" 3987 "s59E5H2" 0 "hypothetical protein"
"DsuE09_00070" 3987 "s63E6H2" 0 "hypothetical protein"
"DsuE09_00071" 3988 "s59E13H2" 0 "hypothetical protein"
"DsuE09_00071" 3988 "s59E25H2" 0 "hypothetical protein"
"DsuE09_00071" 3988 "s59E5H2" 0 "hypothetical protein"
"DsuE09_00071" 3988 "s63E6H2" 0 "hypothetical protein"
"DsuE09_00072" 3989 "s59E13H2" 0 "3-oxoadipyl-CoA/3-oxo-5,6-dehydrosuberyl-CoA thiolase"
"DsuE09_00072" 3989 "s59E25H2" 0 "3-oxoadipyl-CoA/3-oxo-5,6-dehydrosuberyl-CoA thiolase"
"DsuE09_00072" 3989 "s59E5H2" 0 "3-oxoadipyl-CoA/3-oxo-5,6-dehydrosuberyl-CoA thiolase"
"DsuE09_00072" 3989 "s63E6H2" 0 "3-oxoadipyl-CoA/3-oxo-5,6-dehydrosuberyl-CoA thiolase"
"DsuE09_00073" 3990 "s59E13H2" 0 "hypothetical protein"
"DsuE09_00073" 3990 "s59E25H2" 0 "hypothetical protein"
"DsuE09_00073" 3990 "s59E5H2" 0 "hypothetical protein"
"DsuE09_00073" 3990 "s63E6H2" 0 "hypothetical protein"
"DsuE09_00074" 3991 "s59E13H2" 0 "Caspase domain protein"
"DsuE09_00074" 3991 "s59E25H2" 0 "Caspase domain protein"
"DsuE09_00074" 3991 "s59E5H2" 0 "Caspase domain protein"
"DsuE09_00074" 3991 "s63E6H2" 0 "Caspase domain protein"
"DsuE09_00075" 3992 "s59E13H2" 0 "Serine/threonine-protein kinase pkn1"
"DsuE09_00075" 3992 "s59E25H2" 0 "Serine/threonine-protein kinase pkn1"
"DsuE09_00075" 3992 "s59E5H2" 0 "Serine/threonine-protein kinase pkn1"
"DsuE09_00075" 3992 "s63E6H2" 0 "Serine/threonine-protein kinase pkn1"
"DsuE09_00076" 3993 "s59E13H2" 0 "hypothetical protein"
"DsuE09_00076" 3993 "s59E25H2" 0 "hypothetical protein"
"DsuE09_00076" 3993 "s59E5H2" 0 "hypothetical protein"
"DsuE09_00076" 3993 "s63E6H2" 0 "hypothetical protein"
"DsuE09_00077" 3994 "s59E13H2" 0 "Transcriptional regulatory protein DevR (DosR)"
"DsuE09_00077" 3994 "s59E25H2" 0 "Transcriptional regulatory protein DevR (DosR)"
"DsuE09_00077" 3994 "s59E5H2" 0 "Transcriptional regulatory protein DevR (DosR)"
"DsuE09_00077" 3994 "s63E6H2" 0 "Transcriptional regulatory protein DevR (DosR)"
"DsuE09_00078" 3995 "s59E13H2" 0 "hypothetical protein"
"DsuE09_00078" 3995 "s59E25H2" 0 "hypothetical protein"

"DsuE09_00078" 3995 "s59E5H2" 0 "hypothetical protein"
"DsuE09_00078" 3995 "s63E6H2" 0 "hypothetical protein"
"DsuE09_00079" 3996 "s59E13H2" 0 "Response regulator UvrY"
"DsuE09_00079" 3996 "s59E25H2" 0 "Response regulator UvrY"
"DsuE09_00079" 3996 "s59E5H2" 0 "Response regulator UvrY"
"DsuE09_00079" 3996 "s63E6H2" 0 "Response regulator UvrY"
"DsuE09_00080" 3997 "s59E13H2" 0 "Helix-turn-helix domain protein"
"DsuE09_00080" 3997 "s59E25H2" 0 "Helix-turn-helix domain protein"
"DsuE09_00080" 3997 "s59E5H2" 0 "Helix-turn-helix domain protein"
"DsuE09_00080" 3997 "s63E6H2" 0 "Helix-turn-helix domain protein"
"DsuE09_00081" 3998 "s59E13H2" 0 "Chemotaxis protein methyltransferase"
"DsuE09_00081" 3998 "s59E25H2" 0 "Chemotaxis protein methyltransferase"
"DsuE09_00081" 3998 "s59E5H2" 0 "Chemotaxis protein methyltransferase"
"DsuE09_00081" 3998 "s63E6H2" 0 "Chemotaxis protein methyltransferase"
"DsuE09_00082" 3999 "s59E13H2" 0 "Chemotaxis response regulator protein-glutamate methylesterase"
"DsuE09_00082" 3999 "s59E25H2" 0 "Chemotaxis response regulator protein-glutamate methylesterase"
"DsuE09_00082" 3999 "s59E5H2" 0 "Chemotaxis response regulator protein-glutamate methylesterase"
"DsuE09_00082" 3999 "s63E6H2" 0 "Chemotaxis response regulator protein-glutamate methylesterase"
"DsuE09_00084" 4001 "s59E13H2" 0 "Phytochrome-like protein cph1"
"DsuE09_00084" 4001 "s59E25H2" 0 "Phytochrome-like protein cph1"
"DsuE09_00084" 4001 "s59E5H2" 0 "Phytochrome-like protein cph1"
"DsuE09_00084" 4001 "s63E6H2" 0 "Phytochrome-like protein cph1"
"DsuE09_00085" 4002 "s59E13H2" 0 "Response regulator rcp1"
"DsuE09_00085" 4002 "s59E25H2" 0 "Response regulator rcp1"
"DsuE09_00085" 4002 "s59E5H2" 0 "Response regulator rcp1"
"DsuE09_00085" 4002 "s63E6H2" 0 "Response regulator rcp1"
"DsuE09_00086" 4003 "s59E13H2" 0 "hypothetical protein"
"DsuE09_00086" 4003 "s59E25H2" 0 "hypothetical protein"
"DsuE09_00086" 4003 "s59E5H2" 0 "hypothetical protein"
"DsuE09_00086" 4003 "s63E6H2" 0 "hypothetical protein"
"DsuE09_00087" 4004 "s59E13H2" 0 "SpoVT / AbrB like domain protein"
"DsuE09_00087" 4004 "s59E25H2" 0 "SpoVT / AbrB like domain protein"
"DsuE09_00087" 4004 "s59E5H2" 0 "SpoVT / AbrB like domain protein"
"DsuE09_00087" 4004 "s63E6H2" 0 "SpoVT / AbrB like domain protein"
"DsuE09_00088" 4005 "s59E13H2" 0 "Luminescence regulatory protein LuxO"
"DsuE09_00088" 4005 "s59E25H2" 0 "Luminescence regulatory protein LuxO"
"DsuE09_00088" 4005 "s59E5H2" 0.223765 "Luminescence regulatory protein LuxO"
"DsuE09_00088" 4005 "s63E6H2" 0.001554011 "Luminescence regulatory protein LuxO"
"DsuE09_00089" 4006 "s59E13H2" 0 "Circadian clock protein KaiB"
"DsuE09_00089" 4006 "s59E25H2" 0 "Circadian clock protein KaiB"
"DsuE09_00089" 4006 "s59E5H2" 0.4890807 "Circadian clock protein KaiB"
"DsuE09_00089" 4006 "s63E6H2" 0 "Circadian clock protein KaiB"
"DsuE09_00090" 4007 "s59E13H2" 0 "Circadian clock protein kinase KaiC"
"DsuE09_00090" 4007 "s59E25H2" 0 "Circadian clock protein kinase KaiC"
"DsuE09_00090" 4007 "s59E5H2" 0.3309976 "Circadian clock protein kinase KaiC"
"DsuE09_00090" 4007 "s63E6H2" 0.0056098 "Circadian clock protein kinase KaiC"
"DsuE09_00092" 4009 "s59E13H2" 0 "hypothetical protein"

"DsuE09_00092" 4009 "s59E25H2" 0 "hypothetical protein"
"DsuE09_00092" 4009 "s59E5H2" 0.0170594 "hypothetical protein"
"DsuE09_00092" 4009 "s63E6H2" 0 "hypothetical protein"
"DsuE09_00094" 4011 "s59E13H2" 0 "Non-motile and phage-resistance protein"
"DsuE09_00094" 4011 "s59E25H2" 0 "Non-motile and phage-resistance protein"
"DsuE09_00094" 4011 "s59E5H2" 0.0342117 "Non-motile and phage-resistance protein"
"DsuE09_00094" 4011 "s63E6H2" 0 "Non-motile and phage-resistance protein"
"DsuE09_00095" 4012 "s59E13H2" 0 "PemK-like protein"
"DsuE09_00095" 4012 "s59E25H2" 0 "PemK-like protein"
"DsuE09_00095" 4012 "s59E5H2" 0 "PemK-like protein"
"DsuE09_00095" 4012 "s63E6H2" 0 "PemK-like protein"
"DsuE09_00096" 4013 "s59E13H2" 0 "hypothetical protein"
"DsuE09_00096" 4013 "s59E25H2" 0 "hypothetical protein"
"DsuE09_00096" 4013 "s59E5H2" 0 "hypothetical protein"
"DsuE09_00096" 4013 "s63E6H2" 0 "hypothetical protein"
"DsuE09_00097" 4014 "s59E13H2" 0.002334091 "putative homocitrate synthase AksA"
"DsuE09_00097" 4014 "s59E25H2" 0.5973154 "putative homocitrate synthase AksA"
"DsuE09_00097" 4014 "s59E5H2" 0 "putative homocitrate synthase AksA"
"DsuE09_00097" 4014 "s63E6H2" 0 "putative homocitrate synthase AksA"
"DsuE09_00098" 4015 "s59E13H2" 0 "metal-dependent hydrolase"
"DsuE09_00098" 4015 "s59E25H2" 0 "metal-dependent hydrolase"
"DsuE09_00098" 4015 "s59E5H2" 0 "metal-dependent hydrolase"
"DsuE09_00098" 4015 "s63E6H2" 0 "metal-dependent hydrolase"
"DsuE09_00099" 4016 "s59E13H2" 0 "Transposase DDE domain protein"
"DsuE09_00099" 4016 "s59E25H2" 0 "Transposase DDE domain protein"
"DsuE09_00099" 4016 "s59E5H2" 0 "Transposase DDE domain protein"
"DsuE09_00099" 4016 "s63E6H2" 0 "Transposase DDE domain protein"
"DsuE09_00100" 4017 "s59E13H2" 0 "Tripartite tricarboxylate transporter family receptor"
"DsuE09_00100" 4017 "s59E25H2" 0 "Tripartite tricarboxylate transporter family receptor"
"DsuE09_00100" 4017 "s59E5H2" 0 "Tripartite tricarboxylate transporter family receptor"
"DsuE09_00100" 4017 "s63E6H2" 0 "Tripartite tricarboxylate transporter family receptor"
"DsuE09_00101" 4018 "s59E13H2" 0 "Tripartite tricarboxylate transporter TctB family protein"
"DsuE09_00101" 4018 "s59E25H2" 0 "Tripartite tricarboxylate transporter TctB family protein"
"DsuE09_00101" 4018 "s59E5H2" 0 "Tripartite tricarboxylate transporter TctB family protein"
"DsuE09_00101" 4018 "s63E6H2" 0 "Tripartite tricarboxylate transporter TctB family protein"
"DsuE09_00103" 4020 "s59E13H2" 0 "ISXO2-like transposase domain protein"
"DsuE09_00103" 4020 "s59E25H2" 0 "ISXO2-like transposase domain protein"
"DsuE09_00103" 4020 "s59E5H2" 0 "ISXO2-like transposase domain protein"
"DsuE09_00103" 4020 "s63E6H2" 0 "ISXO2-like transposase domain protein"
"DsuE09_00104" 4021 "s59E13H2" 0 "Transposase zinc-ribbon domain protein"
"DsuE09_00104" 4021 "s59E25H2" 0 "Transposase zinc-ribbon domain protein"
"DsuE09_00104" 4021 "s59E5H2" 0 "Transposase zinc-ribbon domain protein"
"DsuE09_00104" 4021 "s63E6H2" 0 "Transposase zinc-ribbon domain protein"
"DsuE09_00105" 4022 "s59E13H2" 0 "Tripartite tricarboxylate transporter TctA family protein"
"DsuE09_00105" 4022 "s59E25H2" 0 "Tripartite tricarboxylate transporter TctA family protein"
"DsuE09_00105" 4022 "s59E5H2" 0 "Tripartite tricarboxylate transporter TctA family protein"
"DsuE09_00105" 4022 "s63E6H2" 0 "Tripartite tricarboxylate transporter TctA family protein"

"DsuE09_00106" 4023 "s59E13H2" 0 "hypothetical protein"
"DsuE09_00106" 4023 "s59E25H2" 0 "hypothetical protein"
"DsuE09_00106" 4023 "s59E5H2" 0.003216614 "hypothetical protein"
"DsuE09_00106" 4023 "s63E6H2" 0 "hypothetical protein"
"DsuE09_00107" 4024 "s59E13H2" 0 "hypothetical protein"
"DsuE09_00107" 4024 "s59E25H2" 0 "hypothetical protein"
"DsuE09_00107" 4024 "s59E5H2" 0 "hypothetical protein"
"DsuE09_00107" 4024 "s63E6H2" 0 "hypothetical protein"
"DsuE09_00109" 4026 "s59E13H2" 0 "hypothetical protein"
"DsuE09_00109" 4026 "s59E25H2" 0 "hypothetical protein"
"DsuE09_00109" 4026 "s59E5H2" 0 "hypothetical protein"
"DsuE09_00109" 4026 "s63E6H2" 0 "hypothetical protein"
"DsuE09_00110" 4027 "s59E13H2" 0 "Nucleotidyltransferase domain protein"
"DsuE09_00110" 4027 "s59E25H2" 0 "Nucleotidyltransferase domain protein"
"DsuE09_00110" 4027 "s59E5H2" 0 "Nucleotidyltransferase domain protein"
"DsuE09_00110" 4027 "s63E6H2" 0 "Nucleotidyltransferase domain protein"
"DsuE09_00111" 4028 "s59E13H2" 0 "hypothetical protein"
"DsuE09_00111" 4028 "s59E25H2" 0 "hypothetical protein"
"DsuE09_00111" 4028 "s59E5H2" 0 "hypothetical protein"
"DsuE09_00111" 4028 "s63E6H2" 0 "hypothetical protein"
"DsuE09_00113" 4030 "s59E13H2" 0 "hypothetical protein"
"DsuE09_00113" 4030 "s59E25H2" 0 "hypothetical protein"
"DsuE09_00113" 4030 "s59E5H2" 0 "hypothetical protein"
"DsuE09_00113" 4030 "s63E6H2" 0 "hypothetical protein"
"DsuE09_00114" 4031 "s59E13H2" 0 "hypothetical protein"
"DsuE09_00114" 4031 "s59E25H2" 0 "hypothetical protein"
"DsuE09_00114" 4031 "s59E5H2" 0 "hypothetical protein"
"DsuE09_00114" 4031 "s63E6H2" 0 "hypothetical protein"
"DsuE09_00115" 4032 "s59E13H2" 0 "UDP-N-acetyl-alpha-D-glucosamine C6 dehydratase"
"DsuE09_00115" 4032 "s59E25H2" 0 "UDP-N-acetyl-alpha-D-glucosamine C6 dehydratase"
"DsuE09_00115" 4032 "s59E5H2" 0 "UDP-N-acetyl-alpha-D-glucosamine C6 dehydratase"
"DsuE09_00115" 4032 "s63E6H2" 0 "UDP-N-acetyl-alpha-D-glucosamine C6 dehydratase"
"DsuE09_00116" 4033 "s59E13H2" 0 "hypothetical protein"
"DsuE09_00116" 4033 "s59E25H2" 0 "hypothetical protein"
"DsuE09_00116" 4033 "s59E5H2" 0 "hypothetical protein"
"DsuE09_00116" 4033 "s63E6H2" 0 "hypothetical protein"
"DsuE09_00117" 4034 "s59E13H2" 0 "hypothetical protein"
"DsuE09_00117" 4034 "s59E25H2" 0 "hypothetical protein"
"DsuE09_00117" 4034 "s59E5H2" 0 "hypothetical protein"
"DsuE09_00117" 4034 "s63E6H2" 0 "hypothetical protein"
"DsuE09_00118" 4035 "s59E13H2" 0 "hypothetical protein"
"DsuE09_00118" 4035 "s59E25H2" 0 "hypothetical protein"
"DsuE09_00118" 4035 "s59E5H2" 0 "hypothetical protein"
"DsuE09_00118" 4035 "s63E6H2" 0 "hypothetical protein"
"DsuE09_00119" 4036 "s59E13H2" 0 "HEPN domain protein"
"DsuE09_00119" 4036 "s59E25H2" 0 "HEPN domain protein"
"DsuE09_00119" 4036 "s59E5H2" 0 "HEPN domain protein"

"DsuE09_00119" 4036 "s63E6H2" 0 "HEPN domain protein"
"DsuE09_00120" 4037 "s59E13H2" 0 "Nucleotidyltransferase domain protein"
"DsuE09_00120" 4037 "s59E25H2" 0 "Nucleotidyltransferase domain protein"
"DsuE09_00120" 4037 "s59E5H2" 0 "Nucleotidyltransferase domain protein"
"DsuE09_00120" 4037 "s63E6H2" 0 "Nucleotidyltransferase domain protein"
"DsuE09_00121" 4038 "s59E13H2" 0 "putative HTH-type transcriptional regulator YbaQ"
"DsuE09_00121" 4038 "s59E25H2" 0 "putative HTH-type transcriptional regulator YbaQ"
"DsuE09_00121" 4038 "s59E5H2" 0 "putative HTH-type transcriptional regulator YbaQ"
"DsuE09_00121" 4038 "s63E6H2" 0 "putative HTH-type transcriptional regulator YbaQ"
"DsuE09_00122" 4039 "s59E13H2" 0 "Toxin HigB-1"
"DsuE09_00122" 4039 "s59E25H2" 0 "Toxin HigB-1"
"DsuE09_00122" 4039 "s59E5H2" 0 "Toxin HigB-1"
"DsuE09_00122" 4039 "s63E6H2" 0 "Toxin HigB-1"
"DsuE09_00124" 4041 "s59E13H2" 0 "hypothetical protein"
"DsuE09_00124" 4041 "s59E25H2" 0 "hypothetical protein"
"DsuE09_00124" 4041 "s59E5H2" 0 "hypothetical protein"
"DsuE09_00124" 4041 "s63E6H2" 0 "hypothetical protein"
"DsuE09_00126" 4043 "s59E13H2" 0 "hypothetical protein"
"DsuE09_00126" 4043 "s59E25H2" 0 "hypothetical protein"
"DsuE09_00126" 4043 "s59E5H2" 0 "hypothetical protein"
"DsuE09_00126" 4043 "s63E6H2" 0 "hypothetical protein"
"DsuE09_00127" 4044 "s59E13H2" 0 "hypothetical protein"
"DsuE09_00127" 4044 "s59E25H2" 0 "hypothetical protein"
"DsuE09_00127" 4044 "s59E5H2" 0 "hypothetical protein"
"DsuE09_00127" 4044 "s63E6H2" 0 "hypothetical protein"
"DsuE09_00128" 4045 "s59E13H2" 0 "hypothetical protein"
"DsuE09_00128" 4045 "s59E25H2" 0 "hypothetical protein"
"DsuE09_00128" 4045 "s59E5H2" 0 "hypothetical protein"
"DsuE09_00128" 4045 "s63E6H2" 0 "hypothetical protein"
"DsuE09_00129" 4046 "s59E13H2" 0 "hypothetical protein"
"DsuE09_00129" 4046 "s59E25H2" 0 "hypothetical protein"
"DsuE09_00129" 4046 "s59E5H2" 0 "hypothetical protein"
"DsuE09_00129" 4046 "s63E6H2" 0 "hypothetical protein"
"DsuE09_00130" 4047 "s59E13H2" 0 "hypothetical protein"
"DsuE09_00130" 4047 "s59E25H2" 0 "hypothetical protein"
"DsuE09_00130" 4047 "s59E5H2" 0 "hypothetical protein"
"DsuE09_00130" 4047 "s63E6H2" 0 "hypothetical protein"
"DsuE09_00131" 4048 "s59E13H2" 0 "YcfA-like protein"
"DsuE09_00131" 4048 "s59E25H2" 0 "YcfA-like protein"
"DsuE09_00131" 4048 "s59E5H2" 0 "YcfA-like protein"
"DsuE09_00131" 4048 "s63E6H2" 0.005821019 "YcfA-like protein"
"DsuE09_00133" 4050 "s59E13H2" 0 "UDP-N-acetyl-alpha-D-glucosamine C6 dehydratase"
"DsuE09_00133" 4050 "s59E25H2" 0 "UDP-N-acetyl-alpha-D-glucosamine C6 dehydratase"
"DsuE09_00133" 4050 "s59E5H2" 0 "UDP-N-acetyl-alpha-D-glucosamine C6 dehydratase"
"DsuE09_00133" 4050 "s63E6H2" 0 "UDP-N-acetyl-alpha-D-glucosamine C6 dehydratase"
"DsuE09_00134" 4051 "s59E13H2" 0 "hypothetical protein"
"DsuE09_00134" 4051 "s59E25H2" 0 "hypothetical protein"

"DsuE09_00134" 4051 "s59E5H2" 0 "hypothetical protein"
"DsuE09_00134" 4051 "s63E6H2" 0 "hypothetical protein"
"DsuE09_00135" 4052 "s59E13H2" 0 "Nucleotidyltransferase domain protein"
"DsuE09_00135" 4052 "s59E25H2" 0 "Nucleotidyltransferase domain protein"
"DsuE09_00135" 4052 "s59E5H2" 0 "Nucleotidyltransferase domain protein"
"DsuE09_00135" 4052 "s63E6H2" 0 "Nucleotidyltransferase domain protein"
"DsuE09_00136" 4053 "s59E13H2" 0 "hypothetical protein"
"DsuE09_00136" 4053 "s59E25H2" 0 "hypothetical protein"
"DsuE09_00136" 4053 "s59E5H2" 0 "hypothetical protein"
"DsuE09_00136" 4053 "s63E6H2" 0 "hypothetical protein"
"DsuE09_00137" 4054 "s59E13H2" 0 "hypothetical protein"
"DsuE09_00137" 4054 "s59E25H2" 0 "hypothetical protein"
"DsuE09_00137" 4054 "s59E5H2" 0 "hypothetical protein"
"DsuE09_00137" 4054 "s63E6H2" 0 "hypothetical protein"
"DsuE09_00138" 4055 "s59E13H2" 0 "hypothetical protein"
"DsuE09_00138" 4055 "s59E25H2" 0 "hypothetical protein"
"DsuE09_00138" 4055 "s59E5H2" 0 "hypothetical protein"
"DsuE09_00138" 4055 "s63E6H2" 0 "hypothetical protein"
"DsuE09_00139" 4056 "s59E13H2" 0 "hypothetical protein"
"DsuE09_00139" 4056 "s59E25H2" 0 "hypothetical protein"
"DsuE09_00139" 4056 "s59E5H2" 0 "hypothetical protein"
"DsuE09_00139" 4056 "s63E6H2" 0 "hypothetical protein"
"DsuE09_00141" 4058 "s59E13H2" 0 "hypothetical protein"
"DsuE09_00141" 4058 "s59E25H2" 0 "hypothetical protein"
"DsuE09_00141" 4058 "s59E5H2" 0 "hypothetical protein"
"DsuE09_00141" 4058 "s63E6H2" 0 "hypothetical protein"
"DsuE09_00142" 4059 "s59E13H2" 0 "hypothetical protein"
"DsuE09_00142" 4059 "s59E25H2" 0 "hypothetical protein"
"DsuE09_00142" 4059 "s59E5H2" 0 "hypothetical protein"
"DsuE09_00142" 4059 "s63E6H2" 0 "hypothetical protein"
"DsuE09_00144" 4061 "s59E13H2" 0 "hypothetical protein"
"DsuE09_00144" 4061 "s59E25H2" 0 "hypothetical protein"
"DsuE09_00144" 4061 "s59E5H2" 0 "hypothetical protein"
"DsuE09_00144" 4061 "s63E6H2" 0 "hypothetical protein"
"DsuE09_00146" 4063 "s59E13H2" 0 "10 kDa chaperonin 1"
"DsuE09_00146" 4063 "s59E25H2" 0 "10 kDa chaperonin 1"
"DsuE09_00146" 4063 "s59E5H2" 0 "10 kDa chaperonin 1"
"DsuE09_00146" 4063 "s63E6H2" 0 "10 kDa chaperonin 1"
"DsuE09_00148" 4065 "s59E13H2" 0 "Transcriptional regulatory protein ZraR"
"DsuE09_00148" 4065 "s59E25H2" 0 "Transcriptional regulatory protein ZraR"
"DsuE09_00148" 4065 "s59E5H2" 0 "Transcriptional regulatory protein ZraR"
"DsuE09_00148" 4065 "s63E6H2" 0 "Transcriptional regulatory protein ZraR"
"DsuE09_00151" 4068 "s59E13H2" 0 "L-lysine 2,3-aminomutase"
"DsuE09_00151" 4068 "s59E25H2" 0 "L-lysine 2,3-aminomutase"
"DsuE09_00151" 4068 "s59E5H2" 0 "L-lysine 2,3-aminomutase"
"DsuE09_00151" 4068 "s63E6H2" 0 "L-lysine 2,3-aminomutase"
"DsuE09_00152" 4069 "s59E13H2" 0 "D-alanine--D-alanine ligase"

"DsuE09_00152" 4069 "s59E25H2" 0 "D-alanine--D-alanine ligase"
"DsuE09_00152" 4069 "s59E5H2" 0 "D-alanine--D-alanine ligase"
"DsuE09_00152" 4069 "s63E6H2" 0 "D-alanine--D-alanine ligase"
"DsuE09_00153" 4070 "s59E13H2" 0 "D-alanine--D-alanine ligase"
"DsuE09_00153" 4070 "s59E25H2" 0 "D-alanine--D-alanine ligase"
"DsuE09_00153" 4070 "s59E5H2" 0 "D-alanine--D-alanine ligase"
"DsuE09_00153" 4070 "s63E6H2" 0 "D-alanine--D-alanine ligase"
"DsuE09_00154" 4071 "s59E13H2" 0 "Acetyltransferase (GNAT) family protein"
"DsuE09_00154" 4071 "s59E25H2" 0 "Acetyltransferase (GNAT) family protein"
"DsuE09_00154" 4071 "s59E5H2" 0 "Acetyltransferase (GNAT) family protein"
"DsuE09_00154" 4071 "s63E6H2" 0 "Acetyltransferase (GNAT) family protein"
"DsuE09_00155" 4072 "s59E13H2" 0 "hypothetical protein"
"DsuE09_00155" 4072 "s59E25H2" 0 "hypothetical protein"
"DsuE09_00155" 4072 "s59E5H2" 0 "hypothetical protein"
"DsuE09_00155" 4072 "s63E6H2" 0 "hypothetical protein"
"DsuE09_00156" 4073 "s59E13H2" 0 "Diacylglycerol kinase"
"DsuE09_00156" 4073 "s59E25H2" 0 "Diacylglycerol kinase"
"DsuE09_00156" 4073 "s59E5H2" 0 "Diacylglycerol kinase"
"DsuE09_00156" 4073 "s63E6H2" 0 "Diacylglycerol kinase"
"DsuE09_00157" 4074 "s59E13H2" 0 "hypothetical protein"
"DsuE09_00157" 4074 "s59E25H2" 0 "hypothetical protein"
"DsuE09_00157" 4074 "s59E5H2" 0 "hypothetical protein"
"DsuE09_00157" 4074 "s63E6H2" 0 "hypothetical protein"
"DsuE09_00158" 4075 "s59E13H2" 0 "Inner membrane ABC transporter permease protein YcjP"
"DsuE09_00158" 4075 "s59E25H2" 0 "Inner membrane ABC transporter permease protein YcjP"
"DsuE09_00158" 4075 "s59E5H2" 0 "Inner membrane ABC transporter permease protein YcjP"
"DsuE09_00158" 4075 "s63E6H2" 0 "Inner membrane ABC transporter permease protein YcjP"
"DsuE09_00159" 4076 "s59E13H2" 0 "Inner membrane ABC transporter permease protein YcjO"
"DsuE09_00159" 4076 "s59E25H2" 0 "Inner membrane ABC transporter permease protein YcjO"
"DsuE09_00159" 4076 "s59E5H2" 0 "Inner membrane ABC transporter permease protein YcjO"
"DsuE09_00159" 4076 "s63E6H2" 0 "Inner membrane ABC transporter permease protein YcjO"
"DsuE09_00160" 4077 "s59E13H2" 0 "Bacterial extracellular solute-binding protein"
"DsuE09_00160" 4077 "s59E25H2" 0 "Bacterial extracellular solute-binding protein"
"DsuE09_00160" 4077 "s59E5H2" 0 "Bacterial extracellular solute-binding protein"
"DsuE09_00160" 4077 "s63E6H2" 0 "Bacterial extracellular solute-binding protein"
"DsuE09_00161" 4078 "s59E13H2" 0 "NAD-dependent protein deacylase 2"
"DsuE09_00161" 4078 "s59E25H2" 0 "NAD-dependent protein deacylase 2"
"DsuE09_00161" 4078 "s59E5H2" 0 "NAD-dependent protein deacylase 2"
"DsuE09_00161" 4078 "s63E6H2" 0 "NAD-dependent protein deacylase 2"
"DsuE09_00162" 4079 "s59E13H2" 0 "putative nicotinate-nucleotide pyrophosphorylase [carboxylating]"
"DsuE09_00162" 4079 "s59E25H2" 0 "putative nicotinate-nucleotide pyrophosphorylase [carboxylating]"
"DsuE09_00162" 4079 "s59E5H2" 0 "putative nicotinate-nucleotide pyrophosphorylase [carboxylating]"
"DsuE09_00162" 4079 "s63E6H2" 0 "putative nicotinate-nucleotide pyrophosphorylase [carboxylating]"
"DsuE09_00163" 4080 "s59E13H2" 0 "Valine--tRNA ligase"
"DsuE09_00163" 4080 "s59E25H2" 0 "Valine--tRNA ligase"
"DsuE09_00163" 4080 "s59E5H2" 0 "Valine--tRNA ligase"
"DsuE09_00163" 4080 "s63E6H2" 0 "Valine--tRNA ligase"

"DsuE09_00164" 4081 "s59E13H2" 0 "hypothetical protein"
"DsuE09_00164" 4081 "s59E25H2" 0 "hypothetical protein"
"DsuE09_00164" 4081 "s59E5H2" 0 "hypothetical protein"
"DsuE09_00164" 4081 "s63E6H2" 0 "hypothetical protein"
"DsuE09_00165" 4082 "s59E13H2" 0 "putative acyl-CoA dehydrogenase"
"DsuE09_00165" 4082 "s59E25H2" 0 "putative acyl-CoA dehydrogenase"
"DsuE09_00165" 4082 "s59E5H2" 0 "putative acyl-CoA dehydrogenase"
"DsuE09_00165" 4082 "s63E6H2" 0 "putative acyl-CoA dehydrogenase"
"DsuE09_00166" 4083 "s59E13H2" 0 "Archaeal Lon protease"
"DsuE09_00166" 4083 "s59E25H2" 0 "Archaeal Lon protease"
"DsuE09_00166" 4083 "s59E5H2" 0 "Archaeal Lon protease"
"DsuE09_00166" 4083 "s63E6H2" 0 "Archaeal Lon protease"
"DsuE09_00168" 4085 "s59E13H2" 0 "hypothetical protein"
"DsuE09_00168" 4085 "s59E25H2" 0 "hypothetical protein"
"DsuE09_00168" 4085 "s59E5H2" 0 "hypothetical protein"
"DsuE09_00168" 4085 "s63E6H2" 0 "hypothetical protein"
"DsuE09_00169" 4086 "s59E13H2" 0 "hypothetical protein"
"DsuE09_00169" 4086 "s59E25H2" 0 "hypothetical protein"
"DsuE09_00169" 4086 "s59E5H2" 0 "hypothetical protein"
"DsuE09_00169" 4086 "s63E6H2" 0 "hypothetical protein"
"DsuE09_00170" 4087 "s59E13H2" 0 "Ribosomal RNA small subunit methyltransferase E"
"DsuE09_00170" 4087 "s59E25H2" 0 "Ribosomal RNA small subunit methyltransferase E"
"DsuE09_00170" 4087 "s59E5H2" 0 "Ribosomal RNA small subunit methyltransferase E"
"DsuE09_00170" 4087 "s63E6H2" 0.002190103 "Ribosomal RNA small subunit methyltransferase E"
"DsuE09_00177" 4094 "s59E13H2" 0 "Corrinoid/iron-sulfur protein small subunit"
"DsuE09_00177" 4094 "s59E25H2" 0 "Corrinoid/iron-sulfur protein small subunit"
"DsuE09_00177" 4094 "s59E5H2" 0 "Corrinoid/iron-sulfur protein small subunit"
"DsuE09_00177" 4094 "s63E6H2" 0 "Corrinoid/iron-sulfur protein small subunit"
"DsuE09_00178" 4095 "s59E13H2" 0 "Carbon monoxide dehydrogenase 1"
"DsuE09_00178" 4095 "s59E25H2" 0 "Carbon monoxide dehydrogenase 1"
"DsuE09_00178" 4095 "s59E5H2" 0 "Carbon monoxide dehydrogenase 1"
"DsuE09_00178" 4095 "s63E6H2" 0 "Carbon monoxide dehydrogenase 1"
"DsuE09_00179" 4096 "s59E13H2" 0 "Carbon monoxide dehydrogenase/acetyl-CoA synthase subunit alpha"
"DsuE09_00179" 4096 "s59E25H2" 0 "Carbon monoxide dehydrogenase/acetyl-CoA synthase subunit alpha"
"DsuE09_00179" 4096 "s59E5H2" 0 "Carbon monoxide dehydrogenase/acetyl-CoA synthase subunit alpha"
"DsuE09_00179" 4096 "s63E6H2" 0 "Carbon monoxide dehydrogenase/acetyl-CoA synthase subunit alpha"
"DsuE09_00180" 4097 "s59E13H2" 0 "Carbon monoxide dehydrogenase/acetyl-CoA synthase subunit alpha"
"DsuE09_00180" 4097 "s59E25H2" 0 "Carbon monoxide dehydrogenase/acetyl-CoA synthase subunit alpha"
"DsuE09_00180" 4097 "s59E5H2" 0 "Carbon monoxide dehydrogenase/acetyl-CoA synthase subunit alpha"
"DsuE09_00180" 4097 "s63E6H2" 0 "Carbon monoxide dehydrogenase/acetyl-CoA synthase subunit alpha"
"DsuE09_00181" 4098 "s59E13H2" 0 "Corrinoid/iron-sulfur protein large subunit"
"DsuE09_00181" 4098 "s59E25H2" 0 "Corrinoid/iron-sulfur protein large subunit"
"DsuE09_00181" 4098 "s59E5H2" 0 "Corrinoid/iron-sulfur protein large subunit"
"DsuE09_00181" 4098 "s63E6H2" 0 "Corrinoid/iron-sulfur protein large subunit"
"DsuE09_00182" 4099 "s59E13H2" 0 "5-methyltetrahydrofolate:corrinoid/iron-sulfur protein co-methyltransferase"
"DsuE09_00182" 4099 "s59E25H2" 0 "5-methyltetrahydrofolate:corrinoid/iron-sulfur protein co-methyltransferase"
"DsuE09_00182" 4099 "s59E5H2" 0 "5-methyltetrahydrofolate:corrinoid/iron-sulfur protein co-methyltransferase"

"DsuE09_00182" 4099 "s63E6H2" 0 "5-methyltetrahydrofolate:corrinoid/iron-sulfur protein co-methyltransferase"
"DsuE09_00183" 4100 "s59E13H2" 0 "ComEC family competence protein"
"DsuE09_00183" 4100 "s59E25H2" 0 "ComEC family competence protein"
"DsuE09_00183" 4100 "s59E5H2" 0 "ComEC family competence protein"
"DsuE09_00183" 4100 "s63E6H2" 0 "ComEC family competence protein"
"DsuE09_00184" 4101 "s59E13H2" 0 "UDP-N-acetylglucosamine 1-carboxyvinyltransferase"
"DsuE09_00184" 4101 "s59E25H2" 0 "UDP-N-acetylglucosamine 1-carboxyvinyltransferase"
"DsuE09_00184" 4101 "s59E5H2" 0 "UDP-N-acetylglucosamine 1-carboxyvinyltransferase"
"DsuE09_00184" 4101 "s63E6H2" 0 "UDP-N-acetylglucosamine 1-carboxyvinyltransferase"
"DsuE09_00185" 4102 "s59E13H2" 0 "Release factor glutamine methyltransferase"
"DsuE09_00185" 4102 "s59E25H2" 0 "Release factor glutamine methyltransferase"
"DsuE09_00185" 4102 "s59E5H2" 0 "Release factor glutamine methyltransferase"
"DsuE09_00185" 4102 "s63E6H2" 0 "Release factor glutamine methyltransferase"
"DsuE09_00186" 4103 "s59E13H2" 0 "Peptide chain release factor 1"
"DsuE09_00186" 4103 "s59E25H2" 0 "Peptide chain release factor 1"
"DsuE09_00186" 4103 "s59E5H2" 0 "Peptide chain release factor 1"
"DsuE09_00186" 4103 "s63E6H2" 0 "Peptide chain release factor 1"
"DsuE09_00187" 4104 "s59E13H2" 0 "50S ribosomal protein L31"
"DsuE09_00187" 4104 "s59E25H2" 0 "50S ribosomal protein L31"
"DsuE09_00187" 4104 "s59E5H2" 0 "50S ribosomal protein L31"
"DsuE09_00187" 4104 "s63E6H2" 0 "50S ribosomal protein L31"
"DsuE09_00188" 4105 "s59E13H2" 0 "hypothetical protein"
"DsuE09_00188" 4105 "s59E25H2" 0 "hypothetical protein"
"DsuE09_00188" 4105 "s59E5H2" 0 "hypothetical protein"
"DsuE09_00188" 4105 "s63E6H2" 0 "hypothetical protein"
"DsuE09_00189" 4106 "s59E13H2" 0 "hypothetical protein"
"DsuE09_00189" 4106 "s59E25H2" 0 "hypothetical protein"
"DsuE09_00189" 4106 "s59E5H2" 0 "hypothetical protein"
"DsuE09_00189" 4106 "s63E6H2" 0 "hypothetical protein"
"DsuE09_00190" 4107 "s59E13H2" 0 "Helix-turn-helix domain protein"
"DsuE09_00190" 4107 "s59E25H2" 0 "Helix-turn-helix domain protein"
"DsuE09_00190" 4107 "s59E5H2" 0 "Helix-turn-helix domain protein"
"DsuE09_00190" 4107 "s63E6H2" 0 "Helix-turn-helix domain protein"
"DsuE09_00191" 4108 "s59E13H2" 0 "hypothetical protein"
"DsuE09_00191" 4108 "s59E25H2" 0 "hypothetical protein"
"DsuE09_00191" 4108 "s59E5H2" 0 "hypothetical protein"
"DsuE09_00191" 4108 "s63E6H2" 0 "hypothetical protein"
"DsuE09_00196" 4113 "s59E13H2" 0 "hypothetical protein"
"DsuE09_00196" 4113 "s59E25H2" 0 "hypothetical protein"
"DsuE09_00196" 4113 "s59E5H2" 0 "hypothetical protein"
"DsuE09_00196" 4113 "s63E6H2" 0 "hypothetical protein"
"DsuE09_00197" 4114 "s59E13H2" 0 "hypothetical protein"
"DsuE09_00197" 4114 "s59E25H2" 0 "hypothetical protein"
"DsuE09_00197" 4114 "s59E5H2" 0 "hypothetical protein"
"DsuE09_00197" 4114 "s63E6H2" 0 "hypothetical protein"
"DsuE09_00199" 4116 "s59E13H2" 0 "30S ribosomal protein S21"
"DsuE09_00199" 4116 "s59E25H2" 0 "30S ribosomal protein S21"

"DsuE09_00199" 4116 "s59E5H2" 0 "30S ribosomal protein S21"
"DsuE09_00199" 4116 "s63E6H2" 0 "30S ribosomal protein S21"
"DsuE09_00201" 4118 "s59E13H2" 0 "hypothetical protein"
"DsuE09_00201" 4118 "s59E25H2" 0 "hypothetical protein"
"DsuE09_00201" 4118 "s59E5H2" 0 "hypothetical protein"
"DsuE09_00201" 4118 "s63E6H2" 0 "hypothetical protein"
"DsuE09_00203" 4120 "s59E13H2" 0 "hypothetical protein"
"DsuE09_00203" 4120 "s59E25H2" 0 "hypothetical protein"
"DsuE09_00203" 4120 "s59E5H2" 0 "hypothetical protein"
"DsuE09_00203" 4120 "s63E6H2" 0 "hypothetical protein"
"DsuE09_00208" 4125 "s59E13H2" 0 "hypothetical protein"
"DsuE09_00208" 4125 "s59E25H2" 0 "hypothetical protein"
"DsuE09_00208" 4125 "s59E5H2" 0 "hypothetical protein"
"DsuE09_00208" 4125 "s63E6H2" 0 "hypothetical protein"
"DsuE09_00209" 4126 "s59E13H2" 0 "hypothetical protein"
"DsuE09_00209" 4126 "s59E25H2" 0 "hypothetical protein"
"DsuE09_00209" 4126 "s59E5H2" 0 "hypothetical protein"
"DsuE09_00209" 4126 "s63E6H2" 0 "hypothetical protein"
"DsuE09_00213" 4130 "s59E13H2" 0 "hypothetical protein"
"DsuE09_00213" 4130 "s59E25H2" 0 "hypothetical protein"
"DsuE09_00213" 4130 "s59E5H2" 0 "hypothetical protein"
"DsuE09_00213" 4130 "s63E6H2" 0 "hypothetical protein"
"DsuE09_00214" 4131 "s59E13H2" 0 "hypothetical protein"
"DsuE09_00214" 4131 "s59E25H2" 0 "hypothetical protein"
"DsuE09_00214" 4131 "s59E5H2" 0 "hypothetical protein"
"DsuE09_00214" 4131 "s63E6H2" 0 "hypothetical protein"
"DsuE09_00216" 4133 "s59E13H2" 0 "hypothetical protein"
"DsuE09_00216" 4133 "s59E25H2" 0 "hypothetical protein"
"DsuE09_00216" 4133 "s59E5H2" 0 "hypothetical protein"
"DsuE09_00216" 4133 "s63E6H2" 0 "hypothetical protein"
"DsuE09_00217" 4134 "s59E13H2" 0 "hypothetical protein"
"DsuE09_00217" 4134 "s59E25H2" 0 "hypothetical protein"
"DsuE09_00217" 4134 "s59E5H2" 0 "hypothetical protein"
"DsuE09_00217" 4134 "s63E6H2" 0 "hypothetical protein"
"DsuE09_00218" 4135 "s59E13H2" 0 "Tyrosine recombinase XerD"
"DsuE09_00218" 4135 "s59E25H2" 0 "Tyrosine recombinase XerD"
"DsuE09_00218" 4135 "s59E5H2" 0 "Tyrosine recombinase XerD"
"DsuE09_00218" 4135 "s63E6H2" 0 "Tyrosine recombinase XerD"
"DsuE09_00219" 4136 "s59E13H2" 0 "hypothetical protein"
"DsuE09_00219" 4136 "s59E25H2" 0 "hypothetical protein"
"DsuE09_00219" 4136 "s59E5H2" 0 "hypothetical protein"
"DsuE09_00219" 4136 "s63E6H2" 0 "hypothetical protein"
"DsuE09_00220" 4137 "s59E13H2" 0 "UDP-3-O-[3-hydroxymyristoyl] N-acetylglucosamine deacetylase"
"DsuE09_00220" 4137 "s59E25H2" 0 "UDP-3-O-[3-hydroxymyristoyl] N-acetylglucosamine deacetylase"
"DsuE09_00220" 4137 "s59E5H2" 0 "UDP-3-O-[3-hydroxymyristoyl] N-acetylglucosamine deacetylase"
"DsuE09_00220" 4137 "s63E6H2" 0 "UDP-3-O-[3-hydroxymyristoyl] N-acetylglucosamine deacetylase"
"DsuE09_00221" 4138 "s59E13H2" 0 "Membrane-bound lytic murein transglycosylase A precursor"

"DsuE09_00221" 4138 "s59E25H2" 0 "Membrane-bound lytic murein transglycosylase A precursor"
"DsuE09_00221" 4138 "s59E5H2" 0 "Membrane-bound lytic murein transglycosylase A precursor"
"DsuE09_00221" 4138 "s63E6H2" 0 "Membrane-bound lytic murein transglycosylase A precursor"
"DsuE09_00222" 4139 "s59E13H2" 0 "Glycine--tRNA ligase beta subunit"
"DsuE09_00222" 4139 "s59E25H2" 0 "Glycine--tRNA ligase beta subunit"
"DsuE09_00222" 4139 "s59E5H2" 0 "Glycine--tRNA ligase beta subunit"
"DsuE09_00222" 4139 "s63E6H2" 0 "Glycine--tRNA ligase beta subunit"
"DsuE09_00223" 4140 "s59E13H2" 0 "hypothetical protein"
"DsuE09_00223" 4140 "s59E25H2" 0 "hypothetical protein"
"DsuE09_00223" 4140 "s59E5H2" 0 "hypothetical protein"
"DsuE09_00223" 4140 "s63E6H2" 0 "hypothetical protein"
"DsuE09_00224" 4141 "s59E13H2" 0 "Glycine--tRNA ligase alpha subunit"
"DsuE09_00224" 4141 "s59E25H2" 0 "Glycine--tRNA ligase alpha subunit"
"DsuE09_00224" 4141 "s59E5H2" 0 "Glycine--tRNA ligase alpha subunit"
"DsuE09_00224" 4141 "s63E6H2" 0 "Glycine--tRNA ligase alpha subunit"
"DsuE09_00225" 4142 "s59E13H2" 0 "DNA repair protein RecO"
"DsuE09_00225" 4142 "s59E25H2" 0 "DNA repair protein RecO"
"DsuE09_00225" 4142 "s59E5H2" 0 "DNA repair protein RecO"
"DsuE09_00225" 4142 "s63E6H2" 0 "DNA repair protein RecO"
"DsuE09_00226" 4143 "s59E13H2" 0 "hypothetical protein"
"DsuE09_00226" 4143 "s59E25H2" 0 "hypothetical protein"
"DsuE09_00226" 4143 "s59E5H2" 0 "hypothetical protein"
"DsuE09_00226" 4143 "s63E6H2" 0 "hypothetical protein"
"DsuE09_00227" 4144 "s59E13H2" 0 "High-affinity branched-chain amino acid transport ATP-binding protein LivF"
"DsuE09_00227" 4144 "s59E25H2" 0 "High-affinity branched-chain amino acid transport ATP-binding protein LivF"
"DsuE09_00227" 4144 "s59E5H2" 0 "High-affinity branched-chain amino acid transport ATP-binding protein LivF"
"DsuE09_00227" 4144 "s63E6H2" 0 "High-affinity branched-chain amino acid transport ATP-binding protein LivF"
"DsuE09_00228" 4145 "s59E13H2" 0 "hypothetical protein"
"DsuE09_00228" 4145 "s59E25H2" 0 "hypothetical protein"
"DsuE09_00228" 4145 "s59E5H2" 0 "hypothetical protein"
"DsuE09_00228" 4145 "s63E6H2" 0 "hypothetical protein"
"DsuE09_00229" 4146 "s59E13H2" 0 "leucine/isoleucine/valine transporter permease subunit"
"DsuE09_00229" 4146 "s59E25H2" 0 "leucine/isoleucine/valine transporter permease subunit"
"DsuE09_00229" 4146 "s59E5H2" 0 "leucine/isoleucine/valine transporter permease subunit"
"DsuE09_00229" 4146 "s63E6H2" 0.005010708 "leucine/isoleucine/valine transporter permease subunit"
"DsuE09_00230" 4147 "s59E13H2" 0 "High-affinity branched-chain amino acid transport system permease protein LivH"
"DsuE09_00230" 4147 "s59E25H2" 0 "High-affinity branched-chain amino acid transport system permease protein LivH"
"DsuE09_00230" 4147 "s59E5H2" 0 "High-affinity branched-chain amino acid transport system permease protein LivH"
"DsuE09_00230" 4147 "s63E6H2" 0 "High-affinity branched-chain amino acid transport system permease protein LivH"
"DsuE09_00231" 4148 "s59E13H2" 0 "Long-chain-fatty-acid--CoA ligase FadD15"
"DsuE09_00231" 4148 "s59E25H2" 0 "Long-chain-fatty-acid--CoA ligase FadD15"
"DsuE09_00231" 4148 "s59E5H2" 0 "Long-chain-fatty-acid--CoA ligase FadD15"
"DsuE09_00231" 4148 "s63E6H2" 0 "Long-chain-fatty-acid--CoA ligase FadD15"
"DsuE09_00232" 4149 "s59E13H2" 0 "putative branched-chain amino acid transport ATP-binding protein LivG"
"DsuE09_00232" 4149 "s59E25H2" 0 "putative branched-chain amino acid transport ATP-binding protein LivG"
"DsuE09_00232" 4149 "s59E5H2" 0 "putative branched-chain amino acid transport ATP-binding protein LivG"
"DsuE09_00232" 4149 "s63E6H2" 0 "putative branched-chain amino acid transport ATP-binding protein LivG"

"DsuE09_00233" 4150 "s59E13H2" 0 "deoxyguanosinetriphosphate triphosphohydrolase-like protein"
"DsuE09_00233" 4150 "s59E25H2" 0 "deoxyguanosinetriphosphate triphosphohydrolase-like protein"
"DsuE09_00233" 4150 "s59E5H2" 0 "deoxyguanosinetriphosphate triphosphohydrolase-like protein"
"DsuE09_00233" 4150 "s63E6H2" 0 "deoxyguanosinetriphosphate triphosphohydrolase-like protein"
"DsuE09_00234" 4151 "s59E13H2" 0 "Alkaline phosphatase synthesis transcriptional regulatory protein PhoP"
"DsuE09_00234" 4151 "s59E25H2" 0 "Alkaline phosphatase synthesis transcriptional regulatory protein PhoP"
"DsuE09_00234" 4151 "s59E5H2" 0 "Alkaline phosphatase synthesis transcriptional regulatory protein PhoP"
"DsuE09_00234" 4151 "s63E6H2" 0 "Alkaline phosphatase synthesis transcriptional regulatory protein PhoP"
"DsuE09_00235" 4152 "s59E13H2" 0 "Type II secretion system protein E"
"DsuE09_00235" 4152 "s59E25H2" 0 "Type II secretion system protein E"
"DsuE09_00235" 4152 "s59E5H2" 0 "Type II secretion system protein E"
"DsuE09_00235" 4152 "s63E6H2" 0 "Type II secretion system protein E"
"DsuE09_00236" 4153 "s59E13H2" 0 "hypothetical protein"
"DsuE09_00236" 4153 "s59E25H2" 0 "hypothetical protein"
"DsuE09_00236" 4153 "s59E5H2" 0 "hypothetical protein"
"DsuE09_00236" 4153 "s63E6H2" 0 "hypothetical protein"
"DsuE09_00238" 4155 "s59E13H2" 0 "Sensor protein ZraS"
"DsuE09_00238" 4155 "s59E25H2" 0 "Sensor protein ZraS"
"DsuE09_00238" 4155 "s59E5H2" 0 "Sensor protein ZraS"
"DsuE09_00238" 4155 "s63E6H2" 0 "Sensor protein ZraS"
"DsuE09_00239" 4156 "s59E13H2" 0 "hypothetical protein"
"DsuE09_00239" 4156 "s59E25H2" 0 "hypothetical protein"
"DsuE09_00239" 4156 "s59E5H2" 0 "hypothetical protein"
"DsuE09_00239" 4156 "s63E6H2" 0 "hypothetical protein"
"DsuE09_00240" 4157 "s59E13H2" 0 "hypothetical protein"
"DsuE09_00240" 4157 "s59E25H2" 0 "hypothetical protein"
"DsuE09_00240" 4157 "s59E5H2" 0 "hypothetical protein"
"DsuE09_00240" 4157 "s63E6H2" 0 "hypothetical protein"
"DsuE09_00241" 4158 "s59E13H2" 0 "Sensor protein ZraS"
"DsuE09_00241" 4158 "s59E25H2" 0 "Sensor protein ZraS"
"DsuE09_00241" 4158 "s59E5H2" 0 "Sensor protein ZraS"
"DsuE09_00241" 4158 "s63E6H2" 0 "Sensor protein ZraS"
"DsuE09_00242" 4159 "s59E13H2" 0 "Response regulator PleD"
"DsuE09_00242" 4159 "s59E25H2" 0 "Response regulator PleD"
"DsuE09_00242" 4159 "s59E5H2" 0 "Response regulator PleD"
"DsuE09_00242" 4159 "s63E6H2" 0 "Response regulator PleD"
"DsuE09_00243" 4160 "s59E13H2" 0 "Phosphate regulon transcriptional regulatory protein PhoB"
"DsuE09_00243" 4160 "s59E25H2" 0 "Phosphate regulon transcriptional regulatory protein PhoB"
"DsuE09_00243" 4160 "s59E5H2" 0 "Phosphate regulon transcriptional regulatory protein PhoB"
"DsuE09_00243" 4160 "s63E6H2" 0 "Phosphate regulon transcriptional regulatory protein PhoB"
"DsuE09_00244" 4161 "s59E13H2" 0 "hypothetical protein"
"DsuE09_00244" 4161 "s59E25H2" 0 "hypothetical protein"
"DsuE09_00244" 4161 "s59E5H2" 0 "hypothetical protein"
"DsuE09_00244" 4161 "s63E6H2" 0 "hypothetical protein"
"DsuE09_00245" 4162 "s59E13H2" 0 "Cyclic di-GMP phosphodiesterase response regulator RpfG"
"DsuE09_00245" 4162 "s59E25H2" 0 "Cyclic di-GMP phosphodiesterase response regulator RpfG"
"DsuE09_00245" 4162 "s59E5H2" 0 "Cyclic di-GMP phosphodiesterase response regulator RpfG"

"DsuE09_00245" 4162 "s63E6H2" 0 "Cyclic di-GMP phosphodiesterase response regulator RpfG"
"DsuE09_00246" 4163 "s59E13H2" 0 "Mutual gliding-motility protein MglA"
"DsuE09_00246" 4163 "s59E25H2" 0 "Mutual gliding-motility protein MglA"
"DsuE09_00246" 4163 "s59E5H2" 0 "Mutual gliding-motility protein MglA"
"DsuE09_00246" 4163 "s63E6H2" 0 "Mutual gliding-motility protein MglA"
"DsuE09_00247" 4164 "s59E13H2" 0 "Putative type II secretion system protein E"
"DsuE09_00247" 4164 "s59E25H2" 0 "Putative type II secretion system protein E"
"DsuE09_00247" 4164 "s59E5H2" 0 "Putative type II secretion system protein E"
"DsuE09_00247" 4164 "s63E6H2" 0 "Putative type II secretion system protein E"
"DsuE09_00248" 4165 "s59E13H2" 0 "Alkaline phosphatase synthesis transcriptional regulatory protein PhoP"
"DsuE09_00248" 4165 "s59E25H2" 0 "Alkaline phosphatase synthesis transcriptional regulatory protein PhoP"
"DsuE09_00248" 4165 "s59E5H2" 0 "Alkaline phosphatase synthesis transcriptional regulatory protein PhoP"
"DsuE09_00248" 4165 "s63E6H2" 0 "Alkaline phosphatase synthesis transcriptional regulatory protein PhoP"
"DsuE09_00249" 4166 "s59E13H2" 0 "Methyl-accepting chemotaxis protein McpB"
"DsuE09_00249" 4166 "s59E25H2" 0 "Methyl-accepting chemotaxis protein McpB"
"DsuE09_00249" 4166 "s59E5H2" 0 "Methyl-accepting chemotaxis protein McpB"
"DsuE09_00249" 4166 "s63E6H2" 0 "Methyl-accepting chemotaxis protein McpB"
"DsuE09_00250" 4167 "s59E13H2" 0 "CheW-like domain protein"
"DsuE09_00250" 4167 "s59E25H2" 0 "CheW-like domain protein"
"DsuE09_00250" 4167 "s59E5H2" 0 "CheW-like domain protein"
"DsuE09_00250" 4167 "s63E6H2" 0 "CheW-like domain protein"
"DsuE09_00251" 4168 "s59E13H2" 0 "Chemotaxis protein CheA"
"DsuE09_00251" 4168 "s59E25H2" 0 "Chemotaxis protein CheA"
"DsuE09_00251" 4168 "s59E5H2" 0 "Chemotaxis protein CheA"
"DsuE09_00251" 4168 "s63E6H2" 0 "Chemotaxis protein CheA"
"DsuE09_00252" 4169 "s59E13H2" 0 "3-oxoacyl-[acyl-carrier-protein] reductase FabG"
"DsuE09_00252" 4169 "s59E25H2" 0 "3-oxoacyl-[acyl-carrier-protein] reductase FabG"
"DsuE09_00252" 4169 "s59E5H2" 0 "3-oxoacyl-[acyl-carrier-protein] reductase FabG"
"DsuE09_00252" 4169 "s63E6H2" 0 "3-oxoacyl-[acyl-carrier-protein] reductase FabG"
"DsuE09_00253" 4170 "s59E13H2" 0 "Alcohol dehydrogenase"
"DsuE09_00253" 4170 "s59E25H2" 0 "Alcohol dehydrogenase"
"DsuE09_00253" 4170 "s59E5H2" 0 "Alcohol dehydrogenase"
"DsuE09_00253" 4170 "s63E6H2" 0 "Alcohol dehydrogenase"
"DsuE09_00254" 4171 "s59E13H2" 0 "1,4-Dihydroxy-2-naphthoyl-CoA synthase"
"DsuE09_00254" 4171 "s59E25H2" 0 "1,4-Dihydroxy-2-naphthoyl-CoA synthase"
"DsuE09_00254" 4171 "s59E5H2" 0 "1,4-Dihydroxy-2-naphthoyl-CoA synthase"
"DsuE09_00254" 4171 "s63E6H2" 0 "1,4-Dihydroxy-2-naphthoyl-CoA synthase"
"DsuE09_00255" 4172 "s59E13H2" 0 "putative enoyl-CoA hydratase echA8"
"DsuE09_00255" 4172 "s59E25H2" 0 "putative enoyl-CoA hydratase echA8"
"DsuE09_00255" 4172 "s59E5H2" 0 "putative enoyl-CoA hydratase echA8"
"DsuE09_00255" 4172 "s63E6H2" 0 "putative enoyl-CoA hydratase echA8"
"DsuE09_00256" 4173 "s59E13H2" 0 "Calcium-gated potassium channel MthK"
"DsuE09_00256" 4173 "s59E25H2" 0 "Calcium-gated potassium channel MthK"
"DsuE09_00256" 4173 "s59E5H2" 0 "Calcium-gated potassium channel MthK"
"DsuE09_00256" 4173 "s63E6H2" 0 "Calcium-gated potassium channel MthK"
"DsuE09_00257" 4174 "s59E13H2" 0 "cAMP receptor protein"
"DsuE09_00257" 4174 "s59E25H2" 0 "cAMP receptor protein"

"DsuE09_00257" 4174 "s59E5H2" 0 "cAMP receptor protein"
"DsuE09_00257" 4174 "s63E6H2" 0 "cAMP receptor protein"
"DsuE09_00258" 4175 "s59E13H2" 0 "RlpA-like protein precursor"
"DsuE09_00258" 4175 "s59E25H2" 0 "RlpA-like protein precursor"
"DsuE09_00258" 4175 "s59E5H2" 0 "RlpA-like protein precursor"
"DsuE09_00258" 4175 "s63E6H2" 0 "RlpA-like protein precursor"
"DsuE09_00259" 4176 "s59E13H2" 0 "Integration host factor subunit alpha"
"DsuE09_00259" 4176 "s59E25H2" 0 "Integration host factor subunit alpha"
"DsuE09_00259" 4176 "s59E5H2" 0 "Integration host factor subunit alpha"
"DsuE09_00259" 4176 "s63E6H2" 0 "Integration host factor subunit alpha"
"DsuE09_00260" 4177 "s59E13H2" 0 "Phosphopantetheine adenylyltransferase"
"DsuE09_00260" 4177 "s59E25H2" 0 "Phosphopantetheine adenylyltransferase"
"DsuE09_00260" 4177 "s59E5H2" 0 "Phosphopantetheine adenylyltransferase"
"DsuE09_00260" 4177 "s63E6H2" 0 "Phosphopantetheine adenylyltransferase"
"DsuE09_00261" 4178 "s59E13H2" 0 "Ribosomal RNA small subunit methyltransferase D"
"DsuE09_00261" 4178 "s59E25H2" 0 "Ribosomal RNA small subunit methyltransferase D"
"DsuE09_00261" 4178 "s59E5H2" 0 "Ribosomal RNA small subunit methyltransferase D"
"DsuE09_00261" 4178 "s63E6H2" 0 "Ribosomal RNA small subunit methyltransferase D"
"DsuE09_00262" 4179 "s59E13H2" 0 "Archaetidylserine synthase"
"DsuE09_00262" 4179 "s59E25H2" 0 "Archaetidylserine synthase"
"DsuE09_00262" 4179 "s59E5H2" 0 "Archaetidylserine synthase"
"DsuE09_00262" 4179 "s63E6H2" 0 "Archaetidylserine synthase"
"DsuE09_00263" 4180 "s59E13H2" 0 "phosphatidylserine decarboxylase"
"DsuE09_00263" 4180 "s59E25H2" 0 "phosphatidylserine decarboxylase"
"DsuE09_00263" 4180 "s59E5H2" 0 "phosphatidylserine decarboxylase"
"DsuE09_00263" 4180 "s63E6H2" 0 "phosphatidylserine decarboxylase"
"DsuE09_00265" 4182 "s59E13H2" 0 "tRNA threonylcarbamoyladenosine biosynthesis protein TsaB"
"DsuE09_00265" 4182 "s59E25H2" 0 "tRNA threonylcarbamoyladenosine biosynthesis protein TsaB"
"DsuE09_00265" 4182 "s59E5H2" 0 "tRNA threonylcarbamoyladenosine biosynthesis protein TsaB"
"DsuE09_00265" 4182 "s63E6H2" 0 "tRNA threonylcarbamoyladenosine biosynthesis protein TsaB"
"DsuE09_00266" 4183 "s59E13H2" 0 "Metalloprotease MmpA"
"DsuE09_00266" 4183 "s59E25H2" 0 "Metalloprotease MmpA"
"DsuE09_00266" 4183 "s59E5H2" 0 "Metalloprotease MmpA"
"DsuE09_00266" 4183 "s63E6H2" 0 "Metalloprotease MmpA"
"DsuE09_00267" 4184 "s59E13H2" 0 "1-deoxy-D-xylulose 5-phosphate reductoisomerase"
"DsuE09_00267" 4184 "s59E25H2" 0 "1-deoxy-D-xylulose 5-phosphate reductoisomerase"
"DsuE09_00267" 4184 "s59E5H2" 0 "1-deoxy-D-xylulose 5-phosphate reductoisomerase"
"DsuE09_00267" 4184 "s63E6H2" 0 "1-deoxy-D-xylulose 5-phosphate reductoisomerase"
"DsuE09_00268" 4185 "s59E13H2" 0 "Phosphatidate cytidylyltransferase"
"DsuE09_00268" 4185 "s59E25H2" 0 "Phosphatidate cytidylyltransferase"
"DsuE09_00268" 4185 "s59E5H2" 0 "Phosphatidate cytidylyltransferase"
"DsuE09_00268" 4185 "s63E6H2" 0 "Phosphatidate cytidylyltransferase"
"DsuE09_00269" 4186 "s59E13H2" 0 "Isoprenyl transferase"
"DsuE09_00269" 4186 "s59E25H2" 0 "Isoprenyl transferase"
"DsuE09_00269" 4186 "s59E5H2" 0 "Isoprenyl transferase"
"DsuE09_00269" 4186 "s63E6H2" 0 "Isoprenyl transferase"
"DsuE09_00270" 4187 "s59E13H2" 0 "Ribosome-recycling factor"

"DsuE09_00270" 4187 "s59E25H2" 0 "Ribosome-recycling factor"
"DsuE09_00270" 4187 "s59E5H2" 0 "Ribosome-recycling factor"
"DsuE09_00270" 4187 "s63E6H2" 0 "Ribosome-recycling factor"
"DsuE09_00271" 4188 "s59E13H2" 0 "Uridylate kinase"
"DsuE09_00271" 4188 "s59E25H2" 0 "Uridylate kinase"
"DsuE09_00271" 4188 "s59E5H2" 0 "Uridylate kinase"
"DsuE09_00271" 4188 "s63E6H2" 0 "Uridylate kinase"
"DsuE09_00272" 4189 "s59E13H2" 0 "Elongation factor Ts"
"DsuE09_00272" 4189 "s59E25H2" 0 "Elongation factor Ts"
"DsuE09_00272" 4189 "s59E5H2" 0 "Elongation factor Ts"
"DsuE09_00272" 4189 "s63E6H2" 0 "Elongation factor Ts"
"DsuE09_00273" 4190 "s59E13H2" 0 "30S ribosomal protein S2"
"DsuE09_00273" 4190 "s59E25H2" 0 "30S ribosomal protein S2"
"DsuE09_00273" 4190 "s59E5H2" 0 "30S ribosomal protein S2"
"DsuE09_00273" 4190 "s63E6H2" 0 "30S ribosomal protein S2"
"DsuE09_00274" 4191 "s59E13H2" 0 "High-affinity branched-chain amino acid transport ATP-binding protein LivF"
"DsuE09_00274" 4191 "s59E25H2" 0 "High-affinity branched-chain amino acid transport ATP-binding protein LivF"
"DsuE09_00274" 4191 "s59E5H2" 0 "High-affinity branched-chain amino acid transport ATP-binding protein LivF"
"DsuE09_00274" 4191 "s63E6H2" 0 "High-affinity branched-chain amino acid transport ATP-binding protein LivF"
"DsuE09_00275" 4192 "s59E13H2" 0 "leucine/isoleucine/valine transporter permease subunit"
"DsuE09_00275" 4192 "s59E25H2" 0 "leucine/isoleucine/valine transporter permease subunit"
"DsuE09_00275" 4192 "s59E5H2" 0 "leucine/isoleucine/valine transporter permease subunit"
"DsuE09_00275" 4192 "s63E6H2" 0 "leucine/isoleucine/valine transporter permease subunit"
"DsuE09_00276" 4193 "s59E13H2" 0 "High-affinity branched-chain amino acid transport system permease protein LivH"
"DsuE09_00276" 4193 "s59E25H2" 0 "High-affinity branched-chain amino acid transport system permease protein LivH"
"DsuE09_00276" 4193 "s59E5H2" 0 "High-affinity branched-chain amino acid transport system permease protein LivH"
"DsuE09_00276" 4193 "s63E6H2" 0 "High-affinity branched-chain amino acid transport system permease protein LivH"
"DsuE09_00277" 4194 "s59E13H2" 0 "hypothetical protein"
"DsuE09_00277" 4194 "s59E25H2" 0 "hypothetical protein"
"DsuE09_00277" 4194 "s59E5H2" 0 "hypothetical protein"
"DsuE09_00277" 4194 "s63E6H2" 0 "hypothetical protein"
"DsuE09_00278" 4195 "s59E13H2" 0 "Long-chain-fatty-acid--CoA ligase FadD15"
"DsuE09_00278" 4195 "s59E25H2" 0 "Long-chain-fatty-acid--CoA ligase FadD15"
"DsuE09_00278" 4195 "s59E5H2" 0 "Long-chain-fatty-acid--CoA ligase FadD15"
"DsuE09_00278" 4195 "s63E6H2" 0 "Long-chain-fatty-acid--CoA ligase FadD15"
"DsuE09_00279" 4196 "s59E13H2" 0 "putative branched-chain amino acid transport ATP-binding protein LivG"
"DsuE09_00279" 4196 "s59E25H2" 0 "putative branched-chain amino acid transport ATP-binding protein LivG"
"DsuE09_00279" 4196 "s59E5H2" 0 "putative branched-chain amino acid transport ATP-binding protein LivG"
"DsuE09_00279" 4196 "s63E6H2" 0 "putative branched-chain amino acid transport ATP-binding protein LivG"
"DsuE09_00280" 4197 "s59E13H2" 0 "hypothetical protein"
"DsuE09_00280" 4197 "s59E25H2" 0 "hypothetical protein"
"DsuE09_00280" 4197 "s59E5H2" 0 "hypothetical protein"
"DsuE09_00280" 4197 "s63E6H2" 0 "hypothetical protein"
"DsuE09_00281" 4198 "s59E13H2" 0 "Long-chain-fatty-acid--CoA ligase"
"DsuE09_00281" 4198 "s59E25H2" 0 "Long-chain-fatty-acid--CoA ligase"
"DsuE09_00281" 4198 "s59E5H2" 0 "Long-chain-fatty-acid--CoA ligase"
"DsuE09_00281" 4198 "s63E6H2" 0 "Long-chain-fatty-acid--CoA ligase"

"DsuE09_00282" 4199 "s59E13H2" 0 "Indolepyruvate oxidoreductase subunit IorA"
"DsuE09_00282" 4199 "s59E25H2" 0 "Indolepyruvate oxidoreductase subunit IorA"
"DsuE09_00282" 4199 "s59E5H2" 0 "Indolepyruvate oxidoreductase subunit IorA"
"DsuE09_00282" 4199 "s63E6H2" 0 "Indolepyruvate oxidoreductase subunit IorA"
"DsuE09_00283" 4200 "s59E13H2" 0 "Indolepyruvate oxidoreductase subunit IorB"
"DsuE09_00283" 4200 "s59E25H2" 0 "Indolepyruvate oxidoreductase subunit IorB"
"DsuE09_00283" 4200 "s59E5H2" 0 "Indolepyruvate oxidoreductase subunit IorB"
"DsuE09_00283" 4200 "s63E6H2" 0 "Indolepyruvate oxidoreductase subunit IorB"
"DsuE09_00284" 4201 "s59E13H2" 0 "MoaE protein"
"DsuE09_00284" 4201 "s59E25H2" 0 "MoaE protein"
"DsuE09_00284" 4201 "s59E5H2" 0 "MoaE protein"
"DsuE09_00284" 4201 "s63E6H2" 0 "MoaE protein"
"DsuE09_00285" 4202 "s59E13H2" 0 "Glutamyl-tRNA reductase"
"DsuE09_00285" 4202 "s59E25H2" 0 "Glutamyl-tRNA reductase"
"DsuE09_00285" 4202 "s59E5H2" 0 "Glutamyl-tRNA reductase"
"DsuE09_00285" 4202 "s63E6H2" 0 "Glutamyl-tRNA reductase"
"DsuE09_00286" 4203 "s59E13H2" 0 "Cytochrome c biogenesis protein CcsA"
"DsuE09_00286" 4203 "s59E25H2" 0 "Cytochrome c biogenesis protein CcsA"
"DsuE09_00286" 4203 "s59E5H2" 0 "Cytochrome c biogenesis protein CcsA"
"DsuE09_00286" 4203 "s63E6H2" 0 "Cytochrome c biogenesis protein CcsA"
"DsuE09_00287" 4204 "s59E13H2" 0 "Siroheme synthase"
"DsuE09_00287" 4204 "s59E25H2" 0 "Siroheme synthase"
"DsuE09_00287" 4204 "s59E5H2" 0 "Siroheme synthase"
"DsuE09_00287" 4204 "s63E6H2" 0 "Siroheme synthase"
"DsuE09_00288" 4205 "s59E13H2" 0 "hypothetical protein"
"DsuE09_00288" 4205 "s59E25H2" 0 "hypothetical protein"
"DsuE09_00288" 4205 "s59E5H2" 0 "hypothetical protein"
"DsuE09_00288" 4205 "s63E6H2" 0 "hypothetical protein"
"DsuE09_00289" 4206 "s59E13H2" 0 "putative transcriptional regulatory protein"
"DsuE09_00289" 4206 "s59E25H2" 0 "putative transcriptional regulatory protein"
"DsuE09_00289" 4206 "s59E5H2" 0 "putative transcriptional regulatory protein"
"DsuE09_00289" 4206 "s63E6H2" 0 "putative transcriptional regulatory protein"
"DsuE09_00290" 4207 "s59E13H2" 0 "Crossover junction endodeoxyribonuclease RuvC"
"DsuE09_00290" 4207 "s59E25H2" 0 "Crossover junction endodeoxyribonuclease RuvC"
"DsuE09_00290" 4207 "s59E5H2" 0.00292287 "Crossover junction endodeoxyribonuclease RuvC"
"DsuE09_00290" 4207 "s63E6H2" 0 "Crossover junction endodeoxyribonuclease RuvC"
"DsuE09_00291" 4208 "s59E13H2" 0 "Holliday junction ATP-dependent DNA helicase RuvA"
"DsuE09_00291" 4208 "s59E25H2" 0 "Holliday junction ATP-dependent DNA helicase RuvA"
"DsuE09_00291" 4208 "s59E5H2" 0.005809838 "Holliday junction ATP-dependent DNA helicase RuvA"
"DsuE09_00291" 4208 "s63E6H2" 0 "Holliday junction ATP-dependent DNA helicase RuvA"
"DsuE09_00292" 4209 "s59E13H2" 0 "hypothetical protein"
"DsuE09_00292" 4209 "s59E25H2" 0 "hypothetical protein"
"DsuE09_00292" 4209 "s59E5H2" 0 "hypothetical protein"
"DsuE09_00292" 4209 "s63E6H2" 0 "hypothetical protein"
"DsuE09_00293" 4210 "s59E13H2" 0 "Transposase"
"DsuE09_00293" 4210 "s59E25H2" 0 "Transposase"
"DsuE09_00293" 4210 "s59E5H2" 0 "Transposase"

"DsuE09_00293" 4210 "s63E6H2" 0 "Transposase"
"DsuE09_00294" 4211 "s59E13H2" 0 "Benzoylformate decarboxylase"
"DsuE09_00294" 4211 "s59E25H2" 0 "Benzoylformate decarboxylase"
"DsuE09_00294" 4211 "s59E5H2" 0 "Benzoylformate decarboxylase"
"DsuE09_00294" 4211 "s63E6H2" 0 "Benzoylformate decarboxylase"
"DsuE09_00295" 4212 "s59E13H2" 0 "Sulfoacetaldehyde acetyltransferase"
"DsuE09_00295" 4212 "s59E25H2" 0 "Sulfoacetaldehyde acetyltransferase"
"DsuE09_00295" 4212 "s59E5H2" 0 "Sulfoacetaldehyde acetyltransferase"
"DsuE09_00295" 4212 "s63E6H2" 0 "Sulfoacetaldehyde acetyltransferase"
"DsuE09_00296" 4213 "s59E13H2" 0 "NTE family protein RssA"
"DsuE09_00296" 4213 "s59E25H2" 0 "NTE family protein RssA"
"DsuE09_00296" 4213 "s59E5H2" 0 "NTE family protein RssA"
"DsuE09_00296" 4213 "s63E6H2" 0 "NTE family protein RssA"
"DsuE09_00297" 4214 "s59E13H2" 0 "Magnesium transporter MgtE"
"DsuE09_00297" 4214 "s59E25H2" 0 "Magnesium transporter MgtE"
"DsuE09_00297" 4214 "s59E5H2" 0 "Magnesium transporter MgtE"
"DsuE09_00297" 4214 "s63E6H2" 0 "Magnesium transporter MgtE"
"DsuE09_00298" 4215 "s59E13H2" 0 "Thiamine-phosphate synthase"
"DsuE09_00298" 4215 "s59E25H2" 0 "Thiamine-phosphate synthase"
"DsuE09_00298" 4215 "s59E5H2" 0 "Thiamine-phosphate synthase"
"DsuE09_00298" 4215 "s63E6H2" 0 "Thiamine-phosphate synthase"
"DsuE09_00299" 4216 "s59E13H2" 0 "Putative ribose 1,5-bisphosphate isomerase"
"DsuE09_00299" 4216 "s59E25H2" 0 "Putative ribose 1,5-bisphosphate isomerase"
"DsuE09_00299" 4216 "s59E5H2" 0 "Putative ribose 1,5-bisphosphate isomerase"
"DsuE09_00299" 4216 "s63E6H2" 0 "Putative ribose 1,5-bisphosphate isomerase"
"DsuE09_00300" 4217 "s59E13H2" 0 "Isoleucine--tRNA ligase"
"DsuE09_00300" 4217 "s59E25H2" 0 "Isoleucine--tRNA ligase"
"DsuE09_00300" 4217 "s59E5H2" 0 "Isoleucine--tRNA ligase"
"DsuE09_00300" 4217 "s63E6H2" 0 "Isoleucine--tRNA ligase"
"DsuE09_00301" 4218 "s59E13H2" 0 "Lipoprotein signal peptidase"
"DsuE09_00301" 4218 "s59E25H2" 0 "Lipoprotein signal peptidase"
"DsuE09_00301" 4218 "s59E5H2" 0 "Lipoprotein signal peptidase"
"DsuE09_00301" 4218 "s63E6H2" 0 "Lipoprotein signal peptidase"
"DsuE09_00302" 4219 "s59E13H2" 0 "Pyruvate formate-lyase 1-activating enzyme"
"DsuE09_00302" 4219 "s59E25H2" 0 "Pyruvate formate-lyase 1-activating enzyme"
"DsuE09_00302" 4219 "s59E5H2" 0 "Pyruvate formate-lyase 1-activating enzyme"
"DsuE09_00302" 4219 "s63E6H2" 0 "Pyruvate formate-lyase 1-activating enzyme"
"DsuE09_00303" 4220 "s59E13H2" 0 "hypothetical protein"
"DsuE09_00303" 4220 "s59E25H2" 0 "hypothetical protein"
"DsuE09_00303" 4220 "s59E5H2" 0 "hypothetical protein"
"DsuE09_00303" 4220 "s63E6H2" 0 "hypothetical protein"
"DsuE09_00304" 4221 "s59E13H2" 0 "putative methyltransferase YcgJ"
"DsuE09_00304" 4221 "s59E25H2" 0 "putative methyltransferase YcgJ"
"DsuE09_00304" 4221 "s59E5H2" 0 "putative methyltransferase YcgJ"
"DsuE09_00304" 4221 "s63E6H2" 0 "putative methyltransferase YcgJ"
"DsuE09_00305" 4222 "s59E13H2" 0 "CCA-adding enzyme"
"DsuE09_00305" 4222 "s59E25H2" 0 "CCA-adding enzyme"

"DsuE09_00305" 4222 "s59E5H2" 0 "CCA-adding enzyme"
"DsuE09_00305" 4222 "s63E6H2" 0 "CCA-adding enzyme"
"DsuE09_00306" 4223 "s59E13H2" 0 "hypothetical protein"
"DsuE09_00306" 4223 "s59E25H2" 0 "hypothetical protein"
"DsuE09_00306" 4223 "s59E5H2" 0 "hypothetical protein"
"DsuE09_00306" 4223 "s63E6H2" 0 "hypothetical protein"
"DsuE09_00307" 4224 "s59E13H2" 0 "putative inner membrane transporter yiJE"
"DsuE09_00307" 4224 "s59E25H2" 0 "putative inner membrane transporter yiJE"
"DsuE09_00307" 4224 "s59E5H2" 0 "putative inner membrane transporter yiJE"
"DsuE09_00307" 4224 "s63E6H2" 0 "putative inner membrane transporter yiJE"
"DsuE09_00308" 4225 "s59E13H2" 0 "methylcobalamin:coenzyme M methyltransferase"
"DsuE09_00308" 4225 "s59E25H2" 0 "methylcobalamin:coenzyme M methyltransferase"
"DsuE09_00308" 4225 "s59E5H2" 0 "methylcobalamin:coenzyme M methyltransferase"
"DsuE09_00308" 4225 "s63E6H2" 0 "methylcobalamin:coenzyme M methyltransferase"
"DsuE09_00309" 4226 "s59E13H2" 0 "Aspartate aminotransferase"
"DsuE09_00309" 4226 "s59E25H2" 0 "Aspartate aminotransferase"
"DsuE09_00309" 4226 "s59E5H2" 0 "Aspartate aminotransferase"
"DsuE09_00309" 4226 "s63E6H2" 0 "Aspartate aminotransferase"
"DsuE09_00310" 4227 "s59E13H2" 0 "hypothetical protein"
"DsuE09_00310" 4227 "s59E25H2" 0 "hypothetical protein"
"DsuE09_00310" 4227 "s59E5H2" 0 "hypothetical protein"
"DsuE09_00310" 4227 "s63E6H2" 0 "hypothetical protein"
"DsuE09_00311" 4228 "s59E13H2" 0 "Mannosylfructose-phosphate synthase"
"DsuE09_00311" 4228 "s59E25H2" 0 "Mannosylfructose-phosphate synthase"
"DsuE09_00311" 4228 "s59E5H2" 0 "Mannosylfructose-phosphate synthase"
"DsuE09_00311" 4228 "s63E6H2" 0 "Mannosylfructose-phosphate synthase"
"DsuE09_00312" 4229 "s59E13H2" 0 "N(1)-aminopropylagmatine ureohydrolase"
"DsuE09_00312" 4229 "s59E25H2" 0 "N(1)-aminopropylagmatine ureohydrolase"
"DsuE09_00312" 4229 "s59E5H2" 0 "N(1)-aminopropylagmatine ureohydrolase"
"DsuE09_00312" 4229 "s63E6H2" 0 "N(1)-aminopropylagmatine ureohydrolase"
"DsuE09_00313" 4230 "s59E13H2" 0 "S-adenosylmethionine decarboxylase proenzyme precursor"
"DsuE09_00313" 4230 "s59E25H2" 0 "S-adenosylmethionine decarboxylase proenzyme precursor"
"DsuE09_00313" 4230 "s59E5H2" 0 "S-adenosylmethionine decarboxylase proenzyme precursor"
"DsuE09_00313" 4230 "s63E6H2" 0 "S-adenosylmethionine decarboxylase proenzyme precursor"
"DsuE09_00314" 4231 "s59E13H2" 0 "putative isochorismatase"
"DsuE09_00314" 4231 "s59E25H2" 0 "putative isochorismatase"
"DsuE09_00314" 4231 "s59E5H2" 0 "putative isochorismatase"
"DsuE09_00314" 4231 "s63E6H2" 0 "putative isochorismatase"
"DsuE09_00315" 4232 "s59E13H2" 0 "LysM domain protein"
"DsuE09_00315" 4232 "s59E25H2" 0 "LysM domain protein"
"DsuE09_00315" 4232 "s59E5H2" 0 "LysM domain protein"
"DsuE09_00315" 4232 "s63E6H2" 0 "LysM domain protein"
"DsuE09_00316" 4233 "s59E13H2" 0 "3-oxoacyl-[acyl-carrier-protein] reductase FabG"
"DsuE09_00316" 4233 "s59E25H2" 0 "3-oxoacyl-[acyl-carrier-protein] reductase FabG"
"DsuE09_00316" 4233 "s59E5H2" 0 "3-oxoacyl-[acyl-carrier-protein] reductase FabG"
"DsuE09_00316" 4233 "s63E6H2" 0 "3-oxoacyl-[acyl-carrier-protein] reductase FabG"
"DsuE09_00317" 4234 "s59E13H2" 0 "hypothetical protein"

"DsuE09_00317" 4234 "s59E25H2" 0 "hypothetical protein"
"DsuE09_00317" 4234 "s59E5H2" 0 "hypothetical protein"
"DsuE09_00317" 4234 "s63E6H2" 0 "hypothetical protein"
"DsuE09_00318" 4235 "s59E13H2" 0 "hypothetical protein"
"DsuE09_00318" 4235 "s59E25H2" 0 "hypothetical protein"
"DsuE09_00318" 4235 "s59E5H2" 0 "hypothetical protein"
"DsuE09_00318" 4235 "s63E6H2" 0 "hypothetical protein"
"DsuE09_00319" 4236 "s59E13H2" 0 "DNA topoisomerase 1"
"DsuE09_00319" 4236 "s59E25H2" 0 "DNA topoisomerase 1"
"DsuE09_00319" 4236 "s59E5H2" 0 "DNA topoisomerase 1"
"DsuE09_00319" 4236 "s63E6H2" 0 "DNA topoisomerase 1"
"DsuE09_00320" 4237 "s59E13H2" 0 "Deoxyuridine 5'-triphosphate nucleotidohydrolase"
"DsuE09_00320" 4237 "s59E25H2" 0 "Deoxyuridine 5'-triphosphate nucleotidohydrolase"
"DsuE09_00320" 4237 "s59E5H2" 0 "Deoxyuridine 5'-triphosphate nucleotidohydrolase"
"DsuE09_00320" 4237 "s63E6H2" 0 "Deoxyuridine 5'-triphosphate nucleotidohydrolase"
"DsuE09_00321" 4238 "s59E13H2" 0 "Protease 3 precursor"
"DsuE09_00321" 4238 "s59E25H2" 0 "Protease 3 precursor"
"DsuE09_00321" 4238 "s59E5H2" 0 "Protease 3 precursor"
"DsuE09_00321" 4238 "s63E6H2" 0 "Protease 3 precursor"
"DsuE09_00322" 4239 "s59E13H2" 0 "Polyribonucleotide nucleotidyltransferase"
"DsuE09_00322" 4239 "s59E25H2" 0 "Polyribonucleotide nucleotidyltransferase"
"DsuE09_00322" 4239 "s59E5H2" 0 "Polyribonucleotide nucleotidyltransferase"
"DsuE09_00322" 4239 "s63E6H2" 0 "Polyribonucleotide nucleotidyltransferase"
"DsuE09_00323" 4240 "s59E13H2" 0 "30S ribosomal protein S15"
"DsuE09_00323" 4240 "s59E25H2" 0 "30S ribosomal protein S15"
"DsuE09_00323" 4240 "s59E5H2" 0 "30S ribosomal protein S15"
"DsuE09_00323" 4240 "s63E6H2" 0 "30S ribosomal protein S15"
"DsuE09_00324" 4241 "s59E13H2" 0 "tRNA pseudouridine synthase B"
"DsuE09_00324" 4241 "s59E25H2" 0 "tRNA pseudouridine synthase B"
"DsuE09_00324" 4241 "s59E5H2" 0 "tRNA pseudouridine synthase B"
"DsuE09_00324" 4241 "s63E6H2" 0 "tRNA pseudouridine synthase B"
"DsuE09_00325" 4242 "s59E13H2" 0 "Bifunctional oligoribonuclease and PAP phosphatase NrnA"
"DsuE09_00325" 4242 "s59E25H2" 0 "Bifunctional oligoribonuclease and PAP phosphatase NrnA"
"DsuE09_00325" 4242 "s59E5H2" 0 "Bifunctional oligoribonuclease and PAP phosphatase NrnA"
"DsuE09_00325" 4242 "s63E6H2" 0 "Bifunctional oligoribonuclease and PAP phosphatase NrnA"
"DsuE09_00326" 4243 "s59E13H2" 0 "Acyl-CoA dehydrogenase"
"DsuE09_00326" 4243 "s59E25H2" 0 "Acyl-CoA dehydrogenase"
"DsuE09_00326" 4243 "s59E5H2" 0 "Acyl-CoA dehydrogenase"
"DsuE09_00326" 4243 "s63E6H2" 0 "Acyl-CoA dehydrogenase"
"DsuE09_00327" 4244 "s59E13H2" 0 "Methylmalonyl-CoA mutase"
"DsuE09_00327" 4244 "s59E25H2" 0 "Methylmalonyl-CoA mutase"
"DsuE09_00327" 4244 "s59E5H2" 0 "Methylmalonyl-CoA mutase"
"DsuE09_00327" 4244 "s63E6H2" 0 "Methylmalonyl-CoA mutase"
"DsuE09_00328" 4245 "s59E13H2" 0 "Methylmalonyl-CoA mutase"
"DsuE09_00328" 4245 "s59E25H2" 0 "Methylmalonyl-CoA mutase"
"DsuE09_00328" 4245 "s59E5H2" 0 "Methylmalonyl-CoA mutase"
"DsuE09_00328" 4245 "s63E6H2" 0 "Methylmalonyl-CoA mutase"

"DsuE09_00329" 4246 "s59E13H2" 0 "Glutaconyl-CoA decarboxylase subunit beta"
"DsuE09_00329" 4246 "s59E25H2" 0 "Glutaconyl-CoA decarboxylase subunit beta"
"DsuE09_00329" 4246 "s59E5H2" 0 "Glutaconyl-CoA decarboxylase subunit beta"
"DsuE09_00329" 4246 "s63E6H2" 0 "Glutaconyl-CoA decarboxylase subunit beta"
"DsuE09_00330" 4247 "s59E13H2" 0 "hypothetical protein"
"DsuE09_00330" 4247 "s59E25H2" 0 "hypothetical protein"
"DsuE09_00330" 4247 "s59E5H2" 0 "hypothetical protein"
"DsuE09_00330" 4247 "s63E6H2" 0 "hypothetical protein"
"DsuE09_00331" 4248 "s59E13H2" 0 "Glutaconyl-CoA decarboxylase subunit gamma"
"DsuE09_00331" 4248 "s59E25H2" 0 "Glutaconyl-CoA decarboxylase subunit gamma"
"DsuE09_00331" 4248 "s59E5H2" 0 "Glutaconyl-CoA decarboxylase subunit gamma"
"DsuE09_00331" 4248 "s63E6H2" 0 "Glutaconyl-CoA decarboxylase subunit gamma"
"DsuE09_00332" 4249 "s59E13H2" 0 "Aspartate--tRNA ligase"
"DsuE09_00332" 4249 "s59E25H2" 0 "Aspartate--tRNA ligase"
"DsuE09_00332" 4249 "s59E5H2" 0 "Aspartate--tRNA ligase"
"DsuE09_00332" 4249 "s63E6H2" 0 "Aspartate--tRNA ligase"
"DsuE09_00335" 4252 "s59E13H2" 0 "hypothetical protein"
"DsuE09_00335" 4252 "s59E25H2" 0 "hypothetical protein"
"DsuE09_00335" 4252 "s59E5H2" 0 "hypothetical protein"
"DsuE09_00335" 4252 "s63E6H2" 0 "hypothetical protein"
"DsuE09_00336" 4253 "s59E13H2" 0 "hypothetical protein"
"DsuE09_00336" 4253 "s59E25H2" 0 "hypothetical protein"
"DsuE09_00336" 4253 "s59E5H2" 0 "hypothetical protein"
"DsuE09_00336" 4253 "s63E6H2" 0 "hypothetical protein"
"DsuE09_00337" 4254 "s59E13H2" 0 "hypothetical protein"
"DsuE09_00337" 4254 "s59E25H2" 0 "hypothetical protein"
"DsuE09_00337" 4254 "s59E5H2" 0 "hypothetical protein"
"DsuE09_00337" 4254 "s63E6H2" 0 "hypothetical protein"
"DsuE09_00338" 4255 "s59E13H2" 0 "hypothetical protein"
"DsuE09_00338" 4255 "s59E25H2" 0 "hypothetical protein"
"DsuE09_00338" 4255 "s59E5H2" 0 "hypothetical protein"
"DsuE09_00338" 4255 "s63E6H2" 0 "hypothetical protein"
"DsuE09_00339" 4256 "s59E13H2" 0 "photosystem I assembly protein Ycf3"
"DsuE09_00339" 4256 "s59E25H2" 0 "photosystem I assembly protein Ycf3"
"DsuE09_00339" 4256 "s59E5H2" 0 "photosystem I assembly protein Ycf3"
"DsuE09_00339" 4256 "s63E6H2" 0 "photosystem I assembly protein Ycf3"
"DsuE09_00340" 4257 "s59E13H2" 0 "Phosphoglycerate kinase"
"DsuE09_00340" 4257 "s59E25H2" 0 "Phosphoglycerate kinase"
"DsuE09_00340" 4257 "s59E5H2" 0 "Phosphoglycerate kinase"
"DsuE09_00340" 4257 "s63E6H2" 0 "Phosphoglycerate kinase"
"DsuE09_00341" 4258 "s59E13H2" 0 "hypothetical protein"
"DsuE09_00341" 4258 "s59E25H2" 0 "hypothetical protein"
"DsuE09_00341" 4258 "s59E5H2" 0 "hypothetical protein"
"DsuE09_00341" 4258 "s63E6H2" 0 "hypothetical protein"
"DsuE09_00342" 4259 "s59E13H2" 0 "putative inner membrane transporter YicL"
"DsuE09_00342" 4259 "s59E25H2" 0 "putative inner membrane transporter YicL"
"DsuE09_00342" 4259 "s59E5H2" 0 "putative inner membrane transporter YicL"

"DsuE09_00342" 4259 "s63E6H2" 0 "putative inner membrane transporter YicL"
"DsuE09_00343" 4260 "s59E13H2" 0 "Inosine-5'-monophosphate dehydrogenase"
"DsuE09_00343" 4260 "s59E25H2" 0 "Inosine-5'-monophosphate dehydrogenase"
"DsuE09_00343" 4260 "s59E5H2" 0 "Inosine-5'-monophosphate dehydrogenase"
"DsuE09_00343" 4260 "s63E6H2" 0 "Inosine-5'-monophosphate dehydrogenase"
"DsuE09_00344" 4261 "s59E13H2" 0 "dTDP-4-dehydrorhamnose reductase"
"DsuE09_00344" 4261 "s59E25H2" 0 "dTDP-4-dehydrorhamnose reductase"
"DsuE09_00344" 4261 "s59E5H2" 0 "dTDP-4-dehydrorhamnose reductase"
"DsuE09_00344" 4261 "s63E6H2" 0 "dTDP-4-dehydrorhamnose reductase"
"DsuE09_00345" 4262 "s59E13H2" 0 "D-tagatose 3-epimerase"
"DsuE09_00345" 4262 "s59E25H2" 0 "D-tagatose 3-epimerase"
"DsuE09_00345" 4262 "s59E5H2" 0 "D-tagatose 3-epimerase"
"DsuE09_00345" 4262 "s63E6H2" 0 "D-tagatose 3-epimerase"
"DsuE09_00346" 4263 "s59E13H2" 0 "hypothetical protein"
"DsuE09_00346" 4263 "s59E25H2" 0 "hypothetical protein"
"DsuE09_00346" 4263 "s59E5H2" 0 "hypothetical protein"
"DsuE09_00346" 4263 "s63E6H2" 0 "hypothetical protein"
"DsuE09_00347" 4264 "s59E13H2" 0 "Murein DD-endopeptidase MepM"
"DsuE09_00347" 4264 "s59E25H2" 0 "Murein DD-endopeptidase MepM"
"DsuE09_00347" 4264 "s59E5H2" 0 "Murein DD-endopeptidase MepM"
"DsuE09_00347" 4264 "s63E6H2" 0 "Murein DD-endopeptidase MepM"
"DsuE09_00349" 4266 "s59E13H2" 0 "UvrABC system protein B"
"DsuE09_00349" 4266 "s59E25H2" 0 "UvrABC system protein B"
"DsuE09_00349" 4266 "s59E5H2" 0 "UvrABC system protein B"
"DsuE09_00349" 4266 "s63E6H2" 0 "UvrABC system protein B"
"DsuE09_00350" 4267 "s59E13H2" 0 "Cysteine--tRNA ligase"
"DsuE09_00350" 4267 "s59E25H2" 0 "Cysteine--tRNA ligase"
"DsuE09_00350" 4267 "s59E5H2" 0 "Cysteine--tRNA ligase"
"DsuE09_00350" 4267 "s63E6H2" 0 "Cysteine--tRNA ligase"
"DsuE09_00351" 4268 "s59E13H2" 0 "2-C-methyl-D-erythritol 2,4-cyclodiphosphate synthase"
"DsuE09_00351" 4268 "s59E25H2" 0 "2-C-methyl-D-erythritol 2,4-cyclodiphosphate synthase"
"DsuE09_00351" 4268 "s59E5H2" 0 "2-C-methyl-D-erythritol 2,4-cyclodiphosphate synthase"
"DsuE09_00351" 4268 "s63E6H2" 0 "2-C-methyl-D-erythritol 2,4-cyclodiphosphate synthase"
"DsuE09_00353" 4270 "s59E13H2" 0 "Phosphoheptose isomerase"
"DsuE09_00353" 4270 "s59E25H2" 0 "Phosphoheptose isomerase"
"DsuE09_00353" 4270 "s59E5H2" 0 "Phosphoheptose isomerase"
"DsuE09_00353" 4270 "s63E6H2" 0 "Phosphoheptose isomerase"
"DsuE09_00354" 4271 "s59E13H2" 0 "DNA-binding protein HU-beta"
"DsuE09_00354" 4271 "s59E25H2" 0 "DNA-binding protein HU-beta"
"DsuE09_00354" 4271 "s59E5H2" 0 "DNA-binding protein HU-beta"
"DsuE09_00354" 4271 "s63E6H2" 0 "DNA-binding protein HU-beta"
"DsuE09_00355" 4272 "s59E13H2" 0 "L-seryl-tRNA(Sec) selenium transferase"
"DsuE09_00355" 4272 "s59E25H2" 0 "L-seryl-tRNA(Sec) selenium transferase"
"DsuE09_00355" 4272 "s59E5H2" 0 "L-seryl-tRNA(Sec) selenium transferase"
"DsuE09_00355" 4272 "s63E6H2" 0 "L-seryl-tRNA(Sec) selenium transferase"
"DsuE09_00357" 4274 "s59E13H2" 0 "Peptidase family M50"
"DsuE09_00357" 4274 "s59E25H2" 0 "Peptidase family M50"

"DsuE09_00357" 4274 "s59E5H2" 0 "Peptidase family M50"
"DsuE09_00357" 4274 "s63E6H2" 0 "Peptidase family M50"
"DsuE09_00358" 4275 "s59E13H2" 0 "hypothetical protein"
"DsuE09_00358" 4275 "s59E25H2" 0 "hypothetical protein"
"DsuE09_00358" 4275 "s59E5H2" 0 "hypothetical protein"
"DsuE09_00358" 4275 "s63E6H2" 0 "hypothetical protein"
"DsuE09_00359" 4276 "s59E13H2" 0 "F420-non-reducing hydrogenase iron-sulfur subunit D"
"DsuE09_00359" 4276 "s59E25H2" 0 "F420-non-reducing hydrogenase iron-sulfur subunit D"
"DsuE09_00359" 4276 "s59E5H2" 0 "F420-non-reducing hydrogenase iron-sulfur subunit D"
"DsuE09_00359" 4276 "s63E6H2" 0.004966414 "F420-non-reducing hydrogenase iron-sulfur subunit D"
"DsuE09_00360" 4277 "s59E13H2" 0 "CoB--CoM heterodisulfide reductase iron-sulfur subunit A"
"DsuE09_00360" 4277 "s59E25H2" 0 "CoB--CoM heterodisulfide reductase iron-sulfur subunit A"
"DsuE09_00360" 4277 "s59E5H2" 0 "CoB--CoM heterodisulfide reductase iron-sulfur subunit A"
"DsuE09_00360" 4277 "s63E6H2" 0.002912435 "CoB--CoM heterodisulfide reductase iron-sulfur subunit A"
"DsuE09_00361" 4278 "s59E13H2" 0 "Heterodisulfide reductase subunit A-like protein"
"DsuE09_00361" 4278 "s59E25H2" 0 "Heterodisulfide reductase subunit A-like protein"
"DsuE09_00361" 4278 "s59E5H2" 0 "Heterodisulfide reductase subunit A-like protein"
"DsuE09_00361" 4278 "s63E6H2" 0.002165226 "Heterodisulfide reductase subunit A-like protein"
"DsuE09_00362" 4279 "s59E13H2" 0 "formate dehydrogenase accessory protein"
"DsuE09_00362" 4279 "s59E25H2" 0 "formate dehydrogenase accessory protein"
"DsuE09_00362" 4279 "s59E5H2" 0 "formate dehydrogenase accessory protein"
"DsuE09_00362" 4279 "s63E6H2" 0 "formate dehydrogenase accessory protein"
"DsuE09_00363" 4280 "s59E13H2" 0 "CoB--CoM heterodisulfide reductase iron-sulfur subunit D"
"DsuE09_00363" 4280 "s59E25H2" 0 "CoB--CoM heterodisulfide reductase iron-sulfur subunit D"
"DsuE09_00363" 4280 "s59E5H2" 0 "CoB--CoM heterodisulfide reductase iron-sulfur subunit D"
"DsuE09_00363" 4280 "s63E6H2" 0.001324567 "CoB--CoM heterodisulfide reductase iron-sulfur subunit D"
"DsuE09_00364" 4281 "s59E13H2" 0 "hydrogenase 2 protein HybA"
"DsuE09_00364" 4281 "s59E25H2" 0 "hydrogenase 2 protein HybA"
"DsuE09_00364" 4281 "s59E5H2" 0 "hydrogenase 2 protein HybA"
"DsuE09_00364" 4281 "s63E6H2" 0 "hydrogenase 2 protein HybA"
"DsuE09_00365" 4282 "s59E13H2" 0 "hypothetical protein"
"DsuE09_00365" 4282 "s59E25H2" 0 "hypothetical protein"
"DsuE09_00365" 4282 "s59E5H2" 0 "hypothetical protein"
"DsuE09_00365" 4282 "s63E6H2" 0 "hypothetical protein"
"DsuE09_00366" 4283 "s59E13H2" 0 "hypothetical protein"
"DsuE09_00366" 4283 "s59E25H2" 0 "hypothetical protein"
"DsuE09_00366" 4283 "s59E5H2" 0 "hypothetical protein"
"DsuE09_00366" 4283 "s63E6H2" 0 "hypothetical protein"
"DsuE09_00367" 4284 "s59E13H2" 0 "hypothetical protein"
"DsuE09_00367" 4284 "s59E25H2" 0 "hypothetical protein"
"DsuE09_00367" 4284 "s59E5H2" 0 "hypothetical protein"
"DsuE09_00367" 4284 "s63E6H2" 0 "hypothetical protein"
"DsuE09_00368" 4285 "s59E13H2" 0 "Modulator of FtsH protease HflK"
"DsuE09_00368" 4285 "s59E25H2" 0 "Modulator of FtsH protease HflK"
"DsuE09_00368" 4285 "s59E5H2" 0 "Modulator of FtsH protease HflK"
"DsuE09_00368" 4285 "s63E6H2" 0 "Modulator of FtsH protease HflK"
"DsuE09_00369" 4286 "s59E13H2" 0 "Modulator of FtsH protease HflC"

"DsuE09_00369" 4286 "s59E25H2" 0 "Modulator of FtsH protease HflC"
"DsuE09_00369" 4286 "s59E5H2" 0 "Modulator of FtsH protease HflC"
"DsuE09_00369" 4286 "s63E6H2" 0 "Modulator of FtsH protease HflC"
"DsuE09_00370" 4287 "s59E13H2" 0 "hypothetical protein"
"DsuE09_00370" 4287 "s59E25H2" 0 "hypothetical protein"
"DsuE09_00370" 4287 "s59E5H2" 0 "hypothetical protein"
"DsuE09_00370" 4287 "s63E6H2" 0 "hypothetical protein"
"DsuE09_00371" 4288 "s59E13H2" 0 "Single-stranded-DNA-specific exonuclease RecJ"
"DsuE09_00371" 4288 "s59E25H2" 0 "Single-stranded-DNA-specific exonuclease RecJ"
"DsuE09_00371" 4288 "s59E5H2" 0 "Single-stranded-DNA-specific exonuclease RecJ"
"DsuE09_00371" 4288 "s63E6H2" 0 "Single-stranded-DNA-specific exonuclease RecJ"
"DsuE09_00372" 4289 "s59E13H2" 0 "Benzoyl-CoA reductase subunit C"
"DsuE09_00372" 4289 "s59E25H2" 0 "Benzoyl-CoA reductase subunit C"
"DsuE09_00372" 4289 "s59E5H2" 0 "Benzoyl-CoA reductase subunit C"
"DsuE09_00372" 4289 "s63E6H2" 0 "Benzoyl-CoA reductase subunit C"
"DsuE09_00373" 4290 "s59E13H2" 0 "Benzoyl-CoA reductase subunit B"
"DsuE09_00373" 4290 "s59E25H2" 0 "Benzoyl-CoA reductase subunit B"
"DsuE09_00373" 4290 "s59E5H2" 0 "Benzoyl-CoA reductase subunit B"
"DsuE09_00373" 4290 "s63E6H2" 0 "Benzoyl-CoA reductase subunit B"
"DsuE09_00374" 4291 "s59E13H2" 0 "R-phenyllactate dehydratase activator"
"DsuE09_00374" 4291 "s59E25H2" 0 "R-phenyllactate dehydratase activator"
"DsuE09_00374" 4291 "s59E5H2" 0 "R-phenyllactate dehydratase activator"
"DsuE09_00374" 4291 "s63E6H2" 0 "R-phenyllactate dehydratase activator"
"DsuE09_00375" 4292 "s59E13H2" 0 "Membrane-bound lytic murein transglycosylase B precursor"
"DsuE09_00375" 4292 "s59E25H2" 0 "Membrane-bound lytic murein transglycosylase B precursor"
"DsuE09_00375" 4292 "s59E5H2" 0 "Membrane-bound lytic murein transglycosylase B precursor"
"DsuE09_00375" 4292 "s63E6H2" 0 "Membrane-bound lytic murein transglycosylase B precursor"
"DsuE09_00376" 4293 "s59E13H2" 0 "hypothetical protein"
"DsuE09_00376" 4293 "s59E25H2" 0 "hypothetical protein"
"DsuE09_00376" 4293 "s59E5H2" 0 "hypothetical protein"
"DsuE09_00376" 4293 "s63E6H2" 0 "hypothetical protein"
"DsuE09_00377" 4294 "s59E13H2" 0 "tRNA-splicing ligase RtcB"
"DsuE09_00377" 4294 "s59E25H2" 0 "tRNA-splicing ligase RtcB"
"DsuE09_00377" 4294 "s59E5H2" 0 "tRNA-splicing ligase RtcB"
"DsuE09_00377" 4294 "s63E6H2" 0 "tRNA-splicing ligase RtcB"
"DsuE09_00378" 4295 "s59E13H2" 0 "hypothetical protein"
"DsuE09_00378" 4295 "s59E25H2" 0 "hypothetical protein"
"DsuE09_00378" 4295 "s59E5H2" 0 "hypothetical protein"
"DsuE09_00378" 4295 "s63E6H2" 0 "hypothetical protein"
"DsuE09_00379" 4296 "s59E13H2" 0 "Undecaprenyl-phosphate mannosyltransferase"
"DsuE09_00379" 4296 "s59E25H2" 0 "Undecaprenyl-phosphate mannosyltransferase"
"DsuE09_00379" 4296 "s59E5H2" 0 "Undecaprenyl-phosphate mannosyltransferase"
"DsuE09_00379" 4296 "s63E6H2" 0 "Undecaprenyl-phosphate mannosyltransferase"
"DsuE09_00380" 4297 "s59E13H2" 0 "putative glycosyl transferase"
"DsuE09_00380" 4297 "s59E25H2" 0 "putative glycosyl transferase"
"DsuE09_00380" 4297 "s59E5H2" 0 "putative glycosyl transferase"
"DsuE09_00380" 4297 "s63E6H2" 0 "putative glycosyl transferase"

"DsuE09_00381" 4298 "s59E13H2" 0 "putative S-adenosylmethionine-dependent methyltransferase/MSMEI_2290"
"DsuE09_00381" 4298 "s59E25H2" 0 "putative S-adenosylmethionine-dependent methyltransferase/MSMEI_2290"
"DsuE09_00381" 4298 "s59E5H2" 0 "putative S-adenosylmethionine-dependent methyltransferase/MSMEI_2290"
"DsuE09_00381" 4298 "s63E6H2" 0 "putative S-adenosylmethionine-dependent methyltransferase/MSMEI_2290"
"DsuE09_00382" 4299 "s59E13H2" 0 "bifunctional 3-demethylubiquinone-9 3-methyltransferase/ 2-octaprenyl-6-hydroxy phenol methylase"
"DsuE09_00382" 4299 "s59E25H2" 0 "bifunctional 3-demethylubiquinone-9 3-methyltransferase/ 2-octaprenyl-6-hydroxy phenol methylase"
"DsuE09_00382" 4299 "s59E5H2" 0 "bifunctional 3-demethylubiquinone-9 3-methyltransferase/ 2-octaprenyl-6-hydroxy phenol methylase"
"DsuE09_00382" 4299 "s63E6H2" 0 "bifunctional 3-demethylubiquinone-9 3-methyltransferase/ 2-octaprenyl-6-hydroxy phenol methylase"
"DsuE09_00383" 4300 "s59E13H2" 0 "hypothetical protein"
"DsuE09_00383" 4300 "s59E25H2" 0 "hypothetical protein"
"DsuE09_00383" 4300 "s59E5H2" 0 "hypothetical protein"
"DsuE09_00383" 4300 "s63E6H2" 0 "hypothetical protein"
"DsuE09_00384" 4301 "s59E13H2" 0 "coproporphyrinogen III oxidase"
"DsuE09_00384" 4301 "s59E25H2" 0 "coproporphyrinogen III oxidase"
"DsuE09_00384" 4301 "s59E5H2" 0 "coproporphyrinogen III oxidase"
"DsuE09_00384" 4301 "s63E6H2" 0 "coproporphyrinogen III oxidase"
"DsuE09_00385" 4302 "s59E13H2" 0 "Demethylmenaquinone methyltransferase"
"DsuE09_00385" 4302 "s59E25H2" 0 "Demethylmenaquinone methyltransferase"
"DsuE09_00385" 4302 "s59E5H2" 0 "Demethylmenaquinone methyltransferase"
"DsuE09_00385" 4302 "s63E6H2" 0 "Demethylmenaquinone methyltransferase"
"DsuE09_00386" 4303 "s59E13H2" 0 "putative methyltransferase YcgJ"
"DsuE09_00386" 4303 "s59E25H2" 0 "putative methyltransferase YcgJ"
"DsuE09_00386" 4303 "s59E5H2" 0 "putative methyltransferase YcgJ"
"DsuE09_00386" 4303 "s63E6H2" 0 "putative methyltransferase YcgJ"
"DsuE09_00387" 4304 "s59E13H2" 0 "hypothetical protein"
"DsuE09_00387" 4304 "s59E25H2" 0 "hypothetical protein"
"DsuE09_00387" 4304 "s59E5H2" 0 "hypothetical protein"
"DsuE09_00387" 4304 "s63E6H2" 0 "hypothetical protein"
"DsuE09_00388" 4305 "s59E13H2" 0 "Undecaprenyl-phosphate mannosyltransferase"
"DsuE09_00388" 4305 "s59E25H2" 0 "Undecaprenyl-phosphate mannosyltransferase"
"DsuE09_00388" 4305 "s59E5H2" 0 "Undecaprenyl-phosphate mannosyltransferase"
"DsuE09_00388" 4305 "s63E6H2" 0 "Undecaprenyl-phosphate mannosyltransferase"
"DsuE09_00389" 4306 "s59E13H2" 0 "Phd_YefM"
"DsuE09_00389" 4306 "s59E25H2" 0 "Phd_YefM"
"DsuE09_00389" 4306 "s59E5H2" 0 "Phd_YefM"
"DsuE09_00389" 4306 "s63E6H2" 0 "Phd_YefM"
"DsuE09_00390" 4307 "s59E13H2" 0 "GDP-mannose-dependent alpha-(1-6)-phosphatidylinositol monomannoside mannosyltransferase"
"DsuE09_00390" 4307 "s59E25H2" 0 "GDP-mannose-dependent alpha-(1-6)-phosphatidylinositol monomannoside mannosyltransferase"
"DsuE09_00390" 4307 "s59E5H2" 0 "GDP-mannose-dependent alpha-(1-6)-phosphatidylinositol monomannoside mannosyltransferase"
"DsuE09_00390" 4307 "s63E6H2" 0 "GDP-mannose-dependent alpha-(1-6)-phosphatidylinositol monomannoside mannosyltransferase"
"DsuE09_00391" 4308 "s59E13H2" 0 "Chondroitin synthase"
"DsuE09_00391" 4308 "s59E25H2" 0 "Chondroitin synthase"
"DsuE09_00391" 4308 "s59E5H2" 0 "Chondroitin synthase"
"DsuE09_00391" 4308 "s63E6H2" 0 "Chondroitin synthase"
"DsuE09_00392" 4309 "s59E13H2" 0 "Polysaccharide biosynthesis protein"
"DsuE09_00392" 4309 "s59E25H2" 0 "Polysaccharide biosynthesis protein"
"DsuE09_00392" 4309 "s59E5H2" 0 "Polysaccharide biosynthesis protein"

"DsuE09_00392" 4309 "s63E6H2" 0 "Polysaccharide biosynthesis protein"
"DsuE09_00393" 4310 "s59E13H2" 0 "Methyltransferase domain protein"
"DsuE09_00393" 4310 "s59E25H2" 0 "Methyltransferase domain protein"
"DsuE09_00393" 4310 "s59E5H2" 0 "Methyltransferase domain protein"
"DsuE09_00393" 4310 "s63E6H2" 0 "Methyltransferase domain protein"
"DsuE09_00394" 4311 "s59E13H2" 0 "dTDP-4-amino-4,6-dideoxy-D-glucose acyltransferase"
"DsuE09_00394" 4311 "s59E25H2" 0 "dTDP-4-amino-4,6-dideoxy-D-glucose acyltransferase"
"DsuE09_00394" 4311 "s59E5H2" 0 "dTDP-4-amino-4,6-dideoxy-D-glucose acyltransferase"
"DsuE09_00394" 4311 "s63E6H2" 0 "dTDP-4-amino-4,6-dideoxy-D-glucose acyltransferase"
"DsuE09_00395" 4312 "s59E13H2" 0 "dTDP-4-amino-4,6-dideoxy-D-glucose transaminase"
"DsuE09_00395" 4312 "s59E25H2" 0 "dTDP-4-amino-4,6-dideoxy-D-glucose transaminase"
"DsuE09_00395" 4312 "s59E5H2" 0 "dTDP-4-amino-4,6-dideoxy-D-glucose transaminase"
"DsuE09_00395" 4312 "s63E6H2" 0 "dTDP-4-amino-4,6-dideoxy-D-glucose transaminase"
"DsuE09_00396" 4313 "s59E13H2" 0 "GalNAc(5)-diNAcBac-PP-undecaprenol beta-1,3-glucosyltransferase"
"DsuE09_00396" 4313 "s59E25H2" 0 "GalNAc(5)-diNAcBac-PP-undecaprenol beta-1,3-glucosyltransferase"
"DsuE09_00396" 4313 "s59E5H2" 0 "GalNAc(5)-diNAcBac-PP-undecaprenol beta-1,3-glucosyltransferase"
"DsuE09_00396" 4313 "s63E6H2" 0 "GalNAc(5)-diNAcBac-PP-undecaprenol beta-1,3-glucosyltransferase"
"DsuE09_00397" 4314 "s59E13H2" 0 "Glycosyltransferase AglJ"
"DsuE09_00397" 4314 "s59E25H2" 0 "Glycosyltransferase AglJ"
"DsuE09_00397" 4314 "s59E5H2" 0 "Glycosyltransferase AglJ"
"DsuE09_00397" 4314 "s63E6H2" 0 "Glycosyltransferase AglJ"
"DsuE09_00398" 4315 "s59E13H2" 0 "Phd_YefM"
"DsuE09_00398" 4315 "s59E25H2" 0 "Phd_YefM"
"DsuE09_00398" 4315 "s59E5H2" 0 "Phd_YefM"
"DsuE09_00398" 4315 "s63E6H2" 0 "Phd_YefM"
"DsuE09_00399" 4316 "s59E13H2" 0 "hypothetical protein"
"DsuE09_00399" 4316 "s59E25H2" 0 "hypothetical protein"
"DsuE09_00399" 4316 "s59E5H2" 0 "hypothetical protein"
"DsuE09_00399" 4316 "s63E6H2" 0 "hypothetical protein"
"DsuE09_00400" 4317 "s59E13H2" 0 "hypothetical protein"
"DsuE09_00400" 4317 "s59E25H2" 0 "hypothetical protein"
"DsuE09_00400" 4317 "s59E5H2" 0 "hypothetical protein"
"DsuE09_00400" 4317 "s63E6H2" 0 "hypothetical protein"
"DsuE09_00401" 4318 "s59E13H2" 0 "tRNA(fMet)-specific endonuclease VapC"
"DsuE09_00401" 4318 "s59E25H2" 0 "tRNA(fMet)-specific endonuclease VapC"
"DsuE09_00401" 4318 "s59E5H2" 0 "tRNA(fMet)-specific endonuclease VapC"
"DsuE09_00401" 4318 "s63E6H2" 0 "tRNA(fMet)-specific endonuclease VapC"
"DsuE09_00402" 4319 "s59E13H2" 0 "Phd_YefM"
"DsuE09_00402" 4319 "s59E25H2" 0 "Phd_YefM"
"DsuE09_00402" 4319 "s59E5H2" 0 "Phd_YefM"
"DsuE09_00402" 4319 "s63E6H2" 0 "Phd_YefM"
"DsuE09_00403" 4320 "s59E13H2" 0 "hypothetical protein"
"DsuE09_00403" 4320 "s59E25H2" 0 "hypothetical protein"
"DsuE09_00403" 4320 "s59E5H2" 0 "hypothetical protein"
"DsuE09_00403" 4320 "s63E6H2" 0 "hypothetical protein"
"DsuE09_00404" 4321 "s59E13H2" 0 "bifunctional 3-demethylubiquinone-9 3-methyltransferase/ 2-octaprenyl-6-hydroxy phenol methylase"
"DsuE09_00404" 4321 "s59E25H2" 0 "bifunctional 3-demethylubiquinone-9 3-methyltransferase/ 2-octaprenyl-6-hydroxy phenol methylase"

"DsuE09_00404" 4321 "s59E5H2" 0 "bifunctional 3-demethylubiquinone-9 3-methyltransferase/ 2-octaprenyl-6-hydroxy phenol methylase"
"DsuE09_00404" 4321 "s63E6H2" 0 "bifunctional 3-demethylubiquinone-9 3-methyltransferase/ 2-octaprenyl-6-hydroxy phenol methylase"
"DsuE09_00405" 4322 "s59E13H2" 0 "Glycosyltransferase AglD"
"DsuE09_00405" 4322 "s59E25H2" 0 "Glycosyltransferase AglD"
"DsuE09_00405" 4322 "s59E5H2" 0 "Glycosyltransferase AglD"
"DsuE09_00405" 4322 "s63E6H2" 0 "Glycosyltransferase AglD"
"DsuE09_00406" 4323 "s59E13H2" 0 "photosystem I assembly protein Ycf3"
"DsuE09_00406" 4323 "s59E25H2" 0 "photosystem I assembly protein Ycf3"
"DsuE09_00406" 4323 "s59E5H2" 0 "photosystem I assembly protein Ycf3"
"DsuE09_00406" 4323 "s63E6H2" 0 "photosystem I assembly protein Ycf3"
"DsuE09_00408" 4325 "s59E13H2" 0 "photosystem I assembly protein Ycf3"
"DsuE09_00408" 4325 "s59E25H2" 0 "photosystem I assembly protein Ycf3"
"DsuE09_00408" 4325 "s59E5H2" 0 "photosystem I assembly protein Ycf3"
"DsuE09_00408" 4325 "s63E6H2" 0 "photosystem I assembly protein Ycf3"
"DsuE09_00409" 4326 "s59E13H2" 0 "photosystem I assembly protein Ycf3"
"DsuE09_00409" 4326 "s59E25H2" 0 "photosystem I assembly protein Ycf3"
"DsuE09_00409" 4326 "s59E5H2" 0 "photosystem I assembly protein Ycf3"
"DsuE09_00409" 4326 "s63E6H2" 0 "photosystem I assembly protein Ycf3"
"DsuE09_00410" 4327 "s59E13H2" 0 "Type II secretion system protein G precursor"
"DsuE09_00410" 4327 "s59E25H2" 0 "Type II secretion system protein G precursor"
"DsuE09_00410" 4327 "s59E5H2" 0 "Type II secretion system protein G precursor"
"DsuE09_00410" 4327 "s63E6H2" 0 "Type II secretion system protein G precursor"
"DsuE09_00412" 4329 "s59E13H2" 0 "Transcriptional regulatory protein ZraR"
"DsuE09_00412" 4329 "s59E25H2" 0 "Transcriptional regulatory protein ZraR"
"DsuE09_00412" 4329 "s59E5H2" 0 "Transcriptional regulatory protein ZraR"
"DsuE09_00412" 4329 "s63E6H2" 0 "Transcriptional regulatory protein ZraR"
"DsuE09_00413" 4330 "s59E13H2" 0 "Dihydroorotase"
"DsuE09_00413" 4330 "s59E25H2" 0 "Dihydroorotase"
"DsuE09_00413" 4330 "s59E5H2" 0 "Dihydroorotase"
"DsuE09_00413" 4330 "s63E6H2" 0 "Dihydroorotase"
"DsuE09_00414" 4331 "s59E13H2" 0 "Aspartate carbamoyltransferase"
"DsuE09_00414" 4331 "s59E25H2" 0 "Aspartate carbamoyltransferase"
"DsuE09_00414" 4331 "s59E5H2" 0 "Aspartate carbamoyltransferase"
"DsuE09_00414" 4331 "s63E6H2" 0 "Aspartate carbamoyltransferase"
"DsuE09_00415" 4332 "s59E13H2" 0 "50S ribosomal protein L17"
"DsuE09_00415" 4332 "s59E25H2" 0 "50S ribosomal protein L17"
"DsuE09_00415" 4332 "s59E5H2" 0 "50S ribosomal protein L17"
"DsuE09_00415" 4332 "s63E6H2" 0 "50S ribosomal protein L17"
"DsuE09_00416" 4333 "s59E13H2" 0 "DNA-directed RNA polymerase subunit alpha"
"DsuE09_00416" 4333 "s59E25H2" 0 "DNA-directed RNA polymerase subunit alpha"
"DsuE09_00416" 4333 "s59E5H2" 0 "DNA-directed RNA polymerase subunit alpha"
"DsuE09_00416" 4333 "s63E6H2" 0 "DNA-directed RNA polymerase subunit alpha"
"DsuE09_00417" 4334 "s59E13H2" 0 "30S ribosomal protein S4"
"DsuE09_00417" 4334 "s59E25H2" 0 "30S ribosomal protein S4"
"DsuE09_00417" 4334 "s59E5H2" 0 "30S ribosomal protein S4"
"DsuE09_00417" 4334 "s63E6H2" 0 "30S ribosomal protein S4"
"DsuE09_00418" 4335 "s59E13H2" 0 "30S ribosomal protein S11"

"DsuE09_00418" 4335 "s59E25H2" 0 "30S ribosomal protein S11"
"DsuE09_00418" 4335 "s59E5H2" 0 "30S ribosomal protein S11"
"DsuE09_00418" 4335 "s63E6H2" 0 "30S ribosomal protein S11"
"DsuE09_00419" 4336 "s59E13H2" 0 "30S ribosomal protein S13"
"DsuE09_00419" 4336 "s59E25H2" 0 "30S ribosomal protein S13"
"DsuE09_00419" 4336 "s59E5H2" 0 "30S ribosomal protein S13"
"DsuE09_00419" 4336 "s63E6H2" 0 "30S ribosomal protein S13"
"DsuE09_00420" 4337 "s59E13H2" 0 "preprotein translocase subunit SecY"
"DsuE09_00420" 4337 "s59E25H2" 0 "preprotein translocase subunit SecY"
"DsuE09_00420" 4337 "s59E5H2" 0 "preprotein translocase subunit SecY"
"DsuE09_00420" 4337 "s63E6H2" 0 "preprotein translocase subunit SecY"
"DsuE09_00421" 4338 "s59E13H2" 0 "50S ribosomal protein L15"
"DsuE09_00421" 4338 "s59E25H2" 0 "50S ribosomal protein L15"
"DsuE09_00421" 4338 "s59E5H2" 0 "50S ribosomal protein L15"
"DsuE09_00421" 4338 "s63E6H2" 0 "50S ribosomal protein L15"
"DsuE09_00422" 4339 "s59E13H2" 0 "30S ribosomal protein S5"
"DsuE09_00422" 4339 "s59E25H2" 0 "30S ribosomal protein S5"
"DsuE09_00422" 4339 "s59E5H2" 0 "30S ribosomal protein S5"
"DsuE09_00422" 4339 "s63E6H2" 0 "30S ribosomal protein S5"
"DsuE09_00423" 4340 "s59E13H2" 0 "50S ribosomal protein L18"
"DsuE09_00423" 4340 "s59E25H2" 0 "50S ribosomal protein L18"
"DsuE09_00423" 4340 "s59E5H2" 0 "50S ribosomal protein L18"
"DsuE09_00423" 4340 "s63E6H2" 0 "50S ribosomal protein L18"
"DsuE09_00424" 4341 "s59E13H2" 0 "50S ribosomal protein L6"
"DsuE09_00424" 4341 "s59E25H2" 0 "50S ribosomal protein L6"
"DsuE09_00424" 4341 "s59E5H2" 0 "50S ribosomal protein L6"
"DsuE09_00424" 4341 "s63E6H2" 0 "50S ribosomal protein L6"
"DsuE09_00425" 4342 "s59E13H2" 0 "30S ribosomal protein S8"
"DsuE09_00425" 4342 "s59E25H2" 0 "30S ribosomal protein S8"
"DsuE09_00425" 4342 "s59E5H2" 0 "30S ribosomal protein S8"
"DsuE09_00425" 4342 "s63E6H2" 0 "30S ribosomal protein S8"
"DsuE09_00426" 4343 "s59E13H2" 0 "50S ribosomal protein L5"
"DsuE09_00426" 4343 "s59E25H2" 0 "50S ribosomal protein L5"
"DsuE09_00426" 4343 "s59E5H2" 0 "50S ribosomal protein L5"
"DsuE09_00426" 4343 "s63E6H2" 0 "50S ribosomal protein L5"
"DsuE09_00427" 4344 "s59E13H2" 0 "50S ribosomal protein L24"
"DsuE09_00427" 4344 "s59E25H2" 0 "50S ribosomal protein L24"
"DsuE09_00427" 4344 "s59E5H2" 0 "50S ribosomal protein L24"
"DsuE09_00427" 4344 "s63E6H2" 0 "50S ribosomal protein L24"
"DsuE09_00428" 4345 "s59E13H2" 0 "50S ribosomal protein L14"
"DsuE09_00428" 4345 "s59E25H2" 0 "50S ribosomal protein L14"
"DsuE09_00428" 4345 "s59E5H2" 0 "50S ribosomal protein L14"
"DsuE09_00428" 4345 "s63E6H2" 0 "50S ribosomal protein L14"
"DsuE09_00429" 4346 "s59E13H2" 0 "30S ribosomal protein S17"
"DsuE09_00429" 4346 "s59E25H2" 0 "30S ribosomal protein S17"
"DsuE09_00429" 4346 "s59E5H2" 0 "30S ribosomal protein S17"
"DsuE09_00429" 4346 "s63E6H2" 0 "30S ribosomal protein S17"

"DsuE09_00430" 4347 "s59E13H2" 0 "50S ribosomal protein L29"
"DsuE09_00430" 4347 "s59E25H2" 0 "50S ribosomal protein L29"
"DsuE09_00430" 4347 "s59E5H2" 0 "50S ribosomal protein L29"
"DsuE09_00430" 4347 "s63E6H2" 0 "50S ribosomal protein L29"
"DsuE09_00431" 4348 "s59E13H2" 0 "50S ribosomal protein L16"
"DsuE09_00431" 4348 "s59E25H2" 0 "50S ribosomal protein L16"
"DsuE09_00431" 4348 "s59E5H2" 0 "50S ribosomal protein L16"
"DsuE09_00431" 4348 "s63E6H2" 0 "50S ribosomal protein L16"
"DsuE09_00432" 4349 "s59E13H2" 0.001251789 "30S ribosomal protein S3"
"DsuE09_00432" 4349 "s59E25H2" 0 "30S ribosomal protein S3"
"DsuE09_00432" 4349 "s59E5H2" 0 "30S ribosomal protein S3"
"DsuE09_00432" 4349 "s63E6H2" 0 "30S ribosomal protein S3"
"DsuE09_00433" 4350 "s59E13H2" 0 "50S ribosomal protein L22"
"DsuE09_00433" 4350 "s59E25H2" 0 "50S ribosomal protein L22"
"DsuE09_00433" 4350 "s59E5H2" 0 "50S ribosomal protein L22"
"DsuE09_00433" 4350 "s63E6H2" 0 "50S ribosomal protein L22"
"DsuE09_00434" 4351 "s59E13H2" 0 "30S ribosomal protein S19"
"DsuE09_00434" 4351 "s59E25H2" 0 "30S ribosomal protein S19"
"DsuE09_00434" 4351 "s59E5H2" 0 "30S ribosomal protein S19"
"DsuE09_00434" 4351 "s63E6H2" 0 "30S ribosomal protein S19"
"DsuE09_00435" 4352 "s59E13H2" 0 "50S ribosomal protein L2"
"DsuE09_00435" 4352 "s59E25H2" 0 "50S ribosomal protein L2"
"DsuE09_00435" 4352 "s59E5H2" 0 "50S ribosomal protein L2"
"DsuE09_00435" 4352 "s63E6H2" 0 "50S ribosomal protein L2"
"DsuE09_00436" 4353 "s59E13H2" 0 "50S ribosomal protein L23"
"DsuE09_00436" 4353 "s59E25H2" 0 "50S ribosomal protein L23"
"DsuE09_00436" 4353 "s59E5H2" 0 "50S ribosomal protein L23"
"DsuE09_00436" 4353 "s63E6H2" 0 "50S ribosomal protein L23"
"DsuE09_00437" 4354 "s59E13H2" 0 "50S ribosomal protein L4"
"DsuE09_00437" 4354 "s59E25H2" 0 "50S ribosomal protein L4"
"DsuE09_00437" 4354 "s59E5H2" 0 "50S ribosomal protein L4"
"DsuE09_00437" 4354 "s63E6H2" 0 "50S ribosomal protein L4"
"DsuE09_00438" 4355 "s59E13H2" 0 "50S ribosomal protein L3"
"DsuE09_00438" 4355 "s59E25H2" 0 "50S ribosomal protein L3"
"DsuE09_00438" 4355 "s59E5H2" 0 "50S ribosomal protein L3"
"DsuE09_00438" 4355 "s63E6H2" 0 "50S ribosomal protein L3"
"DsuE09_00439" 4356 "s59E13H2" 0 "30S ribosomal protein S10"
"DsuE09_00439" 4356 "s59E25H2" 0 "30S ribosomal protein S10"
"DsuE09_00439" 4356 "s59E5H2" 0 "30S ribosomal protein S10"
"DsuE09_00439" 4356 "s63E6H2" 0 "30S ribosomal protein S10"
"DsuE09_00440" 4357 "s59E13H2" 0 "Elongation factor Tu"
"DsuE09_00440" 4357 "s59E25H2" 0 "Elongation factor Tu"
"DsuE09_00440" 4357 "s59E5H2" 0 "Elongation factor Tu"
"DsuE09_00440" 4357 "s63E6H2" 0 "Elongation factor Tu"
"DsuE09_00441" 4358 "s59E13H2" 0 "30S ribosomal protein S7"
"DsuE09_00441" 4358 "s59E25H2" 0 "30S ribosomal protein S7"
"DsuE09_00441" 4358 "s59E5H2" 0 "30S ribosomal protein S7"

"DsuE09_00441" 4358 "s63E6H2" 0 "30S ribosomal protein S7"
"DsuE09_00442" 4359 "s59E13H2" 0 "30S ribosomal protein S12"
"DsuE09_00442" 4359 "s59E25H2" 0 "30S ribosomal protein S12"
"DsuE09_00442" 4359 "s59E5H2" 0 "30S ribosomal protein S12"
"DsuE09_00442" 4359 "s63E6H2" 0 "30S ribosomal protein S12"
"DsuE09_00443" 4360 "s59E13H2" 0 "DNA-directed RNA polymerase subunit beta'"
"DsuE09_00443" 4360 "s59E25H2" 0 "DNA-directed RNA polymerase subunit beta'"
"DsuE09_00443" 4360 "s59E5H2" 0 "DNA-directed RNA polymerase subunit beta'"
"DsuE09_00443" 4360 "s63E6H2" 0 "DNA-directed RNA polymerase subunit beta'"
"DsuE09_00444" 4361 "s59E13H2" 0 "DNA-directed RNA polymerase subunit beta"
"DsuE09_00444" 4361 "s59E25H2" 0 "DNA-directed RNA polymerase subunit beta"
"DsuE09_00444" 4361 "s59E5H2" 0 "DNA-directed RNA polymerase subunit beta"
"DsuE09_00444" 4361 "s63E6H2" 0 "DNA-directed RNA polymerase subunit beta"
"DsuE09_00445" 4362 "s59E13H2" 0 "50S ribosomal protein L7/L12"
"DsuE09_00445" 4362 "s59E25H2" 0 "50S ribosomal protein L7/L12"
"DsuE09_00445" 4362 "s59E5H2" 0 "50S ribosomal protein L7/L12"
"DsuE09_00445" 4362 "s63E6H2" 0 "50S ribosomal protein L7/L12"
"DsuE09_00446" 4363 "s59E13H2" 0 "50S ribosomal protein L10"
"DsuE09_00446" 4363 "s59E25H2" 0 "50S ribosomal protein L10"
"DsuE09_00446" 4363 "s59E5H2" 0 "50S ribosomal protein L10"
"DsuE09_00446" 4363 "s63E6H2" 0 "50S ribosomal protein L10"
"DsuE09_00447" 4364 "s59E13H2" 0 "50S ribosomal protein L1"
"DsuE09_00447" 4364 "s59E25H2" 0 "50S ribosomal protein L1"
"DsuE09_00447" 4364 "s59E5H2" 0 "50S ribosomal protein L1"
"DsuE09_00447" 4364 "s63E6H2" 0 "50S ribosomal protein L1"
"DsuE09_00448" 4365 "s59E13H2" 0 "50S ribosomal protein L11"
"DsuE09_00448" 4365 "s59E25H2" 0 "50S ribosomal protein L11"
"DsuE09_00448" 4365 "s59E5H2" 0 "50S ribosomal protein L11"
"DsuE09_00448" 4365 "s63E6H2" 0 "50S ribosomal protein L11"
"DsuE09_00449" 4366 "s59E13H2" 0 "hypothetical protein"
"DsuE09_00449" 4366 "s59E25H2" 0 "hypothetical protein"
"DsuE09_00449" 4366 "s59E5H2" 0 "hypothetical protein"
"DsuE09_00449" 4366 "s63E6H2" 0 "hypothetical protein"
"DsuE09_00450" 4367 "s59E13H2" 0 "preprotein translocase subunit SecE"
"DsuE09_00450" 4367 "s59E25H2" 0 "preprotein translocase subunit SecE"
"DsuE09_00450" 4367 "s59E5H2" 0 "preprotein translocase subunit SecE"
"DsuE09_00450" 4367 "s63E6H2" 0 "preprotein translocase subunit SecE"
"DsuE09_00458" 4375 "s59E13H2" 0 "putative HTH-type transcriptional regulator"
"DsuE09_00458" 4375 "s59E25H2" 0 "putative HTH-type transcriptional regulator"
"DsuE09_00458" 4375 "s59E5H2" 0 "putative HTH-type transcriptional regulator"
"DsuE09_00458" 4375 "s63E6H2" 0 "putative HTH-type transcriptional regulator"
"DsuE09_00459" 4376 "s59E13H2" 0 "Antilisterial bacteriocin subtilosin biosynthesis protein AlbA"
"DsuE09_00459" 4376 "s59E25H2" 0 "Antilisterial bacteriocin subtilosin biosynthesis protein AlbA"
"DsuE09_00459" 4376 "s59E5H2" 0 "Antilisterial bacteriocin subtilosin biosynthesis protein AlbA"
"DsuE09_00459" 4376 "s63E6H2" 0 "Antilisterial bacteriocin subtilosin biosynthesis protein AlbA"
"DsuE09_00460" 4377 "s59E13H2" 0 "Delta-aminolevulinic acid dehydratase"
"DsuE09_00460" 4377 "s59E25H2" 0 "Delta-aminolevulinic acid dehydratase"

"DsuE09_00460" 4377 "s59E5H2" 0 "Delta-aminolevulinic acid dehydratase"
"DsuE09_00460" 4377 "s63E6H2" 0 "Delta-aminolevulinic acid dehydratase"
"DsuE09_00461" 4378 "s59E13H2" 0 "ATP-dependent Clp protease ATP-binding subunit ClpX"
"DsuE09_00461" 4378 "s59E25H2" 0 "ATP-dependent Clp protease ATP-binding subunit ClpX"
"DsuE09_00461" 4378 "s59E5H2" 0 "ATP-dependent Clp protease ATP-binding subunit ClpX"
"DsuE09_00461" 4378 "s63E6H2" 0 "ATP-dependent Clp protease ATP-binding subunit ClpX"
"DsuE09_00464" 4381 "s59E13H2" 0 "Rhomboid family protein"
"DsuE09_00464" 4381 "s59E25H2" 0 "Rhomboid family protein"
"DsuE09_00464" 4381 "s59E5H2" 0 "Rhomboid family protein"
"DsuE09_00464" 4381 "s63E6H2" 0 "Rhomboid family protein"
"DsuE09_00465" 4382 "s59E13H2" 0 "hypothetical protein"
"DsuE09_00465" 4382 "s59E25H2" 0 "hypothetical protein"
"DsuE09_00465" 4382 "s59E5H2" 0 "hypothetical protein"
"DsuE09_00465" 4382 "s63E6H2" 0 "hypothetical protein"
"DsuE09_00466" 4383 "s59E13H2" 0 "D-inositol 3-phosphate glycosyltransferase"
"DsuE09_00466" 4383 "s59E25H2" 0 "D-inositol 3-phosphate glycosyltransferase"
"DsuE09_00466" 4383 "s59E5H2" 0 "D-inositol 3-phosphate glycosyltransferase"
"DsuE09_00466" 4383 "s63E6H2" 0 "D-inositol 3-phosphate glycosyltransferase"
"DsuE09_00467" 4384 "s59E13H2" 0 "Mycothiol acetyltransferase"
"DsuE09_00467" 4384 "s59E25H2" 0 "Mycothiol acetyltransferase"
"DsuE09_00467" 4384 "s59E5H2" 0 "Mycothiol acetyltransferase"
"DsuE09_00467" 4384 "s63E6H2" 0 "Mycothiol acetyltransferase"
"DsuE09_00468" 4385 "s59E13H2" 0 "Diacetylchitobiose deacetylase"
"DsuE09_00468" 4385 "s59E25H2" 0 "Diacetylchitobiose deacetylase"
"DsuE09_00468" 4385 "s59E5H2" 0 "Diacetylchitobiose deacetylase"
"DsuE09_00468" 4385 "s63E6H2" 0 "Diacetylchitobiose deacetylase"
"DsuE09_00469" 4386 "s59E13H2" 0 "Hydroxyacylglutathione hydrolase"
"DsuE09_00469" 4386 "s59E25H2" 0 "Hydroxyacylglutathione hydrolase"
"DsuE09_00469" 4386 "s59E5H2" 0 "Hydroxyacylglutathione hydrolase"
"DsuE09_00469" 4386 "s63E6H2" 0 "Hydroxyacylglutathione hydrolase"
"DsuE09_00470" 4387 "s59E13H2" 0 "FecR protein"
"DsuE09_00470" 4387 "s59E25H2" 0 "FecR protein"
"DsuE09_00470" 4387 "s59E5H2" 0 "FecR protein"
"DsuE09_00470" 4387 "s63E6H2" 0 "FecR protein"
"DsuE09_00472" 4389 "s59E13H2" 0 "putative phosphatase"
"DsuE09_00472" 4389 "s59E25H2" 0 "putative phosphatase"
"DsuE09_00472" 4389 "s59E5H2" 0 "putative phosphatase"
"DsuE09_00472" 4389 "s63E6H2" 0 "putative phosphatase"
"DsuE09_00473" 4390 "s59E13H2" 0 "Integration host factor subunit alpha"
"DsuE09_00473" 4390 "s59E25H2" 0 "Integration host factor subunit alpha"
"DsuE09_00473" 4390 "s59E5H2" 0 "Integration host factor subunit alpha"
"DsuE09_00473" 4390 "s63E6H2" 0 "Integration host factor subunit alpha"
"DsuE09_00475" 4392 "s59E13H2" 0 "SsrA-binding protein"
"DsuE09_00475" 4392 "s59E25H2" 0 "SsrA-binding protein"
"DsuE09_00475" 4392 "s59E5H2" 0 "SsrA-binding protein"
"DsuE09_00475" 4392 "s63E6H2" 0 "SsrA-binding protein"
"DsuE09_00476" 4393 "s59E13H2" 0 "Phosphoenolpyruvate-protein phosphotransferase"

"DsuE09_00476" 4393 "s59E25H2" 0 "Phosphoenolpyruvate-protein phosphotransferase"
"DsuE09_00476" 4393 "s59E5H2" 0.009233639 "Phosphoenolpyruvate-protein phosphotransferase"
"DsuE09_00476" 4393 "s63E6H2" 0 "Phosphoenolpyruvate-protein phosphotransferase"
"DsuE09_00477" 4394 "s59E13H2" 0 "Ribosomal RNA small subunit methyltransferase I"
"DsuE09_00477" 4394 "s59E25H2" 0 "Ribosomal RNA small subunit methyltransferase I"
"DsuE09_00477" 4394 "s59E5H2" 0 "Ribosomal RNA small subunit methyltransferase I"
"DsuE09_00477" 4394 "s63E6H2" 0 "Ribosomal RNA small subunit methyltransferase I"
"DsuE09_00478" 4395 "s59E13H2" 0 "hypothetical protein"
"DsuE09_00478" 4395 "s59E25H2" 0 "hypothetical protein"
"DsuE09_00478" 4395 "s59E5H2" 0 "hypothetical protein"
"DsuE09_00478" 4395 "s63E6H2" 0 "hypothetical protein"
"DsuE09_00479" 4396 "s59E13H2" 0 "Ribonuclease HII"
"DsuE09_00479" 4396 "s59E25H2" 0 "Ribonuclease HII"
"DsuE09_00479" 4396 "s59E5H2" 0 "Ribonuclease HII"
"DsuE09_00479" 4396 "s63E6H2" 0 "Ribonuclease HII"
"DsuE09_00480" 4397 "s59E13H2" 0 "50S ribosomal protein L19"
"DsuE09_00480" 4397 "s59E25H2" 0 "50S ribosomal protein L19"
"DsuE09_00480" 4397 "s59E5H2" 0 "50S ribosomal protein L19"
"DsuE09_00480" 4397 "s63E6H2" 0 "50S ribosomal protein L19"
"DsuE09_00481" 4398 "s59E13H2" 0 "hypothetical protein"
"DsuE09_00481" 4398 "s59E25H2" 0 "hypothetical protein"
"DsuE09_00481" 4398 "s59E5H2" 0 "hypothetical protein"
"DsuE09_00481" 4398 "s63E6H2" 0 "hypothetical protein"
"DsuE09_00482" 4399 "s59E13H2" 0 "tRNA (guanine-N(1)-)-methyltransferase"
"DsuE09_00482" 4399 "s59E25H2" 0 "tRNA (guanine-N(1)-)-methyltransferase"
"DsuE09_00482" 4399 "s59E5H2" 0 "tRNA (guanine-N(1)-)-methyltransferase"
"DsuE09_00482" 4399 "s63E6H2" 0 "tRNA (guanine-N(1)-)-methyltransferase"
"DsuE09_00483" 4400 "s59E13H2" 0 "Ribosome maturation factor RimM"
"DsuE09_00483" 4400 "s59E25H2" 0 "Ribosome maturation factor RimM"
"DsuE09_00483" 4400 "s59E5H2" 0 "Ribosome maturation factor RimM"
"DsuE09_00483" 4400 "s63E6H2" 0 "Ribosome maturation factor RimM"
"DsuE09_00484" 4401 "s59E13H2" 0 "hypothetical protein"
"DsuE09_00484" 4401 "s59E25H2" 0 "hypothetical protein"
"DsuE09_00484" 4401 "s59E5H2" 0.01812704 "hypothetical protein"
"DsuE09_00484" 4401 "s63E6H2" 0 "hypothetical protein"
"DsuE09_00485" 4402 "s59E13H2" 0 "30S ribosomal protein S16"
"DsuE09_00485" 4402 "s59E25H2" 0 "30S ribosomal protein S16"
"DsuE09_00485" 4402 "s59E5H2" 0 "30S ribosomal protein S16"
"DsuE09_00485" 4402 "s63E6H2" 0 "30S ribosomal protein S16"
"DsuE09_00486" 4403 "s59E13H2" 0 "Signal recognition particle protein"
"DsuE09_00486" 4403 "s59E25H2" 0 "Signal recognition particle protein"
"DsuE09_00486" 4403 "s59E5H2" 0 "Signal recognition particle protein"
"DsuE09_00486" 4403 "s63E6H2" 0 "Signal recognition particle protein"
"DsuE09_00488" 4405 "s59E13H2" 0 "hypothetical protein"
"DsuE09_00488" 4405 "s59E25H2" 0 "hypothetical protein"
"DsuE09_00488" 4405 "s59E5H2" 0 "hypothetical protein"
"DsuE09_00488" 4405 "s63E6H2" 0 "hypothetical protein"

"DsuE09_00489" 4406 "s59E13H2" 0 "hypothetical protein"
"DsuE09_00489" 4406 "s59E25H2" 0 "hypothetical protein"
"DsuE09_00489" 4406 "s59E5H2" 0 "hypothetical protein"
"DsuE09_00489" 4406 "s63E6H2" 0 "hypothetical protein"
"DsuE09_00490" 4407 "s59E13H2" 0 "YcfA-like protein"
"DsuE09_00490" 4407 "s59E25H2" 0 "YcfA-like protein"
"DsuE09_00490" 4407 "s59E5H2" 0 "YcfA-like protein"
"DsuE09_00490" 4407 "s63E6H2" 0 "YcfA-like protein"
"DsuE09_00491" 4408 "s59E13H2" 0 "Phd_YefM"
"DsuE09_00491" 4408 "s59E25H2" 0 "Phd_YefM"
"DsuE09_00491" 4408 "s59E5H2" 0 "Phd_YefM"
"DsuE09_00491" 4408 "s63E6H2" 0 "Phd_YefM"
"DsuE09_00492" 4409 "s59E13H2" 0 "hypothetical protein"
"DsuE09_00492" 4409 "s59E25H2" 0 "hypothetical protein"
"DsuE09_00492" 4409 "s59E5H2" 0 "hypothetical protein"
"DsuE09_00492" 4409 "s63E6H2" 0 "hypothetical protein"
"DsuE09_00493" 4410 "s59E13H2" 0 "Nucleotidyltransferase domain protein"
"DsuE09_00493" 4410 "s59E25H2" 0 "Nucleotidyltransferase domain protein"
"DsuE09_00493" 4410 "s59E5H2" 0 "Nucleotidyltransferase domain protein"
"DsuE09_00493" 4410 "s63E6H2" 0 "Nucleotidyltransferase domain protein"
"DsuE09_00494" 4411 "s59E13H2" 0 "HEPN domain protein"
"DsuE09_00494" 4411 "s59E25H2" 0 "HEPN domain protein"
"DsuE09_00494" 4411 "s59E5H2" 0 "HEPN domain protein"
"DsuE09_00494" 4411 "s63E6H2" 0 "HEPN domain protein"
"DsuE09_00495" 4412 "s59E13H2" 0 "hypothetical protein"
"DsuE09_00495" 4412 "s59E25H2" 0 "hypothetical protein"
"DsuE09_00495" 4412 "s59E5H2" 0 "hypothetical protein"
"DsuE09_00495" 4412 "s63E6H2" 0 "hypothetical protein"
"DsuE09_00496" 4413 "s59E13H2" 0 "hypothetical protein"
"DsuE09_00496" 4413 "s59E25H2" 0 "hypothetical protein"
"DsuE09_00496" 4413 "s59E5H2" 0 "hypothetical protein"
"DsuE09_00496" 4413 "s63E6H2" 0 "hypothetical protein"
"DsuE09_00498" 4415 "s59E13H2" 0 "YcfA-like protein"
"DsuE09_00498" 4415 "s59E25H2" 0 "YcfA-like protein"
"DsuE09_00498" 4415 "s59E5H2" 0 "YcfA-like protein"
"DsuE09_00498" 4415 "s63E6H2" 0 "YcfA-like protein"
"DsuE09_00499" 4416 "s59E13H2" 0 "hypothetical protein"
"DsuE09_00499" 4416 "s59E25H2" 0 "hypothetical protein"
"DsuE09_00499" 4416 "s59E5H2" 0 "hypothetical protein"
"DsuE09_00499" 4416 "s63E6H2" 0 "hypothetical protein"
"DsuE09_00500" 4417 "s59E13H2" 0 "hypothetical protein"
"DsuE09_00500" 4417 "s59E25H2" 0 "hypothetical protein"
"DsuE09_00500" 4417 "s59E5H2" 0 "hypothetical protein"
"DsuE09_00500" 4417 "s63E6H2" 0 "hypothetical protein"
"DsuE09_00501" 4418 "s59E13H2" 0 "YcfA-like protein"
"DsuE09_00501" 4418 "s59E25H2" 0 "YcfA-like protein"
"DsuE09_00501" 4418 "s59E5H2" 0 "YcfA-like protein"

"DsuE09_00501" 4418 "s63E6H2" 0 "YcfA-like protein"
"DsuE09_00503" 4420 "s59E13H2" 0 "hypothetical protein"
"DsuE09_00503" 4420 "s59E25H2" 0 "hypothetical protein"
"DsuE09_00503" 4420 "s59E5H2" 0 "hypothetical protein"
"DsuE09_00503" 4420 "s63E6H2" 0 "hypothetical protein"
"DsuE09_00508" 4425 "s59E13H2" 0 "hypothetical protein"
"DsuE09_00508" 4425 "s59E25H2" 0 "hypothetical protein"
"DsuE09_00508" 4425 "s59E5H2" 0 "hypothetical protein"
"DsuE09_00508" 4425 "s63E6H2" 0 "hypothetical protein"
"DsuE09_00510" 4427 "s59E13H2" 0 "hypothetical protein"
"DsuE09_00510" 4427 "s59E25H2" 0 "hypothetical protein"
"DsuE09_00510" 4427 "s59E5H2" 0 "hypothetical protein"
"DsuE09_00510" 4427 "s63E6H2" 0 "hypothetical protein"
"DsuE09_00511" 4428 "s59E13H2" 0 "hypothetical protein"
"DsuE09_00511" 4428 "s59E25H2" 0 "hypothetical protein"
"DsuE09_00511" 4428 "s59E5H2" 0 "hypothetical protein"
"DsuE09_00511" 4428 "s63E6H2" 0 "hypothetical protein"
"DsuE09_00512" 4429 "s59E13H2" 0 "Helix-turn-helix domain protein"
"DsuE09_00512" 4429 "s59E25H2" 0 "Helix-turn-helix domain protein"
"DsuE09_00512" 4429 "s59E5H2" 0 "Helix-turn-helix domain protein"
"DsuE09_00512" 4429 "s63E6H2" 0 "Helix-turn-helix domain protein"
"DsuE09_00514" 4431 "s59E13H2" 0 "hypothetical protein"
"DsuE09_00514" 4431 "s59E25H2" 0 "hypothetical protein"
"DsuE09_00514" 4431 "s59E5H2" 0 "hypothetical protein"
"DsuE09_00514" 4431 "s63E6H2" 0 "hypothetical protein"
"DsuE09_00515" 4432 "s59E13H2" 0 "hypothetical protein"
"DsuE09_00515" 4432 "s59E25H2" 0 "hypothetical protein"
"DsuE09_00515" 4432 "s59E5H2" 0 "hypothetical protein"
"DsuE09_00515" 4432 "s63E6H2" 0 "hypothetical protein"
"DsuE09_00517" 4434 "s59E13H2" 0 "hypothetical protein"
"DsuE09_00517" 4434 "s59E25H2" 0 "hypothetical protein"
"DsuE09_00517" 4434 "s59E5H2" 0 "hypothetical protein"
"DsuE09_00517" 4434 "s63E6H2" 0 "hypothetical protein"
"DsuE09_00518" 4435 "s59E13H2" 0 "putative HTH-type transcriptional regulator YbaQ"
"DsuE09_00518" 4435 "s59E25H2" 0 "putative HTH-type transcriptional regulator YbaQ"
"DsuE09_00518" 4435 "s59E5H2" 0 "putative HTH-type transcriptional regulator YbaQ"
"DsuE09_00518" 4435 "s63E6H2" 0 "putative HTH-type transcriptional regulator YbaQ"
"DsuE09_00519" 4436 "s59E13H2" 0 "Toxin HigB-1"
"DsuE09_00519" 4436 "s59E25H2" 0 "Toxin HigB-1"
"DsuE09_00519" 4436 "s59E5H2" 0 "Toxin HigB-1"
"DsuE09_00519" 4436 "s63E6H2" 0 "Toxin HigB-1"
"DsuE09_00520" 4437 "s59E13H2" 0 "hypothetical protein"
"DsuE09_00520" 4437 "s59E25H2" 0 "hypothetical protein"
"DsuE09_00520" 4437 "s59E5H2" 0 "hypothetical protein"
"DsuE09_00520" 4437 "s63E6H2" 0 "hypothetical protein"
"DsuE09_00522" 4439 "s59E13H2" 0 "UDP-glucose:undecaprenyl-phosphate glucose-1-phosphate transferase"
"DsuE09_00522" 4439 "s59E25H2" 0 "UDP-glucose:undecaprenyl-phosphate glucose-1-phosphate transferase"

"DsuE09_00522" 4439 "s59E5H2" 0 "UDP-glucose:undecaprenyl-phosphate glucose-1-phosphate transferase"
"DsuE09_00522" 4439 "s63E6H2" 0 "UDP-glucose:undecaprenyl-phosphate glucose-1-phosphate transferase"
"DsuE09_00525" 4442 "s59E13H2" 0 "hypothetical protein"
"DsuE09_00525" 4442 "s59E25H2" 0 "hypothetical protein"
"DsuE09_00525" 4442 "s59E5H2" 0 "hypothetical protein"
"DsuE09_00525" 4442 "s63E6H2" 0 "hypothetical protein"
"DsuE09_00526" 4443 "s59E13H2" 0 "hypothetical protein"
"DsuE09_00526" 4443 "s59E25H2" 0 "hypothetical protein"
"DsuE09_00526" 4443 "s59E5H2" 0 "hypothetical protein"
"DsuE09_00526" 4443 "s63E6H2" 0 "hypothetical protein"
"DsuE09_00527" 4444 "s59E13H2" 0 "tRNA(fMet)-specific endonuclease VapC"
"DsuE09_00527" 4444 "s59E25H2" 0 "tRNA(fMet)-specific endonuclease VapC"
"DsuE09_00527" 4444 "s59E5H2" 0 "tRNA(fMet)-specific endonuclease VapC"
"DsuE09_00527" 4444 "s63E6H2" 0 "tRNA(fMet)-specific endonuclease VapC"
"DsuE09_00528" 4445 "s59E13H2" 0 "Putative antitoxin VapB21"
"DsuE09_00528" 4445 "s59E25H2" 0 "Putative antitoxin VapB21"
"DsuE09_00528" 4445 "s59E5H2" 0 "Putative antitoxin VapB21"
"DsuE09_00528" 4445 "s63E6H2" 0 "Putative antitoxin VapB21"
"DsuE09_00531" 4448 "s59E13H2" 0 "B3/4 domain protein"
"DsuE09_00531" 4448 "s59E25H2" 0 "B3/4 domain protein"
"DsuE09_00531" 4448 "s59E5H2" 0 "B3/4 domain protein"
"DsuE09_00531" 4448 "s63E6H2" 0 "B3/4 domain protein"
"DsuE09_00532" 4449 "s59E13H2" 0 "pyruvate formate lyase-activating enzyme 1"
"DsuE09_00532" 4449 "s59E25H2" 0 "pyruvate formate lyase-activating enzyme 1"
"DsuE09_00532" 4449 "s59E5H2" 0 "pyruvate formate lyase-activating enzyme 1"
"DsuE09_00532" 4449 "s63E6H2" 0 "pyruvate formate lyase-activating enzyme 1"
"DsuE09_00534" 4451 "s59E13H2" 0 "DNA mismatch repair protein"
"DsuE09_00534" 4451 "s59E25H2" 0 "DNA mismatch repair protein"
"DsuE09_00534" 4451 "s59E5H2" 0 "DNA mismatch repair protein"
"DsuE09_00534" 4451 "s63E6H2" 0 "DNA mismatch repair protein"
"DsuE09_00535" 4452 "s59E13H2" 0 "hypothetical protein"
"DsuE09_00535" 4452 "s59E25H2" 0 "hypothetical protein"
"DsuE09_00535" 4452 "s59E5H2" 0 "hypothetical protein"
"DsuE09_00535" 4452 "s63E6H2" 0 "hypothetical protein"
"DsuE09_00536" 4453 "s59E13H2" 0 "hypothetical protein"
"DsuE09_00536" 4453 "s59E25H2" 0 "hypothetical protein"
"DsuE09_00536" 4453 "s59E5H2" 0 "hypothetical protein"
"DsuE09_00536" 4453 "s63E6H2" 0 "hypothetical protein"
"DsuE09_00537" 4454 "s59E13H2" 0 "site-specific tyrosine recombinase XerC"
"DsuE09_00537" 4454 "s59E25H2" 0 "site-specific tyrosine recombinase XerC"
"DsuE09_00537" 4454 "s59E5H2" 0 "site-specific tyrosine recombinase XerC"
"DsuE09_00537" 4454 "s63E6H2" 0 "site-specific tyrosine recombinase XerC"
"DsuE09_00539" 4456 "s59E13H2" 0 "PilZ domain protein"
"DsuE09_00539" 4456 "s59E25H2" 0 "PilZ domain protein"
"DsuE09_00539" 4456 "s59E5H2" 0 "PilZ domain protein"
"DsuE09_00539" 4456 "s63E6H2" 0 "PilZ domain protein"
"DsuE09_00540" 4457 "s59E13H2" 0 "hypothetical protein"

"DsuE09_00540" 4457 "s59E25H2" 0 "hypothetical protein"
"DsuE09_00540" 4457 "s59E5H2" 0 "hypothetical protein"
"DsuE09_00540" 4457 "s63E6H2" 0 "hypothetical protein"
"DsuE09_00541" 4458 "s59E13H2" 0 "Mutual gliding-motility protein MglA"
"DsuE09_00541" 4458 "s59E25H2" 0 "Mutual gliding-motility protein MglA"
"DsuE09_00541" 4458 "s59E5H2" 0 "Mutual gliding-motility protein MglA"
"DsuE09_00541" 4458 "s63E6H2" 0 "Mutual gliding-motility protein MglA"
"DsuE09_00542" 4459 "s59E13H2" 0 "DNA-binding transcriptional dual regulator Crp"
"DsuE09_00542" 4459 "s59E25H2" 0 "DNA-binding transcriptional dual regulator Crp"
"DsuE09_00542" 4459 "s59E5H2" 0 "DNA-binding transcriptional dual regulator Crp"
"DsuE09_00542" 4459 "s63E6H2" 0 "DNA-binding transcriptional dual regulator Crp"
"DsuE09_00543" 4460 "s59E13H2" 0 "hypothetical protein"
"DsuE09_00543" 4460 "s59E25H2" 0 "hypothetical protein"
"DsuE09_00543" 4460 "s59E5H2" 0 "hypothetical protein"
"DsuE09_00543" 4460 "s63E6H2" 0 "hypothetical protein"
"DsuE09_00544" 4461 "s59E13H2" 0 "hypothetical protein"
"DsuE09_00544" 4461 "s59E25H2" 0 "hypothetical protein"
"DsuE09_00544" 4461 "s59E5H2" 0 "hypothetical protein"
"DsuE09_00544" 4461 "s63E6H2" 0 "hypothetical protein"
"DsuE09_00545" 4462 "s59E13H2" 0 "Vacuole effluxer Atg22 like protein"
"DsuE09_00545" 4462 "s59E25H2" 0 "Vacuole effluxer Atg22 like protein"
"DsuE09_00545" 4462 "s59E5H2" 0 "Vacuole effluxer Atg22 like protein"
"DsuE09_00545" 4462 "s63E6H2" 0 "Vacuole effluxer Atg22 like protein"
"DsuE09_00547" 4464 "s59E13H2" 0 "Hemolysin C"
"DsuE09_00547" 4464 "s59E25H2" 0 "Hemolysin C"
"DsuE09_00547" 4464 "s59E5H2" 0 "Hemolysin C"
"DsuE09_00547" 4464 "s63E6H2" 0 "Hemolysin C"
"DsuE09_00548" 4465 "s59E13H2" 0 "Magnesium and cobalt efflux protein CorC"
"DsuE09_00548" 4465 "s59E25H2" 0 "Magnesium and cobalt efflux protein CorC"
"DsuE09_00548" 4465 "s59E5H2" 0 "Magnesium and cobalt efflux protein CorC"
"DsuE09_00548" 4465 "s63E6H2" 0 "Magnesium and cobalt efflux protein CorC"
"DsuE09_00549" 4466 "s59E13H2" 0 "hypothetical protein"
"DsuE09_00549" 4466 "s59E25H2" 0 "hypothetical protein"
"DsuE09_00549" 4466 "s59E5H2" 0 "hypothetical protein"
"DsuE09_00549" 4466 "s63E6H2" 0 "hypothetical protein"
"DsuE09_00550" 4467 "s59E13H2" 0 "Adenylate cyclase 1"
"DsuE09_00550" 4467 "s59E25H2" 0 "Adenylate cyclase 1"
"DsuE09_00550" 4467 "s59E5H2" 0 "Adenylate cyclase 1"
"DsuE09_00550" 4467 "s63E6H2" 0 "Adenylate cyclase 1"
"DsuE09_00551" 4468 "s59E13H2" 0 "hypothetical protein"
"DsuE09_00551" 4468 "s59E25H2" 0 "hypothetical protein"
"DsuE09_00551" 4468 "s59E5H2" 0 "hypothetical protein"
"DsuE09_00551" 4468 "s63E6H2" 0 "hypothetical protein"
"DsuE09_00552" 4469 "s59E13H2" 0 "putative hydrogenase nickel incorporation protein HypA"
"DsuE09_00552" 4469 "s59E25H2" 0 "putative hydrogenase nickel incorporation protein HypA"
"DsuE09_00552" 4469 "s59E5H2" 0 "putative hydrogenase nickel incorporation protein HypA"
"DsuE09_00552" 4469 "s63E6H2" 0 "putative hydrogenase nickel incorporation protein HypA"

"DsuE09_00553" 4470 "s59E13H2" 0 "hypothetical protein"
"DsuE09_00553" 4470 "s59E25H2" 0 "hypothetical protein"
"DsuE09_00553" 4470 "s59E5H2" 0 "hypothetical protein"
"DsuE09_00553" 4470 "s63E6H2" 0 "hypothetical protein"
"DsuE09_00554" 4471 "s59E13H2" 0 "hypothetical protein"
"DsuE09_00554" 4471 "s59E25H2" 0 "hypothetical protein"
"DsuE09_00554" 4471 "s59E5H2" 0 "hypothetical protein"
"DsuE09_00554" 4471 "s63E6H2" 0 "hypothetical protein"
"DsuE09_00555" 4472 "s59E13H2" 0 "hypothetical protein"
"DsuE09_00555" 4472 "s59E25H2" 0 "hypothetical protein"
"DsuE09_00555" 4472 "s59E5H2" 0 "hypothetical protein"
"DsuE09_00555" 4472 "s63E6H2" 0 "hypothetical protein"
"DsuE09_00556" 4473 "s59E13H2" 0 "Acyl-CoA dehydrogenase"
"DsuE09_00556" 4473 "s59E25H2" 0 "Acyl-CoA dehydrogenase"
"DsuE09_00556" 4473 "s59E5H2" 0 "Acyl-CoA dehydrogenase"
"DsuE09_00556" 4473 "s63E6H2" 0 "Acyl-CoA dehydrogenase"
"DsuE09_00557" 4474 "s59E13H2" 0 "hypothetical protein"
"DsuE09_00557" 4474 "s59E25H2" 0 "hypothetical protein"
"DsuE09_00557" 4474 "s59E5H2" 0 "hypothetical protein"
"DsuE09_00557" 4474 "s63E6H2" 0 "hypothetical protein"
"DsuE09_00559" 4476 "s59E13H2" 0 "Fructose-1,6-bisphosphatase"
"DsuE09_00559" 4476 "s59E25H2" 0 "Fructose-1,6-bisphosphatase"
"DsuE09_00559" 4476 "s59E5H2" 0 "Fructose-1,6-bisphosphatase"
"DsuE09_00559" 4476 "s63E6H2" 0 "Fructose-1,6-bisphosphatase"
"DsuE09_00561" 4478 "s59E13H2" 0 "Methylmalonyl-CoA mutase large subunit"
"DsuE09_00561" 4478 "s59E25H2" 0 "Methylmalonyl-CoA mutase large subunit"
"DsuE09_00561" 4478 "s59E5H2" 0 "Methylmalonyl-CoA mutase large subunit"
"DsuE09_00561" 4478 "s63E6H2" 0 "Methylmalonyl-CoA mutase large subunit"
"DsuE09_00564" 4481 "s59E13H2" 0 "putative lipoprotein YiaD precursor"
"DsuE09_00564" 4481 "s59E25H2" 0 "putative lipoprotein YiaD precursor"
"DsuE09_00564" 4481 "s59E5H2" 0 "putative lipoprotein YiaD precursor"
"DsuE09_00564" 4481 "s63E6H2" 0 "putative lipoprotein YiaD precursor"
"DsuE09_00565" 4482 "s59E13H2" 0 "Leucine-, isoleucine-, valine-, threonine-, and alanine-binding protein precursor"
"DsuE09_00565" 4482 "s59E25H2" 0 "Leucine-, isoleucine-, valine-, threonine-, and alanine-binding protein precursor"
"DsuE09_00565" 4482 "s59E5H2" 0 "Leucine-, isoleucine-, valine-, threonine-, and alanine-binding protein precursor"
"DsuE09_00565" 4482 "s63E6H2" 0 "Leucine-, isoleucine-, valine-, threonine-, and alanine-binding protein precursor"
"DsuE09_00566" 4483 "s59E13H2" 0 "leucine/isoleucine/valine transporter permease subunit"
"DsuE09_00566" 4483 "s59E25H2" 0 "leucine/isoleucine/valine transporter permease subunit"
"DsuE09_00566" 4483 "s59E5H2" 0 "leucine/isoleucine/valine transporter permease subunit"
"DsuE09_00566" 4483 "s63E6H2" 0 "leucine/isoleucine/valine transporter permease subunit"
"DsuE09_00567" 4484 "s59E13H2" 0 "High-affinity branched-chain amino acid transport system permease protein LivH"
"DsuE09_00567" 4484 "s59E25H2" 0 "High-affinity branched-chain amino acid transport system permease protein LivH"
"DsuE09_00567" 4484 "s59E5H2" 0 "High-affinity branched-chain amino acid transport system permease protein LivH"
"DsuE09_00567" 4484 "s63E6H2" 0 "High-affinity branched-chain amino acid transport system permease protein LivH"
"DsuE09_00568" 4485 "s59E13H2" 0 "High-affinity branched-chain amino acid transport ATP-binding protein LivF"
"DsuE09_00568" 4485 "s59E25H2" 0 "High-affinity branched-chain amino acid transport ATP-binding protein LivF"
"DsuE09_00568" 4485 "s59E5H2" 0 "High-affinity branched-chain amino acid transport ATP-binding protein LivF"

"DsuE09_00568" 4485 "s63E6H2" 0 "High-affinity branched-chain amino acid transport ATP-binding protein LivF"
"DsuE09_00569" 4486 "s59E13H2" 0 "putative branched-chain amino acid transport ATP-binding protein LivG"
"DsuE09_00569" 4486 "s59E25H2" 0 "putative branched-chain amino acid transport ATP-binding protein LivG"
"DsuE09_00569" 4486 "s59E5H2" 0 "putative branched-chain amino acid transport ATP-binding protein LivG"
"DsuE09_00569" 4486 "s63E6H2" 0 "putative branched-chain amino acid transport ATP-binding protein LivG"
"DsuE09_00570" 4487 "s59E13H2" 0 "DNA integrity scanning protein DisA"
"DsuE09_00570" 4487 "s59E25H2" 0 "DNA integrity scanning protein DisA"
"DsuE09_00570" 4487 "s59E5H2" 0 "DNA integrity scanning protein DisA"
"DsuE09_00570" 4487 "s63E6H2" 0 "DNA integrity scanning protein DisA"
"DsuE09_00571" 4488 "s59E13H2" 0 "Phosphoglycerate transporter protein"
"DsuE09_00571" 4488 "s59E25H2" 0 "Phosphoglycerate transporter protein"
"DsuE09_00571" 4488 "s59E5H2" 0 "Phosphoglycerate transporter protein"
"DsuE09_00571" 4488 "s63E6H2" 0 "Phosphoglycerate transporter protein"
"DsuE09_00572" 4489 "s59E13H2" 0 "Altronate dehydratase"
"DsuE09_00572" 4489 "s59E25H2" 0 "Altronate dehydratase"
"DsuE09_00572" 4489 "s59E5H2" 0 "Altronate dehydratase"
"DsuE09_00572" 4489 "s63E6H2" 0 "Altronate dehydratase"
"DsuE09_00573" 4490 "s59E13H2" 0 "Butyrate--acetoacetate CoA-transferase subunit B"
"DsuE09_00573" 4490 "s59E25H2" 0 "Butyrate--acetoacetate CoA-transferase subunit B"
"DsuE09_00573" 4490 "s59E5H2" 0 "Butyrate--acetoacetate CoA-transferase subunit B"
"DsuE09_00573" 4490 "s63E6H2" 0 "Butyrate--acetoacetate CoA-transferase subunit B"
"DsuE09_00574" 4491 "s59E13H2" 0 "3-oxoadipate CoA-transferase subunit A"
"DsuE09_00574" 4491 "s59E25H2" 0 "3-oxoadipate CoA-transferase subunit A"
"DsuE09_00574" 4491 "s59E5H2" 0 "3-oxoadipate CoA-transferase subunit A"
"DsuE09_00574" 4491 "s63E6H2" 0 "3-oxoadipate CoA-transferase subunit A"
"DsuE09_00575" 4492 "s59E13H2" 0 "Glucose 1-dehydrogenase 1"
"DsuE09_00575" 4492 "s59E25H2" 0 "Glucose 1-dehydrogenase 1"
"DsuE09_00575" 4492 "s59E5H2" 0 "Glucose 1-dehydrogenase 1"
"DsuE09_00575" 4492 "s63E6H2" 0 "Glucose 1-dehydrogenase 1"
"DsuE09_00576" 4493 "s59E13H2" 0 "hypothetical protein"
"DsuE09_00576" 4493 "s59E25H2" 0 "hypothetical protein"
"DsuE09_00576" 4493 "s59E5H2" 0 "hypothetical protein"
"DsuE09_00576" 4493 "s63E6H2" 0 "hypothetical protein"
"DsuE09_00577" 4494 "s59E13H2" 0 "Glycosyl transferases group 1"
"DsuE09_00577" 4494 "s59E25H2" 0 "Glycosyl transferases group 1"
"DsuE09_00577" 4494 "s59E5H2" 0 "Glycosyl transferases group 1"
"DsuE09_00577" 4494 "s63E6H2" 0 "Glycosyl transferases group 1"
"DsuE09_00578" 4495 "s59E13H2" 0 "hypothetical protein"
"DsuE09_00578" 4495 "s59E25H2" 0 "hypothetical protein"
"DsuE09_00578" 4495 "s59E5H2" 0 "hypothetical protein"
"DsuE09_00578" 4495 "s63E6H2" 0 "hypothetical protein"
"DsuE09_00579" 4496 "s59E13H2" 0 "hypothetical protein"
"DsuE09_00579" 4496 "s59E25H2" 0 "hypothetical protein"
"DsuE09_00579" 4496 "s59E5H2" 0 "hypothetical protein"
"DsuE09_00579" 4496 "s63E6H2" 0 "hypothetical protein"
"DsuE09_00580" 4497 "s59E13H2" 0 "hypothetical protein"
"DsuE09_00580" 4497 "s59E25H2" 0 "hypothetical protein"

"DsuE09_00580" 4497 "s59E5H2" 0 "hypothetical protein"
"DsuE09_00580" 4497 "s63E6H2" 0 "hypothetical protein"
"DsuE09_00581" 4498 "s59E13H2" 0 "hypothetical protein"
"DsuE09_00581" 4498 "s59E25H2" 0 "hypothetical protein"
"DsuE09_00581" 4498 "s59E5H2" 0 "hypothetical protein"
"DsuE09_00581" 4498 "s63E6H2" 0 "hypothetical protein"
"DsuE09_00583" 4500 "s59E13H2" 0 "PemK-like protein"
"DsuE09_00583" 4500 "s59E25H2" 0 "PemK-like protein"
"DsuE09_00583" 4500 "s59E5H2" 0 "PemK-like protein"
"DsuE09_00583" 4500 "s63E6H2" 0 "PemK-like protein"
"DsuE09_00584" 4501 "s59E13H2" 0 "hypothetical protein"
"DsuE09_00584" 4501 "s59E25H2" 0 "hypothetical protein"
"DsuE09_00584" 4501 "s59E5H2" 0 "hypothetical protein"
"DsuE09_00584" 4501 "s63E6H2" 0 "hypothetical protein"
"DsuE09_00586" 4503 "s59E13H2" 0 "hypothetical protein"
"DsuE09_00586" 4503 "s59E25H2" 0 "hypothetical protein"
"DsuE09_00586" 4503 "s59E5H2" 0 "hypothetical protein"
"DsuE09_00586" 4503 "s63E6H2" 0 "hypothetical protein"
"DsuE09_00588" 4505 "s59E13H2" 0 "PemK-like protein"
"DsuE09_00588" 4505 "s59E25H2" 0 "PemK-like protein"
"DsuE09_00588" 4505 "s59E5H2" 0 "PemK-like protein"
"DsuE09_00588" 4505 "s63E6H2" 0 "PemK-like protein"
"DsuE09_00591" 4508 "s59E13H2" 0 "Ribonuclease VapC20"
"DsuE09_00591" 4508 "s59E25H2" 0 "Ribonuclease VapC20"
"DsuE09_00591" 4508 "s59E5H2" 0 "Ribonuclease VapC20"
"DsuE09_00591" 4508 "s63E6H2" 0 "Ribonuclease VapC20"
"DsuE09_00592" 4509 "s59E13H2" 0 "hypothetical protein"
"DsuE09_00592" 4509 "s59E25H2" 0 "hypothetical protein"
"DsuE09_00592" 4509 "s59E5H2" 0 "hypothetical protein"
"DsuE09_00592" 4509 "s63E6H2" 0 "hypothetical protein"
"DsuE09_00593" 4510 "s59E13H2" 0 "hypothetical protein"
"DsuE09_00593" 4510 "s59E25H2" 0 "hypothetical protein"
"DsuE09_00593" 4510 "s59E5H2" 0 "hypothetical protein"
"DsuE09_00593" 4510 "s63E6H2" 0 "hypothetical protein"
"DsuE09_00594" 4511 "s59E13H2" 0 "hypothetical protein"
"DsuE09_00594" 4511 "s59E25H2" 0 "hypothetical protein"
"DsuE09_00594" 4511 "s59E5H2" 0 "hypothetical protein"
"DsuE09_00594" 4511 "s63E6H2" 0 "hypothetical protein"
"DsuE09_00595" 4512 "s59E13H2" 0 "hypothetical protein"
"DsuE09_00595" 4512 "s59E25H2" 0 "hypothetical protein"
"DsuE09_00595" 4512 "s59E5H2" 0 "hypothetical protein"
"DsuE09_00595" 4512 "s63E6H2" 0 "hypothetical protein"
"DsuE09_00596" 4513 "s59E13H2" 0 "ATP-dependent RecD-like DNA helicase"
"DsuE09_00596" 4513 "s59E25H2" 0 "ATP-dependent RecD-like DNA helicase"
"DsuE09_00596" 4513 "s59E5H2" 0 "ATP-dependent RecD-like DNA helicase"
"DsuE09_00596" 4513 "s63E6H2" 0 "ATP-dependent RecD-like DNA helicase"
"DsuE09_00597" 4514 "s59E13H2" 0 "Arginine--tRNA ligase"

"DsuE09_00597" 4514 "s59E25H2" 0 "Arginine--tRNA ligase"
"DsuE09_00597" 4514 "s59E5H2" 0 "Arginine--tRNA ligase"
"DsuE09_00597" 4514 "s63E6H2" 0 "Arginine--tRNA ligase"
"DsuE09_00598" 4515 "s59E13H2" 0 "Sporulation related domain protein"
"DsuE09_00598" 4515 "s59E25H2" 0 "Sporulation related domain protein"
"DsuE09_00598" 4515 "s59E5H2" 0 "Sporulation related domain protein"
"DsuE09_00598" 4515 "s63E6H2" 0 "Sporulation related domain protein"
"DsuE09_00599" 4516 "s59E13H2" 0 "Amino-acid acetyltransferase"
"DsuE09_00599" 4516 "s59E25H2" 0 "Amino-acid acetyltransferase"
"DsuE09_00599" 4516 "s59E5H2" 0 "Amino-acid acetyltransferase"
"DsuE09_00599" 4516 "s63E6H2" 0 "Amino-acid acetyltransferase"
"DsuE09_00600" 4517 "s59E13H2" 0 "hypothetical protein"
"DsuE09_00600" 4517 "s59E25H2" 0 "hypothetical protein"
"DsuE09_00600" 4517 "s59E5H2" 0 "hypothetical protein"
"DsuE09_00600" 4517 "s63E6H2" 0 "hypothetical protein"
"DsuE09_00601" 4518 "s59E13H2" 0 "Sec-independent protein translocase protein TatAd"
"DsuE09_00601" 4518 "s59E25H2" 0 "Sec-independent protein translocase protein TatAd"
"DsuE09_00601" 4518 "s59E5H2" 0 "Sec-independent protein translocase protein TatAd"
"DsuE09_00601" 4518 "s63E6H2" 0 "Sec-independent protein translocase protein TatAd"
"DsuE09_00602" 4519 "s59E13H2" 0 "Sec-independent protein translocase protein TatC"
"DsuE09_00602" 4519 "s59E25H2" 0 "Sec-independent protein translocase protein TatC"
"DsuE09_00602" 4519 "s59E5H2" 0 "Sec-independent protein translocase protein TatC"
"DsuE09_00602" 4519 "s63E6H2" 0 "Sec-independent protein translocase protein TatC"
"DsuE09_00603" 4520 "s59E13H2" 0 "Penicillin-binding protein activator LpoA"
"DsuE09_00603" 4520 "s59E25H2" 0 "Penicillin-binding protein activator LpoA"
"DsuE09_00603" 4520 "s59E5H2" 0 "Penicillin-binding protein activator LpoA"
"DsuE09_00603" 4520 "s63E6H2" 0 "Penicillin-binding protein activator LpoA"
"DsuE09_00604" 4521 "s59E13H2" 0 "Chaperone protein DnaK"
"DsuE09_00604" 4521 "s59E25H2" 0 "Chaperone protein DnaK"
"DsuE09_00604" 4521 "s59E5H2" 0 "Chaperone protein DnaK"
"DsuE09_00604" 4521 "s63E6H2" 0 "Chaperone protein DnaK"
"DsuE09_00605" 4522 "s59E13H2" 0 "heat shock protein GrpE"
"DsuE09_00605" 4522 "s59E25H2" 0 "heat shock protein GrpE"
"DsuE09_00605" 4522 "s59E5H2" 0 "heat shock protein GrpE"
"DsuE09_00605" 4522 "s63E6H2" 0 "heat shock protein GrpE"
"DsuE09_00606" 4523 "s59E13H2" 0 "Phosphoribosylaminoimidazole-succinocarboxamide synthase"
"DsuE09_00606" 4523 "s59E25H2" 0 "Phosphoribosylaminoimidazole-succinocarboxamide synthase"
"DsuE09_00606" 4523 "s59E5H2" 0 "Phosphoribosylaminoimidazole-succinocarboxamide synthase"
"DsuE09_00606" 4523 "s63E6H2" 0 "Phosphoribosylaminoimidazole-succinocarboxamide synthase"
"DsuE09_00607" 4524 "s59E13H2" 0 "Phosphoribosyl-AMP cyclohydrolase"
"DsuE09_00607" 4524 "s59E25H2" 0 "Phosphoribosyl-AMP cyclohydrolase"
"DsuE09_00607" 4524 "s59E5H2" 0 "Phosphoribosyl-AMP cyclohydrolase"
"DsuE09_00607" 4524 "s63E6H2" 0 "Phosphoribosyl-AMP cyclohydrolase"
"DsuE09_00608" 4525 "s59E13H2" 0 "ATP phosphoribosyltransferase"
"DsuE09_00608" 4525 "s59E25H2" 0 "ATP phosphoribosyltransferase"
"DsuE09_00608" 4525 "s59E5H2" 0 "ATP phosphoribosyltransferase"
"DsuE09_00608" 4525 "s63E6H2" 0 "ATP phosphoribosyltransferase"

"DsuE09_00609" 4526 "s59E13H2" 0.007986051 "putative aspartate aminotransferase 2"
"DsuE09_00609" 4526 "s59E25H2" 0 "putative aspartate aminotransferase 2"
"DsuE09_00609" 4526 "s59E5H2" 0 "putative aspartate aminotransferase 2"
"DsuE09_00609" 4526 "s63E6H2" 0 "putative aspartate aminotransferase 2"
"DsuE09_00610" 4527 "s59E13H2" 0.1788302 "Transcriptional regulatory protein ZraR"
"DsuE09_00610" 4527 "s59E25H2" 0 "Transcriptional regulatory protein ZraR"
"DsuE09_00610" 4527 "s59E5H2" 0 "Transcriptional regulatory protein ZraR"
"DsuE09_00610" 4527 "s63E6H2" 0 "Transcriptional regulatory protein ZraR"
"DsuE09_00611" 4528 "s59E13H2" 0 "Lipid-A-disaccharide synthase"
"DsuE09_00611" 4528 "s59E25H2" 0 "Lipid-A-disaccharide synthase"
"DsuE09_00611" 4528 "s59E5H2" 0 "Lipid-A-disaccharide synthase"
"DsuE09_00611" 4528 "s63E6H2" 0 "Lipid-A-disaccharide synthase"
"DsuE09_00612" 4529 "s59E13H2" 0 "Putative oxidoreductase YceM"
"DsuE09_00612" 4529 "s59E25H2" 0 "Putative oxidoreductase YceM"
"DsuE09_00612" 4529 "s59E5H2" 0 "Putative oxidoreductase YceM"
"DsuE09_00612" 4529 "s63E6H2" 0 "Putative oxidoreductase YceM"
"DsuE09_00613" 4530 "s59E13H2" 0 "hypothetical protein"
"DsuE09_00613" 4530 "s59E25H2" 0 "hypothetical protein"
"DsuE09_00613" 4530 "s59E5H2" 0 "hypothetical protein"
"DsuE09_00613" 4530 "s63E6H2" 0 "hypothetical protein"
"DsuE09_00614" 4531 "s59E13H2" 0 "Acyl-[acyl-carrier-protein]--UDP-N-acetylglucosamine O-acyltransferase"
"DsuE09_00614" 4531 "s59E25H2" 0 "Acyl-[acyl-carrier-protein]--UDP-N-acetylglucosamine O-acyltransferase"
"DsuE09_00614" 4531 "s59E5H2" 0 "Acyl-[acyl-carrier-protein]--UDP-N-acetylglucosamine O-acyltransferase"
"DsuE09_00614" 4531 "s63E6H2" 0 "Acyl-[acyl-carrier-protein]--UDP-N-acetylglucosamine O-acyltransferase"
"DsuE09_00615" 4532 "s59E13H2" 0 "3-hydroxyacyl-[acyl-carrier-protein] dehydratase FabZ"
"DsuE09_00615" 4532 "s59E25H2" 0 "3-hydroxyacyl-[acyl-carrier-protein] dehydratase FabZ"
"DsuE09_00615" 4532 "s59E5H2" 0 "3-hydroxyacyl-[acyl-carrier-protein] dehydratase FabZ"
"DsuE09_00615" 4532 "s63E6H2" 0 "3-hydroxyacyl-[acyl-carrier-protein] dehydratase FabZ"
"DsuE09_00616" 4533 "s59E13H2" 0 "UDP-3-O-(3-hydroxymyristoyl)glucosamine N-acyltransferase"
"DsuE09_00616" 4533 "s59E25H2" 0 "UDP-3-O-(3-hydroxymyristoyl)glucosamine N-acyltransferase"
"DsuE09_00616" 4533 "s59E5H2" 0 "UDP-3-O-(3-hydroxymyristoyl)glucosamine N-acyltransferase"
"DsuE09_00616" 4533 "s63E6H2" 0 "UDP-3-O-(3-hydroxymyristoyl)glucosamine N-acyltransferase"
"DsuE09_00617" 4534 "s59E13H2" 0 "Outer membrane protein (OmpH-like)"
"DsuE09_00617" 4534 "s59E25H2" 0 "Outer membrane protein (OmpH-like)"
"DsuE09_00617" 4534 "s59E5H2" 0 "Outer membrane protein (OmpH-like)"
"DsuE09_00617" 4534 "s63E6H2" 0 "Outer membrane protein (OmpH-like)"
"DsuE09_00618" 4535 "s59E13H2" 0 "Outer membrane protein assembly factor BamA precursor"
"DsuE09_00618" 4535 "s59E25H2" 0 "Outer membrane protein assembly factor BamA precursor"
"DsuE09_00618" 4535 "s59E5H2" 0 "Outer membrane protein assembly factor BamA precursor"
"DsuE09_00618" 4535 "s63E6H2" 0 "Outer membrane protein assembly factor BamA precursor"
"DsuE09_00619" 4536 "s59E13H2" 0 "Lipoprotein-releasing system ATP-binding protein LolD"
"DsuE09_00619" 4536 "s59E25H2" 0 "Lipoprotein-releasing system ATP-binding protein LolD"
"DsuE09_00619" 4536 "s59E5H2" 0 "Lipoprotein-releasing system ATP-binding protein LolD"
"DsuE09_00619" 4536 "s63E6H2" 0 "Lipoprotein-releasing system ATP-binding protein LolD"
"DsuE09_00620" 4537 "s59E13H2" 0 "Lipoprotein-releasing system transmembrane protein LolC"
"DsuE09_00620" 4537 "s59E25H2" 0 "Lipoprotein-releasing system transmembrane protein LolC"
"DsuE09_00620" 4537 "s59E5H2" 0 "Lipoprotein-releasing system transmembrane protein LolC"

"DsuE09_00620" 4537 "s63E6H2" 0 "Lipoprotein-releasing system transmembrane protein LolC"
"DsuE09_00621" 4538 "s59E13H2" 0 "Lysine--tRNA ligase"
"DsuE09_00621" 4538 "s59E25H2" 0 "Lysine--tRNA ligase"
"DsuE09_00621" 4538 "s59E5H2" 0 "Lysine--tRNA ligase"
"DsuE09_00621" 4538 "s63E6H2" 0 "Lysine--tRNA ligase"
"DsuE09_00622" 4539 "s59E13H2" 0 "Acetyl-coenzyme A synthetase"
"DsuE09_00622" 4539 "s59E25H2" 0 "Acetyl-coenzyme A synthetase"
"DsuE09_00622" 4539 "s59E5H2" 0 "Acetyl-coenzyme A synthetase"
"DsuE09_00622" 4539 "s63E6H2" 0 "Acetyl-coenzyme A synthetase"
"DsuE09_00623" 4540 "s59E13H2" 0 "F(420)H(2) dehydrogenase subunit M"
"DsuE09_00623" 4540 "s59E25H2" 0 "F(420)H(2) dehydrogenase subunit M"
"DsuE09_00623" 4540 "s59E5H2" 0 "F(420)H(2) dehydrogenase subunit M"
"DsuE09_00623" 4540 "s63E6H2" 0 "F(420)H(2) dehydrogenase subunit M"
"DsuE09_00624" 4541 "s59E13H2" 0 "NADH-quinone oxidoreductase subunit L"
"DsuE09_00624" 4541 "s59E25H2" 0 "NADH-quinone oxidoreductase subunit L"
"DsuE09_00624" 4541 "s59E5H2" 0 "NADH-quinone oxidoreductase subunit L"
"DsuE09_00624" 4541 "s63E6H2" 0 "NADH-quinone oxidoreductase subunit L"
"DsuE09_00625" 4542 "s59E13H2" 0 "hypothetical protein"
"DsuE09_00625" 4542 "s59E25H2" 0 "hypothetical protein"
"DsuE09_00625" 4542 "s59E5H2" 0 "hypothetical protein"
"DsuE09_00625" 4542 "s63E6H2" 0 "hypothetical protein"
"DsuE09_00626" 4543 "s59E13H2" 0 "Na(+)/H(+) antiporter subunit D"
"DsuE09_00626" 4543 "s59E25H2" 0 "Na(+)/H(+) antiporter subunit D"
"DsuE09_00626" 4543 "s59E5H2" 0 "Na(+)/H(+) antiporter subunit D"
"DsuE09_00626" 4543 "s63E6H2" 0 "Na(+)/H(+) antiporter subunit D"
"DsuE09_00627" 4544 "s59E13H2" 0 "Na(+)/H(+) antiporter subunit C1"
"DsuE09_00627" 4544 "s59E25H2" 0 "Na(+)/H(+) antiporter subunit C1"
"DsuE09_00627" 4544 "s59E5H2" 0 "Na(+)/H(+) antiporter subunit C1"
"DsuE09_00627" 4544 "s63E6H2" 0 "Na(+)/H(+) antiporter subunit C1"
"DsuE09_00628" 4545 "s59E13H2" 0 "putative monovalent cation/H+ antiporter subunit B"
"DsuE09_00628" 4545 "s59E25H2" 0 "putative monovalent cation/H+ antiporter subunit B"
"DsuE09_00628" 4545 "s59E5H2" 0 "putative monovalent cation/H+ antiporter subunit B"
"DsuE09_00628" 4545 "s63E6H2" 0 "putative monovalent cation/H+ antiporter subunit B"
"DsuE09_00629" 4546 "s59E13H2" 0 "putative monovalent cation/H+ antiporter subunit B"
"DsuE09_00629" 4546 "s59E25H2" 0 "putative monovalent cation/H+ antiporter subunit B"
"DsuE09_00629" 4546 "s59E5H2" 0 "putative monovalent cation/H+ antiporter subunit B"
"DsuE09_00629" 4546 "s63E6H2" 0 "putative monovalent cation/H+ antiporter subunit B"
"DsuE09_00630" 4547 "s59E13H2" 0 "Na(+)/H(+) antiporter subunit G"
"DsuE09_00630" 4547 "s59E25H2" 0 "Na(+)/H(+) antiporter subunit G"
"DsuE09_00630" 4547 "s59E5H2" 0 "Na(+)/H(+) antiporter subunit G"
"DsuE09_00630" 4547 "s63E6H2" 0 "Na(+)/H(+) antiporter subunit G"
"DsuE09_00631" 4548 "s59E13H2" 0 "putative monovalent cation/H+ antiporter subunit F"
"DsuE09_00631" 4548 "s59E25H2" 0 "putative monovalent cation/H+ antiporter subunit F"
"DsuE09_00631" 4548 "s59E5H2" 0 "putative monovalent cation/H+ antiporter subunit F"
"DsuE09_00631" 4548 "s63E6H2" 0 "putative monovalent cation/H+ antiporter subunit F"
"DsuE09_00632" 4549 "s59E13H2" 0 "Na(+)/H(+) antiporter subunit B"
"DsuE09_00632" 4549 "s59E25H2" 0 "Na(+)/H(+) antiporter subunit B"

"DsuE09_00632" 4549 "s59E5H2" 0 "Na(+)/H(+) antiporter subunit B"
"DsuE09_00632" 4549 "s63E6H2" 0 "Na(+)/H(+) antiporter subunit B"
"DsuE09_00633" 4550 "s59E13H2" 0 "Na(+)/H(+) antiporter subunit E1"
"DsuE09_00633" 4550 "s59E25H2" 0 "Na(+)/H(+) antiporter subunit E1"
"DsuE09_00633" 4550 "s59E5H2" 0 "Na(+)/H(+) antiporter subunit E1"
"DsuE09_00633" 4550 "s63E6H2" 0 "Na(+)/H(+) antiporter subunit E1"
"DsuE09_00634" 4551 "s59E13H2" 0 "Universal stress protein family protein"
"DsuE09_00634" 4551 "s59E25H2" 0 "Universal stress protein family protein"
"DsuE09_00634" 4551 "s59E5H2" 0 "Universal stress protein family protein"
"DsuE09_00634" 4551 "s63E6H2" 0 "Universal stress protein family protein"
"DsuE09_00636" 4553 "s59E13H2" 0 "Phosphate regulon transcriptional regulatory protein PhoB"
"DsuE09_00636" 4553 "s59E25H2" 0 "Phosphate regulon transcriptional regulatory protein PhoB"
"DsuE09_00636" 4553 "s59E5H2" 0 "Phosphate regulon transcriptional regulatory protein PhoB"
"DsuE09_00636" 4553 "s63E6H2" 0 "Phosphate regulon transcriptional regulatory protein PhoB"
"DsuE09_00637" 4554 "s59E13H2" 0 "Sporulation kinase E"
"DsuE09_00637" 4554 "s59E25H2" 0 "Sporulation kinase E"
"DsuE09_00637" 4554 "s59E5H2" 0 "Sporulation kinase E"
"DsuE09_00637" 4554 "s63E6H2" 0 "Sporulation kinase E"
"DsuE09_00638" 4555 "s59E13H2" 0 "Acetyl-CoA acetyltransferase"
"DsuE09_00638" 4555 "s59E25H2" 0 "Acetyl-CoA acetyltransferase"
"DsuE09_00638" 4555 "s59E5H2" 0 "Acetyl-CoA acetyltransferase"
"DsuE09_00638" 4555 "s63E6H2" 0 "Acetyl-CoA acetyltransferase"
"DsuE09_00639" 4556 "s59E13H2" 0 "3-hydroxypropionyl-coenzyme A dehydratase"
"DsuE09_00639" 4556 "s59E25H2" 0 "3-hydroxypropionyl-coenzyme A dehydratase"
"DsuE09_00639" 4556 "s59E5H2" 0 "3-hydroxypropionyl-coenzyme A dehydratase"
"DsuE09_00639" 4556 "s63E6H2" 0 "3-hydroxypropionyl-coenzyme A dehydratase"
"DsuE09_00640" 4557 "s59E13H2" 0 "Acryloyl-CoA reductase (NADH)"
"DsuE09_00640" 4557 "s59E25H2" 0 "Acryloyl-CoA reductase (NADH)"
"DsuE09_00640" 4557 "s59E5H2" 0 "Acryloyl-CoA reductase (NADH)"
"DsuE09_00640" 4557 "s63E6H2" 0 "Acryloyl-CoA reductase (NADH)"
"DsuE09_00641" 4558 "s59E13H2" 0 "Methylmalonyl-CoA mutase"
"DsuE09_00641" 4558 "s59E25H2" 0 "Methylmalonyl-CoA mutase"
"DsuE09_00641" 4558 "s59E5H2" 0 "Methylmalonyl-CoA mutase"
"DsuE09_00641" 4558 "s63E6H2" 0 "Methylmalonyl-CoA mutase"
"DsuE09_00642" 4559 "s59E13H2" 0 "Methylmalonyl-CoA mutase"
"DsuE09_00642" 4559 "s59E25H2" 0 "Methylmalonyl-CoA mutase"
"DsuE09_00642" 4559 "s59E5H2" 0 "Methylmalonyl-CoA mutase"
"DsuE09_00642" 4559 "s63E6H2" 0 "Methylmalonyl-CoA mutase"
"DsuE09_00643" 4560 "s59E13H2" 0 "putative GTPase/MT1543"
"DsuE09_00643" 4560 "s59E25H2" 0 "putative GTPase/MT1543"
"DsuE09_00643" 4560 "s59E5H2" 0 "putative GTPase/MT1543"
"DsuE09_00643" 4560 "s63E6H2" 0 "putative GTPase/MT1543"
"DsuE09_00644" 4561 "s59E13H2" 0 "hypothetical protein"
"DsuE09_00644" 4561 "s59E25H2" 0 "hypothetical protein"
"DsuE09_00644" 4561 "s59E5H2" 0 "hypothetical protein"
"DsuE09_00644" 4561 "s63E6H2" 0 "hypothetical protein"
"DsuE09_00645" 4562 "s59E13H2" 0 "DNA-binding transcriptional repressor PuuR"

"DsuE09_00645" 4562 "s59E25H2" 0 "DNA-binding transcriptional repressor PuuR"
"DsuE09_00645" 4562 "s59E5H2" 0 "DNA-binding transcriptional repressor PuuR"
"DsuE09_00645" 4562 "s63E6H2" 0 "DNA-binding transcriptional repressor PuuR"
"DsuE09_00646" 4563 "s59E13H2" 0 "Acyl-CoA dehydrogenase"
"DsuE09_00646" 4563 "s59E25H2" 0 "Acyl-CoA dehydrogenase"
"DsuE09_00646" 4563 "s59E5H2" 0 "Acyl-CoA dehydrogenase"
"DsuE09_00646" 4563 "s63E6H2" 0 "Acyl-CoA dehydrogenase"
"DsuE09_00647" 4564 "s59E13H2" 0 "Hydroxymethylglutaryl-CoA lyase YngG"
"DsuE09_00647" 4564 "s59E25H2" 0 "Hydroxymethylglutaryl-CoA lyase YngG"
"DsuE09_00647" 4564 "s59E5H2" 0 "Hydroxymethylglutaryl-CoA lyase YngG"
"DsuE09_00647" 4564 "s63E6H2" 0 "Hydroxymethylglutaryl-CoA lyase YngG"
"DsuE09_00648" 4565 "s59E13H2" 0 "hypothetical protein"
"DsuE09_00648" 4565 "s59E25H2" 0 "hypothetical protein"
"DsuE09_00648" 4565 "s59E5H2" 0 "hypothetical protein"
"DsuE09_00648" 4565 "s63E6H2" 0 "hypothetical protein"
"DsuE09_00649" 4566 "s59E13H2" 0 "Putative N-acetyl-LL-diaminopimelate aminotransferase"
"DsuE09_00649" 4566 "s59E25H2" 0 "Putative N-acetyl-LL-diaminopimelate aminotransferase"
"DsuE09_00649" 4566 "s59E5H2" 0 "Putative N-acetyl-LL-diaminopimelate aminotransferase"
"DsuE09_00649" 4566 "s63E6H2" 0 "Putative N-acetyl-LL-diaminopimelate aminotransferase"
"DsuE09_00650" 4567 "s59E13H2" 0 "hypothetical protein"
"DsuE09_00650" 4567 "s59E25H2" 0 "hypothetical protein"
"DsuE09_00650" 4567 "s59E5H2" 0 "hypothetical protein"
"DsuE09_00650" 4567 "s63E6H2" 0 "hypothetical protein"
"DsuE09_00651" 4568 "s59E13H2" 0 "putative acetyl-CoA acyltransferase"
"DsuE09_00651" 4568 "s59E25H2" 0 "putative acetyl-CoA acyltransferase"
"DsuE09_00651" 4568 "s59E5H2" 0 "putative acetyl-CoA acyltransferase"
"DsuE09_00651" 4568 "s63E6H2" 0 "putative acetyl-CoA acyltransferase"
"DsuE09_00652" 4569 "s59E13H2" 0 "Putative ski2-type helicase"
"DsuE09_00652" 4569 "s59E25H2" 0 "Putative ski2-type helicase"
"DsuE09_00652" 4569 "s59E5H2" 0 "Putative ski2-type helicase"
"DsuE09_00652" 4569 "s63E6H2" 0 "Putative ski2-type helicase"
"DsuE09_00653" 4570 "s59E13H2" 0 "Sec-independent protein translocase protein TatAy"
"DsuE09_00653" 4570 "s59E25H2" 0 "Sec-independent protein translocase protein TatAy"
"DsuE09_00653" 4570 "s59E5H2" 0 "Sec-independent protein translocase protein TatAy"
"DsuE09_00653" 4570 "s63E6H2" 0 "Sec-independent protein translocase protein TatAy"
"DsuE09_00654" 4571 "s59E13H2" 0 "Inositol-1-monophosphatase"
"DsuE09_00654" 4571 "s59E25H2" 0 "Inositol-1-monophosphatase"
"DsuE09_00654" 4571 "s59E5H2" 0 "Inositol-1-monophosphatase"
"DsuE09_00654" 4571 "s63E6H2" 0 "Inositol-1-monophosphatase"
"DsuE09_00655" 4572 "s59E13H2" 0 "outer membrane protease"
"DsuE09_00655" 4572 "s59E25H2" 0 "outer membrane protease"
"DsuE09_00655" 4572 "s59E5H2" 0 "outer membrane protease"
"DsuE09_00655" 4572 "s63E6H2" 0 "outer membrane protease"
"DsuE09_00658" 4575 "s59E13H2" 0 "Tyrosine recombinase XerD"
"DsuE09_00658" 4575 "s59E25H2" 0 "Tyrosine recombinase XerD"
"DsuE09_00658" 4575 "s59E5H2" 0 "Tyrosine recombinase XerD"
"DsuE09_00658" 4575 "s63E6H2" 0 "Tyrosine recombinase XerD"

"DsuE09_00659" 4576 "s59E13H2" 0 "Xaa-Pro dipeptidase"
"DsuE09_00659" 4576 "s59E25H2" 0 "Xaa-Pro dipeptidase"
"DsuE09_00659" 4576 "s59E5H2" 0 "Xaa-Pro dipeptidase"
"DsuE09_00659" 4576 "s63E6H2" 0 "Xaa-Pro dipeptidase"
"DsuE09_00661" 4578 "s59E13H2" 0 "3'(2'),5'-bisphosphate nucleotidase CysQ"
"DsuE09_00661" 4578 "s59E25H2" 0 "3'(2'),5'-bisphosphate nucleotidase CysQ"
"DsuE09_00661" 4578 "s59E5H2" 0 "3'(2'),5'-bisphosphate nucleotidase CysQ"
"DsuE09_00661" 4578 "s63E6H2" 0 "3'(2'),5'-bisphosphate nucleotidase CysQ"
"DsuE09_00662" 4579 "s59E13H2" 0 "Uroporphyrinogen-III C-methyltransferase"
"DsuE09_00662" 4579 "s59E25H2" 0 "Uroporphyrinogen-III C-methyltransferase"
"DsuE09_00662" 4579 "s59E5H2" 0 "Uroporphyrinogen-III C-methyltransferase"
"DsuE09_00662" 4579 "s63E6H2" 0 "Uroporphyrinogen-III C-methyltransferase"
"DsuE09_00663" 4580 "s59E13H2" 0 "Porphobilinogen deaminase"
"DsuE09_00663" 4580 "s59E25H2" 0 "Porphobilinogen deaminase"
"DsuE09_00663" 4580 "s59E5H2" 0 "Porphobilinogen deaminase"
"DsuE09_00663" 4580 "s63E6H2" 0 "Porphobilinogen deaminase"
"DsuE09_00664" 4581 "s59E13H2" 0 "2-C-methyl-D-erythritol 4-phosphate cytidylyltransferase"
"DsuE09_00664" 4581 "s59E25H2" 0 "2-C-methyl-D-erythritol 4-phosphate cytidylyltransferase"
"DsuE09_00664" 4581 "s59E5H2" 0 "2-C-methyl-D-erythritol 4-phosphate cytidylyltransferase"
"DsuE09_00664" 4581 "s63E6H2" 0 "2-C-methyl-D-erythritol 4-phosphate cytidylyltransferase"
"DsuE09_00665" 4582 "s59E13H2" 0 "hypothetical protein"
"DsuE09_00665" 4582 "s59E25H2" 0 "hypothetical protein"
"DsuE09_00665" 4582 "s59E5H2" 0 "hypothetical protein"
"DsuE09_00665" 4582 "s63E6H2" 0 "hypothetical protein"
"DsuE09_00666" 4583 "s59E13H2" 0 "Modulator of FtsH protease HflC"
"DsuE09_00666" 4583 "s59E25H2" 0 "Modulator of FtsH protease HflC"
"DsuE09_00666" 4583 "s59E5H2" 0.005513746 "Modulator of FtsH protease HflC"
"DsuE09_00666" 4583 "s63E6H2" 0 "Modulator of FtsH protease HflC"
"DsuE09_00667" 4584 "s59E13H2" 0 "hypothetical protein"
"DsuE09_00667" 4584 "s59E25H2" 0 "hypothetical protein"
"DsuE09_00667" 4584 "s59E5H2" 0 "hypothetical protein"
"DsuE09_00667" 4584 "s63E6H2" 0 "hypothetical protein"
"DsuE09_00668" 4585 "s59E13H2" 0 "Uracil DNA glycosylase superfamily protein"
"DsuE09_00668" 4585 "s59E25H2" 0 "Uracil DNA glycosylase superfamily protein"
"DsuE09_00668" 4585 "s59E5H2" 0 "Uracil DNA glycosylase superfamily protein"
"DsuE09_00668" 4585 "s63E6H2" 0 "Uracil DNA glycosylase superfamily protein"
"DsuE09_00669" 4586 "s59E13H2" 0 "Coenzyme A biosynthesis bifunctional protein CoaBC"
"DsuE09_00669" 4586 "s59E25H2" 0 "Coenzyme A biosynthesis bifunctional protein CoaBC"
"DsuE09_00669" 4586 "s59E5H2" 0 "Coenzyme A biosynthesis bifunctional protein CoaBC"
"DsuE09_00669" 4586 "s63E6H2" 0 "Coenzyme A biosynthesis bifunctional protein CoaBC"
"DsuE09_00670" 4587 "s59E13H2" 0 "hypothetical protein"
"DsuE09_00670" 4587 "s59E25H2" 0 "hypothetical protein"
"DsuE09_00670" 4587 "s59E5H2" 0 "hypothetical protein"
"DsuE09_00670" 4587 "s63E6H2" 0 "hypothetical protein"
"DsuE09_00671" 4588 "s59E13H2" 0 "YGGT family protein"
"DsuE09_00671" 4588 "s59E25H2" 0 "YGGT family protein"
"DsuE09_00671" 4588 "s59E5H2" 0 "YGGT family protein"

"DsuE09_00671" 4588 "s63E6H2" 0 "YGGT family protein"
"DsuE09_00675" 4592 "s59E13H2" 0 "Putative zinc ribbon domain protein"
"DsuE09_00675" 4592 "s59E25H2" 0 "Putative zinc ribbon domain protein"
"DsuE09_00675" 4592 "s59E5H2" 0 "Putative zinc ribbon domain protein"
"DsuE09_00675" 4592 "s63E6H2" 0 "Putative zinc ribbon domain protein"
"DsuE09_00677" 4594 "s59E13H2" 0 "RNA polymerase sigma factor RpoD"
"DsuE09_00677" 4594 "s59E25H2" 0 "RNA polymerase sigma factor RpoD"
"DsuE09_00677" 4594 "s59E5H2" 0 "RNA polymerase sigma factor RpoD"
"DsuE09_00677" 4594 "s63E6H2" 0 "RNA polymerase sigma factor RpoD"
"DsuE09_00678" 4595 "s59E13H2" 0 "DNA primase"
"DsuE09_00678" 4595 "s59E25H2" 0 "DNA primase"
"DsuE09_00678" 4595 "s59E5H2" 0 "DNA primase"
"DsuE09_00678" 4595 "s63E6H2" 0 "DNA primase"
"DsuE09_00679" 4596 "s59E13H2" 0 "Tyrosine-protein kinase etk"
"DsuE09_00679" 4596 "s59E25H2" 0 "Tyrosine-protein kinase etk"
"DsuE09_00679" 4596 "s59E5H2" 0 "Tyrosine-protein kinase etk"
"DsuE09_00679" 4596 "s63E6H2" 0 "Tyrosine-protein kinase etk"
"DsuE09_00680" 4597 "s59E13H2" 0 "Polysialic acid transport protein KpsD precursor"
"DsuE09_00680" 4597 "s59E25H2" 0 "Polysialic acid transport protein KpsD precursor"
"DsuE09_00680" 4597 "s59E5H2" 0 "Polysialic acid transport protein KpsD precursor"
"DsuE09_00680" 4597 "s63E6H2" 0 "Polysialic acid transport protein KpsD precursor"
"DsuE09_00681" 4598 "s59E13H2" 0 "GIY-YIG nuclease superfamily protein"
"DsuE09_00681" 4598 "s59E25H2" 0 "GIY-YIG nuclease superfamily protein"
"DsuE09_00681" 4598 "s59E5H2" 0 "GIY-YIG nuclease superfamily protein"
"DsuE09_00681" 4598 "s63E6H2" 0 "GIY-YIG nuclease superfamily protein"
"DsuE09_00682" 4599 "s59E13H2" 0 "hypothetical protein"
"DsuE09_00682" 4599 "s59E25H2" 0 "hypothetical protein"
"DsuE09_00682" 4599 "s59E5H2" 0 "hypothetical protein"
"DsuE09_00682" 4599 "s63E6H2" 0 "hypothetical protein"
"DsuE09_00683" 4600 "s59E13H2" 0 "hypothetical protein"
"DsuE09_00683" 4600 "s59E25H2" 0 "hypothetical protein"
"DsuE09_00683" 4600 "s59E5H2" 0 "hypothetical protein"
"DsuE09_00683" 4600 "s63E6H2" 0 "hypothetical protein"
"DsuE09_00684" 4601 "s59E13H2" 0 "UDP-N-acetylgalactosamine-undecaprenyl-phosphate N-acetylgalactosaminephosphotransferase"
"DsuE09_00684" 4601 "s59E25H2" 0 "UDP-N-acetylgalactosamine-undecaprenyl-phosphate N-acetylgalactosaminephosphotransferase"
"DsuE09_00684" 4601 "s59E5H2" 0 "UDP-N-acetylgalactosamine-undecaprenyl-phosphate N-acetylgalactosaminephosphotransferase"
"DsuE09_00684" 4601 "s63E6H2" 0 "UDP-N-acetylgalactosamine-undecaprenyl-phosphate N-acetylgalactosaminephosphotransferase"
"DsuE09_00685" 4602 "s59E13H2" 0 "hypothetical protein"
"DsuE09_00685" 4602 "s59E25H2" 0 "hypothetical protein"
"DsuE09_00685" 4602 "s59E5H2" 0 "hypothetical protein"
"DsuE09_00685" 4602 "s63E6H2" 0 "hypothetical protein"
"DsuE09_00687" 4604 "s59E13H2" 0 "hypothetical protein"
"DsuE09_00687" 4604 "s59E25H2" 0 "hypothetical protein"
"DsuE09_00687" 4604 "s59E5H2" 0 "hypothetical protein"
"DsuE09_00687" 4604 "s63E6H2" 0 "hypothetical protein"
"DsuE09_00689" 4606 "s59E13H2" 0 "hypothetical protein"
"DsuE09_00689" 4606 "s59E25H2" 0 "hypothetical protein"

"DsuE09_00689" 4606 "s59E5H2" 0 "hypothetical protein"
"DsuE09_00689" 4606 "s63E6H2" 0 "hypothetical protein"
"DsuE09_00690" 4607 "s59E13H2" 0 "hypothetical protein"
"DsuE09_00690" 4607 "s59E25H2" 0 "hypothetical protein"
"DsuE09_00690" 4607 "s59E5H2" 0 "hypothetical protein"
"DsuE09_00690" 4607 "s63E6H2" 0 "hypothetical protein"
"DsuE09_00691" 4608 "s59E13H2" 0 "Dissimilatory sulfite reductase D (DsrD)"
"DsuE09_00691" 4608 "s59E25H2" 0 "Dissimilatory sulfite reductase D (DsrD)"
"DsuE09_00691" 4608 "s59E5H2" 0 "Dissimilatory sulfite reductase D (DsrD)"
"DsuE09_00691" 4608 "s63E6H2" 0 "Dissimilatory sulfite reductase D (DsrD)"
"DsuE09_00692" 4609 "s59E13H2" 0 "Sulfite reductase, dissimilatory-type subunit gamma"
"DsuE09_00692" 4609 "s59E25H2" 0 "Sulfite reductase, dissimilatory-type subunit gamma"
"DsuE09_00692" 4609 "s59E5H2" 0 "Sulfite reductase, dissimilatory-type subunit gamma"
"DsuE09_00692" 4609 "s63E6H2" 0 "Sulfite reductase, dissimilatory-type subunit gamma"
"DsuE09_00693" 4610 "s59E13H2" 0 "Cobyrinic acid A,C-diamide synthase"
"DsuE09_00693" 4610 "s59E25H2" 0 "Cobyrinic acid A,C-diamide synthase"
"DsuE09_00693" 4610 "s59E5H2" 0 "Cobyrinic acid A,C-diamide synthase"
"DsuE09_00693" 4610 "s63E6H2" 0 "Cobyrinic acid A,C-diamide synthase"
"DsuE09_00694" 4611 "s59E13H2" 0 "Sulfite reductase, dissimilatory-type subunit beta"
"DsuE09_00694" 4611 "s59E25H2" 0 "Sulfite reductase, dissimilatory-type subunit beta"
"DsuE09_00694" 4611 "s59E5H2" 0 "Sulfite reductase, dissimilatory-type subunit beta"
"DsuE09_00694" 4611 "s63E6H2" 0 "Sulfite reductase, dissimilatory-type subunit beta"
"DsuE09_00695" 4612 "s59E13H2" 0 "Sulfite reductase, dissimilatory-type subunit alpha"
"DsuE09_00695" 4612 "s59E25H2" 0 "Sulfite reductase, dissimilatory-type subunit alpha"
"DsuE09_00695" 4612 "s59E5H2" 0 "Sulfite reductase, dissimilatory-type subunit alpha"
"DsuE09_00695" 4612 "s63E6H2" 0 "Sulfite reductase, dissimilatory-type subunit alpha"
"DsuE09_00696" 4613 "s59E13H2" 0 "Yip1 domain protein"
"DsuE09_00696" 4613 "s59E25H2" 0 "Yip1 domain protein"
"DsuE09_00696" 4613 "s59E5H2" 0 "Yip1 domain protein"
"DsuE09_00696" 4613 "s63E6H2" 0 "Yip1 domain protein"
"DsuE09_00697" 4614 "s59E13H2" 0 "ATP-dependent DNA helicase PcrA"
"DsuE09_00697" 4614 "s59E25H2" 0 "ATP-dependent DNA helicase PcrA"
"DsuE09_00697" 4614 "s59E5H2" 0 "ATP-dependent DNA helicase PcrA"
"DsuE09_00697" 4614 "s63E6H2" 0 "ATP-dependent DNA helicase PcrA"
"DsuE09_00698" 4615 "s59E13H2" 0 "Endonuclease V"
"DsuE09_00698" 4615 "s59E25H2" 0 "Endonuclease V"
"DsuE09_00698" 4615 "s59E5H2" 0 "Endonuclease V"
"DsuE09_00698" 4615 "s63E6H2" 0 "Endonuclease V"
"DsuE09_00699" 4616 "s59E13H2" 0 "GTPase Era"
"DsuE09_00699" 4616 "s59E25H2" 0 "GTPase Era"
"DsuE09_00699" 4616 "s59E5H2" 0 "GTPase Era"
"DsuE09_00699" 4616 "s63E6H2" 0 "GTPase Era"
"DsuE09_00700" 4617 "s59E13H2" 0 "Transcriptional regulatory protein ZraR"
"DsuE09_00700" 4617 "s59E25H2" 0 "Transcriptional regulatory protein ZraR"
"DsuE09_00700" 4617 "s59E5H2" 0 "Transcriptional regulatory protein ZraR"
"DsuE09_00700" 4617 "s63E6H2" 0 "Transcriptional regulatory protein ZraR"
"DsuE09_00701" 4618 "s59E13H2" 0 "Sensor protein ZraS"

"DsuE09_00701" 4618 "s59E25H2" 0 "Sensor protein ZraS"
"DsuE09_00701" 4618 "s59E5H2" 0 "Sensor protein ZraS"
"DsuE09_00701" 4618 "s63E6H2" 0 "Sensor protein ZraS"
"DsuE09_00702" 4619 "s59E13H2" 0 "Bifunctional purine biosynthesis protein PurH"
"DsuE09_00702" 4619 "s59E25H2" 0 "Bifunctional purine biosynthesis protein PurH"
"DsuE09_00702" 4619 "s59E5H2" 0 "Bifunctional purine biosynthesis protein PurH"
"DsuE09_00702" 4619 "s63E6H2" 0 "Bifunctional purine biosynthesis protein PurH"
"DsuE09_00703" 4620 "s59E13H2" 0 "Phosphoserine phosphatase 1"
"DsuE09_00703" 4620 "s59E25H2" 0 "Phosphoserine phosphatase 1"
"DsuE09_00703" 4620 "s59E5H2" 0 "Phosphoserine phosphatase 1"
"DsuE09_00703" 4620 "s63E6H2" 0 "Phosphoserine phosphatase 1"
"DsuE09_00704" 4621 "s59E13H2" 0 "YfaZ precursor"
"DsuE09_00704" 4621 "s59E25H2" 0 "YfaZ precursor"
"DsuE09_00704" 4621 "s59E5H2" 0 "YfaZ precursor"
"DsuE09_00704" 4621 "s63E6H2" 0 "YfaZ precursor"
"DsuE09_00706" 4623 "s59E13H2" 0 "hypothetical protein"
"DsuE09_00706" 4623 "s59E25H2" 0 "hypothetical protein"
"DsuE09_00706" 4623 "s59E5H2" 0 "hypothetical protein"
"DsuE09_00706" 4623 "s63E6H2" 0 "hypothetical protein"
"DsuE09_00707" 4624 "s59E13H2" 0 "3-chloro-4-hydroxyphenylacetate reductive dehalogenase precursor"
"DsuE09_00707" 4624 "s59E25H2" 0 "3-chloro-4-hydroxyphenylacetate reductive dehalogenase precursor"
"DsuE09_00707" 4624 "s59E5H2" 0 "3-chloro-4-hydroxyphenylacetate reductive dehalogenase precursor"
"DsuE09_00707" 4624 "s63E6H2" 0 "3-chloro-4-hydroxyphenylacetate reductive dehalogenase precursor"
"DsuE09_00708" 4625 "s59E13H2" 0 "putative sulfoacetate transporter SauU"
"DsuE09_00708" 4625 "s59E25H2" 0 "putative sulfoacetate transporter SauU"
"DsuE09_00708" 4625 "s59E5H2" 0 "putative sulfoacetate transporter SauU"
"DsuE09_00708" 4625 "s63E6H2" 0 "putative sulfoacetate transporter SauU"
"DsuE09_00709" 4626 "s59E13H2" 0 "Imidazoleglycerol-phosphate dehydratase"
"DsuE09_00709" 4626 "s59E25H2" 0 "Imidazoleglycerol-phosphate dehydratase"
"DsuE09_00709" 4626 "s59E5H2" 0 "Imidazoleglycerol-phosphate dehydratase"
"DsuE09_00709" 4626 "s63E6H2" 0 "Imidazoleglycerol-phosphate dehydratase"
"DsuE09_00710" 4627 "s59E13H2" 0 "putative peptidoglycan biosynthesis protein MurJ"
"DsuE09_00710" 4627 "s59E25H2" 0 "putative peptidoglycan biosynthesis protein MurJ"
"DsuE09_00710" 4627 "s59E5H2" 0 "putative peptidoglycan biosynthesis protein MurJ"
"DsuE09_00710" 4627 "s63E6H2" 0 "putative peptidoglycan biosynthesis protein MurJ"
"DsuE09_00711" 4628 "s59E13H2" 0 "30S ribosomal protein S20"
"DsuE09_00711" 4628 "s59E25H2" 0 "30S ribosomal protein S20"
"DsuE09_00711" 4628 "s59E5H2" 0 "30S ribosomal protein S20"
"DsuE09_00711" 4628 "s63E6H2" 0 "30S ribosomal protein S20"
"DsuE09_00712" 4629 "s59E13H2" 0 "DNA polymerase III subunit delta"
"DsuE09_00712" 4629 "s59E25H2" 0 "DNA polymerase III subunit delta"
"DsuE09_00712" 4629 "s59E5H2" 0 "DNA polymerase III subunit delta"
"DsuE09_00712" 4629 "s63E6H2" 0 "DNA polymerase III subunit delta"
"DsuE09_00713" 4630 "s59E13H2" 0 "hypothetical protein"
"DsuE09_00713" 4630 "s59E25H2" 0 "hypothetical protein"
"DsuE09_00713" 4630 "s59E5H2" 0 "hypothetical protein"
"DsuE09_00713" 4630 "s63E6H2" 0 "hypothetical protein"

"DsuE09_00714" 4631 "s59E13H2" 0 "Leucine--tRNA ligase"
"DsuE09_00714" 4631 "s59E25H2" 0 "Leucine--tRNA ligase"
"DsuE09_00714" 4631 "s59E5H2" 0 "Leucine--tRNA ligase"
"DsuE09_00714" 4631 "s63E6H2" 0 "Leucine--tRNA ligase"
"DsuE09_00715" 4632 "s59E13H2" 0 "hypothetical protein"
"DsuE09_00715" 4632 "s59E25H2" 0 "hypothetical protein"
"DsuE09_00715" 4632 "s59E5H2" 0 "hypothetical protein"
"DsuE09_00715" 4632 "s63E6H2" 0 "hypothetical protein"
"DsuE09_00716" 4633 "s59E13H2" 0 "hypothetical protein"
"DsuE09_00716" 4633 "s59E25H2" 0 "hypothetical protein"
"DsuE09_00716" 4633 "s59E5H2" 0 "hypothetical protein"
"DsuE09_00716" 4633 "s63E6H2" 0 "hypothetical protein"
"DsuE09_00717" 4634 "s59E13H2" 0 "6,7-dimethyl-8-ribityllumazine synthase"
"DsuE09_00717" 4634 "s59E25H2" 0 "6,7-dimethyl-8-ribityllumazine synthase"
"DsuE09_00717" 4634 "s59E5H2" 0 "6,7-dimethyl-8-ribityllumazine synthase"
"DsuE09_00717" 4634 "s63E6H2" 0 "6,7-dimethyl-8-ribityllumazine synthase"
"DsuE09_00718" 4635 "s59E13H2" 0 "Riboflavin biosynthesis protein RibBA"
"DsuE09_00718" 4635 "s59E25H2" 0 "Riboflavin biosynthesis protein RibBA"
"DsuE09_00718" 4635 "s59E5H2" 0 "Riboflavin biosynthesis protein RibBA"
"DsuE09_00718" 4635 "s63E6H2" 0 "Riboflavin biosynthesis protein RibBA"
"DsuE09_00719" 4636 "s59E13H2" 0 "Riboflavin synthase"
"DsuE09_00719" 4636 "s59E25H2" 0 "Riboflavin synthase"
"DsuE09_00719" 4636 "s59E5H2" 0 "Riboflavin synthase"
"DsuE09_00719" 4636 "s63E6H2" 0 "Riboflavin synthase"
"DsuE09_00721" 4638 "s59E13H2" 0 "Riboflavin biosynthesis protein RibD"
"DsuE09_00721" 4638 "s59E25H2" 0 "Riboflavin biosynthesis protein RibD"
"DsuE09_00721" 4638 "s59E5H2" 0 "Riboflavin biosynthesis protein RibD"
"DsuE09_00721" 4638 "s63E6H2" 0 "Riboflavin biosynthesis protein RibD"
"DsuE09_00722" 4639 "s59E13H2" 0 "Transcriptional repressor NrdR"
"DsuE09_00722" 4639 "s59E25H2" 0 "Transcriptional repressor NrdR"
"DsuE09_00722" 4639 "s59E5H2" 0 "Transcriptional repressor NrdR"
"DsuE09_00722" 4639 "s63E6H2" 0 "Transcriptional repressor NrdR"
"DsuE09_00723" 4640 "s59E13H2" 0 "tRNA-specific adenosine deaminase"
"DsuE09_00723" 4640 "s59E25H2" 0 "tRNA-specific adenosine deaminase"
"DsuE09_00723" 4640 "s59E5H2" 0 "tRNA-specific adenosine deaminase"
"DsuE09_00723" 4640 "s63E6H2" 0 "tRNA-specific adenosine deaminase"
"DsuE09_00724" 4641 "s59E13H2" 0 "Ribose-5-phosphate isomerase B"
"DsuE09_00724" 4641 "s59E25H2" 0 "Ribose-5-phosphate isomerase B"
"DsuE09_00724" 4641 "s59E5H2" 0 "Ribose-5-phosphate isomerase B"
"DsuE09_00724" 4641 "s63E6H2" 0 "Ribose-5-phosphate isomerase B"
"DsuE09_00725" 4642 "s59E13H2" 0 "3-oxoacyl-[acyl-carrier-protein] synthase 2"
"DsuE09_00725" 4642 "s59E25H2" 0 "3-oxoacyl-[acyl-carrier-protein] synthase 2"
"DsuE09_00725" 4642 "s59E5H2" 0 "3-oxoacyl-[acyl-carrier-protein] synthase 2"
"DsuE09_00725" 4642 "s63E6H2" 0 "3-oxoacyl-[acyl-carrier-protein] synthase 2"
"DsuE09_00726" 4643 "s59E13H2" 0 "Acyl carrier protein"
"DsuE09_00726" 4643 "s59E25H2" 0 "Acyl carrier protein"
"DsuE09_00726" 4643 "s59E5H2" 0 "Acyl carrier protein"

"DsuE09_00726" 4643 "s63E6H2" 0 "Acyl carrier protein"
"DsuE09_00727" 4644 "s59E13H2" 0 "3-oxoacyl-[acyl-carrier-protein] reductase FabG"
"DsuE09_00727" 4644 "s59E25H2" 0 "3-oxoacyl-[acyl-carrier-protein] reductase FabG"
"DsuE09_00727" 4644 "s59E5H2" 0 "3-oxoacyl-[acyl-carrier-protein] reductase FabG"
"DsuE09_00727" 4644 "s63E6H2" 0 "3-oxoacyl-[acyl-carrier-protein] reductase FabG"
"DsuE09_00728" 4645 "s59E13H2" 0 "Phosphate acyltransferase"
"DsuE09_00728" 4645 "s59E25H2" 0 "Phosphate acyltransferase"
"DsuE09_00728" 4645 "s59E5H2" 0 "Phosphate acyltransferase"
"DsuE09_00728" 4645 "s63E6H2" 0 "Phosphate acyltransferase"
"DsuE09_00729" 4646 "s59E13H2" 0 "hypothetical protein"
"DsuE09_00729" 4646 "s59E25H2" 0 "hypothetical protein"
"DsuE09_00729" 4646 "s59E5H2" 0 "hypothetical protein"
"DsuE09_00729" 4646 "s63E6H2" 0 "hypothetical protein"
"DsuE09_00730" 4647 "s59E13H2" 0 "4-hydroxybutyryl-CoA dehydratase/vinylacetyl-CoA-Delta-isomerase"
"DsuE09_00730" 4647 "s59E25H2" 0 "4-hydroxybutyryl-CoA dehydratase/vinylacetyl-CoA-Delta-isomerase"
"DsuE09_00730" 4647 "s59E5H2" 0 "4-hydroxybutyryl-CoA dehydratase/vinylacetyl-CoA-Delta-isomerase"
"DsuE09_00730" 4647 "s63E6H2" 0 "4-hydroxybutyryl-CoA dehydratase/vinylacetyl-CoA-Delta-isomerase"
"DsuE09_00732" 4649 "s59E13H2" 0 "Phosphosulfolactate synthase"
"DsuE09_00732" 4649 "s59E25H2" 0 "Phosphosulfolactate synthase"
"DsuE09_00732" 4649 "s59E5H2" 0 "Phosphosulfolactate synthase"
"DsuE09_00732" 4649 "s63E6H2" 0 "Phosphosulfolactate synthase"
"DsuE09_00733" 4650 "s59E13H2" 0 "L(+)-tartrate dehydratase subunit alpha"
"DsuE09_00733" 4650 "s59E25H2" 0 "L(+)-tartrate dehydratase subunit alpha"
"DsuE09_00733" 4650 "s59E5H2" 0 "L(+)-tartrate dehydratase subunit alpha"
"DsuE09_00733" 4650 "s63E6H2" 0 "L(+)-tartrate dehydratase subunit alpha"
"DsuE09_00734" 4651 "s59E13H2" 0 "L(+)-tartrate dehydratase subunit beta"
"DsuE09_00734" 4651 "s59E25H2" 0 "L(+)-tartrate dehydratase subunit beta"
"DsuE09_00734" 4651 "s59E5H2" 0 "L(+)-tartrate dehydratase subunit beta"
"DsuE09_00734" 4651 "s63E6H2" 0 "L(+)-tartrate dehydratase subunit beta"
"DsuE09_00735" 4652 "s59E13H2" 0 "hypothetical protein"
"DsuE09_00735" 4652 "s59E25H2" 0 "hypothetical protein"
"DsuE09_00735" 4652 "s59E5H2" 0 "hypothetical protein"
"DsuE09_00735" 4652 "s63E6H2" 0 "hypothetical protein"
"DsuE09_00736" 4653 "s59E13H2" 0 "hypothetical protein"
"DsuE09_00736" 4653 "s59E25H2" 0 "hypothetical protein"
"DsuE09_00736" 4653 "s59E5H2" 0 "hypothetical protein"
"DsuE09_00736" 4653 "s63E6H2" 0 "hypothetical protein"
"DsuE09_00737" 4654 "s59E13H2" 0 "HTH-type transcriptional regulator KipR"
"DsuE09_00737" 4654 "s59E25H2" 0 "HTH-type transcriptional regulator KipR"
"DsuE09_00737" 4654 "s59E5H2" 0 "HTH-type transcriptional regulator KipR"
"DsuE09_00737" 4654 "s63E6H2" 0 "HTH-type transcriptional regulator KipR"
"DsuE09_00738" 4655 "s59E13H2" 0 "3-keto-5-aminohexanoate cleavage enzyme"
"DsuE09_00738" 4655 "s59E25H2" 0 "3-keto-5-aminohexanoate cleavage enzyme"
"DsuE09_00738" 4655 "s59E5H2" 0 "3-keto-5-aminohexanoate cleavage enzyme"
"DsuE09_00738" 4655 "s63E6H2" 0 "3-keto-5-aminohexanoate cleavage enzyme"
"DsuE09_00739" 4656 "s59E13H2" 0 "Pyruvate carboxylase subunit B"
"DsuE09_00739" 4656 "s59E25H2" 0 "Pyruvate carboxylase subunit B"

"DsuE09_00739" 4656 "s59E5H2" 0 "Pyruvate carboxylase subunit B"
"DsuE09_00739" 4656 "s63E6H2" 0 "Pyruvate carboxylase subunit B"
"DsuE09_00740" 4657 "s59E13H2" 0 "hypothetical protein"
"DsuE09_00740" 4657 "s59E25H2" 0 "hypothetical protein"
"DsuE09_00740" 4657 "s59E5H2" 0 "hypothetical protein"
"DsuE09_00740" 4657 "s63E6H2" 0 "hypothetical protein"
"DsuE09_00741" 4658 "s59E13H2" 0 "Putative aliphatic sulfonates-binding protein precursor"
"DsuE09_00741" 4658 "s59E25H2" 0 "Putative aliphatic sulfonates-binding protein precursor"
"DsuE09_00741" 4658 "s59E5H2" 0 "Putative aliphatic sulfonates-binding protein precursor"
"DsuE09_00741" 4658 "s63E6H2" 0 "Putative aliphatic sulfonates-binding protein precursor"
"DsuE09_00742" 4659 "s59E13H2" 0 "imidazolonepropionase"
"DsuE09_00742" 4659 "s59E25H2" 0 "imidazolonepropionase"
"DsuE09_00742" 4659 "s59E5H2" 0 "imidazolonepropionase"
"DsuE09_00742" 4659 "s63E6H2" 0 "imidazolonepropionase"
"DsuE09_00743" 4660 "s59E13H2" 0 "Bicarbonate transport ATP-binding protein CmpD"
"DsuE09_00743" 4660 "s59E25H2" 0 "Bicarbonate transport ATP-binding protein CmpD"
"DsuE09_00743" 4660 "s59E5H2" 0 "Bicarbonate transport ATP-binding protein CmpD"
"DsuE09_00743" 4660 "s63E6H2" 0 "Bicarbonate transport ATP-binding protein CmpD"
"DsuE09_00744" 4661 "s59E13H2" 0 "Bicarbonate transport system permease protein CmpB"
"DsuE09_00744" 4661 "s59E25H2" 0 "Bicarbonate transport system permease protein CmpB"
"DsuE09_00744" 4661 "s59E5H2" 0 "Bicarbonate transport system permease protein CmpB"
"DsuE09_00744" 4661 "s63E6H2" 0 "Bicarbonate transport system permease protein CmpB"
"DsuE09_00745" 4662 "s59E13H2" 0 "Putative transposase"
"DsuE09_00745" 4662 "s59E25H2" 0 "Putative transposase"
"DsuE09_00745" 4662 "s59E5H2" 0 "Putative transposase"
"DsuE09_00745" 4662 "s63E6H2" 0 "Putative transposase"
"DsuE09_00746" 4663 "s59E13H2" 0 "hypothetical protein"
"DsuE09_00746" 4663 "s59E25H2" 0 "hypothetical protein"
"DsuE09_00746" 4663 "s59E5H2" 0 "hypothetical protein"
"DsuE09_00746" 4663 "s63E6H2" 0 "hypothetical protein"
"DsuE09_00748" 4665 "s59E13H2" 0 "hypothetical protein"
"DsuE09_00748" 4665 "s59E25H2" 0 "hypothetical protein"
"DsuE09_00748" 4665 "s59E5H2" 0.4558379 "hypothetical protein"
"DsuE09_00748" 4665 "s63E6H2" 0 "hypothetical protein"
"DsuE09_00749" 4666 "s59E13H2" 0 "hypothetical protein"
"DsuE09_00749" 4666 "s59E25H2" 0 "hypothetical protein"
"DsuE09_00749" 4666 "s59E5H2" 0.5614995 "hypothetical protein"
"DsuE09_00749" 4666 "s63E6H2" 0 "hypothetical protein"
"DsuE09_00750" 4667 "s59E13H2" 0 "Guanidinobutyrase"
"DsuE09_00750" 4667 "s59E25H2" 0 "Guanidinobutyrase"
"DsuE09_00750" 4667 "s59E5H2" 0 "Guanidinobutyrase"
"DsuE09_00750" 4667 "s63E6H2" 0 "Guanidinobutyrase"
"DsuE09_00751" 4668 "s59E13H2" 0 "hypothetical protein"
"DsuE09_00751" 4668 "s59E25H2" 0 "hypothetical protein"
"DsuE09_00751" 4668 "s59E5H2" 0 "hypothetical protein"
"DsuE09_00751" 4668 "s63E6H2" 0 "hypothetical protein"
"DsuE09_00752" 4669 "s59E13H2" 0 "putative HTH-type transcriptional regulator YdfH"

"DsuE09_00752" 4669 "s59E25H2" 0 "putative HTH-type transcriptional regulator YdfH"
"DsuE09_00752" 4669 "s59E5H2" 0 "putative HTH-type transcriptional regulator YdfH"
"DsuE09_00752" 4669 "s63E6H2" 0 "putative HTH-type transcriptional regulator YdfH"
"DsuE09_00753" 4670 "s59E13H2" 0 "Succinate dehydrogenase flavoprotein subunit"
"DsuE09_00753" 4670 "s59E25H2" 0 "Succinate dehydrogenase flavoprotein subunit"
"DsuE09_00753" 4670 "s59E5H2" 0 "Succinate dehydrogenase flavoprotein subunit"
"DsuE09_00753" 4670 "s63E6H2" 0 "Succinate dehydrogenase flavoprotein subunit"
"DsuE09_00754" 4671 "s59E13H2" 0 "Succinate dehydrogenase/Fumarate reductase transmembrane subunit"
"DsuE09_00754" 4671 "s59E25H2" 0 "Succinate dehydrogenase/Fumarate reductase transmembrane subunit"
"DsuE09_00754" 4671 "s59E5H2" 0 "Succinate dehydrogenase/Fumarate reductase transmembrane subunit"
"DsuE09_00754" 4671 "s63E6H2" 0 "Succinate dehydrogenase/Fumarate reductase transmembrane subunit"
"DsuE09_00755" 4672 "s59E13H2" 0 "Succinate dehydrogenase iron-sulfur subunit"
"DsuE09_00755" 4672 "s59E25H2" 0 "Succinate dehydrogenase iron-sulfur subunit"
"DsuE09_00755" 4672 "s59E5H2" 0 "Succinate dehydrogenase iron-sulfur subunit"
"DsuE09_00755" 4672 "s63E6H2" 0 "Succinate dehydrogenase iron-sulfur subunit"
"DsuE09_00756" 4673 "s59E13H2" 0 "Cytochrome b6"
"DsuE09_00756" 4673 "s59E25H2" 0 "Cytochrome b6"
"DsuE09_00756" 4673 "s59E5H2" 0 "Cytochrome b6"
"DsuE09_00756" 4673 "s63E6H2" 0 "Cytochrome b6"
"DsuE09_00757" 4674 "s59E13H2" 0 "L-aspartate oxidase"
"DsuE09_00757" 4674 "s59E25H2" 0 "L-aspartate oxidase"
"DsuE09_00757" 4674 "s59E5H2" 0 "L-aspartate oxidase"
"DsuE09_00757" 4674 "s63E6H2" 0 "L-aspartate oxidase"
"DsuE09_00758" 4675 "s59E13H2" 0 "Sulfide dehydrogenase subunit alpha precursor"
"DsuE09_00758" 4675 "s59E25H2" 0 "Sulfide dehydrogenase subunit alpha precursor"
"DsuE09_00758" 4675 "s59E5H2" 0 "Sulfide dehydrogenase subunit alpha precursor"
"DsuE09_00758" 4675 "s63E6H2" 0 "Sulfide dehydrogenase subunit alpha precursor"
"DsuE09_00759" 4676 "s59E13H2" 0 "hypothetical protein"
"DsuE09_00759" 4676 "s59E25H2" 0 "hypothetical protein"
"DsuE09_00759" 4676 "s59E5H2" 0 "hypothetical protein"
"DsuE09_00759" 4676 "s63E6H2" 0 "hypothetical protein"
"DsuE09_00760" 4677 "s59E13H2" 0 "Methylaspartate mutase S chain"
"DsuE09_00760" 4677 "s59E25H2" 0 "Methylaspartate mutase S chain"
"DsuE09_00760" 4677 "s59E5H2" 0 "Methylaspartate mutase S chain"
"DsuE09_00760" 4677 "s63E6H2" 0 "Methylaspartate mutase S chain"
"DsuE09_00761" 4678 "s59E13H2" 0 "hypothetical protein"
"DsuE09_00761" 4678 "s59E25H2" 0 "hypothetical protein"
"DsuE09_00761" 4678 "s59E5H2" 0 "hypothetical protein"
"DsuE09_00761" 4678 "s63E6H2" 0 "hypothetical protein"
"DsuE09_00762" 4679 "s59E13H2" 0 "Methylaspartate mutase E chain"
"DsuE09_00762" 4679 "s59E25H2" 0 "Methylaspartate mutase E chain"
"DsuE09_00762" 4679 "s59E5H2" 0 "Methylaspartate mutase E chain"
"DsuE09_00762" 4679 "s63E6H2" 0 "Methylaspartate mutase E chain"
"DsuE09_00763" 4680 "s59E13H2" 0 "L-Ala-D/L-Glu epimerase"
"DsuE09_00763" 4680 "s59E25H2" 0 "L-Ala-D/L-Glu epimerase"
"DsuE09_00763" 4680 "s59E5H2" 0 "L-Ala-D/L-Glu epimerase"
"DsuE09_00763" 4680 "s63E6H2" 0 "L-Ala-D/L-Glu epimerase"

"DsuE09_00764" 4681 "s59E13H2" 0 "Tripartite tricarboxylate transporter TctA family protein"
"DsuE09_00764" 4681 "s59E25H2" 0 "Tripartite tricarboxylate transporter TctA family protein"
"DsuE09_00764" 4681 "s59E5H2" 0 "Tripartite tricarboxylate transporter TctA family protein"
"DsuE09_00764" 4681 "s63E6H2" 0 "Tripartite tricarboxylate transporter TctA family protein"
"DsuE09_00766" 4683 "s59E13H2" 0 "GIY-YIG nuclease superfamily protein"
"DsuE09_00766" 4683 "s59E25H2" 0 "GIY-YIG nuclease superfamily protein"
"DsuE09_00766" 4683 "s59E5H2" 0 "GIY-YIG nuclease superfamily protein"
"DsuE09_00766" 4683 "s63E6H2" 0 "GIY-YIG nuclease superfamily protein"
"DsuE09_00767" 4684 "s59E13H2" 0 "NADH dehydrogenase"
"DsuE09_00767" 4684 "s59E25H2" 0 "NADH dehydrogenase"
"DsuE09_00767" 4684 "s59E5H2" 0 "NADH dehydrogenase"
"DsuE09_00767" 4684 "s63E6H2" 0 "NADH dehydrogenase"
"DsuE09_00768" 4685 "s59E13H2" 0 "Long-chain-fatty-acid--CoA ligase"
"DsuE09_00768" 4685 "s59E25H2" 0 "Long-chain-fatty-acid--CoA ligase"
"DsuE09_00768" 4685 "s59E5H2" 0 "Long-chain-fatty-acid--CoA ligase"
"DsuE09_00768" 4685 "s63E6H2" 0 "Long-chain-fatty-acid--CoA ligase"
"DsuE09_00769" 4686 "s59E13H2" 0 "hypothetical protein"
"DsuE09_00769" 4686 "s59E25H2" 0 "hypothetical protein"
"DsuE09_00769" 4686 "s59E5H2" 0 "hypothetical protein"
"DsuE09_00769" 4686 "s63E6H2" 0 "hypothetical protein"
"DsuE09_00771" 4688 "s59E13H2" 0 "hypothetical protein"
"DsuE09_00771" 4688 "s59E25H2" 0 "hypothetical protein"
"DsuE09_00771" 4688 "s59E5H2" 0 "hypothetical protein"
"DsuE09_00771" 4688 "s63E6H2" 0 "hypothetical protein"
"DsuE09_00772" 4689 "s59E13H2" 0 "Thioredoxin-1"
"DsuE09_00772" 4689 "s59E25H2" 0 "Thioredoxin-1"
"DsuE09_00772" 4689 "s59E5H2" 0 "Thioredoxin-1"
"DsuE09_00772" 4689 "s63E6H2" 0 "Thioredoxin-1"
"DsuE09_00773" 4690 "s59E13H2" 0 "Outer membrane protein assembly factor BamD precursor"
"DsuE09_00773" 4690 "s59E25H2" 0 "Outer membrane protein assembly factor BamD precursor"
"DsuE09_00773" 4690 "s59E5H2" 0 "Outer membrane protein assembly factor BamD precursor"
"DsuE09_00773" 4690 "s63E6H2" 0 "Outer membrane protein assembly factor BamD precursor"
"DsuE09_00774" 4691 "s59E13H2" 0 "Molybdopterin molybdenumtransferase"
"DsuE09_00774" 4691 "s59E25H2" 0 "Molybdopterin molybdenumtransferase"
"DsuE09_00774" 4691 "s59E5H2" 0 "Molybdopterin molybdenumtransferase"
"DsuE09_00774" 4691 "s63E6H2" 0 "Molybdopterin molybdenumtransferase"
"DsuE09_00775" 4692 "s59E13H2" 0 "Molybdopterin molybdenumtransferase"
"DsuE09_00775" 4692 "s59E25H2" 0 "Molybdopterin molybdenumtransferase"
"DsuE09_00775" 4692 "s59E5H2" 0 "Molybdopterin molybdenumtransferase"
"DsuE09_00775" 4692 "s63E6H2" 0 "Molybdopterin molybdenumtransferase"
"DsuE09_00776" 4693 "s59E13H2" 0 "DNA-binding transcriptional regulator ModE"
"DsuE09_00776" 4693 "s59E25H2" 0 "DNA-binding transcriptional regulator ModE"
"DsuE09_00776" 4693 "s59E5H2" 0 "DNA-binding transcriptional regulator ModE"
"DsuE09_00776" 4693 "s63E6H2" 0 "DNA-binding transcriptional regulator ModE"
"DsuE09_00777" 4694 "s59E13H2" 0 "ferredoxin"
"DsuE09_00777" 4694 "s59E25H2" 0 "ferredoxin"
"DsuE09_00777" 4694 "s59E5H2" 0 "ferredoxin"

"DsuE09_00777" 4694 "s63E6H2" 0 "ferredoxin"
"DsuE09_00778" 4695 "s59E13H2" 0 "Cyclic pyranopterin monophosphate synthase"
"DsuE09_00778" 4695 "s59E25H2" 0 "Cyclic pyranopterin monophosphate synthase"
"DsuE09_00778" 4695 "s59E5H2" 0 "Cyclic pyranopterin monophosphate synthase"
"DsuE09_00778" 4695 "s63E6H2" 0 "Cyclic pyranopterin monophosphate synthase"
"DsuE09_00779" 4696 "s59E13H2" 0 "Electron transport protein HydN"
"DsuE09_00779" 4696 "s59E25H2" 0 "Electron transport protein HydN"
"DsuE09_00779" 4696 "s59E5H2" 0 "Electron transport protein HydN"
"DsuE09_00779" 4696 "s63E6H2" 0 "Electron transport protein HydN"
"DsuE09_00780" 4697 "s59E13H2" 0 "Tungsten-containing aldehyde ferredoxin oxidoreductase"
"DsuE09_00780" 4697 "s59E25H2" 0 "Tungsten-containing aldehyde ferredoxin oxidoreductase"
"DsuE09_00780" 4697 "s59E5H2" 0 "Tungsten-containing aldehyde ferredoxin oxidoreductase"
"DsuE09_00780" 4697 "s63E6H2" 0 "Tungsten-containing aldehyde ferredoxin oxidoreductase"
"DsuE09_00781" 4698 "s59E13H2" 0 "hypothetical protein"
"DsuE09_00781" 4698 "s59E25H2" 0 "hypothetical protein"
"DsuE09_00781" 4698 "s59E5H2" 0 "hypothetical protein"
"DsuE09_00781" 4698 "s63E6H2" 0 "hypothetical protein"
"DsuE09_00782" 4699 "s59E13H2" 0 "formate hydrogenlyase complex iron-sulfur subunit"
"DsuE09_00782" 4699 "s59E25H2" 0 "formate hydrogenlyase complex iron-sulfur subunit"
"DsuE09_00782" 4699 "s59E5H2" 0 "formate hydrogenlyase complex iron-sulfur subunit"
"DsuE09_00782" 4699 "s63E6H2" 0 "formate hydrogenlyase complex iron-sulfur subunit"
"DsuE09_00783" 4700 "s59E13H2" 0 "Selenocysteine-specific elongation factor"
"DsuE09_00783" 4700 "s59E25H2" 0 "Selenocysteine-specific elongation factor"
"DsuE09_00783" 4700 "s59E5H2" 0 "Selenocysteine-specific elongation factor"
"DsuE09_00783" 4700 "s63E6H2" 0 "Selenocysteine-specific elongation factor"
"DsuE09_00785" 4702 "s59E13H2" 0 "GIY-YIG nuclease superfamily protein"
"DsuE09_00785" 4702 "s59E25H2" 0 "GIY-YIG nuclease superfamily protein"
"DsuE09_00785" 4702 "s59E5H2" 0 "GIY-YIG nuclease superfamily protein"
"DsuE09_00785" 4702 "s63E6H2" 0 "GIY-YIG nuclease superfamily protein"
"DsuE09_00786" 4703 "s59E13H2" 0 "hypothetical protein"
"DsuE09_00786" 4703 "s59E25H2" 0 "hypothetical protein"
"DsuE09_00786" 4703 "s59E5H2" 0 "hypothetical protein"
"DsuE09_00786" 4703 "s63E6H2" 0 "hypothetical protein"
"DsuE09_00787" 4704 "s59E13H2" 0 "Oxygen sensor histidine kinase NreB"
"DsuE09_00787" 4704 "s59E25H2" 0 "Oxygen sensor histidine kinase NreB"
"DsuE09_00787" 4704 "s59E5H2" 0 "Oxygen sensor histidine kinase NreB"
"DsuE09_00787" 4704 "s63E6H2" 0 "Oxygen sensor histidine kinase NreB"
"DsuE09_00788" 4705 "s59E13H2" 0 "hypothetical protein"
"DsuE09_00788" 4705 "s59E25H2" 0 "hypothetical protein"
"DsuE09_00788" 4705 "s59E5H2" 0 "hypothetical protein"
"DsuE09_00788" 4705 "s63E6H2" 0 "hypothetical protein"
"DsuE09_00790" 4707 "s59E13H2" 0 "tRNA(fMet)-specific endonuclease VapC"
"DsuE09_00790" 4707 "s59E25H2" 0 "tRNA(fMet)-specific endonuclease VapC"
"DsuE09_00790" 4707 "s59E5H2" 0 "tRNA(fMet)-specific endonuclease VapC"
"DsuE09_00790" 4707 "s63E6H2" 0 "tRNA(fMet)-specific endonuclease VapC"
"DsuE09_00791" 4708 "s59E13H2" 0 "hypothetical protein"
"DsuE09_00791" 4708 "s59E25H2" 0 "hypothetical protein"

"DsuE09_00791" 4708 "s59E5H2" 0 "hypothetical protein"
"DsuE09_00791" 4708 "s63E6H2" 0 "hypothetical protein"
"DsuE09_00792" 4709 "s59E13H2" 0 "Beta-1,4-mannooligosaccharide phosphorylase"
"DsuE09_00792" 4709 "s59E25H2" 0 "Beta-1,4-mannooligosaccharide phosphorylase"
"DsuE09_00792" 4709 "s59E5H2" 0 "Beta-1,4-mannooligosaccharide phosphorylase"
"DsuE09_00792" 4709 "s63E6H2" 0 "Beta-1,4-mannooligosaccharide phosphorylase"
"DsuE09_00793" 4710 "s59E13H2" 0 "hypothetical protein"
"DsuE09_00793" 4710 "s59E25H2" 0 "hypothetical protein"
"DsuE09_00793" 4710 "s59E5H2" 0 "hypothetical protein"
"DsuE09_00793" 4710 "s63E6H2" 0 "hypothetical protein"
"DsuE09_00797" 4714 "s59E13H2" 0 "Chromosomal replication initiator protein DnaA"
"DsuE09_00797" 4714 "s59E25H2" 0 "Chromosomal replication initiator protein DnaA"
"DsuE09_00797" 4714 "s59E5H2" 0 "Chromosomal replication initiator protein DnaA"
"DsuE09_00797" 4714 "s63E6H2" 0 "Chromosomal replication initiator protein DnaA"
"DsuE09_00798" 4715 "s59E13H2" 0 "DNA polymerase V subunit UmuD"
"DsuE09_00798" 4715 "s59E25H2" 0 "DNA polymerase V subunit UmuD"
"DsuE09_00798" 4715 "s59E5H2" 0 "DNA polymerase V subunit UmuD"
"DsuE09_00798" 4715 "s63E6H2" 0 "DNA polymerase V subunit UmuD"
"DsuE09_00799" 4716 "s59E13H2" 0 "Succinyl-CoA:(R)-benzylsuccinate CoA-transferase subunit BbsF"
"DsuE09_00799" 4716 "s59E25H2" 0 "Succinyl-CoA:(R)-benzylsuccinate CoA-transferase subunit BbsF"
"DsuE09_00799" 4716 "s59E5H2" 0 "Succinyl-CoA:(R)-benzylsuccinate CoA-transferase subunit BbsF"
"DsuE09_00799" 4716 "s63E6H2" 0 "Succinyl-CoA:(R)-benzylsuccinate CoA-transferase subunit BbsF"
"DsuE09_00800" 4717 "s59E13H2" 0 "Dihydroorotase"
"DsuE09_00800" 4717 "s59E25H2" 0 "Dihydroorotase"
"DsuE09_00800" 4717 "s59E5H2" 0 "Dihydroorotase"
"DsuE09_00800" 4717 "s63E6H2" 0 "Dihydroorotase"
"DsuE09_00801" 4718 "s59E13H2" 0 "Iron-sulfur flavoprotein"
"DsuE09_00801" 4718 "s59E25H2" 0 "Iron-sulfur flavoprotein"
"DsuE09_00801" 4718 "s59E5H2" 0 "Iron-sulfur flavoprotein"
"DsuE09_00801" 4718 "s63E6H2" 0 "Iron-sulfur flavoprotein"
"DsuE09_00802" 4719 "s59E13H2" 0 "3-oxoacyl-[acyl-carrier-protein] reductase FabG"
"DsuE09_00802" 4719 "s59E25H2" 0 "3-oxoacyl-[acyl-carrier-protein] reductase FabG"
"DsuE09_00802" 4719 "s59E5H2" 0 "3-oxoacyl-[acyl-carrier-protein] reductase FabG"
"DsuE09_00802" 4719 "s63E6H2" 0 "3-oxoacyl-[acyl-carrier-protein] reductase FabG"
"DsuE09_00803" 4720 "s59E13H2" 0 "putative 3-hydroxybutyryl-CoA dehydrogenase"
"DsuE09_00803" 4720 "s59E25H2" 0 "putative 3-hydroxybutyryl-CoA dehydrogenase"
"DsuE09_00803" 4720 "s59E5H2" 0 "putative 3-hydroxybutyryl-CoA dehydrogenase"
"DsuE09_00803" 4720 "s63E6H2" 0 "putative 3-hydroxybutyryl-CoA dehydrogenase"
"DsuE09_00804" 4721 "s59E13H2" 0 "Acyl-CoA dehydrogenase"
"DsuE09_00804" 4721 "s59E25H2" 0 "Acyl-CoA dehydrogenase"
"DsuE09_00804" 4721 "s59E5H2" 0 "Acyl-CoA dehydrogenase"
"DsuE09_00804" 4721 "s63E6H2" 0 "Acyl-CoA dehydrogenase"
"DsuE09_00805" 4722 "s59E13H2" 0 "Acyl-CoA dehydrogenase"
"DsuE09_00805" 4722 "s59E25H2" 0 "Acyl-CoA dehydrogenase"
"DsuE09_00805" 4722 "s59E5H2" 0 "Acyl-CoA dehydrogenase"
"DsuE09_00805" 4722 "s63E6H2" 0 "Acyl-CoA dehydrogenase"
"DsuE09_00806" 4723 "s59E13H2" 0 "NMT1/THI5 like protein"

"DsuE09_00806" 4723 "s59E25H2" 0 "NMT1/THI5 like protein"
"DsuE09_00806" 4723 "s59E5H2" 0 "NMT1/THI5 like protein"
"DsuE09_00806" 4723 "s63E6H2" 0 "NMT1/THI5 like protein"
"DsuE09_00807" 4724 "s59E13H2" 0 "Sialic acid TRAP transporter permease protein SiaT"
"DsuE09_00807" 4724 "s59E25H2" 0 "Sialic acid TRAP transporter permease protein SiaT"
"DsuE09_00807" 4724 "s59E5H2" 0 "Sialic acid TRAP transporter permease protein SiaT"
"DsuE09_00807" 4724 "s63E6H2" 0 "Sialic acid TRAP transporter permease protein SiaT"
"DsuE09_00808" 4725 "s59E13H2" 0 "2-dehydro-3-deoxy-D-gluconate 5-dehydrogenase"
"DsuE09_00808" 4725 "s59E25H2" 0 "2-dehydro-3-deoxy-D-gluconate 5-dehydrogenase"
"DsuE09_00808" 4725 "s59E5H2" 0 "2-dehydro-3-deoxy-D-gluconate 5-dehydrogenase"
"DsuE09_00808" 4725 "s63E6H2" 0 "2-dehydro-3-deoxy-D-gluconate 5-dehydrogenase"
"DsuE09_00809" 4726 "s59E13H2" 0 "Acryloyl-CoA reductase (NADH)"
"DsuE09_00809" 4726 "s59E25H2" 0 "Acryloyl-CoA reductase (NADH)"
"DsuE09_00809" 4726 "s59E5H2" 0 "Acryloyl-CoA reductase (NADH)"
"DsuE09_00809" 4726 "s63E6H2" 0 "Acryloyl-CoA reductase (NADH)"
"DsuE09_00810" 4727 "s59E13H2" 0 "4-hydroxybutyryl-CoA dehydratase/vinylacetyl-CoA-Delta-isomerase"
"DsuE09_00810" 4727 "s59E25H2" 0 "4-hydroxybutyryl-CoA dehydratase/vinylacetyl-CoA-Delta-isomerase"
"DsuE09_00810" 4727 "s59E5H2" 0 "4-hydroxybutyryl-CoA dehydratase/vinylacetyl-CoA-Delta-isomerase"
"DsuE09_00810" 4727 "s63E6H2" 0 "4-hydroxybutyryl-CoA dehydratase/vinylacetyl-CoA-Delta-isomerase"
"DsuE09_00811" 4728 "s59E13H2" 0 "Carbon starvation protein A"
"DsuE09_00811" 4728 "s59E25H2" 0 "Carbon starvation protein A"
"DsuE09_00811" 4728 "s59E5H2" 0 "Carbon starvation protein A"
"DsuE09_00811" 4728 "s63E6H2" 0 "Carbon starvation protein A"
"DsuE09_00812" 4729 "s59E13H2" 0 "hypothetical protein"
"DsuE09_00812" 4729 "s59E25H2" 0 "hypothetical protein"
"DsuE09_00812" 4729 "s59E5H2" 0 "hypothetical protein"
"DsuE09_00812" 4729 "s63E6H2" 0 "hypothetical protein"
"DsuE09_00814" 4731 "s59E13H2" 0 "hypothetical protein"
"DsuE09_00814" 4731 "s59E25H2" 0 "hypothetical protein"
"DsuE09_00814" 4731 "s59E5H2" 0 "hypothetical protein"
"DsuE09_00814" 4731 "s63E6H2" 0 "hypothetical protein"
"DsuE09_00816" 4733 "s59E13H2" 0 "Putative arsenical pump-driving ATPase"
"DsuE09_00816" 4733 "s59E25H2" 0 "Putative arsenical pump-driving ATPase"
"DsuE09_00816" 4733 "s59E5H2" 0 "Putative arsenical pump-driving ATPase"
"DsuE09_00816" 4733 "s63E6H2" 0 "Putative arsenical pump-driving ATPase"
"DsuE09_00817" 4734 "s59E13H2" 0 "Putative arsenical pump-driving ATPase"
"DsuE09_00817" 4734 "s59E25H2" 0 "Putative arsenical pump-driving ATPase"
"DsuE09_00817" 4734 "s59E5H2" 0 "Putative arsenical pump-driving ATPase"
"DsuE09_00817" 4734 "s63E6H2" 0 "Putative arsenical pump-driving ATPase"
"DsuE09_00818" 4735 "s59E13H2" 0 "hypothetical protein"
"DsuE09_00818" 4735 "s59E25H2" 0 "hypothetical protein"
"DsuE09_00818" 4735 "s59E5H2" 0 "hypothetical protein"
"DsuE09_00818" 4735 "s63E6H2" 0 "hypothetical protein"
"DsuE09_00819" 4736 "s59E13H2" 0 "Adenylosuccinate synthetase"
"DsuE09_00819" 4736 "s59E25H2" 0 "Adenylosuccinate synthetase"
"DsuE09_00819" 4736 "s59E5H2" 0 "Adenylosuccinate synthetase"
"DsuE09_00819" 4736 "s63E6H2" 0 "Adenylosuccinate synthetase"

"DsuE09_00820" 4737 "s59E13H2" 0 "PAC2 family protein"
"DsuE09_00820" 4737 "s59E25H2" 0 "PAC2 family protein"
"DsuE09_00820" 4737 "s59E5H2" 0 "PAC2 family protein"
"DsuE09_00820" 4737 "s63E6H2" 0 "PAC2 family protein"
"DsuE09_00821" 4738 "s59E13H2" 0 "dTDP-3-amino-3,6-dideoxy-alpha-D-galactopyranose transaminase"
"DsuE09_00821" 4738 "s59E25H2" 0 "dTDP-3-amino-3,6-dideoxy-alpha-D-galactopyranose transaminase"
"DsuE09_00821" 4738 "s59E5H2" 0 "dTDP-3-amino-3,6-dideoxy-alpha-D-galactopyranose transaminase"
"DsuE09_00821" 4738 "s63E6H2" 0 "dTDP-3-amino-3,6-dideoxy-alpha-D-galactopyranose transaminase"
"DsuE09_00822" 4739 "s59E13H2" 0 "Divergent polysaccharide deacetylase"
"DsuE09_00822" 4739 "s59E25H2" 0 "Divergent polysaccharide deacetylase"
"DsuE09_00822" 4739 "s59E5H2" 0 "Divergent polysaccharide deacetylase"
"DsuE09_00822" 4739 "s63E6H2" 0 "Divergent polysaccharide deacetylase"
"DsuE09_00823" 4740 "s59E13H2" 0 "Carboxy-terminal processing protease CtpA precursor"
"DsuE09_00823" 4740 "s59E25H2" 0 "Carboxy-terminal processing protease CtpA precursor"
"DsuE09_00823" 4740 "s59E5H2" 0 "Carboxy-terminal processing protease CtpA precursor"
"DsuE09_00823" 4740 "s63E6H2" 0 "Carboxy-terminal processing protease CtpA precursor"
"DsuE09_00824" 4741 "s59E13H2" 0 "Murein hydrolase activator EnvC precursor"
"DsuE09_00824" 4741 "s59E25H2" 0 "Murein hydrolase activator EnvC precursor"
"DsuE09_00824" 4741 "s59E5H2" 0 "Murein hydrolase activator EnvC precursor"
"DsuE09_00824" 4741 "s63E6H2" 0 "Murein hydrolase activator EnvC precursor"
"DsuE09_00825" 4742 "s59E13H2" 0 "Cell division protein FtsX"
"DsuE09_00825" 4742 "s59E25H2" 0 "Cell division protein FtsX"
"DsuE09_00825" 4742 "s59E5H2" 0 "Cell division protein FtsX"
"DsuE09_00825" 4742 "s63E6H2" 0 "Cell division protein FtsX"
"DsuE09_00826" 4743 "s59E13H2" 0 "Cell division ATP-binding protein FtsE"
"DsuE09_00826" 4743 "s59E25H2" 0 "Cell division ATP-binding protein FtsE"
"DsuE09_00826" 4743 "s59E5H2" 0 "Cell division ATP-binding protein FtsE"
"DsuE09_00826" 4743 "s63E6H2" 0 "Cell division ATP-binding protein FtsE"
"DsuE09_00827" 4744 "s59E13H2" 0 "Ribonuclease Y"
"DsuE09_00827" 4744 "s59E25H2" 0 "Ribonuclease Y"
"DsuE09_00827" 4744 "s59E5H2" 0 "Ribonuclease Y"
"DsuE09_00827" 4744 "s63E6H2" 0 "Ribonuclease Y"
"DsuE09_00829" 4746 "s59E13H2" 0 "Cell division protein ZapA"
"DsuE09_00829" 4746 "s59E25H2" 0 "Cell division protein ZapA"
"DsuE09_00829" 4746 "s59E5H2" 0 "Cell division protein ZapA"
"DsuE09_00829" 4746 "s63E6H2" 0 "Cell division protein ZapA"
"DsuE09_00830" 4747 "s59E13H2" 0 "hypothetical protein"
"DsuE09_00830" 4747 "s59E25H2" 0 "hypothetical protein"
"DsuE09_00830" 4747 "s59E5H2" 0 "hypothetical protein"
"DsuE09_00830" 4747 "s63E6H2" 0 "hypothetical protein"
"DsuE09_00831" 4748 "s59E13H2" 0 "V-type sodium ATPase subunit D"
"DsuE09_00831" 4748 "s59E25H2" 0 "V-type sodium ATPase subunit D"
"DsuE09_00831" 4748 "s59E5H2" 0 "V-type sodium ATPase subunit D"
"DsuE09_00831" 4748 "s63E6H2" 0 "V-type sodium ATPase subunit D"
"DsuE09_00832" 4749 "s59E13H2" 0 "V-type ATP synthase beta chain"
"DsuE09_00832" 4749 "s59E25H2" 0 "V-type ATP synthase beta chain"
"DsuE09_00832" 4749 "s59E5H2" 0 "V-type ATP synthase beta chain"

"DsuE09_00832" 4749 "s63E6H2" 0 "V-type ATP synthase beta chain"
"DsuE09_00833" 4750 "s59E13H2" 0 "V-type ATP synthase alpha chain"
"DsuE09_00833" 4750 "s59E25H2" 0 "V-type ATP synthase alpha chain"
"DsuE09_00833" 4750 "s59E5H2" 0 "V-type ATP synthase alpha chain"
"DsuE09_00833" 4750 "s63E6H2" 0 "V-type ATP synthase alpha chain"
"DsuE09_00834" 4751 "s59E13H2" 0 "V-type ATP synthase subunit F"
"DsuE09_00834" 4751 "s59E25H2" 0 "V-type ATP synthase subunit F"
"DsuE09_00834" 4751 "s59E5H2" 0 "V-type ATP synthase subunit F"
"DsuE09_00834" 4751 "s63E6H2" 0 "V-type ATP synthase subunit F"
"DsuE09_00835" 4752 "s59E13H2" 0 "V-type ATP synthase subunit C"
"DsuE09_00835" 4752 "s59E25H2" 0 "V-type ATP synthase subunit C"
"DsuE09_00835" 4752 "s59E5H2" 0 "V-type ATP synthase subunit C"
"DsuE09_00835" 4752 "s63E6H2" 0 "V-type ATP synthase subunit C"
"DsuE09_00836" 4753 "s59E13H2" 0 "V-type ATP synthase subunit E"
"DsuE09_00836" 4753 "s59E25H2" 0 "V-type ATP synthase subunit E"
"DsuE09_00836" 4753 "s59E5H2" 0 "V-type ATP synthase subunit E"
"DsuE09_00836" 4753 "s63E6H2" 0 "V-type ATP synthase subunit E"
"DsuE09_00837" 4754 "s59E13H2" 0 "V-type sodium ATPase subunit K"
"DsuE09_00837" 4754 "s59E25H2" 0 "V-type sodium ATPase subunit K"
"DsuE09_00837" 4754 "s59E5H2" 0 "V-type sodium ATPase subunit K"
"DsuE09_00837" 4754 "s63E6H2" 0 "V-type sodium ATPase subunit K"
"DsuE09_00838" 4755 "s59E13H2" 0 "V-type ATP synthase subunit I"
"DsuE09_00838" 4755 "s59E25H2" 0 "V-type ATP synthase subunit I"
"DsuE09_00838" 4755 "s59E5H2" 0 "V-type ATP synthase subunit I"
"DsuE09_00838" 4755 "s63E6H2" 0 "V-type ATP synthase subunit I"
"DsuE09_00839" 4756 "s59E13H2" 0 "V-type ATP synthase subunit H"
"DsuE09_00839" 4756 "s59E25H2" 0 "V-type ATP synthase subunit H"
"DsuE09_00839" 4756 "s59E5H2" 0 "V-type ATP synthase subunit H"
"DsuE09_00839" 4756 "s63E6H2" 0 "V-type ATP synthase subunit H"
"DsuE09_00840" 4757 "s59E13H2" 0 "Polymer-forming cytoskeletal"
"DsuE09_00840" 4757 "s59E25H2" 0 "Polymer-forming cytoskeletal"
"DsuE09_00840" 4757 "s59E5H2" 0 "Polymer-forming cytoskeletal"
"DsuE09_00840" 4757 "s63E6H2" 0 "Polymer-forming cytoskeletal"
"DsuE09_00841" 4758 "s59E13H2" 0 "Rod shape-determining protein RodA"
"DsuE09_00841" 4758 "s59E25H2" 0 "Rod shape-determining protein RodA"
"DsuE09_00841" 4758 "s59E5H2" 0 "Rod shape-determining protein RodA"
"DsuE09_00841" 4758 "s63E6H2" 0 "Rod shape-determining protein RodA"
"DsuE09_00842" 4759 "s59E13H2" 0 "Stage V sporulation protein D"
"DsuE09_00842" 4759 "s59E25H2" 0 "Stage V sporulation protein D"
"DsuE09_00842" 4759 "s59E5H2" 0 "Stage V sporulation protein D"
"DsuE09_00842" 4759 "s63E6H2" 0 "Stage V sporulation protein D"
"DsuE09_00843" 4760 "s59E13H2" 0 "hypothetical protein"
"DsuE09_00843" 4760 "s59E25H2" 0 "hypothetical protein"
"DsuE09_00843" 4760 "s59E5H2" 0 "hypothetical protein"
"DsuE09_00843" 4760 "s63E6H2" 0 "hypothetical protein"
"DsuE09_00844" 4761 "s59E13H2" 0 "Cell shape-determining protein MreC"
"DsuE09_00844" 4761 "s59E25H2" 0 "Cell shape-determining protein MreC"

"DsuE09_00844" 4761 "s59E5H2" 0 "Cell shape-determining protein MreC"
"DsuE09_00844" 4761 "s63E6H2" 0 "Cell shape-determining protein MreC"
"DsuE09_00845" 4762 "s59E13H2" 0 "Rod shape-determining protein MreB"
"DsuE09_00845" 4762 "s59E25H2" 0 "Rod shape-determining protein MreB"
"DsuE09_00845" 4762 "s59E5H2" 0 "Rod shape-determining protein MreB"
"DsuE09_00845" 4762 "s63E6H2" 0 "Rod shape-determining protein MreB"
"DsuE09_00846" 4763 "s59E13H2" 0 "Ribonuclease P protein component"
"DsuE09_00846" 4763 "s59E25H2" 0 "Ribonuclease P protein component"
"DsuE09_00846" 4763 "s59E5H2" 0 "Ribonuclease P protein component"
"DsuE09_00846" 4763 "s63E6H2" 0 "Ribonuclease P protein component"
"DsuE09_00847" 4764 "s59E13H2" 0 "Putative membrane protein insertion efficiency factor"
"DsuE09_00847" 4764 "s59E25H2" 0 "Putative membrane protein insertion efficiency factor"
"DsuE09_00847" 4764 "s59E5H2" 0 "Putative membrane protein insertion efficiency factor"
"DsuE09_00847" 4764 "s63E6H2" 0 "Putative membrane protein insertion efficiency factor"
"DsuE09_00848" 4765 "s59E13H2" 0 "Membrane protein insertase YidC"
"DsuE09_00848" 4765 "s59E25H2" 0 "Membrane protein insertase YidC"
"DsuE09_00848" 4765 "s59E5H2" 0 "Membrane protein insertase YidC"
"DsuE09_00848" 4765 "s63E6H2" 0 "Membrane protein insertase YidC"
"DsuE09_00849" 4766 "s59E13H2" 0 "hypothetical protein"
"DsuE09_00849" 4766 "s59E25H2" 0 "hypothetical protein"
"DsuE09_00849" 4766 "s59E5H2" 0 "hypothetical protein"
"DsuE09_00849" 4766 "s63E6H2" 0 "hypothetical protein"
"DsuE09_00850" 4767 "s59E13H2" 0 "tRNA modification GTPase MnmE"
"DsuE09_00850" 4767 "s59E25H2" 0 "tRNA modification GTPase MnmE"
"DsuE09_00850" 4767 "s59E5H2" 0 "tRNA modification GTPase MnmE"
"DsuE09_00850" 4767 "s63E6H2" 0 "tRNA modification GTPase MnmE"
"DsuE09_00851" 4768 "s59E13H2" 0 "Glutaryl-CoA dehydrogenase"
"DsuE09_00851" 4768 "s59E25H2" 0 "Glutaryl-CoA dehydrogenase"
"DsuE09_00851" 4768 "s59E5H2" 0 "Glutaryl-CoA dehydrogenase"
"DsuE09_00851" 4768 "s63E6H2" 0 "Glutaryl-CoA dehydrogenase"
"DsuE09_00852" 4769 "s59E13H2" 0 "DNA polymerase III subunit beta"
"DsuE09_00852" 4769 "s59E25H2" 0 "DNA polymerase III subunit beta"
"DsuE09_00852" 4769 "s59E5H2" 0 "DNA polymerase III subunit beta"
"DsuE09_00852" 4769 "s63E6H2" 0 "DNA polymerase III subunit beta"
"DsuE09_00853" 4770 "s59E13H2" 0 "DNA gyrase subunit B"
"DsuE09_00853" 4770 "s59E25H2" 0 "DNA gyrase subunit B"
"DsuE09_00853" 4770 "s59E5H2" 0 "DNA gyrase subunit B"
"DsuE09_00853" 4770 "s63E6H2" 0 "DNA gyrase subunit B"
"DsuE09_00854" 4771 "s59E13H2" 0 "DNA gyrase subunit A"
"DsuE09_00854" 4771 "s59E25H2" 0 "DNA gyrase subunit A"
"DsuE09_00854" 4771 "s59E5H2" 0.001669935 "DNA gyrase subunit A"
"DsuE09_00854" 4771 "s63E6H2" 0 "DNA gyrase subunit A"
"DsuE09_00856" 4773 "s59E13H2" 0 "Bifunctional ligase/repressor BirA"
"DsuE09_00856" 4773 "s59E25H2" 0 "Bifunctional ligase/repressor BirA"
"DsuE09_00856" 4773 "s59E5H2" 0 "Bifunctional ligase/repressor BirA"
"DsuE09_00856" 4773 "s63E6H2" 0 "Bifunctional ligase/repressor BirA"
"DsuE09_00857" 4774 "s59E13H2" 0 "asparagine synthetase A"

"DsuE09_00857" 4774 "s59E25H2" 0 "asparagine synthetase A"
"DsuE09_00857" 4774 "s59E5H2" 0 "asparagine synthetase A"
"DsuE09_00857" 4774 "s63E6H2" 0 "asparagine synthetase A"
"DsuE09_00858" 4775 "s59E13H2" 0 "putative glutamine synthetase 2"
"DsuE09_00858" 4775 "s59E25H2" 0 "putative glutamine synthetase 2"
"DsuE09_00858" 4775 "s59E5H2" 0 "putative glutamine synthetase 2"
"DsuE09_00858" 4775 "s63E6H2" 0 "putative glutamine synthetase 2"
"DsuE09_00859" 4776 "s59E13H2" 0 "Glutamine-dependent NAD(+) synthetase"
"DsuE09_00859" 4776 "s59E25H2" 0 "Glutamine-dependent NAD(+) synthetase"
"DsuE09_00859" 4776 "s59E5H2" 0 "Glutamine-dependent NAD(+) synthetase"
"DsuE09_00859" 4776 "s63E6H2" 0 "Glutamine-dependent NAD(+) synthetase"
"DsuE09_00860" 4777 "s59E13H2" 0 "Amidophosphoribosyltransferase precursor"
"DsuE09_00860" 4777 "s59E25H2" 0 "Amidophosphoribosyltransferase precursor"
"DsuE09_00860" 4777 "s59E5H2" 0.005473789 "Amidophosphoribosyltransferase precursor"
"DsuE09_00860" 4777 "s63E6H2" 0 "Amidophosphoribosyltransferase precursor"
"DsuE09_00861" 4778 "s59E13H2" 0 "Carbamoyl-phosphate synthase large chain"
"DsuE09_00861" 4778 "s59E25H2" 0 "Carbamoyl-phosphate synthase large chain"
"DsuE09_00861" 4778 "s59E5H2" 0 "Carbamoyl-phosphate synthase large chain"
"DsuE09_00861" 4778 "s63E6H2" 0 "Carbamoyl-phosphate synthase large chain"
"DsuE09_00863" 4780 "s59E13H2" 0 "hypothetical protein"
"DsuE09_00863" 4780 "s59E25H2" 0 "hypothetical protein"
"DsuE09_00863" 4780 "s59E5H2" 0 "hypothetical protein"
"DsuE09_00863" 4780 "s63E6H2" 0 "hypothetical protein"
"DsuE09_00864" 4781 "s59E13H2" 0 "hypothetical protein"
"DsuE09_00864" 4781 "s59E25H2" 0 "hypothetical protein"
"DsuE09_00864" 4781 "s59E5H2" 0 "hypothetical protein"
"DsuE09_00864" 4781 "s63E6H2" 0 "hypothetical protein"
"DsuE09_00865" 4782 "s59E13H2" 0 "phosphoenolpyruvate synthase"
"DsuE09_00865" 4782 "s59E25H2" 0 "phosphoenolpyruvate synthase"
"DsuE09_00865" 4782 "s59E5H2" 0 "phosphoenolpyruvate synthase"
"DsuE09_00865" 4782 "s63E6H2" 0 "phosphoenolpyruvate synthase"
"DsuE09_00866" 4783 "s59E13H2" 0 "Glutamate dehydrogenase"
"DsuE09_00866" 4783 "s59E25H2" 0 "Glutamate dehydrogenase"
"DsuE09_00866" 4783 "s59E5H2" 0 "Glutamate dehydrogenase"
"DsuE09_00866" 4783 "s63E6H2" 0 "Glutamate dehydrogenase"
"DsuE09_00867" 4784 "s59E13H2" 0 "putative transcriptional regulatory protein pdtaR"
"DsuE09_00867" 4784 "s59E25H2" 0 "putative transcriptional regulatory protein pdtaR"
"DsuE09_00867" 4784 "s59E5H2" 0 "putative transcriptional regulatory protein pdtaR"
"DsuE09_00867" 4784 "s63E6H2" 0 "putative transcriptional regulatory protein pdtaR"
"DsuE09_00868" 4785 "s59E13H2" 0 "Glutamine--fructose-6-phosphate aminotransferase [isomerizing]"
"DsuE09_00868" 4785 "s59E25H2" 0 "Glutamine--fructose-6-phosphate aminotransferase [isomerizing]"
"DsuE09_00868" 4785 "s59E5H2" 0 "Glutamine--fructose-6-phosphate aminotransferase [isomerizing]"
"DsuE09_00868" 4785 "s63E6H2" 0 "Glutamine--fructose-6-phosphate aminotransferase [isomerizing]"
"DsuE09_00869" 4786 "s59E13H2" 0 "Glucosamine-6-phosphate deaminase 1"
"DsuE09_00869" 4786 "s59E25H2" 0 "Glucosamine-6-phosphate deaminase 1"
"DsuE09_00869" 4786 "s59E5H2" 0 "Glucosamine-6-phosphate deaminase 1"
"DsuE09_00869" 4786 "s63E6H2" 0 "Glucosamine-6-phosphate deaminase 1"

"DsuE09_00870" 4787 "s59E13H2" 0 "Phosphoglucosamine mutase"
"DsuE09_00870" 4787 "s59E25H2" 0 "Phosphoglucosamine mutase"
"DsuE09_00870" 4787 "s59E5H2" 0 "Phosphoglucosamine mutase"
"DsuE09_00870" 4787 "s63E6H2" 0 "Phosphoglucosamine mutase"
"DsuE09_00872" 4789 "s59E13H2" 0 "hypothetical protein"
"DsuE09_00872" 4789 "s59E25H2" 0 "hypothetical protein"
"DsuE09_00872" 4789 "s59E5H2" 0 "hypothetical protein"
"DsuE09_00872" 4789 "s63E6H2" 0 "hypothetical protein"
"DsuE09_00873" 4790 "s59E13H2" 0 "3-deoxy-D-manno-octulosonic acid transferase"
"DsuE09_00873" 4790 "s59E25H2" 0 "3-deoxy-D-manno-octulosonic acid transferase"
"DsuE09_00873" 4790 "s59E5H2" 0 "3-deoxy-D-manno-octulosonic acid transferase"
"DsuE09_00873" 4790 "s63E6H2" 0 "3-deoxy-D-manno-octulosonic acid transferase"
"DsuE09_00875" 4792 "s59E13H2" 0 "GMP synthase [glutamine-hydrolyzing]"
"DsuE09_00875" 4792 "s59E25H2" 0 "GMP synthase [glutamine-hydrolyzing]"
"DsuE09_00875" 4792 "s59E5H2" 0 "GMP synthase [glutamine-hydrolyzing]"
"DsuE09_00875" 4792 "s63E6H2" 0 "GMP synthase [glutamine-hydrolyzing]"
"DsuE09_00876" 4793 "s59E13H2" 0 "GMP synthase [glutamine-hydrolyzing]"
"DsuE09_00876" 4793 "s59E25H2" 0 "GMP synthase [glutamine-hydrolyzing]"
"DsuE09_00876" 4793 "s59E5H2" 0 "GMP synthase [glutamine-hydrolyzing]"
"DsuE09_00876" 4793 "s63E6H2" 0 "GMP synthase [glutamine-hydrolyzing]"
"DsuE09_00877" 4794 "s59E13H2" 0 "hypothetical protein"
"DsuE09_00877" 4794 "s59E25H2" 0 "hypothetical protein"
"DsuE09_00877" 4794 "s59E5H2" 0 "hypothetical protein"
"DsuE09_00877" 4794 "s63E6H2" 0 "hypothetical protein"
"DsuE09_00878" 4795 "s59E13H2" 0 "Long-chain-fatty-acid--CoA ligase"
"DsuE09_00878" 4795 "s59E25H2" 0 "Long-chain-fatty-acid--CoA ligase"
"DsuE09_00878" 4795 "s59E5H2" 0 "Long-chain-fatty-acid--CoA ligase"
"DsuE09_00878" 4795 "s63E6H2" 0 "Long-chain-fatty-acid--CoA ligase"
"DsuE09_00879" 4796 "s59E13H2" 0 "3-keto-5-aminohexanoate cleavage enzyme"
"DsuE09_00879" 4796 "s59E25H2" 0 "3-keto-5-aminohexanoate cleavage enzyme"
"DsuE09_00879" 4796 "s59E5H2" 0 "3-keto-5-aminohexanoate cleavage enzyme"
"DsuE09_00879" 4796 "s63E6H2" 0 "3-keto-5-aminohexanoate cleavage enzyme"
"DsuE09_00880" 4797 "s59E13H2" 0 "hypothetical protein"
"DsuE09_00880" 4797 "s59E25H2" 0 "hypothetical protein"
"DsuE09_00880" 4797 "s59E5H2" 0 "hypothetical protein"
"DsuE09_00880" 4797 "s63E6H2" 0 "hypothetical protein"
"DsuE09_00881" 4798 "s59E13H2" 0 "4-hydroxybutyryl-CoA dehydratase/vinylacetyl-CoA-Delta-isomerase"
"DsuE09_00881" 4798 "s59E25H2" 0 "4-hydroxybutyryl-CoA dehydratase/vinylacetyl-CoA-Delta-isomerase"
"DsuE09_00881" 4798 "s59E5H2" 0 "4-hydroxybutyryl-CoA dehydratase/vinylacetyl-CoA-Delta-isomerase"
"DsuE09_00881" 4798 "s63E6H2" 0 "4-hydroxybutyryl-CoA dehydratase/vinylacetyl-CoA-Delta-isomerase"
"DsuE09_00883" 4800 "s59E13H2" 0 "NMT1/THI5 like protein"
"DsuE09_00883" 4800 "s59E25H2" 0 "NMT1/THI5 like protein"
"DsuE09_00883" 4800 "s59E5H2" 0 "NMT1/THI5 like protein"
"DsuE09_00883" 4800 "s63E6H2" 0 "NMT1/THI5 like protein"
"DsuE09_00885" 4802 "s59E13H2" 0 "Sialic acid TRAP transporter permease protein SiaT"
"DsuE09_00885" 4802 "s59E25H2" 0 "Sialic acid TRAP transporter permease protein SiaT"
"DsuE09_00885" 4802 "s59E5H2" 0 "Sialic acid TRAP transporter permease protein SiaT"

"DsuE09_00885" 4802 "s63E6H2" 0 "Sialic acid TRAP transporter permease protein SiaT"
"DsuE09_00886" 4803 "s59E13H2" 0 "Indolepyruvate oxidoreductase subunit IorA"
"DsuE09_00886" 4803 "s59E25H2" 0 "Indolepyruvate oxidoreductase subunit IorA"
"DsuE09_00886" 4803 "s59E5H2" 0 "Indolepyruvate oxidoreductase subunit IorA"
"DsuE09_00886" 4803 "s63E6H2" 0 "Indolepyruvate oxidoreductase subunit IorA"
"DsuE09_00887" 4804 "s59E13H2" 0 "Indolepyruvate oxidoreductase subunit IorB"
"DsuE09_00887" 4804 "s59E25H2" 0 "Indolepyruvate oxidoreductase subunit IorB"
"DsuE09_00887" 4804 "s59E5H2" 0 "Indolepyruvate oxidoreductase subunit IorB"
"DsuE09_00887" 4804 "s63E6H2" 0 "Indolepyruvate oxidoreductase subunit IorB"
"DsuE09_00888" 4805 "s59E13H2" 0 "Lactate-binding periplasmic protein precursor"
"DsuE09_00888" 4805 "s59E25H2" 0 "Lactate-binding periplasmic protein precursor"
"DsuE09_00888" 4805 "s59E5H2" 0 "Lactate-binding periplasmic protein precursor"
"DsuE09_00888" 4805 "s63E6H2" 0 "Lactate-binding periplasmic protein precursor"
"DsuE09_00889" 4806 "s59E13H2" 0 "Tripartite ATP-independent periplasmic transporters, DctQ component"
"DsuE09_00889" 4806 "s59E25H2" 0 "Tripartite ATP-independent periplasmic transporters, DctQ component"
"DsuE09_00889" 4806 "s59E5H2" 0 "Tripartite ATP-independent periplasmic transporters, DctQ component"
"DsuE09_00889" 4806 "s63E6H2" 0 "Tripartite ATP-independent periplasmic transporters, DctQ component"
"DsuE09_00890" 4807 "s59E13H2" 0 "Sialic acid TRAP transporter permease protein SiaT"
"DsuE09_00890" 4807 "s59E25H2" 0 "Sialic acid TRAP transporter permease protein SiaT"
"DsuE09_00890" 4807 "s59E5H2" 0 "Sialic acid TRAP transporter permease protein SiaT"
"DsuE09_00890" 4807 "s63E6H2" 0 "Sialic acid TRAP transporter permease protein SiaT"
"DsuE09_00891" 4808 "s59E13H2" 0 "putative 3-hydroxybutyryl-CoA dehydrogenase"
"DsuE09_00891" 4808 "s59E25H2" 0 "putative 3-hydroxybutyryl-CoA dehydrogenase"
"DsuE09_00891" 4808 "s59E5H2" 0 "putative 3-hydroxybutyryl-CoA dehydrogenase"
"DsuE09_00891" 4808 "s63E6H2" 0 "putative 3-hydroxybutyryl-CoA dehydrogenase"
"DsuE09_00893" 4810 "s59E13H2" 0 "hypothetical protein"
"DsuE09_00893" 4810 "s59E25H2" 0 "hypothetical protein"
"DsuE09_00893" 4810 "s59E5H2" 0 "hypothetical protein"
"DsuE09_00893" 4810 "s63E6H2" 0 "hypothetical protein"
"DsuE09_00894" 4811 "s59E13H2" 0 "Putative ketoacyl reductase"
"DsuE09_00894" 4811 "s59E25H2" 0 "Putative ketoacyl reductase"
"DsuE09_00894" 4811 "s59E5H2" 0 "Putative ketoacyl reductase"
"DsuE09_00894" 4811 "s63E6H2" 0 "Putative ketoacyl reductase"
"DsuE09_00896" 4813 "s59E13H2" 0 "putative periplasmic serine endoprotease DegP-like precursor"
"DsuE09_00896" 4813 "s59E25H2" 0 "putative periplasmic serine endoprotease DegP-like precursor"
"DsuE09_00896" 4813 "s59E5H2" 0 "putative periplasmic serine endoprotease DegP-like precursor"
"DsuE09_00896" 4813 "s63E6H2" 0 "putative periplasmic serine endoprotease DegP-like precursor"
"DsuE09_00897" 4814 "s59E13H2" 0 "Chaperone protein ClpB 1"
"DsuE09_00897" 4814 "s59E25H2" 0 "Chaperone protein ClpB 1"
"DsuE09_00897" 4814 "s59E5H2" 0 "Chaperone protein ClpB 1"
"DsuE09_00897" 4814 "s63E6H2" 0 "Chaperone protein ClpB 1"
"DsuE09_00898" 4815 "s59E13H2" 0 "hypothetical protein"
"DsuE09_00898" 4815 "s59E25H2" 0 "hypothetical protein"
"DsuE09_00898" 4815 "s59E5H2" 0.1865091 "hypothetical protein"
"DsuE09_00898" 4815 "s63E6H2" 0 "hypothetical protein"
"DsuE09_00900" 4817 "s59E13H2" 0 "hypothetical protein"
"DsuE09_00900" 4817 "s59E25H2" 0 "hypothetical protein"

"DsuE09_00900" 4817 "s59E5H2" 0 "hypothetical protein"
"DsuE09_00900" 4817 "s63E6H2" 0 "hypothetical protein"
"DsuE09_00902" 4819 "s59E13H2" 0 "hypothetical protein"
"DsuE09_00902" 4819 "s59E25H2" 0 "hypothetical protein"
"DsuE09_00902" 4819 "s59E5H2" 0 "hypothetical protein"
"DsuE09_00902" 4819 "s63E6H2" 0 "hypothetical protein"
"DsuE09_00903" 4820 "s59E13H2" 0 "Foldase protein PrsA precursor"
"DsuE09_00903" 4820 "s59E25H2" 0 "Foldase protein PrsA precursor"
"DsuE09_00903" 4820 "s59E5H2" 0 "Foldase protein PrsA precursor"
"DsuE09_00903" 4820 "s63E6H2" 0 "Foldase protein PrsA precursor"
"DsuE09_00904" 4821 "s59E13H2" 0 "Purine catabolism protein PucG"
"DsuE09_00904" 4821 "s59E25H2" 0 "Purine catabolism protein PucG"
"DsuE09_00904" 4821 "s59E5H2" 0 "Purine catabolism protein PucG"
"DsuE09_00904" 4821 "s63E6H2" 0 "Purine catabolism protein PucG"
"DsuE09_00905" 4822 "s59E13H2" 0 "Glycogen synthase"
"DsuE09_00905" 4822 "s59E25H2" 0 "Glycogen synthase"
"DsuE09_00905" 4822 "s59E5H2" 0 "Glycogen synthase"
"DsuE09_00905" 4822 "s63E6H2" 0 "Glycogen synthase"
"DsuE09_00906" 4823 "s59E13H2" 0 "Demethylrebeccamycin-D-glucose O-methyltransferase"
"DsuE09_00906" 4823 "s59E25H2" 0 "Demethylrebeccamycin-D-glucose O-methyltransferase"
"DsuE09_00906" 4823 "s59E5H2" 0 "Demethylrebeccamycin-D-glucose O-methyltransferase"
"DsuE09_00906" 4823 "s63E6H2" 0 "Demethylrebeccamycin-D-glucose O-methyltransferase"
"DsuE09_00907" 4824 "s59E13H2" 0 "hypothetical protein"
"DsuE09_00907" 4824 "s59E25H2" 0 "hypothetical protein"
"DsuE09_00907" 4824 "s59E5H2" 0 "hypothetical protein"
"DsuE09_00907" 4824 "s63E6H2" 0 "hypothetical protein"
"DsuE09_00908" 4825 "s59E13H2" 0 "Bifunctional purine biosynthesis protein PurH"
"DsuE09_00908" 4825 "s59E25H2" 0 "Bifunctional purine biosynthesis protein PurH"
"DsuE09_00908" 4825 "s59E5H2" 0 "Bifunctional purine biosynthesis protein PurH"
"DsuE09_00908" 4825 "s63E6H2" 0 "Bifunctional purine biosynthesis protein PurH"
"DsuE09_00909" 4826 "s59E13H2" 0 "Hydrogenase transcriptional regulatory protein hupR1"
"DsuE09_00909" 4826 "s59E25H2" 0 "Hydrogenase transcriptional regulatory protein hupR1"
"DsuE09_00909" 4826 "s59E5H2" 0 "Hydrogenase transcriptional regulatory protein hupR1"
"DsuE09_00909" 4826 "s63E6H2" 0 "Hydrogenase transcriptional regulatory protein hupR1"
"DsuE09_00910" 4827 "s59E13H2" 0 "Transcriptional regulatory protein ZraR"
"DsuE09_00910" 4827 "s59E25H2" 0 "Transcriptional regulatory protein ZraR"
"DsuE09_00910" 4827 "s59E5H2" 0 "Transcriptional regulatory protein ZraR"
"DsuE09_00910" 4827 "s63E6H2" 0 "Transcriptional regulatory protein ZraR"
"DsuE09_00911" 4828 "s59E13H2" 0 "Sensor protein ZraS"
"DsuE09_00911" 4828 "s59E25H2" 0 "Sensor protein ZraS"
"DsuE09_00911" 4828 "s59E5H2" 0 "Sensor protein ZraS"
"DsuE09_00911" 4828 "s63E6H2" 0 "Sensor protein ZraS"
"DsuE09_00913" 4830 "s59E13H2" 0 "GIY-YIG nuclease superfamily protein"
"DsuE09_00913" 4830 "s59E25H2" 0 "GIY-YIG nuclease superfamily protein"
"DsuE09_00913" 4830 "s59E5H2" 0 "GIY-YIG nuclease superfamily protein"
"DsuE09_00913" 4830 "s63E6H2" 0 "GIY-YIG nuclease superfamily protein"
"DsuE09_00914" 4831 "s59E13H2" 0 "Sensor protein FixL"

"DsuE09_00914" 4831 "s59E25H2" 0 "Sensor protein FixL"
"DsuE09_00914" 4831 "s59E5H2" 0 "Sensor protein FixL"
"DsuE09_00914" 4831 "s63E6H2" 0 "Sensor protein FixL"
"DsuE09_00915" 4832 "s59E13H2" 0 "metal-dependent hydrolase"
"DsuE09_00915" 4832 "s59E25H2" 0 "metal-dependent hydrolase"
"DsuE09_00915" 4832 "s59E5H2" 0 "metal-dependent hydrolase"
"DsuE09_00915" 4832 "s63E6H2" 0 "metal-dependent hydrolase"
"DsuE09_00916" 4833 "s59E13H2" 0 "Sensor protein kinase WalK"
"DsuE09_00916" 4833 "s59E25H2" 0 "Sensor protein kinase WalK"
"DsuE09_00916" 4833 "s59E5H2" 0 "Sensor protein kinase WalK"
"DsuE09_00916" 4833 "s63E6H2" 5.366949e-05 "Sensor protein kinase WalK"
"DsuE09_00917" 4834 "s59E13H2" 0 "GIY-YIG catalytic domain protein"
"DsuE09_00917" 4834 "s59E25H2" 0 "GIY-YIG catalytic domain protein"
"DsuE09_00917" 4834 "s59E5H2" 0 "GIY-YIG catalytic domain protein"
"DsuE09_00917" 4834 "s63E6H2" 0 "GIY-YIG catalytic domain protein"
"DsuE09_00918" 4835 "s59E13H2" 0 "hypothetical protein"
"DsuE09_00918" 4835 "s59E25H2" 0 "hypothetical protein"
"DsuE09_00918" 4835 "s59E5H2" 0 "hypothetical protein"
"DsuE09_00918" 4835 "s63E6H2" 0 "hypothetical protein"
"DsuE09_00919" 4836 "s59E13H2" 0 "Chemotaxis protein CheA"
"DsuE09_00919" 4836 "s59E25H2" 0 "Chemotaxis protein CheA"
"DsuE09_00919" 4836 "s59E5H2" 0 "Chemotaxis protein CheA"
"DsuE09_00919" 4836 "s63E6H2" 0 "Chemotaxis protein CheA"
"DsuE09_00920" 4837 "s59E13H2" 0 "CheW-like domain protein"
"DsuE09_00920" 4837 "s59E25H2" 0 "CheW-like domain protein"
"DsuE09_00920" 4837 "s59E5H2" 0 "CheW-like domain protein"
"DsuE09_00920" 4837 "s63E6H2" 0 "CheW-like domain protein"
"DsuE09_00921" 4838 "s59E13H2" 0 "Methyl-accepting chemotaxis protein McpB"
"DsuE09_00921" 4838 "s59E25H2" 0 "Methyl-accepting chemotaxis protein McpB"
"DsuE09_00921" 4838 "s59E5H2" 0 "Methyl-accepting chemotaxis protein McpB"
"DsuE09_00921" 4838 "s63E6H2" 0 "Methyl-accepting chemotaxis protein McpB"
"DsuE09_00922" 4839 "s59E13H2" 0 "Sensor histidine kinase TmoS"
"DsuE09_00922" 4839 "s59E25H2" 0 "Sensor histidine kinase TmoS"
"DsuE09_00922" 4839 "s59E5H2" 0 "Sensor histidine kinase TmoS"
"DsuE09_00922" 4839 "s63E6H2" 0 "Sensor histidine kinase TmoS"
"DsuE09_00923" 4840 "s59E13H2" 0 "hypothetical protein"
"DsuE09_00923" 4840 "s59E25H2" 0 "hypothetical protein"
"DsuE09_00923" 4840 "s59E5H2" 0 "hypothetical protein"
"DsuE09_00923" 4840 "s63E6H2" 0 "hypothetical protein"
"DsuE09_00924" 4841 "s59E13H2" 0 "Type II secretion system protein E"
"DsuE09_00924" 4841 "s59E25H2" 0 "Type II secretion system protein E"
"DsuE09_00924" 4841 "s59E5H2" 0 "Type II secretion system protein E"
"DsuE09_00924" 4841 "s63E6H2" 0 "Type II secretion system protein E"
"DsuE09_00926" 4843 "s59E13H2" 0 "Transcriptional regulatory protein ZraR"
"DsuE09_00926" 4843 "s59E25H2" 0 "Transcriptional regulatory protein ZraR"
"DsuE09_00926" 4843 "s59E5H2" 0 "Transcriptional regulatory protein ZraR"
"DsuE09_00926" 4843 "s63E6H2" 0 "Transcriptional regulatory protein ZraR"

"DsuE09_00929" 4846 "s59E13H2" 0 "Gas vesicle structural protein 1"
"DsuE09_00929" 4846 "s59E25H2" 0 "Gas vesicle structural protein 1"
"DsuE09_00929" 4846 "s59E5H2" 0 "Gas vesicle structural protein 1"
"DsuE09_00929" 4846 "s63E6H2" 0 "Gas vesicle structural protein 1"
"DsuE09_00931" 4848 "s59E13H2" 0 "Gas vesicle protein K"
"DsuE09_00931" 4848 "s59E25H2" 0 "Gas vesicle protein K"
"DsuE09_00931" 4848 "s59E5H2" 0 "Gas vesicle protein K"
"DsuE09_00931" 4848 "s63E6H2" 0 "Gas vesicle protein K"
"DsuE09_00932" 4849 "s59E13H2" 0 "Gas vesicle synthesis protein GvpL/GvpF"
"DsuE09_00932" 4849 "s59E25H2" 0 "Gas vesicle synthesis protein GvpL/GvpF"
"DsuE09_00932" 4849 "s59E5H2" 0 "Gas vesicle synthesis protein GvpL/GvpF"
"DsuE09_00932" 4849 "s63E6H2" 0 "Gas vesicle synthesis protein GvpL/GvpF"
"DsuE09_00933" 4850 "s59E13H2" 0 "Gas vesicle synthesis protein GvpO"
"DsuE09_00933" 4850 "s59E25H2" 0 "Gas vesicle synthesis protein GvpO"
"DsuE09_00933" 4850 "s59E5H2" 0 "Gas vesicle synthesis protein GvpO"
"DsuE09_00933" 4850 "s63E6H2" 0 "Gas vesicle synthesis protein GvpO"
"DsuE09_00934" 4851 "s59E13H2" 0 "Putative arsenical pump-driving ATPase"
"DsuE09_00934" 4851 "s59E25H2" 0 "Putative arsenical pump-driving ATPase"
"DsuE09_00934" 4851 "s59E5H2" 0.02885376 "Putative arsenical pump-driving ATPase"
"DsuE09_00934" 4851 "s63E6H2" 0.005265016 "Putative arsenical pump-driving ATPase"
"DsuE09_00935" 4852 "s59E13H2" 0 "Gas vesicle protein G"
"DsuE09_00935" 4852 "s59E25H2" 0 "Gas vesicle protein G"
"DsuE09_00935" 4852 "s59E5H2" 0 "Gas vesicle protein G"
"DsuE09_00935" 4852 "s63E6H2" 0 "Gas vesicle protein G"
"DsuE09_00936" 4853 "s59E13H2" 0 "Gas vesicle synthesis protein GvpL/GvpF"
"DsuE09_00936" 4853 "s59E25H2" 0 "Gas vesicle synthesis protein GvpL/GvpF"
"DsuE09_00936" 4853 "s59E5H2" 0 "Gas vesicle synthesis protein GvpL/GvpF"
"DsuE09_00936" 4853 "s63E6H2" 0 "Gas vesicle synthesis protein GvpL/GvpF"
"DsuE09_00937" 4854 "s59E13H2" 0 "Gas vesicle synthesis protein GvpL/GvpF"
"DsuE09_00937" 4854 "s59E25H2" 0 "Gas vesicle synthesis protein GvpL/GvpF"
"DsuE09_00937" 4854 "s59E5H2" 0 "Gas vesicle synthesis protein GvpL/GvpF"
"DsuE09_00937" 4854 "s63E6H2" 0 "Gas vesicle synthesis protein GvpL/GvpF"
"DsuE09_00938" 4855 "s59E13H2" 0 "chaperone protein DnaJ"
"DsuE09_00938" 4855 "s59E25H2" 0 "chaperone protein DnaJ"
"DsuE09_00938" 4855 "s59E5H2" 0 "chaperone protein DnaJ"
"DsuE09_00938" 4855 "s63E6H2" 0 "chaperone protein DnaJ"
"DsuE09_00940" 4857 "s59E13H2" 0 "VCP-like ATPase"
"DsuE09_00940" 4857 "s59E25H2" 0 "VCP-like ATPase"
"DsuE09_00940" 4857 "s59E5H2" 0 "VCP-like ATPase"
"DsuE09_00940" 4857 "s63E6H2" 0 "VCP-like ATPase"
"DsuE09_00941" 4858 "s59E13H2" 0 "Hsp20/alpha crystallin family protein"
"DsuE09_00941" 4858 "s59E25H2" 0 "Hsp20/alpha crystallin family protein"
"DsuE09_00941" 4858 "s59E5H2" 0 "Hsp20/alpha crystallin family protein"
"DsuE09_00941" 4858 "s63E6H2" 0 "Hsp20/alpha crystallin family protein"
"DsuE09_00942" 4859 "s59E13H2" 0 "hypothetical protein"
"DsuE09_00942" 4859 "s59E25H2" 0 "hypothetical protein"
"DsuE09_00942" 4859 "s59E5H2" 0 "hypothetical protein"

"DsuE09_00942" 4859 "s63E6H2" 0 "hypothetical protein"
"DsuE09_00944" 4861 "s59E13H2" 0 "chaperone protein DnaJ"
"DsuE09_00944" 4861 "s59E25H2" 0 "chaperone protein DnaJ"
"DsuE09_00944" 4861 "s59E5H2" 0 "chaperone protein DnaJ"
"DsuE09_00944" 4861 "s63E6H2" 0 "chaperone protein DnaJ"
"DsuE09_00945" 4862 "s59E13H2" 0 "hypothetical protein"
"DsuE09_00945" 4862 "s59E25H2" 0 "hypothetical protein"
"DsuE09_00945" 4862 "s59E5H2" 0 "hypothetical protein"
"DsuE09_00945" 4862 "s63E6H2" 0 "hypothetical protein"
"DsuE09_00946" 4863 "s59E13H2" 0 "hypothetical protein"
"DsuE09_00946" 4863 "s59E25H2" 0 "hypothetical protein"
"DsuE09_00946" 4863 "s59E5H2" 0 "hypothetical protein"
"DsuE09_00946" 4863 "s63E6H2" 0 "hypothetical protein"
"DsuE09_00947" 4864 "s59E13H2" 0 "Denitrification regulatory protein NirQ"
"DsuE09_00947" 4864 "s59E25H2" 0 "Denitrification regulatory protein NirQ"
"DsuE09_00947" 4864 "s59E5H2" 0 "Denitrification regulatory protein NirQ"
"DsuE09_00947" 4864 "s63E6H2" 0 "Denitrification regulatory protein NirQ"
"DsuE09_00948" 4865 "s59E13H2" 0 "hypothetical protein"
"DsuE09_00948" 4865 "s59E25H2" 0 "hypothetical protein"
"DsuE09_00948" 4865 "s59E5H2" 0 "hypothetical protein"
"DsuE09_00948" 4865 "s63E6H2" 0 "hypothetical protein"
"DsuE09_00949" 4866 "s59E13H2" 0 "chaperone protein DnaJ"
"DsuE09_00949" 4866 "s59E25H2" 0 "chaperone protein DnaJ"
"DsuE09_00949" 4866 "s59E5H2" 0 "chaperone protein DnaJ"
"DsuE09_00949" 4866 "s63E6H2" 0 "chaperone protein DnaJ"
"DsuE09_00950" 4867 "s59E13H2" 0 "hypothetical protein"
"DsuE09_00950" 4867 "s59E25H2" 0 "hypothetical protein"
"DsuE09_00950" 4867 "s59E5H2" 0 "hypothetical protein"
"DsuE09_00950" 4867 "s63E6H2" 0 "hypothetical protein"
"DsuE09_00951" 4868 "s59E13H2" 0 "hypothetical protein"
"DsuE09_00951" 4868 "s59E25H2" 0 "hypothetical protein"
"DsuE09_00951" 4868 "s59E5H2" 0 "hypothetical protein"
"DsuE09_00951" 4868 "s63E6H2" 0 "hypothetical protein"
"DsuE09_00952" 4869 "s59E13H2" 0 "hypothetical protein"
"DsuE09_00952" 4869 "s59E25H2" 0 "hypothetical protein"
"DsuE09_00952" 4869 "s59E5H2" 0 "hypothetical protein"
"DsuE09_00952" 4869 "s63E6H2" 0 "hypothetical protein"
"DsuE09_00953" 4870 "s59E13H2" 0 "Gas vesicle structural protein"
"DsuE09_00953" 4870 "s59E25H2" 0 "Gas vesicle structural protein"
"DsuE09_00953" 4870 "s59E5H2" 0 "Gas vesicle structural protein"
"DsuE09_00953" 4870 "s63E6H2" 0 "Gas vesicle structural protein"
"DsuE09_00954" 4871 "s59E13H2" 0 "Response regulator ArlR"
"DsuE09_00954" 4871 "s59E25H2" 0 "Response regulator ArlR"
"DsuE09_00954" 4871 "s59E5H2" 0 "Response regulator ArlR"
"DsuE09_00954" 4871 "s63E6H2" 0 "Response regulator ArlR"
"DsuE09_00955" 4872 "s59E13H2" 0 "Transcriptional regulatory protein ZraR"
"DsuE09_00955" 4872 "s59E25H2" 0 "Transcriptional regulatory protein ZraR"

"DsuE09_00955" 4872 "s59E5H2" 0 "Transcriptional regulatory protein ZraR"
"DsuE09_00955" 4872 "s63E6H2" 0 "Transcriptional regulatory protein ZraR"
"DsuE09_00956" 4873 "s59E13H2" 0 "Sporulation initiation phosphotransferase F"
"DsuE09_00956" 4873 "s59E25H2" 0 "Sporulation initiation phosphotransferase F"
"DsuE09_00956" 4873 "s59E5H2" 0 "Sporulation initiation phosphotransferase F"
"DsuE09_00956" 4873 "s63E6H2" 0 "Sporulation initiation phosphotransferase F"
"DsuE09_00957" 4874 "s59E13H2" 0 "DNA polymerase III subunits gamma and tau"
"DsuE09_00957" 4874 "s59E25H2" 0 "DNA polymerase III subunits gamma and tau"
"DsuE09_00957" 4874 "s59E5H2" 0 "DNA polymerase III subunits gamma and tau"
"DsuE09_00957" 4874 "s63E6H2" 0.00200045 "DNA polymerase III subunits gamma and tau"
"DsuE09_00958" 4875 "s59E13H2" 0 "Nucleoid-associated protein"
"DsuE09_00958" 4875 "s59E25H2" 0 "Nucleoid-associated protein"
"DsuE09_00958" 4875 "s59E5H2" 0 "Nucleoid-associated protein"
"DsuE09_00958" 4875 "s63E6H2" 0.00560463 "Nucleoid-associated protein"
"DsuE09_00959" 4876 "s59E13H2" 0 "hypothetical protein"
"DsuE09_00959" 4876 "s59E25H2" 0 "hypothetical protein"
"DsuE09_00959" 4876 "s59E5H2" 0 "hypothetical protein"
"DsuE09_00959" 4876 "s63E6H2" 0 "hypothetical protein"
"DsuE09_00960" 4877 "s59E13H2" 0 "Antilisterial bacteriocin subtilosin biosynthesis protein AlbA"
"DsuE09_00960" 4877 "s59E25H2" 0 "Antilisterial bacteriocin subtilosin biosynthesis protein AlbA"
"DsuE09_00960" 4877 "s59E5H2" 0 "Antilisterial bacteriocin subtilosin biosynthesis protein AlbA"
"DsuE09_00960" 4877 "s63E6H2" 0 "Antilisterial bacteriocin subtilosin biosynthesis protein AlbA"
"DsuE09_00961" 4878 "s59E13H2" 0 "hypothetical protein"
"DsuE09_00961" 4878 "s59E25H2" 0 "hypothetical protein"
"DsuE09_00961" 4878 "s59E5H2" 0 "hypothetical protein"
"DsuE09_00961" 4878 "s63E6H2" 0 "hypothetical protein"
"DsuE09_00963" 4880 "s59E13H2" 0 "putative methyltransferase"
"DsuE09_00963" 4880 "s59E25H2" 0 "putative methyltransferase"
"DsuE09_00963" 4880 "s59E5H2" 0 "putative methyltransferase"
"DsuE09_00963" 4880 "s63E6H2" 0 "putative methyltransferase"
"DsuE09_00964" 4881 "s59E13H2" 0 "Beta-lactamase superfamily domain protein"
"DsuE09_00964" 4881 "s59E25H2" 0 "Beta-lactamase superfamily domain protein"
"DsuE09_00964" 4881 "s59E5H2" 0 "Beta-lactamase superfamily domain protein"
"DsuE09_00964" 4881 "s63E6H2" 0 "Beta-lactamase superfamily domain protein"
"DsuE09_00965" 4882 "s59E13H2" 0 "hypothetical protein"
"DsuE09_00965" 4882 "s59E25H2" 0 "hypothetical protein"
"DsuE09_00965" 4882 "s59E5H2" 0 "hypothetical protein"
"DsuE09_00965" 4882 "s63E6H2" 0 "hypothetical protein"
"DsuE09_00966" 4883 "s59E13H2" 0 "Asparagine synthetase [glutamine-hydrolyzing] 1"
"DsuE09_00966" 4883 "s59E25H2" 0 "Asparagine synthetase [glutamine-hydrolyzing] 1"
"DsuE09_00966" 4883 "s59E5H2" 0 "Asparagine synthetase [glutamine-hydrolyzing] 1"
"DsuE09_00966" 4883 "s63E6H2" 0 "Asparagine synthetase [glutamine-hydrolyzing] 1"
"DsuE09_00967" 4884 "s59E13H2" 0 "hypothetical protein"
"DsuE09_00967" 4884 "s59E25H2" 0 "hypothetical protein"
"DsuE09_00967" 4884 "s59E5H2" 0 "hypothetical protein"
"DsuE09_00967" 4884 "s63E6H2" 0 "hypothetical protein"
"DsuE09_00968" 4885 "s59E13H2" 0 "hypothetical protein"

"DsuE09_00968" 4885 "s59E25H2" 0 "hypothetical protein"
"DsuE09_00968" 4885 "s59E5H2" 0 "hypothetical protein"
"DsuE09_00968" 4885 "s63E6H2" 0 "hypothetical protein"
"DsuE09_00971" 4888 "s59E13H2" 0 "membrane-bound lytic murein transglycosylase D"
"DsuE09_00971" 4888 "s59E25H2" 0 "membrane-bound lytic murein transglycosylase D"
"DsuE09_00971" 4888 "s59E5H2" 0 "membrane-bound lytic murein transglycosylase D"
"DsuE09_00971" 4888 "s63E6H2" 0 "membrane-bound lytic murein transglycosylase D"
"DsuE09_00972" 4889 "s59E13H2" 0 "3-keto-5-aminohexanoate cleavage enzyme"
"DsuE09_00972" 4889 "s59E25H2" 0 "3-keto-5-aminohexanoate cleavage enzyme"
"DsuE09_00972" 4889 "s59E5H2" 0 "3-keto-5-aminohexanoate cleavage enzyme"
"DsuE09_00972" 4889 "s63E6H2" 0 "3-keto-5-aminohexanoate cleavage enzyme"
"DsuE09_00974" 4891 "s59E13H2" 0 "Phosphoenolpyruvate synthase"
"DsuE09_00974" 4891 "s59E25H2" 0 "Phosphoenolpyruvate synthase"
"DsuE09_00974" 4891 "s59E5H2" 0 "Phosphoenolpyruvate synthase"
"DsuE09_00974" 4891 "s63E6H2" 0 "Phosphoenolpyruvate synthase"
"DsuE09_00975" 4892 "s59E13H2" 0 "Phosphoenolpyruvate synthase"
"DsuE09_00975" 4892 "s59E25H2" 0 "Phosphoenolpyruvate synthase"
"DsuE09_00975" 4892 "s59E5H2" 0 "Phosphoenolpyruvate synthase"
"DsuE09_00975" 4892 "s63E6H2" 0 "Phosphoenolpyruvate synthase"
"DsuE09_00976" 4893 "s59E13H2" 0 "hypothetical protein"
"DsuE09_00976" 4893 "s59E25H2" 0 "hypothetical protein"
"DsuE09_00976" 4893 "s59E5H2" 0 "hypothetical protein"
"DsuE09_00976" 4893 "s63E6H2" 0 "hypothetical protein"
"DsuE09_00977" 4894 "s59E13H2" 0 "Spore protein SP21"
"DsuE09_00977" 4894 "s59E25H2" 0 "Spore protein SP21"
"DsuE09_00977" 4894 "s59E5H2" 0 "Spore protein SP21"
"DsuE09_00977" 4894 "s63E6H2" 0 "Spore protein SP21"
"DsuE09_00978" 4895 "s59E13H2" 0 "Archaeal Lon protease"
"DsuE09_00978" 4895 "s59E25H2" 0 "Archaeal Lon protease"
"DsuE09_00978" 4895 "s59E5H2" 0 "Archaeal Lon protease"
"DsuE09_00978" 4895 "s63E6H2" 0 "Archaeal Lon protease"
"DsuE09_00979" 4896 "s59E13H2" 0 "hypothetical protein"
"DsuE09_00979" 4896 "s59E25H2" 0 "hypothetical protein"
"DsuE09_00979" 4896 "s59E5H2" 0 "hypothetical protein"
"DsuE09_00979" 4896 "s63E6H2" 0 "hypothetical protein"
"DsuE09_00980" 4897 "s59E13H2" 0 "Integration host factor subunit beta"
"DsuE09_00980" 4897 "s59E25H2" 0 "Integration host factor subunit beta"
"DsuE09_00980" 4897 "s59E5H2" 0 "Integration host factor subunit beta"
"DsuE09_00980" 4897 "s63E6H2" 0 "Integration host factor subunit beta"
"DsuE09_00984" 4901 "s59E13H2" 0 "Dihydroorotate dehydrogenase B (NAD(+)), electron transfer subunit"
"DsuE09_00984" 4901 "s59E25H2" 0 "Dihydroorotate dehydrogenase B (NAD(+)), electron transfer subunit"
"DsuE09_00984" 4901 "s59E5H2" 0 "Dihydroorotate dehydrogenase B (NAD(+)), electron transfer subunit"
"DsuE09_00984" 4901 "s63E6H2" 0 "Dihydroorotate dehydrogenase B (NAD(+)), electron transfer subunit"
"DsuE09_00985" 4902 "s59E13H2" 0 "Dihydroorotate dehydrogenase B (NAD(+)), catalytic subunit"
"DsuE09_00985" 4902 "s59E25H2" 0 "Dihydroorotate dehydrogenase B (NAD(+)), catalytic subunit"
"DsuE09_00985" 4902 "s59E5H2" 0.001234227 "Dihydroorotate dehydrogenase B (NAD(+)), catalytic subunit"
"DsuE09_00985" 4902 "s63E6H2" 0 "Dihydroorotate dehydrogenase B (NAD(+)), catalytic subunit"

"DsuE09_00986" 4903 "s59E13H2" 0 "putative M18 family aminopeptidase 1"
"DsuE09_00986" 4903 "s59E25H2" 0 "putative M18 family aminopeptidase 1"
"DsuE09_00986" 4903 "s59E5H2" 0 "putative M18 family aminopeptidase 1"
"DsuE09_00986" 4903 "s63E6H2" 0 "putative M18 family aminopeptidase 1"
"DsuE09_00987" 4904 "s59E13H2" 0 "Glyoxylate reductase"
"DsuE09_00987" 4904 "s59E25H2" 0 "Glyoxylate reductase"
"DsuE09_00987" 4904 "s59E5H2" 0 "Glyoxylate reductase"
"DsuE09_00987" 4904 "s63E6H2" 0 "Glyoxylate reductase"
"DsuE09_00988" 4905 "s59E13H2" 0 "Archaemetzincin"
"DsuE09_00988" 4905 "s59E25H2" 0 "Archaemetzincin"
"DsuE09_00988" 4905 "s59E5H2" 0 "Archaemetzincin"
"DsuE09_00988" 4905 "s63E6H2" 0 "Archaemetzincin"
"DsuE09_00989" 4906 "s59E13H2" 0 "GYD domain protein"
"DsuE09_00989" 4906 "s59E25H2" 0 "GYD domain protein"
"DsuE09_00989" 4906 "s59E5H2" 0 "GYD domain protein"
"DsuE09_00989" 4906 "s63E6H2" 0 "GYD domain protein"
"DsuE09_00990" 4907 "s59E13H2" 0 "Radical SAM superfamily protein"
"DsuE09_00990" 4907 "s59E25H2" 0 "Radical SAM superfamily protein"
"DsuE09_00990" 4907 "s59E5H2" 0 "Radical SAM superfamily protein"
"DsuE09_00990" 4907 "s63E6H2" 0 "Radical SAM superfamily protein"
"DsuE09_00991" 4908 "s59E13H2" 0 "Cell division protein FtsZ"
"DsuE09_00991" 4908 "s59E25H2" 0 "Cell division protein FtsZ"
"DsuE09_00991" 4908 "s59E5H2" 0 "Cell division protein FtsZ"
"DsuE09_00991" 4908 "s63E6H2" 0 "Cell division protein FtsZ"
"DsuE09_00993" 4910 "s59E13H2" 0 "Cell division protein FtsA"
"DsuE09_00993" 4910 "s59E25H2" 0 "Cell division protein FtsA"
"DsuE09_00993" 4910 "s59E5H2" 0 "Cell division protein FtsA"
"DsuE09_00993" 4910 "s63E6H2" 0 "Cell division protein FtsA"
"DsuE09_00994" 4911 "s59E13H2" 0 "Cell division protein DivIB"
"DsuE09_00994" 4911 "s59E25H2" 0 "Cell division protein DivIB"
"DsuE09_00994" 4911 "s59E5H2" 0 "Cell division protein DivIB"
"DsuE09_00994" 4911 "s63E6H2" 0 "Cell division protein DivIB"
"DsuE09_00995" 4912 "s59E13H2" 0 "UDP-N-acetylenolpyruvoylglucosamine reductase"
"DsuE09_00995" 4912 "s59E25H2" 0 "UDP-N-acetylenolpyruvoylglucosamine reductase"
"DsuE09_00995" 4912 "s59E5H2" 0 "UDP-N-acetylenolpyruvoylglucosamine reductase"
"DsuE09_00995" 4912 "s63E6H2" 0 "UDP-N-acetylenolpyruvoylglucosamine reductase"
"DsuE09_00996" 4913 "s59E13H2" 0 "UDP-N-acetylenolpyruvoylglucosamine reductase"
"DsuE09_00996" 4913 "s59E25H2" 0 "UDP-N-acetylenolpyruvoylglucosamine reductase"
"DsuE09_00996" 4913 "s59E5H2" 0 "UDP-N-acetylenolpyruvoylglucosamine reductase"
"DsuE09_00996" 4913 "s63E6H2" 0 "UDP-N-acetylenolpyruvoylglucosamine reductase"
"DsuE09_00997" 4914 "s59E13H2" 0 "UDP-N-acetylmuramate--L-alanine ligase"
"DsuE09_00997" 4914 "s59E25H2" 0 "UDP-N-acetylmuramate--L-alanine ligase"
"DsuE09_00997" 4914 "s59E5H2" 0 "UDP-N-acetylmuramate--L-alanine ligase"
"DsuE09_00997" 4914 "s63E6H2" 0 "UDP-N-acetylmuramate--L-alanine ligase"
"DsuE09_00998" 4915 "s59E13H2" 0 "UDP-N-acetylglucosamine--N-acetylmuramyl-(pentapeptide) pyrophosphoryl-undecaprenol N-acetylglucosamine transferase"
"DsuE09_00998" 4915 "s59E25H2" 0 "UDP-N-acetylglucosamine--N-acetylmuramyl-(pentapeptide) pyrophosphoryl-undecaprenol N-acetylglucosamine transferase"
"DsuE09_00998" 4915 "s59E5H2" 0 "UDP-N-acetylglucosamine--N-acetylmuramyl-(pentapeptide) pyrophosphoryl-undecaprenol N-acetylglucosamine transferase"

"DsuE09_00998" 4915 "s63E6H2" 0 "UDP-N-acetylglucosamine--N-acetylmuramyl-(pentapeptide) pyrophosphoryl-undecaprenol N-acetylglucosamine transferase"
"DsuE09_00999" 4916 "s59E13H2" 0 "Lipid II flippase FtsW"
"DsuE09_00999" 4916 "s59E25H2" 0 "Lipid II flippase FtsW"
"DsuE09_00999" 4916 "s59E5H2" 0 "Lipid II flippase FtsW"
"DsuE09_00999" 4916 "s63E6H2" 0 "Lipid II flippase FtsW"
"DsuE09_01000" 4917 "s59E13H2" 0 "UDP-N-acetylmuramoylalanine--D-glutamate ligase"
"DsuE09_01000" 4917 "s59E25H2" 0 "UDP-N-acetylmuramoylalanine--D-glutamate ligase"
"DsuE09_01000" 4917 "s59E5H2" 0 "UDP-N-acetylmuramoylalanine--D-glutamate ligase"
"DsuE09_01000" 4917 "s63E6H2" 0 "UDP-N-acetylmuramoylalanine--D-glutamate ligase"
"DsuE09_01001" 4918 "s59E13H2" 0 "Phospho-N-acetylmuramoyl-pentapeptide-transferase"
"DsuE09_01001" 4918 "s59E25H2" 0 "Phospho-N-acetylmuramoyl-pentapeptide-transferase"
"DsuE09_01001" 4918 "s59E5H2" 0 "Phospho-N-acetylmuramoyl-pentapeptide-transferase"
"DsuE09_01001" 4918 "s63E6H2" 0 "Phospho-N-acetylmuramoyl-pentapeptide-transferase"
"DsuE09_01002" 4919 "s59E13H2" 0 "NADP-reducing hydrogenase subunit HndC"
"DsuE09_01002" 4919 "s59E25H2" 0 "NADP-reducing hydrogenase subunit HndC"
"DsuE09_01002" 4919 "s59E5H2" 0 "NADP-reducing hydrogenase subunit HndC"
"DsuE09_01002" 4919 "s63E6H2" 0 "NADP-reducing hydrogenase subunit HndC"
"DsuE09_01003" 4920 "s59E13H2" 0 "NADP-reducing hydrogenase subunit HndA"
"DsuE09_01003" 4920 "s59E25H2" 0 "NADP-reducing hydrogenase subunit HndA"
"DsuE09_01003" 4920 "s59E5H2" 0 "NADP-reducing hydrogenase subunit HndA"
"DsuE09_01003" 4920 "s63E6H2" 0 "NADP-reducing hydrogenase subunit HndA"
"DsuE09_01004" 4921 "s59E13H2" 0 "NADP-reducing hydrogenase subunit HndC"
"DsuE09_01004" 4921 "s59E25H2" 0 "NADP-reducing hydrogenase subunit HndC"
"DsuE09_01004" 4921 "s59E5H2" 0 "NADP-reducing hydrogenase subunit HndC"
"DsuE09_01004" 4921 "s63E6H2" 0 "NADP-reducing hydrogenase subunit HndC"
"DsuE09_01005" 4922 "s59E13H2" 0 "NADP-reducing hydrogenase subunit HndC"
"DsuE09_01005" 4922 "s59E25H2" 0 "NADP-reducing hydrogenase subunit HndC"
"DsuE09_01005" 4922 "s59E5H2" 0 "NADP-reducing hydrogenase subunit HndC"
"DsuE09_01005" 4922 "s63E6H2" 0 "NADP-reducing hydrogenase subunit HndC"
"DsuE09_01006" 4923 "s59E13H2" 0 "Putative formate dehydrogenase"
"DsuE09_01006" 4923 "s59E25H2" 0 "Putative formate dehydrogenase"
"DsuE09_01006" 4923 "s59E5H2" 0 "Putative formate dehydrogenase"
"DsuE09_01006" 4923 "s63E6H2" 0 "Putative formate dehydrogenase"
"DsuE09_01007" 4924 "s59E13H2" 0 "Formate dehydrogenase subunit alpha"
"DsuE09_01007" 4924 "s59E25H2" 0 "Formate dehydrogenase subunit alpha"
"DsuE09_01007" 4924 "s59E5H2" 0 "Formate dehydrogenase subunit alpha"
"DsuE09_01007" 4924 "s63E6H2" 0 "Formate dehydrogenase subunit alpha"
"DsuE09_01008" 4925 "s59E13H2" 0 "hypothetical protein"
"DsuE09_01008" 4925 "s59E25H2" 0 "hypothetical protein"
"DsuE09_01008" 4925 "s59E5H2" 0 "hypothetical protein"
"DsuE09_01008" 4925 "s63E6H2" 0 "hypothetical protein"
"DsuE09_01009" 4926 "s59E13H2" 0 "Hdr-like menaquinol oxidoreductase cytochrome b-like subunit"
"DsuE09_01009" 4926 "s59E25H2" 0 "Hdr-like menaquinol oxidoreductase cytochrome b-like subunit"
"DsuE09_01009" 4926 "s59E5H2" 0 "Hdr-like menaquinol oxidoreductase cytochrome b-like subunit"
"DsuE09_01009" 4926 "s63E6H2" 0 "Hdr-like menaquinol oxidoreductase cytochrome b-like subunit"
"DsuE09_01010" 4927 "s59E13H2" 0 "Hdr-like menaquinol oxidoreductase iron-sulfur subunit 2"
"DsuE09_01010" 4927 "s59E25H2" 0 "Hdr-like menaquinol oxidoreductase iron-sulfur subunit 2"

"DsuE09_01010" 4927 "s59E5H2" 0 "Hdr-like menaquinol oxidoreductase iron-sulfur subunit 2"
"DsuE09_01010" 4927 "s63E6H2" 0 "Hdr-like menaquinol oxidoreductase iron-sulfur subunit 2"
"DsuE09_01011" 4928 "s59E13H2" 0 "Hdr-like menaquinol oxidoreductase cytochrome c subunit"
"DsuE09_01011" 4928 "s59E25H2" 0 "Hdr-like menaquinol oxidoreductase cytochrome c subunit"
"DsuE09_01011" 4928 "s59E5H2" 0 "Hdr-like menaquinol oxidoreductase cytochrome c subunit"
"DsuE09_01011" 4928 "s63E6H2" 0 "Hdr-like menaquinol oxidoreductase cytochrome c subunit"
"DsuE09_01012" 4929 "s59E13H2" 0 "Hdr-like menaquinol oxidoreductase iron-sulfur subunit 1 precursor"
"DsuE09_01012" 4929 "s59E25H2" 0 "Hdr-like menaquinol oxidoreductase iron-sulfur subunit 1 precursor"
"DsuE09_01012" 4929 "s59E5H2" 0 "Hdr-like menaquinol oxidoreductase iron-sulfur subunit 1 precursor"
"DsuE09_01012" 4929 "s63E6H2" 0 "Hdr-like menaquinol oxidoreductase iron-sulfur subunit 1 precursor"
"DsuE09_01013" 4930 "s59E13H2" 0 "Polysulphide reductase, NrfD"
"DsuE09_01013" 4930 "s59E25H2" 0 "Polysulphide reductase, NrfD"
"DsuE09_01013" 4930 "s59E5H2" 0 "Polysulphide reductase, NrfD"
"DsuE09_01013" 4930 "s63E6H2" 0 "Polysulphide reductase, NrfD"
"DsuE09_01015" 4932 "s59E13H2" 0 "Sulfurtransferase TusD"
"DsuE09_01015" 4932 "s59E25H2" 0 "Sulfurtransferase TusD"
"DsuE09_01015" 4932 "s59E5H2" 0 "Sulfurtransferase TusD"
"DsuE09_01015" 4932 "s63E6H2" 0 "Sulfurtransferase TusD"
"DsuE09_01016" 4933 "s59E13H2" 0 "Acidic cytochrome c3 precursor"
"DsuE09_01016" 4933 "s59E25H2" 0 "Acidic cytochrome c3 precursor"
"DsuE09_01016" 4933 "s59E5H2" 0 "Acidic cytochrome c3 precursor"
"DsuE09_01016" 4933 "s63E6H2" 0 "Acidic cytochrome c3 precursor"
"DsuE09_01017" 4934 "s59E13H2" 0 "Universal stress protein"
"DsuE09_01017" 4934 "s59E25H2" 0 "Universal stress protein"
"DsuE09_01017" 4934 "s59E5H2" 0 "Universal stress protein"
"DsuE09_01017" 4934 "s63E6H2" 0 "Universal stress protein"
"DsuE09_01018" 4935 "s59E13H2" 0 "Hdr-like menaquinol oxidoreductase iron-sulfur subunit"
"DsuE09_01018" 4935 "s59E25H2" 0 "Hdr-like menaquinol oxidoreductase iron-sulfur subunit"
"DsuE09_01018" 4935 "s59E5H2" 0 "Hdr-like menaquinol oxidoreductase iron-sulfur subunit"
"DsuE09_01018" 4935 "s63E6H2" 0 "Hdr-like menaquinol oxidoreductase iron-sulfur subunit"
"DsuE09_01019" 4936 "s59E13H2" 0 "hypothetical protein"
"DsuE09_01019" 4936 "s59E25H2" 0 "hypothetical protein"
"DsuE09_01019" 4936 "s59E5H2" 0 "hypothetical protein"
"DsuE09_01019" 4936 "s63E6H2" 0 "hypothetical protein"
"DsuE09_01021" 4938 "s59E13H2" 0 "Transcriptional regulatory protein ZraR"
"DsuE09_01021" 4938 "s59E25H2" 0 "Transcriptional regulatory protein ZraR"
"DsuE09_01021" 4938 "s59E5H2" 0 "Transcriptional regulatory protein ZraR"
"DsuE09_01021" 4938 "s63E6H2" 0 "Transcriptional regulatory protein ZraR"
"DsuE09_01022" 4939 "s59E13H2" 0 "Dinitrogenase iron-molybdenum cofactor"
"DsuE09_01022" 4939 "s59E25H2" 0 "Dinitrogenase iron-molybdenum cofactor"
"DsuE09_01022" 4939 "s59E5H2" 0 "Dinitrogenase iron-molybdenum cofactor"
"DsuE09_01022" 4939 "s63E6H2" 0 "Dinitrogenase iron-molybdenum cofactor"
"DsuE09_01024" 4941 "s59E13H2" 0 "hypothetical protein"
"DsuE09_01024" 4941 "s59E25H2" 0 "hypothetical protein"
"DsuE09_01024" 4941 "s59E5H2" 0 "hypothetical protein"
"DsuE09_01024" 4941 "s63E6H2" 0 "hypothetical protein"
"DsuE09_01025" 4942 "s59E13H2" 0 "7,8-dihydropterin-6-methyl-4-(beta-D-ribofuranosyl)-aminobenzene-5'-phosphate synthase"

"DsuE09_01025" 4942 "s59E25H2" 0 "7,8-dihydropterin-6-methyl-4-(beta-D-ribofuranosyl)-aminobenzene-5'-phosphate synthase"
"DsuE09_01025" 4942 "s59E5H2" 0 "7,8-dihydropterin-6-methyl-4-(beta-D-ribofuranosyl)-aminobenzene-5'-phosphate synthase"
"DsuE09_01025" 4942 "s63E6H2" 0 "7,8-dihydropterin-6-methyl-4-(beta-D-ribofuranosyl)-aminobenzene-5'-phosphate synthase"
"DsuE09_01026" 4943 "s59E13H2" 0 "4-hydroxybutyryl-CoA dehydratase/vinylacetyl-CoA-Delta-isomerase"
"DsuE09_01026" 4943 "s59E25H2" 0 "4-hydroxybutyryl-CoA dehydratase/vinylacetyl-CoA-Delta-isomerase"
"DsuE09_01026" 4943 "s59E5H2" 0 "4-hydroxybutyryl-CoA dehydratase/vinylacetyl-CoA-Delta-isomerase"
"DsuE09_01026" 4943 "s63E6H2" 0 "4-hydroxybutyryl-CoA dehydratase/vinylacetyl-CoA-Delta-isomerase"
"DsuE09_01027" 4944 "s59E13H2" 0 "Dinitrogenase iron-molybdenum cofactor"
"DsuE09_01027" 4944 "s59E25H2" 0 "Dinitrogenase iron-molybdenum cofactor"
"DsuE09_01027" 4944 "s59E5H2" 0 "Dinitrogenase iron-molybdenum cofactor"
"DsuE09_01027" 4944 "s63E6H2" 0 "Dinitrogenase iron-molybdenum cofactor"
"DsuE09_01029" 4946 "s59E13H2" 0 "antiporter inner membrane protein"
"DsuE09_01029" 4946 "s59E25H2" 0 "antiporter inner membrane protein"
"DsuE09_01029" 4946 "s59E5H2" 0 "antiporter inner membrane protein"
"DsuE09_01029" 4946 "s63E6H2" 0 "antiporter inner membrane protein"
"DsuE09_01030" 4947 "s59E13H2" 0 "Dinitrogenase iron-molybdenum cofactor"
"DsuE09_01030" 4947 "s59E25H2" 0 "Dinitrogenase iron-molybdenum cofactor"
"DsuE09_01030" 4947 "s59E5H2" 0 "Dinitrogenase iron-molybdenum cofactor"
"DsuE09_01030" 4947 "s63E6H2" 0 "Dinitrogenase iron-molybdenum cofactor"
"DsuE09_01031" 4948 "s59E13H2" 0 "Dinitrogenase iron-molybdenum cofactor"
"DsuE09_01031" 4948 "s59E25H2" 0 "Dinitrogenase iron-molybdenum cofactor"
"DsuE09_01031" 4948 "s59E5H2" 0 "Dinitrogenase iron-molybdenum cofactor"
"DsuE09_01031" 4948 "s63E6H2" 0 "Dinitrogenase iron-molybdenum cofactor"
"DsuE09_01032" 4949 "s59E13H2" 0 "Electron transport complex subunit RsxB"
"DsuE09_01032" 4949 "s59E25H2" 0 "Electron transport complex subunit RsxB"
"DsuE09_01032" 4949 "s59E5H2" 0 "Electron transport complex subunit RsxB"
"DsuE09_01032" 4949 "s63E6H2" 0 "Electron transport complex subunit RsxB"
"DsuE09_01033" 4950 "s59E13H2" 0 "CoB--CoM heterodisulfide reductase iron-sulfur subunit A"
"DsuE09_01033" 4950 "s59E25H2" 0 "CoB--CoM heterodisulfide reductase iron-sulfur subunit A"
"DsuE09_01033" 4950 "s59E5H2" 0 "CoB--CoM heterodisulfide reductase iron-sulfur subunit A"
"DsuE09_01033" 4950 "s63E6H2" 0 "CoB--CoM heterodisulfide reductase iron-sulfur subunit A"
"DsuE09_01034" 4951 "s59E13H2" 0 "hypothetical protein"
"DsuE09_01034" 4951 "s59E25H2" 0 "hypothetical protein"
"DsuE09_01034" 4951 "s59E5H2" 0 "hypothetical protein"
"DsuE09_01034" 4951 "s63E6H2" 0 "hypothetical protein"
"DsuE09_01036" 4953 "s59E13H2" 0 "NifU-like protein"
"DsuE09_01036" 4953 "s59E25H2" 0 "NifU-like protein"
"DsuE09_01036" 4953 "s59E5H2" 0 "NifU-like protein"
"DsuE09_01036" 4953 "s63E6H2" 0 "NifU-like protein"
"DsuE09_01038" 4955 "s59E13H2" 0 "Chaperone protein DnaJ"
"DsuE09_01038" 4955 "s59E25H2" 0 "Chaperone protein DnaJ"
"DsuE09_01038" 4955 "s59E5H2" 0 "Chaperone protein DnaJ"
"DsuE09_01038" 4955 "s63E6H2" 0 "Chaperone protein DnaJ"
"DsuE09_01039" 4956 "s59E13H2" 0 "putative RNA-binding protein"
"DsuE09_01039" 4956 "s59E25H2" 0 "putative RNA-binding protein"
"DsuE09_01039" 4956 "s59E5H2" 0 "putative RNA-binding protein"
"DsuE09_01039" 4956 "s63E6H2" 0 "putative RNA-binding protein"

"DsuE09_01040" 4957 "s59E13H2" 0 "Thioredoxin-1"
"DsuE09_01040" 4957 "s59E25H2" 0 "Thioredoxin-1"
"DsuE09_01040" 4957 "s59E5H2" 0 "Thioredoxin-1"
"DsuE09_01040" 4957 "s63E6H2" 0 "Thioredoxin-1"
"DsuE09_01041" 4958 "s59E13H2" 0 "Acetyl-CoA decarbonylase/synthase complex subunit gamma"
"DsuE09_01041" 4958 "s59E25H2" 0 "Acetyl-CoA decarbonylase/synthase complex subunit gamma"
"DsuE09_01041" 4958 "s59E5H2" 0 "Acetyl-CoA decarbonylase/synthase complex subunit gamma"
"DsuE09_01041" 4958 "s63E6H2" 0 "Acetyl-CoA decarbonylase/synthase complex subunit gamma"
"DsuE09_01042" 4959 "s59E13H2" 0 "hypothetical protein"
"DsuE09_01042" 4959 "s59E25H2" 0 "hypothetical protein"
"DsuE09_01042" 4959 "s59E5H2" 0 "hypothetical protein"
"DsuE09_01042" 4959 "s63E6H2" 0 "hypothetical protein"
"DsuE09_01043" 4960 "s59E13H2" 0 "hypothetical protein"
"DsuE09_01043" 4960 "s59E25H2" 0 "hypothetical protein"
"DsuE09_01043" 4960 "s59E5H2" 0 "hypothetical protein"
"DsuE09_01043" 4960 "s63E6H2" 0 "hypothetical protein"
"DsuE09_01044" 4961 "s59E13H2" 0 "Dinitrogenase iron-molybdenum cofactor"
"DsuE09_01044" 4961 "s59E25H2" 0 "Dinitrogenase iron-molybdenum cofactor"
"DsuE09_01044" 4961 "s59E5H2" 0 "Dinitrogenase iron-molybdenum cofactor"
"DsuE09_01044" 4961 "s63E6H2" 0 "Dinitrogenase iron-molybdenum cofactor"
"DsuE09_01045" 4962 "s59E13H2" 0 "Dinitrogenase iron-molybdenum cofactor"
"DsuE09_01045" 4962 "s59E25H2" 0 "Dinitrogenase iron-molybdenum cofactor"
"DsuE09_01045" 4962 "s59E5H2" 0 "Dinitrogenase iron-molybdenum cofactor"
"DsuE09_01045" 4962 "s63E6H2" 0 "Dinitrogenase iron-molybdenum cofactor"
"DsuE09_01046" 4963 "s59E13H2" 0 "Response regulator receiver domain protein"
"DsuE09_01046" 4963 "s59E25H2" 0 "Response regulator receiver domain protein"
"DsuE09_01046" 4963 "s59E5H2" 0 "Response regulator receiver domain protein"
"DsuE09_01046" 4963 "s63E6H2" 0 "Response regulator receiver domain protein"
"DsuE09_01047" 4964 "s59E13H2" 0 "NifU-like protein"
"DsuE09_01047" 4964 "s59E25H2" 0 "NifU-like protein"
"DsuE09_01047" 4964 "s59E5H2" 0 "NifU-like protein"
"DsuE09_01047" 4964 "s63E6H2" 0 "NifU-like protein"
"DsuE09_01048" 4965 "s59E13H2" 0 "Zinc ribbon domain protein"
"DsuE09_01048" 4965 "s59E25H2" 0 "Zinc ribbon domain protein"
"DsuE09_01048" 4965 "s59E5H2" 0 "Zinc ribbon domain protein"
"DsuE09_01048" 4965 "s63E6H2" 0 "Zinc ribbon domain protein"
"DsuE09_01049" 4966 "s59E13H2" 0 "hypothetical protein"
"DsuE09_01049" 4966 "s59E25H2" 0 "hypothetical protein"
"DsuE09_01049" 4966 "s59E5H2" 0 "hypothetical protein"
"DsuE09_01049" 4966 "s63E6H2" 0 "hypothetical protein"
"DsuE09_01050" 4967 "s59E13H2" 0 "Sulfite exporter TauE/SafE"
"DsuE09_01050" 4967 "s59E25H2" 0 "Sulfite exporter TauE/SafE"
"DsuE09_01050" 4967 "s59E5H2" 0 "Sulfite exporter TauE/SafE"
"DsuE09_01050" 4967 "s63E6H2" 0 "Sulfite exporter TauE/SafE"
"DsuE09_01051" 4968 "s59E13H2" 0 "hypothetical protein"
"DsuE09_01051" 4968 "s59E25H2" 0 "hypothetical protein"
"DsuE09_01051" 4968 "s59E5H2" 0 "hypothetical protein"

"DsuE09_01051" 4968 "s63E6H2" 0 "hypothetical protein"
"DsuE09_01052" 4969 "s59E13H2" 0 "Coenzyme A disulfide reductase"
"DsuE09_01052" 4969 "s59E25H2" 0 "Coenzyme A disulfide reductase"
"DsuE09_01052" 4969 "s59E5H2" 0 "Coenzyme A disulfide reductase"
"DsuE09_01052" 4969 "s63E6H2" 0 "Coenzyme A disulfide reductase"
"DsuE09_01055" 4972 "s59E13H2" 0 "(R)-stereoselective amidase"
"DsuE09_01055" 4972 "s59E25H2" 0 "(R)-stereoselective amidase"
"DsuE09_01055" 4972 "s59E5H2" 0 "(R)-stereoselective amidase"
"DsuE09_01055" 4972 "s63E6H2" 0 "(R)-stereoselective amidase"
"JS1_A09_00001" 10487 "s59E13H2" 0.02123696 "ATP-dependent protease subunit HslV"
"JS1_A09_00001" 10487 "s59E25H2" 0 "ATP-dependent protease subunit HslV"
"JS1_A09_00001" 10487 "s59E5H2" 0.1647966 "ATP-dependent protease subunit HslV"
"JS1_A09_00001" 10487 "s63E6H2" 0.0444282 "ATP-dependent protease subunit HslV"
"JS1_A09_00002" 10488 "s59E13H2" 0 "Formate dehydrogenase subunit beta"
"JS1_A09_00002" 10488 "s59E25H2" 0 "Formate dehydrogenase subunit beta"
"JS1_A09_00002" 10488 "s59E5H2" 0.003573786 "Formate dehydrogenase subunit beta"
"JS1_A09_00002" 10488 "s63E6H2" 0 "Formate dehydrogenase subunit beta"
"JS1_A09_00003" 10489 "s59E13H2" 0 "F420-non-reducing hydrogenase vhc iron-sulfur subunit D"
"JS1_A09_00003" 10489 "s59E25H2" 0 "F420-non-reducing hydrogenase vhc iron-sulfur subunit D"
"JS1_A09_00003" 10489 "s59E5H2" 0 "F420-non-reducing hydrogenase vhc iron-sulfur subunit D"
"JS1_A09_00003" 10489 "s63E6H2" 0 "F420-non-reducing hydrogenase vhc iron-sulfur subunit D"
"JS1_A09_00004" 10490 "s59E13H2" 0 "CoB--CoM heterodisulfide reductase iron-sulfur subunit A"
"JS1_A09_00004" 10490 "s59E25H2" 0 "CoB--CoM heterodisulfide reductase iron-sulfur subunit A"
"JS1_A09_00004" 10490 "s59E5H2" 0.00562814 "CoB--CoM heterodisulfide reductase iron-sulfur subunit A"
"JS1_A09_00004" 10490 "s63E6H2" 0 "CoB--CoM heterodisulfide reductase iron-sulfur subunit A"
"JS1_A09_00005" 10491 "s59E13H2" 0 "hypothetical protein"
"JS1_A09_00005" 10491 "s59E25H2" 0 "hypothetical protein"
"JS1_A09_00005" 10491 "s59E5H2" 0 "hypothetical protein"
"JS1_A09_00005" 10491 "s63E6H2" 0 "hypothetical protein"
"JS1_A09_00006" 10492 "s59E13H2" 0.02996592 "hypothetical protein"
"JS1_A09_00006" 10492 "s59E25H2" 0 "hypothetical protein"
"JS1_A09_00006" 10492 "s59E5H2" 0.990751 "hypothetical protein"
"JS1_A09_00006" 10492 "s63E6H2" 1.467307 "hypothetical protein"
"JS1_A09_00009" 10495 "s59E13H2" 0.17658 "Transposase IS200 like protein"
"JS1_A09_00009" 10495 "s59E25H2" 0 "Transposase IS200 like protein"
"JS1_A09_00009" 10495 "s59E5H2" 0.02169815 "Transposase IS200 like protein"
"JS1_A09_00009" 10495 "s63E6H2" 0.2101454 "Transposase IS200 like protein"
"JS1_A09_00010" 10496 "s59E13H2" 0 "Enamine/imine deaminase"
"JS1_A09_00010" 10496 "s59E25H2" 0 "Enamine/imine deaminase"
"JS1_A09_00010" 10496 "s59E5H2" 0 "Enamine/imine deaminase"
"JS1_A09_00010" 10496 "s63E6H2" 0 "Enamine/imine deaminase"
"JS1_A09_00011" 10497 "s59E13H2" 0 "hypothetical protein"
"JS1_A09_00011" 10497 "s59E25H2" 0 "hypothetical protein"
"JS1_A09_00011" 10497 "s59E5H2" 0 "hypothetical protein"
"JS1_A09_00011" 10497 "s63E6H2" 0 "hypothetical protein"
"JS1_A09_00012" 10498 "s59E13H2" 0 "2-oxoglutarate synthase subunit KorC"
"JS1_A09_00012" 10498 "s59E25H2" 0 "2-oxoglutarate synthase subunit KorC"

"JS1_A09_00012" 10498 "s59E5H2" 0 "2-oxoglutarate synthase subunit KorC"
"JS1_A09_00012" 10498 "s63E6H2" 0 "2-oxoglutarate synthase subunit KorC"
"JS1_A09_00013" 10499 "s59E13H2" 0.1810703 "Integrase core domain protein"
"JS1_A09_00013" 10499 "s59E25H2" 0 "Integrase core domain protein"
"JS1_A09_00013" 10499 "s59E5H2" 16.86801 "Integrase core domain protein"
"JS1_A09_00013" 10499 "s63E6H2" 0.09171818 "Integrase core domain protein"
"JS1_A09_00014" 10500 "s59E13H2" 0 "CoB--CoM heterodisulfide reductase subunit B"
"JS1_A09_00014" 10500 "s59E25H2" 0 "CoB--CoM heterodisulfide reductase subunit B"
"JS1_A09_00014" 10500 "s59E5H2" 0 "CoB--CoM heterodisulfide reductase subunit B"
"JS1_A09_00014" 10500 "s63E6H2" 0 "CoB--CoM heterodisulfide reductase subunit B"
"JS1_A09_00015" 10501 "s59E13H2" 0 "CoB--CoM heterodisulfide reductase iron-sulfur subunit A"
"JS1_A09_00015" 10501 "s59E25H2" 0 "CoB--CoM heterodisulfide reductase iron-sulfur subunit A"
"JS1_A09_00015" 10501 "s59E5H2" 0.002085327 "CoB--CoM heterodisulfide reductase iron-sulfur subunit A"
"JS1_A09_00015" 10501 "s63E6H2" 0 "CoB--CoM heterodisulfide reductase iron-sulfur subunit A"
"JS1_A09_00016" 10502 "s59E13H2" 0 "CoB--CoM heterodisulfide reductase subunit B"
"JS1_A09_00016" 10502 "s59E25H2" 0 "CoB--CoM heterodisulfide reductase subunit B"
"JS1_A09_00016" 10502 "s59E5H2" 0.0176093 "CoB--CoM heterodisulfide reductase subunit B"
"JS1_A09_00016" 10502 "s63E6H2" 0 "CoB--CoM heterodisulfide reductase subunit B"
"JS1_A09_00017" 10503 "s59E13H2" 0 "L-Ala-D/L-Glu epimerase"
"JS1_A09_00017" 10503 "s59E25H2" 0 "L-Ala-D/L-Glu epimerase"
"JS1_A09_00017" 10503 "s59E5H2" 0 "L-Ala-D/L-Glu epimerase"
"JS1_A09_00017" 10503 "s63E6H2" 0 "L-Ala-D/L-Glu epimerase"
"JS1_A09_00018" 10504 "s59E13H2" 0 "hypothetical protein"
"JS1_A09_00018" 10504 "s59E25H2" 0 "hypothetical protein"
"JS1_A09_00018" 10504 "s59E5H2" 0 "hypothetical protein"
"JS1_A09_00018" 10504 "s63E6H2" 0 "hypothetical protein"
"JS1_A09_00019" 10505 "s59E13H2" 0.03527236 "CoB--CoM heterodisulfide reductase iron-sulfur subunit D"
"JS1_A09_00019" 10505 "s59E25H2" 0 "CoB--CoM heterodisulfide reductase iron-sulfur subunit D"
"JS1_A09_00019" 10505 "s59E5H2" 0.04617165 "CoB--CoM heterodisulfide reductase iron-sulfur subunit D"
"JS1_A09_00019" 10505 "s63E6H2" 0.03926208 "CoB--CoM heterodisulfide reductase iron-sulfur subunit D"
"JS1_A09_00020" 10506 "s59E13H2" 0 "CoB--CoM heterodisulfide reductase subunit B"
"JS1_A09_00020" 10506 "s59E25H2" 0 "CoB--CoM heterodisulfide reductase subunit B"
"JS1_A09_00020" 10506 "s59E5H2" 0.0146942 "CoB--CoM heterodisulfide reductase subunit B"
"JS1_A09_00020" 10506 "s63E6H2" 0.2602158 "CoB--CoM heterodisulfide reductase subunit B"
"JS1_A09_00021" 10507 "s59E13H2" 0 "CoB--CoM heterodisulfide reductase iron-sulfur subunit C"
"JS1_A09_00021" 10507 "s59E25H2" 0 "CoB--CoM heterodisulfide reductase iron-sulfur subunit C"
"JS1_A09_00021" 10507 "s59E5H2" 0.002802028 "CoB--CoM heterodisulfide reductase iron-sulfur subunit C"
"JS1_A09_00021" 10507 "s63E6H2" 0.1503911 "CoB--CoM heterodisulfide reductase iron-sulfur subunit C"
"JS1_A09_00022" 10508 "s59E13H2" 8.085155 "hypothetical protein"
"JS1_A09_00022" 10508 "s59E25H2" 0 "hypothetical protein"
"JS1_A09_00022" 10508 "s59E5H2" 1.483425 "hypothetical protein"
"JS1_A09_00022" 10508 "s63E6H2" 1.921744 "hypothetical protein"
"JS1_A09_00023" 10509 "s59E13H2" 0 "Formate dehydrogenase subunit beta"
"JS1_A09_00023" 10509 "s59E25H2" 0 "Formate dehydrogenase subunit beta"
"JS1_A09_00023" 10509 "s59E5H2" 0.004049331 "Formate dehydrogenase subunit beta"
"JS1_A09_00023" 10509 "s63E6H2" 0.004004225 "Formate dehydrogenase subunit beta"
"JS1_A09_00026" 10512 "s59E13H2" 0 "Succinyl-CoA ligase [ADP-forming] subunit beta"

"JS1_A09_00026" 10512 "s59E25H2" 0 "Succinyl-CoA ligase [ADP-forming] subunit beta"
"JS1_A09_00026" 10512 "s59E5H2" 0.01646906 "Succinyl-CoA ligase [ADP-forming] subunit beta"
"JS1_A09_00026" 10512 "s63E6H2" 0 "Succinyl-CoA ligase [ADP-forming] subunit beta"
"JS1_A09_00027" 10513 "s59E13H2" 0 "hypothetical protein"
"JS1_A09_00027" 10513 "s59E25H2" 0 "hypothetical protein"
"JS1_A09_00027" 10513 "s59E5H2" 0 "hypothetical protein"
"JS1_A09_00027" 10513 "s63E6H2" 0 "hypothetical protein"
"JS1_A09_00030" 10516 "s59E13H2" 0 "Formate dehydrogenase subunit beta"
"JS1_A09_00030" 10516 "s59E25H2" 0 "Formate dehydrogenase subunit beta"
"JS1_A09_00030" 10516 "s59E5H2" 0.03609948 "Formate dehydrogenase subunit beta"
"JS1_A09_00030" 10516 "s63E6H2" 0.0002032782 "Formate dehydrogenase subunit beta"
"JS1_A09_00031" 10517 "s59E13H2" 0 "putative aspartate aminotransferase 2"
"JS1_A09_00031" 10517 "s59E25H2" 0 "putative aspartate aminotransferase 2"
"JS1_A09_00031" 10517 "s59E5H2" 0 "putative aspartate aminotransferase 2"
"JS1_A09_00031" 10517 "s63E6H2" 0 "putative aspartate aminotransferase 2"
"JS1_A09_00032" 10518 "s59E13H2" 0 "Aspartate aminotransferase"
"JS1_A09_00032" 10518 "s59E25H2" 0 "Aspartate aminotransferase"
"JS1_A09_00032" 10518 "s59E5H2" 0 "Aspartate aminotransferase"
"JS1_A09_00032" 10518 "s63E6H2" 0 "Aspartate aminotransferase"
"JS1_A09_00033" 10519 "s59E13H2" 1.381994 "Endonuclease MutS2"
"JS1_A09_00033" 10519 "s59E25H2" 0 "Endonuclease MutS2"
"JS1_A09_00033" 10519 "s59E5H2" 0.3454048 "Endonuclease MutS2"
"JS1_A09_00033" 10519 "s63E6H2" 1.802055 "Endonuclease MutS2"
"JS1_A09_00034" 10520 "s59E13H2" 0.6951119 "Penicillin-binding protein 2D"
"JS1_A09_00034" 10520 "s59E25H2" 0 "Penicillin-binding protein 2D"
"JS1_A09_00034" 10520 "s59E5H2" 0.4310114 "Penicillin-binding protein 2D"
"JS1_A09_00034" 10520 "s63E6H2" 0.8260356 "Penicillin-binding protein 2D"
"JS1_A09_00035" 10521 "s59E13H2" 0.9439096 "Queuosine precursor transporter QueT"
"JS1_A09_00035" 10521 "s59E25H2" 0 "Queuosine precursor transporter QueT"
"JS1_A09_00035" 10521 "s59E5H2" 0.4185952 "Queuosine precursor transporter QueT"
"JS1_A09_00035" 10521 "s63E6H2" 0.7338794 "Queuosine precursor transporter QueT"
"JS1_A09_00036" 10522 "s59E13H2" 1.171473 "Tyrosine--tRNA ligase"
"JS1_A09_00036" 10522 "s59E25H2" 0 "Tyrosine--tRNA ligase"
"JS1_A09_00036" 10522 "s59E5H2" 0.834486 "Tyrosine--tRNA ligase"
"JS1_A09_00036" 10522 "s63E6H2" 1.218522 "Tyrosine--tRNA ligase"
"JS1_A09_00039" 10525 "s59E13H2" 0.6089416 "DNA replication protein DnaC"
"JS1_A09_00039" 10525 "s59E25H2" 0 "DNA replication protein DnaC"
"JS1_A09_00039" 10525 "s59E5H2" 0.9310851 "DNA replication protein DnaC"
"JS1_A09_00039" 10525 "s63E6H2" 0.2132491 "DNA replication protein DnaC"
"JS1_A09_00040" 10526 "s59E13H2" 0 "hypothetical protein"
"JS1_A09_00040" 10526 "s59E25H2" 0 "hypothetical protein"
"JS1_A09_00040" 10526 "s59E5H2" 0 "hypothetical protein"
"JS1_A09_00040" 10526 "s63E6H2" 0 "hypothetical protein"
"JS1_A09_00041" 10527 "s59E13H2" 0.776488 "putative epimerase/dehydratase"
"JS1_A09_00041" 10527 "s59E25H2" 0 "putative epimerase/dehydratase"
"JS1_A09_00041" 10527 "s59E5H2" 1.457321 "putative epimerase/dehydratase"
"JS1_A09_00041" 10527 "s63E6H2" 2.063023 "putative epimerase/dehydratase"

"JS1_A09_00042" 10528 "s59E13H2" 0.441636 "putative HTH-type transcriptional regulator YdfH"
"JS1_A09_00042" 10528 "s59E25H2" 0 "putative HTH-type transcriptional regulator YdfH"
"JS1_A09_00042" 10528 "s59E5H2" 0.3893132 "putative HTH-type transcriptional regulator YdfH"
"JS1_A09_00042" 10528 "s63E6H2" 1.567222 "putative HTH-type transcriptional regulator YdfH"
"JS1_A09_00043" 10529 "s59E13H2" 0.8831805 "Malate dehydrogenase"
"JS1_A09_00043" 10529 "s59E25H2" 0 "Malate dehydrogenase"
"JS1_A09_00043" 10529 "s59E5H2" 1.819832 "Malate dehydrogenase"
"JS1_A09_00043" 10529 "s63E6H2" 0.9259267 "Malate dehydrogenase"
"JS1_A09_00044" 10530 "s59E13H2" 0.4903079 "hypothetical protein"
"JS1_A09_00044" 10530 "s59E25H2" 0 "hypothetical protein"
"JS1_A09_00044" 10530 "s59E5H2" 0.6361127 "hypothetical protein"
"JS1_A09_00044" 10530 "s63E6H2" 0.793805 "hypothetical protein"
"JS1_A09_00046" 10532 "s59E13H2" 0.004767683 "hypothetical protein"
"JS1_A09_00046" 10532 "s59E25H2" 0 "hypothetical protein"
"JS1_A09_00046" 10532 "s59E5H2" 0.2256704 "hypothetical protein"
"JS1_A09_00046" 10532 "s63E6H2" 0.9291609 "hypothetical protein"
"JS1_A09_00047" 10533 "s59E13H2" 0.004252926 "Flavoredoxin"
"JS1_A09_00047" 10533 "s59E25H2" 0 "Flavoredoxin"
"JS1_A09_00047" 10533 "s59E5H2" 0.1032818 "Flavoredoxin"
"JS1_A09_00047" 10533 "s63E6H2" 0.7417093 "Flavoredoxin"
"JS1_A09_00048" 10534 "s59E13H2" 0.005972602 "Endonuclease YhcR precursor"
"JS1_A09_00048" 10534 "s59E25H2" 0.293381 "Endonuclease YhcR precursor"
"JS1_A09_00048" 10534 "s59E5H2" 0.1174263 "Endonuclease YhcR precursor"
"JS1_A09_00048" 10534 "s63E6H2" 0.7602411 "Endonuclease YhcR precursor"
"JS1_A09_00049" 10535 "s59E13H2" 0.0896187 "DNA replication protein DnaC"
"JS1_A09_00049" 10535 "s59E25H2" 0 "DNA replication protein DnaC"
"JS1_A09_00049" 10535 "s59E5H2" 0.1253141 "DNA replication protein DnaC"
"JS1_A09_00049" 10535 "s63E6H2" 0.04721255 "DNA replication protein DnaC"
"JS1_A09_00051" 10537 "s59E13H2" 0 "hypothetical protein"
"JS1_A09_00051" 10537 "s59E25H2" 0 "hypothetical protein"
"JS1_A09_00051" 10537 "s59E5H2" 0 "hypothetical protein"
"JS1_A09_00051" 10537 "s63E6H2" 0 "hypothetical protein"
"JS1_A09_00052" 10538 "s59E13H2" 0 "hypothetical protein"
"JS1_A09_00052" 10538 "s59E25H2" 0 "hypothetical protein"
"JS1_A09_00052" 10538 "s59E5H2" 0 "hypothetical protein"
"JS1_A09_00052" 10538 "s63E6H2" 0 "hypothetical protein"
"JS1_A09_00054" 10540 "s59E13H2" 0.4995429 "RDD family protein"
"JS1_A09_00054" 10540 "s59E25H2" 0 "RDD family protein"
"JS1_A09_00054" 10540 "s59E5H2" 1.760363 "RDD family protein"
"JS1_A09_00054" 10540 "s63E6H2" 1.149724 "RDD family protein"
"JS1_A09_00058" 10544 "s59E13H2" 0.05183462 "Bifunctional homocysteine S-methyltransferase/5,10-methylenetetrahydrofolate reductase"
"JS1_A09_00058" 10544 "s59E25H2" 0 "Bifunctional homocysteine S-methyltransferase/5,10-methylenetetrahydrofolate reductase"
"JS1_A09_00058" 10544 "s59E5H2" 0.363359 "Bifunctional homocysteine S-methyltransferase/5,10-methylenetetrahydrofolate reductase"
"JS1_A09_00058" 10544 "s63E6H2" 0.04374387 "Bifunctional homocysteine S-methyltransferase/5,10-methylenetetrahydrofolate reductase"
"JS1_A09_00059" 10545 "s59E13H2" 0.02624689 "hypothetical protein"
"JS1_A09_00059" 10545 "s59E25H2" 0 "hypothetical protein"
"JS1_A09_00059" 10545 "s59E5H2" 0.09948405 "hypothetical protein"

"JS1_A09_00059" 10545 "s63E6H2" 0.3350787 "hypothetical protein"
"JS1_A09_00060" 10546 "s59E13H2" 0.00502731 "D-3-phosphoglycerate dehydrogenase"
"JS1_A09_00060" 10546 "s59E25H2" 0 "D-3-phosphoglycerate dehydrogenase"
"JS1_A09_00060" 10546 "s59E5H2" 0.4615439 "D-3-phosphoglycerate dehydrogenase"
"JS1_A09_00060" 10546 "s63E6H2" 0.1138613 "D-3-phosphoglycerate dehydrogenase"
"JS1_A09_00061" 10547 "s59E13H2" 0.7881576 "Soluble hydrogenase 42 kDa subunit"
"JS1_A09_00061" 10547 "s59E25H2" 0 "Soluble hydrogenase 42 kDa subunit"
"JS1_A09_00061" 10547 "s59E5H2" 0.3000457 "Soluble hydrogenase 42 kDa subunit"
"JS1_A09_00061" 10547 "s63E6H2" 0.04757285 "Soluble hydrogenase 42 kDa subunit"
"JS1_A09_00062" 10548 "s59E13H2" 0.237995 "Phosphorylated carbohydrates phosphatase"
"JS1_A09_00062" 10548 "s59E25H2" 0 "Phosphorylated carbohydrates phosphatase"
"JS1_A09_00062" 10548 "s59E5H2" 0.03795728 "Phosphorylated carbohydrates phosphatase"
"JS1_A09_00062" 10548 "s63E6H2" 0.03716745 "Phosphorylated carbohydrates phosphatase"
"JS1_A09_00063" 10549 "s59E13H2" 0 "hypothetical protein"
"JS1_A09_00063" 10549 "s59E25H2" 0 "hypothetical protein"
"JS1_A09_00063" 10549 "s59E5H2" 0.2174084 "hypothetical protein"
"JS1_A09_00063" 10549 "s63E6H2" 0.001806062 "hypothetical protein"
"JS1_A09_00064" 10550 "s59E13H2" 0 "hypothetical protein"
"JS1_A09_00064" 10550 "s59E25H2" 0 "hypothetical protein"
"JS1_A09_00064" 10550 "s59E5H2" 0.2483945 "hypothetical protein"
"JS1_A09_00064" 10550 "s63E6H2" 0.007857382 "hypothetical protein"
"JS1_A09_00065" 10551 "s59E13H2" 0.003463996 "Low molecular weight protein-tyrosine-phosphatase YwlE"
"JS1_A09_00065" 10551 "s59E25H2" 0 "Low molecular weight protein-tyrosine-phosphatase YwlE"
"JS1_A09_00065" 10551 "s59E5H2" 0.02750205 "Low molecular weight protein-tyrosine-phosphatase YwlE"
"JS1_A09_00065" 10551 "s63E6H2" 0.009143058 "Low molecular weight protein-tyrosine-phosphatase YwlE"
"JS1_A09_00066" 10552 "s59E13H2" 0 "Putative sugar phosphate isomerase YwlF"
"JS1_A09_00066" 10552 "s59E25H2" 0 "Putative sugar phosphate isomerase YwlF"
"JS1_A09_00066" 10552 "s59E5H2" 0.007572359 "Putative sugar phosphate isomerase YwlF"
"JS1_A09_00066" 10552 "s63E6H2" 0.03075785 "Putative sugar phosphate isomerase YwlF"
"JS1_A09_00071" 10557 "s59E13H2" 0 "2-oxoglutarate synthase subunit KorA"
"JS1_A09_00071" 10557 "s59E25H2" 0 "2-oxoglutarate synthase subunit KorA"
"JS1_A09_00071" 10557 "s59E5H2" 0 "2-oxoglutarate synthase subunit KorA"
"JS1_A09_00071" 10557 "s63E6H2" 0.01364507 "2-oxoglutarate synthase subunit KorA"
"JS1_A09_00072" 10558 "s59E13H2" 0 "L-Ala-D/L-Glu epimerase"
"JS1_A09_00072" 10558 "s59E25H2" 0 "L-Ala-D/L-Glu epimerase"
"JS1_A09_00072" 10558 "s59E5H2" 0 "L-Ala-D/L-Glu epimerase"
"JS1_A09_00072" 10558 "s63E6H2" 0 "L-Ala-D/L-Glu epimerase"
"JS1_A09_00073" 10559 "s59E13H2" 0 "Muconate cycloisomerase 1"
"JS1_A09_00073" 10559 "s59E25H2" 0 "Muconate cycloisomerase 1"
"JS1_A09_00073" 10559 "s59E5H2" 0 "Muconate cycloisomerase 1"
"JS1_A09_00073" 10559 "s63E6H2" 0 "Muconate cycloisomerase 1"
"JS1_A09_00074" 10560 "s59E13H2" 0 "L-allo-threonine aldolase"
"JS1_A09_00074" 10560 "s59E25H2" 0 "L-allo-threonine aldolase"
"JS1_A09_00074" 10560 "s59E5H2" 0 "L-allo-threonine aldolase"
"JS1_A09_00074" 10560 "s63E6H2" 0.009716321 "L-allo-threonine aldolase"
"JS1_A09_00075" 10561 "s59E13H2" 0 "Cysteine desulfurase"
"JS1_A09_00075" 10561 "s59E25H2" 0 "Cysteine desulfurase"

"JS1_A09_00075" 10561 "s59E5H2" 0 "Cysteine desulfurase"
"JS1_A09_00075" 10561 "s63E6H2" 0 "Cysteine desulfurase"
"JS1_A09_00076" 10562 "s59E13H2" 0.2810059 "hypothetical protein"
"JS1_A09_00076" 10562 "s59E25H2" 0 "hypothetical protein"
"JS1_A09_00076" 10562 "s59E5H2" 0.06426672 "hypothetical protein"
"JS1_A09_00076" 10562 "s63E6H2" 3.241264 "hypothetical protein"
"JS1_A09_00077" 10563 "s59E13H2" 0 "hypothetical protein"
"JS1_A09_00077" 10563 "s59E25H2" 0 "hypothetical protein"
"JS1_A09_00077" 10563 "s59E5H2" 0 "hypothetical protein"
"JS1_A09_00077" 10563 "s63E6H2" 0 "hypothetical protein"
"JS1_A09_00083" 10569 "s59E13H2" 0 "hypothetical protein"
"JS1_A09_00083" 10569 "s59E25H2" 0 "hypothetical protein"
"JS1_A09_00083" 10569 "s59E5H2" 0 "hypothetical protein"
"JS1_A09_00083" 10569 "s63E6H2" 0 "hypothetical protein"
"JS1_A09_00084" 10570 "s59E13H2" 0 "Cysteine desulfurase"
"JS1_A09_00084" 10570 "s59E25H2" 0 "Cysteine desulfurase"
"JS1_A09_00084" 10570 "s59E5H2" 0 "Cysteine desulfurase"
"JS1_A09_00084" 10570 "s63E6H2" 0 "Cysteine desulfurase"
"JS1_A09_00085" 10571 "s59E13H2" 0 "Sialic acid TRAP transporter permease protein SiaT"
"JS1_A09_00085" 10571 "s59E25H2" 0 "Sialic acid TRAP transporter permease protein SiaT"
"JS1_A09_00085" 10571 "s59E5H2" 0 "Sialic acid TRAP transporter permease protein SiaT"
"JS1_A09_00085" 10571 "s63E6H2" 0 "Sialic acid TRAP transporter permease protein SiaT"
"JS1_A09_00086" 10572 "s59E13H2" 0.001598987 "Sulfide dehydrogenase subunit alpha precursor"
"JS1_A09_00086" 10572 "s59E25H2" 0 "Sulfide dehydrogenase subunit alpha precursor"
"JS1_A09_00086" 10572 "s59E5H2" 0.01317809 "Sulfide dehydrogenase subunit alpha precursor"
"JS1_A09_00086" 10572 "s63E6H2" 0.001400333 "Sulfide dehydrogenase subunit alpha precursor"
"JS1_A09_00088" 10574 "s59E13H2" 0 "Cysteine desulfurase"
"JS1_A09_00088" 10574 "s59E25H2" 0 "Cysteine desulfurase"
"JS1_A09_00088" 10574 "s59E5H2" 0 "Cysteine desulfurase"
"JS1_A09_00088" 10574 "s63E6H2" 0 "Cysteine desulfurase"
"JS1_A09_00089" 10575 "s59E13H2" 0 "hypothetical protein"
"JS1_A09_00089" 10575 "s59E25H2" 0 "hypothetical protein"
"JS1_A09_00089" 10575 "s59E5H2" 0 "hypothetical protein"
"JS1_A09_00089" 10575 "s63E6H2" 0 "hypothetical protein"
"JS1_A09_00090" 10576 "s59E13H2" 0 "Formate dehydrogenase subunit beta"
"JS1_A09_00090" 10576 "s59E25H2" 0 "Formate dehydrogenase subunit beta"
"JS1_A09_00090" 10576 "s59E5H2" 0.001413331 "Formate dehydrogenase subunit beta"
"JS1_A09_00090" 10576 "s63E6H2" 0 "Formate dehydrogenase subunit beta"
"JS1_A09_00091" 10577 "s59E13H2" 0.7599733 "Poly-beta-1,6-N-acetyl-D-glucosamine N-deacetylase precursor"
"JS1_A09_00091" 10577 "s59E25H2" 0 "Poly-beta-1,6-N-acetyl-D-glucosamine N-deacetylase precursor"
"JS1_A09_00091" 10577 "s59E5H2" 0.4631965 "Poly-beta-1,6-N-acetyl-D-glucosamine N-deacetylase precursor"
"JS1_A09_00091" 10577 "s63E6H2" 0.7137718 "Poly-beta-1,6-N-acetyl-D-glucosamine N-deacetylase precursor"
"JS1_A09_00092" 10578 "s59E13H2" 0.726006 "High-affinity branched-chain amino acid transport ATP-binding protein LivF"
"JS1_A09_00092" 10578 "s59E25H2" 0 "High-affinity branched-chain amino acid transport ATP-binding protein LivF"
"JS1_A09_00092" 10578 "s59E5H2" 0.3689146 "High-affinity branched-chain amino acid transport ATP-binding protein LivF"
"JS1_A09_00092" 10578 "s63E6H2" 0.6478884 "High-affinity branched-chain amino acid transport ATP-binding protein LivF"
"JS1_A09_00093" 10579 "s59E13H2" 0.02867541 "YibE/F-like protein"

"JS1_A09_00093" 10579 "s59E25H2" 0 "YibE/F-like protein"
"JS1_A09_00093" 10579 "s59E5H2" 0.6382902 "YibE/F-like protein"
"JS1_A09_00093" 10579 "s63E6H2" 2.833796 "YibE/F-like protein"
"JS1_A09_00094" 10580 "s59E13H2" 2.905628 "Sorbitol dehydrogenase"
"JS1_A09_00094" 10580 "s59E25H2" 0.7261209 "Sorbitol dehydrogenase"
"JS1_A09_00094" 10580 "s59E5H2" 0.611274 "Sorbitol dehydrogenase"
"JS1_A09_00094" 10580 "s63E6H2" 1.473647 "Sorbitol dehydrogenase"
"JS1_A09_00095" 10581 "s59E13H2" 1.981086 "Folate transporter FolT"
"JS1_A09_00095" 10581 "s59E25H2" 0 "Folate transporter FolT"
"JS1_A09_00095" 10581 "s59E5H2" 0.4961218 "Folate transporter FolT"
"JS1_A09_00095" 10581 "s63E6H2" 1.368264 "Folate transporter FolT"
"JS1_A09_00096" 10582 "s59E13H2" 0.5226631 "hypothetical protein"
"JS1_A09_00096" 10582 "s59E25H2" 0 "hypothetical protein"
"JS1_A09_00096" 10582 "s59E5H2" 0.2954781 "hypothetical protein"
"JS1_A09_00096" 10582 "s63E6H2" 0.508851 "hypothetical protein"
"JS1_A09_00097" 10583 "s59E13H2" 0.476754 "Membrane dipeptidase (Peptidase family M19)"
"JS1_A09_00097" 10583 "s59E25H2" 0 "Membrane dipeptidase (Peptidase family M19)"
"JS1_A09_00097" 10583 "s59E5H2" 0.5306705 "Membrane dipeptidase (Peptidase family M19)"
"JS1_A09_00097" 10583 "s63E6H2" 1.563047 "Membrane dipeptidase (Peptidase family M19)"
"JS1_A09_00098" 10584 "s59E13H2" 0.9677836 "lipoprotein NlpI"
"JS1_A09_00098" 10584 "s59E25H2" 0 "lipoprotein NlpI"
"JS1_A09_00098" 10584 "s59E5H2" 0.4103309 "lipoprotein NlpI"
"JS1_A09_00098" 10584 "s63E6H2" 0.7849267 "lipoprotein NlpI"
"JS1_A09_00099" 10585 "s59E13H2" 1.118745 "Inosine-5'-monophosphate dehydrogenase"
"JS1_A09_00099" 10585 "s59E25H2" 0 "Inosine-5'-monophosphate dehydrogenase"
"JS1_A09_00099" 10585 "s59E5H2" 1.194385 "Inosine-5'-monophosphate dehydrogenase"
"JS1_A09_00099" 10585 "s63E6H2" 1.066926 "Inosine-5'-monophosphate dehydrogenase"
"JS1_A09_00100" 10586 "s59E13H2" 0.611775 "Quinolinate synthase A"
"JS1_A09_00100" 10586 "s59E25H2" 0 "Quinolinate synthase A"
"JS1_A09_00100" 10586 "s59E5H2" 0.4621312 "Quinolinate synthase A"
"JS1_A09_00100" 10586 "s63E6H2" 0.693303 "Quinolinate synthase A"
"JS1_A09_00101" 10587 "s59E13H2" 5.748585 "Ribosomal RNA small subunit methyltransferase D"
"JS1_A09_00101" 10587 "s59E25H2" 0 "Ribosomal RNA small subunit methyltransferase D"
"JS1_A09_00101" 10587 "s59E5H2" 0.07539327 "Ribosomal RNA small subunit methyltransferase D"
"JS1_A09_00101" 10587 "s63E6H2" 0.1617875 "Ribosomal RNA small subunit methyltransferase D"
"JS1_A09_00102" 10588 "s59E13H2" 6.435317 "Phosphopantetheine adenylyltransferase"
"JS1_A09_00102" 10588 "s59E25H2" 0 "Phosphopantetheine adenylyltransferase"
"JS1_A09_00102" 10588 "s59E5H2" 0.106089 "Phosphopantetheine adenylyltransferase"
"JS1_A09_00102" 10588 "s63E6H2" 0.3477254 "Phosphopantetheine adenylyltransferase"
"JS1_A09_00103" 10589 "s59E13H2" 5.803717 "Lon protease"
"JS1_A09_00103" 10589 "s59E25H2" 0 "Lon protease"
"JS1_A09_00103" 10589 "s59E5H2" 0.2630611 "Lon protease"
"JS1_A09_00103" 10589 "s63E6H2" 0.5231005 "Lon protease"
"JS1_A09_00107" 10593 "s59E13H2" 0 "2-oxoglutarate synthase subunit KorA"
"JS1_A09_00107" 10593 "s59E25H2" 0 "2-oxoglutarate synthase subunit KorA"
"JS1_A09_00107" 10593 "s59E5H2" 0 "2-oxoglutarate synthase subunit KorA"
"JS1_A09_00107" 10593 "s63E6H2" 0.01198082 "2-oxoglutarate synthase subunit KorA"

"JS1_A09_00108" 10594 "s59E13H2" 0 "2-oxoglutarate synthase subunit KorA"
"JS1_A09_00108" 10594 "s59E25H2" 0 "2-oxoglutarate synthase subunit KorA"
"JS1_A09_00108" 10594 "s59E5H2" 0.009920612 "2-oxoglutarate synthase subunit KorA"
"JS1_A09_00108" 10594 "s63E6H2" 0.01075218 "2-oxoglutarate synthase subunit KorA"
"JS1_A09_00110" 10596 "s59E13H2" 0.6790865 "DNA replication protein DnaC"
"JS1_A09_00110" 10596 "s59E25H2" 0 "DNA replication protein DnaC"
"JS1_A09_00110" 10596 "s59E5H2" 0.6645285 "DNA replication protein DnaC"
"JS1_A09_00110" 10596 "s63E6H2" 0.1967189 "DNA replication protein DnaC"
"JS1_A09_00112" 10598 "s59E13H2" 0.7227023 "ISXO2-like transposase domain protein"
"JS1_A09_00112" 10598 "s59E25H2" 0 "ISXO2-like transposase domain protein"
"JS1_A09_00112" 10598 "s59E5H2" 0 "ISXO2-like transposase domain protein"
"JS1_A09_00112" 10598 "s63E6H2" 0 "ISXO2-like transposase domain protein"
"JS1_A09_00113" 10599 "s59E13H2" 0.03180842 "hypothetical protein"
"JS1_A09_00113" 10599 "s59E25H2" 0 "hypothetical protein"
"JS1_A09_00113" 10599 "s59E5H2" 0.158457 "hypothetical protein"
"JS1_A09_00113" 10599 "s63E6H2" 0.5001688 "hypothetical protein"
"JS1_A09_00114" 10600 "s59E13H2" 0 "Pectin degradation repressor protein KdgR"
"JS1_A09_00114" 10600 "s59E25H2" 0 "Pectin degradation repressor protein KdgR"
"JS1_A09_00114" 10600 "s59E5H2" 0 "Pectin degradation repressor protein KdgR"
"JS1_A09_00114" 10600 "s63E6H2" 0 "Pectin degradation repressor protein KdgR"
"JS1_A09_00115" 10601 "s59E13H2" 0 "Malate dehydrogenase"
"JS1_A09_00115" 10601 "s59E25H2" 0 "Malate dehydrogenase"
"JS1_A09_00115" 10601 "s59E5H2" 0 "Malate dehydrogenase"
"JS1_A09_00115" 10601 "s63E6H2" 0.001365638 "Malate dehydrogenase"
"JS1_A09_00116" 10602 "s59E13H2" 0.04075733 "hypothetical protein"
"JS1_A09_00116" 10602 "s59E25H2" 0 "hypothetical protein"
"JS1_A09_00116" 10602 "s59E5H2" 0.3184752 "hypothetical protein"
"JS1_A09_00116" 10602 "s63E6H2" 0.2013631 "hypothetical protein"
"JS1_A09_00117" 10603 "s59E13H2" 0.06811248 "5'-nucleotidase SurE"
"JS1_A09_00117" 10603 "s59E25H2" 0 "5'-nucleotidase SurE"
"JS1_A09_00117" 10603 "s59E5H2" 0.5687984 "5'-nucleotidase SurE"
"JS1_A09_00117" 10603 "s63E6H2" 0.281863 "5'-nucleotidase SurE"
"JS1_A09_00118" 10604 "s59E13H2" 0.04920438 "hypothetical protein"
"JS1_A09_00118" 10604 "s59E25H2" 0 "hypothetical protein"
"JS1_A09_00118" 10604 "s59E5H2" 0.4925815 "hypothetical protein"
"JS1_A09_00118" 10604 "s63E6H2" 0.2419614 "hypothetical protein"
"JS1_A09_00119" 10605 "s59E13H2" 0.05587441 "Protein-L-isoaspartate O-methyltransferase"
"JS1_A09_00119" 10605 "s59E25H2" 0 "Protein-L-isoaspartate O-methyltransferase"
"JS1_A09_00119" 10605 "s59E5H2" 0.3907639 "Protein-L-isoaspartate O-methyltransferase"
"JS1_A09_00119" 10605 "s63E6H2" 0.5271681 "Protein-L-isoaspartate O-methyltransferase"
"JS1_A09_00121" 10607 "s59E13H2" 0.08439862 "Vancomycin B-type resistance protein VanW"
"JS1_A09_00121" 10607 "s59E25H2" 0.3377483 "Vancomycin B-type resistance protein VanW"
"JS1_A09_00121" 10607 "s59E5H2" 0.3743849 "Vancomycin B-type resistance protein VanW"
"JS1_A09_00121" 10607 "s63E6H2" 0.5710596 "Vancomycin B-type resistance protein VanW"
"JS1_A09_00122" 10608 "s59E13H2" 0.1992583 "aspartoacylase"
"JS1_A09_00122" 10608 "s59E25H2" 0 "aspartoacylase"
"JS1_A09_00122" 10608 "s59E5H2" 0.5273334 "aspartoacylase"

"JS1_A09_00122" 10608 "s63E6H2" 0.2534851 "aspartoacylase"
"JS1_A09_00123" 10609 "s59E13H2" 6.074481 "Putative peptidyl-prolyl cis-trans isomerase Cbf2 precursor"
"JS1_A09_00123" 10609 "s59E25H2" 0 "Putative peptidyl-prolyl cis-trans isomerase Cbf2 precursor"
"JS1_A09_00123" 10609 "s59E5H2" 0.3041226 "Putative peptidyl-prolyl cis-trans isomerase Cbf2 precursor"
"JS1_A09_00123" 10609 "s63E6H2" 0.2799655 "Putative peptidyl-prolyl cis-trans isomerase Cbf2 precursor"
"JS1_A09_00124" 10610 "s59E13H2" 0.03248941 "hypothetical protein"
"JS1_A09_00124" 10610 "s59E25H2" 0 "hypothetical protein"
"JS1_A09_00124" 10610 "s59E5H2" 0.03398408 "hypothetical protein"
"JS1_A09_00124" 10610 "s63E6H2" 0.3134328 "hypothetical protein"
"JS1_A09_00126" 10612 "s59E13H2" 0.0270191 "Peptidase M15"
"JS1_A09_00126" 10612 "s59E25H2" 0 "Peptidase M15"
"JS1_A09_00126" 10612 "s59E5H2" 0.05365615 "Peptidase M15"
"JS1_A09_00126" 10612 "s63E6H2" 1.125466 "Peptidase M15"
"JS1_A09_00127" 10613 "s59E13H2" 1.003125 "hypothetical protein"
"JS1_A09_00127" 10613 "s59E25H2" 0 "hypothetical protein"
"JS1_A09_00127" 10613 "s59E5H2" 1.110444 "hypothetical protein"
"JS1_A09_00127" 10613 "s63E6H2" 4.724103 "hypothetical protein"
"JS1_A09_00128" 10614 "s59E13H2" 0.3768969 "Inner membrane symporter YicJ"
"JS1_A09_00128" 10614 "s59E25H2" 0 "Inner membrane symporter YicJ"
"JS1_A09_00128" 10614 "s59E5H2" 3.819074 "Inner membrane symporter YicJ"
"JS1_A09_00128" 10614 "s63E6H2" 1.79467 "Inner membrane symporter YicJ"
"JS1_A09_00129" 10615 "s59E13H2" 0.2602169 "Carbon-nitrogen hydrolase"
"JS1_A09_00129" 10615 "s59E25H2" 0 "Carbon-nitrogen hydrolase"
"JS1_A09_00129" 10615 "s59E5H2" 2.478628 "Carbon-nitrogen hydrolase"
"JS1_A09_00129" 10615 "s63E6H2" 0.9498612 "Carbon-nitrogen hydrolase"
"JS1_A09_00130" 10616 "s59E13H2" 0.109356 "Formamidase"
"JS1_A09_00130" 10616 "s59E25H2" 0 "Formamidase"
"JS1_A09_00130" 10616 "s59E5H2" 0.8043234 "Formamidase"
"JS1_A09_00130" 10616 "s63E6H2" 0.6170013 "Formamidase"
"JS1_A09_00131" 10617 "s59E13H2" 0.1402885 "hypothetical protein"
"JS1_A09_00131" 10617 "s59E25H2" 0 "hypothetical protein"
"JS1_A09_00131" 10617 "s59E5H2" 0.9200855 "hypothetical protein"
"JS1_A09_00131" 10617 "s63E6H2" 1.404515 "hypothetical protein"
"JS1_A09_00132" 10618 "s59E13H2" 1.017925 "protease TldD"
"JS1_A09_00132" 10618 "s59E25H2" 0 "protease TldD"
"JS1_A09_00132" 10618 "s59E5H2" 0.1553686 "protease TldD"
"JS1_A09_00132" 10618 "s63E6H2" 1.1436 "protease TldD"
"JS1_A09_00133" 10619 "s59E13H2" 0.8544167 "protease TldD"
"JS1_A09_00133" 10619 "s59E25H2" 0.1298322 "protease TldD"
"JS1_A09_00133" 10619 "s59E5H2" 11.54644 "protease TldD"
"JS1_A09_00133" 10619 "s63E6H2" 0.7863701 "protease TldD"
"JS1_A09_00134" 10620 "s59E13H2" 0.06238657 "3'-5' exoribonuclease YhaM"
"JS1_A09_00134" 10620 "s59E25H2" 0 "3'-5' exoribonuclease YhaM"
"JS1_A09_00134" 10620 "s59E5H2" 0.03859389 "3'-5' exoribonuclease YhaM"
"JS1_A09_00134" 10620 "s63E6H2" 0.3867607 "3'-5' exoribonuclease YhaM"
"JS1_A09_00135" 10621 "s59E13H2" 0.6712499 "Valine--tRNA ligase"
"JS1_A09_00135" 10621 "s59E25H2" 0 "Valine--tRNA ligase"

"JS1_A09_00135" 10621 "s59E5H2" 0.4159561 "Valine--tRNA ligase"
"JS1_A09_00135" 10621 "s63E6H2" 0.1971546 "Valine--tRNA ligase"
"JS1_A09_00136" 10622 "s59E13H2" 0 "L-threonine dehydratase catabolic TdcB"
"JS1_A09_00136" 10622 "s59E25H2" 0 "L-threonine dehydratase catabolic TdcB"
"JS1_A09_00136" 10622 "s59E5H2" 0 "L-threonine dehydratase catabolic TdcB"
"JS1_A09_00136" 10622 "s63E6H2" 0.008322837 "L-threonine dehydratase catabolic TdcB"
"JS1_A09_00137" 10623 "s59E13H2" 0 "hypothetical protein"
"JS1_A09_00137" 10623 "s59E25H2" 0 "hypothetical protein"
"JS1_A09_00137" 10623 "s59E5H2" 0 "hypothetical protein"
"JS1_A09_00137" 10623 "s63E6H2" 0.02269488 "hypothetical protein"
"JS1_A09_00138" 10624 "s59E13H2" 0 "L-ribulose-5-phosphate 3-epimerase UlaE"
"JS1_A09_00138" 10624 "s59E25H2" 0 "L-ribulose-5-phosphate 3-epimerase UlaE"
"JS1_A09_00138" 10624 "s59E5H2" 0 "L-ribulose-5-phosphate 3-epimerase UlaE"
"JS1_A09_00138" 10624 "s63E6H2" 0.01151012 "L-ribulose-5-phosphate 3-epimerase UlaE"
"JS1_A09_00139" 10625 "s59E13H2" 0 "hypothetical protein"
"JS1_A09_00139" 10625 "s59E25H2" 0 "hypothetical protein"
"JS1_A09_00139" 10625 "s59E5H2" 0 "hypothetical protein"
"JS1_A09_00139" 10625 "s63E6H2" 0.00354271 "hypothetical protein"
"JS1_A09_00140" 10626 "s59E13H2" 0 "Aspartate aminotransferase"
"JS1_A09_00140" 10626 "s59E25H2" 0 "Aspartate aminotransferase"
"JS1_A09_00140" 10626 "s59E5H2" 0 "Aspartate aminotransferase"
"JS1_A09_00140" 10626 "s63E6H2" 0 "Aspartate aminotransferase"
"JS1_A09_00141" 10627 "s59E13H2" 0 "hypothetical protein"
"JS1_A09_00141" 10627 "s59E25H2" 0 "hypothetical protein"
"JS1_A09_00141" 10627 "s59E5H2" 0 "hypothetical protein"
"JS1_A09_00141" 10627 "s63E6H2" 0.0288734 "hypothetical protein"
"JS1_A09_00142" 10628 "s59E13H2" 0 "Sialic acid TRAP transporter permease protein SiaT"
"JS1_A09_00142" 10628 "s59E25H2" 0 "Sialic acid TRAP transporter permease protein SiaT"
"JS1_A09_00142" 10628 "s59E5H2" 0 "Sialic acid TRAP transporter permease protein SiaT"
"JS1_A09_00142" 10628 "s63E6H2" 0.0007118606 "Sialic acid TRAP transporter permease protein SiaT"
"JS1_A09_00143" 10629 "s59E13H2" 0 "2,3-diketo-L-gulonate TRAP transporter small permease protein YiaM"
"JS1_A09_00143" 10629 "s59E25H2" 0 "2,3-diketo-L-gulonate TRAP transporter small permease protein YiaM"
"JS1_A09_00143" 10629 "s59E5H2" 0 "2,3-diketo-L-gulonate TRAP transporter small permease protein YiaM"
"JS1_A09_00143" 10629 "s63E6H2" 0.0010562 "2,3-diketo-L-gulonate TRAP transporter small permease protein YiaM"
"JS1_A09_00144" 10630 "s59E13H2" 0 "2,3-diketo-L-gulonate-binding periplasmic protein YiaO precursor"
"JS1_A09_00144" 10630 "s59E25H2" 0 "2,3-diketo-L-gulonate-binding periplasmic protein YiaO precursor"
"JS1_A09_00144" 10630 "s59E5H2" 0 "2,3-diketo-L-gulonate-binding periplasmic protein YiaO precursor"
"JS1_A09_00144" 10630 "s63E6H2" 0.00295521 "2,3-diketo-L-gulonate-binding periplasmic protein YiaO precursor"
"JS1_A09_00145" 10631 "s59E13H2" 0 "Succinyl-CoA ligase [ADP-forming] subunit beta"
"JS1_A09_00145" 10631 "s59E25H2" 0 "Succinyl-CoA ligase [ADP-forming] subunit beta"
"JS1_A09_00145" 10631 "s59E5H2" 0 "Succinyl-CoA ligase [ADP-forming] subunit beta"
"JS1_A09_00145" 10631 "s63E6H2" 0 "Succinyl-CoA ligase [ADP-forming] subunit beta"
"JS1_A09_00146" 10632 "s59E13H2" 0.01299327 "Succinyl-CoA ligase [ADP-forming] subunit alpha"
"JS1_A09_00146" 10632 "s59E25H2" 0 "Succinyl-CoA ligase [ADP-forming] subunit alpha"
"JS1_A09_00146" 10632 "s59E5H2" 0.7501846 "Succinyl-CoA ligase [ADP-forming] subunit alpha"
"JS1_A09_00146" 10632 "s63E6H2" 0.06980463 "Succinyl-CoA ligase [ADP-forming] subunit alpha"
"JS1_A09_00147" 10633 "s59E13H2" 0.007867015 "Succinyl-CoA ligase [ADP-forming] subunit beta"

"JS1_A09_00147" 10633 "s59E25H2" 0 "Succinyl-CoA ligase [ADP-forming] subunit beta"
"JS1_A09_00147" 10633 "s59E5H2" 0.9772507 "Succinyl-CoA ligase [ADP-forming] subunit beta"
"JS1_A09_00147" 10633 "s63E6H2" 0.0581795 "Succinyl-CoA ligase [ADP-forming] subunit beta"
"JS1_A09_00148" 10634 "s59E13H2" 0.02045947 "hypothetical protein"
"JS1_A09_00148" 10634 "s59E25H2" 0 "hypothetical protein"
"JS1_A09_00148" 10634 "s59E5H2" 0.04567765 "hypothetical protein"
"JS1_A09_00148" 10634 "s63E6H2" 0.3780935 "hypothetical protein"
"JS1_A09_00149" 10635 "s59E13H2" 0.0298502 "SigmaW regulon antibacterial"
"JS1_A09_00149" 10635 "s59E25H2" 0 "SigmaW regulon antibacterial"
"JS1_A09_00149" 10635 "s59E5H2" 0.2127356 "SigmaW regulon antibacterial"
"JS1_A09_00149" 10635 "s63E6H2" 0.9733246 "SigmaW regulon antibacterial"
"JS1_A09_00150" 10636 "s59E13H2" 0.07763707 "hypothetical protein"
"JS1_A09_00150" 10636 "s59E25H2" 0 "hypothetical protein"
"JS1_A09_00150" 10636 "s59E5H2" 0.2911584 "hypothetical protein"
"JS1_A09_00150" 10636 "s63E6H2" 0.9088606 "hypothetical protein"
"JS1_A09_00151" 10637 "s59E13H2" 0.0501308 "glutamyl-tRNA(Gln) amidotransferase subunit E"
"JS1_A09_00151" 10637 "s59E25H2" 0 "glutamyl-tRNA(Gln) amidotransferase subunit E"
"JS1_A09_00151" 10637 "s59E5H2" 0.2763269 "glutamyl-tRNA(Gln) amidotransferase subunit E"
"JS1_A09_00151" 10637 "s63E6H2" 0.07283457 "glutamyl-tRNA(Gln) amidotransferase subunit E"
"JS1_A09_00152" 10638 "s59E13H2" 0.04902037 "30S ribosomal protein S21"
"JS1_A09_00152" 10638 "s59E25H2" 0 "30S ribosomal protein S21"
"JS1_A09_00152" 10638 "s59E5H2" 0.3167721 "30S ribosomal protein S21"
"JS1_A09_00152" 10638 "s63E6H2" 0.1865924 "30S ribosomal protein S21"
"JS1_A09_00153" 10639 "s59E13H2" 0.04361554 "N-6 DNA Methylase"
"JS1_A09_00153" 10639 "s59E25H2" 0 "N-6 DNA Methylase"
"JS1_A09_00153" 10639 "s59E5H2" 0.3602758 "N-6 DNA Methylase"
"JS1_A09_00153" 10639 "s63E6H2" 0.4035224 "N-6 DNA Methylase"
"JS1_A09_00155" 10641 "s59E13H2" 0.002596134 "Sulfide dehydrogenase subunit alpha precursor"
"JS1_A09_00155" 10641 "s59E25H2" 0 "Sulfide dehydrogenase subunit alpha precursor"
"JS1_A09_00155" 10641 "s59E5H2" 0.0009072787 "Sulfide dehydrogenase subunit alpha precursor"
"JS1_A09_00155" 10641 "s63E6H2" 0.002447981 "Sulfide dehydrogenase subunit alpha precursor"
"JS1_A09_00156" 10642 "s59E13H2" 0.04624859 "F420-non-reducing hydrogenase iron-sulfur subunit D"
"JS1_A09_00156" 10642 "s59E25H2" 0 "F420-non-reducing hydrogenase iron-sulfur subunit D"
"JS1_A09_00156" 10642 "s59E5H2" 0 "F420-non-reducing hydrogenase iron-sulfur subunit D"
"JS1_A09_00156" 10642 "s63E6H2" 0.004026196 "F420-non-reducing hydrogenase iron-sulfur subunit D"
"JS1_A09_00157" 10643 "s59E13H2" 0.0435319 "Formate dehydrogenase subunit beta"
"JS1_A09_00157" 10643 "s59E25H2" 0 "Formate dehydrogenase subunit beta"
"JS1_A09_00157" 10643 "s59E5H2" 0 "Formate dehydrogenase subunit beta"
"JS1_A09_00157" 10643 "s63E6H2" 0.002123854 "Formate dehydrogenase subunit beta"
"JS1_A09_00158" 10644 "s59E13H2" 0.04350613 "Sulfhydrogenase 2 subunit beta"
"JS1_A09_00158" 10644 "s59E25H2" 0 "Sulfhydrogenase 2 subunit beta"
"JS1_A09_00158" 10644 "s59E5H2" 0 "Sulfhydrogenase 2 subunit beta"
"JS1_A09_00158" 10644 "s63E6H2" 0 "Sulfhydrogenase 2 subunit beta"
"JS1_A09_00159" 10645 "s59E13H2" 0.01152659 "Sulfide dehydrogenase subunit alpha precursor"
"JS1_A09_00159" 10645 "s59E25H2" 0 "Sulfide dehydrogenase subunit alpha precursor"
"JS1_A09_00159" 10645 "s59E5H2" 0.01736843 "Sulfide dehydrogenase subunit alpha precursor"
"JS1_A09_00159" 10645 "s63E6H2" 0.01400238 "Sulfide dehydrogenase subunit alpha precursor"

"JS1_A09_00160" 10646 "s59E13H2" 0 "CoB--CoM heterodisulfide reductase subunit B"
"JS1_A09_00160" 10646 "s59E25H2" 0 "CoB--CoM heterodisulfide reductase subunit B"
"JS1_A09_00160" 10646 "s59E5H2" 0.01115423 "CoB--CoM heterodisulfide reductase subunit B"
"JS1_A09_00160" 10646 "s63E6H2" 0 "CoB--CoM heterodisulfide reductase subunit B"
"JS1_A09_00161" 10647 "s59E13H2" 0 "hypothetical protein"
"JS1_A09_00161" 10647 "s59E25H2" 0 "hypothetical protein"
"JS1_A09_00161" 10647 "s59E5H2" 0 "hypothetical protein"
"JS1_A09_00161" 10647 "s63E6H2" 0 "hypothetical protein"
"JS1_A09_00163" 10649 "s59E13H2" 0 "DctM-like transporters"
"JS1_A09_00163" 10649 "s59E25H2" 0 "DctM-like transporters"
"JS1_A09_00163" 10649 "s59E5H2" 0 "DctM-like transporters"
"JS1_A09_00163" 10649 "s63E6H2" 0 "DctM-like transporters"
"JS1_A09_00164" 10650 "s59E13H2" 0.04266845 "DNA replication protein DnaC"
"JS1_A09_00164" 10650 "s59E25H2" 0 "DNA replication protein DnaC"
"JS1_A09_00164" 10650 "s59E5H2" 0.06595822 "DNA replication protein DnaC"
"JS1_A09_00164" 10650 "s63E6H2" 0.02332029 "DNA replication protein DnaC"
"JS1_A09_00166" 10652 "s59E13H2" 0.001882372 "Ferrous-iron efflux pump FieF"
"JS1_A09_00166" 10652 "s59E25H2" 0 "Ferrous-iron efflux pump FieF"
"JS1_A09_00166" 10652 "s59E5H2" 0.08391221 "Ferrous-iron efflux pump FieF"
"JS1_A09_00166" 10652 "s63E6H2" 0.02756747 "Ferrous-iron efflux pump FieF"
"JS1_A09_00168" 10654 "s59E13H2" 4.376177 "HTH-type transcriptional repressor YtrA"
"JS1_A09_00168" 10654 "s59E25H2" 0 "HTH-type transcriptional repressor YtrA"
"JS1_A09_00168" 10654 "s59E5H2" 1.853588 "HTH-type transcriptional repressor YtrA"
"JS1_A09_00168" 10654 "s63E6H2" 3.227826 "HTH-type transcriptional repressor YtrA"
"JS1_A09_00169" 10655 "s59E13H2" 5.748571 "ABC transporter ATP-binding protein YtrB"
"JS1_A09_00169" 10655 "s59E25H2" 0 "ABC transporter ATP-binding protein YtrB"
"JS1_A09_00169" 10655 "s59E5H2" 4.893721 "ABC transporter ATP-binding protein YtrB"
"JS1_A09_00169" 10655 "s63E6H2" 3.460686 "ABC transporter ATP-binding protein YtrB"
"JS1_A09_00170" 10656 "s59E13H2" 7.565338 "hypothetical protein"
"JS1_A09_00170" 10656 "s59E25H2" 0 "hypothetical protein"
"JS1_A09_00170" 10656 "s59E5H2" 5.043265 "hypothetical protein"
"JS1_A09_00170" 10656 "s63E6H2" 1.590437 "hypothetical protein"
"JS1_A09_00171" 10657 "s59E13H2" 7.612231 "hypothetical protein"
"JS1_A09_00171" 10657 "s59E25H2" 0.07407407 "hypothetical protein"
"JS1_A09_00171" 10657 "s59E5H2" 5.845981 "hypothetical protein"
"JS1_A09_00171" 10657 "s63E6H2" 2.521442 "hypothetical protein"
"JS1_A09_00172" 10658 "s59E13H2" 7.299792 "hypothetical protein"
"JS1_A09_00172" 10658 "s59E25H2" 1.294118 "hypothetical protein"
"JS1_A09_00172" 10658 "s59E5H2" 4.157197 "hypothetical protein"
"JS1_A09_00172" 10658 "s63E6H2" 2.263403 "hypothetical protein"
"JS1_A09_00173" 10659 "s59E13H2" 1.674165 "CGGC domain protein"
"JS1_A09_00173" 10659 "s59E25H2" 0.1044444 "CGGC domain protein"
"JS1_A09_00173" 10659 "s59E5H2" 2.46739 "CGGC domain protein"
"JS1_A09_00173" 10659 "s63E6H2" 0.2623416 "CGGC domain protein"
"JS1_A09_00174" 10660 "s59E13H2" 4.57348 "Pyruvate kinase"
"JS1_A09_00174" 10660 "s59E25H2" 0 "Pyruvate kinase"
"JS1_A09_00174" 10660 "s59E5H2" 2.978021 "Pyruvate kinase"

"JS1_A09_00174" 10660 "s63E6H2" 2.703454 "Pyruvate kinase"
"JS1_A09_00175" 10661 "s59E13H2" 0.02995808 "Trifunctional nucleotide phosphoesterase protein YfkN precursor"
"JS1_A09_00175" 10661 "s59E25H2" 0 "Trifunctional nucleotide phosphoesterase protein YfkN precursor"
"JS1_A09_00175" 10661 "s59E5H2" 0.1588634 "Trifunctional nucleotide phosphoesterase protein YfkN precursor"
"JS1_A09_00175" 10661 "s63E6H2" 0 "Trifunctional nucleotide phosphoesterase protein YfkN precursor"
"JS1_A09_00176" 10662 "s59E13H2" 0.02270796 "hypothetical protein"
"JS1_A09_00176" 10662 "s59E25H2" 0 "hypothetical protein"
"JS1_A09_00176" 10662 "s59E5H2" 0.575437 "hypothetical protein"
"JS1_A09_00176" 10662 "s63E6H2" 0.007509711 "hypothetical protein"
"JS1_A09_00177" 10663 "s59E13H2" 0.154713 "hypothetical protein"
"JS1_A09_00177" 10663 "s59E25H2" 0 "hypothetical protein"
"JS1_A09_00177" 10663 "s59E5H2" 0.006924193 "hypothetical protein"
"JS1_A09_00177" 10663 "s63E6H2" 0.3450839 "hypothetical protein"
"JS1_A09_00178" 10664 "s59E13H2" 0.0180132 "hypothetical protein"
"JS1_A09_00178" 10664 "s59E25H2" 0 "hypothetical protein"
"JS1_A09_00178" 10664 "s59E5H2" 0 "hypothetical protein"
"JS1_A09_00178" 10664 "s63E6H2" 0.00602733 "hypothetical protein"
"JS1_A09_00179" 10665 "s59E13H2" 0.003679658 "hypothetical protein"
"JS1_A09_00179" 10665 "s59E25H2" 0 "hypothetical protein"
"JS1_A09_00179" 10665 "s59E5H2" 0 "hypothetical protein"
"JS1_A09_00179" 10665 "s63E6H2" 0.08008179 "hypothetical protein"
"JS1_A09_00180" 10666 "s59E13H2" 0.1532037 "hypothetical protein"
"JS1_A09_00180" 10666 "s59E25H2" 0 "hypothetical protein"
"JS1_A09_00180" 10666 "s59E5H2" 0.7303 "hypothetical protein"
"JS1_A09_00180" 10666 "s63E6H2" 0.7969659 "hypothetical protein"
"JS1_A09_00181" 10667 "s59E13H2" 0.1343038 "Galactose-1-phosphate uridylyltransferase"
"JS1_A09_00181" 10667 "s59E25H2" 0 "Galactose-1-phosphate uridylyltransferase"
"JS1_A09_00181" 10667 "s59E5H2" 0.1645563 "Galactose-1-phosphate uridylyltransferase"
"JS1_A09_00181" 10667 "s63E6H2" 0.2342051 "Galactose-1-phosphate uridylyltransferase"
"JS1_A09_00182" 10668 "s59E13H2" 0.1011513 "6-phosphofructokinase"
"JS1_A09_00182" 10668 "s59E25H2" 0 "6-phosphofructokinase"
"JS1_A09_00182" 10668 "s59E5H2" 0.1822561 "6-phosphofructokinase"
"JS1_A09_00182" 10668 "s63E6H2" 0.3603017 "6-phosphofructokinase"
"JS1_A09_00183" 10669 "s59E13H2" 0.03646798 "Transposase IS200 like protein"
"JS1_A09_00183" 10669 "s59E25H2" 0 "Transposase IS200 like protein"
"JS1_A09_00183" 10669 "s59E5H2" 0 "Transposase IS200 like protein"
"JS1_A09_00183" 10669 "s63E6H2" 0.01617482 "Transposase IS200 like protein"
"JS1_A09_00184" 10670 "s59E13H2" 0.2863071 "Transcription-repair-coupling factor"
"JS1_A09_00184" 10670 "s59E25H2" 0 "Transcription-repair-coupling factor"
"JS1_A09_00184" 10670 "s59E5H2" 0.4249814 "Transcription-repair-coupling factor"
"JS1_A09_00184" 10670 "s63E6H2" 0.4059342 "Transcription-repair-coupling factor"
"JS1_A09_00185" 10671 "s59E13H2" 0 "NTE family protein RssA"
"JS1_A09_00185" 10671 "s59E25H2" 0 "NTE family protein RssA"
"JS1_A09_00185" 10671 "s59E5H2" 0.2749819 "NTE family protein RssA"
"JS1_A09_00185" 10671 "s63E6H2" 0.001576112 "NTE family protein RssA"
"JS1_A09_00186" 10672 "s59E13H2" 0.01841432 "Type I phosphodiesterase / nucleotide pyrophosphatase"
"JS1_A09_00186" 10672 "s59E25H2" 0 "Type I phosphodiesterase / nucleotide pyrophosphatase"

"JS1_A09_00186" 10672 "s59E5H2" 0.007345027 "Type I phosphodiesterase / nucleotide pyrophosphatase"
"JS1_A09_00186" 10672 "s63E6H2" 0.05036446 "Type I phosphodiesterase / nucleotide pyrophosphatase"
"JS1_A09_00187" 10673 "s59E13H2" 0.1454503 "Nucleoside triphosphate pyrophosphohydrolase/pyrophosphatase MazG"
"JS1_A09_00187" 10673 "s59E25H2" 0 "Nucleoside triphosphate pyrophosphohydrolase/pyrophosphatase MazG"
"JS1_A09_00187" 10673 "s59E5H2" 0.3649389 "Nucleoside triphosphate pyrophosphohydrolase/pyrophosphatase MazG"
"JS1_A09_00187" 10673 "s63E6H2" 0.1102582 "Nucleoside triphosphate pyrophosphohydrolase/pyrophosphatase MazG"
"JS1_A09_00188" 10674 "s59E13H2" 0.111271 "AsmA family protein"
"JS1_A09_00188" 10674 "s59E25H2" 0 "AsmA family protein"
"JS1_A09_00188" 10674 "s59E5H2" 0.3223758 "AsmA family protein"
"JS1_A09_00188" 10674 "s63E6H2" 0.3218818 "AsmA family protein"
"JS1_A09_00189" 10675 "s59E13H2" 0.2969744 "Outer membrane protein assembly factor BamA precursor"
"JS1_A09_00189" 10675 "s59E25H2" 0 "Outer membrane protein assembly factor BamA precursor"
"JS1_A09_00189" 10675 "s59E5H2" 0.8057379 "Outer membrane protein assembly factor BamA precursor"
"JS1_A09_00189" 10675 "s63E6H2" 0.543274 "Outer membrane protein assembly factor BamA precursor"
"JS1_A09_00190" 10676 "s59E13H2" 0.350048 "Outer membrane protein (OmpH-like)"
"JS1_A09_00190" 10676 "s59E25H2" 0 "Outer membrane protein (OmpH-like)"
"JS1_A09_00190" 10676 "s59E5H2" 0.8472456 "Outer membrane protein (OmpH-like)"
"JS1_A09_00190" 10676 "s63E6H2" 1.365299 "Outer membrane protein (OmpH-like)"
"JS1_A09_00191" 10677 "s59E13H2" 0.6057689 "3-hydroxyacyl-[acyl-carrier-protein] dehydratase FabZ"
"JS1_A09_00191" 10677 "s59E25H2" 0 "3-hydroxyacyl-[acyl-carrier-protein] dehydratase FabZ"
"JS1_A09_00191" 10677 "s59E5H2" 1.009133 "3-hydroxyacyl-[acyl-carrier-protein] dehydratase FabZ"
"JS1_A09_00191" 10677 "s63E6H2" 1.419521 "3-hydroxyacyl-[acyl-carrier-protein] dehydratase FabZ"
"JS1_A09_00192" 10678 "s59E13H2" 0.5726194 "Lipopolysaccharide export system ATP-binding protein LptB"
"JS1_A09_00192" 10678 "s59E25H2" 0 "Lipopolysaccharide export system ATP-binding protein LptB"
"JS1_A09_00192" 10678 "s59E5H2" 0.8644015 "Lipopolysaccharide export system ATP-binding protein LptB"
"JS1_A09_00192" 10678 "s63E6H2" 1.718244 "Lipopolysaccharide export system ATP-binding protein LptB"
"JS1_A09_00193" 10679 "s59E13H2" 0.2231464 "hypothetical protein"
"JS1_A09_00193" 10679 "s59E25H2" 0 "hypothetical protein"
"JS1_A09_00193" 10679 "s59E5H2" 0.3625029 "hypothetical protein"
"JS1_A09_00193" 10679 "s63E6H2" 0.7676543 "hypothetical protein"
"JS1_A09_00194" 10680 "s59E13H2" 0.1357512 "Outer membrane protein alpha precursor"
"JS1_A09_00194" 10680 "s59E25H2" 0.008865248 "Outer membrane protein alpha precursor"
"JS1_A09_00194" 10680 "s59E5H2" 0.3841376 "Outer membrane protein alpha precursor"
"JS1_A09_00194" 10680 "s63E6H2" 0.4971113 "Outer membrane protein alpha precursor"
"JS1_A09_00203" 10689 "s59E13H2" 0 "hypothetical protein"
"JS1_A09_00203" 10689 "s59E25H2" 0 "hypothetical protein"
"JS1_A09_00203" 10689 "s59E5H2" 0 "hypothetical protein"
"JS1_A09_00203" 10689 "s63E6H2" 0.00260818 "hypothetical protein"
"JS1_A09_00204" 10690 "s59E13H2" 5.09867 "50S ribosomal protein L28"
"JS1_A09_00204" 10690 "s59E25H2" 0 "50S ribosomal protein L28"
"JS1_A09_00204" 10690 "s59E5H2" 0.1063627 "50S ribosomal protein L28"
"JS1_A09_00204" 10690 "s63E6H2" 0.3459084 "50S ribosomal protein L28"
"JS1_A09_00205" 10691 "s59E13H2" 6.133039 "4-hydroxy-3-methylbut-2-enyl diphosphate reductase"
"JS1_A09_00205" 10691 "s59E25H2" 0 "4-hydroxy-3-methylbut-2-enyl diphosphate reductase"
"JS1_A09_00205" 10691 "s59E5H2" 0.1426858 "4-hydroxy-3-methylbut-2-enyl diphosphate reductase"
"JS1_A09_00205" 10691 "s63E6H2" 0.4145285 "4-hydroxy-3-methylbut-2-enyl diphosphate reductase"
"JS1_A09_00206" 10692 "s59E13H2" 5.036201 "Cytidylate kinase"

"JS1_A09_00206" 10692 "s59E25H2" 0.1082982 "Cytidylate kinase"
"JS1_A09_00206" 10692 "s59E5H2" 0.04510099 "Cytidylate kinase"
"JS1_A09_00206" 10692 "s63E6H2" 0.1426025 "Cytidylate kinase"
"JS1_A09_00207" 10693 "s59E13H2" 4.053673 "Ribosomal large subunit pseudouridine synthase B"
"JS1_A09_00207" 10693 "s59E25H2" 0 "Ribosomal large subunit pseudouridine synthase B"
"JS1_A09_00207" 10693 "s59E5H2" 0.03635358 "Ribosomal large subunit pseudouridine synthase B"
"JS1_A09_00207" 10693 "s63E6H2" 0.08467462 "Ribosomal large subunit pseudouridine synthase B"
"JS1_A09_00208" 10694 "s59E13H2" 5.110618 "Segregation and condensation protein B"
"JS1_A09_00208" 10694 "s59E25H2" 0 "Segregation and condensation protein B"
"JS1_A09_00208" 10694 "s59E5H2" 0.2101484 "Segregation and condensation protein B"
"JS1_A09_00208" 10694 "s63E6H2" 0.2173692 "Segregation and condensation protein B"
"JS1_A09_00209" 10695 "s59E13H2" 1.343156 "Segregation and condensation protein A"
"JS1_A09_00209" 10695 "s59E25H2" 0 "Segregation and condensation protein A"
"JS1_A09_00209" 10695 "s59E5H2" 0.4332509 "Segregation and condensation protein A"
"JS1_A09_00209" 10695 "s63E6H2" 0.0861118 "Segregation and condensation protein A"
"JS1_A09_00210" 10696 "s59E13H2" 0.08382652 "Tryptophan--tRNA ligase"
"JS1_A09_00210" 10696 "s59E25H2" 0 "Tryptophan--tRNA ligase"
"JS1_A09_00210" 10696 "s59E5H2" 0.2406301 "Tryptophan--tRNA ligase"
"JS1_A09_00210" 10696 "s63E6H2" 0.06681059 "Tryptophan--tRNA ligase"
"JS1_A09_00211" 10697 "s59E13H2" 0.04903263 "Peptidase family M50"
"JS1_A09_00211" 10697 "s59E25H2" 0 "Peptidase family M50"
"JS1_A09_00211" 10697 "s59E5H2" 0.2475041 "Peptidase family M50"
"JS1_A09_00211" 10697 "s63E6H2" 0.06687218 "Peptidase family M50"
"JS1_A09_00212" 10698 "s59E13H2" 0.03764547 "Multifunctional CCA protein"
"JS1_A09_00212" 10698 "s59E25H2" 0 "Multifunctional CCA protein"
"JS1_A09_00212" 10698 "s59E5H2" 0.232831 "Multifunctional CCA protein"
"JS1_A09_00212" 10698 "s63E6H2" 0.06649957 "Multifunctional CCA protein"
"JS1_A09_00214" 10700 "s59E13H2" 3.317645 "Integrase core domain protein"
"JS1_A09_00214" 10700 "s59E25H2" 0 "Integrase core domain protein"
"JS1_A09_00214" 10700 "s59E5H2" 1.653472 "Integrase core domain protein"
"JS1_A09_00214" 10700 "s63E6H2" 0.4986216 "Integrase core domain protein"
"JS1_A09_00216" 10702 "s59E13H2" 0.007893234 "TPR repeat-containing protein YrrB"
"JS1_A09_00216" 10702 "s59E25H2" 0 "TPR repeat-containing protein YrrB"
"JS1_A09_00216" 10702 "s59E5H2" 0.04095847 "TPR repeat-containing protein YrrB"
"JS1_A09_00216" 10702 "s63E6H2" 0.3043906 "TPR repeat-containing protein YrrB"
"JS1_A09_00217" 10703 "s59E13H2" 0 "putative major pilin subunit"
"JS1_A09_00217" 10703 "s59E25H2" 0 "putative major pilin subunit"
"JS1_A09_00217" 10703 "s59E5H2" 0 "putative major pilin subunit"
"JS1_A09_00217" 10703 "s63E6H2" 0.07466003 "putative major pilin subunit"
"JS1_A09_00219" 10705 "s59E13H2" 0.127188 "hypothetical protein"
"JS1_A09_00219" 10705 "s59E25H2" 0 "hypothetical protein"
"JS1_A09_00219" 10705 "s59E5H2" 0.01493795 "hypothetical protein"
"JS1_A09_00219" 10705 "s63E6H2" 0.4408353 "hypothetical protein"
"JS1_A09_00220" 10706 "s59E13H2" 0.0439467 "Toxin RelE"
"JS1_A09_00220" 10706 "s59E25H2" 0 "Toxin RelE"
"JS1_A09_00220" 10706 "s59E5H2" 0.0190365 "Toxin RelE"
"JS1_A09_00220" 10706 "s63E6H2" 0.2527971 "Toxin RelE"

"JS1_A09_00222" 10708 "s59E13H2" 0.06342838 "hypothetical protein"
"JS1_A09_00222" 10708 "s59E25H2" 0 "hypothetical protein"
"JS1_A09_00222" 10708 "s59E5H2" 0.1657457 "hypothetical protein"
"JS1_A09_00222" 10708 "s63E6H2" 0.01267966 "hypothetical protein"
"JS1_A09_00225" 10711 "s59E13H2" 0.03360699 "hypothetical protein"
"JS1_A09_00225" 10711 "s59E25H2" 0 "hypothetical protein"
"JS1_A09_00225" 10711 "s59E5H2" 0.7561108 "hypothetical protein"
"JS1_A09_00225" 10711 "s63E6H2" 0.4719842 "hypothetical protein"
"JS1_A09_00226" 10712 "s59E13H2" 0.1555777 "hypothetical protein"
"JS1_A09_00226" 10712 "s59E25H2" 0 "hypothetical protein"
"JS1_A09_00226" 10712 "s59E5H2" 0.1361485 "hypothetical protein"
"JS1_A09_00226" 10712 "s63E6H2" 0.009456673 "hypothetical protein"
"JS1_A09_00227" 10713 "s59E13H2" 0.1662763 "AIR carboxylase"
"JS1_A09_00227" 10713 "s59E25H2" 0 "AIR carboxylase"
"JS1_A09_00227" 10713 "s59E5H2" 0.129986 "AIR carboxylase"
"JS1_A09_00227" 10713 "s63E6H2" 0.0223455 "AIR carboxylase"
"JS1_A09_00229" 10715 "s59E13H2" 0 "CoB--CoM heterodisulfide reductase iron-sulfur subunit A"
"JS1_A09_00229" 10715 "s59E25H2" 0 "CoB--CoM heterodisulfide reductase iron-sulfur subunit A"
"JS1_A09_00229" 10715 "s59E5H2" 0 "CoB--CoM heterodisulfide reductase iron-sulfur subunit A"
"JS1_A09_00229" 10715 "s63E6H2" 0 "CoB--CoM heterodisulfide reductase iron-sulfur subunit A"
"JS1_A09_00230" 10716 "s59E13H2" 2.234266 "putative zinc-type alcohol dehydrogenase-like protein YjmD"
"JS1_A09_00230" 10716 "s59E25H2" 0.1360544 "putative zinc-type alcohol dehydrogenase-like protein YjmD"
"JS1_A09_00230" 10716 "s59E5H2" 0 "putative zinc-type alcohol dehydrogenase-like protein YjmD"
"JS1_A09_00230" 10716 "s63E6H2" 0.002014614 "putative zinc-type alcohol dehydrogenase-like protein YjmD"
"JS1_A09_00231" 10717 "s59E13H2" 0.005262697 "Glucose-6-phosphate isomerase"
"JS1_A09_00231" 10717 "s59E25H2" 0 "Glucose-6-phosphate isomerase"
"JS1_A09_00231" 10717 "s59E5H2" 0 "Glucose-6-phosphate isomerase"
"JS1_A09_00231" 10717 "s63E6H2" 0.003581098 "Glucose-6-phosphate isomerase"
"JS1_A09_00232" 10718 "s59E13H2" 0 "putative L-threonine 3-dehydrogenase"
"JS1_A09_00232" 10718 "s59E25H2" 0 "putative L-threonine 3-dehydrogenase"
"JS1_A09_00232" 10718 "s59E5H2" 0.0008885625 "putative L-threonine 3-dehydrogenase"
"JS1_A09_00232" 10718 "s63E6H2" 0.07948637 "putative L-threonine 3-dehydrogenase"
"JS1_A09_00233" 10719 "s59E13H2" 0 "ureidoglycolate hydrolase"
"JS1_A09_00233" 10719 "s59E25H2" 0 "ureidoglycolate hydrolase"
"JS1_A09_00233" 10719 "s59E5H2" 0 "ureidoglycolate hydrolase"
"JS1_A09_00233" 10719 "s63E6H2" 0.01095027 "ureidoglycolate hydrolase"
"JS1_A09_00234" 10720 "s59E13H2" 0 "General stress protein 69"
"JS1_A09_00234" 10720 "s59E25H2" 0 "General stress protein 69"
"JS1_A09_00234" 10720 "s59E5H2" 0 "General stress protein 69"
"JS1_A09_00234" 10720 "s63E6H2" 0.003148093 "General stress protein 69"
"JS1_A09_00235" 10721 "s59E13H2" 0 "D-tagatose 3-epimerase"
"JS1_A09_00235" 10721 "s59E25H2" 0 "D-tagatose 3-epimerase"
"JS1_A09_00235" 10721 "s59E5H2" 0.004459171 "D-tagatose 3-epimerase"
"JS1_A09_00235" 10721 "s63E6H2" 0.00261452 "D-tagatose 3-epimerase"
"JS1_A09_00236" 10722 "s59E13H2" 0 "3-oxoacyl-[acyl-carrier-protein] reductase FabG"
"JS1_A09_00236" 10722 "s59E25H2" 0 "3-oxoacyl-[acyl-carrier-protein] reductase FabG"
"JS1_A09_00236" 10722 "s59E5H2" 0.03056075 "3-oxoacyl-[acyl-carrier-protein] reductase FabG"

"JS1_A09_00236" 10722 "s63E6H2" 0.0009671597 "3-oxoacyl-[acyl-carrier-protein] reductase FabG"
"JS1_A09_00237" 10723 "s59E13H2" 8.429853 "ferredoxin"
"JS1_A09_00237" 10723 "s59E25H2" 0 "ferredoxin"
"JS1_A09_00237" 10723 "s59E5H2" 0.3127859 "ferredoxin"
"JS1_A09_00237" 10723 "s63E6H2" 0.4536199 "ferredoxin"
"JS1_A09_00238" 10724 "s59E13H2" 0 "Ribose transport system permease protein RbsC"
"JS1_A09_00238" 10724 "s59E25H2" 0 "Ribose transport system permease protein RbsC"
"JS1_A09_00238" 10724 "s59E5H2" 0 "Ribose transport system permease protein RbsC"
"JS1_A09_00238" 10724 "s63E6H2" 0.005957946 "Ribose transport system permease protein RbsC"
"JS1_A09_00239" 10725 "s59E13H2" 0 "Ribose import ATP-binding protein RbsA"
"JS1_A09_00239" 10725 "s59E25H2" 0 "Ribose import ATP-binding protein RbsA"
"JS1_A09_00239" 10725 "s59E5H2" 0 "Ribose import ATP-binding protein RbsA"
"JS1_A09_00239" 10725 "s63E6H2" 0 "Ribose import ATP-binding protein RbsA"
"JS1_A09_00240" 10726 "s59E13H2" 0 "D-allose transport system permease protein AlsC"
"JS1_A09_00240" 10726 "s59E25H2" 0 "D-allose transport system permease protein AlsC"
"JS1_A09_00240" 10726 "s59E5H2" 0 "D-allose transport system permease protein AlsC"
"JS1_A09_00240" 10726 "s63E6H2" 0.001284191 "D-allose transport system permease protein AlsC"
"JS1_A09_00241" 10727 "s59E13H2" 0 "D-allose-binding periplasmic protein precursor"
"JS1_A09_00241" 10727 "s59E25H2" 0 "D-allose-binding periplasmic protein precursor"
"JS1_A09_00241" 10727 "s59E5H2" 0 "D-allose-binding periplasmic protein precursor"
"JS1_A09_00241" 10727 "s63E6H2" 0.001459964 "D-allose-binding periplasmic protein precursor"
"JS1_A09_00242" 10728 "s59E13H2" 0 "hypothetical protein"
"JS1_A09_00242" 10728 "s59E25H2" 0 "hypothetical protein"
"JS1_A09_00242" 10728 "s59E5H2" 0 "hypothetical protein"
"JS1_A09_00242" 10728 "s63E6H2" 0 "hypothetical protein"
"JS1_A09_00243" 10729 "s59E13H2" 0 "Putative alcohol dehydrogenase D"
"JS1_A09_00243" 10729 "s59E25H2" 0 "Putative alcohol dehydrogenase D"
"JS1_A09_00243" 10729 "s59E5H2" 0 "Putative alcohol dehydrogenase D"
"JS1_A09_00243" 10729 "s63E6H2" 0 "Putative alcohol dehydrogenase D"
"JS1_A09_00244" 10730 "s59E13H2" 0.002346691 "HTH-type transcriptional repressor YvoA"
"JS1_A09_00244" 10730 "s59E25H2" 0 "HTH-type transcriptional repressor YvoA"
"JS1_A09_00244" 10730 "s59E5H2" 0 "HTH-type transcriptional repressor YvoA"
"JS1_A09_00244" 10730 "s63E6H2" 0.01347945 "HTH-type transcriptional repressor YvoA"
"JS1_A09_00245" 10731 "s59E13H2" 0.006646455 "hypothetical protein"
"JS1_A09_00245" 10731 "s59E25H2" 0 "hypothetical protein"
"JS1_A09_00245" 10731 "s59E5H2" 0.1145029 "hypothetical protein"
"JS1_A09_00245" 10731 "s63E6H2" 0.03598039 "hypothetical protein"
"JS1_A09_00246" 10732 "s59E13H2" 0.07600474 "Lipoate-protein ligase A subunit 2"
"JS1_A09_00246" 10732 "s59E25H2" 0 "Lipoate-protein ligase A subunit 2"
"JS1_A09_00246" 10732 "s59E5H2" 0 "Lipoate-protein ligase A subunit 2"
"JS1_A09_00246" 10732 "s63E6H2" 0.1413075 "Lipoate-protein ligase A subunit 2"
"JS1_A09_00247" 10733 "s59E13H2" 0.002727683 "CoB--CoM heterodisulfide reductase iron-sulfur subunit C"
"JS1_A09_00247" 10733 "s59E25H2" 0 "CoB--CoM heterodisulfide reductase iron-sulfur subunit C"
"JS1_A09_00247" 10733 "s59E5H2" 0 "CoB--CoM heterodisulfide reductase iron-sulfur subunit C"
"JS1_A09_00247" 10733 "s63E6H2" 0.1171604 "CoB--CoM heterodisulfide reductase iron-sulfur subunit C"
"JS1_A09_00249" 10735 "s59E13H2" 1.027587 "colicin V production protein"
"JS1_A09_00249" 10735 "s59E25H2" 0 "colicin V production protein"

"JS1_A09_00249" 10735 "s59E5H2" 0.4143741 "colicin V production protein"
"JS1_A09_00249" 10735 "s63E6H2" 0.5837685 "colicin V production protein"
"JS1_A09_00250" 10736 "s59E13H2" 0.4212542 "Phenylalanine--tRNA ligase beta subunit"
"JS1_A09_00250" 10736 "s59E25H2" 0 "Phenylalanine--tRNA ligase beta subunit"
"JS1_A09_00250" 10736 "s59E5H2" 0.3111852 "Phenylalanine--tRNA ligase beta subunit"
"JS1_A09_00250" 10736 "s63E6H2" 0.3317641 "Phenylalanine--tRNA ligase beta subunit"
"JS1_A09_00251" 10737 "s59E13H2" 0.03582812 "Phenylalanine--tRNA ligase alpha subunit"
"JS1_A09_00251" 10737 "s59E25H2" 0 "Phenylalanine--tRNA ligase alpha subunit"
"JS1_A09_00251" 10737 "s59E5H2" 0.09110358 "Phenylalanine--tRNA ligase alpha subunit"
"JS1_A09_00251" 10737 "s63E6H2" 0.4271357 "Phenylalanine--tRNA ligase alpha subunit"
"JS1_A09_00253" 10739 "s59E13H2" 0.2847896 "50S ribosomal protein L20"
"JS1_A09_00253" 10739 "s59E25H2" 0 "50S ribosomal protein L20"
"JS1_A09_00253" 10739 "s59E5H2" 0.2940363 "50S ribosomal protein L20"
"JS1_A09_00253" 10739 "s63E6H2" 0.384613 "50S ribosomal protein L20"
"JS1_A09_00254" 10740 "s59E13H2" 0.02950857 "50S ribosomal protein L35"
"JS1_A09_00254" 10740 "s59E25H2" 0 "50S ribosomal protein L35"
"JS1_A09_00254" 10740 "s59E5H2" 0.3054028 "50S ribosomal protein L35"
"JS1_A09_00254" 10740 "s63E6H2" 0.3160616 "50S ribosomal protein L35"
"JS1_A09_00255" 10741 "s59E13H2" 0.09884821 "Translation initiation factor IF-3"
"JS1_A09_00255" 10741 "s59E25H2" 0 "Translation initiation factor IF-3"
"JS1_A09_00255" 10741 "s59E5H2" 0.2822749 "Translation initiation factor IF-3"
"JS1_A09_00255" 10741 "s63E6H2" 0.2949184 "Translation initiation factor IF-3"
"JS1_A09_00256" 10742 "s59E13H2" 0.09744371 "Threonine--tRNA ligase 2"
"JS1_A09_00256" 10742 "s59E25H2" 0 "Threonine--tRNA ligase 2"
"JS1_A09_00256" 10742 "s59E5H2" 0.1784815 "Threonine--tRNA ligase 2"
"JS1_A09_00256" 10742 "s63E6H2" 0.2621082 "Threonine--tRNA ligase 2"
"JS1_A09_00264" 10750 "s59E13H2" 0.05117885 "Single-stranded DNA-binding protein ssb"
"JS1_A09_00264" 10750 "s59E25H2" 0 "Single-stranded DNA-binding protein ssb"
"JS1_A09_00264" 10750 "s59E5H2" 0.3112508 "Single-stranded DNA-binding protein ssb"
"JS1_A09_00264" 10750 "s63E6H2" 0.2786681 "Single-stranded DNA-binding protein ssb"
"JS1_A09_00265" 10751 "s59E13H2" 0.06584739 "Leucine--tRNA ligase"
"JS1_A09_00265" 10751 "s59E25H2" 0.1830536 "Leucine--tRNA ligase"
"JS1_A09_00265" 10751 "s59E5H2" 0.9754513 "Leucine--tRNA ligase"
"JS1_A09_00265" 10751 "s63E6H2" 0.6577879 "Leucine--tRNA ligase"
"JS1_A09_00268" 10754 "s59E13H2" 0.1194043 "hypothetical protein"
"JS1_A09_00268" 10754 "s59E25H2" 0 "hypothetical protein"
"JS1_A09_00268" 10754 "s59E5H2" 0.6700138 "hypothetical protein"
"JS1_A09_00268" 10754 "s63E6H2" 1.157965 "hypothetical protein"
"JS1_A09_00269" 10755 "s59E13H2" 0.1404202 "Elongation factor G"
"JS1_A09_00269" 10755 "s59E25H2" 0.2366667 "Elongation factor G"
"JS1_A09_00269" 10755 "s59E5H2" 1.200324 "Elongation factor G"
"JS1_A09_00269" 10755 "s63E6H2" 1.220003 "Elongation factor G"
"JS1_A09_00270" 10756 "s59E13H2" 0.1607112 "Lipoyl synthase"
"JS1_A09_00270" 10756 "s59E25H2" 0 "Lipoyl synthase"
"JS1_A09_00270" 10756 "s59E5H2" 1.028807 "Lipoyl synthase"
"JS1_A09_00270" 10756 "s63E6H2" 0.8991157 "Lipoyl synthase"
"JS1_A09_00271" 10757 "s59E13H2" 0.482666 "Inosose isomerase"

"JS1_A09_00271" 10757 "s59E25H2" 0 "Inosose isomerase"
"JS1_A09_00271" 10757 "s59E5H2" 1.484842 "Inosose isomerase"
"JS1_A09_00271" 10757 "s63E6H2" 1.114507 "Inosose isomerase"
"JS1_A09_00272" 10758 "s59E13H2" 0.622352 "Thioredoxin reductase"
"JS1_A09_00272" 10758 "s59E25H2" 0 "Thioredoxin reductase"
"JS1_A09_00272" 10758 "s59E5H2" 0.6876396 "Thioredoxin reductase"
"JS1_A09_00272" 10758 "s63E6H2" 0.6358136 "Thioredoxin reductase"
"JS1_A09_00274" 10760 "s59E13H2" 2.569066 "L-aspartate oxidase"
"JS1_A09_00274" 10760 "s59E25H2" 0 "L-aspartate oxidase"
"JS1_A09_00274" 10760 "s59E5H2" 2.357025 "L-aspartate oxidase"
"JS1_A09_00274" 10760 "s63E6H2" 1.989326 "L-aspartate oxidase"
"JS1_A09_00275" 10761 "s59E13H2" 6.236725 "putative nicotinate-nucleotide pyrophosphorylase [carboxylating]"
"JS1_A09_00275" 10761 "s59E25H2" 0.7588652 "putative nicotinate-nucleotide pyrophosphorylase [carboxylating]"
"JS1_A09_00275" 10761 "s59E5H2" 3.573786 "putative nicotinate-nucleotide pyrophosphorylase [carboxylating]"
"JS1_A09_00275" 10761 "s63E6H2" 3.413385 "putative nicotinate-nucleotide pyrophosphorylase [carboxylating]"
"JS1_A09_00276" 10762 "s59E13H2" 8.04139 "Type III pantothenate kinase"
"JS1_A09_00276" 10762 "s59E25H2" 0 "Type III pantothenate kinase"
"JS1_A09_00276" 10762 "s59E5H2" 1.448787 "Type III pantothenate kinase"
"JS1_A09_00276" 10762 "s63E6H2" 3.675491 "Type III pantothenate kinase"
"JS1_A09_00277" 10763 "s59E13H2" 6.40838 "Pantothenic acid transporter PanT"
"JS1_A09_00277" 10763 "s59E25H2" 0 "Pantothenic acid transporter PanT"
"JS1_A09_00277" 10763 "s59E5H2" 4.056618 "Pantothenic acid transporter PanT"
"JS1_A09_00277" 10763 "s63E6H2" 4.396627 "Pantothenic acid transporter PanT"
"JS1_A09_00278" 10764 "s59E13H2" 7.968768 "V-type ATP synthase subunit D"
"JS1_A09_00278" 10764 "s59E25H2" 0 "V-type ATP synthase subunit D"
"JS1_A09_00278" 10764 "s59E5H2" 5.515085 "V-type ATP synthase subunit D"
"JS1_A09_00278" 10764 "s63E6H2" 4.400609 "V-type ATP synthase subunit D"
"JS1_A09_00279" 10765 "s59E13H2" 7.333316 "V-type sodium ATPase subunit B"
"JS1_A09_00279" 10765 "s59E25H2" 0.1259098 "V-type sodium ATPase subunit B"
"JS1_A09_00279" 10765 "s59E5H2" 5.394767 "V-type sodium ATPase subunit B"
"JS1_A09_00279" 10765 "s63E6H2" 3.471863 "V-type sodium ATPase subunit B"
"JS1_A09_00280" 10766 "s59E13H2" 5.296401 "V-type ATP synthase alpha chain"
"JS1_A09_00280" 10766 "s59E25H2" 0 "V-type ATP synthase alpha chain"
"JS1_A09_00280" 10766 "s59E5H2" 3.924266 "V-type ATP synthase alpha chain"
"JS1_A09_00280" 10766 "s63E6H2" 2.983511 "V-type ATP synthase alpha chain"
"JS1_A09_00281" 10767 "s59E13H2" 3.503963 "V-type ATP synthase subunit E"
"JS1_A09_00281" 10767 "s59E25H2" 0 "V-type ATP synthase subunit E"
"JS1_A09_00281" 10767 "s59E5H2" 3.154597 "V-type ATP synthase subunit E"
"JS1_A09_00281" 10767 "s63E6H2" 1.497184 "V-type ATP synthase subunit E"
"JS1_A09_00282" 10768 "s59E13H2" 1.627091 "V-type ATP synthase subunit F"
"JS1_A09_00282" 10768 "s59E25H2" 0 "V-type ATP synthase subunit F"
"JS1_A09_00282" 10768 "s59E5H2" 2.94403 "V-type ATP synthase subunit F"
"JS1_A09_00282" 10768 "s63E6H2" 1.42942 "V-type ATP synthase subunit F"
"JS1_A09_00283" 10769 "s59E13H2" 1.106636 "V-type ATP synthase subunit K"
"JS1_A09_00283" 10769 "s59E25H2" 0 "V-type ATP synthase subunit K"
"JS1_A09_00283" 10769 "s59E5H2" 1.91374 "V-type ATP synthase subunit K"
"JS1_A09_00283" 10769 "s63E6H2" 0.9348927 "V-type ATP synthase subunit K"

"JS1_A09_00284" 10770 "s59E13H2" 1.336591 "V-type ATP synthase subunit I"
"JS1_A09_00284" 10770 "s59E25H2" 0.1161103 "V-type ATP synthase subunit I"
"JS1_A09_00284" 10770 "s59E5H2" 1.992961 "V-type ATP synthase subunit I"
"JS1_A09_00284" 10770 "s63E6H2" 0.1888639 "V-type ATP synthase subunit I"
"JS1_A09_00285" 10771 "s59E13H2" 1.190117 "V-type ATP synthase subunit C"
"JS1_A09_00285" 10771 "s59E25H2" 0.4737345 "V-type ATP synthase subunit C"
"JS1_A09_00285" 10771 "s59E5H2" 1.159023 "V-type ATP synthase subunit C"
"JS1_A09_00285" 10771 "s63E6H2" 0.2923459 "V-type ATP synthase subunit C"
"JS1_A09_00286" 10772 "s59E13H2" 1.07261 "hypothetical protein"
"JS1_A09_00286" 10772 "s59E25H2" 0 "hypothetical protein"
"JS1_A09_00286" 10772 "s59E5H2" 0.1984007 "hypothetical protein"
"JS1_A09_00286" 10772 "s63E6H2" 0.1488521 "hypothetical protein"
"JS1_A09_00287" 10773 "s59E13H2" 0.9661208 "Transcriptional regulatory protein ZraR"
"JS1_A09_00287" 10773 "s59E25H2" 0 "Transcriptional regulatory protein ZraR"
"JS1_A09_00287" 10773 "s59E5H2" 0.9850976 "Transcriptional regulatory protein ZraR"
"JS1_A09_00287" 10773 "s63E6H2" 0.3232819 "Transcriptional regulatory protein ZraR"
"JS1_A09_00288" 10774 "s59E13H2" 0.8508968 "Sensor protein ZraS"
"JS1_A09_00288" 10774 "s59E25H2" 0 "Sensor protein ZraS"
"JS1_A09_00288" 10774 "s59E5H2" 0.6350823 "Sensor protein ZraS"
"JS1_A09_00288" 10774 "s63E6H2" 0.1696887 "Sensor protein ZraS"
"JS1_A09_00289" 10775 "s59E13H2" 0.5481007 "Putative serine protease HtrA"
"JS1_A09_00289" 10775 "s59E25H2" 0 "Putative serine protease HtrA"
"JS1_A09_00289" 10775 "s59E5H2" 0.1640226 "Putative serine protease HtrA"
"JS1_A09_00289" 10775 "s63E6H2" 0.06770783 "Putative serine protease HtrA"
"JS1_A09_00290" 10776 "s59E13H2" 0.1254056 "hypothetical protein"
"JS1_A09_00290" 10776 "s59E25H2" 0 "hypothetical protein"
"JS1_A09_00290" 10776 "s59E5H2" 0.04224604 "hypothetical protein"
"JS1_A09_00290" 10776 "s63E6H2" 0.1262768 "hypothetical protein"
"JS1_A09_00291" 10777 "s59E13H2" 0.1724503 "hypothetical protein"
"JS1_A09_00291" 10777 "s59E25H2" 0 "hypothetical protein"
"JS1_A09_00291" 10777 "s59E5H2" 0.07544691 "hypothetical protein"
"JS1_A09_00291" 10777 "s63E6H2" 0.09081078 "hypothetical protein"
"JS1_A09_00292" 10778 "s59E13H2" 0.333567 "hypothetical protein"
"JS1_A09_00292" 10778 "s59E25H2" 0 "hypothetical protein"
"JS1_A09_00292" 10778 "s59E5H2" 0.05522697 "hypothetical protein"
"JS1_A09_00292" 10778 "s63E6H2" 0.09018554 "hypothetical protein"
"JS1_A09_00293" 10779 "s59E13H2" 0.3613002 "Disulfide bond reductase DsbH precursor"
"JS1_A09_00293" 10779 "s59E25H2" 0 "Disulfide bond reductase DsbH precursor"
"JS1_A09_00293" 10779 "s59E5H2" 0.02367148 "Disulfide bond reductase DsbH precursor"
"JS1_A09_00293" 10779 "s63E6H2" 0.05873343 "Disulfide bond reductase DsbH precursor"
"JS1_A09_00294" 10780 "s59E13H2" 0.5225389 "Thiol:disulfide interchange protein DsbD precursor"
"JS1_A09_00294" 10780 "s59E25H2" 0 "Thiol:disulfide interchange protein DsbD precursor"
"JS1_A09_00294" 10780 "s59E5H2" 0.1066597 "Thiol:disulfide interchange protein DsbD precursor"
"JS1_A09_00294" 10780 "s63E6H2" 0.08828479 "Thiol:disulfide interchange protein DsbD precursor"
"JS1_A09_00295" 10781 "s59E13H2" 0.6438814 "NifU-like protein"
"JS1_A09_00295" 10781 "s59E25H2" 0 "NifU-like protein"
"JS1_A09_00295" 10781 "s59E5H2" 0.1381375 "NifU-like protein"

"JS1_A09_00295" 10781 "s63E6H2" 0.1000233 "NifU-like protein"
"JS1_A09_00296" 10782 "s59E13H2" 0.4158194 "putative cysteine desulfurase 2"
"JS1_A09_00296" 10782 "s59E25H2" 0 "putative cysteine desulfurase 2"
"JS1_A09_00296" 10782 "s59E5H2" 0.08651758 "putative cysteine desulfurase 2"
"JS1_A09_00296" 10782 "s63E6H2" 0.1300112 "putative cysteine desulfurase 2"
"JS1_A09_00297" 10783 "s59E13H2" 0.1993436 "hypothetical protein"
"JS1_A09_00297" 10783 "s59E25H2" 0 "hypothetical protein"
"JS1_A09_00297" 10783 "s59E5H2" 0.06519682 "hypothetical protein"
"JS1_A09_00297" 10783 "s63E6H2" 0.03372038 "hypothetical protein"
"JS1_A09_00298" 10784 "s59E13H2" 0.1557832 "Acylphosphatase"
"JS1_A09_00298" 10784 "s59E25H2" 0 "Acylphosphatase"
"JS1_A09_00298" 10784 "s59E5H2" 0.02444626 "Acylphosphatase"
"JS1_A09_00298" 10784 "s63E6H2" 0.01053744 "Acylphosphatase"
"JS1_A09_00299" 10785 "s59E13H2" 0.5882655 "Peptidase family M50"
"JS1_A09_00299" 10785 "s59E25H2" 0 "Peptidase family M50"
"JS1_A09_00299" 10785 "s59E5H2" 0.48365 "Peptidase family M50"
"JS1_A09_00299" 10785 "s63E6H2" 0.2980684 "Peptidase family M50"
"JS1_A09_00300" 10786 "s59E13H2" 17.74046 "Sodium:neurotransmitter symporter family protein"
"JS1_A09_00300" 10786 "s59E25H2" 0 "Sodium:neurotransmitter symporter family protein"
"JS1_A09_00300" 10786 "s59E5H2" 0.6184423 "Sodium:neurotransmitter symporter family protein"
"JS1_A09_00300" 10786 "s63E6H2" 1.515091 "Sodium:neurotransmitter symporter family protein"
"JS1_A09_00301" 10787 "s59E13H2" 8.139702 "TPR repeat-containing protein YfgC precursor"
"JS1_A09_00301" 10787 "s59E25H2" 0 "TPR repeat-containing protein YfgC precursor"
"JS1_A09_00301" 10787 "s59E5H2" 0.17562 "TPR repeat-containing protein YfgC precursor"
"JS1_A09_00301" 10787 "s63E6H2" 1.173843 "TPR repeat-containing protein YfgC precursor"
"JS1_A09_00302" 10788 "s59E13H2" 3.145291 "Alanine--tRNA ligase"
"JS1_A09_00302" 10788 "s59E25H2" 0.08699187 "Alanine--tRNA ligase"
"JS1_A09_00302" 10788 "s59E5H2" 0.2377062 "Alanine--tRNA ligase"
"JS1_A09_00302" 10788 "s63E6H2" 1.105442 "Alanine--tRNA ligase"
"JS1_A09_00303" 10789 "s59E13H2" 11.96015 "Bicarbonate transport ATP-binding protein CmpD"
"JS1_A09_00303" 10789 "s59E25H2" 0 "Bicarbonate transport ATP-binding protein CmpD"
"JS1_A09_00303" 10789 "s59E5H2" 0.2632189 "Bicarbonate transport ATP-binding protein CmpD"
"JS1_A09_00303" 10789 "s63E6H2" 0.7379078 "Bicarbonate transport ATP-binding protein CmpD"
"JS1_A09_00304" 10790 "s59E13H2" 16.54602 "beta-methylgalactoside transporter inner membrane component"
"JS1_A09_00304" 10790 "s59E25H2" 0 "beta-methylgalactoside transporter inner membrane component"
"JS1_A09_00304" 10790 "s59E5H2" 0.6283805 "beta-methylgalactoside transporter inner membrane component"
"JS1_A09_00304" 10790 "s63E6H2" 1.163801 "beta-methylgalactoside transporter inner membrane component"
"JS1_A09_00305" 10791 "s59E13H2" 7.687768 "ABC transporter substrate binding protein"
"JS1_A09_00305" 10791 "s59E25H2" 0 "ABC transporter substrate binding protein"
"JS1_A09_00305" 10791 "s59E5H2" 0.2942616 "ABC transporter substrate binding protein"
"JS1_A09_00305" 10791 "s63E6H2" 2.037876 "ABC transporter substrate binding protein"
"JS1_A09_00306" 10792 "s59E13H2" 1.822967 "putative deoxyribonuclease YcfH"
"JS1_A09_00306" 10792 "s59E25H2" 0 "putative deoxyribonuclease YcfH"
"JS1_A09_00306" 10792 "s59E5H2" 0.07605271 "putative deoxyribonuclease YcfH"
"JS1_A09_00306" 10792 "s63E6H2" 1.791733 "putative deoxyribonuclease YcfH"
"JS1_A09_00307" 10793 "s59E13H2" 4.62283 "hypothetical protein"
"JS1_A09_00307" 10793 "s59E25H2" 0 "hypothetical protein"

"JS1_A09_00307" 10793 "s59E5H2" 0.3638729 "hypothetical protein"
"JS1_A09_00307" 10793 "s63E6H2" 1.719835 "hypothetical protein"
"JS1_A09_00308" 10794 "s59E13H2" 1.202227 "hypothetical protein"
"JS1_A09_00308" 10794 "s59E25H2" 0 "hypothetical protein"
"JS1_A09_00308" 10794 "s59E5H2" 0.02360994 "hypothetical protein"
"JS1_A09_00308" 10794 "s63E6H2" 0.7206371 "hypothetical protein"
"JS1_A09_00310" 10796 "s59E13H2" 9.698486 "hypothetical protein"
"JS1_A09_00310" 10796 "s59E25H2" 0 "hypothetical protein"
"JS1_A09_00310" 10796 "s59E5H2" 0.4174313 "hypothetical protein"
"JS1_A09_00310" 10796 "s63E6H2" 2.320574 "hypothetical protein"
"JS1_A09_00311" 10797 "s59E13H2" 11.00566 "Major Facilitator Superfamily protein"
"JS1_A09_00311" 10797 "s59E25H2" 0 "Major Facilitator Superfamily protein"
"JS1_A09_00311" 10797 "s59E5H2" 0.5182066 "Major Facilitator Superfamily protein"
"JS1_A09_00311" 10797 "s63E6H2" 1.608665 "Major Facilitator Superfamily protein"
"JS1_A09_00312" 10798 "s59E13H2" 9.251134 "Fosmidomycin resistance protein"
"JS1_A09_00312" 10798 "s59E25H2" 0 "Fosmidomycin resistance protein"
"JS1_A09_00312" 10798 "s59E5H2" 0.6241651 "Fosmidomycin resistance protein"
"JS1_A09_00312" 10798 "s63E6H2" 1.745361 "Fosmidomycin resistance protein"
"JS1_A09_00313" 10799 "s59E13H2" 0.009223353 "hypothetical protein"
"JS1_A09_00313" 10799 "s59E25H2" 0 "hypothetical protein"
"JS1_A09_00313" 10799 "s59E5H2" 0.03108235 "hypothetical protein"
"JS1_A09_00313" 10799 "s63E6H2" 0.0464156 "hypothetical protein"
"JS1_A09_00314" 10800 "s59E13H2" 0.1767002 "hypothetical protein"
"JS1_A09_00314" 10800 "s59E25H2" 0 "hypothetical protein"
"JS1_A09_00314" 10800 "s59E5H2" 0.009233256 "hypothetical protein"
"JS1_A09_00314" 10800 "s63E6H2" 0.7726035 "hypothetical protein"
"JS1_A09_00315" 10801 "s59E13H2" 0.4009586 "hypothetical protein"
"JS1_A09_00315" 10801 "s59E25H2" 0 "hypothetical protein"
"JS1_A09_00315" 10801 "s59E5H2" 0 "hypothetical protein"
"JS1_A09_00315" 10801 "s63E6H2" 0.8089315 "hypothetical protein"
"JS1_A09_00316" 10802 "s59E13H2" 0.02724697 "hypothetical protein"
"JS1_A09_00316" 10802 "s59E25H2" 0 "hypothetical protein"
"JS1_A09_00316" 10802 "s59E5H2" 0.06748972 "hypothetical protein"
"JS1_A09_00316" 10802 "s63E6H2" 0.425358 "hypothetical protein"
"JS1_A09_00317" 10803 "s59E13H2" 1.607828 "ABC-2 type transporter"
"JS1_A09_00317" 10803 "s59E25H2" 0 "ABC-2 type transporter"
"JS1_A09_00317" 10803 "s59E5H2" 1.386113 "ABC-2 type transporter"
"JS1_A09_00317" 10803 "s63E6H2" 1.448022 "ABC-2 type transporter"
"JS1_A09_00318" 10804 "s59E13H2" 0.8406493 "putative ABC transporter ATP-binding protein YbhF"
"JS1_A09_00318" 10804 "s59E25H2" 0 "putative ABC transporter ATP-binding protein YbhF"
"JS1_A09_00318" 10804 "s59E5H2" 1.242935 "putative ABC transporter ATP-binding protein YbhF"
"JS1_A09_00318" 10804 "s63E6H2" 1.869719 "putative ABC transporter ATP-binding protein YbhF"
"JS1_A09_00319" 10805 "s59E13H2" 0 "hypothetical protein"
"JS1_A09_00319" 10805 "s59E25H2" 0 "hypothetical protein"
"JS1_A09_00319" 10805 "s59E5H2" 0 "hypothetical protein"
"JS1_A09_00319" 10805 "s63E6H2" 0 "hypothetical protein"
"JS1_A09_00320" 10806 "s59E13H2" 0 "hypothetical protein"

"JS1_A09_00320" 10806 "s59E25H2" 0 "hypothetical protein"
"JS1_A09_00320" 10806 "s59E5H2" 0 "hypothetical protein"
"JS1_A09_00320" 10806 "s63E6H2" 0.06991856 "hypothetical protein"
"JS1_A09_00321" 10807 "s59E13H2" 0.3718301 "haloacid dehalogenase-like hydrolase"
"JS1_A09_00321" 10807 "s59E25H2" 0.6218905 "haloacid dehalogenase-like hydrolase"
"JS1_A09_00321" 10807 "s59E5H2" 0.7890718 "haloacid dehalogenase-like hydrolase"
"JS1_A09_00321" 10807 "s63E6H2" 1.577016 "haloacid dehalogenase-like hydrolase"
"JS1_A09_00323" 10809 "s59E13H2" 0.1172948 "ATP synthase subunit beta"
"JS1_A09_00323" 10809 "s59E25H2" 0 "ATP synthase subunit beta"
"JS1_A09_00323" 10809 "s59E5H2" 1.554297 "ATP synthase subunit beta"
"JS1_A09_00323" 10809 "s63E6H2" 0.4600735 "ATP synthase subunit beta"
"JS1_A09_00324" 10810 "s59E13H2" 0.1553593 "F0F1 ATP synthase subunit epsilon"
"JS1_A09_00324" 10810 "s59E25H2" 0 "F0F1 ATP synthase subunit epsilon"
"JS1_A09_00324" 10810 "s59E5H2" 1.528729 "F0F1 ATP synthase subunit epsilon"
"JS1_A09_00324" 10810 "s63E6H2" 0.5299501 "F0F1 ATP synthase subunit epsilon"
"JS1_A09_00325" 10811 "s59E13H2" 0.1846023 "Putative F0F1-ATPase subunit (ATPase_gene1)"
"JS1_A09_00325" 10811 "s59E25H2" 0 "Putative F0F1-ATPase subunit (ATPase_gene1)"
"JS1_A09_00325" 10811 "s59E5H2" 1.651845 "Putative F0F1-ATPase subunit (ATPase_gene1)"
"JS1_A09_00325" 10811 "s63E6H2" 0.6725308 "Putative F0F1-ATPase subunit (ATPase_gene1)"
"JS1_A09_00326" 10812 "s59E13H2" 0.2532029 "N-ATPase, AtpR subunit"
"JS1_A09_00326" 10812 "s59E25H2" 0 "N-ATPase, AtpR subunit"
"JS1_A09_00326" 10812 "s59E5H2" 2.089512 "N-ATPase, AtpR subunit"
"JS1_A09_00326" 10812 "s63E6H2" 0.8706471 "N-ATPase, AtpR subunit"
"JS1_A09_00327" 10813 "s59E13H2" 0.1932165 "ATP synthase subunit a"
"JS1_A09_00327" 10813 "s59E25H2" 0 "ATP synthase subunit a"
"JS1_A09_00327" 10813 "s59E5H2" 2.393144 "ATP synthase subunit a"
"JS1_A09_00327" 10813 "s63E6H2" 0.9023677 "ATP synthase subunit a"
"JS1_A09_00328" 10814 "s59E13H2" 0.2633901 "ATP synthase subunit a"
"JS1_A09_00328" 10814 "s59E25H2" 0 "ATP synthase subunit a"
"JS1_A09_00328" 10814 "s59E5H2" 2.692426 "ATP synthase subunit a"
"JS1_A09_00328" 10814 "s63E6H2" 0.94497 "ATP synthase subunit a"
"JS1_A09_00329" 10815 "s59E13H2" 0.2810832 "ATP synthase subunit c, sodium ion specific"
"JS1_A09_00329" 10815 "s59E25H2" 0 "ATP synthase subunit c, sodium ion specific"
"JS1_A09_00329" 10815 "s59E5H2" 2.326384 "ATP synthase subunit c, sodium ion specific"
"JS1_A09_00329" 10815 "s63E6H2" 1.044444 "ATP synthase subunit c, sodium ion specific"
"JS1_A09_00330" 10816 "s59E13H2" 0.4094176 "ATP synthase subunit b, sodium ion specific"
"JS1_A09_00330" 10816 "s59E25H2" 0 "ATP synthase subunit b, sodium ion specific"
"JS1_A09_00330" 10816 "s59E5H2" 0.5614969 "ATP synthase subunit b, sodium ion specific"
"JS1_A09_00330" 10816 "s63E6H2" 1.04576 "ATP synthase subunit b, sodium ion specific"
"JS1_A09_00331" 10817 "s59E13H2" 0.4978442 "ATP synthase subunit alpha"
"JS1_A09_00331" 10817 "s59E25H2" 0 "ATP synthase subunit alpha"
"JS1_A09_00331" 10817 "s59E5H2" 1.153168 "ATP synthase subunit alpha"
"JS1_A09_00331" 10817 "s63E6H2" 2.070724 "ATP synthase subunit alpha"
"JS1_A09_00332" 10818 "s59E13H2" 0.3468632 "ATP synthase gamma chain"
"JS1_A09_00332" 10818 "s59E25H2" 0 "ATP synthase gamma chain"
"JS1_A09_00332" 10818 "s59E5H2" 1.500717 "ATP synthase gamma chain"
"JS1_A09_00332" 10818 "s63E6H2" 1.661207 "ATP synthase gamma chain"

"JS1_A09_00333" 10819 "s59E13H2" 0 "hypothetical protein"
"JS1_A09_00333" 10819 "s59E25H2" 0 "hypothetical protein"
"JS1_A09_00333" 10819 "s59E5H2" 0.1342888 "hypothetical protein"
"JS1_A09_00333" 10819 "s63E6H2" 0.004890127 "hypothetical protein"
"JS1_A09_00334" 10820 "s59E13H2" 0.3311062 "Demethylmenaquinone methyltransferase"
"JS1_A09_00334" 10820 "s59E25H2" 0 "Demethylmenaquinone methyltransferase"
"JS1_A09_00334" 10820 "s59E5H2" 2.521816 "Demethylmenaquinone methyltransferase"
"JS1_A09_00334" 10820 "s63E6H2" 2.27443 "Demethylmenaquinone methyltransferase"
"JS1_A09_00335" 10821 "s59E13H2" 0.47234 "Glutathione amide reductase"
"JS1_A09_00335" 10821 "s59E25H2" 0 "Glutathione amide reductase"
"JS1_A09_00335" 10821 "s59E5H2" 1.998436 "Glutathione amide reductase"
"JS1_A09_00335" 10821 "s63E6H2" 2.933052 "Glutathione amide reductase"
"JS1_A09_00336" 10822 "s59E13H2" 0 "Mercuric reductase"
"JS1_A09_00336" 10822 "s59E25H2" 0 "Mercuric reductase"
"JS1_A09_00336" 10822 "s59E5H2" 1.404135 "Mercuric reductase"
"JS1_A09_00336" 10822 "s63E6H2" 0.1391835 "Mercuric reductase"
"JS1_A09_00337" 10823 "s59E13H2" 0 "Antilisterial bacteriocin subtilosin biosynthesis protein AlbA"
"JS1_A09_00337" 10823 "s59E25H2" 0 "Antilisterial bacteriocin subtilosin biosynthesis protein AlbA"
"JS1_A09_00337" 10823 "s59E5H2" 1.047543 "Antilisterial bacteriocin subtilosin biosynthesis protein AlbA"
"JS1_A09_00337" 10823 "s63E6H2" 0.002965651 "Antilisterial bacteriocin subtilosin biosynthesis protein AlbA"
"JS1_A09_00338" 10824 "s59E13H2" 0 "6-phosphogluconolactonase"
"JS1_A09_00338" 10824 "s59E25H2" 0 "6-phosphogluconolactonase"
"JS1_A09_00338" 10824 "s59E5H2" 1.679389 "6-phosphogluconolactonase"
"JS1_A09_00338" 10824 "s63E6H2" 0.2479833 "6-phosphogluconolactonase"
"JS1_A09_00339" 10825 "s59E13H2" 0.001106371 "Glucose-6-phosphate 1-dehydrogenase"
"JS1_A09_00339" 10825 "s59E25H2" 0 "Glucose-6-phosphate 1-dehydrogenase"
"JS1_A09_00339" 10825 "s59E5H2" 2.817092 "Glucose-6-phosphate 1-dehydrogenase"
"JS1_A09_00339" 10825 "s63E6H2" 0.2510346 "Glucose-6-phosphate 1-dehydrogenase"
"JS1_A09_00340" 10826 "s59E13H2" 0.1153801 "Ribose-5-phosphate isomerase B"
"JS1_A09_00340" 10826 "s59E25H2" 0 "Ribose-5-phosphate isomerase B"
"JS1_A09_00340" 10826 "s59E5H2" 1.973254 "Ribose-5-phosphate isomerase B"
"JS1_A09_00340" 10826 "s63E6H2" 0.6030242 "Ribose-5-phosphate isomerase B"
"JS1_A09_00341" 10827 "s59E13H2" 0 "Transaldolase"
"JS1_A09_00341" 10827 "s59E25H2" 0 "Transaldolase"
"JS1_A09_00341" 10827 "s59E5H2" 0 "Transaldolase"
"JS1_A09_00341" 10827 "s63E6H2" 0 "Transaldolase"
"JS1_A09_00342" 10828 "s59E13H2" 0 "hypothetical protein"
"JS1_A09_00342" 10828 "s59E25H2" 0 "hypothetical protein"
"JS1_A09_00342" 10828 "s59E5H2" 0 "hypothetical protein"
"JS1_A09_00342" 10828 "s63E6H2" 0 "hypothetical protein"
"JS1_A09_00343" 10829 "s59E13H2" 0.9786881 "Ribosome maturation factor RimM"
"JS1_A09_00343" 10829 "s59E25H2" 0 "Ribosome maturation factor RimM"
"JS1_A09_00343" 10829 "s59E5H2" 0.03095535 "Ribosome maturation factor RimM"
"JS1_A09_00343" 10829 "s63E6H2" 0.007379484 "Ribosome maturation factor RimM"
"JS1_A09_00344" 10830 "s59E13H2" 0.01608367 "tRNA (guanine-N(1)-)-methyltransferase"
"JS1_A09_00344" 10830 "s59E25H2" 0 "tRNA (guanine-N(1)-)-methyltransferase"
"JS1_A09_00344" 10830 "s59E5H2" 0.0732081 "tRNA (guanine-N(1)-)-methyltransferase"

"JS1_A09_00344" 10830 "s63E6H2" 0.01362654 "tRNA (guanine-N(1)-)-methyltransferase"
"JS1_A09_00345" 10831 "s59E13H2" 0.01749987 "50S ribosomal protein L19"
"JS1_A09_00345" 10831 "s59E25H2" 0 "50S ribosomal protein L19"
"JS1_A09_00345" 10831 "s59E5H2" 0.06968695 "50S ribosomal protein L19"
"JS1_A09_00345" 10831 "s63E6H2" 0.04343608 "50S ribosomal protein L19"
"JS1_A09_00346" 10832 "s59E13H2" 0.0308642 "Ribonuclease HII"
"JS1_A09_00346" 10832 "s59E25H2" 0 "Ribonuclease HII"
"JS1_A09_00346" 10832 "s59E5H2" 0.1192093 "Ribonuclease HII"
"JS1_A09_00346" 10832 "s63E6H2" 0.04112613 "Ribonuclease HII"
"JS1_A09_00347" 10833 "s59E13H2" 0.03381039 "hypothetical protein"
"JS1_A09_00347" 10833 "s59E25H2" 0 "hypothetical protein"
"JS1_A09_00347" 10833 "s59E5H2" 0.09565348 "hypothetical protein"
"JS1_A09_00347" 10833 "s63E6H2" 0.02283324 "hypothetical protein"
"JS1_A09_00348" 10834 "s59E13H2" 0.2348328 "Competence protein ComM"
"JS1_A09_00348" 10834 "s59E25H2" 0 "Competence protein ComM"
"JS1_A09_00348" 10834 "s59E5H2" 0.2053381 "Competence protein ComM"
"JS1_A09_00348" 10834 "s63E6H2" 0.04882825 "Competence protein ComM"
"JS1_A09_00349" 10835 "s59E13H2" 0.0167273 "Ribulose-phosphate 3-epimerase"
"JS1_A09_00349" 10835 "s59E25H2" 0 "Ribulose-phosphate 3-epimerase"
"JS1_A09_00349" 10835 "s59E5H2" 0.1448939 "Ribulose-phosphate 3-epimerase"
"JS1_A09_00349" 10835 "s63E6H2" 0.0345881 "Ribulose-phosphate 3-epimerase"
"JS1_A09_00350" 10836 "s59E13H2" 0.03317807 "GTPase Der"
"JS1_A09_00350" 10836 "s59E25H2" 0 "GTPase Der"
"JS1_A09_00350" 10836 "s59E5H2" 0.1716536 "GTPase Der"
"JS1_A09_00350" 10836 "s63E6H2" 0.08520633 "GTPase Der"
"JS1_A09_00351" 10837 "s59E13H2" 0.007970671 "Glycerol-3-phosphate acyltransferase"
"JS1_A09_00351" 10837 "s59E25H2" 0 "Glycerol-3-phosphate acyltransferase"
"JS1_A09_00351" 10837 "s59E5H2" 0.09135567 "Glycerol-3-phosphate acyltransferase"
"JS1_A09_00351" 10837 "s63E6H2" 0.05160843 "Glycerol-3-phosphate acyltransferase"
"JS1_A09_00352" 10838 "s59E13H2" 0.03349617 "ferredoxin"
"JS1_A09_00352" 10838 "s59E25H2" 0 "ferredoxin"
"JS1_A09_00352" 10838 "s59E5H2" 0.2468811 "ferredoxin"
"JS1_A09_00352" 10838 "s63E6H2" 0.05348347 "ferredoxin"
"JS1_A09_00355" 10841 "s59E13H2" 0.004705991 "Formate dehydrogenase subunit beta"
"JS1_A09_00355" 10841 "s59E25H2" 0 "Formate dehydrogenase subunit beta"
"JS1_A09_00355" 10841 "s59E5H2" 0.04165393 "Formate dehydrogenase subunit beta"
"JS1_A09_00355" 10841 "s63E6H2" 0.002738419 "Formate dehydrogenase subunit beta"
"JS1_A09_00359" 10845 "s59E13H2" 2.315676 "Benzylsuccinate synthase alpha subunit"
"JS1_A09_00359" 10845 "s59E25H2" 0 "Benzylsuccinate synthase alpha subunit"
"JS1_A09_00359" 10845 "s59E5H2" 6.556908 "Benzylsuccinate synthase alpha subunit"
"JS1_A09_00359" 10845 "s63E6H2" 1.514513 "Benzylsuccinate synthase alpha subunit"
"JS1_A09_00360" 10846 "s59E13H2" 0.1545811 "Pyruvate formate-lyase-activating enzyme"
"JS1_A09_00360" 10846 "s59E25H2" 0 "Pyruvate formate-lyase-activating enzyme"
"JS1_A09_00360" 10846 "s59E5H2" 0.2522113 "Pyruvate formate-lyase-activating enzyme"
"JS1_A09_00360" 10846 "s63E6H2" 0.08699058 "Pyruvate formate-lyase-activating enzyme"
"JS1_A09_00361" 10847 "s59E13H2" 1.030916 "Benzylsuccinate synthase activating enzyme"
"JS1_A09_00361" 10847 "s59E25H2" 0 "Benzylsuccinate synthase activating enzyme"

"JS1_A09_00361" 10847 "s59E5H2" 0.6726027 "Benzylsuccinate synthase activating enzyme"
"JS1_A09_00361" 10847 "s63E6H2" 0.4553941 "Benzylsuccinate synthase activating enzyme"
"JS1_A09_00362" 10848 "s59E13H2" 1.132486 "Transketolase 2"
"JS1_A09_00362" 10848 "s59E25H2" 0.2537994 "Transketolase 2"
"JS1_A09_00362" 10848 "s59E5H2" 1.681339 "Transketolase 2"
"JS1_A09_00362" 10848 "s63E6H2" 0.826999 "Transketolase 2"
"JS1_A09_00363" 10849 "s59E13H2" 1.309183 "Thiamine pyrophosphokinase"
"JS1_A09_00363" 10849 "s59E25H2" 0 "Thiamine pyrophosphokinase"
"JS1_A09_00363" 10849 "s59E5H2" 1.272671 "Thiamine pyrophosphokinase"
"JS1_A09_00363" 10849 "s63E6H2" 0.6965992 "Thiamine pyrophosphokinase"
"JS1_A09_00364" 10850 "s59E13H2" 1.34154 "Thiamine transporter ThiT"
"JS1_A09_00364" 10850 "s59E25H2" 0 "Thiamine transporter ThiT"
"JS1_A09_00364" 10850 "s59E5H2" 1.553172 "Thiamine transporter ThiT"
"JS1_A09_00364" 10850 "s63E6H2" 1.068334 "Thiamine transporter ThiT"
"JS1_A09_00366" 10852 "s59E13H2" 1.048281 "RNA recognition motif. (a.k.a. RRM, RBD, or RNP domain)"
"JS1_A09_00366" 10852 "s59E25H2" 0 "RNA recognition motif. (a.k.a. RRM, RBD, or RNP domain)"
"JS1_A09_00366" 10852 "s59E5H2" 2.292473 "RNA recognition motif. (a.k.a. RRM, RBD, or RNP domain)"
"JS1_A09_00366" 10852 "s63E6H2" 1.118684 "RNA recognition motif. (a.k.a. RRM, RBD, or RNP domain)"
"JS1_A09_00367" 10853 "s59E13H2" 0.2493375 "NAD-dependent protein deacetylase"
"JS1_A09_00367" 10853 "s59E25H2" 0 "NAD-dependent protein deacetylase"
"JS1_A09_00367" 10853 "s59E5H2" 0.5890475 "NAD-dependent protein deacetylase"
"JS1_A09_00367" 10853 "s63E6H2" 0.158888 "NAD-dependent protein deacetylase"
"JS1_A09_00368" 10854 "s59E13H2" 0.5307749 "Methionine--tRNA ligase"
"JS1_A09_00368" 10854 "s59E25H2" 0 "Methionine--tRNA ligase"
"JS1_A09_00368" 10854 "s59E5H2" 1.189155 "Methionine--tRNA ligase"
"JS1_A09_00368" 10854 "s63E6H2" 0.1610643 "Methionine--tRNA ligase"
"JS1_A09_00369" 10855 "s59E13H2" 0 "Serine acetyltransferase"
"JS1_A09_00369" 10855 "s59E25H2" 0 "Serine acetyltransferase"
"JS1_A09_00369" 10855 "s59E5H2" 0 "Serine acetyltransferase"
"JS1_A09_00369" 10855 "s63E6H2" 0.1637258 "Serine acetyltransferase"
"JS1_A09_00370" 10856 "s59E13H2" 0 "Ferredoxin"
"JS1_A09_00370" 10856 "s59E25H2" 0 "Ferredoxin"
"JS1_A09_00370" 10856 "s59E5H2" 0 "Ferredoxin"
"JS1_A09_00370" 10856 "s63E6H2" 0.5481493 "Ferredoxin"
"JS1_A09_00371" 10857 "s59E13H2" 0.001441872 "hypothetical protein"
"JS1_A09_00371" 10857 "s59E25H2" 0 "hypothetical protein"
"JS1_A09_00371" 10857 "s59E5H2" 0.003374084 "hypothetical protein"
"JS1_A09_00371" 10857 "s63E6H2" 0.8204067 "hypothetical protein"
"JS1_A09_00372" 10858 "s59E13H2" 0 "O-acetylserine sulfhydrylase"
"JS1_A09_00372" 10858 "s59E25H2" 0 "O-acetylserine sulfhydrylase"
"JS1_A09_00372" 10858 "s59E5H2" 0 "O-acetylserine sulfhydrylase"
"JS1_A09_00372" 10858 "s63E6H2" 0.06654322 "O-acetylserine sulfhydrylase"
"JS1_A09_00373" 10859 "s59E13H2" 0 "HTH-type transcriptional regulator CymR"
"JS1_A09_00373" 10859 "s59E25H2" 0 "HTH-type transcriptional regulator CymR"
"JS1_A09_00373" 10859 "s59E5H2" 0 "HTH-type transcriptional regulator CymR"
"JS1_A09_00373" 10859 "s63E6H2" 0.08258042 "HTH-type transcriptional regulator CymR"
"JS1_A09_00374" 10860 "s59E13H2" 0 "putative adenylyltransferase"

"JS1_A09_00374" 10860 "s59E25H2" 0 "putative adenylyltransferase"
"JS1_A09_00374" 10860 "s59E5H2" 0 "putative adenylyltransferase"
"JS1_A09_00374" 10860 "s63E6H2" 0.09258812 "putative adenylyltransferase"
"JS1_A09_00375" 10861 "s59E13H2" 0 "formate dehydrogenase accessory protein"
"JS1_A09_00375" 10861 "s59E25H2" 0 "formate dehydrogenase accessory protein"
"JS1_A09_00375" 10861 "s59E5H2" 0 "formate dehydrogenase accessory protein"
"JS1_A09_00375" 10861 "s63E6H2" 0.04678469 "formate dehydrogenase accessory protein"
"JS1_A09_00376" 10862 "s59E13H2" 0.0001979234 "hypothetical protein"
"JS1_A09_00376" 10862 "s59E25H2" 0 "hypothetical protein"
"JS1_A09_00376" 10862 "s59E5H2" 0.002499182 "hypothetical protein"
"JS1_A09_00376" 10862 "s63E6H2" 0.0004600338 "hypothetical protein"
"JS1_A09_00378" 10864 "s59E13H2" 0 "hypothetical protein"
"JS1_A09_00378" 10864 "s59E25H2" 0 "hypothetical protein"
"JS1_A09_00378" 10864 "s59E5H2" 0 "hypothetical protein"
"JS1_A09_00378" 10864 "s63E6H2" 0.007822019 "hypothetical protein"
"JS1_A09_00379" 10865 "s59E13H2" 0 "hypothetical protein"
"JS1_A09_00379" 10865 "s59E25H2" 0 "hypothetical protein"
"JS1_A09_00379" 10865 "s59E5H2" 0 "hypothetical protein"
"JS1_A09_00379" 10865 "s63E6H2" 0.1141856 "hypothetical protein"
"JS1_A09_00380" 10866 "s59E13H2" 0 "hypothetical protein"
"JS1_A09_00380" 10866 "s59E25H2" 0 "hypothetical protein"
"JS1_A09_00380" 10866 "s59E5H2" 0.0125691 "hypothetical protein"
"JS1_A09_00380" 10866 "s63E6H2" 0.2959788 "hypothetical protein"
"JS1_A09_00381" 10867 "s59E13H2" 0.003086356 "hypothetical protein"
"JS1_A09_00381" 10867 "s59E25H2" 0 "hypothetical protein"
"JS1_A09_00381" 10867 "s59E5H2" 0.002947588 "hypothetical protein"
"JS1_A09_00381" 10867 "s63E6H2" 0.09478165 "hypothetical protein"
"JS1_A09_00382" 10868 "s59E13H2" 0 "hypothetical protein"
"JS1_A09_00382" 10868 "s59E25H2" 0 "hypothetical protein"
"JS1_A09_00382" 10868 "s59E5H2" 0 "hypothetical protein"
"JS1_A09_00382" 10868 "s63E6H2" 0.04833362 "hypothetical protein"
"JS1_A09_00383" 10869 "s59E13H2" 0 "hypothetical protein"
"JS1_A09_00383" 10869 "s59E25H2" 0 "hypothetical protein"
"JS1_A09_00383" 10869 "s59E5H2" 0 "hypothetical protein"
"JS1_A09_00383" 10869 "s63E6H2" 0 "hypothetical protein"
"JS1_A09_00385" 10871 "s59E13H2" 0 "hypothetical protein"
"JS1_A09_00385" 10871 "s59E25H2" 0 "hypothetical protein"
"JS1_A09_00385" 10871 "s59E5H2" 0 "hypothetical protein"
"JS1_A09_00385" 10871 "s63E6H2" 0 "hypothetical protein"
"JS1_A09_00386" 10872 "s59E13H2" 0 "Peptidase M15"
"JS1_A09_00386" 10872 "s59E25H2" 0 "Peptidase M15"
"JS1_A09_00386" 10872 "s59E5H2" 0 "Peptidase M15"
"JS1_A09_00386" 10872 "s63E6H2" 0.03226799 "Peptidase M15"
"JS1_A09_00387" 10873 "s59E13H2" 0.0007728436 "hypothetical protein"
"JS1_A09_00387" 10873 "s59E25H2" 0 "hypothetical protein"
"JS1_A09_00387" 10873 "s59E5H2" 0.0008313709 "hypothetical protein"
"JS1_A09_00387" 10873 "s63E6H2" 0.002883766 "hypothetical protein"

"JS1_A09_00388" 10874 "s59E13H2" 0.08620946 "hypothetical protein"
"JS1_A09_00388" 10874 "s59E25H2" 0 "hypothetical protein"
"JS1_A09_00388" 10874 "s59E5H2" 0.03788548 "hypothetical protein"
"JS1_A09_00388" 10874 "s63E6H2" 0.008258246 "hypothetical protein"
"JS1_A09_00389" 10875 "s59E13H2" 0 "Modification methylase PvuII"
"JS1_A09_00389" 10875 "s59E25H2" 0 "Modification methylase PvuII"
"JS1_A09_00389" 10875 "s59E5H2" 0 "Modification methylase PvuII"
"JS1_A09_00389" 10875 "s63E6H2" 0 "Modification methylase PvuII"
"JS1_A09_00392" 10878 "s59E13H2" 0 "hypothetical protein"
"JS1_A09_00392" 10878 "s59E25H2" 0 "hypothetical protein"
"JS1_A09_00392" 10878 "s59E5H2" 0 "hypothetical protein"
"JS1_A09_00392" 10878 "s63E6H2" 0 "hypothetical protein"
"JS1_A09_00395" 10881 "s59E13H2" 0.002565841 "hypothetical protein"
"JS1_A09_00395" 10881 "s59E25H2" 0 "hypothetical protein"
"JS1_A09_00395" 10881 "s59E5H2" 0.007997788 "hypothetical protein"
"JS1_A09_00395" 10881 "s63E6H2" 0.01188839 "hypothetical protein"
"JS1_A09_00398" 10884 "s59E13H2" 0 "hypothetical protein"
"JS1_A09_00398" 10884 "s59E25H2" 0 "hypothetical protein"
"JS1_A09_00398" 10884 "s59E5H2" 0 "hypothetical protein"
"JS1_A09_00398" 10884 "s63E6H2" 0 "hypothetical protein"
"JS1_A09_00404" 10890 "s59E13H2" 0 "hypothetical protein"
"JS1_A09_00404" 10890 "s59E25H2" 0 "hypothetical protein"
"JS1_A09_00404" 10890 "s59E5H2" 0 "hypothetical protein"
"JS1_A09_00404" 10890 "s63E6H2" 0 "hypothetical protein"
"JS1_A09_00406" 10892 "s59E13H2" 0 "hypothetical protein"
"JS1_A09_00406" 10892 "s59E25H2" 0 "hypothetical protein"
"JS1_A09_00406" 10892 "s59E5H2" 0 "hypothetical protein"
"JS1_A09_00406" 10892 "s63E6H2" 0 "hypothetical protein"
"JS1_A09_00407" 10893 "s59E13H2" 0 "hypothetical protein"
"JS1_A09_00407" 10893 "s59E25H2" 0 "hypothetical protein"
"JS1_A09_00407" 10893 "s59E5H2" 0 "hypothetical protein"
"JS1_A09_00407" 10893 "s63E6H2" 0 "hypothetical protein"
"JS1_A09_00408" 10894 "s59E13H2" 0 "hypothetical protein"
"JS1_A09_00408" 10894 "s59E25H2" 0 "hypothetical protein"
"JS1_A09_00408" 10894 "s59E5H2" 0 "hypothetical protein"
"JS1_A09_00408" 10894 "s63E6H2" 0 "hypothetical protein"
"JS1_A09_00411" 10897 "s59E13H2" 0.04976495 "Lipoate-protein ligase A subunit 2"
"JS1_A09_00411" 10897 "s59E25H2" 0 "Lipoate-protein ligase A subunit 2"
"JS1_A09_00411" 10897 "s59E5H2" 0 "Lipoate-protein ligase A subunit 2"
"JS1_A09_00411" 10897 "s63E6H2" 0.09971656 "Lipoate-protein ligase A subunit 2"
"JS1_A09_00418" 10904 "s59E13H2" 0.01811786 "Sulfhydrogenase 1 subunit gamma"
"JS1_A09_00418" 10904 "s59E25H2" 0 "Sulfhydrogenase 1 subunit gamma"
"JS1_A09_00418" 10904 "s59E5H2" 0 "Sulfhydrogenase 1 subunit gamma"
"JS1_A09_00418" 10904 "s63E6H2" 0.003033578 "Sulfhydrogenase 1 subunit gamma"
"JS1_A09_00419" 10905 "s59E13H2" 0 "2-oxoglutarate synthase subunit KorC"
"JS1_A09_00419" 10905 "s59E25H2" 0 "2-oxoglutarate synthase subunit KorC"
"JS1_A09_00419" 10905 "s59E5H2" 0 "2-oxoglutarate synthase subunit KorC"

"JS1_A09_00419" 10905 "s63E6H2" 0.006914747 "2-oxoglutarate synthase subunit KorC"
"JS1_A09_00422" 10908 "s59E13H2" 0 "putative transcriptional regulatory protein pdtaR"
"JS1_A09_00422" 10908 "s59E25H2" 0 "putative transcriptional regulatory protein pdtaR"
"JS1_A09_00422" 10908 "s59E5H2" 0 "putative transcriptional regulatory protein pdtaR"
"JS1_A09_00422" 10908 "s63E6H2" 0 "putative transcriptional regulatory protein pdtaR"
"JS1_A09_00423" 10909 "s59E13H2" 0.0119407 "Glutamate dehydrogenase"
"JS1_A09_00423" 10909 "s59E25H2" 0 "Glutamate dehydrogenase"
"JS1_A09_00423" 10909 "s59E5H2" 0 "Glutamate dehydrogenase"
"JS1_A09_00423" 10909 "s63E6H2" 0.07789353 "Glutamate dehydrogenase"
"JS1_A09_00424" 10910 "s59E13H2" 0.007520489 "(R)-citramalate synthase CimA"
"JS1_A09_00424" 10910 "s59E25H2" 0 "(R)-citramalate synthase CimA"
"JS1_A09_00424" 10910 "s59E5H2" 0 "(R)-citramalate synthase CimA"
"JS1_A09_00424" 10910 "s63E6H2" 0.1773727 "(R)-citramalate synthase CimA"
"JS1_A09_00425" 10911 "s59E13H2" 0 "Methanogen homoaconitase large subunit"
"JS1_A09_00425" 10911 "s59E25H2" 0 "Methanogen homoaconitase large subunit"
"JS1_A09_00425" 10911 "s59E5H2" 0 "Methanogen homoaconitase large subunit"
"JS1_A09_00425" 10911 "s63E6H2" 0.1056822 "Methanogen homoaconitase large subunit"
"JS1_A09_00426" 10912 "s59E13H2" 0 "Isocitrate dehydrogenase [NADP]"
"JS1_A09_00426" 10912 "s59E25H2" 0 "Isocitrate dehydrogenase [NADP]"
"JS1_A09_00426" 10912 "s59E5H2" 0 "Isocitrate dehydrogenase [NADP]"
"JS1_A09_00426" 10912 "s63E6H2" 0.09212264 "Isocitrate dehydrogenase [NADP]"
"JS1_A09_00427" 10913 "s59E13H2" 0.02685143 "Dihydroxy-acid dehydratase"
"JS1_A09_00427" 10913 "s59E25H2" 0 "Dihydroxy-acid dehydratase"
"JS1_A09_00427" 10913 "s59E5H2" 0.465585 "Dihydroxy-acid dehydratase"
"JS1_A09_00427" 10913 "s63E6H2" 0.08652929 "Dihydroxy-acid dehydratase"
"JS1_A09_00430" 10916 "s59E13H2" 0.03363327 "Acetolactate synthase large subunit"
"JS1_A09_00430" 10916 "s59E25H2" 0 "Acetolactate synthase large subunit"
"JS1_A09_00430" 10916 "s59E5H2" 0.7171193 "Acetolactate synthase large subunit"
"JS1_A09_00430" 10916 "s63E6H2" 0.5103693 "Acetolactate synthase large subunit"
"JS1_A09_00431" 10917 "s59E13H2" 0.006684419 "Putative acetolactate synthase small subunit"
"JS1_A09_00431" 10917 "s59E25H2" 0 "Putative acetolactate synthase small subunit"
"JS1_A09_00431" 10917 "s59E5H2" 0.7736441 "Putative acetolactate synthase small subunit"
"JS1_A09_00431" 10917 "s63E6H2" 0.4879193 "Putative acetolactate synthase small subunit"
"JS1_A09_00432" 10918 "s59E13H2" 0.001742369 "Ketol-acid reductoisomerase"
"JS1_A09_00432" 10918 "s59E25H2" 0 "Ketol-acid reductoisomerase"
"JS1_A09_00432" 10918 "s59E5H2" 0.9689827 "Ketol-acid reductoisomerase"
"JS1_A09_00432" 10918 "s63E6H2" 0.2861331 "Ketol-acid reductoisomerase"
"JS1_A09_00433" 10919 "s59E13H2" 0.02035907 "P-protein"
"JS1_A09_00433" 10919 "s59E25H2" 0 "P-protein"
"JS1_A09_00433" 10919 "s59E5H2" 2.152058 "P-protein"
"JS1_A09_00433" 10919 "s63E6H2" 0.5986473 "P-protein"
"JS1_A09_00434" 10920 "s59E13H2" 0.09637103 "2-isopropylmalate synthase"
"JS1_A09_00434" 10920 "s59E25H2" 0 "2-isopropylmalate synthase"
"JS1_A09_00434" 10920 "s59E5H2" 1.925653 "2-isopropylmalate synthase"
"JS1_A09_00434" 10920 "s63E6H2" 0.8303723 "2-isopropylmalate synthase"
"JS1_A09_00435" 10921 "s59E13H2" 0.1255975 "(R)-citramalate synthase CimA"
"JS1_A09_00435" 10921 "s59E25H2" 0 "(R)-citramalate synthase CimA"

"JS1_A09_00435" 10921 "s59E5H2" 1.881538 "(R)-citramalate synthase CimA"
"JS1_A09_00435" 10921 "s63E6H2" 0.6596497 "(R)-citramalate synthase CimA"
"JS1_A09_00436" 10922 "s59E13H2" 0.1571209 "hypothetical protein"
"JS1_A09_00436" 10922 "s59E25H2" 0 "hypothetical protein"
"JS1_A09_00436" 10922 "s59E5H2" 1.218589 "hypothetical protein"
"JS1_A09_00436" 10922 "s63E6H2" 0.5249501 "hypothetical protein"
"JS1_A09_00437" 10923 "s59E13H2" 0.1533129 "Ferredoxin"
"JS1_A09_00437" 10923 "s59E25H2" 0 "Ferredoxin"
"JS1_A09_00437" 10923 "s59E5H2" 2.569321 "Ferredoxin"
"JS1_A09_00437" 10923 "s63E6H2" 0.8310299 "Ferredoxin"
"JS1_A09_00438" 10924 "s59E13H2" 0.2433349 "hypothetical protein"
"JS1_A09_00438" 10924 "s59E25H2" 0 "hypothetical protein"
"JS1_A09_00438" 10924 "s59E5H2" 1.955182 "hypothetical protein"
"JS1_A09_00438" 10924 "s63E6H2" 1.041117 "hypothetical protein"
"JS1_A09_00439" 10925 "s59E13H2" 0.02588462 "Pyrroline-5-carboxylate reductase"
"JS1_A09_00439" 10925 "s59E25H2" 0 "Pyrroline-5-carboxylate reductase"
"JS1_A09_00439" 10925 "s59E5H2" 1.535973 "Pyrroline-5-carboxylate reductase"
"JS1_A09_00439" 10925 "s63E6H2" 0.4269407 "Pyrroline-5-carboxylate reductase"
"JS1_A09_00440" 10926 "s59E13H2" 0.002396074 "Bifunctional homocysteine S-methyltransferase/5,10-methylenetetrahydrofolate reductase"
"JS1_A09_00440" 10926 "s59E25H2" 0 "Bifunctional homocysteine S-methyltransferase/5,10-methylenetetrahydrofolate reductase"
"JS1_A09_00440" 10926 "s59E5H2" 0.01050058 "Bifunctional homocysteine S-methyltransferase/5,10-methylenetetrahydrofolate reductase"
"JS1_A09_00440" 10926 "s63E6H2" 0.007834625 "Bifunctional homocysteine S-methyltransferase/5,10-methylenetetrahydrofolate reductase"
"JS1_A09_00441" 10927 "s59E13H2" 0.08649128 "Vitamin B12 dependent methionine synthase, activation domain"
"JS1_A09_00441" 10927 "s59E25H2" 0 "Vitamin B12 dependent methionine synthase, activation domain"
"JS1_A09_00441" 10927 "s59E5H2" 0.05622417 "Vitamin B12 dependent methionine synthase, activation domain"
"JS1_A09_00441" 10927 "s63E6H2" 0.2254712 "Vitamin B12 dependent methionine synthase, activation domain"
"JS1_A09_00442" 10928 "s59E13H2" 0.2749965 "2,3-dimethylmalate dehydratase large subunit"
"JS1_A09_00442" 10928 "s59E25H2" 0.6642914 "2,3-dimethylmalate dehydratase large subunit"
"JS1_A09_00442" 10928 "s59E5H2" 0.7403486 "2,3-dimethylmalate dehydratase large subunit"
"JS1_A09_00442" 10928 "s63E6H2" 1.117121 "2,3-dimethylmalate dehydratase large subunit"
"JS1_A09_00443" 10929 "s59E13H2" 0.02499922 "2,3-dimethylmalate dehydratase small subunit"
"JS1_A09_00443" 10929 "s59E25H2" 0.1019608 "2,3-dimethylmalate dehydratase small subunit"
"JS1_A09_00443" 10929 "s59E5H2" 0.4609218 "2,3-dimethylmalate dehydratase small subunit"
"JS1_A09_00443" 10929 "s63E6H2" 0.7125404 "2,3-dimethylmalate dehydratase small subunit"
"JS1_A09_00444" 10930 "s59E13H2" 0.1591236 "Glyoxylate reductase"
"JS1_A09_00444" 10930 "s59E25H2" 0 "Glyoxylate reductase"
"JS1_A09_00444" 10930 "s59E5H2" 0.1936401 "Glyoxylate reductase"
"JS1_A09_00444" 10930 "s63E6H2" 0.02249912 "Glyoxylate reductase"
"JS1_A09_00445" 10931 "s59E13H2" 0 "Cyclopentanol dehydrogenase"
"JS1_A09_00445" 10931 "s59E25H2" 0 "Cyclopentanol dehydrogenase"
"JS1_A09_00445" 10931 "s59E5H2" 0.08851606 "Cyclopentanol dehydrogenase"
"JS1_A09_00445" 10931 "s63E6H2" 0.06760981 "Cyclopentanol dehydrogenase"
"JS1_A09_00446" 10932 "s59E13H2" 0 "hypothetical protein"
"JS1_A09_00446" 10932 "s59E25H2" 0.1763441 "hypothetical protein"
"JS1_A09_00446" 10932 "s59E5H2" 0.0859861 "hypothetical protein"
"JS1_A09_00446" 10932 "s63E6H2" 0.3458963 "hypothetical protein"
"JS1_A09_00447" 10933 "s59E13H2" 0 "Ribose import ATP-binding protein RbsA"

"JS1_A09_00447" 10933 "s59E25H2" 0 "Ribose import ATP-binding protein RbsA"
"JS1_A09_00447" 10933 "s59E5H2" 0.1249865 "Ribose import ATP-binding protein RbsA"
"JS1_A09_00447" 10933 "s63E6H2" 0.228381 "Ribose import ATP-binding protein RbsA"
"JS1_A09_00448" 10934 "s59E13H2" 0 "Ribose transport system permease protein RbsC"
"JS1_A09_00448" 10934 "s59E25H2" 0 "Ribose transport system permease protein RbsC"
"JS1_A09_00448" 10934 "s59E5H2" 0.02983026 "Ribose transport system permease protein RbsC"
"JS1_A09_00448" 10934 "s63E6H2" 0.1346603 "Ribose transport system permease protein RbsC"
"JS1_A09_00449" 10935 "s59E13H2" 0 "Ribose transport system permease protein RbsC"
"JS1_A09_00449" 10935 "s59E25H2" 0 "Ribose transport system permease protein RbsC"
"JS1_A09_00449" 10935 "s59E5H2" 0.1021661 "Ribose transport system permease protein RbsC"
"JS1_A09_00449" 10935 "s63E6H2" 0.09261229 "Ribose transport system permease protein RbsC"
"JS1_A09_00450" 10936 "s59E13H2" 0.03793473 "Deoxyribose operon repressor"
"JS1_A09_00450" 10936 "s59E25H2" 0 "Deoxyribose operon repressor"
"JS1_A09_00450" 10936 "s59E5H2" 0.6876477 "Deoxyribose operon repressor"
"JS1_A09_00450" 10936 "s63E6H2" 0.5121724 "Deoxyribose operon repressor"
"JS1_A09_00451" 10937 "s59E13H2" 0.02422809 "hypothetical protein"
"JS1_A09_00451" 10937 "s59E25H2" 0 "hypothetical protein"
"JS1_A09_00451" 10937 "s59E5H2" 0.5663247 "hypothetical protein"
"JS1_A09_00451" 10937 "s63E6H2" 0.5476984 "hypothetical protein"
"JS1_A09_00452" 10938 "s59E13H2" 0.04527525 "Sporulation protein YtfJ (Spore_YtfJ)"
"JS1_A09_00452" 10938 "s59E25H2" 0 "Sporulation protein YtfJ (Spore_YtfJ)"
"JS1_A09_00452" 10938 "s59E5H2" 0.7402081 "Sporulation protein YtfJ (Spore_YtfJ)"
"JS1_A09_00452" 10938 "s63E6H2" 0.6740193 "Sporulation protein YtfJ (Spore_YtfJ)"
"JS1_A09_00453" 10939 "s59E13H2" 4.448193 "General stress protein 69"
"JS1_A09_00453" 10939 "s59E25H2" 0 "General stress protein 69"
"JS1_A09_00453" 10939 "s59E5H2" 0.5082647 "General stress protein 69"
"JS1_A09_00453" 10939 "s63E6H2" 1.150949 "General stress protein 69"
"JS1_A09_00454" 10940 "s59E13H2" 0.0008927676 "Intracellular protease 1"
"JS1_A09_00454" 10940 "s59E25H2" 0 "Intracellular protease 1"
"JS1_A09_00454" 10940 "s59E5H2" 0.01142896 "Intracellular protease 1"
"JS1_A09_00454" 10940 "s63E6H2" 0.1006111 "Intracellular protease 1"
"JS1_A09_00455" 10941 "s59E13H2" 0.04352214 "hypothetical protein"
"JS1_A09_00455" 10941 "s59E25H2" 0 "hypothetical protein"
"JS1_A09_00455" 10941 "s59E5H2" 0.3257477 "hypothetical protein"
"JS1_A09_00455" 10941 "s63E6H2" 0.4127548 "hypothetical protein"
"JS1_A09_00456" 10942 "s59E13H2" 0.003518119 "DNA-binding transcriptional repressor AcrR"
"JS1_A09_00456" 10942 "s59E25H2" 0 "DNA-binding transcriptional repressor AcrR"
"JS1_A09_00456" 10942 "s59E5H2" 0.01506046 "DNA-binding transcriptional repressor AcrR"
"JS1_A09_00456" 10942 "s63E6H2" 0.1208633 "DNA-binding transcriptional repressor AcrR"
"JS1_A09_00457" 10943 "s59E13H2" 0 "hypothetical protein"
"JS1_A09_00457" 10943 "s59E25H2" 0 "hypothetical protein"
"JS1_A09_00457" 10943 "s59E5H2" 0.1350978 "hypothetical protein"
"JS1_A09_00457" 10943 "s63E6H2" 0.1679685 "hypothetical protein"
"JS1_A09_00459" 10945 "s59E13H2" 0.1278881 "ThiS family protein"
"JS1_A09_00459" 10945 "s59E25H2" 0 "ThiS family protein"
"JS1_A09_00459" 10945 "s59E5H2" 0.1909177 "ThiS family protein"
"JS1_A09_00459" 10945 "s63E6H2" 0.1074238 "ThiS family protein"

"JS1_A09_00460" 10946 "s59E13H2" 0.3447671 "Tungsten-containing aldehyde ferredoxin oxidoreductase"
"JS1_A09_00460" 10946 "s59E25H2" 0 "Tungsten-containing aldehyde ferredoxin oxidoreductase"
"JS1_A09_00460" 10946 "s59E5H2" 0.7713549 "Tungsten-containing aldehyde ferredoxin oxidoreductase"
"JS1_A09_00460" 10946 "s63E6H2" 0.08801638 "Tungsten-containing aldehyde ferredoxin oxidoreductase"
"JS1_A09_00461" 10947 "s59E13H2" 0.02969897 "Ribose transport system permease protein RbsC"
"JS1_A09_00461" 10947 "s59E25H2" 0 "Ribose transport system permease protein RbsC"
"JS1_A09_00461" 10947 "s59E5H2" 1.379415 "Ribose transport system permease protein RbsC"
"JS1_A09_00461" 10947 "s63E6H2" 0.1241328 "Ribose transport system permease protein RbsC"
"JS1_A09_00462" 10948 "s59E13H2" 0.09522403 "Ribose import ATP-binding protein RbsA"
"JS1_A09_00462" 10948 "s59E25H2" 0 "Ribose import ATP-binding protein RbsA"
"JS1_A09_00462" 10948 "s59E5H2" 0.9694222 "Ribose import ATP-binding protein RbsA"
"JS1_A09_00462" 10948 "s63E6H2" 0.6081218 "Ribose import ATP-binding protein RbsA"
"JS1_A09_00463" 10949 "s59E13H2" 0.03139831 "D-allose-binding periplasmic protein precursor"
"JS1_A09_00463" 10949 "s59E25H2" 0 "D-allose-binding periplasmic protein precursor"
"JS1_A09_00463" 10949 "s59E5H2" 0.6047662 "D-allose-binding periplasmic protein precursor"
"JS1_A09_00463" 10949 "s63E6H2" 0.4779855 "D-allose-binding periplasmic protein precursor"
"JS1_A09_00464" 10950 "s59E13H2" 0.1917809 "Glucose-6-phosphate isomerase"
"JS1_A09_00464" 10950 "s59E25H2" 0 "Glucose-6-phosphate isomerase"
"JS1_A09_00464" 10950 "s59E5H2" 0.5138917 "Glucose-6-phosphate isomerase"
"JS1_A09_00464" 10950 "s63E6H2" 0.08534846 "Glucose-6-phosphate isomerase"
"JS1_A09_00465" 10951 "s59E13H2" 0.378367 "Xylulose kinase"
"JS1_A09_00465" 10951 "s59E25H2" 0 "Xylulose kinase"
"JS1_A09_00465" 10951 "s59E5H2" 0.4852996 "Xylulose kinase"
"JS1_A09_00465" 10951 "s63E6H2" 0.06736882 "Xylulose kinase"
"JS1_A09_00466" 10952 "s59E13H2" 0.004942429 "preprotein translocase subunit SecF"
"JS1_A09_00466" 10952 "s59E25H2" 0 "preprotein translocase subunit SecF"
"JS1_A09_00466" 10952 "s59E5H2" 0.454113 "preprotein translocase subunit SecF"
"JS1_A09_00466" 10952 "s63E6H2" 0.1427002 "preprotein translocase subunit SecF"
"JS1_A09_00467" 10953 "s59E13H2" 0.01615112 "Single-stranded-DNA-specific exonuclease RecJ"
"JS1_A09_00467" 10953 "s59E25H2" 0 "Single-stranded-DNA-specific exonuclease RecJ"
"JS1_A09_00467" 10953 "s59E5H2" 0.438825 "Single-stranded-DNA-specific exonuclease RecJ"
"JS1_A09_00467" 10953 "s63E6H2" 0.3574141 "Single-stranded-DNA-specific exonuclease RecJ"
"JS1_A09_00468" 10954 "s59E13H2" 0.02053173 "Adenine phosphoribosyltransferase"
"JS1_A09_00468" 10954 "s59E25H2" 0 "Adenine phosphoribosyltransferase"
"JS1_A09_00468" 10954 "s59E5H2" 0.1078283 "Adenine phosphoribosyltransferase"
"JS1_A09_00468" 10954 "s63E6H2" 0.2233641 "Adenine phosphoribosyltransferase"
"JS1_A09_00469" 10955 "s59E13H2" 0.06197226 "GTP pyrophosphokinase"
"JS1_A09_00469" 10955 "s59E25H2" 0 "GTP pyrophosphokinase"
"JS1_A09_00469" 10955 "s59E5H2" 0.1320688 "GTP pyrophosphokinase"
"JS1_A09_00469" 10955 "s63E6H2" 0.3348982 "GTP pyrophosphokinase"
"JS1_A09_00470" 10956 "s59E13H2" 0.01775995 "D-tyrosyl-tRNA(Tyr) deacylase"
"JS1_A09_00470" 10956 "s59E25H2" 0 "D-tyrosyl-tRNA(Tyr) deacylase"
"JS1_A09_00470" 10956 "s59E5H2" 0.06808928 "D-tyrosyl-tRNA(Tyr) deacylase"
"JS1_A09_00470" 10956 "s63E6H2" 0.2636791 "D-tyrosyl-tRNA(Tyr) deacylase"
"JS1_A09_00471" 10957 "s59E13H2" 0.03150434 "putative glycerophosphoryl diester phosphodiesterase 1"
"JS1_A09_00471" 10957 "s59E25H2" 0 "putative glycerophosphoryl diester phosphodiesterase 1"
"JS1_A09_00471" 10957 "s59E5H2" 0.05950754 "putative glycerophosphoryl diester phosphodiesterase 1"

"JS1_A09_00471" 10957 "s63E6H2" 0.2822545 "putative glycerophosphoryl diester phosphodiesterase 1"
"JS1_A09_00472" 10958 "s59E13H2" 0.01723441 "Beta-lactamase hydrolase-like protein"
"JS1_A09_00472" 10958 "s59E25H2" 0 "Beta-lactamase hydrolase-like protein"
"JS1_A09_00472" 10958 "s59E5H2" 0.1808766 "Beta-lactamase hydrolase-like protein"
"JS1_A09_00472" 10958 "s63E6H2" 0.4622769 "Beta-lactamase hydrolase-like protein"
"JS1_A09_00473" 10959 "s59E13H2" 0.01046382 "Adenosylhomocysteinase"
"JS1_A09_00473" 10959 "s59E25H2" 0 "Adenosylhomocysteinase"
"JS1_A09_00473" 10959 "s59E5H2" 0.2126484 "Adenosylhomocysteinase"
"JS1_A09_00473" 10959 "s63E6H2" 0.4060356 "Adenosylhomocysteinase"
"JS1_A09_00474" 10960 "s59E13H2" 0.116313 "DNA utilization protein GntX"
"JS1_A09_00474" 10960 "s59E25H2" 0 "DNA utilization protein GntX"
"JS1_A09_00474" 10960 "s59E5H2" 0.1768923 "DNA utilization protein GntX"
"JS1_A09_00474" 10960 "s63E6H2" 0.2424961 "DNA utilization protein GntX"
"JS1_A09_00475" 10961 "s59E13H2" 0.003967264 "Putative sigma-54 modulation protein"
"JS1_A09_00475" 10961 "s59E25H2" 0 "Putative sigma-54 modulation protein"
"JS1_A09_00475" 10961 "s59E5H2" 0.1387004 "Putative sigma-54 modulation protein"
"JS1_A09_00475" 10961 "s63E6H2" 0.1923757 "Putative sigma-54 modulation protein"
"JS1_A09_00477" 10963 "s59E13H2" 0.01517992 "Cyclic di-GMP phosphodiesterase response regulator RpfG"
"JS1_A09_00477" 10963 "s59E25H2" 0 "Cyclic di-GMP phosphodiesterase response regulator RpfG"
"JS1_A09_00477" 10963 "s59E5H2" 0.1800955 "Cyclic di-GMP phosphodiesterase response regulator RpfG"
"JS1_A09_00477" 10963 "s63E6H2" 0.09038957 "Cyclic di-GMP phosphodiesterase response regulator RpfG"
"JS1_A09_00478" 10964 "s59E13H2" 0.01973047 "hypothetical protein"
"JS1_A09_00478" 10964 "s59E25H2" 0 "hypothetical protein"
"JS1_A09_00478" 10964 "s59E5H2" 0.256552 "hypothetical protein"
"JS1_A09_00478" 10964 "s63E6H2" 0.09312549 "hypothetical protein"
"JS1_A09_00479" 10965 "s59E13H2" 0.03521166 "preprotein translocase subunit SecA"
"JS1_A09_00479" 10965 "s59E25H2" 0 "preprotein translocase subunit SecA"
"JS1_A09_00479" 10965 "s59E5H2" 0.2299777 "preprotein translocase subunit SecA"
"JS1_A09_00479" 10965 "s63E6H2" 0.1163052 "preprotein translocase subunit SecA"
"JS1_A09_00480" 10966 "s59E13H2" 0.02849422 "Peptide chain release factor 2"
"JS1_A09_00480" 10966 "s59E25H2" 0 "Peptide chain release factor 2"
"JS1_A09_00480" 10966 "s59E5H2" 0.187571 "Peptide chain release factor 2"
"JS1_A09_00480" 10966 "s63E6H2" 0.1933686 "Peptide chain release factor 2"
"JS1_A09_00481" 10967 "s59E13H2" 0.01848153 "hypothetical protein"
"JS1_A09_00481" 10967 "s59E25H2" 0 "hypothetical protein"
"JS1_A09_00481" 10967 "s59E5H2" 0.1133266 "hypothetical protein"
"JS1_A09_00481" 10967 "s63E6H2" 0.07392824 "hypothetical protein"
"JS1_A09_00482" 10968 "s59E13H2" 0 "Methenyltetrahydrofolate cyclohydrolase"
"JS1_A09_00482" 10968 "s59E25H2" 0 "Methenyltetrahydrofolate cyclohydrolase"
"JS1_A09_00482" 10968 "s59E5H2" 0.01244458 "Methenyltetrahydrofolate cyclohydrolase"
"JS1_A09_00482" 10968 "s63E6H2" 0.05211237 "Methenyltetrahydrofolate cyclohydrolase"
"JS1_A09_00483" 10969 "s59E13H2" 0.03141419 "Glutamate 2,3-aminomutase"
"JS1_A09_00483" 10969 "s59E25H2" 0 "Glutamate 2,3-aminomutase"
"JS1_A09_00483" 10969 "s59E5H2" 0.07269589 "Glutamate 2,3-aminomutase"
"JS1_A09_00483" 10969 "s63E6H2" 0.1766648 "Glutamate 2,3-aminomutase"
"JS1_A09_00484" 10970 "s59E13H2" 0.05552638 "HTH-type transcriptional repressor YtrA"
"JS1_A09_00484" 10970 "s59E25H2" 0 "HTH-type transcriptional repressor YtrA"

"JS1_A09_00484" 10970 "s59E5H2" 0.3109926 "HTH-type transcriptional repressor YtrA"
"JS1_A09_00484" 10970 "s63E6H2" 0.2610078 "HTH-type transcriptional repressor YtrA"
"JS1_A09_00485" 10971 "s59E13H2" 0.02767925 "Ferredoxin"
"JS1_A09_00485" 10971 "s59E25H2" 0 "Ferredoxin"
"JS1_A09_00485" 10971 "s59E5H2" 0.2507148 "Ferredoxin"
"JS1_A09_00485" 10971 "s63E6H2" 0.2597583 "Ferredoxin"
"JS1_A09_00486" 10972 "s59E13H2" 0.1309699 "2-oxoglutarate synthase subunit KorA"
"JS1_A09_00486" 10972 "s59E25H2" 0 "2-oxoglutarate synthase subunit KorA"
"JS1_A09_00486" 10972 "s59E5H2" 0.3142409 "2-oxoglutarate synthase subunit KorA"
"JS1_A09_00486" 10972 "s63E6H2" 0.3311502 "2-oxoglutarate synthase subunit KorA"
"JS1_A09_00487" 10973 "s59E13H2" 0.1069128 "2-oxoglutarate synthase subunit KorB"
"JS1_A09_00487" 10973 "s59E25H2" 0 "2-oxoglutarate synthase subunit KorB"
"JS1_A09_00487" 10973 "s59E5H2" 0.3446755 "2-oxoglutarate synthase subunit KorB"
"JS1_A09_00487" 10973 "s63E6H2" 0.1960571 "2-oxoglutarate synthase subunit KorB"
"JS1_A09_00492" 10978 "s59E13H2" 0 "hypothetical protein"
"JS1_A09_00492" 10978 "s59E25H2" 0 "hypothetical protein"
"JS1_A09_00492" 10978 "s59E5H2" 0 "hypothetical protein"
"JS1_A09_00492" 10978 "s63E6H2" 1.041078 "hypothetical protein"
"JS1_A09_00493" 10979 "s59E13H2" 0.004637062 "hypothetical protein"
"JS1_A09_00493" 10979 "s59E25H2" 0 "hypothetical protein"
"JS1_A09_00493" 10979 "s59E5H2" 0.01048359 "hypothetical protein"
"JS1_A09_00493" 10979 "s63E6H2" 0.6926743 "hypothetical protein"
"JS1_A09_00494" 10980 "s59E13H2" 0 "Integrase core domain protein"
"JS1_A09_00494" 10980 "s59E25H2" 0 "Integrase core domain protein"
"JS1_A09_00494" 10980 "s59E5H2" 4.07384 "Integrase core domain protein"
"JS1_A09_00494" 10980 "s63E6H2" 3.609196 "Integrase core domain protein"
"JS1_A09_00495" 10981 "s59E13H2" 0 "Transposase"
"JS1_A09_00495" 10981 "s59E25H2" 0 "Transposase"
"JS1_A09_00495" 10981 "s59E5H2" 2.920382 "Transposase"
"JS1_A09_00495" 10981 "s63E6H2" 0.01292862 "Transposase"
"JS1_A09_00496" 10982 "s59E13H2" 0.06295881 "Transposase IS116/IS110/IS902 family protein"
"JS1_A09_00496" 10982 "s59E25H2" 0 "Transposase IS116/IS110/IS902 family protein"
"JS1_A09_00496" 10982 "s59E5H2" 0.01396123 "Transposase IS116/IS110/IS902 family protein"
"JS1_A09_00496" 10982 "s63E6H2" 0.2201532 "Transposase IS116/IS110/IS902 family protein"
"JS1_A09_00499" 10985 "s59E13H2" 0 "putative methyltransferase"
"JS1_A09_00499" 10985 "s59E25H2" 0 "putative methyltransferase"
"JS1_A09_00499" 10985 "s59E5H2" 0.02491041 "putative methyltransferase"
"JS1_A09_00499" 10985 "s63E6H2" 0 "putative methyltransferase"
"JS1_A09_00502" 10988 "s59E13H2" 0 "hypothetical protein"
"JS1_A09_00502" 10988 "s59E25H2" 0 "hypothetical protein"
"JS1_A09_00502" 10988 "s59E5H2" 0 "hypothetical protein"
"JS1_A09_00502" 10988 "s63E6H2" 0 "hypothetical protein"
"JS1_A09_00503" 10989 "s59E13H2" 0 "transcriptional repressor MprA"
"JS1_A09_00503" 10989 "s59E25H2" 0 "transcriptional repressor MprA"
"JS1_A09_00503" 10989 "s59E5H2" 0 "transcriptional repressor MprA"
"JS1_A09_00503" 10989 "s63E6H2" 0.02030565 "transcriptional repressor MprA"
"JS1_A09_00504" 10990 "s59E13H2" 0 "hypothetical protein"

"JS1_A09_00504" 10990 "s59E25H2" 0 "hypothetical protein"
"JS1_A09_00504" 10990 "s59E5H2" 0.3857224 "hypothetical protein"
"JS1_A09_00504" 10990 "s63E6H2" 0.09593594 "hypothetical protein"
"JS1_A09_00505" 10991 "s59E13H2" 0.02176673 "Heterodisulfide reductase subunit A-like protein"
"JS1_A09_00505" 10991 "s59E25H2" 0 "Heterodisulfide reductase subunit A-like protein"
"JS1_A09_00505" 10991 "s59E5H2" 0 "Heterodisulfide reductase subunit A-like protein"
"JS1_A09_00505" 10991 "s63E6H2" 0 "Heterodisulfide reductase subunit A-like protein"
"JS1_A09_00506" 10992 "s59E13H2" 0.0008300707 "Sulfide dehydrogenase subunit alpha precursor"
"JS1_A09_00506" 10992 "s59E25H2" 0 "Sulfide dehydrogenase subunit alpha precursor"
"JS1_A09_00506" 10992 "s59E5H2" 0.02137866 "Sulfide dehydrogenase subunit alpha precursor"
"JS1_A09_00506" 10992 "s63E6H2" 0.01320603 "Sulfide dehydrogenase subunit alpha precursor"
"JS1_A09_00508" 10994 "s59E13H2" 0 "methylcobalamin:coenzyme M methyltransferase"
"JS1_A09_00508" 10994 "s59E25H2" 0 "methylcobalamin:coenzyme M methyltransferase"
"JS1_A09_00508" 10994 "s59E5H2" 0 "methylcobalamin:coenzyme M methyltransferase"
"JS1_A09_00508" 10994 "s63E6H2" 0.001367428 "methylcobalamin:coenzyme M methyltransferase"
"JS1_A09_00509" 10995 "s59E13H2" 0.05769808 "4-hydroxy-tetrahydrodipicolinate synthase"
"JS1_A09_00509" 10995 "s59E25H2" 0 "4-hydroxy-tetrahydrodipicolinate synthase"
"JS1_A09_00509" 10995 "s59E5H2" 0.1298705 "4-hydroxy-tetrahydrodipicolinate synthase"
"JS1_A09_00509" 10995 "s63E6H2" 0.4044847 "4-hydroxy-tetrahydrodipicolinate synthase"
"JS1_A09_00510" 10996 "s59E13H2" 0 "4-hydroxy-tetrahydrodipicolinate synthase"
"JS1_A09_00510" 10996 "s59E25H2" 0 "4-hydroxy-tetrahydrodipicolinate synthase"
"JS1_A09_00510" 10996 "s59E5H2" 0 "4-hydroxy-tetrahydrodipicolinate synthase"
"JS1_A09_00510" 10996 "s63E6H2" 0 "4-hydroxy-tetrahydrodipicolinate synthase"
"JS1_A09_00511" 10997 "s59E13H2" 0.02098479 "putative oxidoreductase YvaA"
"JS1_A09_00511" 10997 "s59E25H2" 0 "putative oxidoreductase YvaA"
"JS1_A09_00511" 10997 "s59E5H2" 0.1196626 "putative oxidoreductase YvaA"
"JS1_A09_00511" 10997 "s63E6H2" 0.006067155 "putative oxidoreductase YvaA"
"JS1_A09_00512" 10998 "s59E13H2" 0 "Tripartite tricarboxylate transporter family receptor"
"JS1_A09_00512" 10998 "s59E25H2" 0 "Tripartite tricarboxylate transporter family receptor"
"JS1_A09_00512" 10998 "s59E5H2" 0.009622011 "Tripartite tricarboxylate transporter family receptor"
"JS1_A09_00512" 10998 "s63E6H2" 0 "Tripartite tricarboxylate transporter family receptor"
"JS1_A09_00513" 10999 "s59E13H2" 0 "Tripartite tricarboxylate transporter TctB family protein"
"JS1_A09_00513" 10999 "s59E25H2" 0 "Tripartite tricarboxylate transporter TctB family protein"
"JS1_A09_00513" 10999 "s59E5H2" 0.009686255 "Tripartite tricarboxylate transporter TctB family protein"
"JS1_A09_00513" 10999 "s63E6H2" 0 "Tripartite tricarboxylate transporter TctB family protein"
"JS1_A09_00514" 11000 "s59E13H2" 0 "Tripartite tricarboxylate transporter TctA family protein"
"JS1_A09_00514" 11000 "s59E25H2" 0 "Tripartite tricarboxylate transporter TctA family protein"
"JS1_A09_00514" 11000 "s59E5H2" 0.04423916 "Tripartite tricarboxylate transporter TctA family protein"
"JS1_A09_00514" 11000 "s63E6H2" 0 "Tripartite tricarboxylate transporter TctA family protein"
"JS1_A09_00515" 11001 "s59E13H2" 0.4298031 "Serine--tRNA ligase"
"JS1_A09_00515" 11001 "s59E25H2" 0.1878931 "Serine--tRNA ligase"
"JS1_A09_00515" 11001 "s59E5H2" 1.532034 "Serine--tRNA ligase"
"JS1_A09_00515" 11001 "s63E6H2" 1.087691 "Serine--tRNA ligase"
"JS1_A09_00516" 11002 "s59E13H2" 0.1026021 "tRNA-splicing ligase RtcB"
"JS1_A09_00516" 11002 "s59E25H2" 0 "tRNA-splicing ligase RtcB"
"JS1_A09_00516" 11002 "s59E5H2" 0.9590941 "tRNA-splicing ligase RtcB"
"JS1_A09_00516" 11002 "s63E6H2" 0.08313366 "tRNA-splicing ligase RtcB"

"JS1_A09_00517" 11003 "s59E13H2" 0.07995666 "hypothetical protein"
"JS1_A09_00517" 11003 "s59E25H2" 0 "hypothetical protein"
"JS1_A09_00517" 11003 "s59E5H2" 0.3926588 "hypothetical protein"
"JS1_A09_00517" 11003 "s63E6H2" 0.0261438 "hypothetical protein"
"JS1_A09_00519" 11005 "s59E13H2" 0.02320732 "Glyoxylate reductase"
"JS1_A09_00519" 11005 "s59E25H2" 0 "Glyoxylate reductase"
"JS1_A09_00519" 11005 "s59E5H2" 0.004309448 "Glyoxylate reductase"
"JS1_A09_00519" 11005 "s63E6H2" 0.01241509 "Glyoxylate reductase"
"JS1_A09_00520" 11006 "s59E13H2" 0.01807933 "ThiS family protein"
"JS1_A09_00520" 11006 "s59E25H2" 0 "ThiS family protein"
"JS1_A09_00520" 11006 "s59E5H2" 0 "ThiS family protein"
"JS1_A09_00520" 11006 "s63E6H2" 0.001434439 "ThiS family protein"
"JS1_A09_00521" 11007 "s59E13H2" 0.06345212 "Tungsten-containing aldehyde ferredoxin oxidoreductase"
"JS1_A09_00521" 11007 "s59E25H2" 0 "Tungsten-containing aldehyde ferredoxin oxidoreductase"
"JS1_A09_00521" 11007 "s59E5H2" 0 "Tungsten-containing aldehyde ferredoxin oxidoreductase"
"JS1_A09_00521" 11007 "s63E6H2" 0.01559383 "Tungsten-containing aldehyde ferredoxin oxidoreductase"
"JS1_A09_00522" 11008 "s59E13H2" 0 "Inositol 2-dehydrogenase/D-chiro-inositol 3-dehydrogenase"
"JS1_A09_00522" 11008 "s59E25H2" 0 "Inositol 2-dehydrogenase/D-chiro-inositol 3-dehydrogenase"
"JS1_A09_00522" 11008 "s59E5H2" 0 "Inositol 2-dehydrogenase/D-chiro-inositol 3-dehydrogenase"
"JS1_A09_00522" 11008 "s63E6H2" 0.002386151 "Inositol 2-dehydrogenase/D-chiro-inositol 3-dehydrogenase"
"JS1_A09_00523" 11009 "s59E13H2" 2.928092 "KHG/KDPG aldolase"
"JS1_A09_00523" 11009 "s59E25H2" 0 "KHG/KDPG aldolase"
"JS1_A09_00523" 11009 "s59E5H2" 0 "KHG/KDPG aldolase"
"JS1_A09_00523" 11009 "s63E6H2" 0.003030349 "KHG/KDPG aldolase"
"JS1_A09_00524" 11010 "s59E13H2" 11.05719 "Xylulose kinase"
"JS1_A09_00524" 11010 "s59E25H2" 0.1584284 "Xylulose kinase"
"JS1_A09_00524" 11010 "s59E5H2" 0 "Xylulose kinase"
"JS1_A09_00524" 11010 "s63E6H2" 0.0009669846 "Xylulose kinase"
"JS1_A09_00525" 11011 "s59E13H2" 6.438912 "Xylulose kinase"
"JS1_A09_00525" 11011 "s59E25H2" 0 "Xylulose kinase"
"JS1_A09_00525" 11011 "s59E5H2" 0.02265916 "Xylulose kinase"
"JS1_A09_00525" 11011 "s63E6H2" 0.05830528 "Xylulose kinase"
"JS1_A09_00528" 11014 "s59E13H2" 0.05671001 "Ribosomal RNA small subunit methyltransferase I"
"JS1_A09_00528" 11014 "s59E25H2" 0 "Ribosomal RNA small subunit methyltransferase I"
"JS1_A09_00528" 11014 "s59E5H2" 0.0694719 "Ribosomal RNA small subunit methyltransferase I"
"JS1_A09_00528" 11014 "s63E6H2" 0.01752588 "Ribosomal RNA small subunit methyltransferase I"
"JS1_A09_00529" 11015 "s59E13H2" 0.1235922 "Methionine--tRNA ligase"
"JS1_A09_00529" 11015 "s59E25H2" 0 "Methionine--tRNA ligase"
"JS1_A09_00529" 11015 "s59E5H2" 0.1368203 "Methionine--tRNA ligase"
"JS1_A09_00529" 11015 "s63E6H2" 0.1262402 "Methionine--tRNA ligase"
"JS1_A09_00530" 11016 "s59E13H2" 0.2078341 "Membrane-bound lytic murein transglycosylase C precursor"
"JS1_A09_00530" 11016 "s59E25H2" 0 "Membrane-bound lytic murein transglycosylase C precursor"
"JS1_A09_00530" 11016 "s59E5H2" 0.1826411 "Membrane-bound lytic murein transglycosylase C precursor"
"JS1_A09_00530" 11016 "s63E6H2" 0.08456181 "Membrane-bound lytic murein transglycosylase C precursor"
"JS1_A09_00532" 11018 "s59E13H2" 0.196931 "Riboflavin transporter RibU"
"JS1_A09_00532" 11018 "s59E25H2" 0 "Riboflavin transporter RibU"
"JS1_A09_00532" 11018 "s59E5H2" 0.1334797 "Riboflavin transporter RibU"

"JS1_A09_00532" 11018 "s63E6H2" 0.1598819 "Riboflavin transporter RibU"
"JS1_A09_00533" 11019 "s59E13H2" 0.2544492 "Mannose-1-phosphate guanylyltransferase RfbM"
"JS1_A09_00533" 11019 "s59E25H2" 0 "Mannose-1-phosphate guanylyltransferase RfbM"
"JS1_A09_00533" 11019 "s59E5H2" 0.2210229 "Mannose-1-phosphate guanylyltransferase RfbM"
"JS1_A09_00533" 11019 "s63E6H2" 0.2169791 "Mannose-1-phosphate guanylyltransferase RfbM"
"JS1_A09_00534" 11020 "s59E13H2" 0.2739876 "S-adenosylmethionine synthase"
"JS1_A09_00534" 11020 "s59E25H2" 0 "S-adenosylmethionine synthase"
"JS1_A09_00534" 11020 "s59E5H2" 0.17023 "S-adenosylmethionine synthase"
"JS1_A09_00534" 11020 "s63E6H2" 0.3096629 "S-adenosylmethionine synthase"
"JS1_A09_00536" 11022 "s59E13H2" 0.2381419 "Twitching mobility protein"
"JS1_A09_00536" 11022 "s59E25H2" 0 "Twitching mobility protein"
"JS1_A09_00536" 11022 "s59E5H2" 0.1732607 "Twitching mobility protein"
"JS1_A09_00536" 11022 "s63E6H2" 0.5730426 "Twitching mobility protein"
"JS1_A09_00538" 11024 "s59E13H2" 0.1396503 "bacteriophage N4 adsorption protein B"
"JS1_A09_00538" 11024 "s59E25H2" 0 "bacteriophage N4 adsorption protein B"
"JS1_A09_00538" 11024 "s59E5H2" 0.2318103 "bacteriophage N4 adsorption protein B"
"JS1_A09_00538" 11024 "s63E6H2" 0.3720394 "bacteriophage N4 adsorption protein B"
"JS1_A09_00539" 11025 "s59E13H2" 0.09815333 "Twitching mobility protein"
"JS1_A09_00539" 11025 "s59E25H2" 0 "Twitching mobility protein"
"JS1_A09_00539" 11025 "s59E5H2" 0.1266475 "Twitching mobility protein"
"JS1_A09_00539" 11025 "s63E6H2" 0.401302 "Twitching mobility protein"
"JS1_A09_00540" 11026 "s59E13H2" 0.09581967 "Histidine--tRNA ligase"
"JS1_A09_00540" 11026 "s59E25H2" 0 "Histidine--tRNA ligase"
"JS1_A09_00540" 11026 "s59E5H2" 0.04990214 "Histidine--tRNA ligase"
"JS1_A09_00540" 11026 "s63E6H2" 0.4920152 "Histidine--tRNA ligase"
"JS1_A09_00541" 11027 "s59E13H2" 0.1195331 "Asparagine--tRNA ligase"
"JS1_A09_00541" 11027 "s59E25H2" 0 "Asparagine--tRNA ligase"
"JS1_A09_00541" 11027 "s59E5H2" 0.1095293 "Asparagine--tRNA ligase"
"JS1_A09_00541" 11027 "s63E6H2" 0.6099038 "Asparagine--tRNA ligase"
"JS1_A09_00542" 11028 "s59E13H2" 0.1233918 "Aspartate--tRNA ligase"
"JS1_A09_00542" 11028 "s59E25H2" 0 "Aspartate--tRNA ligase"
"JS1_A09_00542" 11028 "s59E5H2" 0.09208085 "Aspartate--tRNA ligase"
"JS1_A09_00542" 11028 "s63E6H2" 0.5263425 "Aspartate--tRNA ligase"
"JS1_A09_00544" 11030 "s59E13H2" 0.09354892 "putative 5-formyltetrahydrofolate cyclo-ligase"
"JS1_A09_00544" 11030 "s59E25H2" 0.3415301 "putative 5-formyltetrahydrofolate cyclo-ligase"
"JS1_A09_00544" 11030 "s59E5H2" 0.09465703 "putative 5-formyltetrahydrofolate cyclo-ligase"
"JS1_A09_00544" 11030 "s63E6H2" 0.3418022 "putative 5-formyltetrahydrofolate cyclo-ligase"
"JS1_A09_00545" 11031 "s59E13H2" 0.08897843 "30S ribosomal protein S1"
"JS1_A09_00545" 11031 "s59E25H2" 0 "30S ribosomal protein S1"
"JS1_A09_00545" 11031 "s59E5H2" 0.09779204 "30S ribosomal protein S1"
"JS1_A09_00545" 11031 "s63E6H2" 0.59077 "30S ribosomal protein S1"
"JS1_A09_00546" 11032 "s59E13H2" 0.01521232 "Type II secretion system protein E"
"JS1_A09_00546" 11032 "s59E25H2" 0 "Type II secretion system protein E"
"JS1_A09_00546" 11032 "s59E5H2" 0.2003793 "Type II secretion system protein E"
"JS1_A09_00546" 11032 "s63E6H2" 0.8346128 "Type II secretion system protein E"
"JS1_A09_00547" 11033 "s59E13H2" 0.06247501 "Alanine--tRNA ligase"
"JS1_A09_00547" 11033 "s59E25H2" 0 "Alanine--tRNA ligase"

"JS1_A09_00547" 11033 "s59E5H2" 0.06872338 "Alanine--tRNA ligase"
"JS1_A09_00547" 11033 "s63E6H2" 0.2355999 "Alanine--tRNA ligase"
"JS1_A09_00548" 11034 "s59E13H2" 0.05004162 "Putative Holliday junction resolvase"
"JS1_A09_00548" 11034 "s59E25H2" 0 "Putative Holliday junction resolvase"
"JS1_A09_00548" 11034 "s59E5H2" 0.0643966 "Putative Holliday junction resolvase"
"JS1_A09_00548" 11034 "s63E6H2" 0.1045159 "Putative Holliday junction resolvase"
"JS1_A09_00549" 11035 "s59E13H2" 0.003798599 "putative aminodeoxychorismate lyase"
"JS1_A09_00549" 11035 "s59E25H2" 0 "putative aminodeoxychorismate lyase"
"JS1_A09_00549" 11035 "s59E5H2" 0.07273188 "putative aminodeoxychorismate lyase"
"JS1_A09_00549" 11035 "s63E6H2" 0.08521201 "putative aminodeoxychorismate lyase"
"JS1_A09_00550" 11036 "s59E13H2" 0.01738364 "Uridine kinase"
"JS1_A09_00550" 11036 "s59E25H2" 0 "Uridine kinase"
"JS1_A09_00550" 11036 "s59E5H2" 0.1277265 "Uridine kinase"
"JS1_A09_00550" 11036 "s63E6H2" 0.07079431 "Uridine kinase"
"JS1_A09_00551" 11037 "s59E13H2" 0.007807031 "Mitochondrial PGP phosphatase"
"JS1_A09_00551" 11037 "s59E25H2" 0 "Mitochondrial PGP phosphatase"
"JS1_A09_00551" 11037 "s59E5H2" 0.1328996 "Mitochondrial PGP phosphatase"
"JS1_A09_00551" 11037 "s63E6H2" 0.2341614 "Mitochondrial PGP phosphatase"
"JS1_A09_00552" 11038 "s59E13H2" 0.3596996 "hypothetical protein"
"JS1_A09_00552" 11038 "s59E25H2" 0 "hypothetical protein"
"JS1_A09_00552" 11038 "s59E5H2" 0.2573745 "hypothetical protein"
"JS1_A09_00552" 11038 "s63E6H2" 0.2959223 "hypothetical protein"
"JS1_A09_00553" 11039 "s59E13H2" 0.8049866 "Putative type II secretion system protein E"
"JS1_A09_00553" 11039 "s59E25H2" 0 "Putative type II secretion system protein E"
"JS1_A09_00553" 11039 "s59E5H2" 0.9875412 "Putative type II secretion system protein E"
"JS1_A09_00553" 11039 "s63E6H2" 0.7612916 "Putative type II secretion system protein E"
"JS1_A09_00554" 11040 "s59E13H2" 0.983044 "Cyclic di-GMP phosphodiesterase response regulator RpfG"
"JS1_A09_00554" 11040 "s59E25H2" 0 "Cyclic di-GMP phosphodiesterase response regulator RpfG"
"JS1_A09_00554" 11040 "s59E5H2" 0.586248 "Cyclic di-GMP phosphodiesterase response regulator RpfG"
"JS1_A09_00554" 11040 "s63E6H2" 0.7253703 "Cyclic di-GMP phosphodiesterase response regulator RpfG"
"JS1_A09_00555" 11041 "s59E13H2" 0.4799015 "Type II secretion system protein F"
"JS1_A09_00555" 11041 "s59E25H2" 0.140264 "Type II secretion system protein F"
"JS1_A09_00555" 11041 "s59E5H2" 1.018035 "Type II secretion system protein F"
"JS1_A09_00555" 11041 "s63E6H2" 1.28896 "Type II secretion system protein F"
"JS1_A09_00556" 11042 "s59E13H2" 0.6747022 "RNA polymerase sigma factor SigF"
"JS1_A09_00556" 11042 "s59E25H2" 0 "RNA polymerase sigma factor SigF"
"JS1_A09_00556" 11042 "s59E5H2" 0.1881378 "RNA polymerase sigma factor SigF"
"JS1_A09_00556" 11042 "s63E6H2" 1.172681 "RNA polymerase sigma factor SigF"
"JS1_A09_00557" 11043 "s59E13H2" 0.4317609 "RNA polymerase sigma-B factor"
"JS1_A09_00557" 11043 "s59E25H2" 0 "RNA polymerase sigma-B factor"
"JS1_A09_00557" 11043 "s59E5H2" 0.1306209 "RNA polymerase sigma-B factor"
"JS1_A09_00557" 11043 "s63E6H2" 0.7921051 "RNA polymerase sigma-B factor"
"JS1_A09_00558" 11044 "s59E13H2" 0.08441108 "Transposase DDE domain protein"
"JS1_A09_00558" 11044 "s59E25H2" 0 "Transposase DDE domain protein"
"JS1_A09_00558" 11044 "s59E5H2" 1.774046 "Transposase DDE domain protein"
"JS1_A09_00558" 11044 "s63E6H2" 1.353576 "Transposase DDE domain protein"
"JS1_A09_00559" 11045 "s59E13H2" 0 "hypothetical protein"

"JS1_A09_00559" 11045 "s59E25H2" 0 "hypothetical protein"
"JS1_A09_00559" 11045 "s59E5H2" 0 "hypothetical protein"
"JS1_A09_00559" 11045 "s63E6H2" 0.3485275 "hypothetical protein"
"JS1_A09_00560" 11046 "s59E13H2" 0.09058371 "Caspase domain protein"
"JS1_A09_00560" 11046 "s59E25H2" 0 "Caspase domain protein"
"JS1_A09_00560" 11046 "s59E5H2" 0.1275709 "Caspase domain protein"
"JS1_A09_00560" 11046 "s63E6H2" 0.04415526 "Caspase domain protein"
"JS1_A09_00561" 11047 "s59E13H2" 0.1524043 "hypothetical protein"
"JS1_A09_00561" 11047 "s59E25H2" 0 "hypothetical protein"
"JS1_A09_00561" 11047 "s59E5H2" 0.5411383 "hypothetical protein"
"JS1_A09_00561" 11047 "s63E6H2" 1.484487 "hypothetical protein"
"JS1_A09_00562" 11048 "s59E13H2" 0.05545058 "hypothetical protein"
"JS1_A09_00562" 11048 "s59E25H2" 0 "hypothetical protein"
"JS1_A09_00562" 11048 "s59E5H2" 0.4385154 "hypothetical protein"
"JS1_A09_00562" 11048 "s63E6H2" 0.5232581 "hypothetical protein"
"JS1_A09_00563" 11049 "s59E13H2" 0.03916724 "Integrase core domain protein"
"JS1_A09_00563" 11049 "s59E25H2" 0 "Integrase core domain protein"
"JS1_A09_00563" 11049 "s59E5H2" 0.1134409 "Integrase core domain protein"
"JS1_A09_00563" 11049 "s63E6H2" 7.400676 "Integrase core domain protein"
"JS1_A09_00564" 11050 "s59E13H2" 0.6881698 "Integrase core domain protein"
"JS1_A09_00564" 11050 "s59E25H2" 0 "Integrase core domain protein"
"JS1_A09_00564" 11050 "s59E5H2" 0.7225327 "Integrase core domain protein"
"JS1_A09_00564" 11050 "s63E6H2" 1.649763 "Integrase core domain protein"
"JS1_A09_00566" 11052 "s59E13H2" 0.3516724 "Lon protease 1"
"JS1_A09_00566" 11052 "s59E25H2" 0 "Lon protease 1"
"JS1_A09_00566" 11052 "s59E5H2" 0.2455731 "Lon protease 1"
"JS1_A09_00566" 11052 "s63E6H2" 0.1318086 "Lon protease 1"
"JS1_A09_00567" 11053 "s59E13H2" 0.1895504 "Lon protease"
"JS1_A09_00567" 11053 "s59E25H2" 0 "Lon protease"
"JS1_A09_00567" 11053 "s59E5H2" 0.08471858 "Lon protease"
"JS1_A09_00567" 11053 "s63E6H2" 0.07908603 "Lon protease"
"JS1_A09_00568" 11054 "s59E13H2" 0.105885 "ATP-dependent Clp protease ATP-binding subunit ClpX"
"JS1_A09_00568" 11054 "s59E25H2" 0 "ATP-dependent Clp protease ATP-binding subunit ClpX"
"JS1_A09_00568" 11054 "s59E5H2" 0.132992 "ATP-dependent Clp protease ATP-binding subunit ClpX"
"JS1_A09_00568" 11054 "s63E6H2" 0.08149483 "ATP-dependent Clp protease ATP-binding subunit ClpX"
"JS1_A09_00569" 11055 "s59E13H2" 0.2605217 "ATP-dependent Clp protease proteolytic subunit"
"JS1_A09_00569" 11055 "s59E25H2" 0 "ATP-dependent Clp protease proteolytic subunit"
"JS1_A09_00569" 11055 "s59E5H2" 0.09219072 "ATP-dependent Clp protease proteolytic subunit"
"JS1_A09_00569" 11055 "s63E6H2" 0.1178065 "ATP-dependent Clp protease proteolytic subunit"
"JS1_A09_00570" 11056 "s59E13H2" 0.2304588 "Trigger factor"
"JS1_A09_00570" 11056 "s59E25H2" 0 "Trigger factor"
"JS1_A09_00570" 11056 "s59E5H2" 0.04118789 "Trigger factor"
"JS1_A09_00570" 11056 "s63E6H2" 0.09586536 "Trigger factor"
"JS1_A09_00578" 11064 "s59E13H2" 0.02204388 "Non-canonical purine NTP pyrophosphatase"
"JS1_A09_00578" 11064 "s59E25H2" 0 "Non-canonical purine NTP pyrophosphatase"
"JS1_A09_00578" 11064 "s59E5H2" 0.03730084 "Non-canonical purine NTP pyrophosphatase"
"JS1_A09_00578" 11064 "s63E6H2" 0.09882742 "Non-canonical purine NTP pyrophosphatase"

"JS1_A09_00579" 11065 "s59E13H2" 0.02208353 "Ribonuclease PH"
"JS1_A09_00579" 11065 "s59E25H2" 0 "Ribonuclease PH"
"JS1_A09_00579" 11065 "s59E5H2" 0.145191 "Ribonuclease PH"
"JS1_A09_00579" 11065 "s63E6H2" 0.2639132 "Ribonuclease PH"
"JS1_A09_00580" 11066 "s59E13H2" 0.0291634 "Farnesyl diphosphate synthase"
"JS1_A09_00580" 11066 "s59E25H2" 0 "Farnesyl diphosphate synthase"
"JS1_A09_00580" 11066 "s59E5H2" 0.06680514 "Farnesyl diphosphate synthase"
"JS1_A09_00580" 11066 "s63E6H2" 0.1697894 "Farnesyl diphosphate synthase"
"JS1_A09_00581" 11067 "s59E13H2" 0.01702832 "Exodeoxyribonuclease 7 small subunit"
"JS1_A09_00581" 11067 "s59E25H2" 0 "Exodeoxyribonuclease 7 small subunit"
"JS1_A09_00581" 11067 "s59E5H2" 0.04839502 "Exodeoxyribonuclease 7 small subunit"
"JS1_A09_00581" 11067 "s63E6H2" 0.2065833 "Exodeoxyribonuclease 7 small subunit"
"JS1_A09_00582" 11068 "s59E13H2" 0.03509464 "Exodeoxyribonuclease 7 large subunit"
"JS1_A09_00582" 11068 "s59E25H2" 0 "Exodeoxyribonuclease 7 large subunit"
"JS1_A09_00582" 11068 "s59E5H2" 0.06785203 "Exodeoxyribonuclease 7 large subunit"
"JS1_A09_00582" 11068 "s63E6H2" 0.197656 "Exodeoxyribonuclease 7 large subunit"
"JS1_A09_00583" 11069 "s59E13H2" 0.03142349 "hypothetical protein"
"JS1_A09_00583" 11069 "s59E25H2" 0 "hypothetical protein"
"JS1_A09_00583" 11069 "s59E5H2" 0.06852619 "hypothetical protein"
"JS1_A09_00583" 11069 "s63E6H2" 0.219803 "hypothetical protein"
"JS1_A09_00584" 11070 "s59E13H2" 0.03345542 "hypothetical protein"
"JS1_A09_00584" 11070 "s59E25H2" 0 "hypothetical protein"
"JS1_A09_00584" 11070 "s59E5H2" 0.09376927 "hypothetical protein"
"JS1_A09_00584" 11070 "s63E6H2" 0.1459315 "hypothetical protein"
"JS1_A09_00585" 11071 "s59E13H2" 0.03280846 "Elongation factor P"
"JS1_A09_00585" 11071 "s59E25H2" 0 "Elongation factor P"
"JS1_A09_00585" 11071 "s59E5H2" 0.1506122 "Elongation factor P"
"JS1_A09_00585" 11071 "s63E6H2" 0.1339514 "Elongation factor P"
"JS1_A09_00586" 11072 "s59E13H2" 0.05022539 "Serine hydroxymethyltransferase"
"JS1_A09_00586" 11072 "s59E25H2" 0 "Serine hydroxymethyltransferase"
"JS1_A09_00586" 11072 "s59E5H2" 0.1757658 "Serine hydroxymethyltransferase"
"JS1_A09_00586" 11072 "s63E6H2" 0.09954775 "Serine hydroxymethyltransferase"
"JS1_A09_00587" 11073 "s59E13H2" 0.07394961 "Xaa-Pro dipeptidase"
"JS1_A09_00587" 11073 "s59E25H2" 0 "Xaa-Pro dipeptidase"
"JS1_A09_00587" 11073 "s59E5H2" 0.1558028 "Xaa-Pro dipeptidase"
"JS1_A09_00587" 11073 "s63E6H2" 0.01857374 "Xaa-Pro dipeptidase"
"JS1_A09_00588" 11074 "s59E13H2" 0.0480108 "Ribosomal protein S12 methylthiotransferase RimO"
"JS1_A09_00588" 11074 "s59E25H2" 0 "Ribosomal protein S12 methylthiotransferase RimO"
"JS1_A09_00588" 11074 "s59E5H2" 0.01262486 "Ribosomal protein S12 methylthiotransferase RimO"
"JS1_A09_00588" 11074 "s63E6H2" 0.05703205 "Ribosomal protein S12 methylthiotransferase RimO"
"JS1_A09_00589" 11075 "s59E13H2" 0 "Helix-turn-helix domain protein"
"JS1_A09_00589" 11075 "s59E25H2" 0 "Helix-turn-helix domain protein"
"JS1_A09_00589" 11075 "s59E5H2" 0 "Helix-turn-helix domain protein"
"JS1_A09_00589" 11075 "s63E6H2" 0 "Helix-turn-helix domain protein"
"JS1_A09_00590" 11076 "s59E13H2" 0 "hypothetical protein"
"JS1_A09_00590" 11076 "s59E25H2" 0 "hypothetical protein"
"JS1_A09_00590" 11076 "s59E5H2" 0 "hypothetical protein"

"JS1_A09_00590" 11076 "s63E6H2" 0 "hypothetical protein"
"JS1_A09_00591" 11077 "s59E13H2" 0 "hypothetical protein"
"JS1_A09_00591" 11077 "s59E25H2" 0 "hypothetical protein"
"JS1_A09_00591" 11077 "s59E5H2" 0 "hypothetical protein"
"JS1_A09_00591" 11077 "s63E6H2" 0 "hypothetical protein"
"JS1_A09_00592" 11078 "s59E13H2" 0.1943078 "Transposase IS200 like protein"
"JS1_A09_00592" 11078 "s59E25H2" 0 "Transposase IS200 like protein"
"JS1_A09_00592" 11078 "s59E5H2" 0 "Transposase IS200 like protein"
"JS1_A09_00592" 11078 "s63E6H2" 0.03874185 "Transposase IS200 like protein"
"JS1_A09_00593" 11079 "s59E13H2" 0.080565 "Formamidase"
"JS1_A09_00593" 11079 "s59E25H2" 0 "Formamidase"
"JS1_A09_00593" 11079 "s59E5H2" 0.09607565 "Formamidase"
"JS1_A09_00593" 11079 "s63E6H2" 0.02428959 "Formamidase"
"JS1_A09_00594" 11080 "s59E13H2" 0 "leucine/isoleucine/valine transporter permease subunit"
"JS1_A09_00594" 11080 "s59E25H2" 0 "leucine/isoleucine/valine transporter permease subunit"
"JS1_A09_00594" 11080 "s59E5H2" 0.04505818 "leucine/isoleucine/valine transporter permease subunit"
"JS1_A09_00594" 11080 "s63E6H2" 0.03682691 "leucine/isoleucine/valine transporter permease subunit"
"JS1_A09_00595" 11081 "s59E13H2" 0 "High-affinity branched-chain amino acid transport system permease protein LivH"
"JS1_A09_00595" 11081 "s59E25H2" 0 "High-affinity branched-chain amino acid transport system permease protein LivH"
"JS1_A09_00595" 11081 "s59E5H2" 0.01839818 "High-affinity branched-chain amino acid transport system permease protein LivH"
"JS1_A09_00595" 11081 "s63E6H2" 0.02078424 "High-affinity branched-chain amino acid transport system permease protein LivH"
"JS1_A09_00596" 11082 "s59E13H2" 0 "High-affinity branched-chain amino acid transport ATP-binding protein LivF"
"JS1_A09_00596" 11082 "s59E25H2" 0 "High-affinity branched-chain amino acid transport ATP-binding protein LivF"
"JS1_A09_00596" 11082 "s59E5H2" 0.001322476 "High-affinity branched-chain amino acid transport ATP-binding protein LivF"
"JS1_A09_00596" 11082 "s63E6H2" 0.04097321 "High-affinity branched-chain amino acid transport ATP-binding protein LivF"
"JS1_A09_00597" 11083 "s59E13H2" 0 "Lipopolysaccharide export system ATP-binding protein LptB"
"JS1_A09_00597" 11083 "s59E25H2" 0 "Lipopolysaccharide export system ATP-binding protein LptB"
"JS1_A09_00597" 11083 "s59E5H2" 0.03024514 "Lipopolysaccharide export system ATP-binding protein LptB"
"JS1_A09_00597" 11083 "s63E6H2" 0.02364168 "Lipopolysaccharide export system ATP-binding protein LptB"
"JS1_A09_00598" 11084 "s59E13H2" 0 "Leucine-, isoleucine-, valine-, threonine-, and alanine-binding protein precursor"
"JS1_A09_00598" 11084 "s59E25H2" 0 "Leucine-, isoleucine-, valine-, threonine-, and alanine-binding protein precursor"
"JS1_A09_00598" 11084 "s59E5H2" 0.06146472 "Leucine-, isoleucine-, valine-, threonine-, and alanine-binding protein precursor"
"JS1_A09_00598" 11084 "s63E6H2" 0.01580615 "Leucine-, isoleucine-, valine-, threonine-, and alanine-binding protein precursor"
"JS1_A09_00601" 11087 "s59E13H2" 0.6393929 "SsrA-binding protein"
"JS1_A09_00601" 11087 "s59E25H2" 0 "SsrA-binding protein"
"JS1_A09_00601" 11087 "s59E5H2" 0.361319 "SsrA-binding protein"
"JS1_A09_00601" 11087 "s63E6H2" 0.2675141 "SsrA-binding protein"
"JS1_A09_00602" 11088 "s59E13H2" 0.08256056 "prephenate dehydrogenase"
"JS1_A09_00602" 11088 "s59E25H2" 0 "prephenate dehydrogenase"
"JS1_A09_00602" 11088 "s59E5H2" 0.2754873 "prephenate dehydrogenase"
"JS1_A09_00602" 11088 "s63E6H2" 0.08607769 "prephenate dehydrogenase"
"JS1_A09_00603" 11089 "s59E13H2" 0.04385115 "3-dehydroquinate dehydratase"
"JS1_A09_00603" 11089 "s59E25H2" 0 "3-dehydroquinate dehydratase"
"JS1_A09_00603" 11089 "s59E5H2" 0.3936929 "3-dehydroquinate dehydratase"
"JS1_A09_00603" 11089 "s63E6H2" 0.1860263 "3-dehydroquinate dehydratase"
"JS1_A09_00604" 11090 "s59E13H2" 0.07042688 "3-dehydroquinate synthase"
"JS1_A09_00604" 11090 "s59E25H2" 0 "3-dehydroquinate synthase"

"JS1_A09_00604" 11090 "s59E5H2" 0.5747745 "3-dehydroquinate synthase"
"JS1_A09_00604" 11090 "s63E6H2" 0.2963837 "3-dehydroquinate synthase"
"JS1_A09_00605" 11091 "s59E13H2" 0.06906494 "Shikimate kinase 1"
"JS1_A09_00605" 11091 "s59E25H2" 0 "Shikimate kinase 1"
"JS1_A09_00605" 11091 "s59E5H2" 0.1896137 "Shikimate kinase 1"
"JS1_A09_00605" 11091 "s63E6H2" 0.09552829 "Shikimate kinase 1"
"JS1_A09_00606" 11092 "s59E13H2" 0.05276928 "Chorismate synthase"
"JS1_A09_00606" 11092 "s59E25H2" 0 "Chorismate synthase"
"JS1_A09_00606" 11092 "s59E5H2" 0.1131012 "Chorismate synthase"
"JS1_A09_00606" 11092 "s63E6H2" 0.07333242 "Chorismate synthase"
"JS1_A09_00607" 11093 "s59E13H2" 0 "Quinate/shikimate dehydrogenase"
"JS1_A09_00607" 11093 "s59E25H2" 0 "Quinate/shikimate dehydrogenase"
"JS1_A09_00607" 11093 "s59E5H2" 0.1402607 "Quinate/shikimate dehydrogenase"
"JS1_A09_00607" 11093 "s63E6H2" 0.03234543 "Quinate/shikimate dehydrogenase"
"JS1_A09_00608" 11094 "s59E13H2" 0 "hypothetical protein"
"JS1_A09_00608" 11094 "s59E25H2" 0 "hypothetical protein"
"JS1_A09_00608" 11094 "s59E5H2" 0.1168328 "hypothetical protein"
"JS1_A09_00608" 11094 "s63E6H2" 0.00236653 "hypothetical protein"
"JS1_A09_00609" 11095 "s59E13H2" 0 "Malate dehydrogenase"
"JS1_A09_00609" 11095 "s59E25H2" 0 "Malate dehydrogenase"
"JS1_A09_00609" 11095 "s59E5H2" 0.055114 "Malate dehydrogenase"
"JS1_A09_00609" 11095 "s63E6H2" 0.02441655 "Malate dehydrogenase"
"JS1_A09_00610" 11096 "s59E13H2" 0 "Sialic acid TRAP transporter permease protein SiaT"
"JS1_A09_00610" 11096 "s59E25H2" 0 "Sialic acid TRAP transporter permease protein SiaT"
"JS1_A09_00610" 11096 "s59E5H2" 0.05539009 "Sialic acid TRAP transporter permease protein SiaT"
"JS1_A09_00610" 11096 "s63E6H2" 0.06776813 "Sialic acid TRAP transporter permease protein SiaT"
"JS1_A09_00611" 11097 "s59E13H2" 0 "Sialic acid TRAP transporter permease protein SiaT"
"JS1_A09_00611" 11097 "s59E25H2" 0 "Sialic acid TRAP transporter permease protein SiaT"
"JS1_A09_00611" 11097 "s59E5H2" 0.04849925 "Sialic acid TRAP transporter permease protein SiaT"
"JS1_A09_00611" 11097 "s63E6H2" 0.03306012 "Sialic acid TRAP transporter permease protein SiaT"
"JS1_A09_00612" 11098 "s59E13H2" 0 "Sialic acid-binding periplasmic protein SiaP precursor"
"JS1_A09_00612" 11098 "s59E25H2" 0 "Sialic acid-binding periplasmic protein SiaP precursor"
"JS1_A09_00612" 11098 "s59E5H2" 0.0902744 "Sialic acid-binding periplasmic protein SiaP precursor"
"JS1_A09_00612" 11098 "s63E6H2" 0.04473071 "Sialic acid-binding periplasmic protein SiaP precursor"
"JS1_A09_00613" 11099 "s59E13H2" 0.3203187 "Transcriptional regulator KdgR"
"JS1_A09_00613" 11099 "s59E25H2" 0 "Transcriptional regulator KdgR"
"JS1_A09_00613" 11099 "s59E5H2" 0.06661399 "Transcriptional regulator KdgR"
"JS1_A09_00613" 11099 "s63E6H2" 0.5305779 "Transcriptional regulator KdgR"
"JS1_A09_00614" 11100 "s59E13H2" 0 "2-oxoglutarate synthase subunit KorB"
"JS1_A09_00614" 11100 "s59E25H2" 0 "2-oxoglutarate synthase subunit KorB"
"JS1_A09_00614" 11100 "s59E5H2" 0 "2-oxoglutarate synthase subunit KorB"
"JS1_A09_00614" 11100 "s63E6H2" 0.01976803 "2-oxoglutarate synthase subunit KorB"
"JS1_A09_00616" 11102 "s59E13H2" 0.1960797 "hypothetical protein"
"JS1_A09_00616" 11102 "s59E25H2" 0 "hypothetical protein"
"JS1_A09_00616" 11102 "s59E5H2" 0.446645 "hypothetical protein"
"JS1_A09_00616" 11102 "s63E6H2" 0.9830498 "hypothetical protein"
"JS1_A09_00617" 11103 "s59E13H2" 0.02964276 "Integrase core domain protein"

"JS1_A09_00617" 11103 "s59E25H2" 0 "Integrase core domain protein"
"JS1_A09_00617" 11103 "s59E5H2" 1.325177 "Integrase core domain protein"
"JS1_A09_00617" 11103 "s63E6H2" 0.7057244 "Integrase core domain protein"
"JS1_A09_00618" 11104 "s59E13H2" 0.002943158 "IS2 transposase TnpB"
"JS1_A09_00618" 11104 "s59E25H2" 0 "IS2 transposase TnpB"
"JS1_A09_00618" 11104 "s59E5H2" 0.08009632 "IS2 transposase TnpB"
"JS1_A09_00618" 11104 "s63E6H2" 0.03916839 "IS2 transposase TnpB"
"JS1_A09_00619" 11105 "s59E13H2" 0.01506071 "Transposase"
"JS1_A09_00619" 11105 "s59E25H2" 0 "Transposase"
"JS1_A09_00619" 11105 "s59E5H2" 0.369464 "Transposase"
"JS1_A09_00619" 11105 "s63E6H2" 0.2526226 "Transposase"
"JS1_A09_00620" 11106 "s59E13H2" 0 "hypothetical protein"
"JS1_A09_00620" 11106 "s59E25H2" 0 "hypothetical protein"
"JS1_A09_00620" 11106 "s59E5H2" 0.008916453 "hypothetical protein"
"JS1_A09_00620" 11106 "s63E6H2" 0.003730473 "hypothetical protein"
"JS1_A09_00622" 11108 "s59E13H2" 0 "hypothetical protein"
"JS1_A09_00622" 11108 "s59E25H2" 0 "hypothetical protein"
"JS1_A09_00622" 11108 "s59E5H2" 0 "hypothetical protein"
"JS1_A09_00622" 11108 "s63E6H2" 0.002318373 "hypothetical protein"
"JS1_A09_00631" 11117 "s59E13H2" 0.06194075 "hypothetical protein"
"JS1_A09_00631" 11117 "s59E25H2" 0 "hypothetical protein"
"JS1_A09_00631" 11117 "s59E5H2" 0.03302908 "hypothetical protein"
"JS1_A09_00631" 11117 "s63E6H2" 0.02835255 "hypothetical protein"
"JS1_A09_00632" 11118 "s59E13H2" 0.03795716 "hypothetical protein"
"JS1_A09_00632" 11118 "s59E25H2" 0 "hypothetical protein"
"JS1_A09_00632" 11118 "s59E5H2" 0 "hypothetical protein"
"JS1_A09_00632" 11118 "s63E6H2" 0.005219592 "hypothetical protein"
"JS1_A09_00634" 11120 "s59E13H2" 0 "Adenylate cyclase 2"
"JS1_A09_00634" 11120 "s59E25H2" 0 "Adenylate cyclase 2"
"JS1_A09_00634" 11120 "s59E5H2" 0.005092553 "Adenylate cyclase 2"
"JS1_A09_00634" 11120 "s63E6H2" 0.01608281 "Adenylate cyclase 2"
"JS1_A09_00636" 11122 "s59E13H2" 0.01866817 "Nucleotidyltransferase domain protein"
"JS1_A09_00636" 11122 "s59E25H2" 0 "Nucleotidyltransferase domain protein"
"JS1_A09_00636" 11122 "s59E5H2" 0 "Nucleotidyltransferase domain protein"
"JS1_A09_00636" 11122 "s63E6H2" 0.08210535 "Nucleotidyltransferase domain protein"
"JS1_A09_00637" 11123 "s59E13H2" 0.5996855 "hypothetical protein"
"JS1_A09_00637" 11123 "s59E25H2" 0 "hypothetical protein"
"JS1_A09_00637" 11123 "s59E5H2" 0.07249289 "hypothetical protein"
"JS1_A09_00637" 11123 "s63E6H2" 0.03748895 "hypothetical protein"
"JS1_A09_00638" 11124 "s59E13H2" 0.1448911 "Microbial collagenase precursor"
"JS1_A09_00638" 11124 "s59E25H2" 0 "Microbial collagenase precursor"
"JS1_A09_00638" 11124 "s59E5H2" 0 "Microbial collagenase precursor"
"JS1_A09_00638" 11124 "s63E6H2" 0.0009309613 "Microbial collagenase precursor"
"JS1_A09_00639" 11125 "s59E13H2" 0.01366167 "Type IV pilus biogenesis and competence protein PilQ precursor"
"JS1_A09_00639" 11125 "s59E25H2" 0 "Type IV pilus biogenesis and competence protein PilQ precursor"
"JS1_A09_00639" 11125 "s59E5H2" 0.08823405 "Type IV pilus biogenesis and competence protein PilQ precursor"
"JS1_A09_00639" 11125 "s63E6H2" 0.02583363 "Type IV pilus biogenesis and competence protein PilQ precursor"

"JS1_A09_00640" 11126 "s59E13H2" 0.02199958 "hypothetical protein"
"JS1_A09_00640" 11126 "s59E25H2" 0 "hypothetical protein"
"JS1_A09_00640" 11126 "s59E5H2" 0.04846247 "hypothetical protein"
"JS1_A09_00640" 11126 "s63E6H2" 0.06150538 "hypothetical protein"
"JS1_A09_00641" 11127 "s59E13H2" 0.07698776 "Pilus assembly protein, PilO"
"JS1_A09_00641" 11127 "s59E25H2" 0 "Pilus assembly protein, PilO"
"JS1_A09_00641" 11127 "s59E5H2" 0.04834309 "Pilus assembly protein, PilO"
"JS1_A09_00641" 11127 "s63E6H2" 0.06623687 "Pilus assembly protein, PilO"
"JS1_A09_00642" 11128 "s59E13H2" 0.1122268 "hypothetical protein"
"JS1_A09_00642" 11128 "s59E25H2" 0 "hypothetical protein"
"JS1_A09_00642" 11128 "s59E5H2" 0.1407029 "hypothetical protein"
"JS1_A09_00642" 11128 "s63E6H2" 0.05561903 "hypothetical protein"
"JS1_A09_00643" 11129 "s59E13H2" 0.08120025 "Competence protein A"
"JS1_A09_00643" 11129 "s59E25H2" 0 "Competence protein A"
"JS1_A09_00643" 11129 "s59E5H2" 0.3163169 "Competence protein A"
"JS1_A09_00643" 11129 "s63E6H2" 0.03909486 "Competence protein A"
"JS1_A09_00644" 11130 "s59E13H2" 0.001439818 "Helix-hairpin-helix motif protein"
"JS1_A09_00644" 11130 "s59E25H2" 0 "Helix-hairpin-helix motif protein"
"JS1_A09_00644" 11130 "s59E5H2" 0.06075082 "Helix-hairpin-helix motif protein"
"JS1_A09_00644" 11130 "s63E6H2" 0.05435273 "Helix-hairpin-helix motif protein"
"JS1_A09_00645" 11131 "s59E13H2" 0.02519877 "hypothetical protein"
"JS1_A09_00645" 11131 "s59E25H2" 0 "hypothetical protein"
"JS1_A09_00645" 11131 "s59E5H2" 0.6621782 "hypothetical protein"
"JS1_A09_00645" 11131 "s63E6H2" 0.09762059 "hypothetical protein"
"JS1_A09_00646" 11132 "s59E13H2" 0.2514054 "Peptidase M15"
"JS1_A09_00646" 11132 "s59E25H2" 0 "Peptidase M15"
"JS1_A09_00646" 11132 "s59E5H2" 0.01157373 "Peptidase M15"
"JS1_A09_00646" 11132 "s63E6H2" 1.282938 "Peptidase M15"
"JS1_A09_00647" 11133 "s59E13H2" 0.003168659 "YvrJ protein family protein"
"JS1_A09_00647" 11133 "s59E25H2" 0 "YvrJ protein family protein"
"JS1_A09_00647" 11133 "s59E5H2" 0.02924116 "YvrJ protein family protein"
"JS1_A09_00647" 11133 "s63E6H2" 0.8502674 "YvrJ protein family protein"
"JS1_A09_00648" 11134 "s59E13H2" 0.02626862 "putative xanthine dehydrogenase subunit D"
"JS1_A09_00648" 11134 "s59E25H2" 0 "putative xanthine dehydrogenase subunit D"
"JS1_A09_00648" 11134 "s59E5H2" 0.02356725 "putative xanthine dehydrogenase subunit D"
"JS1_A09_00648" 11134 "s63E6H2" 0.3734549 "putative xanthine dehydrogenase subunit D"
"JS1_A09_00649" 11135 "s59E13H2" 0.00361917 "Nicotinate dehydrogenase small FeS subunit"
"JS1_A09_00649" 11135 "s59E25H2" 0 "Nicotinate dehydrogenase small FeS subunit"
"JS1_A09_00649" 11135 "s59E5H2" 0.07710458 "Nicotinate dehydrogenase small FeS subunit"
"JS1_A09_00649" 11135 "s63E6H2" 0.1099956 "Nicotinate dehydrogenase small FeS subunit"
"JS1_A09_00650" 11136 "s59E13H2" 0.2737254 "Glyceraldehyde dehydrogenase medium chain"
"JS1_A09_00650" 11136 "s59E25H2" 0 "Glyceraldehyde dehydrogenase medium chain"
"JS1_A09_00650" 11136 "s59E5H2" 0.02258606 "Glyceraldehyde dehydrogenase medium chain"
"JS1_A09_00650" 11136 "s63E6H2" 0.03005395 "Glyceraldehyde dehydrogenase medium chain"
"JS1_A09_00651" 11137 "s59E13H2" 2.157026 "Glyceraldehyde dehydrogenase small chain"
"JS1_A09_00651" 11137 "s59E25H2" 0 "Glyceraldehyde dehydrogenase small chain"
"JS1_A09_00651" 11137 "s59E5H2" 0.1603388 "Glyceraldehyde dehydrogenase small chain"

"JS1_A09_00651" 11137 "s63E6H2" 0.06750063 "Glyceraldehyde dehydrogenase small chain"
"JS1_A09_00652" 11138 "s59E13H2" 1.860844 "Glyceraldehyde dehydrogenase medium chain"
"JS1_A09_00652" 11138 "s59E25H2" 0 "Glyceraldehyde dehydrogenase medium chain"
"JS1_A09_00652" 11138 "s59E5H2" 0.2328048 "Glyceraldehyde dehydrogenase medium chain"
"JS1_A09_00652" 11138 "s63E6H2" 0.04967823 "Glyceraldehyde dehydrogenase medium chain"
"JS1_A09_00653" 11139 "s59E13H2" 1.798943 "Xanthine dehydrogenase molybdenum-binding subunit"
"JS1_A09_00653" 11139 "s59E25H2" 0 "Xanthine dehydrogenase molybdenum-binding subunit"
"JS1_A09_00653" 11139 "s59E5H2" 0.1217622 "Xanthine dehydrogenase molybdenum-binding subunit"
"JS1_A09_00653" 11139 "s63E6H2" 0.0793305 "Xanthine dehydrogenase molybdenum-binding subunit"
"JS1_A09_00654" 11140 "s59E13H2" 0.01002994 "succinyl-CoA synthetase subunit beta"
"JS1_A09_00654" 11140 "s59E25H2" 0 "succinyl-CoA synthetase subunit beta"
"JS1_A09_00654" 11140 "s59E5H2" 0.07534528 "succinyl-CoA synthetase subunit beta"
"JS1_A09_00654" 11140 "s63E6H2" 0.07327285 "succinyl-CoA synthetase subunit beta"
"JS1_A09_00655" 11141 "s59E13H2" 0 "High-affinity branched-chain amino acid transport ATP-binding protein LivF"
"JS1_A09_00655" 11141 "s59E25H2" 0 "High-affinity branched-chain amino acid transport ATP-binding protein LivF"
"JS1_A09_00655" 11141 "s59E5H2" 0 "High-affinity branched-chain amino acid transport ATP-binding protein LivF"
"JS1_A09_00655" 11141 "s63E6H2" 0.05033583 "High-affinity branched-chain amino acid transport ATP-binding protein LivF"
"JS1_A09_00656" 11142 "s59E13H2" 0 "putative branched-chain amino acid transport ATP-binding protein LivG"
"JS1_A09_00656" 11142 "s59E25H2" 0 "putative branched-chain amino acid transport ATP-binding protein LivG"
"JS1_A09_00656" 11142 "s59E5H2" 0 "putative branched-chain amino acid transport ATP-binding protein LivG"
"JS1_A09_00656" 11142 "s63E6H2" 0.03111786 "putative branched-chain amino acid transport ATP-binding protein LivG"
"JS1_A09_00657" 11143 "s59E13H2" 0 "leucine/isoleucine/valine transporter permease subunit"
"JS1_A09_00657" 11143 "s59E25H2" 0 "leucine/isoleucine/valine transporter permease subunit"
"JS1_A09_00657" 11143 "s59E5H2" 0.001596977 "leucine/isoleucine/valine transporter permease subunit"
"JS1_A09_00657" 11143 "s63E6H2" 0.02085106 "leucine/isoleucine/valine transporter permease subunit"
"JS1_A09_00658" 11144 "s59E13H2" 0 "High-affinity branched-chain amino acid transport system permease protein LivH"
"JS1_A09_00658" 11144 "s59E25H2" 0 "High-affinity branched-chain amino acid transport system permease protein LivH"
"JS1_A09_00658" 11144 "s59E5H2" 0.01221509 "High-affinity branched-chain amino acid transport system permease protein LivH"
"JS1_A09_00658" 11144 "s63E6H2" 0.0207498 "High-affinity branched-chain amino acid transport system permease protein LivH"
"JS1_A09_00659" 11145 "s59E13H2" 0.004112632 "hypothetical protein"
"JS1_A09_00659" 11145 "s59E25H2" 0 "hypothetical protein"
"JS1_A09_00659" 11145 "s59E5H2" 0 "hypothetical protein"
"JS1_A09_00659" 11145 "s63E6H2" 0.01402096 "hypothetical protein"
"JS1_A09_00660" 11146 "s59E13H2" 0.0196997 "Succinyl-CoA ligase [ADP-forming] subunit alpha"
"JS1_A09_00660" 11146 "s59E25H2" 0 "Succinyl-CoA ligase [ADP-forming] subunit alpha"
"JS1_A09_00660" 11146 "s59E5H2" 0.2829585 "Succinyl-CoA ligase [ADP-forming] subunit alpha"
"JS1_A09_00660" 11146 "s63E6H2" 0.4858104 "Succinyl-CoA ligase [ADP-forming] subunit alpha"
"JS1_A09_00661" 11147 "s59E13H2" 0.0546156 "Indolepyruvate oxidoreductase subunit IorB"
"JS1_A09_00661" 11147 "s59E25H2" 0 "Indolepyruvate oxidoreductase subunit IorB"
"JS1_A09_00661" 11147 "s59E5H2" 0.3166526 "Indolepyruvate oxidoreductase subunit IorB"
"JS1_A09_00661" 11147 "s63E6H2" 0.4829417 "Indolepyruvate oxidoreductase subunit IorB"
"JS1_A09_00662" 11148 "s59E13H2" 0.03812122 "Indolepyruvate oxidoreductase subunit IorA"
"JS1_A09_00662" 11148 "s59E25H2" 0 "Indolepyruvate oxidoreductase subunit IorA"
"JS1_A09_00662" 11148 "s59E5H2" 0.2587691 "Indolepyruvate oxidoreductase subunit IorA"
"JS1_A09_00662" 11148 "s63E6H2" 0.274222 "Indolepyruvate oxidoreductase subunit IorA"
"JS1_A09_00663" 11149 "s59E13H2" 0 "hypothetical protein"
"JS1_A09_00663" 11149 "s59E25H2" 0 "hypothetical protein"

"JS1_A09_00663" 11149 "s59E5H2" 0 "hypothetical protein"
"JS1_A09_00663" 11149 "s63E6H2" 0.02184308 "hypothetical protein"
"JS1_A09_00664" 11150 "s59E13H2" 0 "Lysine-arginine-ornithine-binding periplasmic protein precursor"
"JS1_A09_00664" 11150 "s59E25H2" 0 "Lysine-arginine-ornithine-binding periplasmic protein precursor"
"JS1_A09_00664" 11150 "s59E5H2" 0 "Lysine-arginine-ornithine-binding periplasmic protein precursor"
"JS1_A09_00664" 11150 "s63E6H2" 0.06992366 "Lysine-arginine-ornithine-binding periplasmic protein precursor"
"JS1_A09_00665" 11151 "s59E13H2" 0 "Inner membrane amino-acid ABC transporter permease protein YecS"
"JS1_A09_00665" 11151 "s59E25H2" 0 "Inner membrane amino-acid ABC transporter permease protein YecS"
"JS1_A09_00665" 11151 "s59E5H2" 0 "Inner membrane amino-acid ABC transporter permease protein YecS"
"JS1_A09_00665" 11151 "s63E6H2" 0.03044229 "Inner membrane amino-acid ABC transporter permease protein YecS"
"JS1_A09_00666" 11152 "s59E13H2" 0 "Glutamine transport ATP-binding protein GlnQ"
"JS1_A09_00666" 11152 "s59E25H2" 0 "Glutamine transport ATP-binding protein GlnQ"
"JS1_A09_00666" 11152 "s59E5H2" 0 "Glutamine transport ATP-binding protein GlnQ"
"JS1_A09_00666" 11152 "s63E6H2" 0.01526106 "Glutamine transport ATP-binding protein GlnQ"
"JS1_A09_00667" 11153 "s59E13H2" 0 "Inner membrane amino-acid ABC transporter permease protein YecS"
"JS1_A09_00667" 11153 "s59E25H2" 0 "Inner membrane amino-acid ABC transporter permease protein YecS"
"JS1_A09_00667" 11153 "s59E5H2" 0 "Inner membrane amino-acid ABC transporter permease protein YecS"
"JS1_A09_00667" 11153 "s63E6H2" 0.02030854 "Inner membrane amino-acid ABC transporter permease protein YecS"
"JS1_A09_00668" 11154 "s59E13H2" 0.09379195 "D-aminopeptidase"
"JS1_A09_00668" 11154 "s59E25H2" 0 "D-aminopeptidase"
"JS1_A09_00668" 11154 "s59E5H2" 0.3265419 "D-aminopeptidase"
"JS1_A09_00668" 11154 "s63E6H2" 0.3738953 "D-aminopeptidase"
"JS1_A09_00669" 11155 "s59E13H2" 0.09934905 "hypothetical protein"
"JS1_A09_00669" 11155 "s59E25H2" 0 "hypothetical protein"
"JS1_A09_00669" 11155 "s59E5H2" 0.1486075 "hypothetical protein"
"JS1_A09_00669" 11155 "s63E6H2" 0.2988477 "hypothetical protein"
"JS1_A09_00670" 11156 "s59E13H2" 0.165841 "putative NH(3)-dependent NAD(+) synthetase"
"JS1_A09_00670" 11156 "s59E25H2" 0 "putative NH(3)-dependent NAD(+) synthetase"
"JS1_A09_00670" 11156 "s59E5H2" 0.2016377 "putative NH(3)-dependent NAD(+) synthetase"
"JS1_A09_00670" 11156 "s63E6H2" 0.4888634 "putative NH(3)-dependent NAD(+) synthetase"
"JS1_A09_00671" 11157 "s59E13H2" 0.08875336 "Acyl carrier protein"
"JS1_A09_00671" 11157 "s59E25H2" 0 "Acyl carrier protein"
"JS1_A09_00671" 11157 "s59E5H2" 0.2142831 "Acyl carrier protein"
"JS1_A09_00671" 11157 "s63E6H2" 0.09978141 "Acyl carrier protein"
"JS1_A09_00672" 11158 "s59E13H2" 0.1664929 "3-oxoacyl-[acyl-carrier-protein] synthase 2"
"JS1_A09_00672" 11158 "s59E25H2" 0 "3-oxoacyl-[acyl-carrier-protein] synthase 2"
"JS1_A09_00672" 11158 "s59E5H2" 0.1258004 "3-oxoacyl-[acyl-carrier-protein] synthase 2"
"JS1_A09_00672" 11158 "s63E6H2" 0.1190857 "3-oxoacyl-[acyl-carrier-protein] synthase 2"
"JS1_A09_00673" 11159 "s59E13H2" 0.04144253 "Ribonuclease 3"
"JS1_A09_00673" 11159 "s59E25H2" 0 "Ribonuclease 3"
"JS1_A09_00673" 11159 "s59E5H2" 0.1072416 "Ribonuclease 3"
"JS1_A09_00673" 11159 "s63E6H2" 0.1196526 "Ribonuclease 3"
"JS1_A09_00674" 11160 "s59E13H2" 0.01751368 "Chromosome partition protein Smc"
"JS1_A09_00674" 11160 "s59E25H2" 0 "Chromosome partition protein Smc"
"JS1_A09_00674" 11160 "s59E5H2" 0.05535603 "Chromosome partition protein Smc"
"JS1_A09_00674" 11160 "s63E6H2" 0.04601699 "Chromosome partition protein Smc"
"JS1_A09_00675" 11161 "s59E13H2" 0.01896362 "Signal recognition particle receptor FtsY"

"JS1_A09_00675" 11161 "s59E25H2" 0 "Signal recognition particle receptor FtsY"
"JS1_A09_00675" 11161 "s59E5H2" 0.03971082 "Signal recognition particle receptor FtsY"
"JS1_A09_00675" 11161 "s63E6H2" 0.05971037 "Signal recognition particle receptor FtsY"
"JS1_A09_00676" 11162 "s59E13H2" 0.0829992 "Signal recognition particle protein"
"JS1_A09_00676" 11162 "s59E25H2" 0 "Signal recognition particle protein"
"JS1_A09_00676" 11162 "s59E5H2" 0.08973433 "Signal recognition particle protein"
"JS1_A09_00676" 11162 "s63E6H2" 0.0222218 "Signal recognition particle protein"
"JS1_A09_00677" 11163 "s59E13H2" 0.01019595 "30S ribosomal protein S16"
"JS1_A09_00677" 11163 "s59E25H2" 0 "30S ribosomal protein S16"
"JS1_A09_00677" 11163 "s59E5H2" 0.02024839 "30S ribosomal protein S16"
"JS1_A09_00677" 11163 "s63E6H2" 0.01440474 "30S ribosomal protein S16"
"JS1_A09_00678" 11164 "s59E13H2" 0.02553395 "hypothetical protein"
"JS1_A09_00678" 11164 "s59E25H2" 0 "hypothetical protein"
"JS1_A09_00678" 11164 "s59E5H2" 0.03740629 "hypothetical protein"
"JS1_A09_00678" 11164 "s63E6H2" 0.02631727 "hypothetical protein"
"JS1_A09_00680" 11166 "s59E13H2" 0.4388373 "Ribonuclease"
"JS1_A09_00680" 11166 "s59E25H2" 0 "Ribonuclease"
"JS1_A09_00680" 11166 "s59E5H2" 0.5303456 "Ribonuclease"
"JS1_A09_00680" 11166 "s63E6H2" 0.8908387 "Ribonuclease"
"JS1_A09_00681" 11167 "s59E13H2" 0.01701636 "Thioredoxin-1"
"JS1_A09_00681" 11167 "s59E25H2" 0 "Thioredoxin-1"
"JS1_A09_00681" 11167 "s59E5H2" 0.2633924 "Thioredoxin-1"
"JS1_A09_00681" 11167 "s63E6H2" 0.4536489 "Thioredoxin-1"
"JS1_A09_00682" 11168 "s59E13H2" 0.01890746 "Multiple antibiotic resistance protein MarR"
"JS1_A09_00682" 11168 "s59E25H2" 0 "Multiple antibiotic resistance protein MarR"
"JS1_A09_00682" 11168 "s59E5H2" 0.2668086 "Multiple antibiotic resistance protein MarR"
"JS1_A09_00682" 11168 "s63E6H2" 0.6534665 "Multiple antibiotic resistance protein MarR"
"JS1_A09_00683" 11169 "s59E13H2" 0.001268822 "23S rRNA (uracil-C(5))-methyltransferase RlmCD"
"JS1_A09_00683" 11169 "s59E25H2" 0 "23S rRNA (uracil-C(5))-methyltransferase RlmCD"
"JS1_A09_00683" 11169 "s59E5H2" 0.0404165 "23S rRNA (uracil-C(5))-methyltransferase RlmCD"
"JS1_A09_00683" 11169 "s63E6H2" 0.03589676 "23S rRNA (uracil-C(5))-methyltransferase RlmCD"
"JS1_A09_00684" 11170 "s59E13H2" 0.01628026 "putative oxidoreductase"
"JS1_A09_00684" 11170 "s59E25H2" 0 "putative oxidoreductase"
"JS1_A09_00684" 11170 "s59E5H2" 0.2273913 "putative oxidoreductase"
"JS1_A09_00684" 11170 "s63E6H2" 0.4231042 "putative oxidoreductase"
"JS1_A09_00685" 11171 "s59E13H2" 0.004493483 "Poly(A) polymerase I precursor"
"JS1_A09_00685" 11171 "s59E25H2" 0 "Poly(A) polymerase I precursor"
"JS1_A09_00685" 11171 "s59E5H2" 0.01699257 "Poly(A) polymerase I precursor"
"JS1_A09_00685" 11171 "s63E6H2" 0.4755185 "Poly(A) polymerase I precursor"
"JS1_A09_00686" 11172 "s59E13H2" 0.06527556 "Transposase IS200 like protein"
"JS1_A09_00686" 11172 "s59E25H2" 0 "Transposase IS200 like protein"
"JS1_A09_00686" 11172 "s59E5H2" 0.4421382 "Transposase IS200 like protein"
"JS1_A09_00686" 11172 "s63E6H2" 0.07707519 "Transposase IS200 like protein"
"JS1_A09_00689" 11175 "s59E13H2" 0.004744622 "Group II intron-encoded protein LtrA"
"JS1_A09_00689" 11175 "s59E25H2" 0 "Group II intron-encoded protein LtrA"
"JS1_A09_00689" 11175 "s59E5H2" 0.1898333 "Group II intron-encoded protein LtrA"
"JS1_A09_00689" 11175 "s63E6H2" 0.001953311 "Group II intron-encoded protein LtrA"

"JS1_A09_00691" 11177 "s59E13H2" 0.06402712 "hypothetical protein"
"JS1_A09_00691" 11177 "s59E25H2" 0 "hypothetical protein"
"JS1_A09_00691" 11177 "s59E5H2" 0.6034367 "hypothetical protein"
"JS1_A09_00691" 11177 "s63E6H2" 0.2940228 "hypothetical protein"
"JS1_A09_00692" 11178 "s59E13H2" 0.005952049 "hypothetical protein"
"JS1_A09_00692" 11178 "s59E25H2" 0 "hypothetical protein"
"JS1_A09_00692" 11178 "s59E5H2" 0.04589291 "hypothetical protein"
"JS1_A09_00692" 11178 "s63E6H2" 0.01100926 "hypothetical protein"
"JS1_A09_00693" 11179 "s59E13H2" 0.04822403 "ADP-ribosyl-[dinitrogen reductase] glycohydrolase"
"JS1_A09_00693" 11179 "s59E25H2" 0 "ADP-ribosyl-[dinitrogen reductase] glycohydrolase"
"JS1_A09_00693" 11179 "s59E5H2" 0 "ADP-ribosyl-[dinitrogen reductase] glycohydrolase"
"JS1_A09_00693" 11179 "s63E6H2" 0.00153984 "ADP-ribosyl-[dinitrogen reductase] glycohydrolase"
"JS1_A09_00695" 11181 "s59E13H2" 0.006631497 "hypothetical protein"
"JS1_A09_00695" 11181 "s59E25H2" 0 "hypothetical protein"
"JS1_A09_00695" 11181 "s59E5H2" 0 "hypothetical protein"
"JS1_A09_00695" 11181 "s63E6H2" 0 "hypothetical protein"
"JS1_A09_00696" 11182 "s59E13H2" 0.01637357 "hypothetical protein"
"JS1_A09_00696" 11182 "s59E25H2" 0 "hypothetical protein"
"JS1_A09_00696" 11182 "s59E5H2" 0 "hypothetical protein"
"JS1_A09_00696" 11182 "s63E6H2" 0.00907456 "hypothetical protein"
"JS1_A09_00697" 11183 "s59E13H2" 0 "DNA helicase II"
"JS1_A09_00697" 11183 "s59E25H2" 0 "DNA helicase II"
"JS1_A09_00697" 11183 "s59E5H2" 0 "DNA helicase II"
"JS1_A09_00697" 11183 "s63E6H2" 0 "DNA helicase II"
"JS1_A09_00698" 11184 "s59E13H2" 0 "hypothetical protein"
"JS1_A09_00698" 11184 "s59E25H2" 0 "hypothetical protein"
"JS1_A09_00698" 11184 "s59E5H2" 0 "hypothetical protein"
"JS1_A09_00698" 11184 "s63E6H2" 0 "hypothetical protein"
"JS1_A09_00699" 11185 "s59E13H2" 0.188986 "DNA integrity scanning protein DisA"
"JS1_A09_00699" 11185 "s59E25H2" 0.5950617 "DNA integrity scanning protein DisA"
"JS1_A09_00699" 11185 "s59E5H2" 0.1168245 "DNA integrity scanning protein DisA"
"JS1_A09_00699" 11185 "s63E6H2" 2.893382 "DNA integrity scanning protein DisA"
"JS1_A09_00700" 11186 "s59E13H2" 0.178085 "YbbR-like protein"
"JS1_A09_00700" 11186 "s59E25H2" 0 "YbbR-like protein"
"JS1_A09_00700" 11186 "s59E5H2" 0.1235008 "YbbR-like protein"
"JS1_A09_00700" 11186 "s63E6H2" 1.034773 "YbbR-like protein"
"JS1_A09_00701" 11187 "s59E13H2" 0.1964555 "Phosphoglucosamine mutase"
"JS1_A09_00701" 11187 "s59E25H2" 0.6867112 "Phosphoglucosamine mutase"
"JS1_A09_00701" 11187 "s59E5H2" 0.1219551 "Phosphoglucosamine mutase"
"JS1_A09_00701" 11187 "s63E6H2" 0.1924612 "Phosphoglucosamine mutase"
"JS1_A09_00703" 11189 "s59E13H2" 0.2100893 "Glutamine--fructose-6-phosphate aminotransferase [isomerizing]"
"JS1_A09_00703" 11189 "s59E25H2" 0.2540984 "Glutamine--fructose-6-phosphate aminotransferase [isomerizing]"
"JS1_A09_00703" 11189 "s59E5H2" 0.2032756 "Glutamine--fructose-6-phosphate aminotransferase [isomerizing]"
"JS1_A09_00703" 11189 "s63E6H2" 0.2128017 "Glutamine--fructose-6-phosphate aminotransferase [isomerizing]"
"JS1_A09_00704" 11190 "s59E13H2" 0.0691948 "Glutamine synthetase"
"JS1_A09_00704" 11190 "s59E25H2" 0.5018811 "Glutamine synthetase"
"JS1_A09_00704" 11190 "s59E5H2" 0.1699243 "Glutamine synthetase"

"JS1_A09_00704" 11190 "s63E6H2" 0.1837647 "Glutamine synthetase"
"JS1_A09_00705" 11191 "s59E13H2" 0.9392277 "L-allo-threonine aldolase"
"JS1_A09_00705" 11191 "s59E25H2" 0 "L-allo-threonine aldolase"
"JS1_A09_00705" 11191 "s59E5H2" 0.3384619 "L-allo-threonine aldolase"
"JS1_A09_00705" 11191 "s63E6H2" 0.200193 "L-allo-threonine aldolase"
"JS1_A09_00706" 11192 "s59E13H2" 2.508586 "Thioesterase superfamily protein"
"JS1_A09_00706" 11192 "s59E25H2" 1.368056 "Thioesterase superfamily protein"
"JS1_A09_00706" 11192 "s59E5H2" 0.4416947 "Thioesterase superfamily protein"
"JS1_A09_00706" 11192 "s63E6H2" 0.09408826 "Thioesterase superfamily protein"
"JS1_A09_00707" 11193 "s59E13H2" 3.668794 "phage T7 F exclusion suppressor FxsA"
"JS1_A09_00707" 11193 "s59E25H2" 0 "phage T7 F exclusion suppressor FxsA"
"JS1_A09_00707" 11193 "s59E5H2" 0.2027285 "phage T7 F exclusion suppressor FxsA"
"JS1_A09_00707" 11193 "s63E6H2" 0.1440607 "phage T7 F exclusion suppressor FxsA"
"JS1_A09_00708" 11194 "s59E13H2" 5.706992 "Holo-[acyl-carrier-protein] synthase"
"JS1_A09_00708" 11194 "s59E25H2" 0 "Holo-[acyl-carrier-protein] synthase"
"JS1_A09_00708" 11194 "s59E5H2" 0.4599975 "Holo-[acyl-carrier-protein] synthase"
"JS1_A09_00708" 11194 "s63E6H2" 0.3258655 "Holo-[acyl-carrier-protein] synthase"
"JS1_A09_00712" 11198 "s59E13H2" 0 "CoB--CoM heterodisulfide reductase subunit B"
"JS1_A09_00712" 11198 "s59E25H2" 0 "CoB--CoM heterodisulfide reductase subunit B"
"JS1_A09_00712" 11198 "s59E5H2" 0 "CoB--CoM heterodisulfide reductase subunit B"
"JS1_A09_00712" 11198 "s63E6H2" 0 "CoB--CoM heterodisulfide reductase subunit B"
"JS1_A09_00713" 11199 "s59E13H2" 0.4971022 "Xylulose kinase"
"JS1_A09_00713" 11199 "s59E25H2" 0 "Xylulose kinase"
"JS1_A09_00713" 11199 "s59E5H2" 0.1877244 "Xylulose kinase"
"JS1_A09_00713" 11199 "s63E6H2" 0.1124287 "Xylulose kinase"
"JS1_A09_00714" 11200 "s59E13H2" 0.4876484 "Sorbitol dehydrogenase"
"JS1_A09_00714" 11200 "s59E25H2" 0 "Sorbitol dehydrogenase"
"JS1_A09_00714" 11200 "s59E5H2" 0.1041206 "Sorbitol dehydrogenase"
"JS1_A09_00714" 11200 "s63E6H2" 0.1350794 "Sorbitol dehydrogenase"
"JS1_A09_00715" 11201 "s59E13H2" 0.8457873 "Glyoxylate reductase"
"JS1_A09_00715" 11201 "s59E25H2" 0 "Glyoxylate reductase"
"JS1_A09_00715" 11201 "s59E5H2" 0.1659796 "Glyoxylate reductase"
"JS1_A09_00715" 11201 "s63E6H2" 0.3195916 "Glyoxylate reductase"
"JS1_A09_00716" 11202 "s59E13H2" 0.09898343 "Vitamin B12 dependent methionine synthase, activation domain"
"JS1_A09_00716" 11202 "s59E25H2" 0 "Vitamin B12 dependent methionine synthase, activation domain"
"JS1_A09_00716" 11202 "s59E5H2" 0.02270104 "Vitamin B12 dependent methionine synthase, activation domain"
"JS1_A09_00716" 11202 "s63E6H2" 0.0712368 "Vitamin B12 dependent methionine synthase, activation domain"
"JS1_A09_00717" 11203 "s59E13H2" 0.5522252 "Chlorosome protein I"
"JS1_A09_00717" 11203 "s59E25H2" 0 "Chlorosome protein I"
"JS1_A09_00717" 11203 "s59E5H2" 0.1131714 "Chlorosome protein I"
"JS1_A09_00717" 11203 "s63E6H2" 0.08105072 "Chlorosome protein I"
"JS1_A09_00718" 11204 "s59E13H2" 0.3681426 "Formate--tetrahydrofolate ligase"
"JS1_A09_00718" 11204 "s59E25H2" 0 "Formate--tetrahydrofolate ligase"
"JS1_A09_00718" 11204 "s59E5H2" 0.1132816 "Formate--tetrahydrofolate ligase"
"JS1_A09_00718" 11204 "s63E6H2" 0.8796352 "Formate--tetrahydrofolate ligase"
"JS1_A09_00719" 11205 "s59E13H2" 2.340467 "5-methyltetrahydrofolate:corrinoid/iron-sulfur protein co-methyltransferase"
"JS1_A09_00719" 11205 "s59E25H2" 0 "5-methyltetrahydrofolate:corrinoid/iron-sulfur protein co-methyltransferase"

"JS1_A09_00719" 11205 "s59E5H2" 0.1594253 "5-methyltetrahydrofolate:corrinoid/iron-sulfur protein co-methyltransferase"
"JS1_A09_00719" 11205 "s63E6H2" 0.3414946 "5-methyltetrahydrofolate:corrinoid/iron-sulfur protein co-methyltransferase"
"JS1_A09_00720" 11206 "s59E13H2" 2.932287 "Acryloyl-CoA reductase electron transfer subunit beta"
"JS1_A09_00720" 11206 "s59E25H2" 0 "Acryloyl-CoA reductase electron transfer subunit beta"
"JS1_A09_00720" 11206 "s59E5H2" 0.1412328 "Acryloyl-CoA reductase electron transfer subunit beta"
"JS1_A09_00720" 11206 "s63E6H2" 0.9140212 "Acryloyl-CoA reductase electron transfer subunit beta"
"JS1_A09_00721" 11207 "s59E13H2" 2.873048 "Acryloyl-CoA reductase electron transfer subunit gamma"
"JS1_A09_00721" 11207 "s59E25H2" 0 "Acryloyl-CoA reductase electron transfer subunit gamma"
"JS1_A09_00721" 11207 "s59E5H2" 0.2288546 "Acryloyl-CoA reductase electron transfer subunit gamma"
"JS1_A09_00721" 11207 "s63E6H2" 1.082923 "Acryloyl-CoA reductase electron transfer subunit gamma"
"JS1_A09_00722" 11208 "s59E13H2" 3.189226 "bifunctional homocysteine S-methyltransferase/5,10-methylenetetrahydrofolate reductase protein"
"JS1_A09_00722" 11208 "s59E25H2" 0 "bifunctional homocysteine S-methyltransferase/5,10-methylenetetrahydrofolate reductase protein"
"JS1_A09_00722" 11208 "s59E5H2" 0.2719833 "bifunctional homocysteine S-methyltransferase/5,10-methylenetetrahydrofolate reductase protein"
"JS1_A09_00722" 11208 "s63E6H2" 0.834493 "bifunctional homocysteine S-methyltransferase/5,10-methylenetetrahydrofolate reductase protein"
"JS1_A09_00723" 11209 "s59E13H2" 2.582701 "Trimethylamine corrinoid protein"
"JS1_A09_00723" 11209 "s59E25H2" 0 "Trimethylamine corrinoid protein"
"JS1_A09_00723" 11209 "s59E5H2" 0.3557873 "Trimethylamine corrinoid protein"
"JS1_A09_00723" 11209 "s63E6H2" 0.42871 "Trimethylamine corrinoid protein"
"JS1_A09_00724" 11210 "s59E13H2" 1.13166 "HTH-type transcriptional activator Btr"
"JS1_A09_00724" 11210 "s59E25H2" 0.2209821 "HTH-type transcriptional activator Btr"
"JS1_A09_00724" 11210 "s59E5H2" 0.03271296 "HTH-type transcriptional activator Btr"
"JS1_A09_00724" 11210 "s63E6H2" 0.1292844 "HTH-type transcriptional activator Btr"
"JS1_A09_00725" 11211 "s59E13H2" 2.501804 "DNA polymerase/3'-5' exonuclease PolX"
"JS1_A09_00725" 11211 "s59E25H2" 0.09656777 "DNA polymerase/3'-5' exonuclease PolX"
"JS1_A09_00725" 11211 "s59E5H2" 0.3569222 "DNA polymerase/3'-5' exonuclease PolX"
"JS1_A09_00725" 11211 "s63E6H2" 0.1067324 "DNA polymerase/3'-5' exonuclease PolX"
"JS1_A09_00727" 11213 "s59E13H2" 2.288024e-05 "NADH dehydrogenase"
"JS1_A09_00727" 11213 "s59E25H2" 0 "NADH dehydrogenase"
"JS1_A09_00727" 11213 "s59E5H2" 1.777602e-05 "NADH dehydrogenase"
"JS1_A09_00727" 11213 "s63E6H2" 0.154402 "NADH dehydrogenase"
"JS1_A09_00728" 11214 "s59E13H2" 0 "Glutaredoxin-3"
"JS1_A09_00728" 11214 "s59E25H2" 0 "Glutaredoxin-3"
"JS1_A09_00728" 11214 "s59E5H2" 0 "Glutaredoxin-3"
"JS1_A09_00728" 11214 "s63E6H2" 0.1471288 "Glutaredoxin-3"
"JS1_A09_00729" 11215 "s59E13H2" 0.1564484 "Carnitine transport permease protein OpuCB"
"JS1_A09_00729" 11215 "s59E25H2" 0 "Carnitine transport permease protein OpuCB"
"JS1_A09_00729" 11215 "s59E5H2" 0.3352326 "Carnitine transport permease protein OpuCB"
"JS1_A09_00729" 11215 "s63E6H2" 0.1982439 "Carnitine transport permease protein OpuCB"
"JS1_A09_00730" 11216 "s59E13H2" 0.4575197 "Glycine betaine/carnitine/choline transport system permease protein OpuCB"
"JS1_A09_00730" 11216 "s59E25H2" 0 "Glycine betaine/carnitine/choline transport system permease protein OpuCB"
"JS1_A09_00730" 11216 "s59E5H2" 0.7312167 "Glycine betaine/carnitine/choline transport system permease protein OpuCB"
"JS1_A09_00730" 11216 "s63E6H2" 0.4626593 "Glycine betaine/carnitine/choline transport system permease protein OpuCB"
"JS1_A09_00731" 11217 "s59E13H2" 0.4029222 "Choline transport ATP-binding protein OpuBA"
"JS1_A09_00731" 11217 "s59E25H2" 0 "Choline transport ATP-binding protein OpuBA"
"JS1_A09_00731" 11217 "s59E5H2" 2.496101 "Choline transport ATP-binding protein OpuBA"
"JS1_A09_00731" 11217 "s63E6H2" 0.5311305 "Choline transport ATP-binding protein OpuBA"
"JS1_A09_00732" 11218 "s59E13H2" 0.3128328 "Glycine betaine/carnitine/choline-binding protein OpuCC precursor"

"JS1_A09_00732" 11218 "s59E25H2" 0 "Glycine betaine/carnitine/choline-binding protein OpuCC precursor"
"JS1_A09_00732" 11218 "s59E5H2" 4.170406 "Glycine betaine/carnitine/choline-binding protein OpuCC precursor"
"JS1_A09_00732" 11218 "s63E6H2" 0.4551214 "Glycine betaine/carnitine/choline-binding protein OpuCC precursor"
"JS1_A09_00733" 11219 "s59E13H2" 0.2067396 "Uracil DNA glycosylase superfamily protein"
"JS1_A09_00733" 11219 "s59E25H2" 0.2142857 "Uracil DNA glycosylase superfamily protein"
"JS1_A09_00733" 11219 "s59E5H2" 0.3556931 "Uracil DNA glycosylase superfamily protein"
"JS1_A09_00733" 11219 "s63E6H2" 0.2090014 "Uracil DNA glycosylase superfamily protein"
"JS1_A09_00734" 11220 "s59E13H2" 0.1225146 "putative HTH-type transcriptional regulator YdfH"
"JS1_A09_00734" 11220 "s59E25H2" 0.1463415 "putative HTH-type transcriptional regulator YdfH"
"JS1_A09_00734" 11220 "s59E5H2" 0.01548682 "putative HTH-type transcriptional regulator YdfH"
"JS1_A09_00734" 11220 "s63E6H2" 0.2495804 "putative HTH-type transcriptional regulator YdfH"
"JS1_A09_00735" 11221 "s59E13H2" 0.005548117 "hypothetical protein"
"JS1_A09_00735" 11221 "s59E25H2" 0 "hypothetical protein"
"JS1_A09_00735" 11221 "s59E5H2" 0.03505671 "hypothetical protein"
"JS1_A09_00735" 11221 "s63E6H2" 0.1760101 "hypothetical protein"
"JS1_A09_00736" 11222 "s59E13H2" 0.005509438 "Opine dehydrogenase"
"JS1_A09_00736" 11222 "s59E25H2" 0 "Opine dehydrogenase"
"JS1_A09_00736" 11222 "s59E5H2" 0 "Opine dehydrogenase"
"JS1_A09_00736" 11222 "s63E6H2" 0.1060498 "Opine dehydrogenase"
"JS1_A09_00737" 11223 "s59E13H2" 0 "Gamma-glutamyl-gamma-aminobutyrate hydrolase PuuD"
"JS1_A09_00737" 11223 "s59E25H2" 0 "Gamma-glutamyl-gamma-aminobutyrate hydrolase PuuD"
"JS1_A09_00737" 11223 "s59E5H2" 0 "Gamma-glutamyl-gamma-aminobutyrate hydrolase PuuD"
"JS1_A09_00737" 11223 "s63E6H2" 0.04072159 "Gamma-glutamyl-gamma-aminobutyrate hydrolase PuuD"
"JS1_A09_00738" 11224 "s59E13H2" 0 "Oligopeptide transport ATP-binding protein OppF"
"JS1_A09_00738" 11224 "s59E25H2" 0 "Oligopeptide transport ATP-binding protein OppF"
"JS1_A09_00738" 11224 "s59E5H2" 0.01195556 "Oligopeptide transport ATP-binding protein OppF"
"JS1_A09_00738" 11224 "s63E6H2" 0.1226438 "Oligopeptide transport ATP-binding protein OppF"
"JS1_A09_00739" 11225 "s59E13H2" 0 "Oligopeptide transport ATP-binding protein OppD"
"JS1_A09_00739" 11225 "s59E25H2" 0 "Oligopeptide transport ATP-binding protein OppD"
"JS1_A09_00739" 11225 "s59E5H2" 0 "Oligopeptide transport ATP-binding protein OppD"
"JS1_A09_00739" 11225 "s63E6H2" 0.02107613 "Oligopeptide transport ATP-binding protein OppD"
"JS1_A09_00740" 11226 "s59E13H2" 0 "Glutathione transport system permease protein GsiD"
"JS1_A09_00740" 11226 "s59E25H2" 0 "Glutathione transport system permease protein GsiD"
"JS1_A09_00740" 11226 "s59E5H2" 0 "Glutathione transport system permease protein GsiD"
"JS1_A09_00740" 11226 "s63E6H2" 0.03003692 "Glutathione transport system permease protein GsiD"
"JS1_A09_00741" 11227 "s59E13H2" 0 "Dipeptide transport system permease protein DppB"
"JS1_A09_00741" 11227 "s59E25H2" 0 "Dipeptide transport system permease protein DppB"
"JS1_A09_00741" 11227 "s59E5H2" 0 "Dipeptide transport system permease protein DppB"
"JS1_A09_00741" 11227 "s63E6H2" 0.0272636 "Dipeptide transport system permease protein DppB"
"JS1_A09_00742" 11228 "s59E13H2" 0 "Periplasmic dipeptide transport protein precursor"
"JS1_A09_00742" 11228 "s59E25H2" 0 "Periplasmic dipeptide transport protein precursor"
"JS1_A09_00742" 11228 "s59E5H2" 0.002760347 "Periplasmic dipeptide transport protein precursor"
"JS1_A09_00742" 11228 "s63E6H2" 0.02220314 "Periplasmic dipeptide transport protein precursor"
"JS1_A09_00743" 11229 "s59E13H2" 0.05728363 "N-acyl-D-glutamate deacylase"
"JS1_A09_00743" 11229 "s59E25H2" 0 "N-acyl-D-glutamate deacylase"
"JS1_A09_00743" 11229 "s59E5H2" 0.2173796 "N-acyl-D-glutamate deacylase"
"JS1_A09_00743" 11229 "s63E6H2" 0.1159915 "N-acyl-D-glutamate deacylase"

"JS1_A09_00744" 11230 "s59E13H2" 0.02045099 "Oligopeptide transport ATP-binding protein OppF"
"JS1_A09_00744" 11230 "s59E25H2" 0 "Oligopeptide transport ATP-binding protein OppF"
"JS1_A09_00744" 11230 "s59E5H2" 0.4260056 "Oligopeptide transport ATP-binding protein OppF"
"JS1_A09_00744" 11230 "s63E6H2" 0.1239756 "Oligopeptide transport ATP-binding protein OppF"
"JS1_A09_00745" 11231 "s59E13H2" 0.01538405 "Oligopeptide transport ATP-binding protein OppD"
"JS1_A09_00745" 11231 "s59E25H2" 0 "Oligopeptide transport ATP-binding protein OppD"
"JS1_A09_00745" 11231 "s59E5H2" 1.636925 "Oligopeptide transport ATP-binding protein OppD"
"JS1_A09_00745" 11231 "s63E6H2" 0.2476926 "Oligopeptide transport ATP-binding protein OppD"
"JS1_A09_00746" 11232 "s59E13H2" 0.007569087 "Glutathione transport system permease protein GsiD"
"JS1_A09_00746" 11232 "s59E25H2" 0 "Glutathione transport system permease protein GsiD"
"JS1_A09_00746" 11232 "s59E5H2" 1.784126 "Glutathione transport system permease protein GsiD"
"JS1_A09_00746" 11232 "s63E6H2" 2.652464 "Glutathione transport system permease protein GsiD"
"JS1_A09_00747" 11233 "s59E13H2" 0.01753625 "Glutathione transport system permease protein GsiC"
"JS1_A09_00747" 11233 "s59E25H2" 0 "Glutathione transport system permease protein GsiC"
"JS1_A09_00747" 11233 "s59E5H2" 0.9548552 "Glutathione transport system permease protein GsiC"
"JS1_A09_00747" 11233 "s63E6H2" 2.813351 "Glutathione transport system permease protein GsiC"
"JS1_A09_00748" 11234 "s59E13H2" 0.002656095 "Glutathione-binding protein GsiB precursor"
"JS1_A09_00748" 11234 "s59E25H2" 0 "Glutathione-binding protein GsiB precursor"
"JS1_A09_00748" 11234 "s59E5H2" 1.221035 "Glutathione-binding protein GsiB precursor"
"JS1_A09_00748" 11234 "s63E6H2" 2.784874 "Glutathione-binding protein GsiB precursor"
"JS1_A09_00749" 11235 "s59E13H2" 0.4589686 "putative protease YhbU precursor"
"JS1_A09_00749" 11235 "s59E25H2" 0 "putative protease YhbU precursor"
"JS1_A09_00749" 11235 "s59E5H2" 0.6744602 "putative protease YhbU precursor"
"JS1_A09_00749" 11235 "s63E6H2" 1.405991 "putative protease YhbU precursor"
"JS1_A09_00750" 11236 "s59E13H2" 0.4107367 "Putative GTP cyclohydrolase 1 type 2"
"JS1_A09_00750" 11236 "s59E25H2" 0.1776557 "Putative GTP cyclohydrolase 1 type 2"
"JS1_A09_00750" 11236 "s59E5H2" 0.3410848 "Putative GTP cyclohydrolase 1 type 2"
"JS1_A09_00750" 11236 "s63E6H2" 0.1663844 "Putative GTP cyclohydrolase 1 type 2"
"JS1_A09_00751" 11237 "s59E13H2" 0.3899548 "hypothetical protein"
"JS1_A09_00751" 11237 "s59E25H2" 0 "hypothetical protein"
"JS1_A09_00751" 11237 "s59E5H2" 0.3371869 "hypothetical protein"
"JS1_A09_00751" 11237 "s63E6H2" 0.7504224 "hypothetical protein"
"JS1_A09_00752" 11238 "s59E13H2" 0 "4-hydroxy-tetrahydrodipicolinate synthase"
"JS1_A09_00752" 11238 "s59E25H2" 0 "4-hydroxy-tetrahydrodipicolinate synthase"
"JS1_A09_00752" 11238 "s59E5H2" 0.04660873 "4-hydroxy-tetrahydrodipicolinate synthase"
"JS1_A09_00752" 11238 "s63E6H2" 3.128233 "4-hydroxy-tetrahydrodipicolinate synthase"
"JS1_A09_00753" 11239 "s59E13H2" 0.002085847 "Aldo/keto reductase family protein"
"JS1_A09_00753" 11239 "s59E25H2" 0 "Aldo/keto reductase family protein"
"JS1_A09_00753" 11239 "s59E5H2" 0.05004912 "Aldo/keto reductase family protein"
"JS1_A09_00753" 11239 "s63E6H2" 0.03710763 "Aldo/keto reductase family protein"
"JS1_A09_00754" 11240 "s59E13H2" 0 "D-allulose-6-phosphate 3-epimerase"
"JS1_A09_00754" 11240 "s59E25H2" 0 "D-allulose-6-phosphate 3-epimerase"
"JS1_A09_00754" 11240 "s59E5H2" 0.005258514 "D-allulose-6-phosphate 3-epimerase"
"JS1_A09_00754" 11240 "s63E6H2" 0.008587337 "D-allulose-6-phosphate 3-epimerase"
"JS1_A09_00755" 11241 "s59E13H2" 0 "4-oxalmesaconate hydratase"
"JS1_A09_00755" 11241 "s59E25H2" 0 "4-oxalmesaconate hydratase"
"JS1_A09_00755" 11241 "s59E5H2" 0 "4-oxalmesaconate hydratase"

"JS1_A09_00755" 11241 "s63E6H2" 0.005691085 "4-oxalmesaconate hydratase"
"JS1_A09_00757" 11243 "s59E13H2" 0 "EamA-like transporter family protein"
"JS1_A09_00757" 11243 "s59E25H2" 0 "EamA-like transporter family protein"
"JS1_A09_00757" 11243 "s59E5H2" 0 "EamA-like transporter family protein"
"JS1_A09_00757" 11243 "s63E6H2" 0 "EamA-like transporter family protein"
"JS1_A09_00758" 11244 "s59E13H2" 0.006326479 "L-threonine dehydratase catabolic TdcB"
"JS1_A09_00758" 11244 "s59E25H2" 0 "L-threonine dehydratase catabolic TdcB"
"JS1_A09_00758" 11244 "s59E5H2" 0 "L-threonine dehydratase catabolic TdcB"
"JS1_A09_00758" 11244 "s63E6H2" 0.001686632 "L-threonine dehydratase catabolic TdcB"
"JS1_A09_00760" 11246 "s59E13H2" 0.003901918 "Integrase core domain protein"
"JS1_A09_00760" 11246 "s59E25H2" 0 "Integrase core domain protein"
"JS1_A09_00760" 11246 "s59E5H2" 0.9697252 "Integrase core domain protein"
"JS1_A09_00760" 11246 "s63E6H2" 0.8781064 "Integrase core domain protein"
"JS1_A09_00761" 11247 "s59E13H2" 0.03129457 "Transposase"
"JS1_A09_00761" 11247 "s59E25H2" 0 "Transposase"
"JS1_A09_00761" 11247 "s59E5H2" 0.1110543 "Transposase"
"JS1_A09_00761" 11247 "s63E6H2" 0.364777 "Transposase"
"JS1_A09_00762" 11248 "s59E13H2" 0 "putative glycolipid permease LtaA"
"JS1_A09_00762" 11248 "s59E25H2" 0 "putative glycolipid permease LtaA"
"JS1_A09_00762" 11248 "s59E5H2" 0.002213816 "putative glycolipid permease LtaA"
"JS1_A09_00762" 11248 "s63E6H2" 0.002939097 "putative glycolipid permease LtaA"
"JS1_A09_00763" 11249 "s59E13H2" 0.04714346 "Transposase"
"JS1_A09_00763" 11249 "s59E25H2" 0 "Transposase"
"JS1_A09_00763" 11249 "s59E5H2" 0 "Transposase"
"JS1_A09_00763" 11249 "s63E6H2" 0.006698476 "Transposase"
"JS1_A09_00764" 11250 "s59E13H2" 0.6440786 "branched-chain amino acid transporter permease subunit LivH"
"JS1_A09_00764" 11250 "s59E25H2" 0 "branched-chain amino acid transporter permease subunit LivH"
"JS1_A09_00764" 11250 "s59E5H2" 0.03293428 "branched-chain amino acid transporter permease subunit LivH"
"JS1_A09_00764" 11250 "s63E6H2" 0.1660866 "branched-chain amino acid transporter permease subunit LivH"
"JS1_A09_00765" 11251 "s59E13H2" 0.03857945 "Aspartate carbamoyltransferase"
"JS1_A09_00765" 11251 "s59E25H2" 0 "Aspartate carbamoyltransferase"
"JS1_A09_00765" 11251 "s59E5H2" 0.0306514 "Aspartate carbamoyltransferase"
"JS1_A09_00765" 11251 "s63E6H2" 0.0237645 "Aspartate carbamoyltransferase"
"JS1_A09_00767" 11253 "s59E13H2" 0.1436556 "Carbamate kinase"
"JS1_A09_00767" 11253 "s59E25H2" 0 "Carbamate kinase"
"JS1_A09_00767" 11253 "s59E5H2" 0.6628742 "Carbamate kinase"
"JS1_A09_00767" 11253 "s63E6H2" 0.04227271 "Carbamate kinase"
"JS1_A09_00768" 11254 "s59E13H2" 0 "HTH-type transcriptional regulator KipR"
"JS1_A09_00768" 11254 "s59E25H2" 0 "HTH-type transcriptional regulator KipR"
"JS1_A09_00768" 11254 "s59E5H2" 0 "HTH-type transcriptional regulator KipR"
"JS1_A09_00768" 11254 "s63E6H2" 0.005145232 "HTH-type transcriptional regulator KipR"
"JS1_A09_00769" 11255 "s59E13H2" 0.09376139 "Trehalose/maltose import ATP-binding protein MalK"
"JS1_A09_00769" 11255 "s59E25H2" 0 "Trehalose/maltose import ATP-binding protein MalK"
"JS1_A09_00769" 11255 "s59E5H2" 0.2567938 "Trehalose/maltose import ATP-binding protein MalK"
"JS1_A09_00769" 11255 "s63E6H2" 0.1157728 "Trehalose/maltose import ATP-binding protein MalK"
"JS1_A09_00770" 11256 "s59E13H2" 0 "methylcobalamin:coenzyme M methyltransferase"
"JS1_A09_00770" 11256 "s59E25H2" 0 "methylcobalamin:coenzyme M methyltransferase"

"JS1_A09_00770" 11256 "s59E5H2" 0.2448366 "methylcobalamin:coenzyme M methyltransferase"
"JS1_A09_00770" 11256 "s63E6H2" 0.1693652 "methylcobalamin:coenzyme M methyltransferase"
"JS1_A09_00772" 11258 "s59E13H2" 0 "Sulfide dehydrogenase subunit alpha precursor"
"JS1_A09_00772" 11258 "s59E25H2" 0 "Sulfide dehydrogenase subunit alpha precursor"
"JS1_A09_00772" 11258 "s59E5H2" 0.005290294 "Sulfide dehydrogenase subunit alpha precursor"
"JS1_A09_00772" 11258 "s63E6H2" 0 "Sulfide dehydrogenase subunit alpha precursor"
"JS1_A09_00773" 11259 "s59E13H2" 0 "hypothetical protein"
"JS1_A09_00773" 11259 "s59E25H2" 0 "hypothetical protein"
"JS1_A09_00773" 11259 "s59E5H2" 0.0574298 "hypothetical protein"
"JS1_A09_00773" 11259 "s63E6H2" 0.1017453 "hypothetical protein"
"JS1_A09_00775" 11261 "s59E13H2" 0 "Helix-turn-helix"
"JS1_A09_00775" 11261 "s59E25H2" 0 "Helix-turn-helix"
"JS1_A09_00775" 11261 "s59E5H2" 0 "Helix-turn-helix"
"JS1_A09_00775" 11261 "s63E6H2" 0.3185998 "Helix-turn-helix"
"JS1_A09_00776" 11262 "s59E13H2" 0.008148192 "Quinone oxidoreductase 1"
"JS1_A09_00776" 11262 "s59E25H2" 0 "Quinone oxidoreductase 1"
"JS1_A09_00776" 11262 "s59E5H2" 0 "Quinone oxidoreductase 1"
"JS1_A09_00776" 11262 "s63E6H2" 0.3056737 "Quinone oxidoreductase 1"
"JS1_A09_00777" 11263 "s59E13H2" 0 "hypothetical protein"
"JS1_A09_00777" 11263 "s59E25H2" 0 "hypothetical protein"
"JS1_A09_00777" 11263 "s59E5H2" 0 "hypothetical protein"
"JS1_A09_00777" 11263 "s63E6H2" 0.3887589 "hypothetical protein"
"JS1_A09_00778" 11264 "s59E13H2" 0.002435432 "hypothetical protein"
"JS1_A09_00778" 11264 "s59E25H2" 0 "hypothetical protein"
"JS1_A09_00778" 11264 "s59E5H2" 0 "hypothetical protein"
"JS1_A09_00778" 11264 "s63E6H2" 0.4723284 "hypothetical protein"
"JS1_A09_00779" 11265 "s59E13H2" 0 "Acryloyl-coenzyme A reductase"
"JS1_A09_00779" 11265 "s59E25H2" 0 "Acryloyl-coenzyme A reductase"
"JS1_A09_00779" 11265 "s59E5H2" 0 "Acryloyl-coenzyme A reductase"
"JS1_A09_00779" 11265 "s63E6H2" 0.5561228 "Acryloyl-coenzyme A reductase"
"JS1_A09_00780" 11266 "s59E13H2" 0.006674559 "Alcohol dehydrogenase"
"JS1_A09_00780" 11266 "s59E25H2" 0 "Alcohol dehydrogenase"
"JS1_A09_00780" 11266 "s59E5H2" 0.001113533 "Alcohol dehydrogenase"
"JS1_A09_00780" 11266 "s63E6H2" 0.7504729 "Alcohol dehydrogenase"
"JS1_A09_00781" 11267 "s59E13H2" 0.06508687 "hypothetical protein"
"JS1_A09_00781" 11267 "s59E25H2" 0 "hypothetical protein"
"JS1_A09_00781" 11267 "s59E5H2" 0.3698155 "hypothetical protein"
"JS1_A09_00781" 11267 "s63E6H2" 0.4906897 "hypothetical protein"
"JS1_A09_00782" 11268 "s59E13H2" 0.05868119 "Cystathionine beta-lyase PatB"
"JS1_A09_00782" 11268 "s59E25H2" 0.4204738 "Cystathionine beta-lyase PatB"
"JS1_A09_00782" 11268 "s59E5H2" 0.8884359 "Cystathionine beta-lyase PatB"
"JS1_A09_00782" 11268 "s63E6H2" 1.050601 "Cystathionine beta-lyase PatB"
"JS1_A09_00783" 11269 "s59E13H2" 0.06995567 "Pyridoxamine 5'-phosphate oxidase"
"JS1_A09_00783" 11269 "s59E25H2" 0 "Pyridoxamine 5'-phosphate oxidase"
"JS1_A09_00783" 11269 "s59E5H2" 0.3068117 "Pyridoxamine 5'-phosphate oxidase"
"JS1_A09_00783" 11269 "s63E6H2" 0.3258359 "Pyridoxamine 5'-phosphate oxidase"
"JS1_A09_00784" 11270 "s59E13H2" 0.07842431 "hypothetical protein"

"JS1_A09_00784" 11270 "s59E25H2" 0 "hypothetical protein"
"JS1_A09_00784" 11270 "s59E5H2" 0.2256133 "hypothetical protein"
"JS1_A09_00784" 11270 "s63E6H2" 0.1041078 "hypothetical protein"
"JS1_A09_00785" 11271 "s59E13H2" 0.2184545 "L-galactonate transporter"
"JS1_A09_00785" 11271 "s59E25H2" 0 "L-galactonate transporter"
"JS1_A09_00785" 11271 "s59E5H2" 0.1718389 "L-galactonate transporter"
"JS1_A09_00785" 11271 "s63E6H2" 0.0651767 "L-galactonate transporter"
"JS1_A09_00786" 11272 "s59E13H2" 0.005925134 "Demethylmenaquinone methyltransferase"
"JS1_A09_00786" 11272 "s59E25H2" 0 "Demethylmenaquinone methyltransferase"
"JS1_A09_00786" 11272 "s59E5H2" 0.2376365 "Demethylmenaquinone methyltransferase"
"JS1_A09_00786" 11272 "s63E6H2" 0.7883103 "Demethylmenaquinone methyltransferase"
"JS1_A09_00787" 11273 "s59E13H2" 0.01798956 "hypothetical protein"
"JS1_A09_00787" 11273 "s59E25H2" 0 "hypothetical protein"
"JS1_A09_00787" 11273 "s59E5H2" 0.432378 "hypothetical protein"
"JS1_A09_00787" 11273 "s63E6H2" 0.4393446 "hypothetical protein"
"JS1_A09_00789" 11275 "s59E13H2" 0.04052055 "Ribokinase"
"JS1_A09_00789" 11275 "s59E25H2" 0 "Ribokinase"
"JS1_A09_00789" 11275 "s59E5H2" 0.8931663 "Ribokinase"
"JS1_A09_00789" 11275 "s63E6H2" 0.4838135 "Ribokinase"
"JS1_A09_00790" 11276 "s59E13H2" 0.08769741 "HTH-type transcriptional regulator DegA"
"JS1_A09_00790" 11276 "s59E25H2" 0 "HTH-type transcriptional regulator DegA"
"JS1_A09_00790" 11276 "s59E5H2" 0.8802686 "HTH-type transcriptional regulator DegA"
"JS1_A09_00790" 11276 "s63E6H2" 1.540916 "HTH-type transcriptional regulator DegA"
"JS1_A09_00791" 11277 "s59E13H2" 0.1725165 "D-ribose pyranase"
"JS1_A09_00791" 11277 "s59E25H2" 0 "D-ribose pyranase"
"JS1_A09_00791" 11277 "s59E5H2" 0.227089 "D-ribose pyranase"
"JS1_A09_00791" 11277 "s63E6H2" 0.2827961 "D-ribose pyranase"
"JS1_A09_00792" 11278 "s59E13H2" 0.0035514 "Galactose/methyl galactoside import ATP-binding protein MglA"
"JS1_A09_00792" 11278 "s59E25H2" 0 "Galactose/methyl galactoside import ATP-binding protein MglA"
"JS1_A09_00792" 11278 "s59E5H2" 0.221614 "Galactose/methyl galactoside import ATP-binding protein MglA"
"JS1_A09_00792" 11278 "s63E6H2" 2.215037 "Galactose/methyl galactoside import ATP-binding protein MglA"
"JS1_A09_00793" 11279 "s59E13H2" 0.001543202 "Ribose transport system permease protein RbsC"
"JS1_A09_00793" 11279 "s59E25H2" 0 "Ribose transport system permease protein RbsC"
"JS1_A09_00793" 11279 "s59E5H2" 0.2281632 "Ribose transport system permease protein RbsC"
"JS1_A09_00793" 11279 "s63E6H2" 2.338741 "Ribose transport system permease protein RbsC"
"JS1_A09_00794" 11280 "s59E13H2" 0.1716342 "D-ribose-binding periplasmic protein precursor"
"JS1_A09_00794" 11280 "s59E25H2" 0 "D-ribose-binding periplasmic protein precursor"
"JS1_A09_00794" 11280 "s59E5H2" 0.3241247 "D-ribose-binding periplasmic protein precursor"
"JS1_A09_00794" 11280 "s63E6H2" 3.055513 "D-ribose-binding periplasmic protein precursor"
"JS1_A09_00795" 11281 "s59E13H2" 0.3876305 "Cyclic di-GMP phosphodiesterase Gmr"
"JS1_A09_00795" 11281 "s59E25H2" 0 "Cyclic di-GMP phosphodiesterase Gmr"
"JS1_A09_00795" 11281 "s59E5H2" 1.225266 "Cyclic di-GMP phosphodiesterase Gmr"
"JS1_A09_00795" 11281 "s63E6H2" 2.188712 "Cyclic di-GMP phosphodiesterase Gmr"
"JS1_A09_00796" 11282 "s59E13H2" 1.100097 "L-asparaginase 1"
"JS1_A09_00796" 11282 "s59E25H2" 0 "L-asparaginase 1"
"JS1_A09_00796" 11282 "s59E5H2" 1.936863 "L-asparaginase 1"
"JS1_A09_00796" 11282 "s63E6H2" 3.553268 "L-asparaginase 1"

"JS1_A09_00797" 11283 "s59E13H2" 0.86474 "Multidrug export protein MepA"
"JS1_A09_00797" 11283 "s59E25H2" 0.3399852 "Multidrug export protein MepA"
"JS1_A09_00797" 11283 "s59E5H2" 0.259855 "Multidrug export protein MepA"
"JS1_A09_00797" 11283 "s63E6H2" 4.082384 "Multidrug export protein MepA"
"JS1_A09_00798" 11284 "s59E13H2" 0.1990754 "putative HTH-type transcriptional regulator YusO"
"JS1_A09_00798" 11284 "s59E25H2" 0 "putative HTH-type transcriptional regulator YusO"
"JS1_A09_00798" 11284 "s59E5H2" 0.1343914 "putative HTH-type transcriptional regulator YusO"
"JS1_A09_00798" 11284 "s63E6H2" 0.188895 "putative HTH-type transcriptional regulator YusO"
"JS1_A09_00799" 11285 "s59E13H2" 0.01198104 "Flavoredoxin"
"JS1_A09_00799" 11285 "s59E25H2" 0 "Flavoredoxin"
"JS1_A09_00799" 11285 "s59E5H2" 0.008147013 "Flavoredoxin"
"JS1_A09_00799" 11285 "s63E6H2" 0.007190226 "Flavoredoxin"
"JS1_A09_00800" 11286 "s59E13H2" 0.05631355 "hypothetical protein"
"JS1_A09_00800" 11286 "s59E25H2" 0 "hypothetical protein"
"JS1_A09_00800" 11286 "s59E5H2" 0 "hypothetical protein"
"JS1_A09_00800" 11286 "s63E6H2" 0.07817343 "hypothetical protein"
"JS1_A09_00801" 11287 "s59E13H2" 0.1634144 "PEGA domain protein"
"JS1_A09_00801" 11287 "s59E25H2" 0 "PEGA domain protein"
"JS1_A09_00801" 11287 "s59E5H2" 0.9276362 "PEGA domain protein"
"JS1_A09_00801" 11287 "s63E6H2" 1.421651 "PEGA domain protein"
"JS1_A09_00802" 11288 "s59E13H2" 0 "hypothetical protein"
"JS1_A09_00802" 11288 "s59E25H2" 0 "hypothetical protein"
"JS1_A09_00802" 11288 "s59E5H2" 0 "hypothetical protein"
"JS1_A09_00802" 11288 "s63E6H2" 0 "hypothetical protein"
"JS1_A09_00803" 11289 "s59E13H2" 0.1244455 "PEGA domain protein"
"JS1_A09_00803" 11289 "s59E25H2" 0 "PEGA domain protein"
"JS1_A09_00803" 11289 "s59E5H2" 0.06365708 "PEGA domain protein"
"JS1_A09_00803" 11289 "s63E6H2" 0.4289013 "PEGA domain protein"
"JS1_A09_00804" 11290 "s59E13H2" 0.07107111 "hypothetical protein"
"JS1_A09_00804" 11290 "s59E25H2" 0 "hypothetical protein"
"JS1_A09_00804" 11290 "s59E5H2" 0.0451511 "hypothetical protein"
"JS1_A09_00804" 11290 "s63E6H2" 0.2013454 "hypothetical protein"
"JS1_A09_00805" 11291 "s59E13H2" 0.009480215 "hypothetical protein"
"JS1_A09_00805" 11291 "s59E25H2" 0 "hypothetical protein"
"JS1_A09_00805" 11291 "s59E5H2" 0 "hypothetical protein"
"JS1_A09_00805" 11291 "s63E6H2" 0.041812 "hypothetical protein"
"JS1_A09_00806" 11292 "s59E13H2" 0 "tetratricopeptide repeat protein"
"JS1_A09_00806" 11292 "s59E25H2" 0 "tetratricopeptide repeat protein"
"JS1_A09_00806" 11292 "s59E5H2" 0 "tetratricopeptide repeat protein"
"JS1_A09_00806" 11292 "s63E6H2" 0.005188751 "tetratricopeptide repeat protein"
"JS1_A09_00807" 11293 "s59E13H2" 0 "hypothetical protein"
"JS1_A09_00807" 11293 "s59E25H2" 0 "hypothetical protein"
"JS1_A09_00807" 11293 "s59E5H2" 0 "hypothetical protein"
"JS1_A09_00807" 11293 "s63E6H2" 0.02450714 "hypothetical protein"
"JS1_A09_00808" 11294 "s59E13H2" 0 "hypothetical protein"
"JS1_A09_00808" 11294 "s59E25H2" 0 "hypothetical protein"
"JS1_A09_00808" 11294 "s59E5H2" 0 "hypothetical protein"

"JS1_A09_00808" 11294 "s63E6H2" 0.00202643 "hypothetical protein"
"JS1_A09_00809" 11295 "s59E13H2" 0 "hypothetical protein"
"JS1_A09_00809" 11295 "s59E25H2" 0 "hypothetical protein"
"JS1_A09_00809" 11295 "s59E5H2" 0 "hypothetical protein"
"JS1_A09_00809" 11295 "s63E6H2" 0 "hypothetical protein"
"JS1_A09_00810" 11296 "s59E13H2" 0 "hypothetical protein"
"JS1_A09_00810" 11296 "s59E25H2" 0 "hypothetical protein"
"JS1_A09_00810" 11296 "s59E5H2" 0.004510702 "hypothetical protein"
"JS1_A09_00810" 11296 "s63E6H2" 0 "hypothetical protein"
"JS1_A09_00811" 11297 "s59E13H2" 0 "hypothetical protein"
"JS1_A09_00811" 11297 "s59E25H2" 0 "hypothetical protein"
"JS1_A09_00811" 11297 "s59E5H2" 0.002575958 "hypothetical protein"
"JS1_A09_00811" 11297 "s63E6H2" 0 "hypothetical protein"
"JS1_A09_00812" 11298 "s59E13H2" 0.1805789 "hypothetical protein"
"JS1_A09_00812" 11298 "s59E25H2" 0 "hypothetical protein"
"JS1_A09_00812" 11298 "s59E5H2" 0.04000136 "hypothetical protein"
"JS1_A09_00812" 11298 "s63E6H2" 0.3576066 "hypothetical protein"
"JS1_A09_00813" 11299 "s59E13H2" 1.215749 "Cyclic di-GMP phosphodiesterase response regulator RpfG"
"JS1_A09_00813" 11299 "s59E25H2" 0 "Cyclic di-GMP phosphodiesterase response regulator RpfG"
"JS1_A09_00813" 11299 "s59E5H2" 0.1543331 "Cyclic di-GMP phosphodiesterase response regulator RpfG"
"JS1_A09_00813" 11299 "s63E6H2" 0.5668606 "Cyclic di-GMP phosphodiesterase response regulator RpfG"
"JS1_A09_00814" 11300 "s59E13H2" 0 "hypothetical protein"
"JS1_A09_00814" 11300 "s59E25H2" 0 "hypothetical protein"
"JS1_A09_00814" 11300 "s59E5H2" 0 "hypothetical protein"
"JS1_A09_00814" 11300 "s63E6H2" 0.003743605 "hypothetical protein"
"JS1_A09_00816" 11302 "s59E13H2" 1.180052 "hypothetical protein"
"JS1_A09_00816" 11302 "s59E25H2" 0 "hypothetical protein"
"JS1_A09_00816" 11302 "s59E5H2" 0.02403809 "hypothetical protein"
"JS1_A09_00816" 11302 "s63E6H2" 0.001746922 "hypothetical protein"
"JS1_A09_00817" 11303 "s59E13H2" 0.1578509 "Anti-sigma-V factor RsiV"
"JS1_A09_00817" 11303 "s59E25H2" 0 "Anti-sigma-V factor RsiV"
"JS1_A09_00817" 11303 "s59E5H2" 1.017959 "Anti-sigma-V factor RsiV"
"JS1_A09_00817" 11303 "s63E6H2" 0.02340818 "Anti-sigma-V factor RsiV"
"JS1_A09_00818" 11304 "s59E13H2" 0.08168661 "Response regulator PleD"
"JS1_A09_00818" 11304 "s59E25H2" 0 "Response regulator PleD"
"JS1_A09_00818" 11304 "s59E5H2" 0.07116873 "Response regulator PleD"
"JS1_A09_00818" 11304 "s63E6H2" 0.04191995 "Response regulator PleD"
"JS1_A09_00819" 11305 "s59E13H2" 7.587254 "D-alanine--D-alanine ligase"
"JS1_A09_00819" 11305 "s59E25H2" 0.2899061 "D-alanine--D-alanine ligase"
"JS1_A09_00819" 11305 "s59E5H2" 5.301275 "D-alanine--D-alanine ligase"
"JS1_A09_00819" 11305 "s63E6H2" 7.760434 "D-alanine--D-alanine ligase"
"JS1_A09_00820" 11306 "s59E13H2" 5.241311 "Methylated-DNA--protein-cysteine methyltransferase"
"JS1_A09_00820" 11306 "s59E25H2" 0 "Methylated-DNA--protein-cysteine methyltransferase"
"JS1_A09_00820" 11306 "s59E5H2" 4.987563 "Methylated-DNA--protein-cysteine methyltransferase"
"JS1_A09_00820" 11306 "s63E6H2" 4.090586 "Methylated-DNA--protein-cysteine methyltransferase"
"JS1_A09_00821" 11307 "s59E13H2" 4.053091 "molybdopterin-guanine dinucleotide biosynthesis protein MobA"
"JS1_A09_00821" 11307 "s59E25H2" 0 "molybdopterin-guanine dinucleotide biosynthesis protein MobA"

"JS1_A09_00821" 11307 "s59E5H2" 8.733395 "molybdopterin-guanine dinucleotide biosynthesis protein MobA"
"JS1_A09_00821" 11307 "s63E6H2" 13.34444 "molybdopterin-guanine dinucleotide biosynthesis protein MobA"
"JS1_A09_00822" 11308 "s59E13H2" 0.3887856 "hypothetical protein"
"JS1_A09_00822" 11308 "s59E25H2" 0 "hypothetical protein"
"JS1_A09_00822" 11308 "s59E5H2" 6.780643 "hypothetical protein"
"JS1_A09_00822" 11308 "s63E6H2" 10.23639 "hypothetical protein"
"JS1_A09_00823" 11309 "s59E13H2" 0.008056701 "Tetratricopeptide repeat protein"
"JS1_A09_00823" 11309 "s59E25H2" 0 "Tetratricopeptide repeat protein"
"JS1_A09_00823" 11309 "s59E5H2" 1.062203 "Tetratricopeptide repeat protein"
"JS1_A09_00823" 11309 "s63E6H2" 0.02010842 "Tetratricopeptide repeat protein"
"JS1_A09_00824" 11310 "s59E13H2" 0.07429379 "Putative competence-damage inducible protein"
"JS1_A09_00824" 11310 "s59E25H2" 0.1457327 "Putative competence-damage inducible protein"
"JS1_A09_00824" 11310 "s59E5H2" 4.414173 "Putative competence-damage inducible protein"
"JS1_A09_00824" 11310 "s63E6H2" 1.515632 "Putative competence-damage inducible protein"
"JS1_A09_00825" 11311 "s59E13H2" 0.2085147 "NADH-dependent butanol dehydrogenase A"
"JS1_A09_00825" 11311 "s59E25H2" 0 "NADH-dependent butanol dehydrogenase A"
"JS1_A09_00825" 11311 "s59E5H2" 4.407603 "NADH-dependent butanol dehydrogenase A"
"JS1_A09_00825" 11311 "s63E6H2" 4.462791 "NADH-dependent butanol dehydrogenase A"
"JS1_A09_00826" 11312 "s59E13H2" 0.2608801 "1,3-propanediol dehydrogenase"
"JS1_A09_00826" 11312 "s59E25H2" 0 "1,3-propanediol dehydrogenase"
"JS1_A09_00826" 11312 "s59E5H2" 9.404651 "1,3-propanediol dehydrogenase"
"JS1_A09_00826" 11312 "s63E6H2" 6.590152 "1,3-propanediol dehydrogenase"
"JS1_A09_00827" 11313 "s59E13H2" 0.1782034 "hypothetical protein"
"JS1_A09_00827" 11313 "s59E25H2" 0 "hypothetical protein"
"JS1_A09_00827" 11313 "s59E5H2" 5.485975 "hypothetical protein"
"JS1_A09_00827" 11313 "s63E6H2" 1.663039 "hypothetical protein"
"JS1_A09_00828" 11314 "s59E13H2" 0.05894111 "methylcobalamin:coenzyme M methyltransferase"
"JS1_A09_00828" 11314 "s59E25H2" 0 "methylcobalamin:coenzyme M methyltransferase"
"JS1_A09_00828" 11314 "s59E5H2" 0.01201188 "methylcobalamin:coenzyme M methyltransferase"
"JS1_A09_00828" 11314 "s63E6H2" 0.3532868 "methylcobalamin:coenzyme M methyltransferase"
"JS1_A09_00829" 11315 "s59E13H2" 2.773328 "Sialic acid-binding periplasmic protein SiaP precursor"
"JS1_A09_00829" 11315 "s59E25H2" 0 "Sialic acid-binding periplasmic protein SiaP precursor"
"JS1_A09_00829" 11315 "s59E5H2" 0.3864037 "Sialic acid-binding periplasmic protein SiaP precursor"
"JS1_A09_00829" 11315 "s63E6H2" 0.09451461 "Sialic acid-binding periplasmic protein SiaP precursor"
"JS1_A09_00830" 11316 "s59E13H2" 2.28275 "Tripartite ATP-independent periplasmic transporters, DctQ component"
"JS1_A09_00830" 11316 "s59E25H2" 0 "Tripartite ATP-independent periplasmic transporters, DctQ component"
"JS1_A09_00830" 11316 "s59E5H2" 0.502885 "Tripartite ATP-independent periplasmic transporters, DctQ component"
"JS1_A09_00830" 11316 "s63E6H2" 0.05178897 "Tripartite ATP-independent periplasmic transporters, DctQ component"
"JS1_A09_00831" 11317 "s59E13H2" 1.373463 "Sialic acid TRAP transporter permease protein SiaT"
"JS1_A09_00831" 11317 "s59E25H2" 0 "Sialic acid TRAP transporter permease protein SiaT"
"JS1_A09_00831" 11317 "s59E5H2" 0.3506022 "Sialic acid TRAP transporter permease protein SiaT"
"JS1_A09_00831" 11317 "s63E6H2" 0.02844524 "Sialic acid TRAP transporter permease protein SiaT"
"JS1_A09_00832" 11318 "s59E13H2" 0.05265866 "Methylated-thiol--coenzyme M methyltransferase"
"JS1_A09_00832" 11318 "s59E25H2" 0 "Methylated-thiol--coenzyme M methyltransferase"
"JS1_A09_00832" 11318 "s59E5H2" 0.01079393 "Methylated-thiol--coenzyme M methyltransferase"
"JS1_A09_00832" 11318 "s63E6H2" 0.05240066 "Methylated-thiol--coenzyme M methyltransferase"
"JS1_A09_00833" 11319 "s59E13H2" 0.05011447 "methylcobalamin:coenzyme M methyltransferase"

"JS1_A09_00833" 11319 "s59E25H2" 0 "methylcobalamin:coenzyme M methyltransferase"
"JS1_A09_00833" 11319 "s59E5H2" 0.001565067 "methylcobalamin:coenzyme M methyltransferase"
"JS1_A09_00833" 11319 "s63E6H2" 0.01177101 "methylcobalamin:coenzyme M methyltransferase"
"JS1_A09_00835" 11321 "s59E13H2" 0.008599108 "N5-carboxyaminoimidazole ribonucleotide mutase"
"JS1_A09_00835" 11321 "s59E25H2" 0 "N5-carboxyaminoimidazole ribonucleotide mutase"
"JS1_A09_00835" 11321 "s59E5H2" 0.01922151 "N5-carboxyaminoimidazole ribonucleotide mutase"
"JS1_A09_00835" 11321 "s63E6H2" 0.08622587 "N5-carboxyaminoimidazole ribonucleotide mutase"
"JS1_A09_00836" 11322 "s59E13H2" 0.01838507 "Phosphoribosylaminoimidazole-succinocarboxamide synthase"
"JS1_A09_00836" 11322 "s59E25H2" 0 "Phosphoribosylaminoimidazole-succinocarboxamide synthase"
"JS1_A09_00836" 11322 "s59E5H2" 0.01308983 "Phosphoribosylaminoimidazole-succinocarboxamide synthase"
"JS1_A09_00836" 11322 "s63E6H2" 0.06912443 "Phosphoribosylaminoimidazole-succinocarboxamide synthase"
"JS1_A09_00837" 11323 "s59E13H2" 0.822984 "Phosphoribosylformylglycinamidine synthase 2"
"JS1_A09_00837" 11323 "s59E25H2" 0 "Phosphoribosylformylglycinamidine synthase 2"
"JS1_A09_00837" 11323 "s59E5H2" 2.142012 "Phosphoribosylformylglycinamidine synthase 2"
"JS1_A09_00837" 11323 "s63E6H2" 0.1559938 "Phosphoribosylformylglycinamidine synthase 2"
"JS1_A09_00838" 11324 "s59E13H2" 0.02559104 "Amidophosphoribosyltransferase precursor"
"JS1_A09_00838" 11324 "s59E25H2" 0 "Amidophosphoribosyltransferase precursor"
"JS1_A09_00838" 11324 "s59E5H2" 0.3122432 "Amidophosphoribosyltransferase precursor"
"JS1_A09_00838" 11324 "s63E6H2" 0.1087099 "Amidophosphoribosyltransferase precursor"
"JS1_A09_00839" 11325 "s59E13H2" 1.173135 "Phosphoribosylformylglycinamidine cyclo-ligase"
"JS1_A09_00839" 11325 "s59E25H2" 0 "Phosphoribosylformylglycinamidine cyclo-ligase"
"JS1_A09_00839" 11325 "s59E5H2" 0.05695754 "Phosphoribosylformylglycinamidine cyclo-ligase"
"JS1_A09_00839" 11325 "s63E6H2" 0.2650234 "Phosphoribosylformylglycinamidine cyclo-ligase"
"JS1_A09_00840" 11326 "s59E13H2" 1.264262 "Phosphoribosylglycinamide formyltransferase"
"JS1_A09_00840" 11326 "s59E25H2" 0 "Phosphoribosylglycinamide formyltransferase"
"JS1_A09_00840" 11326 "s59E5H2" 0.02856353 "Phosphoribosylglycinamide formyltransferase"
"JS1_A09_00840" 11326 "s63E6H2" 0.3001638 "Phosphoribosylglycinamide formyltransferase"
"JS1_A09_00841" 11327 "s59E13H2" 1.616418 "Phosphoribosylamine--glycine ligase"
"JS1_A09_00841" 11327 "s59E25H2" 0 "Phosphoribosylamine--glycine ligase"
"JS1_A09_00841" 11327 "s59E5H2" 0.1523599 "Phosphoribosylamine--glycine ligase"
"JS1_A09_00841" 11327 "s63E6H2" 0.484032 "Phosphoribosylamine--glycine ligase"
"JS1_A09_00842" 11328 "s59E13H2" 0.8281971 "Bifunctional purine biosynthesis protein PurH"
"JS1_A09_00842" 11328 "s59E25H2" 0 "Bifunctional purine biosynthesis protein PurH"
"JS1_A09_00842" 11328 "s59E5H2" 0.2927984 "Bifunctional purine biosynthesis protein PurH"
"JS1_A09_00842" 11328 "s63E6H2" 0.3939925 "Bifunctional purine biosynthesis protein PurH"
"JS1_A09_00844" 11330 "s59E13H2" 0 "hypothetical protein"
"JS1_A09_00844" 11330 "s59E25H2" 0 "hypothetical protein"
"JS1_A09_00844" 11330 "s59E5H2" 0.02023053 "hypothetical protein"
"JS1_A09_00844" 11330 "s63E6H2" 0.001368023 "hypothetical protein"
"JS1_A09_00845" 11331 "s59E13H2" 0 "hypothetical protein"
"JS1_A09_00845" 11331 "s59E25H2" 0 "hypothetical protein"
"JS1_A09_00845" 11331 "s59E5H2" 0.007997245 "hypothetical protein"
"JS1_A09_00845" 11331 "s63E6H2" 0.005101715 "hypothetical protein"
"JS1_A09_00846" 11332 "s59E13H2" 0 "DctM-like transporters"
"JS1_A09_00846" 11332 "s59E25H2" 0 "DctM-like transporters"
"JS1_A09_00846" 11332 "s59E5H2" 0 "DctM-like transporters"
"JS1_A09_00846" 11332 "s63E6H2" 0 "DctM-like transporters"

"JS1_A09_00850" 11336 "s59E13H2" 0 "Nucleotidyltransferase domain protein"
"JS1_A09_00850" 11336 "s59E25H2" 0 "Nucleotidyltransferase domain protein"
"JS1_A09_00850" 11336 "s59E5H2" 0.02156214 "Nucleotidyltransferase domain protein"
"JS1_A09_00850" 11336 "s63E6H2" 0.02347448 "Nucleotidyltransferase domain protein"
"JS1_A09_00851" 11337 "s59E13H2" 0 "HEPN domain protein"
"JS1_A09_00851" 11337 "s59E25H2" 0 "HEPN domain protein"
"JS1_A09_00851" 11337 "s59E5H2" 0.006847704 "HEPN domain protein"
"JS1_A09_00851" 11337 "s63E6H2" 0.02734907 "HEPN domain protein"
"JS1_A09_00852" 11338 "s59E13H2" 2.508503 "hypothetical protein"
"JS1_A09_00852" 11338 "s59E25H2" 0 "hypothetical protein"
"JS1_A09_00852" 11338 "s59E5H2" 0.00471614 "hypothetical protein"
"JS1_A09_00852" 11338 "s63E6H2" 0.06129029 "hypothetical protein"
"JS1_A09_00854" 11340 "s59E13H2" 0 "Adenylate cyclase 1"
"JS1_A09_00854" 11340 "s59E25H2" 0 "Adenylate cyclase 1"
"JS1_A09_00854" 11340 "s59E5H2" 0 "Adenylate cyclase 1"
"JS1_A09_00854" 11340 "s63E6H2" 0 "Adenylate cyclase 1"
"JS1_A09_00855" 11341 "s59E13H2" 0.01469302 "Adenylate cyclase 2"
"JS1_A09_00855" 11341 "s59E25H2" 0 "Adenylate cyclase 2"
"JS1_A09_00855" 11341 "s59E5H2" 0 "Adenylate cyclase 2"
"JS1_A09_00855" 11341 "s63E6H2" 0 "Adenylate cyclase 2"
"JS1_A09_00856" 11342 "s59E13H2" 0 "methylcobalamin:coenzyme M methyltransferase"
"JS1_A09_00856" 11342 "s59E25H2" 0 "methylcobalamin:coenzyme M methyltransferase"
"JS1_A09_00856" 11342 "s59E5H2" 0.0003899729 "methylcobalamin:coenzyme M methyltransferase"
"JS1_A09_00856" 11342 "s63E6H2" 0 "methylcobalamin:coenzyme M methyltransferase"
"JS1_A09_00857" 11343 "s59E13H2" 0 "4-hydroxy-tetrahydrodipicolinate synthase"
"JS1_A09_00857" 11343 "s59E25H2" 0 "4-hydroxy-tetrahydrodipicolinate synthase"
"JS1_A09_00857" 11343 "s59E5H2" 0.002094676 "4-hydroxy-tetrahydrodipicolinate synthase"
"JS1_A09_00857" 11343 "s63E6H2" 0.006165099 "4-hydroxy-tetrahydrodipicolinate synthase"
"JS1_A09_00858" 11344 "s59E13H2" 0 "4-hydroxy-tetrahydrodipicolinate synthase"
"JS1_A09_00858" 11344 "s59E25H2" 0 "4-hydroxy-tetrahydrodipicolinate synthase"
"JS1_A09_00858" 11344 "s59E5H2" 0.03427412 "4-hydroxy-tetrahydrodipicolinate synthase"
"JS1_A09_00858" 11344 "s63E6H2" 0.004261055 "4-hydroxy-tetrahydrodipicolinate synthase"
"JS1_A09_00859" 11345 "s59E13H2" 0 "Methylcobamide:CoM methyltransferase MtbA"
"JS1_A09_00859" 11345 "s59E25H2" 0 "Methylcobamide:CoM methyltransferase MtbA"
"JS1_A09_00859" 11345 "s59E5H2" 0 "Methylcobamide:CoM methyltransferase MtbA"
"JS1_A09_00859" 11345 "s63E6H2" 0 "Methylcobamide:CoM methyltransferase MtbA"
"JS1_A09_00860" 11346 "s59E13H2" 0 "Dimethylamine corrinoid protein"
"JS1_A09_00860" 11346 "s59E25H2" 0 "Dimethylamine corrinoid protein"
"JS1_A09_00860" 11346 "s59E5H2" 0 "Dimethylamine corrinoid protein"
"JS1_A09_00860" 11346 "s63E6H2" 0 "Dimethylamine corrinoid protein"
"JS1_A09_00861" 11347 "s59E13H2" 0 "Methylated-thiol--coenzyme M methyltransferase"
"JS1_A09_00861" 11347 "s59E25H2" 0 "Methylated-thiol--coenzyme M methyltransferase"
"JS1_A09_00861" 11347 "s59E5H2" 0 "Methylated-thiol--coenzyme M methyltransferase"
"JS1_A09_00861" 11347 "s63E6H2" 0 "Methylated-thiol--coenzyme M methyltransferase"
"JS1_A09_00862" 11348 "s59E13H2" 0.0004830273 "2Fe-2S ferredoxin"
"JS1_A09_00862" 11348 "s59E25H2" 0 "2Fe-2S ferredoxin"
"JS1_A09_00862" 11348 "s59E5H2" 0.02733998 "2Fe-2S ferredoxin"

"JS1_A09_00862" 11348 "s63E6H2" 0.009430693 "2Fe-2S ferredoxin"
"JS1_A09_00863" 11349 "s59E13H2" 0 "Vitamin B12 dependent methionine synthase, activation domain"
"JS1_A09_00863" 11349 "s59E25H2" 0 "Vitamin B12 dependent methionine synthase, activation domain"
"JS1_A09_00863" 11349 "s59E5H2" 0 "Vitamin B12 dependent methionine synthase, activation domain"
"JS1_A09_00863" 11349 "s63E6H2" 0 "Vitamin B12 dependent methionine synthase, activation domain"
"JS1_A09_00864" 11350 "s59E13H2" 0 "Ribulokinase"
"JS1_A09_00864" 11350 "s59E25H2" 0 "Ribulokinase"
"JS1_A09_00864" 11350 "s59E5H2" 0 "Ribulokinase"
"JS1_A09_00864" 11350 "s63E6H2" 0.003908671 "Ribulokinase"
"JS1_A09_00868" 11354 "s59E13H2" 0.23883 "50S ribosomal protein L17"
"JS1_A09_00868" 11354 "s59E25H2" 0 "50S ribosomal protein L17"
"JS1_A09_00868" 11354 "s59E5H2" 0.1174979 "50S ribosomal protein L17"
"JS1_A09_00868" 11354 "s63E6H2" 1.829011 "50S ribosomal protein L17"
"JS1_A09_00869" 11355 "s59E13H2" 0.2579945 "Energy-coupling factor transporter ATP-binding protein EcfA1"
"JS1_A09_00869" 11355 "s59E25H2" 0 "Energy-coupling factor transporter ATP-binding protein EcfA1"
"JS1_A09_00869" 11355 "s59E5H2" 0.2316155 "Energy-coupling factor transporter ATP-binding protein EcfA1"
"JS1_A09_00869" 11355 "s63E6H2" 2.911502 "Energy-coupling factor transporter ATP-binding protein EcfA1"
"JS1_A09_00870" 11356 "s59E13H2" 0.2842312 "Energy-coupling factor transporter ATP-binding protein EcfA2"
"JS1_A09_00870" 11356 "s59E25H2" 0 "Energy-coupling factor transporter ATP-binding protein EcfA2"
"JS1_A09_00870" 11356 "s59E5H2" 0.6114404 "Energy-coupling factor transporter ATP-binding protein EcfA2"
"JS1_A09_00870" 11356 "s63E6H2" 1.785542 "Energy-coupling factor transporter ATP-binding protein EcfA2"
"JS1_A09_00871" 11357 "s59E13H2" 0.2747372 "Energy-coupling factor transporter transmembrane protein EcfT"
"JS1_A09_00871" 11357 "s59E25H2" 0 "Energy-coupling factor transporter transmembrane protein EcfT"
"JS1_A09_00871" 11357 "s59E5H2" 0.5575983 "Energy-coupling factor transporter transmembrane protein EcfT"
"JS1_A09_00871" 11357 "s63E6H2" 1.002138 "Energy-coupling factor transporter transmembrane protein EcfT"
"JS1_A09_00872" 11358 "s59E13H2" 0.2547591 "tRNA pseudouridine synthase A"
"JS1_A09_00872" 11358 "s59E25H2" 0 "tRNA pseudouridine synthase A"
"JS1_A09_00872" 11358 "s59E5H2" 0.356593 "tRNA pseudouridine synthase A"
"JS1_A09_00872" 11358 "s63E6H2" 0.9839334 "tRNA pseudouridine synthase A"
"JS1_A09_00873" 11359 "s59E13H2" 0.1725183 "50S ribosomal protein L13"
"JS1_A09_00873" 11359 "s59E25H2" 0 "50S ribosomal protein L13"
"JS1_A09_00873" 11359 "s59E5H2" 0.4538524 "50S ribosomal protein L13"
"JS1_A09_00873" 11359 "s63E6H2" 1.627723 "50S ribosomal protein L13"
"JS1_A09_00874" 11360 "s59E13H2" 0.1344217 "30S ribosomal protein S9"
"JS1_A09_00874" 11360 "s59E25H2" 0 "30S ribosomal protein S9"
"JS1_A09_00874" 11360 "s59E5H2" 0.4510822 "30S ribosomal protein S9"
"JS1_A09_00874" 11360 "s63E6H2" 1.417898 "30S ribosomal protein S9"
"JS1_A09_00875" 11361 "s59E13H2" 0.2132474 "alanine racemase"
"JS1_A09_00875" 11361 "s59E25H2" 0 "alanine racemase"
"JS1_A09_00875" 11361 "s59E5H2" 0.4328637 "alanine racemase"
"JS1_A09_00875" 11361 "s63E6H2" 1.91592 "alanine racemase"
"JS1_A09_00876" 11362 "s59E13H2" 0.161 "Ornithine carbamoyltransferase"
"JS1_A09_00876" 11362 "s59E25H2" 0 "Ornithine carbamoyltransferase"
"JS1_A09_00876" 11362 "s59E5H2" 0.3576138 "Ornithine carbamoyltransferase"
"JS1_A09_00876" 11362 "s63E6H2" 1.975789 "Ornithine carbamoyltransferase"
"JS1_A09_00880" 11366 "s59E13H2" 0.1021317 "putative inner membrane transporter YicL"
"JS1_A09_00880" 11366 "s59E25H2" 0 "putative inner membrane transporter YicL"

"JS1_A09_00880" 11366 "s59E5H2" 0.5110401 "putative inner membrane transporter YicL"
"JS1_A09_00880" 11366 "s63E6H2" 0.1683292 "putative inner membrane transporter YicL"
"JS1_A09_00881" 11367 "s59E13H2" 0.02689218 "Acetyltransferase (GNAT) family protein"
"JS1_A09_00881" 11367 "s59E25H2" 0 "Acetyltransferase (GNAT) family protein"
"JS1_A09_00881" 11367 "s59E5H2" 0.4584911 "Acetyltransferase (GNAT) family protein"
"JS1_A09_00881" 11367 "s63E6H2" 0.6245894 "Acetyltransferase (GNAT) family protein"
"JS1_A09_00882" 11368 "s59E13H2" 0.07005103 "tRNA 2'-O-methylase"
"JS1_A09_00882" 11368 "s59E25H2" 0 "tRNA 2'-O-methylase"
"JS1_A09_00882" 11368 "s59E5H2" 1.783009 "tRNA 2'-O-methylase"
"JS1_A09_00882" 11368 "s63E6H2" 1.070082 "tRNA 2'-O-methylase"
"JS1_A09_00883" 11369 "s59E13H2" 8.1322 "Bifunctional NAD(P)H-hydrate repair enzyme Nnr"
"JS1_A09_00883" 11369 "s59E25H2" 0.2168285 "Bifunctional NAD(P)H-hydrate repair enzyme Nnr"
"JS1_A09_00883" 11369 "s59E5H2" 0.7929374 "Bifunctional NAD(P)H-hydrate repair enzyme Nnr"
"JS1_A09_00883" 11369 "s63E6H2" 0.1721954 "Bifunctional NAD(P)H-hydrate repair enzyme Nnr"
"JS1_A09_00891" 11377 "s59E13H2" 0 "hypothetical protein"
"JS1_A09_00891" 11377 "s59E25H2" 0 "hypothetical protein"
"JS1_A09_00891" 11377 "s59E5H2" 0 "hypothetical protein"
"JS1_A09_00891" 11377 "s63E6H2" 0 "hypothetical protein"
"JS1_A09_00892" 11378 "s59E13H2" 0 "Aspartate aminotransferase"
"JS1_A09_00892" 11378 "s59E25H2" 0 "Aspartate aminotransferase"
"JS1_A09_00892" 11378 "s59E5H2" 0 "Aspartate aminotransferase"
"JS1_A09_00892" 11378 "s63E6H2" 0 "Aspartate aminotransferase"
"JS1_A09_00894" 11380 "s59E13H2" 0 "Sulfide dehydrogenase subunit alpha precursor"
"JS1_A09_00894" 11380 "s59E25H2" 0 "Sulfide dehydrogenase subunit alpha precursor"
"JS1_A09_00894" 11380 "s59E5H2" 0.01953518 "Sulfide dehydrogenase subunit alpha precursor"
"JS1_A09_00894" 11380 "s63E6H2" 0.001652879 "Sulfide dehydrogenase subunit alpha precursor"
"JS1_A09_00895" 11381 "s59E13H2" 0.001694311 "CoB--CoM heterodisulfide reductase subunit B"
"JS1_A09_00895" 11381 "s59E25H2" 0 "CoB--CoM heterodisulfide reductase subunit B"
"JS1_A09_00895" 11381 "s59E5H2" 0.05594433 "CoB--CoM heterodisulfide reductase subunit B"
"JS1_A09_00895" 11381 "s63E6H2" 0.7406601 "CoB--CoM heterodisulfide reductase subunit B"
"JS1_A09_00901" 11387 "s59E13H2" 0 "hypothetical protein"
"JS1_A09_00901" 11387 "s59E25H2" 0 "hypothetical protein"
"JS1_A09_00901" 11387 "s59E5H2" 0 "hypothetical protein"
"JS1_A09_00901" 11387 "s63E6H2" 0 "hypothetical protein"
"JS1_A09_00903" 11389 "s59E13H2" 0 "hypothetical protein"
"JS1_A09_00903" 11389 "s59E25H2" 0 "hypothetical protein"
"JS1_A09_00903" 11389 "s59E5H2" 0 "hypothetical protein"
"JS1_A09_00903" 11389 "s63E6H2" 0 "hypothetical protein"
"JS1_A09_00904" 11390 "s59E13H2" 0 "hypothetical protein"
"JS1_A09_00904" 11390 "s59E25H2" 0 "hypothetical protein"
"JS1_A09_00904" 11390 "s59E5H2" 0 "hypothetical protein"
"JS1_A09_00904" 11390 "s63E6H2" 0 "hypothetical protein"
"JS1_A09_00906" 11392 "s59E13H2" 0.63162 "Integrase core domain protein"
"JS1_A09_00906" 11392 "s59E25H2" 0 "Integrase core domain protein"
"JS1_A09_00906" 11392 "s59E5H2" 5.881475 "Integrase core domain protein"
"JS1_A09_00906" 11392 "s63E6H2" 3.420201 "Integrase core domain protein"
"JS1_A09_00908" 11394 "s59E13H2" 0 "CoB--CoM heterodisulfide reductase iron-sulfur subunit A"

"JS1_A09_00908" 11394 "s59E25H2" 0 "CoB--CoM heterodisulfide reductase iron-sulfur subunit A"
"JS1_A09_00908" 11394 "s59E5H2" 0 "CoB--CoM heterodisulfide reductase iron-sulfur subunit A"
"JS1_A09_00908" 11394 "s63E6H2" 0 "CoB--CoM heterodisulfide reductase iron-sulfur subunit A"
"JS1_A09_00910" 11396 "s59E13H2" 0.5127004 "hypothetical protein"
"JS1_A09_00910" 11396 "s59E25H2" 0 "hypothetical protein"
"JS1_A09_00910" 11396 "s59E5H2" 1.452088 "hypothetical protein"
"JS1_A09_00910" 11396 "s63E6H2" 5.13468 "hypothetical protein"
"JS1_A09_00911" 11397 "s59E13H2" 0.3347837 "hypothetical protein"
"JS1_A09_00911" 11397 "s59E25H2" 0 "hypothetical protein"
"JS1_A09_00911" 11397 "s59E5H2" 0.7005886 "hypothetical protein"
"JS1_A09_00911" 11397 "s63E6H2" 3.723938 "hypothetical protein"
"JS1_A09_00912" 11398 "s59E13H2" 0.003158578 "sporulation sigma factor SigG"
"JS1_A09_00912" 11398 "s59E25H2" 0 "sporulation sigma factor SigG"
"JS1_A09_00912" 11398 "s59E5H2" 0 "sporulation sigma factor SigG"
"JS1_A09_00912" 11398 "s63E6H2" 0.8018201 "sporulation sigma factor SigG"
"JS1_A09_00913" 11399 "s59E13H2" 0.05895079 "RNA polymerase factor sigma-70"
"JS1_A09_00913" 11399 "s59E25H2" 0 "RNA polymerase factor sigma-70"
"JS1_A09_00913" 11399 "s59E5H2" 0 "RNA polymerase factor sigma-70"
"JS1_A09_00913" 11399 "s63E6H2" 0.8576114 "RNA polymerase factor sigma-70"
"JS1_A09_00914" 11400 "s59E13H2" 0.01277478 "hypothetical protein"
"JS1_A09_00914" 11400 "s59E25H2" 0 "hypothetical protein"
"JS1_A09_00914" 11400 "s59E5H2" 0.0674195 "hypothetical protein"
"JS1_A09_00914" 11400 "s63E6H2" 0.5787712 "hypothetical protein"
"JS1_A09_00915" 11401 "s59E13H2" 0.01607125 "Putative ski2-type helicase"
"JS1_A09_00915" 11401 "s59E25H2" 0 "Putative ski2-type helicase"
"JS1_A09_00915" 11401 "s59E5H2" 0.3143019 "Putative ski2-type helicase"
"JS1_A09_00915" 11401 "s63E6H2" 0.196764 "Putative ski2-type helicase"
"JS1_A09_00917" 11403 "s59E13H2" 0.2641392 "Cypemycin methyltransferase"
"JS1_A09_00917" 11403 "s59E25H2" 0 "Cypemycin methyltransferase"
"JS1_A09_00917" 11403 "s59E5H2" 0.008807798 "Cypemycin methyltransferase"
"JS1_A09_00917" 11403 "s63E6H2" 0.09910756 "Cypemycin methyltransferase"
"JS1_A09_00923" 11409 "s59E13H2" 0.07978987 "Ribosome-recycling factor"
"JS1_A09_00923" 11409 "s59E25H2" 0 "Ribosome-recycling factor"
"JS1_A09_00923" 11409 "s59E5H2" 0.09744472 "Ribosome-recycling factor"
"JS1_A09_00923" 11409 "s63E6H2" 0.159358 "Ribosome-recycling factor"
"JS1_A09_00924" 11410 "s59E13H2" 0.06508309 "Uridylate kinase"
"JS1_A09_00924" 11410 "s59E25H2" 0 "Uridylate kinase"
"JS1_A09_00924" 11410 "s59E5H2" 0.1807608 "Uridylate kinase"
"JS1_A09_00924" 11410 "s63E6H2" 0.08265301 "Uridylate kinase"
"JS1_A09_00925" 11411 "s59E13H2" 0.06370164 "Elongation factor Ts"
"JS1_A09_00925" 11411 "s59E25H2" 0 "Elongation factor Ts"
"JS1_A09_00925" 11411 "s59E5H2" 0.05066166 "Elongation factor Ts"
"JS1_A09_00925" 11411 "s63E6H2" 0.01744756 "Elongation factor Ts"
"JS1_A09_00926" 11412 "s59E13H2" 0.04586405 "30S ribosomal protein S2"
"JS1_A09_00926" 11412 "s59E25H2" 0 "30S ribosomal protein S2"
"JS1_A09_00926" 11412 "s59E5H2" 0.08139925 "30S ribosomal protein S2"
"JS1_A09_00926" 11412 "s63E6H2" 0.02603707 "30S ribosomal protein S2"

"JS1_A09_00927" 11413 "s59E13H2" 0.04255814 "ATP-dependent protease ATPase subunit HslU"
"JS1_A09_00927" 11413 "s59E25H2" 0 "ATP-dependent protease ATPase subunit HslU"
"JS1_A09_00927" 11413 "s59E5H2" 1.702548 "ATP-dependent protease ATPase subunit HslU"
"JS1_A09_00927" 11413 "s63E6H2" 0.0856527 "ATP-dependent protease ATPase subunit HslU"
"JS1_A09_00928" 11414 "s59E13H2" 0 "CoB--CoM heterodisulfide reductase subunit B"
"JS1_A09_00928" 11414 "s59E25H2" 0 "CoB--CoM heterodisulfide reductase subunit B"
"JS1_A09_00928" 11414 "s59E5H2" 0.001602855 "CoB--CoM heterodisulfide reductase subunit B"
"JS1_A09_00928" 11414 "s63E6H2" 0 "CoB--CoM heterodisulfide reductase subunit B"
"JS1_A09_00929" 11415 "s59E13H2" 0 "hypothetical protein"
"JS1_A09_00929" 11415 "s59E25H2" 0 "hypothetical protein"
"JS1_A09_00929" 11415 "s59E5H2" 0 "hypothetical protein"
"JS1_A09_00929" 11415 "s63E6H2" 0.006900122 "hypothetical protein"
"JS1_A09_00930" 11416 "s59E13H2" 0 "Uroporphyrinogen decarboxylase (URO-D)"
"JS1_A09_00930" 11416 "s59E25H2" 0 "Uroporphyrinogen decarboxylase (URO-D)"
"JS1_A09_00930" 11416 "s59E5H2" 0 "Uroporphyrinogen decarboxylase (URO-D)"
"JS1_A09_00930" 11416 "s63E6H2" 0 "Uroporphyrinogen decarboxylase (URO-D)"
"JS1_A09_00931" 11417 "s59E13H2" 0.1691406 "DNA replication protein DnaC"
"JS1_A09_00931" 11417 "s59E25H2" 0 "DNA replication protein DnaC"
"JS1_A09_00931" 11417 "s59E5H2" 0.1713999 "DNA replication protein DnaC"
"JS1_A09_00931" 11417 "s63E6H2" 0.1576121 "DNA replication protein DnaC"
"JS1_A09_00933" 11419 "s59E13H2" 2.483099 "Phosphate acetyltransferase"
"JS1_A09_00933" 11419 "s59E25H2" 0 "Phosphate acetyltransferase"
"JS1_A09_00933" 11419 "s59E5H2" 1.514411 "Phosphate acetyltransferase"
"JS1_A09_00933" 11419 "s63E6H2" 0.1926823 "Phosphate acetyltransferase"
"JS1_A09_00934" 11420 "s59E13H2" 0 "Branched-chain-amino-acid aminotransferase"
"JS1_A09_00934" 11420 "s59E25H2" 0 "Branched-chain-amino-acid aminotransferase"
"JS1_A09_00934" 11420 "s59E5H2" 0 "Branched-chain-amino-acid aminotransferase"
"JS1_A09_00934" 11420 "s63E6H2" 0 "Branched-chain-amino-acid aminotransferase"
"JS1_A09_00935" 11421 "s59E13H2" 0 "succinyl-CoA synthetase subunit alpha"
"JS1_A09_00935" 11421 "s59E25H2" 0 "succinyl-CoA synthetase subunit alpha"
"JS1_A09_00935" 11421 "s59E5H2" 0.0007245467 "succinyl-CoA synthetase subunit alpha"
"JS1_A09_00935" 11421 "s63E6H2" 0.1813258 "succinyl-CoA synthetase subunit alpha"
"JS1_A09_00941" 11427 "s59E13H2" 0.04135203 "hypothetical protein"
"JS1_A09_00941" 11427 "s59E25H2" 0 "hypothetical protein"
"JS1_A09_00941" 11427 "s59E5H2" 0 "hypothetical protein"
"JS1_A09_00941" 11427 "s63E6H2" 0.003873303 "hypothetical protein"
"JS1_A09_00942" 11428 "s59E13H2" 0.003453717 "L-glyceraldehyde 3-phosphate reductase"
"JS1_A09_00942" 11428 "s59E25H2" 0 "L-glyceraldehyde 3-phosphate reductase"
"JS1_A09_00942" 11428 "s59E5H2" 0.05977976 "L-glyceraldehyde 3-phosphate reductase"
"JS1_A09_00942" 11428 "s63E6H2" 0.2663042 "L-glyceraldehyde 3-phosphate reductase"
"JS1_A09_00943" 11429 "s59E13H2" 0 "Bacterial extracellular solute-binding protein"
"JS1_A09_00943" 11429 "s59E25H2" 0 "Bacterial extracellular solute-binding protein"
"JS1_A09_00943" 11429 "s59E5H2" 0.1596893 "Bacterial extracellular solute-binding protein"
"JS1_A09_00943" 11429 "s63E6H2" 0.1655589 "Bacterial extracellular solute-binding protein"
"JS1_A09_00944" 11430 "s59E13H2" 0 "Trehalose/maltose transport system permease protein MalG"
"JS1_A09_00944" 11430 "s59E25H2" 0 "Trehalose/maltose transport system permease protein MalG"
"JS1_A09_00944" 11430 "s59E5H2" 0.04783462 "Trehalose/maltose transport system permease protein MalG"

"JS1_A09_00944" 11430 "s63E6H2" 0.177009 "Trehalose/maltose transport system permease protein MalG"
"JS1_A09_00946" 11432 "s59E13H2" 0.01167497 "Oligopeptide transport ATP-binding protein OppF"
"JS1_A09_00946" 11432 "s59E25H2" 0 "Oligopeptide transport ATP-binding protein OppF"
"JS1_A09_00946" 11432 "s59E5H2" 0.2816109 "Oligopeptide transport ATP-binding protein OppF"
"JS1_A09_00946" 11432 "s63E6H2" 0.09719669 "Oligopeptide transport ATP-binding protein OppF"
"JS1_A09_00947" 11433 "s59E13H2" 0 "Oligopeptide transport ATP-binding protein OppD"
"JS1_A09_00947" 11433 "s59E25H2" 0 "Oligopeptide transport ATP-binding protein OppD"
"JS1_A09_00947" 11433 "s59E5H2" 0.09565246 "Oligopeptide transport ATP-binding protein OppD"
"JS1_A09_00947" 11433 "s63E6H2" 0.002105303 "Oligopeptide transport ATP-binding protein OppD"
"JS1_A09_00948" 11434 "s59E13H2" 0 "putative D,D-dipeptide transport system permease protein DdpC"
"JS1_A09_00948" 11434 "s59E25H2" 0 "putative D,D-dipeptide transport system permease protein DdpC"
"JS1_A09_00948" 11434 "s59E5H2" 0.09938334 "putative D,D-dipeptide transport system permease protein DdpC"
"JS1_A09_00948" 11434 "s63E6H2" 0.0009676378 "putative D,D-dipeptide transport system permease protein DdpC"
"JS1_A09_00949" 11435 "s59E13H2" 0 "putative D,D-dipeptide transport system permease protein DdpB"
"JS1_A09_00949" 11435 "s59E25H2" 0 "putative D,D-dipeptide transport system permease protein DdpB"
"JS1_A09_00949" 11435 "s59E5H2" 0.02626552 "putative D,D-dipeptide transport system permease protein DdpB"
"JS1_A09_00949" 11435 "s63E6H2" 0.007870088 "putative D,D-dipeptide transport system permease protein DdpB"
"JS1_A09_00950" 11436 "s59E13H2" 0 "putative D,D-dipeptide-binding periplasmic protein DdpA precursor"
"JS1_A09_00950" 11436 "s59E25H2" 0 "putative D,D-dipeptide-binding periplasmic protein DdpA precursor"
"JS1_A09_00950" 11436 "s59E5H2" 0 "putative D,D-dipeptide-binding periplasmic protein DdpA precursor"
"JS1_A09_00950" 11436 "s63E6H2" 0.0001460827 "putative D,D-dipeptide-binding periplasmic protein DdpA precursor"
"JS1_A09_00951" 11437 "s59E13H2" 0 "2,5-dihydroxypyridine 5,6-dioxygenase"
"JS1_A09_00951" 11437 "s59E25H2" 0 "2,5-dihydroxypyridine 5,6-dioxygenase"
"JS1_A09_00951" 11437 "s59E5H2" 0.08624071 "2,5-dihydroxypyridine 5,6-dioxygenase"
"JS1_A09_00951" 11437 "s63E6H2" 0.00287028 "2,5-dihydroxypyridine 5,6-dioxygenase"
"JS1_A09_00952" 11438 "s59E13H2" 0 "Creatinine amidohydrolase"
"JS1_A09_00952" 11438 "s59E25H2" 0 "Creatinine amidohydrolase"
"JS1_A09_00952" 11438 "s59E5H2" 0.01925143 "Creatinine amidohydrolase"
"JS1_A09_00952" 11438 "s63E6H2" 0.007584212 "Creatinine amidohydrolase"
"JS1_A09_00953" 11439 "s59E13H2" 0 "2,5-dihydroxypyridine 5,6-dioxygenase"
"JS1_A09_00953" 11439 "s59E25H2" 0 "2,5-dihydroxypyridine 5,6-dioxygenase"
"JS1_A09_00953" 11439 "s59E5H2" 0.02378686 "2,5-dihydroxypyridine 5,6-dioxygenase"
"JS1_A09_00953" 11439 "s63E6H2" 0.306407 "2,5-dihydroxypyridine 5,6-dioxygenase"
"JS1_A09_00955" 11441 "s59E13H2" 0.00221092 "Asp/Glu/Hydantoin racemase"
"JS1_A09_00955" 11441 "s59E25H2" 0 "Asp/Glu/Hydantoin racemase"
"JS1_A09_00955" 11441 "s59E5H2" 0.004879481 "Asp/Glu/Hydantoin racemase"
"JS1_A09_00955" 11441 "s63E6H2" 0.156702 "Asp/Glu/Hydantoin racemase"
"JS1_A09_00956" 11442 "s59E13H2" 0.008139178 "Indolepyruvate oxidoreductase subunit IorB"
"JS1_A09_00956" 11442 "s59E25H2" 0 "Indolepyruvate oxidoreductase subunit IorB"
"JS1_A09_00956" 11442 "s59E5H2" 0 "Indolepyruvate oxidoreductase subunit IorB"
"JS1_A09_00956" 11442 "s63E6H2" 0 "Indolepyruvate oxidoreductase subunit IorB"
"JS1_A09_00959" 11445 "s59E13H2" 0.06447278 "hypothetical protein"
"JS1_A09_00959" 11445 "s59E25H2" 0 "hypothetical protein"
"JS1_A09_00959" 11445 "s59E5H2" 0.5153098 "hypothetical protein"
"JS1_A09_00959" 11445 "s63E6H2" 0.9360325 "hypothetical protein"
"JS1_A09_00960" 11446 "s59E13H2" 0 "CoB--CoM heterodisulfide reductase subunit B"
"JS1_A09_00960" 11446 "s59E25H2" 0 "CoB--CoM heterodisulfide reductase subunit B"

"JS1_A09_00960" 11446 "s59E5H2" 0 "CoB--CoM heterodisulfide reductase subunit B"
"JS1_A09_00960" 11446 "s63E6H2" 0 "CoB--CoM heterodisulfide reductase subunit B"
"JS1_A09_00961" 11447 "s59E13H2" 0 "CoB--CoM heterodisulfide reductase subunit B"
"JS1_A09_00961" 11447 "s59E25H2" 0 "CoB--CoM heterodisulfide reductase subunit B"
"JS1_A09_00961" 11447 "s59E5H2" 0 "CoB--CoM heterodisulfide reductase subunit B"
"JS1_A09_00961" 11447 "s63E6H2" 0 "CoB--CoM heterodisulfide reductase subunit B"
"JS1_A09_00962" 11448 "s59E13H2" 0.02649094 "Tyrosine recombinase XerD"
"JS1_A09_00962" 11448 "s59E25H2" 0 "Tyrosine recombinase XerD"
"JS1_A09_00962" 11448 "s59E5H2" 0.1157471 "Tyrosine recombinase XerD"
"JS1_A09_00962" 11448 "s63E6H2" 0.06809607 "Tyrosine recombinase XerD"
"JS1_A09_00963" 11449 "s59E13H2" 0.1197803 "50S ribosomal protein L31"
"JS1_A09_00963" 11449 "s59E25H2" 0 "50S ribosomal protein L31"
"JS1_A09_00963" 11449 "s59E5H2" 0.04578719 "50S ribosomal protein L31"
"JS1_A09_00963" 11449 "s63E6H2" 0.05312532 "50S ribosomal protein L31"
"JS1_A09_00964" 11450 "s59E13H2" 0.1761509 "hypothetical protein"
"JS1_A09_00964" 11450 "s59E25H2" 0 "hypothetical protein"
"JS1_A09_00964" 11450 "s59E5H2" 0.08909754 "hypothetical protein"
"JS1_A09_00964" 11450 "s63E6H2" 0.1508434 "hypothetical protein"
"JS1_A09_00965" 11451 "s59E13H2" 0.0004589745 "hypothetical protein"
"JS1_A09_00965" 11451 "s59E25H2" 0 "hypothetical protein"
"JS1_A09_00965" 11451 "s59E5H2" 0.0026027 "hypothetical protein"
"JS1_A09_00965" 11451 "s63E6H2" 0.005665732 "hypothetical protein"
"JS1_A09_00967" 11453 "s59E13H2" 0.007298332 "Lipoate-protein ligase A subunit 2"
"JS1_A09_00967" 11453 "s59E25H2" 0 "Lipoate-protein ligase A subunit 2"
"JS1_A09_00967" 11453 "s59E5H2" 0 "Lipoate-protein ligase A subunit 2"
"JS1_A09_00967" 11453 "s63E6H2" 0.02507487 "Lipoate-protein ligase A subunit 2"
"JS1_A09_00968" 11454 "s59E13H2" 0.1183529 "Lipoate-protein ligase LplJ"
"JS1_A09_00968" 11454 "s59E25H2" 0 "Lipoate-protein ligase LplJ"
"JS1_A09_00968" 11454 "s59E5H2" 0.0110843 "Lipoate-protein ligase LplJ"
"JS1_A09_00968" 11454 "s63E6H2" 0.1819734 "Lipoate-protein ligase LplJ"
"JS1_A09_00969" 11455 "s59E13H2" 0 "hypothetical protein"
"JS1_A09_00969" 11455 "s59E25H2" 0 "hypothetical protein"
"JS1_A09_00969" 11455 "s59E5H2" 0 "hypothetical protein"
"JS1_A09_00969" 11455 "s63E6H2" 0 "hypothetical protein"
"JS1_A09_00970" 11456 "s59E13H2" 0 "hypothetical protein"
"JS1_A09_00970" 11456 "s59E25H2" 0 "hypothetical protein"
"JS1_A09_00970" 11456 "s59E5H2" 0 "hypothetical protein"
"JS1_A09_00970" 11456 "s63E6H2" 0 "hypothetical protein"
"JS1_A09_00971" 11457 "s59E13H2" 0 "hypothetical protein"
"JS1_A09_00971" 11457 "s59E25H2" 0 "hypothetical protein"
"JS1_A09_00971" 11457 "s59E5H2" 0 "hypothetical protein"
"JS1_A09_00971" 11457 "s63E6H2" 0 "hypothetical protein"
"JS1_A09_00978" 11464 "s59E13H2" 0.01075151 "3-phosphoshikimate 1-carboxyvinyltransferase 1"
"JS1_A09_00978" 11464 "s59E25H2" 0 "3-phosphoshikimate 1-carboxyvinyltransferase 1"
"JS1_A09_00978" 11464 "s59E5H2" 0.7668904 "3-phosphoshikimate 1-carboxyvinyltransferase 1"
"JS1_A09_00978" 11464 "s63E6H2" 0.1465062 "3-phosphoshikimate 1-carboxyvinyltransferase 1"
"JS1_A09_00979" 11465 "s59E13H2" 0 "CoB--CoM heterodisulfide reductase subunit B"

"JS1_A09_00979" 11465 "s59E25H2" 0 "CoB--CoM heterodisulfide reductase subunit B"
"JS1_A09_00979" 11465 "s59E5H2" 0 "CoB--CoM heterodisulfide reductase subunit B"
"JS1_A09_00979" 11465 "s63E6H2" 0.002543519 "CoB--CoM heterodisulfide reductase subunit B"
"JS1_A09_00980" 11466 "s59E13H2" 0 "CoB--CoM heterodisulfide reductase iron-sulfur subunit A"
"JS1_A09_00980" 11466 "s59E25H2" 0 "CoB--CoM heterodisulfide reductase iron-sulfur subunit A"
"JS1_A09_00980" 11466 "s59E5H2" 0.003490898 "CoB--CoM heterodisulfide reductase iron-sulfur subunit A"
"JS1_A09_00980" 11466 "s63E6H2" 0 "CoB--CoM heterodisulfide reductase iron-sulfur subunit A"
"JS1_A09_00983" 11469 "s59E13H2" 0 "CoB--CoM heterodisulfide reductase iron-sulfur subunit A"
"JS1_A09_00983" 11469 "s59E25H2" 0 "CoB--CoM heterodisulfide reductase iron-sulfur subunit A"
"JS1_A09_00983" 11469 "s59E5H2" 0 "CoB--CoM heterodisulfide reductase iron-sulfur subunit A"
"JS1_A09_00983" 11469 "s63E6H2" 0 "CoB--CoM heterodisulfide reductase iron-sulfur subunit A"
"JS1_A09_00984" 11470 "s59E13H2" 0.01936194 "putative FAD-linked oxidoreductase"
"JS1_A09_00984" 11470 "s59E25H2" 0 "putative FAD-linked oxidoreductase"
"JS1_A09_00984" 11470 "s59E5H2" 0 "putative FAD-linked oxidoreductase"
"JS1_A09_00984" 11470 "s63E6H2" 0.003705839 "putative FAD-linked oxidoreductase"
"JS1_A09_00986" 11472 "s59E13H2" 0 "Succinyl-CoA ligase [ADP-forming] subunit alpha"
"JS1_A09_00986" 11472 "s59E25H2" 0 "Succinyl-CoA ligase [ADP-forming] subunit alpha"
"JS1_A09_00986" 11472 "s59E5H2" 0.01770057 "Succinyl-CoA ligase [ADP-forming] subunit alpha"
"JS1_A09_00986" 11472 "s63E6H2" 0.009222324 "Succinyl-CoA ligase [ADP-forming] subunit alpha"
"JS1_A09_00988" 11474 "s59E13H2" 0.01130284 "Putative sugar phosphate isomerase YwlF"
"JS1_A09_00988" 11474 "s59E25H2" 0 "Putative sugar phosphate isomerase YwlF"
"JS1_A09_00988" 11474 "s59E5H2" 0.02889591 "Putative sugar phosphate isomerase YwlF"
"JS1_A09_00988" 11474 "s63E6H2" 0.01422723 "Putative sugar phosphate isomerase YwlF"
"JS1_A09_00992" 11478 "s59E13H2" 0 "succinyl-CoA synthetase subunit beta"
"JS1_A09_00992" 11478 "s59E25H2" 0 "succinyl-CoA synthetase subunit beta"
"JS1_A09_00992" 11478 "s59E5H2" 0 "succinyl-CoA synthetase subunit beta"
"JS1_A09_00992" 11478 "s63E6H2" 0 "succinyl-CoA synthetase subunit beta"
"JS1_A09_00993" 11479 "s59E13H2" 0 "Succinyl-CoA ligase [ADP-forming] subunit beta"
"JS1_A09_00993" 11479 "s59E25H2" 0 "Succinyl-CoA ligase [ADP-forming] subunit beta"
"JS1_A09_00993" 11479 "s59E5H2" 0 "Succinyl-CoA ligase [ADP-forming] subunit beta"
"JS1_A09_00993" 11479 "s63E6H2" 0 "Succinyl-CoA ligase [ADP-forming] subunit beta"
"JS1_A09_00996" 11482 "s59E13H2" 0 "CoB--CoM heterodisulfide reductase iron-sulfur subunit A"
"JS1_A09_00996" 11482 "s59E25H2" 0 "CoB--CoM heterodisulfide reductase iron-sulfur subunit A"
"JS1_A09_00996" 11482 "s59E5H2" 0.009358947 "CoB--CoM heterodisulfide reductase iron-sulfur subunit A"
"JS1_A09_00996" 11482 "s63E6H2" 0 "CoB--CoM heterodisulfide reductase iron-sulfur subunit A"
"JS1_A09_00997" 11483 "s59E13H2" 0.0004425485 "Sulfide dehydrogenase subunit alpha precursor"
"JS1_A09_00997" 11483 "s59E25H2" 0 "Sulfide dehydrogenase subunit alpha precursor"
"JS1_A09_00997" 11483 "s59E5H2" 0.01464604 "Sulfide dehydrogenase subunit alpha precursor"
"JS1_A09_00997" 11483 "s63E6H2" 0.3613055 "Sulfide dehydrogenase subunit alpha precursor"
"JS1_A09_00998" 11484 "s59E13H2" 0 "L-aspartate oxidase"
"JS1_A09_00998" 11484 "s59E25H2" 0 "L-aspartate oxidase"
"JS1_A09_00998" 11484 "s59E5H2" 0.003255953 "L-aspartate oxidase"
"JS1_A09_00998" 11484 "s63E6H2" 0.4508296 "L-aspartate oxidase"
"JS1_A09_00999" 11485 "s59E13H2" 0 "acetyl-CoA decarbonylase/synthase complex subunit alpha"
"JS1_A09_00999" 11485 "s59E25H2" 0 "acetyl-CoA decarbonylase/synthase complex subunit alpha"
"JS1_A09_00999" 11485 "s59E5H2" 0.0110139 "acetyl-CoA decarbonylase/synthase complex subunit alpha"
"JS1_A09_00999" 11485 "s63E6H2" 0.520346 "acetyl-CoA decarbonylase/synthase complex subunit alpha"

"JS1_A09_01000" 11486 "s59E13H2" 0.002455491 "CoB--CoM heterodisulfide reductase iron-sulfur subunit A"
"JS1_A09_01000" 11486 "s59E25H2" 0 "CoB--CoM heterodisulfide reductase iron-sulfur subunit A"
"JS1_A09_01000" 11486 "s59E5H2" 0.005586321 "CoB--CoM heterodisulfide reductase iron-sulfur subunit A"
"JS1_A09_01000" 11486 "s63E6H2" 0.4100314 "CoB--CoM heterodisulfide reductase iron-sulfur subunit A"
"JS1_A09_01001" 11487 "s59E13H2" 0 "Asp/Glu/Hydantoin racemase"
"JS1_A09_01001" 11487 "s59E25H2" 0 "Asp/Glu/Hydantoin racemase"
"JS1_A09_01001" 11487 "s59E5H2" 0 "Asp/Glu/Hydantoin racemase"
"JS1_A09_01001" 11487 "s63E6H2" 0.00407884 "Asp/Glu/Hydantoin racemase"
"JS1_A09_01002" 11488 "s59E13H2" 0 "Enamine/imine deaminase"
"JS1_A09_01002" 11488 "s59E25H2" 0 "Enamine/imine deaminase"
"JS1_A09_01002" 11488 "s59E5H2" 0 "Enamine/imine deaminase"
"JS1_A09_01002" 11488 "s63E6H2" 0.04349755 "Enamine/imine deaminase"
"JS1_A09_01003" 11489 "s59E13H2" 0.003753812 "L-hydantoinase"
"JS1_A09_01003" 11489 "s59E25H2" 0 "L-hydantoinase"
"JS1_A09_01003" 11489 "s59E5H2" 0.01152133 "L-hydantoinase"
"JS1_A09_01003" 11489 "s63E6H2" 0.07134173 "L-hydantoinase"
"JS1_A09_01004" 11490 "s59E13H2" 0.01907776 "Phosphoenolpyruvate carboxykinase [GTP]"
"JS1_A09_01004" 11490 "s59E25H2" 0 "Phosphoenolpyruvate carboxykinase [GTP]"
"JS1_A09_01004" 11490 "s59E5H2" 0.001736657 "Phosphoenolpyruvate carboxykinase [GTP]"
"JS1_A09_01004" 11490 "s63E6H2" 0.03476666 "Phosphoenolpyruvate carboxykinase [GTP]"
"JS1_A09_01005" 11491 "s59E13H2" 1.372698 "N(G),N(G)-dimethylarginine dimethylaminohydrolase"
"JS1_A09_01005" 11491 "s59E25H2" 0 "N(G),N(G)-dimethylarginine dimethylaminohydrolase"
"JS1_A09_01005" 11491 "s59E5H2" 0.07849445 "N(G),N(G)-dimethylarginine dimethylaminohydrolase"
"JS1_A09_01005" 11491 "s63E6H2" 0.09149132 "N(G),N(G)-dimethylarginine dimethylaminohydrolase"
"JS1_A09_01006" 11492 "s59E13H2" 0.4131903 "HTH-type transcriptional regulator LutR"
"JS1_A09_01006" 11492 "s59E25H2" 0 "HTH-type transcriptional regulator LutR"
"JS1_A09_01006" 11492 "s59E5H2" 0.05366481 "HTH-type transcriptional regulator LutR"
"JS1_A09_01006" 11492 "s63E6H2" 0.08647855 "HTH-type transcriptional regulator LutR"
"JS1_A09_01007" 11493 "s59E13H2" 3.747964 "hypothetical protein"
"JS1_A09_01007" 11493 "s59E25H2" 0.3679176 "hypothetical protein"
"JS1_A09_01007" 11493 "s59E5H2" 0.2052055 "hypothetical protein"
"JS1_A09_01007" 11493 "s63E6H2" 0.2639727 "hypothetical protein"
"JS1_A09_01008" 11494 "s59E13H2" 1.919322 "hypothetical protein"
"JS1_A09_01008" 11494 "s59E25H2" 0 "hypothetical protein"
"JS1_A09_01008" 11494 "s59E5H2" 0.1917897 "hypothetical protein"
"JS1_A09_01008" 11494 "s63E6H2" 0.1995112 "hypothetical protein"
"JS1_A09_01009" 11495 "s59E13H2" 1.602223 "Dihydroneopterin 2',3'-cyclic phosphate phosphodiesterase"
"JS1_A09_01009" 11495 "s59E25H2" 0 "Dihydroneopterin 2',3'-cyclic phosphate phosphodiesterase"
"JS1_A09_01009" 11495 "s59E5H2" 0.2796906 "Dihydroneopterin 2',3'-cyclic phosphate phosphodiesterase"
"JS1_A09_01009" 11495 "s63E6H2" 0.1987275 "Dihydroneopterin 2',3'-cyclic phosphate phosphodiesterase"
"JS1_A09_01010" 11496 "s59E13H2" 0.9657287 "Isopropylmalate/citramalate isomerase large subunit"
"JS1_A09_01010" 11496 "s59E25H2" 0 "Isopropylmalate/citramalate isomerase large subunit"
"JS1_A09_01010" 11496 "s59E5H2" 0.1072136 "Isopropylmalate/citramalate isomerase large subunit"
"JS1_A09_01010" 11496 "s63E6H2" 0.06545057 "Isopropylmalate/citramalate isomerase large subunit"
"JS1_A09_01011" 11497 "s59E13H2" 0.1010378 "Beta-galactosidase"
"JS1_A09_01011" 11497 "s59E25H2" 0 "Beta-galactosidase"
"JS1_A09_01011" 11497 "s59E5H2" 0.4792442 "Beta-galactosidase"

"JS1_A09_01011" 11497 "s63E6H2" 0.2184915 "Beta-galactosidase"
"JS1_A09_01012" 11498 "s59E13H2" 0.153101 "Alpha-galactosidase"
"JS1_A09_01012" 11498 "s59E25H2" 0 "Alpha-galactosidase"
"JS1_A09_01012" 11498 "s59E5H2" 0.5862259 "Alpha-galactosidase"
"JS1_A09_01012" 11498 "s63E6H2" 0.1128897 "Alpha-galactosidase"
"JS1_A09_01013" 11499 "s59E13H2" 0.2745 "Galactose-1-phosphate uridylyltransferase"
"JS1_A09_01013" 11499 "s59E25H2" 0 "Galactose-1-phosphate uridylyltransferase"
"JS1_A09_01013" 11499 "s59E5H2" 0.4468707 "Galactose-1-phosphate uridylyltransferase"
"JS1_A09_01013" 11499 "s63E6H2" 0.636606 "Galactose-1-phosphate uridylyltransferase"
"JS1_A09_01014" 11500 "s59E13H2" 0.04743254 "Galactokinase"
"JS1_A09_01014" 11500 "s59E25H2" 0 "Galactokinase"
"JS1_A09_01014" 11500 "s59E5H2" 0.101203 "Galactokinase"
"JS1_A09_01014" 11500 "s63E6H2" 0.05681738 "Galactokinase"
"JS1_A09_01015" 11501 "s59E13H2" 1.923945 "Glycerol kinase"
"JS1_A09_01015" 11501 "s59E25H2" 0.2573333 "Glycerol kinase"
"JS1_A09_01015" 11501 "s59E5H2" 1.543216 "Glycerol kinase"
"JS1_A09_01015" 11501 "s63E6H2" 2.865339 "Glycerol kinase"
"JS1_A09_01016" 11502 "s59E13H2" 0.1716304 "HTH-type transcriptional regulator EthR"
"JS1_A09_01016" 11502 "s59E25H2" 0 "HTH-type transcriptional regulator EthR"
"JS1_A09_01016" 11502 "s59E5H2" 1.04607 "HTH-type transcriptional regulator EthR"
"JS1_A09_01016" 11502 "s63E6H2" 1.160627 "HTH-type transcriptional regulator EthR"
"JS1_A09_01017" 11503 "s59E13H2" 0.1935494 "hypothetical protein"
"JS1_A09_01017" 11503 "s59E25H2" 0 "hypothetical protein"
"JS1_A09_01017" 11503 "s59E5H2" 0.8447982 "hypothetical protein"
"JS1_A09_01017" 11503 "s63E6H2" 0.59509 "hypothetical protein"
"JS1_A09_01018" 11504 "s59E13H2" 0.07894509 "hypothetical protein"
"JS1_A09_01018" 11504 "s59E25H2" 0 "hypothetical protein"
"JS1_A09_01018" 11504 "s59E5H2" 0.4374693 "hypothetical protein"
"JS1_A09_01018" 11504 "s63E6H2" 1.4169 "hypothetical protein"
"JS1_A09_01019" 11505 "s59E13H2" 0 "Membrane transport protein mmpL8"
"JS1_A09_01019" 11505 "s59E25H2" 0 "Membrane transport protein mmpL8"
"JS1_A09_01019" 11505 "s59E5H2" 0.1332344 "Membrane transport protein mmpL8"
"JS1_A09_01019" 11505 "s63E6H2" 0.0227589 "Membrane transport protein mmpL8"
"JS1_A09_01021" 11507 "s59E13H2" 0.04288093 "UDP-N-acetylglucosamine 2-epimerase"
"JS1_A09_01021" 11507 "s59E25H2" 0 "UDP-N-acetylglucosamine 2-epimerase"
"JS1_A09_01021" 11507 "s59E5H2" 1.659832 "UDP-N-acetylglucosamine 2-epimerase"
"JS1_A09_01021" 11507 "s63E6H2" 0.08667404 "UDP-N-acetylglucosamine 2-epimerase"
"JS1_A09_01022" 11508 "s59E13H2" 0.01634307 "Rhomboid protease GluP"
"JS1_A09_01022" 11508 "s59E25H2" 0 "Rhomboid protease GluP"
"JS1_A09_01022" 11508 "s59E5H2" 0.6620872 "Rhomboid protease GluP"
"JS1_A09_01022" 11508 "s63E6H2" 0.1461011 "Rhomboid protease GluP"
"JS1_A09_01023" 11509 "s59E13H2" 0.03058075 "Tagatose-6-phosphate kinase"
"JS1_A09_01023" 11509 "s59E25H2" 0 "Tagatose-6-phosphate kinase"
"JS1_A09_01023" 11509 "s59E5H2" 0.8589959 "Tagatose-6-phosphate kinase"
"JS1_A09_01023" 11509 "s63E6H2" 0.08753395 "Tagatose-6-phosphate kinase"
"JS1_A09_01024" 11510 "s59E13H2" 0.0228168 "inosine 5'-monophosphate dehydrogenase"
"JS1_A09_01024" 11510 "s59E25H2" 0 "inosine 5'-monophosphate dehydrogenase"

"JS1_A09_01024" 11510 "s59E5H2" 0.7421967 "inosine 5'-monophosphate dehydrogenase"
"JS1_A09_01024" 11510 "s63E6H2" 0.1081926 "inosine 5'-monophosphate dehydrogenase"
"JS1_A09_01025" 11511 "s59E13H2" 0.03867016 "Thymidylate kinase"
"JS1_A09_01025" 11511 "s59E25H2" 0 "Thymidylate kinase"
"JS1_A09_01025" 11511 "s59E5H2" 0.7478915 "Thymidylate kinase"
"JS1_A09_01025" 11511 "s63E6H2" 0.09351836 "Thymidylate kinase"
"JS1_A09_01026" 11512 "s59E13H2" 0.04661903 "DNA polymerase III subunit tau"
"JS1_A09_01026" 11512 "s59E25H2" 0 "DNA polymerase III subunit tau"
"JS1_A09_01026" 11512 "s59E5H2" 0.4226309 "DNA polymerase III subunit tau"
"JS1_A09_01026" 11512 "s63E6H2" 0.07144371 "DNA polymerase III subunit tau"
"JS1_A09_01027" 11513 "s59E13H2" 0.05105581 "hypothetical protein"
"JS1_A09_01027" 11513 "s59E25H2" 0 "hypothetical protein"
"JS1_A09_01027" 11513 "s59E5H2" 0.2634266 "hypothetical protein"
"JS1_A09_01027" 11513 "s63E6H2" 0.1028851 "hypothetical protein"
"JS1_A09_01028" 11514 "s59E13H2" 0.05322603 "Tetracycline resistance protein, class B"
"JS1_A09_01028" 11514 "s59E25H2" 0 "Tetracycline resistance protein, class B"
"JS1_A09_01028" 11514 "s59E5H2" 0.5113034 "Tetracycline resistance protein, class B"
"JS1_A09_01028" 11514 "s63E6H2" 0.2169156 "Tetracycline resistance protein, class B"
"JS1_A09_01029" 11515 "s59E13H2" 0.006118345 "hypothetical protein"
"JS1_A09_01029" 11515 "s59E25H2" 0 "hypothetical protein"
"JS1_A09_01029" 11515 "s59E5H2" 0.02621897 "hypothetical protein"
"JS1_A09_01029" 11515 "s63E6H2" 0.006979925 "hypothetical protein"
"JS1_A09_01030" 11516 "s59E13H2" 0 "hypothetical protein"
"JS1_A09_01030" 11516 "s59E25H2" 0 "hypothetical protein"
"JS1_A09_01030" 11516 "s59E5H2" 0.02496807 "hypothetical protein"
"JS1_A09_01030" 11516 "s63E6H2" 0.05710592 "hypothetical protein"
"JS1_A09_01031" 11517 "s59E13H2" 0 "Putative N-acetyl-LL-diaminopimelate aminotransferase"
"JS1_A09_01031" 11517 "s59E25H2" 0 "Putative N-acetyl-LL-diaminopimelate aminotransferase"
"JS1_A09_01031" 11517 "s59E5H2" 0 "Putative N-acetyl-LL-diaminopimelate aminotransferase"
"JS1_A09_01031" 11517 "s63E6H2" 0.002683501 "Putative N-acetyl-LL-diaminopimelate aminotransferase"
"JS1_A09_01032" 11518 "s59E13H2" 0 "hypothetical protein"
"JS1_A09_01032" 11518 "s59E25H2" 0 "hypothetical protein"
"JS1_A09_01032" 11518 "s59E5H2" 0 "hypothetical protein"
"JS1_A09_01032" 11518 "s63E6H2" 0.04953603 "hypothetical protein"
"JS1_A09_01035" 11521 "s59E13H2" 0 "2-oxoglutarate synthase subunit KorA"
"JS1_A09_01035" 11521 "s59E25H2" 0 "2-oxoglutarate synthase subunit KorA"
"JS1_A09_01035" 11521 "s59E5H2" 0.0002526504 "2-oxoglutarate synthase subunit KorA"
"JS1_A09_01035" 11521 "s63E6H2" 0.02506457 "2-oxoglutarate synthase subunit KorA"
"JS1_A09_01036" 11522 "s59E13H2" 0 "Succinyl-CoA ligase [ADP-forming] subunit beta"
"JS1_A09_01036" 11522 "s59E25H2" 0 "Succinyl-CoA ligase [ADP-forming] subunit beta"
"JS1_A09_01036" 11522 "s59E5H2" 0 "Succinyl-CoA ligase [ADP-forming] subunit beta"
"JS1_A09_01036" 11522 "s63E6H2" 0 "Succinyl-CoA ligase [ADP-forming] subunit beta"
"JS1_A09_01039" 11525 "s59E13H2" 0 "hypothetical protein"
"JS1_A09_01039" 11525 "s59E25H2" 0 "hypothetical protein"
"JS1_A09_01039" 11525 "s59E5H2" 0 "hypothetical protein"
"JS1_A09_01039" 11525 "s63E6H2" 0 "hypothetical protein"
"JS1_A09_01040" 11526 "s59E13H2" 0 "hypothetical protein"

"JS1_A09_01040" 11526 "s59E25H2" 0 "hypothetical protein"
"JS1_A09_01040" 11526 "s59E5H2" 0 "hypothetical protein"
"JS1_A09_01040" 11526 "s63E6H2" 0 "hypothetical protein"
"JS1_A09_01041" 11527 "s59E13H2" 1.595446 "Group II intron-encoded protein LtrA"
"JS1_A09_01041" 11527 "s59E25H2" 0 "Group II intron-encoded protein LtrA"
"JS1_A09_01041" 11527 "s59E5H2" 1.907204 "Group II intron-encoded protein LtrA"
"JS1_A09_01041" 11527 "s63E6H2" 12.62315 "Group II intron-encoded protein LtrA"
"JS1_A09_01045" 11531 "s59E13H2" 1.204358 "hypothetical protein"
"JS1_A09_01045" 11531 "s59E25H2" 0 "hypothetical protein"
"JS1_A09_01045" 11531 "s59E5H2" 1.064709 "hypothetical protein"
"JS1_A09_01045" 11531 "s63E6H2" 0.06535669 "hypothetical protein"
"JS1_A09_01046" 11532 "s59E13H2" 0.709776 "hypothetical protein"
"JS1_A09_01046" 11532 "s59E25H2" 0 "hypothetical protein"
"JS1_A09_01046" 11532 "s59E5H2" 1.189656 "hypothetical protein"
"JS1_A09_01046" 11532 "s63E6H2" 0.1050916 "hypothetical protein"
"JS1_A09_01047" 11533 "s59E13H2" 0.6193994 "hypothetical protein"
"JS1_A09_01047" 11533 "s59E25H2" 0 "hypothetical protein"
"JS1_A09_01047" 11533 "s59E5H2" 2.446704 "hypothetical protein"
"JS1_A09_01047" 11533 "s63E6H2" 0.1372004 "hypothetical protein"
"JS1_A09_01048" 11534 "s59E13H2" 0.01530855 "putative DMT superfamily transporter inner membrane protein"
"JS1_A09_01048" 11534 "s59E25H2" 0 "putative DMT superfamily transporter inner membrane protein"
"JS1_A09_01048" 11534 "s59E5H2" 1.684819 "putative DMT superfamily transporter inner membrane protein"
"JS1_A09_01048" 11534 "s63E6H2" 0.08398426 "putative DMT superfamily transporter inner membrane protein"
"JS1_A09_01049" 11535 "s59E13H2" 0.1065035 "Outer membrane efflux protein"
"JS1_A09_01049" 11535 "s59E25H2" 0 "Outer membrane efflux protein"
"JS1_A09_01049" 11535 "s59E5H2" 1.529609 "Outer membrane efflux protein"
"JS1_A09_01049" 11535 "s63E6H2" 0.08347985 "Outer membrane efflux protein"
"JS1_A09_01050" 11536 "s59E13H2" 0.2963791 "Outer membrane efflux protein"
"JS1_A09_01050" 11536 "s59E25H2" 0 "Outer membrane efflux protein"
"JS1_A09_01050" 11536 "s59E5H2" 0.3091841 "Outer membrane efflux protein"
"JS1_A09_01050" 11536 "s63E6H2" 0.08865212 "Outer membrane efflux protein"
"JS1_A09_01051" 11537 "s59E13H2" 0.1774476 "Putative efflux system component YknX"
"JS1_A09_01051" 11537 "s59E25H2" 0 "Putative efflux system component YknX"
"JS1_A09_01051" 11537 "s59E5H2" 0.5739481 "Putative efflux system component YknX"
"JS1_A09_01051" 11537 "s63E6H2" 0.5928006 "Putative efflux system component YknX"
"JS1_A09_01052" 11538 "s59E13H2" 0.1313663 "putative ABC transporter ATP-binding protein"
"JS1_A09_01052" 11538 "s59E25H2" 0 "putative ABC transporter ATP-binding protein"
"JS1_A09_01052" 11538 "s59E5H2" 0.5149736 "putative ABC transporter ATP-binding protein"
"JS1_A09_01052" 11538 "s63E6H2" 1.347397 "putative ABC transporter ATP-binding protein"
"JS1_A09_01053" 11539 "s59E13H2" 0.2172109 "Macrolide export ATP-binding/permease protein MacB"
"JS1_A09_01053" 11539 "s59E25H2" 0.1978654 "Macrolide export ATP-binding/permease protein MacB"
"JS1_A09_01053" 11539 "s59E5H2" 0.9026395 "Macrolide export ATP-binding/permease protein MacB"
"JS1_A09_01053" 11539 "s63E6H2" 0.8805337 "Macrolide export ATP-binding/permease protein MacB"
"JS1_A09_01054" 11540 "s59E13H2" 0.004236945 "Trifunctional nucleotide phosphoesterase protein YfkN precursor"
"JS1_A09_01054" 11540 "s59E25H2" 0 "Trifunctional nucleotide phosphoesterase protein YfkN precursor"
"JS1_A09_01054" 11540 "s59E5H2" 0.1415342 "Trifunctional nucleotide phosphoesterase protein YfkN precursor"
"JS1_A09_01054" 11540 "s63E6H2" 0.2287555 "Trifunctional nucleotide phosphoesterase protein YfkN precursor"

"JS1_A09_01055" 11541 "s59E13H2" 0.0644627 "hypothetical protein"
"JS1_A09_01055" 11541 "s59E25H2" 0 "hypothetical protein"
"JS1_A09_01055" 11541 "s59E5H2" 0.4183945 "hypothetical protein"
"JS1_A09_01055" 11541 "s63E6H2" 1.566928 "hypothetical protein"
"JS1_A09_01056" 11542 "s59E13H2" 0.07397523 "LemA family protein"
"JS1_A09_01056" 11542 "s59E25H2" 0 "LemA family protein"
"JS1_A09_01056" 11542 "s59E5H2" 1.055763 "LemA family protein"
"JS1_A09_01056" 11542 "s63E6H2" 1.867797 "LemA family protein"
"JS1_A09_01057" 11543 "s59E13H2" 0.03536993 "galactoside permease"
"JS1_A09_01057" 11543 "s59E25H2" 0 "galactoside permease"
"JS1_A09_01057" 11543 "s59E5H2" 0.6437425 "galactoside permease"
"JS1_A09_01057" 11543 "s63E6H2" 1.098703 "galactoside permease"
"JS1_A09_01064" 11550 "s59E13H2" 0 "hypothetical protein"
"JS1_A09_01064" 11550 "s59E25H2" 0 "hypothetical protein"
"JS1_A09_01064" 11550 "s59E5H2" 0 "hypothetical protein"
"JS1_A09_01064" 11550 "s63E6H2" 0 "hypothetical protein"
"JS1_A09_01066" 11552 "s59E13H2" 0 "hypothetical protein"
"JS1_A09_01066" 11552 "s59E25H2" 0 "hypothetical protein"
"JS1_A09_01066" 11552 "s59E5H2" 0 "hypothetical protein"
"JS1_A09_01066" 11552 "s63E6H2" 0 "hypothetical protein"
"JS1_A09_01069" 11555 "s59E13H2" 0 "hypothetical protein"
"JS1_A09_01069" 11555 "s59E25H2" 0 "hypothetical protein"
"JS1_A09_01069" 11555 "s59E5H2" 0 "hypothetical protein"
"JS1_A09_01069" 11555 "s63E6H2" 0 "hypothetical protein"
"JS1_A09_01073" 11559 "s59E13H2" 0 "hypothetical protein"
"JS1_A09_01073" 11559 "s59E25H2" 0 "hypothetical protein"
"JS1_A09_01073" 11559 "s59E5H2" 0 "hypothetical protein"
"JS1_A09_01073" 11559 "s63E6H2" 0 "hypothetical protein"
"JS1_A09_01074" 11560 "s59E13H2" 0 "hypothetical protein"
"JS1_A09_01074" 11560 "s59E25H2" 0 "hypothetical protein"
"JS1_A09_01074" 11560 "s59E5H2" 0 "hypothetical protein"
"JS1_A09_01074" 11560 "s63E6H2" 0 "hypothetical protein"
"JS1_A09_01075" 11561 "s59E13H2" 0 "Alanine dehydrogenase"
"JS1_A09_01075" 11561 "s59E25H2" 0 "Alanine dehydrogenase"
"JS1_A09_01075" 11561 "s59E5H2" 0 "Alanine dehydrogenase"
"JS1_A09_01075" 11561 "s63E6H2" 0 "Alanine dehydrogenase"
"JS1_A09_01076" 11562 "s59E13H2" 0 "L-Ala-D/L-Glu epimerase"
"JS1_A09_01076" 11562 "s59E25H2" 0 "L-Ala-D/L-Glu epimerase"
"JS1_A09_01076" 11562 "s59E5H2" 0 "L-Ala-D/L-Glu epimerase"
"JS1_A09_01076" 11562 "s63E6H2" 0 "L-Ala-D/L-Glu epimerase"
"JS1_A09_01078" 11564 "s59E13H2" 0 "hypothetical protein"
"JS1_A09_01078" 11564 "s59E25H2" 0 "hypothetical protein"
"JS1_A09_01078" 11564 "s59E5H2" 0 "hypothetical protein"
"JS1_A09_01078" 11564 "s63E6H2" 0.007822615 "hypothetical protein"
"JS1_A09_01079" 11565 "s59E13H2" 0.09515843 "putative tyrosine recombinase XerC-like protein"
"JS1_A09_01079" 11565 "s59E25H2" 0 "putative tyrosine recombinase XerC-like protein"
"JS1_A09_01079" 11565 "s59E5H2" 0 "putative tyrosine recombinase XerC-like protein"

"JS1_A09_01079" 11565 "s63E6H2" 0.009974059 "putative tyrosine recombinase XerC-like protein"
"JS1_A09_01080" 11566 "s59E13H2" 0 "hypothetical protein"
"JS1_A09_01080" 11566 "s59E25H2" 0 "hypothetical protein"
"JS1_A09_01080" 11566 "s59E5H2" 0 "hypothetical protein"
"JS1_A09_01080" 11566 "s63E6H2" 0 "hypothetical protein"
"JS1_A09_01081" 11567 "s59E13H2" 0 "hypothetical protein"
"JS1_A09_01081" 11567 "s59E25H2" 0 "hypothetical protein"
"JS1_A09_01081" 11567 "s59E5H2" 0 "hypothetical protein"
"JS1_A09_01081" 11567 "s63E6H2" 0 "hypothetical protein"
"JS1_A09_01082" 11568 "s59E13H2" 0 "hypothetical protein"
"JS1_A09_01082" 11568 "s59E25H2" 0 "hypothetical protein"
"JS1_A09_01082" 11568 "s59E5H2" 0 "hypothetical protein"
"JS1_A09_01082" 11568 "s63E6H2" 0 "hypothetical protein"
"JS1_A09_01083" 11569 "s59E13H2" 0.0363145 "Restriction endonuclease BglII"
"JS1_A09_01083" 11569 "s59E25H2" 0 "Restriction endonuclease BglII"
"JS1_A09_01083" 11569 "s59E5H2" 0 "Restriction endonuclease BglII"
"JS1_A09_01083" 11569 "s63E6H2" 0 "Restriction endonuclease BglII"
"JS1_A09_01084" 11570 "s59E13H2" 0 "Tetratricopeptide repeat protein"
"JS1_A09_01084" 11570 "s59E25H2" 0 "Tetratricopeptide repeat protein"
"JS1_A09_01084" 11570 "s59E5H2" 0 "Tetratricopeptide repeat protein"
"JS1_A09_01084" 11570 "s63E6H2" 0 "Tetratricopeptide repeat protein"
"JS1_A09_01085" 11571 "s59E13H2" 0 "hypothetical protein"
"JS1_A09_01085" 11571 "s59E25H2" 0 "hypothetical protein"
"JS1_A09_01085" 11571 "s59E5H2" 0 "hypothetical protein"
"JS1_A09_01085" 11571 "s63E6H2" 0 "hypothetical protein"
"JS1_A09_01086" 11572 "s59E13H2" 0 "hypothetical protein"
"JS1_A09_01086" 11572 "s59E25H2" 0 "hypothetical protein"
"JS1_A09_01086" 11572 "s59E5H2" 0 "hypothetical protein"
"JS1_A09_01086" 11572 "s63E6H2" 0 "hypothetical protein"
"JS1_A09_01087" 11573 "s59E13H2" 0 "hypothetical protein"
"JS1_A09_01087" 11573 "s59E25H2" 0 "hypothetical protein"
"JS1_A09_01087" 11573 "s59E5H2" 0 "hypothetical protein"
"JS1_A09_01087" 11573 "s63E6H2" 0 "hypothetical protein"
"JS1_A09_01089" 11575 "s59E13H2" 0.02574012 "Transcription elongation factor GreA"
"JS1_A09_01089" 11575 "s59E25H2" 0 "Transcription elongation factor GreA"
"JS1_A09_01089" 11575 "s59E5H2" 0.1491755 "Transcription elongation factor GreA"
"JS1_A09_01089" 11575 "s63E6H2" 0.06426493 "Transcription elongation factor GreA"
"JS1_A09_01090" 11576 "s59E13H2" 0.03121518 "Threonylcarbamoyl-AMP synthase"
"JS1_A09_01090" 11576 "s59E25H2" 0 "Threonylcarbamoyl-AMP synthase"
"JS1_A09_01090" 11576 "s59E5H2" 0.1535281 "Threonylcarbamoyl-AMP synthase"
"JS1_A09_01090" 11576 "s63E6H2" 0.08013609 "Threonylcarbamoyl-AMP synthase"
"JS1_A09_01091" 11577 "s59E13H2" 0 "Chorismate mutase AroH"
"JS1_A09_01091" 11577 "s59E25H2" 0 "Chorismate mutase AroH"
"JS1_A09_01091" 11577 "s59E5H2" 0 "Chorismate mutase AroH"
"JS1_A09_01091" 11577 "s63E6H2" 0 "Chorismate mutase AroH"
"JS1_A09_01092" 11578 "s59E13H2" 0.01604867 "Putative pyridoxal phosphate-dependent acyltransferase"
"JS1_A09_01092" 11578 "s59E25H2" 0 "Putative pyridoxal phosphate-dependent acyltransferase"

"JS1_A09_01092" 11578 "s59E5H2" 0.1476207 "Putative pyridoxal phosphate-dependent acyltransferase"
"JS1_A09_01092" 11578 "s63E6H2" 0.1958134 "Putative pyridoxal phosphate-dependent acyltransferase"
"JS1_A09_01094" 11580 "s59E13H2" 0.01767383 "hypothetical protein"
"JS1_A09_01094" 11580 "s59E25H2" 0 "hypothetical protein"
"JS1_A09_01094" 11580 "s59E5H2" 0.3005192 "hypothetical protein"
"JS1_A09_01094" 11580 "s63E6H2" 0.08996191 "hypothetical protein"
"JS1_A09_01095" 11581 "s59E13H2" 0.07545824 "tRNA-specific 2-thiouridylase MnmA"
"JS1_A09_01095" 11581 "s59E25H2" 0 "tRNA-specific 2-thiouridylase MnmA"
"JS1_A09_01095" 11581 "s59E5H2" 0.3791847 "tRNA-specific 2-thiouridylase MnmA"
"JS1_A09_01095" 11581 "s63E6H2" 0.1152112 "tRNA-specific 2-thiouridylase MnmA"
"JS1_A09_01096" 11582 "s59E13H2" 0.03544238 "hypothetical protein"
"JS1_A09_01096" 11582 "s59E25H2" 0 "hypothetical protein"
"JS1_A09_01096" 11582 "s59E5H2" 0.1638756 "hypothetical protein"
"JS1_A09_01096" 11582 "s63E6H2" 0.08205529 "hypothetical protein"
"JS1_A09_01097" 11583 "s59E13H2" 0.03635338 "HTH-type transcriptional regulator CymR"
"JS1_A09_01097" 11583 "s59E25H2" 0 "HTH-type transcriptional regulator CymR"
"JS1_A09_01097" 11583 "s59E5H2" 0.1473259 "HTH-type transcriptional regulator CymR"
"JS1_A09_01097" 11583 "s63E6H2" 0.09817793 "HTH-type transcriptional regulator CymR"
"JS1_A09_01098" 11584 "s59E13H2" 0.3687576 "Oligo-1,6-glucosidase"
"JS1_A09_01098" 11584 "s59E25H2" 0 "Oligo-1,6-glucosidase"
"JS1_A09_01098" 11584 "s59E5H2" 0.7604925 "Oligo-1,6-glucosidase"
"JS1_A09_01098" 11584 "s63E6H2" 1.181628 "Oligo-1,6-glucosidase"
"JS1_A09_01099" 11585 "s59E13H2" 0.06226377 "Ferredoxin"
"JS1_A09_01099" 11585 "s59E25H2" 0 "Ferredoxin"
"JS1_A09_01099" 11585 "s59E5H2" 0.5502401 "Ferredoxin"
"JS1_A09_01099" 11585 "s63E6H2" 0.1209648 "Ferredoxin"
"JS1_A09_01100" 11586 "s59E13H2" 0.2107662 "Ribonuclease Z"
"JS1_A09_01100" 11586 "s59E25H2" 0 "Ribonuclease Z"
"JS1_A09_01100" 11586 "s59E5H2" 0.4007445 "Ribonuclease Z"
"JS1_A09_01100" 11586 "s63E6H2" 0.2308014 "Ribonuclease Z"
"JS1_A09_01101" 11587 "s59E13H2" 0.2334582 "Molybdopterin adenylyltransferase"
"JS1_A09_01101" 11587 "s59E25H2" 0 "Molybdopterin adenylyltransferase"
"JS1_A09_01101" 11587 "s59E5H2" 0.4012004 "Molybdopterin adenylyltransferase"
"JS1_A09_01101" 11587 "s63E6H2" 0.1903645 "Molybdopterin adenylyltransferase"
"JS1_A09_01102" 11588 "s59E13H2" 0.01391119 "MOSC domain protein"
"JS1_A09_01102" 11588 "s59E25H2" 0 "MOSC domain protein"
"JS1_A09_01102" 11588 "s59E5H2" 0.5802995 "MOSC domain protein"
"JS1_A09_01102" 11588 "s63E6H2" 0.1091377 "MOSC domain protein"
"JS1_A09_01103" 11589 "s59E13H2" 0 "Cyclic pyranopterin monophosphate synthase accessory protein"
"JS1_A09_01103" 11589 "s59E25H2" 0 "Cyclic pyranopterin monophosphate synthase accessory protein"
"JS1_A09_01103" 11589 "s59E5H2" 0.4410047 "Cyclic pyranopterin monophosphate synthase accessory protein"
"JS1_A09_01103" 11589 "s63E6H2" 0.2154363 "Cyclic pyranopterin monophosphate synthase accessory protein"
"JS1_A09_01104" 11590 "s59E13H2" 0.1554723 "Cyclic pyranopterin monophosphate synthase"
"JS1_A09_01104" 11590 "s59E25H2" 0 "Cyclic pyranopterin monophosphate synthase"
"JS1_A09_01104" 11590 "s59E5H2" 0.3671292 "Cyclic pyranopterin monophosphate synthase"
"JS1_A09_01104" 11590 "s63E6H2" 0.2919262 "Cyclic pyranopterin monophosphate synthase"
"JS1_A09_01105" 11591 "s59E13H2" 0.184754 "Molybdopterin molybdenumtransferase"

"JS1_A09_01105" 11591 "s59E25H2" 0 "Molybdopterin molybdenumtransferase"
"JS1_A09_01105" 11591 "s59E5H2" 0.3909264 "Molybdopterin molybdenumtransferase"
"JS1_A09_01105" 11591 "s63E6H2" 0.4381496 "Molybdopterin molybdenumtransferase"
"JS1_A09_01106" 11592 "s59E13H2" 0.437322 "Lipopolysaccharide export system permease protein LptF"
"JS1_A09_01106" 11592 "s59E25H2" 0 "Lipopolysaccharide export system permease protein LptF"
"JS1_A09_01106" 11592 "s59E5H2" 0.7848241 "Lipopolysaccharide export system permease protein LptF"
"JS1_A09_01106" 11592 "s63E6H2" 0.7104533 "Lipopolysaccharide export system permease protein LptF"
"JS1_A09_01107" 11593 "s59E13H2" 0.03396522 "LPS-assembly protein LptD"
"JS1_A09_01107" 11593 "s59E25H2" 0 "LPS-assembly protein LptD"
"JS1_A09_01107" 11593 "s59E5H2" 0.07088962 "LPS-assembly protein LptD"
"JS1_A09_01107" 11593 "s63E6H2" 0.7811722 "LPS-assembly protein LptD"
"JS1_A09_01108" 11594 "s59E13H2" 0.01114084 "hypothetical protein"
"JS1_A09_01108" 11594 "s59E25H2" 0 "hypothetical protein"
"JS1_A09_01108" 11594 "s59E5H2" 0 "hypothetical protein"
"JS1_A09_01108" 11594 "s63E6H2" 0.9654431 "hypothetical protein"
"JS1_A09_01109" 11595 "s59E13H2" 0.2631126 "LPS-assembly protein LptD"
"JS1_A09_01109" 11595 "s59E25H2" 0 "LPS-assembly protein LptD"
"JS1_A09_01109" 11595 "s59E5H2" 0.8034051 "LPS-assembly protein LptD"
"JS1_A09_01109" 11595 "s63E6H2" 3.018874 "LPS-assembly protein LptD"
"JS1_A09_01110" 11596 "s59E13H2" 0.150003 "Lipopolysaccharide-assembly, LptC-related"
"JS1_A09_01110" 11596 "s59E25H2" 0 "Lipopolysaccharide-assembly, LptC-related"
"JS1_A09_01110" 11596 "s59E5H2" 0.3962868 "Lipopolysaccharide-assembly, LptC-related"
"JS1_A09_01110" 11596 "s63E6H2" 1.505191 "Lipopolysaccharide-assembly, LptC-related"
"JS1_A09_01111" 11597 "s59E13H2" 0.1076842 "Clostripain family protein"
"JS1_A09_01111" 11597 "s59E25H2" 0 "Clostripain family protein"
"JS1_A09_01111" 11597 "s59E5H2" 0.05919984 "Clostripain family protein"
"JS1_A09_01111" 11597 "s63E6H2" 0.256931 "Clostripain family protein"
"JS1_A09_01112" 11598 "s59E13H2" 0 "Succinyl-CoA ligase [ADP-forming] subunit beta"
"JS1_A09_01112" 11598 "s59E25H2" 0 "Succinyl-CoA ligase [ADP-forming] subunit beta"
"JS1_A09_01112" 11598 "s59E5H2" 0 "Succinyl-CoA ligase [ADP-forming] subunit beta"
"JS1_A09_01112" 11598 "s63E6H2" 0 "Succinyl-CoA ligase [ADP-forming] subunit beta"
"JS1_A09_01113" 11599 "s59E13H2" 8.166361 "Alanine racemase"
"JS1_A09_01113" 11599 "s59E25H2" 0.6335878 "Alanine racemase"
"JS1_A09_01113" 11599 "s59E5H2" 0.4919209 "Alanine racemase"
"JS1_A09_01113" 11599 "s63E6H2" 0.2626985 "Alanine racemase"
"JS1_A09_01115" 11601 "s59E13H2" 0 "hypothetical protein"
"JS1_A09_01115" 11601 "s59E25H2" 0 "hypothetical protein"
"JS1_A09_01115" 11601 "s59E5H2" 0.1088213 "hypothetical protein"
"JS1_A09_01115" 11601 "s63E6H2" 0.02702751 "hypothetical protein"
"JS1_A09_01116" 11602 "s59E13H2" 0 "Putative sugar phosphate isomerase YwlF"
"JS1_A09_01116" 11602 "s59E25H2" 0 "Putative sugar phosphate isomerase YwlF"
"JS1_A09_01116" 11602 "s59E5H2" 0 "Putative sugar phosphate isomerase YwlF"
"JS1_A09_01116" 11602 "s63E6H2" 0.00120382 "Putative sugar phosphate isomerase YwlF"
"JS1_A09_01117" 11603 "s59E13H2" 0 "hypothetical protein"
"JS1_A09_01117" 11603 "s59E25H2" 0 "hypothetical protein"
"JS1_A09_01117" 11603 "s59E5H2" 0 "hypothetical protein"
"JS1_A09_01117" 11603 "s63E6H2" 0 "hypothetical protein"

"JS1_A09_01118" 11604 "s59E13H2" 0 "hypothetical protein"
"JS1_A09_01118" 11604 "s59E25H2" 0 "hypothetical protein"
"JS1_A09_01118" 11604 "s59E5H2" 0 "hypothetical protein"
"JS1_A09_01118" 11604 "s63E6H2" 0 "hypothetical protein"
"JS1_A09_01119" 11605 "s59E13H2" 0 "hypothetical protein"
"JS1_A09_01119" 11605 "s59E25H2" 0 "hypothetical protein"
"JS1_A09_01119" 11605 "s59E5H2" 0 "hypothetical protein"
"JS1_A09_01119" 11605 "s63E6H2" 0 "hypothetical protein"
"JS1_A09_01120" 11606 "s59E13H2" 0 "hypothetical protein"
"JS1_A09_01120" 11606 "s59E25H2" 0 "hypothetical protein"
"JS1_A09_01120" 11606 "s59E5H2" 0 "hypothetical protein"
"JS1_A09_01120" 11606 "s63E6H2" 0 "hypothetical protein"
"JS1_A09_01122" 11608 "s59E13H2" 0 "methylcobalamin:coenzyme M methyltransferase"
"JS1_A09_01122" 11608 "s59E25H2" 0 "methylcobalamin:coenzyme M methyltransferase"
"JS1_A09_01122" 11608 "s59E5H2" 0 "methylcobalamin:coenzyme M methyltransferase"
"JS1_A09_01122" 11608 "s63E6H2" 0.001481308 "methylcobalamin:coenzyme M methyltransferase"
"JS1_A09_01124" 11610 "s59E13H2" 1.06539 "Putative nickel-responsive regulator"
"JS1_A09_01124" 11610 "s59E25H2" 0 "Putative nickel-responsive regulator"
"JS1_A09_01124" 11610 "s59E5H2" 1.253552 "Putative nickel-responsive regulator"
"JS1_A09_01124" 11610 "s63E6H2" 0.1986339 "Putative nickel-responsive regulator"
"JS1_A09_01125" 11611 "s59E13H2" 0.938702 "putative peptidyl-prolyl cis-trans isomerase"
"JS1_A09_01125" 11611 "s59E25H2" 0 "putative peptidyl-prolyl cis-trans isomerase"
"JS1_A09_01125" 11611 "s59E5H2" 0.7192728 "putative peptidyl-prolyl cis-trans isomerase"
"JS1_A09_01125" 11611 "s63E6H2" 0.1908759 "putative peptidyl-prolyl cis-trans isomerase"
"JS1_A09_01126" 11612 "s59E13H2" 0.5672112 "Nitroreductase family protein"
"JS1_A09_01126" 11612 "s59E25H2" 0 "Nitroreductase family protein"
"JS1_A09_01126" 11612 "s59E5H2" 0.4396207 "Nitroreductase family protein"
"JS1_A09_01126" 11612 "s63E6H2" 0.07716183 "Nitroreductase family protein"
"JS1_A09_01127" 11613 "s59E13H2" 18.14928 "Adenosylcobalamin-dependent ribonucleoside-triphosphate reductase"
"JS1_A09_01127" 11613 "s59E25H2" 0.1239243 "Adenosylcobalamin-dependent ribonucleoside-triphosphate reductase"
"JS1_A09_01127" 11613 "s59E5H2" 4.52844 "Adenosylcobalamin-dependent ribonucleoside-triphosphate reductase"
"JS1_A09_01127" 11613 "s63E6H2" 4.684192 "Adenosylcobalamin-dependent ribonucleoside-triphosphate reductase"
"JS1_A09_01128" 11614 "s59E13H2" 29.18942 "Adenosylcobalamin-dependent ribonucleoside-triphosphate reductase"
"JS1_A09_01128" 11614 "s59E25H2" 0.3350254 "Adenosylcobalamin-dependent ribonucleoside-triphosphate reductase"
"JS1_A09_01128" 11614 "s59E5H2" 10.32273 "Adenosylcobalamin-dependent ribonucleoside-triphosphate reductase"
"JS1_A09_01128" 11614 "s63E6H2" 11.89276 "Adenosylcobalamin-dependent ribonucleoside-triphosphate reductase"
"JS1_A09_01129" 11615 "s59E13H2" 25.08448 "Methionine aminopeptidase"
"JS1_A09_01129" 11615 "s59E25H2" 0.3359788 "Methionine aminopeptidase"
"JS1_A09_01129" 11615 "s59E5H2" 9.620076 "Methionine aminopeptidase"
"JS1_A09_01129" 11615 "s63E6H2" 11.33426 "Methionine aminopeptidase"
"JS1_A09_01130" 11616 "s59E13H2" 0.9049795 "D-alanine--D-alanine ligase"
"JS1_A09_01130" 11616 "s59E25H2" 0 "D-alanine--D-alanine ligase"
"JS1_A09_01130" 11616 "s59E5H2" 5.341146 "D-alanine--D-alanine ligase"
"JS1_A09_01130" 11616 "s63E6H2" 8.584871 "D-alanine--D-alanine ligase"
"JS1_A09_01131" 11617 "s59E13H2" 0.2774054 "putative adenylyltransferase"
"JS1_A09_01131" 11617 "s59E25H2" 0 "putative adenylyltransferase"
"JS1_A09_01131" 11617 "s59E5H2" 2.594307 "putative adenylyltransferase"

"JS1_A09_01131" 11617 "s63E6H2" 5.577541 "putative adenylyltransferase"
"JS1_A09_01132" 11618 "s59E13H2" 0.5320141 "Replication-associated recombination protein A"
"JS1_A09_01132" 11618 "s59E25H2" 0.1614114 "Replication-associated recombination protein A"
"JS1_A09_01132" 11618 "s59E5H2" 2.718374 "Replication-associated recombination protein A"
"JS1_A09_01132" 11618 "s63E6H2" 5.108009 "Replication-associated recombination protein A"
"JS1_A09_01133" 11619 "s59E13H2" 0.504274 "Spermidine N(1)-acetyltransferase"
"JS1_A09_01133" 11619 "s59E25H2" 0 "Spermidine N(1)-acetyltransferase"
"JS1_A09_01133" 11619 "s59E5H2" 2.279303 "Spermidine N(1)-acetyltransferase"
"JS1_A09_01133" 11619 "s63E6H2" 3.613566 "Spermidine N(1)-acetyltransferase"
"JS1_A09_01134" 11620 "s59E13H2" 0.4856697 "(R)-stereoselective amidase"
"JS1_A09_01134" 11620 "s59E25H2" 0 "(R)-stereoselective amidase"
"JS1_A09_01134" 11620 "s59E5H2" 2.273598 "(R)-stereoselective amidase"
"JS1_A09_01134" 11620 "s63E6H2" 3.663577 "(R)-stereoselective amidase"
"JS1_A09_01135" 11621 "s59E13H2" 0.9576303 "Diacylglycerol kinase"
"JS1_A09_01135" 11621 "s59E25H2" 0 "Diacylglycerol kinase"
"JS1_A09_01135" 11621 "s59E5H2" 2.569658 "Diacylglycerol kinase"
"JS1_A09_01135" 11621 "s63E6H2" 4.094661 "Diacylglycerol kinase"
"JS1_A09_01136" 11622 "s59E13H2" 0.3865871 "putative diguanylate cyclase YeaP"
"JS1_A09_01136" 11622 "s59E25H2" 0 "putative diguanylate cyclase YeaP"
"JS1_A09_01136" 11622 "s59E5H2" 1.349976 "putative diguanylate cyclase YeaP"
"JS1_A09_01136" 11622 "s63E6H2" 0.8883806 "putative diguanylate cyclase YeaP"
"JS1_A09_01137" 11623 "s59E13H2" 0.5919635 "L-fuculose phosphate aldolase"
"JS1_A09_01137" 11623 "s59E25H2" 0 "L-fuculose phosphate aldolase"
"JS1_A09_01137" 11623 "s59E5H2" 0.9760786 "L-fuculose phosphate aldolase"
"JS1_A09_01137" 11623 "s63E6H2" 1.002347 "L-fuculose phosphate aldolase"
"JS1_A09_01138" 11624 "s59E13H2" 0.1107578 "Aromatic amino acid exporter YddG"
"JS1_A09_01138" 11624 "s59E25H2" 0 "Aromatic amino acid exporter YddG"
"JS1_A09_01138" 11624 "s59E5H2" 0.8069381 "Aromatic amino acid exporter YddG"
"JS1_A09_01138" 11624 "s63E6H2" 0.5690589 "Aromatic amino acid exporter YddG"
"JS1_A09_01140" 11626 "s59E13H2" 0.0462946 "hypothetical protein"
"JS1_A09_01140" 11626 "s59E25H2" 0 "hypothetical protein"
"JS1_A09_01140" 11626 "s59E5H2" 0.8656888 "hypothetical protein"
"JS1_A09_01140" 11626 "s63E6H2" 7.351971 "hypothetical protein"
"JS1_A09_01142" 11628 "s59E13H2" 0.6933659 "Heterodisulfide reductase subunit A-like protein"
"JS1_A09_01142" 11628 "s59E25H2" 0 "Heterodisulfide reductase subunit A-like protein"
"JS1_A09_01142" 11628 "s59E5H2" 1.221753 "Heterodisulfide reductase subunit A-like protein"
"JS1_A09_01142" 11628 "s63E6H2" 16.40452 "Heterodisulfide reductase subunit A-like protein"
"JS1_A09_01144" 11630 "s59E13H2" 0.4778004 "Ribosomal protein S6 modification protein"
"JS1_A09_01144" 11630 "s59E25H2" 0 "Ribosomal protein S6 modification protein"
"JS1_A09_01144" 11630 "s59E5H2" 0.9708908 "Ribosomal protein S6 modification protein"
"JS1_A09_01144" 11630 "s63E6H2" 8.479082 "Ribosomal protein S6 modification protein"
"JS1_A09_01145" 11631 "s59E13H2" 8.605055 "putative transporter"
"JS1_A09_01145" 11631 "s59E25H2" 0.1370641 "putative transporter"
"JS1_A09_01145" 11631 "s59E5H2" 0.8817053 "putative transporter"
"JS1_A09_01145" 11631 "s63E6H2" 7.461487 "putative transporter"
"JS1_A09_01146" 11632 "s59E13H2" 16.57486 "Cyclic nucleotide-binding domain protein"
"JS1_A09_01146" 11632 "s59E25H2" 0 "Cyclic nucleotide-binding domain protein"

"JS1_A09_01146" 11632 "s59E5H2" 2.280951 "Cyclic nucleotide-binding domain protein"
"JS1_A09_01146" 11632 "s63E6H2" 2.270202 "Cyclic nucleotide-binding domain protein"
"JS1_A09_01148" 11634 "s59E13H2" 3.381022 "hypothetical protein"
"JS1_A09_01148" 11634 "s59E25H2" 0.7438017 "hypothetical protein"
"JS1_A09_01148" 11634 "s59E5H2" 1.207752 "hypothetical protein"
"JS1_A09_01148" 11634 "s63E6H2" 1.874992 "hypothetical protein"
"JS1_A09_01149" 11635 "s59E13H2" 9.403549 "Glycogen synthase"
"JS1_A09_01149" 11635 "s59E25H2" 0 "Glycogen synthase"
"JS1_A09_01149" 11635 "s59E5H2" 1.063121 "Glycogen synthase"
"JS1_A09_01149" 11635 "s63E6H2" 1.574683 "Glycogen synthase"
"JS1_A09_01150" 11636 "s59E13H2" 11.83729 "hypothetical protein"
"JS1_A09_01150" 11636 "s59E25H2" 0 "hypothetical protein"
"JS1_A09_01150" 11636 "s59E5H2" 0.7111685 "hypothetical protein"
"JS1_A09_01150" 11636 "s63E6H2" 0.368592 "hypothetical protein"
"JS1_A09_01151" 11637 "s59E13H2" 10.26257 "hypothetical protein"
"JS1_A09_01151" 11637 "s59E25H2" 0 "hypothetical protein"
"JS1_A09_01151" 11637 "s59E5H2" 0.4903884 "hypothetical protein"
"JS1_A09_01151" 11637 "s63E6H2" 0.2953294 "hypothetical protein"
"JS1_A09_01152" 11638 "s59E13H2" 11.39761 "Aspartate racemase"
"JS1_A09_01152" 11638 "s59E25H2" 0.6869565 "Aspartate racemase"
"JS1_A09_01152" 11638 "s59E5H2" 1.311788 "Aspartate racemase"
"JS1_A09_01152" 11638 "s63E6H2" 0.3196473 "Aspartate racemase"
"JS1_A09_01153" 11639 "s59E13H2" 6.877567 "1-acyl-sn-glycerol-3-phosphate acyltransferase"
"JS1_A09_01153" 11639 "s59E25H2" 0 "1-acyl-sn-glycerol-3-phosphate acyltransferase"
"JS1_A09_01153" 11639 "s59E5H2" 0.7629716 "1-acyl-sn-glycerol-3-phosphate acyltransferase"
"JS1_A09_01153" 11639 "s63E6H2" 0.1506278 "1-acyl-sn-glycerol-3-phosphate acyltransferase"
"JS1_A09_01154" 11640 "s59E13H2" 3.277012 "Alpha/beta hydrolase family protein"
"JS1_A09_01154" 11640 "s59E25H2" 0 "Alpha/beta hydrolase family protein"
"JS1_A09_01154" 11640 "s59E5H2" 0.2153575 "Alpha/beta hydrolase family protein"
"JS1_A09_01154" 11640 "s63E6H2" 0.08906305 "Alpha/beta hydrolase family protein"
"JS1_A09_01155" 11641 "s59E13H2" 0.00771423 "Zinc transporter ZupT"
"JS1_A09_01155" 11641 "s59E25H2" 0 "Zinc transporter ZupT"
"JS1_A09_01155" 11641 "s59E5H2" 1.077901 "Zinc transporter ZupT"
"JS1_A09_01155" 11641 "s63E6H2" 0.0268949 "Zinc transporter ZupT"
"JS1_A09_01156" 11642 "s59E13H2" 0.08105746 "DNA alkylation repair enzyme"
"JS1_A09_01156" 11642 "s59E25H2" 0.3144105 "DNA alkylation repair enzyme"
"JS1_A09_01156" 11642 "s59E5H2" 1.616029 "DNA alkylation repair enzyme"
"JS1_A09_01156" 11642 "s63E6H2" 3.117056 "DNA alkylation repair enzyme"
"JS1_A09_01157" 11643 "s59E13H2" 0.07180143 "Demethylrebeccamycin-D-glucose O-methyltransferase"
"JS1_A09_01157" 11643 "s59E25H2" 0 "Demethylrebeccamycin-D-glucose O-methyltransferase"
"JS1_A09_01157" 11643 "s59E5H2" 2.615386 "Demethylrebeccamycin-D-glucose O-methyltransferase"
"JS1_A09_01157" 11643 "s63E6H2" 2.447137 "Demethylrebeccamycin-D-glucose O-methyltransferase"
"JS1_A09_01158" 11644 "s59E13H2" 0 "hypothetical protein"
"JS1_A09_01158" 11644 "s59E25H2" 0 "hypothetical protein"
"JS1_A09_01158" 11644 "s59E5H2" 0 "hypothetical protein"
"JS1_A09_01158" 11644 "s63E6H2" 2.08525 "hypothetical protein"
"JS1_A09_01159" 11645 "s59E13H2" 0 "hypothetical protein"

"JS1_A09_01159" 11645 "s59E25H2" 0 "hypothetical protein"
"JS1_A09_01159" 11645 "s59E5H2" 0 "hypothetical protein"
"JS1_A09_01159" 11645 "s63E6H2" 1.975609 "hypothetical protein"
"JS1_A09_01161" 11647 "s59E13H2" 0.03525437 "hypothetical protein"
"JS1_A09_01161" 11647 "s59E25H2" 0 "hypothetical protein"
"JS1_A09_01161" 11647 "s59E5H2" 1.068679 "hypothetical protein"
"JS1_A09_01161" 11647 "s63E6H2" 1.249808 "hypothetical protein"
"JS1_A09_01162" 11648 "s59E13H2" 0 "HTH-type transcriptional repressor AseR"
"JS1_A09_01162" 11648 "s59E25H2" 0 "HTH-type transcriptional repressor AseR"
"JS1_A09_01162" 11648 "s59E5H2" 0 "HTH-type transcriptional repressor AseR"
"JS1_A09_01162" 11648 "s63E6H2" 0.007882626 "HTH-type transcriptional repressor AseR"
"JS1_A09_01164" 11650 "s59E13H2" 0 "hypothetical protein"
"JS1_A09_01164" 11650 "s59E25H2" 0 "hypothetical protein"
"JS1_A09_01164" 11650 "s59E5H2" 0 "hypothetical protein"
"JS1_A09_01164" 11650 "s63E6H2" 0.004470811 "hypothetical protein"
"JS1_A09_01165" 11651 "s59E13H2" 0 "putative permease"
"JS1_A09_01165" 11651 "s59E25H2" 0 "putative permease"
"JS1_A09_01165" 11651 "s59E5H2" 0 "putative permease"
"JS1_A09_01165" 11651 "s63E6H2" 0.01127059 "putative permease"
"JS1_A09_01166" 11652 "s59E13H2" 0 "hypothetical protein"
"JS1_A09_01166" 11652 "s59E25H2" 0 "hypothetical protein"
"JS1_A09_01166" 11652 "s59E5H2" 0 "hypothetical protein"
"JS1_A09_01166" 11652 "s63E6H2" 0.009118934 "hypothetical protein"
"JS1_A09_01167" 11653 "s59E13H2" 0 "Sulfate transporter family protein"
"JS1_A09_01167" 11653 "s59E25H2" 0 "Sulfate transporter family protein"
"JS1_A09_01167" 11653 "s59E5H2" 0 "Sulfate transporter family protein"
"JS1_A09_01167" 11653 "s63E6H2" 0.004458146 "Sulfate transporter family protein"
"JS1_A09_01168" 11654 "s59E13H2" 0 "hypothetical protein"
"JS1_A09_01168" 11654 "s59E25H2" 0 "hypothetical protein"
"JS1_A09_01168" 11654 "s59E5H2" 0 "hypothetical protein"
"JS1_A09_01168" 11654 "s63E6H2" 0.1709353 "hypothetical protein"
"JS1_A09_01169" 11655 "s59E13H2" 0 "HTH-type transcriptional repressor AseR"
"JS1_A09_01169" 11655 "s59E25H2" 0 "HTH-type transcriptional repressor AseR"
"JS1_A09_01169" 11655 "s59E5H2" 0 "HTH-type transcriptional repressor AseR"
"JS1_A09_01169" 11655 "s63E6H2" 0.006255501 "HTH-type transcriptional repressor AseR"
"JS1_A09_01170" 11656 "s59E13H2" 0.2057548 "putative permease"
"JS1_A09_01170" 11656 "s59E25H2" 0 "putative permease"
"JS1_A09_01170" 11656 "s59E5H2" 0.1021339 "putative permease"
"JS1_A09_01170" 11656 "s63E6H2" 0.3049829 "putative permease"
"JS1_A09_01171" 11657 "s59E13H2" 0.004150457 "Thioredoxin"
"JS1_A09_01171" 11657 "s59E25H2" 0 "Thioredoxin"
"JS1_A09_01171" 11657 "s59E5H2" 0.3067553 "Thioredoxin"
"JS1_A09_01171" 11657 "s63E6H2" 0.1891585 "Thioredoxin"
"JS1_A09_01172" 11658 "s59E13H2" 0.0205095 "Cadmium, cobalt and zinc/H(+)-K(+) antiporter"
"JS1_A09_01172" 11658 "s59E25H2" 0 "Cadmium, cobalt and zinc/H(+)-K(+) antiporter"
"JS1_A09_01172" 11658 "s59E5H2" 0.4159417 "Cadmium, cobalt and zinc/H(+)-K(+) antiporter"
"JS1_A09_01172" 11658 "s63E6H2" 2.227624 "Cadmium, cobalt and zinc/H(+)-K(+) antiporter"

"JS1_A09_01173" 11659 "s59E13H2" 0.0512352 "Sporulation thiol-disulfide oxidoreductase A precursor"
"JS1_A09_01173" 11659 "s59E25H2" 0 "Sporulation thiol-disulfide oxidoreductase A precursor"
"JS1_A09_01173" 11659 "s59E5H2" 1.140781 "Sporulation thiol-disulfide oxidoreductase A precursor"
"JS1_A09_01173" 11659 "s63E6H2" 14.29874 "Sporulation thiol-disulfide oxidoreductase A precursor"
"JS1_A09_01174" 11660 "s59E13H2" 0.141515 "Thioredoxin C-1"
"JS1_A09_01174" 11660 "s59E25H2" 0.5742092 "Thioredoxin C-1"
"JS1_A09_01174" 11660 "s59E5H2" 3.948168 "Thioredoxin C-1"
"JS1_A09_01174" 11660 "s63E6H2" 17.31862 "Thioredoxin C-1"
"JS1_A09_01175" 11661 "s59E13H2" 0.1496245 "thiol:disulfide interchange protein precursor"
"JS1_A09_01175" 11661 "s59E25H2" 0 "thiol:disulfide interchange protein precursor"
"JS1_A09_01175" 11661 "s59E5H2" 4.33202 "thiol:disulfide interchange protein precursor"
"JS1_A09_01175" 11661 "s63E6H2" 23.87592 "thiol:disulfide interchange protein precursor"
"JS1_A09_01176" 11662 "s59E13H2" 0.2431111 "Sodium Bile acid symporter family protein"
"JS1_A09_01176" 11662 "s59E25H2" 0 "Sodium Bile acid symporter family protein"
"JS1_A09_01176" 11662 "s59E5H2" 5.671293 "Sodium Bile acid symporter family protein"
"JS1_A09_01176" 11662 "s63E6H2" 29.79249 "Sodium Bile acid symporter family protein"
"JS1_A09_01178" 11664 "s59E13H2" 0.3815677 "Biotin transporter BioY"
"JS1_A09_01178" 11664 "s59E25H2" 0 "Biotin transporter BioY"
"JS1_A09_01178" 11664 "s59E5H2" 2.310779 "Biotin transporter BioY"
"JS1_A09_01178" 11664 "s63E6H2" 1.978558 "Biotin transporter BioY"
"JS1_A09_01179" 11665 "s59E13H2" 0.1786352 "Bifunctional ligase/repressor BirA"
"JS1_A09_01179" 11665 "s59E25H2" 0 "Bifunctional ligase/repressor BirA"
"JS1_A09_01179" 11665 "s59E5H2" 0.7494433 "Bifunctional ligase/repressor BirA"
"JS1_A09_01179" 11665 "s63E6H2" 2.598066 "Bifunctional ligase/repressor BirA"
"JS1_A09_01180" 11666 "s59E13H2" 0.1607969 "hypothetical protein"
"JS1_A09_01180" 11666 "s59E25H2" 0 "hypothetical protein"
"JS1_A09_01180" 11666 "s59E5H2" 2.305523 "hypothetical protein"
"JS1_A09_01180" 11666 "s63E6H2" 0.1578976 "hypothetical protein"
"JS1_A09_01181" 11667 "s59E13H2" 0.1264037 "Deoxyadenosine/deoxycytidine kinase"
"JS1_A09_01181" 11667 "s59E25H2" 0 "Deoxyadenosine/deoxycytidine kinase"
"JS1_A09_01181" 11667 "s59E5H2" 1.731597 "Deoxyadenosine/deoxycytidine kinase"
"JS1_A09_01181" 11667 "s63E6H2" 12.40563 "Deoxyadenosine/deoxycytidine kinase"
"JS1_A09_01182" 11668 "s59E13H2" 0.1991397 "Deoxyguanosine kinase"
"JS1_A09_01182" 11668 "s59E25H2" 0 "Deoxyguanosine kinase"
"JS1_A09_01182" 11668 "s59E5H2" 1.441789 "Deoxyguanosine kinase"
"JS1_A09_01182" 11668 "s63E6H2" 10.46612 "Deoxyguanosine kinase"
"JS1_A09_01183" 11669 "s59E13H2" 0.1161092 "Octanoyltransferase LipM"
"JS1_A09_01183" 11669 "s59E25H2" 0 "Octanoyltransferase LipM"
"JS1_A09_01183" 11669 "s59E5H2" 0.6730645 "Octanoyltransferase LipM"
"JS1_A09_01183" 11669 "s63E6H2" 4.993179 "Octanoyltransferase LipM"
"JS1_A09_01185" 11671 "s59E13H2" 0.2025618 "hypothetical protein"
"JS1_A09_01185" 11671 "s59E25H2" 0 "hypothetical protein"
"JS1_A09_01185" 11671 "s59E5H2" 0.7792365 "hypothetical protein"
"JS1_A09_01185" 11671 "s63E6H2" 0.03149988 "hypothetical protein"
"JS1_A09_01187" 11673 "s59E13H2" 0.561418 "Septum site-determining protein MinD"
"JS1_A09_01187" 11673 "s59E25H2" 0 "Septum site-determining protein MinD"
"JS1_A09_01187" 11673 "s59E5H2" 0.5296591 "Septum site-determining protein MinD"

"JS1_A09_01187" 11673 "s63E6H2" 0.1067324 "Septum site-determining protein MinD"
"JS1_A09_01188" 11674 "s59E13H2" 1.200825 "Radical SAM superfamily protein"
"JS1_A09_01188" 11674 "s59E25H2" 0 "Radical SAM superfamily protein"
"JS1_A09_01188" 11674 "s59E5H2" 0.4771874 "Radical SAM superfamily protein"
"JS1_A09_01188" 11674 "s63E6H2" 0.1198706 "Radical SAM superfamily protein"
"JS1_A09_01189" 11675 "s59E13H2" 0.5876996 "Heterodisulfide reductase subunit A-like protein"
"JS1_A09_01189" 11675 "s59E25H2" 0 "Heterodisulfide reductase subunit A-like protein"
"JS1_A09_01189" 11675 "s59E5H2" 0.8600231 "Heterodisulfide reductase subunit A-like protein"
"JS1_A09_01189" 11675 "s63E6H2" 0.2609063 "Heterodisulfide reductase subunit A-like protein"
"JS1_A09_01190" 11676 "s59E13H2" 0.4357103 "antiporter inner membrane protein"
"JS1_A09_01190" 11676 "s59E25H2" 0 "antiporter inner membrane protein"
"JS1_A09_01190" 11676 "s59E5H2" 0.5091991 "antiporter inner membrane protein"
"JS1_A09_01190" 11676 "s63E6H2" 0.3522404 "antiporter inner membrane protein"
"JS1_A09_01191" 11677 "s59E13H2" 0.1022071 "Dinitrogenase iron-molybdenum cofactor"
"JS1_A09_01191" 11677 "s59E25H2" 0 "Dinitrogenase iron-molybdenum cofactor"
"JS1_A09_01191" 11677 "s59E5H2" 0.1292876 "Dinitrogenase iron-molybdenum cofactor"
"JS1_A09_01191" 11677 "s63E6H2" 0.01974626 "Dinitrogenase iron-molybdenum cofactor"
"JS1_A09_01192" 11678 "s59E13H2" 0.1682882 "Dinitrogenase iron-molybdenum cofactor"
"JS1_A09_01192" 11678 "s59E25H2" 0 "Dinitrogenase iron-molybdenum cofactor"
"JS1_A09_01192" 11678 "s59E5H2" 0.3507787 "Dinitrogenase iron-molybdenum cofactor"
"JS1_A09_01192" 11678 "s63E6H2" 0.08609895 "Dinitrogenase iron-molybdenum cofactor"
"JS1_A09_01193" 11679 "s59E13H2" 0.1436729 "hypothetical protein"
"JS1_A09_01193" 11679 "s59E25H2" 0 "hypothetical protein"
"JS1_A09_01193" 11679 "s59E5H2" 0.02197 "hypothetical protein"
"JS1_A09_01193" 11679 "s63E6H2" 0.02705782 "hypothetical protein"
"JS1_A09_01194" 11680 "s59E13H2" 0.5776765 "Ferric uptake regulation protein"
"JS1_A09_01194" 11680 "s59E25H2" 0 "Ferric uptake regulation protein"
"JS1_A09_01194" 11680 "s59E5H2" 0.2749577 "Ferric uptake regulation protein"
"JS1_A09_01194" 11680 "s63E6H2" 0.1635456 "Ferric uptake regulation protein"
"JS1_A09_01195" 11681 "s59E13H2" 0.769967 "hypothetical protein"
"JS1_A09_01195" 11681 "s59E25H2" 0 "hypothetical protein"
"JS1_A09_01195" 11681 "s59E5H2" 0.6155453 "hypothetical protein"
"JS1_A09_01195" 11681 "s63E6H2" 0.926791 "hypothetical protein"
"JS1_A09_01196" 11682 "s59E13H2" 0.6213061 "FeoA domain protein"
"JS1_A09_01196" 11682 "s59E25H2" 0 "FeoA domain protein"
"JS1_A09_01196" 11682 "s59E5H2" 0.5151962 "FeoA domain protein"
"JS1_A09_01196" 11682 "s63E6H2" 3.020144 "FeoA domain protein"
"JS1_A09_01197" 11683 "s59E13H2" 0.4932147 "Transcriptional regulator MntR"
"JS1_A09_01197" 11683 "s59E25H2" 0 "Transcriptional regulator MntR"
"JS1_A09_01197" 11683 "s59E5H2" 0.3528684 "Transcriptional regulator MntR"
"JS1_A09_01197" 11683 "s63E6H2" 2.945837 "Transcriptional regulator MntR"
"JS1_A09_01198" 11684 "s59E13H2" 0.5256039 "Sulfur carrier protein ThiS"
"JS1_A09_01198" 11684 "s59E25H2" 0 "Sulfur carrier protein ThiS"
"JS1_A09_01198" 11684 "s59E5H2" 0.1570409 "Sulfur carrier protein ThiS"
"JS1_A09_01198" 11684 "s63E6H2" 2.821773 "Sulfur carrier protein ThiS"
"JS1_A09_01199" 11685 "s59E13H2" 0.5811292 "Tungsten-containing aldehyde ferredoxin oxidoreductase"
"JS1_A09_01199" 11685 "s59E25H2" 0 "Tungsten-containing aldehyde ferredoxin oxidoreductase"

"JS1_A09_01199" 11685 "s59E5H2" 0.1973101 "Tungsten-containing aldehyde ferredoxin oxidoreductase"
"JS1_A09_01199" 11685 "s63E6H2" 1.094951 "Tungsten-containing aldehyde ferredoxin oxidoreductase"
"JS1_A09_01200" 11686 "s59E13H2" 0.952734 "Malate-2H(+)/Na(+)-lactate antiporter"
"JS1_A09_01200" 11686 "s59E25H2" 0 "Malate-2H(+)/Na(+)-lactate antiporter"
"JS1_A09_01200" 11686 "s59E5H2" 0.563763 "Malate-2H(+)/Na(+)-lactate antiporter"
"JS1_A09_01200" 11686 "s63E6H2" 0.8407151 "Malate-2H(+)/Na(+)-lactate antiporter"
"JS1_A09_01201" 11687 "s59E13H2" 0.7289472 "Cyclic 2,3-diphosphoglycerate synthetase"
"JS1_A09_01201" 11687 "s59E25H2" 0 "Cyclic 2,3-diphosphoglycerate synthetase"
"JS1_A09_01201" 11687 "s59E5H2" 0.2908942 "Cyclic 2,3-diphosphoglycerate synthetase"
"JS1_A09_01201" 11687 "s63E6H2" 1.098412 "Cyclic 2,3-diphosphoglycerate synthetase"
"JS1_A09_01202" 11688 "s59E13H2" 0.2673986 "5-aminovalerate aminotransferase DavT"
"JS1_A09_01202" 11688 "s59E25H2" 0 "5-aminovalerate aminotransferase DavT"
"JS1_A09_01202" 11688 "s59E5H2" 0.209063 "5-aminovalerate aminotransferase DavT"
"JS1_A09_01202" 11688 "s63E6H2" 1.234459 "5-aminovalerate aminotransferase DavT"
"JS1_A09_01203" 11689 "s59E13H2" 0.6189192 "Lysine 6-dehydrogenase"
"JS1_A09_01203" 11689 "s59E25H2" 0 "Lysine 6-dehydrogenase"
"JS1_A09_01203" 11689 "s59E5H2" 0.4453734 "Lysine 6-dehydrogenase"
"JS1_A09_01203" 11689 "s63E6H2" 1.26752 "Lysine 6-dehydrogenase"
"JS1_A09_01205" 11691 "s59E13H2" 0.3433506 "Lysine 6-dehydrogenase"
"JS1_A09_01205" 11691 "s59E25H2" 0 "Lysine 6-dehydrogenase"
"JS1_A09_01205" 11691 "s59E5H2" 0.3845902 "Lysine 6-dehydrogenase"
"JS1_A09_01205" 11691 "s63E6H2" 0.8332797 "Lysine 6-dehydrogenase"
"JS1_A09_01206" 11692 "s59E13H2" 0.3348404 "2-methylcitrate dehydratase"
"JS1_A09_01206" 11692 "s59E25H2" 0.1804949 "2-methylcitrate dehydratase"
"JS1_A09_01206" 11692 "s59E5H2" 0.1674386 "2-methylcitrate dehydratase"
"JS1_A09_01206" 11692 "s63E6H2" 0.4774278 "2-methylcitrate dehydratase"
"JS1_A09_01207" 11693 "s59E13H2" 0.208178 "3-isopropylmalate/3-methylmalate dehydrogenase"
"JS1_A09_01207" 11693 "s59E25H2" 0 "3-isopropylmalate/3-methylmalate dehydrogenase"
"JS1_A09_01207" 11693 "s59E5H2" 0.3248415 "3-isopropylmalate/3-methylmalate dehydrogenase"
"JS1_A09_01207" 11693 "s63E6H2" 0.2370977 "3-isopropylmalate/3-methylmalate dehydrogenase"
"JS1_A09_01208" 11694 "s59E13H2" 1.068705 "Isopropylmalate/citramalate isomerase small subunit"
"JS1_A09_01208" 11694 "s59E25H2" 0 "Isopropylmalate/citramalate isomerase small subunit"
"JS1_A09_01208" 11694 "s59E5H2" 0.1857273 "Isopropylmalate/citramalate isomerase small subunit"
"JS1_A09_01208" 11694 "s63E6H2" 0.2919024 "Isopropylmalate/citramalate isomerase small subunit"
"JS1_A09_01209" 11695 "s59E13H2" 1.313881 "Methanogen homoaconitase large subunit"
"JS1_A09_01209" 11695 "s59E25H2" 0 "Methanogen homoaconitase large subunit"
"JS1_A09_01209" 11695 "s59E5H2" 0.1509189 "Methanogen homoaconitase large subunit"
"JS1_A09_01209" 11695 "s63E6H2" 0.1299357 "Methanogen homoaconitase large subunit"
"JS1_A09_01210" 11696 "s59E13H2" 0 "Uroporphyrinogen decarboxylase (URO-D)"
"JS1_A09_01210" 11696 "s59E25H2" 0 "Uroporphyrinogen decarboxylase (URO-D)"
"JS1_A09_01210" 11696 "s59E5H2" 0 "Uroporphyrinogen decarboxylase (URO-D)"
"JS1_A09_01210" 11696 "s63E6H2" 0 "Uroporphyrinogen decarboxylase (URO-D)"
"JS1_A09_01211" 11697 "s59E13H2" 0 "Sialic acid TRAP transporter permease protein SiaT"
"JS1_A09_01211" 11697 "s59E25H2" 0 "Sialic acid TRAP transporter permease protein SiaT"
"JS1_A09_01211" 11697 "s59E5H2" 0 "Sialic acid TRAP transporter permease protein SiaT"
"JS1_A09_01211" 11697 "s63E6H2" 0 "Sialic acid TRAP transporter permease protein SiaT"
"JS1_A09_01212" 11698 "s59E13H2" 0 "2,3-diketo-L-gulonate TRAP transporter small permease protein YiaM"

"JS1_A09_01212" 11698 "s59E25H2" 0 "2,3-diketo-L-gulonate TRAP transporter small permease protein YiaM"
"JS1_A09_01212" 11698 "s59E5H2" 0 "2,3-diketo-L-gulonate TRAP transporter small permease protein YiaM"
"JS1_A09_01212" 11698 "s63E6H2" 0 "2,3-diketo-L-gulonate TRAP transporter small permease protein YiaM"
"JS1_A09_01214" 11700 "s59E13H2" 0.03029809 "hypothetical protein"
"JS1_A09_01214" 11700 "s59E25H2" 0 "hypothetical protein"
"JS1_A09_01214" 11700 "s59E5H2" 0.4458684 "hypothetical protein"
"JS1_A09_01214" 11700 "s63E6H2" 0.2061463 "hypothetical protein"
"JS1_A09_01217" 11703 "s59E13H2" 0.03151372 "Release factor glutamine methyltransferase"
"JS1_A09_01217" 11703 "s59E25H2" 0 "Release factor glutamine methyltransferase"
"JS1_A09_01217" 11703 "s59E5H2" 0.1269919 "Release factor glutamine methyltransferase"
"JS1_A09_01217" 11703 "s63E6H2" 0.0801773 "Release factor glutamine methyltransferase"
"JS1_A09_01218" 11704 "s59E13H2" 0.3303085 "Peptide chain release factor 1"
"JS1_A09_01218" 11704 "s59E25H2" 0 "Peptide chain release factor 1"
"JS1_A09_01218" 11704 "s59E5H2" 0.06723506 "Peptide chain release factor 1"
"JS1_A09_01218" 11704 "s63E6H2" 0.1157246 "Peptide chain release factor 1"
"JS1_A09_01219" 11705 "s59E13H2" 0.03782055 "hypothetical protein"
"JS1_A09_01219" 11705 "s59E25H2" 0 "hypothetical protein"
"JS1_A09_01219" 11705 "s59E5H2" 0.1167496 "hypothetical protein"
"JS1_A09_01219" 11705 "s63E6H2" 0.2577605 "hypothetical protein"
"JS1_A09_01220" 11706 "s59E13H2" 0.08150835 "Thymidylate synthase ThyX"
"JS1_A09_01220" 11706 "s59E25H2" 0 "Thymidylate synthase ThyX"
"JS1_A09_01220" 11706 "s59E5H2" 0.1773508 "Thymidylate synthase ThyX"
"JS1_A09_01220" 11706 "s63E6H2" 0.2684233 "Thymidylate synthase ThyX"
"JS1_A09_01221" 11707 "s59E13H2" 0.07591086 "Thymidine kinase"
"JS1_A09_01221" 11707 "s59E25H2" 0 "Thymidine kinase"
"JS1_A09_01221" 11707 "s59E5H2" 0.1719172 "Thymidine kinase"
"JS1_A09_01221" 11707 "s63E6H2" 0.2043544 "Thymidine kinase"
"JS1_A09_01222" 11708 "s59E13H2" 0.04136747 "hypothetical protein"
"JS1_A09_01222" 11708 "s59E25H2" 0 "hypothetical protein"
"JS1_A09_01222" 11708 "s59E5H2" 0 "hypothetical protein"
"JS1_A09_01222" 11708 "s63E6H2" 0.07849749 "hypothetical protein"
"JS1_A09_01223" 11709 "s59E13H2" 0.09085685 "2-C-methyl-D-erythritol 4-phosphate cytidylyltransferase"
"JS1_A09_01223" 11709 "s59E25H2" 0 "2-C-methyl-D-erythritol 4-phosphate cytidylyltransferase"
"JS1_A09_01223" 11709 "s59E5H2" 0.1107694 "2-C-methyl-D-erythritol 4-phosphate cytidylyltransferase"
"JS1_A09_01223" 11709 "s63E6H2" 0.1279557 "2-C-methyl-D-erythritol 4-phosphate cytidylyltransferase"
"JS1_A09_01224" 11710 "s59E13H2" 0.06623818 "CTP synthase"
"JS1_A09_01224" 11710 "s59E25H2" 0 "CTP synthase"
"JS1_A09_01224" 11710 "s59E5H2" 0.489437 "CTP synthase"
"JS1_A09_01224" 11710 "s63E6H2" 0.4265786 "CTP synthase"
"JS1_A09_01225" 11711 "s59E13H2" 0.04125461 "metal-dependent hydrolase"
"JS1_A09_01225" 11711 "s59E25H2" 0 "metal-dependent hydrolase"
"JS1_A09_01225" 11711 "s59E5H2" 0.6308368 "metal-dependent hydrolase"
"JS1_A09_01225" 11711 "s63E6H2" 0.5087861 "metal-dependent hydrolase"
"JS1_A09_01226" 11712 "s59E13H2" 0.05658143 "DNA-directed RNA polymerase subunit delta"
"JS1_A09_01226" 11712 "s59E25H2" 0 "DNA-directed RNA polymerase subunit delta"
"JS1_A09_01226" 11712 "s59E5H2" 0.1730332 "DNA-directed RNA polymerase subunit delta"
"JS1_A09_01226" 11712 "s63E6H2" 0.154495 "DNA-directed RNA polymerase subunit delta"

"JS1_A09_01227" 11713 "s59E13H2" 0.0433718 "Carbonic anhydrase precursor"
"JS1_A09_01227" 11713 "s59E25H2" 0 "Carbonic anhydrase precursor"
"JS1_A09_01227" 11713 "s59E5H2" 0.1611142 "Carbonic anhydrase precursor"
"JS1_A09_01227" 11713 "s63E6H2" 0.2263049 "Carbonic anhydrase precursor"
"JS1_A09_01228" 11714 "s59E13H2" 0.04865669 "UvrABC system protein C"
"JS1_A09_01228" 11714 "s59E25H2" 0 "UvrABC system protein C"
"JS1_A09_01228" 11714 "s59E5H2" 0.2312225 "UvrABC system protein C"
"JS1_A09_01228" 11714 "s63E6H2" 0.1617172 "UvrABC system protein C"
"JS1_A09_01229" 11715 "s59E13H2" 0.04540723 "UvrABC system protein A"
"JS1_A09_01229" 11715 "s59E25H2" 0 "UvrABC system protein A"
"JS1_A09_01229" 11715 "s59E5H2" 0.9444038 "UvrABC system protein A"
"JS1_A09_01229" 11715 "s63E6H2" 0.2085694 "UvrABC system protein A"
"JS1_A09_01230" 11716 "s59E13H2" 0.06066532 "UvrABC system protein B"
"JS1_A09_01230" 11716 "s59E25H2" 0 "UvrABC system protein B"
"JS1_A09_01230" 11716 "s59E5H2" 0.776928 "UvrABC system protein B"
"JS1_A09_01230" 11716 "s63E6H2" 0.8196619 "UvrABC system protein B"
"JS1_A09_01231" 11717 "s59E13H2" 0.07082685 "Adenine deaminase"
"JS1_A09_01231" 11717 "s59E25H2" 0 "Adenine deaminase"
"JS1_A09_01231" 11717 "s59E5H2" 1.058211 "Adenine deaminase"
"JS1_A09_01231" 11717 "s63E6H2" 2.198155 "Adenine deaminase"
"JS1_A09_01232" 11718 "s59E13H2" 0.03859672 "Dephospho-CoA kinase"
"JS1_A09_01232" 11718 "s59E25H2" 0 "Dephospho-CoA kinase"
"JS1_A09_01232" 11718 "s59E5H2" 0.2030705 "Dephospho-CoA kinase"
"JS1_A09_01232" 11718 "s63E6H2" 0.6580434 "Dephospho-CoA kinase"
"JS1_A09_01233" 11719 "s59E13H2" 0.02492225 "DNA polymerase I"
"JS1_A09_01233" 11719 "s59E25H2" 0 "DNA polymerase I"
"JS1_A09_01233" 11719 "s59E5H2" 0.1653086 "DNA polymerase I"
"JS1_A09_01233" 11719 "s63E6H2" 1.329188 "DNA polymerase I"
"JS1_A09_01236" 11722 "s59E13H2" 0.1079562 "DNA polymerase III subunit alpha"
"JS1_A09_01236" 11722 "s59E25H2" 0 "DNA polymerase III subunit alpha"
"JS1_A09_01236" 11722 "s59E5H2" 0.5875208 "DNA polymerase III subunit alpha"
"JS1_A09_01236" 11722 "s63E6H2" 0.6263916 "DNA polymerase III subunit alpha"
"JS1_A09_01237" 11723 "s59E13H2" 0.7148209 "hypothetical protein"
"JS1_A09_01237" 11723 "s59E25H2" 0 "hypothetical protein"
"JS1_A09_01237" 11723 "s59E5H2" 0.02916726 "hypothetical protein"
"JS1_A09_01237" 11723 "s63E6H2" 0.4619221 "hypothetical protein"
"JS1_A09_01238" 11724 "s59E13H2" 0.005273653 "6-phosphofructokinase 1"
"JS1_A09_01238" 11724 "s59E25H2" 0 "6-phosphofructokinase 1"
"JS1_A09_01238" 11724 "s59E5H2" 0.5559 "6-phosphofructokinase 1"
"JS1_A09_01238" 11724 "s63E6H2" 1.229188 "6-phosphofructokinase 1"
"JS1_A09_01239" 11725 "s59E13H2" 0.0718183 "Fructose-bisphosphate aldolase"
"JS1_A09_01239" 11725 "s59E25H2" 0 "Fructose-bisphosphate aldolase"
"JS1_A09_01239" 11725 "s59E5H2" 0.8934591 "Fructose-bisphosphate aldolase"
"JS1_A09_01239" 11725 "s63E6H2" 0.2766392 "Fructose-bisphosphate aldolase"
"JS1_A09_01240" 11726 "s59E13H2" 0.04450215 "Modulator of FtsH protease HflK"
"JS1_A09_01240" 11726 "s59E25H2" 0 "Modulator of FtsH protease HflK"
"JS1_A09_01240" 11726 "s59E5H2" 0.7294167 "Modulator of FtsH protease HflK"

"JS1_A09_01240" 11726 "s63E6H2" 0.4588076 "Modulator of FtsH protease HflK"
"JS1_A09_01241" 11727 "s59E13H2" 0.1276906 "Modulator of FtsH protease HflC"
"JS1_A09_01241" 11727 "s59E25H2" 0 "Modulator of FtsH protease HflC"
"JS1_A09_01241" 11727 "s59E5H2" 1.289137 "Modulator of FtsH protease HflC"
"JS1_A09_01241" 11727 "s63E6H2" 0.5368255 "Modulator of FtsH protease HflC"
"JS1_A09_01242" 11728 "s59E13H2" 0.1152141 "Glyoxylate reductase"
"JS1_A09_01242" 11728 "s59E25H2" 0 "Glyoxylate reductase"
"JS1_A09_01242" 11728 "s59E5H2" 0.9316048 "Glyoxylate reductase"
"JS1_A09_01242" 11728 "s63E6H2" 0.9241294 "Glyoxylate reductase"
"JS1_A09_01243" 11729 "s59E13H2" 0.09876942 "hypothetical protein"
"JS1_A09_01243" 11729 "s59E25H2" 0 "hypothetical protein"
"JS1_A09_01243" 11729 "s59E5H2" 0.5963298 "hypothetical protein"
"JS1_A09_01243" 11729 "s63E6H2" 0.541634 "hypothetical protein"
"JS1_A09_01245" 11731 "s59E13H2" 0.0628592 "putative transporter"
"JS1_A09_01245" 11731 "s59E25H2" 0 "putative transporter"
"JS1_A09_01245" 11731 "s59E5H2" 0.3937379 "putative transporter"
"JS1_A09_01245" 11731 "s63E6H2" 0.6237912 "putative transporter"
"JS1_A09_01246" 11732 "s59E13H2" 0.03641289 "Aspartyl/glutamyl-tRNA(Asn/Gln) amidotransferase subunit B"
"JS1_A09_01246" 11732 "s59E25H2" 0 "Aspartyl/glutamyl-tRNA(Asn/Gln) amidotransferase subunit B"
"JS1_A09_01246" 11732 "s59E5H2" 0.4802684 "Aspartyl/glutamyl-tRNA(Asn/Gln) amidotransferase subunit B"
"JS1_A09_01246" 11732 "s63E6H2" 0.4059378 "Aspartyl/glutamyl-tRNA(Asn/Gln) amidotransferase subunit B"
"JS1_A09_01247" 11733 "s59E13H2" 0.1342415 "Glutamyl-tRNA(Gln) amidotransferase subunit A"
"JS1_A09_01247" 11733 "s59E25H2" 0 "Glutamyl-tRNA(Gln) amidotransferase subunit A"
"JS1_A09_01247" 11733 "s59E5H2" 0.6561348 "Glutamyl-tRNA(Gln) amidotransferase subunit A"
"JS1_A09_01247" 11733 "s63E6H2" 0.7392229 "Glutamyl-tRNA(Gln) amidotransferase subunit A"
"JS1_A09_01248" 11734 "s59E13H2" 0.0260953 "Aspartyl/glutamyl-tRNA(Asn/Gln) amidotransferase subunit C"
"JS1_A09_01248" 11734 "s59E25H2" 0 "Aspartyl/glutamyl-tRNA(Asn/Gln) amidotransferase subunit C"
"JS1_A09_01248" 11734 "s59E5H2" 0.1375792 "Aspartyl/glutamyl-tRNA(Asn/Gln) amidotransferase subunit C"
"JS1_A09_01248" 11734 "s63E6H2" 0.06530067 "Aspartyl/glutamyl-tRNA(Asn/Gln) amidotransferase subunit C"
"JS1_A09_01249" 11735 "s59E13H2" 0 "DctM-like transporters"
"JS1_A09_01249" 11735 "s59E25H2" 0 "DctM-like transporters"
"JS1_A09_01249" 11735 "s59E5H2" 0 "DctM-like transporters"
"JS1_A09_01249" 11735 "s63E6H2" 0 "DctM-like transporters"
"JS1_A09_01255" 11741 "s59E13H2" 0.06456427 "hypothetical protein"
"JS1_A09_01255" 11741 "s59E25H2" 0 "hypothetical protein"
"JS1_A09_01255" 11741 "s59E5H2" 0.5061875 "hypothetical protein"
"JS1_A09_01255" 11741 "s63E6H2" 1.276794 "hypothetical protein"
"JS1_A09_01256" 11742 "s59E13H2" 0.04633339 "R-phenyllactate dehydratase activator"
"JS1_A09_01256" 11742 "s59E25H2" 0 "R-phenyllactate dehydratase activator"
"JS1_A09_01256" 11742 "s59E5H2" 0.4172845 "R-phenyllactate dehydratase activator"
"JS1_A09_01256" 11742 "s63E6H2" 0.784323 "R-phenyllactate dehydratase activator"
"JS1_A09_01257" 11743 "s59E13H2" 0.08170675 "hypothetical protein"
"JS1_A09_01257" 11743 "s59E25H2" 0 "hypothetical protein"
"JS1_A09_01257" 11743 "s59E5H2" 0.8562197 "hypothetical protein"
"JS1_A09_01257" 11743 "s63E6H2" 0.09653687 "hypothetical protein"
"JS1_A09_01259" 11745 "s59E13H2" 0.251356 "EamA-like transporter family protein"
"JS1_A09_01259" 11745 "s59E25H2" 0 "EamA-like transporter family protein"

"JS1_A09_01259" 11745 "s59E5H2" 1.109391 "EamA-like transporter family protein"
"JS1_A09_01259" 11745 "s63E6H2" 0.6038364 "EamA-like transporter family protein"
"JS1_A09_01261" 11747 "s59E13H2" 0.09622973 "tRNA dimethylallyltransferase"
"JS1_A09_01261" 11747 "s59E25H2" 0 "tRNA dimethylallyltransferase"
"JS1_A09_01261" 11747 "s59E5H2" 0.3547792 "tRNA dimethylallyltransferase"
"JS1_A09_01261" 11747 "s63E6H2" 0.02020195 "tRNA dimethylallyltransferase"
"JS1_A09_01262" 11748 "s59E13H2" 0.1175438 "(Dimethylallyl)adenosine tRNA methylthiotransferase MiaB"
"JS1_A09_01262" 11748 "s59E25H2" 0 "(Dimethylallyl)adenosine tRNA methylthiotransferase MiaB"
"JS1_A09_01262" 11748 "s59E5H2" 0.7963388 "(Dimethylallyl)adenosine tRNA methylthiotransferase MiaB"
"JS1_A09_01262" 11748 "s63E6H2" 0.08333979 "(Dimethylallyl)adenosine tRNA methylthiotransferase MiaB"
"JS1_A09_01263" 11749 "s59E13H2" 0.09171185 "Ribonuclease Y"
"JS1_A09_01263" 11749 "s59E25H2" 0 "Ribonuclease Y"
"JS1_A09_01263" 11749 "s59E5H2" 0.668975 "Ribonuclease Y"
"JS1_A09_01263" 11749 "s63E6H2" 0.1280939 "Ribonuclease Y"
"JS1_A09_01264" 11750 "s59E13H2" 0.0795374 "Regulatory protein RecX"
"JS1_A09_01264" 11750 "s59E25H2" 0 "Regulatory protein RecX"
"JS1_A09_01264" 11750 "s59E5H2" 0.2389322 "Regulatory protein RecX"
"JS1_A09_01264" 11750 "s63E6H2" 0.2456216 "Regulatory protein RecX"
"JS1_A09_01265" 11751 "s59E13H2" 0.07308036 "DNA repair and recombination protein RadA"
"JS1_A09_01265" 11751 "s59E25H2" 0 "DNA repair and recombination protein RadA"
"JS1_A09_01265" 11751 "s59E5H2" 0.3169984 "DNA repair and recombination protein RadA"
"JS1_A09_01265" 11751 "s63E6H2" 0.4004121 "DNA repair and recombination protein RadA"
"JS1_A09_01266" 11752 "s59E13H2" 0.06939812 "2'-5'-RNA ligase"
"JS1_A09_01266" 11752 "s59E25H2" 0 "2'-5'-RNA ligase"
"JS1_A09_01266" 11752 "s59E5H2" 0.07596597 "2'-5'-RNA ligase"
"JS1_A09_01266" 11752 "s63E6H2" 0.1810949 "2'-5'-RNA ligase"
"JS1_A09_01267" 11753 "s59E13H2" 0.06270006 "hypothetical protein"
"JS1_A09_01267" 11753 "s59E25H2" 0 "hypothetical protein"
"JS1_A09_01267" 11753 "s59E5H2" 0.130333 "hypothetical protein"
"JS1_A09_01267" 11753 "s63E6H2" 0.1832176 "hypothetical protein"
"JS1_A09_01268" 11754 "s59E13H2" 0.4870007 "Helix-turn-helix domain protein"
"JS1_A09_01268" 11754 "s59E25H2" 0 "Helix-turn-helix domain protein"
"JS1_A09_01268" 11754 "s59E5H2" 0.07545896 "Helix-turn-helix domain protein"
"JS1_A09_01268" 11754 "s63E6H2" 0.2098163 "Helix-turn-helix domain protein"
"JS1_A09_01269" 11755 "s59E13H2" 0.9326824 "L-arabinose transporter permease protein"
"JS1_A09_01269" 11755 "s59E25H2" 0 "L-arabinose transporter permease protein"
"JS1_A09_01269" 11755 "s59E5H2" 0.08814379 "L-arabinose transporter permease protein"
"JS1_A09_01269" 11755 "s63E6H2" 0.4489218 "L-arabinose transporter permease protein"
"JS1_A09_01270" 11756 "s59E13H2" 2.167344 "D-allose transporter subunit"
"JS1_A09_01270" 11756 "s59E25H2" 0 "D-allose transporter subunit"
"JS1_A09_01270" 11756 "s59E5H2" 0.2472974 "D-allose transporter subunit"
"JS1_A09_01270" 11756 "s63E6H2" 0.3039375 "D-allose transporter subunit"
"JS1_A09_01271" 11757 "s59E13H2" 1.827246 "Galactose/methyl galactoside import ATP-binding protein MglA"
"JS1_A09_01271" 11757 "s59E25H2" 0 "Galactose/methyl galactoside import ATP-binding protein MglA"
"JS1_A09_01271" 11757 "s59E5H2" 0.2724709 "Galactose/methyl galactoside import ATP-binding protein MglA"
"JS1_A09_01271" 11757 "s63E6H2" 0.1990444 "Galactose/methyl galactoside import ATP-binding protein MglA"
"JS1_A09_01272" 11758 "s59E13H2" 0.8836517 "Membrane lipoprotein TmpC precursor"

"JS1_A09_01272" 11758 "s59E25H2" 0 "Membrane lipoprotein TmpC precursor"
"JS1_A09_01272" 11758 "s59E5H2" 0.2828561 "Membrane lipoprotein TmpC precursor"
"JS1_A09_01272" 11758 "s63E6H2" 0.06168452 "Membrane lipoprotein TmpC precursor"
"JS1_A09_01273" 11759 "s59E13H2" 3.780002 "DNA translocase SpoIIIE"
"JS1_A09_01273" 11759 "s59E25H2" 0 "DNA translocase SpoIIIE"
"JS1_A09_01273" 11759 "s59E5H2" 0.192271 "DNA translocase SpoIIIE"
"JS1_A09_01273" 11759 "s63E6H2" 0.1019359 "DNA translocase SpoIIIE"
"JS1_A09_01274" 11760 "s59E13H2" 4.440818 "Undecaprenyl-diphosphatase"
"JS1_A09_01274" 11760 "s59E25H2" 0 "Undecaprenyl-diphosphatase"
"JS1_A09_01274" 11760 "s59E5H2" 0.2117902 "Undecaprenyl-diphosphatase"
"JS1_A09_01274" 11760 "s63E6H2" 0.142041 "Undecaprenyl-diphosphatase"
"JS1_A09_01275" 11761 "s59E13H2" 4.795472 "hypothetical protein"
"JS1_A09_01275" 11761 "s59E25H2" 0 "hypothetical protein"
"JS1_A09_01275" 11761 "s59E5H2" 0.04884613 "hypothetical protein"
"JS1_A09_01275" 11761 "s63E6H2" 0.1105384 "hypothetical protein"
"JS1_A09_01276" 11762 "s59E13H2" 3.732775 "Deoxyuridine 5'-triphosphate nucleotidohydrolase"
"JS1_A09_01276" 11762 "s59E25H2" 0 "Deoxyuridine 5'-triphosphate nucleotidohydrolase"
"JS1_A09_01276" 11762 "s59E5H2" 0.1454899 "Deoxyuridine 5'-triphosphate nucleotidohydrolase"
"JS1_A09_01276" 11762 "s63E6H2" 0.08361621 "Deoxyuridine 5'-triphosphate nucleotidohydrolase"
"JS1_A09_01277" 11763 "s59E13H2" 1.938737 "Polyribonucleotide nucleotidyltransferase"
"JS1_A09_01277" 11763 "s59E25H2" 0 "Polyribonucleotide nucleotidyltransferase"
"JS1_A09_01277" 11763 "s59E5H2" 0.1067235 "Polyribonucleotide nucleotidyltransferase"
"JS1_A09_01277" 11763 "s63E6H2" 0.02732275 "Polyribonucleotide nucleotidyltransferase"
"JS1_A09_01278" 11764 "s59E13H2" 0.03717378 "30S ribosomal protein S15"
"JS1_A09_01278" 11764 "s59E25H2" 0 "30S ribosomal protein S15"
"JS1_A09_01278" 11764 "s59E5H2" 0 "30S ribosomal protein S15"
"JS1_A09_01278" 11764 "s63E6H2" 0.02861769 "30S ribosomal protein S15"
"JS1_A09_01279" 11765 "s59E13H2" 0.08321282 "Riboflavin biosynthesis protein RibF"
"JS1_A09_01279" 11765 "s59E25H2" 0 "Riboflavin biosynthesis protein RibF"
"JS1_A09_01279" 11765 "s59E5H2" 0.02633971 "Riboflavin biosynthesis protein RibF"
"JS1_A09_01279" 11765 "s63E6H2" 0.02702342 "Riboflavin biosynthesis protein RibF"
"JS1_A09_01280" 11766 "s59E13H2" 0.04846405 "tRNA pseudouridine synthase B"
"JS1_A09_01280" 11766 "s59E25H2" 0 "tRNA pseudouridine synthase B"
"JS1_A09_01280" 11766 "s59E5H2" 0.08081383 "tRNA pseudouridine synthase B"
"JS1_A09_01280" 11766 "s63E6H2" 0.05101488 "tRNA pseudouridine synthase B"
"JS1_A09_01281" 11767 "s59E13H2" 0.03945705 "Bifunctional oligoribonuclease and PAP phosphatase NrnA"
"JS1_A09_01281" 11767 "s59E25H2" 0 "Bifunctional oligoribonuclease and PAP phosphatase NrnA"
"JS1_A09_01281" 11767 "s59E5H2" 0.04765564 "Bifunctional oligoribonuclease and PAP phosphatase NrnA"
"JS1_A09_01281" 11767 "s63E6H2" 0.05552785 "Bifunctional oligoribonuclease and PAP phosphatase NrnA"
"JS1_A09_01282" 11768 "s59E13H2" 0.02081201 "Ribosome-binding factor A"
"JS1_A09_01282" 11768 "s59E25H2" 0 "Ribosome-binding factor A"
"JS1_A09_01282" 11768 "s59E5H2" 0.06974024 "Ribosome-binding factor A"
"JS1_A09_01282" 11768 "s63E6H2" 0.04301775 "Ribosome-binding factor A"
"JS1_A09_01283" 11769 "s59E13H2" 0.03721796 "Translation initiation factor IF-2"
"JS1_A09_01283" 11769 "s59E25H2" 0 "Translation initiation factor IF-2"
"JS1_A09_01283" 11769 "s59E5H2" 0.128653 "Translation initiation factor IF-2"
"JS1_A09_01283" 11769 "s63E6H2" 0.07625808 "Translation initiation factor IF-2"

"JS1_A09_01284" 11770 "s59E13H2" 0.01458742 "hypothetical protein"
"JS1_A09_01284" 11770 "s59E25H2" 0 "hypothetical protein"
"JS1_A09_01284" 11770 "s59E5H2" 0 "hypothetical protein"
"JS1_A09_01284" 11770 "s63E6H2" 0.1006038 "hypothetical protein"
"JS1_A09_01285" 11771 "s59E13H2" 0.2368241 "hypothetical protein"
"JS1_A09_01285" 11771 "s59E25H2" 0 "hypothetical protein"
"JS1_A09_01285" 11771 "s59E5H2" 0.02029835 "hypothetical protein"
"JS1_A09_01285" 11771 "s63E6H2" 0.1820756 "hypothetical protein"
"JS1_A09_01286" 11772 "s59E13H2" 0.7439259 "Threonylcarbamoyladenosine tRNA methylthiotransferase MtaB"
"JS1_A09_01286" 11772 "s59E25H2" 0 "Threonylcarbamoyladenosine tRNA methylthiotransferase MtaB"
"JS1_A09_01286" 11772 "s59E5H2" 0.1806693 "Threonylcarbamoyladenosine tRNA methylthiotransferase MtaB"
"JS1_A09_01286" 11772 "s63E6H2" 0.414599 "Threonylcarbamoyladenosine tRNA methylthiotransferase MtaB"
"JS1_A09_01287" 11773 "s59E13H2" 0.04456732 "Chaperone protein DnaJ"
"JS1_A09_01287" 11773 "s59E25H2" 0 "Chaperone protein DnaJ"
"JS1_A09_01287" 11773 "s59E5H2" 0.03725553 "Chaperone protein DnaJ"
"JS1_A09_01287" 11773 "s63E6H2" 0.4185703 "Chaperone protein DnaJ"
"JS1_A09_01288" 11774 "s59E13H2" 0.2048774 "Chaperone protein DnaK"
"JS1_A09_01288" 11774 "s59E25H2" 0 "Chaperone protein DnaK"
"JS1_A09_01288" 11774 "s59E5H2" 0.08844429 "Chaperone protein DnaK"
"JS1_A09_01288" 11774 "s63E6H2" 0.2931017 "Chaperone protein DnaK"
"JS1_A09_01289" 11775 "s59E13H2" 0.1277672 "heat shock protein GrpE"
"JS1_A09_01289" 11775 "s59E25H2" 0 "heat shock protein GrpE"
"JS1_A09_01289" 11775 "s59E5H2" 0.03187051 "heat shock protein GrpE"
"JS1_A09_01289" 11775 "s63E6H2" 0.1147148 "heat shock protein GrpE"
"JS1_A09_01290" 11776 "s59E13H2" 0.08470726 "Heat-inducible transcription repressor HrcA"
"JS1_A09_01290" 11776 "s59E25H2" 0 "Heat-inducible transcription repressor HrcA"
"JS1_A09_01290" 11776 "s59E5H2" 0.1510425 "Heat-inducible transcription repressor HrcA"
"JS1_A09_01290" 11776 "s63E6H2" 0.1329903 "Heat-inducible transcription repressor HrcA"
"JS1_A09_01291" 11777 "s59E13H2" 0.09092024 "Oxygen-independent coproporphyrinogen-III oxidase 1"
"JS1_A09_01291" 11777 "s59E25H2" 0 "Oxygen-independent coproporphyrinogen-III oxidase 1"
"JS1_A09_01291" 11777 "s59E5H2" 0.07428638 "Oxygen-independent coproporphyrinogen-III oxidase 1"
"JS1_A09_01291" 11777 "s63E6H2" 0.09549442 "Oxygen-independent coproporphyrinogen-III oxidase 1"
"JS1_A09_01292" 11778 "s59E13H2" 0.2135683 "Oxygen-independent coproporphyrinogen-III oxidase 1"
"JS1_A09_01292" 11778 "s59E25H2" 0 "Oxygen-independent coproporphyrinogen-III oxidase 1"
"JS1_A09_01292" 11778 "s59E5H2" 0.2069862 "Oxygen-independent coproporphyrinogen-III oxidase 1"
"JS1_A09_01292" 11778 "s63E6H2" 0.1066178 "Oxygen-independent coproporphyrinogen-III oxidase 1"
"JS1_A09_01293" 11779 "s59E13H2" 1.298419 "Elongation factor 4"
"JS1_A09_01293" 11779 "s59E25H2" 0 "Elongation factor 4"
"JS1_A09_01293" 11779 "s59E5H2" 0.2593699 "Elongation factor 4"
"JS1_A09_01293" 11779 "s63E6H2" 0.1166353 "Elongation factor 4"
"JS1_A09_01294" 11780 "s59E13H2" 0.3713019 "putative peptidoglycan biosynthesis protein MurJ"
"JS1_A09_01294" 11780 "s59E25H2" 0.126195 "putative peptidoglycan biosynthesis protein MurJ"
"JS1_A09_01294" 11780 "s59E5H2" 0.4391943 "putative peptidoglycan biosynthesis protein MurJ"
"JS1_A09_01294" 11780 "s63E6H2" 0.129583 "putative peptidoglycan biosynthesis protein MurJ"
"JS1_A09_01295" 11781 "s59E13H2" 0.1266786 "30S ribosomal protein S20"
"JS1_A09_01295" 11781 "s59E25H2" 0 "30S ribosomal protein S20"
"JS1_A09_01295" 11781 "s59E5H2" 0.3417737 "30S ribosomal protein S20"

"JS1_A09_01295" 11781 "s63E6H2" 0.04292239 "30S ribosomal protein S20"
"JS1_A09_01296" 11782 "s59E13H2" 0.7494482 "DNA polymerase III subunit delta"
"JS1_A09_01296" 11782 "s59E25H2" 0 "DNA polymerase III subunit delta"
"JS1_A09_01296" 11782 "s59E5H2" 0.6418329 "DNA polymerase III subunit delta"
"JS1_A09_01296" 11782 "s63E6H2" 0.05487359 "DNA polymerase III subunit delta"
"JS1_A09_01297" 11783 "s59E13H2" 0.6834318 "ComEC family competence protein"
"JS1_A09_01297" 11783 "s59E25H2" 0 "ComEC family competence protein"
"JS1_A09_01297" 11783 "s59E5H2" 0.9002296 "ComEC family competence protein"
"JS1_A09_01297" 11783 "s63E6H2" 0.07476313 "ComEC family competence protein"
"JS1_A09_01298" 11784 "s59E13H2" 0.467585 "ComE operon protein 1"
"JS1_A09_01298" 11784 "s59E25H2" 0 "ComE operon protein 1"
"JS1_A09_01298" 11784 "s59E5H2" 1.000287 "ComE operon protein 1"
"JS1_A09_01298" 11784 "s63E6H2" 0.08894202 "ComE operon protein 1"
"JS1_A09_01299" 11785 "s59E13H2" 0.6724534 "Folylpolyglutamate synthase"
"JS1_A09_01299" 11785 "s59E25H2" 0.3805175 "Folylpolyglutamate synthase"
"JS1_A09_01299" 11785 "s59E5H2" 0.2616949 "Folylpolyglutamate synthase"
"JS1_A09_01299" 11785 "s63E6H2" 0.07976639 "Folylpolyglutamate synthase"
"JS1_A09_01301" 11787 "s59E13H2" 0 "CoB--CoM heterodisulfide reductase iron-sulfur subunit A"
"JS1_A09_01301" 11787 "s59E25H2" 0 "CoB--CoM heterodisulfide reductase iron-sulfur subunit A"
"JS1_A09_01301" 11787 "s59E5H2" 0.006168848 "CoB--CoM heterodisulfide reductase iron-sulfur subunit A"
"JS1_A09_01301" 11787 "s63E6H2" 0 "CoB--CoM heterodisulfide reductase iron-sulfur subunit A"
"JS1_A09_01302" 11788 "s59E13H2" 2.484695 "putative inner membrane transporter yiJE"
"JS1_A09_01302" 11788 "s59E25H2" 0 "putative inner membrane transporter yiJE"
"JS1_A09_01302" 11788 "s59E5H2" 0.8857259 "putative inner membrane transporter yiJE"
"JS1_A09_01302" 11788 "s63E6H2" 1.163451 "putative inner membrane transporter yiJE"
"JS1_A09_01303" 11789 "s59E13H2" 3.461504 "hypothetical protein"
"JS1_A09_01303" 11789 "s59E25H2" 0 "hypothetical protein"
"JS1_A09_01303" 11789 "s59E5H2" 0.8614234 "hypothetical protein"
"JS1_A09_01303" 11789 "s63E6H2" 0.6814126 "hypothetical protein"
"JS1_A09_01304" 11790 "s59E13H2" 3.717277 "Ribose operon repressor"
"JS1_A09_01304" 11790 "s59E25H2" 0 "Ribose operon repressor"
"JS1_A09_01304" 11790 "s59E5H2" 0.5878345 "Ribose operon repressor"
"JS1_A09_01304" 11790 "s63E6H2" 0.3155904 "Ribose operon repressor"
"JS1_A09_01305" 11791 "s59E13H2" 0.02847192 "Galactose/methyl galactoside import ATP-binding protein MglA"
"JS1_A09_01305" 11791 "s59E25H2" 0 "Galactose/methyl galactoside import ATP-binding protein MglA"
"JS1_A09_01305" 11791 "s59E5H2" 0.4628439 "Galactose/methyl galactoside import ATP-binding protein MglA"
"JS1_A09_01305" 11791 "s63E6H2" 0.1519428 "Galactose/methyl galactoside import ATP-binding protein MglA"
"JS1_A09_01306" 11792 "s59E13H2" 0.01125304 "Inner membrane ABC transporter permease protein YjfF"
"JS1_A09_01306" 11792 "s59E25H2" 0 "Inner membrane ABC transporter permease protein YjfF"
"JS1_A09_01306" 11792 "s59E5H2" 0.4113975 "Inner membrane ABC transporter permease protein YjfF"
"JS1_A09_01306" 11792 "s63E6H2" 0.1605218 "Inner membrane ABC transporter permease protein YjfF"
"JS1_A09_01307" 11793 "s59E13H2" 0.01993244 "Periplasmic binding proteins and sugar binding domain of LacI family protein"
"JS1_A09_01307" 11793 "s59E25H2" 0 "Periplasmic binding proteins and sugar binding domain of LacI family protein"
"JS1_A09_01307" 11793 "s59E5H2" 0.3857908 "Periplasmic binding proteins and sugar binding domain of LacI family protein"
"JS1_A09_01307" 11793 "s63E6H2" 0.2172877 "Periplasmic binding proteins and sugar binding domain of LacI family protein"
"JS1_A09_01308" 11794 "s59E13H2" 5.096001 "Lactose-binding protein precursor"
"JS1_A09_01308" 11794 "s59E25H2" 0 "Lactose-binding protein precursor"

"JS1_A09_01308" 11794 "s59E5H2" 0.6232975 "Lactose-binding protein precursor"
"JS1_A09_01308" 11794 "s63E6H2" 0.4553915 "Lactose-binding protein precursor"
"JS1_A09_01309" 11795 "s59E13H2" 6.18074 "Lactose transport system permease protein LacF"
"JS1_A09_01309" 11795 "s59E25H2" 0 "Lactose transport system permease protein LacF"
"JS1_A09_01309" 11795 "s59E5H2" 0.7220511 "Lactose transport system permease protein LacF"
"JS1_A09_01309" 11795 "s63E6H2" 0.5537137 "Lactose transport system permease protein LacF"
"JS1_A09_01310" 11796 "s59E13H2" 0.9747815 "Lactose transport system permease protein LacG"
"JS1_A09_01310" 11796 "s59E25H2" 0 "Lactose transport system permease protein LacG"
"JS1_A09_01310" 11796 "s59E5H2" 0.617032 "Lactose transport system permease protein LacG"
"JS1_A09_01310" 11796 "s63E6H2" 0.4177921 "Lactose transport system permease protein LacG"
"JS1_A09_01311" 11797 "s59E13H2" 0.1487305 "Beta-galactosidase"
"JS1_A09_01311" 11797 "s59E25H2" 0 "Beta-galactosidase"
"JS1_A09_01311" 11797 "s59E5H2" 0.2618618 "Beta-galactosidase"
"JS1_A09_01311" 11797 "s63E6H2" 0.2421136 "Beta-galactosidase"
"JS1_A09_01312" 11798 "s59E13H2" 4.852442 "Elongation factor Tu domain 2"
"JS1_A09_01312" 11798 "s59E25H2" 0 "Elongation factor Tu domain 2"
"JS1_A09_01312" 11798 "s59E5H2" 2.967627 "Elongation factor Tu domain 2"
"JS1_A09_01312" 11798 "s63E6H2" 1.784625 "Elongation factor Tu domain 2"
"JS1_A09_01313" 11799 "s59E13H2" 5.413827 "Proline--tRNA ligase"
"JS1_A09_01313" 11799 "s59E25H2" 0 "Proline--tRNA ligase"
"JS1_A09_01313" 11799 "s59E5H2" 3.588118 "Proline--tRNA ligase"
"JS1_A09_01313" 11799 "s63E6H2" 2.980769 "Proline--tRNA ligase"
"JS1_A09_01314" 11800 "s59E13H2" 1.081035 "Aspartate racemase"
"JS1_A09_01314" 11800 "s59E25H2" 0 "Aspartate racemase"
"JS1_A09_01314" 11800 "s59E5H2" 0.814989 "Aspartate racemase"
"JS1_A09_01314" 11800 "s63E6H2" 0.8698275 "Aspartate racemase"
"JS1_A09_01315" 11801 "s59E13H2" 0.5494779 "Phosphodiesterase"
"JS1_A09_01315" 11801 "s59E25H2" 0 "Phosphodiesterase"
"JS1_A09_01315" 11801 "s59E5H2" 0.7485719 "Phosphodiesterase"
"JS1_A09_01315" 11801 "s63E6H2" 0.9564993 "Phosphodiesterase"
"JS1_A09_01316" 11802 "s59E13H2" 1.078523 "hypothetical protein"
"JS1_A09_01316" 11802 "s59E25H2" 0 "hypothetical protein"
"JS1_A09_01316" 11802 "s59E5H2" 3.849769 "hypothetical protein"
"JS1_A09_01316" 11802 "s63E6H2" 1.789989 "hypothetical protein"
"JS1_A09_01317" 11803 "s59E13H2" 0.4753623 "Peptidase family S58"
"JS1_A09_01317" 11803 "s59E25H2" 0 "Peptidase family S58"
"JS1_A09_01317" 11803 "s59E5H2" 1.60299 "Peptidase family S58"
"JS1_A09_01317" 11803 "s63E6H2" 2.18831 "Peptidase family S58"
"JS1_A09_01318" 11804 "s59E13H2" 0.007002432 "1,3-propanediol dehydrogenase"
"JS1_A09_01318" 11804 "s59E25H2" 0 "1,3-propanediol dehydrogenase"
"JS1_A09_01318" 11804 "s59E5H2" 1.165389 "1,3-propanediol dehydrogenase"
"JS1_A09_01318" 11804 "s63E6H2" 0.2115368 "1,3-propanediol dehydrogenase"
"JS1_A09_01319" 11805 "s59E13H2" 0.5950986 "Xylulose kinase"
"JS1_A09_01319" 11805 "s59E25H2" 0 "Xylulose kinase"
"JS1_A09_01319" 11805 "s59E5H2" 0.651475 "Xylulose kinase"
"JS1_A09_01319" 11805 "s63E6H2" 1.10131 "Xylulose kinase"
"JS1_A09_01320" 11806 "s59E13H2" 0.002367445 "Glycerol-1-phosphate dehydrogenase [NAD(P)+]"

"JS1_A09_01320" 11806 "s59E25H2" 0 "Glycerol-1-phosphate dehydrogenase [NAD(P)+]"
"JS1_A09_01320" 11806 "s59E5H2" 0.002200327 "Glycerol-1-phosphate dehydrogenase [NAD(P)+]"
"JS1_A09_01320" 11806 "s63E6H2" 0.0882523 "Glycerol-1-phosphate dehydrogenase [NAD(P)+]"
"JS1_A09_01321" 11807 "s59E13H2" 0.004504444 "Xylulose kinase"
"JS1_A09_01321" 11807 "s59E25H2" 0 "Xylulose kinase"
"JS1_A09_01321" 11807 "s59E5H2" 0 "Xylulose kinase"
"JS1_A09_01321" 11807 "s63E6H2" 0.1057061 "Xylulose kinase"
"JS1_A09_01322" 11808 "s59E13H2" 0.003357179 "Putative 2-hydroxyacid dehydrogenase YoaD"
"JS1_A09_01322" 11808 "s59E25H2" 0 "Putative 2-hydroxyacid dehydrogenase YoaD"
"JS1_A09_01322" 11808 "s59E5H2" 0 "Putative 2-hydroxyacid dehydrogenase YoaD"
"JS1_A09_01322" 11808 "s63E6H2" 0.2388552 "Putative 2-hydroxyacid dehydrogenase YoaD"
"JS1_A09_01323" 11809 "s59E13H2" 0.0054135 "L-fuculose phosphate aldolase"
"JS1_A09_01323" 11809 "s59E25H2" 0 "L-fuculose phosphate aldolase"
"JS1_A09_01323" 11809 "s59E5H2" 0 "L-fuculose phosphate aldolase"
"JS1_A09_01323" 11809 "s63E6H2" 0.2352355 "L-fuculose phosphate aldolase"
"JS1_A09_01324" 11810 "s59E13H2" 0.001938367 "L-xylulose/3-keto-L-gulonate kinase"
"JS1_A09_01324" 11810 "s59E25H2" 0 "L-xylulose/3-keto-L-gulonate kinase"
"JS1_A09_01324" 11810 "s59E5H2" 0 "L-xylulose/3-keto-L-gulonate kinase"
"JS1_A09_01324" 11810 "s63E6H2" 0.306397 "L-xylulose/3-keto-L-gulonate kinase"
"JS1_A09_01325" 11811 "s59E13H2" 0 "putative L-threonine 3-dehydrogenase"
"JS1_A09_01325" 11811 "s59E25H2" 0 "putative L-threonine 3-dehydrogenase"
"JS1_A09_01325" 11811 "s59E5H2" 0 "putative L-threonine 3-dehydrogenase"
"JS1_A09_01325" 11811 "s63E6H2" 0.1205246 "putative L-threonine 3-dehydrogenase"
"JS1_A09_01326" 11812 "s59E13H2" 0 "Trehalose/maltose import ATP-binding protein MalK"
"JS1_A09_01326" 11812 "s59E25H2" 0 "Trehalose/maltose import ATP-binding protein MalK"
"JS1_A09_01326" 11812 "s59E5H2" 0 "Trehalose/maltose import ATP-binding protein MalK"
"JS1_A09_01326" 11812 "s63E6H2" 0.03683077 "Trehalose/maltose import ATP-binding protein MalK"
"JS1_A09_01327" 11813 "s59E13H2" 0 "Trehalose/maltose import ATP-binding protein MalK"
"JS1_A09_01327" 11813 "s59E25H2" 0 "Trehalose/maltose import ATP-binding protein MalK"
"JS1_A09_01327" 11813 "s59E5H2" 0 "Trehalose/maltose import ATP-binding protein MalK"
"JS1_A09_01327" 11813 "s63E6H2" 0.02662141 "Trehalose/maltose import ATP-binding protein MalK"
"JS1_A09_01328" 11814 "s59E13H2" 0 "Inner membrane ABC transporter permease protein YcjP"
"JS1_A09_01328" 11814 "s59E25H2" 0 "Inner membrane ABC transporter permease protein YcjP"
"JS1_A09_01328" 11814 "s59E5H2" 0 "Inner membrane ABC transporter permease protein YcjP"
"JS1_A09_01328" 11814 "s63E6H2" 0.06418631 "Inner membrane ABC transporter permease protein YcjP"
"JS1_A09_01329" 11815 "s59E13H2" 0 "Trehalose/maltose transport system permease protein MalF"
"JS1_A09_01329" 11815 "s59E25H2" 0 "Trehalose/maltose transport system permease protein MalF"
"JS1_A09_01329" 11815 "s59E5H2" 0 "Trehalose/maltose transport system permease protein MalF"
"JS1_A09_01329" 11815 "s63E6H2" 0.01083936 "Trehalose/maltose transport system permease protein MalF"
"JS1_A09_01330" 11816 "s59E13H2" 0 "Bacterial extracellular solute-binding protein"
"JS1_A09_01330" 11816 "s59E25H2" 0 "Bacterial extracellular solute-binding protein"
"JS1_A09_01330" 11816 "s59E5H2" 0 "Bacterial extracellular solute-binding protein"
"JS1_A09_01330" 11816 "s63E6H2" 0.008627193 "Bacterial extracellular solute-binding protein"
"JS1_A09_01331" 11817 "s59E13H2" 0.007838336 "PTS-dependent dihydroxyacetone kinase, ADP-binding subunit DhaL"
"JS1_A09_01331" 11817 "s59E25H2" 0 "PTS-dependent dihydroxyacetone kinase, ADP-binding subunit DhaL"
"JS1_A09_01331" 11817 "s59E5H2" 0.01187373 "PTS-dependent dihydroxyacetone kinase, ADP-binding subunit DhaL"
"JS1_A09_01331" 11817 "s63E6H2" 0.01120114 "PTS-dependent dihydroxyacetone kinase, ADP-binding subunit DhaL"

"JS1_A09_01332" 11818 "s59E13H2" 0.01010267 "PTS-dependent dihydroxyacetone kinase, dihydroxyacetone-binding subunit DhaK"
"JS1_A09_01332" 11818 "s59E25H2" 0 "PTS-dependent dihydroxyacetone kinase, dihydroxyacetone-binding subunit DhaK"
"JS1_A09_01332" 11818 "s59E5H2" 0.004073218 "PTS-dependent dihydroxyacetone kinase, dihydroxyacetone-binding subunit DhaK"
"JS1_A09_01332" 11818 "s63E6H2" 0.005154313 "PTS-dependent dihydroxyacetone kinase, dihydroxyacetone-binding subunit DhaK"
"JS1_A09_01333" 11819 "s59E13H2" 0.01897817 "Ribose-5-phosphate isomerase B"
"JS1_A09_01333" 11819 "s59E25H2" 0 "Ribose-5-phosphate isomerase B"
"JS1_A09_01333" 11819 "s59E5H2" 0 "Ribose-5-phosphate isomerase B"
"JS1_A09_01333" 11819 "s63E6H2" 0 "Ribose-5-phosphate isomerase B"
"JS1_A09_01334" 11820 "s59E13H2" 0.03243247 "Glucitol operon repressor"
"JS1_A09_01334" 11820 "s59E25H2" 0 "Glucitol operon repressor"
"JS1_A09_01334" 11820 "s59E5H2" 0 "Glucitol operon repressor"
"JS1_A09_01334" 11820 "s63E6H2" 0.07505645 "Glucitol operon repressor"
"JS1_A09_01335" 11821 "s59E13H2" 0.05055421 "Ferredoxin"
"JS1_A09_01335" 11821 "s59E25H2" 0 "Ferredoxin"
"JS1_A09_01335" 11821 "s59E5H2" 0.3550557 "Ferredoxin"
"JS1_A09_01335" 11821 "s63E6H2" 0.3890809 "Ferredoxin"
"JS1_A09_01336" 11822 "s59E13H2" 0.08774659 "Lipoate-protein ligase A subunit 2"
"JS1_A09_01336" 11822 "s59E25H2" 0 "Lipoate-protein ligase A subunit 2"
"JS1_A09_01336" 11822 "s59E5H2" 0 "Lipoate-protein ligase A subunit 2"
"JS1_A09_01336" 11822 "s63E6H2" 0.2043367 "Lipoate-protein ligase A subunit 2"
"JS1_A09_01338" 11824 "s59E13H2" 0 "hypothetical protein"
"JS1_A09_01338" 11824 "s59E25H2" 0 "hypothetical protein"
"JS1_A09_01338" 11824 "s59E5H2" 0 "hypothetical protein"
"JS1_A09_01338" 11824 "s63E6H2" 0 "hypothetical protein"
"JS1_A09_01346" 11832 "s59E13H2" 0 "hypothetical protein"
"JS1_A09_01346" 11832 "s59E25H2" 0 "hypothetical protein"
"JS1_A09_01346" 11832 "s59E5H2" 0 "hypothetical protein"
"JS1_A09_01346" 11832 "s63E6H2" 0 "hypothetical protein"
"JS1_A09_01347" 11833 "s59E13H2" 0 "Modification methylase DpnIIB"
"JS1_A09_01347" 11833 "s59E25H2" 0 "Modification methylase DpnIIB"
"JS1_A09_01347" 11833 "s59E5H2" 0 "Modification methylase DpnIIB"
"JS1_A09_01347" 11833 "s63E6H2" 0 "Modification methylase DpnIIB"
"JS1_A09_01348" 11834 "s59E13H2" 0 "hypothetical protein"
"JS1_A09_01348" 11834 "s59E25H2" 0 "hypothetical protein"
"JS1_A09_01348" 11834 "s59E5H2" 0 "hypothetical protein"
"JS1_A09_01348" 11834 "s63E6H2" 0 "hypothetical protein"
"JS1_A09_01350" 11836 "s59E13H2" 0 "hypothetical protein"
"JS1_A09_01350" 11836 "s59E25H2" 0 "hypothetical protein"
"JS1_A09_01350" 11836 "s59E5H2" 0 "hypothetical protein"
"JS1_A09_01350" 11836 "s63E6H2" 0 "hypothetical protein"
"JS1_A09_01351" 11837 "s59E13H2" 0 "hypothetical protein"
"JS1_A09_01351" 11837 "s59E25H2" 0 "hypothetical protein"
"JS1_A09_01351" 11837 "s59E5H2" 0 "hypothetical protein"
"JS1_A09_01351" 11837 "s63E6H2" 0 "hypothetical protein"
"JS1_A09_01357" 11843 "s59E13H2" 0.0311109 "Tyrosine recombinase XerC"
"JS1_A09_01357" 11843 "s59E25H2" 0 "Tyrosine recombinase XerC"
"JS1_A09_01357" 11843 "s59E5H2" 0.1381857 "Tyrosine recombinase XerC"

"JS1_A09_01357" 11843 "s63E6H2" 0.047435 "Tyrosine recombinase XerC"
"JS1_A09_01358" 11844 "s59E13H2" 0.02437508 "ATP-dependent protease subunit HslV"
"JS1_A09_01358" 11844 "s59E25H2" 0 "ATP-dependent protease subunit HslV"
"JS1_A09_01358" 11844 "s59E5H2" 0.1103535 "ATP-dependent protease subunit HslV"
"JS1_A09_01358" 11844 "s63E6H2" 0.03106304 "ATP-dependent protease subunit HslV"
"JS1_A09_01359" 11845 "s59E13H2" 0.01717543 "ATP-dependent protease subunit HslV"
"JS1_A09_01359" 11845 "s59E25H2" 0 "ATP-dependent protease subunit HslV"
"JS1_A09_01359" 11845 "s59E5H2" 0.0121588 "ATP-dependent protease subunit HslV"
"JS1_A09_01359" 11845 "s63E6H2" 0.01154532 "ATP-dependent protease subunit HslV"
"JS1_A09_01361" 11847 "s59E13H2" 0.6730491 "NTE family protein RssA"
"JS1_A09_01361" 11847 "s59E25H2" 0 "NTE family protein RssA"
"JS1_A09_01361" 11847 "s59E5H2" 0.1508009 "NTE family protein RssA"
"JS1_A09_01361" 11847 "s63E6H2" 0.4357182 "NTE family protein RssA"
"JS1_A09_01362" 11848 "s59E13H2" 0.8244513 "NTE family protein RssA"
"JS1_A09_01362" 11848 "s59E25H2" 0 "NTE family protein RssA"
"JS1_A09_01362" 11848 "s59E5H2" 0.1609489 "NTE family protein RssA"
"JS1_A09_01362" 11848 "s63E6H2" 0.7290666 "NTE family protein RssA"
"JS1_A09_01363" 11849 "s59E13H2" 0.8486869 "spermidine synthase"
"JS1_A09_01363" 11849 "s59E25H2" 0 "spermidine synthase"
"JS1_A09_01363" 11849 "s59E5H2" 0.6537192 "spermidine synthase"
"JS1_A09_01363" 11849 "s63E6H2" 0.7921054 "spermidine synthase"
"JS1_A09_01364" 11850 "s59E13H2" 0.5080088 "Capsule biosynthesis protein CapA"
"JS1_A09_01364" 11850 "s59E25H2" 0 "Capsule biosynthesis protein CapA"
"JS1_A09_01364" 11850 "s59E5H2" 0.2733471 "Capsule biosynthesis protein CapA"
"JS1_A09_01364" 11850 "s63E6H2" 0.5259958 "Capsule biosynthesis protein CapA"
"JS1_A09_01365" 11851 "s59E13H2" 0.5237825 "hypothetical protein"
"JS1_A09_01365" 11851 "s59E25H2" 0 "hypothetical protein"
"JS1_A09_01365" 11851 "s59E5H2" 1.181534 "hypothetical protein"
"JS1_A09_01365" 11851 "s63E6H2" 1.573953 "hypothetical protein"
"JS1_A09_01366" 11852 "s59E13H2" 1.391214 "Protein-L-isoaspartate O-methyltransferase"
"JS1_A09_01366" 11852 "s59E25H2" 0 "Protein-L-isoaspartate O-methyltransferase"
"JS1_A09_01366" 11852 "s59E5H2" 2.395518 "Protein-L-isoaspartate O-methyltransferase"
"JS1_A09_01366" 11852 "s63E6H2" 1.21147 "Protein-L-isoaspartate O-methyltransferase"
"JS1_A09_01367" 11853 "s59E13H2" 0 "PEGA domain protein"
"JS1_A09_01367" 11853 "s59E25H2" 0 "PEGA domain protein"
"JS1_A09_01367" 11853 "s59E5H2" 1.186691 "PEGA domain protein"
"JS1_A09_01367" 11853 "s63E6H2" 0.392895 "PEGA domain protein"
"JS1_A09_01368" 11854 "s59E13H2" 1.789131 "Inner membrane transport permease YbhS"
"JS1_A09_01368" 11854 "s59E25H2" 0 "Inner membrane transport permease YbhS"
"JS1_A09_01368" 11854 "s59E5H2" 2.237029 "Inner membrane transport permease YbhS"
"JS1_A09_01368" 11854 "s63E6H2" 1.678128 "Inner membrane transport permease YbhS"
"JS1_A09_01369" 11855 "s59E13H2" 2.105887 "putative ABC transporter ATP-binding protein YbhF"
"JS1_A09_01369" 11855 "s59E25H2" 0 "putative ABC transporter ATP-binding protein YbhF"
"JS1_A09_01369" 11855 "s59E5H2" 3.315788 "putative ABC transporter ATP-binding protein YbhF"
"JS1_A09_01369" 11855 "s63E6H2" 1.724866 "putative ABC transporter ATP-binding protein YbhF"
"JS1_A09_01370" 11856 "s59E13H2" 1.147378 "Epoxyqueuosine reductase"
"JS1_A09_01370" 11856 "s59E25H2" 0 "Epoxyqueuosine reductase"

"JS1_A09_01370" 11856 "s59E5H2" 0.6024241 "Epoxyqueuosine reductase"
"JS1_A09_01370" 11856 "s63E6H2" 0.8685089 "Epoxyqueuosine reductase"
"JS1_A09_01371" 11857 "s59E13H2" 1.110606 "Phage shock protein C"
"JS1_A09_01371" 11857 "s59E25H2" 0 "Phage shock protein C"
"JS1_A09_01371" 11857 "s59E5H2" 1.217453 "Phage shock protein C"
"JS1_A09_01371" 11857 "s63E6H2" 0.7692353 "Phage shock protein C"
"JS1_A09_01372" 11858 "s59E13H2" 0.5488438 "Adenosine specific kinase"
"JS1_A09_01372" 11858 "s59E25H2" 0 "Adenosine specific kinase"
"JS1_A09_01372" 11858 "s59E5H2" 1.106493 "Adenosine specific kinase"
"JS1_A09_01372" 11858 "s63E6H2" 0.5570417 "Adenosine specific kinase"
"JS1_A09_01373" 11859 "s59E13H2" 0.5341493 "putative sulfoacetate transporter SauU"
"JS1_A09_01373" 11859 "s59E25H2" 0 "putative sulfoacetate transporter SauU"
"JS1_A09_01373" 11859 "s59E5H2" 0.4839257 "putative sulfoacetate transporter SauU"
"JS1_A09_01373" 11859 "s63E6H2" 0.6806478 "putative sulfoacetate transporter SauU"
"JS1_A09_01374" 11860 "s59E13H2" 0.4973431 "Pyrimidine 5'-nucleotidase YjjG"
"JS1_A09_01374" 11860 "s59E25H2" 0 "Pyrimidine 5'-nucleotidase YjjG"
"JS1_A09_01374" 11860 "s59E5H2" 0.9631726 "Pyrimidine 5'-nucleotidase YjjG"
"JS1_A09_01374" 11860 "s63E6H2" 0.9365297 "Pyrimidine 5'-nucleotidase YjjG"
"JS1_A09_01376" 11862 "s59E13H2" 0 "hypothetical protein"
"JS1_A09_01376" 11862 "s59E25H2" 0 "hypothetical protein"
"JS1_A09_01376" 11862 "s59E5H2" 0.3642815 "hypothetical protein"
"JS1_A09_01376" 11862 "s63E6H2" 0.07823467 "hypothetical protein"
"JS1_A09_01377" 11863 "s59E13H2" 3.724548e-05 "transposase/IS protein"
"JS1_A09_01377" 11863 "s59E25H2" 0 "transposase/IS protein"
"JS1_A09_01377" 11863 "s59E5H2" 1.92338 "transposase/IS protein"
"JS1_A09_01377" 11863 "s63E6H2" 0.4515846 "transposase/IS protein"
"JS1_A09_01378" 11864 "s59E13H2" 0.006809255 "Integrase core domain protein"
"JS1_A09_01378" 11864 "s59E25H2" 0 "Integrase core domain protein"
"JS1_A09_01378" 11864 "s59E5H2" 0.8747088 "Integrase core domain protein"
"JS1_A09_01378" 11864 "s63E6H2" 0.3442732 "Integrase core domain protein"
"JS1_A09_01384" 11870 "s59E13H2" 0.01856505 "Sulfhydrogenase 1 subunit gamma"
"JS1_A09_01384" 11870 "s59E25H2" 0 "Sulfhydrogenase 1 subunit gamma"
"JS1_A09_01384" 11870 "s59E5H2" 0 "Sulfhydrogenase 1 subunit gamma"
"JS1_A09_01384" 11870 "s63E6H2" 0.008582958 "Sulfhydrogenase 1 subunit gamma"
"JS1_A09_01385" 11871 "s59E13H2" 0.2290236 "hypothetical protein"
"JS1_A09_01385" 11871 "s59E25H2" 0 "hypothetical protein"
"JS1_A09_01385" 11871 "s59E5H2" 0 "hypothetical protein"
"JS1_A09_01385" 11871 "s63E6H2" 0.01278764 "hypothetical protein"
"JS1_A09_01386" 11872 "s59E13H2" 0.009631769 "NADP-dependent malic enzyme"
"JS1_A09_01386" 11872 "s59E25H2" 0 "NADP-dependent malic enzyme"
"JS1_A09_01386" 11872 "s59E5H2" 0.01210131 "NADP-dependent malic enzyme"
"JS1_A09_01386" 11872 "s63E6H2" 0.2977019 "NADP-dependent malic enzyme"
"JS1_A09_01387" 11873 "s59E13H2" 0.008315333 "NAD-dependent malic enzyme"
"JS1_A09_01387" 11873 "s59E25H2" 0 "NAD-dependent malic enzyme"
"JS1_A09_01387" 11873 "s59E5H2" 0.1170983 "NAD-dependent malic enzyme"
"JS1_A09_01387" 11873 "s63E6H2" 0.3906286 "NAD-dependent malic enzyme"
"JS1_A09_01388" 11874 "s59E13H2" 0.009948306 "L(+)-tartrate dehydratase subunit alpha"

"JS1_A09_01388" 11874 "s59E25H2" 0 "L(+)-tartrate dehydratase subunit alpha"
"JS1_A09_01388" 11874 "s59E5H2" 0.02014011 "L(+)-tartrate dehydratase subunit alpha"
"JS1_A09_01388" 11874 "s63E6H2" 0.5102004 "L(+)-tartrate dehydratase subunit alpha"
"JS1_A09_01389" 11875 "s59E13H2" 0.003145682 "Fumarate hydratase class I, anaerobic"
"JS1_A09_01389" 11875 "s59E25H2" 0 "Fumarate hydratase class I, anaerobic"
"JS1_A09_01389" 11875 "s59E5H2" 0 "Fumarate hydratase class I, anaerobic"
"JS1_A09_01389" 11875 "s63E6H2" 0.5230927 "Fumarate hydratase class I, anaerobic"
"JS1_A09_01390" 11876 "s59E13H2" 0.7953474 "hypothetical protein"
"JS1_A09_01390" 11876 "s59E25H2" 0 "hypothetical protein"
"JS1_A09_01390" 11876 "s59E5H2" 1.230517 "hypothetical protein"
"JS1_A09_01390" 11876 "s63E6H2" 2.687445 "hypothetical protein"
"JS1_A09_01391" 11877 "s59E13H2" 0.08083823 "Peptidase M15"
"JS1_A09_01391" 11877 "s59E25H2" 0 "Peptidase M15"
"JS1_A09_01391" 11877 "s59E5H2" 0.4852882 "Peptidase M15"
"JS1_A09_01391" 11877 "s63E6H2" 1.757617 "Peptidase M15"
"JS1_A09_01392" 11878 "s59E13H2" 1.326457 "hypothetical protein"
"JS1_A09_01392" 11878 "s59E25H2" 0.7518519 "hypothetical protein"
"JS1_A09_01392" 11878 "s59E5H2" 1.500126 "hypothetical protein"
"JS1_A09_01392" 11878 "s63E6H2" 1.824102 "hypothetical protein"
"JS1_A09_01393" 11879 "s59E13H2" 0.002218746 "hypothetical protein"
"JS1_A09_01393" 11879 "s59E25H2" 0 "hypothetical protein"
"JS1_A09_01393" 11879 "s59E5H2" 2.539961 "hypothetical protein"
"JS1_A09_01393" 11879 "s63E6H2" 0.3777573 "hypothetical protein"
"JS1_A09_01394" 11880 "s59E13H2" 0 "hypothetical protein"
"JS1_A09_01394" 11880 "s59E25H2" 0 "hypothetical protein"
"JS1_A09_01394" 11880 "s59E5H2" 2.520494 "hypothetical protein"
"JS1_A09_01394" 11880 "s63E6H2" 0.5111826 "hypothetical protein"
"JS1_A09_01401" 11887 "s59E13H2" 4.223376 "hypothetical protein"
"JS1_A09_01401" 11887 "s59E25H2" 0 "hypothetical protein"
"JS1_A09_01401" 11887 "s59E5H2" 0.1942531 "hypothetical protein"
"JS1_A09_01401" 11887 "s63E6H2" 0.5449324 "hypothetical protein"
"JS1_A09_01402" 11888 "s59E13H2" 4.255669 "hypothetical protein"
"JS1_A09_01402" 11888 "s59E25H2" 0 "hypothetical protein"
"JS1_A09_01402" 11888 "s59E5H2" 0.2325824 "hypothetical protein"
"JS1_A09_01402" 11888 "s63E6H2" 0.4386194 "hypothetical protein"
"JS1_A09_01403" 11889 "s59E13H2" 0.454551 "hypothetical protein"
"JS1_A09_01403" 11889 "s59E25H2" 0 "hypothetical protein"
"JS1_A09_01403" 11889 "s59E5H2" 2.068451 "hypothetical protein"
"JS1_A09_01403" 11889 "s63E6H2" 0.1163249 "hypothetical protein"
"JS1_A09_01404" 11890 "s59E13H2" 0.00778724 "Phage shock protein C"
"JS1_A09_01404" 11890 "s59E25H2" 0 "Phage shock protein C"
"JS1_A09_01404" 11890 "s59E5H2" 1.923264 "Phage shock protein C"
"JS1_A09_01404" 11890 "s63E6H2" 0.1146915 "Phage shock protein C"
"JS1_A09_01405" 11891 "s59E13H2" 0.1195822 "hypothetical protein"
"JS1_A09_01405" 11891 "s59E25H2" 0 "hypothetical protein"
"JS1_A09_01405" 11891 "s59E5H2" 0.8386355 "hypothetical protein"
"JS1_A09_01405" 11891 "s63E6H2" 0.1418394 "hypothetical protein"

"JS1_A09_01406" 11892 "s59E13H2" 0.05283329 "hypothetical protein"
"JS1_A09_01406" 11892 "s59E25H2" 0 "hypothetical protein"
"JS1_A09_01406" 11892 "s59E5H2" 0.3415101 "hypothetical protein"
"JS1_A09_01406" 11892 "s63E6H2" 0.01294043 "hypothetical protein"
"JS1_A09_01408" 11894 "s59E13H2" 0.1290313 "UvrABC system protein C"
"JS1_A09_01408" 11894 "s59E25H2" 0 "UvrABC system protein C"
"JS1_A09_01408" 11894 "s59E5H2" 0.1178288 "UvrABC system protein C"
"JS1_A09_01408" 11894 "s63E6H2" 2.000769 "UvrABC system protein C"
"JS1_A09_01410" 11896 "s59E13H2" 0.3588799 "B3/4 domain protein"
"JS1_A09_01410" 11896 "s59E25H2" 0 "B3/4 domain protein"
"JS1_A09_01410" 11896 "s59E5H2" 1.226305 "B3/4 domain protein"
"JS1_A09_01410" 11896 "s63E6H2" 2.716635 "B3/4 domain protein"
"JS1_A09_01411" 11897 "s59E13H2" 0 "hypothetical protein"
"JS1_A09_01411" 11897 "s59E25H2" 0 "hypothetical protein"
"JS1_A09_01411" 11897 "s59E5H2" 0.2180224 "hypothetical protein"
"JS1_A09_01411" 11897 "s63E6H2" 0.003680287 "hypothetical protein"
"JS1_A09_01412" 11898 "s59E13H2" 0 "Acryloyl-coenzyme A reductase"
"JS1_A09_01412" 11898 "s59E25H2" 0 "Acryloyl-coenzyme A reductase"
"JS1_A09_01412" 11898 "s59E5H2" 0.4546786 "Acryloyl-coenzyme A reductase"
"JS1_A09_01412" 11898 "s63E6H2" 0.2095213 "Acryloyl-coenzyme A reductase"
"JS1_A09_01413" 11899 "s59E13H2" 0.3972896 "Acetyltransferase (GNAT) family protein"
"JS1_A09_01413" 11899 "s59E25H2" 0 "Acetyltransferase (GNAT) family protein"
"JS1_A09_01413" 11899 "s59E5H2" 0.04078107 "Acetyltransferase (GNAT) family protein"
"JS1_A09_01413" 11899 "s63E6H2" 0.1174952 "Acetyltransferase (GNAT) family protein"
"JS1_A09_01414" 11900 "s59E13H2" 0 "hypothetical protein"
"JS1_A09_01414" 11900 "s59E25H2" 0 "hypothetical protein"
"JS1_A09_01414" 11900 "s59E5H2" 0 "hypothetical protein"
"JS1_A09_01414" 11900 "s63E6H2" 0 "hypothetical protein"
"JS1_A09_01415" 11901 "s59E13H2" 0.02541151 "NADH dehydrogenase"
"JS1_A09_01415" 11901 "s59E25H2" 0 "NADH dehydrogenase"
"JS1_A09_01415" 11901 "s59E5H2" 1.424122 "NADH dehydrogenase"
"JS1_A09_01415" 11901 "s63E6H2" 0.02841485 "NADH dehydrogenase"
"JS1_A09_01416" 11902 "s59E13H2" 0.02321181 "protoporphyrinogen oxidase"
"JS1_A09_01416" 11902 "s59E25H2" 0 "protoporphyrinogen oxidase"
"JS1_A09_01416" 11902 "s59E5H2" 0.1396371 "protoporphyrinogen oxidase"
"JS1_A09_01416" 11902 "s63E6H2" 0.2654739 "protoporphyrinogen oxidase"
"JS1_A09_01418" 11904 "s59E13H2" 0.02281379 "Response regulator PleD"
"JS1_A09_01418" 11904 "s59E25H2" 0 "Response regulator PleD"
"JS1_A09_01418" 11904 "s59E5H2" 0.3394762 "Response regulator PleD"
"JS1_A09_01418" 11904 "s63E6H2" 0.0085086 "Response regulator PleD"
"JS1_A09_01419" 11905 "s59E13H2" 5.680024 "Group II intron-encoded protein LtrA"
"JS1_A09_01419" 11905 "s59E25H2" 0 "Group II intron-encoded protein LtrA"
"JS1_A09_01419" 11905 "s59E5H2" 0.4990997 "Group II intron-encoded protein LtrA"
"JS1_A09_01419" 11905 "s63E6H2" 2.022602 "Group II intron-encoded protein LtrA"
"JS1_A09_01420" 11906 "s59E13H2" 0 "Sulfide dehydrogenase subunit alpha precursor"
"JS1_A09_01420" 11906 "s59E25H2" 0 "Sulfide dehydrogenase subunit alpha precursor"
"JS1_A09_01420" 11906 "s59E5H2" 0.01015782 "Sulfide dehydrogenase subunit alpha precursor"

"JS1_A09_01420" 11906 "s63E6H2" 0 "Sulfide dehydrogenase subunit alpha precursor"
"JS1_C14_00001" 4975 "s59E13H2" 3.908067 "Tripartite ATP-independent periplasmic transporters, DctQ component"
"JS1_C14_00001" 4975 "s59E25H2" 0 "Tripartite ATP-independent periplasmic transporters, DctQ component"
"JS1_C14_00001" 4975 "s59E5H2" 0.9807624 "Tripartite ATP-independent periplasmic transporters, DctQ component"
"JS1_C14_00001" 4975 "s63E6H2" 0.1639394 "Tripartite ATP-independent periplasmic transporters, DctQ component"
"JS1_C14_00002" 4976 "s59E13H2" 9.007492 "Lactate-binding periplasmic protein precursor"
"JS1_C14_00002" 4976 "s59E25H2" 0 "Lactate-binding periplasmic protein precursor"
"JS1_C14_00002" 4976 "s59E5H2" 0.5533013 "Lactate-binding periplasmic protein precursor"
"JS1_C14_00002" 4976 "s63E6H2" 0.1207273 "Lactate-binding periplasmic protein precursor"
"JS1_C14_00003" 4977 "s59E13H2" 7.222968 "hypothetical protein"
"JS1_C14_00003" 4977 "s59E25H2" 0 "hypothetical protein"
"JS1_C14_00003" 4977 "s59E5H2" 0.4965034 "hypothetical protein"
"JS1_C14_00003" 4977 "s63E6H2" 3.467613 "hypothetical protein"
"JS1_C14_00004" 4978 "s59E13H2" 5.133837 "FMN reductase [NAD(P)H]"
"JS1_C14_00004" 4978 "s59E25H2" 0 "FMN reductase [NAD(P)H]"
"JS1_C14_00004" 4978 "s59E5H2" 0.1641398 "FMN reductase [NAD(P)H]"
"JS1_C14_00004" 4978 "s63E6H2" 0.08847559 "FMN reductase [NAD(P)H]"
"JS1_C14_00005" 4979 "s59E13H2" 3.435185 "hypothetical protein"
"JS1_C14_00005" 4979 "s59E25H2" 0 "hypothetical protein"
"JS1_C14_00005" 4979 "s59E5H2" 0.09712434 "hypothetical protein"
"JS1_C14_00005" 4979 "s63E6H2" 0.09266809 "hypothetical protein"
"JS1_C14_00006" 4980 "s59E13H2" 5.231774 "Winged helix-turn-helix transcription repressor, HrcA DNA-binding"
"JS1_C14_00006" 4980 "s59E25H2" 0 "Winged helix-turn-helix transcription repressor, HrcA DNA-binding"
"JS1_C14_00006" 4980 "s59E5H2" 0.479833 "Winged helix-turn-helix transcription repressor, HrcA DNA-binding"
"JS1_C14_00006" 4980 "s63E6H2" 0.2139974 "Winged helix-turn-helix transcription repressor, HrcA DNA-binding"
"JS1_C14_00007" 4981 "s59E13H2" 8.599794 "pyrroloquinoline quinone biosynthesis protein PqqE"
"JS1_C14_00007" 4981 "s59E25H2" 0.3128964 "pyrroloquinoline quinone biosynthesis protein PqqE"
"JS1_C14_00007" 4981 "s59E5H2" 0.6063691 "pyrroloquinoline quinone biosynthesis protein PqqE"
"JS1_C14_00007" 4981 "s63E6H2" 0.5771127 "pyrroloquinoline quinone biosynthesis protein PqqE"
"JS1_C14_00008" 4982 "s59E13H2" 12.11495 "putative inner membrane transporter yiJE"
"JS1_C14_00008" 4982 "s59E25H2" 0 "putative inner membrane transporter yiJE"
"JS1_C14_00008" 4982 "s59E5H2" 0.4090345 "putative inner membrane transporter yiJE"
"JS1_C14_00008" 4982 "s63E6H2" 0.6952709 "putative inner membrane transporter yiJE"
"JS1_C14_00009" 4983 "s59E13H2" 11.29507 "Radical SAM superfamily protein"
"JS1_C14_00009" 4983 "s59E25H2" 0 "Radical SAM superfamily protein"
"JS1_C14_00009" 4983 "s59E5H2" 0.2980371 "Radical SAM superfamily protein"
"JS1_C14_00009" 4983 "s63E6H2" 0.510116 "Radical SAM superfamily protein"
"JS1_C14_00010" 4984 "s59E13H2" 10.16486 "Putative HAD-hydrolase YfnB"
"JS1_C14_00010" 4984 "s59E25H2" 0 "Putative HAD-hydrolase YfnB"
"JS1_C14_00010" 4984 "s59E5H2" 0.3221793 "Putative HAD-hydrolase YfnB"
"JS1_C14_00010" 4984 "s63E6H2" 0.3185825 "Putative HAD-hydrolase YfnB"
"JS1_C14_00011" 4985 "s59E13H2" 13.2849 "Signal peptidase I S"
"JS1_C14_00011" 4985 "s59E25H2" 0 "Signal peptidase I S"
"JS1_C14_00011" 4985 "s59E5H2" 1.21742 "Signal peptidase I S"
"JS1_C14_00011" 4985 "s63E6H2" 0.6068988 "Signal peptidase I S"
"JS1_C14_00013" 4987 "s59E13H2" 0.00538272 "hypothetical protein"
"JS1_C14_00013" 4987 "s59E25H2" 0 "hypothetical protein"

"JS1_C14_00013" 4987 "s59E5H2" 0 "hypothetical protein"
"JS1_C14_00013" 4987 "s63E6H2" 0.07519175 "hypothetical protein"
"JS1_C14_00014" 4988 "s59E13H2" 0.003846305 "Xylose isomerase-like TIM barrel"
"JS1_C14_00014" 4988 "s59E25H2" 0 "Xylose isomerase-like TIM barrel"
"JS1_C14_00014" 4988 "s59E5H2" 0.008803722 "Xylose isomerase-like TIM barrel"
"JS1_C14_00014" 4988 "s63E6H2" 0.07625952 "Xylose isomerase-like TIM barrel"
"JS1_C14_00015" 4989 "s59E13H2" 0.005630718 "putative L-threonine 3-dehydrogenase"
"JS1_C14_00015" 4989 "s59E25H2" 0 "putative L-threonine 3-dehydrogenase"
"JS1_C14_00015" 4989 "s59E5H2" 0.02288391 "putative L-threonine 3-dehydrogenase"
"JS1_C14_00015" 4989 "s63E6H2" 0.06008985 "putative L-threonine 3-dehydrogenase"
"JS1_C14_00016" 4990 "s59E13H2" 0.03800145 "Transaldolase"
"JS1_C14_00016" 4990 "s59E25H2" 0 "Transaldolase"
"JS1_C14_00016" 4990 "s59E5H2" 0.01942945 "Transaldolase"
"JS1_C14_00016" 4990 "s63E6H2" 0.2029432 "Transaldolase"
"JS1_C14_00017" 4991 "s59E13H2" 0.006795694 "4-hydroxy-2-oxo-heptane-1,7-dioate aldolase"
"JS1_C14_00017" 4991 "s59E25H2" 0 "4-hydroxy-2-oxo-heptane-1,7-dioate aldolase"
"JS1_C14_00017" 4991 "s59E5H2" 0 "4-hydroxy-2-oxo-heptane-1,7-dioate aldolase"
"JS1_C14_00017" 4991 "s63E6H2" 0.06732638 "4-hydroxy-2-oxo-heptane-1,7-dioate aldolase"
"JS1_C14_00018" 4992 "s59E13H2" 3.960352 "hypothetical protein"
"JS1_C14_00018" 4992 "s59E25H2" 0 "hypothetical protein"
"JS1_C14_00018" 4992 "s59E5H2" 0.09214721 "hypothetical protein"
"JS1_C14_00018" 4992 "s63E6H2" 0.1474557 "hypothetical protein"
"JS1_C14_00019" 4993 "s59E13H2" 7.916705 "hypothetical protein"
"JS1_C14_00019" 4993 "s59E25H2" 0 "hypothetical protein"
"JS1_C14_00019" 4993 "s59E5H2" 0.3929686 "hypothetical protein"
"JS1_C14_00019" 4993 "s63E6H2" 0.5782613 "hypothetical protein"
"JS1_C14_00020" 4994 "s59E13H2" 3.356369 "hypothetical protein"
"JS1_C14_00020" 4994 "s59E25H2" 0 "hypothetical protein"
"JS1_C14_00020" 4994 "s59E5H2" 0.8859622 "hypothetical protein"
"JS1_C14_00020" 4994 "s63E6H2" 0.6314909 "hypothetical protein"
"JS1_C14_00021" 4995 "s59E13H2" 0.2366558 "Cupin domain protein"
"JS1_C14_00021" 4995 "s59E25H2" 0 "Cupin domain protein"
"JS1_C14_00021" 4995 "s59E5H2" 0.4054381 "Cupin domain protein"
"JS1_C14_00021" 4995 "s63E6H2" 0.0439482 "Cupin domain protein"
"JS1_C14_00022" 4996 "s59E13H2" 0.2265924 "ADP-ribose pyrophosphatase"
"JS1_C14_00022" 4996 "s59E25H2" 0 "ADP-ribose pyrophosphatase"
"JS1_C14_00022" 4996 "s59E5H2" 0.5514971 "ADP-ribose pyrophosphatase"
"JS1_C14_00022" 4996 "s63E6H2" 0.4047829 "ADP-ribose pyrophosphatase"
"JS1_C14_00023" 4997 "s59E13H2" 0.2241466 "hypothetical protein"
"JS1_C14_00023" 4997 "s59E25H2" 0 "hypothetical protein"
"JS1_C14_00023" 4997 "s59E5H2" 0.5972087 "hypothetical protein"
"JS1_C14_00023" 4997 "s63E6H2" 0.5840138 "hypothetical protein"
"JS1_C14_00024" 4998 "s59E13H2" 0.5219359 "hypothetical protein"
"JS1_C14_00024" 4998 "s59E25H2" 0 "hypothetical protein"
"JS1_C14_00024" 4998 "s59E5H2" 0.4645117 "hypothetical protein"
"JS1_C14_00024" 4998 "s63E6H2" 0.6353328 "hypothetical protein"
"JS1_C14_00025" 4999 "s59E13H2" 1.816592 "Putative ski2-type helicase"

"JS1_C14_00025" 4999 "s59E25H2" 0 "Putative ski2-type helicase"
"JS1_C14_00025" 4999 "s59E5H2" 0.6616915 "Putative ski2-type helicase"
"JS1_C14_00025" 4999 "s63E6H2" 0.5978527 "Putative ski2-type helicase"
"JS1_C14_00026" 5000 "s59E13H2" 9.385925 "putative endonuclease 4"
"JS1_C14_00026" 5000 "s59E25H2" 0.2862069 "putative endonuclease 4"
"JS1_C14_00026" 5000 "s59E5H2" 0.2404181 "putative endonuclease 4"
"JS1_C14_00026" 5000 "s63E6H2" 0.2606757 "putative endonuclease 4"
"JS1_C14_00027" 5001 "s59E13H2" 7.613501 "hypothetical protein"
"JS1_C14_00027" 5001 "s59E25H2" 0 "hypothetical protein"
"JS1_C14_00027" 5001 "s59E5H2" 0.2276233 "hypothetical protein"
"JS1_C14_00027" 5001 "s63E6H2" 0.1571323 "hypothetical protein"
"JS1_C14_00029" 5003 "s59E13H2" 4.334707 "hypothetical protein"
"JS1_C14_00029" 5003 "s59E25H2" 0 "hypothetical protein"
"JS1_C14_00029" 5003 "s59E5H2" 0.5573298 "hypothetical protein"
"JS1_C14_00029" 5003 "s63E6H2" 3.66613 "hypothetical protein"
"JS1_C14_00031" 5005 "s59E13H2" 5.580577 "Plasmid pRiA4b ORF-3-like protein"
"JS1_C14_00031" 5005 "s59E25H2" 0 "Plasmid pRiA4b ORF-3-like protein"
"JS1_C14_00031" 5005 "s59E5H2" 0.2099729 "Plasmid pRiA4b ORF-3-like protein"
"JS1_C14_00031" 5005 "s63E6H2" 4.995563 "Plasmid pRiA4b ORF-3-like protein"
"JS1_C14_00032" 5006 "s59E13H2" 0.3890926 "Demethylmenaquinone methyltransferase"
"JS1_C14_00032" 5006 "s59E25H2" 0 "Demethylmenaquinone methyltransferase"
"JS1_C14_00032" 5006 "s59E5H2" 0.05585635 "Demethylmenaquinone methyltransferase"
"JS1_C14_00032" 5006 "s63E6H2" 0.3111556 "Demethylmenaquinone methyltransferase"
"JS1_C14_00033" 5007 "s59E13H2" 5.501252 "Acetyl-CoA decarbonylase/synthase complex subunit gamma"
"JS1_C14_00033" 5007 "s59E25H2" 0 "Acetyl-CoA decarbonylase/synthase complex subunit gamma"
"JS1_C14_00033" 5007 "s59E5H2" 0.1710171 "Acetyl-CoA decarbonylase/synthase complex subunit gamma"
"JS1_C14_00033" 5007 "s63E6H2" 0.2472714 "Acetyl-CoA decarbonylase/synthase complex subunit gamma"
"JS1_C14_00034" 5008 "s59E13H2" 3.028246 "hypothetical protein"
"JS1_C14_00034" 5008 "s59E25H2" 0 "hypothetical protein"
"JS1_C14_00034" 5008 "s59E5H2" 0.1819589 "hypothetical protein"
"JS1_C14_00034" 5008 "s63E6H2" 0.4677994 "hypothetical protein"
"JS1_C14_00035" 5009 "s59E13H2" 1.216309 "Putative membrane protein YdgH"
"JS1_C14_00035" 5009 "s59E25H2" 0 "Putative membrane protein YdgH"
"JS1_C14_00035" 5009 "s59E5H2" 0.2452114 "Putative membrane protein YdgH"
"JS1_C14_00035" 5009 "s63E6H2" 0.1928188 "Putative membrane protein YdgH"
"JS1_C14_00036" 5010 "s59E13H2" 1.679241 "hypothetical protein"
"JS1_C14_00036" 5010 "s59E25H2" 0 "hypothetical protein"
"JS1_C14_00036" 5010 "s59E5H2" 0.2680027 "hypothetical protein"
"JS1_C14_00036" 5010 "s63E6H2" 0.01518941 "hypothetical protein"
"JS1_C14_00039" 5013 "s59E13H2" 0.9865637 "Cysteine/O-acetylserine efflux protein"
"JS1_C14_00039" 5013 "s59E25H2" 0 "Cysteine/O-acetylserine efflux protein"
"JS1_C14_00039" 5013 "s59E5H2" 2.830789 "Cysteine/O-acetylserine efflux protein"
"JS1_C14_00039" 5013 "s63E6H2" 0.2587378 "Cysteine/O-acetylserine efflux protein"
"JS1_C14_00040" 5014 "s59E13H2" 0.599248 "Biotin transporter BioY"
"JS1_C14_00040" 5014 "s59E25H2" 0 "Biotin transporter BioY"
"JS1_C14_00040" 5014 "s59E5H2" 2.004329 "Biotin transporter BioY"
"JS1_C14_00040" 5014 "s63E6H2" 1.153637 "Biotin transporter BioY"

"JS1_C14_00041" 5015 "s59E13H2" 0.6301646 "Pyruvate, phosphate dikinase"
"JS1_C14_00041" 5015 "s59E25H2" 0 "Pyruvate, phosphate dikinase"
"JS1_C14_00041" 5015 "s59E5H2" 3.412649 "Pyruvate, phosphate dikinase"
"JS1_C14_00041" 5015 "s63E6H2" 0.1488139 "Pyruvate, phosphate dikinase"
"JS1_C14_00042" 5016 "s59E13H2" 0.1716357 "hypothetical protein"
"JS1_C14_00042" 5016 "s59E25H2" 0 "hypothetical protein"
"JS1_C14_00042" 5016 "s59E5H2" 0.06304563 "hypothetical protein"
"JS1_C14_00042" 5016 "s63E6H2" 0.06535236 "hypothetical protein"
"JS1_C14_00045" 5019 "s59E13H2" 0.02421017 "Cell division protein DivIB"
"JS1_C14_00045" 5019 "s59E25H2" 0 "Cell division protein DivIB"
"JS1_C14_00045" 5019 "s59E5H2" 0.1982805 "Cell division protein DivIB"
"JS1_C14_00045" 5019 "s63E6H2" 1.15809 "Cell division protein DivIB"
"JS1_C14_00046" 5020 "s59E13H2" 0.1245578 "Cell division protein FtsA"
"JS1_C14_00046" 5020 "s59E25H2" 0 "Cell division protein FtsA"
"JS1_C14_00046" 5020 "s59E5H2" 0.2845422 "Cell division protein FtsA"
"JS1_C14_00046" 5020 "s63E6H2" 1.13379 "Cell division protein FtsA"
"JS1_C14_00047" 5021 "s59E13H2" 0.1214421 "Ribonucleoside-diphosphate reductase NrdZ"
"JS1_C14_00047" 5021 "s59E25H2" 0 "Ribonucleoside-diphosphate reductase NrdZ"
"JS1_C14_00047" 5021 "s59E5H2" 0.1893047 "Ribonucleoside-diphosphate reductase NrdZ"
"JS1_C14_00047" 5021 "s63E6H2" 1.110786 "Ribonucleoside-diphosphate reductase NrdZ"
"JS1_C14_00048" 5022 "s59E13H2" 0.1017062 "Transcriptional repressor NrdR"
"JS1_C14_00048" 5022 "s59E25H2" 0 "Transcriptional repressor NrdR"
"JS1_C14_00048" 5022 "s59E5H2" 0.3950979 "Transcriptional repressor NrdR"
"JS1_C14_00048" 5022 "s63E6H2" 1.638274 "Transcriptional repressor NrdR"
"JS1_C14_00049" 5023 "s59E13H2" 0.2724307 "Cell division protein FtsZ"
"JS1_C14_00049" 5023 "s59E25H2" 0 "Cell division protein FtsZ"
"JS1_C14_00049" 5023 "s59E5H2" 0.5717736 "Cell division protein FtsZ"
"JS1_C14_00049" 5023 "s63E6H2" 1.611827 "Cell division protein FtsZ"
"JS1_C14_00051" 5025 "s59E13H2" 0.8666847 "L-arabinose transport system permease protein AraQ"
"JS1_C14_00051" 5025 "s59E25H2" 0 "L-arabinose transport system permease protein AraQ"
"JS1_C14_00051" 5025 "s59E5H2" 0.5481644 "L-arabinose transport system permease protein AraQ"
"JS1_C14_00051" 5025 "s63E6H2" 0.3690441 "L-arabinose transport system permease protein AraQ"
"JS1_C14_00052" 5026 "s59E13H2" 0.1624717 "hypothetical protein"
"JS1_C14_00052" 5026 "s59E25H2" 0 "hypothetical protein"
"JS1_C14_00052" 5026 "s59E5H2" 0.02231292 "hypothetical protein"
"JS1_C14_00052" 5026 "s63E6H2" 0 "hypothetical protein"
"JS1_C14_00053" 5027 "s59E13H2" 0.2996057 "hypothetical protein"
"JS1_C14_00053" 5027 "s59E25H2" 0 "hypothetical protein"
"JS1_C14_00053" 5027 "s59E5H2" 0.05450611 "hypothetical protein"
"JS1_C14_00053" 5027 "s63E6H2" 0.05128619 "hypothetical protein"
"JS1_C14_00054" 5028 "s59E13H2" 0.4334088 "hypothetical protein"
"JS1_C14_00054" 5028 "s59E25H2" 0 "hypothetical protein"
"JS1_C14_00054" 5028 "s59E5H2" 0.4040998 "hypothetical protein"
"JS1_C14_00054" 5028 "s63E6H2" 1.160585 "hypothetical protein"
"JS1_C14_00055" 5029 "s59E13H2" 0.7522843 "Phospholipase YtpA"
"JS1_C14_00055" 5029 "s59E25H2" 0 "Phospholipase YtpA"
"JS1_C14_00055" 5029 "s59E5H2" 0.2316709 "Phospholipase YtpA"

"JS1_C14_00055" 5029 "s63E6H2" 1.257716 "Phospholipase YtpA"
"JS1_C14_00056" 5030 "s59E13H2" 0.06226911 "Low molecular weight protein-tyrosine-phosphatase YwlE"
"JS1_C14_00056" 5030 "s59E25H2" 0 "Low molecular weight protein-tyrosine-phosphatase YwlE"
"JS1_C14_00056" 5030 "s59E5H2" 0.3827127 "Low molecular weight protein-tyrosine-phosphatase YwlE"
"JS1_C14_00056" 5030 "s63E6H2" 0.0874501 "Low molecular weight protein-tyrosine-phosphatase YwlE"
"JS1_C14_00057" 5031 "s59E13H2" 0.02806643 "Release factor glutamine methyltransferase"
"JS1_C14_00057" 5031 "s59E25H2" 0 "Release factor glutamine methyltransferase"
"JS1_C14_00057" 5031 "s59E5H2" 0.1642149 "Release factor glutamine methyltransferase"
"JS1_C14_00057" 5031 "s63E6H2" 0.07941739 "Release factor glutamine methyltransferase"
"JS1_C14_00058" 5032 "s59E13H2" 0.319931 "Peptide chain release factor 1"
"JS1_C14_00058" 5032 "s59E25H2" 0 "Peptide chain release factor 1"
"JS1_C14_00058" 5032 "s59E5H2" 0.06723506 "Peptide chain release factor 1"
"JS1_C14_00058" 5032 "s63E6H2" 0.11627 "Peptide chain release factor 1"
"JS1_C14_00059" 5033 "s59E13H2" 0.02376494 "hypothetical protein"
"JS1_C14_00059" 5033 "s59E25H2" 0 "hypothetical protein"
"JS1_C14_00059" 5033 "s59E5H2" 0.08327813 "hypothetical protein"
"JS1_C14_00059" 5033 "s63E6H2" 0.0773662 "hypothetical protein"
"JS1_C14_00060" 5034 "s59E13H2" 0.03212287 "hypothetical protein"
"JS1_C14_00060" 5034 "s59E25H2" 0 "hypothetical protein"
"JS1_C14_00060" 5034 "s59E5H2" 0.111935 "hypothetical protein"
"JS1_C14_00060" 5034 "s63E6H2" 0.294388 "hypothetical protein"
"JS1_C14_00061" 5035 "s59E13H2" 0.08139842 "Thymidylate synthase ThyX"
"JS1_C14_00061" 5035 "s59E25H2" 0 "Thymidylate synthase ThyX"
"JS1_C14_00061" 5035 "s59E5H2" 0.1860855 "Thymidylate synthase ThyX"
"JS1_C14_00061" 5035 "s63E6H2" 0.2650081 "Thymidylate synthase ThyX"
"JS1_C14_00062" 5036 "s59E13H2" 0.07591086 "Thymidine kinase"
"JS1_C14_00062" 5036 "s59E25H2" 0 "Thymidine kinase"
"JS1_C14_00062" 5036 "s59E5H2" 0.1913208 "Thymidine kinase"
"JS1_C14_00062" 5036 "s63E6H2" 0.202496 "Thymidine kinase"
"JS1_C14_00063" 5037 "s59E13H2" 0.141963 "50S ribosomal protein L31"
"JS1_C14_00063" 5037 "s59E25H2" 0 "50S ribosomal protein L31"
"JS1_C14_00063" 5037 "s59E5H2" 0.06360549 "50S ribosomal protein L31"
"JS1_C14_00063" 5037 "s63E6H2" 0.04981184 "50S ribosomal protein L31"
"JS1_C14_00064" 5038 "s59E13H2" 0.1697532 "hypothetical protein"
"JS1_C14_00064" 5038 "s59E25H2" 0 "hypothetical protein"
"JS1_C14_00064" 5038 "s59E5H2" 0.1560395 "hypothetical protein"
"JS1_C14_00064" 5038 "s63E6H2" 0.252983 "hypothetical protein"
"JS1_C14_00065" 5039 "s59E13H2" 0.1034379 "2-C-methyl-D-erythritol 4-phosphate cytidylyltransferase"
"JS1_C14_00065" 5039 "s59E25H2" 0 "2-C-methyl-D-erythritol 4-phosphate cytidylyltransferase"
"JS1_C14_00065" 5039 "s59E5H2" 0.1263353 "2-C-methyl-D-erythritol 4-phosphate cytidylyltransferase"
"JS1_C14_00065" 5039 "s63E6H2" 0.1282302 "2-C-methyl-D-erythritol 4-phosphate cytidylyltransferase"
"JS1_C14_00066" 5040 "s59E13H2" 0.06554175 "CTP synthase"
"JS1_C14_00066" 5040 "s59E25H2" 0 "CTP synthase"
"JS1_C14_00066" 5040 "s59E5H2" 0.480575 "CTP synthase"
"JS1_C14_00066" 5040 "s63E6H2" 0.410042 "CTP synthase"
"JS1_C14_00067" 5041 "s59E13H2" 0.04383438 "metal-dependent hydrolase"
"JS1_C14_00067" 5041 "s59E25H2" 0 "metal-dependent hydrolase"

"JS1_C14_00067" 5041 "s59E5H2" 0.6232003 "metal-dependent hydrolase"
"JS1_C14_00067" 5041 "s63E6H2" 0.5160261 "metal-dependent hydrolase"
"JS1_C14_00070" 5044 "s59E13H2" 0.04465267 "Carbonic anhydrase precursor"
"JS1_C14_00070" 5044 "s59E25H2" 0 "Carbonic anhydrase precursor"
"JS1_C14_00070" 5044 "s59E5H2" 0.1520257 "Carbonic anhydrase precursor"
"JS1_C14_00070" 5044 "s63E6H2" 0.2166937 "Carbonic anhydrase precursor"
"JS1_C14_00071" 5045 "s59E13H2" 0.05098449 "UvrABC system protein C"
"JS1_C14_00071" 5045 "s59E25H2" 0 "UvrABC system protein C"
"JS1_C14_00071" 5045 "s59E5H2" 0.2501146 "UvrABC system protein C"
"JS1_C14_00071" 5045 "s63E6H2" 0.1709699 "UvrABC system protein C"
"JS1_C14_00072" 5046 "s59E13H2" 0.04618336 "UvrABC system protein A"
"JS1_C14_00072" 5046 "s59E25H2" 0 "UvrABC system protein A"
"JS1_C14_00072" 5046 "s59E5H2" 0.9584275 "UvrABC system protein A"
"JS1_C14_00072" 5046 "s63E6H2" 0.2130809 "UvrABC system protein A"
"JS1_C14_00073" 5047 "s59E13H2" 0.05872682 "UvrABC system protein B"
"JS1_C14_00073" 5047 "s59E25H2" 0 "UvrABC system protein B"
"JS1_C14_00073" 5047 "s59E5H2" 0.7619514 "UvrABC system protein B"
"JS1_C14_00073" 5047 "s63E6H2" 0.9455657 "UvrABC system protein B"
"JS1_C14_00074" 5048 "s59E13H2" 0.07763221 "Adenine deaminase"
"JS1_C14_00074" 5048 "s59E25H2" 0 "Adenine deaminase"
"JS1_C14_00074" 5048 "s59E5H2" 1.111663 "Adenine deaminase"
"JS1_C14_00074" 5048 "s63E6H2" 2.592687 "Adenine deaminase"
"JS1_C14_00075" 5049 "s59E13H2" 0.03951583 "Dephospho-CoA kinase"
"JS1_C14_00075" 5049 "s59E25H2" 0 "Dephospho-CoA kinase"
"JS1_C14_00075" 5049 "s59E5H2" 0.4646319 "Dephospho-CoA kinase"
"JS1_C14_00075" 5049 "s63E6H2" 0.4982962 "Dephospho-CoA kinase"
"JS1_C14_00076" 5050 "s59E13H2" 0.02547776 "DNA polymerase I"
"JS1_C14_00076" 5050 "s59E25H2" 0 "DNA polymerase I"
"JS1_C14_00076" 5050 "s59E5H2" 0.1700743 "DNA polymerase I"
"JS1_C14_00076" 5050 "s63E6H2" 1.493294 "DNA polymerase I"
"JS1_C14_00078" 5052 "s59E13H2" 0.03486601 "hypothetical protein"
"JS1_C14_00078" 5052 "s59E25H2" 0 "hypothetical protein"
"JS1_C14_00078" 5052 "s59E5H2" 0.2071271 "hypothetical protein"
"JS1_C14_00078" 5052 "s63E6H2" 0.1824813 "hypothetical protein"
"JS1_C14_00081" 5055 "s59E13H2" 0.09516948 "alkanesulfonate transporter substrate-binding subunit"
"JS1_C14_00081" 5055 "s59E25H2" 0 "alkanesulfonate transporter substrate-binding subunit"
"JS1_C14_00081" 5055 "s59E5H2" 0.2763325 "alkanesulfonate transporter substrate-binding subunit"
"JS1_C14_00081" 5055 "s63E6H2" 0.03769032 "alkanesulfonate transporter substrate-binding subunit"
"JS1_C14_00082" 5056 "s59E13H2" 0.07616564 "Glutamate 2,3-aminomutase"
"JS1_C14_00082" 5056 "s59E25H2" 0 "Glutamate 2,3-aminomutase"
"JS1_C14_00082" 5056 "s59E5H2" 0.1361343 "Glutamate 2,3-aminomutase"
"JS1_C14_00082" 5056 "s63E6H2" 0.1421624 "Glutamate 2,3-aminomutase"
"JS1_C14_00083" 5057 "s59E13H2" 0.02055605 "putative HTH-type transcriptional regulator YbbH"
"JS1_C14_00083" 5057 "s59E25H2" 0 "putative HTH-type transcriptional regulator YbbH"
"JS1_C14_00083" 5057 "s59E5H2" 0.1170847 "putative HTH-type transcriptional regulator YbbH"
"JS1_C14_00083" 5057 "s63E6H2" 0.01536187 "putative HTH-type transcriptional regulator YbbH"
"JS1_C14_00084" 5058 "s59E13H2" 0.003450063 "Methenyltetrahydrofolate cyclohydrolase"

"JS1_C14_00084" 5058 "s59E25H2" 0 "Methenyltetrahydrofolate cyclohydrolase"
"JS1_C14_00084" 5058 "s59E5H2" 0.0214088 "Methenyltetrahydrofolate cyclohydrolase"
"JS1_C14_00084" 5058 "s63E6H2" 0.04436815 "Methenyltetrahydrofolate cyclohydrolase"
"JS1_C14_00085" 5059 "s59E13H2" 0.01931528 "hypothetical protein"
"JS1_C14_00085" 5059 "s59E25H2" 0 "hypothetical protein"
"JS1_C14_00085" 5059 "s59E5H2" 0.1322536 "hypothetical protein"
"JS1_C14_00085" 5059 "s63E6H2" 0.1326626 "hypothetical protein"
"JS1_C14_00086" 5060 "s59E13H2" 0.02849422 "Peptide chain release factor 2"
"JS1_C14_00086" 5060 "s59E25H2" 0 "Peptide chain release factor 2"
"JS1_C14_00086" 5060 "s59E5H2" 0.190887 "Peptide chain release factor 2"
"JS1_C14_00086" 5060 "s63E6H2" 0.192413 "Peptide chain release factor 2"
"JS1_C14_00087" 5061 "s59E13H2" 0.03521166 "preprotein translocase subunit SecA"
"JS1_C14_00087" 5061 "s59E25H2" 0 "preprotein translocase subunit SecA"
"JS1_C14_00087" 5061 "s59E5H2" 0.2208356 "preprotein translocase subunit SecA"
"JS1_C14_00087" 5061 "s63E6H2" 0.1128602 "preprotein translocase subunit SecA"
"JS1_C14_00088" 5062 "s59E13H2" 0.02231299 "hypothetical protein"
"JS1_C14_00088" 5062 "s59E25H2" 0 "hypothetical protein"
"JS1_C14_00088" 5062 "s59E5H2" 0.2907679 "hypothetical protein"
"JS1_C14_00088" 5062 "s63E6H2" 0.07533905 "hypothetical protein"
"JS1_C14_00089" 5063 "s59E13H2" 0.01872789 "Cyclic di-GMP phosphodiesterase response regulator RpfG"
"JS1_C14_00089" 5063 "s59E25H2" 0 "Cyclic di-GMP phosphodiesterase response regulator RpfG"
"JS1_C14_00089" 5063 "s59E5H2" 0.1777926 "Cyclic di-GMP phosphodiesterase response regulator RpfG"
"JS1_C14_00089" 5063 "s63E6H2" 0.05136239 "Cyclic di-GMP phosphodiesterase response regulator RpfG"
"JS1_C14_00090" 5064 "s59E13H2" 0.01290649 "hypothetical protein"
"JS1_C14_00090" 5064 "s59E25H2" 0 "hypothetical protein"
"JS1_C14_00090" 5064 "s59E5H2" 0.2687545 "hypothetical protein"
"JS1_C14_00090" 5064 "s63E6H2" 0.1755564 "hypothetical protein"
"JS1_C14_00092" 5066 "s59E13H2" 0.003967264 "Putative sigma-54 modulation protein"
"JS1_C14_00092" 5066 "s59E25H2" 0 "Putative sigma-54 modulation protein"
"JS1_C14_00092" 5066 "s59E5H2" 0.09979222 "Putative sigma-54 modulation protein"
"JS1_C14_00092" 5066 "s63E6H2" 0.1787086 "Putative sigma-54 modulation protein"
"JS1_C14_00093" 5067 "s59E13H2" 0.1180059 "DNA utilization protein GntX"
"JS1_C14_00093" 5067 "s59E25H2" 0 "DNA utilization protein GntX"
"JS1_C14_00093" 5067 "s59E5H2" 0.1975859 "DNA utilization protein GntX"
"JS1_C14_00093" 5067 "s63E6H2" 0.2685663 "DNA utilization protein GntX"
"JS1_C14_00094" 5068 "s59E13H2" 0.0107903 "Adenosylhomocysteinase"
"JS1_C14_00094" 5068 "s59E25H2" 0 "Adenosylhomocysteinase"
"JS1_C14_00094" 5068 "s59E5H2" 0.2158985 "Adenosylhomocysteinase"
"JS1_C14_00094" 5068 "s63E6H2" 0.4022246 "Adenosylhomocysteinase"
"JS1_C14_00095" 5069 "s59E13H2" 0.01723441 "Beta-lactamase hydrolase-like protein"
"JS1_C14_00095" 5069 "s59E25H2" 0 "Beta-lactamase hydrolase-like protein"
"JS1_C14_00095" 5069 "s59E5H2" 0.1692968 "Beta-lactamase hydrolase-like protein"
"JS1_C14_00095" 5069 "s63E6H2" 0.4128706 "Beta-lactamase hydrolase-like protein"
"JS1_C14_00096" 5070 "s59E13H2" 0.03150434 "putative glycerophosphoryl diester phosphodiesterase 1"
"JS1_C14_00096" 5070 "s59E25H2" 0 "putative glycerophosphoryl diester phosphodiesterase 1"
"JS1_C14_00096" 5070 "s59E5H2" 0.05800735 "putative glycerophosphoryl diester phosphodiesterase 1"
"JS1_C14_00096" 5070 "s63E6H2" 0.2855754 "putative glycerophosphoryl diester phosphodiesterase 1"

"JS1_C14_00097" 5071 "s59E13H2" 0.01775995 "D-tyrosyl-tRNA(Tyr) deacylase"
"JS1_C14_00097" 5071 "s59E25H2" 0 "D-tyrosyl-tRNA(Tyr) deacylase"
"JS1_C14_00097" 5071 "s59E5H2" 0.06808928 "D-tyrosyl-tRNA(Tyr) deacylase"
"JS1_C14_00097" 5071 "s63E6H2" 0.2569918 "D-tyrosyl-tRNA(Tyr) deacylase"
"JS1_C14_00098" 5072 "s59E13H2" 0.06656953 "GTP pyrophosphokinase"
"JS1_C14_00098" 5072 "s59E25H2" 0 "GTP pyrophosphokinase"
"JS1_C14_00098" 5072 "s59E5H2" 0.1535622 "GTP pyrophosphokinase"
"JS1_C14_00098" 5072 "s63E6H2" 0.3498273 "GTP pyrophosphokinase"
"JS1_C14_00099" 5073 "s59E13H2" 0.9656209 "Transketolase 2"
"JS1_C14_00099" 5073 "s59E25H2" 0.2518854 "Transketolase 2"
"JS1_C14_00099" 5073 "s59E5H2" 1.623635 "Transketolase 2"
"JS1_C14_00099" 5073 "s63E6H2" 0.8758517 "Transketolase 2"
"JS1_C14_00100" 5074 "s59E13H2" 0.05713098 "protease TldD"
"JS1_C14_00100" 5074 "s59E25H2" 0.1892552 "protease TldD"
"JS1_C14_00100" 5074 "s59E5H2" 1.437361 "protease TldD"
"JS1_C14_00100" 5074 "s63E6H2" 1.229442 "protease TldD"
"JS1_C14_00101" 5075 "s59E13H2" 0.07505401 "protease TldD"
"JS1_C14_00101" 5075 "s59E25H2" 0 "protease TldD"
"JS1_C14_00101" 5075 "s59E5H2" 1.583152 "protease TldD"
"JS1_C14_00101" 5075 "s63E6H2" 0.6863658 "protease TldD"
"JS1_C14_00102" 5076 "s59E13H2" 10.77915 "Lactose transport system permease protein LacF"
"JS1_C14_00102" 5076 "s59E25H2" 0 "Lactose transport system permease protein LacF"
"JS1_C14_00102" 5076 "s59E5H2" 0.7752447 "Lactose transport system permease protein LacF"
"JS1_C14_00102" 5076 "s63E6H2" 2.573389 "Lactose transport system permease protein LacF"
"JS1_C14_00103" 5077 "s59E13H2" 10.59919 "Multiple sugar-binding protein precursor"
"JS1_C14_00103" 5077 "s59E25H2" 0 "Multiple sugar-binding protein precursor"
"JS1_C14_00103" 5077 "s59E5H2" 0.9573655 "Multiple sugar-binding protein precursor"
"JS1_C14_00103" 5077 "s63E6H2" 2.543872 "Multiple sugar-binding protein precursor"
"JS1_C14_00104" 5078 "s59E13H2" 0.04083132 "Inner membrane amino-acid ABC transporter permease protein YecS"
"JS1_C14_00104" 5078 "s59E25H2" 0 "Inner membrane amino-acid ABC transporter permease protein YecS"
"JS1_C14_00104" 5078 "s59E5H2" 0.3446521 "Inner membrane amino-acid ABC transporter permease protein YecS"
"JS1_C14_00104" 5078 "s63E6H2" 0.1507444 "Inner membrane amino-acid ABC transporter permease protein YecS"
"JS1_C14_00105" 5079 "s59E13H2" 0.06141886 "Glutamine transport ATP-binding protein GlnQ"
"JS1_C14_00105" 5079 "s59E25H2" 0 "Glutamine transport ATP-binding protein GlnQ"
"JS1_C14_00105" 5079 "s59E5H2" 0.5542425 "Glutamine transport ATP-binding protein GlnQ"
"JS1_C14_00105" 5079 "s63E6H2" 0.1738537 "Glutamine transport ATP-binding protein GlnQ"
"JS1_C14_00106" 5080 "s59E13H2" 0.1260522 "Inner membrane amino-acid ABC transporter permease protein YecS"
"JS1_C14_00106" 5080 "s59E25H2" 0 "Inner membrane amino-acid ABC transporter permease protein YecS"
"JS1_C14_00106" 5080 "s59E5H2" 0.5660238 "Inner membrane amino-acid ABC transporter permease protein YecS"
"JS1_C14_00106" 5080 "s63E6H2" 0.568037 "Inner membrane amino-acid ABC transporter permease protein YecS"
"JS1_C14_00107" 5081 "s59E13H2" 0.1869909 "Putative ABC transporter arginine-binding protein 2 precursor"
"JS1_C14_00107" 5081 "s59E25H2" 0 "Putative ABC transporter arginine-binding protein 2 precursor"
"JS1_C14_00107" 5081 "s59E5H2" 0.3211312 "Putative ABC transporter arginine-binding protein 2 precursor"
"JS1_C14_00107" 5081 "s63E6H2" 0.3248554 "Putative ABC transporter arginine-binding protein 2 precursor"
"JS1_C14_00108" 5082 "s59E13H2" 0.5009167 "hypothetical protein"
"JS1_C14_00108" 5082 "s59E25H2" 0 "hypothetical protein"
"JS1_C14_00108" 5082 "s59E5H2" 0.2577931 "hypothetical protein"

"JS1_C14_00108" 5082 "s63E6H2" 8.246307 "hypothetical protein"
"JS1_C14_00109" 5083 "s59E13H2" 0.5999607 "Heterodisulfide reductase subunit A-like protein"
"JS1_C14_00109" 5083 "s59E25H2" 0 "Heterodisulfide reductase subunit A-like protein"
"JS1_C14_00109" 5083 "s59E5H2" 1.37508 "Heterodisulfide reductase subunit A-like protein"
"JS1_C14_00109" 5083 "s63E6H2" 16.38721 "Heterodisulfide reductase subunit A-like protein"
"JS1_C14_00111" 5085 "s59E13H2" 0.05461851 "hypothetical protein"
"JS1_C14_00111" 5085 "s59E25H2" 0 "hypothetical protein"
"JS1_C14_00111" 5085 "s59E5H2" 1.225802 "hypothetical protein"
"JS1_C14_00111" 5085 "s63E6H2" 2.268788 "hypothetical protein"
"JS1_C14_00112" 5086 "s59E13H2" 0.0141527 "Pseudouridine kinase"
"JS1_C14_00112" 5086 "s59E25H2" 0 "Pseudouridine kinase"
"JS1_C14_00112" 5086 "s59E5H2" 0.01393196 "Pseudouridine kinase"
"JS1_C14_00112" 5086 "s63E6H2" 0.333638 "Pseudouridine kinase"
"JS1_C14_00113" 5087 "s59E13H2" 0.06493397 "Pseudouridine-5'-phosphate glycosidase"
"JS1_C14_00113" 5087 "s59E25H2" 0 "Pseudouridine-5'-phosphate glycosidase"
"JS1_C14_00113" 5087 "s59E5H2" 0.04615327 "Pseudouridine-5'-phosphate glycosidase"
"JS1_C14_00113" 5087 "s63E6H2" 0.3063605 "Pseudouridine-5'-phosphate glycosidase"
"JS1_C14_00114" 5088 "s59E13H2" 0.04747798 "Nucleotidyltransferase domain protein"
"JS1_C14_00114" 5088 "s59E25H2" 0 "Nucleotidyltransferase domain protein"
"JS1_C14_00114" 5088 "s59E5H2" 0.1075943 "Nucleotidyltransferase domain protein"
"JS1_C14_00114" 5088 "s63E6H2" 0.05468336 "Nucleotidyltransferase domain protein"
"JS1_C14_00115" 5089 "s59E13H2" 0.05904525 "hypothetical protein"
"JS1_C14_00115" 5089 "s59E25H2" 0 "hypothetical protein"
"JS1_C14_00115" 5089 "s59E5H2" 0.03298387 "hypothetical protein"
"JS1_C14_00115" 5089 "s63E6H2" 0.0286252 "hypothetical protein"
"JS1_C14_00116" 5090 "s59E13H2" 0.05010394 "Digeranylgeranylglycerophospholipid reductase"
"JS1_C14_00116" 5090 "s59E25H2" 0 "Digeranylgeranylglycerophospholipid reductase"
"JS1_C14_00116" 5090 "s59E5H2" 0.007710917 "Digeranylgeranylglycerophospholipid reductase"
"JS1_C14_00116" 5090 "s63E6H2" 0.3976692 "Digeranylgeranylglycerophospholipid reductase"
"JS1_C14_00117" 5091 "s59E13H2" 0.0488194 "Glucose-1-phosphate thymidylyltransferase"
"JS1_C14_00117" 5091 "s59E25H2" 0 "Glucose-1-phosphate thymidylyltransferase"
"JS1_C14_00117" 5091 "s59E5H2" 0.1376024 "Glucose-1-phosphate thymidylyltransferase"
"JS1_C14_00117" 5091 "s63E6H2" 0.780221 "Glucose-1-phosphate thymidylyltransferase"
"JS1_C14_00118" 5092 "s59E13H2" 0.1092454 "translocation protein TolB"
"JS1_C14_00118" 5092 "s59E25H2" 0.1255048 "translocation protein TolB"
"JS1_C14_00118" 5092 "s59E5H2" 0.08646761 "translocation protein TolB"
"JS1_C14_00118" 5092 "s63E6H2" 1.920683 "translocation protein TolB"
"JS1_C14_00119" 5093 "s59E13H2" 0.1146367 "Transposase IS200 like protein"
"JS1_C14_00119" 5093 "s59E25H2" 0 "Transposase IS200 like protein"
"JS1_C14_00119" 5093 "s59E5H2" 0.03613058 "Transposase IS200 like protein"
"JS1_C14_00119" 5093 "s63E6H2" 8.556209 "Transposase IS200 like protein"
"JS1_C14_00120" 5094 "s59E13H2" 0.009170126 "Amidohydrolase"
"JS1_C14_00120" 5094 "s59E25H2" 0 "Amidohydrolase"
"JS1_C14_00120" 5094 "s59E5H2" 0.1747645 "Amidohydrolase"
"JS1_C14_00120" 5094 "s63E6H2" 12.65868 "Amidohydrolase"
"JS1_C14_00121" 5095 "s59E13H2" 0.4828264 "Ribosomal protein S6 modification protein"
"JS1_C14_00121" 5095 "s59E25H2" 0 "Ribosomal protein S6 modification protein"

"JS1_C14_00121" 5095 "s59E5H2" 0.9726506 "Ribosomal protein S6 modification protein"
"JS1_C14_00121" 5095 "s63E6H2" 8.47023 "Ribosomal protein S6 modification protein"
"JS1_C14_00122" 5096 "s59E13H2" 12.25848 "putative transporter"
"JS1_C14_00122" 5096 "s59E25H2" 0.1370641 "putative transporter"
"JS1_C14_00122" 5096 "s59E5H2" 0.672399 "putative transporter"
"JS1_C14_00122" 5096 "s63E6H2" 5.594539 "putative transporter"
"JS1_C14_00123" 5097 "s59E13H2" 18.92976 "DNA-binding transcriptional dual regulator Crp"
"JS1_C14_00123" 5097 "s59E25H2" 0.7066895 "DNA-binding transcriptional dual regulator Crp"
"JS1_C14_00123" 5097 "s59E5H2" 1.305865 "DNA-binding transcriptional dual regulator Crp"
"JS1_C14_00123" 5097 "s63E6H2" 0.7928833 "DNA-binding transcriptional dual regulator Crp"
"JS1_C14_00124" 5098 "s59E13H2" 15.31049 "D-inositol 3-phosphate glycosyltransferase"
"JS1_C14_00124" 5098 "s59E25H2" 0 "D-inositol 3-phosphate glycosyltransferase"
"JS1_C14_00124" 5098 "s59E5H2" 1.217265 "D-inositol 3-phosphate glycosyltransferase"
"JS1_C14_00124" 5098 "s63E6H2" 1.334287 "D-inositol 3-phosphate glycosyltransferase"
"JS1_C14_00125" 5099 "s59E13H2" 11.86599 "hypothetical protein"
"JS1_C14_00125" 5099 "s59E25H2" 0 "hypothetical protein"
"JS1_C14_00125" 5099 "s59E5H2" 0.7589835 "hypothetical protein"
"JS1_C14_00125" 5099 "s63E6H2" 0.3616967 "hypothetical protein"
"JS1_C14_00126" 5100 "s59E13H2" 10.44761 "hypothetical protein"
"JS1_C14_00126" 5100 "s59E25H2" 0 "hypothetical protein"
"JS1_C14_00126" 5100 "s59E5H2" 0.5278264 "hypothetical protein"
"JS1_C14_00126" 5100 "s63E6H2" 0.3041896 "hypothetical protein"
"JS1_C14_00127" 5101 "s59E13H2" 10.05631 "Aspartate racemase"
"JS1_C14_00127" 5101 "s59E25H2" 0.6869565 "Aspartate racemase"
"JS1_C14_00127" 5101 "s59E5H2" 1.26569 "Aspartate racemase"
"JS1_C14_00127" 5101 "s63E6H2" 0.299524 "Aspartate racemase"
"JS1_C14_00128" 5102 "s59E13H2" 1.280414 "Thiamine pyrophosphokinase"
"JS1_C14_00128" 5102 "s59E25H2" 0 "Thiamine pyrophosphokinase"
"JS1_C14_00128" 5102 "s59E5H2" 1.3513 "Thiamine pyrophosphokinase"
"JS1_C14_00128" 5102 "s63E6H2" 0.5826544 "Thiamine pyrophosphokinase"
"JS1_C14_00129" 5103 "s59E13H2" 1.332142 "Thiamine transporter ThiT"
"JS1_C14_00129" 5103 "s59E25H2" 0 "Thiamine transporter ThiT"
"JS1_C14_00129" 5103 "s59E5H2" 1.418896 "Thiamine transporter ThiT"
"JS1_C14_00129" 5103 "s63E6H2" 0.8960031 "Thiamine transporter ThiT"
"JS1_C14_00131" 5105 "s59E13H2" 1.055201 "RNA recognition motif. (a.k.a. RRM, RBD, or RNP domain)"
"JS1_C14_00131" 5105 "s59E25H2" 0 "RNA recognition motif. (a.k.a. RRM, RBD, or RNP domain)"
"JS1_C14_00131" 5105 "s59E5H2" 2.541472 "RNA recognition motif. (a.k.a. RRM, RBD, or RNP domain)"
"JS1_C14_00131" 5105 "s63E6H2" 1.143032 "RNA recognition motif. (a.k.a. RRM, RBD, or RNP domain)"
"JS1_C14_00132" 5106 "s59E13H2" 1.101911 "NAD-dependent protein deacetylase"
"JS1_C14_00132" 5106 "s59E25H2" 0 "NAD-dependent protein deacetylase"
"JS1_C14_00132" 5106 "s59E5H2" 1.141306 "NAD-dependent protein deacetylase"
"JS1_C14_00132" 5106 "s63E6H2" 0.1350238 "NAD-dependent protein deacetylase"
"JS1_C14_00133" 5107 "s59E13H2" 0.8132142 "Methionine--tRNA ligase"
"JS1_C14_00133" 5107 "s59E25H2" 0 "Methionine--tRNA ligase"
"JS1_C14_00133" 5107 "s59E5H2" 1.428548 "Methionine--tRNA ligase"
"JS1_C14_00133" 5107 "s63E6H2" 0.1490715 "Methionine--tRNA ligase"
"JS1_C14_00134" 5108 "s59E13H2" 0.001859493 "Quinolinate synthase A"

"JS1_C14_00134" 5108 "s59E25H2" 0 "Quinolinate synthase A"
"JS1_C14_00134" 5108 "s59E5H2" 0.05726695 "Quinolinate synthase A"
"JS1_C14_00134" 5108 "s63E6H2" 0.07873156 "Quinolinate synthase A"
"JS1_C14_00135" 5109 "s59E13H2" 0.1190937 "Thiosulfate sulfurtransferase PspE precursor"
"JS1_C14_00135" 5109 "s59E25H2" 0 "Thiosulfate sulfurtransferase PspE precursor"
"JS1_C14_00135" 5109 "s59E5H2" 0.08187102 "Thiosulfate sulfurtransferase PspE precursor"
"JS1_C14_00135" 5109 "s63E6H2" 0.07789173 "Thiosulfate sulfurtransferase PspE precursor"
"JS1_C14_00136" 5110 "s59E13H2" 0.01353963 "hypothetical protein"
"JS1_C14_00136" 5110 "s59E25H2" 0 "hypothetical protein"
"JS1_C14_00136" 5110 "s59E5H2" 0.1502357 "hypothetical protein"
"JS1_C14_00136" 5110 "s63E6H2" 0.01378232 "hypothetical protein"
"JS1_C14_00137" 5111 "s59E13H2" 0.2700189 "putative ABC transporter ATP-binding protein"
"JS1_C14_00137" 5111 "s59E25H2" 0 "putative ABC transporter ATP-binding protein"
"JS1_C14_00137" 5111 "s59E5H2" 0.1255245 "putative ABC transporter ATP-binding protein"
"JS1_C14_00137" 5111 "s63E6H2" 0.03817899 "putative ABC transporter ATP-binding protein"
"JS1_C14_00138" 5112 "s59E13H2" 0.2384024 "putative ABC transporter ATP-binding protein"
"JS1_C14_00138" 5112 "s59E25H2" 0 "putative ABC transporter ATP-binding protein"
"JS1_C14_00138" 5112 "s59E5H2" 0.1847953 "putative ABC transporter ATP-binding protein"
"JS1_C14_00138" 5112 "s63E6H2" 0.1332508 "putative ABC transporter ATP-binding protein"
"JS1_C14_00139" 5113 "s59E13H2" 1.263141 "Macrolide export ATP-binding/permease protein MacB"
"JS1_C14_00139" 5113 "s59E25H2" 0 "Macrolide export ATP-binding/permease protein MacB"
"JS1_C14_00139" 5113 "s59E5H2" 0.1192691 "Macrolide export ATP-binding/permease protein MacB"
"JS1_C14_00139" 5113 "s63E6H2" 0.4603536 "Macrolide export ATP-binding/permease protein MacB"
"JS1_C14_00140" 5114 "s59E13H2" 0.882316 "hypothetical protein"
"JS1_C14_00140" 5114 "s59E25H2" 0 "hypothetical protein"
"JS1_C14_00140" 5114 "s59E5H2" 0.05651418 "hypothetical protein"
"JS1_C14_00140" 5114 "s63E6H2" 0.1065345 "hypothetical protein"
"JS1_C14_00141" 5115 "s59E13H2" 0.2142931 "YHS domain protein"
"JS1_C14_00141" 5115 "s59E25H2" 0 "YHS domain protein"
"JS1_C14_00141" 5115 "s59E5H2" 0.1694702 "YHS domain protein"
"JS1_C14_00141" 5115 "s63E6H2" 0.1004578 "YHS domain protein"
"JS1_C14_00142" 5116 "s59E13H2" 0.2472369 "putative copper-exporting P-type ATPase A"
"JS1_C14_00142" 5116 "s59E25H2" 0 "putative copper-exporting P-type ATPase A"
"JS1_C14_00142" 5116 "s59E5H2" 0.04262557 "putative copper-exporting P-type ATPase A"
"JS1_C14_00142" 5116 "s63E6H2" 0.1897651 "putative copper-exporting P-type ATPase A"
"JS1_C14_00143" 5117 "s59E13H2" 0.3024573 "Cysteine synthase B"
"JS1_C14_00143" 5117 "s59E25H2" 0 "Cysteine synthase B"
"JS1_C14_00143" 5117 "s59E5H2" 0.3090217 "Cysteine synthase B"
"JS1_C14_00143" 5117 "s63E6H2" 0.381972 "Cysteine synthase B"
"JS1_C14_00144" 5118 "s59E13H2" 0 "putative diguanylate cyclase YedQ"
"JS1_C14_00144" 5118 "s59E25H2" 0 "putative diguanylate cyclase YedQ"
"JS1_C14_00144" 5118 "s59E5H2" 0 "putative diguanylate cyclase YedQ"
"JS1_C14_00144" 5118 "s63E6H2" 0.009867435 "putative diguanylate cyclase YedQ"
"JS1_C14_00145" 5119 "s59E13H2" 0 "putative trifunctional 2-polyprenylphenol hydroxylase/glutamate synthase subunit beta/ferritin domain-containing protein"
"JS1_C14_00145" 5119 "s59E25H2" 0 "putative trifunctional 2-polyprenylphenol hydroxylase/glutamate synthase subunit beta/ferritin domain-containing protein"
"JS1_C14_00145" 5119 "s59E5H2" 0 "putative trifunctional 2-polyprenylphenol hydroxylase/glutamate synthase subunit beta/ferritin domain-containing protein"
"JS1_C14_00145" 5119 "s63E6H2" 0 "putative trifunctional 2-polyprenylphenol hydroxylase/glutamate synthase subunit beta/ferritin domain-containing protein"

"JS1_C14_00146" 5120 "s59E13H2" 0.001751684 "Aerobic respiration control sensor protein ArcB"
"JS1_C14_00146" 5120 "s59E25H2" 0 "Aerobic respiration control sensor protein ArcB"
"JS1_C14_00146" 5120 "s59E5H2" 0.02267855 "Aerobic respiration control sensor protein ArcB"
"JS1_C14_00146" 5120 "s63E6H2" 0.0008791129 "Aerobic respiration control sensor protein ArcB"
"JS1_C14_00147" 5121 "s59E13H2" 0.006288529 "Cyclic di-GMP phosphodiesterase response regulator RpfG"
"JS1_C14_00147" 5121 "s59E25H2" 0 "Cyclic di-GMP phosphodiesterase response regulator RpfG"
"JS1_C14_00147" 5121 "s59E5H2" 0.003501976 "Cyclic di-GMP phosphodiesterase response regulator RpfG"
"JS1_C14_00147" 5121 "s63E6H2" 0.02874361 "Cyclic di-GMP phosphodiesterase response regulator RpfG"
"JS1_C14_00148" 5122 "s59E13H2" 0.007147414 "hypothetical protein"
"JS1_C14_00148" 5122 "s59E25H2" 0 "hypothetical protein"
"JS1_C14_00148" 5122 "s59E5H2" 0.6703571 "hypothetical protein"
"JS1_C14_00148" 5122 "s63E6H2" 0.04719832 "hypothetical protein"
"JS1_C14_00150" 5124 "s59E13H2" 0 "Nucleotidyltransferase domain protein"
"JS1_C14_00150" 5124 "s59E25H2" 0 "Nucleotidyltransferase domain protein"
"JS1_C14_00150" 5124 "s59E5H2" 0.1480434 "Nucleotidyltransferase domain protein"
"JS1_C14_00150" 5124 "s63E6H2" 0 "Nucleotidyltransferase domain protein"
"JS1_C14_00151" 5125 "s59E13H2" 0 "HEPN domain protein"
"JS1_C14_00151" 5125 "s59E25H2" 0 "HEPN domain protein"
"JS1_C14_00151" 5125 "s59E5H2" 0.08548687 "HEPN domain protein"
"JS1_C14_00151" 5125 "s63E6H2" 0 "HEPN domain protein"
"JS1_C14_00152" 5126 "s59E13H2" 0.008251242 "hypothetical protein"
"JS1_C14_00152" 5126 "s59E25H2" 0 "hypothetical protein"
"JS1_C14_00152" 5126 "s59E5H2" 0.002304609 "hypothetical protein"
"JS1_C14_00152" 5126 "s63E6H2" 0 "hypothetical protein"
"JS1_C14_00153" 5127 "s59E13H2" 0.00460026 "Peptidase M15"
"JS1_C14_00153" 5127 "s59E25H2" 0 "Peptidase M15"
"JS1_C14_00153" 5127 "s59E5H2" 0.1121526 "Peptidase M15"
"JS1_C14_00153" 5127 "s63E6H2" 0.01217466 "Peptidase M15"
"JS1_C14_00154" 5128 "s59E13H2" 0.00976121 "hypothetical protein"
"JS1_C14_00154" 5128 "s59E25H2" 0 "hypothetical protein"
"JS1_C14_00154" 5128 "s59E5H2" 0.6495138 "hypothetical protein"
"JS1_C14_00154" 5128 "s63E6H2" 0.03795724 "hypothetical protein"
"JS1_C14_00155" 5129 "s59E13H2" 0.007643508 "hypothetical protein"
"JS1_C14_00155" 5129 "s59E25H2" 0 "hypothetical protein"
"JS1_C14_00155" 5129 "s59E5H2" 0.01187673 "hypothetical protein"
"JS1_C14_00155" 5129 "s63E6H2" 0.02001935 "hypothetical protein"
"JS1_C14_00156" 5130 "s59E13H2" 0 "hypothetical protein"
"JS1_C14_00156" 5130 "s59E25H2" 0 "hypothetical protein"
"JS1_C14_00156" 5130 "s59E5H2" 0 "hypothetical protein"
"JS1_C14_00156" 5130 "s63E6H2" 0.0005066075 "hypothetical protein"
"JS1_C14_00157" 5131 "s59E13H2" 1.363734 "hypothetical protein"
"JS1_C14_00157" 5131 "s59E25H2" 0 "hypothetical protein"
"JS1_C14_00157" 5131 "s59E5H2" 0.201848 "hypothetical protein"
"JS1_C14_00157" 5131 "s63E6H2" 0.05708415 "hypothetical protein"
"JS1_C14_00158" 5132 "s59E13H2" 6.644728 "Bifunctional phosphoglucose/phosphomannose isomerase"
"JS1_C14_00158" 5132 "s59E25H2" 0 "Bifunctional phosphoglucose/phosphomannose isomerase"
"JS1_C14_00158" 5132 "s59E5H2" 0.8040757 "Bifunctional phosphoglucose/phosphomannose isomerase"

"JS1_C14_00158" 5132 "s63E6H2" 0.6102012 "Bifunctional phosphoglucose/phosphomannose isomerase"
"JS1_C14_00159" 5133 "s59E13H2" 6.167849 "putative undecaprenyl-phosphate N-acetylglucosaminyl 1-phosphate transferase"
"JS1_C14_00159" 5133 "s59E25H2" 0 "putative undecaprenyl-phosphate N-acetylglucosaminyl 1-phosphate transferase"
"JS1_C14_00159" 5133 "s59E5H2" 0.6858581 "putative undecaprenyl-phosphate N-acetylglucosaminyl 1-phosphate transferase"
"JS1_C14_00159" 5133 "s63E6H2" 0.303721 "putative undecaprenyl-phosphate N-acetylglucosaminyl 1-phosphate transferase"
"JS1_C14_00160" 5134 "s59E13H2" 4.725153 "UDP-glucose 4-epimerase"
"JS1_C14_00160" 5134 "s59E25H2" 0 "UDP-glucose 4-epimerase"
"JS1_C14_00160" 5134 "s59E5H2" 0.6483792 "UDP-glucose 4-epimerase"
"JS1_C14_00160" 5134 "s63E6H2" 0.6264201 "UDP-glucose 4-epimerase"
"JS1_C14_00161" 5135 "s59E13H2" 0.05294528 "L-2-hydroxyglutarate oxidase LhgO"
"JS1_C14_00161" 5135 "s59E25H2" 0 "L-2-hydroxyglutarate oxidase LhgO"
"JS1_C14_00161" 5135 "s59E5H2" 0.4276543 "L-2-hydroxyglutarate oxidase LhgO"
"JS1_C14_00161" 5135 "s63E6H2" 0.05627556 "L-2-hydroxyglutarate oxidase LhgO"
"JS1_C14_00162" 5136 "s59E13H2" 0.03048501 "Benzene 1,2-dioxygenase system ferredoxin--NAD(+) reductase subunit"
"JS1_C14_00162" 5136 "s59E25H2" 0 "Benzene 1,2-dioxygenase system ferredoxin--NAD(+) reductase subunit"
"JS1_C14_00162" 5136 "s59E5H2" 0.03759071 "Benzene 1,2-dioxygenase system ferredoxin--NAD(+) reductase subunit"
"JS1_C14_00162" 5136 "s63E6H2" 0.01569867 "Benzene 1,2-dioxygenase system ferredoxin--NAD(+) reductase subunit"
"JS1_C14_00163" 5137 "s59E13H2" 0.05738813 "hypothetical protein"
"JS1_C14_00163" 5137 "s59E25H2" 0 "hypothetical protein"
"JS1_C14_00163" 5137 "s59E5H2" 0 "hypothetical protein"
"JS1_C14_00163" 5137 "s63E6H2" 0.02255778 "hypothetical protein"
"JS1_C14_00164" 5138 "s59E13H2" 0.1521235 "Bifunctional protein PyrR"
"JS1_C14_00164" 5138 "s59E25H2" 0 "Bifunctional protein PyrR"
"JS1_C14_00164" 5138 "s59E5H2" 0.09862876 "Bifunctional protein PyrR"
"JS1_C14_00164" 5138 "s63E6H2" 0.1353279 "Bifunctional protein PyrR"
"JS1_C14_00165" 5139 "s59E13H2" 0.008390874 "Dihydroorotate dehydrogenase B (NAD(+)), electron transfer subunit"
"JS1_C14_00165" 5139 "s59E25H2" 0 "Dihydroorotate dehydrogenase B (NAD(+)), electron transfer subunit"
"JS1_C14_00165" 5139 "s59E5H2" 0.02593085 "Dihydroorotate dehydrogenase B (NAD(+)), electron transfer subunit"
"JS1_C14_00165" 5139 "s63E6H2" 0.05224923 "Dihydroorotate dehydrogenase B (NAD(+)), electron transfer subunit"
"JS1_C14_00166" 5140 "s59E13H2" 0.04026204 "Dihydroorotate dehydrogenase B (NAD(+)), catalytic subunit"
"JS1_C14_00166" 5140 "s59E25H2" 0 "Dihydroorotate dehydrogenase B (NAD(+)), catalytic subunit"
"JS1_C14_00166" 5140 "s59E5H2" 0.007880006 "Dihydroorotate dehydrogenase B (NAD(+)), catalytic subunit"
"JS1_C14_00166" 5140 "s63E6H2" 0.02712154 "Dihydroorotate dehydrogenase B (NAD(+)), catalytic subunit"
"JS1_C14_00167" 5141 "s59E13H2" 1.632946 "orotidine 5'-phosphate decarboxylase"
"JS1_C14_00167" 5141 "s59E25H2" 0 "orotidine 5'-phosphate decarboxylase"
"JS1_C14_00167" 5141 "s59E5H2" 0 "orotidine 5'-phosphate decarboxylase"
"JS1_C14_00167" 5141 "s63E6H2" 0.004432815 "orotidine 5'-phosphate decarboxylase"
"JS1_C14_00168" 5142 "s59E13H2" 3.322888 "Argininosuccinate lyase 1"
"JS1_C14_00168" 5142 "s59E25H2" 0 "Argininosuccinate lyase 1"
"JS1_C14_00168" 5142 "s59E5H2" 0.02123972 "Argininosuccinate lyase 1"
"JS1_C14_00168" 5142 "s63E6H2" 0.06325104 "Argininosuccinate lyase 1"
"JS1_C14_00169" 5143 "s59E13H2" 2.948205 "Argininosuccinate synthase"
"JS1_C14_00169" 5143 "s59E25H2" 0 "Argininosuccinate synthase"
"JS1_C14_00169" 5143 "s59E5H2" 0.1392949 "Argininosuccinate synthase"
"JS1_C14_00169" 5143 "s63E6H2" 0.1147724 "Argininosuccinate synthase"
"JS1_C14_00170" 5144 "s59E13H2" 1.667082 "Acetylornithine aminotransferase"
"JS1_C14_00170" 5144 "s59E25H2" 0 "Acetylornithine aminotransferase"

"JS1_C14_00170" 5144 "s59E5H2" 0.07011472 "Acetylornithine aminotransferase"
"JS1_C14_00170" 5144 "s63E6H2" 0.07022064 "Acetylornithine aminotransferase"
"JS1_C14_00171" 5145 "s59E13H2" 0.2159625 "Acetylglutamate kinase"
"JS1_C14_00171" 5145 "s59E25H2" 0 "Acetylglutamate kinase"
"JS1_C14_00171" 5145 "s59E5H2" 0.03233156 "Acetylglutamate kinase"
"JS1_C14_00171" 5145 "s63E6H2" 0.1230057 "Acetylglutamate kinase"
"JS1_C14_00172" 5146 "s59E13H2" 0.1089007 "Arginine biosynthesis bifunctional protein ArgJ"
"JS1_C14_00172" 5146 "s59E25H2" 0 "Arginine biosynthesis bifunctional protein ArgJ"
"JS1_C14_00172" 5146 "s59E5H2" 0.05628255 "Arginine biosynthesis bifunctional protein ArgJ"
"JS1_C14_00172" 5146 "s63E6H2" 0.192517 "Arginine biosynthesis bifunctional protein ArgJ"
"JS1_C14_00173" 5147 "s59E13H2" 0.739578 "preprotein translocase subunit SecY"
"JS1_C14_00173" 5147 "s59E25H2" 0 "preprotein translocase subunit SecY"
"JS1_C14_00173" 5147 "s59E5H2" 0.1182649 "preprotein translocase subunit SecY"
"JS1_C14_00173" 5147 "s63E6H2" 0.8928753 "preprotein translocase subunit SecY"
"JS1_C14_00174" 5148 "s59E13H2" 0.1468154 "Adenylate kinase"
"JS1_C14_00174" 5148 "s59E25H2" 0 "Adenylate kinase"
"JS1_C14_00174" 5148 "s59E5H2" 0.02779154 "Adenylate kinase"
"JS1_C14_00174" 5148 "s63E6H2" 0.96473 "Adenylate kinase"
"JS1_C14_00175" 5149 "s59E13H2" 0.1640683 "Translation initiation factor IF-1"
"JS1_C14_00175" 5149 "s59E25H2" 0 "Translation initiation factor IF-1"
"JS1_C14_00175" 5149 "s59E5H2" 0.07805649 "Translation initiation factor IF-1"
"JS1_C14_00175" 5149 "s63E6H2" 0.7242517 "Translation initiation factor IF-1"
"JS1_C14_00176" 5150 "s59E13H2" 0.3360086 "50S ribosomal protein L36"
"JS1_C14_00176" 5150 "s59E25H2" 0 "50S ribosomal protein L36"
"JS1_C14_00176" 5150 "s59E5H2" 0.0714979 "50S ribosomal protein L36"
"JS1_C14_00176" 5150 "s63E6H2" 0.8852381 "50S ribosomal protein L36"
"JS1_C14_00177" 5151 "s59E13H2" 0.2417344 "30S ribosomal protein S13"
"JS1_C14_00177" 5151 "s59E25H2" 0 "30S ribosomal protein S13"
"JS1_C14_00177" 5151 "s59E5H2" 0.1293079 "30S ribosomal protein S13"
"JS1_C14_00177" 5151 "s63E6H2" 1.263302 "30S ribosomal protein S13"
"JS1_C14_00178" 5152 "s59E13H2" 0.2085448 "30S ribosomal protein S11"
"JS1_C14_00178" 5152 "s59E25H2" 0 "30S ribosomal protein S11"
"JS1_C14_00178" 5152 "s59E5H2" 0.1413205 "30S ribosomal protein S11"
"JS1_C14_00178" 5152 "s63E6H2" 0.9587316 "30S ribosomal protein S11"
"JS1_C14_00179" 5153 "s59E13H2" 0.2795203 "30S ribosomal protein S4"
"JS1_C14_00179" 5153 "s59E25H2" 0 "30S ribosomal protein S4"
"JS1_C14_00179" 5153 "s59E5H2" 0.1449836 "30S ribosomal protein S4"
"JS1_C14_00179" 5153 "s63E6H2" 1.151598 "30S ribosomal protein S4"
"JS1_C14_00180" 5154 "s59E13H2" 0.2015461 "DNA-directed RNA polymerase subunit alpha"
"JS1_C14_00180" 5154 "s59E25H2" 0 "DNA-directed RNA polymerase subunit alpha"
"JS1_C14_00180" 5154 "s59E5H2" 0.142707 "DNA-directed RNA polymerase subunit alpha"
"JS1_C14_00180" 5154 "s63E6H2" 1.313625 "DNA-directed RNA polymerase subunit alpha"
"JS1_C14_00181" 5155 "s59E13H2" 0.284747 "50S ribosomal protein L17"
"JS1_C14_00181" 5155 "s59E25H2" 0 "50S ribosomal protein L17"
"JS1_C14_00181" 5155 "s59E5H2" 0.198525 "50S ribosomal protein L17"
"JS1_C14_00181" 5155 "s63E6H2" 2.565254 "50S ribosomal protein L17"
"JS1_C14_00182" 5156 "s59E13H2" 0.286736 "Energy-coupling factor transporter ATP-binding protein EcfA1"

"JS1_C14_00182" 5156 "s59E25H2" 0 "Energy-coupling factor transporter ATP-binding protein EcfA1"
"JS1_C14_00182" 5156 "s59E5H2" 0.220441 "Energy-coupling factor transporter ATP-binding protein EcfA1"
"JS1_C14_00182" 5156 "s63E6H2" 3.153356 "Energy-coupling factor transporter ATP-binding protein EcfA1"
"JS1_C14_00183" 5157 "s59E13H2" 0.2829484 "Energy-coupling factor transporter ATP-binding protein EcfA2"
"JS1_C14_00183" 5157 "s59E25H2" 0 "Energy-coupling factor transporter ATP-binding protein EcfA2"
"JS1_C14_00183" 5157 "s59E5H2" 0.5764172 "Energy-coupling factor transporter ATP-binding protein EcfA2"
"JS1_C14_00183" 5157 "s63E6H2" 1.786517 "Energy-coupling factor transporter ATP-binding protein EcfA2"
"JS1_C14_00184" 5158 "s59E13H2" 0.2768378 "Energy-coupling factor transporter transmembrane protein EcfT"
"JS1_C14_00184" 5158 "s59E25H2" 0 "Energy-coupling factor transporter transmembrane protein EcfT"
"JS1_C14_00184" 5158 "s59E5H2" 0.5534336 "Energy-coupling factor transporter transmembrane protein EcfT"
"JS1_C14_00184" 5158 "s63E6H2" 0.9904214 "Energy-coupling factor transporter transmembrane protein EcfT"
"JS1_C14_00185" 5159 "s59E13H2" 0.2517142 "tRNA pseudouridine synthase A"
"JS1_C14_00185" 5159 "s59E25H2" 0 "tRNA pseudouridine synthase A"
"JS1_C14_00185" 5159 "s59E5H2" 0.3544228 "tRNA pseudouridine synthase A"
"JS1_C14_00185" 5159 "s63E6H2" 0.9383184 "tRNA pseudouridine synthase A"
"JS1_C14_00186" 5160 "s59E13H2" 0.1725183 "50S ribosomal protein L13"
"JS1_C14_00186" 5160 "s59E25H2" 0 "50S ribosomal protein L13"
"JS1_C14_00186" 5160 "s59E5H2" 0.4568588 "50S ribosomal protein L13"
"JS1_C14_00186" 5160 "s63E6H2" 1.607044 "50S ribosomal protein L13"
"JS1_C14_00187" 5161 "s59E13H2" 0.1249529 "30S ribosomal protein S9"
"JS1_C14_00187" 5161 "s59E25H2" 0 "30S ribosomal protein S9"
"JS1_C14_00187" 5161 "s59E5H2" 0.4501334 "30S ribosomal protein S9"
"JS1_C14_00187" 5161 "s63E6H2" 1.291532 "30S ribosomal protein S9"
"JS1_C14_00188" 5162 "s59E13H2" 0.2094343 "alanine racemase"
"JS1_C14_00188" 5162 "s59E25H2" 0 "alanine racemase"
"JS1_C14_00188" 5162 "s59E5H2" 0.4023994 "alanine racemase"
"JS1_C14_00188" 5162 "s63E6H2" 1.835624 "alanine racemase"
"JS1_C14_00189" 5163 "s59E13H2" 0.1780423 "Ornithine carbamoyltransferase"
"JS1_C14_00189" 5163 "s59E25H2" 0 "Ornithine carbamoyltransferase"
"JS1_C14_00189" 5163 "s59E5H2" 0.3555982 "Ornithine carbamoyltransferase"
"JS1_C14_00189" 5163 "s63E6H2" 2.30437 "Ornithine carbamoyltransferase"
"JS1_C14_00190" 5164 "s59E13H2" 0.1850531 "DNA integrity scanning protein DisA"
"JS1_C14_00190" 5164 "s59E25H2" 0.5950617 "DNA integrity scanning protein DisA"
"JS1_C14_00190" 5164 "s59E5H2" 0.1268318 "DNA integrity scanning protein DisA"
"JS1_C14_00190" 5164 "s63E6H2" 2.902418 "DNA integrity scanning protein DisA"
"JS1_C14_00191" 5165 "s59E13H2" 0.03242106 "MmgE/PrpD family protein"
"JS1_C14_00191" 5165 "s59E25H2" 0 "MmgE/PrpD family protein"
"JS1_C14_00191" 5165 "s59E5H2" 0.5078015 "MmgE/PrpD family protein"
"JS1_C14_00191" 5165 "s63E6H2" 0 "MmgE/PrpD family protein"
"JS1_C14_00192" 5166 "s59E13H2" 0.009947947 "alkanesulfonate transporter substrate-binding subunit"
"JS1_C14_00192" 5166 "s59E25H2" 0 "alkanesulfonate transporter substrate-binding subunit"
"JS1_C14_00192" 5166 "s59E5H2" 1.533968 "alkanesulfonate transporter substrate-binding subunit"
"JS1_C14_00192" 5166 "s63E6H2" 0 "alkanesulfonate transporter substrate-binding subunit"
"JS1_C14_00193" 5167 "s59E13H2" 0.001956826 "Sialic acid TRAP transporter permease protein SiaT"
"JS1_C14_00193" 5167 "s59E25H2" 0 "Sialic acid TRAP transporter permease protein SiaT"
"JS1_C14_00193" 5167 "s59E5H2" 1.254019 "Sialic acid TRAP transporter permease protein SiaT"
"JS1_C14_00193" 5167 "s63E6H2" 0.001577517 "Sialic acid TRAP transporter permease protein SiaT"

"JS1_C14_00194" 5168 "s59E13H2" 0.03704474 "HTH-type transcriptional activator Btr"
"JS1_C14_00194" 5168 "s59E25H2" 0 "HTH-type transcriptional activator Btr"
"JS1_C14_00194" 5168 "s59E5H2" 1.561742 "HTH-type transcriptional activator Btr"
"JS1_C14_00194" 5168 "s63E6H2" 0.1348571 "HTH-type transcriptional activator Btr"
"JS1_C14_00195" 5169 "s59E13H2" 0.05461761 "Cation/acetate symporter ActP"
"JS1_C14_00195" 5169 "s59E25H2" 0 "Cation/acetate symporter ActP"
"JS1_C14_00195" 5169 "s59E5H2" 1.684254 "Cation/acetate symporter ActP"
"JS1_C14_00195" 5169 "s63E6H2" 0.108085 "Cation/acetate symporter ActP"
"JS1_C14_00196" 5170 "s59E13H2" 0.415502 "Uracil DNA glycosylase superfamily protein"
"JS1_C14_00196" 5170 "s59E25H2" 0.2142857 "Uracil DNA glycosylase superfamily protein"
"JS1_C14_00196" 5170 "s59E5H2" 0.2966361 "Uracil DNA glycosylase superfamily protein"
"JS1_C14_00196" 5170 "s63E6H2" 0.1930691 "Uracil DNA glycosylase superfamily protein"
"JS1_C14_00197" 5171 "s59E13H2" 0.5962085 "hypothetical protein"
"JS1_C14_00197" 5171 "s59E25H2" 0.5373134 "hypothetical protein"
"JS1_C14_00197" 5171 "s59E5H2" 0.1108205 "hypothetical protein"
"JS1_C14_00197" 5171 "s63E6H2" 0.1530408 "hypothetical protein"
"JS1_C14_00198" 5172 "s59E13H2" 0.5294959 "putative protease YhbU precursor"
"JS1_C14_00198" 5172 "s59E25H2" 0 "putative protease YhbU precursor"
"JS1_C14_00198" 5172 "s59E5H2" 0.6717588 "putative protease YhbU precursor"
"JS1_C14_00198" 5172 "s63E6H2" 1.261715 "putative protease YhbU precursor"
"JS1_C14_00199" 5173 "s59E13H2" 0.3815767 "Putative GTP cyclohydrolase 1 type 2"
"JS1_C14_00199" 5173 "s59E25H2" 0 "Putative GTP cyclohydrolase 1 type 2"
"JS1_C14_00199" 5173 "s59E5H2" 0.338754 "Putative GTP cyclohydrolase 1 type 2"
"JS1_C14_00199" 5173 "s63E6H2" 0.1343679 "Putative GTP cyclohydrolase 1 type 2"
"JS1_C14_00200" 5174 "s59E13H2" 0.4970121 "hypothetical protein"
"JS1_C14_00200" 5174 "s59E25H2" 0 "hypothetical protein"
"JS1_C14_00200" 5174 "s59E5H2" 0.3722265 "hypothetical protein"
"JS1_C14_00200" 5174 "s63E6H2" 1.161466 "hypothetical protein"
"JS1_C14_00201" 5175 "s59E13H2" 0.4438905 "Transketolase 2"
"JS1_C14_00201" 5175 "s59E25H2" 0 "Transketolase 2"
"JS1_C14_00201" 5175 "s59E5H2" 0.8077686 "Transketolase 2"
"JS1_C14_00201" 5175 "s63E6H2" 2.18988 "Transketolase 2"
"JS1_C14_00202" 5176 "s59E13H2" 0.249994 "2-hydroxy-3-oxopropionate reductase"
"JS1_C14_00202" 5176 "s59E25H2" 0 "2-hydroxy-3-oxopropionate reductase"
"JS1_C14_00202" 5176 "s59E5H2" 0.8192037 "2-hydroxy-3-oxopropionate reductase"
"JS1_C14_00202" 5176 "s63E6H2" 0.1698915 "2-hydroxy-3-oxopropionate reductase"
"JS1_C14_00203" 5177 "s59E13H2" 0.1676054 "hypothetical protein"
"JS1_C14_00203" 5177 "s59E25H2" 0 "hypothetical protein"
"JS1_C14_00203" 5177 "s59E5H2" 0.4991033 "hypothetical protein"
"JS1_C14_00203" 5177 "s63E6H2" 0.6101865 "hypothetical protein"
"JS1_C14_00204" 5178 "s59E13H2" 0.1138708 "2-dehydro-3-deoxygluconokinase"
"JS1_C14_00204" 5178 "s59E25H2" 0 "2-dehydro-3-deoxygluconokinase"
"JS1_C14_00204" 5178 "s59E5H2" 0.7221967 "2-dehydro-3-deoxygluconokinase"
"JS1_C14_00204" 5178 "s63E6H2" 0.2270885 "2-dehydro-3-deoxygluconokinase"
"JS1_C14_00205" 5179 "s59E13H2" 1.054551 "Trehalose/maltose import ATP-binding protein MalK"
"JS1_C14_00205" 5179 "s59E25H2" 0 "Trehalose/maltose import ATP-binding protein MalK"
"JS1_C14_00205" 5179 "s59E5H2" 1.641947 "Trehalose/maltose import ATP-binding protein MalK"

"JS1_C14_00205" 5179 "s63E6H2" 0.6065946 "Trehalose/maltose import ATP-binding protein MalK"
"JS1_C14_00206" 5180 "s59E13H2" 0.1762974 "Putative 2-aminoethylphosphonate transport system permease protein PhnV"
"JS1_C14_00206" 5180 "s59E25H2" 0 "Putative 2-aminoethylphosphonate transport system permease protein PhnV"
"JS1_C14_00206" 5180 "s59E5H2" 0.8468082 "Putative 2-aminoethylphosphonate transport system permease protein PhnV"
"JS1_C14_00206" 5180 "s63E6H2" 0.53152 "Putative 2-aminoethylphosphonate transport system permease protein PhnV"
"JS1_C14_00207" 5181 "s59E13H2" 0.07206824 "putative binding protein component of ABC iron transporter precursor"
"JS1_C14_00207" 5181 "s59E25H2" 0 "putative binding protein component of ABC iron transporter precursor"
"JS1_C14_00207" 5181 "s59E5H2" 0.03350425 "putative binding protein component of ABC iron transporter precursor"
"JS1_C14_00207" 5181 "s63E6H2" 0.7840106 "putative binding protein component of ABC iron transporter precursor"
"JS1_C14_00208" 5182 "s59E13H2" 0.05125419 "hypothetical protein"
"JS1_C14_00208" 5182 "s59E25H2" 0 "hypothetical protein"
"JS1_C14_00208" 5182 "s59E5H2" 0.330446 "hypothetical protein"
"JS1_C14_00208" 5182 "s63E6H2" 0.1361959 "hypothetical protein"
"JS1_C14_00209" 5183 "s59E13H2" 1.002897 "6-phosphofructokinase 1"
"JS1_C14_00209" 5183 "s59E25H2" 0 "6-phosphofructokinase 1"
"JS1_C14_00209" 5183 "s59E5H2" 0.4582097 "6-phosphofructokinase 1"
"JS1_C14_00209" 5183 "s63E6H2" 0.7173793 "6-phosphofructokinase 1"
"JS1_C14_00210" 5184 "s59E13H2" 0.0711114 "Transcription elongation factor GreA"
"JS1_C14_00210" 5184 "s59E25H2" 0 "Transcription elongation factor GreA"
"JS1_C14_00210" 5184 "s59E5H2" 0.2280245 "Transcription elongation factor GreA"
"JS1_C14_00210" 5184 "s63E6H2" 0.182725 "Transcription elongation factor GreA"
"JS1_C14_00211" 5185 "s59E13H2" 0.01681373 "Putative pyridoxal phosphate-dependent acyltransferase"
"JS1_C14_00211" 5185 "s59E25H2" 0 "Putative pyridoxal phosphate-dependent acyltransferase"
"JS1_C14_00211" 5185 "s59E5H2" 0.1664459 "Putative pyridoxal phosphate-dependent acyltransferase"
"JS1_C14_00211" 5185 "s63E6H2" 0.198243 "Putative pyridoxal phosphate-dependent acyltransferase"
"JS1_C14_00212" 5186 "s59E13H2" 0.04146574 "hypothetical protein"
"JS1_C14_00212" 5186 "s59E25H2" 0 "hypothetical protein"
"JS1_C14_00212" 5186 "s59E5H2" 0.1570523 "hypothetical protein"
"JS1_C14_00212" 5186 "s63E6H2" 0.1623886 "hypothetical protein"
"JS1_C14_00213" 5187 "s59E13H2" 0.01569977 "hypothetical protein"
"JS1_C14_00213" 5187 "s59E25H2" 0 "hypothetical protein"
"JS1_C14_00213" 5187 "s59E5H2" 0.2942507 "hypothetical protein"
"JS1_C14_00213" 5187 "s63E6H2" 0.09304208 "hypothetical protein"
"JS1_C14_00214" 5188 "s59E13H2" 0.002668726 "Molybdate/tungstate import ATP-binding protein WtpC"
"JS1_C14_00214" 5188 "s59E25H2" 0 "Molybdate/tungstate import ATP-binding protein WtpC"
"JS1_C14_00214" 5188 "s59E5H2" 0.1936683 "Molybdate/tungstate import ATP-binding protein WtpC"
"JS1_C14_00214" 5188 "s63E6H2" 0.2389287 "Molybdate/tungstate import ATP-binding protein WtpC"
"JS1_C14_00215" 5189 "s59E13H2" 0.1571178 "Molybdopterin molybdenumtransferase"
"JS1_C14_00215" 5189 "s59E25H2" 0 "Molybdopterin molybdenumtransferase"
"JS1_C14_00215" 5189 "s59E5H2" 0.278411 "Molybdopterin molybdenumtransferase"
"JS1_C14_00215" 5189 "s63E6H2" 0.6234198 "Molybdopterin molybdenumtransferase"
"JS1_C14_00216" 5190 "s59E13H2" 0.2372081 "Molybdopterin molybdenumtransferase"
"JS1_C14_00216" 5190 "s59E25H2" 0 "Molybdopterin molybdenumtransferase"
"JS1_C14_00216" 5190 "s59E5H2" 0.4270396 "Molybdopterin molybdenumtransferase"
"JS1_C14_00216" 5190 "s63E6H2" 0.5670851 "Molybdopterin molybdenumtransferase"
"JS1_C14_00217" 5191 "s59E13H2" 0.1585407 "Cyclic pyranopterin monophosphate synthase"
"JS1_C14_00217" 5191 "s59E25H2" 0 "Cyclic pyranopterin monophosphate synthase"

"JS1_C14_00217" 5191 "s59E5H2" 0.4018297 "Cyclic pyranopterin monophosphate synthase"
"JS1_C14_00217" 5191 "s63E6H2" 0.3230502 "Cyclic pyranopterin monophosphate synthase"
"JS1_C14_00218" 5192 "s59E13H2" 0 "Cyclic pyranopterin monophosphate synthase accessory protein"
"JS1_C14_00218" 5192 "s59E25H2" 0 "Cyclic pyranopterin monophosphate synthase accessory protein"
"JS1_C14_00218" 5192 "s59E5H2" 0.4210818 "Cyclic pyranopterin monophosphate synthase accessory protein"
"JS1_C14_00218" 5192 "s63E6H2" 0.2065616 "Cyclic pyranopterin monophosphate synthase accessory protein"
"JS1_C14_00219" 5193 "s59E13H2" 0.1142845 "Molybdopterin adenylyltransferase"
"JS1_C14_00219" 5193 "s59E25H2" 0 "Molybdopterin adenylyltransferase"
"JS1_C14_00219" 5193 "s59E5H2" 0.4844795 "Molybdopterin adenylyltransferase"
"JS1_C14_00219" 5193 "s63E6H2" 0.1411787 "Molybdopterin adenylyltransferase"
"JS1_C14_00220" 5194 "s59E13H2" 0.2215664 "Ribonuclease Z"
"JS1_C14_00220" 5194 "s59E25H2" 0 "Ribonuclease Z"
"JS1_C14_00220" 5194 "s59E5H2" 0.5367315 "Ribonuclease Z"
"JS1_C14_00220" 5194 "s63E6H2" 0.2512003 "Ribonuclease Z"
"JS1_C14_00221" 5195 "s59E13H2" 0.06592855 "Ferredoxin"
"JS1_C14_00221" 5195 "s59E25H2" 0 "Ferredoxin"
"JS1_C14_00221" 5195 "s59E5H2" 0.7962924 "Ferredoxin"
"JS1_C14_00221" 5195 "s63E6H2" 0.1297569 "Ferredoxin"
"JS1_C14_00222" 5196 "s59E13H2" 0.08982087 "Oligo-1,6-glucosidase"
"JS1_C14_00222" 5196 "s59E25H2" 0 "Oligo-1,6-glucosidase"
"JS1_C14_00222" 5196 "s59E5H2" 1.187182 "Oligo-1,6-glucosidase"
"JS1_C14_00222" 5196 "s63E6H2" 2.931425 "Oligo-1,6-glucosidase"
"JS1_C14_00223" 5197 "s59E13H2" 0.6710239 "Oligo-1,6-glucosidase"
"JS1_C14_00223" 5197 "s59E25H2" 0 "Oligo-1,6-glucosidase"
"JS1_C14_00223" 5197 "s59E5H2" 0.6798154 "Oligo-1,6-glucosidase"
"JS1_C14_00223" 5197 "s63E6H2" 0.8809776 "Oligo-1,6-glucosidase"
"JS1_C14_00224" 5198 "s59E13H2" 0.03708163 "HTH-type transcriptional regulator CymR"
"JS1_C14_00224" 5198 "s59E25H2" 0 "HTH-type transcriptional regulator CymR"
"JS1_C14_00224" 5198 "s59E5H2" 0.1809964 "HTH-type transcriptional regulator CymR"
"JS1_C14_00224" 5198 "s63E6H2" 0.09711275 "HTH-type transcriptional regulator CymR"
"JS1_C14_00225" 5199 "s59E13H2" 0.03537317 "hypothetical protein"
"JS1_C14_00225" 5199 "s59E25H2" 0 "hypothetical protein"
"JS1_C14_00225" 5199 "s59E5H2" 0.166634 "hypothetical protein"
"JS1_C14_00225" 5199 "s63E6H2" 0.07998651 "hypothetical protein"
"JS1_C14_00226" 5200 "s59E13H2" 0.03179012 "V-type ATP synthase subunit I"
"JS1_C14_00226" 5200 "s59E25H2" 0 "V-type ATP synthase subunit I"
"JS1_C14_00226" 5200 "s59E5H2" 0.2115376 "V-type ATP synthase subunit I"
"JS1_C14_00226" 5200 "s63E6H2" 0.04813344 "V-type ATP synthase subunit I"
"JS1_C14_00227" 5201 "s59E13H2" 0.06405853 "V-type sodium ATPase subunit K"
"JS1_C14_00227" 5201 "s59E25H2" 0 "V-type sodium ATPase subunit K"
"JS1_C14_00227" 5201 "s59E5H2" 1.017125 "V-type sodium ATPase subunit K"
"JS1_C14_00227" 5201 "s63E6H2" 0.1069037 "V-type sodium ATPase subunit K"
"JS1_C14_00228" 5202 "s59E13H2" 0.9232064 "V-type ATP synthase subunit E"
"JS1_C14_00228" 5202 "s59E25H2" 0 "V-type ATP synthase subunit E"
"JS1_C14_00228" 5202 "s59E5H2" 0.2500734 "V-type ATP synthase subunit E"
"JS1_C14_00228" 5202 "s63E6H2" 0.08215696 "V-type ATP synthase subunit E"
"JS1_C14_00229" 5203 "s59E13H2" 0.02442362 "V-type sodium ATPase subunit C"

"JS1_C14_00229" 5203 "s59E25H2" 0 "V-type sodium ATPase subunit C"
"JS1_C14_00229" 5203 "s59E5H2" 0.3518529 "V-type sodium ATPase subunit C"
"JS1_C14_00229" 5203 "s63E6H2" 0.04436845 "V-type sodium ATPase subunit C"
"JS1_C14_00230" 5204 "s59E13H2" 0.105759 "V-type sodium ATPase subunit G"
"JS1_C14_00230" 5204 "s59E25H2" 0 "V-type sodium ATPase subunit G"
"JS1_C14_00230" 5204 "s59E5H2" 0.5159991 "V-type sodium ATPase subunit G"
"JS1_C14_00230" 5204 "s63E6H2" 0.06610995 "V-type sodium ATPase subunit G"
"JS1_C14_00231" 5205 "s59E13H2" 0.1125498 "V-type sodium ATPase catalytic subunit A"
"JS1_C14_00231" 5205 "s59E25H2" 0 "V-type sodium ATPase catalytic subunit A"
"JS1_C14_00231" 5205 "s59E5H2" 0.5621176 "V-type sodium ATPase catalytic subunit A"
"JS1_C14_00231" 5205 "s63E6H2" 0.4415989 "V-type sodium ATPase catalytic subunit A"
"JS1_C14_00232" 5206 "s59E13H2" 0.2243068 "V-type sodium ATPase subunit B"
"JS1_C14_00232" 5206 "s59E25H2" 0 "V-type sodium ATPase subunit B"
"JS1_C14_00232" 5206 "s59E5H2" 0.7773256 "V-type sodium ATPase subunit B"
"JS1_C14_00232" 5206 "s63E6H2" 0.6961496 "V-type sodium ATPase subunit B"
"JS1_C14_00233" 5207 "s59E13H2" 0.1056507 "V-type sodium ATPase subunit D"
"JS1_C14_00233" 5207 "s59E25H2" 0 "V-type sodium ATPase subunit D"
"JS1_C14_00233" 5207 "s59E5H2" 0.3737093 "V-type sodium ATPase subunit D"
"JS1_C14_00233" 5207 "s63E6H2" 0.5445981 "V-type sodium ATPase subunit D"
"JS1_C14_00234" 5208 "s59E13H2" 0.8327424 "Transposase IS116/IS110/IS902 family protein"
"JS1_C14_00234" 5208 "s59E25H2" 0 "Transposase IS116/IS110/IS902 family protein"
"JS1_C14_00234" 5208 "s59E5H2" 0.517632 "Transposase IS116/IS110/IS902 family protein"
"JS1_C14_00234" 5208 "s63E6H2" 1.372486 "Transposase IS116/IS110/IS902 family protein"
"JS1_C14_00235" 5209 "s59E13H2" 0.2559185 "phosphodiesterase"
"JS1_C14_00235" 5209 "s59E25H2" 0 "phosphodiesterase"
"JS1_C14_00235" 5209 "s59E5H2" 0.5579483 "phosphodiesterase"
"JS1_C14_00235" 5209 "s63E6H2" 0.4771953 "phosphodiesterase"
"JS1_C14_00237" 5211 "s59E13H2" 0.1616638 "1-acyl-sn-glycerol-3-phosphate acyltransferase"
"JS1_C14_00237" 5211 "s59E25H2" 0 "1-acyl-sn-glycerol-3-phosphate acyltransferase"
"JS1_C14_00237" 5211 "s59E5H2" 0.2576743 "1-acyl-sn-glycerol-3-phosphate acyltransferase"
"JS1_C14_00237" 5211 "s63E6H2" 0.5450602 "1-acyl-sn-glycerol-3-phosphate acyltransferase"
"JS1_C14_00238" 5212 "s59E13H2" 0.1112063 "Serine/threonine-protein kinase PknD"
"JS1_C14_00238" 5212 "s59E25H2" 0 "Serine/threonine-protein kinase PknD"
"JS1_C14_00238" 5212 "s59E5H2" 0.691655 "Serine/threonine-protein kinase PknD"
"JS1_C14_00238" 5212 "s63E6H2" 0.7169757 "Serine/threonine-protein kinase PknD"
"JS1_C14_00239" 5213 "s59E13H2" 0.08968499 "hypothetical protein"
"JS1_C14_00239" 5213 "s59E25H2" 0 "hypothetical protein"
"JS1_C14_00239" 5213 "s59E5H2" 0.2131144 "hypothetical protein"
"JS1_C14_00239" 5213 "s63E6H2" 1.126922 "hypothetical protein"
"JS1_C14_00240" 5214 "s59E13H2" 0.0123655 "Acetyltransferase (GNAT) family protein"
"JS1_C14_00240" 5214 "s59E25H2" 0.8367003 "Acetyltransferase (GNAT) family protein"
"JS1_C14_00240" 5214 "s59E5H2" 0.06962311 "Acetyltransferase (GNAT) family protein"
"JS1_C14_00240" 5214 "s63E6H2" 0.510732 "Acetyltransferase (GNAT) family protein"
"JS1_C14_00241" 5215 "s59E13H2" 0.06325303 "hypothetical protein"
"JS1_C14_00241" 5215 "s59E25H2" 0 "hypothetical protein"
"JS1_C14_00241" 5215 "s59E5H2" 0.5788996 "hypothetical protein"
"JS1_C14_00241" 5215 "s63E6H2" 0.787004 "hypothetical protein"

"JS1_C14_00242" 5216 "s59E13H2" 0.05340828 "hypothetical protein"
"JS1_C14_00242" 5216 "s59E25H2" 0 "hypothetical protein"
"JS1_C14_00242" 5216 "s59E5H2" 0.4805177 "hypothetical protein"
"JS1_C14_00242" 5216 "s63E6H2" 1.051825 "hypothetical protein"
"JS1_C14_00244" 5218 "s59E13H2" 0.008000477 "hypothetical protein"
"JS1_C14_00244" 5218 "s59E25H2" 0 "hypothetical protein"
"JS1_C14_00244" 5218 "s59E5H2" 0 "hypothetical protein"
"JS1_C14_00244" 5218 "s63E6H2" 0 "hypothetical protein"
"JS1_C14_00245" 5219 "s59E13H2" 0.006161281 "hypothetical protein"
"JS1_C14_00245" 5219 "s59E25H2" 0 "hypothetical protein"
"JS1_C14_00245" 5219 "s59E5H2" 0 "hypothetical protein"
"JS1_C14_00245" 5219 "s63E6H2" 0 "hypothetical protein"
"JS1_C14_00246" 5220 "s59E13H2" 0.001917632 "Secreted effector protein pipB2"
"JS1_C14_00246" 5220 "s59E25H2" 0 "Secreted effector protein pipB2"
"JS1_C14_00246" 5220 "s59E5H2" 0.01484129 "Secreted effector protein pipB2"
"JS1_C14_00246" 5220 "s63E6H2" 0 "Secreted effector protein pipB2"
"JS1_C14_00247" 5221 "s59E13H2" 0 "NYN domain protein"
"JS1_C14_00247" 5221 "s59E25H2" 0 "NYN domain protein"
"JS1_C14_00247" 5221 "s59E5H2" 0.01357261 "NYN domain protein"
"JS1_C14_00247" 5221 "s63E6H2" 0 "NYN domain protein"
"JS1_C14_00248" 5222 "s59E13H2" 0.004717941 "hypothetical protein"
"JS1_C14_00248" 5222 "s59E25H2" 0 "hypothetical protein"
"JS1_C14_00248" 5222 "s59E5H2" 0 "hypothetical protein"
"JS1_C14_00248" 5222 "s63E6H2" 0 "hypothetical protein"
"JS1_C14_00249" 5223 "s59E13H2" 0.008020224 "hypothetical protein"
"JS1_C14_00249" 5223 "s59E25H2" 0 "hypothetical protein"
"JS1_C14_00249" 5223 "s59E5H2" 0 "hypothetical protein"
"JS1_C14_00249" 5223 "s63E6H2" 0 "hypothetical protein"
"JS1_C14_00250" 5224 "s59E13H2" 0.1577679 "hypothetical protein"
"JS1_C14_00250" 5224 "s59E25H2" 0 "hypothetical protein"
"JS1_C14_00250" 5224 "s59E5H2" 0 "hypothetical protein"
"JS1_C14_00250" 5224 "s63E6H2" 0 "hypothetical protein"
"JS1_C14_00251" 5225 "s59E13H2" 0.008782314 "hypothetical protein"
"JS1_C14_00251" 5225 "s59E25H2" 0 "hypothetical protein"
"JS1_C14_00251" 5225 "s59E5H2" 0 "hypothetical protein"
"JS1_C14_00251" 5225 "s63E6H2" 0 "hypothetical protein"
"JS1_C14_00252" 5226 "s59E13H2" 0 "hypothetical protein"
"JS1_C14_00252" 5226 "s59E25H2" 0 "hypothetical protein"
"JS1_C14_00252" 5226 "s59E5H2" 0.006221608 "hypothetical protein"
"JS1_C14_00252" 5226 "s63E6H2" 0.004172846 "hypothetical protein"
"JS1_C14_00255" 5229 "s59E13H2" 0.003251273 "Dipeptide transport system permease protein DppB"
"JS1_C14_00255" 5229 "s59E25H2" 0 "Dipeptide transport system permease protein DppB"
"JS1_C14_00255" 5229 "s59E5H2" 0.9405911 "Dipeptide transport system permease protein DppB"
"JS1_C14_00255" 5229 "s63E6H2" 0.03961437 "Dipeptide transport system permease protein DppB"
"JS1_C14_00256" 5230 "s59E13H2" 0.002452579 "Dipeptide transport system permease protein DppC"
"JS1_C14_00256" 5230 "s59E25H2" 0.1548894 "Dipeptide transport system permease protein DppC"
"JS1_C14_00256" 5230 "s59E5H2" 0.2893233 "Dipeptide transport system permease protein DppC"

"JS1_C14_00256" 5230 "s63E6H2" 0.05444023 "Dipeptide transport system permease protein DppC"
"JS1_C14_00257" 5231 "s59E13H2" 0.003594786 "Oligopeptide transport ATP-binding protein OppD"
"JS1_C14_00257" 5231 "s59E25H2" 0 "Oligopeptide transport ATP-binding protein OppD"
"JS1_C14_00257" 5231 "s59E5H2" 0.4616791 "Oligopeptide transport ATP-binding protein OppD"
"JS1_C14_00257" 5231 "s63E6H2" 0.0263188 "Oligopeptide transport ATP-binding protein OppD"
"JS1_C14_00258" 5232 "s59E13H2" 0.05959043 "Oligopeptide transport ATP-binding protein OppF"
"JS1_C14_00258" 5232 "s59E25H2" 0 "Oligopeptide transport ATP-binding protein OppF"
"JS1_C14_00258" 5232 "s59E5H2" 0.8323162 "Oligopeptide transport ATP-binding protein OppF"
"JS1_C14_00258" 5232 "s63E6H2" 0.02098454 "Oligopeptide transport ATP-binding protein OppF"
"JS1_C14_00259" 5233 "s59E13H2" 0.05750677 "ECF RNA polymerase sigma factor SigW"
"JS1_C14_00259" 5233 "s59E25H2" 0 "ECF RNA polymerase sigma factor SigW"
"JS1_C14_00259" 5233 "s59E5H2" 0.05006317 "ECF RNA polymerase sigma factor SigW"
"JS1_C14_00259" 5233 "s63E6H2" 0.01112961 "ECF RNA polymerase sigma factor SigW"
"JS1_C14_00260" 5234 "s59E13H2" 0.07346033 "Anti-sigma-W factor RsiW"
"JS1_C14_00260" 5234 "s59E25H2" 0 "Anti-sigma-W factor RsiW"
"JS1_C14_00260" 5234 "s59E5H2" 0.07798924 "Anti-sigma-W factor RsiW"
"JS1_C14_00260" 5234 "s63E6H2" 0.04394921 "Anti-sigma-W factor RsiW"
"JS1_C14_00261" 5235 "s59E13H2" 0.5021507 "Lipopolysaccharide export system permease protein LptF"
"JS1_C14_00261" 5235 "s59E25H2" 0 "Lipopolysaccharide export system permease protein LptF"
"JS1_C14_00261" 5235 "s59E5H2" 0.7697274 "Lipopolysaccharide export system permease protein LptF"
"JS1_C14_00261" 5235 "s63E6H2" 0.8556541 "Lipopolysaccharide export system permease protein LptF"
"JS1_C14_00262" 5236 "s59E13H2" 0.5878652 "LPS-assembly protein LptD"
"JS1_C14_00262" 5236 "s59E25H2" 0 "LPS-assembly protein LptD"
"JS1_C14_00262" 5236 "s59E5H2" 0.2467225 "LPS-assembly protein LptD"
"JS1_C14_00262" 5236 "s63E6H2" 2.797721 "LPS-assembly protein LptD"
"JS1_C14_00263" 5237 "s59E13H2" 0.3357643 "hypothetical protein"
"JS1_C14_00263" 5237 "s59E25H2" 0 "hypothetical protein"
"JS1_C14_00263" 5237 "s59E5H2" 0.02732915 "hypothetical protein"
"JS1_C14_00263" 5237 "s63E6H2" 2.123171 "hypothetical protein"
"JS1_C14_00264" 5238 "s59E13H2" 0.274207 "LPS-assembly protein LptD"
"JS1_C14_00264" 5238 "s59E25H2" 0 "LPS-assembly protein LptD"
"JS1_C14_00264" 5238 "s59E5H2" 0.7167529 "LPS-assembly protein LptD"
"JS1_C14_00264" 5238 "s63E6H2" 2.844939 "LPS-assembly protein LptD"
"JS1_C14_00265" 5239 "s59E13H2" 0.1716545 "Lipopolysaccharide-assembly, LptC-related"
"JS1_C14_00265" 5239 "s59E25H2" 0 "Lipopolysaccharide-assembly, LptC-related"
"JS1_C14_00265" 5239 "s59E5H2" 0.4705005 "Lipopolysaccharide-assembly, LptC-related"
"JS1_C14_00265" 5239 "s63E6H2" 1.236372 "Lipopolysaccharide-assembly, LptC-related"
"JS1_C14_00266" 5240 "s59E13H2" 0.1831125 "Clostripain precursor"
"JS1_C14_00266" 5240 "s59E25H2" 0 "Clostripain precursor"
"JS1_C14_00266" 5240 "s59E5H2" 0.04934063 "Clostripain precursor"
"JS1_C14_00266" 5240 "s63E6H2" 0.3175156 "Clostripain precursor"
"JS1_C14_00267" 5241 "s59E13H2" 0.07133941 "hypothetical protein"
"JS1_C14_00267" 5241 "s59E25H2" 0 "hypothetical protein"
"JS1_C14_00267" 5241 "s59E5H2" 0 "hypothetical protein"
"JS1_C14_00267" 5241 "s63E6H2" 0 "hypothetical protein"
"JS1_C14_00268" 5242 "s59E13H2" 0.741499 "hypothetical protein"
"JS1_C14_00268" 5242 "s59E25H2" 0 "hypothetical protein"

"JS1_C14_00268" 5242 "s59E5H2" 0.1670908 "hypothetical protein"
"JS1_C14_00268" 5242 "s63E6H2" 0.006473816 "hypothetical protein"
"JS1_C14_00269" 5243 "s59E13H2" 0.3189733 "PEGA domain protein"
"JS1_C14_00269" 5243 "s59E25H2" 0 "PEGA domain protein"
"JS1_C14_00269" 5243 "s59E5H2" 0.1047041 "PEGA domain protein"
"JS1_C14_00269" 5243 "s63E6H2" 1.977005e-05 "PEGA domain protein"
"JS1_C14_00270" 5244 "s59E13H2" 0.2020035 "Bacterial Ig-like domain (group 2)"
"JS1_C14_00270" 5244 "s59E25H2" 0 "Bacterial Ig-like domain (group 2)"
"JS1_C14_00270" 5244 "s59E5H2" 0.1141038 "Bacterial Ig-like domain (group 2)"
"JS1_C14_00270" 5244 "s63E6H2" 0.02817275 "Bacterial Ig-like domain (group 2)"
"JS1_C14_00271" 5245 "s59E13H2" 0.1966129 "hypothetical protein"
"JS1_C14_00271" 5245 "s59E25H2" 0 "hypothetical protein"
"JS1_C14_00271" 5245 "s59E5H2" 0.5914937 "hypothetical protein"
"JS1_C14_00271" 5245 "s63E6H2" 1.499226 "hypothetical protein"
"JS1_C14_00272" 5246 "s59E13H2" 0.1617186 "Outer membrane protein alpha precursor"
"JS1_C14_00272" 5246 "s59E25H2" 0 "Outer membrane protein alpha precursor"
"JS1_C14_00272" 5246 "s59E5H2" 0.6177017 "Outer membrane protein alpha precursor"
"JS1_C14_00272" 5246 "s63E6H2" 0.738907 "Outer membrane protein alpha precursor"
"JS1_C14_00273" 5247 "s59E13H2" 0.03560601 "hypothetical protein"
"JS1_C14_00273" 5247 "s59E25H2" 0 "hypothetical protein"
"JS1_C14_00273" 5247 "s59E5H2" 0.014163 "hypothetical protein"
"JS1_C14_00273" 5247 "s63E6H2" 0.01306045 "hypothetical protein"
"JS1_C14_00274" 5248 "s59E13H2" 0.1905376 "hypothetical protein"
"JS1_C14_00274" 5248 "s59E25H2" 0 "hypothetical protein"
"JS1_C14_00274" 5248 "s59E5H2" 0.02210288 "hypothetical protein"
"JS1_C14_00274" 5248 "s63E6H2" 0.08967783 "hypothetical protein"
"JS1_C14_00275" 5249 "s59E13H2" 0.0604099 "hypothetical protein"
"JS1_C14_00275" 5249 "s59E25H2" 0 "hypothetical protein"
"JS1_C14_00275" 5249 "s59E5H2" 0.03161736 "hypothetical protein"
"JS1_C14_00275" 5249 "s63E6H2" 0.0478954 "hypothetical protein"
"JS1_C14_00276" 5250 "s59E13H2" 0.08634381 "Putative serine protease HtrA"
"JS1_C14_00276" 5250 "s59E25H2" 0 "Putative serine protease HtrA"
"JS1_C14_00276" 5250 "s59E5H2" 0.02363933 "Putative serine protease HtrA"
"JS1_C14_00276" 5250 "s63E6H2" 0.03029638 "Putative serine protease HtrA"
"JS1_C14_00278" 5252 "s59E13H2" 1.186616 "V-type ATP synthase subunit C"
"JS1_C14_00278" 5252 "s59E25H2" 0.4737345 "V-type ATP synthase subunit C"
"JS1_C14_00278" 5252 "s59E5H2" 1.162367 "V-type ATP synthase subunit C"
"JS1_C14_00278" 5252 "s63E6H2" 0.2461503 "V-type ATP synthase subunit C"
"JS1_C14_00279" 5253 "s59E13H2" 1.074639 "hypothetical protein"
"JS1_C14_00279" 5253 "s59E25H2" 0 "hypothetical protein"
"JS1_C14_00279" 5253 "s59E5H2" 0.2238281 "hypothetical protein"
"JS1_C14_00279" 5253 "s63E6H2" 0.1488883 "hypothetical protein"
"JS1_C14_00280" 5254 "s59E13H2" 0.9587308 "Transcriptional regulatory protein ZraR"
"JS1_C14_00280" 5254 "s59E25H2" 0 "Transcriptional regulatory protein ZraR"
"JS1_C14_00280" 5254 "s59E5H2" 0.9910504 "Transcriptional regulatory protein ZraR"
"JS1_C14_00280" 5254 "s63E6H2" 0.3004285 "Transcriptional regulatory protein ZraR"
"JS1_C14_00281" 5255 "s59E13H2" 0.8327172 "Sensor protein ZraS"

"JS1_C14_00281" 5255 "s59E25H2" 0 "Sensor protein ZraS"
"JS1_C14_00281" 5255 "s59E5H2" 0.698212 "Sensor protein ZraS"
"JS1_C14_00281" 5255 "s63E6H2" 0.1642769 "Sensor protein ZraS"
"JS1_C14_00286" 5260 "s59E13H2" 0.3247132 "putative outer membrane protein PmpB precursor"
"JS1_C14_00286" 5260 "s59E25H2" 0 "putative outer membrane protein PmpB precursor"
"JS1_C14_00286" 5260 "s59E5H2" 0.01729503 "putative outer membrane protein PmpB precursor"
"JS1_C14_00286" 5260 "s63E6H2" 0 "putative outer membrane protein PmpB precursor"
"JS1_C14_00287" 5261 "s59E13H2" 0.3317893 "hypothetical protein"
"JS1_C14_00287" 5261 "s59E25H2" 0 "hypothetical protein"
"JS1_C14_00287" 5261 "s59E5H2" 0.01263465 "hypothetical protein"
"JS1_C14_00287" 5261 "s63E6H2" 0 "hypothetical protein"
"JS1_C14_00288" 5262 "s59E13H2" 0.1829514 "hypothetical protein"
"JS1_C14_00288" 5262 "s59E25H2" 0 "hypothetical protein"
"JS1_C14_00288" 5262 "s59E5H2" 0.007970769 "hypothetical protein"
"JS1_C14_00288" 5262 "s63E6H2" 0 "hypothetical protein"
"JS1_C14_00289" 5263 "s59E13H2" 0.08995348 "hypothetical protein"
"JS1_C14_00289" 5263 "s59E25H2" 0 "hypothetical protein"
"JS1_C14_00289" 5263 "s59E5H2" 0.02457493 "hypothetical protein"
"JS1_C14_00289" 5263 "s63E6H2" 0 "hypothetical protein"
"JS1_C14_00290" 5264 "s59E13H2" 0.1318724 "hypothetical protein"
"JS1_C14_00290" 5264 "s59E25H2" 0 "hypothetical protein"
"JS1_C14_00290" 5264 "s59E5H2" 0.03804794 "hypothetical protein"
"JS1_C14_00290" 5264 "s63E6H2" 0 "hypothetical protein"
"JS1_C14_00291" 5265 "s59E13H2" 0.09190242 "hypothetical protein"
"JS1_C14_00291" 5265 "s59E25H2" 0 "hypothetical protein"
"JS1_C14_00291" 5265 "s59E5H2" 0.0009665532 "hypothetical protein"
"JS1_C14_00291" 5265 "s63E6H2" 0 "hypothetical protein"
"JS1_C14_00292" 5266 "s59E13H2" 0.4088567 "hypothetical protein"
"JS1_C14_00292" 5266 "s59E25H2" 0 "hypothetical protein"
"JS1_C14_00292" 5266 "s59E5H2" 0.7670987 "hypothetical protein"
"JS1_C14_00292" 5266 "s63E6H2" 1.111371 "hypothetical protein"
"JS1_C14_00293" 5267 "s59E13H2" 0.670979 "Lipopolysaccharide export system ATP-binding protein LptB"
"JS1_C14_00293" 5267 "s59E25H2" 0 "Lipopolysaccharide export system ATP-binding protein LptB"
"JS1_C14_00293" 5267 "s59E5H2" 0.9711119 "Lipopolysaccharide export system ATP-binding protein LptB"
"JS1_C14_00293" 5267 "s63E6H2" 1.495345 "Lipopolysaccharide export system ATP-binding protein LptB"
"JS1_C14_00294" 5268 "s59E13H2" 0.6218851 "3-hydroxyacyl-[acyl-carrier-protein] dehydratase FabZ"
"JS1_C14_00294" 5268 "s59E25H2" 0 "3-hydroxyacyl-[acyl-carrier-protein] dehydratase FabZ"
"JS1_C14_00294" 5268 "s59E5H2" 0.9304174 "3-hydroxyacyl-[acyl-carrier-protein] dehydratase FabZ"
"JS1_C14_00294" 5268 "s63E6H2" 1.133171 "3-hydroxyacyl-[acyl-carrier-protein] dehydratase FabZ"
"JS1_C14_00295" 5269 "s59E13H2" 0.3700751 "Outer membrane protein (OmpH-like)"
"JS1_C14_00295" 5269 "s59E25H2" 0 "Outer membrane protein (OmpH-like)"
"JS1_C14_00295" 5269 "s59E5H2" 0.8461648 "Outer membrane protein (OmpH-like)"
"JS1_C14_00295" 5269 "s63E6H2" 1.267384 "Outer membrane protein (OmpH-like)"
"JS1_C14_00296" 5270 "s59E13H2" 0.3489158 "Outer membrane protein assembly factor BamA precursor"
"JS1_C14_00296" 5270 "s59E25H2" 0 "Outer membrane protein assembly factor BamA precursor"
"JS1_C14_00296" 5270 "s59E5H2" 0.7804879 "Outer membrane protein assembly factor BamA precursor"
"JS1_C14_00296" 5270 "s63E6H2" 0.4840454 "Outer membrane protein assembly factor BamA precursor"

"JS1_C14_00297" 5271 "s59E13H2" 0.03631497 "N-acetyl-gamma-glutamyl-phosphate reductase"
"JS1_C14_00297" 5271 "s59E25H2" 0 "N-acetyl-gamma-glutamyl-phosphate reductase"
"JS1_C14_00297" 5271 "s59E5H2" 0.04405098 "N-acetyl-gamma-glutamyl-phosphate reductase"
"JS1_C14_00297" 5271 "s63E6H2" 0.05663758 "N-acetyl-gamma-glutamyl-phosphate reductase"
"JS1_C14_00299" 5273 "s59E13H2" 0.01563522 "Orotate phosphoribosyltransferase"
"JS1_C14_00299" 5273 "s59E25H2" 0 "Orotate phosphoribosyltransferase"
"JS1_C14_00299" 5273 "s59E5H2" 0.05608982 "Orotate phosphoribosyltransferase"
"JS1_C14_00299" 5273 "s63E6H2" 0.1105703 "Orotate phosphoribosyltransferase"
"JS1_C14_00300" 5274 "s59E13H2" 0.02456194 "Orotidine 5'-phosphate decarboxylase"
"JS1_C14_00300" 5274 "s59E25H2" 0 "Orotidine 5'-phosphate decarboxylase"
"JS1_C14_00300" 5274 "s59E5H2" 0.009220423 "Orotidine 5'-phosphate decarboxylase"
"JS1_C14_00300" 5274 "s63E6H2" 0.08960619 "Orotidine 5'-phosphate decarboxylase"
"JS1_C14_00301" 5275 "s59E13H2" 0.08678276 "Trimethylamine methyltransferase MttB"
"JS1_C14_00301" 5275 "s59E25H2" 0 "Trimethylamine methyltransferase MttB"
"JS1_C14_00301" 5275 "s59E5H2" 1.862781 "Trimethylamine methyltransferase MttB"
"JS1_C14_00301" 5275 "s63E6H2" 0.2994378 "Trimethylamine methyltransferase MttB"
"JS1_C14_00302" 5276 "s59E13H2" 0.0228677 "UDP-D-galactose:(glucosyl)lipopolysaccharide-1,6-D-galactosyltransferase"
"JS1_C14_00302" 5276 "s59E25H2" 0 "UDP-D-galactose:(glucosyl)lipopolysaccharide-1,6-D-galactosyltransferase"
"JS1_C14_00302" 5276 "s59E5H2" 0 "UDP-D-galactose:(glucosyl)lipopolysaccharide-1,6-D-galactosyltransferase"
"JS1_C14_00302" 5276 "s63E6H2" 0 "UDP-D-galactose:(glucosyl)lipopolysaccharide-1,6-D-galactosyltransferase"
"JS1_C14_00303" 5277 "s59E13H2" 0.02994722 "N,N'-diacetylbacillosaminyl-diphospho-undecaprenol alpha-1,3-N-acetylgalactosaminyltransferase"
"JS1_C14_00303" 5277 "s59E25H2" 0 "N,N'-diacetylbacillosaminyl-diphospho-undecaprenol alpha-1,3-N-acetylgalactosaminyltransferase"
"JS1_C14_00303" 5277 "s59E5H2" 0 "N,N'-diacetylbacillosaminyl-diphospho-undecaprenol alpha-1,3-N-acetylgalactosaminyltransferase"
"JS1_C14_00303" 5277 "s63E6H2" 0 "N,N'-diacetylbacillosaminyl-diphospho-undecaprenol alpha-1,3-N-acetylgalactosaminyltransferase"
"JS1_C14_00304" 5278 "s59E13H2" 0.00290642 "GDP-mannose 4,6-dehydratase"
"JS1_C14_00304" 5278 "s59E25H2" 0 "GDP-mannose 4,6-dehydratase"
"JS1_C14_00304" 5278 "s59E5H2" 0 "GDP-mannose 4,6-dehydratase"
"JS1_C14_00304" 5278 "s63E6H2" 0 "GDP-mannose 4,6-dehydratase"
"JS1_C14_00305" 5279 "s59E13H2" 0.01563522 "GDP-L-fucose synthase"
"JS1_C14_00305" 5279 "s59E25H2" 0 "GDP-L-fucose synthase"
"JS1_C14_00305" 5279 "s59E5H2" 0 "GDP-L-fucose synthase"
"JS1_C14_00305" 5279 "s63E6H2" 0.003104594 "GDP-L-fucose synthase"
"JS1_C14_00306" 5280 "s59E13H2" 0.03671669 "putative glycosyl transferase"
"JS1_C14_00306" 5280 "s59E25H2" 0 "putative glycosyl transferase"
"JS1_C14_00306" 5280 "s59E5H2" 0 "putative glycosyl transferase"
"JS1_C14_00306" 5280 "s63E6H2" 0 "putative glycosyl transferase"
"JS1_C14_00307" 5281 "s59E13H2" 0.02027119 "hypothetical protein"
"JS1_C14_00307" 5281 "s59E25H2" 0 "hypothetical protein"
"JS1_C14_00307" 5281 "s59E5H2" 0 "hypothetical protein"
"JS1_C14_00307" 5281 "s63E6H2" 0.004647775 "hypothetical protein"
"JS1_C14_00308" 5282 "s59E13H2" 0.01745523 "Phosphoglycerate kinase"
"JS1_C14_00308" 5282 "s59E25H2" 0 "Phosphoglycerate kinase"
"JS1_C14_00308" 5282 "s59E5H2" 0.005944302 "Phosphoglycerate kinase"
"JS1_C14_00308" 5282 "s63E6H2" 0.002393117 "Phosphoglycerate kinase"
"JS1_C14_00309" 5283 "s59E13H2" 0.03447792 "Glyceraldehyde-3-phosphate dehydrogenase"
"JS1_C14_00309" 5283 "s59E25H2" 0 "Glyceraldehyde-3-phosphate dehydrogenase"
"JS1_C14_00309" 5283 "s59E5H2" 0 "Glyceraldehyde-3-phosphate dehydrogenase"

"JS1_C14_00309" 5283 "s63E6H2" 0.01628296 "Glyceraldehyde-3-phosphate dehydrogenase"
"JS1_C14_00310" 5284 "s59E13H2" 0.1245191 "Dihydrolipoyllysine-residue acetyltransferase component of pyruvate dehydrogenase complex"
"JS1_C14_00310" 5284 "s59E25H2" 0 "Dihydrolipoyllysine-residue acetyltransferase component of pyruvate dehydrogenase complex"
"JS1_C14_00310" 5284 "s59E5H2" 0.01293032 "Dihydrolipoyllysine-residue acetyltransferase component of pyruvate dehydrogenase complex"
"JS1_C14_00310" 5284 "s63E6H2" 0 "Dihydrolipoyllysine-residue acetyltransferase component of pyruvate dehydrogenase complex"
"JS1_C14_00311" 5285 "s59E13H2" 0.08477789 "Acetoin:2,6-dichlorophenolindophenol oxidoreductase subunit beta"
"JS1_C14_00311" 5285 "s59E25H2" 0 "Acetoin:2,6-dichlorophenolindophenol oxidoreductase subunit beta"
"JS1_C14_00311" 5285 "s59E5H2" 0.004122741 "Acetoin:2,6-dichlorophenolindophenol oxidoreductase subunit beta"
"JS1_C14_00311" 5285 "s63E6H2" 0 "Acetoin:2,6-dichlorophenolindophenol oxidoreductase subunit beta"
"JS1_C14_00312" 5286 "s59E13H2" 0 "Acetoin:2,6-dichlorophenolindophenol oxidoreductase subunit alpha"
"JS1_C14_00312" 5286 "s59E25H2" 0 "Acetoin:2,6-dichlorophenolindophenol oxidoreductase subunit alpha"
"JS1_C14_00312" 5286 "s59E5H2" 0 "Acetoin:2,6-dichlorophenolindophenol oxidoreductase subunit alpha"
"JS1_C14_00312" 5286 "s63E6H2" 0 "Acetoin:2,6-dichlorophenolindophenol oxidoreductase subunit alpha"
"JS1_C14_00313" 5287 "s59E13H2" 8.936768 "Tripartite tricarboxylate transporter TctA family protein"
"JS1_C14_00313" 5287 "s59E25H2" 0.5142672 "Tripartite tricarboxylate transporter TctA family protein"
"JS1_C14_00313" 5287 "s59E5H2" 1.068019 "Tripartite tricarboxylate transporter TctA family protein"
"JS1_C14_00313" 5287 "s63E6H2" 12.4574 "Tripartite tricarboxylate transporter TctA family protein"
"JS1_C14_00314" 5288 "s59E13H2" 8.76366 "3-keto-5-aminohexanoate cleavage enzyme"
"JS1_C14_00314" 5288 "s59E25H2" 1.560847 "3-keto-5-aminohexanoate cleavage enzyme"
"JS1_C14_00314" 5288 "s59E5H2" 0.8995367 "3-keto-5-aminohexanoate cleavage enzyme"
"JS1_C14_00314" 5288 "s63E6H2" 13.49588 "3-keto-5-aminohexanoate cleavage enzyme"
"JS1_C14_00315" 5289 "s59E13H2" 6.747736 "3-keto-5-aminohexanoate cleavage enzyme"
"JS1_C14_00315" 5289 "s59E25H2" 0 "3-keto-5-aminohexanoate cleavage enzyme"
"JS1_C14_00315" 5289 "s59E5H2" 0.423628 "3-keto-5-aminohexanoate cleavage enzyme"
"JS1_C14_00315" 5289 "s63E6H2" 11.05581 "3-keto-5-aminohexanoate cleavage enzyme"
"JS1_C14_00317" 5291 "s59E13H2" 0.06913007 "Formamidase"
"JS1_C14_00317" 5291 "s59E25H2" 0 "Formamidase"
"JS1_C14_00317" 5291 "s59E5H2" 0.0854884 "Formamidase"
"JS1_C14_00317" 5291 "s63E6H2" 0.01548082 "Formamidase"
"JS1_C14_00318" 5292 "s59E13H2" 0.05643792 "hypothetical protein"
"JS1_C14_00318" 5292 "s59E25H2" 0 "hypothetical protein"
"JS1_C14_00318" 5292 "s59E5H2" 0.003232004 "hypothetical protein"
"JS1_C14_00318" 5292 "s63E6H2" 0.02749739 "hypothetical protein"
"JS1_C14_00320" 5294 "s59E13H2" 5.204848 "Diacetylchitobiose deacetylase"
"JS1_C14_00320" 5294 "s59E25H2" 0 "Diacetylchitobiose deacetylase"
"JS1_C14_00320" 5294 "s59E5H2" 5.109161 "Diacetylchitobiose deacetylase"
"JS1_C14_00320" 5294 "s63E6H2" 2.660614 "Diacetylchitobiose deacetylase"
"JS1_C14_00321" 5295 "s59E13H2" 6.794953 "Undecaprenyl-phosphate mannosyltransferase"
"JS1_C14_00321" 5295 "s59E25H2" 0 "Undecaprenyl-phosphate mannosyltransferase"
"JS1_C14_00321" 5295 "s59E5H2" 7.432895 "Undecaprenyl-phosphate mannosyltransferase"
"JS1_C14_00321" 5295 "s63E6H2" 1.867717 "Undecaprenyl-phosphate mannosyltransferase"
"JS1_C14_00322" 5296 "s59E13H2" 7.84966 "Na+/Pi-cotransporter"
"JS1_C14_00322" 5296 "s59E25H2" 0.3103662 "Na+/Pi-cotransporter"
"JS1_C14_00322" 5296 "s59E5H2" 2.974224 "Na+/Pi-cotransporter"
"JS1_C14_00322" 5296 "s63E6H2" 1.418173 "Na+/Pi-cotransporter"
"JS1_C14_00323" 5297 "s59E13H2" 7.876921 "L-2-hydroxyglutarate oxidase LhgO"
"JS1_C14_00323" 5297 "s59E25H2" 0.3306826 "L-2-hydroxyglutarate oxidase LhgO"

"JS1_C14_00323" 5297 "s59E5H2" 0.9053216 "L-2-hydroxyglutarate oxidase LhgO"
"JS1_C14_00323" 5297 "s63E6H2" 2.537844 "L-2-hydroxyglutarate oxidase LhgO"
"JS1_C14_00324" 5298 "s59E13H2" 2.759793 "Thioredoxin reductase"
"JS1_C14_00324" 5298 "s59E25H2" 0 "Thioredoxin reductase"
"JS1_C14_00324" 5298 "s59E5H2" 1.687685 "Thioredoxin reductase"
"JS1_C14_00324" 5298 "s63E6H2" 4.450371 "Thioredoxin reductase"
"JS1_C14_00325" 5299 "s59E13H2" 0.3743159 "hypothetical protein"
"JS1_C14_00325" 5299 "s59E25H2" 0 "hypothetical protein"
"JS1_C14_00325" 5299 "s59E5H2" 3.377655 "hypothetical protein"
"JS1_C14_00325" 5299 "s63E6H2" 4.980331 "hypothetical protein"
"JS1_C14_00326" 5300 "s59E13H2" 0.3201556 "Glycerol-3-phosphate responsive antiterminator"
"JS1_C14_00326" 5300 "s59E25H2" 0 "Glycerol-3-phosphate responsive antiterminator"
"JS1_C14_00326" 5300 "s59E5H2" 2.022392 "Glycerol-3-phosphate responsive antiterminator"
"JS1_C14_00326" 5300 "s63E6H2" 2.305698 "Glycerol-3-phosphate responsive antiterminator"
"JS1_C14_00327" 5301 "s59E13H2" 2.877265 "sn-glycerol-3-phosphate-binding periplasmic protein UgpB precursor"
"JS1_C14_00327" 5301 "s59E25H2" 0.3795455 "sn-glycerol-3-phosphate-binding periplasmic protein UgpB precursor"
"JS1_C14_00327" 5301 "s59E5H2" 1.822592 "sn-glycerol-3-phosphate-binding periplasmic protein UgpB precursor"
"JS1_C14_00327" 5301 "s63E6H2" 4.993155 "sn-glycerol-3-phosphate-binding periplasmic protein UgpB precursor"
"JS1_C14_00328" 5302 "s59E13H2" 2.797175 "sn-glycerol-3-phosphate transport system permease protein UgpA"
"JS1_C14_00328" 5302 "s59E25H2" 0 "sn-glycerol-3-phosphate transport system permease protein UgpA"
"JS1_C14_00328" 5302 "s59E5H2" 1.357053 "sn-glycerol-3-phosphate transport system permease protein UgpA"
"JS1_C14_00328" 5302 "s63E6H2" 0.950688 "sn-glycerol-3-phosphate transport system permease protein UgpA"
"JS1_C14_00329" 5303 "s59E13H2" 0.8666433 "Lactose transport system permease protein LacG"
"JS1_C14_00329" 5303 "s59E25H2" 0 "Lactose transport system permease protein LacG"
"JS1_C14_00329" 5303 "s59E5H2" 1.186256 "Lactose transport system permease protein LacG"
"JS1_C14_00329" 5303 "s63E6H2" 1.099331 "Lactose transport system permease protein LacG"
"JS1_C14_00330" 5304 "s59E13H2" 0.354017 "hypothetical protein"
"JS1_C14_00330" 5304 "s59E25H2" 0 "hypothetical protein"
"JS1_C14_00330" 5304 "s59E5H2" 0.04901729 "hypothetical protein"
"JS1_C14_00330" 5304 "s63E6H2" 2.082999 "hypothetical protein"
"JS1_C14_00331" 5305 "s59E13H2" 0.1464165 "hypothetical protein"
"JS1_C14_00331" 5305 "s59E25H2" 0 "hypothetical protein"
"JS1_C14_00331" 5305 "s59E5H2" 0.7759164 "hypothetical protein"
"JS1_C14_00331" 5305 "s63E6H2" 0.09402925 "hypothetical protein"
"JS1_C14_00332" 5306 "s59E13H2" 0.06648317 "hypothetical protein"
"JS1_C14_00332" 5306 "s59E25H2" 0 "hypothetical protein"
"JS1_C14_00332" 5306 "s59E5H2" 0.4076372 "hypothetical protein"
"JS1_C14_00332" 5306 "s63E6H2" 0.03215431 "hypothetical protein"
"JS1_C14_00333" 5307 "s59E13H2" 0.0256433 "hypothetical protein"
"JS1_C14_00333" 5307 "s59E25H2" 0 "hypothetical protein"
"JS1_C14_00333" 5307 "s59E5H2" 0.4143134 "hypothetical protein"
"JS1_C14_00333" 5307 "s63E6H2" 0.04040671 "hypothetical protein"
"JS1_C14_00334" 5308 "s59E13H2" 0.6070063 "ATP-dependent RecD-like DNA helicase"
"JS1_C14_00334" 5308 "s59E25H2" 0 "ATP-dependent RecD-like DNA helicase"
"JS1_C14_00334" 5308 "s59E5H2" 0.7238436 "ATP-dependent RecD-like DNA helicase"
"JS1_C14_00334" 5308 "s63E6H2" 0.1363335 "ATP-dependent RecD-like DNA helicase"
"JS1_C14_00335" 5309 "s59E13H2" 0.2675285 "2,3-bisphosphoglycerate-independent phosphoglycerate mutase"

"JS1_C14_00335" 5309 "s59E25H2" 0 "2,3-bisphosphoglycerate-independent phosphoglycerate mutase"
"JS1_C14_00335" 5309 "s59E5H2" 0.4405729 "2,3-bisphosphoglycerate-independent phosphoglycerate mutase"
"JS1_C14_00335" 5309 "s63E6H2" 0.07456055 "2,3-bisphosphoglycerate-independent phosphoglycerate mutase"
"JS1_C14_00336" 5310 "s59E13H2" 0.05490397 "putative 2-phosphosulfolactate phosphatase"
"JS1_C14_00336" 5310 "s59E25H2" 0 "putative 2-phosphosulfolactate phosphatase"
"JS1_C14_00336" 5310 "s59E5H2" 0.3239207 "putative 2-phosphosulfolactate phosphatase"
"JS1_C14_00336" 5310 "s63E6H2" 0.08372266 "putative 2-phosphosulfolactate phosphatase"
"JS1_C14_00337" 5311 "s59E13H2" 0.3030435 "Soluble hydrogenase 42 kDa subunit"
"JS1_C14_00337" 5311 "s59E25H2" 0 "Soluble hydrogenase 42 kDa subunit"
"JS1_C14_00337" 5311 "s59E5H2" 0.2092569 "Soluble hydrogenase 42 kDa subunit"
"JS1_C14_00337" 5311 "s63E6H2" 0.07493558 "Soluble hydrogenase 42 kDa subunit"
"JS1_C14_00338" 5312 "s59E13H2" 0.02881336 "VanZ like family protein"
"JS1_C14_00338" 5312 "s59E25H2" 0 "VanZ like family protein"
"JS1_C14_00338" 5312 "s59E5H2" 0.1333982 "VanZ like family protein"
"JS1_C14_00338" 5312 "s63E6H2" 0.0564949 "VanZ like family protein"
"JS1_C14_00339" 5313 "s59E13H2" 0.4807221 "Tetrahedral aminopeptidase"
"JS1_C14_00339" 5313 "s59E25H2" 0 "Tetrahedral aminopeptidase"
"JS1_C14_00339" 5313 "s59E5H2" 0.3695312 "Tetrahedral aminopeptidase"
"JS1_C14_00339" 5313 "s63E6H2" 0.1343325 "Tetrahedral aminopeptidase"
"JS1_C14_00340" 5314 "s59E13H2" 0.145985 "putative A/G-specific adenine glycosylase YfhQ"
"JS1_C14_00340" 5314 "s59E25H2" 0 "putative A/G-specific adenine glycosylase YfhQ"
"JS1_C14_00340" 5314 "s59E5H2" 0.2276367 "putative A/G-specific adenine glycosylase YfhQ"
"JS1_C14_00340" 5314 "s63E6H2" 0.1182264 "putative A/G-specific adenine glycosylase YfhQ"
"JS1_C14_00341" 5315 "s59E13H2" 0.06349728 "Trehalose synthase"
"JS1_C14_00341" 5315 "s59E25H2" 0 "Trehalose synthase"
"JS1_C14_00341" 5315 "s59E5H2" 0.2878799 "Trehalose synthase"
"JS1_C14_00341" 5315 "s63E6H2" 0.1573614 "Trehalose synthase"
"JS1_C14_00342" 5316 "s59E13H2" 0.05151986 "4-alpha-glucanotransferase"
"JS1_C14_00342" 5316 "s59E25H2" 0 "4-alpha-glucanotransferase"
"JS1_C14_00342" 5316 "s59E5H2" 0.1824753 "4-alpha-glucanotransferase"
"JS1_C14_00342" 5316 "s63E6H2" 0.1347836 "4-alpha-glucanotransferase"
"JS1_C14_00343" 5317 "s59E13H2" 0.06787464 "Glycosyl hydrolase family 57"
"JS1_C14_00343" 5317 "s59E25H2" 0 "Glycosyl hydrolase family 57"
"JS1_C14_00343" 5317 "s59E5H2" 0.1006122 "Glycosyl hydrolase family 57"
"JS1_C14_00343" 5317 "s63E6H2" 0.3543318 "Glycosyl hydrolase family 57"
"JS1_C14_00344" 5318 "s59E13H2" 11.14693 "Transposase IS116/IS110/IS902 family protein"
"JS1_C14_00344" 5318 "s59E25H2" 0 "Transposase IS116/IS110/IS902 family protein"
"JS1_C14_00344" 5318 "s59E5H2" 0.5080886 "Transposase IS116/IS110/IS902 family protein"
"JS1_C14_00344" 5318 "s63E6H2" 2.747274 "Transposase IS116/IS110/IS902 family protein"
"JS1_C14_00345" 5319 "s59E13H2" 0.1303971 "Transposase IS116/IS110/IS902 family protein"
"JS1_C14_00345" 5319 "s59E25H2" 0 "Transposase IS116/IS110/IS902 family protein"
"JS1_C14_00345" 5319 "s59E5H2" 0.5107639 "Transposase IS116/IS110/IS902 family protein"
"JS1_C14_00345" 5319 "s63E6H2" 2.385256 "Transposase IS116/IS110/IS902 family protein"
"JS1_C14_00346" 5320 "s59E13H2" 0.006660507 "hypothetical protein"
"JS1_C14_00346" 5320 "s59E25H2" 0 "hypothetical protein"
"JS1_C14_00346" 5320 "s59E5H2" 0 "hypothetical protein"
"JS1_C14_00346" 5320 "s63E6H2" 0 "hypothetical protein"

"JS1_C14_00347" 5321 "s59E13H2" 0.08537185 "6-phosphofructokinase 1"
"JS1_C14_00347" 5321 "s59E25H2" 0 "6-phosphofructokinase 1"
"JS1_C14_00347" 5321 "s59E5H2" 0.07954312 "6-phosphofructokinase 1"
"JS1_C14_00347" 5321 "s63E6H2" 0.3873696 "6-phosphofructokinase 1"
"JS1_C14_00348" 5322 "s59E13H2" 0.07262858 "Fructose-bisphosphate aldolase"
"JS1_C14_00348" 5322 "s59E25H2" 0 "Fructose-bisphosphate aldolase"
"JS1_C14_00348" 5322 "s59E5H2" 0.7139894 "Fructose-bisphosphate aldolase"
"JS1_C14_00348" 5322 "s63E6H2" 0.215054 "Fructose-bisphosphate aldolase"
"JS1_C14_00350" 5324 "s59E13H2" 0.007016028 "hypothetical protein"
"JS1_C14_00350" 5324 "s59E25H2" 0 "hypothetical protein"
"JS1_C14_00350" 5324 "s59E5H2" 0 "hypothetical protein"
"JS1_C14_00350" 5324 "s63E6H2" 0 "hypothetical protein"
"JS1_C14_00351" 5325 "s59E13H2" 0.01476005 "Retroviral aspartyl protease"
"JS1_C14_00351" 5325 "s59E25H2" 0 "Retroviral aspartyl protease"
"JS1_C14_00351" 5325 "s59E5H2" 0 "Retroviral aspartyl protease"
"JS1_C14_00351" 5325 "s63E6H2" 0.001384173 "Retroviral aspartyl protease"
"JS1_C14_00353" 5327 "s59E13H2" 0.07422005 "Exodeoxyribonuclease"
"JS1_C14_00353" 5327 "s59E25H2" 0 "Exodeoxyribonuclease"
"JS1_C14_00353" 5327 "s59E5H2" 0.7568209 "Exodeoxyribonuclease"
"JS1_C14_00353" 5327 "s63E6H2" 0.03679113 "Exodeoxyribonuclease"
"JS1_C14_00354" 5328 "s59E13H2" 0.8869989 "Trifunctional nucleotide phosphoesterase protein YfkN precursor"
"JS1_C14_00354" 5328 "s59E25H2" 0 "Trifunctional nucleotide phosphoesterase protein YfkN precursor"
"JS1_C14_00354" 5328 "s59E5H2" 0.1436232 "Trifunctional nucleotide phosphoesterase protein YfkN precursor"
"JS1_C14_00354" 5328 "s63E6H2" 0.006645683 "Trifunctional nucleotide phosphoesterase protein YfkN precursor"
"JS1_C14_00355" 5329 "s59E13H2" 1.125399 "Ribosome maturation factor RimM"
"JS1_C14_00355" 5329 "s59E25H2" 0 "Ribosome maturation factor RimM"
"JS1_C14_00355" 5329 "s59E5H2" 0.03512542 "Ribosome maturation factor RimM"
"JS1_C14_00355" 5329 "s63E6H2" 0.006092799 "Ribosome maturation factor RimM"
"JS1_C14_00356" 5330 "s59E13H2" 0.01426401 "tRNA (guanine-N(1)-)-methyltransferase"
"JS1_C14_00356" 5330 "s59E25H2" 0 "tRNA (guanine-N(1)-)-methyltransferase"
"JS1_C14_00356" 5330 "s59E5H2" 0.05262939 "tRNA (guanine-N(1)-)-methyltransferase"
"JS1_C14_00356" 5330 "s63E6H2" 0.004449335 "tRNA (guanine-N(1)-)-methyltransferase"
"JS1_C14_00357" 5331 "s59E13H2" 0.09048912 "Signal recognition particle protein"
"JS1_C14_00357" 5331 "s59E25H2" 0 "Signal recognition particle protein"
"JS1_C14_00357" 5331 "s59E5H2" 0.08941784 "Signal recognition particle protein"
"JS1_C14_00357" 5331 "s63E6H2" 0.02195827 "Signal recognition particle protein"
"JS1_C14_00358" 5332 "s59E13H2" 0.01896362 "Signal recognition particle receptor FtsY"
"JS1_C14_00358" 5332 "s59E25H2" 0 "Signal recognition particle receptor FtsY"
"JS1_C14_00358" 5332 "s59E5H2" 0.03971082 "Signal recognition particle receptor FtsY"
"JS1_C14_00358" 5332 "s63E6H2" 0.05850244 "Signal recognition particle receptor FtsY"
"JS1_C14_00359" 5333 "s59E13H2" 0.007785383 "Chromosome partition protein Smc"
"JS1_C14_00359" 5333 "s59E25H2" 0 "Chromosome partition protein Smc"
"JS1_C14_00359" 5333 "s59E5H2" 0.0122299 "Chromosome partition protein Smc"
"JS1_C14_00359" 5333 "s63E6H2" 0.03867281 "Chromosome partition protein Smc"
"JS1_C14_00360" 5334 "s59E13H2" 0.6328403 "4-diphosphocytidyl-2-C-methyl-D-erythritol kinase"
"JS1_C14_00360" 5334 "s59E25H2" 0 "4-diphosphocytidyl-2-C-methyl-D-erythritol kinase"
"JS1_C14_00360" 5334 "s59E5H2" 0.3366168 "4-diphosphocytidyl-2-C-methyl-D-erythritol kinase"

"JS1_C14_00360" 5334 "s63E6H2" 0.3416289 "4-diphosphocytidyl-2-C-methyl-D-erythritol kinase"
"JS1_C14_00361" 5335 "s59E13H2" 0.9330711 "Putative 2-C-methyl-D-erythritol 4-phosphate cytidylyltransferase 2"
"JS1_C14_00361" 5335 "s59E25H2" 0 "Putative 2-C-methyl-D-erythritol 4-phosphate cytidylyltransferase 2"
"JS1_C14_00361" 5335 "s59E5H2" 0.1851984 "Putative 2-C-methyl-D-erythritol 4-phosphate cytidylyltransferase 2"
"JS1_C14_00361" 5335 "s63E6H2" 0.2643117 "Putative 2-C-methyl-D-erythritol 4-phosphate cytidylyltransferase 2"
"JS1_C14_00362" 5336 "s59E13H2" 0.9544517 "hypothetical protein"
"JS1_C14_00362" 5336 "s59E25H2" 0 "hypothetical protein"
"JS1_C14_00362" 5336 "s59E5H2" 0.1671295 "hypothetical protein"
"JS1_C14_00362" 5336 "s63E6H2" 0.1130194 "hypothetical protein"
"JS1_C14_00363" 5337 "s59E13H2" 1.658953 "Rod shape-determining protein MreB"
"JS1_C14_00363" 5337 "s59E25H2" 0 "Rod shape-determining protein MreB"
"JS1_C14_00363" 5337 "s59E5H2" 0.2187707 "Rod shape-determining protein MreB"
"JS1_C14_00363" 5337 "s63E6H2" 0.3994637 "Rod shape-determining protein MreB"
"JS1_C14_00364" 5338 "s59E13H2" 0.6844733 "UDP-N-acetylglucosamine 1-carboxyvinyltransferase 1"
"JS1_C14_00364" 5338 "s59E25H2" 0 "UDP-N-acetylglucosamine 1-carboxyvinyltransferase 1"
"JS1_C14_00364" 5338 "s59E5H2" 0.302465 "UDP-N-acetylglucosamine 1-carboxyvinyltransferase 1"
"JS1_C14_00364" 5338 "s63E6H2" 0.1566175 "UDP-N-acetylglucosamine 1-carboxyvinyltransferase 1"
"JS1_C14_00365" 5339 "s59E13H2" 0.4347077 "Methylthioribose-1-phosphate isomerase"
"JS1_C14_00365" 5339 "s59E25H2" 0 "Methylthioribose-1-phosphate isomerase"
"JS1_C14_00365" 5339 "s59E5H2" 0.2855145 "Methylthioribose-1-phosphate isomerase"
"JS1_C14_00365" 5339 "s63E6H2" 0.1456151 "Methylthioribose-1-phosphate isomerase"
"JS1_C14_00366" 5340 "s59E13H2" 0.3739805 "Putative small multi-drug export protein"
"JS1_C14_00366" 5340 "s59E25H2" 0 "Putative small multi-drug export protein"
"JS1_C14_00366" 5340 "s59E5H2" 0.2575158 "Putative small multi-drug export protein"
"JS1_C14_00366" 5340 "s63E6H2" 0.2331851 "Putative small multi-drug export protein"
"JS1_C14_00367" 5341 "s59E13H2" 0.3729041 "Purine nucleoside phosphorylase 1"
"JS1_C14_00367" 5341 "s59E25H2" 0 "Purine nucleoside phosphorylase 1"
"JS1_C14_00367" 5341 "s59E5H2" 0.2699204 "Purine nucleoside phosphorylase 1"
"JS1_C14_00367" 5341 "s63E6H2" 0.1809712 "Purine nucleoside phosphorylase 1"
"JS1_C14_00368" 5342 "s59E13H2" 0.2733147 "Tyrosine recombinase XerD"
"JS1_C14_00368" 5342 "s59E25H2" 0 "Tyrosine recombinase XerD"
"JS1_C14_00368" 5342 "s59E5H2" 0.224329 "Tyrosine recombinase XerD"
"JS1_C14_00368" 5342 "s63E6H2" 0.1075259 "Tyrosine recombinase XerD"
"JS1_C14_00369" 5343 "s59E13H2" 0.7537009 "Putative lipoprotein YerB precursor"
"JS1_C14_00369" 5343 "s59E25H2" 0 "Putative lipoprotein YerB precursor"
"JS1_C14_00369" 5343 "s59E5H2" 0.3117938 "Putative lipoprotein YerB precursor"
"JS1_C14_00369" 5343 "s63E6H2" 0.1066158 "Putative lipoprotein YerB precursor"
"JS1_C14_00370" 5344 "s59E13H2" 0.05630517 "Restriction endonuclease BglII"
"JS1_C14_00370" 5344 "s59E25H2" 0 "Restriction endonuclease BglII"
"JS1_C14_00370" 5344 "s59E5H2" 0.01388641 "Restriction endonuclease BglII"
"JS1_C14_00370" 5344 "s63E6H2" 0 "Restriction endonuclease BglII"
"JS1_C14_00371" 5345 "s59E13H2" 0.04733127 "DNA replication and repair protein RecF"
"JS1_C14_00371" 5345 "s59E25H2" 0 "DNA replication and repair protein RecF"
"JS1_C14_00371" 5345 "s59E5H2" 0.003514432 "DNA replication and repair protein RecF"
"JS1_C14_00371" 5345 "s63E6H2" 0 "DNA replication and repair protein RecF"
"JS1_C14_00372" 5346 "s59E13H2" 0.1122092 "hypothetical protein"
"JS1_C14_00372" 5346 "s59E25H2" 0 "hypothetical protein"

"JS1_C14_00372" 5346 "s59E5H2" 0.009087812 "hypothetical protein"
"JS1_C14_00372" 5346 "s63E6H2" 0.001164836 "hypothetical protein"
"JS1_C14_00373" 5347 "s59E13H2" 0.1307552 "Inner membrane protein YhaI"
"JS1_C14_00373" 5347 "s59E25H2" 0 "Inner membrane protein YhaI"
"JS1_C14_00373" 5347 "s59E5H2" 0 "Inner membrane protein YhaI"
"JS1_C14_00373" 5347 "s63E6H2" 0.006675298 "Inner membrane protein YhaI"
"JS1_C14_00374" 5348 "s59E13H2" 0.05065486 "hypothetical protein"
"JS1_C14_00374" 5348 "s59E25H2" 0 "hypothetical protein"
"JS1_C14_00374" 5348 "s59E5H2" 0.006960472 "hypothetical protein"
"JS1_C14_00374" 5348 "s63E6H2" 0.1728413 "hypothetical protein"
"JS1_C14_00375" 5349 "s59E13H2" 2.581823 "hypothetical protein"
"JS1_C14_00375" 5349 "s59E25H2" 0.1657559 "hypothetical protein"
"JS1_C14_00375" 5349 "s59E5H2" 1.189805 "hypothetical protein"
"JS1_C14_00375" 5349 "s63E6H2" 0.3019823 "hypothetical protein"
"JS1_C14_00376" 5350 "s59E13H2" 2.277772 "hypothetical protein"
"JS1_C14_00376" 5350 "s59E25H2" 0 "hypothetical protein"
"JS1_C14_00376" 5350 "s59E5H2" 0.7889433 "hypothetical protein"
"JS1_C14_00376" 5350 "s63E6H2" 0.06445086 "hypothetical protein"
"JS1_C14_00377" 5351 "s59E13H2" 2.410201 "putative ATP-dependent RNA helicase"
"JS1_C14_00377" 5351 "s59E25H2" 0 "putative ATP-dependent RNA helicase"
"JS1_C14_00377" 5351 "s59E5H2" 1.394722 "putative ATP-dependent RNA helicase"
"JS1_C14_00377" 5351 "s63E6H2" 0.4386485 "putative ATP-dependent RNA helicase"
"JS1_C14_00378" 5352 "s59E13H2" 1.801467 "Aminopeptidase 2"
"JS1_C14_00378" 5352 "s59E25H2" 0 "Aminopeptidase 2"
"JS1_C14_00378" 5352 "s59E5H2" 1.30838 "Aminopeptidase 2"
"JS1_C14_00378" 5352 "s63E6H2" 0.7849903 "Aminopeptidase 2"
"JS1_C14_00379" 5353 "s59E13H2" 0.02725441 "HTH-type transcriptional regulator MurR"
"JS1_C14_00379" 5353 "s59E25H2" 0 "HTH-type transcriptional regulator MurR"
"JS1_C14_00379" 5353 "s59E5H2" 0 "HTH-type transcriptional regulator MurR"
"JS1_C14_00379" 5353 "s63E6H2" 0.001618424 "HTH-type transcriptional regulator MurR"
"JS1_C14_00380" 5354 "s59E13H2" 0.000920692 "Alanine dehydrogenase"
"JS1_C14_00380" 5354 "s59E25H2" 0 "Alanine dehydrogenase"
"JS1_C14_00380" 5354 "s59E5H2" 0.06032018 "Alanine dehydrogenase"
"JS1_C14_00380" 5354 "s63E6H2" 0 "Alanine dehydrogenase"
"JS1_C14_00381" 5355 "s59E13H2" 0.03143379 "alkanesulfonate transporter substrate-binding subunit"
"JS1_C14_00381" 5355 "s59E25H2" 0 "alkanesulfonate transporter substrate-binding subunit"
"JS1_C14_00381" 5355 "s59E5H2" 0.02016787 "alkanesulfonate transporter substrate-binding subunit"
"JS1_C14_00381" 5355 "s63E6H2" 0.00354783 "alkanesulfonate transporter substrate-binding subunit"
"JS1_C14_00382" 5356 "s59E13H2" 0 "hypothetical protein"
"JS1_C14_00382" 5356 "s59E25H2" 0 "hypothetical protein"
"JS1_C14_00382" 5356 "s59E5H2" 0.2143862 "hypothetical protein"
"JS1_C14_00382" 5356 "s63E6H2" 0.0008851073 "hypothetical protein"
"JS1_C14_00383" 5357 "s59E13H2" 0.3169126 "Sialic acid TRAP transporter permease protein SiaT"
"JS1_C14_00383" 5357 "s59E25H2" 0 "Sialic acid TRAP transporter permease protein SiaT"
"JS1_C14_00383" 5357 "s59E5H2" 0.7115568 "Sialic acid TRAP transporter permease protein SiaT"
"JS1_C14_00383" 5357 "s63E6H2" 0.01366662 "Sialic acid TRAP transporter permease protein SiaT"
"JS1_C14_00384" 5358 "s59E13H2" 0.7637513 "Opine dehydrogenase"

"JS1_C14_00384" 5358 "s59E25H2" 0 "Opine dehydrogenase"
"JS1_C14_00384" 5358 "s59E5H2" 0.1818408 "Opine dehydrogenase"
"JS1_C14_00384" 5358 "s63E6H2" 0.01157392 "Opine dehydrogenase"
"JS1_C14_00385" 5359 "s59E13H2" 10.99218 "Integrase core domain protein"
"JS1_C14_00385" 5359 "s59E25H2" 0 "Integrase core domain protein"
"JS1_C14_00385" 5359 "s59E5H2" 2.697251 "Integrase core domain protein"
"JS1_C14_00385" 5359 "s63E6H2" 2.363429 "Integrase core domain protein"
"JS1_C14_00386" 5360 "s59E13H2" 0.9760388 "hypothetical protein"
"JS1_C14_00386" 5360 "s59E25H2" 0 "hypothetical protein"
"JS1_C14_00386" 5360 "s59E5H2" 1.438588 "hypothetical protein"
"JS1_C14_00386" 5360 "s63E6H2" 0.1032149 "hypothetical protein"
"JS1_C14_00387" 5361 "s59E13H2" 1.535766 "Iron-sulfur flavoprotein"
"JS1_C14_00387" 5361 "s59E25H2" 0 "Iron-sulfur flavoprotein"
"JS1_C14_00387" 5361 "s59E5H2" 0.2552004 "Iron-sulfur flavoprotein"
"JS1_C14_00387" 5361 "s63E6H2" 0.002302279 "Iron-sulfur flavoprotein"
"JS1_C14_00388" 5362 "s59E13H2" 1.115423 "hypothetical protein"
"JS1_C14_00388" 5362 "s59E25H2" 0 "hypothetical protein"
"JS1_C14_00388" 5362 "s59E5H2" 0.169967 "hypothetical protein"
"JS1_C14_00388" 5362 "s63E6H2" 0.005301909 "hypothetical protein"
"JS1_C14_00389" 5363 "s59E13H2" 0.005548887 "PLAT/LH2 domain protein"
"JS1_C14_00389" 5363 "s59E25H2" 0 "PLAT/LH2 domain protein"
"JS1_C14_00389" 5363 "s59E5H2" 0.1715901 "PLAT/LH2 domain protein"
"JS1_C14_00389" 5363 "s63E6H2" 0.0008565498 "PLAT/LH2 domain protein"
"JS1_C14_00390" 5364 "s59E13H2" 0 "hypothetical protein"
"JS1_C14_00390" 5364 "s59E25H2" 0 "hypothetical protein"
"JS1_C14_00390" 5364 "s59E5H2" 0.5755462 "hypothetical protein"
"JS1_C14_00390" 5364 "s63E6H2" 0 "hypothetical protein"
"JS1_C14_00391" 5365 "s59E13H2" 2.118783 "23S rRNA (uridine(2479)-2'-O)-methyltransferase"
"JS1_C14_00391" 5365 "s59E25H2" 0 "23S rRNA (uridine(2479)-2'-O)-methyltransferase"
"JS1_C14_00391" 5365 "s59E5H2" 0.5089783 "23S rRNA (uridine(2479)-2'-O)-methyltransferase"
"JS1_C14_00391" 5365 "s63E6H2" 0.3212897 "23S rRNA (uridine(2479)-2'-O)-methyltransferase"
"JS1_C14_00392" 5366 "s59E13H2" 0.03031043 "dihydrolipoamide dehydrogenase"
"JS1_C14_00392" 5366 "s59E25H2" 0 "dihydrolipoamide dehydrogenase"
"JS1_C14_00392" 5366 "s59E5H2" 0.3881751 "dihydrolipoamide dehydrogenase"
"JS1_C14_00392" 5366 "s63E6H2" 0.2160741 "dihydrolipoamide dehydrogenase"
"JS1_C14_00393" 5367 "s59E13H2" 0.06419714 "5'-nucleotidase SurE"
"JS1_C14_00393" 5367 "s59E25H2" 0 "5'-nucleotidase SurE"
"JS1_C14_00393" 5367 "s59E5H2" 0.5912551 "5'-nucleotidase SurE"
"JS1_C14_00393" 5367 "s63E6H2" 0.2722355 "5'-nucleotidase SurE"
"JS1_C14_00394" 5368 "s59E13H2" 0.04798071 "hypothetical protein"
"JS1_C14_00394" 5368 "s59E25H2" 0 "hypothetical protein"
"JS1_C14_00394" 5368 "s59E5H2" 0.7023583 "hypothetical protein"
"JS1_C14_00394" 5368 "s63E6H2" 0.234503 "hypothetical protein"
"JS1_C14_00395" 5369 "s59E13H2" 0.05357775 "Protein-L-isoaspartate O-methyltransferase"
"JS1_C14_00395" 5369 "s59E25H2" 0 "Protein-L-isoaspartate O-methyltransferase"
"JS1_C14_00395" 5369 "s59E5H2" 0.4088874 "Protein-L-isoaspartate O-methyltransferase"
"JS1_C14_00395" 5369 "s63E6H2" 0.4668436 "Protein-L-isoaspartate O-methyltransferase"

"JS1_C14_00397" 5371 "s59E13H2" 0.08838567 "Vancomycin B-type resistance protein VanW"
"JS1_C14_00397" 5371 "s59E25H2" 0.3377483 "Vancomycin B-type resistance protein VanW"
"JS1_C14_00397" 5371 "s59E5H2" 0.3904176 "Vancomycin B-type resistance protein VanW"
"JS1_C14_00397" 5371 "s63E6H2" 0.5987183 "Vancomycin B-type resistance protein VanW"
"JS1_C14_00398" 5372 "s59E13H2" 0.2313091 "aspartoacylase"
"JS1_C14_00398" 5372 "s59E25H2" 0 "aspartoacylase"
"JS1_C14_00398" 5372 "s59E5H2" 0.5110437 "aspartoacylase"
"JS1_C14_00398" 5372 "s63E6H2" 0.2559771 "aspartoacylase"
"JS1_C14_00399" 5373 "s59E13H2" 6.404402 "Putative peptidyl-prolyl cis-trans isomerase Cbf2 precursor"
"JS1_C14_00399" 5373 "s59E25H2" 0 "Putative peptidyl-prolyl cis-trans isomerase Cbf2 precursor"
"JS1_C14_00399" 5373 "s59E5H2" 0.35115 "Putative peptidyl-prolyl cis-trans isomerase Cbf2 precursor"
"JS1_C14_00399" 5373 "s63E6H2" 0.3325801 "Putative peptidyl-prolyl cis-trans isomerase Cbf2 precursor"
"JS1_C14_00400" 5374 "s59E13H2" 0.150865 "Integrase core domain protein"
"JS1_C14_00400" 5374 "s59E25H2" 0 "Integrase core domain protein"
"JS1_C14_00400" 5374 "s59E5H2" 1.036351 "Integrase core domain protein"
"JS1_C14_00400" 5374 "s63E6H2" 1.571068 "Integrase core domain protein"
"JS1_C14_00401" 5375 "s59E13H2" 0.04902037 "30S ribosomal protein S21"
"JS1_C14_00401" 5375 "s59E25H2" 0 "30S ribosomal protein S21"
"JS1_C14_00401" 5375 "s59E5H2" 0.3331554 "30S ribosomal protein S21"
"JS1_C14_00401" 5375 "s63E6H2" 0.1957274 "30S ribosomal protein S21"
"JS1_C14_00402" 5376 "s59E13H2" 0.04967007 "glutamyl-tRNA(Gln) amidotransferase subunit E"
"JS1_C14_00402" 5376 "s59E25H2" 0 "glutamyl-tRNA(Gln) amidotransferase subunit E"
"JS1_C14_00402" 5376 "s59E5H2" 0.2865458 "glutamyl-tRNA(Gln) amidotransferase subunit E"
"JS1_C14_00402" 5376 "s63E6H2" 0.1344718 "glutamyl-tRNA(Gln) amidotransferase subunit E"
"JS1_C14_00403" 5377 "s59E13H2" 0.08685932 "hypothetical protein"
"JS1_C14_00403" 5377 "s59E25H2" 0 "hypothetical protein"
"JS1_C14_00403" 5377 "s59E5H2" 0.295438 "hypothetical protein"
"JS1_C14_00403" 5377 "s63E6H2" 0.9146633 "hypothetical protein"
"JS1_C14_00404" 5378 "s59E13H2" 0.0318213 "SigmaW regulon antibacterial"
"JS1_C14_00404" 5378 "s59E25H2" 0 "SigmaW regulon antibacterial"
"JS1_C14_00404" 5378 "s59E5H2" 0.2231799 "SigmaW regulon antibacterial"
"JS1_C14_00404" 5378 "s63E6H2" 0.911156 "SigmaW regulon antibacterial"
"JS1_C14_00405" 5379 "s59E13H2" 0.02072767 "hypothetical protein"
"JS1_C14_00405" 5379 "s59E25H2" 0 "hypothetical protein"
"JS1_C14_00405" 5379 "s59E5H2" 0.1421229 "hypothetical protein"
"JS1_C14_00405" 5379 "s63E6H2" 0.2566676 "hypothetical protein"
"JS1_C14_00406" 5380 "s59E13H2" 0.058398 "PhoH-like protein"
"JS1_C14_00406" 5380 "s59E25H2" 0 "PhoH-like protein"
"JS1_C14_00406" 5380 "s59E5H2" 0.3158554 "PhoH-like protein"
"JS1_C14_00406" 5380 "s63E6H2" 0.5633113 "PhoH-like protein"
"JS1_C14_00407" 5381 "s59E13H2" 0.05827848 "7TM-HD extracellular"
"JS1_C14_00407" 5381 "s59E25H2" 0 "7TM-HD extracellular"
"JS1_C14_00407" 5381 "s59E5H2" 0.2460494 "7TM-HD extracellular"
"JS1_C14_00407" 5381 "s63E6H2" 0.4612118 "7TM-HD extracellular"
"JS1_C14_00408" 5382 "s59E13H2" 0.0997106 "Glycine--tRNA ligase beta subunit"
"JS1_C14_00408" 5382 "s59E25H2" 0 "Glycine--tRNA ligase beta subunit"
"JS1_C14_00408" 5382 "s59E5H2" 0.1039263 "Glycine--tRNA ligase beta subunit"

"JS1_C14_00408" 5382 "s63E6H2" 0.05299214 "Glycine--tRNA ligase beta subunit"
"JS1_C14_00409" 5383 "s59E13H2" 0.07289781 "Glycine--tRNA ligase alpha subunit"
"JS1_C14_00409" 5383 "s59E25H2" 0 "Glycine--tRNA ligase alpha subunit"
"JS1_C14_00409" 5383 "s59E5H2" 0.2404927 "Glycine--tRNA ligase alpha subunit"
"JS1_C14_00409" 5383 "s63E6H2" 0.3647504 "Glycine--tRNA ligase alpha subunit"
"JS1_C14_00410" 5384 "s59E13H2" 0.09155392 "DNA repair protein RecO"
"JS1_C14_00410" 5384 "s59E25H2" 0 "DNA repair protein RecO"
"JS1_C14_00410" 5384 "s59E5H2" 0.3671827 "DNA repair protein RecO"
"JS1_C14_00410" 5384 "s63E6H2" 0.7849474 "DNA repair protein RecO"
"JS1_C14_00411" 5385 "s59E13H2" 0.1002765 "Cytidine deaminase"
"JS1_C14_00411" 5385 "s59E25H2" 0 "Cytidine deaminase"
"JS1_C14_00411" 5385 "s59E5H2" 0.475319 "Cytidine deaminase"
"JS1_C14_00411" 5385 "s63E6H2" 0.6967401 "Cytidine deaminase"
"JS1_C14_00412" 5386 "s59E13H2" 0.1895696 "Putative lipoprotein YerB precursor"
"JS1_C14_00412" 5386 "s59E25H2" 0 "Putative lipoprotein YerB precursor"
"JS1_C14_00412" 5386 "s59E5H2" 0.3113311 "Putative lipoprotein YerB precursor"
"JS1_C14_00412" 5386 "s63E6H2" 0.5239685 "Putative lipoprotein YerB precursor"
"JS1_C14_00413" 5387 "s59E13H2" 0.2123015 "Magnesium and cobalt efflux protein CorC"
"JS1_C14_00413" 5387 "s59E25H2" 0 "Magnesium and cobalt efflux protein CorC"
"JS1_C14_00413" 5387 "s59E5H2" 0.3245823 "Magnesium and cobalt efflux protein CorC"
"JS1_C14_00413" 5387 "s63E6H2" 0.3468395 "Magnesium and cobalt efflux protein CorC"
"JS1_C14_00414" 5388 "s59E13H2" 0.2955235 "Magnesium and cobalt efflux protein CorC"
"JS1_C14_00414" 5388 "s59E25H2" 0 "Magnesium and cobalt efflux protein CorC"
"JS1_C14_00414" 5388 "s59E5H2" 0.2735455 "Magnesium and cobalt efflux protein CorC"
"JS1_C14_00414" 5388 "s63E6H2" 0.263578 "Magnesium and cobalt efflux protein CorC"
"JS1_C14_00415" 5389 "s59E13H2" 0.6606531 "Sialic acid TRAP transporter permease protein SiaT"
"JS1_C14_00415" 5389 "s59E25H2" 0 "Sialic acid TRAP transporter permease protein SiaT"
"JS1_C14_00415" 5389 "s59E5H2" 0.04079911 "Sialic acid TRAP transporter permease protein SiaT"
"JS1_C14_00415" 5389 "s63E6H2" 0.304503 "Sialic acid TRAP transporter permease protein SiaT"
"JS1_C14_00416" 5390 "s59E13H2" 0.1519592 "hypothetical protein"
"JS1_C14_00416" 5390 "s59E25H2" 0 "hypothetical protein"
"JS1_C14_00416" 5390 "s59E5H2" 0.0380181 "hypothetical protein"
"JS1_C14_00416" 5390 "s63E6H2" 0.6140321 "hypothetical protein"
"JS1_C14_00417" 5391 "s59E13H2" 0.1492617 "NMT1/THI5 like protein"
"JS1_C14_00417" 5391 "s59E25H2" 0 "NMT1/THI5 like protein"
"JS1_C14_00417" 5391 "s59E5H2" 0.1005328 "NMT1/THI5 like protein"
"JS1_C14_00417" 5391 "s63E6H2" 0.5530146 "NMT1/THI5 like protein"
"JS1_C14_00418" 5392 "s59E13H2" 0.1837946 "Endoribonuclease YbeY"
"JS1_C14_00418" 5392 "s59E25H2" 0 "Endoribonuclease YbeY"
"JS1_C14_00418" 5392 "s59E5H2" 0.3085849 "Endoribonuclease YbeY"
"JS1_C14_00418" 5392 "s63E6H2" 0.4343855 "Endoribonuclease YbeY"
"JS1_C14_00419" 5393 "s59E13H2" 0.1453369 "hypothetical protein"
"JS1_C14_00419" 5393 "s59E25H2" 0 "hypothetical protein"
"JS1_C14_00419" 5393 "s59E5H2" 0.4008709 "hypothetical protein"
"JS1_C14_00419" 5393 "s63E6H2" 0.6075343 "hypothetical protein"
"JS1_C14_00420" 5394 "s59E13H2" 0.4369918 "hypothetical protein"
"JS1_C14_00420" 5394 "s59E25H2" 0.7374631 "hypothetical protein"

"JS1_C14_00420" 5394 "s59E5H2" 0.7180506 "hypothetical protein"
"JS1_C14_00420" 5394 "s63E6H2" 1.717462 "hypothetical protein"
"JS1_C14_00421" 5395 "s59E13H2" 0.1211607 "ATP synthase subunit beta"
"JS1_C14_00421" 5395 "s59E25H2" 0 "ATP synthase subunit beta"
"JS1_C14_00421" 5395 "s59E5H2" 1.568375 "ATP synthase subunit beta"
"JS1_C14_00421" 5395 "s63E6H2" 0.487626 "ATP synthase subunit beta"
"JS1_C14_00422" 5396 "s59E13H2" 0.1610936 "F0F1 ATP synthase subunit epsilon"
"JS1_C14_00422" 5396 "s59E25H2" 0 "F0F1 ATP synthase subunit epsilon"
"JS1_C14_00422" 5396 "s59E5H2" 1.20961 "F0F1 ATP synthase subunit epsilon"
"JS1_C14_00422" 5396 "s63E6H2" 0.5424799 "F0F1 ATP synthase subunit epsilon"
"JS1_C14_00423" 5397 "s59E13H2" 0.1893467 "Putative F0F1-ATPase subunit (ATPase_gene1)"
"JS1_C14_00423" 5397 "s59E25H2" 0 "Putative F0F1-ATPase subunit (ATPase_gene1)"
"JS1_C14_00423" 5397 "s59E5H2" 1.535211 "Putative F0F1-ATPase subunit (ATPase_gene1)"
"JS1_C14_00423" 5397 "s63E6H2" 0.6888736 "Putative F0F1-ATPase subunit (ATPase_gene1)"
"JS1_C14_00424" 5398 "s59E13H2" 0.2603034 "N-ATPase, AtpR subunit"
"JS1_C14_00424" 5398 "s59E25H2" 0 "N-ATPase, AtpR subunit"
"JS1_C14_00424" 5398 "s59E5H2" 2.055006 "N-ATPase, AtpR subunit"
"JS1_C14_00424" 5398 "s63E6H2" 0.8592696 "N-ATPase, AtpR subunit"
"JS1_C14_00425" 5399 "s59E13H2" 0.2143569 "ATP synthase subunit a"
"JS1_C14_00425" 5399 "s59E25H2" 0 "ATP synthase subunit a"
"JS1_C14_00425" 5399 "s59E5H2" 2.361645 "ATP synthase subunit a"
"JS1_C14_00425" 5399 "s63E6H2" 0.9177003 "ATP synthase subunit a"
"JS1_C14_00426" 5400 "s59E13H2" 0.2869311 "ATP synthase subunit c, sodium ion specific"
"JS1_C14_00426" 5400 "s59E25H2" 0 "ATP synthase subunit c, sodium ion specific"
"JS1_C14_00426" 5400 "s59E5H2" 2.263998 "ATP synthase subunit c, sodium ion specific"
"JS1_C14_00426" 5400 "s63E6H2" 1.008442 "ATP synthase subunit c, sodium ion specific"
"JS1_C14_00427" 5401 "s59E13H2" 0.4326466 "ATP synthase subunit b, sodium ion specific"
"JS1_C14_00427" 5401 "s59E25H2" 0 "ATP synthase subunit b, sodium ion specific"
"JS1_C14_00427" 5401 "s59E5H2" 0.5358844 "ATP synthase subunit b, sodium ion specific"
"JS1_C14_00427" 5401 "s63E6H2" 1.073847 "ATP synthase subunit b, sodium ion specific"
"JS1_C14_00428" 5402 "s59E13H2" 0.5138811 "ATP synthase subunit alpha"
"JS1_C14_00428" 5402 "s59E25H2" 0 "ATP synthase subunit alpha"
"JS1_C14_00428" 5402 "s59E5H2" 0.9173777 "ATP synthase subunit alpha"
"JS1_C14_00428" 5402 "s63E6H2" 1.986418 "ATP synthase subunit alpha"
"JS1_C14_00429" 5403 "s59E13H2" 0.315046 "ATP synthase gamma chain, sodium ion specific"
"JS1_C14_00429" 5403 "s59E25H2" 0 "ATP synthase gamma chain, sodium ion specific"
"JS1_C14_00429" 5403 "s59E5H2" 1.154611 "ATP synthase gamma chain, sodium ion specific"
"JS1_C14_00429" 5403 "s63E6H2" 2.053766 "ATP synthase gamma chain, sodium ion specific"
"JS1_C14_00430" 5404 "s59E13H2" 0.285856 "ubiquinone/menaquinone biosynthesis methyltransferase"
"JS1_C14_00430" 5404 "s59E25H2" 0 "ubiquinone/menaquinone biosynthesis methyltransferase"
"JS1_C14_00430" 5404 "s59E5H2" 2.437241 "ubiquinone/menaquinone biosynthesis methyltransferase"
"JS1_C14_00430" 5404 "s63E6H2" 2.117784 "ubiquinone/menaquinone biosynthesis methyltransferase"
"JS1_C14_00431" 5405 "s59E13H2" 2.073416 "hypothetical protein"
"JS1_C14_00431" 5405 "s59E25H2" 0 "hypothetical protein"
"JS1_C14_00431" 5405 "s59E5H2" 1.110569 "hypothetical protein"
"JS1_C14_00431" 5405 "s63E6H2" 4.506245 "hypothetical protein"
"JS1_C14_00432" 5406 "s59E13H2" 0.1636914 "ferredoxin"

"JS1_C14_00432" 5406 "s59E25H2" 0 "ferredoxin"
"JS1_C14_00432" 5406 "s59E5H2" 0.07194869 "ferredoxin"
"JS1_C14_00432" 5406 "s63E6H2" 0.104457 "ferredoxin"
"JS1_C14_00433" 5407 "s59E13H2" 0.7569706 "tetratricopeptide repeat protein"
"JS1_C14_00433" 5407 "s59E25H2" 0 "tetratricopeptide repeat protein"
"JS1_C14_00433" 5407 "s59E5H2" 0.09318282 "tetratricopeptide repeat protein"
"JS1_C14_00433" 5407 "s63E6H2" 2.733849 "tetratricopeptide repeat protein"
"JS1_C14_00434" 5408 "s59E13H2" 0.20425 "hypothetical protein"
"JS1_C14_00434" 5408 "s59E25H2" 0 "hypothetical protein"
"JS1_C14_00434" 5408 "s59E5H2" 0.08470995 "hypothetical protein"
"JS1_C14_00434" 5408 "s63E6H2" 2.056169 "hypothetical protein"
"JS1_C14_00435" 5409 "s59E13H2" 0.2017224 "hypothetical protein"
"JS1_C14_00435" 5409 "s59E25H2" 0 "hypothetical protein"
"JS1_C14_00435" 5409 "s59E5H2" 0.06686155 "hypothetical protein"
"JS1_C14_00435" 5409 "s63E6H2" 0.9309312 "hypothetical protein"
"JS1_C14_00436" 5410 "s59E13H2" 0.1808666 "PEGA domain protein"
"JS1_C14_00436" 5410 "s59E25H2" 0 "PEGA domain protein"
"JS1_C14_00436" 5410 "s59E5H2" 0.08684592 "PEGA domain protein"
"JS1_C14_00436" 5410 "s63E6H2" 0.7851187 "PEGA domain protein"
"JS1_C14_00437" 5411 "s59E13H2" 0.2272096 "PEGA domain protein"
"JS1_C14_00437" 5411 "s59E25H2" 0 "PEGA domain protein"
"JS1_C14_00437" 5411 "s59E5H2" 1.030725 "PEGA domain protein"
"JS1_C14_00437" 5411 "s63E6H2" 1.533558 "PEGA domain protein"
"JS1_C14_00438" 5412 "s59E13H2" 0.7459873 "hypothetical protein"
"JS1_C14_00438" 5412 "s59E25H2" 0 "hypothetical protein"
"JS1_C14_00438" 5412 "s59E5H2" 0.08700296 "hypothetical protein"
"JS1_C14_00438" 5412 "s63E6H2" 0.1501247 "hypothetical protein"
"JS1_C14_00439" 5413 "s59E13H2" 0.7575446 "lipoprotein NlpI"
"JS1_C14_00439" 5413 "s59E25H2" 0 "lipoprotein NlpI"
"JS1_C14_00439" 5413 "s59E5H2" 0.3376456 "lipoprotein NlpI"
"JS1_C14_00439" 5413 "s63E6H2" 0.2622559 "lipoprotein NlpI"
"JS1_C14_00440" 5414 "s59E13H2" 0.1902045 "putative nicotinate-nucleotide adenylyltransferase"
"JS1_C14_00440" 5414 "s59E25H2" 0 "putative nicotinate-nucleotide adenylyltransferase"
"JS1_C14_00440" 5414 "s59E5H2" 0.3337976 "putative nicotinate-nucleotide adenylyltransferase"
"JS1_C14_00440" 5414 "s63E6H2" 0.2278195 "putative nicotinate-nucleotide adenylyltransferase"
"JS1_C14_00441" 5415 "s59E13H2" 0.03662308 "putative nicotinate-nucleotide adenylyltransferase"
"JS1_C14_00441" 5415 "s59E25H2" 0 "putative nicotinate-nucleotide adenylyltransferase"
"JS1_C14_00441" 5415 "s59E5H2" 0.3419459 "putative nicotinate-nucleotide adenylyltransferase"
"JS1_C14_00441" 5415 "s63E6H2" 0.3103302 "putative nicotinate-nucleotide adenylyltransferase"
"JS1_C14_00442" 5416 "s59E13H2" 0.02999433 "hypothetical protein"
"JS1_C14_00442" 5416 "s59E25H2" 0 "hypothetical protein"
"JS1_C14_00442" 5416 "s59E5H2" 0.1203768 "hypothetical protein"
"JS1_C14_00442" 5416 "s63E6H2" 0.2250735 "hypothetical protein"
"JS1_C14_00443" 5417 "s59E13H2" 0.03921578 "Ribosomal silencing factor RsfS"
"JS1_C14_00443" 5417 "s59E25H2" 0 "Ribosomal silencing factor RsfS"
"JS1_C14_00443" 5417 "s59E5H2" 0.2159031 "Ribosomal silencing factor RsfS"
"JS1_C14_00443" 5417 "s63E6H2" 0.3726889 "Ribosomal silencing factor RsfS"

"JS1_C14_00444" 5418 "s59E13H2" 0.08943172 "hypothetical protein"
"JS1_C14_00444" 5418 "s59E25H2" 0 "hypothetical protein"
"JS1_C14_00444" 5418 "s59E5H2" 0.4041602 "hypothetical protein"
"JS1_C14_00444" 5418 "s63E6H2" 0.5026036 "hypothetical protein"
"JS1_C14_00445" 5419 "s59E13H2" 0.04855962 "hypothetical protein"
"JS1_C14_00445" 5419 "s59E25H2" 0 "hypothetical protein"
"JS1_C14_00445" 5419 "s59E5H2" 0.4877105 "hypothetical protein"
"JS1_C14_00445" 5419 "s63E6H2" 0.375982 "hypothetical protein"
"JS1_C14_00447" 5421 "s59E13H2" 0 "hypothetical protein"
"JS1_C14_00447" 5421 "s59E25H2" 0 "hypothetical protein"
"JS1_C14_00447" 5421 "s59E5H2" 0.0005721788 "hypothetical protein"
"JS1_C14_00447" 5421 "s63E6H2" 0.007509711 "hypothetical protein"
"JS1_C14_00448" 5422 "s59E13H2" 0.005166553 "Disaggregatase related repeat protein"
"JS1_C14_00448" 5422 "s59E25H2" 0 "Disaggregatase related repeat protein"
"JS1_C14_00448" 5422 "s59E5H2" 0.3655026 "Disaggregatase related repeat protein"
"JS1_C14_00448" 5422 "s63E6H2" 0.001752967 "Disaggregatase related repeat protein"
"JS1_C14_00449" 5423 "s59E13H2" 0.01075352 "Glutamine synthetase"
"JS1_C14_00449" 5423 "s59E25H2" 0 "Glutamine synthetase"
"JS1_C14_00449" 5423 "s59E5H2" 0.8999094 "Glutamine synthetase"
"JS1_C14_00449" 5423 "s63E6H2" 0 "Glutamine synthetase"
"JS1_C14_00450" 5424 "s59E13H2" 0 "Phosphate acetyltransferase"
"JS1_C14_00450" 5424 "s59E25H2" 0 "Phosphate acetyltransferase"
"JS1_C14_00450" 5424 "s59E5H2" 0.04508636 "Phosphate acetyltransferase"
"JS1_C14_00450" 5424 "s63E6H2" 0 "Phosphate acetyltransferase"
"JS1_C14_00451" 5425 "s59E13H2" 0.29068 "Transcription-repair-coupling factor"
"JS1_C14_00451" 5425 "s59E25H2" 0 "Transcription-repair-coupling factor"
"JS1_C14_00451" 5425 "s59E5H2" 0.4441342 "Transcription-repair-coupling factor"
"JS1_C14_00451" 5425 "s63E6H2" 0.3886714 "Transcription-repair-coupling factor"
"JS1_C14_00452" 5426 "s59E13H2" 0.0240792 "Transposase IS200 like protein"
"JS1_C14_00452" 5426 "s59E25H2" 0 "Transposase IS200 like protein"
"JS1_C14_00452" 5426 "s59E5H2" 0 "Transposase IS200 like protein"
"JS1_C14_00452" 5426 "s63E6H2" 0.02172919 "Transposase IS200 like protein"
"JS1_C14_00453" 5427 "s59E13H2" 0.1009137 "6-phosphofructokinase"
"JS1_C14_00453" 5427 "s59E25H2" 0 "6-phosphofructokinase"
"JS1_C14_00453" 5427 "s59E5H2" 0.182189 "6-phosphofructokinase"
"JS1_C14_00453" 5427 "s63E6H2" 0.3930267 "6-phosphofructokinase"
"JS1_C14_00454" 5428 "s59E13H2" 0.1463615 "Galactose-1-phosphate uridylyltransferase"
"JS1_C14_00454" 5428 "s59E25H2" 0 "Galactose-1-phosphate uridylyltransferase"
"JS1_C14_00454" 5428 "s59E5H2" 0.1736903 "Galactose-1-phosphate uridylyltransferase"
"JS1_C14_00454" 5428 "s63E6H2" 0.3005751 "Galactose-1-phosphate uridylyltransferase"
"JS1_C14_00455" 5429 "s59E13H2" 0.2902563 "Peptidyl-tRNA hydrolase"
"JS1_C14_00455" 5429 "s59E25H2" 0 "Peptidyl-tRNA hydrolase"
"JS1_C14_00455" 5429 "s59E5H2" 0.1088265 "Peptidyl-tRNA hydrolase"
"JS1_C14_00455" 5429 "s63E6H2" 0.510359 "Peptidyl-tRNA hydrolase"
"JS1_C14_00456" 5430 "s59E13H2" 0.07405186 "50S ribosomal protein L25"
"JS1_C14_00456" 5430 "s59E25H2" 0 "50S ribosomal protein L25"
"JS1_C14_00456" 5430 "s59E5H2" 0.5280561 "50S ribosomal protein L25"

"JS1_C14_00456" 5430 "s63E6H2" 0.5457264 "50S ribosomal protein L25"
"JS1_C14_00457" 5431 "s59E13H2" 0.1383985 "Ribose-phosphate pyrophosphokinase"
"JS1_C14_00457" 5431 "s59E25H2" 0 "Ribose-phosphate pyrophosphokinase"
"JS1_C14_00457" 5431 "s59E5H2" 0.3377361 "Ribose-phosphate pyrophosphokinase"
"JS1_C14_00457" 5431 "s63E6H2" 0.7228743 "Ribose-phosphate pyrophosphokinase"
"JS1_C14_00458" 5432 "s59E13H2" 0.08613418 "Bifunctional protein GlmU"
"JS1_C14_00458" 5432 "s59E25H2" 0 "Bifunctional protein GlmU"
"JS1_C14_00458" 5432 "s59E5H2" 0.214932 "Bifunctional protein GlmU"
"JS1_C14_00458" 5432 "s63E6H2" 0.6567928 "Bifunctional protein GlmU"
"JS1_C14_00460" 5434 "s59E13H2" 0.159441 "Putative septation protein SpoVG"
"JS1_C14_00460" 5434 "s59E25H2" 0 "Putative septation protein SpoVG"
"JS1_C14_00460" 5434 "s59E5H2" 0.1300463 "Putative septation protein SpoVG"
"JS1_C14_00460" 5434 "s63E6H2" 0.2162847 "Putative septation protein SpoVG"
"JS1_C14_00461" 5435 "s59E13H2" 0.1845913 "Ribosomal RNA small subunit methyltransferase A"
"JS1_C14_00461" 5435 "s59E25H2" 0 "Ribosomal RNA small subunit methyltransferase A"
"JS1_C14_00461" 5435 "s59E5H2" 0.2046318 "Ribosomal RNA small subunit methyltransferase A"
"JS1_C14_00461" 5435 "s63E6H2" 0.5443721 "Ribosomal RNA small subunit methyltransferase A"
"JS1_C14_00466" 5440 "s59E13H2" 0.1543686 "Replicative DNA helicase"
"JS1_C14_00466" 5440 "s59E25H2" 0 "Replicative DNA helicase"
"JS1_C14_00466" 5440 "s59E5H2" 0.4638799 "Replicative DNA helicase"
"JS1_C14_00466" 5440 "s63E6H2" 1.357396 "Replicative DNA helicase"
"JS1_C14_00467" 5441 "s59E13H2" 0.06887733 "50S ribosomal protein L9"
"JS1_C14_00467" 5441 "s59E25H2" 0 "50S ribosomal protein L9"
"JS1_C14_00467" 5441 "s59E5H2" 0.533735 "50S ribosomal protein L9"
"JS1_C14_00467" 5441 "s63E6H2" 0.6080896 "50S ribosomal protein L9"
"JS1_C14_00468" 5442 "s59E13H2" 0.005391721 "2-keto-3-deoxy-L-rhamnonate aldolase"
"JS1_C14_00468" 5442 "s59E25H2" 0 "2-keto-3-deoxy-L-rhamnonate aldolase"
"JS1_C14_00468" 5442 "s59E5H2" 0 "2-keto-3-deoxy-L-rhamnonate aldolase"
"JS1_C14_00468" 5442 "s63E6H2" 0 "2-keto-3-deoxy-L-rhamnonate aldolase"
"JS1_C14_00469" 5443 "s59E13H2" 1.01253 "hypothetical protein"
"JS1_C14_00469" 5443 "s59E25H2" 0 "hypothetical protein"
"JS1_C14_00469" 5443 "s59E5H2" 0.3564852 "hypothetical protein"
"JS1_C14_00469" 5443 "s63E6H2" 1.819053 "hypothetical protein"
"JS1_C14_00470" 5444 "s59E13H2" 1.294703 "hypothetical protein"
"JS1_C14_00470" 5444 "s59E25H2" 0 "hypothetical protein"
"JS1_C14_00470" 5444 "s59E5H2" 0.1477455 "hypothetical protein"
"JS1_C14_00470" 5444 "s63E6H2" 1.974654 "hypothetical protein"
"JS1_C14_00471" 5445 "s59E13H2" 1.542081 "N-acetylglucosamine-6-phosphate deacetylase"
"JS1_C14_00471" 5445 "s59E25H2" 0 "N-acetylglucosamine-6-phosphate deacetylase"
"JS1_C14_00471" 5445 "s59E5H2" 0.2682939 "N-acetylglucosamine-6-phosphate deacetylase"
"JS1_C14_00471" 5445 "s63E6H2" 1.187837 "N-acetylglucosamine-6-phosphate deacetylase"
"JS1_C14_00472" 5446 "s59E13H2" 1.226515 "hypothetical protein"
"JS1_C14_00472" 5446 "s59E25H2" 0 "hypothetical protein"
"JS1_C14_00472" 5446 "s59E5H2" 0.4390535 "hypothetical protein"
"JS1_C14_00472" 5446 "s63E6H2" 0.02953255 "hypothetical protein"
"JS1_C14_00473" 5447 "s59E13H2" 1.359383 "hypothetical protein"
"JS1_C14_00473" 5447 "s59E25H2" 0 "hypothetical protein"

"JS1_C14_00473" 5447 "s59E5H2" 0.4693484 "hypothetical protein"
"JS1_C14_00473" 5447 "s63E6H2" 0 "hypothetical protein"
"JS1_C14_00474" 5448 "s59E13H2" 1.234474 "Ankyrin repeats (3 copies)"
"JS1_C14_00474" 5448 "s59E25H2" 0 "Ankyrin repeats (3 copies)"
"JS1_C14_00474" 5448 "s59E5H2" 0.1638469 "Ankyrin repeats (3 copies)"
"JS1_C14_00474" 5448 "s63E6H2" 0.2835001 "Ankyrin repeats (3 copies)"
"JS1_C14_00475" 5449 "s59E13H2" 1.171562 "protease TldD"
"JS1_C14_00475" 5449 "s59E25H2" 0 "protease TldD"
"JS1_C14_00475" 5449 "s59E5H2" 0.1453257 "protease TldD"
"JS1_C14_00475" 5449 "s63E6H2" 0.8777587 "protease TldD"
"JS1_C14_00476" 5450 "s59E13H2" 0.8591045 "protease TldD"
"JS1_C14_00476" 5450 "s59E25H2" 0.1298322 "protease TldD"
"JS1_C14_00476" 5450 "s59E5H2" 11.25826 "protease TldD"
"JS1_C14_00476" 5450 "s63E6H2" 0.8216441 "protease TldD"
"JS1_C14_00477" 5451 "s59E13H2" 0.1903033 "3'-5' exoribonuclease YhaM"
"JS1_C14_00477" 5451 "s59E25H2" 0 "3'-5' exoribonuclease YhaM"
"JS1_C14_00477" 5451 "s59E5H2" 0.09058141 "3'-5' exoribonuclease YhaM"
"JS1_C14_00477" 5451 "s63E6H2" 0.5768282 "3'-5' exoribonuclease YhaM"
"JS1_C14_00480" 5454 "s59E13H2" 0.06101397 "hypothetical protein"
"JS1_C14_00480" 5454 "s59E25H2" 0 "hypothetical protein"
"JS1_C14_00480" 5454 "s59E5H2" 0.7250129 "hypothetical protein"
"JS1_C14_00480" 5454 "s63E6H2" 1.51481 "hypothetical protein"
"JS1_C14_00481" 5455 "s59E13H2" 0.5580749 "Multidrug export protein MepA"
"JS1_C14_00481" 5455 "s59E25H2" 0 "Multidrug export protein MepA"
"JS1_C14_00481" 5455 "s59E5H2" 3.950994 "Multidrug export protein MepA"
"JS1_C14_00481" 5455 "s63E6H2" 0.4201975 "Multidrug export protein MepA"
"JS1_C14_00482" 5456 "s59E13H2" 0.08080853 "putative HTH-type transcriptional regulator YusO"
"JS1_C14_00482" 5456 "s59E25H2" 0.6755556 "putative HTH-type transcriptional regulator YusO"
"JS1_C14_00482" 5456 "s59E5H2" 2.82113 "putative HTH-type transcriptional regulator YusO"
"JS1_C14_00482" 5456 "s63E6H2" 0.3117189 "putative HTH-type transcriptional regulator YusO"
"JS1_C14_00484" 5458 "s59E13H2" 0.2657091 "2,3-diketo-L-gulonate-binding periplasmic protein YiaO precursor"
"JS1_C14_00484" 5458 "s59E25H2" 0 "2,3-diketo-L-gulonate-binding periplasmic protein YiaO precursor"
"JS1_C14_00484" 5458 "s59E5H2" 1.710073 "2,3-diketo-L-gulonate-binding periplasmic protein YiaO precursor"
"JS1_C14_00484" 5458 "s63E6H2" 0 "2,3-diketo-L-gulonate-binding periplasmic protein YiaO precursor"
"JS1_C14_00485" 5459 "s59E13H2" 0.217249 "2,3-diketo-L-gulonate TRAP transporter small permease protein YiaM"
"JS1_C14_00485" 5459 "s59E25H2" 0 "2,3-diketo-L-gulonate TRAP transporter small permease protein YiaM"
"JS1_C14_00485" 5459 "s59E5H2" 3.491982 "2,3-diketo-L-gulonate TRAP transporter small permease protein YiaM"
"JS1_C14_00485" 5459 "s63E6H2" 0 "2,3-diketo-L-gulonate TRAP transporter small permease protein YiaM"
"JS1_C14_00486" 5460 "s59E13H2" 0.2277385 "Sialic acid TRAP transporter permease protein SiaT"
"JS1_C14_00486" 5460 "s59E25H2" 0 "Sialic acid TRAP transporter permease protein SiaT"
"JS1_C14_00486" 5460 "s59E5H2" 4.610259 "Sialic acid TRAP transporter permease protein SiaT"
"JS1_C14_00486" 5460 "s63E6H2" 0 "Sialic acid TRAP transporter permease protein SiaT"
"JS1_C14_00487" 5461 "s59E13H2" 1.82955 "hypothetical protein"
"JS1_C14_00487" 5461 "s59E25H2" 0 "hypothetical protein"
"JS1_C14_00487" 5461 "s59E5H2" 2.763268 "hypothetical protein"
"JS1_C14_00487" 5461 "s63E6H2" 0.2159881 "hypothetical protein"
"JS1_C14_00489" 5463 "s59E13H2" 4.473843 "Isoprenylcysteine carboxyl methyltransferase (ICMT) family protein"

"JS1_C14_00489" 5463 "s59E25H2" 0 "Isoprenylcysteine carboxyl methyltransferase (ICMT) family protein"
"JS1_C14_00489" 5463 "s59E5H2" 0.7395638 "Isoprenylcysteine carboxyl methyltransferase (ICMT) family protein"
"JS1_C14_00489" 5463 "s63E6H2" 4.234623 "Isoprenylcysteine carboxyl methyltransferase (ICMT) family protein"
"JS1_C14_00490" 5464 "s59E13H2" 0.2653951 "Exodeoxyribonuclease"
"JS1_C14_00490" 5464 "s59E25H2" 0 "Exodeoxyribonuclease"
"JS1_C14_00490" 5464 "s59E5H2" 1.04245 "Exodeoxyribonuclease"
"JS1_C14_00490" 5464 "s63E6H2" 0.1981091 "Exodeoxyribonuclease"
"JS1_C14_00491" 5465 "s59E13H2" 0.114197 "hypothetical protein"
"JS1_C14_00491" 5465 "s59E25H2" 0 "hypothetical protein"
"JS1_C14_00491" 5465 "s59E5H2" 0.4702056 "hypothetical protein"
"JS1_C14_00491" 5465 "s63E6H2" 3.588889 "hypothetical protein"
"JS1_C14_00492" 5466 "s59E13H2" 0.2062322 "Rubrerythrin"
"JS1_C14_00492" 5466 "s59E25H2" 0 "Rubrerythrin"
"JS1_C14_00492" 5466 "s59E5H2" 0.5816573 "Rubrerythrin"
"JS1_C14_00492" 5466 "s63E6H2" 1.059393 "Rubrerythrin"
"JS1_C14_00493" 5467 "s59E13H2" 0.4467274 "Glyoxalase-like domain protein"
"JS1_C14_00493" 5467 "s59E25H2" 0 "Glyoxalase-like domain protein"
"JS1_C14_00493" 5467 "s59E5H2" 0.1153943 "Glyoxalase-like domain protein"
"JS1_C14_00493" 5467 "s63E6H2" 10.90692 "Glyoxalase-like domain protein"
"JS1_C14_00494" 5468 "s59E13H2" 0.2545409 "FMN reductase [NAD(P)H]"
"JS1_C14_00494" 5468 "s59E25H2" 0 "FMN reductase [NAD(P)H]"
"JS1_C14_00494" 5468 "s59E5H2" 0.06348042 "FMN reductase [NAD(P)H]"
"JS1_C14_00494" 5468 "s63E6H2" 9.317788 "FMN reductase [NAD(P)H]"
"JS1_C14_00497" 5471 "s59E13H2" 0.3292117 "DNA alkylation repair enzyme"
"JS1_C14_00497" 5471 "s59E25H2" 0 "DNA alkylation repair enzyme"
"JS1_C14_00497" 5471 "s59E5H2" 0.505632 "DNA alkylation repair enzyme"
"JS1_C14_00497" 5471 "s63E6H2" 0.2892729 "DNA alkylation repair enzyme"
"JS1_C14_00498" 5472 "s59E13H2" 0.3006952 "Dihydrolipoyl dehydrogenase"
"JS1_C14_00498" 5472 "s59E25H2" 0 "Dihydrolipoyl dehydrogenase"
"JS1_C14_00498" 5472 "s59E5H2" 0.5948432 "Dihydrolipoyl dehydrogenase"
"JS1_C14_00498" 5472 "s63E6H2" 0.06873943 "Dihydrolipoyl dehydrogenase"
"JS1_C14_00499" 5473 "s59E13H2" 0.1969945 "hypothetical protein"
"JS1_C14_00499" 5473 "s59E25H2" 0 "hypothetical protein"
"JS1_C14_00499" 5473 "s59E5H2" 0.3113592 "hypothetical protein"
"JS1_C14_00499" 5473 "s63E6H2" 0.03471362 "hypothetical protein"
"JS1_C14_00500" 5474 "s59E13H2" 2.808052 "putative tRNA-dihydrouridine synthase"
"JS1_C14_00500" 5474 "s59E25H2" 0 "putative tRNA-dihydrouridine synthase"
"JS1_C14_00500" 5474 "s59E5H2" 0.2820565 "putative tRNA-dihydrouridine synthase"
"JS1_C14_00500" 5474 "s63E6H2" 0.3339207 "putative tRNA-dihydrouridine synthase"
"JS1_C14_00501" 5475 "s59E13H2" 0.1742823 "1-acyl-sn-glycerol-3-phosphate acyltransferase"
"JS1_C14_00501" 5475 "s59E25H2" 0 "1-acyl-sn-glycerol-3-phosphate acyltransferase"
"JS1_C14_00501" 5475 "s59E5H2" 0.1408634 "1-acyl-sn-glycerol-3-phosphate acyltransferase"
"JS1_C14_00501" 5475 "s63E6H2" 8.080947 "1-acyl-sn-glycerol-3-phosphate acyltransferase"
"JS1_C14_00502" 5476 "s59E13H2" 0.2099607 "Diacylglycerol kinase"
"JS1_C14_00502" 5476 "s59E25H2" 0 "Diacylglycerol kinase"
"JS1_C14_00502" 5476 "s59E5H2" 0.1789086 "Diacylglycerol kinase"
"JS1_C14_00502" 5476 "s63E6H2" 8.27821 "Diacylglycerol kinase"

"JS1_C14_00503" 5477 "s59E13H2" 1.196551 "Indolepyruvate oxidoreductase subunit IorA"
"JS1_C14_00503" 5477 "s59E25H2" 0 "Indolepyruvate oxidoreductase subunit IorA"
"JS1_C14_00503" 5477 "s59E5H2" 0.9743147 "Indolepyruvate oxidoreductase subunit IorA"
"JS1_C14_00503" 5477 "s63E6H2" 1.081318 "Indolepyruvate oxidoreductase subunit IorA"
"JS1_C14_00504" 5478 "s59E13H2" 0.7464997 "Indolepyruvate oxidoreductase subunit IorB"
"JS1_C14_00504" 5478 "s59E25H2" 0 "Indolepyruvate oxidoreductase subunit IorB"
"JS1_C14_00504" 5478 "s59E5H2" 1.063239 "Indolepyruvate oxidoreductase subunit IorB"
"JS1_C14_00504" 5478 "s63E6H2" 0.6638173 "Indolepyruvate oxidoreductase subunit IorB"
"JS1_C14_00505" 5479 "s59E13H2" 0.7247031 "Aspartate aminotransferase"
"JS1_C14_00505" 5479 "s59E25H2" 0 "Aspartate aminotransferase"
"JS1_C14_00505" 5479 "s59E5H2" 1.168631 "Aspartate aminotransferase"
"JS1_C14_00505" 5479 "s63E6H2" 0.3932954 "Aspartate aminotransferase"
"JS1_C14_00506" 5480 "s59E13H2" 0.7615267 "Butyrate kinase 2"
"JS1_C14_00506" 5480 "s59E25H2" 0 "Butyrate kinase 2"
"JS1_C14_00506" 5480 "s59E5H2" 1.854346 "Butyrate kinase 2"
"JS1_C14_00506" 5480 "s63E6H2" 1.033263 "Butyrate kinase 2"
"JS1_C14_00507" 5481 "s59E13H2" 1.733836 "Phosphate acetyltransferase"
"JS1_C14_00507" 5481 "s59E25H2" 0.2766667 "Phosphate acetyltransferase"
"JS1_C14_00507" 5481 "s59E5H2" 1.93732 "Phosphate acetyltransferase"
"JS1_C14_00507" 5481 "s63E6H2" 1.312107 "Phosphate acetyltransferase"
"JS1_C14_00508" 5482 "s59E13H2" 2.785189 "GTP-binding protein TypA/BipA"
"JS1_C14_00508" 5482 "s59E25H2" 0.1376096 "GTP-binding protein TypA/BipA"
"JS1_C14_00508" 5482 "s59E5H2" 1.202281 "GTP-binding protein TypA/BipA"
"JS1_C14_00508" 5482 "s63E6H2" 8.497524 "GTP-binding protein TypA/BipA"
"JS1_C14_00509" 5483 "s59E13H2" 2.97926 "Transcriptional repressor PagR"
"JS1_C14_00509" 5483 "s59E25H2" 0 "Transcriptional repressor PagR"
"JS1_C14_00509" 5483 "s59E5H2" 0.3280599 "Transcriptional repressor PagR"
"JS1_C14_00509" 5483 "s63E6H2" 0.3454038 "Transcriptional repressor PagR"
"JS1_C14_00510" 5484 "s59E13H2" 1.521951 "Coenzyme A disulfide reductase"
"JS1_C14_00510" 5484 "s59E25H2" 0 "Coenzyme A disulfide reductase"
"JS1_C14_00510" 5484 "s59E5H2" 0.5252786 "Coenzyme A disulfide reductase"
"JS1_C14_00510" 5484 "s63E6H2" 0.1377557 "Coenzyme A disulfide reductase"
"JS1_C14_00511" 5485 "s59E13H2" 1.25756 "sn-glycerol-3-phosphate-binding periplasmic protein UgpB precursor"
"JS1_C14_00511" 5485 "s59E25H2" 0.5641892 "sn-glycerol-3-phosphate-binding periplasmic protein UgpB precursor"
"JS1_C14_00511" 5485 "s59E5H2" 0.09073458 "sn-glycerol-3-phosphate-binding periplasmic protein UgpB precursor"
"JS1_C14_00511" 5485 "s63E6H2" 2.89483 "sn-glycerol-3-phosphate-binding periplasmic protein UgpB precursor"
"JS1_C14_00512" 5486 "s59E13H2" 5.122769 "sn-glycerol-3-phosphate transport system permease protein UgpA"
"JS1_C14_00512" 5486 "s59E25H2" 0 "sn-glycerol-3-phosphate transport system permease protein UgpA"
"JS1_C14_00512" 5486 "s59E5H2" 0.148941 "sn-glycerol-3-phosphate transport system permease protein UgpA"
"JS1_C14_00512" 5486 "s63E6H2" 5.21666 "sn-glycerol-3-phosphate transport system permease protein UgpA"
"JS1_D03_00001" 11910 "s59E13H2" 0.02900085 "30S ribosomal protein S5"
"JS1_D03_00001" 11910 "s59E25H2" 0 "30S ribosomal protein S5"
"JS1_D03_00001" 11910 "s59E5H2" 0.01492793 "30S ribosomal protein S5"
"JS1_D03_00001" 11910 "s63E6H2" 0.03059797 "30S ribosomal protein S5"
"JS1_D03_00002" 11911 "s59E13H2" 0.03566121 "50S ribosomal protein L15"
"JS1_D03_00002" 11911 "s59E25H2" 0 "50S ribosomal protein L15"
"JS1_D03_00002" 11911 "s59E5H2" 0.005146582 "50S ribosomal protein L15"

"JS1_D03_00002" 11911 "s63E6H2" 0.04106622 "50S ribosomal protein L15"
"JS1_D03_00003" 11912 "s59E13H2" 0.02079199 "LL-diaminopimelate aminotransferase"
"JS1_D03_00003" 11912 "s59E25H2" 0 "LL-diaminopimelate aminotransferase"
"JS1_D03_00003" 11912 "s59E5H2" 0 "LL-diaminopimelate aminotransferase"
"JS1_D03_00003" 11912 "s63E6H2" 0.002614748 "LL-diaminopimelate aminotransferase"
"JS1_D03_00004" 11913 "s59E13H2" 0.03643014 "hypothetical protein"
"JS1_D03_00004" 11913 "s59E25H2" 0 "hypothetical protein"
"JS1_D03_00004" 11913 "s59E5H2" 0.09256857 "hypothetical protein"
"JS1_D03_00004" 11913 "s63E6H2" 0.06419953 "hypothetical protein"
"JS1_D03_00005" 11914 "s59E13H2" 0.04615518 "hypothetical protein"
"JS1_D03_00005" 11914 "s59E25H2" 0 "hypothetical protein"
"JS1_D03_00005" 11914 "s59E5H2" 0.1247921 "hypothetical protein"
"JS1_D03_00005" 11914 "s63E6H2" 0.04552294 "hypothetical protein"
"JS1_D03_00007" 11916 "s59E13H2" 0.05873834 "L-seryl-tRNA(Sec) selenium transferase"
"JS1_D03_00007" 11916 "s59E25H2" 0 "L-seryl-tRNA(Sec) selenium transferase"
"JS1_D03_00007" 11916 "s59E5H2" 1.075861 "L-seryl-tRNA(Sec) selenium transferase"
"JS1_D03_00007" 11916 "s63E6H2" 2.591792 "L-seryl-tRNA(Sec) selenium transferase"
"JS1_D03_00008" 11917 "s59E13H2" 0.03584862 "Selenocysteine-specific elongation factor"
"JS1_D03_00008" 11917 "s59E25H2" 0 "Selenocysteine-specific elongation factor"
"JS1_D03_00008" 11917 "s59E5H2" 1.122034 "Selenocysteine-specific elongation factor"
"JS1_D03_00008" 11917 "s63E6H2" 1.50022 "Selenocysteine-specific elongation factor"
"JS1_D03_00009" 11918 "s59E13H2" 0.117692 "Selenide, water dikinase"
"JS1_D03_00009" 11918 "s59E25H2" 0 "Selenide, water dikinase"
"JS1_D03_00009" 11918 "s59E5H2" 0.9204331 "Selenide, water dikinase"
"JS1_D03_00009" 11918 "s63E6H2" 3.729908 "Selenide, water dikinase"
"JS1_D03_00010" 11919 "s59E13H2" 0.004505824 "Putative Ig domain protein"
"JS1_D03_00010" 11919 "s59E25H2" 0 "Putative Ig domain protein"
"JS1_D03_00010" 11919 "s59E5H2" 0.006882339 "Putative Ig domain protein"
"JS1_D03_00010" 11919 "s63E6H2" 0.2429422 "Putative Ig domain protein"
"JS1_D03_00011" 11920 "s59E13H2" 0.02737155 "hypothetical protein"
"JS1_D03_00011" 11920 "s59E25H2" 0 "hypothetical protein"
"JS1_D03_00011" 11920 "s59E5H2" 0.2413747 "hypothetical protein"
"JS1_D03_00011" 11920 "s63E6H2" 0.4967568 "hypothetical protein"
"JS1_D03_00013" 11922 "s59E13H2" 0.01935789 "Uridine phosphorylase"
"JS1_D03_00013" 11922 "s59E25H2" 0 "Uridine phosphorylase"
"JS1_D03_00013" 11922 "s59E5H2" 0.3373799 "Uridine phosphorylase"
"JS1_D03_00013" 11922 "s63E6H2" 1.024425 "Uridine phosphorylase"
"JS1_D03_00014" 11923 "s59E13H2" 0.1696298 "Uridine phosphorylase"
"JS1_D03_00014" 11923 "s59E25H2" 0 "Uridine phosphorylase"
"JS1_D03_00014" 11923 "s59E5H2" 1.063582 "Uridine phosphorylase"
"JS1_D03_00014" 11923 "s63E6H2" 2.369358 "Uridine phosphorylase"
"JS1_D03_00015" 11924 "s59E13H2" 0.01553357 "Purine-binding protein precursor"
"JS1_D03_00015" 11924 "s59E25H2" 0.01950806 "Purine-binding protein precursor"
"JS1_D03_00015" 11924 "s59E5H2" 0.429109 "Purine-binding protein precursor"
"JS1_D03_00015" 11924 "s63E6H2" 2.265858 "Purine-binding protein precursor"
"JS1_D03_00016" 11925 "s59E13H2" 0.005070851 "L-arabinose transporter permease protein"
"JS1_D03_00016" 11925 "s59E25H2" 0.02688172 "L-arabinose transporter permease protein"

"JS1_D03_00016" 11925 "s59E5H2" 0.439704 "L-arabinose transporter permease protein"
"JS1_D03_00016" 11925 "s63E6H2" 1.742579 "L-arabinose transporter permease protein"
"JS1_D03_00017" 11926 "s59E13H2" 0.01234618 "D-allose transporter subunit"
"JS1_D03_00017" 11926 "s59E25H2" 0 "D-allose transporter subunit"
"JS1_D03_00017" 11926 "s59E5H2" 0.3043302 "D-allose transporter subunit"
"JS1_D03_00017" 11926 "s63E6H2" 3.323784 "D-allose transporter subunit"
"JS1_D03_00018" 11927 "s59E13H2" 0.01605311 "Galactose/methyl galactoside import ATP-binding protein MglA"
"JS1_D03_00018" 11927 "s59E25H2" 0 "Galactose/methyl galactoside import ATP-binding protein MglA"
"JS1_D03_00018" 11927 "s59E5H2" 0.08243075 "Galactose/methyl galactoside import ATP-binding protein MglA"
"JS1_D03_00018" 11927 "s63E6H2" 2.335417 "Galactose/methyl galactoside import ATP-binding protein MglA"
"JS1_D03_00019" 11928 "s59E13H2" 0.006883589 "hypothetical protein"
"JS1_D03_00019" 11928 "s59E25H2" 0 "hypothetical protein"
"JS1_D03_00019" 11928 "s59E5H2" 0.2723772 "hypothetical protein"
"JS1_D03_00019" 11928 "s63E6H2" 0.5228567 "hypothetical protein"
"JS1_D03_00020" 11929 "s59E13H2" 0.01953265 "Cell wall-binding protein YocH precursor"
"JS1_D03_00020" 11929 "s59E25H2" 0 "Cell wall-binding protein YocH precursor"
"JS1_D03_00020" 11929 "s59E5H2" 0.08341344 "Cell wall-binding protein YocH precursor"
"JS1_D03_00020" 11929 "s63E6H2" 0.1742277 "Cell wall-binding protein YocH precursor"
"JS1_D03_00021" 11930 "s59E13H2" 0 "Cupin domain protein"
"JS1_D03_00021" 11930 "s59E25H2" 0 "Cupin domain protein"
"JS1_D03_00021" 11930 "s59E5H2" 0 "Cupin domain protein"
"JS1_D03_00021" 11930 "s63E6H2" 0.004007828 "Cupin domain protein"
"JS1_D03_00022" 11931 "s59E13H2" 0.0263759 "5-methylthioadenosine/S-adenosylhomocysteine deaminase"
"JS1_D03_00022" 11931 "s59E25H2" 0.3033033 "5-methylthioadenosine/S-adenosylhomocysteine deaminase"
"JS1_D03_00022" 11931 "s59E5H2" 0.27678 "5-methylthioadenosine/S-adenosylhomocysteine deaminase"
"JS1_D03_00022" 11931 "s63E6H2" 0.6737605 "5-methylthioadenosine/S-adenosylhomocysteine deaminase"
"JS1_D03_00023" 11932 "s59E13H2" 0.9849662 "8-oxoguanine deaminase"
"JS1_D03_00023" 11932 "s59E25H2" 0 "8-oxoguanine deaminase"
"JS1_D03_00023" 11932 "s59E5H2" 0.355501 "8-oxoguanine deaminase"
"JS1_D03_00023" 11932 "s63E6H2" 0.2091159 "8-oxoguanine deaminase"
"JS1_D03_00024" 11933 "s59E13H2" 1.106313 "Nicotinate dehydrogenase small FeS subunit"
"JS1_D03_00024" 11933 "s59E25H2" 0 "Nicotinate dehydrogenase small FeS subunit"
"JS1_D03_00024" 11933 "s59E5H2" 0.1061956 "Nicotinate dehydrogenase small FeS subunit"
"JS1_D03_00024" 11933 "s63E6H2" 0.5793367 "Nicotinate dehydrogenase small FeS subunit"
"JS1_D03_00025" 11934 "s59E13H2" 1.574512 "Glyceraldehyde dehydrogenase medium chain"
"JS1_D03_00025" 11934 "s59E25H2" 0 "Glyceraldehyde dehydrogenase medium chain"
"JS1_D03_00025" 11934 "s59E5H2" 0.2232018 "Glyceraldehyde dehydrogenase medium chain"
"JS1_D03_00025" 11934 "s63E6H2" 0.693779 "Glyceraldehyde dehydrogenase medium chain"
"JS1_D03_00026" 11935 "s59E13H2" 1.976304 "putative xanthine dehydrogenase subunit D"
"JS1_D03_00026" 11935 "s59E25H2" 0 "putative xanthine dehydrogenase subunit D"
"JS1_D03_00026" 11935 "s59E5H2" 0.6687181 "putative xanthine dehydrogenase subunit D"
"JS1_D03_00026" 11935 "s63E6H2" 1.46145 "putative xanthine dehydrogenase subunit D"
"JS1_D03_00027" 11936 "s59E13H2" 0.1258933 "Aspartate carbamoyltransferase"
"JS1_D03_00027" 11936 "s59E25H2" 0 "Aspartate carbamoyltransferase"
"JS1_D03_00027" 11936 "s59E5H2" 0.8135095 "Aspartate carbamoyltransferase"
"JS1_D03_00027" 11936 "s63E6H2" 1.57906 "Aspartate carbamoyltransferase"
"JS1_D03_00028" 11937 "s59E13H2" 0.05310944 "Cysteine synthase"

"JS1_D03_00028" 11937 "s59E25H2" 0 "Cysteine synthase"
"JS1_D03_00028" 11937 "s59E5H2" 0.4827933 "Cysteine synthase"
"JS1_D03_00028" 11937 "s63E6H2" 1.347325 "Cysteine synthase"
"JS1_D03_00029" 11938 "s59E13H2" 0.05763029 "putative succinyl-diaminopimelate desuccinylase"
"JS1_D03_00029" 11938 "s59E25H2" 0 "putative succinyl-diaminopimelate desuccinylase"
"JS1_D03_00029" 11938 "s59E5H2" 0.2225951 "putative succinyl-diaminopimelate desuccinylase"
"JS1_D03_00029" 11938 "s63E6H2" 1.025918 "putative succinyl-diaminopimelate desuccinylase"
"JS1_D03_00030" 11939 "s59E13H2" 0.02063605 "Threonine synthase"
"JS1_D03_00030" 11939 "s59E25H2" 0 "Threonine synthase"
"JS1_D03_00030" 11939 "s59E5H2" 0.1963771 "Threonine synthase"
"JS1_D03_00030" 11939 "s63E6H2" 0.6820756 "Threonine synthase"
"JS1_D03_00031" 11940 "s59E13H2" 0.01552992 "Ornithine carbamoyltransferase"
"JS1_D03_00031" 11940 "s59E25H2" 0 "Ornithine carbamoyltransferase"
"JS1_D03_00031" 11940 "s59E5H2" 0.2143936 "Ornithine carbamoyltransferase"
"JS1_D03_00031" 11940 "s63E6H2" 0.8637039 "Ornithine carbamoyltransferase"
"JS1_D03_00032" 11941 "s59E13H2" 0.01479618 "Carbamate kinase"
"JS1_D03_00032" 11941 "s59E25H2" 0 "Carbamate kinase"
"JS1_D03_00032" 11941 "s59E5H2" 0.07846751 "Carbamate kinase"
"JS1_D03_00032" 11941 "s63E6H2" 0.6580144 "Carbamate kinase"
"JS1_D03_00033" 11942 "s59E13H2" 0.01257765 "Allantoinase"
"JS1_D03_00033" 11942 "s59E25H2" 0 "Allantoinase"
"JS1_D03_00033" 11942 "s59E5H2" 0.1282693 "Allantoinase"
"JS1_D03_00033" 11942 "s63E6H2" 0.1746836 "Allantoinase"
"JS1_D03_00034" 11943 "s59E13H2" 0 "Sialic acid TRAP transporter permease protein SiaT"
"JS1_D03_00034" 11943 "s59E25H2" 0 "Sialic acid TRAP transporter permease protein SiaT"
"JS1_D03_00034" 11943 "s59E5H2" 0.09728302 "Sialic acid TRAP transporter permease protein SiaT"
"JS1_D03_00034" 11943 "s63E6H2" 0.2800829 "Sialic acid TRAP transporter permease protein SiaT"
"JS1_D03_00036" 11945 "s59E13H2" 0.009683276 "hypothetical protein"
"JS1_D03_00036" 11945 "s59E25H2" 0 "hypothetical protein"
"JS1_D03_00036" 11945 "s59E5H2" 0.1443616 "hypothetical protein"
"JS1_D03_00036" 11945 "s63E6H2" 1.450396 "hypothetical protein"
"JS1_D03_00038" 11947 "s59E13H2" 0 "GDP-mannose 4,6-dehydratase"
"JS1_D03_00038" 11947 "s59E25H2" 0 "GDP-mannose 4,6-dehydratase"
"JS1_D03_00038" 11947 "s59E5H2" 0 "GDP-mannose 4,6-dehydratase"
"JS1_D03_00038" 11947 "s63E6H2" 0.006506549 "GDP-mannose 4,6-dehydratase"
"JS1_D03_00039" 11948 "s59E13H2" 0 "GDP-6-deoxy-D-mannose reductase"
"JS1_D03_00039" 11948 "s59E25H2" 0 "GDP-6-deoxy-D-mannose reductase"
"JS1_D03_00039" 11948 "s59E5H2" 0 "GDP-6-deoxy-D-mannose reductase"
"JS1_D03_00039" 11948 "s63E6H2" 0.0006584299 "GDP-6-deoxy-D-mannose reductase"
"JS1_D03_00041" 11950 "s59E13H2" 0 "hypothetical protein"
"JS1_D03_00041" 11950 "s59E25H2" 0 "hypothetical protein"
"JS1_D03_00041" 11950 "s59E5H2" 0 "hypothetical protein"
"JS1_D03_00041" 11950 "s63E6H2" 0.007388675 "hypothetical protein"
"JS1_D03_00042" 11951 "s59E13H2" 0 "50S ribosomal protein L3"
"JS1_D03_00042" 11951 "s59E25H2" 0 "50S ribosomal protein L3"
"JS1_D03_00042" 11951 "s59E5H2" 0 "50S ribosomal protein L3"
"JS1_D03_00042" 11951 "s63E6H2" 0.004107628 "50S ribosomal protein L3"

"JS1_D03_00043" 11952 "s59E13H2" 0.2788765 "30S ribosomal protein S10"
"JS1_D03_00043" 11952 "s59E25H2" 0 "30S ribosomal protein S10"
"JS1_D03_00043" 11952 "s59E5H2" 0 "30S ribosomal protein S10"
"JS1_D03_00043" 11952 "s63E6H2" 0.08546419 "30S ribosomal protein S10"
"JS1_D03_00044" 11953 "s59E13H2" 0.03444225 "Elongation factor Tu"
"JS1_D03_00044" 11953 "s59E25H2" 0 "Elongation factor Tu"
"JS1_D03_00044" 11953 "s59E5H2" 0.04623889 "Elongation factor Tu"
"JS1_D03_00044" 11953 "s63E6H2" 0.1745939 "Elongation factor Tu"
"JS1_D03_00045" 11954 "s59E13H2" 0 "hypothetical protein"
"JS1_D03_00045" 11954 "s59E25H2" 0 "hypothetical protein"
"JS1_D03_00045" 11954 "s59E5H2" 0.07512754 "hypothetical protein"
"JS1_D03_00045" 11954 "s63E6H2" 0.01341892 "hypothetical protein"
"JS1_D03_00047" 11956 "s59E13H2" 0 "hypothetical protein"
"JS1_D03_00047" 11956 "s59E25H2" 0 "hypothetical protein"
"JS1_D03_00047" 11956 "s59E5H2" 0 "hypothetical protein"
"JS1_D03_00047" 11956 "s63E6H2" 0 "hypothetical protein"
"JS1_D03_00048" 11957 "s59E13H2" 0.001402809 "hypothetical protein"
"JS1_D03_00048" 11957 "s59E25H2" 0 "hypothetical protein"
"JS1_D03_00048" 11957 "s59E5H2" 0.2749864 "hypothetical protein"
"JS1_D03_00048" 11957 "s63E6H2" 1.75522 "hypothetical protein"
"JS1_D03_00049" 11958 "s59E13H2" 0 "HNH endonuclease"
"JS1_D03_00049" 11958 "s59E25H2" 0 "HNH endonuclease"
"JS1_D03_00049" 11958 "s59E5H2" 0.1209051 "HNH endonuclease"
"JS1_D03_00049" 11958 "s63E6H2" 0 "HNH endonuclease"
"JS1_D03_00050" 11959 "s59E13H2" 0.001336375 "DNA-directed RNA polymerase subunit beta'"
"JS1_D03_00050" 11959 "s59E25H2" 0 "DNA-directed RNA polymerase subunit beta'"
"JS1_D03_00050" 11959 "s59E5H2" 0.004647113 "DNA-directed RNA polymerase subunit beta'"
"JS1_D03_00050" 11959 "s63E6H2" 0.00492535 "DNA-directed RNA polymerase subunit beta'"
"JS1_D03_00051" 11960 "s59E13H2" 0.002673521 "hypothetical protein"
"JS1_D03_00051" 11960 "s59E25H2" 0.400319 "hypothetical protein"
"JS1_D03_00051" 11960 "s59E5H2" 0.8617557 "hypothetical protein"
"JS1_D03_00051" 11960 "s63E6H2" 1.943424 "hypothetical protein"
"JS1_D03_00052" 11961 "s59E13H2" 0.00274397 "Putative ski2-type helicase"
"JS1_D03_00052" 11961 "s59E25H2" 0 "Putative ski2-type helicase"
"JS1_D03_00052" 11961 "s59E5H2" 0.0904814 "Putative ski2-type helicase"
"JS1_D03_00052" 11961 "s63E6H2" 0.0904496 "Putative ski2-type helicase"
"JS1_D03_00053" 11962 "s59E13H2" 0.9747835 "Cyclic di-GMP phosphodiesterase response regulator RpfG"
"JS1_D03_00053" 11962 "s59E25H2" 0 "Cyclic di-GMP phosphodiesterase response regulator RpfG"
"JS1_D03_00053" 11962 "s59E5H2" 0.2073188 "Cyclic di-GMP phosphodiesterase response regulator RpfG"
"JS1_D03_00053" 11962 "s63E6H2" 0.4309058 "Cyclic di-GMP phosphodiesterase response regulator RpfG"
"JS1_D03_00054" 11963 "s59E13H2" 0 "Helix-hairpin-helix motif protein"
"JS1_D03_00054" 11963 "s59E25H2" 0 "Helix-hairpin-helix motif protein"
"JS1_D03_00054" 11963 "s59E5H2" 0 "Helix-hairpin-helix motif protein"
"JS1_D03_00054" 11963 "s63E6H2" 0.007463193 "Helix-hairpin-helix motif protein"
"JS1_D03_00055" 11964 "s59E13H2" 0 "ATP-dependent helicase HepA"
"JS1_D03_00055" 11964 "s59E25H2" 0 "ATP-dependent helicase HepA"
"JS1_D03_00055" 11964 "s59E5H2" 0.001005944 "ATP-dependent helicase HepA"

"JS1_D03_00055" 11964 "s63E6H2" 0.09276384 "ATP-dependent helicase HepA"
"JS1_D03_00056" 11965 "s59E13H2" 0 "hypothetical protein"
"JS1_D03_00056" 11965 "s59E25H2" 0 "hypothetical protein"
"JS1_D03_00056" 11965 "s59E5H2" 0 "hypothetical protein"
"JS1_D03_00056" 11965 "s63E6H2" 0.01166175 "hypothetical protein"
"JS1_D03_00057" 11966 "s59E13H2" 0 "hypothetical protein"
"JS1_D03_00057" 11966 "s59E25H2" 0 "hypothetical protein"
"JS1_D03_00057" 11966 "s59E5H2" 0 "hypothetical protein"
"JS1_D03_00057" 11966 "s63E6H2" 0.005437067 "hypothetical protein"
"JS1_D03_00059" 11968 "s59E13H2" 0 "Divergent AAA domain protein"
"JS1_D03_00059" 11968 "s59E25H2" 0 "Divergent AAA domain protein"
"JS1_D03_00059" 11968 "s59E5H2" 0.04450414 "Divergent AAA domain protein"
"JS1_D03_00059" 11968 "s63E6H2" 0 "Divergent AAA domain protein"
"JS1_D03_00060" 11969 "s59E13H2" 0 "hypothetical protein"
"JS1_D03_00060" 11969 "s59E25H2" 0 "hypothetical protein"
"JS1_D03_00060" 11969 "s59E5H2" 0 "hypothetical protein"
"JS1_D03_00060" 11969 "s63E6H2" 0 "hypothetical protein"
"JS1_D03_00061" 11970 "s59E13H2" 0 "hypothetical protein"
"JS1_D03_00061" 11970 "s59E25H2" 0 "hypothetical protein"
"JS1_D03_00061" 11970 "s59E5H2" 0 "hypothetical protein"
"JS1_D03_00061" 11970 "s63E6H2" 0.02096307 "hypothetical protein"
"JS1_D03_00062" 11971 "s59E13H2" 0.001238422 "PHP domain protein"
"JS1_D03_00062" 11971 "s59E25H2" 0 "PHP domain protein"
"JS1_D03_00062" 11971 "s59E5H2" 0 "PHP domain protein"
"JS1_D03_00062" 11971 "s63E6H2" 0.004325133 "PHP domain protein"
"JS1_D03_00063" 11972 "s59E13H2" 0 "hypothetical protein"
"JS1_D03_00063" 11972 "s59E25H2" 0 "hypothetical protein"
"JS1_D03_00063" 11972 "s59E5H2" 0.006265118 "hypothetical protein"
"JS1_D03_00063" 11972 "s63E6H2" 0 "hypothetical protein"
"JS1_D03_00064" 11973 "s59E13H2" 0 "hypothetical protein"
"JS1_D03_00064" 11973 "s59E25H2" 0 "hypothetical protein"
"JS1_D03_00064" 11973 "s59E5H2" 0 "hypothetical protein"
"JS1_D03_00064" 11973 "s63E6H2" 0 "hypothetical protein"
"JS1_D03_00066" 11975 "s59E13H2" 0 "Divergent AAA domain protein"
"JS1_D03_00066" 11975 "s59E25H2" 0 "Divergent AAA domain protein"
"JS1_D03_00066" 11975 "s59E5H2" 0 "Divergent AAA domain protein"
"JS1_D03_00066" 11975 "s63E6H2" 0 "Divergent AAA domain protein"
"JS1_D03_00070" 11979 "s59E13H2" 0 "exonuclease III"
"JS1_D03_00070" 11979 "s59E25H2" 0 "exonuclease III"
"JS1_D03_00070" 11979 "s59E5H2" 0 "exonuclease III"
"JS1_D03_00070" 11979 "s63E6H2" 0.1434522 "exonuclease III"
"JS1_D03_00071" 11980 "s59E13H2" 0.03045817 "hypothetical protein"
"JS1_D03_00071" 11980 "s59E25H2" 0 "hypothetical protein"
"JS1_D03_00071" 11980 "s59E5H2" 0 "hypothetical protein"
"JS1_D03_00071" 11980 "s63E6H2" 0.01348109 "hypothetical protein"
"JS1_D03_00072" 11981 "s59E13H2" 0.02257076 "hypothetical protein"
"JS1_D03_00072" 11981 "s59E25H2" 0 "hypothetical protein"

"JS1_D03_00072" 11981 "s59E5H2" 0 "hypothetical protein"
"JS1_D03_00072" 11981 "s63E6H2" 0.03430282 "hypothetical protein"
"JS1_D03_00073" 11982 "s59E13H2" 0.06014542 "Beta-lactamase superfamily domain protein"
"JS1_D03_00073" 11982 "s59E25H2" 0 "Beta-lactamase superfamily domain protein"
"JS1_D03_00073" 11982 "s59E5H2" 0.08947018 "Beta-lactamase superfamily domain protein"
"JS1_D03_00073" 11982 "s63E6H2" 0.1751028 "Beta-lactamase superfamily domain protein"
"JS1_D03_00074" 11983 "s59E13H2" 0 "hypothetical protein"
"JS1_D03_00074" 11983 "s59E25H2" 0 "hypothetical protein"
"JS1_D03_00074" 11983 "s59E5H2" 0.1044981 "hypothetical protein"
"JS1_D03_00074" 11983 "s63E6H2" 0.1549966 "hypothetical protein"
"JS1_D03_00075" 11984 "s59E13H2" 0 "Glucosamine-6-phosphate deaminase 1"
"JS1_D03_00075" 11984 "s59E25H2" 0 "Glucosamine-6-phosphate deaminase 1"
"JS1_D03_00075" 11984 "s59E5H2" 0.03299 "Glucosamine-6-phosphate deaminase 1"
"JS1_D03_00075" 11984 "s63E6H2" 0 "Glucosamine-6-phosphate deaminase 1"
"JS1_D03_00076" 11985 "s59E13H2" 0 "Enoyl-[acyl-carrier-protein] reductase [NADPH] FabL"
"JS1_D03_00076" 11985 "s59E25H2" 0 "Enoyl-[acyl-carrier-protein] reductase [NADPH] FabL"
"JS1_D03_00076" 11985 "s59E5H2" 0.002273074 "Enoyl-[acyl-carrier-protein] reductase [NADPH] FabL"
"JS1_D03_00076" 11985 "s63E6H2" 0 "Enoyl-[acyl-carrier-protein] reductase [NADPH] FabL"
"JS1_D03_00077" 11986 "s59E13H2" 0 "Cupin domain protein"
"JS1_D03_00077" 11986 "s59E25H2" 0 "Cupin domain protein"
"JS1_D03_00077" 11986 "s59E5H2" 0 "Cupin domain protein"
"JS1_D03_00077" 11986 "s63E6H2" 0 "Cupin domain protein"
"JS1_D03_00078" 11987 "s59E13H2" 0 "N-acetylglucosamine-6-phosphate deacetylase"
"JS1_D03_00078" 11987 "s59E25H2" 0 "N-acetylglucosamine-6-phosphate deacetylase"
"JS1_D03_00078" 11987 "s59E5H2" 0 "N-acetylglucosamine-6-phosphate deacetylase"
"JS1_D03_00078" 11987 "s63E6H2" 0 "N-acetylglucosamine-6-phosphate deacetylase"
"JS1_D03_00079" 11988 "s59E13H2" 0 "Trehalose/maltose transport system permease protein MalG"
"JS1_D03_00079" 11988 "s59E25H2" 0 "Trehalose/maltose transport system permease protein MalG"
"JS1_D03_00079" 11988 "s59E5H2" 0 "Trehalose/maltose transport system permease protein MalG"
"JS1_D03_00079" 11988 "s63E6H2" 0 "Trehalose/maltose transport system permease protein MalG"
"JS1_D03_00080" 11989 "s59E13H2" 0 "hypothetical protein"
"JS1_D03_00080" 11989 "s59E25H2" 0 "hypothetical protein"
"JS1_D03_00080" 11989 "s59E5H2" 0 "hypothetical protein"
"JS1_D03_00080" 11989 "s63E6H2" 0 "hypothetical protein"
"JS1_D03_00081" 11990 "s59E13H2" 0 "Trehalose/maltose transport system permease protein MalF"
"JS1_D03_00081" 11990 "s59E25H2" 0 "Trehalose/maltose transport system permease protein MalF"
"JS1_D03_00081" 11990 "s59E5H2" 0 "Trehalose/maltose transport system permease protein MalF"
"JS1_D03_00081" 11990 "s63E6H2" 0 "Trehalose/maltose transport system permease protein MalF"
"JS1_D03_00082" 11991 "s59E13H2" 0 "Trehalose/maltose-binding protein MalE precursor"
"JS1_D03_00082" 11991 "s59E25H2" 0 "Trehalose/maltose-binding protein MalE precursor"
"JS1_D03_00082" 11991 "s59E5H2" 0.04776122 "Trehalose/maltose-binding protein MalE precursor"
"JS1_D03_00082" 11991 "s63E6H2" 0 "Trehalose/maltose-binding protein MalE precursor"
"JS1_D03_00083" 11992 "s59E13H2" 0 "hypothetical protein"
"JS1_D03_00083" 11992 "s59E25H2" 0 "hypothetical protein"
"JS1_D03_00083" 11992 "s59E5H2" 0.05406081 "hypothetical protein"
"JS1_D03_00083" 11992 "s63E6H2" 0 "hypothetical protein"
"JS1_D03_00084" 11993 "s59E13H2" 0 "hypothetical protein"

"JS1_D03_00084" 11993 "s59E25H2" 0 "hypothetical protein"
"JS1_D03_00084" 11993 "s59E5H2" 0 "hypothetical protein"
"JS1_D03_00084" 11993 "s63E6H2" 0 "hypothetical protein"
"JS1_D03_00085" 11994 "s59E13H2" 0.01885198 "Demethylmenaquinone methyltransferase"
"JS1_D03_00085" 11994 "s59E25H2" 0 "Demethylmenaquinone methyltransferase"
"JS1_D03_00085" 11994 "s59E5H2" 0.07220946 "Demethylmenaquinone methyltransferase"
"JS1_D03_00085" 11994 "s63E6H2" 0.2644093 "Demethylmenaquinone methyltransferase"
"JS1_D03_00087" 11996 "s59E13H2" 0 "Trehalose/maltose-binding protein MalE precursor"
"JS1_D03_00087" 11996 "s59E25H2" 0 "Trehalose/maltose-binding protein MalE precursor"
"JS1_D03_00087" 11996 "s59E5H2" 0.05260126 "Trehalose/maltose-binding protein MalE precursor"
"JS1_D03_00087" 11996 "s63E6H2" 0 "Trehalose/maltose-binding protein MalE precursor"
"JS1_D03_00088" 11997 "s59E13H2" 0 "Inner membrane ABC transporter permease protein YcjO"
"JS1_D03_00088" 11997 "s59E25H2" 0 "Inner membrane ABC transporter permease protein YcjO"
"JS1_D03_00088" 11997 "s59E5H2" 0.03915498 "Inner membrane ABC transporter permease protein YcjO"
"JS1_D03_00088" 11997 "s63E6H2" 0 "Inner membrane ABC transporter permease protein YcjO"
"JS1_D03_00089" 11998 "s59E13H2" 0 "Inner membrane ABC transporter permease protein YcjP"
"JS1_D03_00089" 11998 "s59E25H2" 0 "Inner membrane ABC transporter permease protein YcjP"
"JS1_D03_00089" 11998 "s59E5H2" 0.02820041 "Inner membrane ABC transporter permease protein YcjP"
"JS1_D03_00089" 11998 "s63E6H2" 0 "Inner membrane ABC transporter permease protein YcjP"
"JS1_D03_00090" 11999 "s59E13H2" 0 "Mannosylglycerate hydrolase"
"JS1_D03_00090" 11999 "s59E25H2" 0 "Mannosylglycerate hydrolase"
"JS1_D03_00090" 11999 "s59E5H2" 0.03356429 "Mannosylglycerate hydrolase"
"JS1_D03_00090" 11999 "s63E6H2" 6.754766e-05 "Mannosylglycerate hydrolase"
"JS1_D03_00091" 12000 "s59E13H2" 0 "Mannosylglycerate hydrolase"
"JS1_D03_00091" 12000 "s59E25H2" 0 "Mannosylglycerate hydrolase"
"JS1_D03_00091" 12000 "s59E5H2" 0.02513061 "Mannosylglycerate hydrolase"
"JS1_D03_00091" 12000 "s63E6H2" 0.01884761 "Mannosylglycerate hydrolase"
"JS1_D03_00092" 12001 "s59E13H2" 0 "Uroporphyrinogen decarboxylase (URO-D)"
"JS1_D03_00092" 12001 "s59E25H2" 0 "Uroporphyrinogen decarboxylase (URO-D)"
"JS1_D03_00092" 12001 "s59E5H2" 0.004077885 "Uroporphyrinogen decarboxylase (URO-D)"
"JS1_D03_00092" 12001 "s63E6H2" 0 "Uroporphyrinogen decarboxylase (URO-D)"
"JS1_D03_00093" 12002 "s59E13H2" 0.02978491 "Transposase IS200 like protein"
"JS1_D03_00093" 12002 "s59E25H2" 0 "Transposase IS200 like protein"
"JS1_D03_00093" 12002 "s59E5H2" 0.3628705 "Transposase IS200 like protein"
"JS1_D03_00093" 12002 "s63E6H2" 1.06078 "Transposase IS200 like protein"
"JS1_D03_00094" 12003 "s59E13H2" 0 "50S ribosomal protein L3"
"JS1_D03_00094" 12003 "s59E25H2" 0 "50S ribosomal protein L3"
"JS1_D03_00094" 12003 "s59E5H2" 0 "50S ribosomal protein L3"
"JS1_D03_00094" 12003 "s63E6H2" 0.05420389 "50S ribosomal protein L3"
"JS1_D03_00098" 12007 "s59E13H2" 0.002683952 "Arginine biosynthesis bifunctional protein ArgJ"
"JS1_D03_00098" 12007 "s59E25H2" 0 "Arginine biosynthesis bifunctional protein ArgJ"
"JS1_D03_00098" 12007 "s59E5H2" 0 "Arginine biosynthesis bifunctional protein ArgJ"
"JS1_D03_00098" 12007 "s63E6H2" 0.08202636 "Arginine biosynthesis bifunctional protein ArgJ"
"JS1_D03_00100" 12009 "s59E13H2" 0.07757896 "Elongation factor Tu-A"
"JS1_D03_00100" 12009 "s59E25H2" 0 "Elongation factor Tu-A"
"JS1_D03_00100" 12009 "s59E5H2" 0.08916453 "Elongation factor Tu-A"
"JS1_D03_00100" 12009 "s63E6H2" 0.2338019 "Elongation factor Tu-A"

"JS1_D03_00101" 12010 "s59E13H2" 0 "Protein of unknown function (Hypoth_ymh)"
"JS1_D03_00101" 12010 "s59E25H2" 0 "Protein of unknown function (Hypoth_ymh)"
"JS1_D03_00101" 12010 "s59E5H2" 0.001375904 "Protein of unknown function (Hypoth_ymh)"
"JS1_D03_00101" 12010 "s63E6H2" 0 "Protein of unknown function (Hypoth_ymh)"
"JS1_D03_00102" 12011 "s59E13H2" 0.01588966 "F0F1 ATP synthase subunit beta"
"JS1_D03_00102" 12011 "s59E25H2" 0 "F0F1 ATP synthase subunit beta"
"JS1_D03_00102" 12011 "s59E5H2" 0.5617906 "F0F1 ATP synthase subunit beta"
"JS1_D03_00102" 12011 "s63E6H2" 0.1191541 "F0F1 ATP synthase subunit beta"
"JS1_D03_00104" 12013 "s59E13H2" 0 "Copper-sensing transcriptional repressor CsoR"
"JS1_D03_00104" 12013 "s59E25H2" 0 "Copper-sensing transcriptional repressor CsoR"
"JS1_D03_00104" 12013 "s59E5H2" 0 "Copper-sensing transcriptional repressor CsoR"
"JS1_D03_00104" 12013 "s63E6H2" 0.03835285 "Copper-sensing transcriptional repressor CsoR"
"JS1_D03_00105" 12014 "s59E13H2" 0 "30S ribosomal protein S12"
"JS1_D03_00105" 12014 "s59E25H2" 0 "30S ribosomal protein S12"
"JS1_D03_00105" 12014 "s59E5H2" 0.03933544 "30S ribosomal protein S12"
"JS1_D03_00105" 12014 "s63E6H2" 0.06737487 "30S ribosomal protein S12"
"JS1_D03_00106" 12015 "s59E13H2" 0.01573743 "preprotein translocase subunit SecY"
"JS1_D03_00106" 12015 "s59E25H2" 0 "preprotein translocase subunit SecY"
"JS1_D03_00106" 12015 "s59E5H2" 0.01961694 "preprotein translocase subunit SecY"
"JS1_D03_00106" 12015 "s63E6H2" 0.09198742 "preprotein translocase subunit SecY"
"JS1_D03_00109" 12018 "s59E13H2" 0.004577612 "DNA-directed RNA polymerase subunit beta"
"JS1_D03_00109" 12018 "s59E25H2" 0 "DNA-directed RNA polymerase subunit beta"
"JS1_D03_00109" 12018 "s59E5H2" 0 "DNA-directed RNA polymerase subunit beta"
"JS1_D03_00109" 12018 "s63E6H2" 0.004278019 "DNA-directed RNA polymerase subunit beta"
"JS1_D03_00110" 12019 "s59E13H2" 0 "Tyrosine-protein kinase ptk"
"JS1_D03_00110" 12019 "s59E25H2" 0 "Tyrosine-protein kinase ptk"
"JS1_D03_00110" 12019 "s59E5H2" 0.2058206 "Tyrosine-protein kinase ptk"
"JS1_D03_00110" 12019 "s63E6H2" 0.1165507 "Tyrosine-protein kinase ptk"
"JS1_D03_00111" 12020 "s59E13H2" 0 "hypothetical protein"
"JS1_D03_00111" 12020 "s59E25H2" 0 "hypothetical protein"
"JS1_D03_00111" 12020 "s59E5H2" 0 "hypothetical protein"
"JS1_D03_00111" 12020 "s63E6H2" 0.05367852 "hypothetical protein"
"JS1_D03_00112" 12021 "s59E13H2" 0 "Phosphoglucomutase"
"JS1_D03_00112" 12021 "s59E25H2" 0.07273877 "Phosphoglucomutase"
"JS1_D03_00112" 12021 "s59E5H2" 0.1333751 "Phosphoglucomutase"
"JS1_D03_00112" 12021 "s63E6H2" 2.977571 "Phosphoglucomutase"
"JS1_D03_00113" 12022 "s59E13H2" 0 "Tetratricopeptide repeat protein"
"JS1_D03_00113" 12022 "s59E25H2" 0 "Tetratricopeptide repeat protein"
"JS1_D03_00113" 12022 "s59E5H2" 0 "Tetratricopeptide repeat protein"
"JS1_D03_00113" 12022 "s63E6H2" 0.009059333 "Tetratricopeptide repeat protein"
"JS1_D03_00114" 12023 "s59E13H2" 1.905552 "Radical SAM superfamily protein"
"JS1_D03_00114" 12023 "s59E25H2" 0 "Radical SAM superfamily protein"
"JS1_D03_00114" 12023 "s59E5H2" 0.02723716 "Radical SAM superfamily protein"
"JS1_D03_00114" 12023 "s63E6H2" 0.06624658 "Radical SAM superfamily protein"
"JS1_D03_00115" 12024 "s59E13H2" 4.58309 "Ktr system potassium uptake protein A"
"JS1_D03_00115" 12024 "s59E25H2" 0 "Ktr system potassium uptake protein A"
"JS1_D03_00115" 12024 "s59E5H2" 3.971825 "Ktr system potassium uptake protein A"

"JS1_D03_00115" 12024 "s63E6H2" 9.780365 "Ktr system potassium uptake protein A"
"JS1_D03_00116" 12025 "s59E13H2" 1.079357 "D-alanine--D-alanine ligase"
"JS1_D03_00116" 12025 "s59E25H2" 0.2950089 "D-alanine--D-alanine ligase"
"JS1_D03_00116" 12025 "s59E5H2" 4.816951 "D-alanine--D-alanine ligase"
"JS1_D03_00116" 12025 "s63E6H2" 8.153324 "D-alanine--D-alanine ligase"
"JS1_D03_00117" 12026 "s59E13H2" 1.770602 "Methylated-DNA--protein-cysteine methyltransferase"
"JS1_D03_00117" 12026 "s59E25H2" 0.1561905 "Methylated-DNA--protein-cysteine methyltransferase"
"JS1_D03_00117" 12026 "s59E5H2" 4.928398 "Methylated-DNA--protein-cysteine methyltransferase"
"JS1_D03_00117" 12026 "s63E6H2" 11.22152 "Methylated-DNA--protein-cysteine methyltransferase"
"JS1_D03_00118" 12027 "s59E13H2" 2.370308 "MobA-like NTP transferase domain protein"
"JS1_D03_00118" 12027 "s59E25H2" 0 "MobA-like NTP transferase domain protein"
"JS1_D03_00118" 12027 "s59E5H2" 3.988487 "MobA-like NTP transferase domain protein"
"JS1_D03_00118" 12027 "s63E6H2" 13.5678 "MobA-like NTP transferase domain protein"
"JS1_D03_00119" 12028 "s59E13H2" 0.1058508 "hypothetical protein"
"JS1_D03_00119" 12028 "s59E25H2" 0 "hypothetical protein"
"JS1_D03_00119" 12028 "s59E5H2" 2.092894 "hypothetical protein"
"JS1_D03_00119" 12028 "s63E6H2" 11.01973 "hypothetical protein"
"JS1_D03_00120" 12029 "s59E13H2" 0.01237847 "Tetratricopeptide repeat protein"
"JS1_D03_00120" 12029 "s59E25H2" 0 "Tetratricopeptide repeat protein"
"JS1_D03_00120" 12029 "s59E5H2" 2.160803 "Tetratricopeptide repeat protein"
"JS1_D03_00120" 12029 "s63E6H2" 0.05763765 "Tetratricopeptide repeat protein"
"JS1_D03_00121" 12030 "s59E13H2" 0.03456719 "Putative competence-damage inducible protein"
"JS1_D03_00121" 12030 "s59E25H2" 0.1443381 "Putative competence-damage inducible protein"
"JS1_D03_00121" 12030 "s59E5H2" 2.325854 "Putative competence-damage inducible protein"
"JS1_D03_00121" 12030 "s63E6H2" 0.8926278 "Putative competence-damage inducible protein"
"JS1_D03_00122" 12031 "s59E13H2" 0.06187097 "1,3-propanediol dehydrogenase"
"JS1_D03_00122" 12031 "s59E25H2" 0 "1,3-propanediol dehydrogenase"
"JS1_D03_00122" 12031 "s59E5H2" 4.110925 "1,3-propanediol dehydrogenase"
"JS1_D03_00122" 12031 "s63E6H2" 1.985445 "1,3-propanediol dehydrogenase"
"JS1_D03_00123" 12032 "s59E13H2" 0.07145291 "RNA recognition motif. (a.k.a. RRM, RBD, or RNP domain)"
"JS1_D03_00123" 12032 "s59E25H2" 0 "RNA recognition motif. (a.k.a. RRM, RBD, or RNP domain)"
"JS1_D03_00123" 12032 "s59E5H2" 1.092695 "RNA recognition motif. (a.k.a. RRM, RBD, or RNP domain)"
"JS1_D03_00123" 12032 "s63E6H2" 0.3496282 "RNA recognition motif. (a.k.a. RRM, RBD, or RNP domain)"
"JS1_D03_00124" 12033 "s59E13H2" 0.6472619 "Benzylsuccinate synthase alpha subunit"
"JS1_D03_00124" 12033 "s59E25H2" 0 "Benzylsuccinate synthase alpha subunit"
"JS1_D03_00124" 12033 "s59E5H2" 3.85029 "Benzylsuccinate synthase alpha subunit"
"JS1_D03_00124" 12033 "s63E6H2" 0.6082766 "Benzylsuccinate synthase alpha subunit"
"JS1_D03_00125" 12034 "s59E13H2" 0.3970785 "Benzylsuccinate synthase activating enzyme"
"JS1_D03_00125" 12034 "s59E25H2" 0 "Benzylsuccinate synthase activating enzyme"
"JS1_D03_00125" 12034 "s59E5H2" 0.8255877 "Benzylsuccinate synthase activating enzyme"
"JS1_D03_00125" 12034 "s63E6H2" 0.438176 "Benzylsuccinate synthase activating enzyme"
"JS1_D03_00126" 12035 "s59E13H2" 0.3187368 "Transketolase 2"
"JS1_D03_00126" 12035 "s59E25H2" 0 "Transketolase 2"
"JS1_D03_00126" 12035 "s59E5H2" 1.58474 "Transketolase 2"
"JS1_D03_00126" 12035 "s63E6H2" 0.361649 "Transketolase 2"
"JS1_D03_00127" 12036 "s59E13H2" 0.5111407 "Thiamine pyrophosphokinase"
"JS1_D03_00127" 12036 "s59E25H2" 0 "Thiamine pyrophosphokinase"

"JS1_D03_00127" 12036 "s59E5H2" 1.503714 "Thiamine pyrophosphokinase"
"JS1_D03_00127" 12036 "s63E6H2" 0.9598961 "Thiamine pyrophosphokinase"
"JS1_D03_00128" 12037 "s59E13H2" 0.6171804 "Thiamine transporter ThiT"
"JS1_D03_00128" 12037 "s59E25H2" 0 "Thiamine transporter ThiT"
"JS1_D03_00128" 12037 "s59E5H2" 0.8190284 "Thiamine transporter ThiT"
"JS1_D03_00128" 12037 "s63E6H2" 1.31466 "Thiamine transporter ThiT"
"JS1_D03_00130" 12039 "s59E13H2" 0.6585427 "RNA recognition motif. (a.k.a. RRM, RBD, or RNP domain)"
"JS1_D03_00130" 12039 "s59E25H2" 0 "RNA recognition motif. (a.k.a. RRM, RBD, or RNP domain)"
"JS1_D03_00130" 12039 "s59E5H2" 1.617509 "RNA recognition motif. (a.k.a. RRM, RBD, or RNP domain)"
"JS1_D03_00130" 12039 "s63E6H2" 1.451553 "RNA recognition motif. (a.k.a. RRM, RBD, or RNP domain)"
"JS1_D03_00131" 12040 "s59E13H2" 0.1292831 "NAD-dependent protein deacetylase"
"JS1_D03_00131" 12040 "s59E25H2" 0 "NAD-dependent protein deacetylase"
"JS1_D03_00131" 12040 "s59E5H2" 1.125158 "NAD-dependent protein deacetylase"
"JS1_D03_00131" 12040 "s63E6H2" 0.2969236 "NAD-dependent protein deacetylase"
"JS1_D03_00132" 12041 "s59E13H2" 0.0129202 "hypothetical protein"
"JS1_D03_00132" 12041 "s59E25H2" 0 "hypothetical protein"
"JS1_D03_00132" 12041 "s59E5H2" 0.06497767 "hypothetical protein"
"JS1_D03_00132" 12041 "s63E6H2" 0.4131302 "hypothetical protein"
"JS1_D03_00133" 12042 "s59E13H2" 0.01352781 "Ribosomal silencing factor RsfS"
"JS1_D03_00133" 12042 "s59E25H2" 0 "Ribosomal silencing factor RsfS"
"JS1_D03_00133" 12042 "s59E5H2" 0.07773973 "Ribosomal silencing factor RsfS"
"JS1_D03_00133" 12042 "s63E6H2" 0.4509684 "Ribosomal silencing factor RsfS"
"JS1_D03_00134" 12043 "s59E13H2" 0 "hypothetical protein"
"JS1_D03_00134" 12043 "s59E25H2" 0 "hypothetical protein"
"JS1_D03_00134" 12043 "s59E5H2" 0 "hypothetical protein"
"JS1_D03_00134" 12043 "s63E6H2" 0.0001282551 "hypothetical protein"
"JS1_D03_00135" 12044 "s59E13H2" 0 "DNA-directed RNA polymerase subunit beta'"
"JS1_D03_00135" 12044 "s59E25H2" 0 "DNA-directed RNA polymerase subunit beta'"
"JS1_D03_00135" 12044 "s59E5H2" 0 "DNA-directed RNA polymerase subunit beta'"
"JS1_D03_00135" 12044 "s63E6H2" 0.03944301 "DNA-directed RNA polymerase subunit beta'"
"JS1_D03_00137" 12046 "s59E13H2" 0.03433154 "hypothetical protein"
"JS1_D03_00137" 12046 "s59E25H2" 0 "hypothetical protein"
"JS1_D03_00137" 12046 "s59E5H2" 0.1089006 "hypothetical protein"
"JS1_D03_00137" 12046 "s63E6H2" 0.09758959 "hypothetical protein"
"JS1_D03_00140" 12049 "s59E13H2" 0.003941502 "hypothetical protein"
"JS1_D03_00140" 12049 "s59E25H2" 0 "hypothetical protein"
"JS1_D03_00140" 12049 "s59E5H2" 0.0385479 "hypothetical protein"
"JS1_D03_00140" 12049 "s63E6H2" 0.1574536 "hypothetical protein"
"JS1_D03_00141" 12050 "s59E13H2" 0.004721757 "Methionine aminopeptidase 1"
"JS1_D03_00141" 12050 "s59E25H2" 0 "Methionine aminopeptidase 1"
"JS1_D03_00141" 12050 "s59E5H2" 0.01095587 "Methionine aminopeptidase 1"
"JS1_D03_00141" 12050 "s63E6H2" 0.04992975 "Methionine aminopeptidase 1"
"JS1_D03_00142" 12051 "s59E13H2" 0 "Adenylate kinase"
"JS1_D03_00142" 12051 "s59E25H2" 0 "Adenylate kinase"
"JS1_D03_00142" 12051 "s59E5H2" 0.00834288 "Adenylate kinase"
"JS1_D03_00142" 12051 "s63E6H2" 0.03136819 "Adenylate kinase"
"JS1_D03_00144" 12053 "s59E13H2" 0 "putative membrane protein YdfK"

"JS1_D03_00144" 12053 "s59E25H2" 0 "putative membrane protein YdfK"
"JS1_D03_00144" 12053 "s59E5H2" 0.004479558 "putative membrane protein YdfK"
"JS1_D03_00144" 12053 "s63E6H2" 0.06422361 "putative membrane protein YdfK"
"JS1_D03_00145" 12054 "s59E13H2" 0.04957641 "Putative ATP:guanido phosphotransferase"
"JS1_D03_00145" 12054 "s59E25H2" 0 "Putative ATP:guanido phosphotransferase"
"JS1_D03_00145" 12054 "s59E5H2" 0.3627564 "Putative ATP:guanido phosphotransferase"
"JS1_D03_00145" 12054 "s63E6H2" 0.6008704 "Putative ATP:guanido phosphotransferase"
"JS1_D03_00146" 12055 "s59E13H2" 0.3922225 "UvrABC system protein B"
"JS1_D03_00146" 12055 "s59E25H2" 0 "UvrABC system protein B"
"JS1_D03_00146" 12055 "s59E5H2" 0.06994725 "UvrABC system protein B"
"JS1_D03_00146" 12055 "s63E6H2" 0.4375381 "UvrABC system protein B"
"JS1_D03_00148" 12057 "s59E13H2" 0 "hypothetical protein"
"JS1_D03_00148" 12057 "s59E25H2" 0 "hypothetical protein"
"JS1_D03_00148" 12057 "s59E5H2" 0.00417144 "hypothetical protein"
"JS1_D03_00148" 12057 "s63E6H2" 0.3827108 "hypothetical protein"
"JS1_D03_00149" 12058 "s59E13H2" 0.0014201 "Phosphoglycerate kinase"
"JS1_D03_00149" 12058 "s59E25H2" 0 "Phosphoglycerate kinase"
"JS1_D03_00149" 12058 "s59E5H2" 0.09232582 "Phosphoglycerate kinase"
"JS1_D03_00149" 12058 "s63E6H2" 0.6888544 "Phosphoglycerate kinase"
"JS1_D03_00150" 12059 "s59E13H2" 0 "putative membrane protein YdfK"
"JS1_D03_00150" 12059 "s59E25H2" 0 "putative membrane protein YdfK"
"JS1_D03_00150" 12059 "s59E5H2" 0 "putative membrane protein YdfK"
"JS1_D03_00150" 12059 "s63E6H2" 0.04267057 "putative membrane protein YdfK"
"JS1_D03_00151" 12060 "s59E13H2" 0 "hypothetical protein"
"JS1_D03_00151" 12060 "s59E25H2" 0 "hypothetical protein"
"JS1_D03_00151" 12060 "s59E5H2" 0.01551977 "hypothetical protein"
"JS1_D03_00151" 12060 "s63E6H2" 0.0527366 "hypothetical protein"
"JS1_D03_00152" 12061 "s59E13H2" 0 "Ribonuclease 3"
"JS1_D03_00152" 12061 "s59E25H2" 0 "Ribonuclease 3"
"JS1_D03_00152" 12061 "s59E5H2" 0 "Ribonuclease 3"
"JS1_D03_00152" 12061 "s63E6H2" 0.1476581 "Ribonuclease 3"
"JS1_D03_00153" 12062 "s59E13H2" 0 "3-oxoacyl-[acyl-carrier-protein] synthase 2"
"JS1_D03_00153" 12062 "s59E25H2" 0 "3-oxoacyl-[acyl-carrier-protein] synthase 2"
"JS1_D03_00153" 12062 "s59E5H2" 0.002493109 "3-oxoacyl-[acyl-carrier-protein] synthase 2"
"JS1_D03_00153" 12062 "s63E6H2" 0.2295935 "3-oxoacyl-[acyl-carrier-protein] synthase 2"
"JS1_D03_00154" 12063 "s59E13H2" 0 "Acyl carrier protein"
"JS1_D03_00154" 12063 "s59E25H2" 0 "Acyl carrier protein"
"JS1_D03_00154" 12063 "s59E5H2" 0 "Acyl carrier protein"
"JS1_D03_00154" 12063 "s63E6H2" 0.1509285 "Acyl carrier protein"
"JS1_D03_00155" 12064 "s59E13H2" 0 "3-oxoacyl-[acyl-carrier-protein] reductase FabG"
"JS1_D03_00155" 12064 "s59E25H2" 0 "3-oxoacyl-[acyl-carrier-protein] reductase FabG"
"JS1_D03_00155" 12064 "s59E5H2" 0 "3-oxoacyl-[acyl-carrier-protein] reductase FabG"
"JS1_D03_00155" 12064 "s63E6H2" 0.1425093 "3-oxoacyl-[acyl-carrier-protein] reductase FabG"
"JS1_D03_00156" 12065 "s59E13H2" 0 "Malonyl CoA-acyl carrier protein transacylase"
"JS1_D03_00156" 12065 "s59E25H2" 0 "Malonyl CoA-acyl carrier protein transacylase"
"JS1_D03_00156" 12065 "s59E5H2" 0 "Malonyl CoA-acyl carrier protein transacylase"
"JS1_D03_00156" 12065 "s63E6H2" 0.128739 "Malonyl CoA-acyl carrier protein transacylase"

"JS1_D03_00157" 12066 "s59E13H2" 0 "3-oxoacyl-[acyl-carrier-protein] synthase 3"
"JS1_D03_00157" 12066 "s59E25H2" 0 "3-oxoacyl-[acyl-carrier-protein] synthase 3"
"JS1_D03_00157" 12066 "s59E5H2" 0.003087949 "3-oxoacyl-[acyl-carrier-protein] synthase 3"
"JS1_D03_00157" 12066 "s63E6H2" 0.09650487 "3-oxoacyl-[acyl-carrier-protein] synthase 3"
"JS1_D03_00158" 12067 "s59E13H2" 0 "Phosphate acyltransferase"
"JS1_D03_00158" 12067 "s59E25H2" 0 "Phosphate acyltransferase"
"JS1_D03_00158" 12067 "s59E5H2" 0 "Phosphate acyltransferase"
"JS1_D03_00158" 12067 "s63E6H2" 0.08999586 "Phosphate acyltransferase"
"JS1_D03_00159" 12068 "s59E13H2" 0.02151739 "Transcription factor FapR"
"JS1_D03_00159" 12068 "s59E25H2" 0 "Transcription factor FapR"
"JS1_D03_00159" 12068 "s59E5H2" 0.03319545 "Transcription factor FapR"
"JS1_D03_00159" 12068 "s63E6H2" 0.1084967 "Transcription factor FapR"
"JS1_D03_00160" 12069 "s59E13H2" 0.003407538 "50S ribosomal protein L32"
"JS1_D03_00160" 12069 "s59E25H2" 0 "50S ribosomal protein L32"
"JS1_D03_00160" 12069 "s59E5H2" 0 "50S ribosomal protein L32"
"JS1_D03_00160" 12069 "s63E6H2" 0.124779 "50S ribosomal protein L32"
"JS1_D03_00161" 12070 "s59E13H2" 0.002406708 "Acetate kinase"
"JS1_D03_00161" 12070 "s59E25H2" 0 "Acetate kinase"
"JS1_D03_00161" 12070 "s59E5H2" 0 "Acetate kinase"
"JS1_D03_00161" 12070 "s63E6H2" 0.09323844 "Acetate kinase"
"JS1_D03_00162" 12071 "s59E13H2" 0.07713627 "Phosphate acetyltransferase"
"JS1_D03_00162" 12071 "s59E25H2" 0 "Phosphate acetyltransferase"
"JS1_D03_00162" 12071 "s59E5H2" 0.05028549 "Phosphate acetyltransferase"
"JS1_D03_00162" 12071 "s63E6H2" 0.1024257 "Phosphate acetyltransferase"
"JS1_D03_00163" 12072 "s59E13H2" 0.003370019 "hypothetical protein"
"JS1_D03_00163" 12072 "s59E25H2" 0 "hypothetical protein"
"JS1_D03_00163" 12072 "s59E5H2" 0 "hypothetical protein"
"JS1_D03_00163" 12072 "s63E6H2" 0.05944194 "hypothetical protein"
"JS1_D03_00164" 12073 "s59E13H2" 0 "Lon protease"
"JS1_D03_00164" 12073 "s59E25H2" 0 "Lon protease"
"JS1_D03_00164" 12073 "s59E5H2" 0 "Lon protease"
"JS1_D03_00164" 12073 "s63E6H2" 0.09411573 "Lon protease"
"JS1_D03_00165" 12074 "s59E13H2" 0.5967443 "Phosphopantetheine adenylyltransferase"
"JS1_D03_00165" 12074 "s59E25H2" 0 "Phosphopantetheine adenylyltransferase"
"JS1_D03_00165" 12074 "s59E5H2" 0.0709802 "Phosphopantetheine adenylyltransferase"
"JS1_D03_00165" 12074 "s63E6H2" 0.1647376 "Phosphopantetheine adenylyltransferase"
"JS1_D03_00166" 12075 "s59E13H2" 0.007786296 "Ribosomal RNA small subunit methyltransferase D"
"JS1_D03_00166" 12075 "s59E25H2" 0 "Ribosomal RNA small subunit methyltransferase D"
"JS1_D03_00166" 12075 "s59E5H2" 0.04034149 "Ribosomal RNA small subunit methyltransferase D"
"JS1_D03_00166" 12075 "s63E6H2" 0.05734688 "Ribosomal RNA small subunit methyltransferase D"
"JS1_D03_00167" 12076 "s59E13H2" 0.4253216 "50S ribosomal protein L28"
"JS1_D03_00167" 12076 "s59E25H2" 0 "50S ribosomal protein L28"
"JS1_D03_00167" 12076 "s59E5H2" 0.09744195 "50S ribosomal protein L28"
"JS1_D03_00167" 12076 "s63E6H2" 0.1621787 "50S ribosomal protein L28"
"JS1_D03_00168" 12077 "s59E13H2" 1.275508 "4-hydroxy-3-methylbut-2-enyl diphosphate reductase"
"JS1_D03_00168" 12077 "s59E25H2" 0.2998805 "4-hydroxy-3-methylbut-2-enyl diphosphate reductase"
"JS1_D03_00168" 12077 "s59E5H2" 0.03620039 "4-hydroxy-3-methylbut-2-enyl diphosphate reductase"

"JS1_D03_00168" 12077 "s63E6H2" 0.1367876 "4-hydroxy-3-methylbut-2-enyl diphosphate reductase"
"JS1_D03_00169" 12078 "s59E13H2" 2.185387 "Cytidylate kinase"
"JS1_D03_00169" 12078 "s59E25H2" 0.1082982 "Cytidylate kinase"
"JS1_D03_00169" 12078 "s59E5H2" 0.01420522 "Cytidylate kinase"
"JS1_D03_00169" 12078 "s63E6H2" 0.0926429 "Cytidylate kinase"
"JS1_D03_00170" 12079 "s59E13H2" 1.005093 "Ribosomal large subunit pseudouridine synthase B"
"JS1_D03_00170" 12079 "s59E25H2" 0 "Ribosomal large subunit pseudouridine synthase B"
"JS1_D03_00170" 12079 "s59E5H2" 0.006475761 "Ribosomal large subunit pseudouridine synthase B"
"JS1_D03_00170" 12079 "s63E6H2" 0.05385112 "Ribosomal large subunit pseudouridine synthase B"
"JS1_D03_00171" 12080 "s59E13H2" 0.001215815 "Segregation and condensation protein B"
"JS1_D03_00171" 12080 "s59E25H2" 0 "Segregation and condensation protein B"
"JS1_D03_00171" 12080 "s59E5H2" 0.04501062 "Segregation and condensation protein B"
"JS1_D03_00171" 12080 "s63E6H2" 0.04198664 "Segregation and condensation protein B"
"JS1_D03_00172" 12081 "s59E13H2" 0 "Segregation and condensation protein A"
"JS1_D03_00172" 12081 "s59E25H2" 0 "Segregation and condensation protein A"
"JS1_D03_00172" 12081 "s59E5H2" 0.007891425 "Segregation and condensation protein A"
"JS1_D03_00172" 12081 "s63E6H2" 0.02102019 "Segregation and condensation protein A"
"JS1_D03_00173" 12082 "s59E13H2" 0 "Tryptophan--tRNA ligase"
"JS1_D03_00173" 12082 "s59E25H2" 0 "Tryptophan--tRNA ligase"
"JS1_D03_00173" 12082 "s59E5H2" 0.04860235 "Tryptophan--tRNA ligase"
"JS1_D03_00173" 12082 "s63E6H2" 0.02667619 "Tryptophan--tRNA ligase"
"JS1_D03_00174" 12083 "s59E13H2" 0 "Peptidase family M50"
"JS1_D03_00174" 12083 "s59E25H2" 0 "Peptidase family M50"
"JS1_D03_00174" 12083 "s59E5H2" 0.02154059 "Peptidase family M50"
"JS1_D03_00174" 12083 "s63E6H2" 0.006792293 "Peptidase family M50"
"JS1_D03_00175" 12084 "s59E13H2" 0.001954943 "Multifunctional CCA protein"
"JS1_D03_00175" 12084 "s59E25H2" 0 "Multifunctional CCA protein"
"JS1_D03_00175" 12084 "s59E5H2" 0.01506954 "Multifunctional CCA protein"
"JS1_D03_00175" 12084 "s63E6H2" 0.02528202 "Multifunctional CCA protein"
"JS1_D03_00176" 12085 "s59E13H2" 0 "Pyrimidine-nucleoside phosphorylase"
"JS1_D03_00176" 12085 "s59E25H2" 0 "Pyrimidine-nucleoside phosphorylase"
"JS1_D03_00176" 12085 "s59E5H2" 0.0117471 "Pyrimidine-nucleoside phosphorylase"
"JS1_D03_00176" 12085 "s63E6H2" 0.01041576 "Pyrimidine-nucleoside phosphorylase"
"JS1_D03_00177" 12086 "s59E13H2" 0.01645626 "Putative K(+)-stimulated pyrophosphate-energized sodium pump"
"JS1_D03_00177" 12086 "s59E25H2" 0 "Putative K(+)-stimulated pyrophosphate-energized sodium pump"
"JS1_D03_00177" 12086 "s59E5H2" 0.1654587 "Putative K(+)-stimulated pyrophosphate-energized sodium pump"
"JS1_D03_00177" 12086 "s63E6H2" 0.07870282 "Putative K(+)-stimulated pyrophosphate-energized sodium pump"
"JS1_D03_00178" 12087 "s59E13H2" 0.02827464 "Pyruvate, phosphate dikinase"
"JS1_D03_00178" 12087 "s59E25H2" 0 "Pyruvate, phosphate dikinase"
"JS1_D03_00178" 12087 "s59E5H2" 0.07497623 "Pyruvate, phosphate dikinase"
"JS1_D03_00178" 12087 "s63E6H2" 0.0831745 "Pyruvate, phosphate dikinase"
"JS1_D03_00179" 12088 "s59E13H2" 0.2549322 "hypothetical protein"
"JS1_D03_00179" 12088 "s59E25H2" 0 "hypothetical protein"
"JS1_D03_00179" 12088 "s59E5H2" 0.006765201 "hypothetical protein"
"JS1_D03_00179" 12088 "s63E6H2" 0.08701273 "hypothetical protein"
"JS1_D03_00180" 12089 "s59E13H2" 0.6552344 "Macrolide export ATP-binding/permease protein MacB"
"JS1_D03_00180" 12089 "s59E25H2" 0 "Macrolide export ATP-binding/permease protein MacB"

"JS1_D03_00180" 12089 "s59E5H2" 0.053615 "Macrolide export ATP-binding/permease protein MacB"
"JS1_D03_00180" 12089 "s63E6H2" 0.3147328 "Macrolide export ATP-binding/permease protein MacB"
"JS1_D03_00181" 12090 "s59E13H2" 0.02718729 "putative ABC transporter ATP-binding protein"
"JS1_D03_00181" 12090 "s59E25H2" 0 "putative ABC transporter ATP-binding protein"
"JS1_D03_00181" 12090 "s59E5H2" 0.005850321 "putative ABC transporter ATP-binding protein"
"JS1_D03_00181" 12090 "s63E6H2" 0.04133917 "putative ABC transporter ATP-binding protein"
"JS1_D03_00182" 12091 "s59E13H2" 0.05211359 "putative ABC transporter ATP-binding protein"
"JS1_D03_00182" 12091 "s59E25H2" 0 "putative ABC transporter ATP-binding protein"
"JS1_D03_00182" 12091 "s59E5H2" 0.05971435 "putative ABC transporter ATP-binding protein"
"JS1_D03_00182" 12091 "s63E6H2" 0.03100692 "putative ABC transporter ATP-binding protein"
"JS1_D03_00183" 12092 "s59E13H2" 0.02309413 "hypothetical protein"
"JS1_D03_00183" 12092 "s59E25H2" 0 "hypothetical protein"
"JS1_D03_00183" 12092 "s59E5H2" 0.0657577 "hypothetical protein"
"JS1_D03_00183" 12092 "s63E6H2" 0.04150766 "hypothetical protein"
"JS1_D03_00184" 12093 "s59E13H2" 0.1304089 "Dihydrolipoyllysine-residue acetyltransferase component of pyruvate dehydrogenase complex"
"JS1_D03_00184" 12093 "s59E25H2" 0 "Dihydrolipoyllysine-residue acetyltransferase component of pyruvate dehydrogenase complex"
"JS1_D03_00184" 12093 "s59E5H2" 0.2238669 "Dihydrolipoyllysine-residue acetyltransferase component of pyruvate dehydrogenase complex"
"JS1_D03_00184" 12093 "s63E6H2" 0.2177918 "Dihydrolipoyllysine-residue acetyltransferase component of pyruvate dehydrogenase complex"
"JS1_D03_00185" 12094 "s59E13H2" 0.2031734 "Rhamnulokinase"
"JS1_D03_00185" 12094 "s59E25H2" 0 "Rhamnulokinase"
"JS1_D03_00185" 12094 "s59E5H2" 0.4924598 "Rhamnulokinase"
"JS1_D03_00185" 12094 "s63E6H2" 0.6852824 "Rhamnulokinase"
"JS1_D03_00186" 12095 "s59E13H2" 0.04019377 "short chain dehydrogenase"
"JS1_D03_00186" 12095 "s59E25H2" 0 "short chain dehydrogenase"
"JS1_D03_00186" 12095 "s59E5H2" 0.09178398 "short chain dehydrogenase"
"JS1_D03_00186" 12095 "s63E6H2" 1.200103 "short chain dehydrogenase"
"JS1_D03_00187" 12096 "s59E13H2" 0.075789 "hypothetical protein"
"JS1_D03_00187" 12096 "s59E25H2" 0 "hypothetical protein"
"JS1_D03_00187" 12096 "s59E5H2" 0.1910471 "hypothetical protein"
"JS1_D03_00187" 12096 "s63E6H2" 1.229656 "hypothetical protein"
"JS1_D03_00188" 12097 "s59E13H2" 0.1960891 "Cell division protein FtsZ"
"JS1_D03_00188" 12097 "s59E25H2" 0 "Cell division protein FtsZ"
"JS1_D03_00188" 12097 "s59E5H2" 0.5308255 "Cell division protein FtsZ"
"JS1_D03_00188" 12097 "s63E6H2" 1.647618 "Cell division protein FtsZ"
"JS1_D03_00189" 12098 "s59E13H2" 0.08504164 "Sporulation initiation inhibitor protein Soj"
"JS1_D03_00189" 12098 "s59E25H2" 0.5646766 "Sporulation initiation inhibitor protein Soj"
"JS1_D03_00189" 12098 "s59E5H2" 0.5184125 "Sporulation initiation inhibitor protein Soj"
"JS1_D03_00189" 12098 "s63E6H2" 1.777776 "Sporulation initiation inhibitor protein Soj"
"JS1_D03_00190" 12099 "s59E13H2" 0.01583716 "Glutamine amidotransferase subunit PdxT"
"JS1_D03_00190" 12099 "s59E25H2" 0 "Glutamine amidotransferase subunit PdxT"
"JS1_D03_00190" 12099 "s59E5H2" 0.08085852 "Glutamine amidotransferase subunit PdxT"
"JS1_D03_00190" 12099 "s63E6H2" 0.08275125 "Glutamine amidotransferase subunit PdxT"
"JS1_D03_00191" 12100 "s59E13H2" 0.02900128 "Pyridoxal biosynthesis lyase PdxS"
"JS1_D03_00191" 12100 "s59E25H2" 0 "Pyridoxal biosynthesis lyase PdxS"
"JS1_D03_00191" 12100 "s59E5H2" 0.5134814 "Pyridoxal biosynthesis lyase PdxS"
"JS1_D03_00191" 12100 "s63E6H2" 0.4547034 "Pyridoxal biosynthesis lyase PdxS"
"JS1_D03_00192" 12101 "s59E13H2" 0.08420299 "Pyruvate-flavodoxin oxidoreductase"

"JS1_D03_00192" 12101 "s59E25H2" 0 "Pyruvate-flavodoxin oxidoreductase"
"JS1_D03_00192" 12101 "s59E5H2" 0.2939728 "Pyruvate-flavodoxin oxidoreductase"
"JS1_D03_00192" 12101 "s63E6H2" 0.4699026 "Pyruvate-flavodoxin oxidoreductase"
"JS1_D03_00193" 12102 "s59E13H2" 0 "hypothetical protein"
"JS1_D03_00193" 12102 "s59E25H2" 0 "hypothetical protein"
"JS1_D03_00193" 12102 "s59E5H2" 0 "hypothetical protein"
"JS1_D03_00193" 12102 "s63E6H2" 0 "hypothetical protein"
"JS1_D03_00194" 12103 "s59E13H2" 0.1055229 "Inositol 2-dehydrogenase/D-chiro-inositol 3-dehydrogenase"
"JS1_D03_00194" 12103 "s59E25H2" 0 "Inositol 2-dehydrogenase/D-chiro-inositol 3-dehydrogenase"
"JS1_D03_00194" 12103 "s59E5H2" 0.09195886 "Inositol 2-dehydrogenase/D-chiro-inositol 3-dehydrogenase"
"JS1_D03_00194" 12103 "s63E6H2" 0.05484062 "Inositol 2-dehydrogenase/D-chiro-inositol 3-dehydrogenase"
"JS1_D03_00195" 12104 "s59E13H2" 0.001412109 "Uronate isomerase"
"JS1_D03_00195" 12104 "s59E25H2" 0 "Uronate isomerase"
"JS1_D03_00195" 12104 "s59E5H2" 0.004592212 "Uronate isomerase"
"JS1_D03_00195" 12104 "s63E6H2" 0.3011041 "Uronate isomerase"
"JS1_D03_00196" 12105 "s59E13H2" 0.02459498 "hypothetical protein"
"JS1_D03_00196" 12105 "s59E25H2" 0 "hypothetical protein"
"JS1_D03_00196" 12105 "s59E5H2" 0.04164254 "hypothetical protein"
"JS1_D03_00196" 12105 "s63E6H2" 0.2545642 "hypothetical protein"
"JS1_D03_00197" 12106 "s59E13H2" 0.002700969 "hypothetical protein"
"JS1_D03_00197" 12106 "s59E25H2" 0 "hypothetical protein"
"JS1_D03_00197" 12106 "s59E5H2" 0 "hypothetical protein"
"JS1_D03_00197" 12106 "s63E6H2" 0.01957489 "hypothetical protein"
"JS1_D03_00198" 12107 "s59E13H2" 0 "Threonylcarbamoyladenosine tRNA methylthiotransferase MtaB"
"JS1_D03_00198" 12107 "s59E25H2" 0 "Threonylcarbamoyladenosine tRNA methylthiotransferase MtaB"
"JS1_D03_00198" 12107 "s59E5H2" 0 "Threonylcarbamoyladenosine tRNA methylthiotransferase MtaB"
"JS1_D03_00198" 12107 "s63E6H2" 0.1363278 "Threonylcarbamoyladenosine tRNA methylthiotransferase MtaB"
"JS1_D03_00199" 12108 "s59E13H2" 0.01586783 "Chaperone protein DnaJ"
"JS1_D03_00199" 12108 "s59E25H2" 0 "Chaperone protein DnaJ"
"JS1_D03_00199" 12108 "s59E5H2" 0.003574015 "Chaperone protein DnaJ"
"JS1_D03_00199" 12108 "s63E6H2" 0.1448897 "Chaperone protein DnaJ"
"JS1_D03_00200" 12109 "s59E13H2" 0.09664297 "Chaperone protein DnaK"
"JS1_D03_00200" 12109 "s59E25H2" 0 "Chaperone protein DnaK"
"JS1_D03_00200" 12109 "s59E5H2" 0.01420057 "Chaperone protein DnaK"
"JS1_D03_00200" 12109 "s63E6H2" 0.08852678 "Chaperone protein DnaK"
"JS1_D03_00201" 12110 "s59E13H2" 0.1818544 "Valine--tRNA ligase"
"JS1_D03_00201" 12110 "s59E25H2" 0 "Valine--tRNA ligase"
"JS1_D03_00201" 12110 "s59E5H2" 0.1195075 "Valine--tRNA ligase"
"JS1_D03_00201" 12110 "s63E6H2" 0.09591159 "Valine--tRNA ligase"
"JS1_D03_00203" 12112 "s59E13H2" 0.05401567 "Lon protease 1"
"JS1_D03_00203" 12112 "s59E25H2" 0 "Lon protease 1"
"JS1_D03_00203" 12112 "s59E5H2" 0.03478412 "Lon protease 1"
"JS1_D03_00203" 12112 "s63E6H2" 0.0413765 "Lon protease 1"
"JS1_D03_00204" 12113 "s59E13H2" 0 "Chromosome partition protein Smc"
"JS1_D03_00204" 12113 "s59E25H2" 0 "Chromosome partition protein Smc"
"JS1_D03_00204" 12113 "s59E5H2" 0 "Chromosome partition protein Smc"
"JS1_D03_00204" 12113 "s63E6H2" 0.05720459 "Chromosome partition protein Smc"

"JS1_D03_00205" 12114 "s59E13H2" 0 "Signal recognition particle receptor FtsY"
"JS1_D03_00205" 12114 "s59E25H2" 0 "Signal recognition particle receptor FtsY"
"JS1_D03_00205" 12114 "s59E5H2" 0 "Signal recognition particle receptor FtsY"
"JS1_D03_00205" 12114 "s63E6H2" 0.08770415 "Signal recognition particle receptor FtsY"
"JS1_D03_00206" 12115 "s59E13H2" 0.0007150118 "Signal recognition particle protein"
"JS1_D03_00206" 12115 "s59E25H2" 0 "Signal recognition particle protein"
"JS1_D03_00206" 12115 "s59E5H2" 0.007536066 "Signal recognition particle protein"
"JS1_D03_00206" 12115 "s63E6H2" 0.01961639 "Signal recognition particle protein"
"JS1_D03_00207" 12116 "s59E13H2" 0 "30S ribosomal protein S16"
"JS1_D03_00207" 12116 "s59E25H2" 0 "30S ribosomal protein S16"
"JS1_D03_00207" 12116 "s59E5H2" 0 "30S ribosomal protein S16"
"JS1_D03_00207" 12116 "s63E6H2" 0.01665091 "30S ribosomal protein S16"
"JS1_D03_00208" 12117 "s59E13H2" 0 "hypothetical protein"
"JS1_D03_00208" 12117 "s59E25H2" 0 "hypothetical protein"
"JS1_D03_00208" 12117 "s59E5H2" 0 "hypothetical protein"
"JS1_D03_00208" 12117 "s63E6H2" 0.02168762 "hypothetical protein"
"JS1_D03_00209" 12118 "s59E13H2" 0.1011698 "Ribosome maturation factor RimM"
"JS1_D03_00209" 12118 "s59E25H2" 0 "Ribosome maturation factor RimM"
"JS1_D03_00209" 12118 "s59E5H2" 0.001398659 "Ribosome maturation factor RimM"
"JS1_D03_00209" 12118 "s63E6H2" 0.03782272 "Ribosome maturation factor RimM"
"JS1_D03_00210" 12119 "s59E13H2" 0 "tRNA (guanine-N(1)-)-methyltransferase"
"JS1_D03_00210" 12119 "s59E25H2" 0 "tRNA (guanine-N(1)-)-methyltransferase"
"JS1_D03_00210" 12119 "s59E5H2" 0.003971246 "tRNA (guanine-N(1)-)-methyltransferase"
"JS1_D03_00210" 12119 "s63E6H2" 0.03863412 "tRNA (guanine-N(1)-)-methyltransferase"
"JS1_D03_00211" 12120 "s59E13H2" 0 "50S ribosomal protein L19"
"JS1_D03_00211" 12120 "s59E25H2" 0 "50S ribosomal protein L19"
"JS1_D03_00211" 12120 "s59E5H2" 0 "50S ribosomal protein L19"
"JS1_D03_00211" 12120 "s63E6H2" 0.01807928 "50S ribosomal protein L19"
"JS1_D03_00212" 12121 "s59E13H2" 0 "tRNA (guanine-N(1)-)-methyltransferase"
"JS1_D03_00212" 12121 "s59E25H2" 0 "tRNA (guanine-N(1)-)-methyltransferase"
"JS1_D03_00212" 12121 "s59E5H2" 0 "tRNA (guanine-N(1)-)-methyltransferase"
"JS1_D03_00212" 12121 "s63E6H2" 0 "tRNA (guanine-N(1)-)-methyltransferase"
"JS1_D03_00213" 12122 "s59E13H2" 0 "Putative ski2-type helicase"
"JS1_D03_00213" 12122 "s59E25H2" 0 "Putative ski2-type helicase"
"JS1_D03_00213" 12122 "s59E5H2" 0.1280842 "Putative ski2-type helicase"
"JS1_D03_00213" 12122 "s63E6H2" 0.04474608 "Putative ski2-type helicase"
"JS1_D03_00214" 12123 "s59E13H2" 0.0116302 "hypothetical protein"
"JS1_D03_00214" 12123 "s59E25H2" 0 "hypothetical protein"
"JS1_D03_00214" 12123 "s59E5H2" 0.7801103 "hypothetical protein"
"JS1_D03_00214" 12123 "s63E6H2" 0.2127177 "hypothetical protein"
"JS1_D03_00215" 12124 "s59E13H2" 0.002441239 "hypothetical protein"
"JS1_D03_00215" 12124 "s59E25H2" 0 "hypothetical protein"
"JS1_D03_00215" 12124 "s59E5H2" 0.2376667 "hypothetical protein"
"JS1_D03_00215" 12124 "s63E6H2" 1.052699 "hypothetical protein"
"JS1_D03_00216" 12125 "s59E13H2" 0.04229752 "RNA polymerase factor sigma-70"
"JS1_D03_00216" 12125 "s59E25H2" 0 "RNA polymerase factor sigma-70"
"JS1_D03_00216" 12125 "s59E5H2" 0.02575377 "RNA polymerase factor sigma-70"

"JS1_D03_00216" 12125 "s63E6H2" 5.886541 "RNA polymerase factor sigma-70"
"JS1_D03_00217" 12126 "s59E13H2" 0.1521233 "putative DNA-binding protein"
"JS1_D03_00217" 12126 "s59E25H2" 0 "putative DNA-binding protein"
"JS1_D03_00217" 12126 "s59E5H2" 0.08509143 "putative DNA-binding protein"
"JS1_D03_00217" 12126 "s63E6H2" 7.939552 "putative DNA-binding protein"
"JS1_D03_00218" 12127 "s59E13H2" 0.02278872 "hypothetical protein"
"JS1_D03_00218" 12127 "s59E25H2" 0 "hypothetical protein"
"JS1_D03_00218" 12127 "s59E5H2" 0.118886 "hypothetical protein"
"JS1_D03_00218" 12127 "s63E6H2" 1.266572 "hypothetical protein"
"JS1_D03_00219" 12128 "s59E13H2" 0.5213637 "hypothetical protein"
"JS1_D03_00219" 12128 "s59E25H2" 0.2197802 "hypothetical protein"
"JS1_D03_00219" 12128 "s59E5H2" 1.560127 "hypothetical protein"
"JS1_D03_00219" 12128 "s63E6H2" 11.87183 "hypothetical protein"
"JS1_D03_00220" 12129 "s59E13H2" 0.1816424 "hypothetical protein"
"JS1_D03_00220" 12129 "s59E25H2" 0 "hypothetical protein"
"JS1_D03_00220" 12129 "s59E5H2" 0.3700835 "hypothetical protein"
"JS1_D03_00220" 12129 "s63E6H2" 1.707473 "hypothetical protein"
"JS1_D03_00221" 12130 "s59E13H2" 0.2803414 "Peptidase M15"
"JS1_D03_00221" 12130 "s59E25H2" 0 "Peptidase M15"
"JS1_D03_00221" 12130 "s59E5H2" 0.03204051 "Peptidase M15"
"JS1_D03_00221" 12130 "s63E6H2" 0.6720248 "Peptidase M15"
"JS1_D03_00222" 12131 "s59E13H2" 0.07182236 "hypothetical protein"
"JS1_D03_00222" 12131 "s59E25H2" 0 "hypothetical protein"
"JS1_D03_00222" 12131 "s59E5H2" 0.05598209 "hypothetical protein"
"JS1_D03_00222" 12131 "s63E6H2" 0.04116613 "hypothetical protein"
"JS1_D03_00223" 12132 "s59E13H2" 6.44823 "D-alanyl-D-alanine carboxypeptidase precursor"
"JS1_D03_00223" 12132 "s59E25H2" 0 "D-alanyl-D-alanine carboxypeptidase precursor"
"JS1_D03_00223" 12132 "s59E5H2" 0.258998 "D-alanyl-D-alanine carboxypeptidase precursor"
"JS1_D03_00223" 12132 "s63E6H2" 0.3751867 "D-alanyl-D-alanine carboxypeptidase precursor"
"JS1_D03_00224" 12133 "s59E13H2" 5.789195 "3-chloro-4-hydroxyphenylacetate reductive dehalogenase precursor"
"JS1_D03_00224" 12133 "s59E25H2" 0 "3-chloro-4-hydroxyphenylacetate reductive dehalogenase precursor"
"JS1_D03_00224" 12133 "s59E5H2" 0.5939112 "3-chloro-4-hydroxyphenylacetate reductive dehalogenase precursor"
"JS1_D03_00224" 12133 "s63E6H2" 0.1938503 "3-chloro-4-hydroxyphenylacetate reductive dehalogenase precursor"
"JS1_D03_00225" 12134 "s59E13H2" 0.04396622 "Phosphoserine phosphatase 1"
"JS1_D03_00225" 12134 "s59E25H2" 0 "Phosphoserine phosphatase 1"
"JS1_D03_00225" 12134 "s59E5H2" 0 "Phosphoserine phosphatase 1"
"JS1_D03_00225" 12134 "s63E6H2" 0.03315464 "Phosphoserine phosphatase 1"
"JS1_D03_00226" 12135 "s59E13H2" 0.7585587 "putative hydrolase"
"JS1_D03_00226" 12135 "s59E25H2" 0 "putative hydrolase"
"JS1_D03_00226" 12135 "s59E5H2" 0.05964234 "putative hydrolase"
"JS1_D03_00226" 12135 "s63E6H2" 0.2098601 "putative hydrolase"
"JS1_D03_00227" 12136 "s59E13H2" 3.503718 "Ferredoxin-2"
"JS1_D03_00227" 12136 "s59E25H2" 0 "Ferredoxin-2"
"JS1_D03_00227" 12136 "s59E5H2" 0.120789 "Ferredoxin-2"
"JS1_D03_00227" 12136 "s63E6H2" 0.2460805 "Ferredoxin-2"
"JS1_D03_00228" 12137 "s59E13H2" 6.506014 "Glucose-6-phosphate 3-dehydrogenase"
"JS1_D03_00228" 12137 "s59E25H2" 0 "Glucose-6-phosphate 3-dehydrogenase"

"JS1_D03_00228" 12137 "s59E5H2" 0.2876144 "Glucose-6-phosphate 3-dehydrogenase"
"JS1_D03_00228" 12137 "s63E6H2" 0.5771904 "Glucose-6-phosphate 3-dehydrogenase"
"JS1_D03_00229" 12138 "s59E13H2" 4.977542 "Pyruvate formate-lyase 1-activating enzyme"
"JS1_D03_00229" 12138 "s59E25H2" 0 "Pyruvate formate-lyase 1-activating enzyme"
"JS1_D03_00229" 12138 "s59E5H2" 0.06238127 "Pyruvate formate-lyase 1-activating enzyme"
"JS1_D03_00229" 12138 "s63E6H2" 1.131649 "Pyruvate formate-lyase 1-activating enzyme"
"JS1_D03_00230" 12139 "s59E13H2" 0 "Helix-turn-helix domain protein"
"JS1_D03_00230" 12139 "s59E25H2" 0 "Helix-turn-helix domain protein"
"JS1_D03_00230" 12139 "s59E5H2" 0 "Helix-turn-helix domain protein"
"JS1_D03_00230" 12139 "s63E6H2" 0 "Helix-turn-helix domain protein"
"JS1_D03_00231" 12140 "s59E13H2" 0.03972676 "Cyclic di-GMP phosphodiesterase response regulator RpfG"
"JS1_D03_00231" 12140 "s59E25H2" 0 "Cyclic di-GMP phosphodiesterase response regulator RpfG"
"JS1_D03_00231" 12140 "s59E5H2" 0.7212405 "Cyclic di-GMP phosphodiesterase response regulator RpfG"
"JS1_D03_00231" 12140 "s63E6H2" 0.16959 "Cyclic di-GMP phosphodiesterase response regulator RpfG"
"JS1_D03_00232" 12141 "s59E13H2" 0.006036985 "Cyclic di-GMP phosphodiesterase response regulator RpfG"
"JS1_D03_00232" 12141 "s59E25H2" 0 "Cyclic di-GMP phosphodiesterase response regulator RpfG"
"JS1_D03_00232" 12141 "s59E5H2" 0.04048145 "Cyclic di-GMP phosphodiesterase response regulator RpfG"
"JS1_D03_00232" 12141 "s63E6H2" 0.1680269 "Cyclic di-GMP phosphodiesterase response regulator RpfG"
"JS1_D03_00233" 12142 "s59E13H2" 0.08627624 "putative diguanylate cyclase YegE"
"JS1_D03_00233" 12142 "s59E25H2" 0 "putative diguanylate cyclase YegE"
"JS1_D03_00233" 12142 "s59E5H2" 0.04992265 "putative diguanylate cyclase YegE"
"JS1_D03_00233" 12142 "s63E6H2" 0.03755565 "putative diguanylate cyclase YegE"
"JS1_D03_00234" 12143 "s59E13H2" 11.19907 "Benzylsuccinate synthase alpha subunit"
"JS1_D03_00234" 12143 "s59E25H2" 0 "Benzylsuccinate synthase alpha subunit"
"JS1_D03_00234" 12143 "s59E5H2" 0.1273318 "Benzylsuccinate synthase alpha subunit"
"JS1_D03_00234" 12143 "s63E6H2" 2.073346 "Benzylsuccinate synthase alpha subunit"
"JS1_D03_00235" 12144 "s59E13H2" 9.253275 "Xylulose kinase"
"JS1_D03_00235" 12144 "s59E25H2" 0 "Xylulose kinase"
"JS1_D03_00235" 12144 "s59E5H2" 0.08837489 "Xylulose kinase"
"JS1_D03_00235" 12144 "s63E6H2" 0.9930312 "Xylulose kinase"
"JS1_D03_00236" 12145 "s59E13H2" 0 "aminoimidazole riboside kinase"
"JS1_D03_00236" 12145 "s59E25H2" 0 "aminoimidazole riboside kinase"
"JS1_D03_00236" 12145 "s59E5H2" 0.03976724 "aminoimidazole riboside kinase"
"JS1_D03_00236" 12145 "s63E6H2" 0 "aminoimidazole riboside kinase"
"JS1_D03_00237" 12146 "s59E13H2" 0 "Trehalose/maltose transport system permease protein MalG"
"JS1_D03_00237" 12146 "s59E25H2" 0 "Trehalose/maltose transport system permease protein MalG"
"JS1_D03_00237" 12146 "s59E5H2" 0.01523603 "Trehalose/maltose transport system permease protein MalG"
"JS1_D03_00237" 12146 "s63E6H2" 0.0008091647 "Trehalose/maltose transport system permease protein MalG"
"JS1_D03_00238" 12147 "s59E13H2" 0 "Lactose transport system permease protein LacF"
"JS1_D03_00238" 12147 "s59E25H2" 0 "Lactose transport system permease protein LacF"
"JS1_D03_00238" 12147 "s59E5H2" 0.03248075 "Lactose transport system permease protein LacF"
"JS1_D03_00238" 12147 "s63E6H2" 0.4013334 "Lactose transport system permease protein LacF"
"JS1_D03_00239" 12148 "s59E13H2" 0 "Trehalose/maltose-binding protein MalE precursor"
"JS1_D03_00239" 12148 "s59E25H2" 0 "Trehalose/maltose-binding protein MalE precursor"
"JS1_D03_00239" 12148 "s59E5H2" 0.002149943 "Trehalose/maltose-binding protein MalE precursor"
"JS1_D03_00239" 12148 "s63E6H2" 0.7490818 "Trehalose/maltose-binding protein MalE precursor"
"JS1_D03_00240" 12149 "s59E13H2" 0 "hypothetical protein"

"JS1_D03_00240" 12149 "s59E25H2" 0 "hypothetical protein"
"JS1_D03_00240" 12149 "s59E5H2" 0 "hypothetical protein"
"JS1_D03_00240" 12149 "s63E6H2" 0.03585222 "hypothetical protein"
"JS1_D03_00241" 12150 "s59E13H2" 0.05312126 "ATP-dependent zinc metalloprotease FtsH"
"JS1_D03_00241" 12150 "s59E25H2" 0 "ATP-dependent zinc metalloprotease FtsH"
"JS1_D03_00241" 12150 "s59E5H2" 0.09874826 "ATP-dependent zinc metalloprotease FtsH"
"JS1_D03_00241" 12150 "s63E6H2" 0.1171355 "ATP-dependent zinc metalloprotease FtsH"
"JS1_D03_00242" 12151 "s59E13H2" 0 "hypothetical protein"
"JS1_D03_00242" 12151 "s59E25H2" 0 "hypothetical protein"
"JS1_D03_00242" 12151 "s59E5H2" 0 "hypothetical protein"
"JS1_D03_00242" 12151 "s63E6H2" 0.007852416 "hypothetical protein"
"JS1_D03_00243" 12152 "s59E13H2" 0.00203684 "Oligopeptide-binding protein OppA precursor"
"JS1_D03_00243" 12152 "s59E25H2" 0 "Oligopeptide-binding protein OppA precursor"
"JS1_D03_00243" 12152 "s59E5H2" 0.04709988 "Oligopeptide-binding protein OppA precursor"
"JS1_D03_00243" 12152 "s63E6H2" 0.03025251 "Oligopeptide-binding protein OppA precursor"
"JS1_D03_00244" 12153 "s59E13H2" 0.02073039 "Dipeptide transport system permease protein DppB"
"JS1_D03_00244" 12153 "s59E25H2" 0 "Dipeptide transport system permease protein DppB"
"JS1_D03_00244" 12153 "s59E5H2" 0.09787083 "Dipeptide transport system permease protein DppB"
"JS1_D03_00244" 12153 "s63E6H2" 0.01824449 "Dipeptide transport system permease protein DppB"
"JS1_D03_00245" 12154 "s59E13H2" 0.00809339 "Oligopeptide transport system permease protein OppC"
"JS1_D03_00245" 12154 "s59E25H2" 0 "Oligopeptide transport system permease protein OppC"
"JS1_D03_00245" 12154 "s59E5H2" 0.08232625 "Oligopeptide transport system permease protein OppC"
"JS1_D03_00245" 12154 "s63E6H2" 0.06200625 "Oligopeptide transport system permease protein OppC"
"JS1_D03_00247" 12156 "s59E13H2" 0 "Protein of unknown function (Hypoth_ymh)"
"JS1_D03_00247" 12156 "s59E25H2" 0 "Protein of unknown function (Hypoth_ymh)"
"JS1_D03_00247" 12156 "s59E5H2" 0 "Protein of unknown function (Hypoth_ymh)"
"JS1_D03_00247" 12156 "s63E6H2" 0 "Protein of unknown function (Hypoth_ymh)"
"JS1_D03_00248" 12157 "s59E13H2" 0.2478061 "Capsule biosynthesis protein CapA"
"JS1_D03_00248" 12157 "s59E25H2" 0 "Capsule biosynthesis protein CapA"
"JS1_D03_00248" 12157 "s59E5H2" 0.1639973 "Capsule biosynthesis protein CapA"
"JS1_D03_00248" 12157 "s63E6H2" 0.5963562 "Capsule biosynthesis protein CapA"
"JS1_D03_00249" 12158 "s59E13H2" 0.7226115 "hypothetical protein"
"JS1_D03_00249" 12158 "s59E25H2" 0 "hypothetical protein"
"JS1_D03_00249" 12158 "s59E5H2" 1.110895 "hypothetical protein"
"JS1_D03_00249" 12158 "s63E6H2" 0.8082949 "hypothetical protein"
"JS1_D03_00250" 12159 "s59E13H2" 4.364117 "Ktr system potassium uptake protein B"
"JS1_D03_00250" 12159 "s59E25H2" 0.05367232 "Ktr system potassium uptake protein B"
"JS1_D03_00250" 12159 "s59E5H2" 1.852351 "Ktr system potassium uptake protein B"
"JS1_D03_00250" 12159 "s63E6H2" 9.771319 "Ktr system potassium uptake protein B"
"JS1_D03_00251" 12160 "s59E13H2" 6.268941 "Trk system potassium uptake protein TrkH"
"JS1_D03_00251" 12160 "s59E25H2" 0.2383922 "Trk system potassium uptake protein TrkH"
"JS1_D03_00251" 12160 "s59E5H2" 0.7665577 "Trk system potassium uptake protein TrkH"
"JS1_D03_00251" 12160 "s63E6H2" 10.40643 "Trk system potassium uptake protein TrkH"
"JS1_D03_00252" 12161 "s59E13H2" 3.975499 "Trk system potassium uptake protein TrkA"
"JS1_D03_00252" 12161 "s59E25H2" 0 "Trk system potassium uptake protein TrkA"
"JS1_D03_00252" 12161 "s59E5H2" 0.4971246 "Trk system potassium uptake protein TrkA"
"JS1_D03_00252" 12161 "s63E6H2" 7.740698 "Trk system potassium uptake protein TrkA"

"JS1_D03_00254" 12163 "s59E13H2" 3.271658 "Zinc ribbon domain protein"
"JS1_D03_00254" 12163 "s59E25H2" 0 "Zinc ribbon domain protein"
"JS1_D03_00254" 12163 "s59E5H2" 0.06540838 "Zinc ribbon domain protein"
"JS1_D03_00254" 12163 "s63E6H2" 1.715018 "Zinc ribbon domain protein"
"JS1_D03_00255" 12164 "s59E13H2" 1.514554 "hypothetical protein"
"JS1_D03_00255" 12164 "s59E25H2" 0 "hypothetical protein"
"JS1_D03_00255" 12164 "s59E5H2" 0.09577125 "hypothetical protein"
"JS1_D03_00255" 12164 "s63E6H2" 4.028974 "hypothetical protein"
"JS1_D03_00256" 12165 "s59E13H2" 0.01844566 "hypothetical protein"
"JS1_D03_00256" 12165 "s59E25H2" 0 "hypothetical protein"
"JS1_D03_00256" 12165 "s59E5H2" 0.4978293 "hypothetical protein"
"JS1_D03_00256" 12165 "s63E6H2" 0.8128674 "hypothetical protein"
"JS1_D03_00257" 12166 "s59E13H2" 0 "L-lactate permease"
"JS1_D03_00257" 12166 "s59E25H2" 0 "L-lactate permease"
"JS1_D03_00257" 12166 "s59E5H2" 0.008816907 "L-lactate permease"
"JS1_D03_00257" 12166 "s63E6H2" 0.6639264 "L-lactate permease"
"JS1_D03_00258" 12167 "s59E13H2" 0 "hypothetical protein"
"JS1_D03_00258" 12167 "s59E25H2" 0 "hypothetical protein"
"JS1_D03_00258" 12167 "s59E5H2" 0 "hypothetical protein"
"JS1_D03_00258" 12167 "s63E6H2" 0 "hypothetical protein"
"JS1_D03_00259" 12168 "s59E13H2" 0 "hypothetical protein"
"JS1_D03_00259" 12168 "s59E25H2" 0 "hypothetical protein"
"JS1_D03_00259" 12168 "s59E5H2" 0 "hypothetical protein"
"JS1_D03_00259" 12168 "s63E6H2" 0 "hypothetical protein"
"JS1_D03_00260" 12169 "s59E13H2" 0 "Ion channel"
"JS1_D03_00260" 12169 "s59E25H2" 0 "Ion channel"
"JS1_D03_00260" 12169 "s59E5H2" 0 "Ion channel"
"JS1_D03_00260" 12169 "s63E6H2" 0.001044423 "Ion channel"
"JS1_D03_00261" 12170 "s59E13H2" 0 "Protein of unknown function (Hypoth_ymh)"
"JS1_D03_00261" 12170 "s59E25H2" 0 "Protein of unknown function (Hypoth_ymh)"
"JS1_D03_00261" 12170 "s59E5H2" 0 "Protein of unknown function (Hypoth_ymh)"
"JS1_D03_00261" 12170 "s63E6H2" 0 "Protein of unknown function (Hypoth_ymh)"
"JS1_D03_00262" 12171 "s59E13H2" 0.2815764 "Thioredoxin reductase"
"JS1_D03_00262" 12171 "s59E25H2" 0 "Thioredoxin reductase"
"JS1_D03_00262" 12171 "s59E5H2" 0.62802 "Thioredoxin reductase"
"JS1_D03_00262" 12171 "s63E6H2" 0.2601776 "Thioredoxin reductase"
"JS1_D03_00263" 12172 "s59E13H2" 0.2196701 "Inosose isomerase"
"JS1_D03_00263" 12172 "s59E25H2" 0 "Inosose isomerase"
"JS1_D03_00263" 12172 "s59E5H2" 0.4843978 "Inosose isomerase"
"JS1_D03_00263" 12172 "s63E6H2" 0.2878951 "Inosose isomerase"
"JS1_D03_00264" 12173 "s59E13H2" 0.04545775 "Lipoyl synthase"
"JS1_D03_00264" 12173 "s59E25H2" 0 "Lipoyl synthase"
"JS1_D03_00264" 12173 "s59E5H2" 0.2751287 "Lipoyl synthase"
"JS1_D03_00264" 12173 "s63E6H2" 0.3879166 "Lipoyl synthase"
"JS1_D03_00265" 12174 "s59E13H2" 0.07394789 "Elongation factor G"
"JS1_D03_00265" 12174 "s59E25H2" 0.2366667 "Elongation factor G"
"JS1_D03_00265" 12174 "s59E5H2" 0.7751138 "Elongation factor G"

"JS1_D03_00265" 12174 "s63E6H2" 0.7119572 "Elongation factor G"
"JS1_D03_00267" 12176 "s59E13H2" 0.05039439 "hypothetical protein"
"JS1_D03_00267" 12176 "s59E25H2" 0 "hypothetical protein"
"JS1_D03_00267" 12176 "s59E5H2" 0.4243988 "hypothetical protein"
"JS1_D03_00267" 12176 "s63E6H2" 1.089938 "hypothetical protein"
"JS1_D03_00270" 12179 "s59E13H2" 0.02704953 "Leucine--tRNA ligase"
"JS1_D03_00270" 12179 "s59E25H2" 0 "Leucine--tRNA ligase"
"JS1_D03_00270" 12179 "s59E5H2" 0.6976293 "Leucine--tRNA ligase"
"JS1_D03_00270" 12179 "s63E6H2" 0.6654101 "Leucine--tRNA ligase"
"JS1_D03_00271" 12180 "s59E13H2" 0.0687779 "Single-stranded DNA-binding protein ssb"
"JS1_D03_00271" 12180 "s59E25H2" 0 "Single-stranded DNA-binding protein ssb"
"JS1_D03_00271" 12180 "s59E5H2" 0.4328206 "Single-stranded DNA-binding protein ssb"
"JS1_D03_00271" 12180 "s63E6H2" 0.1780674 "Single-stranded DNA-binding protein ssb"
"JS1_D03_00279" 12188 "s59E13H2" 0.02692112 "Threonine--tRNA ligase 2"
"JS1_D03_00279" 12188 "s59E25H2" 0 "Threonine--tRNA ligase 2"
"JS1_D03_00279" 12188 "s59E5H2" 0.1130502 "Threonine--tRNA ligase 2"
"JS1_D03_00279" 12188 "s63E6H2" 0.1340048 "Threonine--tRNA ligase 2"
"JS1_D03_00280" 12189 "s59E13H2" 0.08687999 "Translation initiation factor IF-3"
"JS1_D03_00280" 12189 "s59E25H2" 0 "Translation initiation factor IF-3"
"JS1_D03_00280" 12189 "s59E5H2" 0.2932811 "Translation initiation factor IF-3"
"JS1_D03_00280" 12189 "s63E6H2" 0.2552977 "Translation initiation factor IF-3"
"JS1_D03_00281" 12190 "s59E13H2" 0.01443812 "50S ribosomal protein L35"
"JS1_D03_00281" 12190 "s59E25H2" 0 "50S ribosomal protein L35"
"JS1_D03_00281" 12190 "s59E5H2" 0.1505726 "50S ribosomal protein L35"
"JS1_D03_00281" 12190 "s63E6H2" 0.1546227 "50S ribosomal protein L35"
"JS1_D03_00282" 12191 "s59E13H2" 0.1058146 "50S ribosomal protein L20"
"JS1_D03_00282" 12191 "s59E25H2" 0 "50S ribosomal protein L20"
"JS1_D03_00282" 12191 "s59E5H2" 0.1603009 "50S ribosomal protein L20"
"JS1_D03_00282" 12191 "s63E6H2" 0.2176283 "50S ribosomal protein L20"
"JS1_D03_00284" 12193 "s59E13H2" 0.01007197 "Phenylalanine--tRNA ligase alpha subunit"
"JS1_D03_00284" 12193 "s59E25H2" 0 "Phenylalanine--tRNA ligase alpha subunit"
"JS1_D03_00284" 12193 "s59E5H2" 0.2273298 "Phenylalanine--tRNA ligase alpha subunit"
"JS1_D03_00284" 12193 "s63E6H2" 0.116621 "Phenylalanine--tRNA ligase alpha subunit"
"JS1_D03_00285" 12194 "s59E13H2" 0.2259909 "Phenylalanine--tRNA ligase beta subunit"
"JS1_D03_00285" 12194 "s59E25H2" 0 "Phenylalanine--tRNA ligase beta subunit"
"JS1_D03_00285" 12194 "s59E5H2" 0.1898641 "Phenylalanine--tRNA ligase beta subunit"
"JS1_D03_00285" 12194 "s63E6H2" 0.199108 "Phenylalanine--tRNA ligase beta subunit"
"JS1_D03_00286" 12195 "s59E13H2" 0.07756998 "colicin V production protein"
"JS1_D03_00286" 12195 "s59E25H2" 0 "colicin V production protein"
"JS1_D03_00286" 12195 "s59E5H2" 0 "colicin V production protein"
"JS1_D03_00286" 12195 "s63E6H2" 0.0376982 "colicin V production protein"
"JS1_D03_00287" 12196 "s59E13H2" 0.05964842 "Endonuclease MutS2"
"JS1_D03_00287" 12196 "s59E25H2" 0 "Endonuclease MutS2"
"JS1_D03_00287" 12196 "s59E5H2" 0.01046923 "Endonuclease MutS2"
"JS1_D03_00287" 12196 "s63E6H2" 0.05182125 "Endonuclease MutS2"
"JS1_D03_00288" 12197 "s59E13H2" 0.2610802 "Penicillin-binding protein 2D"
"JS1_D03_00288" 12197 "s59E25H2" 0 "Penicillin-binding protein 2D"

"JS1_D03_00288" 12197 "s59E5H2" 0.08647015 "Penicillin-binding protein 2D"
"JS1_D03_00288" 12197 "s63E6H2" 0.341219 "Penicillin-binding protein 2D"
"JS1_D03_00289" 12198 "s59E13H2" 0.08013794 "Queuosine precursor transporter QueT"
"JS1_D03_00289" 12198 "s59E25H2" 0 "Queuosine precursor transporter QueT"
"JS1_D03_00289" 12198 "s59E5H2" 0.07267567 "Queuosine precursor transporter QueT"
"JS1_D03_00289" 12198 "s63E6H2" 0.0780825 "Queuosine precursor transporter QueT"
"JS1_D03_00290" 12199 "s59E13H2" 0.3237284 "Tyrosine--tRNA ligase"
"JS1_D03_00290" 12199 "s59E25H2" 0 "Tyrosine--tRNA ligase"
"JS1_D03_00290" 12199 "s59E5H2" 0.2645128 "Tyrosine--tRNA ligase"
"JS1_D03_00290" 12199 "s63E6H2" 0.2829434 "Tyrosine--tRNA ligase"
"JS1_D03_00295" 12204 "s59E13H2" 0.2853717 "putative NH(3)-dependent NAD(+) synthetase"
"JS1_D03_00295" 12204 "s59E25H2" 0 "putative NH(3)-dependent NAD(+) synthetase"
"JS1_D03_00295" 12204 "s59E5H2" 0.1311738 "putative NH(3)-dependent NAD(+) synthetase"
"JS1_D03_00295" 12204 "s63E6H2" 1.11346 "putative NH(3)-dependent NAD(+) synthetase"
"JS1_D03_00296" 12205 "s59E13H2" 0.1016517 "putative transcriptional regulatory protein"
"JS1_D03_00296" 12205 "s59E25H2" 0 "putative transcriptional regulatory protein"
"JS1_D03_00296" 12205 "s59E5H2" 0.04467233 "putative transcriptional regulatory protein"
"JS1_D03_00296" 12205 "s63E6H2" 0.2401598 "putative transcriptional regulatory protein"
"JS1_D03_00297" 12206 "s59E13H2" 0.1849951 "Crossover junction endodeoxyribonuclease RuvC"
"JS1_D03_00297" 12206 "s59E25H2" 0 "Crossover junction endodeoxyribonuclease RuvC"
"JS1_D03_00297" 12206 "s59E5H2" 2.487382 "Crossover junction endodeoxyribonuclease RuvC"
"JS1_D03_00297" 12206 "s63E6H2" 0.08241245 "Crossover junction endodeoxyribonuclease RuvC"
"JS1_D03_00298" 12207 "s59E13H2" 0.3220993 "Holliday junction ATP-dependent DNA helicase RuvA"
"JS1_D03_00298" 12207 "s59E25H2" 0 "Holliday junction ATP-dependent DNA helicase RuvA"
"JS1_D03_00298" 12207 "s59E5H2" 0.08539276 "Holliday junction ATP-dependent DNA helicase RuvA"
"JS1_D03_00298" 12207 "s63E6H2" 0.06755109 "Holliday junction ATP-dependent DNA helicase RuvA"
"JS1_D03_00299" 12208 "s59E13H2" 0.2685677 "Holliday junction ATP-dependent DNA helicase RuvB"
"JS1_D03_00299" 12208 "s59E25H2" 0 "Holliday junction ATP-dependent DNA helicase RuvB"
"JS1_D03_00299" 12208 "s59E5H2" 0.1038187 "Holliday junction ATP-dependent DNA helicase RuvB"
"JS1_D03_00299" 12208 "s63E6H2" 0.07318988 "Holliday junction ATP-dependent DNA helicase RuvB"
"JS1_D03_00300" 12209 "s59E13H2" 0.1824093 "Amidase enhancer precursor"
"JS1_D03_00300" 12209 "s59E25H2" 0 "Amidase enhancer precursor"
"JS1_D03_00300" 12209 "s59E5H2" 0.1135863 "Amidase enhancer precursor"
"JS1_D03_00300" 12209 "s63E6H2" 0.01431953 "Amidase enhancer precursor"
"JS1_D03_00301" 12210 "s59E13H2" 0.6924972 "S-adenosylmethionine:tRNA ribosyltransferase-isomerase"
"JS1_D03_00301" 12210 "s59E25H2" 0 "S-adenosylmethionine:tRNA ribosyltransferase-isomerase"
"JS1_D03_00301" 12210 "s59E5H2" 0.151404 "S-adenosylmethionine:tRNA ribosyltransferase-isomerase"
"JS1_D03_00301" 12210 "s63E6H2" 0.05643282 "S-adenosylmethionine:tRNA ribosyltransferase-isomerase"
"JS1_D03_00302" 12211 "s59E13H2" 0.01658786 "Queuine tRNA-ribosyltransferase"
"JS1_D03_00302" 12211 "s59E25H2" 0 "Queuine tRNA-ribosyltransferase"
"JS1_D03_00302" 12211 "s59E5H2" 0.07629912 "Queuine tRNA-ribosyltransferase"
"JS1_D03_00302" 12211 "s63E6H2" 0.08241913 "Queuine tRNA-ribosyltransferase"
"JS1_D03_00303" 12212 "s59E13H2" 0.004530463 "preprotein translocase subunit YajC"
"JS1_D03_00303" 12212 "s59E25H2" 0 "preprotein translocase subunit YajC"
"JS1_D03_00303" 12212 "s59E5H2" 0.04441145 "preprotein translocase subunit YajC"
"JS1_D03_00303" 12212 "s63E6H2" 0.04539669 "preprotein translocase subunit YajC"
"JS1_D03_00304" 12213 "s59E13H2" 0.06220797 "preprotein translocase subunit SecD"

"JS1_D03_00304" 12213 "s59E25H2" 0 "preprotein translocase subunit SecD"
"JS1_D03_00304" 12213 "s59E5H2" 0.3431761 "preprotein translocase subunit SecD"
"JS1_D03_00304" 12213 "s63E6H2" 0.13628 "preprotein translocase subunit SecD"
"JS1_D03_00305" 12214 "s59E13H2" 0.003121099 "preprotein translocase subunit SecF"
"JS1_D03_00305" 12214 "s59E25H2" 0 "preprotein translocase subunit SecF"
"JS1_D03_00305" 12214 "s59E5H2" 0.02043787 "preprotein translocase subunit SecF"
"JS1_D03_00305" 12214 "s63E6H2" 0.01460069 "preprotein translocase subunit SecF"
"JS1_D03_00306" 12215 "s59E13H2" 0 "Cytochrome C biogenesis protein transmembrane region"
"JS1_D03_00306" 12215 "s59E25H2" 0 "Cytochrome C biogenesis protein transmembrane region"
"JS1_D03_00306" 12215 "s59E5H2" 0 "Cytochrome C biogenesis protein transmembrane region"
"JS1_D03_00306" 12215 "s63E6H2" 0.009376019 "Cytochrome C biogenesis protein transmembrane region"
"JS1_D03_00307" 12216 "s59E13H2" 0.1852814 "Thiol:disulfide interchange protein DsbD precursor"
"JS1_D03_00307" 12216 "s59E25H2" 0 "Thiol:disulfide interchange protein DsbD precursor"
"JS1_D03_00307" 12216 "s59E5H2" 0.004328643 "Thiol:disulfide interchange protein DsbD precursor"
"JS1_D03_00307" 12216 "s63E6H2" 0.04761076 "Thiol:disulfide interchange protein DsbD precursor"
"JS1_D03_00308" 12217 "s59E13H2" 0.1268732 "NAD synthetase"
"JS1_D03_00308" 12217 "s59E25H2" 0 "NAD synthetase"
"JS1_D03_00308" 12217 "s59E5H2" 0.03692394 "NAD synthetase"
"JS1_D03_00308" 12217 "s63E6H2" 0.07201668 "NAD synthetase"
"JS1_D03_00309" 12218 "s59E13H2" 0.01580932 "hypothetical protein"
"JS1_D03_00309" 12218 "s59E25H2" 0 "hypothetical protein"
"JS1_D03_00309" 12218 "s59E5H2" 0.04323129 "hypothetical protein"
"JS1_D03_00309" 12218 "s63E6H2" 0.0724059 "hypothetical protein"
"JS1_D03_00310" 12219 "s59E13H2" 0.0124495 "hypothetical protein"
"JS1_D03_00310" 12219 "s59E25H2" 0 "hypothetical protein"
"JS1_D03_00310" 12219 "s59E5H2" 0.02787126 "hypothetical protein"
"JS1_D03_00310" 12219 "s63E6H2" 0.1128964 "hypothetical protein"
"JS1_D03_00311" 12220 "s59E13H2" 0.01744874 "Putative serine protease HtrA"
"JS1_D03_00311" 12220 "s59E25H2" 0 "Putative serine protease HtrA"
"JS1_D03_00311" 12220 "s59E5H2" 0.002339838 "Putative serine protease HtrA"
"JS1_D03_00311" 12220 "s63E6H2" 0.1167934 "Putative serine protease HtrA"
"JS1_D03_00312" 12221 "s59E13H2" 0.4173645 "Sensor protein ZraS"
"JS1_D03_00312" 12221 "s59E25H2" 0 "Sensor protein ZraS"
"JS1_D03_00312" 12221 "s59E5H2" 0.4752064 "Sensor protein ZraS"
"JS1_D03_00312" 12221 "s63E6H2" 0.1830735 "Sensor protein ZraS"
"JS1_D03_00313" 12222 "s59E13H2" 0.6077639 "Transcriptional regulatory protein ZraR"
"JS1_D03_00313" 12222 "s59E25H2" 0 "Transcriptional regulatory protein ZraR"
"JS1_D03_00313" 12222 "s59E5H2" 0.7477036 "Transcriptional regulatory protein ZraR"
"JS1_D03_00313" 12222 "s63E6H2" 0.3820438 "Transcriptional regulatory protein ZraR"
"JS1_D03_00314" 12223 "s59E13H2" 2.424235 "Pantothenic acid transporter PanT"
"JS1_D03_00314" 12223 "s59E25H2" 0 "Pantothenic acid transporter PanT"
"JS1_D03_00314" 12223 "s59E5H2" 4.199361 "Pantothenic acid transporter PanT"
"JS1_D03_00314" 12223 "s63E6H2" 1.853608 "Pantothenic acid transporter PanT"
"JS1_D03_00315" 12224 "s59E13H2" 0.709325 "Type III pantothenate kinase"
"JS1_D03_00315" 12224 "s59E25H2" 0 "Type III pantothenate kinase"
"JS1_D03_00315" 12224 "s59E5H2" 4.555327 "Type III pantothenate kinase"
"JS1_D03_00315" 12224 "s63E6H2" 1.283978 "Type III pantothenate kinase"

"JS1_D03_00316" 12225 "s59E13H2" 0.5464729 "putative nicotinate-nucleotide pyrophosphorylase [carboxylating]"
"JS1_D03_00316" 12225 "s59E25H2" 0.4826389 "putative nicotinate-nucleotide pyrophosphorylase [carboxylating]"
"JS1_D03_00316" 12225 "s59E5H2" 0.2091576 "putative nicotinate-nucleotide pyrophosphorylase [carboxylating]"
"JS1_D03_00316" 12225 "s63E6H2" 0.1349757 "putative nicotinate-nucleotide pyrophosphorylase [carboxylating]"
"JS1_D03_00317" 12226 "s59E13H2" 0.9699013 "L-aspartate oxidase"
"JS1_D03_00317" 12226 "s59E25H2" 0 "L-aspartate oxidase"
"JS1_D03_00317" 12226 "s59E5H2" 0.5841516 "L-aspartate oxidase"
"JS1_D03_00317" 12226 "s63E6H2" 1.005189 "L-aspartate oxidase"
"JS1_D03_00318" 12227 "s59E13H2" 0.0004025745 "Quinolinate synthase A"
"JS1_D03_00318" 12227 "s59E25H2" 0 "Quinolinate synthase A"
"JS1_D03_00318" 12227 "s59E5H2" 0 "Quinolinate synthase A"
"JS1_D03_00318" 12227 "s63E6H2" 0.0418448 "Quinolinate synthase A"
"JS1_D03_00319" 12228 "s59E13H2" 4.292755 "hypothetical protein"
"JS1_D03_00319" 12228 "s59E25H2" 0 "hypothetical protein"
"JS1_D03_00319" 12228 "s59E5H2" 1.769633 "hypothetical protein"
"JS1_D03_00319" 12228 "s63E6H2" 1.179169 "hypothetical protein"
"JS1_D03_00320" 12229 "s59E13H2" 0.007339948 "hypothetical protein"
"JS1_D03_00320" 12229 "s59E25H2" 0 "hypothetical protein"
"JS1_D03_00320" 12229 "s59E5H2" 0.01430447 "hypothetical protein"
"JS1_D03_00320" 12229 "s63E6H2" 0.003348486 "hypothetical protein"
"JS1_D03_00321" 12230 "s59E13H2" 0.7370234 "Diaminopimelate epimerase"
"JS1_D03_00321" 12230 "s59E25H2" 0 "Diaminopimelate epimerase"
"JS1_D03_00321" 12230 "s59E5H2" 0.4335116 "Diaminopimelate epimerase"
"JS1_D03_00321" 12230 "s63E6H2" 0.2183906 "Diaminopimelate epimerase"
"JS1_D03_00322" 12231 "s59E13H2" 0.02787447 "4-hydroxy-tetrahydrodipicolinate reductase"
"JS1_D03_00322" 12231 "s59E25H2" 0 "4-hydroxy-tetrahydrodipicolinate reductase"
"JS1_D03_00322" 12231 "s59E5H2" 2.41741 "4-hydroxy-tetrahydrodipicolinate reductase"
"JS1_D03_00322" 12231 "s63E6H2" 0.6197232 "4-hydroxy-tetrahydrodipicolinate reductase"
"JS1_D03_00323" 12232 "s59E13H2" 0.9615458 "4-hydroxy-tetrahydrodipicolinate synthase"
"JS1_D03_00323" 12232 "s59E25H2" 0 "4-hydroxy-tetrahydrodipicolinate synthase"
"JS1_D03_00323" 12232 "s59E5H2" 2.075047 "4-hydroxy-tetrahydrodipicolinate synthase"
"JS1_D03_00323" 12232 "s63E6H2" 1.26895 "4-hydroxy-tetrahydrodipicolinate synthase"
"JS1_D03_00324" 12233 "s59E13H2" 0.9164637 "aspartate kinase"
"JS1_D03_00324" 12233 "s59E25H2" 0 "aspartate kinase"
"JS1_D03_00324" 12233 "s59E5H2" 0.3691672 "aspartate kinase"
"JS1_D03_00324" 12233 "s63E6H2" 0.6326839 "aspartate kinase"
"JS1_D03_00325" 12234 "s59E13H2" 2.200863 "Diaminopimelate decarboxylase"
"JS1_D03_00325" 12234 "s59E25H2" 0 "Diaminopimelate decarboxylase"
"JS1_D03_00325" 12234 "s59E5H2" 1.314701 "Diaminopimelate decarboxylase"
"JS1_D03_00325" 12234 "s63E6H2" 1.377295 "Diaminopimelate decarboxylase"
"JS1_D03_00326" 12235 "s59E13H2" 0.002724956 "preprotein translocase subunit SecA"
"JS1_D03_00326" 12235 "s59E25H2" 0 "preprotein translocase subunit SecA"
"JS1_D03_00326" 12235 "s59E5H2" 0.03697547 "preprotein translocase subunit SecA"
"JS1_D03_00326" 12235 "s63E6H2" 0.3672177 "preprotein translocase subunit SecA"
"JS1_D03_00327" 12236 "s59E13H2" 0 "Peptide chain release factor 2"
"JS1_D03_00327" 12236 "s59E25H2" 0 "Peptide chain release factor 2"
"JS1_D03_00327" 12236 "s59E5H2" 0.01557502 "Peptide chain release factor 2"

"JS1_D03_00327" 12236 "s63E6H2" 0.587983 "Peptide chain release factor 2"
"JS1_D03_00328" 12237 "s59E13H2" 0 "Acryloyl-CoA reductase electron transfer subunit gamma"
"JS1_D03_00328" 12237 "s59E25H2" 0 "Acryloyl-CoA reductase electron transfer subunit gamma"
"JS1_D03_00328" 12237 "s59E5H2" 0 "Acryloyl-CoA reductase electron transfer subunit gamma"
"JS1_D03_00328" 12237 "s63E6H2" 0.05636878 "Acryloyl-CoA reductase electron transfer subunit gamma"
"JS1_D03_00329" 12238 "s59E13H2" 0 "Acryloyl-CoA reductase electron transfer subunit beta"
"JS1_D03_00329" 12238 "s59E25H2" 0 "Acryloyl-CoA reductase electron transfer subunit beta"
"JS1_D03_00329" 12238 "s59E5H2" 0 "Acryloyl-CoA reductase electron transfer subunit beta"
"JS1_D03_00329" 12238 "s63E6H2" 0.02324822 "Acryloyl-CoA reductase electron transfer subunit beta"
"JS1_D03_00330" 12239 "s59E13H2" 0 "hypothetical protein"
"JS1_D03_00330" 12239 "s59E25H2" 0 "hypothetical protein"
"JS1_D03_00330" 12239 "s59E5H2" 0 "hypothetical protein"
"JS1_D03_00330" 12239 "s63E6H2" 0.01754177 "hypothetical protein"
"JS1_D03_00331" 12240 "s59E13H2" 0 "Methenyltetrahydrofolate cyclohydrolase"
"JS1_D03_00331" 12240 "s59E25H2" 0 "Methenyltetrahydrofolate cyclohydrolase"
"JS1_D03_00331" 12240 "s59E5H2" 0.006464008 "Methenyltetrahydrofolate cyclohydrolase"
"JS1_D03_00331" 12240 "s63E6H2" 0.0738338 "Methenyltetrahydrofolate cyclohydrolase"
"JS1_D03_00332" 12241 "s59E13H2" 0.007373688 "Glutamate 2,3-aminomutase"
"JS1_D03_00332" 12241 "s59E25H2" 0 "Glutamate 2,3-aminomutase"
"JS1_D03_00332" 12241 "s59E5H2" 0.01355407 "Glutamate 2,3-aminomutase"
"JS1_D03_00332" 12241 "s63E6H2" 0.04655141 "Glutamate 2,3-aminomutase"
"JS1_D03_00333" 12242 "s59E13H2" 0.005201477 "Adenosylhomocysteinase"
"JS1_D03_00333" 12242 "s59E25H2" 0 "Adenosylhomocysteinase"
"JS1_D03_00333" 12242 "s59E5H2" 0.09313041 "Adenosylhomocysteinase"
"JS1_D03_00333" 12242 "s63E6H2" 0.5192255 "Adenosylhomocysteinase"
"JS1_D03_00334" 12243 "s59E13H2" 0 "putative metallo-hydrolase"
"JS1_D03_00334" 12243 "s59E25H2" 0.6682692 "putative metallo-hydrolase"
"JS1_D03_00334" 12243 "s59E5H2" 0 "putative metallo-hydrolase"
"JS1_D03_00334" 12243 "s63E6H2" 0.2314027 "putative metallo-hydrolase"
"JS1_D03_00335" 12244 "s59E13H2" 0 "putative glycerophosphoryl diester phosphodiesterase 1"
"JS1_D03_00335" 12244 "s59E25H2" 0 "putative glycerophosphoryl diester phosphodiesterase 1"
"JS1_D03_00335" 12244 "s59E5H2" 0 "putative glycerophosphoryl diester phosphodiesterase 1"
"JS1_D03_00335" 12244 "s63E6H2" 0.1937448 "putative glycerophosphoryl diester phosphodiesterase 1"
"JS1_D03_00336" 12245 "s59E13H2" 0 "D-tyrosyl-tRNA(Tyr) deacylase"
"JS1_D03_00336" 12245 "s59E25H2" 0 "D-tyrosyl-tRNA(Tyr) deacylase"
"JS1_D03_00336" 12245 "s59E5H2" 0.03162018 "D-tyrosyl-tRNA(Tyr) deacylase"
"JS1_D03_00336" 12245 "s63E6H2" 0.4339161 "D-tyrosyl-tRNA(Tyr) deacylase"
"JS1_D03_00337" 12246 "s59E13H2" 0.028103 "GTP pyrophosphokinase"
"JS1_D03_00337" 12246 "s59E25H2" 0 "GTP pyrophosphokinase"
"JS1_D03_00337" 12246 "s59E5H2" 0.1240408 "GTP pyrophosphokinase"
"JS1_D03_00337" 12246 "s63E6H2" 0.4960344 "GTP pyrophosphokinase"
"JS1_D03_00338" 12247 "s59E13H2" 0.002251521 "Adenine phosphoribosyltransferase"
"JS1_D03_00338" 12247 "s59E25H2" 0 "Adenine phosphoribosyltransferase"
"JS1_D03_00338" 12247 "s59E5H2" 0.04585515 "Adenine phosphoribosyltransferase"
"JS1_D03_00338" 12247 "s63E6H2" 0.4802287 "Adenine phosphoribosyltransferase"
"JS1_D03_00339" 12248 "s59E13H2" 0.009971934 "Single-stranded-DNA-specific exonuclease RecJ"
"JS1_D03_00339" 12248 "s59E25H2" 0 "Single-stranded-DNA-specific exonuclease RecJ"

"JS1_D03_00339" 12248 "s59E5H2" 0.2599963 "Single-stranded-DNA-specific exonuclease RecJ"
"JS1_D03_00339" 12248 "s63E6H2" 0.5197773 "Single-stranded-DNA-specific exonuclease RecJ"
"JS1_D03_00340" 12249 "s59E13H2" 0 "Photosystem I assembly protein Ycf3"
"JS1_D03_00340" 12249 "s59E25H2" 0 "Photosystem I assembly protein Ycf3"
"JS1_D03_00340" 12249 "s59E5H2" 0.04291503 "Photosystem I assembly protein Ycf3"
"JS1_D03_00340" 12249 "s63E6H2" 0.02319824 "Photosystem I assembly protein Ycf3"
"JS1_D03_00341" 12250 "s59E13H2" 0 "hypothetical protein"
"JS1_D03_00341" 12250 "s59E25H2" 0 "hypothetical protein"
"JS1_D03_00341" 12250 "s59E5H2" 0.009633058 "hypothetical protein"
"JS1_D03_00341" 12250 "s63E6H2" 0.01656386 "hypothetical protein"
"JS1_D03_00342" 12251 "s59E13H2" 0 "hypothetical protein"
"JS1_D03_00342" 12251 "s59E25H2" 0 "hypothetical protein"
"JS1_D03_00342" 12251 "s59E5H2" 0 "hypothetical protein"
"JS1_D03_00342" 12251 "s63E6H2" 0.004004611 "hypothetical protein"
"JS1_D03_00343" 12252 "s59E13H2" 0.01010699 "Sialic acid TRAP transporter permease protein SiaT"
"JS1_D03_00343" 12252 "s59E25H2" 0 "Sialic acid TRAP transporter permease protein SiaT"
"JS1_D03_00343" 12252 "s59E5H2" 0.04297945 "Sialic acid TRAP transporter permease protein SiaT"
"JS1_D03_00343" 12252 "s63E6H2" 0.007775195 "Sialic acid TRAP transporter permease protein SiaT"
"JS1_D03_00344" 12253 "s59E13H2" 0.01415658 "Putative pyruvate, phosphate dikinase regulatory protein"
"JS1_D03_00344" 12253 "s59E25H2" 0 "Putative pyruvate, phosphate dikinase regulatory protein"
"JS1_D03_00344" 12253 "s59E5H2" 0.03879377 "Putative pyruvate, phosphate dikinase regulatory protein"
"JS1_D03_00344" 12253 "s63E6H2" 0.02334092 "Putative pyruvate, phosphate dikinase regulatory protein"
"JS1_D03_00345" 12254 "s59E13H2" 0.003684513 "Glycine--tRNA ligase beta subunit"
"JS1_D03_00345" 12254 "s59E25H2" 0 "Glycine--tRNA ligase beta subunit"
"JS1_D03_00345" 12254 "s59E5H2" 0.03805093 "Glycine--tRNA ligase beta subunit"
"JS1_D03_00345" 12254 "s63E6H2" 0.02236589 "Glycine--tRNA ligase beta subunit"
"JS1_D03_00346" 12255 "s59E13H2" 0.009901491 "Glycine--tRNA ligase alpha subunit"
"JS1_D03_00346" 12255 "s59E25H2" 0 "Glycine--tRNA ligase alpha subunit"
"JS1_D03_00346" 12255 "s59E5H2" 0.1809039 "Glycine--tRNA ligase alpha subunit"
"JS1_D03_00346" 12255 "s63E6H2" 0.1071266 "Glycine--tRNA ligase alpha subunit"
"JS1_D03_00347" 12256 "s59E13H2" 0 "Hypoxanthine-guanine phosphoribosyltransferase"
"JS1_D03_00347" 12256 "s59E25H2" 0 "Hypoxanthine-guanine phosphoribosyltransferase"
"JS1_D03_00347" 12256 "s59E5H2" 0 "Hypoxanthine-guanine phosphoribosyltransferase"
"JS1_D03_00347" 12256 "s63E6H2" 0.020571 "Hypoxanthine-guanine phosphoribosyltransferase"
"JS1_D03_00348" 12257 "s59E13H2" 0.005756514 "tRNA(Ile)-lysidine synthase"
"JS1_D03_00348" 12257 "s59E25H2" 0 "tRNA(Ile)-lysidine synthase"
"JS1_D03_00348" 12257 "s59E5H2" 0.06493427 "tRNA(Ile)-lysidine synthase"
"JS1_D03_00348" 12257 "s63E6H2" 0.1306085 "tRNA(Ile)-lysidine synthase"
"JS1_D03_00352" 12261 "s59E13H2" 0.01825843 "cell division protein FtsB"
"JS1_D03_00352" 12261 "s59E25H2" 0 "cell division protein FtsB"
"JS1_D03_00352" 12261 "s59E5H2" 0.2488614 "cell division protein FtsB"
"JS1_D03_00352" 12261 "s63E6H2" 0.320197 "cell division protein FtsB"
"JS1_D03_00353" 12262 "s59E13H2" 0.007232513 "Enolase"
"JS1_D03_00353" 12262 "s59E25H2" 0 "Enolase"
"JS1_D03_00353" 12262 "s59E5H2" 0.3100858 "Enolase"
"JS1_D03_00353" 12262 "s63E6H2" 0.4401719 "Enolase"
"JS1_D03_00354" 12263 "s59E13H2" 0.030207 "Magnesium transporter MgtE"

"JS1_D03_00354" 12263 "s59E25H2" 0 "Magnesium transporter MgtE"
"JS1_D03_00354" 12263 "s59E5H2" 0.3166384 "Magnesium transporter MgtE"
"JS1_D03_00354" 12263 "s63E6H2" 0.2531526 "Magnesium transporter MgtE"
"JS1_D03_00355" 12264 "s59E13H2" 0 "S4 domain protein"
"JS1_D03_00355" 12264 "s59E25H2" 0 "S4 domain protein"
"JS1_D03_00355" 12264 "s59E5H2" 0.01493665 "S4 domain protein"
"JS1_D03_00355" 12264 "s63E6H2" 0.0185794 "S4 domain protein"
"JS1_D03_00356" 12265 "s59E13H2" 0.03910526 "Lysine--tRNA ligase"
"JS1_D03_00356" 12265 "s59E25H2" 0 "Lysine--tRNA ligase"
"JS1_D03_00356" 12265 "s59E5H2" 0.1914888 "Lysine--tRNA ligase"
"JS1_D03_00356" 12265 "s63E6H2" 0.1734361 "Lysine--tRNA ligase"
"JS1_D03_00357" 12266 "s59E13H2" 0.03976721 "Transcription elongation factor GreA"
"JS1_D03_00357" 12266 "s59E25H2" 0 "Transcription elongation factor GreA"
"JS1_D03_00357" 12266 "s59E5H2" 0.1328062 "Transcription elongation factor GreA"
"JS1_D03_00357" 12266 "s63E6H2" 0.1325388 "Transcription elongation factor GreA"
"JS1_D03_00358" 12267 "s59E13H2" 0.004274974 "Putative pyridoxal phosphate-dependent acyltransferase"
"JS1_D03_00358" 12267 "s59E25H2" 0 "Putative pyridoxal phosphate-dependent acyltransferase"
"JS1_D03_00358" 12267 "s59E5H2" 0.04841741 "Putative pyridoxal phosphate-dependent acyltransferase"
"JS1_D03_00358" 12267 "s63E6H2" 0.1671779 "Putative pyridoxal phosphate-dependent acyltransferase"
"JS1_D03_00359" 12268 "s59E13H2" 0.02633025 "hypothetical protein"
"JS1_D03_00359" 12268 "s59E25H2" 0 "hypothetical protein"
"JS1_D03_00359" 12268 "s59E5H2" 0.1018896 "hypothetical protein"
"JS1_D03_00359" 12268 "s63E6H2" 0.101997 "hypothetical protein"
"JS1_D03_00360" 12269 "s59E13H2" 0 "hypothetical protein"
"JS1_D03_00360" 12269 "s59E25H2" 0 "hypothetical protein"
"JS1_D03_00360" 12269 "s59E5H2" 0.1168789 "hypothetical protein"
"JS1_D03_00360" 12269 "s63E6H2" 0.05997074 "hypothetical protein"
"JS1_D03_00361" 12270 "s59E13H2" 0.01945063 "tRNA-specific 2-thiouridylase MnmA"
"JS1_D03_00361" 12270 "s59E25H2" 0 "tRNA-specific 2-thiouridylase MnmA"
"JS1_D03_00361" 12270 "s59E5H2" 0.07837206 "tRNA-specific 2-thiouridylase MnmA"
"JS1_D03_00361" 12270 "s63E6H2" 0.08327936 "tRNA-specific 2-thiouridylase MnmA"
"JS1_D03_00362" 12271 "s59E13H2" 0.0194229 "hypothetical protein"
"JS1_D03_00362" 12271 "s59E25H2" 0 "hypothetical protein"
"JS1_D03_00362" 12271 "s59E5H2" 0.06373055 "hypothetical protein"
"JS1_D03_00362" 12271 "s63E6H2" 0.04911969 "hypothetical protein"
"JS1_D03_00363" 12272 "s59E13H2" 0 "HTH-type transcriptional regulator CymR"
"JS1_D03_00363" 12272 "s59E25H2" 0 "HTH-type transcriptional regulator CymR"
"JS1_D03_00363" 12272 "s59E5H2" 0 "HTH-type transcriptional regulator CymR"
"JS1_D03_00363" 12272 "s63E6H2" 0.05360946 "HTH-type transcriptional regulator CymR"
"JS1_D03_00364" 12273 "s59E13H2" 0 "Ferredoxin"
"JS1_D03_00364" 12273 "s59E25H2" 0 "Ferredoxin"
"JS1_D03_00364" 12273 "s59E5H2" 0.01621173 "Ferredoxin"
"JS1_D03_00364" 12273 "s63E6H2" 0.01013215 "Ferredoxin"
"JS1_D03_00365" 12274 "s59E13H2" 0.001394053 "Ribonuclease Z"
"JS1_D03_00365" 12274 "s59E25H2" 0 "Ribonuclease Z"
"JS1_D03_00365" 12274 "s59E5H2" 0.09022479 "Ribonuclease Z"
"JS1_D03_00365" 12274 "s63E6H2" 0.03098001 "Ribonuclease Z"

"JS1_D03_00366" 12275 "s59E13H2" 0 "Molybdopterin adenylyltransferase"
"JS1_D03_00366" 12275 "s59E25H2" 0 "Molybdopterin adenylyltransferase"
"JS1_D03_00366" 12275 "s59E5H2" 0 "Molybdopterin adenylyltransferase"
"JS1_D03_00366" 12275 "s63E6H2" 0.01030002 "Molybdopterin adenylyltransferase"
"JS1_D03_00367" 12276 "s59E13H2" 0 "MOSC domain protein"
"JS1_D03_00367" 12276 "s59E25H2" 0 "MOSC domain protein"
"JS1_D03_00367" 12276 "s59E5H2" 0 "MOSC domain protein"
"JS1_D03_00367" 12276 "s63E6H2" 0.01651731 "MOSC domain protein"
"JS1_D03_00368" 12277 "s59E13H2" 0 "Cyclic pyranopterin monophosphate synthase accessory protein"
"JS1_D03_00368" 12277 "s59E25H2" 0 "Cyclic pyranopterin monophosphate synthase accessory protein"
"JS1_D03_00368" 12277 "s59E5H2" 0.007617561 "Cyclic pyranopterin monophosphate synthase accessory protein"
"JS1_D03_00368" 12277 "s63E6H2" 0.01198768 "Cyclic pyranopterin monophosphate synthase accessory protein"
"JS1_D03_00369" 12278 "s59E13H2" 0 "Cyclic pyranopterin monophosphate synthase"
"JS1_D03_00369" 12278 "s59E25H2" 0 "Cyclic pyranopterin monophosphate synthase"
"JS1_D03_00369" 12278 "s59E5H2" 0 "Cyclic pyranopterin monophosphate synthase"
"JS1_D03_00369" 12278 "s63E6H2" 0.04102924 "Cyclic pyranopterin monophosphate synthase"
"JS1_D03_00370" 12279 "s59E13H2" 0.02355655 "Molybdopterin molybdenumtransferase"
"JS1_D03_00370" 12279 "s59E25H2" 0 "Molybdopterin molybdenumtransferase"
"JS1_D03_00370" 12279 "s59E5H2" 0.02630121 "Molybdopterin molybdenumtransferase"
"JS1_D03_00370" 12279 "s63E6H2" 0.04874758 "Molybdopterin molybdenumtransferase"
"JS1_D03_00371" 12280 "s59E13H2" 0.0009968031 "Molybdopterin molybdenumtransferase"
"JS1_D03_00371" 12280 "s59E25H2" 0 "Molybdopterin molybdenumtransferase"
"JS1_D03_00371" 12280 "s59E5H2" 0.008689899 "Molybdopterin molybdenumtransferase"
"JS1_D03_00371" 12280 "s63E6H2" 0.01781429 "Molybdopterin molybdenumtransferase"
"JS1_D03_00372" 12281 "s59E13H2" 0.001697983 "Sulfate/thiosulfate import ATP-binding protein CysA"
"JS1_D03_00372" 12281 "s59E25H2" 0 "Sulfate/thiosulfate import ATP-binding protein CysA"
"JS1_D03_00372" 12281 "s59E5H2" 0.07249187 "Sulfate/thiosulfate import ATP-binding protein CysA"
"JS1_D03_00372" 12281 "s63E6H2" 0.08004398 "Sulfate/thiosulfate import ATP-binding protein CysA"
"JS1_D03_00373" 12282 "s59E13H2" 0.004627788 "Molybdenum transport system permease protein ModB"
"JS1_D03_00373" 12282 "s59E25H2" 0 "Molybdenum transport system permease protein ModB"
"JS1_D03_00373" 12282 "s59E5H2" 0.09569245 "Molybdenum transport system permease protein ModB"
"JS1_D03_00373" 12282 "s63E6H2" 0.08769578 "Molybdenum transport system permease protein ModB"
"JS1_D03_00374" 12283 "s59E13H2" 0.4981274 "PBP superfamily domain protein"
"JS1_D03_00374" 12283 "s59E25H2" 0 "PBP superfamily domain protein"
"JS1_D03_00374" 12283 "s59E5H2" 0.0145094 "PBP superfamily domain protein"
"JS1_D03_00374" 12283 "s63E6H2" 0.07473295 "PBP superfamily domain protein"
"JS1_D03_00376" 12285 "s59E13H2" 0.02159029 "Formamidopyrimidine-DNA glycosylase"
"JS1_D03_00376" 12285 "s59E25H2" 0 "Formamidopyrimidine-DNA glycosylase"
"JS1_D03_00376" 12285 "s59E5H2" 0.2392093 "Formamidopyrimidine-DNA glycosylase"
"JS1_D03_00376" 12285 "s63E6H2" 0.114443 "Formamidopyrimidine-DNA glycosylase"
"JS1_D03_00377" 12286 "s59E13H2" 0.9697993 "GMP/IMP nucleotidase YrfG"
"JS1_D03_00377" 12286 "s59E25H2" 0 "GMP/IMP nucleotidase YrfG"
"JS1_D03_00377" 12286 "s59E5H2" 0.3790352 "GMP/IMP nucleotidase YrfG"
"JS1_D03_00377" 12286 "s63E6H2" 0.3800938 "GMP/IMP nucleotidase YrfG"
"JS1_D03_00378" 12287 "s59E13H2" 0 "Serine acetyltransferase"
"JS1_D03_00378" 12287 "s59E25H2" 0 "Serine acetyltransferase"
"JS1_D03_00378" 12287 "s59E5H2" 0 "Serine acetyltransferase"

"JS1_D03_00378" 12287 "s63E6H2" 0.6361118 "Serine acetyltransferase"
"JS1_D03_00379" 12288 "s59E13H2" 0 "Ferredoxin"
"JS1_D03_00379" 12288 "s59E25H2" 0 "Ferredoxin"
"JS1_D03_00379" 12288 "s59E5H2" 0 "Ferredoxin"
"JS1_D03_00379" 12288 "s63E6H2" 1.049393 "Ferredoxin"
"JS1_D03_00380" 12289 "s59E13H2" 0.001441872 "hypothetical protein"
"JS1_D03_00380" 12289 "s59E25H2" 0 "hypothetical protein"
"JS1_D03_00380" 12289 "s59E5H2" 0.006748167 "hypothetical protein"
"JS1_D03_00380" 12289 "s63E6H2" 1.223888 "hypothetical protein"
"JS1_D03_00382" 12291 "s59E13H2" 0 "O-acetylserine sulfhydrylase"
"JS1_D03_00382" 12291 "s59E25H2" 0 "O-acetylserine sulfhydrylase"
"JS1_D03_00382" 12291 "s59E5H2" 0 "O-acetylserine sulfhydrylase"
"JS1_D03_00382" 12291 "s63E6H2" 0.244869 "O-acetylserine sulfhydrylase"
"JS1_D03_00383" 12292 "s59E13H2" 0 "HTH-type transcriptional regulator CymR"
"JS1_D03_00383" 12292 "s59E25H2" 0 "HTH-type transcriptional regulator CymR"
"JS1_D03_00383" 12292 "s59E5H2" 0 "HTH-type transcriptional regulator CymR"
"JS1_D03_00383" 12292 "s63E6H2" 0.07682753 "HTH-type transcriptional regulator CymR"
"JS1_D03_00384" 12293 "s59E13H2" 0 "hypothetical protein"
"JS1_D03_00384" 12293 "s59E25H2" 0 "hypothetical protein"
"JS1_D03_00384" 12293 "s59E5H2" 0 "hypothetical protein"
"JS1_D03_00384" 12293 "s63E6H2" 0.02463712 "hypothetical protein"
"JS1_D03_00385" 12294 "s59E13H2" 0 "formate dehydrogenase accessory protein"
"JS1_D03_00385" 12294 "s59E25H2" 0 "formate dehydrogenase accessory protein"
"JS1_D03_00385" 12294 "s59E5H2" 0 "formate dehydrogenase accessory protein"
"JS1_D03_00385" 12294 "s63E6H2" 0.04818312 "formate dehydrogenase accessory protein"
"JS1_D03_00386" 12295 "s59E13H2" 0 "putative cysteine desulfurase"
"JS1_D03_00386" 12295 "s59E25H2" 0 "putative cysteine desulfurase"
"JS1_D03_00386" 12295 "s59E5H2" 0 "putative cysteine desulfurase"
"JS1_D03_00386" 12295 "s63E6H2" 0.04507219 "putative cysteine desulfurase"
"JS1_D03_00390" 12299 "s59E13H2" 0.1552221 "UDP-N-acetylmuramate--L-alanine ligase"
"JS1_D03_00390" 12299 "s59E25H2" 0 "UDP-N-acetylmuramate--L-alanine ligase"
"JS1_D03_00390" 12299 "s59E5H2" 0.3678671 "UDP-N-acetylmuramate--L-alanine ligase"
"JS1_D03_00390" 12299 "s63E6H2" 0.2498985 "UDP-N-acetylmuramate--L-alanine ligase"
"JS1_D03_00391" 12300 "s59E13H2" 0.04487297 "UDP-N-acetylglucosamine--N-acetylmuramyl-(pentapeptide) pyrophosphoryl-undecaprenol N-acetylglucosamine transferase"
"JS1_D03_00391" 12300 "s59E25H2" 0 "UDP-N-acetylglucosamine--N-acetylmuramyl-(pentapeptide) pyrophosphoryl-undecaprenol N-acetylglucosamine transferase"
"JS1_D03_00391" 12300 "s59E5H2" 0.2729267 "UDP-N-acetylglucosamine--N-acetylmuramyl-(pentapeptide) pyrophosphoryl-undecaprenol N-acetylglucosamine transferase"
"JS1_D03_00391" 12300 "s63E6H2" 0.3022886 "UDP-N-acetylglucosamine--N-acetylmuramyl-(pentapeptide) pyrophosphoryl-undecaprenol N-acetylglucosamine transferase"
"JS1_D03_00392" 12301 "s59E13H2" 0.3879907 "Lipid II flippase FtsW"
"JS1_D03_00392" 12301 "s59E25H2" 0 "Lipid II flippase FtsW"
"JS1_D03_00392" 12301 "s59E5H2" 0.6149562 "Lipid II flippase FtsW"
"JS1_D03_00392" 12301 "s63E6H2" 0.8732753 "Lipid II flippase FtsW"
"JS1_D03_00393" 12302 "s59E13H2" 0.006151055 "Phospho-N-acetylmuramoyl-pentapeptide-transferase"
"JS1_D03_00393" 12302 "s59E25H2" 0 "Phospho-N-acetylmuramoyl-pentapeptide-transferase"
"JS1_D03_00393" 12302 "s59E5H2" 0.06380706 "Phospho-N-acetylmuramoyl-pentapeptide-transferase"
"JS1_D03_00393" 12302 "s63E6H2" 0.06742833 "Phospho-N-acetylmuramoyl-pentapeptide-transferase"
"JS1_D03_00394" 12303 "s59E13H2" 0.09302741 "UDP-N-acetylmuramoyl-tripeptide--D-alanyl-D-alanine ligase"
"JS1_D03_00394" 12303 "s59E25H2" 0 "UDP-N-acetylmuramoyl-tripeptide--D-alanyl-D-alanine ligase"

"JS1_D03_00394" 12303 "s59E5H2" 0.4231556 "UDP-N-acetylmuramoyl-tripeptide--D-alanyl-D-alanine ligase"
"JS1_D03_00394" 12303 "s63E6H2" 0.2782398 "UDP-N-acetylmuramoyl-tripeptide--D-alanyl-D-alanine ligase"
"JS1_D03_00395" 12304 "s59E13H2" 0.012929 "UDP-N-acetylmuramoyl-L-alanyl-D-glutamate--L-lysine ligase"
"JS1_D03_00395" 12304 "s59E25H2" 0 "UDP-N-acetylmuramoyl-L-alanyl-D-glutamate--L-lysine ligase"
"JS1_D03_00395" 12304 "s59E5H2" 0.2206028 "UDP-N-acetylmuramoyl-L-alanyl-D-glutamate--L-lysine ligase"
"JS1_D03_00395" 12304 "s63E6H2" 0.2721937 "UDP-N-acetylmuramoyl-L-alanyl-D-glutamate--L-lysine ligase"
"JS1_D03_00396" 12305 "s59E13H2" 0 "photosystem I assembly protein Ycf3"
"JS1_D03_00396" 12305 "s59E25H2" 0 "photosystem I assembly protein Ycf3"
"JS1_D03_00396" 12305 "s59E5H2" 0.003191486 "photosystem I assembly protein Ycf3"
"JS1_D03_00396" 12305 "s63E6H2" 0.01633064 "photosystem I assembly protein Ycf3"
"JS1_D03_00397" 12306 "s59E13H2" 0.01913842 "hypothetical protein"
"JS1_D03_00397" 12306 "s59E25H2" 0 "hypothetical protein"
"JS1_D03_00397" 12306 "s59E5H2" 0.01225705 "hypothetical protein"
"JS1_D03_00397" 12306 "s63E6H2" 0.1993178 "hypothetical protein"
"JS1_D03_00398" 12307 "s59E13H2" 0 "Cystathionine beta-lyase PatB"
"JS1_D03_00398" 12307 "s59E25H2" 0 "Cystathionine beta-lyase PatB"
"JS1_D03_00398" 12307 "s59E5H2" 0 "Cystathionine beta-lyase PatB"
"JS1_D03_00398" 12307 "s63E6H2" 0.1300421 "Cystathionine beta-lyase PatB"
"JS1_D03_00399" 12308 "s59E13H2" 1.478817 "Radical SAM superfamily protein"
"JS1_D03_00399" 12308 "s59E25H2" 0 "Radical SAM superfamily protein"
"JS1_D03_00399" 12308 "s59E5H2" 0.0871923 "Radical SAM superfamily protein"
"JS1_D03_00399" 12308 "s63E6H2" 0.581504 "Radical SAM superfamily protein"
"JS1_D03_00400" 12309 "s59E13H2" 0.4342592 "Cyclic di-GMP phosphodiesterase response regulator RpfG"
"JS1_D03_00400" 12309 "s59E25H2" 0 "Cyclic di-GMP phosphodiesterase response regulator RpfG"
"JS1_D03_00400" 12309 "s59E5H2" 0.9590705 "Cyclic di-GMP phosphodiesterase response regulator RpfG"
"JS1_D03_00400" 12309 "s63E6H2" 0.03752106 "Cyclic di-GMP phosphodiesterase response regulator RpfG"
"JS1_D03_00402" 12311 "s59E13H2" 0.02308559 "dihydrolipoamide dehydrogenase"
"JS1_D03_00402" 12311 "s59E25H2" 0 "dihydrolipoamide dehydrogenase"
"JS1_D03_00402" 12311 "s59E5H2" 0.2661513 "dihydrolipoamide dehydrogenase"
"JS1_D03_00402" 12311 "s63E6H2" 0.2897446 "dihydrolipoamide dehydrogenase"
"JS1_D03_00403" 12312 "s59E13H2" 0.02636088 "5'-nucleotidase SurE"
"JS1_D03_00403" 12312 "s59E25H2" 0 "5'-nucleotidase SurE"
"JS1_D03_00403" 12312 "s59E5H2" 0.4087207 "5'-nucleotidase SurE"
"JS1_D03_00403" 12312 "s63E6H2" 0.3260108 "5'-nucleotidase SurE"
"JS1_D03_00404" 12313 "s59E13H2" 0.03609824 "hypothetical protein"
"JS1_D03_00404" 12313 "s59E25H2" 0 "hypothetical protein"
"JS1_D03_00404" 12313 "s59E5H2" 0.4743433 "hypothetical protein"
"JS1_D03_00404" 12313 "s63E6H2" 0.249082 "hypothetical protein"
"JS1_D03_00405" 12314 "s59E13H2" 0.02114979 "Protein-L-isoaspartate O-methyltransferase"
"JS1_D03_00405" 12314 "s59E25H2" 0 "Protein-L-isoaspartate O-methyltransferase"
"JS1_D03_00405" 12314 "s59E5H2" 0.211336 "Protein-L-isoaspartate O-methyltransferase"
"JS1_D03_00405" 12314 "s63E6H2" 0.292253 "Protein-L-isoaspartate O-methyltransferase"
"JS1_D03_00407" 12316 "s59E13H2" 0 "hypothetical protein"
"JS1_D03_00407" 12316 "s59E25H2" 0 "hypothetical protein"
"JS1_D03_00407" 12316 "s59E5H2" 0 "hypothetical protein"
"JS1_D03_00407" 12316 "s63E6H2" 0.1880739 "hypothetical protein"
"JS1_D03_00408" 12317 "s59E13H2" 0.03433102 "Vancomycin B-type resistance protein VanW"

"JS1_D03_00408" 12317 "s59E25H2" 0.6144578 "Vancomycin B-type resistance protein VanW"
"JS1_D03_00408" 12317 "s59E5H2" 0.1381144 "Vancomycin B-type resistance protein VanW"
"JS1_D03_00408" 12317 "s63E6H2" 1.431571 "Vancomycin B-type resistance protein VanW"
"JS1_D03_00409" 12318 "s59E13H2" 0 "Succinylglutamate desuccinylase / Aspartoacylase family protein"
"JS1_D03_00409" 12318 "s59E25H2" 0 "Succinylglutamate desuccinylase / Aspartoacylase family protein"
"JS1_D03_00409" 12318 "s59E5H2" 0 "Succinylglutamate desuccinylase / Aspartoacylase family protein"
"JS1_D03_00409" 12318 "s63E6H2" 1.04628 "Succinylglutamate desuccinylase / Aspartoacylase family protein"
"JS1_D03_00410" 12319 "s59E13H2" 0.1155141 "Putative peptidyl-prolyl cis-trans isomerase Cbf2 precursor"
"JS1_D03_00410" 12319 "s59E25H2" 0 "Putative peptidyl-prolyl cis-trans isomerase Cbf2 precursor"
"JS1_D03_00410" 12319 "s59E5H2" 0.01102631 "Putative peptidyl-prolyl cis-trans isomerase Cbf2 precursor"
"JS1_D03_00410" 12319 "s63E6H2" 0.8227797 "Putative peptidyl-prolyl cis-trans isomerase Cbf2 precursor"
"JS1_D03_00411" 12320 "s59E13H2" 0.006955593 "30S ribosomal protein S21"
"JS1_D03_00411" 12320 "s59E25H2" 0 "30S ribosomal protein S21"
"JS1_D03_00411" 12320 "s59E5H2" 0.2849491 "30S ribosomal protein S21"
"JS1_D03_00411" 12320 "s63E6H2" 0.5078298 "30S ribosomal protein S21"
"JS1_D03_00412" 12321 "s59E13H2" 0.000787706 "glutamyl-tRNA(Gln) amidotransferase subunit E"
"JS1_D03_00412" 12321 "s59E25H2" 0 "glutamyl-tRNA(Gln) amidotransferase subunit E"
"JS1_D03_00412" 12321 "s59E5H2" 0.08843708 "glutamyl-tRNA(Gln) amidotransferase subunit E"
"JS1_D03_00412" 12321 "s63E6H2" 0.7185383 "glutamyl-tRNA(Gln) amidotransferase subunit E"
"JS1_D03_00413" 12322 "s59E13H2" 0.04552276 "hypothetical protein"
"JS1_D03_00413" 12322 "s59E25H2" 0 "hypothetical protein"
"JS1_D03_00413" 12322 "s59E5H2" 0.2198892 "hypothetical protein"
"JS1_D03_00413" 12322 "s63E6H2" 0.9620556 "hypothetical protein"
"JS1_D03_00414" 12323 "s59E13H2" 0.02605688 "SigmaW regulon antibacterial"
"JS1_D03_00414" 12323 "s59E25H2" 0 "SigmaW regulon antibacterial"
"JS1_D03_00414" 12323 "s59E5H2" 0.1525324 "SigmaW regulon antibacterial"
"JS1_D03_00414" 12323 "s63E6H2" 0.8577252 "SigmaW regulon antibacterial"
"JS1_D03_00415" 12324 "s59E13H2" 0.005145233 "hypothetical protein"
"JS1_D03_00415" 12324 "s59E25H2" 0 "hypothetical protein"
"JS1_D03_00415" 12324 "s59E5H2" 0.03642088 "hypothetical protein"
"JS1_D03_00415" 12324 "s63E6H2" 0.2710142 "hypothetical protein"
"JS1_D03_00416" 12325 "s59E13H2" 0.007016076 "PhoH-like protein"
"JS1_D03_00416" 12325 "s59E25H2" 0 "PhoH-like protein"
"JS1_D03_00416" 12325 "s59E5H2" 0.06191002 "PhoH-like protein"
"JS1_D03_00416" 12325 "s63E6H2" 0.2436474 "PhoH-like protein"
"JS1_D03_00417" 12326 "s59E13H2" 0.05317124 "Ribonuclease Y"
"JS1_D03_00417" 12326 "s59E25H2" 0 "Ribonuclease Y"
"JS1_D03_00417" 12326 "s59E5H2" 0.1441633 "Ribonuclease Y"
"JS1_D03_00417" 12326 "s63E6H2" 0.2310767 "Ribonuclease Y"
"JS1_D03_00418" 12327 "s59E13H2" 0.1057193 "NMT1/THI5 like protein"
"JS1_D03_00418" 12327 "s59E25H2" 0 "NMT1/THI5 like protein"
"JS1_D03_00418" 12327 "s59E5H2" 0.07818104 "NMT1/THI5 like protein"
"JS1_D03_00418" 12327 "s63E6H2" 0.2452984 "NMT1/THI5 like protein"
"JS1_D03_00419" 12328 "s59E13H2" 0.04177376 "hypothetical protein"
"JS1_D03_00419" 12328 "s59E25H2" 0 "hypothetical protein"
"JS1_D03_00419" 12328 "s59E5H2" 0.04526166 "hypothetical protein"
"JS1_D03_00419" 12328 "s63E6H2" 0.1828121 "hypothetical protein"

"JS1_D03_00420" 12329 "s59E13H2" 0.157653 "Sialic acid TRAP transporter permease protein SiaT"
"JS1_D03_00420" 12329 "s59E25H2" 0 "Sialic acid TRAP transporter permease protein SiaT"
"JS1_D03_00420" 12329 "s59E5H2" 0.06399193 "Sialic acid TRAP transporter permease protein SiaT"
"JS1_D03_00420" 12329 "s63E6H2" 0.2472649 "Sialic acid TRAP transporter permease protein SiaT"
"JS1_D03_00421" 12330 "s59E13H2" 0.00494585 "Magnesium and cobalt efflux protein CorC"
"JS1_D03_00421" 12330 "s59E25H2" 0 "Magnesium and cobalt efflux protein CorC"
"JS1_D03_00421" 12330 "s59E5H2" 0.01274737 "Magnesium and cobalt efflux protein CorC"
"JS1_D03_00421" 12330 "s63E6H2" 0.04374893 "Magnesium and cobalt efflux protein CorC"
"JS1_D03_00422" 12331 "s59E13H2" 0.03799814 "Magnesium and cobalt efflux protein CorC"
"JS1_D03_00422" 12331 "s59E25H2" 0 "Magnesium and cobalt efflux protein CorC"
"JS1_D03_00422" 12331 "s59E5H2" 0.04105076 "Magnesium and cobalt efflux protein CorC"
"JS1_D03_00422" 12331 "s63E6H2" 0.0988566 "Magnesium and cobalt efflux protein CorC"
"JS1_D03_00423" 12332 "s59E13H2" 0.0005901715 "Putative lipoprotein YerB precursor"
"JS1_D03_00423" 12332 "s59E25H2" 0 "Putative lipoprotein YerB precursor"
"JS1_D03_00423" 12332 "s59E5H2" 0.06135895 "Putative lipoprotein YerB precursor"
"JS1_D03_00423" 12332 "s63E6H2" 0.07070398 "Putative lipoprotein YerB precursor"
"JS1_D03_00424" 12333 "s59E13H2" 0.2048844 "Trehalose/maltose-binding protein MalE precursor"
"JS1_D03_00424" 12333 "s59E25H2" 0 "Trehalose/maltose-binding protein MalE precursor"
"JS1_D03_00424" 12333 "s59E5H2" 0.3416108 "Trehalose/maltose-binding protein MalE precursor"
"JS1_D03_00424" 12333 "s63E6H2" 0.5172356 "Trehalose/maltose-binding protein MalE precursor"
"JS1_D03_00425" 12334 "s59E13H2" 0.05724748 "Inner membrane ABC transporter permease protein YcjO"
"JS1_D03_00425" 12334 "s59E25H2" 0 "Inner membrane ABC transporter permease protein YcjO"
"JS1_D03_00425" 12334 "s59E5H2" 0.1898441 "Inner membrane ABC transporter permease protein YcjO"
"JS1_D03_00425" 12334 "s63E6H2" 0.2799601 "Inner membrane ABC transporter permease protein YcjO"
"JS1_D03_00426" 12335 "s59E13H2" 0.09233871 "Trehalose/maltose transport system permease protein MalG"
"JS1_D03_00426" 12335 "s59E25H2" 0 "Trehalose/maltose transport system permease protein MalG"
"JS1_D03_00426" 12335 "s59E5H2" 0.2670433 "Trehalose/maltose transport system permease protein MalG"
"JS1_D03_00426" 12335 "s63E6H2" 0.2296972 "Trehalose/maltose transport system permease protein MalG"
"JS1_D03_00427" 12336 "s59E13H2" 0.1748232 "Ribokinase"
"JS1_D03_00427" 12336 "s59E25H2" 0 "Ribokinase"
"JS1_D03_00427" 12336 "s59E5H2" 0.1460955 "Ribokinase"
"JS1_D03_00427" 12336 "s63E6H2" 0.2775432 "Ribokinase"
"JS1_D03_00428" 12337 "s59E13H2" 0.1201109 "hypothetical protein"
"JS1_D03_00428" 12337 "s59E25H2" 0 "hypothetical protein"
"JS1_D03_00428" 12337 "s59E5H2" 0.13481 "hypothetical protein"
"JS1_D03_00428" 12337 "s63E6H2" 0.8026701 "hypothetical protein"
"JS1_D03_00429" 12338 "s59E13H2" 0.08895837 "hypothetical protein"
"JS1_D03_00429" 12338 "s59E25H2" 0 "hypothetical protein"
"JS1_D03_00429" 12338 "s59E5H2" 0.3079992 "hypothetical protein"
"JS1_D03_00429" 12338 "s63E6H2" 1.524462 "hypothetical protein"
"JS1_D03_00430" 12339 "s59E13H2" 0.05416346 "hypothetical protein"
"JS1_D03_00430" 12339 "s59E25H2" 0 "hypothetical protein"
"JS1_D03_00430" 12339 "s59E5H2" 0.292962 "hypothetical protein"
"JS1_D03_00430" 12339 "s63E6H2" 1.627017 "hypothetical protein"
"JS1_D03_00431" 12340 "s59E13H2" 0.06243302 "Carbon dioxide concentrating mechanism protein CcmL"
"JS1_D03_00431" 12340 "s59E25H2" 0 "Carbon dioxide concentrating mechanism protein CcmL"
"JS1_D03_00431" 12340 "s59E5H2" 0.4005579 "Carbon dioxide concentrating mechanism protein CcmL"

"JS1_D03_00431" 12340 "s63E6H2" 1.627537 "Carbon dioxide concentrating mechanism protein CcmL"
"JS1_D03_00432" 12341 "s59E13H2" 0.0991655 "Carbon dioxide concentrating mechanism protein CcmL"
"JS1_D03_00432" 12341 "s59E25H2" 0 "Carbon dioxide concentrating mechanism protein CcmL"
"JS1_D03_00432" 12341 "s59E5H2" 0.486352 "Carbon dioxide concentrating mechanism protein CcmL"
"JS1_D03_00432" 12341 "s63E6H2" 1.71075 "Carbon dioxide concentrating mechanism protein CcmL"
"JS1_D03_00433" 12342 "s59E13H2" 0.1073096 "Electron transport complex protein RnfC"
"JS1_D03_00433" 12342 "s59E25H2" 0 "Electron transport complex protein RnfC"
"JS1_D03_00433" 12342 "s59E5H2" 0.7046896 "Electron transport complex protein RnfC"
"JS1_D03_00433" 12342 "s63E6H2" 2.580923 "Electron transport complex protein RnfC"
"JS1_D03_00434" 12343 "s59E13H2" 0.125539 "BMC domain protein"
"JS1_D03_00434" 12343 "s59E25H2" 0 "BMC domain protein"
"JS1_D03_00434" 12343 "s59E5H2" 0.7607246 "BMC domain protein"
"JS1_D03_00434" 12343 "s63E6H2" 3.053184 "BMC domain protein"
"JS1_D03_00435" 12344 "s59E13H2" 0.08504815 "Aldehyde-alcohol dehydrogenase"
"JS1_D03_00435" 12344 "s59E25H2" 0 "Aldehyde-alcohol dehydrogenase"
"JS1_D03_00435" 12344 "s59E5H2" 0.5648941 "Aldehyde-alcohol dehydrogenase"
"JS1_D03_00435" 12344 "s63E6H2" 2.590582 "Aldehyde-alcohol dehydrogenase"
"JS1_D03_00436" 12345 "s59E13H2" 0.07352319 "Carbon dioxide concentrating mechanism protein CcmL"
"JS1_D03_00436" 12345 "s59E25H2" 0 "Carbon dioxide concentrating mechanism protein CcmL"
"JS1_D03_00436" 12345 "s59E5H2" 0.5684914 "Carbon dioxide concentrating mechanism protein CcmL"
"JS1_D03_00436" 12345 "s63E6H2" 2.012875 "Carbon dioxide concentrating mechanism protein CcmL"
"JS1_D03_00437" 12346 "s59E13H2" 0.09856332 "Carbon dioxide concentrating mechanism protein CcmL"
"JS1_D03_00437" 12346 "s59E25H2" 0 "Carbon dioxide concentrating mechanism protein CcmL"
"JS1_D03_00437" 12346 "s59E5H2" 0.5923287 "Carbon dioxide concentrating mechanism protein CcmL"
"JS1_D03_00437" 12346 "s63E6H2" 4.348201 "Carbon dioxide concentrating mechanism protein CcmL"
"JS1_D03_00438" 12347 "s59E13H2" 0.1308599 "Putative sugar phosphate isomerase YwlF"
"JS1_D03_00438" 12347 "s59E25H2" 0 "Putative sugar phosphate isomerase YwlF"
"JS1_D03_00438" 12347 "s59E5H2" 0.4290008 "Putative sugar phosphate isomerase YwlF"
"JS1_D03_00438" 12347 "s63E6H2" 5.939411 "Putative sugar phosphate isomerase YwlF"
"JS1_D03_00439" 12348 "s59E13H2" 0.0783868 "Deoxyribose-phosphate aldolase 1"
"JS1_D03_00439" 12348 "s59E25H2" 0 "Deoxyribose-phosphate aldolase 1"
"JS1_D03_00439" 12348 "s59E5H2" 0.1213099 "Deoxyribose-phosphate aldolase 1"
"JS1_D03_00439" 12348 "s63E6H2" 2.551737 "Deoxyribose-phosphate aldolase 1"
"JS1_D03_00440" 12349 "s59E13H2" 0.1194202 "Phosphate propanoyltransferase"
"JS1_D03_00440" 12349 "s59E25H2" 0 "Phosphate propanoyltransferase"
"JS1_D03_00440" 12349 "s59E5H2" 0.4455636 "Phosphate propanoyltransferase"
"JS1_D03_00440" 12349 "s63E6H2" 3.081635 "Phosphate propanoyltransferase"
"JS1_D03_00441" 12350 "s59E13H2" 0.02463885 "Deoxyribose operon repressor"
"JS1_D03_00441" 12350 "s59E25H2" 0 "Deoxyribose operon repressor"
"JS1_D03_00441" 12350 "s59E5H2" 0.3361233 "Deoxyribose operon repressor"
"JS1_D03_00441" 12350 "s63E6H2" 0.2852044 "Deoxyribose operon repressor"
"JS1_D03_00442" 12351 "s59E13H2" 0.006599909 "hypothetical protein"
"JS1_D03_00442" 12351 "s59E25H2" 0 "hypothetical protein"
"JS1_D03_00442" 12351 "s59E5H2" 0.3617857 "hypothetical protein"
"JS1_D03_00442" 12351 "s63E6H2" 0.1629549 "hypothetical protein"
"JS1_D03_00443" 12352 "s59E13H2" 0.0156707 "HTH-type transcriptional regulator MtrR"
"JS1_D03_00443" 12352 "s59E25H2" 0 "HTH-type transcriptional regulator MtrR"

"JS1_D03_00443" 12352 "s59E5H2" 0.06587218 "HTH-type transcriptional regulator MtrR"
"JS1_D03_00443" 12352 "s63E6H2" 0.06343606 "HTH-type transcriptional regulator MtrR"
"JS1_D03_00444" 12353 "s59E13H2" 0.0009520537 "Lipoprotein-releasing system transmembrane protein LolE"
"JS1_D03_00444" 12353 "s59E25H2" 0 "Lipoprotein-releasing system transmembrane protein LolE"
"JS1_D03_00444" 12353 "s59E5H2" 0.3136487 "Lipoprotein-releasing system transmembrane protein LolE"
"JS1_D03_00444" 12353 "s63E6H2" 0.05963284 "Lipoprotein-releasing system transmembrane protein LolE"
"JS1_D03_00445" 12354 "s59E13H2" 0.04688473 "putative ABC transporter ATP-binding protein"
"JS1_D03_00445" 12354 "s59E25H2" 0 "putative ABC transporter ATP-binding protein"
"JS1_D03_00445" 12354 "s59E5H2" 0.1219169 "putative ABC transporter ATP-binding protein"
"JS1_D03_00445" 12354 "s63E6H2" 0.03304843 "putative ABC transporter ATP-binding protein"
"JS1_D03_00446" 12355 "s59E13H2" 0.01204738 "Lipoprotein-releasing system transmembrane protein LolE"
"JS1_D03_00446" 12355 "s59E25H2" 0 "Lipoprotein-releasing system transmembrane protein LolE"
"JS1_D03_00446" 12355 "s59E5H2" 0.1224189 "Lipoprotein-releasing system transmembrane protein LolE"
"JS1_D03_00446" 12355 "s63E6H2" 0.01641261 "Lipoprotein-releasing system transmembrane protein LolE"
"JS1_D03_00447" 12356 "s59E13H2" 0.002742585 "Lipoprotein-releasing system transmembrane protein LolE"
"JS1_D03_00447" 12356 "s59E25H2" 0 "Lipoprotein-releasing system transmembrane protein LolE"
"JS1_D03_00447" 12356 "s59E5H2" 0.02170549 "Lipoprotein-releasing system transmembrane protein LolE"
"JS1_D03_00447" 12356 "s63E6H2" 0.09296548 "Lipoprotein-releasing system transmembrane protein LolE"
"JS1_D03_00448" 12357 "s59E13H2" 0 "hypothetical protein"
"JS1_D03_00448" 12357 "s59E25H2" 0 "hypothetical protein"
"JS1_D03_00448" 12357 "s59E5H2" 0 "hypothetical protein"
"JS1_D03_00448" 12357 "s63E6H2" 0.02662504 "hypothetical protein"
"JS1_D03_00449" 12358 "s59E13H2" 0.02623502 "hypothetical protein"
"JS1_D03_00449" 12358 "s59E25H2" 0 "hypothetical protein"
"JS1_D03_00449" 12358 "s59E5H2" 0.9482188 "hypothetical protein"
"JS1_D03_00449" 12358 "s63E6H2" 0.2551991 "hypothetical protein"
"JS1_D03_00450" 12359 "s59E13H2" 1.57688 "Anaerobic regulatory protein"
"JS1_D03_00450" 12359 "s59E25H2" 0 "Anaerobic regulatory protein"
"JS1_D03_00450" 12359 "s59E5H2" 0.2442375 "Anaerobic regulatory protein"
"JS1_D03_00450" 12359 "s63E6H2" 0.1718575 "Anaerobic regulatory protein"
"JS1_D03_00451" 12360 "s59E13H2" 2.882567 "ferredoxin"
"JS1_D03_00451" 12360 "s59E25H2" 0 "ferredoxin"
"JS1_D03_00451" 12360 "s59E5H2" 0.3494963 "ferredoxin"
"JS1_D03_00451" 12360 "s63E6H2" 0.3811153 "ferredoxin"
"JS1_D03_00452" 12361 "s59E13H2" 2.299697 "Hydroxylamine reductase"
"JS1_D03_00452" 12361 "s59E25H2" 0 "Hydroxylamine reductase"
"JS1_D03_00452" 12361 "s59E5H2" 0.3670548 "Hydroxylamine reductase"
"JS1_D03_00452" 12361 "s63E6H2" 0.3523859 "Hydroxylamine reductase"
"JS1_D03_00453" 12362 "s59E13H2" 0.0374782 "3-oxoacyl-[acyl-carrier-protein] synthase 3"
"JS1_D03_00453" 12362 "s59E25H2" 0 "3-oxoacyl-[acyl-carrier-protein] synthase 3"
"JS1_D03_00453" 12362 "s59E5H2" 0.3082041 "3-oxoacyl-[acyl-carrier-protein] synthase 3"
"JS1_D03_00453" 12362 "s63E6H2" 0.1718573 "3-oxoacyl-[acyl-carrier-protein] synthase 3"
"JS1_D03_00454" 12363 "s59E13H2" 0.02567185 "3-oxoacyl-[acyl-carrier-protein] synthase 2"
"JS1_D03_00454" 12363 "s59E25H2" 0 "3-oxoacyl-[acyl-carrier-protein] synthase 2"
"JS1_D03_00454" 12363 "s59E5H2" 0.2704566 "3-oxoacyl-[acyl-carrier-protein] synthase 2"
"JS1_D03_00454" 12363 "s63E6H2" 0.05316197 "3-oxoacyl-[acyl-carrier-protein] synthase 2"
"JS1_D03_00455" 12364 "s59E13H2" 0.3636772 "isopentenyl pyrophosphate isomerase"

"JS1_D03_00455" 12364 "s59E25H2" 0 "isopentenyl pyrophosphate isomerase"
"JS1_D03_00455" 12364 "s59E5H2" 0.5013369 "isopentenyl pyrophosphate isomerase"
"JS1_D03_00455" 12364 "s63E6H2" 0.06531676 "isopentenyl pyrophosphate isomerase"
"JS1_D03_00456" 12365 "s59E13H2" 0.2617992 "hypothetical protein"
"JS1_D03_00456" 12365 "s59E25H2" 0 "hypothetical protein"
"JS1_D03_00456" 12365 "s59E5H2" 0.2910619 "hypothetical protein"
"JS1_D03_00456" 12365 "s63E6H2" 0.02642873 "hypothetical protein"
"JS1_D03_00457" 12366 "s59E13H2" 0.06278269 "hypothetical protein"
"JS1_D03_00457" 12366 "s59E25H2" 0 "hypothetical protein"
"JS1_D03_00457" 12366 "s59E5H2" 0.15896 "hypothetical protein"
"JS1_D03_00457" 12366 "s63E6H2" 0.06498237 "hypothetical protein"
"JS1_D03_00458" 12367 "s59E13H2" 0.2808857 "hypothetical protein"
"JS1_D03_00458" 12367 "s59E25H2" 0 "hypothetical protein"
"JS1_D03_00458" 12367 "s59E5H2" 0.9037779 "hypothetical protein"
"JS1_D03_00458" 12367 "s63E6H2" 0.09606064 "hypothetical protein"
"JS1_D03_00459" 12368 "s59E13H2" 0.03406757 "putative inner membrane transporter YedA"
"JS1_D03_00459" 12368 "s59E25H2" 0 "putative inner membrane transporter YedA"
"JS1_D03_00459" 12368 "s59E5H2" 1.825139 "putative inner membrane transporter YedA"
"JS1_D03_00459" 12368 "s63E6H2" 0.07795273 "putative inner membrane transporter YedA"
"JS1_D03_00460" 12369 "s59E13H2" 0.09828498 "Outer membrane efflux protein"
"JS1_D03_00460" 12369 "s59E25H2" 0 "Outer membrane efflux protein"
"JS1_D03_00460" 12369 "s59E5H2" 1.449863 "Outer membrane efflux protein"
"JS1_D03_00460" 12369 "s63E6H2" 0.07372129 "Outer membrane efflux protein"
"JS1_D03_00461" 12370 "s59E13H2" 0.1893175 "Outer membrane efflux protein"
"JS1_D03_00461" 12370 "s59E25H2" 0 "Outer membrane efflux protein"
"JS1_D03_00461" 12370 "s59E5H2" 0.2954251 "Outer membrane efflux protein"
"JS1_D03_00461" 12370 "s63E6H2" 0.0631167 "Outer membrane efflux protein"
"JS1_D03_00462" 12371 "s59E13H2" 0.1389325 "Putative efflux system component YknX"
"JS1_D03_00462" 12371 "s59E25H2" 0 "Putative efflux system component YknX"
"JS1_D03_00462" 12371 "s59E5H2" 0.4230999 "Putative efflux system component YknX"
"JS1_D03_00462" 12371 "s63E6H2" 0.5262505 "Putative efflux system component YknX"
"JS1_D03_00463" 12372 "s59E13H2" 0.1206559 "putative ABC transporter ATP-binding protein"
"JS1_D03_00463" 12372 "s59E25H2" 0 "putative ABC transporter ATP-binding protein"
"JS1_D03_00463" 12372 "s59E5H2" 0.5049309 "putative ABC transporter ATP-binding protein"
"JS1_D03_00463" 12372 "s63E6H2" 1.414242 "putative ABC transporter ATP-binding protein"
"JS1_D03_00464" 12373 "s59E13H2" 0.1763054 "Macrolide export ATP-binding/permease protein MacB"
"JS1_D03_00464" 12373 "s59E25H2" 0.1978654 "Macrolide export ATP-binding/permease protein MacB"
"JS1_D03_00464" 12373 "s59E5H2" 0.6917139 "Macrolide export ATP-binding/permease protein MacB"
"JS1_D03_00464" 12373 "s63E6H2" 1.093638 "Macrolide export ATP-binding/permease protein MacB"
"JS1_D03_00465" 12374 "s59E13H2" 0.01062094 "Trifunctional nucleotide phosphoesterase protein YfkN precursor"
"JS1_D03_00465" 12374 "s59E25H2" 0 "Trifunctional nucleotide phosphoesterase protein YfkN precursor"
"JS1_D03_00465" 12374 "s59E5H2" 0.3059496 "Trifunctional nucleotide phosphoesterase protein YfkN precursor"
"JS1_D03_00465" 12374 "s63E6H2" 1.008541 "Trifunctional nucleotide phosphoesterase protein YfkN precursor"
"JS1_D03_00466" 12375 "s59E13H2" 0.05353555 "hypothetical protein"
"JS1_D03_00466" 12375 "s59E25H2" 0 "hypothetical protein"
"JS1_D03_00466" 12375 "s59E5H2" 0.5659929 "hypothetical protein"
"JS1_D03_00466" 12375 "s63E6H2" 1.553564 "hypothetical protein"

"JS1_D03_00467" 12376 "s59E13H2" 0.02112297 "LemA family protein"
"JS1_D03_00467" 12376 "s59E25H2" 0 "LemA family protein"
"JS1_D03_00467" 12376 "s59E5H2" 0.5336138 "LemA family protein"
"JS1_D03_00467" 12376 "s63E6H2" 0.8492197 "LemA family protein"
"JS1_D03_00468" 12377 "s59E13H2" 0.01837833 "galactoside permease"
"JS1_D03_00468" 12377 "s59E25H2" 0 "galactoside permease"
"JS1_D03_00468" 12377 "s59E5H2" 0.3493252 "galactoside permease"
"JS1_D03_00468" 12377 "s63E6H2" 0.8134128 "galactoside permease"
"JS1_D03_00469" 12378 "s59E13H2" 0.02806643 "hypothetical protein"
"JS1_D03_00469" 12378 "s59E25H2" 0 "hypothetical protein"
"JS1_D03_00469" 12378 "s59E5H2" 0.4561185 "hypothetical protein"
"JS1_D03_00469" 12378 "s63E6H2" 0.270795 "hypothetical protein"
"JS1_D03_00470" 12379 "s59E13H2" 0.02952635 "Acetyltransferase (GNAT) family protein"
"JS1_D03_00470" 12379 "s59E25H2" 0 "Acetyltransferase (GNAT) family protein"
"JS1_D03_00470" 12379 "s59E5H2" 1.109429 "Acetyltransferase (GNAT) family protein"
"JS1_D03_00470" 12379 "s63E6H2" 0.884854 "Acetyltransferase (GNAT) family protein"
"JS1_D03_00471" 12380 "s59E13H2" 0.06778795 "putative inner membrane transporter YicL"
"JS1_D03_00471" 12380 "s59E25H2" 0 "putative inner membrane transporter YicL"
"JS1_D03_00471" 12380 "s59E5H2" 0.2698883 "putative inner membrane transporter YicL"
"JS1_D03_00471" 12380 "s63E6H2" 0.1858961 "putative inner membrane transporter YicL"
"JS1_D03_00472" 12381 "s59E13H2" 0.05353061 "Response regulator aspartate phosphatase G"
"JS1_D03_00472" 12381 "s59E25H2" 0 "Response regulator aspartate phosphatase G"
"JS1_D03_00472" 12381 "s59E5H2" 0.6022434 "Response regulator aspartate phosphatase G"
"JS1_D03_00472" 12381 "s63E6H2" 0.1632935 "Response regulator aspartate phosphatase G"
"JS1_D03_00473" 12382 "s59E13H2" 0.02880562 "hypothetical protein"
"JS1_D03_00473" 12382 "s59E25H2" 0 "hypothetical protein"
"JS1_D03_00473" 12382 "s59E5H2" 0.8428686 "hypothetical protein"
"JS1_D03_00473" 12382 "s63E6H2" 0.193402 "hypothetical protein"
"JS1_D03_00474" 12383 "s59E13H2" 0.0004091525 "EamA-like transporter family protein"
"JS1_D03_00474" 12383 "s59E25H2" 0 "EamA-like transporter family protein"
"JS1_D03_00474" 12383 "s59E5H2" 0.07351631 "EamA-like transporter family protein"
"JS1_D03_00474" 12383 "s63E6H2" 0.01951658 "EamA-like transporter family protein"
"JS1_D03_00475" 12384 "s59E13H2" 0 "L-lysine 2,3-aminomutase"
"JS1_D03_00475" 12384 "s59E25H2" 0 "L-lysine 2,3-aminomutase"
"JS1_D03_00475" 12384 "s59E5H2" 0.003070404 "L-lysine 2,3-aminomutase"
"JS1_D03_00475" 12384 "s63E6H2" 0.01571404 "L-lysine 2,3-aminomutase"
"JS1_D03_00478" 12387 "s59E13H2" 0.01092093 "recombination protein F"
"JS1_D03_00478" 12387 "s59E25H2" 0 "recombination protein F"
"JS1_D03_00478" 12387 "s59E5H2" 0.004764196 "recombination protein F"
"JS1_D03_00478" 12387 "s63E6H2" 0 "recombination protein F"
"JS1_D03_00479" 12388 "s59E13H2" 0.009318555 "ATP-dependent DNA helicase PcrA"
"JS1_D03_00479" 12388 "s59E25H2" 0 "ATP-dependent DNA helicase PcrA"
"JS1_D03_00479" 12388 "s59E5H2" 0.017558 "ATP-dependent DNA helicase PcrA"
"JS1_D03_00479" 12388 "s63E6H2" 0.03908468 "ATP-dependent DNA helicase PcrA"
"JS1_D03_00481" 12390 "s59E13H2" 0 "Type-1 restriction enzyme R protein"
"JS1_D03_00481" 12390 "s59E25H2" 0 "Type-1 restriction enzyme R protein"
"JS1_D03_00481" 12390 "s59E5H2" 0 "Type-1 restriction enzyme R protein"

"JS1_D03_00481" 12390 "s63E6H2" 0 "Type-1 restriction enzyme R protein"
"JS1_D03_00482" 12391 "s59E13H2" 0 "Type-1 restriction enzyme MjaXIP specificity protein"
"JS1_D03_00482" 12391 "s59E25H2" 0 "Type-1 restriction enzyme MjaXIP specificity protein"
"JS1_D03_00482" 12391 "s59E5H2" 0 "Type-1 restriction enzyme MjaXIP specificity protein"
"JS1_D03_00482" 12391 "s63E6H2" 0 "Type-1 restriction enzyme MjaXIP specificity protein"
"JS1_D03_00484" 12393 "s59E13H2" 0 "Type I restriction enzyme EcoKI M protein"
"JS1_D03_00484" 12393 "s59E25H2" 0 "Type I restriction enzyme EcoKI M protein"
"JS1_D03_00484" 12393 "s59E5H2" 0 "Type I restriction enzyme EcoKI M protein"
"JS1_D03_00484" 12393 "s63E6H2" 0 "Type I restriction enzyme EcoKI M protein"
"JS1_D03_00485" 12394 "s59E13H2" 0 "hypothetical protein"
"JS1_D03_00485" 12394 "s59E25H2" 0 "hypothetical protein"
"JS1_D03_00485" 12394 "s59E5H2" 0 "hypothetical protein"
"JS1_D03_00485" 12394 "s63E6H2" 0 "hypothetical protein"
"JS1_D03_00486" 12395 "s59E13H2" 4.196836 "Adenosine monophosphate-protein transferase SoFic"
"JS1_D03_00486" 12395 "s59E25H2" 0 "Adenosine monophosphate-protein transferase SoFic"
"JS1_D03_00486" 12395 "s59E5H2" 0.6984321 "Adenosine monophosphate-protein transferase SoFic"
"JS1_D03_00486" 12395 "s63E6H2" 0.5445532 "Adenosine monophosphate-protein transferase SoFic"
"JS1_D03_00487" 12396 "s59E13H2" 17.59294 "DNA-binding transcriptional dual regulator Crp"
"JS1_D03_00487" 12396 "s59E25H2" 0.7066895 "DNA-binding transcriptional dual regulator Crp"
"JS1_D03_00487" 12396 "s59E5H2" 1.127054 "DNA-binding transcriptional dual regulator Crp"
"JS1_D03_00487" 12396 "s63E6H2" 0.3894708 "DNA-binding transcriptional dual regulator Crp"
"JS1_D03_00488" 12397 "s59E13H2" 12.4021 "D-inositol 3-phosphate glycosyltransferase"
"JS1_D03_00488" 12397 "s59E25H2" 0 "D-inositol 3-phosphate glycosyltransferase"
"JS1_D03_00488" 12397 "s59E5H2" 0.8845484 "D-inositol 3-phosphate glycosyltransferase"
"JS1_D03_00488" 12397 "s63E6H2" 1.033215 "D-inositol 3-phosphate glycosyltransferase"
"JS1_D03_00489" 12398 "s59E13H2" 5.914174 "hypothetical protein"
"JS1_D03_00489" 12398 "s59E25H2" 0 "hypothetical protein"
"JS1_D03_00489" 12398 "s59E5H2" 0.1949703 "hypothetical protein"
"JS1_D03_00489" 12398 "s63E6H2" 0.2649011 "hypothetical protein"
"JS1_D03_00490" 12399 "s59E13H2" 0.4221554 "hypothetical protein"
"JS1_D03_00490" 12399 "s59E25H2" 0 "hypothetical protein"
"JS1_D03_00490" 12399 "s59E5H2" 0.1079213 "hypothetical protein"
"JS1_D03_00490" 12399 "s63E6H2" 0.09187883 "hypothetical protein"
"JS1_D03_00491" 12400 "s59E13H2" 5.922022 "Aspartate racemase"
"JS1_D03_00491" 12400 "s59E25H2" 0 "Aspartate racemase"
"JS1_D03_00491" 12400 "s59E5H2" 0.8311685 "Aspartate racemase"
"JS1_D03_00491" 12400 "s63E6H2" 0.2591539 "Aspartate racemase"
"JS1_D03_00492" 12401 "s59E13H2" 0.4549171 "1-acyl-sn-glycerol-3-phosphate acyltransferase"
"JS1_D03_00492" 12401 "s59E25H2" 0 "1-acyl-sn-glycerol-3-phosphate acyltransferase"
"JS1_D03_00492" 12401 "s59E5H2" 0.1698096 "1-acyl-sn-glycerol-3-phosphate acyltransferase"
"JS1_D03_00492" 12401 "s63E6H2" 0.1634584 "1-acyl-sn-glycerol-3-phosphate acyltransferase"
"JS1_D03_00493" 12402 "s59E13H2" 0 "hypothetical protein"
"JS1_D03_00493" 12402 "s59E25H2" 0 "hypothetical protein"
"JS1_D03_00493" 12402 "s59E5H2" 0.02341695 "hypothetical protein"
"JS1_D03_00493" 12402 "s63E6H2" 0.05962616 "hypothetical protein"
"JS1_D03_00494" 12403 "s59E13H2" 0.03005033 "Alpha/beta hydrolase family protein"
"JS1_D03_00494" 12403 "s59E25H2" 0 "Alpha/beta hydrolase family protein"

"JS1_D03_00494" 12403 "s59E5H2" 0.2281253 "Alpha/beta hydrolase family protein"
"JS1_D03_00494" 12403 "s63E6H2" 0.03399811 "Alpha/beta hydrolase family protein"
"JS1_D03_00495" 12404 "s59E13H2" 0.03554472 "Ubiquinone biosynthesis O-methyltransferase"
"JS1_D03_00495" 12404 "s59E25H2" 0 "Ubiquinone biosynthesis O-methyltransferase"
"JS1_D03_00495" 12404 "s59E5H2" 1.393209 "Ubiquinone biosynthesis O-methyltransferase"
"JS1_D03_00495" 12404 "s63E6H2" 0.9568977 "Ubiquinone biosynthesis O-methyltransferase"
"JS1_D03_00496" 12405 "s59E13H2" 0.003970087 "hypothetical protein"
"JS1_D03_00496" 12405 "s59E25H2" 0 "hypothetical protein"
"JS1_D03_00496" 12405 "s59E5H2" 0.3727588 "hypothetical protein"
"JS1_D03_00496" 12405 "s63E6H2" 0.1357986 "hypothetical protein"
"JS1_D03_00497" 12406 "s59E13H2" 0.1679177 "putative permease"
"JS1_D03_00497" 12406 "s59E25H2" 0 "putative permease"
"JS1_D03_00497" 12406 "s59E5H2" 0.4405229 "putative permease"
"JS1_D03_00497" 12406 "s63E6H2" 2.550572 "putative permease"
"JS1_D03_00498" 12407 "s59E13H2" 0 "Thioredoxin"
"JS1_D03_00498" 12407 "s59E25H2" 0 "Thioredoxin"
"JS1_D03_00498" 12407 "s59E5H2" 0.06984042 "Thioredoxin"
"JS1_D03_00498" 12407 "s63E6H2" 0.02778004 "Thioredoxin"
"JS1_D03_00499" 12408 "s59E13H2" 0.05261019 "Thioredoxin-1"
"JS1_D03_00499" 12408 "s59E25H2" 0.2058824 "Thioredoxin-1"
"JS1_D03_00499" 12408 "s59E5H2" 3.944179 "Thioredoxin-1"
"JS1_D03_00499" 12408 "s63E6H2" 16.8801 "Thioredoxin-1"
"JS1_D03_00500" 12409 "s59E13H2" 0.1418564 "thiol:disulfide interchange protein precursor"
"JS1_D03_00500" 12409 "s59E25H2" 0.8547009 "thiol:disulfide interchange protein precursor"
"JS1_D03_00500" 12409 "s59E5H2" 6.479774 "thiol:disulfide interchange protein precursor"
"JS1_D03_00500" 12409 "s63E6H2" 29.64071 "thiol:disulfide interchange protein precursor"
"JS1_D03_00501" 12410 "s59E13H2" 0.1974038 "Sodium Bile acid symporter family protein"
"JS1_D03_00501" 12410 "s59E25H2" 0 "Sodium Bile acid symporter family protein"
"JS1_D03_00501" 12410 "s59E5H2" 4.772962 "Sodium Bile acid symporter family protein"
"JS1_D03_00501" 12410 "s63E6H2" 32.20641 "Sodium Bile acid symporter family protein"
"JS1_D03_00502" 12411 "s59E13H2" 0.09773405 "hypothetical protein"
"JS1_D03_00502" 12411 "s59E25H2" 0 "hypothetical protein"
"JS1_D03_00502" 12411 "s59E5H2" 0.9454557 "hypothetical protein"
"JS1_D03_00502" 12411 "s63E6H2" 0.07478491 "hypothetical protein"
"JS1_D03_00503" 12412 "s59E13H2" 0.0001575701 "Na(+)/H(+) antiporter subunit D1"
"JS1_D03_00503" 12412 "s59E25H2" 0 "Na(+)/H(+) antiporter subunit D1"
"JS1_D03_00503" 12412 "s59E5H2" 1.924317 "Na(+)/H(+) antiporter subunit D1"
"JS1_D03_00503" 12412 "s63E6H2" 0.5307967 "Na(+)/H(+) antiporter subunit D1"
"JS1_D03_00504" 12413 "s59E13H2" 0.08651302 "Na(+)/H(+) antiporter subunit D"
"JS1_D03_00504" 12413 "s59E25H2" 0 "Na(+)/H(+) antiporter subunit D"
"JS1_D03_00504" 12413 "s59E5H2" 4.704705 "Na(+)/H(+) antiporter subunit D"
"JS1_D03_00504" 12413 "s63E6H2" 0.7746591 "Na(+)/H(+) antiporter subunit D"
"JS1_D03_00505" 12414 "s59E13H2" 0.1302375 "Na(+)/H(+) antiporter subunit C"
"JS1_D03_00505" 12414 "s59E25H2" 0 "Na(+)/H(+) antiporter subunit C"
"JS1_D03_00505" 12414 "s59E5H2" 6.173176 "Na(+)/H(+) antiporter subunit C"
"JS1_D03_00505" 12414 "s63E6H2" 1.087162 "Na(+)/H(+) antiporter subunit C"
"JS1_D03_00506" 12415 "s59E13H2" 0.2691455 "Na(+)/H(+) antiporter subunit B"

"JS1_D03_00506" 12415 "s59E25H2" 0 "Na(+)/H(+) antiporter subunit B"
"JS1_D03_00506" 12415 "s59E5H2" 3.084568 "Na(+)/H(+) antiporter subunit B"
"JS1_D03_00506" 12415 "s63E6H2" 1.149485 "Na(+)/H(+) antiporter subunit B"
"JS1_D03_00507" 12416 "s59E13H2" 0.3067416 "putative monovalent cation/H+ antiporter subunit B"
"JS1_D03_00507" 12416 "s59E25H2" 0 "putative monovalent cation/H+ antiporter subunit B"
"JS1_D03_00507" 12416 "s59E5H2" 2.700274 "putative monovalent cation/H+ antiporter subunit B"
"JS1_D03_00507" 12416 "s63E6H2" 1.263592 "putative monovalent cation/H+ antiporter subunit B"
"JS1_D03_00508" 12417 "s59E13H2" 0.8892243 "Glutamine-binding periplasmic protein precursor"
"JS1_D03_00508" 12417 "s59E25H2" 0 "Glutamine-binding periplasmic protein precursor"
"JS1_D03_00508" 12417 "s59E5H2" 2.463979 "Glutamine-binding periplasmic protein precursor"
"JS1_D03_00508" 12417 "s63E6H2" 1.80745 "Glutamine-binding periplasmic protein precursor"
"JS1_D03_00509" 12418 "s59E13H2" 1.673534 "Na(+)/H(+) antiporter subunit G1"
"JS1_D03_00509" 12418 "s59E25H2" 0 "Na(+)/H(+) antiporter subunit G1"
"JS1_D03_00509" 12418 "s59E5H2" 2.448681 "Na(+)/H(+) antiporter subunit G1"
"JS1_D03_00509" 12418 "s63E6H2" 1.829436 "Na(+)/H(+) antiporter subunit G1"
"JS1_D03_00510" 12419 "s59E13H2" 1.717224 "putative monovalent cation/H+ antiporter subunit F"
"JS1_D03_00510" 12419 "s59E25H2" 0 "putative monovalent cation/H+ antiporter subunit F"
"JS1_D03_00510" 12419 "s59E5H2" 2.774756 "putative monovalent cation/H+ antiporter subunit F"
"JS1_D03_00510" 12419 "s63E6H2" 1.464517 "putative monovalent cation/H+ antiporter subunit F"
"JS1_D03_00511" 12420 "s59E13H2" 0.7034034 "Na(+)/H(+) antiporter subunit E1"
"JS1_D03_00511" 12420 "s59E25H2" 0 "Na(+)/H(+) antiporter subunit E1"
"JS1_D03_00511" 12420 "s59E5H2" 1.716243 "Na(+)/H(+) antiporter subunit E1"
"JS1_D03_00511" 12420 "s63E6H2" 1.273872 "Na(+)/H(+) antiporter subunit E1"
"JS1_D03_00512" 12421 "s59E13H2" 1.263014 "Pyruvate formate-lyase 1-activating enzyme"
"JS1_D03_00512" 12421 "s59E25H2" 0 "Pyruvate formate-lyase 1-activating enzyme"
"JS1_D03_00512" 12421 "s59E5H2" 0.4545614 "Pyruvate formate-lyase 1-activating enzyme"
"JS1_D03_00512" 12421 "s63E6H2" 1.297006 "Pyruvate formate-lyase 1-activating enzyme"
"JS1_D03_00513" 12422 "s59E13H2" 0.8530736 "hypothetical protein"
"JS1_D03_00513" 12422 "s59E25H2" 0 "hypothetical protein"
"JS1_D03_00513" 12422 "s59E5H2" 4.824982 "hypothetical protein"
"JS1_D03_00513" 12422 "s63E6H2" 1.03004 "hypothetical protein"
"JS1_D03_00514" 12423 "s59E13H2" 0.3872635 "Hydrogenase expression/formation protein HypE"
"JS1_D03_00514" 12423 "s59E25H2" 0 "Hydrogenase expression/formation protein HypE"
"JS1_D03_00514" 12423 "s59E5H2" 0.05638027 "Hydrogenase expression/formation protein HypE"
"JS1_D03_00514" 12423 "s63E6H2" 0.9489344 "Hydrogenase expression/formation protein HypE"
"JS1_D03_00515" 12424 "s59E13H2" 0 "Transposase IS200 like protein"
"JS1_D03_00515" 12424 "s59E25H2" 0 "Transposase IS200 like protein"
"JS1_D03_00515" 12424 "s59E5H2" 0 "Transposase IS200 like protein"
"JS1_D03_00515" 12424 "s63E6H2" 0.4396059 "Transposase IS200 like protein"
"JS1_D03_00516" 12425 "s59E13H2" 0.3856059 "Hydrogenase expression/formation protein HypD"
"JS1_D03_00516" 12425 "s59E25H2" 0 "Hydrogenase expression/formation protein HypD"
"JS1_D03_00516" 12425 "s59E5H2" 0.4200449 "Hydrogenase expression/formation protein HypD"
"JS1_D03_00516" 12425 "s63E6H2" 0.9391119 "Hydrogenase expression/formation protein HypD"
"JS1_D03_00517" 12426 "s59E13H2" 0.4903693 "Hydrogenase isoenzymes formation protein HypC"
"JS1_D03_00517" 12426 "s59E25H2" 0 "Hydrogenase isoenzymes formation protein HypC"
"JS1_D03_00517" 12426 "s59E5H2" 0.3017184 "Hydrogenase isoenzymes formation protein HypC"
"JS1_D03_00517" 12426 "s63E6H2" 0.8158632 "Hydrogenase isoenzymes formation protein HypC"

"JS1_D03_00518" 12427 "s59E13H2" 0.2950905 "Carbamoyltransferase HypF"
"JS1_D03_00518" 12427 "s59E25H2" 0 "Carbamoyltransferase HypF"
"JS1_D03_00518" 12427 "s59E5H2" 0.1332846 "Carbamoyltransferase HypF"
"JS1_D03_00518" 12427 "s63E6H2" 0.7262917 "Carbamoyltransferase HypF"
"JS1_D03_00519" 12428 "s59E13H2" 0.236605 "Hydrogenase isoenzymes nickel incorporation protein HypB"
"JS1_D03_00519" 12428 "s59E25H2" 0 "Hydrogenase isoenzymes nickel incorporation protein HypB"
"JS1_D03_00519" 12428 "s59E5H2" 0.1846823 "Hydrogenase isoenzymes nickel incorporation protein HypB"
"JS1_D03_00519" 12428 "s63E6H2" 0.7646395 "Hydrogenase isoenzymes nickel incorporation protein HypB"
"JS1_D03_00520" 12429 "s59E13H2" 0 "Holliday junction resolvase-like protein"
"JS1_D03_00520" 12429 "s59E25H2" 0 "Holliday junction resolvase-like protein"
"JS1_D03_00520" 12429 "s59E5H2" 0.03956294 "Holliday junction resolvase-like protein"
"JS1_D03_00520" 12429 "s63E6H2" 0.1786217 "Holliday junction resolvase-like protein"
"JS1_D03_00521" 12430 "s59E13H2" 0 "formate dehydrogenase accessory protein"
"JS1_D03_00521" 12430 "s59E25H2" 0 "formate dehydrogenase accessory protein"
"JS1_D03_00521" 12430 "s59E5H2" 0.000610119 "formate dehydrogenase accessory protein"
"JS1_D03_00521" 12430 "s63E6H2" 0.4905513 "formate dehydrogenase accessory protein"
"JS1_D03_00522" 12431 "s59E13H2" 0 "putative molybdenum cofactor guanylyltransferase"
"JS1_D03_00522" 12431 "s59E25H2" 0 "putative molybdenum cofactor guanylyltransferase"
"JS1_D03_00522" 12431 "s59E5H2" 0 "putative molybdenum cofactor guanylyltransferase"
"JS1_D03_00522" 12431 "s63E6H2" 0.5518549 "putative molybdenum cofactor guanylyltransferase"
"JS1_D03_00523" 12432 "s59E13H2" 0 "Formate dehydrogenase subunit alpha"
"JS1_D03_00523" 12432 "s59E25H2" 0 "Formate dehydrogenase subunit alpha"
"JS1_D03_00523" 12432 "s59E5H2" 0.01760474 "Formate dehydrogenase subunit alpha"
"JS1_D03_00523" 12432 "s63E6H2" 0.09832913 "Formate dehydrogenase subunit alpha"
"JS1_D03_00524" 12433 "s59E13H2" 0 "NADP-reducing hydrogenase subunit HndC"
"JS1_D03_00524" 12433 "s59E25H2" 0 "NADP-reducing hydrogenase subunit HndC"
"JS1_D03_00524" 12433 "s59E5H2" 0 "NADP-reducing hydrogenase subunit HndC"
"JS1_D03_00524" 12433 "s63E6H2" 0.0604227 "NADP-reducing hydrogenase subunit HndC"
"JS1_D03_00525" 12434 "s59E13H2" 0 "NADP-reducing hydrogenase subunit HndA"
"JS1_D03_00525" 12434 "s59E25H2" 0 "NADP-reducing hydrogenase subunit HndA"
"JS1_D03_00525" 12434 "s59E5H2" 0 "NADP-reducing hydrogenase subunit HndA"
"JS1_D03_00525" 12434 "s63E6H2" 0.1192523 "NADP-reducing hydrogenase subunit HndA"
"JS1_D03_00526" 12435 "s59E13H2" 0 "NADH-quinone oxidoreductase subunit I"
"JS1_D03_00526" 12435 "s59E25H2" 0 "NADH-quinone oxidoreductase subunit I"
"JS1_D03_00526" 12435 "s59E5H2" 0.1910876 "NADH-quinone oxidoreductase subunit I"
"JS1_D03_00526" 12435 "s63E6H2" 0.3879279 "NADH-quinone oxidoreductase subunit I"
"JS1_D03_00527" 12436 "s59E13H2" 0 "Na(+)/H(+) antiporter subunit G"
"JS1_D03_00527" 12436 "s59E25H2" 0 "Na(+)/H(+) antiporter subunit G"
"JS1_D03_00527" 12436 "s59E5H2" 0.2759854 "Na(+)/H(+) antiporter subunit G"
"JS1_D03_00527" 12436 "s63E6H2" 0.3463567 "Na(+)/H(+) antiporter subunit G"
"JS1_D03_00528" 12437 "s59E13H2" 0 "Na(+)/H(+) antiporter subunit F"
"JS1_D03_00528" 12437 "s59E25H2" 0 "Na(+)/H(+) antiporter subunit F"
"JS1_D03_00528" 12437 "s59E5H2" 0.3099121 "Na(+)/H(+) antiporter subunit F"
"JS1_D03_00528" 12437 "s63E6H2" 0.2851829 "Na(+)/H(+) antiporter subunit F"
"JS1_D03_00529" 12438 "s59E13H2" 0 "Na(+)/H(+) antiporter subunit E"
"JS1_D03_00529" 12438 "s59E25H2" 0 "Na(+)/H(+) antiporter subunit E"
"JS1_D03_00529" 12438 "s59E5H2" 0.1071016 "Na(+)/H(+) antiporter subunit E"

"JS1_D03_00529" 12438 "s63E6H2" 0.807752 "Na(+)/H(+) antiporter subunit E"
"JS1_D03_00530" 12439 "s59E13H2" 0.001159265 "Na(+)/H(+) antiporter subunit D"
"JS1_D03_00530" 12439 "s59E25H2" 0.5285525 "Na(+)/H(+) antiporter subunit D"
"JS1_D03_00530" 12439 "s59E5H2" 0.3237825 "Na(+)/H(+) antiporter subunit D"
"JS1_D03_00530" 12439 "s63E6H2" 5.321667 "Na(+)/H(+) antiporter subunit D"
"JS1_D03_00531" 12440 "s59E13H2" 0.003405983 "Na(+)/H(+) antiporter subunit C"
"JS1_D03_00531" 12440 "s59E25H2" 0.5870206 "Na(+)/H(+) antiporter subunit C"
"JS1_D03_00531" 12440 "s59E5H2" 0.3998416 "Na(+)/H(+) antiporter subunit C"
"JS1_D03_00531" 12440 "s63E6H2" 5.888572 "Na(+)/H(+) antiporter subunit C"
"JS1_D03_00532" 12441 "s59E13H2" 0.001305133 "Na(+)/H(+) antiporter subunit B"
"JS1_D03_00532" 12441 "s59E25H2" 0 "Na(+)/H(+) antiporter subunit B"
"JS1_D03_00532" 12441 "s59E5H2" 0.4118782 "Na(+)/H(+) antiporter subunit B"
"JS1_D03_00532" 12441 "s63E6H2" 5.791349 "Na(+)/H(+) antiporter subunit B"
"JS1_D03_00533" 12442 "s59E13H2" 0 "Na(+)/H(+) antiporter subunit A"
"JS1_D03_00533" 12442 "s59E25H2" 0 "Na(+)/H(+) antiporter subunit A"
"JS1_D03_00533" 12442 "s59E5H2" 0.3289594 "Na(+)/H(+) antiporter subunit A"
"JS1_D03_00533" 12442 "s63E6H2" 5.371578 "Na(+)/H(+) antiporter subunit A"
"JS1_D03_00534" 12443 "s59E13H2" 0 "Na(+)/H(+) antiporter subunit A"
"JS1_D03_00534" 12443 "s59E25H2" 0 "Na(+)/H(+) antiporter subunit A"
"JS1_D03_00534" 12443 "s59E5H2" 0.3848892 "Na(+)/H(+) antiporter subunit A"
"JS1_D03_00534" 12443 "s63E6H2" 6.001546 "Na(+)/H(+) antiporter subunit A"
"JS1_D03_00535" 12444 "s59E13H2" 0 "Membrane-bound hydrogenase subunit alpha"
"JS1_D03_00535" 12444 "s59E25H2" 0 "Membrane-bound hydrogenase subunit alpha"
"JS1_D03_00535" 12444 "s59E5H2" 0.8517795 "Membrane-bound hydrogenase subunit alpha"
"JS1_D03_00535" 12444 "s63E6H2" 8.110249 "Membrane-bound hydrogenase subunit alpha"
"JS1_D03_00536" 12445 "s59E13H2" 0 "F(420)H(2) dehydrogenase subunit C"
"JS1_D03_00536" 12445 "s59E25H2" 0 "F(420)H(2) dehydrogenase subunit C"
"JS1_D03_00536" 12445 "s59E5H2" 0.8750231 "F(420)H(2) dehydrogenase subunit C"
"JS1_D03_00536" 12445 "s63E6H2" 6.382331 "F(420)H(2) dehydrogenase subunit C"
"JS1_D03_00537" 12446 "s59E13H2" 0.00732336 "putative membrane-bound hydrogenase subunit mbhJ"
"JS1_D03_00537" 12446 "s59E25H2" 0 "putative membrane-bound hydrogenase subunit mbhJ"
"JS1_D03_00537" 12446 "s59E5H2" 0.5173965 "putative membrane-bound hydrogenase subunit mbhJ"
"JS1_D03_00537" 12446 "s63E6H2" 6.188186 "putative membrane-bound hydrogenase subunit mbhJ"
"JS1_D03_00538" 12447 "s59E13H2" 0.0002860525 "F(420)H(2) dehydrogenase subunit H"
"JS1_D03_00538" 12447 "s59E25H2" 0 "F(420)H(2) dehydrogenase subunit H"
"JS1_D03_00538" 12447 "s59E5H2" 0.3943802 "F(420)H(2) dehydrogenase subunit H"
"JS1_D03_00538" 12447 "s63E6H2" 5.886108 "F(420)H(2) dehydrogenase subunit H"
"JS1_D03_00539" 12448 "s59E13H2" 0.001310474 "Anaerobic dimethyl sulfoxide reductase chain B"
"JS1_D03_00539" 12448 "s59E25H2" 0 "Anaerobic dimethyl sulfoxide reductase chain B"
"JS1_D03_00539" 12448 "s59E5H2" 1.03852 "Anaerobic dimethyl sulfoxide reductase chain B"
"JS1_D03_00539" 12448 "s63E6H2" 6.972053 "Anaerobic dimethyl sulfoxide reductase chain B"
"JS1_D03_00540" 12449 "s59E13H2" 0.001656093 "Anaerobic sulfite reductase subunit B"
"JS1_D03_00540" 12449 "s59E25H2" 0 "Anaerobic sulfite reductase subunit B"
"JS1_D03_00540" 12449 "s59E5H2" 0.9129415 "Anaerobic sulfite reductase subunit B"
"JS1_D03_00540" 12449 "s63E6H2" 4.24006 "Anaerobic sulfite reductase subunit B"
"JS1_D03_00541" 12450 "s59E13H2" 0 "Sulfhydrogenase 1 subunit gamma"
"JS1_D03_00541" 12450 "s59E25H2" 0 "Sulfhydrogenase 1 subunit gamma"

"JS1_D03_00541" 12450 "s59E5H2" 0.1653597 "Sulfhydrogenase 1 subunit gamma"
"JS1_D03_00541" 12450 "s63E6H2" 0.442024 "Sulfhydrogenase 1 subunit gamma"
"JS1_D03_00542" 12451 "s59E13H2" 0 "Sulfhydrogenase 2 subunit beta"
"JS1_D03_00542" 12451 "s59E25H2" 0 "Sulfhydrogenase 2 subunit beta"
"JS1_D03_00542" 12451 "s59E5H2" 0.1872092 "Sulfhydrogenase 2 subunit beta"
"JS1_D03_00542" 12451 "s63E6H2" 0.8689059 "Sulfhydrogenase 2 subunit beta"
"JS1_D03_00543" 12452 "s59E13H2" 0.1356666 "Redox-sensing transcriptional repressor Rex"
"JS1_D03_00543" 12452 "s59E25H2" 0 "Redox-sensing transcriptional repressor Rex"
"JS1_D03_00543" 12452 "s59E5H2" 0.5535978 "Redox-sensing transcriptional repressor Rex"
"JS1_D03_00543" 12452 "s63E6H2" 1.063734 "Redox-sensing transcriptional repressor Rex"
"JS1_D03_00544" 12453 "s59E13H2" 0.1280488 "Sporulation-specific N-acetylmuramoyl-L-alanine amidase"
"JS1_D03_00544" 12453 "s59E25H2" 0 "Sporulation-specific N-acetylmuramoyl-L-alanine amidase"
"JS1_D03_00544" 12453 "s59E5H2" 0.3377307 "Sporulation-specific N-acetylmuramoyl-L-alanine amidase"
"JS1_D03_00544" 12453 "s63E6H2" 0.3795904 "Sporulation-specific N-acetylmuramoyl-L-alanine amidase"
"JS1_D03_00545" 12454 "s59E13H2" 0.0696023 "Hydrogenase 3 maturation protease"
"JS1_D03_00545" 12454 "s59E25H2" 0 "Hydrogenase 3 maturation protease"
"JS1_D03_00545" 12454 "s59E5H2" 1.474151 "Hydrogenase 3 maturation protease"
"JS1_D03_00545" 12454 "s63E6H2" 0.6028088 "Hydrogenase 3 maturation protease"
"JS1_D03_00546" 12455 "s59E13H2" 0.2849717 "NAD(P)H-quinone oxidoreductase subunit I"
"JS1_D03_00546" 12455 "s59E25H2" 0 "NAD(P)H-quinone oxidoreductase subunit I"
"JS1_D03_00546" 12455 "s59E5H2" 1.140628 "NAD(P)H-quinone oxidoreductase subunit I"
"JS1_D03_00546" 12455 "s63E6H2" 5.120486 "NAD(P)H-quinone oxidoreductase subunit I"
"JS1_D03_00547" 12456 "s59E13H2" 0.6812834 "Hydrogenase-4 component C"
"JS1_D03_00547" 12456 "s59E25H2" 0 "Hydrogenase-4 component C"
"JS1_D03_00547" 12456 "s59E5H2" 1.09903 "Hydrogenase-4 component C"
"JS1_D03_00547" 12456 "s63E6H2" 4.34386 "Hydrogenase-4 component C"
"JS1_D03_00548" 12457 "s59E13H2" 0.3126778 "Membrane-bound hydrogenase subunit alpha"
"JS1_D03_00548" 12457 "s59E25H2" 0 "Membrane-bound hydrogenase subunit alpha"
"JS1_D03_00548" 12457 "s59E5H2" 0.5054246 "Membrane-bound hydrogenase subunit alpha"
"JS1_D03_00548" 12457 "s63E6H2" 2.66849 "Membrane-bound hydrogenase subunit alpha"
"JS1_D03_00549" 12458 "s59E13H2" 0.006918595 "Membrane-bound hydrogenase subunit beta"
"JS1_D03_00549" 12458 "s59E25H2" 0 "Membrane-bound hydrogenase subunit beta"
"JS1_D03_00549" 12458 "s59E5H2" 0.02092578 "Membrane-bound hydrogenase subunit beta"
"JS1_D03_00549" 12458 "s63E6H2" 0.2033759 "Membrane-bound hydrogenase subunit beta"
"JS1_D03_00550" 12459 "s59E13H2" 0.2323905 "putative membrane-bound hydrogenase subunit mbhJ"
"JS1_D03_00550" 12459 "s59E25H2" 0 "putative membrane-bound hydrogenase subunit mbhJ"
"JS1_D03_00550" 12459 "s59E5H2" 0.5783974 "putative membrane-bound hydrogenase subunit mbhJ"
"JS1_D03_00550" 12459 "s63E6H2" 1.408221 "putative membrane-bound hydrogenase subunit mbhJ"
"JS1_D03_00551" 12460 "s59E13H2" 0.467245 "hypothetical protein"
"JS1_D03_00551" 12460 "s59E25H2" 0 "hypothetical protein"
"JS1_D03_00551" 12460 "s59E5H2" 1.269018 "hypothetical protein"
"JS1_D03_00551" 12460 "s63E6H2" 4.374902 "hypothetical protein"
"JS1_D03_00552" 12461 "s59E13H2" 0.1989806 "Na(+)/H(+) antiporter subunit A"
"JS1_D03_00552" 12461 "s59E25H2" 0 "Na(+)/H(+) antiporter subunit A"
"JS1_D03_00552" 12461 "s59E5H2" 1.185278 "Na(+)/H(+) antiporter subunit A"
"JS1_D03_00552" 12461 "s63E6H2" 4.459237 "Na(+)/H(+) antiporter subunit A"
"JS1_D03_00553" 12462 "s59E13H2" 0.5394582 "Na(+)/H(+) antiporter subunit C1"

"JS1_D03_00553" 12462 "s59E25H2" 0 "Na(+)/H(+) antiporter subunit C1"
"JS1_D03_00553" 12462 "s59E5H2" 1.664777 "Na(+)/H(+) antiporter subunit C1"
"JS1_D03_00553" 12462 "s63E6H2" 3.836032 "Na(+)/H(+) antiporter subunit C1"
"JS1_D03_00554" 12463 "s59E13H2" 0.7544558 "Na(+)/H(+) antiporter subunit B"
"JS1_D03_00554" 12463 "s59E25H2" 0 "Na(+)/H(+) antiporter subunit B"
"JS1_D03_00554" 12463 "s59E5H2" 0.7419332 "Na(+)/H(+) antiporter subunit B"
"JS1_D03_00554" 12463 "s63E6H2" 2.561535 "Na(+)/H(+) antiporter subunit B"
"JS1_D03_00555" 12464 "s59E13H2" 1.399299 "Na(+)/H(+) antiporter subunit A"
"JS1_D03_00555" 12464 "s59E25H2" 0 "Na(+)/H(+) antiporter subunit A"
"JS1_D03_00555" 12464 "s59E5H2" 0.8508427 "Na(+)/H(+) antiporter subunit A"
"JS1_D03_00555" 12464 "s63E6H2" 4.550109 "Na(+)/H(+) antiporter subunit A"
"JS1_D03_00556" 12465 "s59E13H2" 0.5631422 "hypothetical protein"
"JS1_D03_00556" 12465 "s59E25H2" 0 "hypothetical protein"
"JS1_D03_00556" 12465 "s59E5H2" 0.8541557 "hypothetical protein"
"JS1_D03_00556" 12465 "s63E6H2" 1.97772 "hypothetical protein"
"JS1_D03_00557" 12466 "s59E13H2" 0.1911926 "Na(+)/H(+) antiporter subunit G"
"JS1_D03_00557" 12466 "s59E25H2" 0 "Na(+)/H(+) antiporter subunit G"
"JS1_D03_00557" 12466 "s59E5H2" 1.139153 "Na(+)/H(+) antiporter subunit G"
"JS1_D03_00557" 12466 "s63E6H2" 0.6736958 "Na(+)/H(+) antiporter subunit G"
"JS1_D03_00558" 12467 "s59E13H2" 1.049632 "Na(+)/H(+) antiporter subunit F"
"JS1_D03_00558" 12467 "s59E25H2" 0 "Na(+)/H(+) antiporter subunit F"
"JS1_D03_00558" 12467 "s59E5H2" 1.237139 "Na(+)/H(+) antiporter subunit F"
"JS1_D03_00558" 12467 "s63E6H2" 2.574835 "Na(+)/H(+) antiporter subunit F"
"JS1_D03_00559" 12468 "s59E13H2" 1.257626 "Na(+)/H(+) antiporter subunit E"
"JS1_D03_00559" 12468 "s59E25H2" 0 "Na(+)/H(+) antiporter subunit E"
"JS1_D03_00559" 12468 "s59E5H2" 1.190195 "Na(+)/H(+) antiporter subunit E"
"JS1_D03_00559" 12468 "s63E6H2" 3.242645 "Na(+)/H(+) antiporter subunit E"
"JS1_D03_00560" 12469 "s59E13H2" 0.09926973 "putative hydrogenase nickel incorporation protein HypA"
"JS1_D03_00560" 12469 "s59E25H2" 0 "putative hydrogenase nickel incorporation protein HypA"
"JS1_D03_00560" 12469 "s59E5H2" 0.2328422 "putative hydrogenase nickel incorporation protein HypA"
"JS1_D03_00560" 12469 "s63E6H2" 0.4354691 "putative hydrogenase nickel incorporation protein HypA"
"JS1_D03_00562" 12471 "s59E13H2" 2.050181 "Oligopeptide transport ATP-binding protein OppF"
"JS1_D03_00562" 12471 "s59E25H2" 0.2317949 "Oligopeptide transport ATP-binding protein OppF"
"JS1_D03_00562" 12471 "s59E5H2" 1.542526 "Oligopeptide transport ATP-binding protein OppF"
"JS1_D03_00562" 12471 "s63E6H2" 4.846509 "Oligopeptide transport ATP-binding protein OppF"
"JS1_D03_00563" 12472 "s59E13H2" 1.837081 "Oligopeptide transport ATP-binding protein OppD"
"JS1_D03_00563" 12472 "s59E25H2" 0.3867833 "Oligopeptide transport ATP-binding protein OppD"
"JS1_D03_00563" 12472 "s59E5H2" 1.195493 "Oligopeptide transport ATP-binding protein OppD"
"JS1_D03_00563" 12472 "s63E6H2" 2.647374 "Oligopeptide transport ATP-binding protein OppD"
"JS1_D03_00564" 12473 "s59E13H2" 0.2636156 "Putative nicotinate phosphoribosyltransferase"
"JS1_D03_00564" 12473 "s59E25H2" 0.2909441 "Putative nicotinate phosphoribosyltransferase"
"JS1_D03_00564" 12473 "s59E5H2" 0.2349296 "Putative nicotinate phosphoribosyltransferase"
"JS1_D03_00564" 12473 "s63E6H2" 0.26237 "Putative nicotinate phosphoribosyltransferase"
"JS1_D03_00565" 12474 "s59E13H2" 0.7892897 "Protein-export membrane protein SecG"
"JS1_D03_00565" 12474 "s59E25H2" 0 "Protein-export membrane protein SecG"
"JS1_D03_00565" 12474 "s59E5H2" 0.2014578 "Protein-export membrane protein SecG"
"JS1_D03_00565" 12474 "s63E6H2" 0.5561874 "Protein-export membrane protein SecG"

"JS1_D03_00566" 12475 "s59E13H2" 0.9225836 "Triosephosphate isomerase"
"JS1_D03_00566" 12475 "s59E25H2" 0 "Triosephosphate isomerase"
"JS1_D03_00566" 12475 "s59E5H2" 0.3140651 "Triosephosphate isomerase"
"JS1_D03_00566" 12475 "s63E6H2" 0.8235842 "Triosephosphate isomerase"
"JS1_D03_00567" 12476 "s59E13H2" 0.05018744 "hypothetical protein"
"JS1_D03_00567" 12476 "s59E25H2" 0 "hypothetical protein"
"JS1_D03_00567" 12476 "s59E5H2" 0.008080376 "hypothetical protein"
"JS1_D03_00567" 12476 "s63E6H2" 0 "hypothetical protein"
"JS1_D03_00568" 12477 "s59E13H2" 0.04245356 "4-hydroxythreonine-4-phosphate dehydrogenase 2"
"JS1_D03_00568" 12477 "s59E25H2" 0 "4-hydroxythreonine-4-phosphate dehydrogenase 2"
"JS1_D03_00568" 12477 "s59E5H2" 0.003848939 "4-hydroxythreonine-4-phosphate dehydrogenase 2"
"JS1_D03_00568" 12477 "s63E6H2" 0 "4-hydroxythreonine-4-phosphate dehydrogenase 2"
"JS1_D03_00569" 12478 "s59E13H2" 0.03872325 "hypothetical protein"
"JS1_D03_00569" 12478 "s59E25H2" 0 "hypothetical protein"
"JS1_D03_00569" 12478 "s59E5H2" 0.003161729 "hypothetical protein"
"JS1_D03_00569" 12478 "s63E6H2" 0 "hypothetical protein"
"JS1_D03_00570" 12479 "s59E13H2" 0.08330632 "Sialic acid TRAP transporter permease protein SiaT"
"JS1_D03_00570" 12479 "s59E25H2" 0 "Sialic acid TRAP transporter permease protein SiaT"
"JS1_D03_00570" 12479 "s59E5H2" 0.3085548 "Sialic acid TRAP transporter permease protein SiaT"
"JS1_D03_00570" 12479 "s63E6H2" 0.03896911 "Sialic acid TRAP transporter permease protein SiaT"
"JS1_D03_00571" 12480 "s59E13H2" 0.1485045 "Tripartite ATP-independent periplasmic transporters, DctQ component"
"JS1_D03_00571" 12480 "s59E25H2" 0 "Tripartite ATP-independent periplasmic transporters, DctQ component"
"JS1_D03_00571" 12480 "s59E5H2" 0.593724 "Tripartite ATP-independent periplasmic transporters, DctQ component"
"JS1_D03_00571" 12480 "s63E6H2" 0.05627948 "Tripartite ATP-independent periplasmic transporters, DctQ component"
"JS1_D03_00572" 12481 "s59E13H2" 0.1236238 "Monocarboxylate 2-oxoacid-binding periplasmic protein precursor"
"JS1_D03_00572" 12481 "s59E25H2" 0 "Monocarboxylate 2-oxoacid-binding periplasmic protein precursor"
"JS1_D03_00572" 12481 "s59E5H2" 0.3284083 "Monocarboxylate 2-oxoacid-binding periplasmic protein precursor"
"JS1_D03_00572" 12481 "s63E6H2" 0.01602456 "Monocarboxylate 2-oxoacid-binding periplasmic protein precursor"
"JS1_D03_00573" 12482 "s59E13H2" 0 "Glycerol kinase"
"JS1_D03_00573" 12482 "s59E25H2" 0 "Glycerol kinase"
"JS1_D03_00573" 12482 "s59E5H2" 0 "Glycerol kinase"
"JS1_D03_00573" 12482 "s63E6H2" 0 "Glycerol kinase"
"JS1_D03_00574" 12483 "s59E13H2" 0 "Fructose-bisphosphate aldolase"
"JS1_D03_00574" 12483 "s59E25H2" 0 "Fructose-bisphosphate aldolase"
"JS1_D03_00574" 12483 "s59E5H2" 0 "Fructose-bisphosphate aldolase"
"JS1_D03_00574" 12483 "s63E6H2" 0 "Fructose-bisphosphate aldolase"
"JS1_D03_00575" 12484 "s59E13H2" 0 "Putative 2-hydroxyacid dehydrogenase YoaD"
"JS1_D03_00575" 12484 "s59E25H2" 0 "Putative 2-hydroxyacid dehydrogenase YoaD"
"JS1_D03_00575" 12484 "s59E5H2" 0 "Putative 2-hydroxyacid dehydrogenase YoaD"
"JS1_D03_00575" 12484 "s63E6H2" 0 "Putative 2-hydroxyacid dehydrogenase YoaD"
"JS1_D03_00576" 12485 "s59E13H2" 0 "HTH-type transcriptional regulator Mce2R"
"JS1_D03_00576" 12485 "s59E25H2" 0 "HTH-type transcriptional regulator Mce2R"
"JS1_D03_00576" 12485 "s59E5H2" 0 "HTH-type transcriptional regulator Mce2R"
"JS1_D03_00576" 12485 "s63E6H2" 0 "HTH-type transcriptional regulator Mce2R"
"JS1_D03_00577" 12486 "s59E13H2" 0.09988275 "NifU-like protein"
"JS1_D03_00577" 12486 "s59E25H2" 0 "NifU-like protein"
"JS1_D03_00577" 12486 "s59E5H2" 0.2104976 "NifU-like protein"

"JS1_D03_00577" 12486 "s63E6H2" 0.6058261 "NifU-like protein"
"JS1_D03_00578" 12487 "s59E13H2" 0.1221806 "putative cysteine desulfurase 2"
"JS1_D03_00578" 12487 "s59E25H2" 0 "putative cysteine desulfurase 2"
"JS1_D03_00578" 12487 "s59E5H2" 0.5494115 "putative cysteine desulfurase 2"
"JS1_D03_00578" 12487 "s63E6H2" 0.8173423 "putative cysteine desulfurase 2"
"JS1_D03_00579" 12488 "s59E13H2" 0.04471452 "hypothetical protein"
"JS1_D03_00579" 12488 "s59E25H2" 0 "hypothetical protein"
"JS1_D03_00579" 12488 "s59E5H2" 0.2799226 "hypothetical protein"
"JS1_D03_00579" 12488 "s63E6H2" 0.4245371 "hypothetical protein"
"JS1_D03_00580" 12489 "s59E13H2" 0.01272059 "hypothetical protein"
"JS1_D03_00580" 12489 "s59E25H2" 0 "hypothetical protein"
"JS1_D03_00580" 12489 "s59E5H2" 0.1587581 "hypothetical protein"
"JS1_D03_00580" 12489 "s63E6H2" 0.3565969 "hypothetical protein"
"JS1_D03_00582" 12491 "s59E13H2" 0.01673909 "hypothetical protein"
"JS1_D03_00582" 12491 "s59E25H2" 0 "hypothetical protein"
"JS1_D03_00582" 12491 "s59E5H2" 2.420277 "hypothetical protein"
"JS1_D03_00582" 12491 "s63E6H2" 4.477312 "hypothetical protein"
"JS1_D03_00583" 12492 "s59E13H2" 9.120864 "Transposase IS116/IS110/IS902 family protein"
"JS1_D03_00583" 12492 "s59E25H2" 0 "Transposase IS116/IS110/IS902 family protein"
"JS1_D03_00583" 12492 "s59E5H2" 0.4363725 "Transposase IS116/IS110/IS902 family protein"
"JS1_D03_00583" 12492 "s63E6H2" 2.54576 "Transposase IS116/IS110/IS902 family protein"
"JS1_D03_00585" 12494 "s59E13H2" 0.4804024 "Fic/DOC family protein"
"JS1_D03_00585" 12494 "s59E25H2" 0 "Fic/DOC family protein"
"JS1_D03_00585" 12494 "s59E5H2" 0.2536543 "Fic/DOC family protein"
"JS1_D03_00585" 12494 "s63E6H2" 0.21128 "Fic/DOC family protein"
"JS1_D03_00586" 12495 "s59E13H2" 0 "Vitamin B12 dependent methionine synthase, activation domain"
"JS1_D03_00586" 12495 "s59E25H2" 0 "Vitamin B12 dependent methionine synthase, activation domain"
"JS1_D03_00586" 12495 "s59E5H2" 0 "Vitamin B12 dependent methionine synthase, activation domain"
"JS1_D03_00586" 12495 "s63E6H2" 0.1640696 "Vitamin B12 dependent methionine synthase, activation domain"
"JS1_D03_00587" 12496 "s59E13H2" 0 "2Fe-2S ferredoxin"
"JS1_D03_00587" 12496 "s59E25H2" 0 "2Fe-2S ferredoxin"
"JS1_D03_00587" 12496 "s59E5H2" 0 "2Fe-2S ferredoxin"
"JS1_D03_00587" 12496 "s63E6H2" 0.2753403 "2Fe-2S ferredoxin"
"JS1_D03_00588" 12497 "s59E13H2" 1.081906 "Bifunctional protein FolD protein"
"JS1_D03_00588" 12497 "s59E25H2" 0 "Bifunctional protein FolD protein"
"JS1_D03_00588" 12497 "s59E5H2" 1.278524 "Bifunctional protein FolD protein"
"JS1_D03_00588" 12497 "s63E6H2" 0.3484047 "Bifunctional protein FolD protein"
"JS1_D03_00589" 12498 "s59E13H2" 2.292659 "5-methyltetrahydrofolate:corrinoid/iron-sulfur protein co-methyltransferase"
"JS1_D03_00589" 12498 "s59E25H2" 0 "5-methyltetrahydrofolate:corrinoid/iron-sulfur protein co-methyltransferase"
"JS1_D03_00589" 12498 "s59E5H2" 0.1312492 "5-methyltetrahydrofolate:corrinoid/iron-sulfur protein co-methyltransferase"
"JS1_D03_00589" 12498 "s63E6H2" 0.3322672 "5-methyltetrahydrofolate:corrinoid/iron-sulfur protein co-methyltransferase"
"JS1_D03_00590" 12499 "s59E13H2" 2.99514 "Acryloyl-CoA reductase electron transfer subunit beta"
"JS1_D03_00590" 12499 "s59E25H2" 0 "Acryloyl-CoA reductase electron transfer subunit beta"
"JS1_D03_00590" 12499 "s59E5H2" 0.1409785 "Acryloyl-CoA reductase electron transfer subunit beta"
"JS1_D03_00590" 12499 "s63E6H2" 0.907328 "Acryloyl-CoA reductase electron transfer subunit beta"
"JS1_D03_00591" 12500 "s59E13H2" 3.196787 "Acryloyl-CoA reductase electron transfer subunit gamma"
"JS1_D03_00591" 12500 "s59E25H2" 0 "Acryloyl-CoA reductase electron transfer subunit gamma"

"JS1_D03_00591" 12500 "s59E5H2" 0.2433609 "Acryloyl-CoA reductase electron transfer subunit gamma"
"JS1_D03_00591" 12500 "s63E6H2" 1.115954 "Acryloyl-CoA reductase electron transfer subunit gamma"
"JS1_D03_00592" 12501 "s59E13H2" 3.230071 "bifunctional homocysteine S-methyltransferase/5,10-methylenetetrahydrofolate reductase protein"
"JS1_D03_00592" 12501 "s59E25H2" 0 "bifunctional homocysteine S-methyltransferase/5,10-methylenetetrahydrofolate reductase protein"
"JS1_D03_00592" 12501 "s59E5H2" 0.2574171 "bifunctional homocysteine S-methyltransferase/5,10-methylenetetrahydrofolate reductase protein"
"JS1_D03_00592" 12501 "s63E6H2" 0.885786 "bifunctional homocysteine S-methyltransferase/5,10-methylenetetrahydrofolate reductase protein"
"JS1_D03_00593" 12502 "s59E13H2" 2.069558 "Trimethylamine corrinoid protein"
"JS1_D03_00593" 12502 "s59E25H2" 0 "Trimethylamine corrinoid protein"
"JS1_D03_00593" 12502 "s59E5H2" 0.3205979 "Trimethylamine corrinoid protein"
"JS1_D03_00593" 12502 "s63E6H2" 0.3960374 "Trimethylamine corrinoid protein"
"JS1_D03_00594" 12503 "s59E13H2" 0 "N-carbamoyl-D-amino acid hydrolase"
"JS1_D03_00594" 12503 "s59E25H2" 0 "N-carbamoyl-D-amino acid hydrolase"
"JS1_D03_00594" 12503 "s59E5H2" 0.4053502 "N-carbamoyl-D-amino acid hydrolase"
"JS1_D03_00594" 12503 "s63E6H2" 0.04416195 "N-carbamoyl-D-amino acid hydrolase"
"JS1_D03_00595" 12504 "s59E13H2" 0 "Trimethylamine methyltransferase MttB"
"JS1_D03_00595" 12504 "s59E25H2" 0 "Trimethylamine methyltransferase MttB"
"JS1_D03_00595" 12504 "s59E5H2" 0.5234745 "Trimethylamine methyltransferase MttB"
"JS1_D03_00595" 12504 "s63E6H2" 0.06894477 "Trimethylamine methyltransferase MttB"
"JS1_D03_00596" 12505 "s59E13H2" 0.4567973 "Putative osmoprotectant uptake system substrate-binding protein OsmF precursor"
"JS1_D03_00596" 12505 "s59E25H2" 0 "Putative osmoprotectant uptake system substrate-binding protein OsmF precursor"
"JS1_D03_00596" 12505 "s59E5H2" 0.7467189 "Putative osmoprotectant uptake system substrate-binding protein OsmF precursor"
"JS1_D03_00596" 12505 "s63E6H2" 0.1024027 "Putative osmoprotectant uptake system substrate-binding protein OsmF precursor"
"JS1_D03_00597" 12506 "s59E13H2" 0.4524463 "Glycine betaine/carnitine/choline transport system permease protein OpuCB"
"JS1_D03_00597" 12506 "s59E25H2" 0 "Glycine betaine/carnitine/choline transport system permease protein OpuCB"
"JS1_D03_00597" 12506 "s59E5H2" 0.6814432 "Glycine betaine/carnitine/choline transport system permease protein OpuCB"
"JS1_D03_00597" 12506 "s63E6H2" 0.07962181 "Glycine betaine/carnitine/choline transport system permease protein OpuCB"
"JS1_D03_00598" 12507 "s59E13H2" 0.2237925 "Carnitine transport ATP-binding protein OpuCA"
"JS1_D03_00598" 12507 "s59E25H2" 0 "Carnitine transport ATP-binding protein OpuCA"
"JS1_D03_00598" 12507 "s59E5H2" 0.6929511 "Carnitine transport ATP-binding protein OpuCA"
"JS1_D03_00598" 12507 "s63E6H2" 0.1147194 "Carnitine transport ATP-binding protein OpuCA"
"JS1_D03_00599" 12508 "s59E13H2" 0.3308093 "Carnitine transport permease protein OpuCB"
"JS1_D03_00599" 12508 "s59E25H2" 0 "Carnitine transport permease protein OpuCB"
"JS1_D03_00599" 12508 "s59E5H2" 1.032647 "Carnitine transport permease protein OpuCB"
"JS1_D03_00599" 12508 "s63E6H2" 0.1329657 "Carnitine transport permease protein OpuCB"
"JS1_D03_00600" 12509 "s59E13H2" 0.07186962 "Trimethylamine methyltransferase MttB"
"JS1_D03_00600" 12509 "s59E25H2" 0 "Trimethylamine methyltransferase MttB"
"JS1_D03_00600" 12509 "s59E5H2" 1.178786 "Trimethylamine methyltransferase MttB"
"JS1_D03_00600" 12509 "s63E6H2" 0.3165165 "Trimethylamine methyltransferase MttB"
"JS1_D03_00601" 12510 "s59E13H2" 0.04538214 "HTH-type transcriptional regulator YesS"
"JS1_D03_00601" 12510 "s59E25H2" 0 "HTH-type transcriptional regulator YesS"
"JS1_D03_00601" 12510 "s59E5H2" 1.365344 "HTH-type transcriptional regulator YesS"
"JS1_D03_00601" 12510 "s63E6H2" 0.1553928 "HTH-type transcriptional regulator YesS"
"JS1_D03_00602" 12511 "s59E13H2" 2.219084 "DNA polymerase/3'-5' exonuclease PolX"
"JS1_D03_00602" 12511 "s59E25H2" 0 "DNA polymerase/3'-5' exonuclease PolX"
"JS1_D03_00602" 12511 "s59E5H2" 0.4826333 "DNA polymerase/3'-5' exonuclease PolX"
"JS1_D03_00602" 12511 "s63E6H2" 0.1569106 "DNA polymerase/3'-5' exonuclease PolX"
"JS1_D03_00603" 12512 "s59E13H2" 0.1577666 "Uracil DNA glycosylase superfamily protein"

"JS1_D03_00603" 12512 "s59E25H2" 0 "Uracil DNA glycosylase superfamily protein"
"JS1_D03_00603" 12512 "s59E5H2" 0.4121033 "Uracil DNA glycosylase superfamily protein"
"JS1_D03_00603" 12512 "s63E6H2" 0.18486 "Uracil DNA glycosylase superfamily protein"
"JS1_D03_00604" 12513 "s59E13H2" 0.2521826 "putative protease YhbU precursor"
"JS1_D03_00604" 12513 "s59E25H2" 0 "putative protease YhbU precursor"
"JS1_D03_00604" 12513 "s59E5H2" 0.4819102 "putative protease YhbU precursor"
"JS1_D03_00604" 12513 "s63E6H2" 2.557587 "putative protease YhbU precursor"
"JS1_D03_00605" 12514 "s59E13H2" 0.03512829 "Putative GTP cyclohydrolase 1 type 2"
"JS1_D03_00605" 12514 "s59E25H2" 0 "Putative GTP cyclohydrolase 1 type 2"
"JS1_D03_00605" 12514 "s59E5H2" 0.1501515 "Putative GTP cyclohydrolase 1 type 2"
"JS1_D03_00605" 12514 "s63E6H2" 2.66672 "Putative GTP cyclohydrolase 1 type 2"
"JS1_D03_00606" 12515 "s59E13H2" 0.2906076 "hypothetical protein"
"JS1_D03_00606" 12515 "s59E25H2" 0 "hypothetical protein"
"JS1_D03_00606" 12515 "s59E5H2" 0.3332841 "hypothetical protein"
"JS1_D03_00606" 12515 "s63E6H2" 2.334222 "hypothetical protein"
"JS1_D03_00607" 12516 "s59E13H2" 0.4144065 "Transketolase 2"
"JS1_D03_00607" 12516 "s59E25H2" 0 "Transketolase 2"
"JS1_D03_00607" 12516 "s59E5H2" 0.8294204 "Transketolase 2"
"JS1_D03_00607" 12516 "s63E6H2" 2.903391 "Transketolase 2"
"JS1_D03_00608" 12517 "s59E13H2" 0.2074772 "2-hydroxy-3-oxopropionate reductase"
"JS1_D03_00608" 12517 "s59E25H2" 0 "2-hydroxy-3-oxopropionate reductase"
"JS1_D03_00608" 12517 "s59E5H2" 0.7736721 "2-hydroxy-3-oxopropionate reductase"
"JS1_D03_00608" 12517 "s63E6H2" 0.1774769 "2-hydroxy-3-oxopropionate reductase"
"JS1_D03_00609" 12518 "s59E13H2" 0.1450184 "Xylose isomerase-like TIM barrel"
"JS1_D03_00609" 12518 "s59E25H2" 0 "Xylose isomerase-like TIM barrel"
"JS1_D03_00609" 12518 "s59E5H2" 0.4196801 "Xylose isomerase-like TIM barrel"
"JS1_D03_00609" 12518 "s63E6H2" 1.174618 "Xylose isomerase-like TIM barrel"
"JS1_D03_00610" 12519 "s59E13H2" 0.1044279 "HTH-type transcriptional regulator LutR"
"JS1_D03_00610" 12519 "s59E25H2" 0 "HTH-type transcriptional regulator LutR"
"JS1_D03_00610" 12519 "s59E5H2" 0.1901106 "HTH-type transcriptional regulator LutR"
"JS1_D03_00610" 12519 "s63E6H2" 0.2804688 "HTH-type transcriptional regulator LutR"
"JS1_D03_00611" 12520 "s59E13H2" 0.05663326 "RutC family protein"
"JS1_D03_00611" 12520 "s59E25H2" 0 "RutC family protein"
"JS1_D03_00611" 12520 "s59E5H2" 0.2950368 "RutC family protein"
"JS1_D03_00611" 12520 "s63E6H2" 0.1612504 "RutC family protein"
"JS1_D03_00612" 12521 "s59E13H2" 0.05738494 "Sialic acid TRAP transporter permease protein SiaT"
"JS1_D03_00612" 12521 "s59E25H2" 0 "Sialic acid TRAP transporter permease protein SiaT"
"JS1_D03_00612" 12521 "s59E5H2" 0.2652409 "Sialic acid TRAP transporter permease protein SiaT"
"JS1_D03_00612" 12521 "s63E6H2" 0.2310358 "Sialic acid TRAP transporter permease protein SiaT"
"JS1_D03_00613" 12522 "s59E13H2" 2.056837 "Tripartite ATP-independent periplasmic transporters, DctQ component"
"JS1_D03_00613" 12522 "s59E25H2" 0 "Tripartite ATP-independent periplasmic transporters, DctQ component"
"JS1_D03_00613" 12522 "s59E5H2" 0.1361468 "Tripartite ATP-independent periplasmic transporters, DctQ component"
"JS1_D03_00613" 12522 "s63E6H2" 0.04235238 "Tripartite ATP-independent periplasmic transporters, DctQ component"
"JS1_D03_00614" 12523 "s59E13H2" 4.736347 "Lactate-binding periplasmic protein precursor"
"JS1_D03_00614" 12523 "s59E25H2" 0 "Lactate-binding periplasmic protein precursor"
"JS1_D03_00614" 12523 "s59E5H2" 0.3461755 "Lactate-binding periplasmic protein precursor"
"JS1_D03_00614" 12523 "s63E6H2" 0.1487539 "Lactate-binding periplasmic protein precursor"

"JS1_D03_00615" 12524 "s59E13H2" 5.014173 "hypothetical protein"
"JS1_D03_00615" 12524 "s59E25H2" 0 "hypothetical protein"
"JS1_D03_00615" 12524 "s59E5H2" 0.5425362 "hypothetical protein"
"JS1_D03_00615" 12524 "s63E6H2" 3.776487 "hypothetical protein"
"JS1_D03_00617" 12526 "s59E13H2" 0 "hypothetical protein"
"JS1_D03_00617" 12526 "s59E25H2" 0 "hypothetical protein"
"JS1_D03_00617" 12526 "s59E5H2" 0.06982422 "hypothetical protein"
"JS1_D03_00617" 12526 "s63E6H2" 0.03546252 "hypothetical protein"
"JS1_D03_00618" 12527 "s59E13H2" 0 "hypothetical protein"
"JS1_D03_00618" 12527 "s59E25H2" 0 "hypothetical protein"
"JS1_D03_00618" 12527 "s59E5H2" 0.0520937 "hypothetical protein"
"JS1_D03_00618" 12527 "s63E6H2" 0.02202729 "hypothetical protein"
"JS1_D03_00619" 12528 "s59E13H2" 6.127701 "FMN reductase [NAD(P)H]"
"JS1_D03_00619" 12528 "s59E25H2" 0 "FMN reductase [NAD(P)H]"
"JS1_D03_00619" 12528 "s59E5H2" 0.2384673 "FMN reductase [NAD(P)H]"
"JS1_D03_00619" 12528 "s63E6H2" 0.1992538 "FMN reductase [NAD(P)H]"
"JS1_D03_00620" 12529 "s59E13H2" 0.6958905 "hypothetical protein"
"JS1_D03_00620" 12529 "s59E25H2" 0 "hypothetical protein"
"JS1_D03_00620" 12529 "s59E5H2" 0.03121034 "hypothetical protein"
"JS1_D03_00620" 12529 "s63E6H2" 0.00434235 "hypothetical protein"
"JS1_D03_00621" 12530 "s59E13H2" 0.6517648 "hypothetical protein"
"JS1_D03_00621" 12530 "s59E25H2" 0 "hypothetical protein"
"JS1_D03_00621" 12530 "s59E5H2" 0.1402229 "hypothetical protein"
"JS1_D03_00621" 12530 "s63E6H2" 0.08214278 "hypothetical protein"
"JS1_D03_00622" 12531 "s59E13H2" 5.208092 "pyrroloquinoline quinone biosynthesis protein PqqE"
"JS1_D03_00622" 12531 "s59E25H2" 0.3128964 "pyrroloquinoline quinone biosynthesis protein PqqE"
"JS1_D03_00622" 12531 "s59E5H2" 0.3550815 "pyrroloquinoline quinone biosynthesis protein PqqE"
"JS1_D03_00622" 12531 "s63E6H2" 0.4551456 "pyrroloquinoline quinone biosynthesis protein PqqE"
"JS1_D03_00623" 12532 "s59E13H2" 4.892141 "putative inner membrane transporter yiJE"
"JS1_D03_00623" 12532 "s59E25H2" 0 "putative inner membrane transporter yiJE"
"JS1_D03_00623" 12532 "s59E5H2" 0.1432036 "putative inner membrane transporter yiJE"
"JS1_D03_00623" 12532 "s63E6H2" 0.2759275 "putative inner membrane transporter yiJE"
"JS1_D03_00624" 12533 "s59E13H2" 3.767012 "Radical SAM superfamily protein"
"JS1_D03_00624" 12533 "s59E25H2" 0 "Radical SAM superfamily protein"
"JS1_D03_00624" 12533 "s59E5H2" 0.196447 "Radical SAM superfamily protein"
"JS1_D03_00624" 12533 "s63E6H2" 0.2105651 "Radical SAM superfamily protein"
"JS1_D03_00625" 12534 "s59E13H2" 2.249117 "dUMP phosphatase"
"JS1_D03_00625" 12534 "s59E25H2" 0 "dUMP phosphatase"
"JS1_D03_00625" 12534 "s59E5H2" 0.2356483 "dUMP phosphatase"
"JS1_D03_00625" 12534 "s63E6H2" 0.1064185 "dUMP phosphatase"
"JS1_D03_00626" 12535 "s59E13H2" 0.1866975 "Signal peptidase IB"
"JS1_D03_00626" 12535 "s59E25H2" 0 "Signal peptidase IB"
"JS1_D03_00626" 12535 "s59E5H2" 0.1161696 "Signal peptidase IB"
"JS1_D03_00626" 12535 "s63E6H2" 0.2194613 "Signal peptidase IB"
"JS1_D03_00628" 12537 "s59E13H2" 0 "hypothetical protein"
"JS1_D03_00628" 12537 "s59E25H2" 0 "hypothetical protein"
"JS1_D03_00628" 12537 "s59E5H2" 0.3510905 "hypothetical protein"

"JS1_D03_00628" 12537 "s63E6H2" 0.2659024 "hypothetical protein"
"JS1_D03_00630" 12539 "s59E13H2" 0.05229575 "Transaldolase"
"JS1_D03_00630" 12539 "s59E25H2" 0 "Transaldolase"
"JS1_D03_00630" 12539 "s59E5H2" 0.01585763 "Transaldolase"
"JS1_D03_00630" 12539 "s63E6H2" 0.1876959 "Transaldolase"
"JS1_D03_00631" 12540 "s59E13H2" 3.834835 "hypothetical protein"
"JS1_D03_00631" 12540 "s59E25H2" 0 "hypothetical protein"
"JS1_D03_00631" 12540 "s59E5H2" 0.08590113 "hypothetical protein"
"JS1_D03_00631" 12540 "s63E6H2" 0.1275861 "hypothetical protein"
"JS1_D03_00632" 12541 "s59E13H2" 1.575381 "hypothetical protein"
"JS1_D03_00632" 12541 "s59E25H2" 0 "hypothetical protein"
"JS1_D03_00632" 12541 "s59E5H2" 0.05332807 "hypothetical protein"
"JS1_D03_00632" 12541 "s63E6H2" 0.3750851 "hypothetical protein"
"JS1_D03_00633" 12542 "s59E13H2" 0.3461652 "Phosphoglycolate phosphatase"
"JS1_D03_00633" 12542 "s59E25H2" 0 "Phosphoglycolate phosphatase"
"JS1_D03_00633" 12542 "s59E5H2" 0.4277095 "Phosphoglycolate phosphatase"
"JS1_D03_00633" 12542 "s63E6H2" 0.5085964 "Phosphoglycolate phosphatase"
"JS1_D03_00634" 12543 "s59E13H2" 0.2046725 "Cupin domain protein"
"JS1_D03_00634" 12543 "s59E25H2" 0 "Cupin domain protein"
"JS1_D03_00634" 12543 "s59E5H2" 0.091191 "Cupin domain protein"
"JS1_D03_00634" 12543 "s63E6H2" 0.2508069 "Cupin domain protein"
"JS1_D03_00635" 12544 "s59E13H2" 0.09020057 "ADP-ribose pyrophosphatase"
"JS1_D03_00635" 12544 "s59E25H2" 0 "ADP-ribose pyrophosphatase"
"JS1_D03_00635" 12544 "s59E5H2" 0.3441353 "ADP-ribose pyrophosphatase"
"JS1_D03_00635" 12544 "s63E6H2" 0.4954924 "ADP-ribose pyrophosphatase"
"JS1_D03_00636" 12545 "s59E13H2" 0.200107 "hypothetical protein"
"JS1_D03_00636" 12545 "s59E25H2" 0 "hypothetical protein"
"JS1_D03_00636" 12545 "s59E5H2" 0.43822 "hypothetical protein"
"JS1_D03_00636" 12545 "s63E6H2" 0.4296195 "hypothetical protein"
"JS1_D03_00637" 12546 "s59E13H2" 0.2509837 "Tetratricopeptide repeat protein"
"JS1_D03_00637" 12546 "s59E25H2" 0 "Tetratricopeptide repeat protein"
"JS1_D03_00637" 12546 "s59E5H2" 0.3174992 "Tetratricopeptide repeat protein"
"JS1_D03_00637" 12546 "s63E6H2" 0.4686725 "Tetratricopeptide repeat protein"
"JS1_D03_00638" 12547 "s59E13H2" 0.1101988 "Putative ski2-type helicase"
"JS1_D03_00638" 12547 "s59E25H2" 0.1103811 "Putative ski2-type helicase"
"JS1_D03_00638" 12547 "s59E5H2" 0.4789527 "Putative ski2-type helicase"
"JS1_D03_00638" 12547 "s63E6H2" 0.4610674 "Putative ski2-type helicase"
"JS1_D03_00639" 12548 "s59E13H2" 2.330677 "putative endonuclease 4"
"JS1_D03_00639" 12548 "s59E25H2" 0 "putative endonuclease 4"
"JS1_D03_00639" 12548 "s59E5H2" 0.2145068 "putative endonuclease 4"
"JS1_D03_00639" 12548 "s63E6H2" 0.3416632 "putative endonuclease 4"
"JS1_D03_00640" 12549 "s59E13H2" 2.604852 "hypothetical protein"
"JS1_D03_00640" 12549 "s59E25H2" 0 "hypothetical protein"
"JS1_D03_00640" 12549 "s59E5H2" 0.1339643 "hypothetical protein"
"JS1_D03_00640" 12549 "s63E6H2" 0.1306207 "hypothetical protein"
"JS1_D03_00642" 12551 "s59E13H2" 0.7256066 "hypothetical protein"
"JS1_D03_00642" 12551 "s59E25H2" 0 "hypothetical protein"

"JS1_D03_00642" 12551 "s59E5H2" 0.296585 "hypothetical protein"
"JS1_D03_00642" 12551 "s63E6H2" 0.8370518 "hypothetical protein"
"JS1_D03_00644" 12553 "s59E13H2" 0.03900689 "Coenzyme A disulfide reductase"
"JS1_D03_00644" 12553 "s59E25H2" 0 "Coenzyme A disulfide reductase"
"JS1_D03_00644" 12553 "s59E5H2" 0.1292891 "Coenzyme A disulfide reductase"
"JS1_D03_00644" 12553 "s63E6H2" 0.02796018 "Coenzyme A disulfide reductase"
"JS1_D03_00645" 12554 "s59E13H2" 0.09403961 "Coenzyme A disulfide reductase"
"JS1_D03_00645" 12554 "s59E25H2" 0 "Coenzyme A disulfide reductase"
"JS1_D03_00645" 12554 "s59E5H2" 0.1271 "Coenzyme A disulfide reductase"
"JS1_D03_00645" 12554 "s63E6H2" 0.01530968 "Coenzyme A disulfide reductase"
"JS1_D03_00646" 12555 "s59E13H2" 0.02504716 "DNA-directed RNA polymerase subunit beta"
"JS1_D03_00646" 12555 "s59E25H2" 0 "DNA-directed RNA polymerase subunit beta"
"JS1_D03_00646" 12555 "s59E5H2" 0.01547484 "DNA-directed RNA polymerase subunit beta"
"JS1_D03_00646" 12555 "s63E6H2" 0.009198763 "DNA-directed RNA polymerase subunit beta"
"JS1_D03_00648" 12557 "s59E13H2" 0 "hypothetical protein"
"JS1_D03_00648" 12557 "s59E25H2" 0 "hypothetical protein"
"JS1_D03_00648" 12557 "s59E5H2" 0.007830817 "hypothetical protein"
"JS1_D03_00648" 12557 "s63E6H2" 0.0006309841 "hypothetical protein"
"JS1_D03_00649" 12558 "s59E13H2" 0 "Modification methylase BamHI"
"JS1_D03_00649" 12558 "s59E25H2" 0 "Modification methylase BamHI"
"JS1_D03_00649" 12558 "s59E5H2" 0 "Modification methylase BamHI"
"JS1_D03_00649" 12558 "s63E6H2" 0 "Modification methylase BamHI"
"JS1_D03_00650" 12559 "s59E13H2" 0.1287707 "N-isopropylammelide isopropyl amidohydrolase"
"JS1_D03_00650" 12559 "s59E25H2" 0 "N-isopropylammelide isopropyl amidohydrolase"
"JS1_D03_00650" 12559 "s59E5H2" 0.01666878 "N-isopropylammelide isopropyl amidohydrolase"
"JS1_D03_00650" 12559 "s63E6H2" 0.1076982 "N-isopropylammelide isopropyl amidohydrolase"
"JS1_D03_00651" 12560 "s59E13H2" 0.1091026 "beta-methylgalactoside transporter inner membrane component"
"JS1_D03_00651" 12560 "s59E25H2" 0 "beta-methylgalactoside transporter inner membrane component"
"JS1_D03_00651" 12560 "s59E5H2" 0.02047349 "beta-methylgalactoside transporter inner membrane component"
"JS1_D03_00651" 12560 "s63E6H2" 0.2781515 "beta-methylgalactoside transporter inner membrane component"
"JS1_D03_00652" 12561 "s59E13H2" 0.2600029 "D-allose transporter subunit"
"JS1_D03_00652" 12561 "s59E25H2" 0 "D-allose transporter subunit"
"JS1_D03_00652" 12561 "s59E5H2" 0.01641018 "D-allose transporter subunit"
"JS1_D03_00652" 12561 "s63E6H2" 0.608696 "D-allose transporter subunit"
"JS1_D03_00653" 12562 "s59E13H2" 0.08947608 "Galactose/methyl galactoside import ATP-binding protein MglA"
"JS1_D03_00653" 12562 "s59E25H2" 0 "Galactose/methyl galactoside import ATP-binding protein MglA"
"JS1_D03_00653" 12562 "s59E5H2" 0.01244925 "Galactose/methyl galactoside import ATP-binding protein MglA"
"JS1_D03_00653" 12562 "s63E6H2" 0.2404537 "Galactose/methyl galactoside import ATP-binding protein MglA"
"JS1_D03_00654" 12563 "s59E13H2" 0.1456292 "Purine-binding protein precursor"
"JS1_D03_00654" 12563 "s59E25H2" 0 "Purine-binding protein precursor"
"JS1_D03_00654" 12563 "s59E5H2" 0.4781473 "Purine-binding protein precursor"
"JS1_D03_00654" 12563 "s63E6H2" 0.1993439 "Purine-binding protein precursor"
"JS1_D03_00655" 12564 "s59E13H2" 0.0707107 "4-hydroxybenzoyl-CoA reductase subunit alpha"
"JS1_D03_00655" 12564 "s59E25H2" 0 "4-hydroxybenzoyl-CoA reductase subunit alpha"
"JS1_D03_00655" 12564 "s59E5H2" 0.6875169 "4-hydroxybenzoyl-CoA reductase subunit alpha"
"JS1_D03_00655" 12564 "s63E6H2" 0.5093686 "4-hydroxybenzoyl-CoA reductase subunit alpha"
"JS1_D03_00656" 12565 "s59E13H2" 0.05987351 "Glyceraldehyde dehydrogenase small chain"

"JS1_D03_00656" 12565 "s59E25H2" 0 "Glyceraldehyde dehydrogenase small chain"
"JS1_D03_00656" 12565 "s59E5H2" 0.7932873 "Glyceraldehyde dehydrogenase small chain"
"JS1_D03_00656" 12565 "s63E6H2" 0.9622483 "Glyceraldehyde dehydrogenase small chain"
"JS1_D03_00657" 12566 "s59E13H2" 0.002840059 "Uracil permease"
"JS1_D03_00657" 12566 "s59E25H2" 0 "Uracil permease"
"JS1_D03_00657" 12566 "s59E5H2" 0.6809588 "Uracil permease"
"JS1_D03_00657" 12566 "s63E6H2" 0.4292365 "Uracil permease"
"JS1_D03_00658" 12567 "s59E13H2" 0.01458178 "4-hydroxybenzoyl-CoA reductase subunit alpha"
"JS1_D03_00658" 12567 "s59E25H2" 0 "4-hydroxybenzoyl-CoA reductase subunit alpha"
"JS1_D03_00658" 12567 "s59E5H2" 0.09212582 "4-hydroxybenzoyl-CoA reductase subunit alpha"
"JS1_D03_00658" 12567 "s63E6H2" 0.2385931 "4-hydroxybenzoyl-CoA reductase subunit alpha"
"JS1_D03_00659" 12568 "s59E13H2" 0.0160137 "Nicotinate dehydrogenase small FeS subunit"
"JS1_D03_00659" 12568 "s59E25H2" 0 "Nicotinate dehydrogenase small FeS subunit"
"JS1_D03_00659" 12568 "s59E5H2" 0 "Nicotinate dehydrogenase small FeS subunit"
"JS1_D03_00659" 12568 "s63E6H2" 0.4729259 "Nicotinate dehydrogenase small FeS subunit"
"JS1_D03_00660" 12569 "s59E13H2" 0.009070027 "Carbon monoxide dehydrogenase medium chain"
"JS1_D03_00660" 12569 "s59E25H2" 0 "Carbon monoxide dehydrogenase medium chain"
"JS1_D03_00660" 12569 "s59E5H2" 0 "Carbon monoxide dehydrogenase medium chain"
"JS1_D03_00660" 12569 "s63E6H2" 0.5479423 "Carbon monoxide dehydrogenase medium chain"
"JS1_D03_00661" 12570 "s59E13H2" 0.05349988 "D-aminopeptidase"
"JS1_D03_00661" 12570 "s59E25H2" 0 "D-aminopeptidase"
"JS1_D03_00661" 12570 "s59E5H2" 0.1705571 "D-aminopeptidase"
"JS1_D03_00661" 12570 "s63E6H2" 0.4036542 "D-aminopeptidase"
"JS1_D03_00662" 12571 "s59E13H2" 0.006752721 "hypothetical protein"
"JS1_D03_00662" 12571 "s59E25H2" 0 "hypothetical protein"
"JS1_D03_00662" 12571 "s59E5H2" 0.05099941 "hypothetical protein"
"JS1_D03_00662" 12571 "s63E6H2" 0.2003126 "hypothetical protein"
"JS1_D03_00663" 12572 "s59E13H2" 0.00479696 "NH(3)-dependent NAD(+) synthetase"
"JS1_D03_00663" 12572 "s59E25H2" 0 "NH(3)-dependent NAD(+) synthetase"
"JS1_D03_00663" 12572 "s59E5H2" 0.01755001 "NH(3)-dependent NAD(+) synthetase"
"JS1_D03_00663" 12572 "s63E6H2" 0.2379816 "NH(3)-dependent NAD(+) synthetase"
"JS1_D03_00664" 12573 "s59E13H2" 0.1351372 "transcriptional regulator SlyA"
"JS1_D03_00664" 12573 "s59E25H2" 0 "transcriptional regulator SlyA"
"JS1_D03_00664" 12573 "s59E5H2" 0.1433966 "transcriptional regulator SlyA"
"JS1_D03_00664" 12573 "s63E6H2" 0.398729 "transcriptional regulator SlyA"
"JS1_D03_00665" 12574 "s59E13H2" 0.274829 "Cadmium, cobalt and zinc/H(+)-K(+) antiporter"
"JS1_D03_00665" 12574 "s59E25H2" 0 "Cadmium, cobalt and zinc/H(+)-K(+) antiporter"
"JS1_D03_00665" 12574 "s59E5H2" 0.3118949 "Cadmium, cobalt and zinc/H(+)-K(+) antiporter"
"JS1_D03_00665" 12574 "s63E6H2" 0.9236922 "Cadmium, cobalt and zinc/H(+)-K(+) antiporter"
"JS1_D03_00666" 12575 "s59E13H2" 0.05022615 "putative ATP-dependent RNA helicase"
"JS1_D03_00666" 12575 "s59E25H2" 0 "putative ATP-dependent RNA helicase"
"JS1_D03_00666" 12575 "s59E5H2" 0.08376062 "putative ATP-dependent RNA helicase"
"JS1_D03_00666" 12575 "s63E6H2" 0.4646976 "putative ATP-dependent RNA helicase"
"JS1_D03_00667" 12576 "s59E13H2" 0 "Alpha/beta hydrolase family protein"
"JS1_D03_00667" 12576 "s59E25H2" 0 "Alpha/beta hydrolase family protein"
"JS1_D03_00667" 12576 "s59E5H2" 0.006696489 "Alpha/beta hydrolase family protein"
"JS1_D03_00667" 12576 "s63E6H2" 0.01523034 "Alpha/beta hydrolase family protein"

"JS1_D03_00668" 12577 "s59E13H2" 0 "ABC-2 type transporter"
"JS1_D03_00668" 12577 "s59E25H2" 0 "ABC-2 type transporter"
"JS1_D03_00668" 12577 "s59E5H2" 0.05804201 "ABC-2 type transporter"
"JS1_D03_00668" 12577 "s63E6H2" 0.01229367 "ABC-2 type transporter"
"JS1_D03_00669" 12578 "s59E13H2" 0 "putative ABC transporter ATP-binding protein YbhF"
"JS1_D03_00669" 12578 "s59E25H2" 0 "putative ABC transporter ATP-binding protein YbhF"
"JS1_D03_00669" 12578 "s59E5H2" 0.04045869 "putative ABC transporter ATP-binding protein YbhF"
"JS1_D03_00669" 12578 "s63E6H2" 0.01025797 "putative ABC transporter ATP-binding protein YbhF"
"JS1_D03_00670" 12579 "s59E13H2" 11.24278 "Swarming motility protein SwrC"
"JS1_D03_00670" 12579 "s59E25H2" 0.1296654 "Swarming motility protein SwrC"
"JS1_D03_00670" 12579 "s59E5H2" 4.612066 "Swarming motility protein SwrC"
"JS1_D03_00670" 12579 "s63E6H2" 5.390075 "Swarming motility protein SwrC"
"JS1_D03_00671" 12580 "s59E13H2" 7.50219 "Macrolide export protein MacA"
"JS1_D03_00671" 12580 "s59E25H2" 0 "Macrolide export protein MacA"
"JS1_D03_00671" 12580 "s59E5H2" 4.026888 "Macrolide export protein MacA"
"JS1_D03_00671" 12580 "s63E6H2" 3.268446 "Macrolide export protein MacA"
"JS1_D03_00672" 12581 "s59E13H2" 1.103349 "Outer membrane protein TolC precursor"
"JS1_D03_00672" 12581 "s59E25H2" 0 "Outer membrane protein TolC precursor"
"JS1_D03_00672" 12581 "s59E5H2" 0.6542326 "Outer membrane protein TolC precursor"
"JS1_D03_00672" 12581 "s63E6H2" 1.017147 "Outer membrane protein TolC precursor"
"JS1_D03_00673" 12582 "s59E13H2" 3.366326 "hypothetical protein"
"JS1_D03_00673" 12582 "s59E25H2" 0 "hypothetical protein"
"JS1_D03_00673" 12582 "s59E5H2" 1.168265 "hypothetical protein"
"JS1_D03_00673" 12582 "s63E6H2" 1.02749 "hypothetical protein"
"JS1_D03_00675" 12584 "s59E13H2" 0.007879382 "hypothetical protein"
"JS1_D03_00675" 12584 "s59E25H2" 0 "hypothetical protein"
"JS1_D03_00675" 12584 "s59E5H2" 0.0156629 "hypothetical protein"
"JS1_D03_00675" 12584 "s63E6H2" 0.06308846 "hypothetical protein"
"JS1_D03_00676" 12585 "s59E13H2" 0.04491013 "Peptidase M15"
"JS1_D03_00676" 12585 "s59E25H2" 0 "Peptidase M15"
"JS1_D03_00676" 12585 "s59E5H2" 0 "Peptidase M15"
"JS1_D03_00676" 12585 "s63E6H2" 0.2253486 "Peptidase M15"
"JS1_D03_00677" 12586 "s59E13H2" 0.03941502 "YvrJ protein family protein"
"JS1_D03_00677" 12586 "s59E25H2" 0 "YvrJ protein family protein"
"JS1_D03_00677" 12586 "s59E5H2" 0 "YvrJ protein family protein"
"JS1_D03_00677" 12586 "s63E6H2" 0.1476562 "YvrJ protein family protein"
"JS1_D03_00678" 12587 "s59E13H2" 0 "Trehalose/maltose transport system permease protein MalF"
"JS1_D03_00678" 12587 "s59E25H2" 0 "Trehalose/maltose transport system permease protein MalF"
"JS1_D03_00678" 12587 "s59E5H2" 0.04017247 "Trehalose/maltose transport system permease protein MalF"
"JS1_D03_00678" 12587 "s63E6H2" 2.408278 "Trehalose/maltose transport system permease protein MalF"
"JS1_D03_00679" 12588 "s59E13H2" 0 "Trehalose/maltose transport system permease protein MalG"
"JS1_D03_00679" 12588 "s59E25H2" 0 "Trehalose/maltose transport system permease protein MalG"
"JS1_D03_00679" 12588 "s59E5H2" 0.05491542 "Trehalose/maltose transport system permease protein MalG"
"JS1_D03_00679" 12588 "s63E6H2" 0.1834193 "Trehalose/maltose transport system permease protein MalG"
"JS1_D03_00680" 12589 "s59E13H2" 0 "Bacterial extracellular solute-binding protein"
"JS1_D03_00680" 12589 "s59E25H2" 0 "Bacterial extracellular solute-binding protein"
"JS1_D03_00680" 12589 "s59E5H2" 0.1110434 "Bacterial extracellular solute-binding protein"

"JS1_D03_00680" 12589 "s63E6H2" 0.1611918 "Bacterial extracellular solute-binding protein"
"JS1_D03_00681" 12590 "s59E13H2" 0.003453717 "L-glyceraldehyde 3-phosphate reductase"
"JS1_D03_00681" 12590 "s59E25H2" 0 "L-glyceraldehyde 3-phosphate reductase"
"JS1_D03_00681" 12590 "s59E5H2" 0.04948507 "L-glyceraldehyde 3-phosphate reductase"
"JS1_D03_00681" 12590 "s63E6H2" 0.4252135 "L-glyceraldehyde 3-phosphate reductase"
"JS1_D03_00682" 12591 "s59E13H2" 4.321942 "Xylulose kinase"
"JS1_D03_00682" 12591 "s59E25H2" 0.1633987 "Xylulose kinase"
"JS1_D03_00682" 12591 "s59E5H2" 0.04522332 "Xylulose kinase"
"JS1_D03_00682" 12591 "s63E6H2" 0.3438732 "Xylulose kinase"
"JS1_D03_00683" 12592 "s59E13H2" 0 "methylcobalamin:coenzyme M methyltransferase"
"JS1_D03_00683" 12592 "s59E25H2" 0 "methylcobalamin:coenzyme M methyltransferase"
"JS1_D03_00683" 12592 "s59E5H2" 0 "methylcobalamin:coenzyme M methyltransferase"
"JS1_D03_00683" 12592 "s63E6H2" 1.371868 "methylcobalamin:coenzyme M methyltransferase"
"JS1_D03_00684" 12593 "s59E13H2" 0 "Bacterial extracellular solute-binding protein"
"JS1_D03_00684" 12593 "s59E25H2" 0 "Bacterial extracellular solute-binding protein"
"JS1_D03_00684" 12593 "s59E5H2" 0.01110928 "Bacterial extracellular solute-binding protein"
"JS1_D03_00684" 12593 "s63E6H2" 1.201736 "Bacterial extracellular solute-binding protein"
"JS1_D03_00685" 12594 "s59E13H2" 0.04862362 "Alpha-glucosidase"
"JS1_D03_00685" 12594 "s59E25H2" 0 "Alpha-glucosidase"
"JS1_D03_00685" 12594 "s59E5H2" 0.0225672 "Alpha-glucosidase"
"JS1_D03_00685" 12594 "s63E6H2" 1.528752 "Alpha-glucosidase"
"JS1_D03_00686" 12595 "s59E13H2" 0.0148992 "Mannosylglycerate hydrolase"
"JS1_D03_00686" 12595 "s59E25H2" 0 "Mannosylglycerate hydrolase"
"JS1_D03_00686" 12595 "s59E5H2" 0.3180386 "Mannosylglycerate hydrolase"
"JS1_D03_00686" 12595 "s63E6H2" 0.9263847 "Mannosylglycerate hydrolase"
"JS1_D03_00687" 12596 "s59E13H2" 0.03235054 "PHP domain protein"
"JS1_D03_00687" 12596 "s59E25H2" 0.1526419 "PHP domain protein"
"JS1_D03_00687" 12596 "s59E5H2" 0.0531931 "PHP domain protein"
"JS1_D03_00687" 12596 "s63E6H2" 0.6938758 "PHP domain protein"
"JS1_D03_00688" 12597 "s59E13H2" 0 "putative D,D-dipeptide transport ATP-binding protein DdpF"
"JS1_D03_00688" 12597 "s59E25H2" 0 "putative D,D-dipeptide transport ATP-binding protein DdpF"
"JS1_D03_00688" 12597 "s59E5H2" 0 "putative D,D-dipeptide transport ATP-binding protein DdpF"
"JS1_D03_00688" 12597 "s63E6H2" 0.7568056 "putative D,D-dipeptide transport ATP-binding protein DdpF"
"JS1_D03_00689" 12598 "s59E13H2" 0 "Oligopeptide transport ATP-binding protein OppD"
"JS1_D03_00689" 12598 "s59E25H2" 0 "Oligopeptide transport ATP-binding protein OppD"
"JS1_D03_00689" 12598 "s59E5H2" 0.009140329 "Oligopeptide transport ATP-binding protein OppD"
"JS1_D03_00689" 12598 "s63E6H2" 0.8490857 "Oligopeptide transport ATP-binding protein OppD"
"JS1_D03_00690" 12599 "s59E13H2" 0.2418319 "hypothetical protein"
"JS1_D03_00690" 12599 "s59E25H2" 0 "hypothetical protein"
"JS1_D03_00690" 12599 "s59E5H2" 0.1248727 "hypothetical protein"
"JS1_D03_00690" 12599 "s63E6H2" 0.0363764 "hypothetical protein"
"JS1_D03_00691" 12600 "s59E13H2" 0.07108884 "5'-methylthioadenosine/S-adenosylhomocysteine nucleosidase"
"JS1_D03_00691" 12600 "s59E25H2" 0 "5'-methylthioadenosine/S-adenosylhomocysteine nucleosidase"
"JS1_D03_00691" 12600 "s59E5H2" 0.02943122 "5'-methylthioadenosine/S-adenosylhomocysteine nucleosidase"
"JS1_D03_00691" 12600 "s63E6H2" 0.009160803 "5'-methylthioadenosine/S-adenosylhomocysteine nucleosidase"
"JS1_D03_00692" 12601 "s59E13H2" 0 "hypothetical protein"
"JS1_D03_00692" 12601 "s59E25H2" 0 "hypothetical protein"

"JS1_D03_00692" 12601 "s59E5H2" 0.009449124 "hypothetical protein"
"JS1_D03_00692" 12601 "s63E6H2" 0.01875895 "hypothetical protein"
"JS1_D03_00693" 12602 "s59E13H2" 0 "hypothetical protein"
"JS1_D03_00693" 12602 "s59E25H2" 0 "hypothetical protein"
"JS1_D03_00693" 12602 "s59E5H2" 0 "hypothetical protein"
"JS1_D03_00693" 12602 "s63E6H2" 0.02023312 "hypothetical protein"
"JS1_D03_00694" 12603 "s59E13H2" 0 "Reverse rubrerythrin-2"
"JS1_D03_00694" 12603 "s59E25H2" 0 "Reverse rubrerythrin-2"
"JS1_D03_00694" 12603 "s59E5H2" 0.7933924 "Reverse rubrerythrin-2"
"JS1_D03_00694" 12603 "s63E6H2" 0.03826268 "Reverse rubrerythrin-2"
"JS1_D03_00695" 12604 "s59E13H2" 0 "hypothetical protein"
"JS1_D03_00695" 12604 "s59E25H2" 0 "hypothetical protein"
"JS1_D03_00695" 12604 "s59E5H2" 0 "hypothetical protein"
"JS1_D03_00695" 12604 "s63E6H2" 0 "hypothetical protein"
"JS1_D03_00696" 12605 "s59E13H2" 0 "hypothetical protein"
"JS1_D03_00696" 12605 "s59E25H2" 0 "hypothetical protein"
"JS1_D03_00696" 12605 "s59E5H2" 0.0212906 "hypothetical protein"
"JS1_D03_00696" 12605 "s63E6H2" 0 "hypothetical protein"
"JS1_D03_00697" 12606 "s59E13H2" 0 "Cupin domain protein"
"JS1_D03_00697" 12606 "s59E25H2" 0 "Cupin domain protein"
"JS1_D03_00697" 12606 "s59E5H2" 0 "Cupin domain protein"
"JS1_D03_00697" 12606 "s63E6H2" 0 "Cupin domain protein"
"JS1_D03_00698" 12607 "s59E13H2" 0.007820211 "Copper-exporting P-type ATPase B"
"JS1_D03_00698" 12607 "s59E25H2" 0 "Copper-exporting P-type ATPase B"
"JS1_D03_00698" 12607 "s59E5H2" 0.3570859 "Copper-exporting P-type ATPase B"
"JS1_D03_00698" 12607 "s63E6H2" 3.82831 "Copper-exporting P-type ATPase B"
"JS1_D03_00701" 12610 "s59E13H2" 0.001995391 "Galactose/methyl galactoside import ATP-binding protein MglA"
"JS1_D03_00701" 12610 "s59E25H2" 0 "Galactose/methyl galactoside import ATP-binding protein MglA"
"JS1_D03_00701" 12610 "s59E5H2" 0 "Galactose/methyl galactoside import ATP-binding protein MglA"
"JS1_D03_00701" 12610 "s63E6H2" 0.4453203 "Galactose/methyl galactoside import ATP-binding protein MglA"
"JS1_D03_00702" 12611 "s59E13H2" 0 "Branched-chain amino acid transport system / permease component"
"JS1_D03_00702" 12611 "s59E25H2" 0.4752381 "Branched-chain amino acid transport system / permease component"
"JS1_D03_00702" 12611 "s59E5H2" 0 "Branched-chain amino acid transport system / permease component"
"JS1_D03_00702" 12611 "s63E6H2" 1.158087 "Branched-chain amino acid transport system / permease component"
"JS1_D03_00703" 12612 "s59E13H2" 0 "L-arabinose transporter permease protein"
"JS1_D03_00703" 12612 "s59E25H2" 0 "L-arabinose transporter permease protein"
"JS1_D03_00703" 12612 "s59E5H2" 0 "L-arabinose transporter permease protein"
"JS1_D03_00703" 12612 "s63E6H2" 0.5953771 "L-arabinose transporter permease protein"
"JS1_D03_00704" 12613 "s59E13H2" 0.009432062 "Xaa-Pro dipeptidase"
"JS1_D03_00704" 12613 "s59E25H2" 0 "Xaa-Pro dipeptidase"
"JS1_D03_00704" 12613 "s59E5H2" 0 "Xaa-Pro dipeptidase"
"JS1_D03_00704" 12613 "s63E6H2" 0.04943495 "Xaa-Pro dipeptidase"
"JS1_D03_00705" 12614 "s59E13H2" 0.5564996 "Tungsten-containing aldehyde ferredoxin oxidoreductase"
"JS1_D03_00705" 12614 "s59E25H2" 0 "Tungsten-containing aldehyde ferredoxin oxidoreductase"
"JS1_D03_00705" 12614 "s59E5H2" 0.06289214 "Tungsten-containing aldehyde ferredoxin oxidoreductase"
"JS1_D03_00705" 12614 "s63E6H2" 0.1639584 "Tungsten-containing aldehyde ferredoxin oxidoreductase"
"JS1_D03_00706" 12615 "s59E13H2" 0.06073476 "4-hydroxy-tetrahydrodipicolinate synthase"

"JS1_D03_00706" 12615 "s59E25H2" 0 "4-hydroxy-tetrahydrodipicolinate synthase"
"JS1_D03_00706" 12615 "s59E5H2" 0.01252562 "4-hydroxy-tetrahydrodipicolinate synthase"
"JS1_D03_00706" 12615 "s63E6H2" 0.2705015 "4-hydroxy-tetrahydrodipicolinate synthase"
"JS1_D03_00707" 12616 "s59E13H2" 0.02306422 "L-serine dehydratase, alpha chain"
"JS1_D03_00707" 12616 "s59E25H2" 0 "L-serine dehydratase, alpha chain"
"JS1_D03_00707" 12616 "s59E5H2" 0.02307898 "L-serine dehydratase, alpha chain"
"JS1_D03_00707" 12616 "s63E6H2" 0.2804195 "L-serine dehydratase, alpha chain"
"JS1_D03_00708" 12617 "s59E13H2" 0 "hypothetical protein"
"JS1_D03_00708" 12617 "s59E25H2" 0 "hypothetical protein"
"JS1_D03_00708" 12617 "s59E5H2" 0.0221976 "hypothetical protein"
"JS1_D03_00708" 12617 "s63E6H2" 0.4043819 "hypothetical protein"
"JS1_D03_00709" 12618 "s59E13H2" 0.007613554 "hypothetical protein"
"JS1_D03_00709" 12618 "s59E25H2" 0 "hypothetical protein"
"JS1_D03_00709" 12618 "s59E5H2" 0.07601988 "hypothetical protein"
"JS1_D03_00709" 12618 "s63E6H2" 0.2361612 "hypothetical protein"
"JS1_D03_00710" 12619 "s59E13H2" 0.01169202 "hypothetical protein"
"JS1_D03_00710" 12619 "s59E25H2" 0 "hypothetical protein"
"JS1_D03_00710" 12619 "s59E5H2" 0.08098146 "hypothetical protein"
"JS1_D03_00710" 12619 "s63E6H2" 0.2933981 "hypothetical protein"
"JS1_D03_00711" 12620 "s59E13H2" 0.0121277 "methylcobalamin:coenzyme M methyltransferase"
"JS1_D03_00711" 12620 "s59E25H2" 0 "methylcobalamin:coenzyme M methyltransferase"
"JS1_D03_00711" 12620 "s59E5H2" 0 "methylcobalamin:coenzyme M methyltransferase"
"JS1_D03_00711" 12620 "s63E6H2" 0.5579626 "methylcobalamin:coenzyme M methyltransferase"
"JS1_D03_00712" 12621 "s59E13H2" 0.2156421 "hypothetical protein"
"JS1_D03_00712" 12621 "s59E25H2" 0 "hypothetical protein"
"JS1_D03_00712" 12621 "s59E5H2" 1.075236 "hypothetical protein"
"JS1_D03_00712" 12621 "s63E6H2" 1.095757 "hypothetical protein"
"JS1_D03_00713" 12622 "s59E13H2" 0.3519876 "Arabinose metabolism transcriptional repressor"
"JS1_D03_00713" 12622 "s59E25H2" 0 "Arabinose metabolism transcriptional repressor"
"JS1_D03_00713" 12622 "s59E5H2" 0.7173318 "Arabinose metabolism transcriptional repressor"
"JS1_D03_00713" 12622 "s63E6H2" 0.4629832 "Arabinose metabolism transcriptional repressor"
"JS1_D03_00714" 12623 "s59E13H2" 0.3790798 "L-ribulose-5-phosphate 4-epimerase UlaF"
"JS1_D03_00714" 12623 "s59E25H2" 0 "L-ribulose-5-phosphate 4-epimerase UlaF"
"JS1_D03_00714" 12623 "s59E5H2" 0.2730743 "L-ribulose-5-phosphate 4-epimerase UlaF"
"JS1_D03_00714" 12623 "s63E6H2" 0.7287951 "L-ribulose-5-phosphate 4-epimerase UlaF"
"JS1_D03_00715" 12624 "s59E13H2" 0.5071313 "Xylulose kinase"
"JS1_D03_00715" 12624 "s59E25H2" 0 "Xylulose kinase"
"JS1_D03_00715" 12624 "s59E5H2" 0.625939 "Xylulose kinase"
"JS1_D03_00715" 12624 "s63E6H2" 1.424896 "Xylulose kinase"
"JS1_D03_00717" 12626 "s59E13H2" 2.454967 "D-tagatose-1,6-bisphosphate aldolase subunit GatY"
"JS1_D03_00717" 12626 "s59E25H2" 0 "D-tagatose-1,6-bisphosphate aldolase subunit GatY"
"JS1_D03_00717" 12626 "s59E5H2" 0.4620255 "D-tagatose-1,6-bisphosphate aldolase subunit GatY"
"JS1_D03_00717" 12626 "s63E6H2" 2.035991 "D-tagatose-1,6-bisphosphate aldolase subunit GatY"
"JS1_D03_00718" 12627 "s59E13H2" 2.46421 "Xylulose kinase"
"JS1_D03_00718" 12627 "s59E25H2" 0 "Xylulose kinase"
"JS1_D03_00718" 12627 "s59E5H2" 0.7180273 "Xylulose kinase"
"JS1_D03_00718" 12627 "s63E6H2" 1.110488 "Xylulose kinase"

"JS1_D03_00719" 12628 "s59E13H2" 4.639833 "Trehalose/maltose-binding protein MalE precursor"
"JS1_D03_00719" 12628 "s59E25H2" 0 "Trehalose/maltose-binding protein MalE precursor"
"JS1_D03_00719" 12628 "s59E5H2" 0.5385474 "Trehalose/maltose-binding protein MalE precursor"
"JS1_D03_00719" 12628 "s63E6H2" 1.262909 "Trehalose/maltose-binding protein MalE precursor"
"JS1_D03_00720" 12629 "s59E13H2" 4.017015 "Lactose transport system permease protein LacF"
"JS1_D03_00720" 12629 "s59E25H2" 0 "Lactose transport system permease protein LacF"
"JS1_D03_00720" 12629 "s59E5H2" 0.9442979 "Lactose transport system permease protein LacF"
"JS1_D03_00720" 12629 "s63E6H2" 1.021581 "Lactose transport system permease protein LacF"
"JS1_D03_00721" 12630 "s59E13H2" 1.932376 "L-arabinose transport system permease protein AraQ"
"JS1_D03_00721" 12630 "s59E25H2" 0 "L-arabinose transport system permease protein AraQ"
"JS1_D03_00721" 12630 "s59E5H2" 0.2270753 "L-arabinose transport system permease protein AraQ"
"JS1_D03_00721" 12630 "s63E6H2" 0.8109675 "L-arabinose transport system permease protein AraQ"
"JS1_D03_00722" 12631 "s59E13H2" 1.30443 "Alpha-D-glucose-1-phosphate phosphatase YihX"
"JS1_D03_00722" 12631 "s59E25H2" 0 "Alpha-D-glucose-1-phosphate phosphatase YihX"
"JS1_D03_00722" 12631 "s59E5H2" 0.4926376 "Alpha-D-glucose-1-phosphate phosphatase YihX"
"JS1_D03_00722" 12631 "s63E6H2" 0.8620753 "Alpha-D-glucose-1-phosphate phosphatase YihX"
"JS1_D03_00723" 12632 "s59E13H2" 0.0751956 "HTH-type transcriptional regulator PuuR"
"JS1_D03_00723" 12632 "s59E25H2" 0 "HTH-type transcriptional regulator PuuR"
"JS1_D03_00723" 12632 "s59E5H2" 0.2800394 "HTH-type transcriptional regulator PuuR"
"JS1_D03_00723" 12632 "s63E6H2" 0.5934044 "HTH-type transcriptional regulator PuuR"
"JS1_D03_00724" 12633 "s59E13H2" 0.003651809 "ATP-dependent protease ATPase subunit HslU"
"JS1_D03_00724" 12633 "s59E25H2" 0 "ATP-dependent protease ATPase subunit HslU"
"JS1_D03_00724" 12633 "s59E5H2" 0.4125138 "ATP-dependent protease ATPase subunit HslU"
"JS1_D03_00724" 12633 "s63E6H2" 0.02740179 "ATP-dependent protease ATPase subunit HslU"
"JS1_D03_00725" 12634 "s59E13H2" 0 "ATP-dependent protease subunit HslV"
"JS1_D03_00725" 12634 "s59E25H2" 0 "ATP-dependent protease subunit HslV"
"JS1_D03_00725" 12634 "s59E5H2" 0.05555484 "ATP-dependent protease subunit HslV"
"JS1_D03_00725" 12634 "s63E6H2" 0.03603091 "ATP-dependent protease subunit HslV"
"JS1_D03_00726" 12635 "s59E13H2" 0.02789893 "NifU-like protein"
"JS1_D03_00726" 12635 "s59E25H2" 0 "NifU-like protein"
"JS1_D03_00726" 12635 "s59E5H2" 0.05860184 "NifU-like protein"
"JS1_D03_00726" 12635 "s63E6H2" 0.1335723 "NifU-like protein"
"JS1_D03_00728" 12637 "s59E13H2" 0.03274149 "Aspartokinase"
"JS1_D03_00728" 12637 "s59E25H2" 0.3098135 "Aspartokinase"
"JS1_D03_00728" 12637 "s59E5H2" 0.1731487 "Aspartokinase"
"JS1_D03_00728" 12637 "s63E6H2" 0.3443994 "Aspartokinase"
"JS1_D03_00729" 12638 "s59E13H2" 0 "hypothetical protein"
"JS1_D03_00729" 12638 "s59E25H2" 0 "hypothetical protein"
"JS1_D03_00729" 12638 "s59E5H2" 0 "hypothetical protein"
"JS1_D03_00729" 12638 "s63E6H2" 0.2288619 "hypothetical protein"
"JS1_D03_00730" 12639 "s59E13H2" 0 "hypothetical protein"
"JS1_D03_00730" 12639 "s59E25H2" 0 "hypothetical protein"
"JS1_D03_00730" 12639 "s59E5H2" 0 "hypothetical protein"
"JS1_D03_00730" 12639 "s63E6H2" 0.2169482 "hypothetical protein"
"JS1_D03_00732" 12641 "s59E13H2" 0.06308694 "DNA alkylation repair enzyme"
"JS1_D03_00732" 12641 "s59E25H2" 0 "DNA alkylation repair enzyme"
"JS1_D03_00732" 12641 "s59E5H2" 0.8936467 "DNA alkylation repair enzyme"

"JS1_D03_00732" 12641 "s63E6H2" 1.487299 "DNA alkylation repair enzyme"
"JS1_D03_00733" 12642 "s59E13H2" 0.0202746 "Isoprenylcysteine carboxyl methyltransferase (ICMT) family protein"
"JS1_D03_00733" 12642 "s59E25H2" 0 "Isoprenylcysteine carboxyl methyltransferase (ICMT) family protein"
"JS1_D03_00733" 12642 "s59E5H2" 1.062465 "Isoprenylcysteine carboxyl methyltransferase (ICMT) family protein"
"JS1_D03_00733" 12642 "s63E6H2" 0.7694205 "Isoprenylcysteine carboxyl methyltransferase (ICMT) family protein"
"JS1_D03_00734" 12643 "s59E13H2" 0 "hypothetical protein"
"JS1_D03_00734" 12643 "s59E25H2" 0 "hypothetical protein"
"JS1_D03_00734" 12643 "s59E5H2" 0.09706518 "hypothetical protein"
"JS1_D03_00734" 12643 "s63E6H2" 0.9665044 "hypothetical protein"
"JS1_D03_00735" 12644 "s59E13H2" 3.431129 "hypothetical protein"
"JS1_D03_00735" 12644 "s59E25H2" 0 "hypothetical protein"
"JS1_D03_00735" 12644 "s59E5H2" 0.1477624 "hypothetical protein"
"JS1_D03_00735" 12644 "s63E6H2" 0.3424577 "hypothetical protein"
"JS1_D03_00737" 12646 "s59E13H2" 0.04261401 "hypothetical protein"
"JS1_D03_00737" 12646 "s59E25H2" 0 "hypothetical protein"
"JS1_D03_00737" 12646 "s59E5H2" 0.04817917 "hypothetical protein"
"JS1_D03_00737" 12646 "s63E6H2" 0.7300876 "hypothetical protein"
"JS1_D03_00742" 12651 "s59E13H2" 0 "Na+/Pi-cotransporter"
"JS1_D03_00742" 12651 "s59E25H2" 0 "Na+/Pi-cotransporter"
"JS1_D03_00742" 12651 "s59E5H2" 0 "Na+/Pi-cotransporter"
"JS1_D03_00742" 12651 "s63E6H2" 0.4128407 "Na+/Pi-cotransporter"
"JS1_D03_00743" 12652 "s59E13H2" 0 "putative membrane protein"
"JS1_D03_00743" 12652 "s59E25H2" 0 "putative membrane protein"
"JS1_D03_00743" 12652 "s59E5H2" 0 "putative membrane protein"
"JS1_D03_00743" 12652 "s63E6H2" 0.4786871 "putative membrane protein"
"JS1_D03_00745" 12654 "s59E13H2" 0.01161847 "Deoxyguanosinetriphosphate triphosphohydrolase"
"JS1_D03_00745" 12654 "s59E25H2" 0 "Deoxyguanosinetriphosphate triphosphohydrolase"
"JS1_D03_00745" 12654 "s59E5H2" 0 "Deoxyguanosinetriphosphate triphosphohydrolase"
"JS1_D03_00745" 12654 "s63E6H2" 0 "Deoxyguanosinetriphosphate triphosphohydrolase"
"JS1_D03_00746" 12655 "s59E13H2" 0 "hypothetical protein"
"JS1_D03_00746" 12655 "s59E25H2" 0 "hypothetical protein"
"JS1_D03_00746" 12655 "s59E5H2" 0 "hypothetical protein"
"JS1_D03_00746" 12655 "s63E6H2" 0 "hypothetical protein"
"JS1_D03_00747" 12656 "s59E13H2" 0.02685426 "putative metallophosphoesterase YhaO"
"JS1_D03_00747" 12656 "s59E25H2" 0 "putative metallophosphoesterase YhaO"
"JS1_D03_00747" 12656 "s59E5H2" 0.006395586 "putative metallophosphoesterase YhaO"
"JS1_D03_00747" 12656 "s63E6H2" 0.04552461 "putative metallophosphoesterase YhaO"
"JS1_D03_00748" 12657 "s59E13H2" 0.01435443 "DNA double-strand break repair Rad50 ATPase"
"JS1_D03_00748" 12657 "s59E25H2" 0 "DNA double-strand break repair Rad50 ATPase"
"JS1_D03_00748" 12657 "s59E5H2" 0.01835078 "DNA double-strand break repair Rad50 ATPase"
"JS1_D03_00748" 12657 "s63E6H2" 0.01909711 "DNA double-strand break repair Rad50 ATPase"
"JS1_D03_00749" 12658 "s59E13H2" 0 "hypothetical protein"
"JS1_D03_00749" 12658 "s59E25H2" 0 "hypothetical protein"
"JS1_D03_00749" 12658 "s59E5H2" 0.005099465 "hypothetical protein"
"JS1_D03_00749" 12658 "s63E6H2" 0.03396411 "hypothetical protein"
"JS1_D03_00750" 12659 "s59E13H2" 0.06083328 "recombination protein F"
"JS1_D03_00750" 12659 "s59E25H2" 0 "recombination protein F"

"JS1_D03_00750" 12659 "s59E5H2" 0.0008237117 "recombination protein F"
"JS1_D03_00750" 12659 "s63E6H2" 0.1896155 "recombination protein F"
"JS1_D03_00755" 12664 "s59E13H2" 0 "hypothetical protein"
"JS1_D03_00755" 12664 "s59E25H2" 0 "hypothetical protein"
"JS1_D03_00755" 12664 "s59E5H2" 0 "hypothetical protein"
"JS1_D03_00755" 12664 "s63E6H2" 0.002897562 "hypothetical protein"
"JS1_D03_00756" 12665 "s59E13H2" 0 "hypothetical protein"
"JS1_D03_00756" 12665 "s59E25H2" 0 "hypothetical protein"
"JS1_D03_00756" 12665 "s59E5H2" 0 "hypothetical protein"
"JS1_D03_00756" 12665 "s63E6H2" 0 "hypothetical protein"
"JS1_D03_00757" 12666 "s59E13H2" 0 "hypothetical protein"
"JS1_D03_00757" 12666 "s59E25H2" 0 "hypothetical protein"
"JS1_D03_00757" 12666 "s59E5H2" 0 "hypothetical protein"
"JS1_D03_00757" 12666 "s63E6H2" 0 "hypothetical protein"
"JS1_D03_00758" 12667 "s59E13H2" 0 "hypothetical protein"
"JS1_D03_00758" 12667 "s59E25H2" 0 "hypothetical protein"
"JS1_D03_00758" 12667 "s59E5H2" 0 "hypothetical protein"
"JS1_D03_00758" 12667 "s63E6H2" 0 "hypothetical protein"
"JS1_D03_00760" 12669 "s59E13H2" 0 "hypothetical protein"
"JS1_D03_00760" 12669 "s59E25H2" 0 "hypothetical protein"
"JS1_D03_00760" 12669 "s59E5H2" 0 "hypothetical protein"
"JS1_D03_00760" 12669 "s63E6H2" 0 "hypothetical protein"
"JS1_D03_00763" 12672 "s59E13H2" 0 "ATP-dependent DNA helicase UvrD1"
"JS1_D03_00763" 12672 "s59E25H2" 0 "ATP-dependent DNA helicase UvrD1"
"JS1_D03_00763" 12672 "s59E5H2" 0 "ATP-dependent DNA helicase UvrD1"
"JS1_D03_00763" 12672 "s63E6H2" 0 "ATP-dependent DNA helicase UvrD1"
"JS1_D03_00764" 12673 "s59E13H2" 0.03555435 "Endonuclease III"
"JS1_D03_00764" 12673 "s59E25H2" 0 "Endonuclease III"
"JS1_D03_00764" 12673 "s59E5H2" 0.1078799 "Endonuclease III"
"JS1_D03_00764" 12673 "s63E6H2" 0.0754148 "Endonuclease III"
"JS1_D03_00765" 12674 "s59E13H2" 0.04770519 "Trehalose synthase"
"JS1_D03_00765" 12674 "s59E25H2" 0 "Trehalose synthase"
"JS1_D03_00765" 12674 "s59E5H2" 0.4596356 "Trehalose synthase"
"JS1_D03_00765" 12674 "s63E6H2" 0.1713767 "Trehalose synthase"
"JS1_D03_00766" 12675 "s59E13H2" 0 "N-acetylglucosamine repressor"
"JS1_D03_00766" 12675 "s59E25H2" 0 "N-acetylglucosamine repressor"
"JS1_D03_00766" 12675 "s59E5H2" 0.2098776 "N-acetylglucosamine repressor"
"JS1_D03_00766" 12675 "s63E6H2" 0.03453133 "N-acetylglucosamine repressor"
"JS1_D03_00767" 12676 "s59E13H2" 0.02874216 "Multiple sugar-binding protein precursor"
"JS1_D03_00767" 12676 "s59E25H2" 0 "Multiple sugar-binding protein precursor"
"JS1_D03_00767" 12676 "s59E5H2" 0.2741407 "Multiple sugar-binding protein precursor"
"JS1_D03_00767" 12676 "s63E6H2" 0.2428576 "Multiple sugar-binding protein precursor"
"JS1_D03_00768" 12677 "s59E13H2" 0.02923261 "Lactose transport system permease protein LacF"
"JS1_D03_00768" 12677 "s59E25H2" 0 "Lactose transport system permease protein LacF"
"JS1_D03_00768" 12677 "s59E5H2" 1.124255 "Lactose transport system permease protein LacF"
"JS1_D03_00768" 12677 "s63E6H2" 0.03552171 "Lactose transport system permease protein LacF"
"JS1_D03_00769" 12678 "s59E13H2" 0.004265451 "L-arabinose transport system permease protein AraQ"

"JS1_D03_00769" 12678 "s59E25H2" 0 "L-arabinose transport system permease protein AraQ"
"JS1_D03_00769" 12678 "s59E5H2" 0.2191502 "L-arabinose transport system permease protein AraQ"
"JS1_D03_00769" 12678 "s63E6H2" 0.03349611 "L-arabinose transport system permease protein AraQ"
"JS1_D03_00770" 12679 "s59E13H2" 0.09622495 "preprotein translocase subunit SecY"
"JS1_D03_00770" 12679 "s59E25H2" 0 "preprotein translocase subunit SecY"
"JS1_D03_00770" 12679 "s59E5H2" 0.03027293 "preprotein translocase subunit SecY"
"JS1_D03_00770" 12679 "s63E6H2" 0.214631 "preprotein translocase subunit SecY"
"JS1_D03_00776" 12685 "s59E13H2" 0.7343641 "LPS-assembly protein LptD"
"JS1_D03_00776" 12685 "s59E25H2" 0 "LPS-assembly protein LptD"
"JS1_D03_00776" 12685 "s59E5H2" 0.3837014 "LPS-assembly protein LptD"
"JS1_D03_00776" 12685 "s63E6H2" 1.326112 "LPS-assembly protein LptD"
"JS1_D03_00777" 12686 "s59E13H2" 0.03480174 "Lipopolysaccharide-assembly, LptC-related"
"JS1_D03_00777" 12686 "s59E25H2" 0 "Lipopolysaccharide-assembly, LptC-related"
"JS1_D03_00777" 12686 "s59E5H2" 0.2499932 "Lipopolysaccharide-assembly, LptC-related"
"JS1_D03_00777" 12686 "s63E6H2" 0.8710115 "Lipopolysaccharide-assembly, LptC-related"
"JS1_D03_00778" 12687 "s59E13H2" 0.1552618 "Clostripain precursor"
"JS1_D03_00778" 12687 "s59E25H2" 0 "Clostripain precursor"
"JS1_D03_00778" 12687 "s59E5H2" 0.01899999 "Clostripain precursor"
"JS1_D03_00778" 12687 "s63E6H2" 0.120356 "Clostripain precursor"
"JS1_D03_00780" 12689 "s59E13H2" 0.1268862 "Acetylglutamate kinase"
"JS1_D03_00780" 12689 "s59E25H2" 0 "Acetylglutamate kinase"
"JS1_D03_00780" 12689 "s59E5H2" 0.03092955 "Acetylglutamate kinase"
"JS1_D03_00780" 12689 "s63E6H2" 0.1584903 "Acetylglutamate kinase"
"JS1_D03_00781" 12690 "s59E13H2" 0.2796347 "Acetylornithine aminotransferase"
"JS1_D03_00781" 12690 "s59E25H2" 0 "Acetylornithine aminotransferase"
"JS1_D03_00781" 12690 "s59E5H2" 0.009770708 "Acetylornithine aminotransferase"
"JS1_D03_00781" 12690 "s63E6H2" 0.04047742 "Acetylornithine aminotransferase"
"JS1_D03_00782" 12691 "s59E13H2" 2.29767 "Argininosuccinate synthase"
"JS1_D03_00782" 12691 "s59E25H2" 0 "Argininosuccinate synthase"
"JS1_D03_00782" 12691 "s59E5H2" 0.1004587 "Argininosuccinate synthase"
"JS1_D03_00782" 12691 "s63E6H2" 0.1131558 "Argininosuccinate synthase"
"JS1_D03_00783" 12692 "s59E13H2" 0.4441376 "Argininosuccinate lyase 1"
"JS1_D03_00783" 12692 "s59E25H2" 0 "Argininosuccinate lyase 1"
"JS1_D03_00783" 12692 "s59E5H2" 0.05921933 "Argininosuccinate lyase 1"
"JS1_D03_00783" 12692 "s63E6H2" 0.1140761 "Argininosuccinate lyase 1"
"JS1_D03_00784" 12693 "s59E13H2" 0.164665 "L(+)-tartrate dehydratase subunit alpha"
"JS1_D03_00784" 12693 "s59E25H2" 0 "L(+)-tartrate dehydratase subunit alpha"
"JS1_D03_00784" 12693 "s59E5H2" 0 "L(+)-tartrate dehydratase subunit alpha"
"JS1_D03_00784" 12693 "s63E6H2" 0.009578833 "L(+)-tartrate dehydratase subunit alpha"
"JS1_D03_00785" 12694 "s59E13H2" 1.624927 "Fumarate hydratase class I, anaerobic"
"JS1_D03_00785" 12694 "s59E25H2" 0 "Fumarate hydratase class I, anaerobic"
"JS1_D03_00785" 12694 "s59E5H2" 0.003855763 "Fumarate hydratase class I, anaerobic"
"JS1_D03_00785" 12694 "s63E6H2" 0.03711105 "Fumarate hydratase class I, anaerobic"
"JS1_D03_00786" 12695 "s59E13H2" 0.580792 "NAD-dependent malic enzyme"
"JS1_D03_00786" 12695 "s59E25H2" 0 "NAD-dependent malic enzyme"
"JS1_D03_00786" 12695 "s59E5H2" 0.1464708 "NAD-dependent malic enzyme"
"JS1_D03_00786" 12695 "s63E6H2" 0.04550162 "NAD-dependent malic enzyme"

"JS1_D03_00789" 12698 "s59E13H2" 0 "50S ribosomal protein L3"
"JS1_D03_00789" 12698 "s59E25H2" 0 "50S ribosomal protein L3"
"JS1_D03_00789" 12698 "s59E5H2" 0 "50S ribosomal protein L3"
"JS1_D03_00789" 12698 "s63E6H2" 0.08894858 "50S ribosomal protein L3"
"JS1_D03_00790" 12699 "s59E13H2" 0 "50S ribosomal protein L4"
"JS1_D03_00790" 12699 "s59E25H2" 0 "50S ribosomal protein L4"
"JS1_D03_00790" 12699 "s59E5H2" 0 "50S ribosomal protein L4"
"JS1_D03_00790" 12699 "s63E6H2" 0.004550782 "50S ribosomal protein L4"
"JS1_D03_00791" 12700 "s59E13H2" 0.002567785 "Glutamate--tRNA ligase"
"JS1_D03_00791" 12700 "s59E25H2" 0 "Glutamate--tRNA ligase"
"JS1_D03_00791" 12700 "s59E5H2" 0.004296822 "Glutamate--tRNA ligase"
"JS1_D03_00791" 12700 "s63E6H2" 0.01404937 "Glutamate--tRNA ligase"
"JS1_D03_00794" 12703 "s59E13H2" 0 "Transcription elongation factor GreA"
"JS1_D03_00794" 12703 "s59E25H2" 0 "Transcription elongation factor GreA"
"JS1_D03_00794" 12703 "s59E5H2" 0.008324037 "Transcription elongation factor GreA"
"JS1_D03_00794" 12703 "s63E6H2" 0.01530521 "Transcription elongation factor GreA"
"JS1_D03_00795" 12704 "s59E13H2" 0 "Threonylcarbamoyl-AMP synthase"
"JS1_D03_00795" 12704 "s59E25H2" 0 "Threonylcarbamoyl-AMP synthase"
"JS1_D03_00795" 12704 "s59E5H2" 0 "Threonylcarbamoyl-AMP synthase"
"JS1_D03_00795" 12704 "s63E6H2" 0.02014545 "Threonylcarbamoyl-AMP synthase"
"JS1_D03_00796" 12705 "s59E13H2" 0.02596052 "Chorismate mutase AroH"
"JS1_D03_00796" 12705 "s59E25H2" 0 "Chorismate mutase AroH"
"JS1_D03_00796" 12705 "s59E5H2" 0.06279999 "Chorismate mutase AroH"
"JS1_D03_00796" 12705 "s63E6H2" 0.1077078 "Chorismate mutase AroH"
"JS1_D03_00797" 12706 "s59E13H2" 0.01047632 "Phospho-2-dehydro-3-deoxyheptonate aldolase"
"JS1_D03_00797" 12706 "s59E25H2" 0 "Phospho-2-dehydro-3-deoxyheptonate aldolase"
"JS1_D03_00797" 12706 "s59E5H2" 0.09884076 "Phospho-2-dehydro-3-deoxyheptonate aldolase"
"JS1_D03_00797" 12706 "s63E6H2" 0.288925 "Phospho-2-dehydro-3-deoxyheptonate aldolase"
"JS1_D03_00798" 12707 "s59E13H2" 0.00159062 "3-phosphoshikimate 1-carboxyvinyltransferase 1"
"JS1_D03_00798" 12707 "s59E25H2" 0 "3-phosphoshikimate 1-carboxyvinyltransferase 1"
"JS1_D03_00798" 12707 "s59E5H2" 0.1453275 "3-phosphoshikimate 1-carboxyvinyltransferase 1"
"JS1_D03_00798" 12707 "s63E6H2" 0.2565979 "3-phosphoshikimate 1-carboxyvinyltransferase 1"
"JS1_D03_00799" 12708 "s59E13H2" 0 "Shikimate dehydrogenase"
"JS1_D03_00799" 12708 "s59E25H2" 0 "Shikimate dehydrogenase"
"JS1_D03_00799" 12708 "s59E5H2" 0.02118557 "Shikimate dehydrogenase"
"JS1_D03_00799" 12708 "s63E6H2" 0.1202886 "Shikimate dehydrogenase"
"JS1_D03_00800" 12709 "s59E13H2" 0.001271257 "Chorismate synthase"
"JS1_D03_00800" 12709 "s59E25H2" 0 "Chorismate synthase"
"JS1_D03_00800" 12709 "s59E5H2" 0.04831068 "Chorismate synthase"
"JS1_D03_00800" 12709 "s63E6H2" 0.1585589 "Chorismate synthase"
"JS1_D03_00801" 12710 "s59E13H2" 0.02231755 "Shikimate kinase"
"JS1_D03_00801" 12710 "s59E25H2" 0 "Shikimate kinase"
"JS1_D03_00801" 12710 "s59E5H2" 0.149445 "Shikimate kinase"
"JS1_D03_00801" 12710 "s63E6H2" 0.205973 "Shikimate kinase"
"JS1_D03_00802" 12711 "s59E13H2" 0.03958396 "3-dehydroquinate synthase"
"JS1_D03_00802" 12711 "s59E25H2" 0 "3-dehydroquinate synthase"
"JS1_D03_00802" 12711 "s59E5H2" 0.3372692 "3-dehydroquinate synthase"

"JS1_D03_00802" 12711 "s63E6H2" 0.3341318 "3-dehydroquinate synthase"
"JS1_D03_00803" 12712 "s59E13H2" 0.02565637 "3-dehydroquinate dehydratase"
"JS1_D03_00803" 12712 "s59E25H2" 0 "3-dehydroquinate dehydratase"
"JS1_D03_00803" 12712 "s59E5H2" 0.09218645 "3-dehydroquinate dehydratase"
"JS1_D03_00803" 12712 "s63E6H2" 0.07979068 "3-dehydroquinate dehydratase"
"JS1_D03_00804" 12713 "s59E13H2" 0.001983898 "prephenate dehydrogenase"
"JS1_D03_00804" 12713 "s59E25H2" 0 "prephenate dehydrogenase"
"JS1_D03_00804" 12713 "s59E5H2" 0.01502324 "prephenate dehydrogenase"
"JS1_D03_00804" 12713 "s63E6H2" 0.04634327 "prephenate dehydrogenase"
"JS1_D03_00805" 12714 "s59E13H2" 0.5696051 "SsrA-binding protein"
"JS1_D03_00805" 12714 "s59E25H2" 0 "SsrA-binding protein"
"JS1_D03_00805" 12714 "s59E5H2" 0.3085295 "SsrA-binding protein"
"JS1_D03_00805" 12714 "s63E6H2" 0.2422217 "SsrA-binding protein"
"JS1_D03_00808" 12717 "s59E13H2" 0 "hypothetical protein"
"JS1_D03_00808" 12717 "s59E25H2" 0 "hypothetical protein"
"JS1_D03_00808" 12717 "s59E5H2" 0 "hypothetical protein"
"JS1_D03_00808" 12717 "s63E6H2" 0.00306406 "hypothetical protein"
"JS1_D03_00810" 12719 "s59E13H2" 0 "hypothetical protein"
"JS1_D03_00810" 12719 "s59E25H2" 0 "hypothetical protein"
"JS1_D03_00810" 12719 "s59E5H2" 0 "hypothetical protein"
"JS1_D03_00810" 12719 "s63E6H2" 0 "hypothetical protein"
"JS1_D03_00811" 12720 "s59E13H2" 0 "hypothetical protein"
"JS1_D03_00811" 12720 "s59E25H2" 0 "hypothetical protein"
"JS1_D03_00811" 12720 "s59E5H2" 0 "hypothetical protein"
"JS1_D03_00811" 12720 "s63E6H2" 0 "hypothetical protein"
"JS1_D03_00812" 12721 "s59E13H2" 0.003566971 "hypothetical protein"
"JS1_D03_00812" 12721 "s59E25H2" 0 "hypothetical protein"
"JS1_D03_00812" 12721 "s59E5H2" 0 "hypothetical protein"
"JS1_D03_00812" 12721 "s63E6H2" 0 "hypothetical protein"
"JS1_D03_00813" 12722 "s59E13H2" 0.045033 "hypothetical protein"
"JS1_D03_00813" 12722 "s59E25H2" 0 "hypothetical protein"
"JS1_D03_00813" 12722 "s59E5H2" 0 "hypothetical protein"
"JS1_D03_00813" 12722 "s63E6H2" 0 "hypothetical protein"
"JS1_D03_00814" 12723 "s59E13H2" 0.01598682 "hypothetical protein"
"JS1_D03_00814" 12723 "s59E25H2" 0 "hypothetical protein"
"JS1_D03_00814" 12723 "s59E5H2" 0 "hypothetical protein"
"JS1_D03_00814" 12723 "s63E6H2" 0.007970624 "hypothetical protein"
"JS1_D03_00815" 12724 "s59E13H2" 0.00290642 "ComEC family competence protein"
"JS1_D03_00815" 12724 "s59E25H2" 0 "ComEC family competence protein"
"JS1_D03_00815" 12724 "s59E5H2" 0 "ComEC family competence protein"
"JS1_D03_00815" 12724 "s63E6H2" 0.003654502 "ComEC family competence protein"
"JS1_D03_00816" 12725 "s59E13H2" 0 "hypothetical protein"
"JS1_D03_00816" 12725 "s59E25H2" 0 "hypothetical protein"
"JS1_D03_00816" 12725 "s59E5H2" 0 "hypothetical protein"
"JS1_D03_00816" 12725 "s63E6H2" 0.00938162 "hypothetical protein"
"JS1_D03_00817" 12726 "s59E13H2" 0 "hypothetical protein"
"JS1_D03_00817" 12726 "s59E25H2" 0 "hypothetical protein"

"JS1_D03_00817" 12726 "s59E5H2" 0 "hypothetical protein"
"JS1_D03_00817" 12726 "s63E6H2" 0 "hypothetical protein"
"JS1_D03_00818" 12727 "s59E13H2" 0 "hypothetical protein"
"JS1_D03_00818" 12727 "s59E25H2" 0 "hypothetical protein"
"JS1_D03_00818" 12727 "s59E5H2" 0 "hypothetical protein"
"JS1_D03_00818" 12727 "s63E6H2" 0 "hypothetical protein"
"JS1_D03_00819" 12728 "s59E13H2" 0 "Ion channel"
"JS1_D03_00819" 12728 "s59E25H2" 0 "Ion channel"
"JS1_D03_00819" 12728 "s59E5H2" 0 "Ion channel"
"JS1_D03_00819" 12728 "s63E6H2" 0.00259256 "Ion channel"
"JS1_D03_00820" 12729 "s59E13H2" 0 "GDP-mannose-dependent alpha-(1-6)-phosphatidylinositol monomannoside mannosyltransferase"
"JS1_D03_00820" 12729 "s59E25H2" 0 "GDP-mannose-dependent alpha-(1-6)-phosphatidylinositol monomannoside mannosyltransferase"
"JS1_D03_00820" 12729 "s59E5H2" 0 "GDP-mannose-dependent alpha-(1-6)-phosphatidylinositol monomannoside mannosyltransferase"
"JS1_D03_00820" 12729 "s63E6H2" 0.001304189 "GDP-mannose-dependent alpha-(1-6)-phosphatidylinositol monomannoside mannosyltransferase"
"JS1_D03_00821" 12730 "s59E13H2" 0 "Polysaccharide biosynthesis protein"
"JS1_D03_00821" 12730 "s59E25H2" 0 "Polysaccharide biosynthesis protein"
"JS1_D03_00821" 12730 "s59E5H2" 0.00317496 "Polysaccharide biosynthesis protein"
"JS1_D03_00821" 12730 "s63E6H2" 0.002765081 "Polysaccharide biosynthesis protein"
"JS1_D03_00822" 12731 "s59E13H2" 0 "coproporphyrinogen III oxidase"
"JS1_D03_00822" 12731 "s59E25H2" 0 "coproporphyrinogen III oxidase"
"JS1_D03_00822" 12731 "s59E5H2" 0 "coproporphyrinogen III oxidase"
"JS1_D03_00822" 12731 "s63E6H2" 0 "coproporphyrinogen III oxidase"
"JS1_D03_00823" 12732 "s59E13H2" 0 "hypothetical protein"
"JS1_D03_00823" 12732 "s59E25H2" 0 "hypothetical protein"
"JS1_D03_00823" 12732 "s59E5H2" 0 "hypothetical protein"
"JS1_D03_00823" 12732 "s63E6H2" 0.02603824 "hypothetical protein"
"JS1_D03_00825" 12734 "s59E13H2" 0 "hypothetical protein"
"JS1_D03_00825" 12734 "s59E25H2" 0 "hypothetical protein"
"JS1_D03_00825" 12734 "s59E5H2" 0 "hypothetical protein"
"JS1_D03_00825" 12734 "s63E6H2" 0 "hypothetical protein"
"JS1_D03_00827" 12736 "s59E13H2" 0 "hypothetical protein"
"JS1_D03_00827" 12736 "s59E25H2" 0 "hypothetical protein"
"JS1_D03_00827" 12736 "s59E5H2" 0 "hypothetical protein"
"JS1_D03_00827" 12736 "s63E6H2" 0 "hypothetical protein"
"JS1_D03_00830" 12739 "s59E13H2" 0 "Inner membrane ABC transporter permease protein YcjP"
"JS1_D03_00830" 12739 "s59E25H2" 0 "Inner membrane ABC transporter permease protein YcjP"
"JS1_D03_00830" 12739 "s59E5H2" 0.07077568 "Inner membrane ABC transporter permease protein YcjP"
"JS1_D03_00830" 12739 "s63E6H2" 0.2901731 "Inner membrane ABC transporter permease protein YcjP"
"JS1_D03_00831" 12740 "s59E13H2" 0 "hypothetical protein"
"JS1_D03_00831" 12740 "s59E25H2" 0 "hypothetical protein"
"JS1_D03_00831" 12740 "s59E5H2" 0.1077566 "hypothetical protein"
"JS1_D03_00831" 12740 "s63E6H2" 0.3053396 "hypothetical protein"
"JS1_D03_00832" 12741 "s59E13H2" 0.001262159 "Methionine--tRNA ligase"
"JS1_D03_00832" 12741 "s59E25H2" 0 "Methionine--tRNA ligase"
"JS1_D03_00832" 12741 "s59E5H2" 0 "Methionine--tRNA ligase"
"JS1_D03_00832" 12741 "s63E6H2" 0 "Methionine--tRNA ligase"
"JS1_D03_00833" 12742 "s59E13H2" 0 "Methionine--tRNA ligase"

"JS1_D03_00833" 12742 "s59E25H2" 0 "Methionine--tRNA ligase"
"JS1_D03_00833" 12742 "s59E5H2" 0 "Methionine--tRNA ligase"
"JS1_D03_00833" 12742 "s63E6H2" 0 "Methionine--tRNA ligase"
"JS1_D03_00834" 12743 "s59E13H2" 0.009949038 "Membrane-bound lytic murein transglycosylase C precursor"
"JS1_D03_00834" 12743 "s59E25H2" 0 "Membrane-bound lytic murein transglycosylase C precursor"
"JS1_D03_00834" 12743 "s59E5H2" 0.00251689 "Membrane-bound lytic murein transglycosylase C precursor"
"JS1_D03_00834" 12743 "s63E6H2" 0.01435156 "Membrane-bound lytic murein transglycosylase C precursor"
"JS1_D03_00836" 12745 "s59E13H2" 0.09160162 "Riboflavin transporter RibU"
"JS1_D03_00836" 12745 "s59E25H2" 0 "Riboflavin transporter RibU"
"JS1_D03_00836" 12745 "s59E5H2" 0.05745853 "Riboflavin transporter RibU"
"JS1_D03_00836" 12745 "s63E6H2" 0.07431388 "Riboflavin transporter RibU"
"JS1_D03_00837" 12746 "s59E13H2" 0.01346926 "Mannose-1-phosphate guanylyltransferase 1"
"JS1_D03_00837" 12746 "s59E25H2" 0 "Mannose-1-phosphate guanylyltransferase 1"
"JS1_D03_00837" 12746 "s59E5H2" 0.02607909 "Mannose-1-phosphate guanylyltransferase 1"
"JS1_D03_00837" 12746 "s63E6H2" 0.08301645 "Mannose-1-phosphate guanylyltransferase 1"
"JS1_D03_00839" 12748 "s59E13H2" 0.1610272 "S-adenosylmethionine synthase"
"JS1_D03_00839" 12748 "s59E25H2" 0 "S-adenosylmethionine synthase"
"JS1_D03_00839" 12748 "s59E5H2" 0.07034588 "S-adenosylmethionine synthase"
"JS1_D03_00839" 12748 "s63E6H2" 0.4117115 "S-adenosylmethionine synthase"
"JS1_D03_00840" 12749 "s59E13H2" 0.05951341 "Twitching mobility protein"
"JS1_D03_00840" 12749 "s59E25H2" 0 "Twitching mobility protein"
"JS1_D03_00840" 12749 "s59E5H2" 0.09367298 "Twitching mobility protein"
"JS1_D03_00840" 12749 "s63E6H2" 0.513989 "Twitching mobility protein"
"JS1_D03_00842" 12751 "s59E13H2" 0.03717129 "bacteriophage N4 adsorption protein B"
"JS1_D03_00842" 12751 "s59E25H2" 0 "bacteriophage N4 adsorption protein B"
"JS1_D03_00842" 12751 "s59E5H2" 0.0861488 "bacteriophage N4 adsorption protein B"
"JS1_D03_00842" 12751 "s63E6H2" 0.1915303 "bacteriophage N4 adsorption protein B"
"JS1_D03_00843" 12752 "s59E13H2" 0.006462987 "Twitching mobility protein"
"JS1_D03_00843" 12752 "s59E25H2" 0 "Twitching mobility protein"
"JS1_D03_00843" 12752 "s59E5H2" 0.06054523 "Twitching mobility protein"
"JS1_D03_00843" 12752 "s63E6H2" 0.1641982 "Twitching mobility protein"
"JS1_D03_00844" 12753 "s59E13H2" 0.01433931 "Histidine--tRNA ligase"
"JS1_D03_00844" 12753 "s59E25H2" 0 "Histidine--tRNA ligase"
"JS1_D03_00844" 12753 "s59E5H2" 0.04529177 "Histidine--tRNA ligase"
"JS1_D03_00844" 12753 "s63E6H2" 0.1103311 "Histidine--tRNA ligase"
"JS1_D03_00845" 12754 "s59E13H2" 0.0248115 "Asparagine--tRNA ligase"
"JS1_D03_00845" 12754 "s59E25H2" 0 "Asparagine--tRNA ligase"
"JS1_D03_00845" 12754 "s59E5H2" 0.06523471 "Asparagine--tRNA ligase"
"JS1_D03_00845" 12754 "s63E6H2" 0.1979053 "Asparagine--tRNA ligase"
"JS1_D03_00846" 12755 "s59E13H2" 0.04303653 "hypothetical protein"
"JS1_D03_00846" 12755 "s59E25H2" 0 "hypothetical protein"
"JS1_D03_00846" 12755 "s59E5H2" 0.1467095 "hypothetical protein"
"JS1_D03_00846" 12755 "s63E6H2" 0.1152553 "hypothetical protein"
"JS1_D03_00847" 12756 "s59E13H2" 0.02956223 "Aspartate--tRNA ligase"
"JS1_D03_00847" 12756 "s59E25H2" 0 "Aspartate--tRNA ligase"
"JS1_D03_00847" 12756 "s59E5H2" 0.04654509 "Aspartate--tRNA ligase"
"JS1_D03_00847" 12756 "s63E6H2" 0.1828242 "Aspartate--tRNA ligase"

"JS1_D03_00849" 12758 "s59E13H2" 0.004831284 "putative 5-formyltetrahydrofolate cyclo-ligase"
"JS1_D03_00849" 12758 "s59E25H2" 0.4066318 "putative 5-formyltetrahydrofolate cyclo-ligase"
"JS1_D03_00849" 12758 "s59E5H2" 0.006507327 "putative 5-formyltetrahydrofolate cyclo-ligase"
"JS1_D03_00849" 12758 "s63E6H2" 0.0678907 "putative 5-formyltetrahydrofolate cyclo-ligase"
"JS1_D03_00850" 12759 "s59E13H2" 0.02629313 "30S ribosomal protein S1"
"JS1_D03_00850" 12759 "s59E25H2" 0 "30S ribosomal protein S1"
"JS1_D03_00850" 12759 "s59E5H2" 0.02664396 "30S ribosomal protein S1"
"JS1_D03_00850" 12759 "s63E6H2" 0.2976811 "30S ribosomal protein S1"
"JS1_D03_00851" 12760 "s59E13H2" 0.005411264 "Type II secretion system protein E"
"JS1_D03_00851" 12760 "s59E25H2" 0 "Type II secretion system protein E"
"JS1_D03_00851" 12760 "s59E5H2" 0.06275754 "Type II secretion system protein E"
"JS1_D03_00851" 12760 "s63E6H2" 0.2730552 "Type II secretion system protein E"
"JS1_D03_00852" 12761 "s59E13H2" 0.009870843 "Alanine--tRNA ligase"
"JS1_D03_00852" 12761 "s59E25H2" 0.09410431 "Alanine--tRNA ligase"
"JS1_D03_00852" 12761 "s59E5H2" 0.0183684 "Alanine--tRNA ligase"
"JS1_D03_00852" 12761 "s63E6H2" 0.1107506 "Alanine--tRNA ligase"
"JS1_D03_00853" 12762 "s59E13H2" 0.006714079 "Putative Holliday junction resolvase"
"JS1_D03_00853" 12762 "s59E25H2" 0 "Putative Holliday junction resolvase"
"JS1_D03_00853" 12762 "s59E5H2" 0 "Putative Holliday junction resolvase"
"JS1_D03_00853" 12762 "s63E6H2" 0.06939115 "Putative Holliday junction resolvase"
"JS1_D03_00854" 12763 "s59E13H2" 0 "putative aminodeoxychorismate lyase"
"JS1_D03_00854" 12763 "s59E25H2" 0 "putative aminodeoxychorismate lyase"
"JS1_D03_00854" 12763 "s59E5H2" 0.004536393 "putative aminodeoxychorismate lyase"
"JS1_D03_00854" 12763 "s63E6H2" 0.01866484 "putative aminodeoxychorismate lyase"
"JS1_D03_00856" 12765 "s59E13H2" 0.03092238 "Methionine--tRNA ligase"
"JS1_D03_00856" 12765 "s59E25H2" 0 "Methionine--tRNA ligase"
"JS1_D03_00856" 12765 "s59E5H2" 0.05119124 "Methionine--tRNA ligase"
"JS1_D03_00856" 12765 "s63E6H2" 0.05984572 "Methionine--tRNA ligase"
"JS1_D03_00857" 12766 "s59E13H2" 0.003095485 "N5-glutamine S-adenosyl-L-methionine-dependent methyltransferase"
"JS1_D03_00857" 12766 "s59E25H2" 0 "N5-glutamine S-adenosyl-L-methionine-dependent methyltransferase"
"JS1_D03_00857" 12766 "s59E5H2" 0 "N5-glutamine S-adenosyl-L-methionine-dependent methyltransferase"
"JS1_D03_00857" 12766 "s63E6H2" 0.005389441 "N5-glutamine S-adenosyl-L-methionine-dependent methyltransferase"
"JS1_D03_00858" 12767 "s59E13H2" 0.004019322 "Ribosomal RNA small subunit methyltransferase I"
"JS1_D03_00858" 12767 "s59E25H2" 0 "Ribosomal RNA small subunit methyltransferase I"
"JS1_D03_00858" 12767 "s59E5H2" 0.03786477 "Ribosomal RNA small subunit methyltransferase I"
"JS1_D03_00858" 12767 "s63E6H2" 0.04060573 "Ribosomal RNA small subunit methyltransferase I"
"JS1_D03_00859" 12768 "s59E13H2" 0.01372609 "Release factor glutamine methyltransferase"
"JS1_D03_00859" 12768 "s59E25H2" 0 "Release factor glutamine methyltransferase"
"JS1_D03_00859" 12768 "s59E5H2" 0.07227163 "Release factor glutamine methyltransferase"
"JS1_D03_00859" 12768 "s63E6H2" 0.09550615 "Release factor glutamine methyltransferase"
"JS1_D03_00860" 12769 "s59E13H2" 0.007951581 "hypothetical protein"
"JS1_D03_00860" 12769 "s59E25H2" 0 "hypothetical protein"
"JS1_D03_00860" 12769 "s59E5H2" 0.08130603 "hypothetical protein"
"JS1_D03_00860" 12769 "s63E6H2" 0.04038589 "hypothetical protein"
"JS1_D03_00861" 12770 "s59E13H2" 0.0002622148 "DNA polymerase III subunit tau"
"JS1_D03_00861" 12770 "s59E25H2" 0 "DNA polymerase III subunit tau"
"JS1_D03_00861" 12770 "s59E5H2" 0.002299879 "DNA polymerase III subunit tau"

"JS1_D03_00861" 12770 "s63E6H2" 0.05011795 "DNA polymerase III subunit tau"
"JS1_D03_00862" 12771 "s59E13H2" 0 "Thymidylate kinase"
"JS1_D03_00862" 12771 "s59E25H2" 0 "Thymidylate kinase"
"JS1_D03_00862" 12771 "s59E5H2" 0.066852 "Thymidylate kinase"
"JS1_D03_00862" 12771 "s63E6H2" 0.04022601 "Thymidylate kinase"
"JS1_D03_00863" 12772 "s59E13H2" 0.01088635 "Methylated protein"
"JS1_D03_00863" 12772 "s59E25H2" 0 "Methylated protein"
"JS1_D03_00863" 12772 "s59E5H2" 0.4223997 "Methylated protein"
"JS1_D03_00863" 12772 "s63E6H2" 0.1211966 "Methylated protein"
"JS1_D03_00864" 12773 "s59E13H2" 0.003727542 "Tagatose-6-phosphate kinase"
"JS1_D03_00864" 12773 "s59E25H2" 0 "Tagatose-6-phosphate kinase"
"JS1_D03_00864" 12773 "s59E5H2" 0.05768805 "Tagatose-6-phosphate kinase"
"JS1_D03_00864" 12773 "s63E6H2" 0.08826373 "Tagatose-6-phosphate kinase"
"JS1_D03_00865" 12774 "s59E13H2" 0.005152291 "Rhomboid protease GluP"
"JS1_D03_00865" 12774 "s59E25H2" 0 "Rhomboid protease GluP"
"JS1_D03_00865" 12774 "s59E5H2" 0.1482593 "Rhomboid protease GluP"
"JS1_D03_00865" 12774 "s63E6H2" 0.08529018 "Rhomboid protease GluP"
"JS1_D03_00866" 12775 "s59E13H2" 0.009075907 "UDP-N-acetylglucosamine 2-epimerase"
"JS1_D03_00866" 12775 "s59E25H2" 0 "UDP-N-acetylglucosamine 2-epimerase"
"JS1_D03_00866" 12775 "s59E5H2" 0.3784278 "UDP-N-acetylglucosamine 2-epimerase"
"JS1_D03_00866" 12775 "s63E6H2" 0.1048336 "UDP-N-acetylglucosamine 2-epimerase"
"JS1_D03_00867" 12776 "s59E13H2" 0.006798935 "tRNA-specific adenosine deaminase"
"JS1_D03_00867" 12776 "s59E25H2" 0 "tRNA-specific adenosine deaminase"
"JS1_D03_00867" 12776 "s59E5H2" 0.2462431 "tRNA-specific adenosine deaminase"
"JS1_D03_00867" 12776 "s63E6H2" 0.08228948 "tRNA-specific adenosine deaminase"
"JS1_D03_00868" 12777 "s59E13H2" 0.02322947 "Uracil phosphoribosyltransferase"
"JS1_D03_00868" 12777 "s59E25H2" 0 "Uracil phosphoribosyltransferase"
"JS1_D03_00868" 12777 "s59E5H2" 0.5535149 "Uracil phosphoribosyltransferase"
"JS1_D03_00868" 12777 "s63E6H2" 0.2090021 "Uracil phosphoribosyltransferase"
"JS1_D03_00869" 12778 "s59E13H2" 0 "Putative sugar phosphate isomerase YwlF"
"JS1_D03_00869" 12778 "s59E25H2" 0 "Putative sugar phosphate isomerase YwlF"
"JS1_D03_00869" 12778 "s59E5H2" 0 "Putative sugar phosphate isomerase YwlF"
"JS1_D03_00869" 12778 "s63E6H2" 0.06881809 "Putative sugar phosphate isomerase YwlF"
"JS1_D03_00870" 12779 "s59E13H2" 0 "Low molecular weight protein-tyrosine-phosphatase YwlE"
"JS1_D03_00870" 12779 "s59E25H2" 0 "Low molecular weight protein-tyrosine-phosphatase YwlE"
"JS1_D03_00870" 12779 "s59E5H2" 0 "Low molecular weight protein-tyrosine-phosphatase YwlE"
"JS1_D03_00870" 12779 "s63E6H2" 0.06058537 "Low molecular weight protein-tyrosine-phosphatase YwlE"
"JS1_D03_00871" 12780 "s59E13H2" 0.007764028 "Release factor glutamine methyltransferase"
"JS1_D03_00871" 12780 "s59E25H2" 0 "Release factor glutamine methyltransferase"
"JS1_D03_00871" 12780 "s59E5H2" 0.01095003 "Release factor glutamine methyltransferase"
"JS1_D03_00871" 12780 "s63E6H2" 0.05248053 "Release factor glutamine methyltransferase"
"JS1_D03_00872" 12781 "s59E13H2" 0.06210026 "Peptide chain release factor 1"
"JS1_D03_00872" 12781 "s59E25H2" 0 "Peptide chain release factor 1"
"JS1_D03_00872" 12781 "s59E5H2" 0.03719162 "Peptide chain release factor 1"
"JS1_D03_00872" 12781 "s63E6H2" 0.1018778 "Peptide chain release factor 1"
"JS1_D03_00875" 12784 "s59E13H2" 0 "AAA-like domain protein"
"JS1_D03_00875" 12784 "s59E25H2" 0 "AAA-like domain protein"

"JS1_D03_00875" 12784 "s59E5H2" 0 "AAA-like domain protein"
"JS1_D03_00875" 12784 "s63E6H2" 0.001318368 "AAA-like domain protein"
"JS1_D03_00876" 12785 "s59E13H2" 0 "hypothetical protein"
"JS1_D03_00876" 12785 "s59E25H2" 0 "hypothetical protein"
"JS1_D03_00876" 12785 "s59E5H2" 0 "hypothetical protein"
"JS1_D03_00876" 12785 "s63E6H2" 0 "hypothetical protein"
"JS1_D03_00877" 12786 "s59E13H2" 0 "hypothetical protein"
"JS1_D03_00877" 12786 "s59E25H2" 0 "hypothetical protein"
"JS1_D03_00877" 12786 "s59E5H2" 0 "hypothetical protein"
"JS1_D03_00877" 12786 "s63E6H2" 0 "hypothetical protein"
"JS1_D03_00878" 12787 "s59E13H2" 0.01852999 "Transposase IS200 like protein"
"JS1_D03_00878" 12787 "s59E25H2" 0 "Transposase IS200 like protein"
"JS1_D03_00878" 12787 "s59E5H2" 0.07310172 "Transposase IS200 like protein"
"JS1_D03_00878" 12787 "s63E6H2" 0.6205662 "Transposase IS200 like protein"
"JS1_D03_00879" 12788 "s59E13H2" 0.08742969 "Naphthalene 1,2-dioxygenase/salicylate 5-hydroxylase systems, ferredoxin component"
"JS1_D03_00879" 12788 "s59E25H2" 0 "Naphthalene 1,2-dioxygenase/salicylate 5-hydroxylase systems, ferredoxin component"
"JS1_D03_00879" 12788 "s59E5H2" 0.01827504 "Naphthalene 1,2-dioxygenase/salicylate 5-hydroxylase systems, ferredoxin component"
"JS1_D03_00879" 12788 "s63E6H2" 0.2588304 "Naphthalene 1,2-dioxygenase/salicylate 5-hydroxylase systems, ferredoxin component"
"JS1_D03_00880" 12789 "s59E13H2" 0.02887075 "hypothetical protein"
"JS1_D03_00880" 12789 "s59E25H2" 0 "hypothetical protein"
"JS1_D03_00880" 12789 "s59E5H2" 0.03091977 "hypothetical protein"
"JS1_D03_00880" 12789 "s63E6H2" 0.03531274 "hypothetical protein"
"JS1_D03_00882" 12791 "s59E13H2" 9.002466 "Sodium-dependent dicarboxylate transporter SdcS"
"JS1_D03_00882" 12791 "s59E25H2" 0 "Sodium-dependent dicarboxylate transporter SdcS"
"JS1_D03_00882" 12791 "s59E5H2" 1.281996 "Sodium-dependent dicarboxylate transporter SdcS"
"JS1_D03_00882" 12791 "s63E6H2" 2.797765 "Sodium-dependent dicarboxylate transporter SdcS"
"JS1_D03_00883" 12792 "s59E13H2" 8.209836 "Beta-lactamase hydrolase-like protein"
"JS1_D03_00883" 12792 "s59E25H2" 0 "Beta-lactamase hydrolase-like protein"
"JS1_D03_00883" 12792 "s59E5H2" 2.136613 "Beta-lactamase hydrolase-like protein"
"JS1_D03_00883" 12792 "s63E6H2" 3.791791 "Beta-lactamase hydrolase-like protein"
"JS1_D03_00884" 12793 "s59E13H2" 9.641028 "putative inner membrane protein"
"JS1_D03_00884" 12793 "s59E25H2" 0 "putative inner membrane protein"
"JS1_D03_00884" 12793 "s59E5H2" 2.617411 "putative inner membrane protein"
"JS1_D03_00884" 12793 "s63E6H2" 3.922533 "putative inner membrane protein"
"JS1_D03_00885" 12794 "s59E13H2" 10.42227 "putative inner membrane protein"
"JS1_D03_00885" 12794 "s59E25H2" 0 "putative inner membrane protein"
"JS1_D03_00885" 12794 "s59E5H2" 2.765254 "putative inner membrane protein"
"JS1_D03_00885" 12794 "s63E6H2" 3.450068 "putative inner membrane protein"
"JS1_D03_00888" 12797 "s59E13H2" 0.2107026 "DNA alkylation repair enzyme"
"JS1_D03_00888" 12797 "s59E25H2" 0 "DNA alkylation repair enzyme"
"JS1_D03_00888" 12797 "s59E5H2" 0.01997395 "DNA alkylation repair enzyme"
"JS1_D03_00888" 12797 "s63E6H2" 0.7095497 "DNA alkylation repair enzyme"
"JS1_D03_00889" 12798 "s59E13H2" 0.6078392 "NADPH-dependent oxidoreductase"
"JS1_D03_00889" 12798 "s59E25H2" 0 "NADPH-dependent oxidoreductase"
"JS1_D03_00889" 12798 "s59E5H2" 0.02420568 "NADPH-dependent oxidoreductase"
"JS1_D03_00889" 12798 "s63E6H2" 0.9324695 "NADPH-dependent oxidoreductase"
"JS1_D03_00890" 12799 "s59E13H2" 0 "hypothetical protein"

"JS1_D03_00890" 12799 "s59E25H2" 0 "hypothetical protein"
"JS1_D03_00890" 12799 "s59E5H2" 0 "hypothetical protein"
"JS1_D03_00890" 12799 "s63E6H2" 0 "hypothetical protein"
"JS1_D03_00891" 12800 "s59E13H2" 0 "hypothetical protein"
"JS1_D03_00891" 12800 "s59E25H2" 0 "hypothetical protein"
"JS1_D03_00891" 12800 "s59E5H2" 0 "hypothetical protein"
"JS1_D03_00891" 12800 "s63E6H2" 0.00368934 "hypothetical protein"
"JS1_D03_00892" 12801 "s59E13H2" 0.6077088 "ADP-ribosyl-[dinitrogen reductase] glycohydrolase"
"JS1_D03_00892" 12801 "s59E25H2" 0 "ADP-ribosyl-[dinitrogen reductase] glycohydrolase"
"JS1_D03_00892" 12801 "s59E5H2" 0.01420785 "ADP-ribosyl-[dinitrogen reductase] glycohydrolase"
"JS1_D03_00892" 12801 "s63E6H2" 0.008321297 "ADP-ribosyl-[dinitrogen reductase] glycohydrolase"
"JS1_D03_00893" 12802 "s59E13H2" 0.2519232 "ubiquinone/menaquinone biosynthesis methyltransferase"
"JS1_D03_00893" 12802 "s59E25H2" 0 "ubiquinone/menaquinone biosynthesis methyltransferase"
"JS1_D03_00893" 12802 "s59E5H2" 0.08907064 "ubiquinone/menaquinone biosynthesis methyltransferase"
"JS1_D03_00893" 12802 "s63E6H2" 0.02479443 "ubiquinone/menaquinone biosynthesis methyltransferase"
"JS1_D03_00894" 12803 "s59E13H2" 0 "Trehalose/maltose-binding protein MalE precursor"
"JS1_D03_00894" 12803 "s59E25H2" 0 "Trehalose/maltose-binding protein MalE precursor"
"JS1_D03_00894" 12803 "s59E5H2" 0 "Trehalose/maltose-binding protein MalE precursor"
"JS1_D03_00894" 12803 "s63E6H2" 0.1339024 "Trehalose/maltose-binding protein MalE precursor"
"JS1_D03_00895" 12804 "s59E13H2" 0 "Trehalose/maltose transport system permease protein MalF"
"JS1_D03_00895" 12804 "s59E25H2" 0 "Trehalose/maltose transport system permease protein MalF"
"JS1_D03_00895" 12804 "s59E5H2" 0.02090842 "Trehalose/maltose transport system permease protein MalF"
"JS1_D03_00895" 12804 "s63E6H2" 0.1811819 "Trehalose/maltose transport system permease protein MalF"
"JS1_D03_00896" 12805 "s59E13H2" 0 "Trehalose/maltose transport system permease protein MalG"
"JS1_D03_00896" 12805 "s59E25H2" 0 "Trehalose/maltose transport system permease protein MalG"
"JS1_D03_00896" 12805 "s59E5H2" 0.0102719 "Trehalose/maltose transport system permease protein MalG"
"JS1_D03_00896" 12805 "s63E6H2" 0.1594432 "Trehalose/maltose transport system permease protein MalG"
"JS1_D03_00898" 12807 "s59E13H2" 0.07941888 "putative tautomerase"
"JS1_D03_00898" 12807 "s59E25H2" 0 "putative tautomerase"
"JS1_D03_00898" 12807 "s59E5H2" 0 "putative tautomerase"
"JS1_D03_00898" 12807 "s63E6H2" 1.862546 "putative tautomerase"
"JS1_D03_00899" 12808 "s59E13H2" 0.002163962 "hypothetical protein"
"JS1_D03_00899" 12808 "s59E25H2" 0 "hypothetical protein"
"JS1_D03_00899" 12808 "s59E5H2" 0.1266316 "hypothetical protein"
"JS1_D03_00899" 12808 "s63E6H2" 1.227341 "hypothetical protein"
"JS1_D03_00900" 12809 "s59E13H2" 1.065714 "Acetyl-CoA decarbonylase/synthase complex subunit gamma"
"JS1_D03_00900" 12809 "s59E25H2" 0 "Acetyl-CoA decarbonylase/synthase complex subunit gamma"
"JS1_D03_00900" 12809 "s59E5H2" 0.0844768 "Acetyl-CoA decarbonylase/synthase complex subunit gamma"
"JS1_D03_00900" 12809 "s63E6H2" 2.276639 "Acetyl-CoA decarbonylase/synthase complex subunit gamma"
"JS1_D03_00901" 12810 "s59E13H2" 1.106867 "Ferredoxin"
"JS1_D03_00901" 12810 "s59E25H2" 0 "Ferredoxin"
"JS1_D03_00901" 12810 "s59E5H2" 0.1161841 "Ferredoxin"
"JS1_D03_00901" 12810 "s63E6H2" 1.754361 "Ferredoxin"
"JS1_D03_00902" 12811 "s59E13H2" 0.02003371 "hypothetical protein"
"JS1_D03_00902" 12811 "s59E25H2" 0 "hypothetical protein"
"JS1_D03_00902" 12811 "s59E5H2" 0.02956945 "hypothetical protein"
"JS1_D03_00902" 12811 "s63E6H2" 0.227083 "hypothetical protein"

"JS1_D03_00903" 12812 "s59E13H2" 0.005761894 "hypothetical protein"
"JS1_D03_00903" 12812 "s59E25H2" 0 "hypothetical protein"
"JS1_D03_00903" 12812 "s59E5H2" 0.02391632 "hypothetical protein"
"JS1_D03_00903" 12812 "s63E6H2" 0.3831558 "hypothetical protein"
"JS1_D03_00904" 12813 "s59E13H2" 2.387085 "hypothetical protein"
"JS1_D03_00904" 12813 "s59E25H2" 0 "hypothetical protein"
"JS1_D03_00904" 12813 "s59E5H2" 0.05790818 "hypothetical protein"
"JS1_D03_00904" 12813 "s63E6H2" 0.05706378 "hypothetical protein"
"JS1_D03_00906" 12815 "s59E13H2" 0.001488012 "50S ribosomal protein L29"
"JS1_D03_00906" 12815 "s59E25H2" 0 "50S ribosomal protein L29"
"JS1_D03_00906" 12815 "s59E5H2" 0 "50S ribosomal protein L29"
"JS1_D03_00906" 12815 "s63E6H2" 0.03922805 "50S ribosomal protein L29"
"JS1_D03_00907" 12816 "s59E13H2" 0.004529223 "30S ribosomal protein S17"
"JS1_D03_00907" 12816 "s59E25H2" 0 "30S ribosomal protein S17"
"JS1_D03_00907" 12816 "s59E5H2" 0 "30S ribosomal protein S17"
"JS1_D03_00907" 12816 "s63E6H2" 0.06651874 "30S ribosomal protein S17"
"JS1_D03_00908" 12817 "s59E13H2" 0.009198724 "50S ribosomal protein L14"
"JS1_D03_00908" 12817 "s59E25H2" 0 "50S ribosomal protein L14"
"JS1_D03_00908" 12817 "s59E5H2" 0.01313634 "50S ribosomal protein L14"
"JS1_D03_00908" 12817 "s63E6H2" 0.08901464 "50S ribosomal protein L14"
"JS1_D03_00909" 12818 "s59E13H2" 0.0032167 "50S ribosomal protein L24"
"JS1_D03_00909" 12818 "s59E25H2" 0 "50S ribosomal protein L24"
"JS1_D03_00909" 12818 "s59E5H2" 0.01611436 "50S ribosomal protein L24"
"JS1_D03_00909" 12818 "s63E6H2" 0.1013032 "50S ribosomal protein L24"
"JS1_D03_00910" 12819 "s59E13H2" 0 "50S ribosomal protein L5"
"JS1_D03_00910" 12819 "s59E25H2" 0 "50S ribosomal protein L5"
"JS1_D03_00910" 12819 "s59E5H2" 0.01305945 "50S ribosomal protein L5"
"JS1_D03_00910" 12819 "s63E6H2" 0.07735333 "50S ribosomal protein L5"
"JS1_D03_00911" 12820 "s59E13H2" 0 "30S ribosomal protein S14 type Z"
"JS1_D03_00911" 12820 "s59E25H2" 0 "30S ribosomal protein S14 type Z"
"JS1_D03_00911" 12820 "s59E5H2" 0 "30S ribosomal protein S14 type Z"
"JS1_D03_00911" 12820 "s63E6H2" 0.009282356 "30S ribosomal protein S14 type Z"
"JS1_D03_00914" 12823 "s59E13H2" 0.001703851 "hypothetical protein"
"JS1_D03_00914" 12823 "s59E25H2" 0 "hypothetical protein"
"JS1_D03_00914" 12823 "s59E5H2" 0.006258483 "hypothetical protein"
"JS1_D03_00914" 12823 "s63E6H2" 0.05773911 "hypothetical protein"
"JS1_D03_00915" 12824 "s59E13H2" 0.0008406544 "LPS-assembly protein LptD"
"JS1_D03_00915" 12824 "s59E25H2" 0 "LPS-assembly protein LptD"
"JS1_D03_00915" 12824 "s59E5H2" 0.01698571 "LPS-assembly protein LptD"
"JS1_D03_00915" 12824 "s63E6H2" 0.187216 "LPS-assembly protein LptD"
"JS1_D03_00917" 12826 "s59E13H2" 0.002227963 "ATP-dependent helicase/deoxyribonuclease subunit B"
"JS1_D03_00917" 12826 "s59E25H2" 0 "ATP-dependent helicase/deoxyribonuclease subunit B"
"JS1_D03_00917" 12826 "s59E5H2" 0.06330899 "ATP-dependent helicase/deoxyribonuclease subunit B"
"JS1_D03_00917" 12826 "s63E6H2" 0.5992307 "ATP-dependent helicase/deoxyribonuclease subunit B"
"JS1_D03_00918" 12827 "s59E13H2" 0.001572989 "ATP-dependent helicase/nuclease subunit A"
"JS1_D03_00918" 12827 "s59E25H2" 0 "ATP-dependent helicase/nuclease subunit A"
"JS1_D03_00918" 12827 "s59E5H2" 0.1806982 "ATP-dependent helicase/nuclease subunit A"

"JS1_D03_00918" 12827 "s63E6H2" 0.5552767 "ATP-dependent helicase/nuclease subunit A"
"JS1_D03_00919" 12828 "s59E13H2" 0.04528248 "DegV domain-containing protein"
"JS1_D03_00919" 12828 "s59E25H2" 0 "DegV domain-containing protein"
"JS1_D03_00919" 12828 "s59E5H2" 0.3038736 "DegV domain-containing protein"
"JS1_D03_00919" 12828 "s63E6H2" 1.46318 "DegV domain-containing protein"
"JS1_D03_00920" 12829 "s59E13H2" 0 "protease TldD"
"JS1_D03_00920" 12829 "s59E25H2" 0 "protease TldD"
"JS1_D03_00920" 12829 "s59E5H2" 0 "protease TldD"
"JS1_D03_00920" 12829 "s63E6H2" 1.157671 "protease TldD"
"JS1_D03_00921" 12830 "s59E13H2" 0 "Putative modulator of DNA gyrase"
"JS1_D03_00921" 12830 "s59E25H2" 0 "Putative modulator of DNA gyrase"
"JS1_D03_00921" 12830 "s59E5H2" 0.004956607 "Putative modulator of DNA gyrase"
"JS1_D03_00921" 12830 "s63E6H2" 1.658971 "Putative modulator of DNA gyrase"
"JS1_D03_00922" 12831 "s59E13H2" 0 "HTH-type transcriptional repressor KstR2"
"JS1_D03_00922" 12831 "s59E25H2" 0 "HTH-type transcriptional repressor KstR2"
"JS1_D03_00922" 12831 "s59E5H2" 0 "HTH-type transcriptional repressor KstR2"
"JS1_D03_00922" 12831 "s63E6H2" 1.524492 "HTH-type transcriptional repressor KstR2"
"JS1_D03_00923" 12832 "s59E13H2" 0 "hypothetical protein"
"JS1_D03_00923" 12832 "s59E25H2" 0 "hypothetical protein"
"JS1_D03_00923" 12832 "s59E5H2" 0.001604081 "hypothetical protein"
"JS1_D03_00923" 12832 "s63E6H2" 1.415459 "hypothetical protein"
"JS1_D03_00924" 12833 "s59E13H2" 0.1003009 "hypothetical protein"
"JS1_D03_00924" 12833 "s59E25H2" 0 "hypothetical protein"
"JS1_D03_00924" 12833 "s59E5H2" 0.4846128 "hypothetical protein"
"JS1_D03_00924" 12833 "s63E6H2" 0.6842811 "hypothetical protein"
"JS1_D03_00925" 12834 "s59E13H2" 0.2038215 "MMPL family protein"
"JS1_D03_00925" 12834 "s59E25H2" 0 "MMPL family protein"
"JS1_D03_00925" 12834 "s59E5H2" 0.4379199 "MMPL family protein"
"JS1_D03_00925" 12834 "s63E6H2" 0.690365 "MMPL family protein"
"JS1_D03_00926" 12835 "s59E13H2" 0.001908037 "Protein-export membrane protein SecF"
"JS1_D03_00926" 12835 "s59E25H2" 0 "Protein-export membrane protein SecF"
"JS1_D03_00926" 12835 "s59E5H2" 0.1612314 "Protein-export membrane protein SecF"
"JS1_D03_00926" 12835 "s63E6H2" 0.2132535 "Protein-export membrane protein SecF"
"JS1_D03_00927" 12836 "s59E13H2" 0.003369958 "Radical SAM superfamily protein"
"JS1_D03_00927" 12836 "s59E25H2" 0 "Radical SAM superfamily protein"
"JS1_D03_00927" 12836 "s59E5H2" 0.06924546 "Radical SAM superfamily protein"
"JS1_D03_00927" 12836 "s63E6H2" 0.05986215 "Radical SAM superfamily protein"
"JS1_D03_00928" 12837 "s59E13H2" 0.1358425 "Biotin transporter BioY"
"JS1_D03_00928" 12837 "s59E25H2" 0.4588015 "Biotin transporter BioY"
"JS1_D03_00928" 12837 "s59E5H2" 1.383765 "Biotin transporter BioY"
"JS1_D03_00928" 12837 "s63E6H2" 4.322341 "Biotin transporter BioY"
"JS1_D03_00929" 12838 "s59E13H2" 0.02334421 "Bifunctional ligase/repressor BirA"
"JS1_D03_00929" 12838 "s59E25H2" 0 "Bifunctional ligase/repressor BirA"
"JS1_D03_00929" 12838 "s59E5H2" 0.3652774 "Bifunctional ligase/repressor BirA"
"JS1_D03_00929" 12838 "s63E6H2" 9.907234 "Bifunctional ligase/repressor BirA"
"JS1_D03_00930" 12839 "s59E13H2" 0.01859939 "hypothetical protein"
"JS1_D03_00930" 12839 "s59E25H2" 0 "hypothetical protein"

"JS1_D03_00930" 12839 "s59E5H2" 0.5634372 "hypothetical protein"
"JS1_D03_00930" 12839 "s63E6H2" 0.05766385 "hypothetical protein"
"JS1_D03_00931" 12840 "s59E13H2" 0.03866041 "Deoxyadenosine/deoxycytidine kinase"
"JS1_D03_00931" 12840 "s59E25H2" 0 "Deoxyadenosine/deoxycytidine kinase"
"JS1_D03_00931" 12840 "s59E5H2" 0.7443377 "Deoxyadenosine/deoxycytidine kinase"
"JS1_D03_00931" 12840 "s63E6H2" 5.922614 "Deoxyadenosine/deoxycytidine kinase"
"JS1_D03_00932" 12841 "s59E13H2" 0.1431969 "Deoxyguanosine kinase"
"JS1_D03_00932" 12841 "s59E25H2" 0 "Deoxyguanosine kinase"
"JS1_D03_00932" 12841 "s59E5H2" 1.305353 "Deoxyguanosine kinase"
"JS1_D03_00932" 12841 "s63E6H2" 6.822738 "Deoxyguanosine kinase"
"JS1_D03_00933" 12842 "s59E13H2" 0.09452658 "Octanoyltransferase LipM"
"JS1_D03_00933" 12842 "s59E25H2" 0 "Octanoyltransferase LipM"
"JS1_D03_00933" 12842 "s59E5H2" 0.4336309 "Octanoyltransferase LipM"
"JS1_D03_00933" 12842 "s63E6H2" 5.141081 "Octanoyltransferase LipM"
"JS1_D03_00934" 12843 "s59E13H2" 0.4907384 "Carbon starvation protein A"
"JS1_D03_00934" 12843 "s59E25H2" 0 "Carbon starvation protein A"
"JS1_D03_00934" 12843 "s59E5H2" 0.04830311 "Carbon starvation protein A"
"JS1_D03_00934" 12843 "s63E6H2" 0.1227669 "Carbon starvation protein A"
"JS1_D03_00937" 12846 "s59E13H2" 0.002482836 "hypothetical protein"
"JS1_D03_00937" 12846 "s59E25H2" 0 "hypothetical protein"
"JS1_D03_00937" 12846 "s59E5H2" 0 "hypothetical protein"
"JS1_D03_00937" 12846 "s63E6H2" 0 "hypothetical protein"
"JS1_D03_00939" 12848 "s59E13H2" 0.5607256 "Radical SAM superfamily protein"
"JS1_D03_00939" 12848 "s59E25H2" 0 "Radical SAM superfamily protein"
"JS1_D03_00939" 12848 "s59E5H2" 0.485917 "Radical SAM superfamily protein"
"JS1_D03_00939" 12848 "s63E6H2" 0.02367723 "Radical SAM superfamily protein"
"JS1_D03_00940" 12849 "s59E13H2" 0.2474891 "Septum site-determining protein MinD"
"JS1_D03_00940" 12849 "s59E25H2" 0 "Septum site-determining protein MinD"
"JS1_D03_00940" 12849 "s59E5H2" 0.7124935 "Septum site-determining protein MinD"
"JS1_D03_00940" 12849 "s63E6H2" 0.1030043 "Septum site-determining protein MinD"
"JS1_D03_00941" 12850 "s59E13H2" 0.2272633 "Heterodisulfide reductase subunit A-like protein"
"JS1_D03_00941" 12850 "s59E25H2" 0 "Heterodisulfide reductase subunit A-like protein"
"JS1_D03_00941" 12850 "s59E5H2" 0.2153293 "Heterodisulfide reductase subunit A-like protein"
"JS1_D03_00941" 12850 "s63E6H2" 0.265483 "Heterodisulfide reductase subunit A-like protein"
"JS1_D03_00942" 12851 "s59E13H2" 0.1348757 "Dinitrogenase iron-molybdenum cofactor"
"JS1_D03_00942" 12851 "s59E25H2" 0 "Dinitrogenase iron-molybdenum cofactor"
"JS1_D03_00942" 12851 "s59E5H2" 0.02275331 "Dinitrogenase iron-molybdenum cofactor"
"JS1_D03_00942" 12851 "s63E6H2" 0.0320115 "Dinitrogenase iron-molybdenum cofactor"
"JS1_D03_00943" 12852 "s59E13H2" 0.2710042 "Dinitrogenase iron-molybdenum cofactor"
"JS1_D03_00943" 12852 "s59E25H2" 0 "Dinitrogenase iron-molybdenum cofactor"
"JS1_D03_00943" 12852 "s59E5H2" 0.2488435 "Dinitrogenase iron-molybdenum cofactor"
"JS1_D03_00943" 12852 "s63E6H2" 0.08621388 "Dinitrogenase iron-molybdenum cofactor"
"JS1_D03_00944" 12853 "s59E13H2" 0.09036425 "hypothetical protein"
"JS1_D03_00944" 12853 "s59E25H2" 0 "hypothetical protein"
"JS1_D03_00944" 12853 "s59E5H2" 0.008401038 "hypothetical protein"
"JS1_D03_00944" 12853 "s63E6H2" 0.02496425 "hypothetical protein"
"JS1_D03_00945" 12854 "s59E13H2" 0.5922832 "Ferric uptake regulation protein"

"JS1_D03_00945" 12854 "s59E25H2" 0 "Ferric uptake regulation protein"
"JS1_D03_00945" 12854 "s59E5H2" 0.2830636 "Ferric uptake regulation protein"
"JS1_D03_00945" 12854 "s63E6H2" 0.1457256 "Ferric uptake regulation protein"
"JS1_D03_00946" 12855 "s59E13H2" 0.2833449 "hypothetical protein"
"JS1_D03_00946" 12855 "s59E25H2" 0 "hypothetical protein"
"JS1_D03_00946" 12855 "s59E5H2" 0.3761275 "hypothetical protein"
"JS1_D03_00946" 12855 "s63E6H2" 0.4676567 "hypothetical protein"
"JS1_D03_00947" 12856 "s59E13H2" 0.05020511 "FeoA domain protein"
"JS1_D03_00947" 12856 "s59E25H2" 0 "FeoA domain protein"
"JS1_D03_00947" 12856 "s59E5H2" 0.03803522 "FeoA domain protein"
"JS1_D03_00947" 12856 "s63E6H2" 0.3786046 "FeoA domain protein"
"JS1_D03_00948" 12857 "s59E13H2" 0.0706098 "Transcriptional regulator MntR"
"JS1_D03_00948" 12857 "s59E25H2" 0 "Transcriptional regulator MntR"
"JS1_D03_00948" 12857 "s59E5H2" 0.1305711 "Transcriptional regulator MntR"
"JS1_D03_00948" 12857 "s63E6H2" 0.3286106 "Transcriptional regulator MntR"
"JS1_D03_00949" 12858 "s59E13H2" 0.1101653 "Sulfur carrier protein ThiS"
"JS1_D03_00949" 12858 "s59E25H2" 0 "Sulfur carrier protein ThiS"
"JS1_D03_00949" 12858 "s59E5H2" 0.01310039 "Sulfur carrier protein ThiS"
"JS1_D03_00949" 12858 "s63E6H2" 0.1031637 "Sulfur carrier protein ThiS"
"JS1_D03_00950" 12859 "s59E13H2" 0.7562648 "Tungsten-containing aldehyde ferredoxin oxidoreductase"
"JS1_D03_00950" 12859 "s59E25H2" 0 "Tungsten-containing aldehyde ferredoxin oxidoreductase"
"JS1_D03_00950" 12859 "s59E5H2" 0.4496892 "Tungsten-containing aldehyde ferredoxin oxidoreductase"
"JS1_D03_00950" 12859 "s63E6H2" 1.139144 "Tungsten-containing aldehyde ferredoxin oxidoreductase"
"JS1_D03_00951" 12860 "s59E13H2" 1.449334 "Malate-2H(+)/Na(+)-lactate antiporter"
"JS1_D03_00951" 12860 "s59E25H2" 0 "Malate-2H(+)/Na(+)-lactate antiporter"
"JS1_D03_00951" 12860 "s59E5H2" 0.5064043 "Malate-2H(+)/Na(+)-lactate antiporter"
"JS1_D03_00951" 12860 "s63E6H2" 0.7465476 "Malate-2H(+)/Na(+)-lactate antiporter"
"JS1_D03_00952" 12861 "s59E13H2" 0.01873184 "Cyclic 2,3-diphosphoglycerate synthetase"
"JS1_D03_00952" 12861 "s59E25H2" 0 "Cyclic 2,3-diphosphoglycerate synthetase"
"JS1_D03_00952" 12861 "s59E5H2" 0.05790254 "Cyclic 2,3-diphosphoglycerate synthetase"
"JS1_D03_00952" 12861 "s63E6H2" 0.3003105 "Cyclic 2,3-diphosphoglycerate synthetase"
"JS1_D03_00953" 12862 "s59E13H2" 0.04616808 "5-aminovalerate aminotransferase DavT"
"JS1_D03_00953" 12862 "s59E25H2" 0 "5-aminovalerate aminotransferase DavT"
"JS1_D03_00953" 12862 "s59E5H2" 0.09976738 "5-aminovalerate aminotransferase DavT"
"JS1_D03_00953" 12862 "s63E6H2" 0.4121013 "5-aminovalerate aminotransferase DavT"
"JS1_D03_00954" 12863 "s59E13H2" 0.3654005 "Lysine 6-dehydrogenase"
"JS1_D03_00954" 12863 "s59E25H2" 0 "Lysine 6-dehydrogenase"
"JS1_D03_00954" 12863 "s59E5H2" 0.2787116 "Lysine 6-dehydrogenase"
"JS1_D03_00954" 12863 "s63E6H2" 0.988346 "Lysine 6-dehydrogenase"
"JS1_D03_00956" 12865 "s59E13H2" 0.1738898 "Lysine 6-dehydrogenase"
"JS1_D03_00956" 12865 "s59E25H2" 0 "Lysine 6-dehydrogenase"
"JS1_D03_00956" 12865 "s59E5H2" 0.1343278 "Lysine 6-dehydrogenase"
"JS1_D03_00956" 12865 "s63E6H2" 0.4966999 "Lysine 6-dehydrogenase"
"JS1_D03_00957" 12866 "s59E13H2" 0 "2-oxoglutarate synthase subunit KorC"
"JS1_D03_00957" 12866 "s59E25H2" 0 "2-oxoglutarate synthase subunit KorC"
"JS1_D03_00957" 12866 "s59E5H2" 0 "2-oxoglutarate synthase subunit KorC"
"JS1_D03_00957" 12866 "s63E6H2" 0.0261851 "2-oxoglutarate synthase subunit KorC"

"JS1_D03_00958" 12867 "s59E13H2" 0 "2-oxoglutarate synthase subunit KorB"
"JS1_D03_00958" 12867 "s59E25H2" 0 "2-oxoglutarate synthase subunit KorB"
"JS1_D03_00958" 12867 "s59E5H2" 0 "2-oxoglutarate synthase subunit KorB"
"JS1_D03_00958" 12867 "s63E6H2" 0.02053152 "2-oxoglutarate synthase subunit KorB"
"JS1_D03_00959" 12868 "s59E13H2" 0.0023246 "2-oxoglutarate synthase subunit KorA"
"JS1_D03_00959" 12868 "s59E25H2" 0 "2-oxoglutarate synthase subunit KorA"
"JS1_D03_00959" 12868 "s59E5H2" 0 "2-oxoglutarate synthase subunit KorA"
"JS1_D03_00959" 12868 "s63E6H2" 0.04805079 "2-oxoglutarate synthase subunit KorA"
"JS1_D03_00960" 12869 "s59E13H2" 0 "hypothetical protein"
"JS1_D03_00960" 12869 "s59E25H2" 0 "hypothetical protein"
"JS1_D03_00960" 12869 "s59E5H2" 0 "hypothetical protein"
"JS1_D03_00960" 12869 "s63E6H2" 0 "hypothetical protein"
"JS1_D03_00961" 12870 "s59E13H2" 0 "ComEC family competence protein"
"JS1_D03_00961" 12870 "s59E25H2" 0 "ComEC family competence protein"
"JS1_D03_00961" 12870 "s59E5H2" 0 "ComEC family competence protein"
"JS1_D03_00961" 12870 "s63E6H2" 0 "ComEC family competence protein"
"JS1_D03_00962" 12871 "s59E13H2" 0 "hypothetical protein"
"JS1_D03_00962" 12871 "s59E25H2" 0 "hypothetical protein"
"JS1_D03_00962" 12871 "s59E5H2" 0 "hypothetical protein"
"JS1_D03_00962" 12871 "s63E6H2" 0 "hypothetical protein"
"JS1_D03_00963" 12872 "s59E13H2" 0 "Tetratricopeptide repeat protein"
"JS1_D03_00963" 12872 "s59E25H2" 0 "Tetratricopeptide repeat protein"
"JS1_D03_00963" 12872 "s59E5H2" 0 "Tetratricopeptide repeat protein"
"JS1_D03_00963" 12872 "s63E6H2" 0.004177389 "Tetratricopeptide repeat protein"
"JS1_D03_00964" 12873 "s59E13H2" 0 "hypothetical protein"
"JS1_D03_00964" 12873 "s59E25H2" 0 "hypothetical protein"
"JS1_D03_00964" 12873 "s59E5H2" 0 "hypothetical protein"
"JS1_D03_00964" 12873 "s63E6H2" 0 "hypothetical protein"
"JS1_D03_00966" 12875 "s59E13H2" 0 "N-6 DNA Methylase"
"JS1_D03_00966" 12875 "s59E25H2" 0 "N-6 DNA Methylase"
"JS1_D03_00966" 12875 "s59E5H2" 0 "N-6 DNA Methylase"
"JS1_D03_00966" 12875 "s63E6H2" 0 "N-6 DNA Methylase"
"JS1_D03_00967" 12876 "s59E13H2" 0 "hypothetical protein"
"JS1_D03_00967" 12876 "s59E25H2" 0 "hypothetical protein"
"JS1_D03_00967" 12876 "s59E5H2" 0 "hypothetical protein"
"JS1_D03_00967" 12876 "s63E6H2" 0.001691568 "hypothetical protein"
"JS1_D03_00970" 12879 "s59E13H2" 0.003930193 "hypothetical protein"
"JS1_D03_00970" 12879 "s59E25H2" 0 "hypothetical protein"
"JS1_D03_00970" 12879 "s59E5H2" 0.00780931 "hypothetical protein"
"JS1_D03_00970" 12879 "s63E6H2" 0.004411192 "hypothetical protein"
"JS1_D03_00971" 12880 "s59E13H2" 0.0236697 "Replicative DNA helicase"
"JS1_D03_00971" 12880 "s59E25H2" 0 "Replicative DNA helicase"
"JS1_D03_00971" 12880 "s59E5H2" 0.01801304 "Replicative DNA helicase"
"JS1_D03_00971" 12880 "s63E6H2" 0.0006659629 "Replicative DNA helicase"
"JS1_D03_00972" 12881 "s59E13H2" 0.01434398 "Helix-turn-helix"
"JS1_D03_00972" 12881 "s59E25H2" 0 "Helix-turn-helix"
"JS1_D03_00972" 12881 "s59E5H2" 0.01628378 "Helix-turn-helix"

"JS1_D03_00972" 12881 "s63E6H2" 0 "Helix-turn-helix"
"JS1_D03_00974" 12883 "s59E13H2" 0.2116071 "hypothetical protein"
"JS1_D03_00974" 12883 "s59E25H2" 0 "hypothetical protein"
"JS1_D03_00974" 12883 "s59E5H2" 0.6182015 "hypothetical protein"
"JS1_D03_00974" 12883 "s63E6H2" 2.68848 "hypothetical protein"
"JS1_D03_00975" 12884 "s59E13H2" 0 "Serine/threonine-protein kinase PK-1"
"JS1_D03_00975" 12884 "s59E25H2" 0.2820513 "Serine/threonine-protein kinase PK-1"
"JS1_D03_00975" 12884 "s59E5H2" 0 "Serine/threonine-protein kinase PK-1"
"JS1_D03_00975" 12884 "s63E6H2" 2.652229 "Serine/threonine-protein kinase PK-1"
"JS1_D03_00976" 12885 "s59E13H2" 0.004632065 "Ribosomal RNA small subunit methyltransferase B"
"JS1_D03_00976" 12885 "s59E25H2" 0.1795977 "Ribosomal RNA small subunit methyltransferase B"
"JS1_D03_00976" 12885 "s59E5H2" 0.008874526 "Ribosomal RNA small subunit methyltransferase B"
"JS1_D03_00976" 12885 "s63E6H2" 3.59597 "Ribosomal RNA small subunit methyltransferase B"
"JS1_D03_00977" 12886 "s59E13H2" 0.02228252 "Putative neutral zinc metallopeptidase"
"JS1_D03_00977" 12886 "s59E25H2" 0.3185841 "Putative neutral zinc metallopeptidase"
"JS1_D03_00977" 12886 "s59E5H2" 0.1482967 "Putative neutral zinc metallopeptidase"
"JS1_D03_00977" 12886 "s63E6H2" 4.176624 "Putative neutral zinc metallopeptidase"
"JS1_D03_00978" 12887 "s59E13H2" 0.008479611 "Methionyl-tRNA formyltransferase"
"JS1_D03_00978" 12887 "s59E25H2" 0 "Methionyl-tRNA formyltransferase"
"JS1_D03_00978" 12887 "s59E5H2" 0.005033549 "Methionyl-tRNA formyltransferase"
"JS1_D03_00978" 12887 "s63E6H2" 2.748843 "Methionyl-tRNA formyltransferase"
"JS1_D03_00979" 12888 "s59E13H2" 0 "Peptide deformylase 1"
"JS1_D03_00979" 12888 "s59E25H2" 0 "Peptide deformylase 1"
"JS1_D03_00979" 12888 "s59E5H2" 0 "Peptide deformylase 1"
"JS1_D03_00979" 12888 "s63E6H2" 2.81408 "Peptide deformylase 1"
"JS1_D03_00980" 12889 "s59E13H2" 0.09260252 "Primosomal protein N'"
"JS1_D03_00980" 12889 "s59E25H2" 0.2958215 "Primosomal protein N'"
"JS1_D03_00980" 12889 "s59E5H2" 0.5216391 "Primosomal protein N'"
"JS1_D03_00980" 12889 "s63E6H2" 2.446123 "Primosomal protein N'"
"JS1_D03_00981" 12890 "s59E13H2" 0.002609804 "Coenzyme A biosynthesis bifunctional protein CoaBC"
"JS1_D03_00981" 12890 "s59E25H2" 0.05862647 "Coenzyme A biosynthesis bifunctional protein CoaBC"
"JS1_D03_00981" 12890 "s59E5H2" 0.06450749 "Coenzyme A biosynthesis bifunctional protein CoaBC"
"JS1_D03_00981" 12890 "s63E6H2" 0.09080239 "Coenzyme A biosynthesis bifunctional protein CoaBC"
"JS1_D03_00982" 12891 "s59E13H2" 0 "DNA-directed RNA polymerase subunit omega"
"JS1_D03_00982" 12891 "s59E25H2" 0 "DNA-directed RNA polymerase subunit omega"
"JS1_D03_00982" 12891 "s59E5H2" 0.005014829 "DNA-directed RNA polymerase subunit omega"
"JS1_D03_00982" 12891 "s63E6H2" 0.02652191 "DNA-directed RNA polymerase subunit omega"
"JS1_D03_00983" 12892 "s59E13H2" 0 "Guanylate kinase"
"JS1_D03_00983" 12892 "s59E25H2" 0 "Guanylate kinase"
"JS1_D03_00983" 12892 "s59E5H2" 0.01789736 "Guanylate kinase"
"JS1_D03_00983" 12892 "s63E6H2" 0.0791609 "Guanylate kinase"
"JS1_D03_00984" 12893 "s59E13H2" 0 "hypothetical protein"
"JS1_D03_00984" 12893 "s59E25H2" 0 "hypothetical protein"
"JS1_D03_00984" 12893 "s59E5H2" 0.01201508 "hypothetical protein"
"JS1_D03_00984" 12893 "s63E6H2" 0.0927415 "hypothetical protein"
"JS1_D03_00985" 12894 "s59E13H2" 0.001985044 "hypothetical protein"
"JS1_D03_00985" 12894 "s59E25H2" 0 "hypothetical protein"

"JS1_D03_00985" 12894 "s59E5H2" 0.003701309 "hypothetical protein"
"JS1_D03_00985" 12894 "s63E6H2" 0.1248753 "hypothetical protein"
"JS1_D03_00986" 12895 "s59E13H2" 0.03867507 "hypothetical protein"
"JS1_D03_00986" 12895 "s59E25H2" 0 "hypothetical protein"
"JS1_D03_00986" 12895 "s59E5H2" 0.1671144 "hypothetical protein"
"JS1_D03_00986" 12895 "s63E6H2" 0.7705209 "hypothetical protein"
"JS1_D03_00987" 12896 "s59E13H2" 0.01208628 "Ribosomal large subunit pseudouridine synthase D"
"JS1_D03_00987" 12896 "s59E25H2" 0 "Ribosomal large subunit pseudouridine synthase D"
"JS1_D03_00987" 12896 "s59E5H2" 0.06416708 "Ribosomal large subunit pseudouridine synthase D"
"JS1_D03_00987" 12896 "s63E6H2" 0.6779846 "Ribosomal large subunit pseudouridine synthase D"
"JS1_D03_00988" 12897 "s59E13H2" 0 "Prolipoprotein diacylglyceryl transferase"
"JS1_D03_00988" 12897 "s59E25H2" 0 "Prolipoprotein diacylglyceryl transferase"
"JS1_D03_00988" 12897 "s59E5H2" 0.006035025 "Prolipoprotein diacylglyceryl transferase"
"JS1_D03_00988" 12897 "s63E6H2" 0.1300095 "Prolipoprotein diacylglyceryl transferase"
"JS1_D03_00989" 12898 "s59E13H2" 0.4798462 "Lipoprotein signal peptidase"
"JS1_D03_00989" 12898 "s59E25H2" 0.1325967 "Lipoprotein signal peptidase"
"JS1_D03_00989" 12898 "s59E5H2" 0.3832298 "Lipoprotein signal peptidase"
"JS1_D03_00989" 12898 "s63E6H2" 0.5821116 "Lipoprotein signal peptidase"
"JS1_D03_00990" 12899 "s59E13H2" 0.4214852 "Isoleucine--tRNA ligase"
"JS1_D03_00990" 12899 "s59E25H2" 0 "Isoleucine--tRNA ligase"
"JS1_D03_00990" 12899 "s59E5H2" 1.82692 "Isoleucine--tRNA ligase"
"JS1_D03_00990" 12899 "s63E6H2" 1.48669 "Isoleucine--tRNA ligase"
"JS1_D03_00992" 12901 "s59E13H2" 0 "hypothetical protein"
"JS1_D03_00992" 12901 "s59E25H2" 0 "hypothetical protein"
"JS1_D03_00992" 12901 "s59E5H2" 0 "hypothetical protein"
"JS1_D03_00992" 12901 "s63E6H2" 0.477175 "hypothetical protein"
"JS1_D03_00993" 12902 "s59E13H2" 0.1434475 "Septum site-determining protein DivIVA"
"JS1_D03_00993" 12902 "s59E25H2" 0 "Septum site-determining protein DivIVA"
"JS1_D03_00993" 12902 "s59E5H2" 0.3530915 "Septum site-determining protein DivIVA"
"JS1_D03_00993" 12902 "s63E6H2" 0.6673135 "Septum site-determining protein DivIVA"
"JS1_D03_00994" 12903 "s59E13H2" 0.1696134 "YGGT family protein"
"JS1_D03_00994" 12903 "s59E25H2" 0 "YGGT family protein"
"JS1_D03_00994" 12903 "s59E5H2" 0.4793869 "YGGT family protein"
"JS1_D03_00994" 12903 "s63E6H2" 0.9691513 "YGGT family protein"
"JS1_D03_00995" 12904 "s59E13H2" 0.09945384 "hypothetical protein"
"JS1_D03_00995" 12904 "s59E25H2" 0 "hypothetical protein"
"JS1_D03_00995" 12904 "s59E5H2" 0.1066387 "hypothetical protein"
"JS1_D03_00995" 12904 "s63E6H2" 0.8942396 "hypothetical protein"
"JS1_D03_00996" 12905 "s59E13H2" 0.1138308 "hypothetical protein"
"JS1_D03_00996" 12905 "s59E25H2" 0 "hypothetical protein"
"JS1_D03_00996" 12905 "s59E5H2" 0.470093 "hypothetical protein"
"JS1_D03_00996" 12905 "s63E6H2" 0.6348035 "hypothetical protein"
"JS1_D03_00997" 12906 "s59E13H2" 0.4892194 "High-affinity branched-chain amino acid transport ATP-binding protein LivF"
"JS1_D03_00997" 12906 "s59E25H2" 0 "High-affinity branched-chain amino acid transport ATP-binding protein LivF"
"JS1_D03_00997" 12906 "s59E5H2" 1.013563 "High-affinity branched-chain amino acid transport ATP-binding protein LivF"
"JS1_D03_00997" 12906 "s63E6H2" 1.526833 "High-affinity branched-chain amino acid transport ATP-binding protein LivF"
"JS1_D03_00998" 12907 "s59E13H2" 0.3524002 "putative branched-chain amino acid transport ATP-binding protein LivG"

"JS1_D03_00998" 12907 "s59E25H2" 0 "putative branched-chain amino acid transport ATP-binding protein LivG"
"JS1_D03_00998" 12907 "s59E5H2" 0.7852534 "putative branched-chain amino acid transport ATP-binding protein LivG"
"JS1_D03_00998" 12907 "s63E6H2" 1.244501 "putative branched-chain amino acid transport ATP-binding protein LivG"
"JS1_D03_00999" 12908 "s59E13H2" 0.07956112 "leucine/isoleucine/valine transporter permease subunit"
"JS1_D03_00999" 12908 "s59E25H2" 0 "leucine/isoleucine/valine transporter permease subunit"
"JS1_D03_00999" 12908 "s59E5H2" 0.6249063 "leucine/isoleucine/valine transporter permease subunit"
"JS1_D03_00999" 12908 "s63E6H2" 1.003612 "leucine/isoleucine/valine transporter permease subunit"
"JS1_D03_01000" 12909 "s59E13H2" 0.04782733 "High-affinity branched-chain amino acid transport system permease protein LivH"
"JS1_D03_01000" 12909 "s59E25H2" 0 "High-affinity branched-chain amino acid transport system permease protein LivH"
"JS1_D03_01000" 12909 "s59E5H2" 0.4707269 "High-affinity branched-chain amino acid transport system permease protein LivH"
"JS1_D03_01000" 12909 "s63E6H2" 1.864249 "High-affinity branched-chain amino acid transport system permease protein LivH"
"JS1_D03_01001" 12910 "s59E13H2" 0.05445865 "Leucine-specific-binding protein precursor"
"JS1_D03_01001" 12910 "s59E25H2" 0 "Leucine-specific-binding protein precursor"
"JS1_D03_01001" 12910 "s59E5H2" 0.1045358 "Leucine-specific-binding protein precursor"
"JS1_D03_01001" 12910 "s63E6H2" 1.891112 "Leucine-specific-binding protein precursor"
"JS1_D03_01002" 12911 "s59E13H2" 0 "Sarcosine oxidase subunit beta"
"JS1_D03_01002" 12911 "s59E25H2" 0 "Sarcosine oxidase subunit beta"
"JS1_D03_01002" 12911 "s59E5H2" 0.01523563 "Sarcosine oxidase subunit beta"
"JS1_D03_01002" 12911 "s63E6H2" 0.2477178 "Sarcosine oxidase subunit beta"
"JS1_D03_01003" 12912 "s59E13H2" 0.00466254 "Hydrogen cyanide synthase subunit HcnB"
"JS1_D03_01003" 12912 "s59E25H2" 0 "Hydrogen cyanide synthase subunit HcnB"
"JS1_D03_01003" 12912 "s59E5H2" 0.09406368 "Hydrogen cyanide synthase subunit HcnB"
"JS1_D03_01003" 12912 "s63E6H2" 0.6801233 "Hydrogen cyanide synthase subunit HcnB"
"JS1_D03_01004" 12913 "s59E13H2" 0.02498706 "ferredoxin"
"JS1_D03_01004" 12913 "s59E25H2" 0 "ferredoxin"
"JS1_D03_01004" 12913 "s59E5H2" 0.08030993 "ferredoxin"
"JS1_D03_01004" 12913 "s63E6H2" 0.7981814 "ferredoxin"
"JS1_D03_01005" 12914 "s59E13H2" 0.07806599 "Hydrogen cyanide synthase subunit HcnB"
"JS1_D03_01005" 12914 "s59E25H2" 0 "Hydrogen cyanide synthase subunit HcnB"
"JS1_D03_01005" 12914 "s59E5H2" 0.08290091 "Hydrogen cyanide synthase subunit HcnB"
"JS1_D03_01005" 12914 "s63E6H2" 0.737062 "Hydrogen cyanide synthase subunit HcnB"
"JS1_D03_01006" 12915 "s59E13H2" 0.0212532 "Hydrogen cyanide synthase subunit HcnA"
"JS1_D03_01006" 12915 "s59E25H2" 0 "Hydrogen cyanide synthase subunit HcnA"
"JS1_D03_01006" 12915 "s59E5H2" 0.06458203 "Hydrogen cyanide synthase subunit HcnA"
"JS1_D03_01006" 12915 "s63E6H2" 0.1164402 "Hydrogen cyanide synthase subunit HcnA"
"JS1_D03_01007" 12916 "s59E13H2" 0.0123235 "Laccase domain protein"
"JS1_D03_01007" 12916 "s59E25H2" 0 "Laccase domain protein"
"JS1_D03_01007" 12916 "s59E5H2" 0.1436344 "Laccase domain protein"
"JS1_D03_01007" 12916 "s63E6H2" 0.2103008 "Laccase domain protein"
"JS1_D03_01009" 12918 "s59E13H2" 0.2576113 "molybdenum cofactor biosynthesis protein A"
"JS1_D03_01009" 12918 "s59E25H2" 0 "molybdenum cofactor biosynthesis protein A"
"JS1_D03_01009" 12918 "s59E5H2" 0.0487036 "molybdenum cofactor biosynthesis protein A"
"JS1_D03_01009" 12918 "s63E6H2" 0.4818029 "molybdenum cofactor biosynthesis protein A"
"JS1_D03_01010" 12919 "s59E13H2" 0.3370871 "hypothetical protein"
"JS1_D03_01010" 12919 "s59E25H2" 0 "hypothetical protein"
"JS1_D03_01010" 12919 "s59E5H2" 0.6352456 "hypothetical protein"
"JS1_D03_01010" 12919 "s63E6H2" 0.6790924 "hypothetical protein"

"JS1_D03_01011" 12920 "s59E13H2" 0.09206774 "Anaerobic ribonucleoside-triphosphate reductase"
"JS1_D03_01011" 12920 "s59E25H2" 0 "Anaerobic ribonucleoside-triphosphate reductase"
"JS1_D03_01011" 12920 "s59E5H2" 0.4157939 "Anaerobic ribonucleoside-triphosphate reductase"
"JS1_D03_01011" 12920 "s63E6H2" 0.6934949 "Anaerobic ribonucleoside-triphosphate reductase"
"JS1_D03_01012" 12921 "s59E13H2" 0.03445337 "Transcriptional repressor NrdR"
"JS1_D03_01012" 12921 "s59E25H2" 0 "Transcriptional repressor NrdR"
"JS1_D03_01012" 12921 "s59E5H2" 0.3435807 "Transcriptional repressor NrdR"
"JS1_D03_01012" 12921 "s63E6H2" 0.6331513 "Transcriptional repressor NrdR"
"JS1_D03_01013" 12922 "s59E13H2" 0.1622709 "Cell division protein FtsZ"
"JS1_D03_01013" 12922 "s59E25H2" 0 "Cell division protein FtsZ"
"JS1_D03_01013" 12922 "s59E5H2" 0.3420079 "Cell division protein FtsZ"
"JS1_D03_01013" 12922 "s63E6H2" 0.6842329 "Cell division protein FtsZ"
"JS1_D03_01014" 12923 "s59E13H2" 0.0158592 "Cell division protein FtsA"
"JS1_D03_01014" 12923 "s59E25H2" 0 "Cell division protein FtsA"
"JS1_D03_01014" 12923 "s59E5H2" 0.2012924 "Cell division protein FtsA"
"JS1_D03_01014" 12923 "s63E6H2" 0.1566043 "Cell division protein FtsA"
"JS1_D03_01015" 12924 "s59E13H2" 0.004113246 "Cell division protein DivIB"
"JS1_D03_01015" 12924 "s59E25H2" 0 "Cell division protein DivIB"
"JS1_D03_01015" 12924 "s59E5H2" 0.03158286 "Cell division protein DivIB"
"JS1_D03_01015" 12924 "s63E6H2" 0.04731338 "Cell division protein DivIB"
"JS1_D03_01016" 12925 "s59E13H2" 0 "UDP-N-acetylenolpyruvoylglucosamine reductase"
"JS1_D03_01016" 12925 "s59E25H2" 0 "UDP-N-acetylenolpyruvoylglucosamine reductase"
"JS1_D03_01016" 12925 "s59E5H2" 0.007253898 "UDP-N-acetylenolpyruvoylglucosamine reductase"
"JS1_D03_01016" 12925 "s63E6H2" 0.0302504 "UDP-N-acetylenolpyruvoylglucosamine reductase"
"JS1_D03_01017" 12926 "s59E13H2" 0 "YcfA-like protein"
"JS1_D03_01017" 12926 "s59E25H2" 0 "YcfA-like protein"
"JS1_D03_01017" 12926 "s59E5H2" 0.2154454 "YcfA-like protein"
"JS1_D03_01017" 12926 "s63E6H2" 0.1153199 "YcfA-like protein"
"JS1_D03_01018" 12927 "s59E13H2" 0 "hypothetical protein"
"JS1_D03_01018" 12927 "s59E25H2" 0 "hypothetical protein"
"JS1_D03_01018" 12927 "s59E5H2" 0.2015298 "hypothetical protein"
"JS1_D03_01018" 12927 "s63E6H2" 0.02507369 "hypothetical protein"
"JS1_D03_01023" 12932 "s59E13H2" 0 "HEPN domain protein"
"JS1_D03_01023" 12932 "s59E25H2" 0 "HEPN domain protein"
"JS1_D03_01023" 12932 "s59E5H2" 0 "HEPN domain protein"
"JS1_D03_01023" 12932 "s63E6H2" 0.01882825 "HEPN domain protein"
"JS1_D03_01025" 12934 "s59E13H2" 0 "hypothetical protein"
"JS1_D03_01025" 12934 "s59E25H2" 0 "hypothetical protein"
"JS1_D03_01025" 12934 "s59E5H2" 0.07163718 "hypothetical protein"
"JS1_D03_01025" 12934 "s63E6H2" 0.00428627 "hypothetical protein"
"JS1_D03_01026" 12935 "s59E13H2" 0 "Archaeal ATPase"
"JS1_D03_01026" 12935 "s59E25H2" 0 "Archaeal ATPase"
"JS1_D03_01026" 12935 "s59E5H2" 0.1437718 "Archaeal ATPase"
"JS1_D03_01026" 12935 "s63E6H2" 0.001164724 "Archaeal ATPase"
"JS1_D03_01027" 12936 "s59E13H2" 0 "Nucleotidyltransferase domain protein"
"JS1_D03_01027" 12936 "s59E25H2" 0 "Nucleotidyltransferase domain protein"
"JS1_D03_01027" 12936 "s59E5H2" 0.5632659 "Nucleotidyltransferase domain protein"

"JS1_D03_01027" 12936 "s63E6H2" 4.828254 "Nucleotidyltransferase domain protein"
"JS1_D03_01028" 12937 "s59E13H2" 0 "hypothetical protein"
"JS1_D03_01028" 12937 "s59E25H2" 0 "hypothetical protein"
"JS1_D03_01028" 12937 "s59E5H2" 0 "hypothetical protein"
"JS1_D03_01028" 12937 "s63E6H2" 0.03233773 "hypothetical protein"
"JS1_D03_01029" 12938 "s59E13H2" 0.004334645 "MarR family protein"
"JS1_D03_01029" 12938 "s59E25H2" 0 "MarR family protein"
"JS1_D03_01029" 12938 "s59E5H2" 0.07659456 "MarR family protein"
"JS1_D03_01029" 12938 "s63E6H2" 2.418226 "MarR family protein"
"JS1_D03_01030" 12939 "s59E13H2" 0 "dTDP-3-amino-3,6-dideoxy-alpha-D-galactopyranose 3-N-acetyltransferase"
"JS1_D03_01030" 12939 "s59E25H2" 0 "dTDP-3-amino-3,6-dideoxy-alpha-D-galactopyranose 3-N-acetyltransferase"
"JS1_D03_01030" 12939 "s59E5H2" 0 "dTDP-3-amino-3,6-dideoxy-alpha-D-galactopyranose 3-N-acetyltransferase"
"JS1_D03_01030" 12939 "s63E6H2" 0 "dTDP-3-amino-3,6-dideoxy-alpha-D-galactopyranose 3-N-acetyltransferase"
"JS1_D03_01031" 12940 "s59E13H2" 0 "hypothetical protein"
"JS1_D03_01031" 12940 "s59E25H2" 0 "hypothetical protein"
"JS1_D03_01031" 12940 "s59E5H2" 0 "hypothetical protein"
"JS1_D03_01031" 12940 "s63E6H2" 0 "hypothetical protein"
"JS1_D03_01032" 12941 "s59E13H2" 0 "dTDP-3-amino-3,6-dideoxy-alpha-D-galactopyranose transaminase"
"JS1_D03_01032" 12941 "s59E25H2" 0 "dTDP-3-amino-3,6-dideoxy-alpha-D-galactopyranose transaminase"
"JS1_D03_01032" 12941 "s59E5H2" 0.003428877 "dTDP-3-amino-3,6-dideoxy-alpha-D-galactopyranose transaminase"
"JS1_D03_01032" 12941 "s63E6H2" 0.004757481 "dTDP-3-amino-3,6-dideoxy-alpha-D-galactopyranose transaminase"
"JS1_D03_01033" 12942 "s59E13H2" 0 "Inositol 2-dehydrogenase"
"JS1_D03_01033" 12942 "s59E25H2" 0 "Inositol 2-dehydrogenase"
"JS1_D03_01033" 12942 "s59E5H2" 0 "Inositol 2-dehydrogenase"
"JS1_D03_01033" 12942 "s63E6H2" 0 "Inositol 2-dehydrogenase"
"JS1_D03_01034" 12943 "s59E13H2" 0.007827653 "UDP-N-acetyl-D-glucosamine 6-dehydrogenase"
"JS1_D03_01034" 12943 "s59E25H2" 0 "UDP-N-acetyl-D-glucosamine 6-dehydrogenase"
"JS1_D03_01034" 12943 "s59E5H2" 0 "UDP-N-acetyl-D-glucosamine 6-dehydrogenase"
"JS1_D03_01034" 12943 "s63E6H2" 0 "UDP-N-acetyl-D-glucosamine 6-dehydrogenase"
"JS1_D03_01035" 12944 "s59E13H2" 0 "dTDP-3-amino-3,6-dideoxy-alpha-D-galactopyranose 3-N-acetyltransferase"
"JS1_D03_01035" 12944 "s59E25H2" 0 "dTDP-3-amino-3,6-dideoxy-alpha-D-galactopyranose 3-N-acetyltransferase"
"JS1_D03_01035" 12944 "s59E5H2" 0 "dTDP-3-amino-3,6-dideoxy-alpha-D-galactopyranose 3-N-acetyltransferase"
"JS1_D03_01035" 12944 "s63E6H2" 0 "dTDP-3-amino-3,6-dideoxy-alpha-D-galactopyranose 3-N-acetyltransferase"
"JS1_D03_01036" 12945 "s59E13H2" 0 "UDP-2-acetamido-2-deoxy-3-oxo-D-glucuronate aminotransferase"
"JS1_D03_01036" 12945 "s59E25H2" 0 "UDP-2-acetamido-2-deoxy-3-oxo-D-glucuronate aminotransferase"
"JS1_D03_01036" 12945 "s59E5H2" 0 "UDP-2-acetamido-2-deoxy-3-oxo-D-glucuronate aminotransferase"
"JS1_D03_01036" 12945 "s63E6H2" 0 "UDP-2-acetamido-2-deoxy-3-oxo-D-glucuronate aminotransferase"
"JS1_D03_01037" 12946 "s59E13H2" 0 "UDP-4-amino-4-deoxy-L-arabinose--oxoglutarate aminotransferase"
"JS1_D03_01037" 12946 "s59E25H2" 0 "UDP-4-amino-4-deoxy-L-arabinose--oxoglutarate aminotransferase"
"JS1_D03_01037" 12946 "s59E5H2" 0 "UDP-4-amino-4-deoxy-L-arabinose--oxoglutarate aminotransferase"
"JS1_D03_01037" 12946 "s63E6H2" 0 "UDP-4-amino-4-deoxy-L-arabinose--oxoglutarate aminotransferase"
"JS1_D03_01038" 12947 "s59E13H2" 0 "ADP-L-glycero-D-manno-heptose-6-epimerase"
"JS1_D03_01038" 12947 "s59E25H2" 0 "ADP-L-glycero-D-manno-heptose-6-epimerase"
"JS1_D03_01038" 12947 "s59E5H2" 0 "ADP-L-glycero-D-manno-heptose-6-epimerase"
"JS1_D03_01038" 12947 "s63E6H2" 0 "ADP-L-glycero-D-manno-heptose-6-epimerase"
"JS1_D03_01039" 12948 "s59E13H2" 0 "dTDP-glucose 4,6-dehydratase"
"JS1_D03_01039" 12948 "s59E25H2" 0 "dTDP-glucose 4,6-dehydratase"

"JS1_D03_01039" 12948 "s59E5H2" 0 "dTDP-glucose 4,6-dehydratase"
"JS1_D03_01039" 12948 "s63E6H2" 0 "dTDP-glucose 4,6-dehydratase"
"JS1_D03_01040" 12949 "s59E13H2" 0 "4-carboxy-2-hydroxymuconate-6-semialdehyde dehydrogenase"
"JS1_D03_01040" 12949 "s59E25H2" 0 "4-carboxy-2-hydroxymuconate-6-semialdehyde dehydrogenase"
"JS1_D03_01040" 12949 "s59E5H2" 0 "4-carboxy-2-hydroxymuconate-6-semialdehyde dehydrogenase"
"JS1_D03_01040" 12949 "s63E6H2" 0.006098287 "4-carboxy-2-hydroxymuconate-6-semialdehyde dehydrogenase"
"JS1_D03_01041" 12950 "s59E13H2" 0 "dTDP-glucose 4,6-dehydratase"
"JS1_D03_01041" 12950 "s59E25H2" 0 "dTDP-glucose 4,6-dehydratase"
"JS1_D03_01041" 12950 "s59E5H2" 0 "dTDP-glucose 4,6-dehydratase"
"JS1_D03_01041" 12950 "s63E6H2" 0 "dTDP-glucose 4,6-dehydratase"
"JS1_D03_01042" 12951 "s59E13H2" 0 "phosphodiesterase"
"JS1_D03_01042" 12951 "s59E25H2" 0 "phosphodiesterase"
"JS1_D03_01042" 12951 "s59E5H2" 0 "phosphodiesterase"
"JS1_D03_01042" 12951 "s63E6H2" 0 "phosphodiesterase"
"JS1_D03_01043" 12952 "s59E13H2" 0 "Coenzyme gamma-F420-2:alpha-L-glutamate ligase"
"JS1_D03_01043" 12952 "s59E25H2" 0 "Coenzyme gamma-F420-2:alpha-L-glutamate ligase"
"JS1_D03_01043" 12952 "s59E5H2" 0 "Coenzyme gamma-F420-2:alpha-L-glutamate ligase"
"JS1_D03_01043" 12952 "s63E6H2" 0 "Coenzyme gamma-F420-2:alpha-L-glutamate ligase"
"JS1_D03_01044" 12953 "s59E13H2" 0 "bifunctional 3-demethylubiquinone-9 3-methyltransferase/ 2-octaprenyl-6-hydroxy phenol methylase"
"JS1_D03_01044" 12953 "s59E25H2" 0 "bifunctional 3-demethylubiquinone-9 3-methyltransferase/ 2-octaprenyl-6-hydroxy phenol methylase"
"JS1_D03_01044" 12953 "s59E5H2" 0 "bifunctional 3-demethylubiquinone-9 3-methyltransferase/ 2-octaprenyl-6-hydroxy phenol methylase"
"JS1_D03_01044" 12953 "s63E6H2" 0 "bifunctional 3-demethylubiquinone-9 3-methyltransferase/ 2-octaprenyl-6-hydroxy phenol methylase"
"JS1_D03_01045" 12954 "s59E13H2" 0 "Imidazole glycerol phosphate synthase subunit HisH 1"
"JS1_D03_01045" 12954 "s59E25H2" 0 "Imidazole glycerol phosphate synthase subunit HisH 1"
"JS1_D03_01045" 12954 "s59E5H2" 0 "Imidazole glycerol phosphate synthase subunit HisH 1"
"JS1_D03_01045" 12954 "s63E6H2" 0 "Imidazole glycerol phosphate synthase subunit HisH 1"
"JS1_D03_01047" 12956 "s59E13H2" 0 "tRNA(Ile)-lysidine synthase"
"JS1_D03_01047" 12956 "s59E25H2" 0 "tRNA(Ile)-lysidine synthase"
"JS1_D03_01047" 12956 "s59E5H2" 0 "tRNA(Ile)-lysidine synthase"
"JS1_D03_01047" 12956 "s63E6H2" 0 "tRNA(Ile)-lysidine synthase"
"JS1_D03_01048" 12957 "s59E13H2" 0 "Glycosyl transferases group 1"
"JS1_D03_01048" 12957 "s59E25H2" 0 "Glycosyl transferases group 1"
"JS1_D03_01048" 12957 "s59E5H2" 0 "Glycosyl transferases group 1"
"JS1_D03_01048" 12957 "s63E6H2" 0.00477403 "Glycosyl transferases group 1"
"JS1_D03_01049" 12958 "s59E13H2" 0.04244852 "Carbon monoxide dehydrogenase medium chain"
"JS1_D03_01049" 12958 "s59E25H2" 0 "Carbon monoxide dehydrogenase medium chain"
"JS1_D03_01049" 12958 "s59E5H2" 0.08812898 "Carbon monoxide dehydrogenase medium chain"
"JS1_D03_01049" 12958 "s63E6H2" 5.915533 "Carbon monoxide dehydrogenase medium chain"
"JS1_D03_01050" 12959 "s59E13H2" 0.0637458 "Nicotinate dehydrogenase small FeS subunit"
"JS1_D03_01050" 12959 "s59E25H2" 0 "Nicotinate dehydrogenase small FeS subunit"
"JS1_D03_01050" 12959 "s59E5H2" 0.3042916 "Nicotinate dehydrogenase small FeS subunit"
"JS1_D03_01050" 12959 "s63E6H2" 5.515098 "Nicotinate dehydrogenase small FeS subunit"
"JS1_D03_01051" 12960 "s59E13H2" 0.00527235 "Nicotinate dehydrogenase large molybdopterin subunit"
"JS1_D03_01051" 12960 "s59E25H2" 0.04996097 "Nicotinate dehydrogenase large molybdopterin subunit"
"JS1_D03_01051" 12960 "s59E5H2" 0.9177663 "Nicotinate dehydrogenase large molybdopterin subunit"
"JS1_D03_01051" 12960 "s63E6H2" 12.07026 "Nicotinate dehydrogenase large molybdopterin subunit"
"JS1_D03_01052" 12961 "s59E13H2" 0.02430605 "Nicotinate dehydrogenase medium molybdopterin subunit"

"JS1_D03_01052" 12961 "s59E25H2" 0.5055388 "Nicotinate dehydrogenase medium molybdopterin subunit"
"JS1_D03_01052" 12961 "s59E5H2" 0.9687857 "Nicotinate dehydrogenase medium molybdopterin subunit"
"JS1_D03_01052" 12961 "s63E6H2" 14.8994 "Nicotinate dehydrogenase medium molybdopterin subunit"
"JS1_D03_01053" 12962 "s59E13H2" 2.498192 "DNA polymerase IV"
"JS1_D03_01053" 12962 "s59E25H2" 0.203749 "DNA polymerase IV"
"JS1_D03_01053" 12962 "s59E5H2" 0.523849 "DNA polymerase IV"
"JS1_D03_01053" 12962 "s63E6H2" 10.79815 "DNA polymerase IV"
"JS1_D03_01054" 12963 "s59E13H2" 6.756939 "DNA polymerase III subunit alpha"
"JS1_D03_01054" 12963 "s59E25H2" 0.2844444 "DNA polymerase III subunit alpha"
"JS1_D03_01054" 12963 "s59E5H2" 3.860492 "DNA polymerase III subunit alpha"
"JS1_D03_01054" 12963 "s63E6H2" 10.54497 "DNA polymerase III subunit alpha"
"JS1_D03_01055" 12964 "s59E13H2" 0.127808 "hypothetical protein"
"JS1_D03_01055" 12964 "s59E25H2" 0.5982906 "hypothetical protein"
"JS1_D03_01055" 12964 "s59E5H2" 5.311312 "hypothetical protein"
"JS1_D03_01055" 12964 "s63E6H2" 3.35726 "hypothetical protein"
"JS1_D03_01056" 12965 "s59E13H2" 0.06337318 "LexA repressor"
"JS1_D03_01056" 12965 "s59E25H2" 0.4084967 "LexA repressor"
"JS1_D03_01056" 12965 "s59E5H2" 1.654391 "LexA repressor"
"JS1_D03_01056" 12965 "s63E6H2" 2.004695 "LexA repressor"
"JS1_D03_01057" 12966 "s59E13H2" 0.07417085 "hypothetical protein"
"JS1_D03_01057" 12966 "s59E25H2" 0 "hypothetical protein"
"JS1_D03_01057" 12966 "s59E5H2" 2.143611 "hypothetical protein"
"JS1_D03_01057" 12966 "s63E6H2" 0.2331217 "hypothetical protein"
"JS1_D03_01058" 12967 "s59E13H2" 0.04774415 "Sporulation thiol-disulfide oxidoreductase A precursor"
"JS1_D03_01058" 12967 "s59E25H2" 0 "Sporulation thiol-disulfide oxidoreductase A precursor"
"JS1_D03_01058" 12967 "s59E5H2" 1.104677 "Sporulation thiol-disulfide oxidoreductase A precursor"
"JS1_D03_01058" 12967 "s63E6H2" 0.09045944 "Sporulation thiol-disulfide oxidoreductase A precursor"
"JS1_D03_01059" 12968 "s59E13H2" 0 "hypothetical protein"
"JS1_D03_01059" 12968 "s59E25H2" 0 "hypothetical protein"
"JS1_D03_01059" 12968 "s59E5H2" 0 "hypothetical protein"
"JS1_D03_01059" 12968 "s63E6H2" 0.003916696 "hypothetical protein"
"JS1_D03_01060" 12969 "s59E13H2" 0.06855368 "hypothetical protein"
"JS1_D03_01060" 12969 "s59E25H2" 0 "hypothetical protein"
"JS1_D03_01060" 12969 "s59E5H2" 1.494828 "hypothetical protein"
"JS1_D03_01060" 12969 "s63E6H2" 0.3746734 "hypothetical protein"
"JS1_D03_01061" 12970 "s59E13H2" 0.03711701 "HTH-type transcriptional repressor AseR"
"JS1_D03_01061" 12970 "s59E25H2" 0 "HTH-type transcriptional repressor AseR"
"JS1_D03_01061" 12970 "s59E5H2" 1.359681 "HTH-type transcriptional repressor AseR"
"JS1_D03_01061" 12970 "s63E6H2" 0.2882372 "HTH-type transcriptional repressor AseR"
"JS1_D03_01062" 12971 "s59E13H2" 0.1582557 "Peptidase family C69"
"JS1_D03_01062" 12971 "s59E25H2" 0 "Peptidase family C69"
"JS1_D03_01062" 12971 "s59E5H2" 2.078089 "Peptidase family C69"
"JS1_D03_01062" 12971 "s63E6H2" 0.6302243 "Peptidase family C69"
"JS1_D03_01063" 12972 "s59E13H2" 0.03811264 "flavodoxin"
"JS1_D03_01063" 12972 "s59E25H2" 0 "flavodoxin"
"JS1_D03_01063" 12972 "s59E5H2" 0.349086 "flavodoxin"
"JS1_D03_01063" 12972 "s63E6H2" 0.08593564 "flavodoxin"

"JS1_D03_01064" 12973 "s59E13H2" 0.00329196 "Possibl zinc metallo-peptidase"
"JS1_D03_01064" 12973 "s59E25H2" 0 "Possibl zinc metallo-peptidase"
"JS1_D03_01064" 12973 "s59E5H2" 0.07091086 "Possibl zinc metallo-peptidase"
"JS1_D03_01064" 12973 "s63E6H2" 0.1490741 "Possibl zinc metallo-peptidase"
"JS1_D03_01065" 12974 "s59E13H2" 0 "3'-5' exoribonuclease YhaM"
"JS1_D03_01065" 12974 "s59E25H2" 0 "3'-5' exoribonuclease YhaM"
"JS1_D03_01065" 12974 "s59E5H2" 0 "3'-5' exoribonuclease YhaM"
"JS1_D03_01065" 12974 "s63E6H2" 0.04600835 "3'-5' exoribonuclease YhaM"
"JS1_D03_01066" 12975 "s59E13H2" 0 "L-asparaginase II"
"JS1_D03_01066" 12975 "s59E25H2" 0 "L-asparaginase II"
"JS1_D03_01066" 12975 "s59E5H2" 0 "L-asparaginase II"
"JS1_D03_01066" 12975 "s63E6H2" 0.02358303 "L-asparaginase II"
"JS1_D03_01067" 12976 "s59E13H2" 0.5928489 "protease TldD"
"JS1_D03_01067" 12976 "s59E25H2" 0 "protease TldD"
"JS1_D03_01067" 12976 "s59E5H2" 1.035316 "protease TldD"
"JS1_D03_01067" 12976 "s63E6H2" 0.7799937 "protease TldD"
"JS1_D03_01068" 12977 "s59E13H2" 0.2435675 "protease TldD"
"JS1_D03_01068" 12977 "s59E25H2" 0 "protease TldD"
"JS1_D03_01068" 12977 "s59E5H2" 0.06274952 "protease TldD"
"JS1_D03_01068" 12977 "s63E6H2" 0.8675433 "protease TldD"
"JS1_D03_01069" 12978 "s59E13H2" 0.09901057 "hypothetical protein"
"JS1_D03_01069" 12978 "s59E25H2" 0 "hypothetical protein"
"JS1_D03_01069" 12978 "s59E5H2" 0.4654864 "hypothetical protein"
"JS1_D03_01069" 12978 "s63E6H2" 1.286115 "hypothetical protein"
"JS1_D03_01070" 12979 "s59E13H2" 0.03004259 "Formamidase"
"JS1_D03_01070" 12979 "s59E25H2" 0 "Formamidase"
"JS1_D03_01070" 12979 "s59E5H2" 0.1197071 "Formamidase"
"JS1_D03_01070" 12979 "s63E6H2" 0.2129255 "Formamidase"
"JS1_D03_01071" 12980 "s59E13H2" 0.06136378 "Carbon-nitrogen hydrolase"
"JS1_D03_01071" 12980 "s59E25H2" 0 "Carbon-nitrogen hydrolase"
"JS1_D03_01071" 12980 "s59E5H2" 0.5767924 "Carbon-nitrogen hydrolase"
"JS1_D03_01071" 12980 "s63E6H2" 0.7658441 "Carbon-nitrogen hydrolase"
"JS1_D03_01072" 12981 "s59E13H2" 0.2527147 "Inner membrane symporter YicJ"
"JS1_D03_01072" 12981 "s59E25H2" 0 "Inner membrane symporter YicJ"
"JS1_D03_01072" 12981 "s59E5H2" 2.616388 "Inner membrane symporter YicJ"
"JS1_D03_01072" 12981 "s63E6H2" 2.567813 "Inner membrane symporter YicJ"
"JS1_D03_01073" 12982 "s59E13H2" 0 "Alpha-monoglucosyldiacylglycerol synthase"
"JS1_D03_01073" 12982 "s59E25H2" 0 "Alpha-monoglucosyldiacylglycerol synthase"
"JS1_D03_01073" 12982 "s59E5H2" 0 "Alpha-monoglucosyldiacylglycerol synthase"
"JS1_D03_01073" 12982 "s63E6H2" 0.04997519 "Alpha-monoglucosyldiacylglycerol synthase"
"JS1_D03_01074" 12983 "s59E13H2" 0 "Diacetylchitobiose deacetylase"
"JS1_D03_01074" 12983 "s59E25H2" 0 "Diacetylchitobiose deacetylase"
"JS1_D03_01074" 12983 "s59E5H2" 0.004889253 "Diacetylchitobiose deacetylase"
"JS1_D03_01074" 12983 "s63E6H2" 0.05613879 "Diacetylchitobiose deacetylase"
"JS1_D03_01075" 12984 "s59E13H2" 0 "hypothetical protein"
"JS1_D03_01075" 12984 "s59E25H2" 0 "hypothetical protein"
"JS1_D03_01075" 12984 "s59E5H2" 0.01154367 "hypothetical protein"

"JS1_D03_01075" 12984 "s63E6H2" 0.0129519 "hypothetical protein"
"JS1_D03_01076" 12985 "s59E13H2" 2.59532 "hypothetical protein"
"JS1_D03_01076" 12985 "s59E25H2" 0 "hypothetical protein"
"JS1_D03_01076" 12985 "s59E5H2" 0.2034667 "hypothetical protein"
"JS1_D03_01076" 12985 "s63E6H2" 2.059974 "hypothetical protein"
"JS1_D03_01077" 12986 "s59E13H2" 0 "hypothetical protein"
"JS1_D03_01077" 12986 "s59E25H2" 0 "hypothetical protein"
"JS1_D03_01077" 12986 "s59E5H2" 0.6807361 "hypothetical protein"
"JS1_D03_01077" 12986 "s63E6H2" 1.042701 "hypothetical protein"
"JS1_D03_01078" 12987 "s59E13H2" 0 "Magnesium transport protein CorA"
"JS1_D03_01078" 12987 "s59E25H2" 0 "Magnesium transport protein CorA"
"JS1_D03_01078" 12987 "s59E5H2" 1.842384 "Magnesium transport protein CorA"
"JS1_D03_01078" 12987 "s63E6H2" 0.2399782 "Magnesium transport protein CorA"
"JS1_D03_01079" 12988 "s59E13H2" 0 "tRNA-specific 2-thiouridylase MnmA"
"JS1_D03_01079" 12988 "s59E25H2" 0 "tRNA-specific 2-thiouridylase MnmA"
"JS1_D03_01079" 12988 "s59E5H2" 0 "tRNA-specific 2-thiouridylase MnmA"
"JS1_D03_01079" 12988 "s63E6H2" 0.1620583 "tRNA-specific 2-thiouridylase MnmA"
"JS1_D03_01080" 12989 "s59E13H2" 0 "GMP synthase [glutamine-hydrolyzing] subunit B"
"JS1_D03_01080" 12989 "s59E25H2" 0 "GMP synthase [glutamine-hydrolyzing] subunit B"
"JS1_D03_01080" 12989 "s59E5H2" 0 "GMP synthase [glutamine-hydrolyzing] subunit B"
"JS1_D03_01080" 12989 "s63E6H2" 0.1277572 "GMP synthase [glutamine-hydrolyzing] subunit B"
"JS1_D03_01081" 12990 "s59E13H2" 0 "thiamine biosynthesis protein ThiI"
"JS1_D03_01081" 12990 "s59E25H2" 0 "thiamine biosynthesis protein ThiI"
"JS1_D03_01081" 12990 "s59E5H2" 0 "thiamine biosynthesis protein ThiI"
"JS1_D03_01081" 12990 "s63E6H2" 0.03497874 "thiamine biosynthesis protein ThiI"
"JS1_D03_01082" 12991 "s59E13H2" 0.005365743 "30S ribosomal protein S3"
"JS1_D03_01082" 12991 "s59E25H2" 0 "30S ribosomal protein S3"
"JS1_D03_01082" 12991 "s59E5H2" 0.01508684 "30S ribosomal protein S3"
"JS1_D03_01082" 12991 "s63E6H2" 0.177507 "30S ribosomal protein S3"
"JS1_D03_01083" 12992 "s59E13H2" 0.01090203 "50S ribosomal protein L16"
"JS1_D03_01083" 12992 "s59E25H2" 0 "50S ribosomal protein L16"
"JS1_D03_01083" 12992 "s59E5H2" 0.06660991 "50S ribosomal protein L16"
"JS1_D03_01083" 12992 "s63E6H2" 0.263364 "50S ribosomal protein L16"
"JS1_D03_01084" 12993 "s59E13H2" 0.001545687 "Adenylate kinase"
"JS1_D03_01084" 12993 "s59E25H2" 0 "Adenylate kinase"
"JS1_D03_01084" 12993 "s59E5H2" 0.02540099 "Adenylate kinase"
"JS1_D03_01084" 12993 "s63E6H2" 0.06635334 "Adenylate kinase"
"JS1_D03_01087" 12996 "s59E13H2" 0 "hypothetical protein"
"JS1_D03_01087" 12996 "s59E25H2" 0 "hypothetical protein"
"JS1_D03_01087" 12996 "s59E5H2" 0.02710814 "hypothetical protein"
"JS1_D03_01087" 12996 "s63E6H2" 0.1424148 "hypothetical protein"
"JS1_D03_01090" 12999 "s59E13H2" 0 "Mannosylfructose-phosphate phosphatase"
"JS1_D03_01090" 12999 "s59E25H2" 0 "Mannosylfructose-phosphate phosphatase"
"JS1_D03_01090" 12999 "s59E5H2" 0.0307543 "Mannosylfructose-phosphate phosphatase"
"JS1_D03_01090" 12999 "s63E6H2" 0.08867782 "Mannosylfructose-phosphate phosphatase"
"JS1_D03_01091" 13000 "s59E13H2" 0.004942979 "D-inositol 3-phosphate glycosyltransferase"
"JS1_D03_01091" 13000 "s59E25H2" 0 "D-inositol 3-phosphate glycosyltransferase"

"JS1_D03_01091" 13000 "s59E5H2" 0.007030088 "D-inositol 3-phosphate glycosyltransferase"
"JS1_D03_01091" 13000 "s63E6H2" 0.05145349 "D-inositol 3-phosphate glycosyltransferase"
"JS1_D03_01094" 13003 "s59E13H2" 0 "Acyl carrier protein"
"JS1_D03_01094" 13003 "s59E25H2" 0 "Acyl carrier protein"
"JS1_D03_01094" 13003 "s59E5H2" 0.01487743 "Acyl carrier protein"
"JS1_D03_01094" 13003 "s63E6H2" 0.03697609 "Acyl carrier protein"
"JS1_D03_01095" 13004 "s59E13H2" 0 "hypothetical protein"
"JS1_D03_01095" 13004 "s59E25H2" 0 "hypothetical protein"
"JS1_D03_01095" 13004 "s59E5H2" 0.004238361 "hypothetical protein"
"JS1_D03_01095" 13004 "s63E6H2" 0.02789321 "hypothetical protein"
"JS1_D03_01097" 13006 "s59E13H2" 6.302462 "hypothetical protein"
"JS1_D03_01097" 13006 "s59E25H2" 0 "hypothetical protein"
"JS1_D03_01097" 13006 "s59E5H2" 0.1528856 "hypothetical protein"
"JS1_D03_01097" 13006 "s63E6H2" 0.07637107 "hypothetical protein"
"JS1_D03_01099" 13008 "s59E13H2" 0.111083 "Glycine betaine transport ATP-binding protein OpuAA"
"JS1_D03_01099" 13008 "s59E25H2" 0 "Glycine betaine transport ATP-binding protein OpuAA"
"JS1_D03_01099" 13008 "s59E5H2" 1.304914 "Glycine betaine transport ATP-binding protein OpuAA"
"JS1_D03_01099" 13008 "s63E6H2" 0.02447185 "Glycine betaine transport ATP-binding protein OpuAA"
"JS1_D03_01100" 13009 "s59E13H2" 0.461525 "Glycine betaine transport system permease protein OpuAB"
"JS1_D03_01100" 13009 "s59E25H2" 0 "Glycine betaine transport system permease protein OpuAB"
"JS1_D03_01100" 13009 "s59E5H2" 1.282139 "Glycine betaine transport system permease protein OpuAB"
"JS1_D03_01100" 13009 "s63E6H2" 0.891029 "Glycine betaine transport system permease protein OpuAB"
"JS1_D03_01101" 13010 "s59E13H2" 0.2287918 "glycine betaine transporter periplasmic subunit"
"JS1_D03_01101" 13010 "s59E25H2" 0 "glycine betaine transporter periplasmic subunit"
"JS1_D03_01101" 13010 "s59E5H2" 0.968918 "glycine betaine transporter periplasmic subunit"
"JS1_D03_01101" 13010 "s63E6H2" 0.3199757 "glycine betaine transporter periplasmic subunit"
"JS1_D03_01102" 13011 "s59E13H2" 2.390274 "hypothetical protein"
"JS1_D03_01102" 13011 "s59E25H2" 0 "hypothetical protein"
"JS1_D03_01102" 13011 "s59E5H2" 0.4724789 "hypothetical protein"
"JS1_D03_01102" 13011 "s63E6H2" 0.5485793 "hypothetical protein"
"JS1_D03_01103" 13012 "s59E13H2" 0 "hypothetical protein"
"JS1_D03_01103" 13012 "s59E25H2" 0 "hypothetical protein"
"JS1_D03_01103" 13012 "s59E5H2" 0 "hypothetical protein"
"JS1_D03_01103" 13012 "s63E6H2" 0.9058704 "hypothetical protein"
"JS1_D03_01104" 13013 "s59E13H2" 0.04498319 "Bacterial Ig-like domain (group 2)"
"JS1_D03_01104" 13013 "s59E25H2" 0 "Bacterial Ig-like domain (group 2)"
"JS1_D03_01104" 13013 "s59E5H2" 0.09498206 "Bacterial Ig-like domain (group 2)"
"JS1_D03_01104" 13013 "s63E6H2" 0.05765052 "Bacterial Ig-like domain (group 2)"
"JS1_D03_01105" 13014 "s59E13H2" 0.003966104 "hypothetical protein"
"JS1_D03_01105" 13014 "s59E25H2" 0 "hypothetical protein"
"JS1_D03_01105" 13014 "s59E5H2" 0.05624585 "hypothetical protein"
"JS1_D03_01105" 13014 "s63E6H2" 0.07000631 "hypothetical protein"
"JS1_D03_01106" 13015 "s59E13H2" 0.05035866 "Outer membrane protein alpha precursor"
"JS1_D03_01106" 13015 "s59E25H2" 0 "Outer membrane protein alpha precursor"
"JS1_D03_01106" 13015 "s59E5H2" 0.1914059 "Outer membrane protein alpha precursor"
"JS1_D03_01106" 13015 "s63E6H2" 0.3130592 "Outer membrane protein alpha precursor"
"JS1_D03_01107" 13016 "s59E13H2" 0.0009358653 "hypothetical protein"

"JS1_D03_01107" 13016 "s59E25H2" 0 "hypothetical protein"
"JS1_D03_01107" 13016 "s59E5H2" 0.001899812 "hypothetical protein"
"JS1_D03_01107" 13016 "s63E6H2" 0.04281361 "hypothetical protein"
"JS1_D03_01108" 13017 "s59E13H2" 0.4096777 "Lipopolysaccharide export system ATP-binding protein LptB"
"JS1_D03_01108" 13017 "s59E25H2" 0 "Lipopolysaccharide export system ATP-binding protein LptB"
"JS1_D03_01108" 13017 "s59E5H2" 0.645644 "Lipopolysaccharide export system ATP-binding protein LptB"
"JS1_D03_01108" 13017 "s63E6H2" 1.137155 "Lipopolysaccharide export system ATP-binding protein LptB"
"JS1_D03_01109" 13018 "s59E13H2" 0.2071599 "3-hydroxyacyl-[acyl-carrier-protein] dehydratase FabZ"
"JS1_D03_01109" 13018 "s59E25H2" 0 "3-hydroxyacyl-[acyl-carrier-protein] dehydratase FabZ"
"JS1_D03_01109" 13018 "s59E5H2" 0.4075607 "3-hydroxyacyl-[acyl-carrier-protein] dehydratase FabZ"
"JS1_D03_01109" 13018 "s63E6H2" 0.52357 "3-hydroxyacyl-[acyl-carrier-protein] dehydratase FabZ"
"JS1_D03_01110" 13019 "s59E13H2" 0.0591664 "Outer membrane protein (OmpH-like)"
"JS1_D03_01110" 13019 "s59E25H2" 0 "Outer membrane protein (OmpH-like)"
"JS1_D03_01110" 13019 "s59E5H2" 0.1492356 "Outer membrane protein (OmpH-like)"
"JS1_D03_01110" 13019 "s63E6H2" 0.2509706 "Outer membrane protein (OmpH-like)"
"JS1_D03_01111" 13020 "s59E13H2" 0.1366702 "Outer membrane protein assembly factor BamA precursor"
"JS1_D03_01111" 13020 "s59E25H2" 0 "Outer membrane protein assembly factor BamA precursor"
"JS1_D03_01111" 13020 "s59E5H2" 0.505571 "Outer membrane protein assembly factor BamA precursor"
"JS1_D03_01111" 13020 "s63E6H2" 0.3091693 "Outer membrane protein assembly factor BamA precursor"
"JS1_D03_01112" 13021 "s59E13H2" 0.04037239 "putative assembly protein"
"JS1_D03_01112" 13021 "s59E25H2" 0 "putative assembly protein"
"JS1_D03_01112" 13021 "s59E5H2" 0.1647958 "putative assembly protein"
"JS1_D03_01112" 13021 "s63E6H2" 0.222422 "putative assembly protein"
"JS1_D03_01113" 13022 "s59E13H2" 0.01439491 "Nucleoside triphosphate pyrophosphohydrolase/pyrophosphatase MazG"
"JS1_D03_01113" 13022 "s59E25H2" 0 "Nucleoside triphosphate pyrophosphohydrolase/pyrophosphatase MazG"
"JS1_D03_01113" 13022 "s59E5H2" 0.1523981 "Nucleoside triphosphate pyrophosphohydrolase/pyrophosphatase MazG"
"JS1_D03_01113" 13022 "s63E6H2" 0.2680573 "Nucleoside triphosphate pyrophosphohydrolase/pyrophosphatase MazG"
"JS1_D03_01114" 13023 "s59E13H2" 0.03138691 "preprotein translocase subunit SecY"
"JS1_D03_01114" 13023 "s59E25H2" 0 "preprotein translocase subunit SecY"
"JS1_D03_01114" 13023 "s59E5H2" 0.01753514 "preprotein translocase subunit SecY"
"JS1_D03_01114" 13023 "s63E6H2" 0.04658032 "preprotein translocase subunit SecY"
"JS1_D03_01119" 13028 "s59E13H2" 0.01111648 "Serine hydroxymethyltransferase"
"JS1_D03_01119" 13028 "s59E25H2" 0 "Serine hydroxymethyltransferase"
"JS1_D03_01119" 13028 "s59E5H2" 0.122863 "Serine hydroxymethyltransferase"
"JS1_D03_01119" 13028 "s63E6H2" 0.1081787 "Serine hydroxymethyltransferase"
"JS1_D03_01120" 13029 "s59E13H2" 0.004630639 "Xaa-Pro dipeptidase"
"JS1_D03_01120" 13029 "s59E25H2" 0 "Xaa-Pro dipeptidase"
"JS1_D03_01120" 13029 "s59E5H2" 0.02094203 "Xaa-Pro dipeptidase"
"JS1_D03_01120" 13029 "s63E6H2" 0.01690188 "Xaa-Pro dipeptidase"
"JS1_D03_01121" 13030 "s59E13H2" 0 "Shikimate kinase 1"
"JS1_D03_01121" 13030 "s59E25H2" 0 "Shikimate kinase 1"
"JS1_D03_01121" 13030 "s59E5H2" 0 "Shikimate kinase 1"
"JS1_D03_01121" 13030 "s63E6H2" 0.0179368 "Shikimate kinase 1"
"JS1_D03_01122" 13031 "s59E13H2" 0.01110509 "Ribosomal protein S12 methylthiotransferase RimO"
"JS1_D03_01122" 13031 "s59E25H2" 0 "Ribosomal protein S12 methylthiotransferase RimO"
"JS1_D03_01122" 13031 "s59E5H2" 0.03481714 "Ribosomal protein S12 methylthiotransferase RimO"
"JS1_D03_01122" 13031 "s63E6H2" 0.04829884 "Ribosomal protein S12 methylthiotransferase RimO"

"JS1_D03_01123" 13032 "s59E13H2" 0.004919318 "hypothetical protein"
"JS1_D03_01123" 13032 "s59E25H2" 0 "hypothetical protein"
"JS1_D03_01123" 13032 "s59E5H2" 0 "hypothetical protein"
"JS1_D03_01123" 13032 "s63E6H2" 0.02833478 "hypothetical protein"
"JS1_D03_01124" 13033 "s59E13H2" 0.2959464 "hypothetical protein"
"JS1_D03_01124" 13033 "s59E25H2" 0 "hypothetical protein"
"JS1_D03_01124" 13033 "s59E5H2" 0.02386727 "hypothetical protein"
"JS1_D03_01124" 13033 "s63E6H2" 0.03856357 "hypothetical protein"
"JS1_D03_01126" 13035 "s59E13H2" 0 "Adenylate cyclase 2"
"JS1_D03_01126" 13035 "s59E25H2" 0 "Adenylate cyclase 2"
"JS1_D03_01126" 13035 "s59E5H2" 0.01359767 "Adenylate cyclase 2"
"JS1_D03_01126" 13035 "s63E6H2" 0.04804956 "Adenylate cyclase 2"
"JS1_D03_01127" 13036 "s59E13H2" 1.628733 "hypothetical protein"
"JS1_D03_01127" 13036 "s59E25H2" 0 "hypothetical protein"
"JS1_D03_01127" 13036 "s59E5H2" 0.008406084 "hypothetical protein"
"JS1_D03_01127" 13036 "s63E6H2" 0.05536861 "hypothetical protein"
"JS1_D03_01128" 13037 "s59E13H2" 2.408869 "hypothetical protein"
"JS1_D03_01128" 13037 "s59E25H2" 0 "hypothetical protein"
"JS1_D03_01128" 13037 "s59E5H2" 0.00471614 "hypothetical protein"
"JS1_D03_01128" 13037 "s63E6H2" 0.06047972 "hypothetical protein"
"JS1_D03_01129" 13038 "s59E13H2" 0 "HEPN domain protein"
"JS1_D03_01129" 13038 "s59E25H2" 0 "HEPN domain protein"
"JS1_D03_01129" 13038 "s59E5H2" 0.006847704 "HEPN domain protein"
"JS1_D03_01129" 13038 "s63E6H2" 0.02597233 "HEPN domain protein"
"JS1_D03_01130" 13039 "s59E13H2" 0 "Nucleotidyltransferase domain protein"
"JS1_D03_01130" 13039 "s59E25H2" 0 "Nucleotidyltransferase domain protein"
"JS1_D03_01130" 13039 "s59E5H2" 0.02156214 "Nucleotidyltransferase domain protein"
"JS1_D03_01130" 13039 "s63E6H2" 0.03852223 "Nucleotidyltransferase domain protein"
"JS1_D03_01133" 13042 "s59E13H2" 0.0974203 "hypothetical protein"
"JS1_D03_01133" 13042 "s59E25H2" 0 "hypothetical protein"
"JS1_D03_01133" 13042 "s59E5H2" 0.0969767 "hypothetical protein"
"JS1_D03_01133" 13042 "s63E6H2" 0.04303961 "hypothetical protein"
"JS1_D03_01134" 13043 "s59E13H2" 0.02944305 "Type IV pilus biogenesis and competence protein PilQ precursor"
"JS1_D03_01134" 13043 "s59E25H2" 0 "Type IV pilus biogenesis and competence protein PilQ precursor"
"JS1_D03_01134" 13043 "s59E5H2" 0.02799724 "Type IV pilus biogenesis and competence protein PilQ precursor"
"JS1_D03_01134" 13043 "s63E6H2" 0.02913993 "Type IV pilus biogenesis and competence protein PilQ precursor"
"JS1_D03_01135" 13044 "s59E13H2" 0.001950166 "hypothetical protein"
"JS1_D03_01135" 13044 "s59E25H2" 0 "hypothetical protein"
"JS1_D03_01135" 13044 "s59E5H2" 0.01805512 "hypothetical protein"
"JS1_D03_01135" 13044 "s63E6H2" 0.0166849 "hypothetical protein"
"JS1_D03_01136" 13045 "s59E13H2" 0.01096602 "Pilus assembly protein, PilO"
"JS1_D03_01136" 13045 "s59E25H2" 0 "Pilus assembly protein, PilO"
"JS1_D03_01136" 13045 "s59E5H2" 0.02237511 "Pilus assembly protein, PilO"
"JS1_D03_01136" 13045 "s63E6H2" 0.01092081 "Pilus assembly protein, PilO"
"JS1_D03_01137" 13046 "s59E13H2" 0.04216291 "hypothetical protein"
"JS1_D03_01137" 13046 "s59E25H2" 0 "hypothetical protein"
"JS1_D03_01137" 13046 "s59E5H2" 0.03885669 "hypothetical protein"

"JS1_D03_01137" 13046 "s63E6H2" 0.0229126 "hypothetical protein"
"JS1_D03_01138" 13047 "s59E13H2" 0.0002808361 "Competence protein A"
"JS1_D03_01138" 13047 "s59E25H2" 0 "Competence protein A"
"JS1_D03_01138" 13047 "s59E5H2" 0.002146342 "Competence protein A"
"JS1_D03_01138" 13047 "s63E6H2" 0.002505923 "Competence protein A"
"JS1_D03_01139" 13048 "s59E13H2" 0 "hypothetical protein"
"JS1_D03_01139" 13048 "s59E25H2" 0 "hypothetical protein"
"JS1_D03_01139" 13048 "s59E5H2" 0 "hypothetical protein"
"JS1_D03_01139" 13048 "s63E6H2" 0.0105718 "hypothetical protein"
"JS1_D03_01140" 13049 "s59E13H2" 0 "hypothetical protein"
"JS1_D03_01140" 13049 "s59E25H2" 0 "hypothetical protein"
"JS1_D03_01140" 13049 "s59E5H2" 0 "hypothetical protein"
"JS1_D03_01140" 13049 "s63E6H2" 0 "hypothetical protein"
"JS1_D03_01141" 13050 "s59E13H2" 0.06010791 "Copper-exporting P-type ATPase B"
"JS1_D03_01141" 13050 "s59E25H2" 0 "Copper-exporting P-type ATPase B"
"JS1_D03_01141" 13050 "s59E5H2" 2.394856 "Copper-exporting P-type ATPase B"
"JS1_D03_01141" 13050 "s63E6H2" 3.310414 "Copper-exporting P-type ATPase B"
"JS1_D03_01142" 13051 "s59E13H2" 0 "hypothetical protein"
"JS1_D03_01142" 13051 "s59E25H2" 0 "hypothetical protein"
"JS1_D03_01142" 13051 "s59E5H2" 0 "hypothetical protein"
"JS1_D03_01142" 13051 "s63E6H2" 0.07522411 "hypothetical protein"
"JS1_D03_01143" 13052 "s59E13H2" 0 "hypothetical protein"
"JS1_D03_01143" 13052 "s59E25H2" 0 "hypothetical protein"
"JS1_D03_01143" 13052 "s59E5H2" 0 "hypothetical protein"
"JS1_D03_01143" 13052 "s63E6H2" 0.005347523 "hypothetical protein"
"JS1_D03_01144" 13053 "s59E13H2" 0 "ubiquinone/menaquinone biosynthesis methyltransferase"
"JS1_D03_01144" 13053 "s59E25H2" 0 "ubiquinone/menaquinone biosynthesis methyltransferase"
"JS1_D03_01144" 13053 "s59E5H2" 0 "ubiquinone/menaquinone biosynthesis methyltransferase"
"JS1_D03_01144" 13053 "s63E6H2" 0.003862457 "ubiquinone/menaquinone biosynthesis methyltransferase"
"JS1_D03_01145" 13054 "s59E13H2" 0 "hypothetical protein"
"JS1_D03_01145" 13054 "s59E25H2" 0 "hypothetical protein"
"JS1_D03_01145" 13054 "s59E5H2" 0 "hypothetical protein"
"JS1_D03_01145" 13054 "s63E6H2" 0 "hypothetical protein"
"JS1_D03_01146" 13055 "s59E13H2" 0 "hypothetical protein"
"JS1_D03_01146" 13055 "s59E25H2" 0 "hypothetical protein"
"JS1_D03_01146" 13055 "s59E5H2" 0 "hypothetical protein"
"JS1_D03_01146" 13055 "s63E6H2" 0 "hypothetical protein"
"JS1_D03_01147" 13056 "s59E13H2" 0 "hypothetical protein"
"JS1_D03_01147" 13056 "s59E25H2" 0 "hypothetical protein"
"JS1_D03_01147" 13056 "s59E5H2" 0.01621173 "hypothetical protein"
"JS1_D03_01147" 13056 "s63E6H2" 0.009705532 "hypothetical protein"
"JS1_D03_01148" 13057 "s59E13H2" 0 "hypothetical protein"
"JS1_D03_01148" 13057 "s59E25H2" 0 "hypothetical protein"
"JS1_D03_01148" 13057 "s59E5H2" 0 "hypothetical protein"
"JS1_D03_01148" 13057 "s63E6H2" 0 "hypothetical protein"
"JS1_D03_01149" 13058 "s59E13H2" 0 "hypothetical protein"
"JS1_D03_01149" 13058 "s59E25H2" 0 "hypothetical protein"

"JS1_D03_01149" 13058 "s59E5H2" 0 "hypothetical protein"
"JS1_D03_01149" 13058 "s63E6H2" 0 "hypothetical protein"
"JS1_D03_01150" 13059 "s59E13H2" 0 "hypothetical protein"
"JS1_D03_01150" 13059 "s59E25H2" 0 "hypothetical protein"
"JS1_D03_01150" 13059 "s59E5H2" 0 "hypothetical protein"
"JS1_D03_01150" 13059 "s63E6H2" 0 "hypothetical protein"
"JS1_D03_01151" 13060 "s59E13H2" 0.009857967 "hypothetical protein"
"JS1_D03_01151" 13060 "s59E25H2" 0 "hypothetical protein"
"JS1_D03_01151" 13060 "s59E5H2" 0 "hypothetical protein"
"JS1_D03_01151" 13060 "s63E6H2" 0 "hypothetical protein"
"JS1_D03_01153" 13062 "s59E13H2" 0 "hypothetical protein"
"JS1_D03_01153" 13062 "s59E25H2" 0 "hypothetical protein"
"JS1_D03_01153" 13062 "s59E5H2" 0.03488841 "hypothetical protein"
"JS1_D03_01153" 13062 "s63E6H2" 0.004728336 "hypothetical protein"
"JS1_D03_01154" 13063 "s59E13H2" 0.3575065 "hypothetical protein"
"JS1_D03_01154" 13063 "s59E25H2" 0 "hypothetical protein"
"JS1_D03_01154" 13063 "s59E5H2" 0.06808564 "hypothetical protein"
"JS1_D03_01154" 13063 "s63E6H2" 0.1814985 "hypothetical protein"
"JS1_D03_01156" 13065 "s59E13H2" 0.141872 "Transposase IS200 like protein"
"JS1_D03_01156" 13065 "s59E25H2" 0 "Transposase IS200 like protein"
"JS1_D03_01156" 13065 "s59E5H2" 0.6232143 "Transposase IS200 like protein"
"JS1_D03_01156" 13065 "s63E6H2" 0.6168756 "Transposase IS200 like protein"
"JS1_D03_01157" 13066 "s59E13H2" 0 "hypothetical protein"
"JS1_D03_01157" 13066 "s59E25H2" 0 "hypothetical protein"
"JS1_D03_01157" 13066 "s59E5H2" 0 "hypothetical protein"
"JS1_D03_01157" 13066 "s63E6H2" 0 "hypothetical protein"
"JS1_D03_01158" 13067 "s59E13H2" 0.04949278 "Demethylrebeccamycin-D-glucose O-methyltransferase"
"JS1_D03_01158" 13067 "s59E25H2" 0 "Demethylrebeccamycin-D-glucose O-methyltransferase"
"JS1_D03_01158" 13067 "s59E5H2" 0.1286174 "Demethylrebeccamycin-D-glucose O-methyltransferase"
"JS1_D03_01158" 13067 "s63E6H2" 0.004464604 "Demethylrebeccamycin-D-glucose O-methyltransferase"
"JS1_D03_01159" 13068 "s59E13H2" 0.8123445 "Mycothiol acetyltransferase"
"JS1_D03_01159" 13068 "s59E25H2" 0 "Mycothiol acetyltransferase"
"JS1_D03_01159" 13068 "s59E5H2" 0.08259311 "Mycothiol acetyltransferase"
"JS1_D03_01159" 13068 "s63E6H2" 0.07552829 "Mycothiol acetyltransferase"
"JS1_D03_01160" 13069 "s59E13H2" 0 "hypothetical protein"
"JS1_D03_01160" 13069 "s59E25H2" 0 "hypothetical protein"
"JS1_D03_01160" 13069 "s59E5H2" 0 "hypothetical protein"
"JS1_D03_01160" 13069 "s63E6H2" 0 "hypothetical protein"
"JS1_D03_01164" 13073 "s59E13H2" 0 "30S ribosomal protein S2"
"JS1_D03_01164" 13073 "s59E25H2" 0 "30S ribosomal protein S2"
"JS1_D03_01164" 13073 "s59E5H2" 0 "30S ribosomal protein S2"
"JS1_D03_01164" 13073 "s63E6H2" 0.04132366 "30S ribosomal protein S2"
"JS1_D03_01165" 13074 "s59E13H2" 0 "Elongation factor Ts"
"JS1_D03_01165" 13074 "s59E25H2" 0 "Elongation factor Ts"
"JS1_D03_01165" 13074 "s59E5H2" 0.004971598 "Elongation factor Ts"
"JS1_D03_01165" 13074 "s63E6H2" 0.01386078 "Elongation factor Ts"
"JS1_D03_01166" 13075 "s59E13H2" 0.00797961 "Uridylate kinase"

"JS1_D03_01166" 13075 "s59E25H2" 0 "Uridylate kinase"
"JS1_D03_01166" 13075 "s59E5H2" 0.06435157 "Uridylate kinase"
"JS1_D03_01166" 13075 "s63E6H2" 0.09325799 "Uridylate kinase"
"JS1_D03_01167" 13076 "s59E13H2" 0.03598709 "Ribosome-recycling factor"
"JS1_D03_01167" 13076 "s59E25H2" 0 "Ribosome-recycling factor"
"JS1_D03_01167" 13076 "s59E5H2" 0.04047122 "Ribosome-recycling factor"
"JS1_D03_01167" 13076 "s63E6H2" 0.07741834 "Ribosome-recycling factor"
"JS1_D03_01168" 13077 "s59E13H2" 0.02546316 "Ferredoxin"
"JS1_D03_01168" 13077 "s59E25H2" 0 "Ferredoxin"
"JS1_D03_01168" 13077 "s59E5H2" 0.073095 "Ferredoxin"
"JS1_D03_01168" 13077 "s63E6H2" 0.02243293 "Ferredoxin"
"JS1_D03_01169" 13078 "s59E13H2" 0.01402203 "Isoprenyl transferase"
"JS1_D03_01169" 13078 "s59E25H2" 0 "Isoprenyl transferase"
"JS1_D03_01169" 13078 "s59E5H2" 0.1021791 "Isoprenyl transferase"
"JS1_D03_01169" 13078 "s63E6H2" 0.08182417 "Isoprenyl transferase"
"JS1_D03_01170" 13079 "s59E13H2" 0.006820204 "Phosphatidate cytidylyltransferase"
"JS1_D03_01170" 13079 "s59E25H2" 0 "Phosphatidate cytidylyltransferase"
"JS1_D03_01170" 13079 "s59E5H2" 0.02114231 "Phosphatidate cytidylyltransferase"
"JS1_D03_01170" 13079 "s63E6H2" 0.0439824 "Phosphatidate cytidylyltransferase"
"JS1_D03_01171" 13080 "s59E13H2" 0.01247985 "1-deoxy-D-xylulose 5-phosphate reductoisomerase"
"JS1_D03_01171" 13080 "s59E25H2" 0 "1-deoxy-D-xylulose 5-phosphate reductoisomerase"
"JS1_D03_01171" 13080 "s59E5H2" 0.01331678 "1-deoxy-D-xylulose 5-phosphate reductoisomerase"
"JS1_D03_01171" 13080 "s63E6H2" 0.05036609 "1-deoxy-D-xylulose 5-phosphate reductoisomerase"
"JS1_D03_01172" 13081 "s59E13H2" 0.007022026 "Regulator of sigma-W protease RasP"
"JS1_D03_01172" 13081 "s59E25H2" 0 "Regulator of sigma-W protease RasP"
"JS1_D03_01172" 13081 "s59E5H2" 0.05845204 "Regulator of sigma-W protease RasP"
"JS1_D03_01172" 13081 "s63E6H2" 0.05883034 "Regulator of sigma-W protease RasP"
"JS1_D03_01173" 13082 "s59E13H2" 0.00778128 "4-hydroxy-3-methylbut-2-en-1-yl diphosphate synthase"
"JS1_D03_01173" 13082 "s59E25H2" 0 "4-hydroxy-3-methylbut-2-en-1-yl diphosphate synthase"
"JS1_D03_01173" 13082 "s59E5H2" 0.008698603 "4-hydroxy-3-methylbut-2-en-1-yl diphosphate synthase"
"JS1_D03_01173" 13082 "s63E6H2" 0.01070948 "4-hydroxy-3-methylbut-2-en-1-yl diphosphate synthase"
"JS1_D03_01174" 13083 "s59E13H2" 0 "V-type ATP synthase subunit H"
"JS1_D03_01174" 13083 "s59E25H2" 0 "V-type ATP synthase subunit H"
"JS1_D03_01174" 13083 "s59E5H2" 0.02334897 "V-type ATP synthase subunit H"
"JS1_D03_01174" 13083 "s63E6H2" 0.01414677 "V-type ATP synthase subunit H"
"JS1_D03_01175" 13084 "s59E13H2" 0.000843736 "V-type ATP synthase subunit I"
"JS1_D03_01175" 13084 "s59E25H2" 0 "V-type ATP synthase subunit I"
"JS1_D03_01175" 13084 "s59E5H2" 0.01623611 "V-type ATP synthase subunit I"
"JS1_D03_01175" 13084 "s63E6H2" 0.01337444 "V-type ATP synthase subunit I"
"JS1_D03_01176" 13085 "s59E13H2" 0 "V-type sodium ATPase subunit K"
"JS1_D03_01176" 13085 "s59E25H2" 0 "V-type sodium ATPase subunit K"
"JS1_D03_01176" 13085 "s59E5H2" 0.05013062 "V-type sodium ATPase subunit K"
"JS1_D03_01176" 13085 "s63E6H2" 0.03911392 "V-type sodium ATPase subunit K"
"JS1_D03_01177" 13086 "s59E13H2" 0 "V-type ATP synthase subunit E"
"JS1_D03_01177" 13086 "s59E25H2" 0 "V-type ATP synthase subunit E"
"JS1_D03_01177" 13086 "s59E5H2" 0.05200246 "V-type ATP synthase subunit E"
"JS1_D03_01177" 13086 "s63E6H2" 0.009480433 "V-type ATP synthase subunit E"

"JS1_D03_01178" 13087 "s59E13H2" 0.003293368 "V-type sodium ATPase subunit C"
"JS1_D03_01178" 13087 "s59E25H2" 0 "V-type sodium ATPase subunit C"
"JS1_D03_01178" 13087 "s59E5H2" 0.1894746 "V-type sodium ATPase subunit C"
"JS1_D03_01178" 13087 "s63E6H2" 0.01387414 "V-type sodium ATPase subunit C"
"JS1_D03_01179" 13088 "s59E13H2" 0.1373389 "V-type sodium ATPase subunit G"
"JS1_D03_01179" 13088 "s59E25H2" 0 "V-type sodium ATPase subunit G"
"JS1_D03_01179" 13088 "s59E5H2" 0.2933552 "V-type sodium ATPase subunit G"
"JS1_D03_01179" 13088 "s63E6H2" 0.04764664 "V-type sodium ATPase subunit G"
"JS1_D03_01180" 13089 "s59E13H2" 0.08004282 "V-type sodium ATPase catalytic subunit A"
"JS1_D03_01180" 13089 "s59E25H2" 0 "V-type sodium ATPase catalytic subunit A"
"JS1_D03_01180" 13089 "s59E5H2" 0.2229737 "V-type sodium ATPase catalytic subunit A"
"JS1_D03_01180" 13089 "s63E6H2" 0.1061953 "V-type sodium ATPase catalytic subunit A"
"JS1_D03_01181" 13090 "s59E13H2" 0.07627524 "V-type sodium ATPase subunit B"
"JS1_D03_01181" 13090 "s59E25H2" 0 "V-type sodium ATPase subunit B"
"JS1_D03_01181" 13090 "s59E5H2" 0.5080709 "V-type sodium ATPase subunit B"
"JS1_D03_01181" 13090 "s63E6H2" 0.3594138 "V-type sodium ATPase subunit B"
"JS1_D03_01182" 13091 "s59E13H2" 0.01319086 "V-type sodium ATPase subunit D"
"JS1_D03_01182" 13091 "s59E25H2" 0 "V-type sodium ATPase subunit D"
"JS1_D03_01182" 13091 "s59E5H2" 0.2123527 "V-type sodium ATPase subunit D"
"JS1_D03_01182" 13091 "s63E6H2" 0.05092635 "V-type sodium ATPase subunit D"
"JS1_D03_01183" 13092 "s59E13H2" 0.07135397 "phosphodiesterase"
"JS1_D03_01183" 13092 "s59E25H2" 0 "phosphodiesterase"
"JS1_D03_01183" 13092 "s59E5H2" 0.3187425 "phosphodiesterase"
"JS1_D03_01183" 13092 "s63E6H2" 0.1691035 "phosphodiesterase"
"JS1_D03_01185" 13094 "s59E13H2" 0.05572391 "1-acyl-sn-glycerol-3-phosphate acyltransferase"
"JS1_D03_01185" 13094 "s59E25H2" 0 "1-acyl-sn-glycerol-3-phosphate acyltransferase"
"JS1_D03_01185" 13094 "s59E5H2" 0.2424643 "1-acyl-sn-glycerol-3-phosphate acyltransferase"
"JS1_D03_01185" 13094 "s63E6H2" 0.2767411 "1-acyl-sn-glycerol-3-phosphate acyltransferase"
"JS1_D03_01186" 13095 "s59E13H2" 0.08538455 "Serine/threonine-protein kinase PknD"
"JS1_D03_01186" 13095 "s59E25H2" 0 "Serine/threonine-protein kinase PknD"
"JS1_D03_01186" 13095 "s59E5H2" 0.6660377 "Serine/threonine-protein kinase PknD"
"JS1_D03_01186" 13095 "s63E6H2" 0.5144267 "Serine/threonine-protein kinase PknD"
"JS1_D03_01187" 13096 "s59E13H2" 0 "Glutamate 2,3-aminomutase"
"JS1_D03_01187" 13096 "s59E25H2" 0 "Glutamate 2,3-aminomutase"
"JS1_D03_01187" 13096 "s59E5H2" 0.007157687 "Glutamate 2,3-aminomutase"
"JS1_D03_01187" 13096 "s63E6H2" 0.01750001 "Glutamate 2,3-aminomutase"
"JS1_D03_01188" 13097 "s59E13H2" 0 "Polysaccharide biosynthesis protein"
"JS1_D03_01188" 13097 "s59E25H2" 0 "Polysaccharide biosynthesis protein"
"JS1_D03_01188" 13097 "s59E5H2" 0 "Polysaccharide biosynthesis protein"
"JS1_D03_01188" 13097 "s63E6H2" 0.01375077 "Polysaccharide biosynthesis protein"
"JS1_D03_01189" 13098 "s59E13H2" 0 "hypothetical protein"
"JS1_D03_01189" 13098 "s59E25H2" 0 "hypothetical protein"
"JS1_D03_01189" 13098 "s59E5H2" 0 "hypothetical protein"
"JS1_D03_01189" 13098 "s63E6H2" 0 "hypothetical protein"
"JS1_D03_01190" 13099 "s59E13H2" 0 "GDP-mannose-dependent alpha-(1-6)-phosphatidylinositol monomannoside mannosyltransferase"
"JS1_D03_01190" 13099 "s59E25H2" 0 "GDP-mannose-dependent alpha-(1-6)-phosphatidylinositol monomannoside mannosyltransferase"
"JS1_D03_01190" 13099 "s59E5H2" 0 "GDP-mannose-dependent alpha-(1-6)-phosphatidylinositol monomannoside mannosyltransferase"

"JS1_D03_01190" 13099 "s63E6H2" 0.001211933 "GDP-mannose-dependent alpha-(1-6)-phosphatidylinositol monomannoside mannosyltransferase"
"JS1_D03_01191" 13100 "s59E13H2" 0 "O-Antigen ligase"
"JS1_D03_01191" 13100 "s59E25H2" 0 "O-Antigen ligase"
"JS1_D03_01191" 13100 "s59E5H2" 0 "O-Antigen ligase"
"JS1_D03_01191" 13100 "s63E6H2" 0.003965298 "O-Antigen ligase"
"JS1_D03_01192" 13101 "s59E13H2" 0 "Putative teichuronic acid biosynthesis glycosyltransferase TuaC"
"JS1_D03_01192" 13101 "s59E25H2" 0 "Putative teichuronic acid biosynthesis glycosyltransferase TuaC"
"JS1_D03_01192" 13101 "s59E5H2" 0 "Putative teichuronic acid biosynthesis glycosyltransferase TuaC"
"JS1_D03_01192" 13101 "s63E6H2" 0.01455345 "Putative teichuronic acid biosynthesis glycosyltransferase TuaC"
"JS1_D03_01193" 13102 "s59E13H2" 0 "hypothetical protein"
"JS1_D03_01193" 13102 "s59E25H2" 0 "hypothetical protein"
"JS1_D03_01193" 13102 "s59E5H2" 0 "hypothetical protein"
"JS1_D03_01193" 13102 "s63E6H2" 0 "hypothetical protein"
"JS1_D03_01195" 13104 "s59E13H2" 0 "putative glycosyl transferase"
"JS1_D03_01195" 13104 "s59E25H2" 0 "putative glycosyl transferase"
"JS1_D03_01195" 13104 "s59E5H2" 0 "putative glycosyl transferase"
"JS1_D03_01195" 13104 "s63E6H2" 0 "putative glycosyl transferase"
"JS1_D03_01196" 13105 "s59E13H2" 0 "Alpha-D-kanosaminyltransferase"
"JS1_D03_01196" 13105 "s59E25H2" 0 "Alpha-D-kanosaminyltransferase"
"JS1_D03_01196" 13105 "s59E5H2" 0 "Alpha-D-kanosaminyltransferase"
"JS1_D03_01196" 13105 "s63E6H2" 0 "Alpha-D-kanosaminyltransferase"
"JS1_D03_01197" 13106 "s59E13H2" 0 "putative sugar transferase EpsL"
"JS1_D03_01197" 13106 "s59E25H2" 0 "putative sugar transferase EpsL"
"JS1_D03_01197" 13106 "s59E5H2" 0 "putative sugar transferase EpsL"
"JS1_D03_01197" 13106 "s63E6H2" 0.0192184 "putative sugar transferase EpsL"
"JS1_D03_01198" 13107 "s59E13H2" 0 "Galactoside O-acetyltransferase"
"JS1_D03_01198" 13107 "s59E25H2" 0 "Galactoside O-acetyltransferase"
"JS1_D03_01198" 13107 "s59E5H2" 0 "Galactoside O-acetyltransferase"
"JS1_D03_01198" 13107 "s63E6H2" 0.02103828 "Galactoside O-acetyltransferase"
"JS1_D03_01199" 13108 "s59E13H2" 0 "Putative pyridoxal phosphate-dependent aminotransferase EpsN"
"JS1_D03_01199" 13108 "s59E25H2" 0 "Putative pyridoxal phosphate-dependent aminotransferase EpsN"
"JS1_D03_01199" 13108 "s59E5H2" 0.004591185 "Putative pyridoxal phosphate-dependent aminotransferase EpsN"
"JS1_D03_01199" 13108 "s63E6H2" 0.02214637 "Putative pyridoxal phosphate-dependent aminotransferase EpsN"
"JS1_D03_01200" 13109 "s59E13H2" 0 "hypothetical protein"
"JS1_D03_01200" 13109 "s59E25H2" 0 "hypothetical protein"
"JS1_D03_01200" 13109 "s59E5H2" 0 "hypothetical protein"
"JS1_D03_01200" 13109 "s63E6H2" 0.03205993 "hypothetical protein"
"JS1_D03_01201" 13110 "s59E13H2" 0.05896943 "mRNA interferase MazF9"
"JS1_D03_01201" 13110 "s59E25H2" 0 "mRNA interferase MazF9"
"JS1_D03_01201" 13110 "s59E5H2" 0.0008666649 "mRNA interferase MazF9"
"JS1_D03_01201" 13110 "s63E6H2" 0.02305542 "mRNA interferase MazF9"
"JS1_D03_01202" 13111 "s59E13H2" 0.1325223 "hypothetical protein"
"JS1_D03_01202" 13111 "s59E25H2" 0 "hypothetical protein"
"JS1_D03_01202" 13111 "s59E5H2" 0.04621766 "hypothetical protein"
"JS1_D03_01202" 13111 "s63E6H2" 0.0376756 "hypothetical protein"
"JS1_D03_01203" 13112 "s59E13H2" 0.006009632 "UDP-N-acetyl-alpha-D-glucosamine C6 dehydratase"
"JS1_D03_01203" 13112 "s59E25H2" 0 "UDP-N-acetyl-alpha-D-glucosamine C6 dehydratase"

"JS1_D03_01203" 13112 "s59E5H2" 0.002040517 "UDP-N-acetyl-alpha-D-glucosamine C6 dehydratase"
"JS1_D03_01203" 13112 "s63E6H2" 0.07320113 "UDP-N-acetyl-alpha-D-glucosamine C6 dehydratase"
"JS1_D03_01204" 13113 "s59E13H2" 0 "Nucleotidyltransferase domain protein"
"JS1_D03_01204" 13113 "s59E25H2" 0 "Nucleotidyltransferase domain protein"
"JS1_D03_01204" 13113 "s59E5H2" 0 "Nucleotidyltransferase domain protein"
"JS1_D03_01204" 13113 "s63E6H2" 0.02474059 "Nucleotidyltransferase domain protein"
"JS1_D03_01205" 13114 "s59E13H2" 0 "hypothetical protein"
"JS1_D03_01205" 13114 "s59E25H2" 0 "hypothetical protein"
"JS1_D03_01205" 13114 "s59E5H2" 0 "hypothetical protein"
"JS1_D03_01205" 13114 "s63E6H2" 0.04207469 "hypothetical protein"
"JS1_D03_01206" 13115 "s59E13H2" 0.01114201 "Pseudouridine kinase"
"JS1_D03_01206" 13115 "s59E25H2" 0 "Pseudouridine kinase"
"JS1_D03_01206" 13115 "s59E5H2" 0.001781007 "Pseudouridine kinase"
"JS1_D03_01206" 13115 "s63E6H2" 0.03044211 "Pseudouridine kinase"
"JS1_D03_01207" 13116 "s59E13H2" 0.004041328 "Pseudouridine-5'-phosphate glycosidase"
"JS1_D03_01207" 13116 "s59E25H2" 0 "Pseudouridine-5'-phosphate glycosidase"
"JS1_D03_01207" 13116 "s59E5H2" 0.009006518 "Pseudouridine-5'-phosphate glycosidase"
"JS1_D03_01207" 13116 "s63E6H2" 0.02017986 "Pseudouridine-5'-phosphate glycosidase"
"JS1_D03_01208" 13117 "s59E13H2" 0.02257587 "Pseudouridine-5'-phosphate glycosidase"
"JS1_D03_01208" 13117 "s59E25H2" 0 "Pseudouridine-5'-phosphate glycosidase"
"JS1_D03_01208" 13117 "s59E5H2" 0.01540887 "Pseudouridine-5'-phosphate glycosidase"
"JS1_D03_01208" 13117 "s63E6H2" 0.1023405 "Pseudouridine-5'-phosphate glycosidase"
"JS1_D03_01209" 13118 "s59E13H2" 0.08136869 "Phage shock protein C"
"JS1_D03_01209" 13118 "s59E25H2" 0 "Phage shock protein C"
"JS1_D03_01209" 13118 "s59E5H2" 0.8143146 "Phage shock protein C"
"JS1_D03_01209" 13118 "s63E6H2" 0.1820965 "Phage shock protein C"
"JS1_D03_01210" 13119 "s59E13H2" 0.1201311 "Epoxyqueuosine reductase"
"JS1_D03_01210" 13119 "s59E25H2" 0 "Epoxyqueuosine reductase"
"JS1_D03_01210" 13119 "s59E5H2" 0.09650041 "Epoxyqueuosine reductase"
"JS1_D03_01210" 13119 "s63E6H2" 0.1971989 "Epoxyqueuosine reductase"
"JS1_D03_01211" 13120 "s59E13H2" 0.8253411 "putative ABC transporter ATP-binding protein YbhF"
"JS1_D03_01211" 13120 "s59E25H2" 0 "putative ABC transporter ATP-binding protein YbhF"
"JS1_D03_01211" 13120 "s59E5H2" 1.870621 "putative ABC transporter ATP-binding protein YbhF"
"JS1_D03_01211" 13120 "s63E6H2" 1.052791 "putative ABC transporter ATP-binding protein YbhF"
"JS1_D03_01212" 13121 "s59E13H2" 0.5924545 "ABC-2 family transporter protein"
"JS1_D03_01212" 13121 "s59E25H2" 0 "ABC-2 family transporter protein"
"JS1_D03_01212" 13121 "s59E5H2" 1.517535 "ABC-2 family transporter protein"
"JS1_D03_01212" 13121 "s63E6H2" 0.8076341 "ABC-2 family transporter protein"
"JS1_D03_01213" 13122 "s59E13H2" 0 "Acyl-CoA dehydrogenase"
"JS1_D03_01213" 13122 "s59E25H2" 0 "Acyl-CoA dehydrogenase"
"JS1_D03_01213" 13122 "s59E5H2" 0 "Acyl-CoA dehydrogenase"
"JS1_D03_01213" 13122 "s63E6H2" 0.06808489 "Acyl-CoA dehydrogenase"
"JS1_D03_01214" 13123 "s59E13H2" 0 "PEGA domain protein"
"JS1_D03_01214" 13123 "s59E25H2" 0 "PEGA domain protein"
"JS1_D03_01214" 13123 "s59E5H2" 0.7417303 "PEGA domain protein"
"JS1_D03_01214" 13123 "s63E6H2" 0.1820591 "PEGA domain protein"
"JS1_D03_01215" 13124 "s59E13H2" 0.1065954 "Protein-L-isoaspartate O-methyltransferase"

"JS1_D03_01215" 13124 "s59E25H2" 0 "Protein-L-isoaspartate O-methyltransferase"
"JS1_D03_01215" 13124 "s59E5H2" 0.3980512 "Protein-L-isoaspartate O-methyltransferase"
"JS1_D03_01215" 13124 "s63E6H2" 0.1965554 "Protein-L-isoaspartate O-methyltransferase"
"JS1_D03_01216" 13125 "s59E13H2" 0.6542615 "hypothetical protein"
"JS1_D03_01216" 13125 "s59E25H2" 0 "hypothetical protein"
"JS1_D03_01216" 13125 "s59E5H2" 0.9999267 "hypothetical protein"
"JS1_D03_01216" 13125 "s63E6H2" 1.219393 "hypothetical protein"
"JS1_D03_01217" 13126 "s59E13H2" 0.8588761 "Alanine--tRNA ligase"
"JS1_D03_01217" 13126 "s59E25H2" 0 "Alanine--tRNA ligase"
"JS1_D03_01217" 13126 "s59E5H2" 0.1184192 "Alanine--tRNA ligase"
"JS1_D03_01217" 13126 "s63E6H2" 0.5200021 "Alanine--tRNA ligase"
"JS1_D03_01218" 13127 "s59E13H2" 3.169673 "putative deoxyribonuclease YcfH"
"JS1_D03_01218" 13127 "s59E25H2" 0 "putative deoxyribonuclease YcfH"
"JS1_D03_01218" 13127 "s59E5H2" 0.08689489 "putative deoxyribonuclease YcfH"
"JS1_D03_01218" 13127 "s63E6H2" 0.3320589 "putative deoxyribonuclease YcfH"
"JS1_D03_01219" 13128 "s59E13H2" 4.81197 "hypothetical protein"
"JS1_D03_01219" 13128 "s59E25H2" 0 "hypothetical protein"
"JS1_D03_01219" 13128 "s59E5H2" 0.1912813 "hypothetical protein"
"JS1_D03_01219" 13128 "s63E6H2" 0.7066879 "hypothetical protein"
"JS1_D03_01220" 13129 "s59E13H2" 0.2183986 "hypothetical protein"
"JS1_D03_01220" 13129 "s59E25H2" 0 "hypothetical protein"
"JS1_D03_01220" 13129 "s59E5H2" 0.05850143 "hypothetical protein"
"JS1_D03_01220" 13129 "s63E6H2" 0.3427123 "hypothetical protein"
"JS1_D03_01221" 13130 "s59E13H2" 0.003518142 "hypothetical protein"
"JS1_D03_01221" 13130 "s59E25H2" 0 "hypothetical protein"
"JS1_D03_01221" 13130 "s59E5H2" 0.003686194 "hypothetical protein"
"JS1_D03_01221" 13130 "s63E6H2" 0.07311133 "hypothetical protein"
"JS1_D03_01222" 13131 "s59E13H2" 0.8037206 "hypothetical protein"
"JS1_D03_01222" 13131 "s59E25H2" 0 "hypothetical protein"
"JS1_D03_01222" 13131 "s59E5H2" 0.1897373 "hypothetical protein"
"JS1_D03_01222" 13131 "s63E6H2" 0.4322407 "hypothetical protein"
"JS1_D03_01223" 13132 "s59E13H2" 0.01490968 "hypothetical protein"
"JS1_D03_01223" 13132 "s59E25H2" 0 "hypothetical protein"
"JS1_D03_01223" 13132 "s59E5H2" 0.03715682 "hypothetical protein"
"JS1_D03_01223" 13132 "s63E6H2" 0.1118086 "hypothetical protein"
"JS1_D03_01224" 13133 "s59E13H2" 0 "hypothetical protein"
"JS1_D03_01224" 13133 "s59E25H2" 0 "hypothetical protein"
"JS1_D03_01224" 13133 "s59E5H2" 0 "hypothetical protein"
"JS1_D03_01224" 13133 "s63E6H2" 0 "hypothetical protein"
"JS1_D03_01225" 13134 "s59E13H2" 0.03073786 "hypothetical protein"
"JS1_D03_01225" 13134 "s59E25H2" 0 "hypothetical protein"
"JS1_D03_01225" 13134 "s59E5H2" 0.09097204 "hypothetical protein"
"JS1_D03_01225" 13134 "s63E6H2" 0.1772497 "hypothetical protein"
"JS1_D03_01226" 13135 "s59E13H2" 0.2406928 "NTE family protein RssA"
"JS1_D03_01226" 13135 "s59E25H2" 0 "NTE family protein RssA"
"JS1_D03_01226" 13135 "s59E5H2" 0.2183785 "NTE family protein RssA"
"JS1_D03_01226" 13135 "s63E6H2" 0.4088162 "NTE family protein RssA"

"JS1_D03_01228" 13137 "s59E13H2" 0.08697273 "50S ribosomal protein L22"
"JS1_D03_01228" 13137 "s59E25H2" 0 "50S ribosomal protein L22"
"JS1_D03_01228" 13137 "s59E5H2" 0.03112653 "50S ribosomal protein L22"
"JS1_D03_01228" 13137 "s63E6H2" 0.05411378 "50S ribosomal protein L22"
"JS1_D03_01229" 13138 "s59E13H2" 0 "Adenylate kinase"
"JS1_D03_01229" 13138 "s59E25H2" 0 "Adenylate kinase"
"JS1_D03_01229" 13138 "s59E5H2" 0 "Adenylate kinase"
"JS1_D03_01229" 13138 "s63E6H2" 0.02190596 "Adenylate kinase"
"JS1_D03_01230" 13139 "s59E13H2" 0.002029327 "Endonuclease YhcR precursor"
"JS1_D03_01230" 13139 "s59E25H2" 0.1705426 "Endonuclease YhcR precursor"
"JS1_D03_01230" 13139 "s59E5H2" 0.01942209 "Endonuclease YhcR precursor"
"JS1_D03_01230" 13139 "s63E6H2" 0.9618601 "Endonuclease YhcR precursor"
"JS1_D03_01231" 13140 "s59E13H2" 0.03634018 "Leucine-, isoleucine-, valine-, threonine-, and alanine-binding protein precursor"
"JS1_D03_01231" 13140 "s59E25H2" 0 "Leucine-, isoleucine-, valine-, threonine-, and alanine-binding protein precursor"
"JS1_D03_01231" 13140 "s59E5H2" 0.1850189 "Leucine-, isoleucine-, valine-, threonine-, and alanine-binding protein precursor"
"JS1_D03_01231" 13140 "s63E6H2" 7.777866 "Leucine-, isoleucine-, valine-, threonine-, and alanine-binding protein precursor"
"JS1_D03_01232" 13141 "s59E13H2" 0.7570626 "High-affinity branched-chain amino acid transport system permease protein LivH"
"JS1_D03_01232" 13141 "s59E25H2" 0.8310962 "High-affinity branched-chain amino acid transport system permease protein LivH"
"JS1_D03_01232" 13141 "s59E5H2" 0.2207625 "High-affinity branched-chain amino acid transport system permease protein LivH"
"JS1_D03_01232" 13141 "s63E6H2" 9.341257 "High-affinity branched-chain amino acid transport system permease protein LivH"
"JS1_D03_01233" 13142 "s59E13H2" 0.7255504 "leucine/isoleucine/valine transporter permease subunit"
"JS1_D03_01233" 13142 "s59E25H2" 0.2350187 "leucine/isoleucine/valine transporter permease subunit"
"JS1_D03_01233" 13142 "s59E5H2" 0.3403553 "leucine/isoleucine/valine transporter permease subunit"
"JS1_D03_01233" 13142 "s63E6H2" 9.585463 "leucine/isoleucine/valine transporter permease subunit"
"JS1_D03_01234" 13143 "s59E13H2" 0.6171274 "putative branched-chain amino acid transport ATP-binding protein LivG"
"JS1_D03_01234" 13143 "s59E25H2" 0 "putative branched-chain amino acid transport ATP-binding protein LivG"
"JS1_D03_01234" 13143 "s59E5H2" 0.5577166 "putative branched-chain amino acid transport ATP-binding protein LivG"
"JS1_D03_01234" 13143 "s63E6H2" 6.388606 "putative branched-chain amino acid transport ATP-binding protein LivG"
"JS1_D03_01235" 13144 "s59E13H2" 0.3505816 "High-affinity branched-chain amino acid transport ATP-binding protein LivF"
"JS1_D03_01235" 13144 "s59E25H2" 0 "High-affinity branched-chain amino acid transport ATP-binding protein LivF"
"JS1_D03_01235" 13144 "s59E5H2" 0.376871 "High-affinity branched-chain amino acid transport ATP-binding protein LivF"
"JS1_D03_01235" 13144 "s63E6H2" 0.4316037 "High-affinity branched-chain amino acid transport ATP-binding protein LivF"
"JS1_D03_01236" 13145 "s59E13H2" 0.008121057 "Poly-beta-1,6-N-acetyl-D-glucosamine N-deacetylase precursor"
"JS1_D03_01236" 13145 "s59E25H2" 0 "Poly-beta-1,6-N-acetyl-D-glucosamine N-deacetylase precursor"
"JS1_D03_01236" 13145 "s59E5H2" 0.5839403 "Poly-beta-1,6-N-acetyl-D-glucosamine N-deacetylase precursor"
"JS1_D03_01236" 13145 "s63E6H2" 0.05665876 "Poly-beta-1,6-N-acetyl-D-glucosamine N-deacetylase precursor"
"JS1_D03_01237" 13146 "s59E13H2" 0.02405224 "Pyrimidine 5'-nucleotidase YjjG"
"JS1_D03_01237" 13146 "s59E25H2" 0 "Pyrimidine 5'-nucleotidase YjjG"
"JS1_D03_01237" 13146 "s59E5H2" 0.04440605 "Pyrimidine 5'-nucleotidase YjjG"
"JS1_D03_01237" 13146 "s63E6H2" 0.05744488 "Pyrimidine 5'-nucleotidase YjjG"
"JS1_D03_01238" 13147 "s59E13H2" 0.2218851 "hypothetical protein"
"JS1_D03_01238" 13147 "s59E25H2" 0 "hypothetical protein"
"JS1_D03_01238" 13147 "s59E5H2" 0.466393 "hypothetical protein"
"JS1_D03_01238" 13147 "s63E6H2" 0.3827734 "hypothetical protein"
"JS1_D03_01239" 13148 "s59E13H2" 0.2871252 "putative sulfoacetate transporter SauU"
"JS1_D03_01239" 13148 "s59E25H2" 0 "putative sulfoacetate transporter SauU"
"JS1_D03_01239" 13148 "s59E5H2" 0.7235589 "putative sulfoacetate transporter SauU"

"JS1_D03_01239" 13148 "s63E6H2" 0.6284569 "putative sulfoacetate transporter SauU"
"JS1_D03_01240" 13149 "s59E13H2" 0.1207652 "Adenosine specific kinase"
"JS1_D03_01240" 13149 "s59E25H2" 0 "Adenosine specific kinase"
"JS1_D03_01240" 13149 "s59E5H2" 0.0815957 "Adenosine specific kinase"
"JS1_D03_01240" 13149 "s63E6H2" 0.08531144 "Adenosine specific kinase"
"JS1_D03_01241" 13150 "s59E13H2" 0.005680614 "DNA-directed RNA polymerase subunit beta'"
"JS1_D03_01241" 13150 "s59E25H2" 0 "DNA-directed RNA polymerase subunit beta'"
"JS1_D03_01241" 13150 "s59E5H2" 0.02401481 "DNA-directed RNA polymerase subunit beta'"
"JS1_D03_01241" 13150 "s63E6H2" 0.02878945 "DNA-directed RNA polymerase subunit beta'"
"JS1_D03_01242" 13151 "s59E13H2" 0 "50S ribosomal protein L18"
"JS1_D03_01242" 13151 "s59E25H2" 0 "50S ribosomal protein L18"
"JS1_D03_01242" 13151 "s59E5H2" 0.01936762 "50S ribosomal protein L18"
"JS1_D03_01242" 13151 "s63E6H2" 0.005284929 "50S ribosomal protein L18"
"JS1_D03_01243" 13152 "s59E13H2" 0.005744804 "50S ribosomal protein L6"
"JS1_D03_01243" 13152 "s59E25H2" 0 "50S ribosomal protein L6"
"JS1_D03_01243" 13152 "s59E5H2" 0 "50S ribosomal protein L6"
"JS1_D03_01243" 13152 "s63E6H2" 0.004469404 "50S ribosomal protein L6"
"JS1_D03_01244" 13153 "s59E13H2" 0.008489658 "30S ribosomal protein S8"
"JS1_D03_01244" 13153 "s59E25H2" 0 "30S ribosomal protein S8"
"JS1_D03_01244" 13153 "s59E5H2" 0.02015293 "30S ribosomal protein S8"
"JS1_D03_01244" 13153 "s63E6H2" 0.0824437 "30S ribosomal protein S8"
"JS1_D03_01245" 13154 "s59E13H2" 0 "30S ribosomal protein S14 type Z"
"JS1_D03_01245" 13154 "s59E25H2" 0 "30S ribosomal protein S14 type Z"
"JS1_D03_01245" 13154 "s59E5H2" 0 "30S ribosomal protein S14 type Z"
"JS1_D03_01245" 13154 "s63E6H2" 0.1064856 "30S ribosomal protein S14 type Z"
"JS1_D03_01251" 13160 "s59E13H2" 0 "hypothetical protein"
"JS1_D03_01251" 13160 "s59E25H2" 0 "hypothetical protein"
"JS1_D03_01251" 13160 "s59E5H2" 0.6352236 "hypothetical protein"
"JS1_D03_01251" 13160 "s63E6H2" 0.07509979 "hypothetical protein"
"JS1_D03_01252" 13161 "s59E13H2" 0 "hypothetical protein"
"JS1_D03_01252" 13161 "s59E25H2" 0 "hypothetical protein"
"JS1_D03_01252" 13161 "s59E5H2" 0 "hypothetical protein"
"JS1_D03_01252" 13161 "s63E6H2" 0.006027988 "hypothetical protein"
"JS1_D03_01253" 13162 "s59E13H2" 0 "sporulation sigma factor SigG"
"JS1_D03_01253" 13162 "s59E25H2" 0 "sporulation sigma factor SigG"
"JS1_D03_01253" 13162 "s59E5H2" 0 "sporulation sigma factor SigG"
"JS1_D03_01253" 13162 "s63E6H2" 0.0243474 "sporulation sigma factor SigG"
"JS1_D03_01254" 13163 "s59E13H2" 0 "RNA polymerase factor sigma-70"
"JS1_D03_01254" 13163 "s59E25H2" 0 "RNA polymerase factor sigma-70"
"JS1_D03_01254" 13163 "s59E5H2" 0 "RNA polymerase factor sigma-70"
"JS1_D03_01254" 13163 "s63E6H2" 0.03908383 "RNA polymerase factor sigma-70"
"JS1_D03_01256" 13165 "s59E13H2" 0.003632857 "30S ribosomal protein S7"
"JS1_D03_01256" 13165 "s59E25H2" 0 "30S ribosomal protein S7"
"JS1_D03_01256" 13165 "s59E5H2" 0.0267442 "30S ribosomal protein S7"
"JS1_D03_01256" 13165 "s63E6H2" 0.1449994 "30S ribosomal protein S7"
"JS1_D03_01257" 13166 "s59E13H2" 0.007982842 "Elongation factor G"
"JS1_D03_01257" 13166 "s59E25H2" 0 "Elongation factor G"

"JS1_D03_01257" 13166 "s59E5H2" 0.02273104 "Elongation factor G"
"JS1_D03_01257" 13166 "s63E6H2" 0.2666198 "Elongation factor G"
"JS1_D03_01258" 13167 "s59E13H2" 0.09150797 "Inosine-5'-monophosphate dehydrogenase"
"JS1_D03_01258" 13167 "s59E25H2" 0 "Inosine-5'-monophosphate dehydrogenase"
"JS1_D03_01258" 13167 "s59E5H2" 0.2438658 "Inosine-5'-monophosphate dehydrogenase"
"JS1_D03_01258" 13167 "s63E6H2" 0.1637528 "Inosine-5'-monophosphate dehydrogenase"
"JS1_D03_01259" 13168 "s59E13H2" 0.2508158 "lipoprotein NlpI"
"JS1_D03_01259" 13168 "s59E25H2" 0 "lipoprotein NlpI"
"JS1_D03_01259" 13168 "s59E5H2" 0.0666583 "lipoprotein NlpI"
"JS1_D03_01259" 13168 "s63E6H2" 0.3374062 "lipoprotein NlpI"
"JS1_D03_01260" 13169 "s59E13H2" 0.1777331 "Membrane dipeptidase (Peptidase family M19)"
"JS1_D03_01260" 13169 "s59E25H2" 0 "Membrane dipeptidase (Peptidase family M19)"
"JS1_D03_01260" 13169 "s59E5H2" 0.2569535 "Membrane dipeptidase (Peptidase family M19)"
"JS1_D03_01260" 13169 "s63E6H2" 0.5551627 "Membrane dipeptidase (Peptidase family M19)"
"JS1_D03_01261" 13170 "s59E13H2" 0.04252149 "Ureidoglycolate lyase"
"JS1_D03_01261" 13170 "s59E25H2" 0.3255208 "Ureidoglycolate lyase"
"JS1_D03_01261" 13170 "s59E5H2" 0.3847542 "Ureidoglycolate lyase"
"JS1_D03_01261" 13170 "s63E6H2" 0.5487271 "Ureidoglycolate lyase"
"JS1_D03_01263" 13172 "s59E13H2" 0.3081026 "Folate transporter FolT"
"JS1_D03_01263" 13172 "s59E25H2" 0 "Folate transporter FolT"
"JS1_D03_01263" 13172 "s59E5H2" 0.004732238 "Folate transporter FolT"
"JS1_D03_01263" 13172 "s63E6H2" 0.3228091 "Folate transporter FolT"
"JS1_D03_01264" 13173 "s59E13H2" 1.654475 "Sorbitol dehydrogenase"
"JS1_D03_01264" 13173 "s59E25H2" 0.7261209 "Sorbitol dehydrogenase"
"JS1_D03_01264" 13173 "s59E5H2" 0.5818053 "Sorbitol dehydrogenase"
"JS1_D03_01264" 13173 "s63E6H2" 1.046847 "Sorbitol dehydrogenase"
"JS1_D03_01265" 13174 "s59E13H2" 0.460117 "1-deoxy-D-xylulose 5-phosphate reductoisomerase"
"JS1_D03_01265" 13174 "s59E25H2" 0 "1-deoxy-D-xylulose 5-phosphate reductoisomerase"
"JS1_D03_01265" 13174 "s59E5H2" 0.2238196 "1-deoxy-D-xylulose 5-phosphate reductoisomerase"
"JS1_D03_01265" 13174 "s63E6H2" 0.2704921 "1-deoxy-D-xylulose 5-phosphate reductoisomerase"
"JS1_D03_01266" 13175 "s59E13H2" 1.087449 "Transposase IS116/IS110/IS902 family protein"
"JS1_D03_01266" 13175 "s59E25H2" 0 "Transposase IS116/IS110/IS902 family protein"
"JS1_D03_01266" 13175 "s59E5H2" 0.5292875 "Transposase IS116/IS110/IS902 family protein"
"JS1_D03_01266" 13175 "s63E6H2" 0.8266876 "Transposase IS116/IS110/IS902 family protein"
"JS1_D03_01267" 13176 "s59E13H2" 1.874377 "hypothetical protein"
"JS1_D03_01267" 13176 "s59E25H2" 0 "hypothetical protein"
"JS1_D03_01267" 13176 "s59E5H2" 0.04365965 "hypothetical protein"
"JS1_D03_01267" 13176 "s63E6H2" 1.986939 "hypothetical protein"
"JS1_D03_01268" 13177 "s59E13H2" 0.004036246 "Leucine carboxyl methyltransferase"
"JS1_D03_01268" 13177 "s59E25H2" 0 "Leucine carboxyl methyltransferase"
"JS1_D03_01268" 13177 "s59E5H2" 0.0006642681 "Leucine carboxyl methyltransferase"
"JS1_D03_01268" 13177 "s63E6H2" 0.04188179 "Leucine carboxyl methyltransferase"
"JS1_D03_01269" 13178 "s59E13H2" 0.07622347 "Phytoene desaturase (lycopene-forming)"
"JS1_D03_01269" 13178 "s59E25H2" 0 "Phytoene desaturase (lycopene-forming)"
"JS1_D03_01269" 13178 "s59E5H2" 0.1531915 "Phytoene desaturase (lycopene-forming)"
"JS1_D03_01269" 13178 "s63E6H2" 1.407886 "Phytoene desaturase (lycopene-forming)"
"JS1_D03_01270" 13179 "s59E13H2" 0.002284435 "DNA-directed RNA polymerase subunit beta'"

"JS1_D03_01270" 13179 "s59E25H2" 0 "DNA-directed RNA polymerase subunit beta'"
"JS1_D03_01270" 13179 "s59E5H2" 0.004552454 "DNA-directed RNA polymerase subunit beta'"
"JS1_D03_01270" 13179 "s63E6H2" 0.004908558 "DNA-directed RNA polymerase subunit beta'"
"JS1_D03_01271" 13180 "s59E13H2" 0 "Type II secretion system protein F"
"JS1_D03_01271" 13180 "s59E25H2" 0 "Type II secretion system protein F"
"JS1_D03_01271" 13180 "s59E5H2" 0 "Type II secretion system protein F"
"JS1_D03_01271" 13180 "s63E6H2" 0.06205941 "Type II secretion system protein F"
"JS1_D03_01276" 13185 "s59E13H2" 0.01578347 "RNA polymerase sigma-B factor"
"JS1_D03_01276" 13185 "s59E25H2" 0 "RNA polymerase sigma-B factor"
"JS1_D03_01276" 13185 "s59E5H2" 0.0245324 "RNA polymerase sigma-B factor"
"JS1_D03_01276" 13185 "s63E6H2" 0.7987016 "RNA polymerase sigma-B factor"
"JS1_D03_01277" 13186 "s59E13H2" 0.002445173 "RNA polymerase sigma factor SigF"
"JS1_D03_01277" 13186 "s59E25H2" 0 "RNA polymerase sigma factor SigF"
"JS1_D03_01277" 13186 "s59E5H2" 0.04211872 "RNA polymerase sigma factor SigF"
"JS1_D03_01277" 13186 "s63E6H2" 2.572442 "RNA polymerase sigma factor SigF"
"JS1_D03_01278" 13187 "s59E13H2" 0.004614106 "Type II secretion system protein F"
"JS1_D03_01278" 13187 "s59E25H2" 0.2054455 "Type II secretion system protein F"
"JS1_D03_01278" 13187 "s59E5H2" 0.006968905 "Type II secretion system protein F"
"JS1_D03_01278" 13187 "s63E6H2" 0.8083399 "Type II secretion system protein F"
"JS1_D03_01279" 13188 "s59E13H2" 0.663293 "hypothetical protein"
"JS1_D03_01279" 13188 "s59E25H2" 0 "hypothetical protein"
"JS1_D03_01279" 13188 "s59E5H2" 0.02463283 "hypothetical protein"
"JS1_D03_01279" 13188 "s63E6H2" 0.7979574 "hypothetical protein"
"JS1_D03_01280" 13189 "s59E13H2" 0.1042495 "Cyclic di-GMP phosphodiesterase response regulator RpfG"
"JS1_D03_01280" 13189 "s59E25H2" 0 "Cyclic di-GMP phosphodiesterase response regulator RpfG"
"JS1_D03_01280" 13189 "s59E5H2" 0.2070742 "Cyclic di-GMP phosphodiesterase response regulator RpfG"
"JS1_D03_01280" 13189 "s63E6H2" 1.013953 "Cyclic di-GMP phosphodiesterase response regulator RpfG"
"JS1_D03_01281" 13190 "s59E13H2" 0.01103524 "Type II secretion system protein E"
"JS1_D03_01281" 13190 "s59E25H2" 0 "Type II secretion system protein E"
"JS1_D03_01281" 13190 "s59E5H2" 0.07498632 "Type II secretion system protein E"
"JS1_D03_01281" 13190 "s63E6H2" 1.252348 "Type II secretion system protein E"
"JS1_D03_01282" 13191 "s59E13H2" 0.2098316 "hypothetical protein"
"JS1_D03_01282" 13191 "s59E25H2" 0 "hypothetical protein"
"JS1_D03_01282" 13191 "s59E5H2" 0.1259773 "hypothetical protein"
"JS1_D03_01282" 13191 "s63E6H2" 0.7393746 "hypothetical protein"
"JS1_D03_01283" 13192 "s59E13H2" 0.003256364 "Mitochondrial PGP phosphatase"
"JS1_D03_01283" 13192 "s59E25H2" 0 "Mitochondrial PGP phosphatase"
"JS1_D03_01283" 13192 "s59E5H2" 0.0620539 "Mitochondrial PGP phosphatase"
"JS1_D03_01283" 13192 "s63E6H2" 0.765 "Mitochondrial PGP phosphatase"
"JS1_D03_01284" 13193 "s59E13H2" 0.005648578 "Uridine kinase"
"JS1_D03_01284" 13193 "s59E25H2" 0 "Uridine kinase"
"JS1_D03_01284" 13193 "s59E5H2" 0.09631676 "Uridine kinase"
"JS1_D03_01284" 13193 "s63E6H2" 0.01480088 "Uridine kinase"
"JS1_D03_01285" 13194 "s59E13H2" 0 "hypothetical protein"
"JS1_D03_01285" 13194 "s59E25H2" 0 "hypothetical protein"
"JS1_D03_01285" 13194 "s59E5H2" 0 "hypothetical protein"
"JS1_D03_01285" 13194 "s63E6H2" 0.07076796 "hypothetical protein"

"JS1_D03_01286" 13195 "s59E13H2" 0 "hypothetical protein"
"JS1_D03_01286" 13195 "s59E25H2" 0 "hypothetical protein"
"JS1_D03_01286" 13195 "s59E5H2" 0 "hypothetical protein"
"JS1_D03_01286" 13195 "s63E6H2" 0.2004392 "hypothetical protein"
"JS1_D03_01287" 13196 "s59E13H2" 0.01455471 "Ribonuclease G"
"JS1_D03_01287" 13196 "s59E25H2" 0 "Ribonuclease G"
"JS1_D03_01287" 13196 "s59E5H2" 0.1377359 "Ribonuclease G"
"JS1_D03_01287" 13196 "s63E6H2" 0.2507069 "Ribonuclease G"
"JS1_D03_01288" 13197 "s59E13H2" 0 "50S ribosomal protein L21"
"JS1_D03_01288" 13197 "s59E25H2" 0 "50S ribosomal protein L21"
"JS1_D03_01288" 13197 "s59E5H2" 0.01413331 "50S ribosomal protein L21"
"JS1_D03_01288" 13197 "s63E6H2" 0.1707462 "50S ribosomal protein L21"
"JS1_D03_01289" 13198 "s59E13H2" 0 "50S ribosomal protein L27"
"JS1_D03_01289" 13198 "s59E25H2" 0 "50S ribosomal protein L27"
"JS1_D03_01289" 13198 "s59E5H2" 0 "50S ribosomal protein L27"
"JS1_D03_01289" 13198 "s63E6H2" 0.1569202 "50S ribosomal protein L27"
"JS1_D03_01290" 13199 "s59E13H2" 0.07035766 "GTPase ObgE"
"JS1_D03_01290" 13199 "s59E25H2" 0 "GTPase ObgE"
"JS1_D03_01290" 13199 "s59E5H2" 0.04895085 "GTPase ObgE"
"JS1_D03_01290" 13199 "s63E6H2" 0.1737522 "GTPase ObgE"
"JS1_D03_01291" 13200 "s59E13H2" 0.06637035 "tol-pal system protein YbgF"
"JS1_D03_01291" 13200 "s59E25H2" 0 "tol-pal system protein YbgF"
"JS1_D03_01291" 13200 "s59E5H2" 0.06996407 "tol-pal system protein YbgF"
"JS1_D03_01291" 13200 "s63E6H2" 0.1714921 "tol-pal system protein YbgF"
"JS1_D03_01292" 13201 "s59E13H2" 0.08324241 "Biopolymer transport protein ExbB"
"JS1_D03_01292" 13201 "s59E25H2" 0 "Biopolymer transport protein ExbB"
"JS1_D03_01292" 13201 "s59E5H2" 0.2040631 "Biopolymer transport protein ExbB"
"JS1_D03_01292" 13201 "s63E6H2" 0.3224612 "Biopolymer transport protein ExbB"
"JS1_D03_01293" 13202 "s59E13H2" 0.01291269 "biopolymer transport protein ExbD"
"JS1_D03_01293" 13202 "s59E25H2" 0 "biopolymer transport protein ExbD"
"JS1_D03_01293" 13202 "s59E5H2" 0.05463078 "biopolymer transport protein ExbD"
"JS1_D03_01293" 13202 "s63E6H2" 0.2474611 "biopolymer transport protein ExbD"
"JS1_D03_01294" 13203 "s59E13H2" 0.4915799 "Putative lipoprotein YerB precursor"
"JS1_D03_01294" 13203 "s59E25H2" 0 "Putative lipoprotein YerB precursor"
"JS1_D03_01294" 13203 "s59E5H2" 0.1706309 "Putative lipoprotein YerB precursor"
"JS1_D03_01294" 13203 "s63E6H2" 0.1361785 "Putative lipoprotein YerB precursor"
"JS1_D03_01295" 13204 "s59E13H2" 0.07998932 "Tyrosine recombinase XerD"
"JS1_D03_01295" 13204 "s59E25H2" 0 "Tyrosine recombinase XerD"
"JS1_D03_01295" 13204 "s59E5H2" 0.07893087 "Tyrosine recombinase XerD"
"JS1_D03_01295" 13204 "s63E6H2" 0.2189937 "Tyrosine recombinase XerD"
"JS1_D03_01296" 13205 "s59E13H2" 0.1153553 "Purine nucleoside phosphorylase 1"
"JS1_D03_01296" 13205 "s59E25H2" 0 "Purine nucleoside phosphorylase 1"
"JS1_D03_01296" 13205 "s59E5H2" 0.1667185 "Purine nucleoside phosphorylase 1"
"JS1_D03_01296" 13205 "s63E6H2" 0.3973198 "Purine nucleoside phosphorylase 1"
"JS1_D03_01297" 13206 "s59E13H2" 0.1475677 "Putative small multi-drug export protein"
"JS1_D03_01297" 13206 "s59E25H2" 0 "Putative small multi-drug export protein"
"JS1_D03_01297" 13206 "s59E5H2" 0.1249963 "Putative small multi-drug export protein"

"JS1_D03_01297" 13206 "s63E6H2" 0.4145837 "Putative small multi-drug export protein"
"JS1_D03_01298" 13207 "s59E13H2" 0.2307876 "Methylthioribose-1-phosphate isomerase"
"JS1_D03_01298" 13207 "s59E25H2" 0 "Methylthioribose-1-phosphate isomerase"
"JS1_D03_01298" 13207 "s59E5H2" 0.1437503 "Methylthioribose-1-phosphate isomerase"
"JS1_D03_01298" 13207 "s63E6H2" 0.335421 "Methylthioribose-1-phosphate isomerase"
"JS1_D03_01299" 13208 "s59E13H2" 0.1159155 "UDP-N-acetylglucosamine 1-carboxyvinyltransferase 1"
"JS1_D03_01299" 13208 "s59E25H2" 0 "UDP-N-acetylglucosamine 1-carboxyvinyltransferase 1"
"JS1_D03_01299" 13208 "s59E5H2" 0.1459826 "UDP-N-acetylglucosamine 1-carboxyvinyltransferase 1"
"JS1_D03_01299" 13208 "s63E6H2" 0.5492828 "UDP-N-acetylglucosamine 1-carboxyvinyltransferase 1"
"JS1_D03_01300" 13209 "s59E13H2" 0.584858 "Rod shape-determining protein MreB"
"JS1_D03_01300" 13209 "s59E25H2" 0 "Rod shape-determining protein MreB"
"JS1_D03_01300" 13209 "s59E5H2" 0.1694182 "Rod shape-determining protein MreB"
"JS1_D03_01300" 13209 "s63E6H2" 0.7076843 "Rod shape-determining protein MreB"
"JS1_D03_01301" 13210 "s59E13H2" 0.01141401 "SpoIVB peptidase S55"
"JS1_D03_01301" 13210 "s59E25H2" 0 "SpoIVB peptidase S55"
"JS1_D03_01301" 13210 "s59E5H2" 0.01304912 "SpoIVB peptidase S55"
"JS1_D03_01301" 13210 "s63E6H2" 0.6653234 "SpoIVB peptidase S55"
"JS1_D03_01302" 13211 "s59E13H2" 0.4761339 "Putative 2-C-methyl-D-erythritol 4-phosphate cytidylyltransferase 2"
"JS1_D03_01302" 13211 "s59E25H2" 0 "Putative 2-C-methyl-D-erythritol 4-phosphate cytidylyltransferase 2"
"JS1_D03_01302" 13211 "s59E5H2" 0.07588373 "Putative 2-C-methyl-D-erythritol 4-phosphate cytidylyltransferase 2"
"JS1_D03_01302" 13211 "s63E6H2" 0.4331408 "Putative 2-C-methyl-D-erythritol 4-phosphate cytidylyltransferase 2"
"JS1_D03_01303" 13212 "s59E13H2" 0.2773887 "4-diphosphocytidyl-2-C-methyl-D-erythritol kinase"
"JS1_D03_01303" 13212 "s59E25H2" 0 "4-diphosphocytidyl-2-C-methyl-D-erythritol kinase"
"JS1_D03_01303" 13212 "s59E5H2" 0.1123673 "4-diphosphocytidyl-2-C-methyl-D-erythritol kinase"
"JS1_D03_01303" 13212 "s63E6H2" 0.4994228 "4-diphosphocytidyl-2-C-methyl-D-erythritol kinase"
"JS1_D03_01304" 13213 "s59E13H2" 0.1605048 "2-C-methyl-D-erythritol 2,4-cyclodiphosphate synthase"
"JS1_D03_01304" 13213 "s59E25H2" 0 "2-C-methyl-D-erythritol 2,4-cyclodiphosphate synthase"
"JS1_D03_01304" 13213 "s59E5H2" 0.04717882 "2-C-methyl-D-erythritol 2,4-cyclodiphosphate synthase"
"JS1_D03_01304" 13213 "s63E6H2" 0.3726331 "2-C-methyl-D-erythritol 2,4-cyclodiphosphate synthase"
"JS1_D03_01305" 13214 "s59E13H2" 0.002988731 "50S ribosomal protein L7/L12"
"JS1_D03_01305" 13214 "s59E25H2" 0 "50S ribosomal protein L7/L12"
"JS1_D03_01305" 13214 "s59E5H2" 0 "50S ribosomal protein L7/L12"
"JS1_D03_01305" 13214 "s63E6H2" 0.00661756 "50S ribosomal protein L7/L12"
"JS1_D03_01307" 13216 "s59E13H2" 0 "50S ribosomal protein L10"
"JS1_D03_01307" 13216 "s59E25H2" 0 "50S ribosomal protein L10"
"JS1_D03_01307" 13216 "s59E5H2" 0 "50S ribosomal protein L10"
"JS1_D03_01307" 13216 "s63E6H2" 0 "50S ribosomal protein L10"
"JS1_D03_01309" 13218 "s59E13H2" 0 "50S ribosomal protein L1"
"JS1_D03_01309" 13218 "s59E25H2" 0 "50S ribosomal protein L1"
"JS1_D03_01309" 13218 "s59E5H2" 0.02653232 "50S ribosomal protein L1"
"JS1_D03_01309" 13218 "s63E6H2" 0.05938934 "50S ribosomal protein L1"
"JS1_D03_01310" 13219 "s59E13H2" 0.01280125 "DNA utilization protein GntX"
"JS1_D03_01310" 13219 "s59E25H2" 0 "DNA utilization protein GntX"
"JS1_D03_01310" 13219 "s59E5H2" 0.1279692 "DNA utilization protein GntX"
"JS1_D03_01310" 13219 "s63E6H2" 0.419277 "DNA utilization protein GntX"
"JS1_D03_01311" 13220 "s59E13H2" 0 "hypothetical protein"
"JS1_D03_01311" 13220 "s59E25H2" 0 "hypothetical protein"

"JS1_D03_01311" 13220 "s59E5H2" 0.04000119 "hypothetical protein"
"JS1_D03_01311" 13220 "s63E6H2" 0.1618446 "hypothetical protein"
"JS1_D03_01314" 13223 "s59E13H2" 0.268692 "Molybdopterin-guanine dinucleotide biosynthesis adapter protein"
"JS1_D03_01314" 13223 "s59E25H2" 0 "Molybdopterin-guanine dinucleotide biosynthesis adapter protein"
"JS1_D03_01314" 13223 "s59E5H2" 0.4623568 "Molybdopterin-guanine dinucleotide biosynthesis adapter protein"
"JS1_D03_01314" 13223 "s63E6H2" 0.287753 "Molybdopterin-guanine dinucleotide biosynthesis adapter protein"
"JS1_D03_01315" 13224 "s59E13H2" 0.03497044 "hypothetical protein"
"JS1_D03_01315" 13224 "s59E25H2" 0 "hypothetical protein"
"JS1_D03_01315" 13224 "s59E5H2" 0.2016372 "hypothetical protein"
"JS1_D03_01315" 13224 "s63E6H2" 0.2143733 "hypothetical protein"
"JS1_D03_01316" 13225 "s59E13H2" 0 "Cyclic di-GMP phosphodiesterase response regulator RpfG"
"JS1_D03_01316" 13225 "s59E25H2" 0 "Cyclic di-GMP phosphodiesterase response regulator RpfG"
"JS1_D03_01316" 13225 "s59E5H2" 0.03906134 "Cyclic di-GMP phosphodiesterase response regulator RpfG"
"JS1_D03_01316" 13225 "s63E6H2" 0.4122907 "Cyclic di-GMP phosphodiesterase response regulator RpfG"
"JS1_D03_01318" 13227 "s59E13H2" 0.1666267 "Zinc ribbon domain protein"
"JS1_D03_01318" 13227 "s59E25H2" 0 "Zinc ribbon domain protein"
"JS1_D03_01318" 13227 "s59E5H2" 0.06286182 "Zinc ribbon domain protein"
"JS1_D03_01318" 13227 "s63E6H2" 0.5362595 "Zinc ribbon domain protein"
"JS1_D03_01319" 13228 "s59E13H2" 2.611149 "NTE family protein RssA"
"JS1_D03_01319" 13228 "s59E25H2" 0 "NTE family protein RssA"
"JS1_D03_01319" 13228 "s59E5H2" 0.1615984 "NTE family protein RssA"
"JS1_D03_01319" 13228 "s63E6H2" 1.195372 "NTE family protein RssA"
"JS1_D03_01320" 13229 "s59E13H2" 1.052173 "Isochorismatase"
"JS1_D03_01320" 13229 "s59E25H2" 0 "Isochorismatase"
"JS1_D03_01320" 13229 "s59E5H2" 0.2253804 "Isochorismatase"
"JS1_D03_01320" 13229 "s63E6H2" 0.9889676 "Isochorismatase"
"JS1_D03_01321" 13230 "s59E13H2" 9.709123 "Fluoroacetyl-CoA thioesterase"
"JS1_D03_01321" 13230 "s59E25H2" 0 "Fluoroacetyl-CoA thioesterase"
"JS1_D03_01321" 13230 "s59E5H2" 0.6522404 "Fluoroacetyl-CoA thioesterase"
"JS1_D03_01321" 13230 "s63E6H2" 1.286331 "Fluoroacetyl-CoA thioesterase"
"JS1_D03_01322" 13231 "s59E13H2" 0.32207 "D-arabitol-phosphate dehydrogenase"
"JS1_D03_01322" 13231 "s59E25H2" 0 "D-arabitol-phosphate dehydrogenase"
"JS1_D03_01322" 13231 "s59E5H2" 0.2297142 "D-arabitol-phosphate dehydrogenase"
"JS1_D03_01322" 13231 "s63E6H2" 0.1786256 "D-arabitol-phosphate dehydrogenase"
"JS1_D03_01323" 13232 "s59E13H2" 0.06491886 "hypothetical protein"
"JS1_D03_01323" 13232 "s59E25H2" 0 "hypothetical protein"
"JS1_D03_01323" 13232 "s59E5H2" 0.08929741 "hypothetical protein"
"JS1_D03_01323" 13232 "s63E6H2" 0.2584529 "hypothetical protein"
"JS1_D03_01325" 13234 "s59E13H2" 0.02159002 "Sialic acid TRAP transporter permease protein SiaT"
"JS1_D03_01325" 13234 "s59E25H2" 0 "Sialic acid TRAP transporter permease protein SiaT"
"JS1_D03_01325" 13234 "s59E5H2" 0.2584199 "Sialic acid TRAP transporter permease protein SiaT"
"JS1_D03_01325" 13234 "s63E6H2" 0.03313969 "Sialic acid TRAP transporter permease protein SiaT"
"JS1_D03_01326" 13235 "s59E13H2" 0.01743424 "HTH-type transcriptional repressor YtrA"
"JS1_D03_01326" 13235 "s59E25H2" 0 "HTH-type transcriptional repressor YtrA"
"JS1_D03_01326" 13235 "s59E5H2" 0.2082047 "HTH-type transcriptional repressor YtrA"
"JS1_D03_01326" 13235 "s63E6H2" 0.147899 "HTH-type transcriptional repressor YtrA"
"JS1_D03_01327" 13236 "s59E13H2" 0.005209539 "Ferredoxin"

"JS1_D03_01327" 13236 "s59E25H2" 0 "Ferredoxin"
"JS1_D03_01327" 13236 "s59E5H2" 0.2437764 "Ferredoxin"
"JS1_D03_01327" 13236 "s63E6H2" 0.1299667 "Ferredoxin"
"JS1_D03_01328" 13237 "s59E13H2" 0.06815879 "2-oxoglutarate synthase subunit KorA"
"JS1_D03_01328" 13237 "s59E25H2" 0 "2-oxoglutarate synthase subunit KorA"
"JS1_D03_01328" 13237 "s59E5H2" 0.1949338 "2-oxoglutarate synthase subunit KorA"
"JS1_D03_01328" 13237 "s63E6H2" 0.2545775 "2-oxoglutarate synthase subunit KorA"
"JS1_D03_01329" 13238 "s59E13H2" 0.03680248 "2-oxoglutarate synthase subunit KorB"
"JS1_D03_01329" 13238 "s59E25H2" 0 "2-oxoglutarate synthase subunit KorB"
"JS1_D03_01329" 13238 "s59E5H2" 0.1733951 "2-oxoglutarate synthase subunit KorB"
"JS1_D03_01329" 13238 "s63E6H2" 0.1004111 "2-oxoglutarate synthase subunit KorB"
"JS1_D03_01330" 13239 "s59E13H2" 0.04595704 "2-oxoglutarate synthase subunit KorC"
"JS1_D03_01330" 13239 "s59E25H2" 0 "2-oxoglutarate synthase subunit KorC"
"JS1_D03_01330" 13239 "s59E5H2" 0.2761837 "2-oxoglutarate synthase subunit KorC"
"JS1_D03_01330" 13239 "s63E6H2" 0.1243899 "2-oxoglutarate synthase subunit KorC"
"JS1_D03_01331" 13240 "s59E13H2" 0.06501938 "Acetate kinase"
"JS1_D03_01331" 13240 "s59E25H2" 0 "Acetate kinase"
"JS1_D03_01331" 13240 "s59E5H2" 0.2232159 "Acetate kinase"
"JS1_D03_01331" 13240 "s63E6H2" 0.06168312 "Acetate kinase"
"JS1_D03_01332" 13241 "s59E13H2" 0.06262465 "Sulfide dehydrogenase subunit beta precursor"
"JS1_D03_01332" 13241 "s59E25H2" 0 "Sulfide dehydrogenase subunit beta precursor"
"JS1_D03_01332" 13241 "s59E5H2" 0.2909987 "Sulfide dehydrogenase subunit beta precursor"
"JS1_D03_01332" 13241 "s63E6H2" 0.130067 "Sulfide dehydrogenase subunit beta precursor"
"JS1_D03_01333" 13242 "s59E13H2" 0.1023985 "Sulfide dehydrogenase subunit alpha precursor"
"JS1_D03_01333" 13242 "s59E25H2" 0 "Sulfide dehydrogenase subunit alpha precursor"
"JS1_D03_01333" 13242 "s59E5H2" 0.1706189 "Sulfide dehydrogenase subunit alpha precursor"
"JS1_D03_01333" 13242 "s63E6H2" 0.2298217 "Sulfide dehydrogenase subunit alpha precursor"
"JS1_D03_01334" 13243 "s59E13H2" 0.04096604 "Glutamate dehydrogenase"
"JS1_D03_01334" 13243 "s59E25H2" 0 "Glutamate dehydrogenase"
"JS1_D03_01334" 13243 "s59E5H2" 0.01838572 "Glutamate dehydrogenase"
"JS1_D03_01334" 13243 "s63E6H2" 0.3668816 "Glutamate dehydrogenase"
"JS1_D03_01335" 13244 "s59E13H2" 0.1110834 "hypothetical protein"
"JS1_D03_01335" 13244 "s59E25H2" 0 "hypothetical protein"
"JS1_D03_01335" 13244 "s59E5H2" 0.2081306 "hypothetical protein"
"JS1_D03_01335" 13244 "s63E6H2" 0.09741123 "hypothetical protein"
"JS1_D03_01336" 13245 "s59E13H2" 0.128252 "Methylmalonyl-CoA mutase"
"JS1_D03_01336" 13245 "s59E25H2" 0 "Methylmalonyl-CoA mutase"
"JS1_D03_01336" 13245 "s59E5H2" 0.07230626 "Methylmalonyl-CoA mutase"
"JS1_D03_01336" 13245 "s63E6H2" 0.1861312 "Methylmalonyl-CoA mutase"
"JS1_D03_01337" 13246 "s59E13H2" 0.1222147 "Methylmalonyl-CoA mutase"
"JS1_D03_01337" 13246 "s59E25H2" 0 "Methylmalonyl-CoA mutase"
"JS1_D03_01337" 13246 "s59E5H2" 0.0740172 "Methylmalonyl-CoA mutase"
"JS1_D03_01337" 13246 "s63E6H2" 0.5071141 "Methylmalonyl-CoA mutase"
"JS1_D03_01338" 13247 "s59E13H2" 0.06877586 "putative GTPase/MT1543"
"JS1_D03_01338" 13247 "s59E25H2" 0 "putative GTPase/MT1543"
"JS1_D03_01338" 13247 "s59E5H2" 0.04770929 "putative GTPase/MT1543"
"JS1_D03_01338" 13247 "s63E6H2" 0.5553996 "putative GTPase/MT1543"

"JS1_D03_01339" 13248 "s59E13H2" 0.04516842 "putative lyase"
"JS1_D03_01339" 13248 "s59E25H2" 0 "putative lyase"
"JS1_D03_01339" 13248 "s59E5H2" 0.1033148 "putative lyase"
"JS1_D03_01339" 13248 "s63E6H2" 0.6872749 "putative lyase"
"JS1_D03_01340" 13249 "s59E13H2" 0.08566503 "Methylmalonyl-CoA carboxyltransferase 12S subunit"
"JS1_D03_01340" 13249 "s59E25H2" 0 "Methylmalonyl-CoA carboxyltransferase 12S subunit"
"JS1_D03_01340" 13249 "s59E5H2" 0.1368158 "Methylmalonyl-CoA carboxyltransferase 12S subunit"
"JS1_D03_01340" 13249 "s63E6H2" 0.8673315 "Methylmalonyl-CoA carboxyltransferase 12S subunit"
"JS1_D03_01341" 13250 "s59E13H2" 0 "Oxaloacetate decarboxylase, gamma chain"
"JS1_D03_01341" 13250 "s59E25H2" 0 "Oxaloacetate decarboxylase, gamma chain"
"JS1_D03_01341" 13250 "s59E5H2" 0 "Oxaloacetate decarboxylase, gamma chain"
"JS1_D03_01341" 13250 "s63E6H2" 0.7650105 "Oxaloacetate decarboxylase, gamma chain"
"JS1_D03_01342" 13251 "s59E13H2" 0.002116919 "2-oxoglutarate carboxylase large subunit"
"JS1_D03_01342" 13251 "s59E25H2" 0 "2-oxoglutarate carboxylase large subunit"
"JS1_D03_01342" 13251 "s59E5H2" 0.003955036 "2-oxoglutarate carboxylase large subunit"
"JS1_D03_01342" 13251 "s63E6H2" 0.8126131 "2-oxoglutarate carboxylase large subunit"
"JS1_D03_01343" 13252 "s59E13H2" 0.08326199 "Glutaconyl-CoA decarboxylase subunit beta"
"JS1_D03_01343" 13252 "s59E25H2" 0 "Glutaconyl-CoA decarboxylase subunit beta"
"JS1_D03_01343" 13252 "s59E5H2" 0.1675502 "Glutaconyl-CoA decarboxylase subunit beta"
"JS1_D03_01343" 13252 "s63E6H2" 0.8542679 "Glutaconyl-CoA decarboxylase subunit beta"
"JS1_D03_01344" 13253 "s59E13H2" 0.03345111 "hypothetical protein"
"JS1_D03_01344" 13253 "s59E25H2" 0 "hypothetical protein"
"JS1_D03_01344" 13253 "s59E5H2" 0.07022668 "hypothetical protein"
"JS1_D03_01344" 13253 "s63E6H2" 0.4118092 "hypothetical protein"
"JS1_D03_01345" 13254 "s59E13H2" 0.02339441 "colanic acid biosynthesis protein"
"JS1_D03_01345" 13254 "s59E25H2" 0 "colanic acid biosynthesis protein"
"JS1_D03_01345" 13254 "s59E5H2" 0.1620887 "colanic acid biosynthesis protein"
"JS1_D03_01345" 13254 "s63E6H2" 0.09809641 "colanic acid biosynthesis protein"
"JS1_D03_01346" 13255 "s59E13H2" 0.03191329 "Cell division ATP-binding protein FtsE"
"JS1_D03_01346" 13255 "s59E25H2" 0 "Cell division ATP-binding protein FtsE"
"JS1_D03_01346" 13255 "s59E5H2" 0.2991605 "Cell division ATP-binding protein FtsE"
"JS1_D03_01346" 13255 "s63E6H2" 0.219737 "Cell division ATP-binding protein FtsE"
"JS1_D03_01347" 13256 "s59E13H2" 0.005438723 "Cell division protein FtsX"
"JS1_D03_01347" 13256 "s59E25H2" 0 "Cell division protein FtsX"
"JS1_D03_01347" 13256 "s59E5H2" 0.03322947 "Cell division protein FtsX"
"JS1_D03_01347" 13256 "s63E6H2" 0.2584969 "Cell division protein FtsX"
"JS1_D03_01348" 13257 "s59E13H2" 0 "Murein hydrolase activator EnvC precursor"
"JS1_D03_01348" 13257 "s59E25H2" 0 "Murein hydrolase activator EnvC precursor"
"JS1_D03_01348" 13257 "s59E5H2" 0.0134097 "Murein hydrolase activator EnvC precursor"
"JS1_D03_01348" 13257 "s63E6H2" 0.1189339 "Murein hydrolase activator EnvC precursor"
"JS1_D03_01349" 13258 "s59E13H2" 0.02470942 "putative CtpA-like serine protease"
"JS1_D03_01349" 13258 "s59E25H2" 0 "putative CtpA-like serine protease"
"JS1_D03_01349" 13258 "s59E5H2" 0.08467956 "putative CtpA-like serine protease"
"JS1_D03_01349" 13258 "s63E6H2" 0.0624953 "putative CtpA-like serine protease"
"JS1_D03_01350" 13259 "s59E13H2" 0 "Digeranylgeranylglycerophospholipid reductase"
"JS1_D03_01350" 13259 "s59E25H2" 0 "Digeranylgeranylglycerophospholipid reductase"
"JS1_D03_01350" 13259 "s59E5H2" 0.03422477 "Digeranylgeranylglycerophospholipid reductase"

"JS1_D03_01350" 13259 "s63E6H2" 0.4823079 "Digeranylgeranylglycerophospholipid reductase"
"JS1_D03_01351" 13260 "s59E13H2" 0.008889873 "Bifunctional protein GlmU"
"JS1_D03_01351" 13260 "s59E25H2" 0 "Bifunctional protein GlmU"
"JS1_D03_01351" 13260 "s59E5H2" 0.0837758 "Bifunctional protein GlmU"
"JS1_D03_01351" 13260 "s63E6H2" 1.756863 "Bifunctional protein GlmU"
"JS1_D03_01352" 13261 "s59E13H2" 0.01091331 "translocation protein TolB"
"JS1_D03_01352" 13261 "s59E25H2" 0 "translocation protein TolB"
"JS1_D03_01352" 13261 "s59E5H2" 0.04273584 "translocation protein TolB"
"JS1_D03_01352" 13261 "s63E6H2" 2.02001 "translocation protein TolB"
"JS1_D03_01353" 13262 "s59E13H2" 0.003764583 "Transposase IS200 like protein"
"JS1_D03_01353" 13262 "s59E25H2" 0 "Transposase IS200 like protein"
"JS1_D03_01353" 13262 "s59E5H2" 0.01124089 "Transposase IS200 like protein"
"JS1_D03_01353" 13262 "s63E6H2" 0.009990935 "Transposase IS200 like protein"
"JS1_D03_01354" 13263 "s59E13H2" 0.006037968 "Amidohydrolase"
"JS1_D03_01354" 13263 "s59E25H2" 0 "Amidohydrolase"
"JS1_D03_01354" 13263 "s59E5H2" 0.1641278 "Amidohydrolase"
"JS1_D03_01354" 13263 "s63E6H2" 12.63 "Amidohydrolase"
"JS1_D03_01355" 13264 "s59E13H2" 0.001332566 "hypothetical protein"
"JS1_D03_01355" 13264 "s59E25H2" 0 "hypothetical protein"
"JS1_D03_01355" 13264 "s59E5H2" 0.047145 "hypothetical protein"
"JS1_D03_01355" 13264 "s63E6H2" 0.4835079 "hypothetical protein"
"JS1_D03_01356" 13265 "s59E13H2" 0.01637692 "Bifunctional phosphoglucose/phosphomannose isomerase"
"JS1_D03_01356" 13265 "s59E25H2" 0 "Bifunctional phosphoglucose/phosphomannose isomerase"
"JS1_D03_01356" 13265 "s59E5H2" 0.07147315 "Bifunctional phosphoglucose/phosphomannose isomerase"
"JS1_D03_01356" 13265 "s63E6H2" 0.04424372 "Bifunctional phosphoglucose/phosphomannose isomerase"
"JS1_D03_01357" 13266 "s59E13H2" 0 "Elongation factor P"
"JS1_D03_01357" 13266 "s59E25H2" 0 "Elongation factor P"
"JS1_D03_01357" 13266 "s59E5H2" 0 "Elongation factor P"
"JS1_D03_01357" 13266 "s63E6H2" 0.006693359 "Elongation factor P"
"JS1_D03_01358" 13267 "s59E13H2" 0 "hypothetical protein"
"JS1_D03_01358" 13267 "s59E25H2" 0 "hypothetical protein"
"JS1_D03_01358" 13267 "s59E5H2" 0 "hypothetical protein"
"JS1_D03_01358" 13267 "s63E6H2" 0.02508777 "hypothetical protein"
"JS1_D03_01359" 13268 "s59E13H2" 0 "hypothetical protein"
"JS1_D03_01359" 13268 "s59E25H2" 0 "hypothetical protein"
"JS1_D03_01359" 13268 "s59E5H2" 0.02782439 "hypothetical protein"
"JS1_D03_01359" 13268 "s63E6H2" 0.01037647 "hypothetical protein"
"JS1_D03_01360" 13269 "s59E13H2" 0.008731248 "Exodeoxyribonuclease 7 large subunit"
"JS1_D03_01360" 13269 "s59E25H2" 0 "Exodeoxyribonuclease 7 large subunit"
"JS1_D03_01360" 13269 "s59E5H2" 0.006563775 "Exodeoxyribonuclease 7 large subunit"
"JS1_D03_01360" 13269 "s63E6H2" 0.03029265 "Exodeoxyribonuclease 7 large subunit"
"JS1_D03_01361" 13270 "s59E13H2" 0.004894676 "Exodeoxyribonuclease 7 small subunit"
"JS1_D03_01361" 13270 "s59E25H2" 0 "Exodeoxyribonuclease 7 small subunit"
"JS1_D03_01361" 13270 "s59E5H2" 0 "Exodeoxyribonuclease 7 small subunit"
"JS1_D03_01361" 13270 "s63E6H2" 0.0644855 "Exodeoxyribonuclease 7 small subunit"
"JS1_D03_01362" 13271 "s59E13H2" 0 "Farnesyl diphosphate synthase"
"JS1_D03_01362" 13271 "s59E25H2" 0 "Farnesyl diphosphate synthase"

"JS1_D03_01362" 13271 "s59E5H2" 0.009339163 "Farnesyl diphosphate synthase"
"JS1_D03_01362" 13271 "s63E6H2" 0.03202042 "Farnesyl diphosphate synthase"
"JS1_D03_01363" 13272 "s59E13H2" 0 "Ribonuclease PH"
"JS1_D03_01363" 13272 "s59E25H2" 0 "Ribonuclease PH"
"JS1_D03_01363" 13272 "s59E5H2" 0.01114277 "Ribonuclease PH"
"JS1_D03_01363" 13272 "s63E6H2" 0.1171059 "Ribonuclease PH"
"JS1_D03_01364" 13273 "s59E13H2" 0 "Non-canonical purine NTP pyrophosphatase"
"JS1_D03_01364" 13273 "s59E25H2" 0 "Non-canonical purine NTP pyrophosphatase"
"JS1_D03_01364" 13273 "s59E5H2" 0.007177502 "Non-canonical purine NTP pyrophosphatase"
"JS1_D03_01364" 13273 "s63E6H2" 0.03734866 "Non-canonical purine NTP pyrophosphatase"
"JS1_D03_01372" 13281 "s59E13H2" 0.04619513 "Trigger factor"
"JS1_D03_01372" 13281 "s59E25H2" 0 "Trigger factor"
"JS1_D03_01372" 13281 "s59E5H2" 0.01881453 "Trigger factor"
"JS1_D03_01372" 13281 "s63E6H2" 0.06418577 "Trigger factor"
"JS1_D03_01373" 13282 "s59E13H2" 0.1091108 "ATP-dependent Clp protease proteolytic subunit"
"JS1_D03_01373" 13282 "s59E25H2" 0 "ATP-dependent Clp protease proteolytic subunit"
"JS1_D03_01373" 13282 "s59E5H2" 0.03318381 "ATP-dependent Clp protease proteolytic subunit"
"JS1_D03_01373" 13282 "s63E6H2" 0.06776829 "ATP-dependent Clp protease proteolytic subunit"
"JS1_D03_01374" 13283 "s59E13H2" 0.01648089 "hypothetical protein"
"JS1_D03_01374" 13283 "s59E25H2" 0 "hypothetical protein"
"JS1_D03_01374" 13283 "s59E5H2" 0 "hypothetical protein"
"JS1_D03_01374" 13283 "s63E6H2" 0.002093978 "hypothetical protein"
"JS1_D03_01375" 13284 "s59E13H2" 0.02081756 "ATP-dependent Clp protease ATP-binding subunit ClpX"
"JS1_D03_01375" 13284 "s59E25H2" 0 "ATP-dependent Clp protease ATP-binding subunit ClpX"
"JS1_D03_01375" 13284 "s59E5H2" 0.06287144 "ATP-dependent Clp protease ATP-binding subunit ClpX"
"JS1_D03_01375" 13284 "s63E6H2" 0.02274476 "ATP-dependent Clp protease ATP-binding subunit ClpX"
"JS1_D03_01376" 13285 "s59E13H2" 0.0007626746 "DNA-directed RNA polymerase subunit beta'"
"JS1_D03_01376" 13285 "s59E25H2" 0 "DNA-directed RNA polymerase subunit beta'"
"JS1_D03_01376" 13285 "s59E5H2" 0 "DNA-directed RNA polymerase subunit beta'"
"JS1_D03_01376" 13285 "s63E6H2" 0.001858449 "DNA-directed RNA polymerase subunit beta'"
"JS1_D03_01377" 13286 "s59E13H2" 0.09374847 "Trehalose/maltose import ATP-binding protein MalK"
"JS1_D03_01377" 13286 "s59E25H2" 0 "Trehalose/maltose import ATP-binding protein MalK"
"JS1_D03_01377" 13286 "s59E5H2" 0.4080839 "Trehalose/maltose import ATP-binding protein MalK"
"JS1_D03_01377" 13286 "s63E6H2" 1.033092 "Trehalose/maltose import ATP-binding protein MalK"
"JS1_D03_01378" 13287 "s59E13H2" 0.05637103 "ureidoglycolate hydrolase"
"JS1_D03_01378" 13287 "s59E25H2" 0 "ureidoglycolate hydrolase"
"JS1_D03_01378" 13287 "s59E5H2" 0.3980013 "ureidoglycolate hydrolase"
"JS1_D03_01378" 13287 "s63E6H2" 0.6701379 "ureidoglycolate hydrolase"
"JS1_D03_01379" 13288 "s59E13H2" 0.05675867 "putative D-lyxose ketol-isomerase"
"JS1_D03_01379" 13288 "s59E25H2" 0 "putative D-lyxose ketol-isomerase"
"JS1_D03_01379" 13288 "s59E5H2" 0.3528241 "putative D-lyxose ketol-isomerase"
"JS1_D03_01379" 13288 "s63E6H2" 1.417776 "putative D-lyxose ketol-isomerase"
"JS1_D03_01380" 13289 "s59E13H2" 0.276195 "putative sugar kinase YdjH"
"JS1_D03_01380" 13289 "s59E25H2" 0 "putative sugar kinase YdjH"
"JS1_D03_01380" 13289 "s59E5H2" 0.2852111 "putative sugar kinase YdjH"
"JS1_D03_01380" 13289 "s63E6H2" 1.452671 "putative sugar kinase YdjH"
"JS1_D03_01381" 13290 "s59E13H2" 0.5889475 "Mannitol-1-phosphate 5-dehydrogenase"

"JS1_D03_01381" 13290 "s59E25H2" 0 "Mannitol-1-phosphate 5-dehydrogenase"
"JS1_D03_01381" 13290 "s59E5H2" 0.4821399 "Mannitol-1-phosphate 5-dehydrogenase"
"JS1_D03_01381" 13290 "s63E6H2" 1.277936 "Mannitol-1-phosphate 5-dehydrogenase"
"JS1_D03_01382" 13291 "s59E13H2" 0.6845127 "Xylose import ATP-binding protein XylG"
"JS1_D03_01382" 13291 "s59E25H2" 0 "Xylose import ATP-binding protein XylG"
"JS1_D03_01382" 13291 "s59E5H2" 0.358682 "Xylose import ATP-binding protein XylG"
"JS1_D03_01382" 13291 "s63E6H2" 0.8554351 "Xylose import ATP-binding protein XylG"
"JS1_D03_01383" 13292 "s59E13H2" 0.8392051 "Ribose transport system permease protein RbsC"
"JS1_D03_01383" 13292 "s59E25H2" 0 "Ribose transport system permease protein RbsC"
"JS1_D03_01383" 13292 "s59E5H2" 1.654452 "Ribose transport system permease protein RbsC"
"JS1_D03_01383" 13292 "s63E6H2" 0.8480046 "Ribose transport system permease protein RbsC"
"JS1_D03_01384" 13293 "s59E13H2" 0.8923943 "Ribose transport system permease protein RbsC"
"JS1_D03_01384" 13293 "s59E25H2" 0 "Ribose transport system permease protein RbsC"
"JS1_D03_01384" 13293 "s59E5H2" 0.5916031 "Ribose transport system permease protein RbsC"
"JS1_D03_01384" 13293 "s63E6H2" 0.6998692 "Ribose transport system permease protein RbsC"
"JS1_D03_01385" 13294 "s59E13H2" 0.9467612 "D-ribose-binding periplasmic protein precursor"
"JS1_D03_01385" 13294 "s59E25H2" 0 "D-ribose-binding periplasmic protein precursor"
"JS1_D03_01385" 13294 "s59E5H2" 1.000596 "D-ribose-binding periplasmic protein precursor"
"JS1_D03_01385" 13294 "s63E6H2" 0.8641224 "D-ribose-binding periplasmic protein precursor"
"JS1_D03_01386" 13295 "s59E13H2" 0 "putative nicotinate-nucleotide adenylyltransferase"
"JS1_D03_01386" 13295 "s59E25H2" 0 "putative nicotinate-nucleotide adenylyltransferase"
"JS1_D03_01386" 13295 "s59E5H2" 0 "putative nicotinate-nucleotide adenylyltransferase"
"JS1_D03_01386" 13295 "s63E6H2" 0.2940915 "putative nicotinate-nucleotide adenylyltransferase"
"JS1_D03_01387" 13296 "s59E13H2" 0 "putative nicotinate-nucleotide adenylyltransferase"
"JS1_D03_01387" 13296 "s59E25H2" 0 "putative nicotinate-nucleotide adenylyltransferase"
"JS1_D03_01387" 13296 "s59E5H2" 0.00404586 "putative nicotinate-nucleotide adenylyltransferase"
"JS1_D03_01387" 13296 "s63E6H2" 0.4047979 "putative nicotinate-nucleotide adenylyltransferase"
"JS1_D03_01391" 13300 "s59E13H2" 0.002622772 "Elongation factor Tu"
"JS1_D03_01391" 13300 "s59E25H2" 0 "Elongation factor Tu"
"JS1_D03_01391" 13300 "s59E5H2" 0 "Elongation factor Tu"
"JS1_D03_01391" 13300 "s63E6H2" 0.03626484 "Elongation factor Tu"
"JS1_D03_01392" 13301 "s59E13H2" 0.06361328 "30S ribosomal protein S10"
"JS1_D03_01392" 13301 "s59E25H2" 0 "30S ribosomal protein S10"
"JS1_D03_01392" 13301 "s59E5H2" 0 "30S ribosomal protein S10"
"JS1_D03_01392" 13301 "s63E6H2" 0.01623111 "30S ribosomal protein S10"
"JS1_D03_01394" 13303 "s59E13H2" 0 "50S ribosomal protein L4"
"JS1_D03_01394" 13303 "s59E25H2" 0 "50S ribosomal protein L4"
"JS1_D03_01394" 13303 "s59E5H2" 0.01379556 "50S ribosomal protein L4"
"JS1_D03_01394" 13303 "s63E6H2" 0.1579446 "50S ribosomal protein L4"
"JS1_D03_01395" 13304 "s59E13H2" 0.00229438 "50S ribosomal protein L23"
"JS1_D03_01395" 13304 "s59E25H2" 0 "50S ribosomal protein L23"
"JS1_D03_01395" 13304 "s59E5H2" 0.02015208 "50S ribosomal protein L23"
"JS1_D03_01395" 13304 "s63E6H2" 0.158737 "50S ribosomal protein L23"
"JS1_D03_01396" 13305 "s59E13H2" 0.02165908 "50S ribosomal protein L2"
"JS1_D03_01396" 13305 "s59E25H2" 0 "50S ribosomal protein L2"
"JS1_D03_01396" 13305 "s59E5H2" 0.03802176 "50S ribosomal protein L2"
"JS1_D03_01396" 13305 "s63E6H2" 0.3097157 "50S ribosomal protein L2"

"JS1_D03_01397" 13306 "s59E13H2" 0 "50S ribosomal protein L6"
"JS1_D03_01397" 13306 "s59E25H2" 0 "50S ribosomal protein L6"
"JS1_D03_01397" 13306 "s59E5H2" 0 "50S ribosomal protein L6"
"JS1_D03_01397" 13306 "s63E6H2" 0.002244966 "50S ribosomal protein L6"
"JS1_D03_01408" 13317 "s59E13H2" 0 "hypothetical protein"
"JS1_D03_01408" 13317 "s59E25H2" 0 "hypothetical protein"
"JS1_D03_01408" 13317 "s59E5H2" 0 "hypothetical protein"
"JS1_D03_01408" 13317 "s63E6H2" 0.05955643 "hypothetical protein"
"JS1_D03_01409" 13318 "s59E13H2" 0 "hypothetical protein"
"JS1_D03_01409" 13318 "s59E25H2" 0 "hypothetical protein"
"JS1_D03_01409" 13318 "s59E5H2" 0.04506595 "hypothetical protein"
"JS1_D03_01409" 13318 "s63E6H2" 0.1270959 "hypothetical protein"
"JS1_D03_01410" 13319 "s59E13H2" 0.07156824 "Glucokinase"
"JS1_D03_01410" 13319 "s59E25H2" 0 "Glucokinase"
"JS1_D03_01410" 13319 "s59E5H2" 0.3403954 "Glucokinase"
"JS1_D03_01410" 13319 "s63E6H2" 0.1621909 "Glucokinase"
"JS1_D03_01411" 13320 "s59E13H2" 0.1023536 "LysM domain/BON superfamily protein"
"JS1_D03_01411" 13320 "s59E25H2" 0 "LysM domain/BON superfamily protein"
"JS1_D03_01411" 13320 "s59E5H2" 0.2983233 "LysM domain/BON superfamily protein"
"JS1_D03_01411" 13320 "s63E6H2" 0.2825306 "LysM domain/BON superfamily protein"
"JS1_D03_01412" 13321 "s59E13H2" 0.03050547 "Long-chain-fatty-acid--CoA ligase FadD15"
"JS1_D03_01412" 13321 "s59E25H2" 0 "Long-chain-fatty-acid--CoA ligase FadD15"
"JS1_D03_01412" 13321 "s59E5H2" 0.03418253 "Long-chain-fatty-acid--CoA ligase FadD15"
"JS1_D03_01412" 13321 "s63E6H2" 1.06991 "Long-chain-fatty-acid--CoA ligase FadD15"
"JS1_D03_01414" 13323 "s59E13H2" 0.5954366 "NAD-dependent malic enzyme"
"JS1_D03_01414" 13323 "s59E25H2" 0 "NAD-dependent malic enzyme"
"JS1_D03_01414" 13323 "s59E5H2" 0.00907085 "NAD-dependent malic enzyme"
"JS1_D03_01414" 13323 "s63E6H2" 0.02749638 "NAD-dependent malic enzyme"
"JS1_D03_01415" 13324 "s59E13H2" 0.06401803 "DNA-directed RNA polymerase subunit beta"
"JS1_D03_01415" 13324 "s59E25H2" 0 "DNA-directed RNA polymerase subunit beta"
"JS1_D03_01415" 13324 "s59E5H2" 0.01524798 "DNA-directed RNA polymerase subunit beta"
"JS1_D03_01415" 13324 "s63E6H2" 0.04014783 "DNA-directed RNA polymerase subunit beta"
"JS1_D03_01417" 13326 "s59E13H2" 0 "mRNA interferase MazF4"
"JS1_D03_01417" 13326 "s59E25H2" 0 "mRNA interferase MazF4"
"JS1_D03_01417" 13326 "s59E5H2" 1.18259 "mRNA interferase MazF4"
"JS1_D03_01417" 13326 "s63E6H2" 0.8690516 "mRNA interferase MazF4"
"JS1_D03_01418" 13327 "s59E13H2" 0 "hypothetical protein"
"JS1_D03_01418" 13327 "s59E25H2" 0 "hypothetical protein"
"JS1_D03_01418" 13327 "s59E5H2" 0 "hypothetical protein"
"JS1_D03_01418" 13327 "s63E6H2" 0 "hypothetical protein"
"JS1_D03_01424" 13333 "s59E13H2" 0 "Ribonuclease HII"
"JS1_D03_01424" 13333 "s59E25H2" 0 "Ribonuclease HII"
"JS1_D03_01424" 13333 "s59E5H2" 0 "Ribonuclease HII"
"JS1_D03_01424" 13333 "s63E6H2" 0.03116179 "Ribonuclease HII"
"JS1_D03_01425" 13334 "s59E13H2" 0 "hypothetical protein"
"JS1_D03_01425" 13334 "s59E25H2" 0 "hypothetical protein"
"JS1_D03_01425" 13334 "s59E5H2" 0 "hypothetical protein"

"JS1_D03_01425" 13334 "s63E6H2" 0.006520528 "hypothetical protein"
"JS1_D03_01426" 13335 "s59E13H2" 0.0175203 "Competence protein ComM"
"JS1_D03_01426" 13335 "s59E25H2" 0 "Competence protein ComM"
"JS1_D03_01426" 13335 "s59E5H2" 0.007145406 "Competence protein ComM"
"JS1_D03_01426" 13335 "s63E6H2" 0.02228281 "Competence protein ComM"
"JS1_D03_01427" 13336 "s59E13H2" 0 "Ribulose-phosphate 3-epimerase"
"JS1_D03_01427" 13336 "s59E25H2" 0 "Ribulose-phosphate 3-epimerase"
"JS1_D03_01427" 13336 "s59E5H2" 0 "Ribulose-phosphate 3-epimerase"
"JS1_D03_01427" 13336 "s63E6H2" 0.008568375 "Ribulose-phosphate 3-epimerase"
"JS1_D03_01428" 13337 "s59E13H2" 0.002475188 "GTPase Der"
"JS1_D03_01428" 13337 "s59E25H2" 0 "GTPase Der"
"JS1_D03_01428" 13337 "s59E5H2" 0.02698304 "GTPase Der"
"JS1_D03_01428" 13337 "s63E6H2" 0.0205564 "GTPase Der"
"JS1_D03_01429" 13338 "s59E13H2" 0 "Glycerol-3-phosphate acyltransferase"
"JS1_D03_01429" 13338 "s59E25H2" 0 "Glycerol-3-phosphate acyltransferase"
"JS1_D03_01429" 13338 "s59E5H2" 0.03124638 "Glycerol-3-phosphate acyltransferase"
"JS1_D03_01429" 13338 "s63E6H2" 0.02441748 "Glycerol-3-phosphate acyltransferase"
"JS1_D03_01430" 13339 "s59E13H2" 0.0008105433 "ferredoxin"
"JS1_D03_01430" 13339 "s59E25H2" 0 "ferredoxin"
"JS1_D03_01430" 13339 "s59E5H2" 0.04283588 "ferredoxin"
"JS1_D03_01430" 13339 "s63E6H2" 0.02035766 "ferredoxin"
"JS1_D03_01431" 13340 "s59E13H2" 0.0002550384 "DNA topoisomerase 1"
"JS1_D03_01431" 13340 "s59E25H2" 0 "DNA topoisomerase 1"
"JS1_D03_01431" 13340 "s59E5H2" 0.008345182 "DNA topoisomerase 1"
"JS1_D03_01431" 13340 "s63E6H2" 0.0192395 "DNA topoisomerase 1"
"JS1_D03_01433" 13342 "s59E13H2" 0.006344112 "Elongation factor Tu"
"JS1_D03_01433" 13342 "s59E25H2" 0 "Elongation factor Tu"
"JS1_D03_01433" 13342 "s59E5H2" 0.04611139 "Elongation factor Tu"
"JS1_D03_01433" 13342 "s63E6H2" 0.177591 "Elongation factor Tu"
"JS1_D03_01434" 13343 "s59E13H2" 0 "UDP-N-acetylmuramoylalanine--D-glutamate ligase"
"JS1_D03_01434" 13343 "s59E25H2" 0 "UDP-N-acetylmuramoylalanine--D-glutamate ligase"
"JS1_D03_01434" 13343 "s59E5H2" 0 "UDP-N-acetylmuramoylalanine--D-glutamate ligase"
"JS1_D03_01434" 13343 "s63E6H2" 0 "UDP-N-acetylmuramoylalanine--D-glutamate ligase"
"JS1_D03_01435" 13344 "s59E13H2" 0.0008637664 "Acyltransferase family protein"
"JS1_D03_01435" 13344 "s59E25H2" 0 "Acyltransferase family protein"
"JS1_D03_01435" 13344 "s59E5H2" 0.4119899 "Acyltransferase family protein"
"JS1_D03_01435" 13344 "s63E6H2" 0.03793795 "Acyltransferase family protein"
"JS1_D03_01436" 13345 "s59E13H2" 0 "7,8-dihydropterin-6-methyl-4-(beta-D-ribofuranosyl)-aminobenzene-5'-phosphate synthase"
"JS1_D03_01436" 13345 "s59E25H2" 0 "7,8-dihydropterin-6-methyl-4-(beta-D-ribofuranosyl)-aminobenzene-5'-phosphate synthase"
"JS1_D03_01436" 13345 "s59E5H2" 2.059566 "7,8-dihydropterin-6-methyl-4-(beta-D-ribofuranosyl)-aminobenzene-5'-phosphate synthase"
"JS1_D03_01436" 13345 "s63E6H2" 0.03609962 "7,8-dihydropterin-6-methyl-4-(beta-D-ribofuranosyl)-aminobenzene-5'-phosphate synthase"
"JS1_D03_01437" 13346 "s59E13H2" 0.03822603 "Purine nucleoside phosphorylase DeoD-type"
"JS1_D03_01437" 13346 "s59E25H2" 0 "Purine nucleoside phosphorylase DeoD-type"
"JS1_D03_01437" 13346 "s59E5H2" 0.9804403 "Purine nucleoside phosphorylase DeoD-type"
"JS1_D03_01437" 13346 "s63E6H2" 0.06800664 "Purine nucleoside phosphorylase DeoD-type"
"JS1_D03_01440" 13349 "s59E13H2" 0.9854424 "tRNA uridine 5-carboxymethylaminomethyl modification enzyme MnmG"
"JS1_D03_01440" 13349 "s59E25H2" 0.1333333 "tRNA uridine 5-carboxymethylaminomethyl modification enzyme MnmG"

"JS1_D03_01440" 13349 "s59E5H2" 2.350943 "tRNA uridine 5-carboxymethylaminomethyl modification enzyme MnmG"
"JS1_D03_01440" 13349 "s63E6H2" 0.3492365 "tRNA uridine 5-carboxymethylaminomethyl modification enzyme MnmG"
"JS1_D03_01441" 13350 "s59E13H2" 1.679592 "RNA polymerase-binding transcription factor CarD"
"JS1_D03_01441" 13350 "s59E25H2" 0 "RNA polymerase-binding transcription factor CarD"
"JS1_D03_01441" 13350 "s59E5H2" 0.9465408 "RNA polymerase-binding transcription factor CarD"
"JS1_D03_01441" 13350 "s63E6H2" 0.2126644 "RNA polymerase-binding transcription factor CarD"
"JS1_D03_01442" 13351 "s59E13H2" 5.965089 "Oligopeptide transport ATP-binding protein OppF"
"JS1_D03_01442" 13351 "s59E25H2" 0 "Oligopeptide transport ATP-binding protein OppF"
"JS1_D03_01442" 13351 "s59E5H2" 2.205183 "Oligopeptide transport ATP-binding protein OppF"
"JS1_D03_01442" 13351 "s63E6H2" 0.35674 "Oligopeptide transport ATP-binding protein OppF"
"JS1_D03_01443" 13352 "s59E13H2" 4.967672 "Oligopeptide transport ATP-binding protein OppD"
"JS1_D03_01443" 13352 "s59E25H2" 0 "Oligopeptide transport ATP-binding protein OppD"
"JS1_D03_01443" 13352 "s59E5H2" 2.090309 "Oligopeptide transport ATP-binding protein OppD"
"JS1_D03_01443" 13352 "s63E6H2" 0.3365171 "Oligopeptide transport ATP-binding protein OppD"
"JS1_D03_01444" 13353 "s59E13H2" 1.206898 "Glutathione transport system permease protein GsiD"
"JS1_D03_01444" 13353 "s59E25H2" 0.4512472 "Glutathione transport system permease protein GsiD"
"JS1_D03_01444" 13353 "s59E5H2" 1.198437 "Glutathione transport system permease protein GsiD"
"JS1_D03_01444" 13353 "s63E6H2" 0.2010094 "Glutathione transport system permease protein GsiD"
"JS1_D03_01445" 13354 "s59E13H2" 1.270133 "Dipeptide transport system permease protein DppB"
"JS1_D03_01445" 13354 "s59E25H2" 0 "Dipeptide transport system permease protein DppB"
"JS1_D03_01445" 13354 "s59E5H2" 1.671518 "Dipeptide transport system permease protein DppB"
"JS1_D03_01445" 13354 "s63E6H2" 0.3153004 "Dipeptide transport system permease protein DppB"
"JS1_D03_01446" 13355 "s59E13H2" 0.3536334 "Periplasmic dipeptide transport protein precursor"
"JS1_D03_01446" 13355 "s59E25H2" 0 "Periplasmic dipeptide transport protein precursor"
"JS1_D03_01446" 13355 "s59E5H2" 1.712901 "Periplasmic dipeptide transport protein precursor"
"JS1_D03_01446" 13355 "s63E6H2" 0.1558529 "Periplasmic dipeptide transport protein precursor"
"JS1_D03_01447" 13356 "s59E13H2" 1.57777 "Murein hydrolase activator NlpD precursor"
"JS1_D03_01447" 13356 "s59E25H2" 0 "Murein hydrolase activator NlpD precursor"
"JS1_D03_01447" 13356 "s59E5H2" 0.473418 "Murein hydrolase activator NlpD precursor"
"JS1_D03_01447" 13356 "s63E6H2" 0.2901781 "Murein hydrolase activator NlpD precursor"
"JS1_D03_01448" 13357 "s59E13H2" 0 "Helix-turn-helix domain protein"
"JS1_D03_01448" 13357 "s59E25H2" 0 "Helix-turn-helix domain protein"
"JS1_D03_01448" 13357 "s59E5H2" 0.1115908 "Helix-turn-helix domain protein"
"JS1_D03_01448" 13357 "s63E6H2" 0.1602906 "Helix-turn-helix domain protein"
"JS1_D03_01449" 13358 "s59E13H2" 0.5964891 "hypothetical protein"
"JS1_D03_01449" 13358 "s59E25H2" 0 "hypothetical protein"
"JS1_D03_01449" 13358 "s59E5H2" 0.5766606 "hypothetical protein"
"JS1_D03_01449" 13358 "s63E6H2" 0.2422953 "hypothetical protein"
"JS1_D03_01450" 13359 "s59E13H2" 1.706658 "Soluble lytic murein transglycosylase precursor"
"JS1_D03_01450" 13359 "s59E25H2" 0 "Soluble lytic murein transglycosylase precursor"
"JS1_D03_01450" 13359 "s59E5H2" 0.4992204 "Soluble lytic murein transglycosylase precursor"
"JS1_D03_01450" 13359 "s63E6H2" 0.2143731 "Soluble lytic murein transglycosylase precursor"
"JS1_D03_01451" 13360 "s59E13H2" 0.1025567 "B3/4 domain protein"
"JS1_D03_01451" 13360 "s59E25H2" 0 "B3/4 domain protein"
"JS1_D03_01451" 13360 "s59E5H2" 0.03401845 "B3/4 domain protein"
"JS1_D03_01451" 13360 "s63E6H2" 0.6724176 "B3/4 domain protein"
"JS1_D03_01452" 13361 "s59E13H2" 0.439875 "hypothetical protein"

"JS1_D03_01452" 13361 "s59E25H2" 0 "hypothetical protein"
"JS1_D03_01452" 13361 "s59E5H2" 0.5919984 "hypothetical protein"
"JS1_D03_01452" 13361 "s63E6H2" 0.103869 "hypothetical protein"
"JS1_D03_01453" 13362 "s59E13H2" 0.005761894 "hypothetical protein"
"JS1_D03_01453" 13362 "s59E25H2" 0 "hypothetical protein"
"JS1_D03_01453" 13362 "s59E5H2" 0.006607752 "hypothetical protein"
"JS1_D03_01453" 13362 "s63E6H2" 0 "hypothetical protein"
"JS1_D03_01454" 13363 "s59E13H2" 0 "Protein-glutamine gamma-glutamyltransferase"
"JS1_D03_01454" 13363 "s59E25H2" 0 "Protein-glutamine gamma-glutamyltransferase"
"JS1_D03_01454" 13363 "s59E5H2" 0.04168032 "Protein-glutamine gamma-glutamyltransferase"
"JS1_D03_01454" 13363 "s63E6H2" 0.0325392 "Protein-glutamine gamma-glutamyltransferase"
"JS1_D03_01455" 13364 "s59E13H2" 0 "hypothetical protein"
"JS1_D03_01455" 13364 "s59E25H2" 0 "hypothetical protein"
"JS1_D03_01455" 13364 "s59E5H2" 0.06779452 "hypothetical protein"
"JS1_D03_01455" 13364 "s63E6H2" 0.0005296351 "hypothetical protein"
"JS1_D03_01456" 13365 "s59E13H2" 0.001909846 "hypothetical protein"
"JS1_D03_01456" 13365 "s59E25H2" 0 "hypothetical protein"
"JS1_D03_01456" 13365 "s59E5H2" 0 "hypothetical protein"
"JS1_D03_01456" 13365 "s63E6H2" 7.78327e-05 "hypothetical protein"
"JS1_D03_01458" 13367 "s59E13H2" 0.02315724 "hypothetical protein"
"JS1_D03_01458" 13367 "s59E25H2" 0 "hypothetical protein"
"JS1_D03_01458" 13367 "s59E5H2" 0.003323471 "hypothetical protein"
"JS1_D03_01458" 13367 "s63E6H2" 0.0116532 "hypothetical protein"
"JS1_D03_01460" 13369 "s59E13H2" 0 "CobQ/CobB/MinD/ParA nucleotide binding domain protein"
"JS1_D03_01460" 13369 "s59E25H2" 0 "CobQ/CobB/MinD/ParA nucleotide binding domain protein"
"JS1_D03_01460" 13369 "s59E5H2" 0 "CobQ/CobB/MinD/ParA nucleotide binding domain protein"
"JS1_D03_01460" 13369 "s63E6H2" 0 "CobQ/CobB/MinD/ParA nucleotide binding domain protein"
"JS1_D03_01463" 13372 "s59E13H2" 0 "hypothetical protein"
"JS1_D03_01463" 13372 "s59E25H2" 0 "hypothetical protein"
"JS1_D03_01463" 13372 "s59E5H2" 0 "hypothetical protein"
"JS1_D03_01463" 13372 "s63E6H2" 0 "hypothetical protein"
"JS1_D03_01464" 13373 "s59E13H2" 0 "hypothetical protein"
"JS1_D03_01464" 13373 "s59E25H2" 0 "hypothetical protein"
"JS1_D03_01464" 13373 "s59E5H2" 0 "hypothetical protein"
"JS1_D03_01464" 13373 "s63E6H2" 0 "hypothetical protein"
"JS1_D03_01465" 13374 "s59E13H2" 0 "hypothetical protein"
"JS1_D03_01465" 13374 "s59E25H2" 0 "hypothetical protein"
"JS1_D03_01465" 13374 "s59E5H2" 0 "hypothetical protein"
"JS1_D03_01465" 13374 "s63E6H2" 0.0003708499 "hypothetical protein"
"JS1_D03_01467" 13376 "s59E13H2" 0 "hypothetical protein"
"JS1_D03_01467" 13376 "s59E25H2" 0 "hypothetical protein"
"JS1_D03_01467" 13376 "s59E5H2" 0 "hypothetical protein"
"JS1_D03_01467" 13376 "s63E6H2" 0.009848449 "hypothetical protein"
"JS1_D03_01468" 13377 "s59E13H2" 0 "hypothetical protein"
"JS1_D03_01468" 13377 "s59E25H2" 0 "hypothetical protein"
"JS1_D03_01468" 13377 "s59E5H2" 0 "hypothetical protein"
"JS1_D03_01468" 13377 "s63E6H2" 0 "hypothetical protein"

"JS1_D03_01469" 13378 "s59E13H2" 0 "Helix-turn-helix domain protein"
"JS1_D03_01469" 13378 "s59E25H2" 0 "Helix-turn-helix domain protein"
"JS1_D03_01469" 13378 "s59E5H2" 0 "Helix-turn-helix domain protein"
"JS1_D03_01469" 13378 "s63E6H2" 0 "Helix-turn-helix domain protein"
"JS1_D03_01470" 13379 "s59E13H2" 0 "hypothetical protein"
"JS1_D03_01470" 13379 "s59E25H2" 0 "hypothetical protein"
"JS1_D03_01470" 13379 "s59E5H2" 0 "hypothetical protein"
"JS1_D03_01470" 13379 "s63E6H2" 0 "hypothetical protein"
"JS1_D03_01471" 13380 "s59E13H2" 0 "hypothetical protein"
"JS1_D03_01471" 13380 "s59E25H2" 0 "hypothetical protein"
"JS1_D03_01471" 13380 "s59E5H2" 0 "hypothetical protein"
"JS1_D03_01471" 13380 "s63E6H2" 0 "hypothetical protein"
"JS1_D03_01472" 13381 "s59E13H2" 0 "hypothetical protein"
"JS1_D03_01472" 13381 "s59E25H2" 0 "hypothetical protein"
"JS1_D03_01472" 13381 "s59E5H2" 0 "hypothetical protein"
"JS1_D03_01472" 13381 "s63E6H2" 0 "hypothetical protein"
"JS1_D03_01473" 13382 "s59E13H2" 0.003267021 "hypothetical protein"
"JS1_D03_01473" 13382 "s59E25H2" 0 "hypothetical protein"
"JS1_D03_01473" 13382 "s59E5H2" 0 "hypothetical protein"
"JS1_D03_01473" 13382 "s63E6H2" 0 "hypothetical protein"
"JS1_D03_01474" 13383 "s59E13H2" 0.003193895 "hypothetical protein"
"JS1_D03_01474" 13383 "s59E25H2" 0 "hypothetical protein"
"JS1_D03_01474" 13383 "s59E5H2" 0 "hypothetical protein"
"JS1_D03_01474" 13383 "s63E6H2" 0 "hypothetical protein"
"JS1_D03_01475" 13384 "s59E13H2" 0 "hypothetical protein"
"JS1_D03_01475" 13384 "s59E25H2" 0 "hypothetical protein"
"JS1_D03_01475" 13384 "s59E5H2" 0 "hypothetical protein"
"JS1_D03_01475" 13384 "s63E6H2" 0 "hypothetical protein"
"JS1_D03_01477" 13386 "s59E13H2" 0 "hypothetical protein"
"JS1_D03_01477" 13386 "s59E25H2" 0 "hypothetical protein"
"JS1_D03_01477" 13386 "s59E5H2" 0 "hypothetical protein"
"JS1_D03_01477" 13386 "s63E6H2" 0 "hypothetical protein"
"JS1_D03_01478" 13387 "s59E13H2" 0 "hypothetical protein"
"JS1_D03_01478" 13387 "s59E25H2" 0 "hypothetical protein"
"JS1_D03_01478" 13387 "s59E5H2" 0 "hypothetical protein"
"JS1_D03_01478" 13387 "s63E6H2" 0 "hypothetical protein"
"JS1_D03_01480" 13389 "s59E13H2" 0.07129197 "tRNA pseudouridine synthase A"
"JS1_D03_01480" 13389 "s59E25H2" 0 "tRNA pseudouridine synthase A"
"JS1_D03_01480" 13389 "s59E5H2" 0.07621378 "tRNA pseudouridine synthase A"
[truncated: 471,993 more chars]
